# *In Vitro* Transformation of Primary Human CD34+ Cells by AML Fusion Oncogenes: Early Gene Expression Profiling Reveals Possible Drug Target in AML

**DOI:** 10.1371/journal.pone.0012464

**Published:** 2010-08-27

**Authors:** Anmaar M. Abdul-Nabi, Enas R. Yassin, Nobish Varghese, Hrishikesh Deshmukh, Nabeel R. Yaseen

**Affiliations:** Department of Pathology and Immunology, Washington University School of Medicine, St. Louis, Missouri, United States of America; George Mason University, United States of America

## Abstract

Different fusion oncogenes in acute myeloid leukemia (AML) have distinct clinical and laboratory features suggesting different modes of malignant transformation. Here we compare the *in vitro* effects of representatives of 4 major groups of AML fusion oncogenes on primary human CD34+ cells. As expected from their clinical similarities, MLL-AF9 and NUP98-HOXA9 had very similar effects *in vitro*. They both caused erythroid hyperplasia and a clear block in erythroid and myeloid maturation. On the other hand, AML1-ETO and PML-RARA had only modest effects on myeloid and erythroid differentiation. All oncogenes except PML-RARA caused a dramatic increase in long-term proliferation and self-renewal. Gene expression profiling revealed two distinct temporal patterns of gene deregulation. Gene deregulation by MLL-AF9 and NUP98-HOXA9 peaked 3 days after transduction. In contrast, the vast majority of gene deregulation by AML1-ETO and PML-RARA occurred within 6 hours, followed by a dramatic drop in the numbers of deregulated genes. Interestingly, the p53 inhibitor MDM2 was upregulated by AML1-ETO at 6 hours. Nutlin-3, an inhibitor of the interaction between MDM2 and p53, specifically inhibited the proliferation and self-renewal of primary human CD34+ cells transduced with AML1-ETO, suggesting that MDM2 upregulation plays a role in cell transformation by AML1-ETO. These data show that differences among AML fusion oncogenes can be recapitulated *in vitro* using primary human CD34+ cells and that early gene expression profiling in these cells can reveal potential drug targets in AML.

## Introduction

Acute myeloid leukemia (AML) is a group of hematopoietic disorders characterized by uncontrolled proliferation and various degrees of blocked differentiation. Approximately 50% of AML patients have chromosomal rearrangements that result in the expression of fusion oncogenes [1], the majority of which involve genes that fall into four groups:

1) Core-binding factor (CBF) transcriptional regulators - these fusions involve the two subunits of CBF: AML1 and CBFB. The most common is t(8;21)(q22;q22) that fuses *AML1* (*RUNX1*) with *ETO* (*RUNX1T1*), resulting in expression of AML1-ETO [2].

2) Retinoic acid receptor alpha (RARA) - rearrangements of the *RARA* gene result in acute promyelocytic leukemia (APL), a subtype of AML. The most common *RARA* rearrangement is the t(15∶17)(q21;q22) translocation that fuses *RARA* to the promyelocytic leukemia gene (*PML*) resulting in expression of PML-RARA [3].

3) Mixed lineage leukemia (MLL) - at least 104 different rearrangements of the *MLL* gene at chromosome 11q23 have been identified and 64 different fusion partners have been molecularly characterized [4]. The most frequent *MLL* gene translocation in AML is t(9;11)(p22;q23) which results in expression of the MLL–AF9 fusion [4], [5], [6].

4) Nucleoporins - two nucleoporins have been implicated in AML: NUP214 and NUP98 [7], [8], [9], [10]. While only a handful of *NUP214* rearrangements have been described, there are at least 24 *NUP98* fusion oncogenes. The prototype of NUP98 fusions is NUP98-HOXA9 that results from the t(7;11)(p15;p15) translocation.

Clinical data indicate that leukemias associated with *MLL* and *NUP98* gene rearrangements have similar features and are biologically distinct from those associated with CBF and *RARA* gene rearrangements. The former tend to follow treatment with topoisomerase II inhibitors, have multiple fusion partners, and respond poorly to treatment [5], [6], [9], [11], [12], [13]; whereas the latter usually occur de novo, have one major fusion partner, and have a favorable prognosis [1].

Numerous studies have sought to recapitulate human AML by expressing fusion oncogenes in mouse bone marrow [14]. On the other hand, studies of the leukemic transformation of primary human hematopoietic cells by AML oncogenes are relatively few [15], [16], [17], [18], [19], [20], [21], [22], [23], [24] in spite of the fact that they offer the potential to identify drug targets and to test drug candidates [25]. In the available studies the source of cells, assays used, culture conditions, and the extent and timing of gene expression profiling have been variable, precluding a comparison of the effects of different AML oncogenes.

This study was undertaken with the goal of determining whether the clinical differences among the various AML oncogenes are reflected in different modes of transformation *in vitro* using primary human CD34+ cells, and whether early *in vitro* gene expression profiling can shed light on mechanisms of leukemogenesis. We compared the *in vitro* effects of 4 representative AML oncogenes, *PML-RARA*, *AML1-ETO*, *MLL-AF9* and *NUP98-HOXA9*, on the differentiation, proliferation, and self-renewal of primary human CD34+ cells under identical conditions. We also performed gene expression profiling in duplicate for each oncogene over 3 time points starting 6 h after transduction. Early gene expression profiling showed upregulation of MDM2 that was confirmed by quantitative RT-PCR and immunoblotting in triplicate. Treatment with the MDM2 inhibitor nutlin-3 suppressed long-term growth and self-renewal of cells transformed by AML1-ETO, suggesting that MDM2 is a possible mediator and drug target in AML1-ETO leukemogenesis. Our data suggest that early gene expression profiling in combination with *in vitro* assays using primary human CD34+ cells can lead to the discovery of potential drug targets that may not be identifiable by other approaches.

## Results

### NUP98-HOXA9 and MLL-AF9 show similar effects on differentiation that differ from those of AML1-ETO and PML-RARA

**Figure 1:**
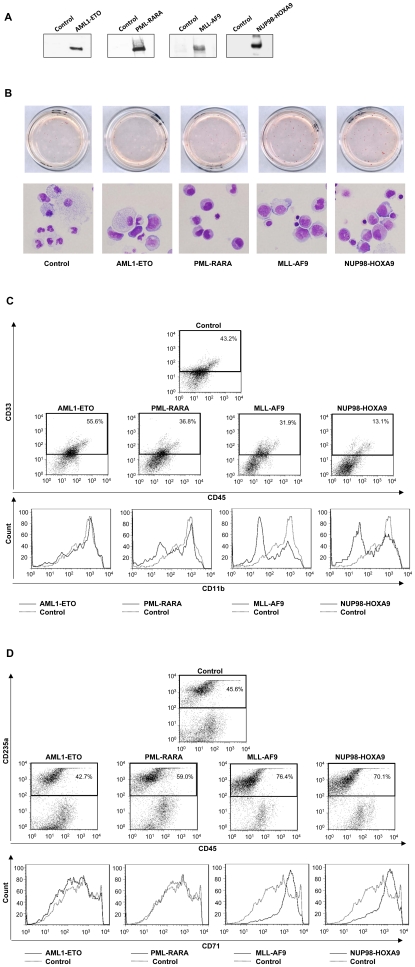
Effects of AML fusion oncogenes on the differentiation of primary human CD34+ cells. (**A**) Immunoblotting shows expression of the indicated AML oncogenes in primary human CD34+ cells. (**B**) CFC assays of primary human CD34+ cells transduced with the indicated oncogenes. Unmagnified plates are shown in the upper panels and representative fields of Giemsa-stained Cytospin preparations from the CFC plates are shown in the lower panels. Differential counts are shown in Table 2. (**C**) Flow cytometric analysis of myeloid differentiation of cells harvested from the CFC plates. Myeloid (CD33+) cells were gated and the level of CD11b expression was plotted on histograms in comparison to control. (**D**) Flow cytometric analysis of erythroid differentiation of cells harvested from the CFC plates. Erythroid (CD235+) cells were gated and their level of CD71 expression was plotted on histograms in comparison to control.

In order to determine whether the clinical differences among the various AML oncogenes are reflected during the *in vitro* transformation of primary human cells, retroviral vectors expressing AML1-ETO, PML-RARA, MLL-AF9, or NUP98-HOXA9 were used to transduce human CD34+ hematopoietic progenitor/stem cells from mobilized peripheral blood. GFP-positive cells were sorted and protein expression was confirmed by immunoblotting (Fig. 1A). Cells were plated for colony-forming cell (CFC) assays, and after 14 days, the NUP98-HOXA9 and MLL-AF9 plates looked markedly different from the others, with large prominent erythroid colonies (Fig. 1B). PML-RARA samples showed increased numbers of small erythroid colonies (Fig. 1B and Table 1). AML1-ETO caused a decrease in the number of erythroid colonies, consistent with previously published data [18].

**Table 1:** CFCs colony counts.

	Erythroid	Myelomonocytic	Mixed
**Control**	102.7±12.7	116.0±17.9	3.3±1.2
**AML1-ETO**	66.3±6.3*	95.7±4.1	2.7±0.5
**PML-RARA**	133.3±14*	107.7±6.6	5.0±1.6
**MLL-AF9**	108.0±2.9	119.7±4.6	9.3±2.1
**NUP98-HOXA9**	120.0±7.5*	117.7±10.4	13.3±4.8

*P<0.05

Colonies on CFC plates were counted at 40X magnification and classified into three categories: erythroid, myelomonocytic, and mixed. The *P* value was obtained by comparing to the control using a paired two-tailed distribution t-test. Averages from 3 independent experiments are shown.

To evaluate differentiation at the cellular level, 500-cell differential counts from Giemsa-stained slides of cells recovered from the CFC plates were performed. Cells were classified into primitive, intermediate myeloid, mature myeloid, intermediate erythroid, and mature erythroid (see Materials and Methods). The average numbers from 3 independent experiments are shown in Table 2 and representative fields are shown in Fig. 1B. Both NUP98-HOXA9 and MLL-AF9 caused a marked increase in the numbers of erythroid cells. In addition, NUP98-HOXA9 caused a marked shift of the erythroid cells towards intermediate differentiation, indicating a block in erythroid maturation. In contrast, AML1-ETO and PML-RARA did not show a significant effect on the numbers and maturation of erythroid cells in spite of their effects on erythroid colony numbers. All fusion oncogenes increased the percentage of primitive cells (blasts and promyelocytes), although the difference was at the border of statistical significance in the case of PML-RARA. On the other hand, all oncogenes except AML1-ETO caused an increase in the percentage of intermediate myeloid cells and a decrease in mature myeloid cells, consistent with a block in myeloid maturation.

**Table 2:** The effects of the fusion oncogenes on hematopoietic differentiation assessed by cell morphology.

	Control	AML1-ETO	PML-RARA	MLL-AF9	NUP98-HOXA9
Primitive cells %	1.1±0.5	6.6±1.6*	3.8±1.7***	10.9±3.1*	9.3±1.5*
Intermediate myeloid %	7.3±2.7	9.3±2.3	17.4±3.5*	16.0±0.5*	22.2±4.3*
Mature myeloid %	63.8±2.6	57.7±2.7	48.6±4.7*	21.8±4.4*	12.8±2.3**
Intermediate erythroid %	5.0±3.0	2.7±0.8	3.4±1.4	14.8±1.5*	30.4±6.0*
Mature erythroid %	22.8±3.1	23.8±4.2	26.5±2	36.7±1.9*	25.0±5.5
**Total Cells x10^6^**	7.5±0.9	7.2±1.1	7±0.6	7.5±1.0	11.6±0.5*

*P<0.05

**P<0.01

***P = 0.0507

Cells from 2 CFC plates for each experimental condition were harvested, Giemsa-stained Cytospin smears were prepared, and 500-cell differential counts were performed. The numbers shown are averages from 3 independent experiments ± standard deviations. The *P* value was obtained by comparing to control using a paired two-tailed distribution t-test.

Differentiation was further assessed by flow cytometry. For myeloid differentiation, CD11b expression on CD33+ myeloid cells was compared to the control sample (Fig. 1C). NUP98-HOXA9, MLL-AF9, and to a lesser extent PML-RARA decreased the expression of CD11b, whereas AML1-ETO had no obvious effect on CD11b expression. For flow cytometric analysis of erythroid differentiation, erythroid cells (CD235a+) were gated and the level of CD71 expression was compared to the control sample (Fig. 1D). Both NUP98-HOXA9 and MLL-AF9 caused an increase in CD71 expression on erythroid cells indicating a block in terminal erythroid differentiation; in contrast, neither AML1-ETO nor PML-RARA caused a block in erythroid differentiation.

In summary, NUP98-HOXA9 and MLL-AF9 showed similar effects on differentiation including a block of both myeloid and erythroid maturation and an increase in erythroid cells. In contrast, PML-RARA and AML1-ETO altered the numbers of erythroid colonies but had no significant effects on the maturation and numbers of erythroid cells, and had limited effects on myeloid differentiation.

### Fusion oncogenes, except PML-RARA, induce long-term proliferation and self-renewal of primary human CD34+ cells

**Figure 2:**
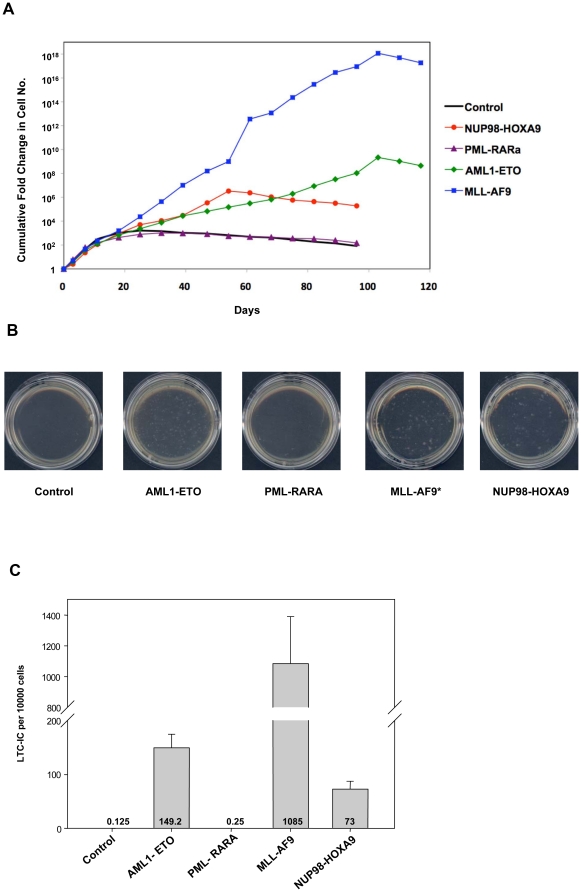
Effects of AML fusion oncogenes on the proliferation and self-renewal of primary human CD34+ cells. (**A**) Long-term liquid culture of primary human CD34+ cells transduced with the indicated oncogenes. Data are representative of 3 independent experiments. (**B**) Representative plates from LTC-IC assays of primary human CD34+ cells transduced with the indicated oncogenes. * Due to the much larger number of LTC-ICs in the MLL-AF9 sample, the MLL-AF9 plate was seeded with 1/10 of the sample in order to allow the visualization of discrete colonies. Data are representative of 3 independent experiments. (**C**) Average numbers of LTC-ICs per 10,000 input cells from 3 independent experiments.

The effects of the fusion oncogenes on the proliferation of primary human CD34+ cells were measured by long-term *in vitro* culture. Sorted primary human CD34+ cells transduced with control, AML1-ETO, PML-RARA, MLL-AF9, or NUP98-HOXA9 retrovirus were continuously cultured in liquid media with a cytokine cocktail and subjected to periodic cell counting and feeding as previously described [20]. The typical growth pattern, which was reproducible in three independent experiments, is shown in Fig. 2A. With the exception of PML-RARA, the fusion oncogenes increased cell numbers several orders of magnitude over the control samples during almost 4 months of continuous culture.

To assess the numbers of self-renewing cells, the long-term culture-initiating cell (LTC-IC) assay [26] was performed. Again all the fusion oncogenes except PML-RARA induced a marked increase in LTC-IC numbers compared to control (Fig. 2B and 2C).

### Gene expression profiling shows 2 distinct temporal patterns of gene deregulation by AML oncogenes

**Figure 3:**
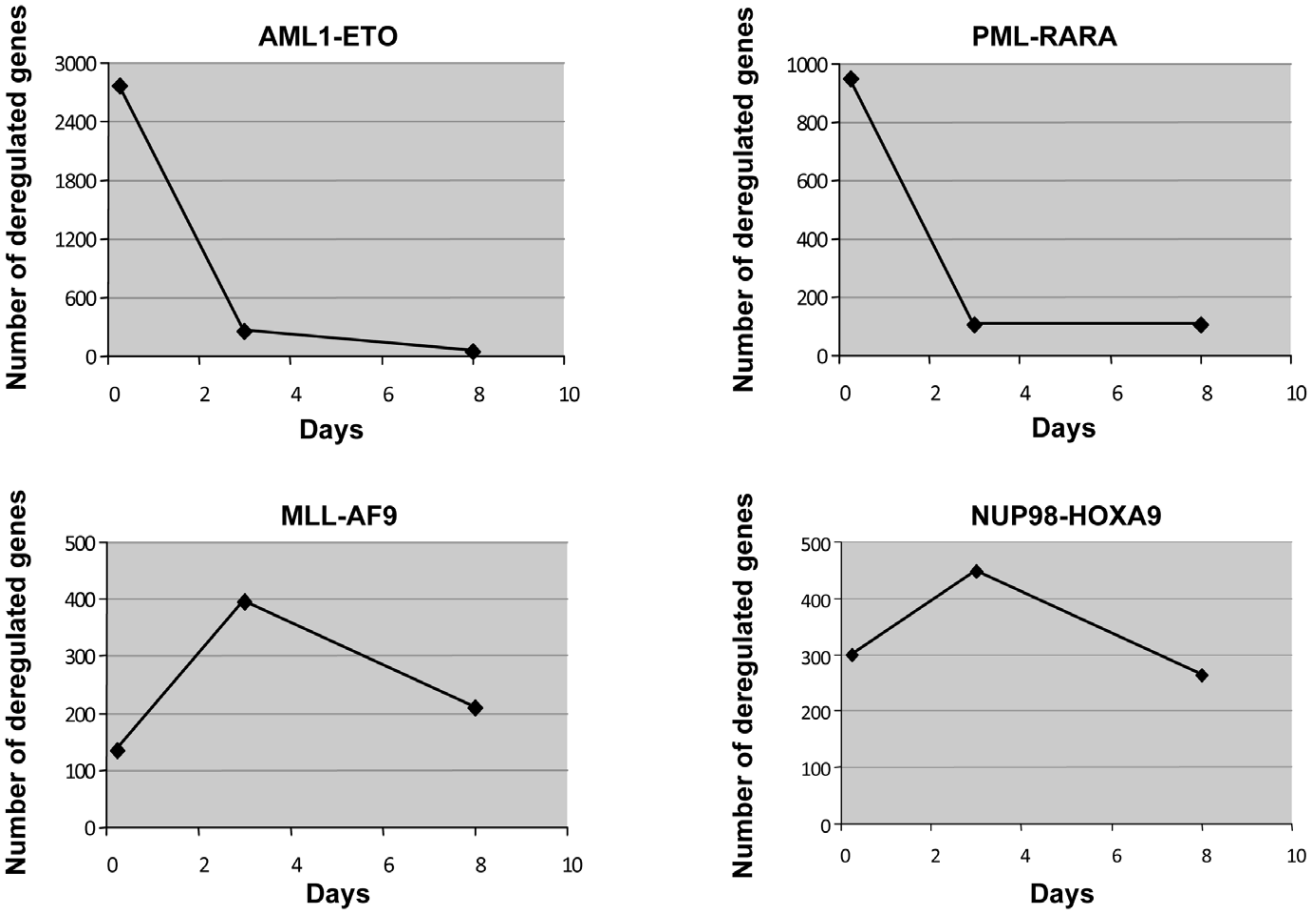
Microarray analysis of the effects of AML fusion oncogenes on primary human CD34+ cells. Temporal patterns of gene deregulation by the indicated oncogenes. Primary human CD34+ cells were transduced with the indicated oncogenes by nucleofection for the 6-hour time point and retrovirally for the 3- and 8-day time points. Cells were sorted for GFP expression and compared to empty vector controls. The numbers shown are of genes that were up- or down-regulated by 2-fold or more in two separate experiments.

In order to explore the molecular basis behind the *in vitro* biological findings, gene expression profiling was performed at 3 time points: 6 h, 3 d, and 8 d. For the 3 d and 8 d time points, the oncogenes were retrovirally expressed in primary human CD34+ cells as described above, with cells transduced with empty retroviral vector as controls. As retroviral transduction requires days, for the 6 h time point the oncogenes were expressed from a pTracer-CMV/Bsd plasmid using nucleofection. Cells nucleofected with empty pTracer-CMV/Bsd vector were used as a control. At all time points, fold changes in gene expression levels were obtained for each oncogene compared to empty vector control as described in Materials and Methods. The entire experiment was performed twice, and only genes that showed a 2-fold or more difference from control in both experiments were considered deregulated (Tables S1, S2, S3, S4, S5, S6, S7, S8, S9, S10, S11 and S12). AML1-ETO and PML-RARA deregulated large numbers of genes within 6 h of introduction into cells and the number of deregulated genes fell sharply thereafter (Fig. 3). This is consistent with the fact that AML1-ETO and PML-RARA are aberrant transcription factors with direct effects on gene transcription. Notably, there was a preponderance of downregulated genes in both cases at 6 h: AML1-ETO upregulated 996 genes and downregulated 1771 genes; PML-RARA upregulated 271 genes and downregulated 675 genes (Tables S1, S2, S3, S4, S5 and S6). This is consistent with data indicating that AML1-ETO and PML-RARA can act as transcriptional repressors [3], [27]. In contrast, gene deregulation by NUP98-HOXA9 and MLL-AF9 peaked at 3 days after introduction of the oncogene with a preponderance of upregulated genes: MLL-AF9 upregulated 244 genes and downregulated 151 genes; NUP98-HOXA9 upregulated 419 genes and downregulated 28 genes (Fig. 3 and Tables S7, S8, S9, S10, S11 and S12). The later peak of gene deregulation by NUP98-HOXA9 and MLL-AF9 suggests that some of the effects of NUP98 and MLL fusions on gene expression may not be due to direct transcriptional modulation. The similar time course of gene deregulation by NUP98-HOXA9 and MLL-AF9 is consistent with the fact that these two oncogenes have similar effects on the proliferation and differentiation of primary human CD34+ cells (Figures 1 and 2) and the fact that MLL and NUP98 fusions share similar clinical presentations [5], [6], [9], [11], [12], [13].

Microarray data were also analyzed using the Significance Analysis of Microarrays (SAM) [28]. The results again showed that, unlike MLL-AF9 and NUP98-HOXA9, maximal gene deregulation by AML1-ETO and PML-RARA occurred at the 6 h time point, with the number of deregulated genes falling dramatically at subsequent time points (Tables S13, S14, S15, S16, S17, S18, S19, S20, S21, S22, S23 and S24).

### Early overexpression of MDM2 may contribute to the transformation of primary human cells by AML1-ETO

**Figure 4:**
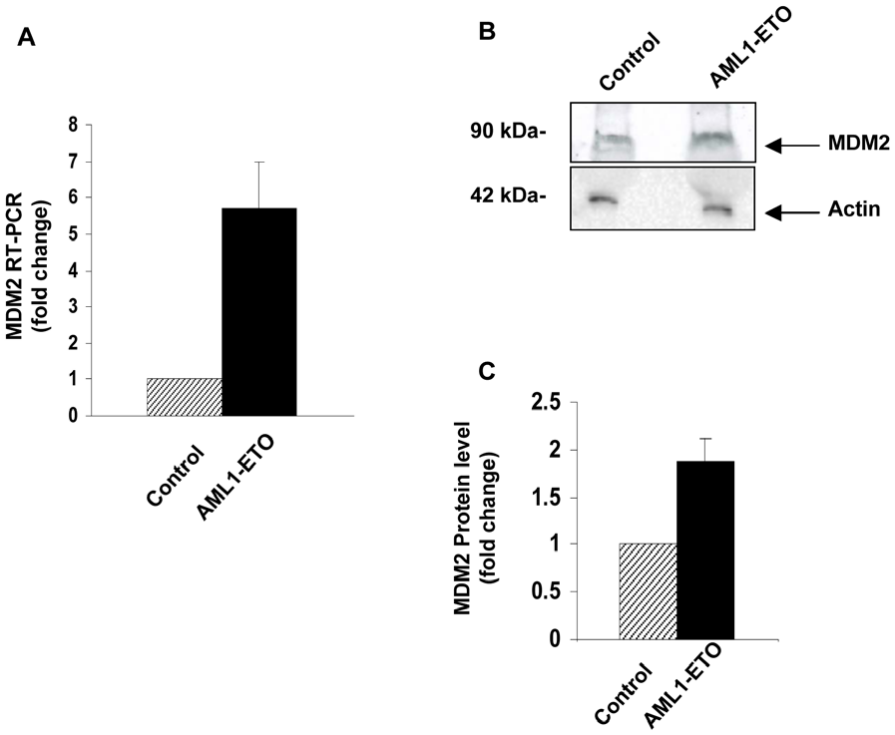
MDM2 overexpression in primary human CD34+ cells transduced with AML1-ETO. (**A**) Quantitative RT-PCR confirms early overexpression of MDM2 RNA by CD34+ cells transduced with AML1-ETO. Fold change shown is the average of 3 independent experiments. (**B**) Immunoblotting shows increased MDM2 protein levels in cells transduced with AML1-ETO. (**C**) MDM2 protein was quantified by using the ChemiDoc XRS imaging system (Bio-Rad) with Quantity One 1-D software and fold change over control was calculated. Fold change shown is the average of 3 independent experiments.

Interestingly, one of the genes upregulated by AML1-ETO and PML-RARA at 6 h but not at later time points was *MDM2* as shown by fold-change analysis (Tables S1and S4) and SAM analysis (Tables S13 and S16). MDM2 is a known inhibitor of the tumor suppressor p53; it acts as an E3 ligase that promotes p53 degradation through a ubiquitin-dependent pathway [29]. AML1-ETO upregulated MDM2 by 7–9 fold in two independent microarray experiments (Table S1). This was verified by quantitative RT-PCR, which showed 5.7-fold upregulation (Fig. 4A). Immunoblotting showed an increase in MDM2 protein levels 7 h after transduction with AML1-ETO, ranging from 1.6- to 2-fold over control (p<0.05) (Fig. 4B, and 4C).

To determine whether upregulation of MDM2 plays a role in leukemic transformation by AML1-ETO, we asked whether MDM2 inhibition can counteract the transforming effects of AML1-ETO. Nutlin-3, a small molecule antagonist of MDM2, was recently developed as a potent and specific inhibitor of the MDM2-p53 interaction leading to cell cycle arrest, apoptosis, and growth inhibition of human tumor xenografts in nude mice [30], [31]. It was therefore important to determine whether nutlin-3 can counteract the ability of AML1-ETO to induce proliferation and self-renewal in primary human CD34+ cells. On the other hand, while PML-RARA also upregulated MDM2, it did not increase cell proliferation or self-renewal (Fig. 2); therefore there is no reliable assay to determine the effect of nutlin-3 on cell transformation by PML-RARA.

**Figure 5:**
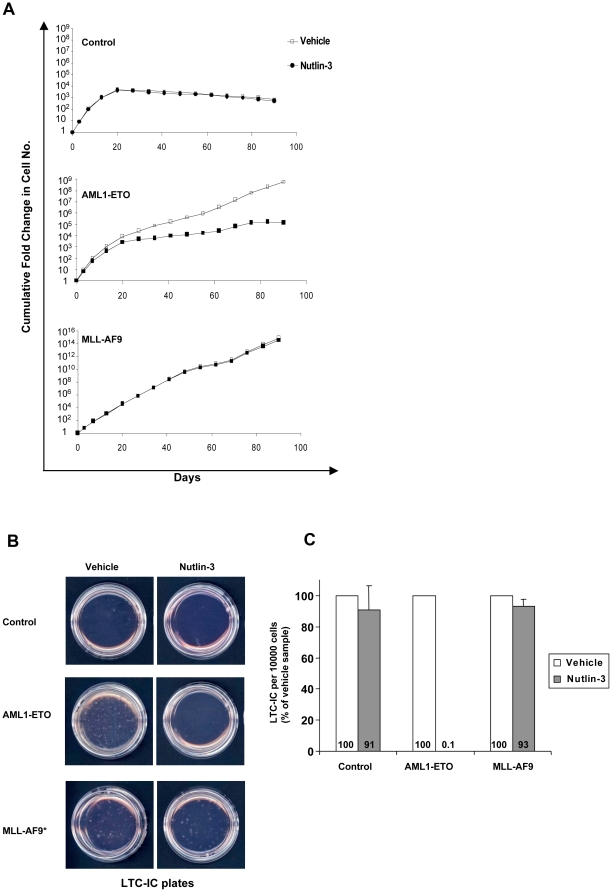
Nutlin-3 specifically inhibits the proliferation and self-renewal of primary human CD34+ cells expressing AML1-ETO. (**A**) Liquid culture of primary human CD34+ cells transduced with empty vector or vector expressing AML1-ETO or MLL-AF9, in the presence or absence of nutlin-3. Data are representative of 3 independent experiments. (**B**) LTC-IC assays of primary human CD34+ cells transduced with empty vector or vector expressing AML1-ETO or MLL-AF9, in the presence or absence of nutlin-3. Shown are representative LTC-IC plates from 3 independent experiments. * Due to the much larger number of LTC-ICs in the MLL-AF9 sample, the MLL-AF9 plates were seeded with only 1/10 of the sample in order to allow the visualization of discrete colonies. (**C**) Average numbers of LTC-ICs as percentage of vehicle sample from 3 separate experiments are shown.

We tested the effects of nutlin-3 on proliferation in liquid culture and LTC-IC numbers induced by AML1-ETO in primary human CD34+ cells. As a control, primary human CD34+ cells transduced with either empty retrovirus or with retrovirus expressing MLL-AF9 were similarly treated with nutlin-3. The results show that nutlin-3 has a marked antiproliferative effect in cells expressing AML1-ETO while it has no significant effect on untransformed cells that express empty vector or cells transformed by MLL-AF9, which does not induce MDM2 (Fig. 5A). Even more remarkable, nutlin-3 abolished the ability of AML1-ETO to increase the numbers of LTC-ICs whereas it had no significant effect on cells transduced with empty vector or those expressing MLL-AF9 (Fig. 5B, C). These data show a specific inhibitory effect of nutlin-3 on the proliferation and self-renewal of cells expressing AML1-ETO and suggest that upregulation of MDM2 may play a significant role in cell transformation by AML1-ETO.

## Discussion

There are clear differences as well as similarities in clinical behavior, pathological findings, and response to treatment among the different categories of AML. Here we show that these similarities and differences among AML oncogenes can be reflected in their transforming effects on primary human CD34+ cells *in vitro*; and we combine this approach with early gene expression profiling to identify MDM2 as a possible mediator and drug target in AML1-ETO leukemogenesis.

Studies of the *in vitro* leukemic transformation of primary human hematopoietic cells by AML fusion oncogenes are not many. Yet there has been a great deal of variability in the techniques and conditions used to study the different oncogenes [15], [16], [17], [19], [20], [21]. Variables have included: a) Source of human cells: cord blood versus peripheral blood mobilized CD34+ cells. b) FACS sorting of the transduced cells versus bulk culture prior to analysis; c) Different cytokines and/or different cytokine concentrations used to supplement culture media; and d) Different assays for proliferation, self-renewal and differentiation. Not surprisingly, the results have been difficult to compare and sometimes contradictory. For example, our finding that AML1-ETO has little effect on myeloid differentiation differs from recently published data indicating that AML1-ETO blocks myeloid differentiation *in vitro* [16], [32]. These studies differ from ours in several respects including the use of cord blood cells, liquid as opposed to semi-solid culture media, different cytokine conditions, and different durations of culture. The effect of differences in cytokine conditions are well illustrated by a study on the effects of PML-RARA on primary human hematopoietic cells [15]. In that study, a limited set of cytokines was used in serum-free media, and cell numbers were monitored over a period of approximately 2 weeks. It was shown that reducing the cytokine concentration accentuated the difference in survival and proliferation between control cells and cells expressing PML-RARA over this time period.

In this study we used identical conditions to compare the effects of 4 representative oncogenes on the differentiation, proliferation, and self-renewal of primary human CD34+ cells. The differentiation data were particularly interesting: both NUP98-HOXA9 and MLL-AF9 caused a profound disruption of myeloid and erythroid differentiation with increased numbers of erythroid cells. On the other hand, AML1-ETO and PML-RARA had no effect on erythroid maturation at the cellular level and only a modest effect on myeloid maturation. It is interesting to note that NUP98 and MLL gene rearrangements are associated with myelodysplasia, a condition that is often associated with erythroid hyperplasia and abnormal erythroid maturation [33]. These data show that the clinical similarities between NUP98 and MLL gene rearrangements are reflected in similar *in vitro* modes of transformation. One possible mechanism that may account for the similarity between NUP98 and MLL rearrangements is the overexpression of homeobox transcription factors, which could also explain the poor clinical outcome of AML with NUP98 and MLL gene rearrangements [34], [35], [36].

The oncogenes we tested caused long-term proliferation and increased self-renewal of primary human CD34+ cells, with the exception of PML-RARA. There is evidence that the ability of PML-RARA to cause leukemia in mice is inversely proportional to its level of expression [37]. There is also evidence that reducing the number and concentrations of cytokines can enhance the short-term proliferation of primary human hematopoietic cells expressing PML-RARA [15]. Future experiments directed at fine-tuning of the expression level of PML-RARA and the cytokine conditions may produce long-term growth of primary human cells and enable the testing of potential drug candidates; however, for the purposes of the current study, uniform conditions were of primary importance in order to compare the effects of the different oncogenes.

A few reports are available on gene expression profiling of *in vitro* transformed primary human hematopoietic cells [19], [20], [21], [23]. These were typically performed at least a few days if not weeks or months after retroviral introduction of the oncogene. Therefore the changes observed may reflect the particular differentiation state of the transformed cells rather than the initial transforming events. Several reports are available comparing the gene expression profiles of leukemic cells from AML patients [38], [39], [40], [41], [42], [43], [44]. However, while the bulk of the leukemic cells consist of blasts, proliferation of the tumor is driven by a small population of leukemic stem cells [45]. Therefore gene expression profiling of patient material may not reflect gene expression in leukemic stem cells or the early events in leukemic transformation. We reasoned that the initial effects of oncogenes are likely to confer the proliferation advantage that leads to leukemia, and sought to identify those early effects using nucleofection to identify deregulated genes in primary human CD34+ cells within 6 h. In addition, we used traditional retroviral transduction techniques to identify genes deregulated at later time points. Our results confirmed some of what was known about AML oncogenes. For example, the vast majority of gene deregulation by AML1-ETO and PML-RARA occurred at the 6 h time point, consistent with their known role as direct transcriptional regulators. Further, these two oncogenes had predominantly repressive effects on gene transcription, consistent with their known roles as transcriptional repressors [3], [27]. In addition, our microarrays showed overexpression of homeobox genes in primary human CD34+ cells expressing MLL-AF9, consistent with previous results showing overexpression of these genes in both mouse and human leukemias with MLL fusions [46], [47]. On the other hand, many of the deregulated genes we identified have not been previously identified in molecular signatures from patients with the corresponding oncogene [38], [39], [40], [41], [42], [43], [44]. In particular, one of the genes upregulated at the 6 h time point by AML1-ETO and PML-RARA was the p53 inhibitor *MDM2*. While MDM2 is known to be upregulated in many human cancers [48], it has not been previously shown to be upregulated in leukemic patients with either AML1-ETO or PML-RARA [38], [39], [40], [41], [42], [43], [44].

The role of the p53 tumor suppressor pathway in AML1-ETO leukemogenesis is not well understood. Leukemic cells from patients with AML1-ETO usually have wild type p53 [49], and there is evidence for p53 activation in leukemic cells with AML1-ETO [23]. These data raise the question of how the p53 pathway is suppressed during AML1-ETO leukemogenesis. The p53 pathway is regulated by an interplay between MDM2 and p14(ARF): MDM2 is an E3 ligase that mediates the degradation of p53, while p14(ARF) inhibits MDM2 and therefore stabilizes p53 [29], [50], [51], [52]. One mechanism by which AML1-ETO suppresses the p53 pathway is by downregulating p14 (ARF) [53]. Our data suggest that early upregulation of MDM2 is another possible mechanism by which AML1-ETO overcomes the p53 tumor suppressor pathway, and show that the MDM2 inhibitor nutlin-3 causes a specific inhibition of the proliferation and self-renewal of cells transformed by AML1-ETO. A possible role for nutlin-3 in the treatment of AML1-ETO leukemias is further supported by the finding that nutlin-3 induced apoptosis in leukemic blasts from patients with AML1-ETO [54], [55]. However, it is possible that the inhibitory effects of nutlin-3 on cells expressing AML1-ETO may have mechanisms other than inhibition of MDM2. It would be of interest to determine the effects of MDM2 knockdown on proliferation and LTC-IC numbers in cells expressing AML1-ETO. MDM2 overexpression as a mechanism of p53 inactivation has been described in several hematopoietic and non-hematopoietic neoplasms [48]. However, the mechanisms by which hematopoietic neoplasms upregulate MDM2 are largely unknown, and the mechanism by which AML1-ETO induces MDM2 expression also remains to be determined. MDM2 is known to be induced by p53 [52], and *in vitro* data suggest that the p53 pathway is activated by AML1-ETO in primary human CD34+ cells [23]. However, this occurs as a late event during culture secondary to accumulating DNA damage [23]; it is therefore unlikely that the early MDM2 upregulation by AML1-ETO is secondary to p53 activation.

MDM2 was also upregulated by PML-RARA; however, there was no robust assay to determine whether MDM2 inhibition has an effect on the transformation of human cells by PML-RARA because PML-RARA did not induce a significant increase in cell proliferation and self-renewal.

To identify additional pathways deregulated by AML oncogenes we analyzed the gene lists generated by SAM (Tables S13, S14, S15, S16, S17, S18, S19, S20, S21, S22, S23 and S24) using GeneGo software. For each oncogene, a list representing the union of all 3 time points was analyzed by GeneGo and the top 10 deregulated pathways based on enrichment score were identified (Table S25). Interestingly, for all 4 oncogenes, the top 10 deregulated pathways included the TGF and WNT cytoskeletal remodeling pathway and the epithelial-to-mesenchymal transition (EMT) pathway. The TGF-β and WNT signaling pathways are well known to be involved in the pathogenesis of malignancies, including AML [56], [57], [58]. On the other hand, the EMT pathway is important during development, and has a well-established role in tumor progression and metastasis, particularly in carcinomas [59], [60], but not in hematopoietic malignancy. Our data suggest a novel role for the EMT pathway in the pathogenesis of AML.

In summary, our data illustrate the potential of studying the effects of AML oncogenes on primary human CD34+ cells using a combination of early gene expression profiling with *in vitro* assays of proliferation and differentiation. The former can suggest possible drug targets, while the latter can be used as assays to measure the effects of potential drugs. MLL and NUP98 gene rearrangements share a poor prognosis in patients with AML and a fairly specific set of *in vitro* effects on primary human CD34+ cells, most prominent among which is their profound effect on the number and differentiation of erythroid precursors. It would be of interest to determine whether drugs that abrogate these effects *in vitro* would be useful clinically in overcoming the resistance of these types of AML to therapy.

## Materials and Methods

### Plasmid Construction

The AML1-ETO cDNA, obtained from pHR'-PGK-ETO-EGFP (kindly provided by Drs. S.D. Nimer and J.C. Mulloy) with *Kpn*I/*Age*I, was subcloned into the *Kpn*I/*Eco*RV sites of pcDNA3.1Hygro(+). PML-RARA and MLL-AF9 cDNAs were kindly provided by Dr. T.J. Ley and Dr. J.L. Hess, respectively. The cDNAs were subcloned into *Hpa*I of MSCV-IRES-GFP, and into pTracer-CMV/Bsd using *Nhe*I/*Eco*RI (AML1-ETO) *Not*I (PML-RARA) and *Eco*RV (MLL-AF9). HA-tagged NUP98-HOXA9 in MSCV-IRES-GFP and pTracer-CMV/Bsd were previously described [20].

### Retrovirus production and transduction

NUP98-HOXA9-expressing retrovirus was previously described [20]. Other retrovirus stocks were generated by transfecting GP293 packaging cells with 4.4 µg retroviral vector and 1.1 µg pVSV-G expression vector using Lipofectamine Plus reagent (Invitrogen). After 48 h, supernatant containing VSV-G-pseudotyped retrovirus was used to transfect PG13 packaging cells by spinoculation with 8 µg/mL hexadimethrine bromide (Sigma-Aldrich) to produce stable cell lines producing GaLV-pseudotyped retrovirus. Primary human CD34+ cells from mobilized peripheral blood of normal donors were purchased from the Fred Hutchinson Cancer Research Center or obtained from deceased autologous transplant patients from the Bone Marrow Transplant Laboratory at Northwestern Memorial Hospital after Institutional Review Board approval. For each experiment all 4 oncogenes and controls were tested using the same cell source. Cells were pre-activated and transduced with retrovirus as previously described [20]. After 48 h, GFP positive cells were sorted using a MoFlo high-speed sorter (Dako).

### Nucleofection

Preactivated primary human CD34+ cells were nucleofected with pTracer-CMV/Bsd constructs expressing the fusion proteins or empty vector as described [20]. Cells were cultured at 2x10^5^/mL in complete cytokine medium [20] for 3.5 h. GFP+ cells were sorted using MoFlo sorter and cultured at 10^5^/mL in complete cytokine medium for 2.5 h before RNA isolation.

### Immunolotting

Total cell lysates were checked for protein expression by immunoblotting using: anti-HA (12CA5) (Roche) for NUP98-HOX9; anti-AML-1 (Ab-2) (EMD Chemicals) for AML1-ETO; anti-MLL/HRX clone N4.4, (Millipore) for MLL-AF9; anti RARA (C-20) for PML-RARA and anti-MDM2 (N-20) for MDM2 (Santa Cruz Biotechnology). MDM2 protein was quantified using the ChemiDoc XRS imaging system (Bio-Rad) with Quantity One 1-D software and fold change over control was calculated.

### Analysis of differentiation

Colony-forming cell (CFC) assays were performed as previously described [20]. Briefly, 1000 sorted cells were seeded into each of two duplicate plates; after 14 days, colonies were counted and cells were collected. Giemsa-stained Cytospin slides were prepared and 500-cell differential counts were performed using an Olympus BX51 microscope. Cells were divided into five categories: primitive cells include blasts and promyelocytes; intermediate myeloid cells include myelocytes/metamyelocytes; mature myeloid cells include bands, segmented neutrophils, monocytes, and macrophages; intermediate erythroid cells include cells with intermediate hemoglobinization; and mature erythroid cells include cells with full hemoglobinization. Photomicrographs were taken with an Olympus DP71 camera with a 60X oil objective. Flow cytometry was performed on a FACSCalibur flow cytometer (BD Biosciences), and analyzed using FlowJO v7.5 (Tree Star) software. Antibodies used were: CD11b (phycoerythrin-conjugated clone D12); CD235a (allophycocyanin-conjugated clone GA-R2) and CD71 (phycoerythrin-conjugated clone M-A712) from BD; CD33 (allophycocyanin-conjugated clone D3HL60.251) and CD45 (phycoerythrin-Cy7-conjugated clone J.33) from Beckman Coulter.

### Liquid culture, long-term culture-initiating cell (LTC-IC) assays, and nutlin-3 treatment

Liquid culture was carried out in Iscove's modified Dulbecco's medium (IMDM) containing 20% FBS, 100 ng/ml Fms-related tyrosine kinase 3 (Flt 3)-ligand, 20 ng/ml granulocyte/macrophage colony-stimulating factor (GM-CSF), 100 ng/ml stem cell factor (SCF), 100 ng/ml thrombopoietin (TPO), 50 ng/ml IL-3, 100 ng/ml IL-6 (all cytokines were from Peprotech, Rocky Hill, NJ, USA), 2 mM L-glutamine, and 100 units/ml penicillin/streptomycin as previously described [20]. LTC-IC assays were performed as described [20] except that cells transduced with MLL-AF9 were seeded at 1/10 cell density in methylcellulose for LTC-IC due to their high number of LTC-ICs. Nutlin-3, (Cayman Chemical, Ann Arbor, Michigan, USA), was dissolved in DMSO at 10 mM and stored at −20°C. Cells in long-term liquid culture were exposed to 1 µM nutlin-3 or vehicle during each round of feeding. LTC-IC plates were exposed to 2 µM nutlin-3 or vehicle weekly.

### Quantitative real-time PCR

Template cDNA was synthesized using SuperScript III First-Strand Synthesis System (Invitrogen). Real-time PCR was performed in triplicate using the StepOne Plus Real-Time PCR System and analyzed with the StepOne software version 2 (Applied Biosystems) with the following two primer pairs:

For MDM2: 5′GCCTTAGTGAAGAAGGACAAG3′

5′TCCCTTTATCTTCAGGAAGCC3′

For GAPDH: 5′GGACCTGACCTGCCG3′

5′TAGCCCAGGATGCCCTTGAG3′.

The expression level of MDM2 was normalized to GAPDH.

### Microarray analysis of primary human CD34+ cells

GFP-sorted cells were collected and snap-frozen 6 h after nucleofection, or 3 and 8 days after retroviral transduction. Total RNA isolated using the RNeasy mini kit (Qiagen) was submitted to the Siteman Cancer Center Laboratory for Clinical Genomics. Target was prepared using linear amplification protocol for the 6 h time point and standard protocol for the 3 and 8 day time points and hybridized to Affymetrix HG-U133 Plus 2.0 GeneChip arrays according to manufacturer's instructions. The experiment was performed two independent times. Microarray data were merged with updated gene annotation data using DecisionSite for Functional Genomics software (Spotfire) 9.1.1. Probesets scored as absent across all chips were filtered out. Fold change was calculated by dividing the absolute signal intensity from oncogene samples by that of the control. Probesets scored as increasing and absent in the numerator, or decreasing and absent in the denominator were filtered out. Probesets induced or repressed by twofold or greater relative to control in both replicates were considered differentially expressed. Data filtering was done using Spotfire 9.1.1. For Significance Analysis for Microarrays (SAM) [28], the absolute signal intensities of the genes induced by each oncogene and the control at each time point were first filtered as described above. The log base 2 of the absolute signal intensities was computed, and assembled into a single tab-delimited file for each comparison. SAM was run with the maximum number of unique permutations available using a two-class unpaired setting, and delta values were chosen to give a median false discovery rate (FDR) of 10% or lower, with the exception of the PML-RARA 3-day time point where the lowest possible FDR was 10.3%. All other parameters were set to the defaults. An arbitrary 2-fold cutoff threshold was then applied to the lists of significant genes from the SAM analysis. The resulting list of statistically significant probes was merged with the updated gene annotation data for each probe set on the array using Spotfire 9.1.1. For each oncogene, a union list of deregulated genes at all time points was generated and analyzed using MetaCore™ version 6.3 build 25177 (GeneGo, Inc.). The top 10 significant pathways at an FDR threshold of 0.05 were chosen based on enrichment score for each of the oncogenes.

## Supporting Information

Table S1 Genes deregulated by AML1-ETO 6 hours after transfection. Primary human CD34+ cells were nucleofected with either control pTracer-CMV/Bsd vector or vector expressing AML1-ETO and sorted for GFP positivity. Total RNA was extracted 6 h after nucleofection and subjected to microarray analysis. Genes that showed up- or down-regulation by 2 fold or more in comparison to the control in 2 independent experiments (Exp.1 and Exp.2) were considered deregulated.
(0.36 MB PDF)

Table S2 Genes deregulated by AML1-ETO 3 days after transduction. Primary human CD34+ cells were retrovirally transduced with either control MSCV-IRES-GFP vector or vector expressing AML1-ETO and sorted for GFP positivity. Total RNA was extracted 3 days after transduction and subjected to microarray analysis. Genes that showed up- or down-regulation by 2 fold or more in comparison to the control in 2 independent experiments (Exp.1 and Exp.2) were considered deregulated.
(0.10 MB PDF)

Table S3 Genes deregulated by AML1-ETO 8 days after transduction. Primary human CD34+ cells were retrovirally transduced with either control MSCV-IRES-GFP vector or vector expressing AML1-ETO and sorted for GFP positivity. Total RNA was extracted 8 days after transduction and subjected to microarray analysis. Genes that showed up- or down-regulation by 2 fold or more in comparison to the control in 2 independent experiments (Exp.1 and Exp.2) were considered deregulated.
(0.08 MB PDF)

Table S4 Genes deregulated by PML-RARA 6 h after transfection. Primary human CD34+ cells were nucleofected with either control pTracer-CMV/Bsd vector or vector expressing PML-RARA and sorted for GFP positivity. Total RNA was extracted 6 h after nucleofection and subjected to microarray analysis. Genes that showed up- or down-regulation by 2 fold or more in comparison to the control in 2 independent experiments (Exp.1 and Exp.2) were considered deregulated.
(0.18 MB PDF)

Table S5 Genes deregulated by PML-RARA 3 days after transduction. Primary human CD34+ cells were retrovirally transduced with either control MSCV-IRES-GFP vector or vector expressing PML-RARA and sorted for GFP positivity. Total RNA was extracted 3 days after transduction and subjected to microarray analysis. Genes that showed up- or down-regulation by 2 fold or more in comparison to the control in 2 independent experiments (Exp.1 and Exp.2) were considered deregulated.
(0.08 MB PDF)

Table S6 Genes deregulated by PML-RARA 8 days after transduction. Primary human CD34+ cells were retrovirally transduced with either control MSCV-IRES-GFP vector or vector expressing PML-RARA and sorted for GFP positivity. Total RNA was extracted 8 days after transduction and subjected to microarray analysis. Genes that showed up- or down-regulation by 2 fold or more in comparison to the control in 2 independent experiments (Exp.1 and Exp.2) were considered deregulated.
(0.08 MB PDF)

Table S7 Genes deregulated by MLL-AF9 6 h after transfection. Primary human CD34+ cells were nucleofected with either control pTracer-CMV/Bsd vector or vector expressing MLL-AF9 and sorted for GFP positivity. Total RNA was extracted 6 h after nucleofection and subjected to microarray analysis. Genes that showed up- or down-regulation by 2 fold or more in comparison to the control in 2 independent experiments (Exp.1 and Exp.2) were considered deregulated.
(0.08 MB PDF)

Table S8 Genes deregulated by MLL-AF9 3 days after transduction. Primary human CD34+ cells were retrovirally transduced with either control MSCV-IRES-GFP vector or vector expressing MLL-AF9 and sorted for GFP positivity. Total RNA was extracted 3 days after transduction and subjected to microarray analysis. Genes that showed up- or down-regulation by 2 fold or more in comparison to the control in 2 independent experiments (Exp.1 and Exp.2) were considered deregulated.
(0.11 MB PDF)

Table S9 Genes deregulated by MLL-AF9 8 days after transduction. Primary human CD34+ cells were retrovirally transduced with either control MSCV-IRES-GFP vector or vector expressing MLL-AF9 and sorted for GFP positivity. Total RNA was extracted 8 days after transduction and subjected to microarray analysis. Genes that showed up- or down-regulation by 2 fold or more in comparison to the control in 2 independent experiments (Exp.1 and Exp.2) were considered deregulated.
(0.10 MB PDF)

Table S10 Genes deregulated by NUP98-HOXA9 6 h after transfection. Primary human CD34+ cells were nucleofected with either control pTracer-CMV/Bsd vector or vector expressing NUP98-HOXA9 and sorted for GFP positivity. Total RNA was extracted 6 h after nucleofection and subjected to microarray analysis. Genes that showed up- or down-regulation by 2 fold or more in comparison to the control in 2 independent experiments (Exp.1 and Exp.2) were considered deregulated.
(0.11 MB PDF)

Table S11 Genes deregulated by NUP98-HOXA9 3 days after transduction. Primary human CD34+ cells were retrovirally transduced with either control MSCV-IRES-GFP vector or vector expressing NUP98-HOXA9 and sorted for GFP positivity. Total RNA was extracted 3 days after transduction and subjected to microarray analysis. Genes that showed up- or down-regulation by 2 fold or more in comparison to the control in 2 independent experiments (Exp.1 and Exp.2) were considered deregulated.
(0.05 MB PDF)

Table S12 Genes deregulated by NUP98-HOXA9 8 days after transduction. Primary human CD34+ cells were retrovirally transduced with either control MSCV-IRES-GFP vector or vector expressing NUP98-HOXA9 and sorted for GFP positivity. Total RNA was extracted 8 days after transduction and subjected to microarray analysis. Genes that showed up- or down-regulation by 2 fold or more in comparison to the control in 2 independent experiments (Exp.1 and Exp.2) were considered deregulated.
(0.11 MB PDF)

Table S13 Genes deregulated by AML1-ETO 6 hours after transfection. Primary human CD34+ cells were nucleofected with either control pTracer-CMV/Bsd vector or vector expressing AML1-ETO and sorted for GFP positivity. Total RNA was extracted 6 h after nucleofection and subjected to microarray analysis. Microarray data were analyzed by SAM as described in Materials and Methods. Significantly deregulated genes are listed and the false discovery rate (FDR) is shown.
(0.79 MB PDF)

Table S14 Genes deregulated by AML1-ETO 3 days after transduction. Primary human CD34+ cells were retrovirally transduced with either control MSCV-IRES-GFP vector or vector expressing AML1-ETO and sorted for GFP positivity. Total RNA was extracted 3 days after transduction and subjected to microarray analysis. Microarray data were analyzed by SAM as described in Materials and Methods. Significantly deregulated genes are listed and the false discovery rate (FDR) is shown.
(0.06 MB PDF)

Table S15 Genes deregulated by AML1-ETO 8 days after transduction. Primary human CD34+ cells were retrovirally transduced with either control MSCV-IRES-GFP vector or vector expressing AML1-ETO and sorted for GFP positivity. Total RNA was extracted 8 days after transduction and subjected to microarray analysis. Microarray data were analyzed by SAM as described in Materials and Methods. Significantly deregulated genes are listed and the false discovery rate (FDR) is shown.
(0.15 MB PDF)

Table S16 Genes deregulated by PML-RARA 6 h after transfection. Primary human CD34+ cells were nucleofected with either control pTracer-CMV/Bsd vector or vector expressing PML-RARA and sorted for GFP positivity. Total RNA was extracted 6 h after nucleofection and subjected to microarray analysis. Microarray data were analyzed by SAM as described in Materials and Methods. Significantly deregulated genes are listed and the false discovery rate (FDR) is shown.
(0.40 MB PDF)

Table S17 Genes deregulated by PML-RARA 3 days after transduction. Primary human CD34+ cells were retrovirally transduced with either control MSCV-IRES-GFP vector or vector expressing PML-RARA and sorted for GFP positivity. Total RNA was extracted 3 days after transduction and subjected to microarray analysis. Microarray data were analyzed by SAM as described in Materials and Methods. Significantly deregulated genes are listed and the false discovery rate (FDR) is shown.
(0.14 MB PDF)

Table S18 Genes deregulated by PML-RARA 8 days after transduction. Primary human CD34+ cells were retrovirally transduced with either control MSCV-IRES-GFP vector or vector expressing PML-RARA and sorted for GFP positivity. Total RNA was extracted 8 days after transduction and subjected to microarray analysis. Microarray data were analyzed by SAM as described in Materials and Methods. Significantly deregulated genes are listed and the false discovery rate (FDR) is shown.
(0.19 MB PDF)

Table S19 Genes deregulated by MLL-AF9 6 h after transfection. Primary human CD34+ cells were nucleofected with either control pTracer-CMV/Bsd vector or vector expressing MLL-AF9 and sorted for GFP positivity. Total RNA was extracted 6 h after nucleofection and subjected to microarray analysis. Microarray data were analyzed by SAM as described in Materials and Methods. Significantly deregulated genes are listed and the false discovery rate (FDR) is shown.
(0.26 MB PDF)

Table S20 Genes deregulated by MLL-AF9 3 days after transduction. Primary human CD34+ cells were retrovirally transduced with either control MSCV-IRES-GFP vector or vector expressing MLL-AF9 and sorted for GFP positivity. Total RNA was extracted 3 days after transduction and subjected to microarray analysis. Microarray data were analyzed by SAM as described in Materials and Methods. Significantly deregulated genes are listed and the false discovery rate (FDR) is shown.
(0.35 MB PDF)

Table S21 Genes deregulated by MLL-AF9 8 days after transduction. Primary human CD34+ cells were retrovirally transduced with either control MSCV-IRES-GFP vector or vector expressing MLL-AF9 and sorted for GFP positivity. Total RNA was extracted 8 days after transduction and subjected to microarray analysis. Microarray data were analyzed by SAM as described in Materials and Methods. Significantly deregulated genes are listed and the false discovery rate (FDR) is shown.
(0.27 MB PDF)

Table S22 Genes deregulated by NUP98-HOXA9 6 h after transfection. Primary human CD34+ cells were nucleofected with either control pTracer-CMV/Bsd vector or vector expressing NUP98-HOXA9 and sorted for GFP positivity. Total RNA was extracted 6 h after nucleofection and subjected to microarray analysis. Microarray data were analyzed by SAM as described in Materials and Methods. Significantly deregulated genes are listed and the false discovery rate (FDR) is shown.
(0.30 MB PDF)

Table S23 Genes deregulated by NUP98-HOXA9 3 days after transduction. Primary human CD34+ cells were retrovirally transduced with either control MSCV-IRES-GFP vector or vector expressing NUP98-HOXA9 and sorted for GFP positivity. Total RNA was extracted 3 days after transduction and subjected to microarray analysis. Microarray data were analyzed by SAM as described in Materials and Methods. Significantly deregulated genes are listed and the false discovery rate (FDR) is shown.
(0.21 MB PDF)

Table S24 Genes deregulated by NUP98-HOXA9 8 days after transduction. Primary human CD34+ cells were retrovirally transduced with either control MSCV-IRES-GFP vector or vector expressing NUP98-HOXA9 and sorted for GFP positivity. Total RNA was extracted 8 days after transduction and subjected to microarray analysis. Microarray data were analyzed by SAM as described in Materials and Methods. Significantly deregulated genes are listed and the false discovery rate (FDR) is shown.
(0.25 MB PDF)

Table S25 Union lists for all 3 time points for each oncogene were generated from the lists shown in Tables S13 - S24 and subjected to GeneGo analysis as described in Materials and Methods. The top 10 enriched pathways for each oncogene are shown along with the corresponding p-values.
(0.05 MB PDF)
