# Supplementary material for: In Vitro Transformation of Primary Human CD34+ Cells by AML Fusion Oncogenes: Early Gene Expression Profiling Reveals Possible Drug Target in AML
Source: PLoS One. 2010 Aug 27;5(8):e12464. doi: 10.1371/journal.pone.0012464 (PMC2929205; doi:10.1371/journal.pone.0012464)
Supplement: Table S1 — Genes deregulated by AML1-ETO 6 hours after transfection. Primary human CD34+ cells were nucleofected with either control pTracer-CMV/Bsd vector or vector expressing AML1-ETO and sorted for GFP positivity. Total RNA was extracted 6 h after nucleofection and subjected to microarray analysis. Genes that showed up- or down-regulation by 2 fold or more in comparison to the control in 2 independent experiments (Exp.1 and Exp.2) were considered deregulated. (0.36 MB PDF) [file pone.0012464.s001.pdf]

Table S1. Genes deregulated by AML1-ETO at 6 h after transfection

Probe set ID	Fold Change		Gene Name	Gene Symbol
	Exp.1	Exp.2		
216831_s_at	19827.19	955.64	runt-related transcription factor 1; translocated to, 1 (cyclin D-related)	RUNX1T1
211181_x_at	16.10	112.28	runt-related transcription factor 1 (acute myeloid leukemia 1; aml1 oncogene)	RUNX1
1560012_at	2.18	95.21		
228547_at	4.30	50.96	neurexin 1	NRXN1
204939_s_at	27.72	49.06	phospholamban	PLN
1563053_at	37.69	47.08		
205943_at	152.82	38.18	tryptophan 2,3-dioxygenase	TDO2
235490_at	2.75	35.60	transmembrane protein 107	TMEM107
1560676_at	4.02	33.91		
1553851_at	26.48	30.70	Spi-C transcription factor (Spi-1/PU.1 related)	SPIC
233021_at	4.42	29.77		
1561657_at	2.09	27.50		
222456_s_at	2.97	27.03	LIM domain and actin binding 1	LIMA1
205513_at	9.21	25.98	transcobalamin I (vitamin B12 binding protein, R binder family)	TCN1
223962_at	4.43	25.57	tetratricopeptide repeat domain 29	TTC29
228202_at	88.20	24.93	phospholamban	PLN
210650_s_at	7.11	24.77	piccolo (presynaptic cytomatrix protein)	PCLO
219936_s_at	30.44	24.73	G protein-coupled receptor 87	GPR87
206030_at	12.87	24.28	aspartoacylase (Canavan disease)	ASPA
227006_at	6.27	23.65	protein phosphatase 1, regulatory (inhibitor) subunit 14A	PPP1R14A
1557733_a_at	2.19	23.06		
240317_at	12.12	21.69	protocadherin beta 4	PCDHB4
204938_s_at	49.40	19.62	phospholamban	PLN
239947_at	26.89	18.37		
236847_at	2.59	18.20	chromosome 19 open reading frame 18	C19orf18
223279_s_at	10.25	17.41	uveal autoantigen with coiled-coil domains and ankyrin repeats	UACA
203038_at	2.68	15.60	protein tyrosine phosphatase, receptor type, K	PTPRK
219106_s_at	19.00	14.82	kelch repeat and BTB (POZ) domain containing 10	KBTBD10
1565614_at	3.76	14.55	zinc finger protein 337	ZNF337
1560746_at	6.54	14.47	chromosome 1 open reading frame 118	C1orf118
243581_at	3.39	14.40		
226534_at	26.11	14.40	KIT ligand	KITLG
210119_at	8.77	13.80	potassium inwardly-rectifying channel, subfamily J, member 15	KCNJ15
216456_at	2.93	13.47		

1565602_at	2.45	13.36		
243401_at	22.09	13.12	formin-like 2	FMNL2
230130_at	3.37	12.27	slit homolog 2 (Drosophila)	SLIT2
211620_x_at	9.98	12.18	runt-related transcription factor 1 (acute myeloid leukemia 1; aml1 oncogene)	RUNX1
214079_at	26.51	11.64	dehydrogenase/reductase (SDR family) member 2	DHRS2
237070_at	2.69	11.60	transient receptor potential cation channel, subfamily M, member 1	TRPM1
243602_at	7.27	11.44		
238844_s_at	3.46	11.42	nephronophthisis 1 (juvenile)	NPHP1
232881_at	4.72	10.66		
207932_at	6.04	10.53	interferon, alpha 8	IFNA8
228964_at	2.33	10.09	PR domain containing 1, with ZNF domain	PRDM1
215300_s_at	4.34	10.02	flavin containing monooxygenase 5	FMO5
215318_at	2.42	9.93		
210118_s_at	2.28	9.93	interleukin 1, alpha	IL1A
237663_at	2.34	9.87		
235355_at	306.27	9.77		
206381_at	27.42	9.54	sodium channel, voltage-gated, type II, alpha subunit	SCN2A
1553506_at	3.80	9.46	carboxypeptidase O	CPO
244334_at	12.27	9.36	translocation associated membrane protein 1-like 1	TRAM1L1
205844_at	12.36	9.32	vanin 1	VNN1
220311_at	12.95	9.20	N-6 adenine-specific DNA methyltransferase 1 (putative)	N6AMT1
211832_s_at	7.15	9.20	Mdm2, transformed 3T3 cell double minute 2, p53 binding protein (mouse)	MDM2
239203_at	13.55	9.13		
240465_at	2.75	8.95		
223799_at	2.74	8.89	KIAA1826	KIAA1826
201939_at	17.84	8.77	polo-like kinase 2 (Drosophila)	PLK2
207813_s_at	10.43	8.77	ferredoxin reductase	FDXR
235944_at	12.69	8.76	hemicentin 1	HMCN1
243272_at	4.47	8.64		
223395_at	102.06	8.63	ABI gene family, member 3 (NESH) binding protein	ABI3BP
240890_at	10.63	8.46		
1552694_at	5.75	8.34	solute carrier family 2 (facilitated glucose transporter), member 13	SLC2A13
214148_at	2.04	8.25		
232295_at	2.15	8.20	G elongation factor, mitochondrial 1	GFM1
206417_at	2.56	7.86	cyclic nucleotide gated channel alpha 1	CNGA1
243222_at	4.02	7.86		
220115_s_at	5.54	7.66	cadherin 10, type 2 (T2-cadherin)	CDH10
241420_at	2.23	7.52		
231260_at	4.34	7.49		
223735_at	6.14	7.46	ADP-ribosylation factor-like 6	ARL6

223557_s_at	7.08	7.25	transmembrane protein with EGF-like and two follistatin-like domains 2	TMEFF2
1552521_a_at	3.68	7.24	transmembrane protein 74	TMEM74
211182_x_at	5.54	7.24	runt-related transcription factor 1 (acute myeloid leukemia 1; aml1 oncogene)	RUNX1
1554706_at	5.06	6.87	olfactory receptor, family 2, subfamily L, member 13	OR2L13
238919_at	3.89	6.83		
206463_s_at	13.19	6.70	dehydrogenase/reductase (SDR family) member 2	DHRS2
219148_at	7.04	6.53	PDZ binding kinase	PBK
207517_at	12.90	6.51	laminin, gamma 2	LAMC2
232549_at	4.14	6.42	RNA binding motif protein 11	RBM11
1557765_at	4.12	6.31		
223204_at	8.67	6.28	chromosome 4 open reading frame 18	C4orf18
206726_at	6.85	6.23		
1558452_at	2.12	6.21	transmembrane protein 144	TMEM144
220369_at	2.81	6.15	SMEK homolog 1, suppressor of mek1 (Dictyostelium)	SMEK1
222376_at	5.98	6.15		
1570119_at	2.08	6.07	androgen-induced proliferation inhibitor	APRIN
206331_at	6.98	5.95	calcitonin receptor-like	CALCRL
241298_x_at	2.38	5.91		
212624_s_at	7.14	5.86	chimerin (chimaerin) 1	CHN1
204959_at	29.27	5.83	myeloid cell nuclear differentiation antigen	MNDA
206091_at	2.92	5.82	matrilin 3	MATN3
209841_s_at	9.04	5.77	leucine rich repeat neuronal 3	LRRN3
219738_s_at	6.19	5.76	protocadherin 9	PCDH9
238756_at	5.11	5.74		
240512_x_at	42.94	5.66	potassium channel tetramerisation domain containing 4	KCTD4
229292_at	4.94	5.52	erythrocyte membrane protein band 4.1 like 5	EPB41L5
236278_at	2.02	5.44		
203811_s_at	9.21	5.42	DnaJ (Hsp40) homolog, subfamily B, member 4	DNAJB4
1561113_at	3.74	5.39		
208173_at	7.25	5.36	interferon, beta 1, fibroblast	IFNB1
230169_at	8.69	5.33	THAP domain containing 6	THAP6
228946_at	3.83	5.28	inturned planar cell polarity effector homolog (Drosophila)	INTU
235675_at	6.31	5.26	dihydrofolate reductase-like 1	DHFRL1
219274_at	6.73	5.24	tetraspanin 12	TSPAN12
202668_at	4.44	5.24	ephrin-B2	EFNB2
210815_s_at	5.96	5.17	calcitonin receptor-like	CALCRL
212224_at	5.69	5.16	aldehyde dehydrogenase 1 family, member A1	ALDH1A1

222717_at	4.12	5.15	serum deprivation response (phosphatidylserine binding protein)	SDPR
219737_s_at	5.64	5.07	protocadherin 9	PCDH9
206254_at	27.72	5.06	epidermal growth factor (beta-urogastrone)	EGF
214534_at	2.49	5.00	histone cluster 1, H1b	HIST1H1B
243056_at	8.48	4.98	chromosome 12 open reading frame 60	C12orf60
1568615_a_at	7.99	4.96		
232695_at	6.30	4.96	kinesin family member 6	KIF6
230506_at	9.57	4.94	chromosome 6 open reading frame 164	C6orf164
229057_at	35.35	4.91	sodium channel, voltage-gated, type II, alpha subunit	SCN2A
205046_at	4.34	4.87	centromere protein E, 312kDa	CENPE
213820_s_at	5.39	4.84	START domain containing 5	STARD5
234611_at	4.50	4.83		
235846_at	4.41	4.82		
205922_at	20.32	4.79	vanin 2	VNN2
1567612_at	18.90	4.79		
215228_at	5.53	4.77	nescient helix loop helix 2	NHLH2
229975_at	3.23	4.76		
203998_s_at	3.75	4.75	synaptotagmin I	SYT1
230147_at	9.76	4.74	coagulation factor II (thrombin) receptor-like 2	F2RL2
206721_at	9.51	4.73	chromosome 1 open reading frame 114	C1orf114
227839_at	15.17	4.71	methyl-CpG binding domain protein 5	MBD5
238578_at	6.95	4.70	transmembrane protein 182	TMEM182
223838_at	15.06	4.69	testis specific, 10	TSGA10
235406_x_at	2.11	4.69		
220623_s_at	12.24	4.68	testis specific, 10	TSGA10
226436_at	5.20	4.65	Ras association (RalGDS/AF-6) domain family 4	RASSF4
209211_at	3.44	4.65	Kruppel-like factor 5 (intestinal)	KLF5
228680_at	5.64	4.65	kinesin family member 3A	KIF3A
221805_at	116.46	4.60	neurofilament, light polypeptide 68kDa	NEFL
231218_at	52.93	4.60		
239975_at	2.24	4.60		
240161_s_at	8.69	4.60	cell division cycle 20 homolog B (S. cerevisiae)	CDC20B
203810_at	6.99	4.59	DnaJ (Hsp40) homolog, subfamily B, member 4	DNAJB4
206914_at	7.22	4.56	cytotoxic and regulatory T cell molecule	CRTAM
239556_at	2.10	4.53		
212104_s_at	6.56	4.53	RNA binding motif protein 9	RBM9
207495_at	4.39	4.52	RAB28, member RAS oncogene family	RAB28
203549_s_at	2.03	4.49	lipoprotein lipase	LPL

213081_at	2.02	4.49	zinc finger and BTB domain containing 22	ZBTB22
201733_at	2.86	4.48	chloride channel 3	CLCN3
206249_at	5.05	4.46	mitogen-activated protein kinase kinase kinase 13	MAP3K13
230450_at	4.86	4.41		
239043_at	6.75	4.40	zinc finger protein 404	ZNF404
238228_at	6.41	4.40	component of oligomeric golgi complex 3	COG3
226103_at	7.22	4.39	nexilin (F actin binding protein)	NEXN
209465_x_at	10.01	4.37	pleiotrophin (heparin binding growth factor 8, neurite growth-promoting factor 1)	PTN
208121_s_at	6.51	4.36	protein tyrosine phosphatase, receptor type, O	PTPRO
1554524_a_at	4.51	4.36	olfactomedin 3	OLFM3
239944_at	3.11	4.35		
237058_x_at	8.11	4.35	solute carrier family 6 (neurotransmitter transporter, GABA), member 13	SLC6A13
206512_at	2.15	4.33	zinc finger (CCCH type), RNA-binding motif and serine/arginine rich 1	ZRSR1
213418_at	2.37	4.33	heat shock 70kDa protein 6 (HSP70B')	HSPA6
221287_at	2.46	4.30	ribonuclease L (2',5'-oligoisoadenylate synthetase-dependent)	RNASEL
227197_at	4.82	4.28		
220497_at	6.84	4.28	zinc finger protein 214	ZNF214
1553954_at	3.09	4.27	asparagine-linked glycosylation 14 homolog (S. cerevisiae)	ALG14
208484_at	12.23	4.26	histone cluster 1, H1a	HIST1H1A
206932_at	8.96	4.24	cholesterol 25-hydroxylase	CH25H
204602_at	4.48	4.24	dickkopf homolog 1 (Xenopus laevis)	DKK1
206291_at	9.68	4.22	neurotensin	NTS
225283_at	4.29	4.22	arrestin domain containing 4	ARRDC4
244786_at	4.71	4.21	small nucleolar RNA host gene (non-protein coding) 10	SNHG10
236076_at	3.31	4.20		
211538_s_at	11.98	4.20	heat shock 70kDa protein 2	HSPA2
214073_at	11.46	4.19	cortactin	CTTN
229147_at	3.89	4.18		
236634_at	3.60	4.18	chromosome 8 open reading frame 48	C8orf48
231119_at	4.63	4.18	replication factor C (activator 1) 3, 38kDa	RFC3
209840_s_at	8.62	4.17	leucine rich repeat neuronal 3	LRRN3
235461_at	2.53	4.17	KIAA1546	KIAA1546
230032_at	5.24	4.17		
1552427_at	2.84	4.16	zinc finger protein 485	ZNF485
213623_at	4.57	4.09	kinesin family member 3A	KIF3A

230708_at	6.23	4.08	prickle homolog 1 (Drosophila)	PRICKLE1
1560258_a_at	5.56	4.07		
200974_at	6.35	4.04	actin, alpha 2, smooth muscle, aorta	ACTA2
232125_at	4.77	4.04		
230789_at	4.28	4.02	suppressor of hairy wing homolog 2 (Drosophila)	SUHW2
1568627_at	4.06	4.00	SMEK homolog 2, suppressor of mek1 (Dictyostelium)	SMEK2
226341_at	3.75	4.00		
244734_at	8.99	4.00		
238488_at	6.84	3.99	importin 11	IPO11
1559322_at	6.99	3.96		
210387_at	4.25	3.96	histone cluster 1, H2bg	HIST1H2BG
232071_at	3.42	3.95	chromosome 2 open reading frame 3	C2orf3
230626_at	2.35	3.95	tetraspanin 12	TSPAN12
242178_at	5.18	3.94	lipase, member I	LIPI
231249_at	2.17	3.94		
244404_at	4.09	3.94		
1569607_s_at	4.87	3.93		
37547_at	3.61	3.93	Bardet-Biedl syndrome 9	BBS9
229989_at	2.62	3.91	asparagine-linked glycosylation 9 homolog (S. cerevisiae, alpha- 1,2-mannosyltransferase)	ALG9
244086_at	2.11	3.90	arylacetamide deacetylase-like 1	AADACL1
239825_at	11.53	3.90	activating transcription factor 6	ATF6
1555112_a_at	8.88	3.90	chromosome 1 open reading frame 114	C1orf114
213558_at	4.89	3.90	piccolo (presynaptic cytomatrix protein)	PCLO
1560017_at	3.25	3.89	transmembrane and tetratricopeptide repeat containing 3	TMTC3
209189_at	2.16	3.86	v-fos FBJ murine osteosarcoma viral oncogene homolog	FOS
242206_at	2.70	3.85		
205352_at	6.82	3.84	serpin peptidase inhibitor, clade I (neuroserpin), member 1	SERPINI1
218711_s_at	2.99	3.84	serum deprivation response (phosphatidylserine binding protein)	SDPR
243087_at	2.67	3.84	WD repeat domain 63	WDR63
244006_at	2.81	3.83		
216350_s_at	3.67	3.81	zinc finger protein 10	ZNF10
223558_at	2.48	3.80		
218769_s_at	5.35	3.79	ankyrin repeat, family A (RFXANK-like), 2	ANKRA2
226733_at	4.42	3.78	6-phosphofructo-2-kinase/fructose-2,6-biphosphatase 2	PFKFB2
235154_at	4.72	3.77	TAF3 RNA polymerase II, TATA box binding protein (TBP)-associated factor, 140kDa	TAF3
226390_at	3.38	3.76	START domain containing 4, sterol regulated	STARD4
1569403_at	5.58	3.76		
223879_s_at	4.55	3.74	oxidation resistance 1	OXR1

1552309_a_at	5.14	3.72	nexilin (F actin binding protein)	NEXN
1553710_at	2.08	3.71		
223854_at	3.86	3.71	protocadherin beta 10	PCDHB10
208498_s_at	6.41	3.70	amylase, alpha 1A; salivary	AMY1A
239363_at	3.31	3.68		
216344_at	8.60	3.67	nephronophthisis 4	NPHP4
217678_at	4.69	3.67	solute carrier family 7, (cationic amino acid transporter, y+ system) member 11	SLC7A11
1562259_at	2.32	3.67	testis expressed sequence 9	TEX9
240436_at	4.58	3.67		
235885_at	321.56	3.65		
232238_at	3.19	3.64	asp (abnormal spindle) homolog, microcephaly associated (Drosophila)	ASPM
226528_at	4.58	3.64	metaxin 3	MTX3
209921_at	3.27	3.62	solute carrier family 7, (cationic amino acid transporter, y+ system) member 11	SLC7A11
235447_at	2.27	3.62	TruB pseudouridine (psi) synthase homolog 1 (E. coli)	TRUB1
210821_x_at	3.23	3.61	centromere protein A	CENPA
243352_at	3.51	3.59	alpha-kinase 1	ALPK1
215796_at	10.40	3.59	T cell receptor alpha locus	TRA@
244165_at	3.72	3.59	chromosome 10 open reading frame 18	C10orf18
231055_at	2.48	3.59		
235512_at	2.09	3.58	cyclin-dependent kinase-like 1 (CDC2-related kinase)	CDKL1
219361_s_at	5.01	3.58	interferon stimulated exonuclease gene 20kDa-like 1	ISG20L1
224448_s_at	2.14	3.57	chromosome 6 open reading frame 125	C6orf125
241703_at	3.41	3.56		
205698_s_at	8.95	3.55	mitogen-activated protein kinase kinase 6	MAP2K6
203413_at	21.12	3.52	NEL-like 2 (chicken)	NELL2
1568807_a_at	6.85	3.51		
237065_s_at	2.40	3.51		
226966_at	5.68	3.51	PRP40 pre-mRNA processing factor 40 homolog B (S. cerevisiae)	PRPF40B
201150_s_at	3.04	3.50	TIMP metallopeptidase inhibitor 3 (Sorsby fundus dystrophy, pseudoinflammatory)	TIMP3
244571_s_at	7.48	3.50	tetratricopeptide repeat domain 12	TTC12
229360_at	7.79	3.48	suppressor of hairy wing homolog 2 (Drosophila)	SUHW2
238720_at	11.02	3.48	oligodendrocyte myelin glycoprotein	OMG
244634_at	2.81	3.45	family with sequence similarity 53, member B	FAM53B
1552705_at	4.75	3.45	dual specificity phosphatase 19	DUSP19
238962_at	4.80	3.45		

231513_at	4.15	3.44	potassium inwardly-rectifying channel, subfamily J, member 2	KCNJ2
235764_at	4.59	3.44		
234628_at	4.46	3.42		
239002_at	4.91	3.41	asp (abnormal spindle) homolog, microcephaly associated (Drosophila)	ASPM
228158_at	3.18	3.41		
227306_at	4.26	3.39		
1554242_a_at	2.81	3.39	coagulation factor C homolog, cochlin (Limulus polyphemus)	COCH
241399_at	6.17	3.39	family with sequence similarity 19 (chemokine (C-C motif)-like), member A2	FAM19A2
244424_at	4.37	3.39		
1569147_at	4.61	3.38		
233876_at	8.18	3.38		
232778_at	2.67	3.37		
214117_s_at	2.06	3.37	biotinidase	BTD
227332_at	3.52	3.36		
225420_at	4.51	3.35	glycerol-3-phosphate acyltransferase, mitochondrial	GPAM
241541_at	4.16	3.35	mindbomb homolog 2 (Drosophila)	MIB2
219793_at	3.37	3.34	sorting nexin 16	SNX16
210852_s_at	3.32	3.34	aminoadipate-semialdehyde synthase	AASS
205433_at	3.89	3.33	butyrylcholinesterase	BCHE
1570048_at	4.04	3.32	DPH4, JJJ3 homolog (S. cerevisiae)	DPH4
1557283_a_at	3.43	3.32	zinc finger protein 519	ZNF519
1560924_at	3.30	3.30		
1563460_at	11.41	3.29		
1569816_at	2.55	3.29		
229072_at	4.36	3.28		
1554966_a_at	2.34	3.28	filamin A interacting protein 1-like	FILIP1L
231822_at	2.31	3.27	CTTNBP2 N-terminal like	CTTNBP2NL
239896_at	2.38	3.26		
214748_at	2.03	3.26		
213929_at	5.69	3.26		
232829_at	6.78	3.25	olfactory receptor, family 52, subfamily K, member 3 pseudogene	OR52K3P
205514_at	5.14	3.25	zinc finger protein 415	ZNF415
238126_at	16.62	3.24		
220351_at	3.83	3.24	chemokine (C-C motif) receptor-like 1	CCRL1
222262_s_at	3.11	3.24	ethanolamine kinase 1	ETNK1
1560814_a_at	4.15	3.23	coiled-coil domain containing 32	CCDC32
235119_at	3.48	3.22	TAF3 RNA polymerase II, TATA box binding protein (TBP)-associated factor, 140kDa	TAF3
220353_at	2.09	3.21	family with sequence similarity 86, member C	FAM86C
242247_at	6.88	3.21	methyltransferase 5 domain containing 1	METT5D1
236550_s_at	3.43	3.21	zinc finger protein 311	ZNF311

226339_at	3.62	3.20	TruB pseudouridine (psi) synthase homolog 1 (E. coli)	TRUB1
219544_at	3.22	3.20	chromosome 13 open reading frame 34	C13orf34
209433_s_at	2.51	3.19	phosphoribosyl pyrophosphate amidotransferase	PPAT
207729_at	23.89	3.17	cadherin 9, type 2 (T1-cadherin)	CDH9
240974_at	2.46	3.17		
216748_at	4.37	3.17	pyrin and HIN domain family, member 1	PYHIN1
236174_at	2.52	3.17		
232063_x_at	3.84	3.16	phenylalanine-tRNA synthetase-like, beta subunit	FARSLB
227082_at	2.85	3.16		
214169_at	4.29	3.16	unc-84 homolog A (C. elegans)	UNC84A
223691_at	4.03	3.16	regulator of G-protein signalling 22	RGS22
244557_at	2.40	3.16		
1554963_at	3.66	3.16	chromosome 6 open reading frame 192	C6orf192
225462_at	3.21	3.15	transmembrane protein 128	TMEM128
207115_x_at	3.06	3.14	mbt domain containing 1	MBTD1
213853_at	3.05	3.14	DPH4, JJJ3 homolog (S. cerevisiae)	DPH4
209693_at	16.28	3.14	astrotactin 2	ASTN2
242872_at	3.08	3.14	citron (rho-interacting, serine/threonine kinase 21)	CIT
204712_at	2.63	3.14	WNT inhibitory factor 1	WIF1
238757_at	3.04	3.13	DBF4 homolog B (S. cerevisiae)	DBF4B
219773_at	31.68	3.13	NADPH oxidase 4	NOX4
235737_at	2.92	3.13		
239025_at	2.61	3.13		
227786_at	3.79	3.12	thyroid hormone receptor associated protein 6	THRAP6
236300_at	14.87	3.11		
235102_x_at	2.01	3.11	GRB2-related adaptor protein	GRAP
204940_at	46.43	3.11	phospholamban	PLN
236862_at	4.40	3.11	golgi associated PDZ and coiled-coil motif containing	GOPC
223582_at	3.27	3.11	G protein-coupled receptor 98	GPR98
203304_at	4.44	3.10	BMP and activin membrane-bound inhibitor homolog (Xenopus laevis)	BAMBI
204290_s_at	3.04	3.10	aldehyde dehydrogenase 6 family, member A1	ALDH6A1
224802_at	3.47	3.08	Nedd4 family interacting protein 2	NDFIP2
221988_at	2.74	3.08	cofactor required for Sp1 transcriptional activation, subunit 7, 70kDa	CRSP7
1556123_a_at	6.69	3.08		
204962_s_at	3.57	3.07	centromere protein A	CENPA
238466_at	3.31	3.07		
117_at	3.37	3.07		
202609_at	2.39	3.07	epidermal growth factor receptor pathway substrate 8	EPS8
1557132_at	4.36	3.07	WD repeat domain 17	WDR17

242996_at	2.19	3.07	mitochondrial translational release factor 1	MTRF1
218647_s_at	4.01	3.06	yrdC domain containing (E. coli)	YRDC
223672_at	4.14	3.06	SH3-domain GRB2-like (endophilin) interacting protein 1	SGIP1
206263_at	3.60	3.05	flavin containing monooxygenase 4	FMO4
223680_at	2.44	3.05	zinc finger protein 607	ZNF607
227765_at	3.82	3.05		
1554742_at	3.01	3.05	PMS1 postmeiotic segregation increased 1 (S. cerevisiae)	PMS1
237563_s_at	4.17	3.04		
1561125_at	9.97	3.04	methylenetetrahydrofolate dehydrogenase (NADP+ dependent) 1-like	MTHFD1L
1552430_at	3.12	3.04	WD repeat domain 17	WDR17
240239_at	2.25	3.04	zinc finger protein 566	ZNF566
1552365_at	5.25	3.03	scinderin	SCIN
227628_at	2.83	3.03		
1552971_at	5.43	3.02	sarcoglycan zeta	SGCZ
226301_at	4.16	3.02	chromosome 6 open reading frame 192	C6orf192
228967_at	2.26	3.02		
209463_s_at	3.02	3.02	TAF12 RNA polymerase II, TATA box binding protein (TBP)-associated factor, 20kDa	TAF12
1569385_s_at	3.06	3.02	KIAA1546	KIAA1546
232737_s_at	2.97	3.02	olfactory receptor, family 2, subfamily A, member 4	OR2A4
234405_s_at	2.07	3.02	RNA U, small nuclear RNA export adaptor (phosphorylation regulated)	RNUXA
206271_at	4.65	3.02	toll-like receptor 3	TLR3
212806_at	2.08	3.00		
1560020_at	3.53	2.99	DnaJ (Hsp40) homolog, subfamily C, member 13	DNAJC13
225687_at	4.51	2.99	family with sequence similarity 83, member D	FAM83D
213218_at	2.21	2.99	zinc finger protein 187	ZNF187
235608_at	3.94	2.99		
241905_at	2.76	2.99	phosphoinositide-3-kinase, class 2, alpha polypeptide	PIK3C2A
207808_s_at	2.82	2.99	protein S (alpha)	PROS1
1557430_at	2.37	2.99		
232985_s_at	3.59	2.99	developmental pluripotency associated 4	DPPA4
219078_at	2.61	2.98	G patch domain containing 2	GPATCH2
218940_at	2.97	2.98	chromosome 14 open reading frame 138	C14orf138
1558856_at	2.38	2.98	DMRT-like family A2	DMRTA2
218597_s_at	2.55	2.98	zinc finger, CDGSH-type domain 1	ZCD1
231947_at	4.22	2.97	myc target 1	MYCT1
240141_at	2.25	2.96	PAP associated domain containing 4	PAPD4
206314_at	3.03	2.96	zinc finger protein 167	ZNF167

209310_s_at	3.47	2.96	caspase 4, apoptosis-related cysteine peptidase	CASP4
1559902_at	2.13	2.95	megakaryoblastic leukemia (translocation) 1	MKL1
235320_at	5.83	2.95	ADP-ribosylation factor-like 6	ARL6
232146_at	5.94	2.95	NADH dehydrogenase (ubiquinone) 1, subcomplex unknown, 1, 6kDa	NDUFC1
230539_at	4.91	2.95		
235158_at	2.74	2.95		
1570125_at	2.17	2.94		
220549_at	2.96	2.94	RAD54 homolog B (S. cerevisiae)	RAD54B
228996_at	3.30	2.94	ring finger and CCCH-type zinc finger domains 1	RC3H1
220047_at	5.16	2.94	sirtuin (silent mating type information regulation 2 homolog) 4 (S. cerevisiae)	SIRT4
242470_at	3.75	2.94	EP300 interacting inhibitor of differentiation 2B	EID2B
201667_at	4.37	2.93	gap junction protein, alpha 1, 43kDa	GJA1
206497_at	2.25	2.93	chromosome 7 open reading frame 44	C7orf44
223373_s_at	2.87	2.93	phospholipase A2, group XIIA	PLA2G12A
1569599_at	6.57	2.93	SAM domain, SH3 domain and nuclear localization signals 1	SAMSN1
1558391_s_at	4.29	2.93	zinc finger protein 599	ZNF599
227963_at	4.50	2.92		
1564053_a_at	2.31	2.92	YTH domain family, member 3	YTHDF3
222798_at	2.75	2.92	phosphotriesterase related	PTER
230431_at	2.76	2.92		
231789_at	2.58	2.92	protocadherin beta 15	PCDHB15
207817_at	32.66	2.92	interferon, omega 1	IFNW1
217127_at	3.08	2.92	cystathionase (cystathionine gamma-lyase)	CTH
225689_at	2.99	2.91	chromosome 3 open reading frame 39	C3orf39
1554743_x_at	2.13	2.91	PMS1 postmeiotic segregation increased 1 (S. cerevisiae)	PMS1
232787_at	2.38	2.91		
203725_at	2.21	2.91	growth arrest and DNA-damage-inducible, alpha	GADD45A
220169_at	3.63	2.90	transmembrane protein 156	TMEM156
205938_at	2.43	2.90	protein phosphatase 1E (PP2C domain containing)	PPM1E
227214_at	3.13	2.90	golgi associated PDZ and coiled-coil motif containing	GOPC
209967_s_at	2.90	2.90	cAMP responsive element modulator	CREM
235425_at	2.08	2.89	shugoshin-like 2 (S. pombe)	SGOL2
217536_x_at	2.26	2.88		
238935_at	3.25	2.88	ribosomal protein S27-like	RPS27L
1554741_s_at	2.50	2.88		
206026_s_at	2.95	2.88	tumor necrosis factor, alpha-induced protein 6	TNFAIP6

207505_at	2.51	2.88	protein kinase, cGMP-dependent, type II	PRKG2
222351_at	3.13	2.88	protein phosphatase 2 (formerly 2A), regulatory subunit A, beta isoform	PPP2R1B
240065_at	4.35	2.88	family with sequence similarity 81, member B	FAM81B
227289_at	2.50	2.87	protocadherin 17	PCDH17
235134_at	2.86	2.87		
242409_at	3.15	2.87		
1553269_at	2.43	2.87	zinc finger protein 718	ZNF718
209146_at	3.03	2.87	sterol-C4-methyl oxidase-like	SC4MOL
212720_at	3.11	2.86	poly(A) polymerase alpha	PAPOLA
244743_x_at	2.05	2.86	zinc finger protein 138	ZNF138
203502_at	2.86	2.86	2,3-bisphosphoglycerate mutase	BPGM
229285_at	3.48	2.85	ribonuclease L (2',5'-oligoisoadenylate synthetase-dependent)	RNASEL
204159_at	2.90	2.85	cyclin-dependent kinase inhibitor 2C (p18, inhibits CDK4)	CDKN2C
1554418_s_at	3.09	2.85	sparc/osteonectin, cwcv and kazal-like domains proteoglycan (testican) 3	SPOCK3
209595_at	2.16	2.84	general transcription factor IIF, polypeptide 2, 30kDa	GTF2F2
225851_at	2.65	2.84	farnesyltransferase, CAAX box, beta	FNTB
221291_at	2.24	2.84	UL16 binding protein 2	ULBP2
231976_at	2.36	2.83	lines homolog 1 (Drosophila)	LINS1
228941_at	3.19	2.83		
233677_at	2.78	2.83		
235866_at	2.20	2.83		
203377_s_at	4.81	2.82	cell division cycle 40 homolog (S. cerevisiae)	CDC40
213805_at	2.24	2.82		
238504_at	3.51	2.81	chromosome 6 open reading frame 57	C6orf57
215342_s_at	4.02	2.81	RAB GTPase activating protein 1-like	RABGAP1L
222939_s_at	3.22	2.81	solute carrier family 16, member 10 (aromatic amino acid transporter)	SLC16A10
241823_at	6.42	2.80		
226600_at	2.58	2.80	transmembrane and tetratricopeptide repeat containing 3	TMTC3
237681_at	5.46	2.79		
235343_at	2.38	2.79		
204523_at	2.15	2.78	zinc finger protein 140	ZNF140
237168_at	2.69	2.78		
244049_at	2.52	2.78		
220145_at	5.05	2.78	microtubule-associated protein 9	MAP9
243815_at	2.12	2.77	piggyBac transposable element derived 4	PGBD4
242819_at	2.37	2.77	BET1 homolog (S. cerevisiae)	BET1
215307_at	3.08	2.77	zinc finger protein 529	ZNF529
204807_at	3.07	2.76	transmembrane protein 5	TMEM5

234295_at	3.48	2.76	debranching enzyme homolog 1 (S. cerevisiae)	DBR1
227546_x_at	3.39	2.76	cyclin L2	CCNL2
231269_at	4.10	2.76	activating signal cointegrator 1 complex subunit 3	ASCC3
235117_at	2.17	2.75	ChaC, cation transport regulator homolog 2 (E. coli)	CHAC2
219003_s_at	4.08	2.75	mannosidase, endo-alpha	MANEA
213032_at	3.37	2.75		
225424_at	3.43	2.75	glycerol-3-phosphate acyltransferase, mitochondrial	GPAM
243512_x_at	2.79	2.74	IMP2 inner mitochondrial membrane peptidase-like (S. cerevisiae)	IMMP2L
204135_at	3.01	2.74	filamin A interacting protein 1-like	FILIP1L
235064_s_at	2.13	2.73	chromosome 20 open reading frame 196	C20orf196
235528_at	4.66	2.73	guanylate cyclase activator 1B (retina)	GUCA1B
223412_at	3.71	2.73	kelch repeat and BTB (POZ) domain containing 7	KBTBD7
242557_at	4.98	2.73	chromosome 6 open reading frame 12	C6orf12
203889_at	6.39	2.73	secretogranin V (7B2 protein)	SCG5
219947_at	3.54	2.73	C-type lectin domain family 4, member A	CLEC4A
207103_at	4.19	2.73	potassium voltage-gated channel, Shal-related subfamily, member 2	KCND2
242255_at	4.01	2.72	WD repeat domain 37	WDR37
204167_at	3.90	2.72	biotinidase	BTD
218954_s_at	2.17	2.72	BRF2, subunit of RNA polymerase III transcription initiation factor, BRF1-like	BRF2
213424_at	2.60	2.71		
239710_at	5.72	2.71		
1554547_at	2.71	2.71	family with sequence similarity 13, member C1	FAM13C1
1556062_at	2.22	2.70	ribonuclease P/MRP 30kDa subunit	RPP30
233840_at	2.37	2.70		
1553122_s_at	3.00	2.70	RB-associated KRAB zinc finger	RBAK
223229_at	2.14	2.70	ubiquitin-conjugating enzyme E2T (putative)	UBE2T
227176_at	3.86	2.70	solute carrier family 2 (facilitated glucose transporter), member 13	SLC2A13
206648_at	4.53	2.69	zinc finger protein 571	ZNF571
232481_s_at	2.48	2.69	SLIT and NTRK-like family, member 6	SLITRK6
45653_at	2.19	2.69	potassium channel tetramerisation domain containing 13	KCTD13
203762_s_at	2.25	2.69	dynein, cytoplasmic 2, light intermediate chain 1	DYNC2LI1
201791_s_at	4.34	2.69	7-dehydrocholesterol reductase	DHCR7
238475_at	2.16	2.68		
230351_at	2.60	2.68		

202058_s_at	2.37	2.68	karyopherin alpha 1 (importin alpha 5)	KPNA1
226945_at	2.40	2.68	rhomboid domain containing 1	RHBDD1
230083_at	2.19	2.67		
203478_at	3.03	2.67	NADH dehydrogenase (ubiquinone) 1, subcomplex unknown, 1, 6kDa	NDUFC1
233852_at	2.11	2.67	polymerase (DNA directed), eta	POLH
229107_at	2.08	2.67		
205443_at	3.07	2.67	small nuclear RNA activating complex, polypeptide 1, 43kDa	SNAPC1
1569251_a_at	2.29	2.67	zinc finger protein 333	ZNF333
222573_s_at	2.04	2.66	salvador homolog 1 (Drosophila)	SAV1
228655_at	2.23	2.66		
222857_s_at	3.58	2.66	potassium large conductance calcium-activated channel, subfamily M, beta member 4	KCNMB4
236958_at	2.70	2.65		
228660_x_at	3.91	2.65	sema domain, immunoglobulin domain (Ig), transmembrane domain (TM) and short cytoplasmic domain, (se	SEMA4F
208375_at	11.09	2.65	interferon, alpha 1	IFNA1
1568720_at	2.94	2.65	zinc finger protein 506	ZNF506
220631_at	6.28	2.65	O-sialoglycoprotein endopeptidase-like 1	OSGEPL1
1558809_s_at	3.02	2.65		
227941_at	3.00	2.65		
1552370_at	2.18	2.64	chromosome 4 open reading frame 33	C4orf33
1563512_at	5.07	2.64	nitric oxide synthase 1 (neuronal) adaptor protein	NOS1AP
1558501_at	6.44	2.64	dynamin 3	DNM3
240230_s_at	2.12	2.64		
1553192_at	2.00	2.64	zinc finger protein 441	ZNF441
1556209_at	3.15	2.64	C-type lectin domain family 2, member B	CLEC2B
223397_s_at	2.24	2.63	nuclear import 7 homolog (S. cerevisiae)	NIP7
1556122_at	4.09	2.63		
225578_at	3.63	2.63		
1568787_at	3.58	2.63		
242667_at	3.12	2.63		
1553983_at	2.28	2.62		
202973_x_at	3.23	2.62	family with sequence similarity 13, member A1	FAM13A1
229220_x_at	2.37	2.62	nucleolar protein with MIF4G domain 1	NOM1
207528_s_at	2.17	2.61	solute carrier family 7, (cationic amino acid transporter, y+ system) member 11	SLC7A11
228430_at	2.15	2.61		
1553801_a_at	2.88	2.61	chromosome 14 open reading frame 126	C14orf126

208900_s_at	2.08	2.61	topoisomerase (DNA) I	TOP1
208115_x_at	2.20	2.61	chromosome 10 open reading frame 137	C10orf137
233110_s_at	2.53	2.61	BCL2-like 12 (proline rich)	BCL2L12
214093_s_at	2.78	2.60	far upstream element (FUSE) binding protein 1	FUBP1
236305_at	2.44	2.60	Rieske (Fe-S) domain containing	RFESD
228146_at	4.25	2.60	chromosome 17 open reading frame 51	C17orf51
221845_s_at	2.67	2.60	ClpB caseinolytic peptidase B homolog (E. coli)	CLPB
1558699_a_at	2.96	2.59	HERPUD family member 2	HERPUD2
243188_at	3.71	2.59	zinc finger protein 283	ZNF283
1565685_at	2.61	2.59		
242579_at	2.55	2.59		
238867_at	6.48	2.59	transmembrane protein 182	TMEM182
1558256_at	2.39	2.59		
209300_s_at	3.30	2.58	NECAP endocytosis associated 1	NECAP1
244290_at	8.64	2.58		
209927_s_at	2.77	2.58	chromosome 1 open reading frame 77	C1orf77
202146_at	2.66	2.58	interferon-related developmental regulator 1	IFRD1
226195_at	2.25	2.58	chromosome 14 open reading frame 179	C14orf179
1560048_at	4.16	2.58		
225290_at	2.88	2.57	ethanolamine kinase 1	ETNK1
210762_s_at	3.52	2.57	deleted in liver cancer 1	DLC1
228915_at	2.62	2.57	dachshund homolog 1 (Drosophila)	DACH1
241014_at	3.27	2.57		
225914_s_at	3.25	2.57	calcium binding protein 39-like	CAB39L
209451_at	2.54	2.57	TRAF family member-associated NFKB activator	TANK
217242_at	3.28	2.57	zinc finger protein 154	ZNF154
1554015_a_at	2.65	2.56	chromodomain helicase DNA binding protein 2	CHD2
206765_at	4.25	2.56	potassium inwardly-rectifying channel, subfamily J, member 2	KCNJ2
241546_at	3.01	2.56	spermatogenesis associated 5	SPATA5
206615_s_at	2.51	2.56	ADAM metallopeptidase domain 22	ADAM22
228863_at	4.87	2.56	protocadherin 17	PCDH17
234996_at	6.53	2.55		
231577_s_at	2.95	2.55	guanylate binding protein 1, interferon-inducible, 67kDa	GBP1
1568673_s_at	96.66	2.54	ELL associated factor 2	EAF2
228717_at	2.41	2.54		
219239_s_at	3.63	2.54	zinc finger protein 654	ZNF654
226181_at	2.65	2.54	tubulin, epsilon 1	TUBE1
229582_at	2.17	2.54	chromosome 18 open reading frame 37	C18orf37
205716_at	2.16	2.54	solute carrier family 25, member 40	SLC25A40
1564190_x_at	2.42	2.54	zinc finger protein 519	ZNF519

235063_at	26.17	2.53	chromosome 20 open reading frame 196	C20orf196
243796_at	3.05	2.53		
1554345_a_at	3.91	2.53	zinc finger, H2C2 domain containing	ZH2C2
223651_x_at	4.19	2.53	cell division cycle 23 homolog (S. cerevisiae)	CDC23
227559_at	2.21	2.53		
203589_s_at	3.67	2.53	transcription factor Dp-2 (E2F dimerization partner 2)	TFDP2
226890_at	2.30	2.53	WD repeat domain 35	WDR35
218739_at	2.21	2.53	abhydrolase domain containing 5	ABHD5
239479_x_at	3.33	2.53		
220121_at	3.33	2.52	lines homolog 1 (Drosophila)	LINS1
218285_s_at	2.89	2.52	3-hydroxybutyrate dehydrogenase, type 2	BDH2
228766_at	2.25	2.52	CD36 molecule (thrombospondin receptor)	CD36
239091_at	2.66	2.51		
238063_at	2.58	2.51		
244784_at	3.63	2.51	DEAH (Asp-Glu-Ala-Asp/His) box polypeptide 57	DHX57
219017_at	2.82	2.51	ethanolamine kinase 1	ETNK1
222581_at	3.49	2.51	xenotropic and polytropic retrovirus receptor	XPR1
202411_at	3.64	2.51	interferon, alpha-inducible protein 27	IFI27
231817_at	2.54	2.51	ubiquitin specific peptidase 53	USP53
243371_at	3.73	2.51		
232018_at	2.00	2.50	leukocyte receptor cluster (LRC) member 1	LENG1
1559108_at	3.36	2.50		
1569714_at	2.53	2.50	zinc finger, FYVE domain containing 20	ZFYVE20
239995_at	4.68	2.50		
222682_s_at	2.69	2.49	chromosome 5 open reading frame 3	C5orf3
1569191_at	3.26	2.49		
202469_s_at	2.88	2.49	cleavage and polyadenylation specific factor 6, 68kDa	CPSF6
239466_at	2.03	2.49		
232163_at	2.13	2.49	WD repeat domain 19	WDR19
1556935_at	4.38	2.49		
235155_at	4.52	2.49	3-hydroxybutyrate dehydrogenase, type 2	BDH2
229018_at	2.44	2.48	chromosome 12 open reading frame 26	C12orf26
231838_at	2.87	2.48	chromosome 20 open reading frame 119	C20orf119
224453_s_at	2.22	2.48	ethanolamine kinase 1	ETNK1
1554518_at	2.11	2.48	glutathione S-transferase, C-terminal domain containing	GSTCD
211734_s_at	2.31	2.48	Fc fragment of IgE, high affinity I, receptor for; alpha polypeptide	FCER1A
209839_at	2.65	2.48	dynamin 3	DNM3
240793_at	2.34	2.47	titin	TTN

212121_at	2.20	2.47	chromosome 10 open reading frame 61	C10orf61
216279_at	4.82	2.47	zinc finger protein 460	ZNF460
224452_s_at	2.56	2.47		
228090_at	3.22	2.47	nicotinamide nucleotide adenylyltransferase 3	NMNAT3
232038_at	3.27	2.47	chromosome 6 open reading frame 170	C6orf170
244257_at	12.87	2.46	transmembrane protein 104	TMEM104
1554915_a_at	2.20	2.46		
223792_at	2.48	2.46	zinc finger protein 2	ZNF2
236551_at	2.83	2.46	zinc finger protein 311	ZNF311
239188_at	2.38	2.46	protein phosphatase 2 (formerly 2A), regulatory subunit B", gamma	PPP2R3C
242433_at	5.30	2.46	zinc finger and BTB domain containing 11	ZBTB11
218185_s_at	2.03	2.45	armadillo repeat containing 1	ARMC1
212107_s_at	3.16	2.45	DEAH (Asp-Glu-Ala-His) box polypeptide 9	DHX9
208922_s_at	2.24	2.45	nuclear RNA export factor 1	NXF1
1556061_at	2.67	2.45	ribonuclease P/MRP 30kDa subunit	RPP30
239311_at	3.14	2.45		
221027_s_at	3.87	2.45	phospholipase A2, group XIIA	PLA2G12A
203913_s_at	2.06	2.45	hydroxyprostaglandin dehydrogenase 15-(NAD)	HPGD
213704_at	2.34	2.44	Rab geranylgeranyltransferase, beta subunit	RABGGTB
218477_at	2.95	2.44	transmembrane protein 14A	TMEM14A
203176_s_at	2.17	2.44	transcription factor A, mitochondrial	TFAM
1556195_a_at	2.52	2.44		
205934_at	2.65	2.44	phospholipase C-like 1	PLCL1
230095_at	2.08	2.44	thioredoxin-like 2	TXNL2
235026_at	2.98	2.44		
240344_x_at	2.59	2.43	Lyrm7 homolog (mouse)	LYRM7
232841_at	2.42	2.43		
1557985_s_at	2.12	2.43	centrosomal protein 78kDa	CEP78
242828_at	3.62	2.43	fidgetin	FIGN
242533_at	2.26	2.43	transmembrane protein 112	TMEM112
218513_at	2.09	2.42		
227458_at	3.06	2.42		
238946_at	3.14	2.42		
221669_s_at	2.05	2.42	acyl-Coenzyme A dehydrogenase family, member 8	ACAD8
218680_x_at	2.15	2.42		
1552695_a_at	2.95	2.42	solute carrier family 2 (facilitated glucose transporter), member 13	SLC2A13
213122_at	2.34	2.41	TSPY-like 5	TSPYL5
229075_at	2.92	2.41		
231850_x_at	2.48	2.41	KIAA1712	KIAA1712
237440_at	3.48	2.41		
208670_s_at	2.29	2.41	EP300 interacting inhibitor of differentiation 1	EID1
218755_at	4.08	2.41	kinesin family member 20A	KIF20A

226588_at	2.47	2.40		
235653_s_at	2.42	2.40	THAP domain containing 6	THAP6
206085_s_at	2.82	2.40	cystathionase (cystathionine gamma-lyase)	CTH
221927_s_at	3.00	2.40	abhydrolase domain containing 11	ABHD11
232278_s_at	2.34	2.40	DEP domain containing 1	DEPDC1
216070_at	4.64	2.40		
232247_at	3.99	2.40	zinc finger protein 502	ZNF502
206613_s_at	2.44	2.40	TATA box binding protein (TBP)-associated factor, RNA polymerase I, A, 48kDa	TAF1A
1557383_a_at	3.86	2.39		
218616_at	2.73	2.39	integrator complex subunit 12	INTS12
200895_s_at	2.37	2.39	FK506 binding protein 4, 59kDa	FKBP4
204641_at	2.72	2.39	NIMA (never in mitosis gene a)-related kinase 2	NEK2
230324_at	2.37	2.39	nuclear receptor coactivator 2	NCOA2
203213_at	2.27	2.39	cell division cycle 2, G1 to S and G2 to M	CDC2
218538_s_at	2.30	2.38	MRS2-like, magnesium homeostasis factor (S. cerevisiae)	MRS2L
223413_s_at	4.51	2.38		
207183_at	2.36	2.38	G protein-coupled receptor 19	GPR19
205018_s_at	2.71	2.38	muscleblind-like 2 (Drosophila)	MBNL2
210223_s_at	2.74	2.38	major histocompatibility complex, class I-related	MR1
206637_at	2.05	2.38	purinergic receptor P2Y, G-protein coupled, 14	P2RY14
238952_x_at	2.43	2.37		
1554014_at	3.55	2.37	chromodomain helicase DNA binding protein 2	CHD2
1555384_a_at	2.17	2.37	La ribonucleoprotein domain family, member 4	LARP4
235606_at	3.79	2.37		
225548_at	7.75	2.37	shroom family member 3	SHROOM3
206025_s_at	2.08	2.37	tumor necrosis factor, alpha-induced protein 6	TNFAIP6
222553_x_at	3.15	2.36	oxidation resistance 1	OXR1
243312_at	3.50	2.36	zinc finger protein 588	ZNF588
221962_s_at	2.04	2.36	ubiquitin-conjugating enzyme E2H (UBC8 homolog, yeast)	UBE2H
1554964_x_at	4.00	2.36	chromosome 6 open reading frame 192	C6orf192
235697_at	2.05	2.35		
205229_s_at	4.17	2.35	coagulation factor C homolog, cochlin (Limulus polyphemus)	COCH
209551_at	3.10	2.35	Yip1 domain family, member 4	YIPF4
227121_at	3.85	2.35		
204109_s_at	2.05	2.35	nuclear transcription factor Y, alpha	NFYA
213599_at	2.14	2.35	Opa interacting protein 5	OIP5
222184_at	9.97	2.35		
209681_at	2.66	2.35	solute carrier family 19 (thiamine transporter), member 2	SLC19A2

1564231_at	2.55	2.35	intraflagellar transport 80 homolog (Chlamydomonas)	IFT80
205357_s_at	2.92	2.35	angiotensin II receptor, type 1	AGTR1
228003_at	2.23	2.35	RAB30, member RAS oncogene family	RAB30
228184_at	2.71	2.35	dispatched homolog 1 (Drosophila)	DISP1
228855_at	2.96	2.34	nudix (nucleoside diphosphate linked moiety X)-type motif 7	NUDT7
202269_x_at	2.79	2.34	guanylate binding protein 1, interferon-inducible, 67kDa	GBP1
226443_at	2.69	2.34	family with sequence similarity 122A	FAM122A
209666_s_at	2.12	2.34	conserved helix-loop-helix ubiquitous kinase	CHUK
201732_s_at	3.01	2.33	chloride channel 3	CLCN3
1556725_a_at	2.37	2.33		
228390_at	2.65	2.33		
233241_at	3.77	2.33	chromosome 20 open reading frame 19	C20orf19
203011_at	2.19	2.33	inositol(myo)-1(or 4)-monophosphatase 1	IMPA1
238944_at	4.73	2.33	zinc finger protein 404	ZNF404
209135_at	4.59	2.33	aspartate beta-hydroxylase	ASPH
203159_at	3.34	2.33	glutaminase	GLS
238729_x_at	3.05	2.33		
227337_at	2.31	2.33	ankyrin repeat domain 37	ANKRD37
1552306_at	2.17	2.33	asparagine-linked glycosylation 10 homolog (yeast, alpha-1,2-glucosyltransferase)	ALG10
1556325_at	3.72	2.33	filamin A interacting protein 1	FILIP1
231840_x_at	3.42	2.32	Lyrm7 homolog (mouse)	LYRM7
226661_at	3.57	2.32	cell division cycle associated 2	CDCA2
202270_at	2.12	2.32	guanylate binding protein 1, interferon-inducible, 67kDa	GBP1
200868_s_at	2.14	2.32	zinc finger protein 313	ZNF313
205624_at	2.00	2.32	carboxypeptidase A3 (mast cell)	CPA3
1563614_at	2.81	2.32	Mdm2, transformed 3T3 cell double minute 2, p53 binding protein (mouse) binding protein, 104kDa	MTBP
239587_at	3.87	2.32		
214349_at	2.56	2.32		
202095_s_at	2.21	2.32	baculoviral IAP repeat-containing 5 (survivin)	BIRC5
239400_at	2.80	2.31		
226065_at	2.83	2.31	prickle homolog 1 (Drosophila)	PRICKLE1
205486_at	4.34	2.31	testis-specific kinase 2	TESK2
236619_at	2.38	2.31		
224734_at	2.05	2.31	high-mobility group box 1	HMGB1
223834_at	5.06	2.30	CD274 molecule	CD274
233981_at	3.43	2.30		
209185_s_at	2.28	2.30	insulin receptor substrate 2	IRS2
225066_at	2.62	2.30	protein phosphatase 2, regulatory subunit B, delta isoform	PPP2R2D

222827_s_at	2.16	2.30	kelch repeat and BTB (POZ) domain containing 10	KBTBD10
206011_at	3.11	2.29	caspase 1, apoptosis-related cysteine peptidase (interleukin 1, beta, convertase)	CASP1
219397_at	4.33	2.29	coenzyme Q10 homolog B (S. cerevisiae)	COQ10B
232242_at	2.11	2.29		
219494_at	2.07	2.29	RAD54 homolog B (S. cerevisiae)	RAD54B
203157_s_at	3.31	2.29	glutaminase	GLS
228201_at	2.12	2.29	ADP-ribosylation factor-like 13B	ARL13B
219467_at	2.85	2.29	zinc finger, H2C2 domain containing	ZH2C2
238623_at	3.00	2.29		
235215_at	2.95	2.29		
237083_at	2.36	2.29		
203854_at	5.20	2.28	complement factor I	CFI
1568941_a_at	4.60	2.28		
223249_at	4.01	2.28	claudin 12	CLDN12
236546_at	2.25	2.28		
238647_at	2.24	2.28	chromosome 14 open reading frame 28	C14orf28
205420_at	2.08	2.28	peroxisomal biogenesis factor 7	PEX7
211080_s_at	2.62	2.27	NIMA (never in mitosis gene a)-related kinase 2	NEK2
218197_s_at	3.66	2.27	oxidation resistance 1	OXR1
229659_s_at	2.26	2.27	polymeric immunoglobulin receptor	PIGR
235342_at	24.93	2.27	sparc/osteonectin, cwcv and kazal-like domains proteoglycan (testican) 3	SPOCK3
232398_at	2.44	2.27		
1553301_a_at	3.86	2.27	transmembrane protein 182	TMEM182
204920_at	3.09	2.27	carbamoyl-phosphate synthetase 1, mitochondrial	CPS1
221220_s_at	2.14	2.26	SCY1-like 2 (S. cerevisiae)	SCYL2
239265_at	2.30	2.26	transmembrane protein 20	TMEM20
215820_x_at	2.30	2.26	sorting nexin 13	SNX13
205988_at	3.16	2.26	CD84 molecule	CD84
207565_s_at	3.48	2.26	major histocompatibility complex, class I-related	MR1
221595_at	4.29	2.26		
1562270_at	29.48	2.26	Rho guanine nucleotide exchange factor (GEF) 7	ARHGEF7
1561965_at	21.11	2.26		
202094_at	3.59	2.25	baculoviral IAP repeat-containing 5 (survivin)	BIRC5
207630_s_at	2.35	2.25	cAMP responsive element modulator	CREM
1568987_at	3.63	2.25		
214668_at	2.16	2.25		
210432_s_at	3.20	2.25	sodium channel, voltage-gated, type III, alpha subunit	SCN3A
221640_s_at	2.78	2.25	leucine-rich repeats and death domain containing	LRDD
206591_at	22.44	2.25	recombination activating gene 1	RAG1

227787_s_at	3.17	2.25	thyroid hormone receptor associated protein 6	THRAP6
226094_at	2.75	2.24	phosphoinositide-3-kinase, class 2, alpha polypeptide	PIK3C2A
232228_at	2.19	2.24	zinc finger protein 530	ZNF530
1552970_s_at	3.41	2.24	zinc finger, MYM-type 6	ZMYM6
207717_s_at	2.07	2.24	plakophilin 2	PKP2
237750_at	4.52	2.24	X-prolyl aminopeptidase (aminopeptidase P) 3, putative	XPNPEP3
230393_at	2.78	2.24		
234995_at	2.18	2.24	coiled-coil domain containing 52	CCDC52
220306_at	3.72	2.24	family with sequence similarity 46, member C	FAM46C
215672_s_at	2.69	2.24		
231270_at	3.09	2.24	carbonic anhydrase XIII	CA13
1556347_at	2.34	2.24		
1567079_at	3.66	2.24	ceroid-lipofuscinosis, neuronal 6, late infantile, variant	CLN6
1559103_s_at	4.80	2.24		
221596_s_at	2.56	2.24		
218346_s_at	3.39	2.23	sestrin 1	SESN1
243300_at	4.38	2.23		
217010_s_at	2.15	2.23	cell division cycle 25 homolog C (S. pombe)	CDC25C
226375_at	4.21	2.23		
219258_at	2.01	2.23	TIMELESS interacting protein	TIPIN
201138_s_at	2.49	2.23	Sjogren syndrome antigen B (autoantigen La)	SSB
213124_at	4.47	2.22	zinc finger protein 473	ZNF473
223515_s_at	2.97	2.22	coenzyme Q3 homolog, methyltransferase (S. cerevisiae)	COQ3
203610_s_at	2.36	2.22	tripartite motif-containing 38	TRIM38
219287_at	3.89	2.22	potassium large conductance calcium-activated channel, subfamily M, beta member 4	KCNMB4
1557826_at	2.06	2.22		
222128_at	3.17	2.22	NOL1/NOP2/Sun domain family, member 6	NSUN6
219294_at	2.67	2.22	centromere protein Q	CENPQ
239482_x_at	2.17	2.22	zinc finger protein 708	ZNF708
201214_s_at	2.12	2.22	protein phosphatase 1, regulatory (inhibitor) subunit 7	PPP1R7
222729_at	2.26	2.22		
221104_s_at	3.07	2.22	nipsnap homolog 3B (C. elegans)	NIPSNAP3B
1557278_s_at	2.16	2.21	transportin 1	TNPO1
237745_at	3.79	2.21		
209375_at	2.73	2.21	xeroderma pigmentosum, complementation group C	XPC
208392_x_at	2.35	2.21	SP110 nuclear body protein	SP110
49077_at	2.21	2.21	protein phosphatase methylesterase 1	PPME1
227022_at	2.25	2.21	glucosamine-6-phosphate deaminase 2	GNPDA2
219826_at	3.42	2.21	zinc finger protein 419	ZNF419

235423_at	2.15	2.21		
213930_at	2.37	2.21		
202768_at	2.14	2.21	FBJ murine osteosarcoma viral oncogene homolog B	FOSB
218784_s_at	2.61	2.21	chromosome 6 open reading frame 64	C6orf64
219651_at	2.43	2.21	developmental pluripotency associated 4	DPPA4
226271_at	3.16	2.20	ganglioside-induced differentiation-associated protein 1	GDAP1
1566472_s_at	2.31	2.20		
243943_x_at	2.52	2.20	chromosome 6 open reading frame 52	C6orf52
214537_at	2.68	2.20	histone cluster 1, H1d	HIST1H1D
1557701_s_at	2.58	2.20	polymerase (DNA directed), eta	POLH
205260_s_at	2.65	2.20	acylphosphatase 1, erythrocyte (common) type	ACYP1
229586_at	2.14	2.20	chromodomain helicase DNA binding protein 9	CHD9
1569318_at	2.21	2.20		
214734_at	3.39	2.19	exophilin 5	EXPH5
215220_s_at	2.67	2.19	translocated promoter region (to activated MET oncogene)	TPR
37577_at	3.30	2.19	Rho GTPase activating protein 19	ARHGAP19
223434_at	2.27	2.19	guanylate binding protein 3	GBP3
227805_at	2.09	2.19		
243055_at	2.55	2.19		
230491_at	3.77	2.19		
219822_at	2.50	2.19	mitochondrial translational release factor 1	MTRF1
222513_s_at	3.61	2.18	sorbin and SH3 domain containing 1	SORBS1
219177_at	2.36	2.18	brix domain containing 2	BXDC2
218043_s_at	2.40	2.18	5-azacytidine induced 2	AZI2
214061_at	2.82	2.18	WD repeat domain 67	WDR67
206828_at	4.37	2.18	TXK tyrosine kinase	TXK
221786_at	2.50	2.18	chromosome 6 open reading frame 120	C6orf120
205011_at	3.10	2.18	loss of heterozygosity, 11, chromosomal region 2, gene A	LOH11CR2A
206383_s_at	2.45	2.18	GTPase activating protein (SH3 domain) binding protein 2	G3BP2
235839_at	2.62	2.18		
229374_at	4.55	2.17	EPH receptor A4	EPHA4
212973_at	3.21	2.17	ribose 5-phosphate isomerase A (ribose 5-phosphate epimerase)	RPIA
204392_at	2.14	2.17	calcium/calmodulin-dependent protein kinase I	CAMK1
201847_at	2.87	2.17	lipase A, lysosomal acid, cholesterol esterase (Wolman disease)	LIPA
1555598_a_at	3.85	2.17	dual specificity phosphatase 19	DUSP19
214862_x_at	2.20	2.17		
226537_at	2.66	2.17	histidine triad nucleotide binding protein 3	HINT3

222805_at	2.25	2.16	mannosidase, endo-alpha	MANEA
204971_at	2.11	2.16	cystatin A (stefin A)	CSTA
1557192_at	2.66	2.16	chromosome 1 open reading frame 136	C1orf136
225847_at	3.34	2.16	arylacetamide deacetylase-like 1	AADACL1
1569114_at	3.20	2.16		
221705_s_at	2.51	2.16		
228841_at	2.86	2.16		
226252_at	4.49	2.15		
203947_at	2.46	2.15	cleavage stimulation factor, 3' pre-RNA, subunit 3, 77kDa	CSTF3
227170_at	2.04	2.15		
219915_s_at	3.07	2.15	solute carrier family 16, member 10 (aromatic amino acid transporter)	SLC16A10
212077_at	3.57	2.15	caldesmon 1	CALD1
241115_at	2.25	2.15	KIAA1467	KIAA1467
218135_at	2.39	2.15	ERGIC and golgi 2	ERGIC2
221928_at	2.31	2.15	acetyl-Coenzyme A carboxylase beta	ACACB
1561948_at	3.13	2.15	coenzyme Q10 homolog B (S. cerevisiae)	COQ10B
219875_s_at	2.83	2.15	chromosome 1 open reading frame 121	C1orf121
231950_at	2.33	2.14	zinc finger protein 658	ZNF658
213156_at	4.39	2.14		
227967_at	2.08	2.14	tubulin, gamma complex associated protein 5	TUBGCP5
214277_at	2.96	2.14	COX11 homolog, cytochrome c oxidase assembly protein (yeast)	COX11
212510_at	2.17	2.14	glycerol-3-phosphate dehydrogenase 1-like	GPD1L
234531_at	3.41	2.13		
210976_s_at	2.47	2.13	phosphofructokinase, muscle	PFKM
210755_at	8.45	2.13	hepatocyte growth factor (hepapoietin A; scatter factor)	HGF
242838_at	2.17	2.13	MAP6 domain containing 1	MAP6D1
219343_at	3.06	2.13	cell division cycle 37 homolog (S. cerevisiae)-like 1	CDC37L1
205776_at	2.27	2.13	flavin containing monooxygenase 5	FMO5
218373_at	2.20	2.13	AKT interacting protein	AKTIP
227812_at	2.28	2.13	tumor necrosis factor receptor superfamily, member 19	TNFRSF19
226948_at	2.16	2.13	rhomboid domain containing 1	RHBDD1
235125_x_at	2.09	2.13		
228029_at	2.20	2.13	ATP-binding cassette, sub-family A (ABC1), member 11 (pseudogene)	ABCA11
210465_s_at	2.09	2.13	small nuclear RNA activating complex, polypeptide 3, 50kDa	SNAPC3
224447_s_at	2.09	2.13	chromosome 17 open reading frame 37	C17orf37
223764_x_at	3.82	2.13	nipsnap homolog 3B (C. elegans)	NIPSNAP3B
207046_at	3.17	2.12	histone cluster 2, H4a	HIST2H4A
222958_s_at	2.21	2.12	DEP domain containing 1	DEPDC1

228050_at	2.26	2.12	UTP15, U3 small nucleolar ribonucleoprotein, homolog (S. cerevisiae)	UTP15
213934_s_at	3.43	2.12	zinc finger protein 23 (KOX 16)	ZNF23
232600_at	3.18	2.12	ankyrin repeat domain 42	ANKRD42
228328_at	2.13	2.12	kelch-like 28 (Drosophila)	KLHL28
201243_s_at	2.43	2.12	ATPase, Na+/K+ transporting, beta 1 polypeptide	ATP1B1
219662_at	2.13	2.12	chromosome 2 open reading frame 49	C2orf49
202007_at	2.08	2.12	nidogen 1	NID1
227084_at	2.23	2.11	dystrobrevin, alpha	DTNA
210215_at	2.42	2.11	transferrin receptor 2	TFR2
244359_s_at	2.35	2.11		
244641_at	2.63	2.11	chromosome 7 open reading frame 30	C7orf30
228305_at	2.45	2.11	zinc finger protein 565	ZNF565
1552735_at	3.27	2.11	protocadherin gamma subfamily A, 4	PCDHGA4
238057_at	2.50	2.11	ubiquitin specific peptidase 45	USP45
219918_s_at	3.10	2.10	asp (abnormal spindle) homolog, microcephaly associated (Drosophila)	ASPM
223462_at	3.18	2.10	transmembrane protein 175	TMEM175
1555350_at	2.68	2.10	periphilin 1	PPHLN1
204271_s_at	4.45	2.10	endothelin receptor type B	EDNRB
237081_at	2.04	2.10		
242900_at	3.65	2.10		
218264_at	2.01	2.10	BRCA2 and CDKN1A interacting protein	BCCIP
1569250_at	3.04	2.09	zinc finger protein 333	ZNF333
227686_at	2.06	2.09	oxidoreductase NAD-binding domain containing 1	OXNAD1
213761_at	2.45	2.09	Mdm4, transformed 3T3 cell double minute 1, p53 binding protein (mouse)	MDM1
212738_at	2.92	2.09	Rho GTPase activating protein 19	ARHGAP19
238634_x_at	4.57	2.09		
202823_at	2.07	2.09	transcription elongation factor B (SIII), polypeptide 1 (15kDa, elongin C)	TCEB1
223036_at	2.42	2.09	phenylalanine-tRNA synthetase-like, beta subunit	FARSLB
225834_at	2.75	2.09		
212936_at	2.44	2.09	chromosome 5 open reading frame 21	C5orf21
222317_at	3.84	2.09	phosphodiesterase 3B, cGMP-inhibited	PDE3B
225023_at	4.81	2.09	golgi associated PDZ and coiled-coil motif containing	GOPC
226787_at	2.57	2.09	zinc finger protein 18	ZNF18
219667_s_at	2.11	2.09	B-cell scaffold protein with ankyrin repeats 1	BANK1

203763_at	2.88	2.09	dynein, cytoplasmic 2, light intermediate chain 1	DYNC2LI1
223538_at	2.27	2.09	small EDRK-rich factor 1A (telomeric)	SERF1A
219587_at	4.28	2.08	tetratricopeptide repeat domain 12	TTC12
239406_at	2.41	2.08	zinc finger protein 193	ZNF193
201707_at	2.84	2.08	peroxisomal biogenesis factor 19	PEX19
236613_at	4.18	2.08	RNA binding motif protein 25	RBM25
215239_x_at	2.43	2.08	zinc finger protein 273	ZNF273
226558_at	2.22	2.08		
209795_at	4.57	2.07	CD69 molecule	CD69
238773_at	4.49	2.07	methyltransferase 5 domain containing 1	METT5D1
229532_at	3.06	2.07	zinc finger protein 502	ZNF502
204008_at	2.61	2.07	dynein, axonemal, light chain 4	DNAL4
236217_at	2.46	2.07	solute carrier family 31 (copper transporters), member 1	SLC31A1
242628_at	2.26	2.07	killer cell lectin-like receptor subfamily B, member 1	KLRB1
220843_s_at	2.42	2.07	WD repeats and SOF1 domain containing	WDSOF1
235453_at	2.99	2.07		
215624_at	2.31	2.07	tuberous sclerosis 2	TSC2
226803_at	2.32	2.07	chromatin modifying protein 4C	CHMP4C
219951_s_at	2.98	2.07	chromosome 20 open reading frame 12	C20orf12
235387_at	3.04	2.06	glutathione S-transferase, C-terminal domain containing	GSTCD
223570_at	3.19	2.06	MCM10 minichromosome maintenance deficient 10 (S. cerevisiae)	MCM10
210559_s_at	2.37	2.06	cell division cycle 2, G1 to S and G2 to M	CDC2
221201_s_at	2.38	2.06	zinc finger protein 155	ZNF155
238614_x_at	2.56	2.06	zinc finger protein 430	ZNF430
219980_at	2.66	2.06	chromosome 4 open reading frame 29	C4orf29
205427_at	2.24	2.06	zinc finger protein 354A	ZNF354A
226811_at	3.37	2.06	family with sequence similarity 46, member C	FAM46C
203712_at	2.24	2.06	KIAA0020	KIAA0020
232696_at	2.40	2.06		
225737_s_at	2.04	2.06	F-box protein 22	FBXO22
AFFX-HUMRGE/M	2.16	2.05		
214751_at	2.10	2.05	zinc finger protein 468	ZNF468
213452_at	2.86	2.05	zinc finger protein 184	ZNF184
208895_s_at	2.06	2.05	DEAD (Asp-Glu-Ala-Asp) box polypeptide 18	DDX18
211792_s_at	2.01	2.05	cyclin-dependent kinase inhibitor 2C (p18, inhibits CDK4)	CDKN2C
222962_s_at	2.65	2.05	MCM10 minichromosome maintenance deficient 10 (S. cerevisiae)	MCM10

1554341_a_at	2.11	2.05		
217564_s_at	2.95	2.05	carbamoyl-phosphate synthetase 1, mitochondrial	CPS1
223559_s_at	2.02	2.05	chromosome 9 open reading frame 80	C9orf80
225062_at	2.32	2.05		
1564002_a_at	2.61	2.04	chromosome 6 open reading frame 199	C6orf199
1555439_at	2.22	2.04	general transcription factor IIIC, polypeptide 3, 102kDa	GTF3C3
225521_at	2.22	2.04	anaphase promoting complex subunit 7	ANAPC7
1568658_at	2.53	2.04		
231332_at	4.74	2.04		
204804_at	2.87	2.04	tripartite motif-containing 21	TRIM21
235456_at	2.97	2.04		
1555125_at	3.56	2.04	chromosome 21 open reading frame 66	C21orf66
238565_at	2.48	2.04		
241757_x_at	2.18	2.04	dynein, cytoplasmic 2, light intermediate chain 1	DYNC2LI1
213158_at	4.04	2.04		
213959_s_at	3.17	2.04		
218196_at	2.33	2.04	osteopetrosis associated transmembrane protein 1	OSTM1
220716_at	2.66	2.03		
219531_at	2.24	2.03	centrosomal protein 72kDa	CEP72
230329_s_at	2.75	2.03	nudix (nucleoside diphosphate linked moiety X)-type motif 6	NUDT6
202124_s_at	2.94	2.03	trafficking protein, kinesin binding 2	TRAK2
208066_s_at	2.60	2.03	general transcription factor IIB	GTF2B
238158_at	3.88	2.03	meiosis expressed gene 1 homolog (mouse)	MEIG1
243293_at	4.00	2.03		
221696_s_at	2.43	2.03	serine/threonine/tyrosine kinase 1	STYK1
242673_at	2.02	2.03	ubiquitin protein ligase E3C	UBE3C
230657_at	2.43	2.03		
1558390_at	31.94	2.03	zinc finger protein 599	ZNF599
236957_at	2.44	2.03	cell division cycle associated 2	CDCA2
239151_at	2.33	2.03	centaurin, gamma-like family, member 6	CTGLF6
219345_at	2.55	2.02	bolA homolog 1 (E. coli)	BOLA1
205130_at	2.31	2.02	renal tumor antigen	RAGE
202742_s_at	2.83	2.02	protein kinase, cAMP-dependent, catalytic, beta	PRKACB
1554606_at	2.28	2.02	coiled-coil domain containing 100	CCDC100
202893_at	2.49	2.02	unc-13 homolog B (C. elegans)	UNC13B
229338_at	2.59	2.02		
1553120_at	2.71	2.02	claspin homolog (Xenopus laevis)	CLSPN
231112_at	4.79	2.02	small nuclear ribonucleoprotein polypeptide E	SNRPE
222130_s_at	2.32	2.02	FtsJ homolog 2 (E. coli)	FTSJ2

1569652_at	2.28	2.02	myeloid/lymphoid or mixed-lineage leukemia (trithorax homolog, Drosophila); translocated to, 3	MLLT3
208896_at	2.25	2.02	DEAD (Asp-Glu-Ala-Asp) box polypeptide 18	DDX18
222851_at	2.24	2.02	zinc finger protein 654	ZNF654
242458_at	2.75	2.02	Ral GEF with PH domain and SH3 binding motif 2	RALGPS2
239346_at	2.57	2.02		
243038_at	3.14	2.02	RNA binding motif protein 43	RBM43
202705_at	2.67	2.01	cyclin B2	CCNB2
233020_at	2.23	2.01		
202577_s_at	2.44	2.01	DEAD (Asp-Glu-Ala-As) box polypeptide 19A	DDX19A
241741_at	2.30	2.01	cardiolipin synthase 1	CRLS1
227414_at	2.07	2.01	rhomboid domain containing 1	RHBDD1
224370_s_at	2.23	2.01	calcyphosine 2	CAPS2
227624_at	2.12	2.01	KIAA1546	KIAA1546
221025_x_at	3.59	2.00	pseudouridylate synthase 7 homolog (S. cerevisiae)-like	PUS7L
204525_at	2.50	2.00	PHD finger protein 14	PHF14
215128_at	2.35	2.00		
1559922_at	2.45	2.00		
224723_x_at	2.52	2.00		
211251_x_at	2.47	2.00	nuclear transcription factor Y, gamma	NFYC
1552684_a_at	2.59	2.00	SUMO/sentrin specific peptidase family member 8	SENP8
224474_x_at	2.53	2.00	SMEK homolog 2, suppressor of mek1 (Dictyostelium)	SMEK2
238831_at	4.17	2.00		
226913_s_at	-46.14	-76.20	SRY (sex determining region Y)-box 8	SOX8
242963_at	-110.21	-62.21		
208978_at	-40.17	-52.19	cysteine-rich protein 2	CRIP2
210612_s_at	-3.62	-49.69	synaptojanin 2	SYNJ2
221195_at	-2.03	-39.03		
1556067_a_at	-4.77	-37.32	jumonji domain containing 3	JMJD3
206251_s_at	-2.94	-30.28	arginine vasopressin receptor 1A	AVPR1A
223887_at	-25.78	-26.03	G protein-coupled receptor 132	GPR132
209683_at	-8.40	-24.14	family with sequence similarity 49, member A	FAM49A
228362_s_at	-3.15	-22.54		
203961_at	-5.93	-22.15	nebulette	NEBL
226084_at	-2.01	-21.79	microtubule-associated protein 1B	MAP1B
229830_at	-17.43	-21.61		
242316_at	-3.23	-21.20		
219049_at	-3.58	-20.25		
205398_s_at	-24.15	-19.88	SMAD family member 3	SMAD3
221530_s_at	-23.62	-19.77	basic helix-loop-helix domain containing, class B, 3	BHLHB3
232151_at	-27.85	-18.54		

227347_x_at	-21.06	-18.17	hairy and enhancer of split 4 (Drosophila)	HES4
1552334_at	-2.92	-17.25	TRIO and F-actin binding protein	TRIOBP
216322_at	-3.50	-16.00	CD58 molecule	CD58
210538_s_at	-33.87	-15.13	baculoviral IAP repeat-containing 3	BIRC3
227478_at	-9.12	-15.04	SET binding protein 1	SETBP1
235735_at	-31.79	-14.79		
226282_at	-7.71	-14.49		
223798_at	-26.28	-14.39	solute carrier family 41, member 2	SLC41A2
209278_s_at	-27.06	-14.32	tissue factor pathway inhibitor 2	TFPI2
1570622_at	-2.03	-14.23		
228885_at	-49.21	-13.91	ribosomal protein L24	RPL24
223520_s_at	-3.01	-13.64	kinesin family member 13A	KIF13A
235359_at	-47.75	-13.16	leucine rich repeat containing 33	LRRC33
202404_s_at	-18.69	-13.08	collagen, type I, alpha 2	COL1A2
219878_s_at	-33.40	-12.76	Kruppel-like factor 13	KLF13
205419_at	-48.28	-12.70	Epstein-Barr virus induced gene 2 (lymphocyte-specific G protein-coupled receptor)	EBI2
211986_at	-9.47	-12.35	AHNAK nucleoprotein (desmoyokin)	AHNAK
205463_s_at	-18.46	-12.07	platelet-derived growth factor alpha polypeptide	PDGFA
1563135_at	-22.89	-12.05		
221565_s_at	-23.55	-11.86	family with sequence similarity 26, member B	FAM26B
35626_at	-5.38	-11.54	N-sulfoglucosamine sulfohydrolase (sulfamidase)	SGSH
224393_s_at	-28.66	-11.19	cat eye syndrome chromosome region, candidate 6	CECR6
227396_at	-9.76	-11.18		
210029_at	-18.98	-10.92	indoleamine-pyrrole 2,3 dioxygenase	INDO
1568623_a_at	-215.69	-10.83	solute carrier family 35, member E4	SLC35E4
230980_x_at	-3.62	-10.82		
212464_s_at	-3.40	-10.76	fibronectin 1	FN1
212827_at	-10.42	-10.55	immunoglobulin heavy constant mu	IGHM
227233_at	-4.88	-10.46	tetraspanin 2	TSPAN2
228188_at	-4.74	-10.29	FOS-like antigen 2	FOSL2
226189_at	-13.24	-10.12	integrin, beta 8	ITGB8
202403_s_at	-16.01	-10.05	collagen, type I, alpha 2	COL1A2
234455_at	-3.52	-9.98	zinc finger protein 1 homolog (mouse)	ZFP1
202207_at	-21.32	-9.74	ADP-ribosylation factor-like 4C	ARL4C
202436_s_at	-7.03	-9.69	cytochrome P450, family 1, subfamily B, polypeptide 1	CYP1B1
240533_at	-2.14	-9.56		
221843_s_at	-19.59	-9.45	KIAA1609	KIAA1609
244533_at	-5.69	-9.44		
213839_at	-12.94	-9.33		
204908_s_at	-42.26	-9.10	B-cell CLL/lymphoma 3	BCL3
228054_at	-5.42	-8.97	transmembrane protein 44	TMEM44
230050_at	-2.56	-8.87	BTB (POZ) domain containing 14A	BTBD14A
226679_at	-8.65	-8.66	solute carrier family 26, member 11	SLC26A11

204567_s_at	-8.69	-8.61	ATP-binding cassette, sub-family G (WHITE), member 1	ABCG1
223474_at	-12.61	-8.59	chromosome 14 open reading frame 4	C14orf4
224989_at	-3.95	-8.54		
203045_at	-133.34	-8.44	ninjurin 1	NINJ1
235874_at	-22.90	-8.39	protease, serine, 35	PRSS35
209277_at	-37.33	-8.31	tissue factor pathway inhibitor 2	TFPI2
201324_at	-18.34	-8.31	epithelial membrane protein 1	EMP1
201170_s_at	-22.99	-8.27	basic helix-loop-helix domain containing, class B, 2	BHLHB2
230913_at	-21.32	-8.27		
229728_at	-5.47	-8.26		
201325_s_at	-28.89	-8.21	epithelial membrane protein 1	EMP1
242814_at	-13.41	-8.14	serpin peptidase inhibitor, clade B (ovalbumin), member 9	SERPINB9
225615_at	-67.29	-8.13		
202948_at	-18.86	-8.13	interleukin 1 receptor, type I	IL1R1
228042_at	-5.89	-8.03	ADP-ribosylarginine hydrolase	ADPRH
227038_at	-14.14	-7.91		
207216_at	-6.75	-7.85	tumor necrosis factor (ligand) superfamily, member 8	TNFSF8
230493_at	-63.61	-7.81	transmembrane protein 46	TMEM46
226530_at	-26.24	-7.80	Bcl2 modifying factor	BMF
226490_at	-3.08	-7.68	NHS-like 1	NHSL1
1568619_s_at	-5.00	-7.67		
221016_s_at	-13.88	-7.62	transcription factor 7-like 1 (T-cell specific, HMG-box)	TCF7L1
242321_at	-5.96	-7.44		
214255_at	-10.83	-7.42	ATPase, Class V, type 10A	ATP10A
221234_s_at	-14.42	-7.41	BTB and CNC homology 1, basic leucine zipper transcription factor 2	BACH2
201373_at	-7.31	-7.41	plectin 1, intermediate filament binding protein 500kDa	PLEC1
227923_at	-7.39	-7.39	SH3 and multiple ankyrin repeat domains 3	SHANK3
227052_at	-10.82	-7.36		
206618_at	-4.20	-7.33	interleukin 18 receptor 1	IL18R1
226056_at	-6.56	-7.31		
244481_at	-3.72	-7.29	solute carrier family 25 (mitochondrial carrier; phosphate carrier), member 24	SLC25A24
202357_s_at	-7.85	-7.27	complement factor B	CFB
231779_at	-6.98	-7.23	interleukin-1 receptor-associated kinase 2	IRAK2
229687_s_at	-4.44	-7.20		
1556423_at	-5.31	-7.17	vasohibin 1	VASH1
219383_at	-4.85	-7.13		
212647_at	-2.58	-7.09	related RAS viral (r-ras) oncogene homolog	RRAS
235306_at	-3.24	-7.02	GTPase, IMAP family member 8	GIMAP8
227329_at	-22.93	-7.01	zinc finger and BTB domain containing 46	ZBTB46

235264_at	-2.71	-6.95		
202071_at	-10.61	-6.92	syndecan 4 (amphiglycan, ryudocan)	SDC4
202464_s_at	-17.97	-6.89	6-phosphofructo-2-kinase/fructose-2,6-biphosphatase 3	PFKFB3
234989_at	-23.17	-6.82		
224516_s_at	-16.94	-6.80	CXXC finger 5	CXXC5
219563_at	-19.69	-6.80	chromosome 14 open reading frame 139	C14orf139
202342_s_at	-4.41	-6.77	tripartite motif-containing 2	TRIM2
237516_at	-3.13	-6.74		
221773_at	-12.19	-6.74	ELK3, ETS-domain protein (SRF accessory protein 2)	ELK3
206924_at	-11.84	-6.69	interleukin 11	IL11
241396_at	-7.10	-6.69	neural precursor cell expressed, developmentally down-regulated 4-like	NEDD4L
233955_x_at	-12.99	-6.67	CXXC finger 5	CXXC5
225407_at	-6.80	-6.64	myelin basic protein	MBP
207176_s_at	-14.21	-6.61	CD80 molecule	CD80
204446_s_at	-5.80	-6.59	arachidonate 5-lipoxygenase	ALOX5
1569104_a_at	-2.18	-6.54		
211434_s_at	-7.56	-6.53	chemokine (C-C motif) receptor-like 2	CCRL2
212828_at	-16.31	-6.53	synaptojanin 2	SYNJ2
223340_at	-9.52	-6.51	spastic paraplegia 3A (autosomal dominant)	SPG3A
225390_s_at	-12.85	-6.47	Kruppel-like factor 13	KLF13
227236_at	-6.25	-6.45	tetraspanin 2	TSPAN2
227367_at	-5.88	-6.41		
235916_at	-8.18	-6.33	yippee-like 4 (Drosophila)	YPEL4
228620_at	-13.32	-6.28		
202435_s_at	-6.69	-6.27		
1569149_at	-20.36	-6.27	PDZ and LIM domain 7 (enigma)	PDLIM7
58780_s_at	-9.72	-6.25		
231283_at	-5.75	-6.24	mannosyl (alpha-1,3-)-glycoprotein beta-1,4-N-acetylglucosaminyltransferase, isozyme A	MGAT4A
205599_at	-23.41	-6.23	TNF receptor-associated factor 1	TRAF1
234994_at	-6.83	-6.22	KIAA1913	KIAA1913
235422_at	-12.84	-6.19		
1552564_at	-2.34	-6.18	nudix (nucleoside diphosphate linked moiety X)-type motif 9 pseudogene 1	NUDT9P1
232081_at	-2.35	-6.16		
228471_at	-5.99	-6.08	ankyrin repeat domain 44	ANKRD44
226041_at	-6.65	-6.08		
204198_s_at	-9.26	-6.06	runt-related transcription factor 3	RUNX3
1553153_at	-4.17	-6.01	ATPase, H+ transporting, lysosomal 38kDa, V0 subunit d2	ATP6V0D2
234986_at	-5.77	-6.00		
213038_at	-4.61	-6.00	IBR domain containing 3	IBRDC3
203521_s_at	-5.41	-5.99	zinc finger protein 318	ZNF318
226382_at	-8.12	-5.98		

201502_s_at	-19.82	-5.96	nuclear factor of kappa light polypeptide gene enhancer in B-cells inhibitor, alpha	NFKBIA
205180_s_at	-7.71	-5.94	ADAM metallopeptidase domain 8	ADAM8
225239_at	-15.45	-5.91		
207986_x_at	-2.93	-5.91	cytochrome b-561	CYB561
240232_at	-3.96	-5.89	chromosome 3 open reading frame 1	C3orf1
231732_at	-11.00	-5.84	sphingomyelin phosphodiesterase 3, neutral membrane (neutral sphingomyelinase II)	SMPD3
227544_at	-10.30	-5.81	chromosome 14 open reading frame 83	C14orf83
230499_at	-9.69	-5.81		
219256_s_at	-13.77	-5.80	SH3 domain and tetratricopeptide repeats 1	SH3TC1
228450_at	-3.01	-5.80	pleckstrin homology domain containing, family A member 7	PLEKHA7
205081_at	-6.24	-5.78	cysteine-rich protein 1 (intestinal)	CRIP1
228770_at	-27.15	-5.76	G protein-coupled receptor 146	GPR146
217853_at	-13.94	-5.74	tensin 3	TNS3
222996_s_at	-12.98	-5.72	CXXC finger 5	CXXC5
204197_s_at	-12.08	-5.71	runt-related transcription factor 3	RUNX3
237753_at	-4.15	-5.69		
202627_s_at	-6.32	-5.68	serpin peptidase inhibitor, clade E (nexin, plasminogen activator inhibitor type 1), member 1	SERPINE1
243894_at	-24.61	-5.64	solute carrier family 41, member 2	SLC41A2
218284_at	-10.79	-5.62	SMAD family member 3	SMAD3
235116_at	-25.32	-5.59	TNF receptor-associated factor 1	TRAF1
202910_s_at	-23.06	-5.58	CD97 molecule	CD97
226509_at	-3.10	-5.56	zinc finger protein 641	ZNF641
205120_s_at	-7.54	-5.51	sarcoglycan, beta (43kDa dystrophin-associated glycoprotein)	SGCB
225612_s_at	-13.90	-5.49	UDP-GlcNAc:betaGal beta-1,3-N-acetylglucosaminyltransferase 5	B3GNT5
229841_at	-3.99	-5.46	eukaryotic translation initiation factor 2C, 2	EIF2C2
225971_at	-5.36	-5.45		
209959_at	-6.51	-5.44	nuclear receptor subfamily 4, group A, member 3	NR4A3
225251_at	-10.21	-5.43	RAB24, member RAS oncogene family	RAB24
220494_s_at	-9.91	-5.42		
203556_at	-13.75	-5.39	zinc fingers and homeoboxes 2	ZHX2
202437_s_at	-6.63	-5.37	cytochrome P450, family 1, subfamily B, polypeptide 1	CYP1B1
243767_at	-2.82	-5.37		
227314_at	-9.15	-5.35	integrin, alpha 2 (CD49B, alpha 2 subunit of VLA-2 receptor)	ITGA2
237444_at	-3.07	-5.34		
203234_at	-6.89	-5.33	uridine phosphorylase 1	UPP1
238790_at	-3.72	-5.32		
203845_at	-7.44	-5.32	p300/CBP-associated factor	PCAF

206552_s_at	-6.93	-5.31	tachykinin, precursor 1 (substance K, substance P, neurokinin 1, neurokinin 2, neuromedin L, neuroki	TAC1
236125_at	-3.10	-5.30		
217936_at	-4.32	-5.30	Rho GTPase activating protein 5	ARHGAP5
225980_at	-9.43	-5.30	chromosome 14 open reading frame 43	C14orf43
210271_at	-4.92	-5.29	neurogenic differentiation 2	NEUROD2
213361_at	-7.96	-5.27	tudor domain containing 7	TDRD7
1555963_x_at	-2.85	-5.27	UDP-GlcNAc:betaGal beta-1,3-N-acetylglucosaminyltransferase 7	B3GNT7
224990_at	-8.61	-5.27	chromosome 4 open reading frame 34	C4orf34
207072_at	-80.55	-5.27	interleukin 18 receptor accessory protein	IL18RAP
223681_s_at	-3.10	-5.22	InaD-like (Drosophila)	INADL
212993_at	-6.84	-5.21		
203232_s_at	-9.82	-5.21	ataxin 1	ATXN1
241762_at	-13.00	-5.19	F-box protein 32	FBXO32
226055_at	-7.87	-5.18	arrestin domain containing 2	ARRDC2
218532_s_at	-5.83	-5.18		
207113_s_at	-5.68	-5.17	tumor necrosis factor (TNF superfamily, member 2)	TNF
228714_at	-4.21	-5.16		
202191_s_at	-7.12	-5.14	growth arrest-specific 7	GAS7
226748_at	-14.47	-5.13	LysM, putative peptidoglycan-binding, domain containing 2	LYSMD2
232322_x_at	-16.44	-5.11	START domain containing 10	STARD10
226991_at	-6.41	-5.09	nuclear factor of activated T-cells, cytoplasmic, calcineurin-dependent 2	NFATC2
209723_at	-5.74	-5.08	serpin peptidase inhibitor, clade B (ovalbumin), member 9	SERPINB9
235299_at	-7.42	-5.06		
242229_at	-5.26	-5.05		
231406_at	-7.17	-5.03		
223674_s_at	-2.32	-5.03	CDC42 small effector 1	CDC42SE1
1556332_at	-3.74	-5.03		
225227_at	-14.50	-5.01		
227792_at	-6.76	-4.99		
213222_at	-7.95	-4.97	phospholipase C, beta 1 (phosphoinositide-specific)	PLCB1
230170_at	-4.75	-4.97	oncostatin M	OSM
230563_at	-5.27	-4.97	RasGEF domain family, member 1A	RASGEF1A
209946_at	-4.28	-4.97	vascular endothelial growth factor C	VEGFC
221012_s_at	-11.51	-4.96	tripartite motif-containing 8	TRIM8
244654_at	-11.50	-4.95	myosin IG	MYO1G
207826_s_at	-46.53	-4.95	inhibitor of DNA binding 3, dominant negative helix-loop-helix protein	ID3
203761_at	-7.51	-4.94	Src-like-adaptor	SLA
223228_at	-10.42	-4.93	leucine zipper, down-regulated in cancer 1-like	LDOC1L
36564_at	-2.76	-4.92	IBR domain containing 3	IBRDC3

212108_at	-8.15	-4.91	UBX domain containing 8	UBXD8
225628_s_at	-3.12	-4.90	myeloid/lymphoid or mixed-lineage leukemia (trithorax homolog, Drosophila); translocated to, 6	MLLT6
203851_at	-5.67	-4.90	insulin-like growth factor binding protein 6	IGFBP6
202887_s_at	-15.22	-4.89	DNA-damage-inducible transcript 4	DDIT4
221534_at	-11.41	-4.88	chromosome 11 open reading frame 68	C11orf68
244808_at	-2.06	-4.88	GRAM domain containing 1A	GRAMD1A
217650_x_at	-8.50	-4.86	ST3 beta-galactoside alpha-2,3-sialyltransferase 2	ST3GAL2
236293_at	-4.31	-4.84		
222061_at	-2.74	-4.84	CD58 molecule	CD58
206337_at	-6.97	-4.84	chemokine (C-C motif) receptor 7	CCR7
205935_at	-3.85	-4.82	forkhead box F1	FOXF1
229691_at	-12.61	-4.81		
230741_at	-4.42	-4.79		
203927_at	-11.97	-4.79	nuclear factor of kappa light polypeptide gene enhancer in B-cells inhibitor, epsilon	NFKBIE
212099_at	-5.48	-4.79	ras homolog gene family, member B	RHOB
226504_at	-7.92	-4.78	family with sequence similarity 109, member B	FAM109B
202073_at	-8.83	-4.78	optineurin	OPTN
242635_s_at	-4.79	-4.76		
205032_at	-6.39	-4.76	integrin, alpha 2 (CD49B, alpha 2 subunit of VLA-2 receptor)	ITGA2
212642_s_at	-3.91	-4.76	human immunodeficiency virus type I enhancer binding protein 2	HIVEP2
229354_at	-9.14	-4.75	aryl-hydrocarbon receptor repressor	AHRR
226438_at	-8.73	-4.74		
55081_at	-6.54	-4.73	MICAL-like 1	MICALL1
210933_s_at	-2.06	-4.73	fascin homolog 1, actin-bundling protein (Strongylocentrotus purpuratus)	FSCN1
203320_at	-11.59	-4.73	SH2B adaptor protein 3	SH2B3
227410_at	-4.70	-4.70	family with sequence similarity 43, member A	FAM43A
223472_at	-15.40	-4.69	Wolf-Hirschhorn syndrome candidate 1	WHSC1
210017_at	-14.05	-4.69	mucosa associated lymphoid tissue lymphoma translocation gene 1	MALT1
202628_s_at	-5.57	-4.68	serpin peptidase inhibitor, clade E (nexin, plasminogen activator inhibitor type 1), member 1	SERPINE1
214724_at	-3.52	-4.66	DIX domain containing 1	DIXDC1
205346_at	-3.74	-4.66	ST3 beta-galactoside alpha-2,3-sialyltransferase 2	ST3GAL2
228145_s_at	-9.03	-4.66	zinc finger protein 398	ZNF398
1555311_at	-4.79	-4.66		
226202_at	-4.64	-4.64	zinc finger protein 398	ZNF398

238778_at	-4.65	-4.64	membrane protein, palmitoylated 7 (MAGUK p55 subfamily member 7)	MPP7
218764_at	-6.65	-4.64	protein kinase C, eta	PRKCH
204794_at	-4.57	-4.63	dual specificity phosphatase 2	DUSP2
1555486_a_at	-6.75	-4.61		
211067_s_at	-4.26	-4.61	growth arrest-specific 7	GAS7
211113_s_at	-3.20	-4.59	ATP-binding cassette, sub-family G (WHITE), member 1	ABCG1
223179_at	-9.39	-4.59	yippee-like 3 (Drosophila)	YPEL3
210136_at	-5.41	-4.57	myelin basic protein	MBP
201005_at	-2.92	-4.56	CD9 molecule	CD9
227062_at	-17.66	-4.56		
207196_s_at	-7.49	-4.55	TNFAIP3 interacting protein 1	TNIP1
1555689_at	-11.96	-4.53	CD80 molecule	CD80
204802_at	-6.27	-4.53	Ras-related associated with diabetes	RRAD
230104_s_at	-6.07	-4.52		
203695_s_at	-4.15	-4.52	deafness, autosomal dominant 5	DFNA5
202393_s_at	-10.08	-4.51	Kruppel-like factor 10	KLF10
209124_at	-4.92	-4.50	myeloid differentiation primary response gene (88)	MYD88
210041_s_at	-4.55	-4.50	phosphoglucomutase 3	PGM3
225136_at	-8.64	-4.50	pleckstrin homology domain containing, family A (phosphoinositide binding specific) member 2	PLEKHA2
201578_at	-3.33	-4.49	podocalyxin-like	PODXL
235626_at	-5.83	-4.49	calcium/calmodulin-dependent protein kinase ID	CAMK1D
230643_at	-21.81	-4.49	wingless-type MMTV integration site family, member 9A	WNT9A
235479_at	-4.05	-4.48	cytoplasmic polyadenylation element binding protein 2	CPEB2
200872_at	-10.60	-4.48	S100 calcium binding protein A10	S100A10
203068_at	-30.39	-4.47	kelch-like 21 (Drosophila)	KLHL21
218699_at	-4.96	-4.47	RAB7, member RAS oncogene family-like 1	RAB7L1
212820_at	-4.24	-4.46	Dmx-like 2	DMXL2
213376_at	-9.74	-4.45	zinc finger and BTB domain containing 1	ZBTB1
1558340_at	-3.16	-4.45	DIX domain containing 1	DIXDC1
210258_at	-4.58	-4.45	regulator of G-protein signalling 13	RGS13
207571_x_at	-6.31	-4.45	chromosome 1 open reading frame 38	C1orf38
204790_at	-5.56	-4.44	SMAD family member 7	SMAD7
239678_at	-3.87	-4.44		
224917_at	-4.62	-4.44	transmembrane protein 49	TMEM49
204088_at	-4.13	-4.44	purinergic receptor P2X, ligand-gated ion channel, 4	P2RX4
226188_at	-3.98	-4.42		
225604_s_at	-3.02	-4.42	chromosome 9 open reading frame 19	C9orf19
230252_at	-7.68	-4.42	G protein-coupled receptor 92	GPR92

235276_at	-3.42	-4.40	epithelial stromal interaction 1 (breast)	EPSTI1
212124_at	-7.02	-4.39	zinc finger, MIZ-type containing 1	ZMIZ1
211352_s_at	-3.73	-4.37	nuclear receptor coactivator 3	NCOA3
204912_at	-6.91	-4.37	interleukin 10 receptor, alpha	IL10RA
225954_s_at	-2.14	-4.37	midnolin	MIDN
219695_at	-2.22	-4.36	sphingomyelin phosphodiesterase 3, neutral membrane (neutral sphingomyelinase II)	SMPD3
223159_s_at	-7.77	-4.36	NIMA (never in mitosis gene a)-related kinase 6	NEK6
31874_at	-5.95	-4.35	growth arrest-specific 2 like 1	GAS2L1
201473_at	-8.37	-4.34	jun B proto-oncogene	JUNB
224570_s_at	-6.85	-4.33		
227131_at	-10.42	-4.33	mitogen-activated protein kinase kinase kinase 3	MAP3K3
232979_at	-3.43	-4.32		
228257_at	-2.88	-4.32	ankyrin repeat domain 52	ANKRD52
223437_at	-3.10	-4.32	peroxisome proliferator-activated receptor alpha	PPARA
212774_at	-4.98	-4.32	zinc finger protein 238	ZNF238
217655_at	-2.25	-4.32	FXYD domain containing ion transport regulator 5	FXYD5
1553155_x_at	-2.15	-4.30	ATPase, H+ transporting, lysosomal 38kDa, V0 subunit d2	ATP6V0D2
203047_at	-8.23	-4.30	serine/threonine kinase 10	STK10
227261_at	-3.22	-4.30	Kruppel-like factor 12	KLF12
222453_at	-6.74	-4.30	cytochrome b reductase 1	CYBRD1
46665_at	-7.82	-4.30	sema domain, immunoglobulin domain (Ig), transmembrane domain (TM) and short cytoplasmic domain, (se	SEMA4C
208683_at	-6.32	-4.29	calpain 2, (m/II) large subunit	CAPN2
211685_s_at	-2.59	-4.29	neurocalcin delta	NCALD
200782_at	-6.61	-4.28	annexin A5	ANXA5
201642_at	-7.95	-4.28	interferon gamma receptor 2 (interferon gamma transducer 1)	IFNGR2
210117_at	-7.30	-4.27	sperm associated antigen 1	SPAG1
227948_at	-4.15	-4.26	FYVE, RhoGEF and PH domain containing 4	FGD4
218368_s_at	-5.71	-4.26	tumor necrosis factor receptor superfamily, member 12A	TNFRSF12A
224571_at	-10.12	-4.26	interferon regulatory factor 2 binding protein 2	IRF2BP2
232077_s_at	-7.04	-4.25		
218651_s_at	-5.23	-4.25	La ribonucleoprotein domain family, member 6	LARP6
229090_at	-5.38	-4.25		
204672_s_at	-5.01	-4.24	ankyrin repeat domain 6	ANKRD6
228242_at	-4.59	-4.23		
216248_s_at	-3.85	-4.22	nuclear receptor subfamily 4, group A, member 2	NR4A2
218404_at	-12.74	-4.22	sorting nexin 10	SNX10

225557_at	-4.04	-4.21	AXIN1 up-regulated 1	AXUD1
202206_at	-5.29	-4.21	ADP-ribosylation factor-like 4C	ARL4C
226893_at	-5.11	-4.20		
212845_at	-2.69	-4.19	sterile alpha motif domain containing 4A	SAMD4A
229033_s_at	-8.04	-4.19	melanoma associated antigen (mutated) 1	MUM1
228754_at	-9.03	-4.18	solute carrier family 6 (neurotransmitter transporter, taurine), member 6	SLC6A6
203037_s_at	-4.52	-4.18	metastasis suppressor 1	MTSS1
205511_at	-5.16	-4.18		
229391_s_at	-6.19	-4.17		
204698_at	-5.73	-4.17	interferon stimulated exonuclease gene 20kDa	ISG20
210785_s_at	-5.25	-4.16	chromosome 1 open reading frame 38	C1orf38
1569150_x_at	-6.45	-4.15	PDZ and LIM domain 7 (enigma)	PDLIM7
225214_at	-12.92	-4.15		
224923_at	-6.05	-4.14	tetratricopeptide repeat domain 7A	TTC7A
1554519_at	-18.91	-4.14	CD80 molecule	CD80
218223_s_at	-18.48	-4.14	pleckstrin homology domain containing, family O member 1	PLEKHO1
214888_at	-3.01	-4.14	calpain 2, (m/II) large subunit	CAPN2
235964_x_at	-4.73	-4.13	chromosome 20 open reading frame 118	C20orf118
212923_s_at	-6.87	-4.13	chromosome 6 open reading frame 145	C6orf145
212397_at	-6.67	-4.12	radixin	RDX
201964_at	-4.65	-4.12	senataxin	SETX
225922_at	-4.88	-4.12		
202085_at	-6.64	-4.12	tight junction protein 2 (zona occludens 2)	TJP2
223161_at	-3.44	-4.12	KIAA1147	KIAA1147
209374_s_at	-11.28	-4.12	immunoglobulin heavy constant mu	IGHM
225176_at	-4.23	-4.11		
1557036_at	-3.24	-4.11	zinc finger and BTB domain containing 1	ZBTB1
206600_s_at	-4.68	-4.09	solute carrier family 16, member 5 (monocarboxylic acid transporter 6)	SLC16A5
227107_at	-4.91	-4.09		
224832_at	-6.39	-4.08	dual specificity phosphatase 16	DUSP16
229396_at	-7.25	-4.08	ovo-like 1(Drosophila)	OVOL1
227645_at	-44.93	-4.07	phosphoinositide-3-kinase, regulatory subunit 5, p101	PIK3R5
227609_at	-5.66	-4.07	epithelial stromal interaction 1 (breast)	EPSTI1
205173_x_at	-8.66	-4.07	CD58 molecule	CD58
223514_at	-4.61	-4.06	caspase recruitment domain family, member 11	CARD11
212398_at	-3.13	-4.06	radixin	RDX
202172_at	-6.52	-4.06	vascular endothelial zinc finger 1	VEZF1

221011_s_at	-2.09	-4.05	limb bud and heart development homolog (mouse)	LBH
227582_at	-9.27	-4.05		
204622_x_at	-3.93	-4.05	nuclear receptor subfamily 4, group A, member 2	NR4A2
231202_at	-3.00	-4.05		
223027_at	-5.46	-4.04	sorting nexin 9	SNX9
236248_x_at	-9.63	-4.03		
241824_at	-2.56	-4.03	FOS-like antigen 2	FOSL2
212817_at	-4.11	-4.03	DnaJ (Hsp40) homolog, subfamily B, member 5	DNAJB5
225266_at	-3.59	-4.02	zinc finger protein 652	ZNF652
217997_at	-3.95	-4.01	pleckstrin homology-like domain, family A, member 1	PHLDA1
205945_at	-7.78	-4.01	interleukin 6 receptor	IL6R
230047_at	-4.26	-4.00		
226939_at	-5.59	-4.00	cytoplasmic polyadenylation element binding protein 2	CPEB2
207700_s_at	-3.11	-3.99	nuclear receptor coactivator 3	NCOA3
65438_at	-4.77	-3.99	KIAA1609	KIAA1609
224569_s_at	-8.53	-3.99	interferon regulatory factor 2 binding protein 2	IRF2BP2
225262_at	-4.45	-3.99	FOS-like antigen 2	FOSL2
212445_s_at	-3.48	-3.99	neural precursor cell expressed, developmentally down-regulated 4-like	NEDD4L
206283_s_at	-5.75	-3.98	T-cell acute lymphocytic leukemia 1	TAL1
203741_s_at	-7.02	-3.97	adenylate cyclase 7	ADCY7
228442_at	-8.34	-3.96		
221680_s_at	-3.23	-3.96	ets variant gene 7 (TEL2 oncogene)	ETV7
1553349_at	-2.36	-3.95	AT rich interactive domain 2 (ARID, RFX-like)	ARID2
205027_s_at	-9.95	-3.95	mitogen-activated protein kinase kinase kinase 8	MAP3K8
242738_s_at	-3.69	-3.95		
203542_s_at	-2.18	-3.95	Kruppel-like factor 9	KLF9
207426_s_at	-3.61	-3.94	tumor necrosis factor (ligand) superfamily, member 4 (tax-transcriptionally activated glycoprotein 1	TNFSF4
207266_x_at	-6.14	-3.93	RNA binding motif, single stranded interacting protein 1	RBMS1
214321_at	-8.40	-3.93	nephroblastoma overexpressed gene	NOV
229543_at	-7.94	-3.92		
221571_at	-5.74	-3.91	TNF receptor-associated factor 3	TRAF3
225803_at	-6.46	-3.91	F-box protein 32	FBXO32
224566_at	-7.40	-3.91		
203665_at	-8.60	-3.91	heme oxygenase (decycling) 1	HMOX1
225142_at	-3.03	-3.91		
207907_at	-74.14	-3.90	tumor necrosis factor (ligand) superfamily, member 14	TNFSF14
235079_at	-2.87	-3.90		

203006_at	-5.65	-3.89	inositol polyphosphate-5-phosphatase, 40kDa	INPP5A
222154_s_at	-4.65	-3.89		
232279_at	-2.56	-3.89	PHD finger protein 15	PHF15
241416_at	-8.74	-3.88		
220985_s_at	-9.98	-3.88	ring finger protein 170	RNF170
1565628_at	-3.90	-3.87		
229218_at	-2.46	-3.87	collagen, type I, alpha 2	COL1A2
1555355_a_at	-3.89	-3.86	v-ets erythroblastosis virus E26 oncogene homolog 1 (avian)	ETS1
1554593_s_at	-4.87	-3.86	solute carrier family 1 (high affinity aspartate/glutamate transporter), member 6	SLC1A6
238327_at	-5.58	-3.85		
225618_at	-9.76	-3.85	Rho GTPase activating protein 27	ARHGAP27
227697_at	-14.24	-3.84	suppressor of cytokine signaling 3	SOCS3
227290_at	-4.29	-3.84		
221778_at	-3.01	-3.83		
227391_x_at	-10.86	-3.83	leucine rich repeat (in FLII) interacting protein 1	LRRFIP1
223092_at	-3.21	-3.82	ankylosis, progressive homolog (mouse)	ANKH
203879_at	-5.48	-3.82	phosphoinositide-3-kinase, catalytic, delta polypeptide	PIK3CD
206513_at	-2.43	-3.81	absent in melanoma 2	AIM2
235094_at	-5.02	-3.81		
203839_s_at	-6.04	-3.81	tyrosine kinase, non-receptor, 2	TNK2
243356_at	-3.82	-3.81	family with sequence similarity 7, member A1	FAM7A1
218301_at	-2.41	-3.81	arginyl aminopeptidase (aminopeptidase B)-like 1	RNPEPL1
212811_x_at	-6.14	-3.80	solute carrier family 1 (glutamate/neutral amino acid transporter), member 4	SLC1A4
232693_s_at	-30.26	-3.80	zinc finger protein 395	ZNF395
236285_at	-11.66	-3.79		
209262_s_at	-48.24	-3.78	nuclear receptor subfamily 2, group F, member 6	NR2F6
209939_x_at	-4.77	-3.78	CASP8 and FADD-like apoptosis regulator	CFLAR
209061_at	-3.86	-3.78	nuclear receptor coactivator 3	NCOA3
232017_at	-6.63	-3.78	tight junction protein 2 (zona occludens 2)	TJP2
221753_at	-3.82	-3.78	slingshot homolog 1 (Drosophila)	SSH1
1554592_a_at	-10.99	-3.77	solute carrier family 1 (high affinity aspartate/glutamate transporter), member 6	SLC1A6
220118_at	-8.98	-3.76	zinc finger and BTB domain containing 32	ZBTB32
204980_at	-3.33	-3.76	clock homolog (mouse)	CLOCK
201109_s_at	-4.38	-3.76	thrombospondin 1	THBS1
219278_at	-5.41	-3.75	mitogen-activated protein kinase kinase kinase 6	MAP3K6

202565_s_at	-6.80	-3.75	supervillin	SVIL
230369_at	-15.78	-3.74	G protein-coupled receptor 161	GPR161
224565_at	-7.31	-3.73		
227112_at	-3.66	-3.73	transmembrane and coiled-coil domain family 1	TMCC1
212733_at	-4.04	-3.72	KIAA0226	KIAA0226
243495_s_at	-5.15	-3.72		
207375_s_at	-3.52	-3.72	interleukin 15 receptor, alpha	IL15RA
210871_x_at	-3.84	-3.71	synovial sarcoma, X breakpoint 2 interacting protein	SSX2IP
204352_at	-6.70	-3.70	TNF receptor-associated factor 5	TRAF5
236685_at	-3.51	-3.70		
231699_at	-3.40	-3.70	nuclear factor of kappa light polypeptide gene enhancer in B-cells inhibitor, alpha	NFKBIA
226333_at	-5.96	-3.70		
231697_s_at	-2.44	-3.68	transmembrane protein 49	TMEM49
202111_at	-2.52	-3.68	solute carrier family 4, anion exchanger, member 2 (erythrocyte membrane protein band 3-like 1)	SLC4A2
226039_at	-3.79	-3.68	mannosyl (alpha-1,3-)-glycoprotein beta-1,4-N-acetylglucosaminyltransferase, isozyme A	MGAT4A
57715_at	-5.73	-3.68	family with sequence similarity 26, member B	FAM26B
243869_at	-3.83	-3.68		
204436_at	-5.20	-3.67	pleckstrin homology domain containing, family Q member 1	PLEKHQ1
207765_s_at	-14.23	-3.67	KIAA1539	KIAA1539
226155_at	-3.76	-3.67	KIAA1600	KIAA1600
211744_s_at	-6.23	-3.67	CD58 molecule	CD58
237244_at	-4.90	-3.67		
211178_s_at	-4.47	-3.66	proline-serine-threonine phosphatase interacting protein 1	PSTPIP1
226685_at	-4.80	-3.66	syntrophin, beta 2 (dystrophin-associated protein A1, 59kDa, basic component 2)	SNTB2
231866_at	-3.28	-3.66	leucyl/cystinyl aminopeptidase	LNPEP
201455_s_at	-8.60	-3.65	aminopeptidase puromycin sensitive	NPEPPS
205590_at	-9.96	-3.65	RAS guanyl releasing protein 1 (calcium and DAG-regulated)	RASGRP1
213295_at	-3.60	-3.65	cylindromatosis (turban tumor syndrome)	CYLD
217538_at	-4.65	-3.65	RUN and TBC1 domain containing 1	RUTBC1
235072_s_at	-3.07	-3.65		
226460_at	-5.01	-3.64		
226100_at	-5.84	-3.64	myeloid/lymphoid or mixed-lineage leukemia 5 (trithorax homolog, Drosophila)	MLL5
212589_at	-2.17	-3.63	related RAS viral (r-ras) oncogene homolog 2	RRAS2
227632_at	-3.01	-3.63	TBC1 domain family, member 24	TBC1D24

1565599_at	-2.56	-3.63		
226673_at	-4.70	-3.63	SH2 domain containing 3C	SH2D3C
1563357_at	-3.39	-3.63		
209722_s_at	-5.27	-3.62	serpin peptidase inhibitor, clade B (ovalbumin), member 9	SERPINB9
239744_at	-4.18	-3.62		
220326_s_at	-10.50	-3.62		
218641_at	-2.38	-3.61		
212062_at	-3.15	-3.61	ATPase, Class II, type 9A	ATP9A
202459_s_at	-2.92	-3.61	lipin 2	LPIN2
1552295_a_at	-4.26	-3.61	solute carrier family 39 (zinc transporter), member 13	SLC39A13
235844_at	-3.41	-3.61	putative homeodomain transcription factor 1	PHTF1
227361_at	-5.58	-3.61	heparan sulfate (glucosamine) 3-O-sulfotransferase 3B1	HS3ST3B1
204401_at	-7.11	-3.61	potassium intermediate/small conductance calcium-activated channel, subfamily N, member 4	KCNN4
212441_at	-3.93	-3.60		
209409_at	-7.02	-3.60	growth factor receptor-bound protein 10	GRB10
226682_at	-4.59	-3.59		
201531_at	-8.53	-3.58	zinc finger protein 36, C3H type, homolog (mouse)	ZFP36
222079_at	-2.19	-3.58		
238001_at	-2.52	-3.58	potassium channel tetramerisation domain containing 6	KCTD6
201189_s_at	-14.08	-3.58	inositol 1,4,5-triphosphate receptor, type 3	ITPR3
230052_s_at	-6.44	-3.58		
230192_at	-8.44	-3.58	tripartite motif-containing 13	TRIM13
204495_s_at	-5.05	-3.58	chromosome 15 open reading frame 39	C15orf39
204205_at	-4.16	-3.57	apolipoprotein B mRNA editing enzyme, catalytic polypeptide-like 3G	APOBEC3G
212810_s_at	-6.87	-3.57	solute carrier family 1 (glutamate/neutral amino acid transporter), member 4	SLC1A4
209099_x_at	-2.84	-3.57	jagged 1 (Alagille syndrome)	JAG1
238597_at	-3.01	-3.57		
235362_at	-3.91	-3.56		
225265_at	-5.22	-3.56	RNA binding motif, single stranded interacting protein 1	RBMS1
218812_s_at	-7.06	-3.55	transmembrane protein 142B	TMEM142B
225269_s_at	-4.10	-3.55	RNA binding motif, single stranded interacting protein 1	RBMS1
213590_at	-2.90	-3.55	solute carrier family 16, member 5 (monocarboxylic acid transporter 6)	SLC16A5
226074_at	-3.82	-3.54	protein phosphatase 1M (PP2C domain containing)	PPM1M

217762_s_at	-2.81	-3.54	RAB31, member RAS oncogene family	RAB31
223164_at	-4.10	-3.54	cerebral cavernous malformation 2	CCM2
210357_s_at	-4.89	-3.54	spermine oxidase	SMOX
229390_at	-5.13	-3.54		
221718_s_at	-4.18	-3.53	A kinase (PRKA) anchor protein 13	AKAP13
216834_at	-6.71	-3.53	regulator of G-protein signalling 1	RGS1
226771_at	-3.31	-3.53	ATPase, Class I, type 8B, member 2	ATP8B2
229393_at	-2.94	-3.53	l(3)mbt-like 3 (Drosophila)	L3MBTL3
205051_s_at	-5.71	-3.53	v-kit Hardy-Zuckerman 4 feline sarcoma viral oncogene homolog	KIT
223484_at	-44.91	-3.53	chromosome 15 open reading frame 48	C15orf48
209824_s_at	-7.07	-3.52	aryl hydrocarbon receptor nuclear translocator-like	ARNTL
207643_s_at	-5.71	-3.52	tumor necrosis factor receptor superfamily, member 1A	TNFRSF1A
215945_s_at	-13.55	-3.52	tripartite motif-containing 2	TRIM2
202083_s_at	-3.35	-3.51	SEC14-like 1 (S. cerevisiae)	SEC14L1
200999_s_at	-6.82	-3.50	cytoskeleton-associated protein 4	CKAP4
209606_at	-4.10	-3.50	pleckstrin homology, Sec7 and coiled-coil domains, binding protein	PSCDBP
212641_at	-3.64	-3.49	human immunodeficiency virus type I enhancer binding protein 2	HIVEP2
226322_at	-5.27	-3.49	transmembrane and tetratricopeptide repeat containing 1	TMTC1
235199_at	-14.79	-3.49	ring finger protein 125	RNF125
232504_at	-5.10	-3.49		
219806_s_at	-7.74	-3.49	chromosome 11 open reading frame 75	C11orf75
225602_at	-3.79	-3.49	chromosome 9 open reading frame 19	C9orf19
225416_at	-4.04	-3.49	ring finger protein 12	RNF12
1569872_a_at	-7.74	-3.49		
238992_at	-5.41	-3.48	polymerase (DNA directed) iota	POLI
228762_at	-25.15	-3.48	LFNG O-fucosylpeptide 3-beta-N-acetylglucosaminyltransferase	LFNG
201169_s_at	-7.25	-3.47	basic helix-loop-helix domain containing, class B, 2	BHLHB2
200759_x_at	-3.02	-3.47	nuclear factor (erythroid-derived 2)-like 1	NFE2L1
204689_at	-5.58	-3.47	homeobox, hematopoietically expressed	HHEX
33304_at	-5.03	-3.47	interferon stimulated exonuclease gene 20kDa	ISG20
218421_at	-4.30	-3.47	ceramide kinase	CERK
215078_at	-5.02	-3.46	superoxide dismutase 2, mitochondrial	SOD2
1569257_at	-9.59	-3.46	formin-like 1	FMNL1
230440_at	-5.32	-3.46	zinc finger protein 469	ZNF469
204621_s_at	-3.12	-3.46	nuclear receptor subfamily 4, group A, member 2	NR4A2
235521_at	-2.28	-3.46	homeobox A3	HOXA3

226641_at	-6.15	-3.45		
222858_s_at	-2.17	-3.45	dual adaptor of phosphotyrosine and 3-phosphoinositides	DAPP1
230733_at	-5.23	-3.45		
212543_at	-4.76	-3.44	absent in melanoma 1	AIM1
218723_s_at	-12.38	-3.44		
210563_x_at	-5.69	-3.44	CASP8 and FADD-like apoptosis regulator	CFLAR
222101_s_at	-3.19	-3.44	dachsous 1 (Drosophila)	DCHS1
227761_at	-2.04	-3.43	myosin VA (heavy chain 12, myoxin)	MYO5A
223190_s_at	-3.17	-3.43	myeloid/lymphoid or mixed-lineage leukemia 5 (trithorax homolog, Drosophila)	MLL5
226715_at	-2.44	-3.43	forkhead box K1	FOXK1
206672_at	-6.04	-3.42	aquaporin 2 (collecting duct)	AQP2
202171_at	-6.33	-3.42	vascular endothelial zinc finger 1	VEZF1
236826_at	-10.66	-3.42	chromosome 9 open reading frame 52	C9orf52
225232_at	-4.39	-3.42	myotubularin related protein 12	MTMR12
207091_at	-6.85	-3.42	purinergic receptor P2X, ligand-gated ion channel, 7	P2RX7
239058_at	-6.45	-3.42		
231963_at	-4.32	-3.42		
227728_at	-2.30	-3.42		
233849_s_at	-3.40	-3.42	Rho GTPase activating protein 5	ARHGAP5
204684_at	-4.71	-3.40	neuronal pentraxin I	NPTX1
1556064_at	-8.15	-3.40		
213541_s_at	-4.23	-3.40	v-ets erythroblastosis virus E26 oncogene homolog (avian)	ERG
226497_s_at	-3.87	-3.40		
229943_at	-3.15	-3.40	tripartite motif-containing 13	TRIM13
210845_s_at	-5.44	-3.39	plasminogen activator, urokinase receptor	PLAUR
1566764_at	-6.28	-3.38		
212122_at	-3.62	-3.38	ras homolog gene family, member Q	RHOQ
211317_s_at	-3.67	-3.38	CASP8 and FADD-like apoptosis regulator	CFLAR
239213_at	-6.48	-3.38	serpin peptidase inhibitor, clade B (ovalbumin), member 1	SERPINB1
232937_at	-2.39	-3.38		
225144_at	-3.92	-3.37	bone morphogenetic protein receptor, type II (serine/threonine kinase)	BMPR2
212606_at	-19.02	-3.37	WD repeat and FYVE domain containing 3	WDFY3
209295_at	-4.05	-3.37	tumor necrosis factor receptor superfamily, member 10b	TNFRSF10B
229307_at	-3.46	-3.36	ankyrin repeat domain 28	ANKRD28
209304_x_at	-5.80	-3.36	growth arrest and DNA-damage-inducible, beta	GADD45B
238725_at	-4.38	-3.36		
1555977_at	-4.07	-3.35		
212688_at	-5.89	-3.35	phosphoinositide-3-kinase, catalytic, beta polypeptide	PIK3CB

205278_at	-4.09	-3.35	glutamate decarboxylase 1 (brain, 67kDa)	GAD1
214352_s_at	-5.61	-3.35	v-Ki-ras2 Kirsten rat sarcoma viral oncogene homolog	KRAS
1569703_a_at	-6.60	-3.35		
227106_at	-2.53	-3.34		
206127_at	-23.69	-3.34	ELK3, ETS-domain protein (SRF accessory protein 2)	ELK3
209920_at	-2.64	-3.34	bone morphogenetic protein receptor, type II (serine/threonine kinase)	BMPR2
237105_at	-16.47	-3.33		
210123_s_at	-2.62	-3.33	cholinergic receptor, nicotinic, alpha 7	CHRNA7
224704_at	-3.15	-3.33	trinucleotide repeat containing 6A	TNRC6A
229739_s_at	-3.80	-3.32	family with sequence similarity 116, member B	FAM116B
201462_at	-5.28	-3.32	secernin 1	SCRN1
212675_s_at	-4.25	-3.31	centrosomal protein 68kDa	CEP68
226423_at	-7.70	-3.31	progestin and adipoQ receptor family member VIII	PAQR8
203574_at	-4.81	-3.30	nuclear factor, interleukin 3 regulated	NFIL3
204951_at	-3.47	-3.30	ras homolog gene family, member H	RHOH
214377_s_at	-6.39	-3.30	chymotrypsin-like	CTRL
203726_s_at	-2.97	-3.30	laminin, alpha 3	LAMA3
205453_at	-3.39	-3.30	homeobox B2	HOXB2
212308_at	-2.13	-3.29	cytoplasmic linker associated protein 2	CLASP2
236769_at	-3.00	-3.29		
235670_at	-6.92	-3.29		
201331_s_at	-5.29	-3.29	signal transducer and activator of transcription 6, interleukin-4 induced	STAT6
223162_s_at	-5.72	-3.28	KIAA1147	KIAA1147
209610_s_at	-7.97	-3.28	solute carrier family 1 (glutamate/neutral amino acid transporter), member 4	SLC1A4
208622_s_at	-3.16	-3.28		
39582_at	-3.67	-3.28	cylindromatosis (turban tumor syndrome)	CYLD
226240_at	-6.78	-3.28		
210360_s_at	-2.33	-3.27	metastasis suppressor 1	MTSS1
1552667_a_at	-3.37	-3.27	SH2 domain containing 3C	SH2D3C
228032_s_at	-2.80	-3.27		
235048_at	-4.40	-3.27		
201000_at	-4.59	-3.26	alanyl-tRNA synthetase	AARS
221752_at	-8.75	-3.26	slingshot homolog 1 (Drosophila)	SSH1
213112_s_at	-3.41	-3.26	sequestosome 1	SQSTM1
224739_at	-4.18	-3.26	pim-3 oncogene	PIM3
236104_at	-6.94	-3.26		
201566_x_at	-2.74	-3.26	inhibitor of DNA binding 2, dominant negative helix-loop-helix protein	ID2
217529_at	-6.82	-3.26		
209163_at	-8.00	-3.26	cytochrome b-561	CYB561

225305_at	-9.00	-3.26	solute carrier family 25, member 29	SLC25A29
219680_at	-10.82	-3.26	NLR family member X1	NLRX1
204036_at	-6.83	-3.25	endothelial differentiation, lysophosphatidic acid G-protein-coupled receptor, 2	EDG2
226075_at	-7.20	-3.25	splA/ryanodine receptor domain and SOCS box containing 1	SPSB1
221755_at	-5.95	-3.25	EH domain binding protein 1-like 1	EHBP1L1
223366_at	-3.06	-3.24		
217419_x_at	-2.07	-3.24	agrin	AGRIN
225627_s_at	-5.13	-3.24	cache domain containing 1	CACHD1
209930_s_at	-11.84	-3.24	nuclear factor (erythroid-derived 2), 45kDa	NFE2
235230_at	-3.04	-3.23		
209403_at	-6.85	-3.23	TBC1 domain family, member 3	TBC1D3
214104_at	-3.91	-3.23	G protein-coupled receptor 161	GPR161
212912_at	-2.14	-3.23	ribosomal protein S6 kinase, 90kDa, polypeptide 2	RPS6KA2
232155_at	-4.35	-3.22	KIAA1618	KIAA1618
223950_s_at	-10.46	-3.22	FLYWCH-type zinc finger 1	FLYWCH1
215127_s_at	-3.71	-3.22	RNA binding motif, single stranded interacting protein 1	RBMS1
220246_at	-47.19	-3.22	calcium/calmodulin-dependent protein kinase ID	CAMK1D
212859_x_at	-5.40	-3.22	metallothionein 1E (functional)	MT1E
1553644_at	-3.72	-3.22	chromosome 14 open reading frame 49	C14orf49
203823_at	-4.20	-3.21	regulator of G-protein signalling 3	RGS3
227489_at	-2.76	-3.21	SMAD specific E3 ubiquitin protein ligase 2	SMURF2
225128_at	-3.23	-3.21	KDEL (Lys-Asp-Glu-Leu) containing 2	KDELC2
224764_at	-7.60	-3.20	Rho GTPase activating protein 21	ARHGAP21
243641_at	-5.34	-3.20		
1555938_x_at	-8.89	-3.20	vimentin	VIM
235529_x_at	-3.39	-3.20		
224663_s_at	-10.21	-3.20	cofilin 2 (muscle)	CFL2
205801_s_at	-2.85	-3.20	RAS guanyl releasing protein 3 (calcium and DAG-regulated)	RASGRP3
226112_at	-3.99	-3.20	sarcoglycan, beta (43kDa dystrophin-associated glycoprotein)	SGCB
224833_at	-4.27	-3.19	v-ets erythroblastosis virus E26 oncogene homolog 1 (avian)	ETS1
227599_at	-4.62	-3.19	chromosome 3 open reading frame 59	C3orf59
238846_at	-6.41	-3.19	tumor necrosis factor receptor superfamily, member 11a, NFKB activator	TNFRSF11A
239442_at	-2.90	-3.18	centrosomal protein 68kDa	CEP68
224793_s_at	-7.27	-3.18	transforming growth factor, beta receptor I (activin A receptor type II-like kinase, 53kDa)	TGFBR1

210094_s_at	-3.08	-3.17	par-3 partitioning defective 3 homolog (C. elegans)	PARD3
202762_at	-5.95	-3.17	Rho-associated, coiled-coil containing protein kinase 2	ROCK2
230748_at	-2.70	-3.17		
226795_at	-3.66	-3.17		
223322_at	-7.05	-3.17	Ras association (RalGDS/AF-6) domain family 5	RASSF5
226867_at	-3.59	-3.17	DENN/MADD domain containing 4C	DENND4C
204009_s_at	-6.24	-3.16	v-Ki-ras2 Kirsten rat sarcoma viral oncogene homolog	KRAS
226104_at	-3.20	-3.16		
205139_s_at	-2.39	-3.16	uronyl-2-sulfotransferase	UST
212959_s_at	-5.96	-3.16	N-acetylglucosamine-1-phosphate transferase, alpha and beta subunits	GNPTAB
243601_at	-3.06	-3.15		
224958_at	-2.68	-3.15	nuclear fragile X mental retardation protein interacting protein 2	NUFIP2
209604_s_at	-3.17	-3.15	GATA binding protein 3	GATA3
224983_at	-6.37	-3.15	scavenger receptor class B, member 2	SCARB2
1553151_at	-6.35	-3.15	ATPase, H+ transporting, lysosomal 38kDa, V0 subunit d2	ATP6V0D2
212561_at	-4.00	-3.14	RAB6 interacting protein 1	RAB6IP1
219636_s_at	-2.25	-3.14	armadillo repeat containing 9	ARMC9
213766_x_at	-6.02	-3.14	guanine nucleotide binding protein (G protein), alpha 11 (Gq class)	GNA11
40420_at	-5.87	-3.14	serine/threonine kinase 10	STK10
206589_at	-2.85	-3.13	growth factor independent 1	GFI1
205569_at	-3.99	-3.13	lysosomal-associated membrane protein 3	LAMP3
226677_at	-2.93	-3.13	zinc finger protein 521	ZNF521
239376_at	-3.63	-3.13		
221864_at	-4.10	-3.13		
204249_s_at	-2.21	-3.13	LIM domain only 2 (rhombotin-like 1)	LMO2
1555912_at	-3.10	-3.13	ST7 overlapping transcript 1 (antisense non-coding RNA)	ST7OT1
230179_at	-4.52	-3.12		
212660_at	-6.20	-3.12	PHD finger protein 15	PHF15
209541_at	-6.21	-3.12		
224466_s_at	-4.24	-3.12	v-maf musculoaponeurotic fibrosarcoma oncogene homolog G (avian)	MAFG
201631_s_at	-6.86	-3.12	immediate early response 3	IER3
229670_at	-4.64	-3.12		
226959_at	-3.72	-3.11		
1568638_a_at	-6.81	-3.11	indoleamine-pyrrole 2,3 dioxygenase-like 1	INDOL1
241819_at	-4.59	-3.11	tumor necrosis factor (ligand) superfamily, member 8	TNFSF8
202192_s_at	-7.94	-3.11	growth arrest-specific 7	GAS7

229897_at	-2.63	-3.11	zinc finger protein 641	ZNF641
208890_s_at	-4.01	-3.11	plexin B2	PLXNB2
210758_at	-4.34	-3.11	PC4 and SFRS1 interacting protein 1	PSIP1
209574_s_at	-3.44	-3.10	chromosome 18 open reading frame 1	C18orf1
204714_s_at	-2.52	-3.10	coagulation factor V (proaccelerin, labile factor)	F5
205248_at	-3.13	-3.10	dopey family member 2	DOPEY2
228226_s_at	-2.31	-3.10	zinc finger protein 775	ZNF775
201454_s_at	-4.10	-3.10	aminopeptidase puromycin sensitive	NPEPPS
222062_at	-7.06	-3.10	interleukin 27 receptor, alpha	IL27RA
212930_at	-4.34	-3.09	ATPase, Ca++ transporting, plasma membrane 1	ATP2B1
200758_s_at	-7.91	-3.09	nuclear factor (erythroid-derived 2)-like 1	NFE2L1
40359_at	-2.77	-3.08	Ras association (RalGDS/AF-6) domain family 7	RASSF7
238649_at	-2.67	-3.08	phosphatidylinositol transfer protein, cytoplasmic 1	PITPNC1
235857_at	-3.22	-3.08	potassium channel tetramerisation domain containing 11	KCTD11
228570_at	-2.57	-3.08	BTB (POZ) domain containing 11	BTBD11
204103_at	-5.20	-3.08	chemokine (C-C motif) ligand 4	CCL4
221779_at	-5.31	-3.07	MICAL-like 1	MICALL1
201651_s_at	-3.58	-3.07	protein kinase C and casein kinase substrate in neurons 2	PACSIN2
227232_at	-2.73	-3.07	Enah/Vasp-like	EVL
230820_at	-2.63	-3.07	SMAD specific E3 ubiquitin protein ligase 2	SMURF2
204472_at	-6.96	-3.07	GTP binding protein overexpressed in skeletal muscle	GEM
239752_at	-7.24	-3.07		
202638_s_at	-5.45	-3.07	intercellular adhesion molecule 1 (CD54), human rhinovirus receptor	ICAM1
232615_at	-2.51	-3.07		
235334_at	-3.05	-3.06	ST6 (alpha-N-acetyl-neuraminyl-2,3-beta-galactosyl-1,3)-N-acetylgalactosaminide alpha-2,6-sialyltran	ST6GALNAC3
222235_s_at	-3.90	-3.06		
216979_at	-5.61	-3.06	nuclear receptor subfamily 4, group A, member 3	NR4A3
212086_x_at	-2.09	-3.06	lamin A/C	LMNA
225913_at	-3.23	-3.05		
239364_at	-2.50	-3.05	ets variant gene 6 (TEL oncogene)	ETV6
209307_at	-3.61	-3.05		
204420_at	-18.63	-3.05	FOS-like antigen 1	FOSL1
203887_s_at	-2.56	-3.05	thrombomodulin	THBD
201110_s_at	-6.32	-3.05	thrombospondin 1	THBS1
218017_s_at	-5.65	-3.04	heparan-alpha-glucosaminide N-acetyltransferase	HGSNAT
238077_at	-5.01	-3.04	potassium channel tetramerisation domain containing 6	KCTD6

207075_at	-5.20	-3.04	NLR family, pyrin domain containing 3	NLRP3
235421_at	-8.39	-3.03		
203940_s_at	-3.20	-3.03	vasohibin 1	VASH1
238469_at	-3.42	-3.03		
229733_s_at	-7.23	-3.03		
219202_at	-3.65	-3.03	rhomboid 5 homolog 2 (Drosophila)	RHBDF2
201646_at	-4.50	-3.02	scavenger receptor class B, member 2	SCARB2
225629_s_at	-3.79	-3.02	zinc finger and BTB domain containing 4	ZBTB4
236961_at	-2.47	-3.02		
226265_at	-4.56	-3.02	glutamine and serine rich 1	QSER1
208343_s_at	-23.74	-3.02	nuclear receptor subfamily 5, group A, member 2	NR5A2
212818_s_at	-2.81	-3.02	ankyrin repeat and SOCS box-containing 1	ASB1
215933_s_at	-8.86	-3.02	homeobox, hematopoietically expressed	HHEX
201865_x_at	-3.06	-3.02		
1552639_at	-3.39	-3.02	kelch domain containing 7B	KLHDC7B
236295_s_at	-7.54	-3.01	NLR family, CARD domain containing 3	NLRC3
212791_at	-3.74	-3.01		
243431_at	-2.05	-3.01	BTB (POZ) domain containing 14A	BTBD14A
235753_at	-3.24	-3.01	homeobox A7	HOXA7
224827_at	-4.04	-3.01	ubiquitin domain containing 2	UBTD2
209012_at	-4.08	-3.01	triple functional domain (PTPRF interacting)	TRIO
201626_at	-8.44	-3.01	insulin induced gene 1	INSIG1
216867_s_at	-3.43	-3.01	platelet-derived growth factor alpha polypeptide	PDGFA
230561_s_at	-3.75	-3.01		
218627_at	-2.86	-3.00		
225965_at	-3.00	-3.00	DDHD domain containing 1	DDHD1
203231_s_at	-6.01	-3.00	ataxin 1	ATXN1
229900_at	-2.94	-3.00	CD109 molecule	CD109
212292_at	-10.03	-3.00	solute carrier family 7 (cationic amino acid transporter, y+ system), member 1	SLC7A1
1554544_a_at	-3.97	-2.99	myelin basic protein	MBP
212830_at	-3.37	-2.99	multiple EGF-like-domains 9	MEGF9
1552696_at	-4.28	-2.99	non imprinted in Prader-Willi/Angelman syndrome 1	NIPA1
212735_at	-5.59	-2.98	KIAA0226	KIAA0226
213150_at	-6.78	-2.98	homeobox A10	HOXA10
236892_s_at	-2.77	-2.98		
1558748_at	-2.27	-2.98		
238642_at	-4.20	-2.98	ankyrin repeat domain 13 family, member D	ANKRD13D
227346_at	-3.21	-2.97	IKAROS family zinc finger 1 (Ikaros)	IKZF1
223264_at	-3.76	-2.97	mesoderm development candidate 1	MESDC1

235109_at	-2.95	-2.96		
220377_at	-5.40	-2.96	family with sequence similarity 30, member A	FAM30A
212677_s_at	-3.56	-2.96	centrosomal protein 68kDa	CEP68
231484_at	-3.06	-2.96		
226430_at	-3.59	-2.96		
207574_s_at	-10.09	-2.96	growth arrest and DNA-damage-inducible, beta	GADD45B
212448_at	-2.19	-2.95	neural precursor cell expressed, developmentally down-regulated 4-like	NEDD4L
202052_s_at	-24.29	-2.94	retinoic acid induced 14	RAI14
225946_at	-3.25	-2.94	Ras association (RalGDS/AF-6) domain family 8	RASSF8
228008_at	-3.17	-2.94		
60084_at	-3.72	-2.94	cylindromatosis (turban tumor syndrome)	CYLD
202723_s_at	-5.48	-2.94	forkhead box O1A (rhabdomyosarcoma)	FOXO1A
226158_at	-3.41	-2.94	kelch-like 24 (Drosophila)	KLHL24
217897_at	-2.52	-2.94	FXYD domain containing ion transport regulator 6	FXYD6
223358_s_at	-2.81	-2.93		
219150_s_at	-2.42	-2.93	centaurin, alpha 1	CENTA1
216321_s_at	-3.15	-2.93	nuclear receptor subfamily 3, group C, member 1 (glucocorticoid receptor)	NR3C1
212458_at	-5.40	-2.93	sprouty-related, EVH1 domain containing 2	SPRED2
235513_at	-3.37	-2.93		
242586_at	-2.97	-2.92	fibronectin type III and SPRY domain containing 1-like	FSD1L
222605_at	-6.09	-2.92	REST corepressor 3	RCOR3
203233_at	-3.47	-2.92	interleukin 4 receptor	IL4R
1552343_s_at	-2.55	-2.92	phosphodiesterase 7A	PDE7A
218157_x_at	-4.37	-2.92	CDC42 small effector 1	CDC42SE1
233880_at	-2.38	-2.92	ring finger protein 213	RNF213
200704_at	-5.16	-2.91	lipopolysaccharide-induced TNF factor	LITAF
226545_at	-2.95	-2.91	CD109 molecule	CD109
214179_s_at	-3.57	-2.90	nuclear factor (erythroid-derived 2)-like 1	NFE2L1
242277_at	-3.05	-2.90		
242523_at	-3.29	-2.90		
226003_at	-3.72	-2.90	kinesin family member 21A	KIF21A
228904_at	-4.30	-2.90	homeobox B3	HOXB3
213391_at	-3.89	-2.90	dpy-19-like 4 (C. elegans)	DPY19L4
218880_at	-5.46	-2.90	FOS-like antigen 2	FOSL2
242612_at	-3.29	-2.90		
226961_at	-5.10	-2.90	proline rich 15	PRR15
202971_s_at	-4.06	-2.90	dual-specificity tyrosine-(Y)-phosphorylation regulated kinase 2	DYRK2

203704_s_at	-2.62	-2.90	ras responsive element binding protein 1	RREB1
213375_s_at	-3.15	-2.90		
235252_at	-5.24	-2.89	kinase suppressor of ras 1	KSR1
218149_s_at	-4.38	-2.89	zinc finger protein 395	ZNF395
238722_x_at	-6.92	-2.89		
229120_s_at	-5.88	-2.89	CDC42 small effector 1	CDC42SE1
228594_at	-4.70	-2.89	chromosome 5 open reading frame 33	C5orf33
226721_at	-3.03	-2.89	dpy-19-like 4 (C. elegans)	DPY19L4
204215_at	-6.28	-2.88	chromosome 7 open reading frame 23	C7orf23
223189_x_at	-4.26	-2.88	myeloid/lymphoid or mixed-lineage leukemia 5 (trithorax homolog, Drosophila)	MLL5
200836_s_at	-3.30	-2.88	microtubule-associated protein 4	MAP4
244286_at	-3.06	-2.88	formin binding protein 1	FNBP1
234987_at	-5.17	-2.88		
1556429_a_at	-2.37	-2.88	WD repeat domain 67	WDR67
206060_s_at	-2.85	-2.88	protein tyrosine phosphatase, non-receptor type 22 (lymphoid)	PTPN22
215248_at	-3.38	-2.88		
238029_s_at	-7.05	-2.87	solute carrier family 16, member 14 (monocarboxylic acid transporter 14)	SLC16A14
201862_s_at	-3.17	-2.87	leucine rich repeat (in FLII) interacting protein 1	LRRFIP1
227811_at	-2.86	-2.87	FYVE, RhoGEF and PH domain containing 3	FGD3
225789_at	-3.18	-2.87	centaurin, gamma 3	CENTG3
219041_s_at	-8.45	-2.87	replication initiator 1	REPIN1
228980_at	-3.71	-2.87	ring finger and FYVE-like domain containing 1	RFFL
229336_at	-6.69	-2.87		
212655_at	-3.32	-2.87	zinc finger, CCHC domain containing 14	ZCCHC14
235221_at	-3.27	-2.87	cerebellin 3 precursor	CBLN3
230604_at	-2.65	-2.86		
212895_s_at	-3.34	-2.86	active BCR-related gene	ABR
240405_at	-2.53	-2.86		
225048_at	-2.70	-2.86	PHD finger protein 10	PHF10
227897_at	-3.03	-2.86	RAP2B, member of RAS oncogene family	RAP2B
210001_s_at	-5.65	-2.86	suppressor of cytokine signaling 1	SOCS1
205290_s_at	-2.45	-2.86	bone morphogenetic protein 2	BMP2
230003_at	-3.79	-2.86		
225606_at	-3.70	-2.85	BCL2-like 11 (apoptosis facilitator)	BCL2L11
223586_at	-3.01	-2.85	aryl hydrocarbon receptor nuclear translocator-like 2	ARNTL2
202284_s_at	-2.64	-2.85	cyclin-dependent kinase inhibitor 1A (p21, Cip1)	CDKN1A
225768_at	-3.08	-2.85	nuclear receptor subfamily 1, group D, member 2	NR1D2

1553768_a_at	-2.33	-2.85	discoidin, CUB and LCCL domain containing 1	DCBLD1
228595_at	-2.16	-2.84		
228173_at	-4.69	-2.84	GNAS complex locus	GNAS
217200_x_at	-3.68	-2.84	cytochrome b-561	CYB561
244023_at	-3.22	-2.84	spleen tyrosine kinase	SYK
232424_at	-6.13	-2.83	PR domain containing 16	PRDM16
213836_s_at	-3.37	-2.83	WD repeat domain, phosphoinositide interacting 1	WIPI1
229968_at	-2.51	-2.83		
212356_at	-4.57	-2.83	KIAA0323	KIAA0323
212239_at	-2.27	-2.83	phosphoinositide-3-kinase, regulatory subunit 1 (p85 alpha)	PIK3R1
202460_s_at	-3.43	-2.83	lipin 2	LPIN2
212276_at	-3.24	-2.83	lipin 1	LPIN1
215285_s_at	-2.25	-2.83	putative homeodomain transcription factor 1	PHTF1
231769_at	-4.91	-2.82	F-box protein 6	FBXO6
1557049_at	-2.42	-2.82		
225234_at	-2.59	-2.82	Cas-Br-M (murine) ecotropic retroviral transforming sequence	CBL
230815_at	-4.03	-2.82		
201627_s_at	-10.37	-2.82	insulin induced gene 1	INSIG1
220974_x_at	-2.03	-2.82	sideroflexin 3	SFXN3
209060_x_at	-4.27	-2.82	nuclear receptor coactivator 3	NCOA3
225150_s_at	-5.30	-2.82	rhotekin	RTKN
226609_at	-3.36	-2.82	discoidin, CUB and LCCL domain containing 1	DCBLD1
241627_x_at	-4.92	-2.82		
239763_at	-3.32	-2.82		
240363_at	-2.88	-2.82	ankyrin 1, erythrocytic	ANK1
217730_at	-2.90	-2.81	transmembrane BAX inhibitor motif containing 1	TMBIM1
204512_at	-4.61	-2.81	human immunodeficiency virus type I enhancer binding protein 1	HIVEP1
235012_at	-5.24	-2.81		
1570259_at	-2.65	-2.81	LIM and senescent cell antigen-like domains 1	LIMS1
223132_s_at	-7.03	-2.80	tripartite motif-containing 8	TRIM8
217849_s_at	-16.98	-2.80	CDC42 binding protein kinase beta (DMPK-like)	CDC42BPB
224762_at	-4.21	-2.80	serine incorporator 2	SERINC2
213386_at	-2.26	-2.80		
203317_at	-6.47	-2.80	pleckstrin and Sec7 domain containing 4	PSD4
229872_s_at	-2.65	-2.80		
244008_at	-2.40	-2.80		
231175_at	-2.21	-2.80	chromosome 6 open reading frame 65	C6orf65
221830_at	-3.36	-2.80	RAP2A, member of RAS oncogene family	RAP2A
227228_s_at	-5.12	-2.80	KIAA1509	KIAA1509

225569_at	-7.86	-2.80	eukaryotic translation initiation factor 2C, 2	EIF2C2
231403_at	-3.06	-2.80	triple functional domain (PTPRF interacting)	TRIO
213518_at	-2.41	-2.79	protein kinase C, iota	PRKCI
1569003_at	-3.53	-2.79	transmembrane protein 49	TMEM49
241803_s_at	-2.92	-2.79		
226551_at	-2.37	-2.79	receptor (TNFRSF)-interacting serine-threonine kinase 1	RIPK1
225685_at	-6.40	-2.79		
231579_s_at	-2.42	-2.79	TIMP metallopeptidase inhibitor 2	TIMP2
225763_at	-7.18	-2.79	RCSD domain containing 1	RCSD1
231858_x_at	-5.32	-2.79		
240393_at	-4.28	-2.79	amino-terminal enhancer of split	AES
203098_at	-4.98	-2.78	chromodomain protein, Y-like	CDYL
218237_s_at	-5.89	-2.78	solute carrier family 38, member 1	SLC38A1
228833_s_at	-4.63	-2.78		
45297_at	-2.31	-2.78	EH-domain containing 2	EHD2
201426_s_at	-4.04	-2.78	vimentin	VIM
225368_at	-5.01	-2.78	homeodomain interacting protein kinase 2	HIPK2
215111_s_at	-2.40	-2.77	TSC22 domain family, member 1	TSC22D1
1562403_a_at	-16.24	-2.77	solute carrier family 8 (sodium-calcium exchanger), member 3	SLC8A3
226135_at	-3.65	-2.77	chromosome 6 open reading frame 107	C6orf107
227502_at	-3.46	-2.77	KIAA1147	KIAA1147
201811_x_at	-3.10	-2.77	SH3-domain binding protein 5 (BTK-associated)	SH3BP5
202259_s_at	-2.78	-2.77		
210427_x_at	-3.61	-2.77	annexin A2	ANXA2
216250_s_at	-4.23	-2.76	leupaxin	LPXN
204047_s_at	-2.66	-2.76	phosphatase and actin regulator 2	PHACTR2
231907_at	-3.14	-2.76		
233539_at	-2.32	-2.76		
222392_x_at	-2.44	-2.76	PERP, TP53 apoptosis effector	PERP
226016_at	-4.04	-2.76	CD47 molecule	CD47
229011_at	-26.66	-2.76		
238909_at	-7.46	-2.76	S100 calcium binding protein A10	S100A10
218845_at	-3.34	-2.75	dual specificity phosphatase 22	DUSP22
211862_x_at	-2.96	-2.75	CASP8 and FADD-like apoptosis regulator	CFLAR
207971_s_at	-4.88	-2.75	centrosomal protein 68kDa	CEP68
222891_s_at	-2.65	-2.75	B-cell CLL/lymphoma 11A (zinc finger protein)	BCL11A
218066_at	-3.05	-2.75	solute carrier family 12 (potassium/chloride transporters), member 7	SLC12A7
207992_s_at	-3.00	-2.75	adenosine monophosphate deaminase (isoform E)	AMPD3
226392_at	-2.94	-2.75		
229312_s_at	-4.22	-2.75	G kinase anchoring protein 1	GKAP1

217437_s_at	-2.47	-2.74	transforming, acidic coiled-coil containing protein 1	TACC1
201590_x_at	-4.03	-2.74	annexin A2	ANXA2
203627_at	-5.48	-2.74	insulin-like growth factor 1 receptor	IGF1R
1559964_at	-2.86	-2.74		
202928_s_at	-2.12	-2.74	PHD finger protein 1	PHF1
202922_at	-2.36	-2.74	glutamate-cysteine ligase, catalytic subunit	GCLC
226249_at	-20.96	-2.73	sorting nexin family member 30	SNX30
210665_at	-4.09	-2.73	tissue factor pathway inhibitor (lipoprotein-associated coagulation inhibitor)	TFPI
218792_s_at	-4.77	-2.73	B-box and SPRY domain containing	BSPRY
202651_at	-4.05	-2.72	lysophosphatidylglycerol acyltransferase 1	LPGAT1
227984_at	-16.27	-2.72		
236471_at	-3.24	-2.72	nuclear factor (erythroid-derived 2)-like 3	NFE2L3
1555058_a_at	-3.18	-2.72	lysophosphatidylglycerol acyltransferase 1	LPGAT1
222537_s_at	-5.14	-2.72	CDC42 small effector 1	CDC42SE1
1555888_at	-2.30	-2.72	E3 ubiquitin protein ligase, HECT domain containing, 1	EDD1
219229_at	-5.38	-2.71	solute carrier organic anion transporter family, member 3A1	SLCO3A1
202101_s_at	-2.29	-2.71	v-ral simian leukemia viral oncogene homolog B (ras related; GTP binding protein)	RALB
203578_s_at	-2.06	-2.71	transient receptor potential cation channel, subfamily V, member 6	TRPV6
219498_s_at	-2.39	-2.71	B-cell CLL/lymphoma 11A (zinc finger protein)	BCL11A
223227_at	-2.58	-2.71	Bardet-Biedl syndrome 2	BBS2
216268_s_at	-2.62	-2.71	jagged 1 (Alagille syndrome)	JAG1
226977_at	-2.76	-2.71		
228220_at	-5.10	-2.71	FCH domain only 2	FCHO2
222491_at	-3.68	-2.71	heparan-alpha-glucosaminide N-acetyltransferase	HGSNAT
227388_at	-9.18	-2.71	tumor suppressor candidate 1	TUSC1
226125_at	-2.62	-2.71		
228557_at	-5.79	-2.70	l(3)mbt-like 4 (Drosophila)	L3MBTL4
221695_s_at	-2.45	-2.70	mitogen-activated protein kinase kinase kinase 2	MAP3K2
218263_s_at	-4.80	-2.70	zinc finger, BED-type containing 5	ZBED5
228647_at	-6.32	-2.70		
238940_at	-2.63	-2.70	Kruppel-like factor 12	KLF12
224579_at	-6.43	-2.70	solute carrier family 38, member 1	SLC38A1
229295_at	-2.82	-2.70		
225752_at	-3.50	-2.70	non imprinted in Prader-Willi/Angelman syndrome 1	NIPA1
205479_s_at	-3.98	-2.70	plasminogen activator, urokinase	PLAU
226738_at	-2.70	-2.70	WD repeat domain 81	WDR81
230860_at	-2.31	-2.70		

244773_at	-2.29	-2.69		
226040_at	-2.71	-2.69		
204440_at	-5.00	-2.69	CD83 molecule	CD83
202807_s_at	-5.00	-2.69	target of myb1 (chicken)	TOM1
241889_at	-7.50	-2.69		
221788_at	-15.69	-2.69		
203822_s_at	-2.77	-2.69	E74-like factor 2 (ets domain transcription factor)	ELF2
227534_at	-6.50	-2.69	chromosome 9 open reading frame 21	C9orf21
224891_at	-3.11	-2.69	forkhead box O3A	FOXO3A
229429_x_at	-2.89	-2.68		
242946_at	-2.69	-2.68	CD53 molecule	CD53
206636_at	-8.41	-2.68	RAS p21 protein activator 2	RASA2
242999_at	-2.08	-2.68	Rho guanine nucleotide exchange factor (GEF) 7	ARHGEF7
210839_s_at	-3.38	-2.68	ectonucleotide pyrophosphatase/phosphodiesterase 2 (autotaxin)	ENPP2
225235_at	-3.69	-2.68	tetraspanin 17	TSPAN17
224558_s_at	-2.42	-2.68	metastasis associated lung adenocarcinoma transcript 1 (non-coding RNA)	MALAT1
225133_at	-6.29	-2.68		
206675_s_at	-5.31	-2.68	SKI-like oncogene	SKIL
203094_at	-3.83	-2.68	MAD2L1 binding protein	MAD2L1BP
225486_at	-3.35	-2.67	AT rich interactive domain 2 (ARID, RFX-like)	ARID2
213293_s_at	-3.19	-2.67	tripartite motif-containing 22	TRIM22
228991_at	-2.94	-2.67		
203373_at	-2.88	-2.67	suppressor of cytokine signaling 2	SOCS2
222627_at	-3.01	-2.67	vacuolar protein sorting 54 homolog (S. cerevisiae)	VPS54
203553_s_at	-3.87	-2.67	mitogen-activated protein kinase kinase kinase kinase 5	MAP4K5
209164_s_at	-5.35	-2.67	cytochrome b-561	CYB561
204020_at	-4.12	-2.67	purine-rich element binding protein A	PURA
212119_at	-3.58	-2.67	ras homolog gene family, member Q	RHOQ
242794_at	-3.37	-2.67	mastermind-like 3 (Drosophila)	MAML3
218927_s_at	-3.15	-2.67	carbohydrate (chondroitin 4) sulfotransferase 12	CHST12
221122_at	-4.37	-2.66	HRAS-like suppressor 2	HRASLS2
212380_at	-2.12	-2.66	KIAA0082	KIAA0082
204774_at	-3.64	-2.66	ecotropic viral integration site 2A	EVI2A
212274_at	-2.60	-2.66	lipin 1	LPIN1
225116_at	-3.25	-2.66	homeodomain interacting protein kinase 2	HIPK2
225970_at	-2.55	-2.66	DDHD domain containing 1	DDHD1
223125_s_at	-3.11	-2.66	chromosome 1 open reading frame 21	C1orf21
212564_at	-2.18	-2.66	potassium channel tetramerisation domain containing 2	KCTD2

1557527_at	-5.16	-2.66		
204342_at	-6.10	-2.66	solute carrier family 25 (mitochondrial carrier; phosphate carrier), member 24	SLC25A24
228394_at	-16.02	-2.66	serine/threonine kinase 10	STK10
207978_s_at	-2.57	-2.66	nuclear receptor subfamily 4, group A, member 3	NR4A3
244271_at	-4.36	-2.66		
207160_at	-4.40	-2.66	interleukin 12A (natural killer cell stimulatory factor 1, cytotoxic lymphocyte maturation factor 1,	IL12A
236630_at	-6.69	-2.66	aquaporin 2 (collecting duct)	AQP2
225490_at	-2.14	-2.66	AT rich interactive domain 2 (ARID, RFX-like)	ARID2
1554690_a_at	-4.72	-2.65	transforming, acidic coiled-coil containing protein 1	TACC1
231875_at	-6.21	-2.65	kinesin family member 21A	KIF21A
225385_s_at	-5.75	-2.65	heterogeneous nuclear ribonucleoprotein L-like	HNRPLL
207996_s_at	-4.76	-2.65	chromosome 18 open reading frame 1	C18orf1
225545_at	-6.25	-2.65	eukaryotic elongation factor-2 kinase	EEF2K
204562_at	-4.22	-2.65	interferon regulatory factor 4	IRF4
212468_at	-2.55	-2.64	sperm associated antigen 9	SPAG9
201389_at	-5.82	-2.64	integrin, alpha 5 (fibronectin receptor, alpha polypeptide)	ITGA5
236600_at	-2.24	-2.64	spastic paraplegia 20, spartin (Troyer syndrome)	SPG20
244447_at	-4.57	-2.64		
224572_s_at	-4.23	-2.64	interferon regulatory factor 2 binding protein 2	IRF2BP2
226810_at	-2.67	-2.64		
221865_at	-3.81	-2.64	chromosome 9 open reading frame 91	C9orf91
1554600_s_at	-3.62	-2.63	lamin A/C	LMNA
227514_at	-6.52	-2.63		
223967_at	-3.41	-2.63	angiopoietin-like 6	ANGPTL6
202082_s_at	-2.68	-2.63	SEC14-like 1 (S. cerevisiae)	SEC14L1
204667_at	-2.66	-2.63	forkhead box A1	FOXA1
205345_at	-3.28	-2.63	BRCA1 associated RING domain 1	BARD1
203445_s_at	-2.96	-2.63	CTD (carboxy-terminal domain, RNA polymerase II, polypeptide A) small phosphatase 2	CTDSP2
225386_s_at	-5.10	-2.63	heterogeneous nuclear ribonucleoprotein L-like	HNRPLL
203955_at	-5.01	-2.62	KIAA0649	KIAA0649
227341_at	-3.79	-2.62	chromosome 10 open reading frame 30	C10orf30
204063_s_at	-7.95	-2.62	unc-51-like kinase 2 (C. elegans)	ULK2
1566766_a_at	-6.00	-2.62		
236192_at	-2.74	-2.62		
243613_at	-3.96	-2.62		
219257_s_at	-8.64	-2.62	sphingosine kinase 1	SPHK1

202606_s_at	-3.81	-2.62	tousled-like kinase 1	TLK1
226676_at	-3.08	-2.62	zinc finger protein 521	ZNF521
1555789_s_at	-2.53	-2.62	PHD finger protein 23	PHF23
213309_at	-2.23	-2.62	phospholipase C-like 2	PLCL2
206674_at	-4.16	-2.61	fms-related tyrosine kinase 3	FLT3
229101_at	-3.86	-2.61		
215047_at	-2.57	-2.61	tripartite motif-containing 58	TRIM58
208908_s_at	-3.37	-2.61	calpastatin	CAST
210105_s_at	-3.00	-2.61	FYN oncogene related to SRC, FGR, YES	FYN
239629_at	-2.52	-2.61	CASP8 and FADD-like apoptosis regulator	CFLAR
218486_at	-4.21	-2.61	Kruppel-like factor 11	KLF11
228899_at	-5.58	-2.61		
201960_s_at	-2.39	-2.61	MYC binding protein 2	MYCBP2
225330_at	-6.14	-2.61	insulin-like growth factor 1 receptor	IGF1R
227749_at	-4.58	-2.61		
204131_s_at	-2.84	-2.61	forkhead box O3A	FOXO3A
227626_at	-3.53	-2.60	progestin and adipoQ receptor family member VIII	PAQR8
216942_s_at	-4.44	-2.60	CD58 molecule	CD58
209288_s_at	-4.60	-2.60	CDC42 effector protein (Rho GTPase binding) 3	CDC42EP3
201367_s_at	-7.39	-2.59	zinc finger protein 36, C3H type-like 2	ZFP36L2
215427_s_at	-3.82	-2.59	zinc finger, CCHC domain containing 14	ZCCHC14
222463_s_at	-7.25	-2.59	beta-site APP-cleaving enzyme 1	BACE1
217901_at	-2.30	-2.59	desmoglein 2	DSG2
225997_at	-6.06	-2.59	MOB1, Mps One Binder kinase activator-like 1A (yeast)	MOBKL1A
217995_at	-2.90	-2.59	sulfide quinone reductase-like (yeast)	SQRDL
41113_at	-3.89	-2.59	zinc finger protein 500	ZNF500
202688_at	-2.65	-2.59	tumor necrosis factor (ligand) superfamily, member 10	TNFSF10
241879_at	-9.78	-2.59		
210999_s_at	-4.18	-2.59	growth factor receptor-bound protein 10	GRB10
203659_s_at	-4.33	-2.59	tripartite motif-containing 13	TRIM13
224880_at	-3.51	-2.59	v-ral simian leukemia viral oncogene homolog A (ras related)	RALA
202956_at	-2.59	-2.59	ADP-ribosylation factor guanine nucleotide-exchange factor 1(brefeldin A-inhibited)	ARFGEF1
1553743_at	-4.22	-2.59	family with sequence similarity 119, member A	FAM119A
213327_s_at	-2.88	-2.59	ubiquitin specific peptidase 12	USP12
209678_s_at	-3.14	-2.59	protein kinase C, iota	PRKCI
202221_s_at	-2.68	-2.58	E1A binding protein p300	EP300
212764_at	-3.30	-2.58		
224912_at	-2.57	-2.58	tetratricopeptide repeat domain 7A	TTC7A
224481_s_at	-3.40	-2.58	HECT domain containing 1	HECTD1
200998_s_at	-25.42	-2.58	cytoskeleton-associated protein 4	CKAP4

225339_at	-4.79	-2.58		
218902_at	-4.54	-2.58	Notch homolog 1, translocation-associated (Drosophila)	NOTCH1
212355_at	-2.96	-2.58	KIAA0323	KIAA0323
203514_at	-5.47	-2.58	mitogen-activated protein kinase kinase kinase 3	MAP3K3
224374_s_at	-2.89	-2.58	elastin microfibril interfacer 2	EMILIN2
212761_at	-2.09	-2.58	transcription factor 7-like 2 (T-cell specific, HMG-box)	TCF7L2
211061_s_at	-3.36	-2.58	mannosyl (alpha-1,6-)-glycoprotein beta-1,2-N-acetylglucosaminyltransferase	MGAT2
242268_at	-2.63	-2.57	CUG triplet repeat, RNA binding protein 2	CUGBP2
226447_at	-2.44	-2.57	ash1 (absent, small, or homeotic)-like (Drosophila)	ASH1L
228954_at	-2.30	-2.57	LysM, putative peptidoglycan-binding, domain containing 4	LYSMD4
212120_at	-4.06	-2.57	ras homolog gene family, member Q	RHOQ
235256_s_at	-2.27	-2.57	galactose mutarotase (aldose 1-epimerase)	GALM
213954_at	-2.96	-2.57		
218898_at	-6.94	-2.57	family with sequence similarity 57, member A	FAM57A
1558143_a_at	-3.66	-2.57	BCL2-like 11 (apoptosis facilitator)	BCL2L11
207949_s_at	-3.27	-2.56	islet cell autoantigen 1, 69kDa	ICA1
213503_x_at	-3.67	-2.56	annexin A2	ANXA2
211671_s_at	-2.75	-2.56	nuclear receptor subfamily 3, group C, member 1 (glucocorticoid receptor)	NR3C1
229264_at	-2.54	-2.56		
225755_at	-2.66	-2.56	kelch domain containing 8B	KLHDC8B
200628_s_at	-3.05	-2.56	tryptophanyl-tRNA synthetase	WARS
242488_at	-2.05	-2.56		
200632_s_at	-2.33	-2.56	N-myc downstream regulated gene 1	NDRG1
226164_x_at	-5.32	-2.56	family with sequence similarity 80, member B	FAM80B
206847_s_at	-5.10	-2.55	homeobox A7	HOXA7
209286_at	-3.19	-2.55	CDC42 effector protein (Rho GTPase binding) 3	CDC42EP3
203018_s_at	-2.18	-2.55	synovial sarcoma, X breakpoint 2 interacting protein	SSX2IP
208816_x_at	-3.32	-2.55	annexin A2 pseudogene 2	ANXA2P2
224800_at	-2.01	-2.55	WD repeat and FYVE domain containing 1	WDFY1
227954_at	-3.05	-2.55		
238013_at	-3.83	-2.55	pleckstrin homology domain containing, family A (phosphoinositide binding specific) member 2	PLEKHA2
226560_at	-3.72	-2.54		
218648_at	-3.51	-2.54	CREB regulated transcription coactivator 3	CRTC3

212474_at	-2.12	-2.53	KIAA0241	KIAA0241
202847_at	-3.47	-2.53	phosphoenolpyruvate carboxykinase 2 (mitochondrial)	PCK2
203298_s_at	-2.31	-2.53	jumonji, AT rich interactive domain 2	JARID2
213142_x_at	-2.67	-2.53		
213639_s_at	-3.08	-2.52	zinc finger protein 500	ZNF500
210512_s_at	-4.27	-2.52	vascular endothelial growth factor A	VEGFA
203185_at	-6.00	-2.52	Ras association (RalGDS/AF-6) domain family 2	RASSF2
226793_at	-4.93	-2.52		
204994_at	-3.09	-2.52	myxovirus (influenza virus) resistance 2 (mouse)	MX2
202724_s_at	-3.10	-2.51	forkhead box O1A (rhabdomyosarcoma)	FOXO1A
235427_at	-4.83	-2.51		
209949_at	-5.15	-2.51	neutrophil cytosolic factor 2 (65kDa, chronic granulomatous disease, autosomal 2)	NCF2
221235_s_at	-4.29	-2.51		
214596_at	-2.16	-2.51		
222982_x_at	-3.58	-2.51	solute carrier family 38, member 2	SLC38A2
228843_at	-5.87	-2.51		
207629_s_at	-5.82	-2.51	rho/rac guanine nucleotide exchange factor (GEF) 2	ARHGEF2
210191_s_at	-3.53	-2.51	putative homeodomain transcription factor 1	PHTF1
223085_at	-2.61	-2.51	ring finger protein 19	RNF19
200813_s_at	-2.98	-2.50	platelet-activating factor acetylhydrolase, isoform Ib, alpha subunit 45kDa	PAFAH1B1
201548_s_at	-2.54	-2.50	jumonji, AT rich interactive domain 1B	JARID1B
209750_at	-3.58	-2.50	nuclear receptor subfamily 1, group D, member 2	NR1D2
209724_s_at	-2.21	-2.50	zinc finger protein 161 homolog (mouse)	ZFP161
227999_at	-5.40	-2.50	PWWP domain containing 2	PWWP2
239979_at	-3.33	-2.49	epithelial stromal interaction 1 (breast)	EPSTI1
227013_at	-3.10	-2.49	LATS, large tumor suppressor, homolog 2 (Drosophila)	LATS2
235142_at	-5.95	-2.49	zinc finger and BTB domain containing 8	ZBTB8
226474_at	-3.04	-2.49	NLR family, CARD domain containing 5	NLRC5
220924_s_at	-4.69	-2.49	solute carrier family 38, member 2	SLC38A2
219190_s_at	-3.36	-2.49	eukaryotic translation initiation factor 2C, 4	EIF2C4
224336_s_at	-2.81	-2.49	dual specificity phosphatase 16	DUSP16
202969_at	-4.30	-2.49		
209392_at	-3.36	-2.49	ectonucleotide pyrophosphatase/phosphodiesterase 2 (autotaxin)	ENPP2

203217_s_at	-2.07	-2.49	ST3 beta-galactoside alpha-2,3-sialyltransferase 5	ST3GAL5
223158_s_at	-6.18	-2.49	NIMA (never in mitosis gene a)-related kinase 6	NEK6
226691_at	-8.61	-2.49		
210214_s_at	-4.20	-2.48	bone morphogenetic protein receptor, type II (serine/threonine kinase)	BMPR2
209281_s_at	-2.63	-2.48	ATPase, Ca++ transporting, plasma membrane 1	ATP2B1
204194_at	-2.62	-2.48	BTB and CNC homology 1, basic leucine zipper transcription factor 1	BACH1
222746_s_at	-3.16	-2.48	B-box and SPRY domain containing	BSPRY
238575_at	-4.56	-2.48	oxysterol binding protein-like 6	OSBPL6
214486_x_at	-2.97	-2.48	CASP8 and FADD-like apoptosis regulator	CFLAR
204021_s_at	-3.33	-2.48	purine-rich element binding protein A	PURA
226538_at	-2.69	-2.47	mannosidase, alpha, class 2A, member 1	MAN2A1
236369_at	-2.33	-2.47		
220095_at	-4.31	-2.47	chromosome 9 open reading frame 39	C9orf39
201218_at	-4.47	-2.47	C-terminal binding protein 2	CTBP2
225527_at	-3.72	-2.47	CCAAT/enhancer binding protein (C/EBP), gamma	CEBPG
236114_at	-2.23	-2.47		
201860_s_at	-11.45	-2.47	plasminogen activator, tissue	PLAT
201354_s_at	-3.45	-2.47	bromodomain adjacent to zinc finger domain, 2A	BAZ2A
202449_s_at	-3.68	-2.47	retinoid X receptor, alpha	RXRA
218815_s_at	-2.87	-2.47	transmembrane protein 51	TMEM51
205705_at	-3.09	-2.47	ankyrin repeat domain 26	ANKRD26
205038_at	-2.40	-2.47	IKAROS family zinc finger 1 (Ikaros)	IKZF1
206099_at	-4.89	-2.46	protein kinase C, eta	PRKCH
230206_at	-2.01	-2.46	dedicator of cytokinesis 5	DOCK5
208309_s_at	-4.37	-2.46	mucosa associated lymphoid tissue lymphoma translocation gene 1	MALT1
212063_at	-3.59	-2.46	CD44 molecule (Indian blood group)	CD44
211962_s_at	-5.30	-2.46	zinc finger protein 36, C3H type-like 1	ZFP36L1
210664_s_at	-2.54	-2.46	tissue factor pathway inhibitor (lipoprotein-associated coagulation inhibitor)	TFPI
213191_at	-4.37	-2.46	toll-like receptor adaptor molecule 1	TICAM1
203378_at	-2.92	-2.46	PCF11, cleavage and polyadenylation factor subunit, homolog (S. cerevisiae)	PCF11
203385_at	-3.14	-2.46	diacylglycerol kinase, alpha 80kDa	DGKA
226299_at	-2.90	-2.45	protein kinase N3	PKN3
200911_s_at	-3.52	-2.45	transforming, acidic coiled-coil containing protein 1	TACC1
213310_at	-4.92	-2.45		

1555978_s_at	-3.62	-2.45		
239474_at	-2.37	-2.45		
205366_s_at	-4.57	-2.45	homeobox B6	HOXB6
217677_at	-2.43	-2.45	pleckstrin homology domain containing, family A (phosphoinositide binding specific) member 2	PLEKHA2
238684_at	-3.95	-2.45		
232682_at	-4.14	-2.45	peroxisomal trans-2-enoyl-CoA reductase	PECR
201647_s_at	-2.46	-2.45	scavenger receptor class B, member 2	SCARB2
204671_s_at	-2.84	-2.45	ankyrin repeat domain 6	ANKRD6
207836_s_at	-3.94	-2.45	RNA binding protein with multiple splicing	RBPMS
212327_at	-6.23	-2.45		
210018_x_at	-4.30	-2.44	mucosa associated lymphoid tissue lymphoma translocation gene 1	MALT1
225641_at	-2.04	-2.44	MADS box transcription enhancer factor 2, polypeptide D (myocyte enhancer factor 2D)	MEF2D
208178_x_at	-2.11	-2.44	triple functional domain (PTPRF interacting)	TRIO
223380_s_at	-3.24	-2.44	LATS, large tumor suppressor, homolog 2 (Drosophila)	LATS2
224204_x_at	-2.01	-2.44	aryl hydrocarbon receptor nuclear translocator-like 2	ARNTL2
212377_s_at	-2.13	-2.44	Notch homolog 2 (Drosophila)	NOTCH2
201431_s_at	-3.05	-2.44	dihydropyrimidinase-like 3	DPYSL3
209426_s_at	-2.57	-2.43	alpha-methylacyl-CoA racemase	AMACR
211272_s_at	-24.88	-2.43	diacylglycerol kinase, alpha 80kDa	DGKA
209410_s_at	-3.86	-2.43	growth factor receptor-bound protein 10	GRB10
227067_x_at	-4.27	-2.43	neuroblastoma breakpoint family, member 1	NBPF1
234974_at	-2.41	-2.43	galactose mutarotase (aldose 1-epimerase)	GALM
37005_at	-4.19	-2.43	neuroblastoma, suppression of tumorigenicity 1	NBL1
225306_s_at	-3.07	-2.43	solute carrier family 25, member 29	SLC25A29
212586_at	-3.32	-2.43	calpastatin	CAST
202084_s_at	-3.06	-2.42	SEC14-like 1 (S. cerevisiae)	SEC14L1
206176_at	-3.71	-2.42	bone morphogenetic protein 6	BMP6
201670_s_at	-3.74	-2.42	myristoylated alanine-rich protein kinase C substrate	MARCKS
202006_at	-6.49	-2.42	protein tyrosine phosphatase, non-receptor type 12	PTPN12
227383_at	-4.51	-2.42	family with sequence similarity 91, member A2	FAM91A2
1565627_a_at	-2.18	-2.42		
203693_s_at	-2.49	-2.42	E2F transcription factor 3	E2F3
207735_at	-4.18	-2.42	ring finger protein 125	RNF125
50221_at	-9.74	-2.42	transcription factor EB	TFEB

201328_at	-3.45	-2.42	v-ets erythroblastosis virus E26 oncogene homolog 2 (avian)	ETS2
225632_s_at	-4.19	-2.42	RAB43, member RAS oncogene family	RAB43
224641_at	-2.86	-2.42	forty-two-three domain containing 1	FYTTD1
219905_at	-2.84	-2.42	erythroblast membrane-associated protein (Scianna blood group)	ERMAP
204048_s_at	-2.25	-2.42	phosphatase and actin regulator 2	PHACTR2
244377_at	-2.41	-2.42	solute carrier family 1 (glutamate/neutral amino acid transporter), member 4	SLC1A4
235233_s_at	-2.70	-2.42		
241900_at	-14.40	-2.41		
205192_at	-8.06	-2.41	mitogen-activated protein kinase kinase kinase 14	MAP3K14
225978_at	-4.49	-2.41		
239448_at	-3.82	-2.41		
221223_x_at	-3.77	-2.41	cytokine inducible SH2-containing protein	CISH
217750_s_at	-2.33	-2.41	ubiquitin-conjugating enzyme E2Z (putative)	UBE2Z
225005_at	-2.71	-2.41	PHD finger protein 13	PHF13
1559584_a_at	-5.88	-2.41	chromosome 16 open reading frame 54	C16orf54
227014_at	-3.06	-2.41	aspartate beta-hydroxylase domain containing 2	ASPHD2
228708_at	-3.09	-2.41		
204490_s_at	-4.94	-2.41	CD44 molecule (Indian blood group)	CD44
212602_at	-2.17	-2.41	WD repeat and FYVE domain containing 3	WDFY3
213940_s_at	-3.23	-2.41	formin binding protein 1	FNBP1
212607_at	-2.58	-2.40	v-akt murine thymoma viral oncogene homolog 3 (protein kinase B, gamma)	AKT3
201959_s_at	-2.20	-2.40	MYC binding protein 2	MYCBP2
203773_x_at	-3.84	-2.40	biliverdin reductase A	BLVRA
225827_at	-3.92	-2.40	eukaryotic translation initiation factor 2C, 2	EIF2C2
212948_at	-2.41	-2.40	calmodulin binding transcription activator 2	CAMTA2
203505_at	-2.13	-2.40	ATP-binding cassette, sub-family A (ABC1), member 1	ABCA1
218229_s_at	-3.91	-2.40	pogo transposable element with KRAB domain	POGK
238948_at	-3.13	-2.40	transmembrane 9 superfamily member 1	TM9SF1
205965_at	-4.30	-2.40	basic leucine zipper transcription factor, ATF-like	BATF
226183_at	-2.04	-2.40		
223216_x_at	-3.69	-2.40	zinc finger protein 395	ZNF395
204049_s_at	-2.25	-2.40	phosphatase and actin regulator 2	PHACTR2
235971_at	-2.33	-2.40		
222173_s_at	-3.94	-2.39	TBC1 domain family, member 2	TBC1D2

201581_at	-3.10	-2.39	thioredoxin domain containing 13	TXNDC13
225701_at	-2.21	-2.39	AT-hook transcription factor	AKNA
201883_s_at	-3.12	-2.39	UDP-Gal:betaGlcNAc beta 1,4-galactosyltransferase, polypeptide 1	B4GALT1
1555781_at	-3.18	-2.39	PQ loop repeat containing 2	PQLC2
224925_at	-10.25	-2.39		
225060_at	-2.56	-2.39	low density lipoprotein receptor-related protein 11	LRP11
1559203_s_at	-2.70	-2.39	v-Ki-ras2 Kirsten rat sarcoma viral oncogene homolog	KRAS
225738_at	-4.10	-2.39	Rap guanine nucleotide exchange factor (GEF) 1	RAPGEF1
201945_at	-20.12	-2.39	furin (paired basic amino acid cleaving enzyme)	FURIN
203820_s_at	-2.62	-2.39	insulin-like growth factor 2 mRNA binding protein 3	IGF2BP3
230424_at	-3.11	-2.39	chromosome 5 open reading frame 13	C5orf13
204489_s_at	-5.63	-2.39	CD44 molecule (Indian blood group)	CD44
209561_at	-2.07	-2.38	thrombospondin 3	THBS3
222303_at	-3.42	-2.38		
222859_s_at	-2.41	-2.38	dual adaptor of phosphotyrosine and 3-phosphoinositides	DAPP1
206157_at	-2.48	-2.38	pentraxin-related gene, rapidly induced by IL-1 beta	PTX3
226251_at	-2.44	-2.38	additional sex combs like 2 (Drosophila)	ASXL2
241692_at	-3.62	-2.38		
202193_at	-2.76	-2.38	LIM domain kinase 2	LIMK2
202239_at	-2.53	-2.38	poly (ADP-ribose) polymerase family, member 4	PARP4
55093_at	-4.09	-2.38		
212322_at	-2.32	-2.38	sphingosine-1-phosphate lyase 1	SGPL1
211924_s_at	-9.51	-2.37	plasminogen activator, urokinase receptor	PLAUR
205702_at	-5.27	-2.37	putative homeodomain transcription factor 1	PHTF1
230466_s_at	-4.57	-2.37		
202365_at	-2.25	-2.37		
244313_at	-2.15	-2.37	complement component (3b/4b) receptor 1 (Knops blood group)	CR1
212115_at	-4.32	-2.37	hematological and neurological expressed 1-like	HN1L
212989_at	-4.19	-2.37	transmembrane protein 23	TMEM23
223081_at	-2.85	-2.37	PHD finger protein 23	PHF23
228588_s_at	-2.37	-2.37	ubiquitin-conjugating enzyme E2B (RAD6 homolog)	UBE2B
226542_at	-3.00	-2.37		
224685_at	-2.80	-2.37	myeloid/lymphoid or mixed-lineage leukemia (trithorax homolog, Drosophila); translocated to, 4	MLLT4
212860_at	-2.19	-2.37	zinc finger, DHHC-type containing 18	ZDHHC18

212372_at	-4.75	-2.36	myosin, heavy chain 10, non-muscle	MYH10
228258_at	-4.68	-2.36	TBC1 domain family, member 10C	TBC1D10C
212473_s_at	-3.46	-2.36	microtubule associated monoxygenase, calponin and LIM domain containing 2	MICAL2
204226_at	-2.50	-2.36	staufen, RNA binding protein, homolog 2 (Drosophila)	STAU2
1556590_s_at	-3.90	-2.36		
225785_at	-4.61	-2.36	receptor accessory protein 3	REEP3
209050_s_at	-5.36	-2.36	ral guanine nucleotide dissociation stimulator	RALGDS
241873_at	-3.59	-2.36		
214121_x_at	-2.42	-2.36	PDZ and LIM domain 7 (enigma)	PDLIM7
218705_s_at	-2.50	-2.36	sorting nexing 24	SNX24
202466_at	-2.18	-2.36	polymerase (DNA directed) sigma	POLS
209435_s_at	-8.40	-2.36	rho/rac guanine nucleotide exchange factor (GEF) 2	ARHGEF2
239449_at	-2.56	-2.36	ankylosis, progressive homolog (mouse)	ANKH
202443_x_at	-2.40	-2.36	Notch homolog 2 (Drosophila)	NOTCH2
202880_s_at	-2.45	-2.36	pleckstrin homology, Sec7 and coiled-coil domains 1(cytohesin 1)	PSCD1
201861_s_at	-3.59	-2.36	leucine rich repeat (in FLII) interacting protein 1	LRRFIP1
224909_s_at	-5.65	-2.36		
202081_at	-4.47	-2.36	immediate early response 2	IER2
210870_s_at	-2.35	-2.35	epilepsy, progressive myoclonus type 2A, Lafora disease (laforin)	EPM2A
220615_s_at	-4.25	-2.35	male sterility domain containing 1	MLSTD1
209269_s_at	-2.15	-2.35		
201250_s_at	-4.25	-2.35	solute carrier family 2 (facilitated glucose transporter), member 1	SLC2A1
231815_at	-2.29	-2.35	PHD finger protein 12	PHF12
221903_s_at	-2.39	-2.35	cylindromatosis (turban tumor syndrome)	CYLD
226946_at	-4.94	-2.35	chromosome 5 open reading frame 33	C5orf33
238451_at	-5.35	-2.35	membrane protein, palmitoylated 7 (MAGUK p55 subfamily member 7)	MPP7
206669_at	-8.18	-2.35	glutamate decarboxylase 1 (brain, 67kDa)	GAD1
235841_at	-3.72	-2.35		
202051_s_at	-2.31	-2.34	zinc finger, MYM-type 4	ZMYM4
222409_at	-2.09	-2.34	coronin, actin binding protein, 1C	CORO1C
218626_at	-3.59	-2.34	eukaryotic translation initiation factor 4E nuclear import factor 1	EIF4ENIF1
206687_s_at	-6.26	-2.34	protein tyrosine phosphatase, non-receptor type 6	PTPN6
200760_s_at	-2.26	-2.34	ADP-ribosylation-like factor 6 interacting protein 5	ARL6IP5
211596_s_at	-3.63	-2.34	leucine-rich repeats and immunoglobulin-like domains 1	LRIG1
225564_at	-2.30	-2.34	spermatogenesis associated 13	SPATA13

235657_at	-2.59	-2.34		
204038_s_at	-9.38	-2.34	endothelial differentiation, lysophosphatidic acid G-protein-coupled receptor, 2	EDG2
225197_at	-2.90	-2.34	chromosome 16 open reading frame 72	C16orf72
208944_at	-2.21	-2.34	transforming growth factor, beta receptor II (70/80kDa)	TGFBR2
224765_at	-3.50	-2.34		
210162_s_at	-4.22	-2.34	nuclear factor of activated T-cells, cytoplasmic, calcineurin-dependent 1	NFATC1
219344_at	-13.18	-2.34	solute carrier family 29 (nucleoside transporters), member 3	SLC29A3
225791_at	-2.93	-2.34	ubiquitin-conjugating enzyme E2F (putative)	UBE2F
226284_at	-2.82	-2.34	zinc finger and BTB domain containing 2	ZBTB2
238015_at	-3.89	-2.33		
1563772_a_at	-2.28	-2.33	laminin, alpha 3	LAMA3
218175_at	-3.42	-2.33	coiled-coil domain containing 92	CCDC92
227420_at	-2.15	-2.33	tumor necrosis factor, alpha-induced protein 8-like 1	TNFAIP8L1
221867_at	-2.94	-2.33		
241925_x_at	-2.43	-2.33		
1569346_a_at	-5.15	-2.33		
214163_at	-2.10	-2.33	chromosome 1 open reading frame 41	C1orf41
227539_at	-2.26	-2.33		
207904_s_at	-2.77	-2.33	leucyl/cystinyl aminopeptidase	LNPEP
224939_at	-2.95	-2.33		
204032_at	-4.87	-2.33	breast cancer anti-estrogen resistance 3	BCAR3
225446_at	-2.69	-2.32	bromodomain and WD repeat domain containing 1	BRWD1
219653_at	-2.23	-2.32	LSM14B, SCD6 homolog B (S. cerevisiae)	LSM14B
242230_at	-6.16	-2.32	ataxin 1	ATXN1
229299_at	-4.16	-2.32	chromosome 5 open reading frame 33	C5orf33
209905_at	-2.15	-2.32	homeobox A9	HOXA9
221551_x_at	-3.02	-2.32	ST6 (alpha-N-acetyl-neuraminyl-2,3-beta-galactosyl-1,3)-N-acetylgalactosaminide alpha-2,6-sialyltran	ST6GALNAC4
226832_at	-2.74	-2.32		
207738_s_at	-2.64	-2.32	NCK-associated protein 1	NCKAP1
214319_at	-2.56	-2.32	furry homolog (Drosophila)	FRY
232690_at	-3.52	-2.32		
222777_s_at	-2.59	-2.31	Wolf-Hirschhorn syndrome candidate 1	WHSC1
225018_at	-3.29	-2.31	spire homolog 1 (Drosophila)	SPIRE1
224733_at	-5.17	-2.31	CKLF-like MARVEL transmembrane domain containing 3	CMTM3

214221_at	-2.32	-2.31	Alstrom syndrome 1	ALMS1
203729_at	-3.58	-2.31	epithelial membrane protein 3	EMP3
214663_at	-4.30	-2.31	receptor interacting protein kinase 5	RIPK5
228062_at	-4.74	-2.31	nucleosome assembly protein 1-like 5	NAP1L5
219777_at	-2.57	-2.31	GTPase, IMAP family member 6	GIMAP6
212475_at	-5.83	-2.31	KIAA0241	KIAA0241
207467_x_at	-2.80	-2.31	calpastatin	CAST
226219_at	-5.72	-2.31	Rho GTPase activating protein 30	ARHGAP30
212689_s_at	-2.86	-2.30	jumonji domain containing 1A	JMJD1A
221937_at	-4.37	-2.30		
203925_at	-3.93	-2.30	glutamate-cysteine ligase, modifier subunit	GCLM
200952_s_at	-4.15	-2.30	cyclin D2	CCND2
218418_s_at	-2.09	-2.30	ankyrin repeat domain 25	ANKRD25
217943_s_at	-4.27	-2.30	MAP7 domain containing 1	MAP7D1
218798_at	-3.71	-2.30		
1552986_at	-16.37	-2.30		
211433_x_at	-2.93	-2.30	KIAA1539	KIAA1539
210252_s_at	-2.54	-2.30	MAP-kinase activating death domain	MADD
202369_s_at	-2.86	-2.30	translocation associated membrane protein 2	TRAM2
226981_at	-2.11	-2.30	myeloid/lymphoid or mixed-lineage leukemia (trithorax homolog, Drosophila)	MLL
1555852_at	-2.91	-2.30	proteasome (prosome, macropain) subunit, beta type, 9 (large multifunctional peptidase 2)	PSMB9
227020_at	-2.75	-2.30	yippee-like 2 (Drosophila)	YPEL2
212267_at	-2.70	-2.30	wings apart-like homolog (Drosophila)	WAPAL
226143_at	-4.52	-2.30	retinoic acid induced 1	RAI1
1558801_at	-2.88	-2.29		
229908_s_at	-2.00	-2.29		
241353_s_at	-4.57	-2.29		
232000_at	-2.46	-2.29	chromosome 9 open reading frame 52	C9orf52
210361_s_at	-2.39	-2.28	E74-like factor 2 (ets domain transcription factor)	ELF2
213620_s_at	-3.64	-2.28	intercellular adhesion molecule 2	ICAM2
204222_s_at	-5.44	-2.28	GLI pathogenesis-related 1 (glioma)	GLIPR1
221799_at	-3.55	-2.28		
216925_s_at	-3.87	-2.28	T-cell acute lymphocytic leukemia 1	TAL1
230546_at	-3.57	-2.28	vasohibin 1	VASH1
241881_at	-5.20	-2.28	olfactory receptor, family 2, subfamily W, member 3	OR2W3
204502_at	-4.57	-2.28	SAM domain and HD domain 1	SAMHD1
201798_s_at	-7.56	-2.28	fer-1-like 3, myoferlin (C. elegans)	FER1L3
204254_s_at	-2.20	-2.28	vitamin D (1,25- dihydroxyvitamin D3) receptor	VDR
240449_at	-4.87	-2.28	zinc finger protein 341	ZNF341
222258_s_at	-4.10	-2.28	SH3-domain binding protein 4	SH3BP4

217894_at	-2.15	-2.28	potassium channel tetramerisation domain containing 3	KCTD3
222108_at	-3.36	-2.28	adhesion molecule with Ig-like domain 2	AMIGO2
215446_s_at	-2.42	-2.27	lysyl oxidase	LOX
1558522_at	-2.45	-2.27		
209420_s_at	-3.51	-2.27	sphingomyelin phosphodiesterase 1, acid lysosomal (acid sphingomyelinase)	SMPD1
228551_at	-4.52	-2.27		
204527_at	-3.12	-2.27	myosin VA (heavy chain 12, myoxin)	MYO5A
224747_at	-3.35	-2.27	ubiquitin-conjugating enzyme E2Q (putative) 2	UBE2Q2
203476_at	-4.64	-2.27	trophoblast glycoprotein	TPBG
226773_at	-2.81	-2.27		
209271_at	-2.90	-2.27	bromodomain PHD finger transcription factor	BPTF
204481_at	-2.28	-2.27	bromodomain and PHD finger containing, 1	BRPF1
223580_at	-3.01	-2.27	splA/ryanodine receptor domain and SOCS box containing 2	SPSB2
240458_at	-2.06	-2.27	inositol 1,4,5-triphosphate receptor, type 2	ITPR2
219892_at	-3.10	-2.27	transmembrane 6 superfamily member 1	TM6SF1
227470_at	-5.11	-2.27	zinc finger protein 553	ZNF553
1556682_s_at	-9.15	-2.27		
1556471_at	-2.78	-2.27	sex comb on midleg-like 4 (Drosophila)	SCML4
202419_at	-2.18	-2.27	follicular lymphoma variant translocation 1	FVT1
202100_at	-3.63	-2.27	v-ral simian leukemia viral oncogene homolog B (ras related; GTP binding protein)	RALB
225935_at	-2.93	-2.27		
225325_at	-2.38	-2.27		
236539_at	-5.36	-2.27	protein tyrosine phosphatase, non-receptor type 22 (lymphoid)	PTPN22
219911_s_at	-5.94	-2.27	solute carrier organic anion transporter family, member 4A1	SLCO4A1
225909_at	-2.23	-2.27	zinc finger protein 775	ZNF775
235690_at	-2.12	-2.27	zinc finger protein 594	ZNF594
1555847_a_at	-10.01	-2.27		
238622_at	-3.58	-2.26	RAP2B, member of RAS oncogene family	RAP2B
239680_at	-2.15	-2.26		
205013_s_at	-3.28	-2.26	adenosine A2a receptor	ADORA2A
210039_s_at	-4.07	-2.26	protein kinase C, theta	PRKCQ
231996_at	-2.63	-2.26		
215716_s_at	-3.19	-2.26	ATPase, Ca++ transporting, plasma membrane 1	ATP2B1
203870_at	-3.40	-2.26	ubiquitin specific peptidase 46	USP46
225658_at	-3.47	-2.26		

210878_s_at	-2.01	-2.26	jumonji domain containing 1B	JMJD1B
212463_at	-2.25	-2.25	CD59 molecule, complement regulatory protein	CD59
64418_at	-2.42	-2.25		
201598_s_at	-2.95	-2.25	inositol polyphosphate phosphatase-like 1	INPPL1
227312_at	-2.09	-2.25	syntrophin, beta 2 (dystrophin-associated protein A1, 59kDa, basic component 2)	SNTB2
228937_at	-2.55	-2.25	chromosome 13 open reading frame 31	C13orf31
225999_at	-3.19	-2.25	family with sequence similarity 80, member B	FAM80B
218363_at	-2.85	-2.25	exonuclease 3'-5' domain-like 2	EXDL2
242088_at	-2.17	-2.25	kelch-like 24 (Drosophila)	KLHL24
229492_at	-3.16	-2.25	vang-like 1 (van gogh, Drosophila)	VANGL1
242834_at	-2.49	-2.25		
1559117_at	-31.61	-2.25		
212486_s_at	-2.83	-2.25		
201965_s_at	-3.53	-2.24	senataxin	SETX
222233_s_at	-3.24	-2.24	DNA cross-link repair 1C (PSO2 homolog, S. cerevisiae)	DCLRE1C
229997_at	-4.88	-2.24	vang-like 1 (van gogh, Drosophila)	VANGL1
237768_x_at	-2.34	-2.24		
228677_s_at	-2.87	-2.24		
212314_at	-3.39	-2.24		
219722_s_at	-4.55	-2.24	glycerophosphodiester phosphodiesterase domain containing 3	GDPD3
201353_s_at	-3.39	-2.24	bromodomain adjacent to zinc finger domain, 2A	BAZ2A
207318_s_at	-2.81	-2.24	cell division cycle 2-like 5 (cholinesterase-related cell division controller)	CDC2L5
222116_s_at	-4.01	-2.24	TBC1 domain family, member 16	TBC1D16
213147_at	-2.46	-2.24	homeobox A10	HOXA10
228914_at	-2.55	-2.24		
202988_s_at	-4.26	-2.24	regulator of G-protein signalling 1	RGS1
229344_x_at	-2.45	-2.23	family with sequence similarity 80, member B	FAM80B
1554414_a_at	-3.19	-2.23	oxidative stress induced growth inhibitor family member 2	OSGIN2
1554453_at	-4.64	-2.23	heterogeneous nuclear ribonucleoprotein L-like	HNRPLL
225711_at	-3.09	-2.23	ADP-ribosylation-like factor 6 interacting protein 6	ARL6IP6
223398_at	-3.71	-2.23	chromosome 9 open reading frame 89	C9orf89
207761_s_at	-2.97	-2.23	methyltransferase like 7A	METTL7A
220416_at	-4.11	-2.23	ATPase, Class I, type 8B, member 4	ATP8B4
60471_at	-8.06	-2.22	Ras and Rab interactor 3	RIN3
229785_at	-4.49	-2.22	KRIT1, ankyrin repeat containing	KRIT1
243444_at	-2.71	-2.22		

219330_at	-4.85	-2.22	vang-like 1 (van gogh, Drosophila)	VANGL1
223961_s_at	-75.34	-2.22	cytokine inducible SH2-containing protein	CISH
229373_at	-10.34	-2.22		
209835_x_at	-4.53	-2.22	CD44 molecule (Indian blood group)	CD44
1555920_at	-5.39	-2.22	chromobox homolog 3 (HP1 gamma homolog, Drosophila)	CBX3
212711_at	-2.62	-2.22	calmodulin regulated spectrin-associated protein 1	CAMSAP1
244783_at	-2.35	-2.22	YY1 associated factor 2	YAF2
201645_at	-5.98	-2.22	tenascin C (hexabrachion)	TNC
220992_s_at	-2.05	-2.22	chromosome 1 open reading frame 25	C1orf25
221983_at	-3.03	-2.22	chromosome 2 open reading frame 17	C2orf17
218700_s_at	-2.93	-2.22	RAB7, member RAS oncogene family-like 1	RAB7L1
1556950_s_at	-2.66	-2.22	serpin peptidase inhibitor, clade B (ovalbumin), member 6	SERPINB6
219394_at	-2.93	-2.21	phosphatidylglycerophosphate synthase 1	PGS1
232884_s_at	-2.65	-2.21		
213983_s_at	-2.08	-2.21		
210233_at	-2.34	-2.21	interleukin 1 receptor accessory protein	IL1RAP
214657_s_at	-3.33	-2.21		
242157_at	-3.21	-2.21	chromodomain helicase DNA binding protein 9	CHD9
222294_s_at	-2.51	-2.21	RAB27A, member RAS oncogene family	RAB27A
206510_at	-4.06	-2.21	sine oculis homeobox homolog 2 (Drosophila)	SIX2
242366_at	-3.22	-2.21		
1554612_at	-3.34	-2.21	KIAA0226	KIAA0226
227143_s_at	-2.64	-2.21	BH3 interacting domain death agonist	BID
224776_at	-2.08	-2.21	1-acylglycerol-3-phosphate O-acyltransferase 6 (lysophosphatidic acid acyltransferase, zeta)	AGPAT6
203837_at	-2.17	-2.20	mitogen-activated protein kinase kinase kinase 5	MAP3K5
217591_at	-14.38	-2.20		
229027_at	-2.03	-2.20		
226215_s_at	-3.53	-2.20	F-box and leucine-rich repeat protein 10	FBXL10
1556589_at	-3.37	-2.20		
212651_at	-3.66	-2.20	Rho-related BTB domain containing 1	RHOBTB1
218543_s_at	-2.19	-2.20	poly (ADP-ribose) polymerase family, member 12	PARP12
208854_s_at	-3.29	-2.20	serine/threonine kinase 24 (STE20 homolog, yeast)	STK24
226765_at	-3.08	-2.20	spectrin, beta, non-erythrocytic 1	SPTBN1

209341_s_at	-2.72	-2.20	inhibitor of kappa light polypeptide gene enhancer in B-cells, kinase beta	IKBKB
203044_at	-2.69	-2.20	carbohydrate (chondroitin) synthase 1	CHSY1
230389_at	-2.53	-2.20	formin binding protein 1	FNBP1
223093_at	-2.63	-2.19	ankylosis, progressive homolog (mouse)	ANKH
218176_at	-2.56	-2.19	melanoma antigen family F, 1	MAGEF1
214964_at	-2.85	-2.19		
212014_x_at	-4.36	-2.19	CD44 molecule (Indian blood group)	CD44
203600_s_at	-3.93	-2.19	chromosome 4 open reading frame 8	C4orf8
207319_s_at	-2.43	-2.19	cell division cycle 2-like 5 (cholinesterase-related cell division controller)	CDC2L5
231049_at	-4.92	-2.19	LIM domain only 2 (rhombotin-like 1)	LMO2
1552625_a_at	-2.56	-2.19	tRNA nucleotidyl transferase, CCA-adding, 1	TRNT1
235295_at	-2.63	-2.19		
243395_at	-3.38	-2.18		
228073_at	-2.58	-2.18	N-acetylneuraminic acid phosphatase	NANP
229814_at	-2.24	-2.18		
227804_at	-2.14	-2.18	TLC domain containing 1	TLCD1
222471_s_at	-2.77	-2.18	potassium channel modulatory factor 1	KCMF1
213725_x_at	-2.92	-2.18	xylosyltransferase I	XYLT1
217889_s_at	-4.31	-2.18	cytochrome b reductase 1	CYBRD1
218262_at	-4.20	-2.18	required for meiotic nuclear division 5 homolog B (S. cerevisiae)	RMND5B
202860_at	-3.70	-2.18	DENN/MADD domain containing 4B	DENND4B
225115_at	-2.62	-2.18	homeodomain interacting protein kinase 2	HIPK2
231964_at	-2.22	-2.18		
202478_at	-2.63	-2.18	tribbles homolog 2 (Drosophila)	TRIB2
226485_at	-3.49	-2.18		
230263_s_at	-3.26	-2.18	dedicator of cytokinesis 5	DOCK5
1570165_at	-3.82	-2.17		
218041_x_at	-3.96	-2.17	solute carrier family 38, member 2	SLC38A2
203075_at	-2.32	-2.17	SMAD family member 2	SMAD2
204549_at	-3.20	-2.17	inhibitor of kappa light polypeptide gene enhancer in B-cells, kinase epsilon	IKBKE
214356_s_at	-2.50	-2.17		
1568592_at	-2.71	-2.17	tripartite motif-containing 69	TRIM69
228869_at	-2.71	-2.17		
225447_at	-2.97	-2.17	glycerol-3-phosphate dehydrogenase 2 (mitochondrial)	GPD2
221485_at	-2.30	-2.17	UDP-Gal:betaGlcNAc beta 1,4-galactosyltransferase, polypeptide 5	B4GALT5
1558426_x_at	-3.17	-2.17	transmembrane protein 142B	TMEM142B
228565_at	-2.48	-2.17		
244774_at	-2.23	-2.17		

223805_at	-2.61	-2.16	oxysterol binding protein-like 6	OSBPL6
226368_at	-2.58	-2.16	carbohydrate (chondroitin 4) sulfotransferase 11	CHST11
211077_s_at	-2.50	-2.16	tousled-like kinase 1	TLK1
222793_at	-2.70	-2.16	DEAD (Asp-Glu-Ala-Asp) box polypeptide 58	DDX58
202173_s_at	-4.50	-2.16	vascular endothelial zinc finger 1	VEZF1
209106_at	-3.89	-2.16	nuclear receptor coactivator 1	NCOA1
244461_at	-2.88	-2.16	sperm antigen with calponin homology and coiled-coil domains 1	SPECC1
205510_s_at	-3.12	-2.16		
226909_at	-2.98	-2.16		
212146_at	-2.04	-2.16	pleckstrin homology domain containing, family M (with RUN domain) member 2	PLEKHM2
217682_at	-2.29	-2.16		
1557316_at	-2.42	-2.16		
200899_s_at	-2.33	-2.16	meningioma expressed antigen 5 (hyaluronidase)	MGEA5
229713_at	-2.14	-2.15		
225411_at	-2.56	-2.15		
227167_s_at	-2.70	-2.15		
1552664_at	-2.55	-2.15	folliculin	FLCN
226473_at	-5.27	-2.15	chromobox homolog 2 (Pc class homolog, Drosophila)	CBX2
201234_at	-5.16	-2.15	integrin-linked kinase	ILK
232001_at	-2.65	-2.15		
224215_s_at	-2.57	-2.15	delta-like 1 (Drosophila)	DLL1
217824_at	-2.20	-2.15	ubiquitin-conjugating enzyme E2, J1 (UBC6 homolog, yeast)	UBE2J1
244653_at	-3.18	-2.15	SET domain containing (lysine methyltransferase) 7	SETD7
239719_at	-2.42	-2.15	CD109 molecule	CD109
213689_x_at	-2.02	-2.15	ribosomal protein L5	RPL5
219501_at	-2.11	-2.15		
224458_at	-4.35	-2.15	chromosome 9 open reading frame 125	C9orf125
222597_at	-15.23	-2.15	synaptosomal-associated protein, 29kDa	SNAP29
204520_x_at	-4.31	-2.15	bromodomain containing 1	BRD1
229274_at	-4.13	-2.15	GNAS complex locus	GNAS
213792_s_at	-2.15	-2.15		
1555294_a_at	-2.89	-2.15	ELKS/RAB6-interacting/CAST family member 1	ERC1
206034_at	-2.57	-2.14	serpin peptidase inhibitor, clade B (ovalbumin), member 8	SERPINB8
212646_at	-2.98	-2.14	raftlin, lipid raft linker 1	RFTN1
222799_at	-2.32	-2.14		
229167_at	-3.42	-2.14		
235292_at	-2.02	-2.14		
227049_at	-4.65	-2.14		
227531_at	-3.39	-2.14		

201963_at	-5.14	-2.14	acyl-CoA synthetase long-chain family member 1	ACSL1
236922_at	-2.28	-2.14		
45749_at	-2.70	-2.14	family with sequence similarity 65, member A	FAM65A
218854_at	-2.01	-2.14	dermatan sulfate epimerase	DSE
204789_at	-2.57	-2.14	formin-like 1	FMNL1
214651_s_at	-2.02	-2.13	homeobox A9	HOXA9
222142_at	-2.03	-2.13	cylindromatosis (turban tumor syndrome)	CYLD
213168_at	-2.17	-2.13	Sp3 transcription factor	SP3
38398_at	-2.61	-2.13	MAP-kinase activating death domain	MADD
213153_at	-4.43	-2.13	SET domain containing 1B	SETD1B
210916_s_at	-4.47	-2.13	CD44 molecule (Indian blood group)	CD44
218928_s_at	-5.24	-2.13	solute carrier family 37 (glycerol-3-phosphate transporter), member 1	SLC37A1
231312_at	-2.33	-2.13		
205210_at	-2.70	-2.13	transforming growth factor, beta receptor associated protein 1	TGFBRAP1
201594_s_at	-3.00	-2.13	protein phosphatase 4, regulatory subunit 1	PPP4R1
218819_at	-2.52	-2.13	integrator complex subunit 6	INTS6
209822_s_at	-2.22	-2.13	very low density lipoprotein receptor	VLDLR
213258_at	-2.93	-2.13	tissue factor pathway inhibitor (lipoprotein-associated coagulation inhibitor)	TFPI
36129_at	-2.47	-2.13	RUN and TBC1 domain containing 1	RUTBC1
217234_s_at	-2.92	-2.13	villin 2 (ezrin)	VIL2
243561_at	-2.53	-2.13		
227692_at	-2.57	-2.13	guanine nucleotide binding protein (G protein), alpha inhibiting activity polypeptide 1	GNAI1
225033_at	-49.39	-2.13		
210754_s_at	-3.66	-2.12	v-yes-1 Yamaguchi sarcoma viral related oncogene homolog	LYN
200791_s_at	-2.26	-2.12	IQ motif containing GTPase activating protein 1	IQGAP1
225897_at	-2.61	-2.12		
232129_s_at	-2.36	-2.12	leucine zipper, putative tumor suppressor 2	LZTS2
209446_s_at	-9.78	-2.12	chromosome 7 open reading frame 44	C7orf44
220305_at	-3.14	-2.12		
221905_at	-3.15	-2.12	cylindromatosis (turban tumor syndrome)	CYLD
205242_at	-30.33	-2.12	chemokine (C-X-C motif) ligand 13 (B-cell chemoattractant)	CXCL13
203297_s_at	-2.08	-2.12	jumonji, AT rich interactive domain 2	JARID2
201995_at	-2.26	-2.12	exostoses (multiple) 1	EXT1
214030_at	-2.01	-2.12		
226440_at	-2.81	-2.12	dual specificity phosphatase 22	DUSP22
203188_at	-4.84	-2.12	UDP-GlcNAc:betaGal beta-1,3-N-acetylglucosaminyltransferase 1	B3GNT1

208623_s_at	-2.59	-2.12	villin 2 (ezrin)	VIL2
202510_s_at	-2.28	-2.12	tumor necrosis factor, alpha-induced protein 2	TNFAIP2
218456_at	-3.37	-2.12	C1q domain containing 1	C1QDC1
209542_x_at	-4.87	-2.12	insulin-like growth factor 1 (somatomedin C)	IGF1
226372_at	-3.47	-2.12	carbohydrate (chondroitin 4) sulfotransferase 11	CHST11
243928_s_at	-2.24	-2.12	ATP-binding cassette, sub-family C (CFTR/MRP), member 4	ABCC4
218032_at	-2.79	-2.12	stannin	SNN
221858_at	-2.07	-2.12	TBC1 domain family, member 12	TBC1D12
226689_at	-3.33	-2.12	zinc finger, CDGSH-type domain 2	ZCD2
205389_s_at	-2.59	-2.11	ankyrin 1, erythrocytic	ANK1
213010_at	-6.55	-2.11	protein kinase C, delta binding protein	PRKCDBP
1559401_a_at	-2.21	-2.11		
210846_x_at	-2.50	-2.11	tripartite motif-containing 14	TRIM14
225814_at	-2.06	-2.11	5'-3' exoribonuclease 1	XRN1
208485_x_at	-3.25	-2.11	CASP8 and FADD-like apoptosis regulator	CFLAR
200984_s_at	-2.90	-2.11	CD59 molecule, complement regulatory protein	CD59
203420_at	-2.42	-2.11	family with sequence similarity 8, member A1	FAM8A1
209457_at	-2.78	-2.11	dual specificity phosphatase 5	DUSP5
205594_at	-3.61	-2.11	zinc finger protein 652	ZNF652
225764_at	-2.58	-2.11	ets variant gene 6 (TEL oncogene)	ETV6
226830_x_at	-3.11	-2.11	chromodomain helicase DNA binding protein 2	CHD2
205247_at	-2.09	-2.11	Notch homolog 4 (Drosophila)	NOTCH4
204413_at	-2.40	-2.11	TNF receptor-associated factor 2	TRAF2
212233_at	-2.47	-2.11		
213579_s_at	-2.88	-2.10	E1A binding protein p300	EP300
224705_s_at	-2.24	-2.10	trinucleotide repeat containing 6A	TNRC6A
235595_at	-2.19	-2.10	rho/rac guanine nucleotide exchange factor (GEF) 2	ARHGEF2
221526_x_at	-5.26	-2.10	par-3 partitioning defective 3 homolog (C. elegans)	PARD3
203003_at	-2.65	-2.10	MADS box transcription enhancer factor 2, polypeptide D (myocyte enhancer factor 2D)	MEF2D
201669_s_at	-2.46	-2.10	myristoylated alanine-rich protein kinase C substrate	MARCKS
226018_at	-2.49	-2.10	chromosome 7 open reading frame 41	C7orf41
223377_x_at	-3.14	-2.10	cytokine inducible SH2-containing protein	CISH
222494_at	-2.17	-2.10	checkpoint suppressor 1	CHES1
209540_at	-2.62	-2.10	insulin-like growth factor 1 (somatomedin C)	IGF1
209925_at	-17.31	-2.10	occludin	OCLN
1556321_a_at	-2.07	-2.10		

235061_at	-2.00	-2.10	protein phosphatase 1K (PP2C domain containing)	PPM1K
202625_at	-4.63	-2.10	v-yes-1 Yamaguchi sarcoma viral related oncogene homolog	LYN
219317_at	-8.73	-2.10	polymerase (DNA directed) iota	POLI
1560156_at	-2.38	-2.10		
218659_at	-3.10	-2.10	additional sex combs like 2 (Drosophila)	ASXL2
230999_at	-4.79	-2.09		
217910_x_at	-2.43	-2.09	MAX-like protein X	MLX
209811_at	-2.51	-2.09	caspase 2, apoptosis-related cysteine peptidase (neural precursor cell expressed, developmentally do	CASP2
212519_at	-2.33	-2.09	ubiquitin-conjugating enzyme E2E 1 (UBC4/5 homolog, yeast)	UBE2E1
227545_at	-2.66	-2.09		
225231_at	-3.10	-2.09	Cas-Br-M (murine) ecotropic retroviral transforming sequence	CBL
235478_at	-3.75	-2.09	DNA cross-link repair 1C (PSO2 homolog, S. cerevisiae)	DCLRE1C
221984_s_at	-3.50	-2.09	chromosome 2 open reading frame 17	C2orf17
201866_s_at	-2.01	-2.09	nuclear receptor subfamily 3, group C, member 1 (glucocorticoid receptor)	NR3C1
209710_at	-3.28	-2.09	GATA binding protein 2	GATA2
201126_s_at	-3.38	-2.09	mannosyl (alpha-1,3-)-glycoprotein beta-1,2-N-acetylglucosaminyltransferase	MGAT1
206748_s_at	-2.74	-2.09	sperm associated antigen 9	SPAG9
206710_s_at	-3.26	-2.09	erythrocyte membrane protein band 4.1-like 3	EPB41L3
226364_at	-3.19	-2.09	huntingtin interacting protein 1	HIP1
212471_at	-2.34	-2.08	KIAA0241	KIAA0241
224162_s_at	-2.56	-2.08	F-box protein 31	FBXO31
201471_s_at	-3.10	-2.08	sequestosome 1	SQSTM1
224318_s_at	-2.18	-2.08		
226801_s_at	-2.40	-2.08		
209305_s_at	-4.13	-2.08	growth arrest and DNA-damage-inducible, beta	GADD45B
202968_s_at	-2.74	-2.08	dual-specificity tyrosine-(Y)-phosphorylation regulated kinase 2	DYRK2
228314_at	-2.42	-2.08		
34726_at	-2.00	-2.08	calcium channel, voltage-dependent, beta 3 subunit	CACNB3
1559172_at	-2.32	-2.08		
225929_s_at	-2.17	-2.08	ring finger protein 213	RNF213
226475_at	-3.54	-2.08	family with sequence similarity 118, member A	FAM118A
212184_s_at	-2.49	-2.08	mitogen-activated protein kinase kinase kinase 7 interacting protein 2	MAP3K7IP2
1562850_at	-2.88	-2.08		

209250_at	-3.39	-2.07	degenerative spermatocyte homolog 1, lipid desaturase (Drosophila)	DEGS1
202377_at	-3.18	-2.07		
1564338_at	-10.25	-2.07		
226607_at	-2.12	-2.07	chromosome 20 open reading frame 194	C20orf194
91703_at	-2.30	-2.07	EH domain binding protein 1-like 1	EHBP1L1
215460_x_at	-5.27	-2.07	bromodomain containing 1	BRD1
203796_s_at	-3.80	-2.07	B-cell CLL/lymphoma 7A	BCL7A
235777_at	-5.77	-2.07	ankyrin repeat domain 44	ANKRD44
217734_s_at	-2.63	-2.07	WD repeat domain 6	WDR6
1569521_s_at	-2.84	-2.07		
204494_s_at	-4.72	-2.07	chromosome 15 open reading frame 39	C15orf39
225157_at	-2.52	-2.07	MLX interacting protein	MLXIP
219648_at	-2.35	-2.07	melanoregulin	MREG
1552733_at	-4.24	-2.07	kelch domain containing 1	KLHDC1
205926_at	-2.49	-2.07	interleukin 27 receptor, alpha	IL27RA
235766_x_at	-3.95	-2.06	RAB27A, member RAS oncogene family	RAB27A
200897_s_at	-2.58	-2.06	palladin, cytoskeletal associated protein	PALLD
219880_at	-2.38	-2.06		
200985_s_at	-3.18	-2.06	CD59 molecule, complement regulatory protein	CD59
202981_x_at	-2.01	-2.06	seven in absentia homolog 1 (Drosophila)	SIAH1
219183_s_at	-3.37	-2.06	pleckstrin homology, Sec7 and coiled-coil domains 4	PSCD4
214743_at	-2.44	-2.06	cut-like 1, CCAAT displacement protein (Drosophila)	CUTL1
226568_at	-3.05	-2.06	family with sequence similarity 102, member B	FAM102B
200660_at	-3.85	-2.06	S100 calcium binding protein A11	S100A11
41386_i_at	-3.87	-2.06	jumonji domain containing 3	JMJD3
1560743_a_at	-3.91	-2.06		
219457_s_at	-8.85	-2.06	Ras and Rab interactor 3	RIN3
214696_at	-2.79	-2.05		
224906_at	-2.68	-2.05	transmembrane protein 16F	TMEM16F
201220_x_at	-3.03	-2.05	C-terminal binding protein 2	CTBP2
209688_s_at	-3.22	-2.05	coiled-coil domain containing 93	CCDC93
209414_at	-5.01	-2.05	fizzy/cell division cycle 20 related 1 (Drosophila)	FZR1
209487_at	-4.12	-2.05	RNA binding protein with multiple splicing	RBPMS
201660_at	-2.52	-2.05		
227930_at	-3.24	-2.05	eukaryotic translation initiation factor 2C, 4	EIF2C4
209941_at	-2.04	-2.05	receptor (TNFRSF)-interacting serine-threonine kinase 1	RIPK1
211105_s_at	-6.12	-2.05	nuclear factor of activated T-cells, cytoplasmic, calcineurin-dependent 1	NFATC1
212856_at	-2.07	-2.05		

233353_at	-2.07	-2.05		
203397_s_at	-4.84	-2.05	UDP-N-acetyl-alpha-D-galactosamine:polypeptide N-acetylgalactosaminyltransferase 3 (GalNAc-T3)	GALNT3
224046_s_at	-2.64	-2.05	phosphodiesterase 7A	PDE7A
218871_x_at	-3.39	-2.05		
228702_at	-4.33	-2.04		
225478_at	-3.29	-2.04	malignant fibrous histiocytoma amplified sequence 1	MFHAS1
214606_at	-3.62	-2.04	tetraspanin 2	TSPAN2
225532_at	-6.49	-2.04	Cdk5 and Abl enzyme substrate 1	CABLES1
221245_s_at	-4.13	-2.04	frizzled homolog 5 (Drosophila)	FZD5
219282_s_at	-2.67	-2.04	transient receptor potential cation channel, subfamily V, member 2	TRPV2
226885_at	-2.86	-2.04		
232752_at	-4.77	-2.04		
212472_at	-2.67	-2.04	microtubule associated monoxygenase, calponin and LIM domain containing 2	MICAL2
1556474_a_at	-4.10	-2.03		
204683_at	-2.86	-2.03	intercellular adhesion molecule 2	ICAM2
45687_at	-2.53	-2.03	proline rich 14	PRR14
212465_at	-2.55	-2.03	SET domain containing 3	SETD3
224783_at	-3.52	-2.03	family with sequence similarity 100, member B	FAM100B
227036_at	-6.71	-2.03		
202417_at	-2.81	-2.03	kelch-like ECH-associated protein 1	KEAP1
202378_s_at	-2.53	-2.03	leptin receptor overlapping transcript	LEPROT
209515_s_at	-2.10	-2.03	RAB27A, member RAS oncogene family	RAB27A
226297_at	-3.26	-2.03	homeodomain interacting protein kinase 3	HIPK3
200083_at	-2.59	-2.03	ubiquitin specific peptidase 22	USP22
210007_s_at	-2.67	-2.03	glycerol-3-phosphate dehydrogenase 2 (mitochondrial)	GPD2
212285_s_at	-2.89	-2.03	agrin	AGRIN
224861_at	-2.46	-2.03		
1558807_at	-2.24	-2.03	ATPase family, AAA domain containing 2B	ATAD2B
213389_at	-2.57	-2.03	zinc finger protein 592	ZNF592
213116_at	-3.64	-2.03	NIMA (never in mitosis gene a)-related kinase 3	NEK3
229268_at	-2.13	-2.03	family with sequence similarity 105, member B	FAM105B
240824_at	-2.10	-2.02	oligonucleotide/oligosaccharide-binding fold containing 1	OBFC1
205469_s_at	-7.54	-2.02	interferon regulatory factor 5	IRF5
226774_at	-2.17	-2.02	family with sequence similarity 120B	FAM120B
226554_at	-5.98	-2.02	zinc finger and BTB domain containing 7A	ZBTB7A

206437_at	-3.38	-2.02	endothelial differentiation, lysophosphatidic acid G-protein-coupled receptor, 6	EDG6
221698_s_at	-2.62	-2.02	C-type lectin domain family 7, member A	CLEC7A
204040_at	-2.67	-2.02	ring finger protein 144	RNF144
222156_x_at	-2.77	-2.02	cell cycle progression 1	CCPG1
205098_at	-2.82	-2.02	chemokine (C-C motif) receptor 1	CCR1
204491_at	-2.08	-2.02	phosphodiesterase 4D, cAMP-specific (phosphodiesterase E3 dunce homolog, Drosophila)	PDE4D
1564301_a_at	-2.18	-2.02	RPA interacting protein	RPAIN
201494_at	-2.64	-2.02	prolylcarboxypeptidase (angiotensinase C)	PRCP
221806_s_at	-2.72	-2.02	SET domain containing 5	SETD5
236346_at	-2.79	-2.02	zinc finger protein 83	ZNF83
205429_s_at	-2.03	-2.02	membrane protein, palmitoylated 6 (MAGUK p55 subfamily member 6)	MPP6
212976_at	-2.05	-2.01		
1561439_at	-2.81	-2.01		
225355_at	-4.21	-2.01		
221221_s_at	-3.11	-2.01	kelch-like 3 (Drosophila)	KLHL3
230707_at	-2.23	-2.01	sortilin-related receptor, L(DLR class) A repeats-containing	SORL1
240432_x_at	-7.54	-2.01		
1568752_s_at	-5.04	-2.01	regulator of G-protein signalling 13	RGS13
227344_at	-2.81	-2.01	IKAROS family zinc finger 1 (Ikaros)	IKZF1
235076_at	-2.01	-2.01	calcium binding and coiled-coil domain 2	CALCOCO2
241812_at	-2.10	-2.01		
202626_s_at	-4.27	-2.01	v-yes-1 Yamaguchi sarcoma viral related oncogene homolog	LYN
224995_at	-2.85	-2.01	spire homolog 1 (Drosophila)	SPIRE1
238787_at	-2.22	-2.01		
235179_at	-2.44	-2.01	zinc finger protein 641	ZNF641
242790_at	-3.62	-2.01		
204713_s_at	-2.87	-2.01	coagulation factor V (proaccelerin, labile factor)	F5
212573_at	-2.28	-2.01	endonuclease domain containing 1	ENDOD1
229115_at	-2.02	-2.01	dynein, cytoplasmic 1, heavy chain 1	DYNC1H1
235458_at	-11.36	-2.00	hepatitis A virus cellular receptor 2	HAVCR2
224834_at	-2.96	-2.00	ubiquitin domain containing 2	UBTD2
225707_at	-2.76	-2.00	ADP-ribosylation-like factor 6 interacting protein 6	ARL6IP6
243_g_at	-2.07	-2.00	microtubule-associated protein 4	MAP4
209110_s_at	-3.30	-2.00	ral guanine nucleotide dissociation stimulator-like 2	RGL2
221704_s_at	-3.22	-2.00	vacuolar protein sorting 37 homolog B (S. cerevisiae)	VPS37B
222780_s_at	-3.63	-2.00	brain and acute leukemia, cytoplasmic	BAALC
223325_at	-4.14	-2.00	thioredoxin domain containing 11	TXNDC11

210249_s_at	-2.99	-2.00	nuclear receptor coactivator 1	NCOA1
