# Supplementary material for: In Vitro Transformation of Primary Human CD34+ Cells by AML Fusion Oncogenes: Early Gene Expression Profiling Reveals Possible Drug Target in AML
Source: PLoS One. 2010 Aug 27;5(8):e12464. doi: 10.1371/journal.pone.0012464 (PMC2929205; doi:10.1371/journal.pone.0012464)
Supplement: Table S2 — Genes deregulated by AML1-ETO 3 days after transduction. Primary human CD34+ cells were retrovirally transduced with either control MSCV-IRES-GFP vector or vector expressing AML1-ETO and sorted for GFP positivity. Total RNA was extracted 3 days after transduction and subjected to microarray analysis. Genes that showed up- or down-regulation by 2 fold or more in comparison to the control in 2 independent experiments (Exp.1 and Exp.2) were considered deregulated. (0.10 MB PDF) [file pone.0012464.s002.pdf]

Table S2. Genes deregulated by AML1-ETO at 3 d after transduction

Probe set ID	Fold Change		Gene Name	Gene Symbol
	Exp.1	Exp.2		
242856_at	50.75	2.22		
201860_s_at	44.28	12.88	plasminogen activator, tissue	PLAT
1561511_at	17.49	3.96		
204888_s_at	17.35	41.67	neuralized homolog (Drosophila)	NEURL
222904_s_at	13.51	2.83	transmembrane channel-like 5	TMC5
237391_at	12.88	18.52		
237494_at	10.92	2.51		
216067_at	10.69	2.45		
1563088_a_at	9.18	2.15		
244557_at	8.54	11.59		
52651_at	7.58	3.29	collagen, type VIII, alpha 2	COL8A2
234612_at	7.17	4.00		
222078_at	6.40	4.23		
205255_x_at	5.97	2.76	transcription factor 7 (T-cell specific, HMG-box)	TCF7
204684_at	5.96	6.80	neuronal pentraxin I	NPTX1
205749_at	5.46	17.67	cytochrome P450, family 1, subfamily A, polypeptide 1	CYP1A1
235839_at	5.17	2.37		
204321_at	5.09	2.81	neogenin homolog 1 (chicken)	NEO1
1562311_at	5.02	6.34		
234765_at	4.86	5.63		
242014_at	4.75	3.55	zinc finger, RAN-binding domain containing 3	ZRANB3
216657_at	4.68	2.80	ataxin 3	ATXN3
1559529_at	4.53	3.73	PTK2 protein tyrosine kinase 2	PTK2
242357_x_at	4.51	2.69		
231598_x_at	4.46	3.87		
1556700_a_at	4.19	4.67		
242868_at	4.17	2.40	endothelial PAS domain protein 1	EPAS1
241184_x_at	4.16	3.60	zinc finger protein 407	ZNF407
225293_at	4.09	2.62	collagen, type XXVII, alpha 1	COL27A1
239492_at	4.06	2.53	SEC14-like 4 (S. cerevisiae)	SEC14L4
205984_at	4.03	12.01	corticotropin releasing hormone binding protein	CRHBP
241746_at	3.86	2.09	cullin 7	CUL7
1561266_at	3.75	26.02		
240254_at	3.66	2.78	TRAF2 and NCK interacting kinase	TNIK
240965_at	3.65	2.87	anaphase promoting complex subunit 10	ANAPC10
232504_at	3.64	2.31		
241030_at	3.62	3.73	fibrous sheath interacting protein 1	FSIP1
237515_at	3.61	3.01	transmembrane protein 56	TMEM56
233373_at	3.43	5.75		
1565873_at	3.42	2.19	KIAA1279	KIAA1279
1555392_at	3.38	3.51		

239913_at	3.34	5.51	solute carrier family 10 (sodium/bile acid cotransporter family), member 4	SLC10A4
238182_at	3.34	3.45		
232626_at	3.32	2.12		
220833_at	3.32	2.18		
1557520_a_at	3.29	2.70	transmembrane protein 59	TMEM59
220905_at	3.24	2.95		
213553_x_at	3.22	2.56	apolipoprotein C-I	APOC1
1567997_x_at	3.16	2.19		
206773_at	3.16	2.19	lymphocyte antigen 6 complex, locus H	LY6H
1555284_at	3.16	3.97	amyotrophic lateral sclerosis 2 (juvenile)	ALS2
1559979_at	3.15	2.43	SYF2 homolog, RNA splicing factor (S. cerevisiae)	SYF2
1553123_at	3.15	3.57	WD repeat domain 62	WDR62
204416_x_at	3.08	2.07	apolipoprotein C-I	APOC1
233223_at	3.08	3.17		
205547_s_at	3.05	3.76	transgelin	TAGLN
1555014_x_at	3.04	4.05		
239555_at	3.03	2.49		
1561225_at	3.03	2.54		
234209_at	3.02	2.77		
241154_x_at	2.99	5.07		
225270_at	2.98	3.93	neogenin homolog 1 (chicken)	NEO1
235538_at	2.98	2.92		
215743_at	2.93	2.46	N-myristoyltransferase 2	NMT2
217617_at	2.93	3.44		
238840_at	2.86	2.08	leucine rich repeat (in FLII) interacting protein 1	LRRFIP1
239956_at	2.86	3.73		
217662_x_at	2.86	3.23		
1561430_s_at	2.83	2.43	chromosome 3 open reading frame 15	C3orf15
216108_at	2.81	28.37		
210922_at	2.80	2.69		
234958_at	2.79	4.48		
243715_at	2.79	2.14		
242498_x_at	2.77	3.13	PRP40 pre-mRNA processing factor 40 homolog A (S. cerevisiae)	PRPF40A
1569238_a_at	2.77	2.04		
1553373_at	2.76	2.39	WD repeat domain 64	WDR64
234632_x_at	2.76	6.73		
1570639_at	2.72	2.30		
1565906_at	2.71	2.19	NAD synthetase 1	NADSYN1
206932_at	2.70	4.97	cholesterol 25-hydroxylase	CH25H
243428_at	2.70	2.31	KCNQ1 overlapping transcript 1	KCNQ1OT1
1569664_at	2.68	2.32		
227140_at	2.68	2.55		
204932_at	2.67	2.86	tumor necrosis factor receptor superfamily, member 11b (osteoprotegerin)	TNFRSF11B

226210_s_at	2.66	2.59	maternally expressed 3	MEG3
241770_x_at	2.66	12.45	solute carrier family 22 (organic anion/cation transporter), member 9	SLC22A9
1566424_at	2.65	2.04		
231217_at	2.65	2.30		
216062_at	2.64	5.50		
215439_x_at	2.64	2.34	synaptopodin 2	SYNPO2
215560_x_at	2.63	2.11	mitochondrial translational release factor 1-like	MTRF1L
244104_at	2.63	2.36	mannosyl (beta-1,4-)-glycoprotein beta-1,4-N-acetylglucosaminyltransferase	MGAT3
1561676_at	2.62	5.46		
229057_at	2.62	5.67	sodium channel, voltage-gated, type II, alpha subunit	SCN2A
1559009_at	2.62	3.21		
1564203_at	2.61	3.16		
215595_x_at	2.61	3.83		
1563884_at	2.60	3.57		
205950_s_at	2.60	2.69	carbonic anhydrase I	CA1
1569294_at	2.59	3.57	ring finger protein 187	RNF187
212884_x_at	2.57	2.28	apolipoprotein E	APOE
224361_s_at	2.54	5.61	interleukin 17 receptor B	IL17RB
1557394_at	2.53	2.57		
1570169_at	2.52	3.75	CUB and Sushi multiple domains 2	CSMD2
236757_at	2.51	2.10		
234199_at	2.51	2.25		
214776_x_at	2.48	5.67	xylulokinase homolog (H. influenzae)	XYLB
1553335_x_at	2.48	5.20		
234753_x_at	2.48	2.81		
236653_at	2.47	2.34		
1557410_at	2.47	4.29		
236742_at	2.46	2.14		
1565876_x_at	2.44	2.38	nucleoporin 153kDa	NUP153
1561571_at	2.42	2.34		
234503_at	2.42	3.57		
214375_at	2.41	2.58	PTPRF interacting protein, binding protein 1 (liprin beta 1)	PPFIBP1
216773_at	2.40	3.76		
227654_at	2.39	2.58	chromosome 20 open reading frame 175	C20orf175
1569768_at	2.39	20.90		
222357_at	2.39	2.05	zinc finger and BTB domain containing 20	ZBTB20
207076_s_at	2.39	3.13	argininosuccinate synthetase 1	ASS1
336_at	2.39	3.74	thromboxane A2 receptor	TBXA2R
1558982_at	2.38	4.68		
241099_at	2.38	2.01	elongation protein 4 homolog (S. cerevisiae)	ELP4
210703_at	2.38	2.13		
244511_at	2.38	2.02	phosphodiesterase 4D interacting protein (myomegalin)	PDE4DIP

206169_x_at	2.37	2.44	zinc finger CCCH-type containing 7B	ZC3H7B
203939_at	2.36	3.52	5'-nucleotidase, ecto (CD73)	NT5E
1553599_a_at	2.36	2.30	synaptonemal complex protein 3	SYCP3
235441_at	2.34	2.10	adenylate cyclase 3	ADCY3
219550_at	2.34	3.24	roundabout, axon guidance receptor, homolog 3 (Drosophila)	ROBO3
214594_x_at	2.31	4.99	ATPase, Class I, type 8B, member 1	ATP8B1
234155_at	2.31	6.63		
213249_at	2.31	3.28	F-box and leucine-rich repeat protein 7	FBXL7
242733_at	2.30	2.28		
240801_at	2.30	2.64	chromosome 21 open reading frame 37	C21orf37
242622_x_at	2.30	2.15	phosphatase and tensin homolog (mutated in multiple advanced cancers 1)	PTEN
205205_at	2.29	2.37	v-rel reticuloendotheliosis viral oncogene homolog B, nuclear factor of kappa light polypeptide gene	RELB
241656_at	2.28	2.52		
211745_x_at	2.27	2.31	hemoglobin, alpha 2	HBA2
239381_at	2.27	27.10	kallikrein-related peptidase 7	KLK7
231247_s_at	2.27	2.37		
1566001_at	2.26	2.56		
1559623_at	2.25	2.45	chromosome 11 open reading frame 54	C11orf54
201392_s_at	2.24	2.03	insulin-like growth factor 2 receptor	IGF2R
214933_at	2.24	5.01	calcium channel, voltage-dependent, P/Q type, alpha 1A subunit	CACNA1A
1559343_at	2.23	2.04	small nuclear ribonucleoprotein polypeptide N	SNRPN
224156_x_at	2.23	4.60	interleukin 17 receptor B	IL17RB
217679_x_at	2.21	2.06		
216769_x_at	2.21	6.15		
239642_at	2.21	3.13		
212319_at	2.21	2.16	RUN and TBC1 domain containing 1	RUTBC1
220728_at	2.20	2.71		
226756_at	2.20	2.34		
241837_at	2.20	2.74	AT rich interactive domain 5B (MRF1-like)	ARID5B
240602_at	2.20	2.20	HBS1-like (S. cerevisiae)	HBS1L
203827_at	2.19	3.30	WD repeat domain, phosphoinositide interacting 1	WIPI1
231050_at	2.18	4.31	HRAS-like suppressor family, member 5	HRASLS5
239336_at	2.18	2.16	thrombospondin 1	THBS1
209802_at	2.18	3.53	pleckstrin homology-like domain, family A, member 2	PHLDA2

214156_at	2.18	2.16	myosin VIIA and Rab interacting protein	MYRIP
205806_at	2.18	8.21	retinal outer segment membrane protein 1	ROM1
225189_s_at	2.17	2.01	Ras association (RalGDS/AF-6) and pleckstrin homology domains 1	RAPH1
244359_s_at	2.16	2.06		
222108_at	2.16	5.83	adhesion molecule with Ig-like domain 2	AMIGO2
244227_at	2.16	2.35	synaptotagmin VI	SYT6
226111_s_at	2.15	3.65	zinc finger protein 385	ZNF385
214403_x_at	2.15	6.24	SAM pointed domain containing ets transcription factor	SPDEF
218353_at	2.15	5.68	regulator of G-protein signalling 5	RGS5
231367_s_at	2.13	2.04		
219255_x_at	2.12	6.04	interleukin 17 receptor B	IL17RB
1556898_at	2.11	2.17		
211699_x_at	2.11	2.43	hemoglobin, alpha 1	HBA1
204018_x_at	2.11	2.17	hemoglobin, alpha 1	HBA1
235062_at	2.10	2.64	PIH1 domain containing 2	PIH1D2
232705_at	2.09	2.55	leucine rich repeat (in FLII) interacting protein 2	LRRFIP2
216563_at	2.09	2.05	ankyrin repeat domain 12	ANKRD12
208849_at	2.09	8.17		
211343_s_at	2.09	3.09	collagen, type XIII, alpha 1	COL13A1
1552829_at	2.09	2.09	transmembrane protein 23	TMEM23
239311_at	2.09	2.16		
1560486_at	2.08	2.97	syntaxin binding protein 3	STXBP3
233411_at	2.07	2.64		
236031_x_at	2.07	2.95	FRAS1 related extracellular matrix 1	FREM1
239725_at	2.06	2.83		
215907_at	2.05	2.07		
212013_at	2.05	2.78	peroxidasin homolog (Drosophila)	PXDN
215387_x_at	2.05	2.89		
1566887_x_at	2.05	3.39		
208154_at	2.04	2.54		
237632_at	2.04	2.53	hect (homologous to the E6-AP (UBE3A) carboxyl terminus) domain and RCC1 (CHC1)-like domain (RLD) 1	HERC1
228698_at	2.04	2.57	SRY (sex determining region Y)-box 7	SOX7
225239_at	2.04	2.40		
232957_x_at	2.04	2.28		
215987_at	2.03	5.62	Rap guanine nucleotide exchange factor (GEF) 2	RAPGEF2
232872_at	2.03	2.86		
241773_at	2.03	4.22		
223781_x_at	2.02	3.90	alcohol dehydrogenase 4 (class II), pi polypeptide	ADH4
1554665_at	2.01	3.22	zinc finger protein 587	ZNF587
220803_at	2.01	3.26	STAM binding protein-like 1	STAMBPL1

238093_at	2.01	2.21		
239342_at	2.00	2.51		
202647_s_at	-2.00	-2.64	neuroblastoma RAS viral (v-ras) oncogene homolog	NRAS
241235_at	-2.01	-8.55		
202437_s_at	-2.01	-2.39	cytochrome P450, family 1, subfamily B, polypeptide 1	CYP1B1
1568672_at	-2.02	-13.98	ELL associated factor 2	EAF2
1558959_at	-2.02	-2.87		
223318_s_at	-2.04	-2.51	alkB, alkylation repair homolog 7 (E. coli)	ALKBH7
221194_s_at	-2.06	-2.12		
202691_at	-2.07	-2.91	small nuclear ribonucleoprotein D1 polypeptide 16kDa	SNRPD1
1555154_a_at	-2.07	-2.59	quaking homolog, KH domain RNA binding (mouse)	QKI
231152_at	-2.07	-10.65		
214513_s_at	-2.09	-2.58	cAMP responsive element binding protein 1	CREB1
222501_s_at	-2.10	-2.27	replication initiator 1	REPIN1
1554101_a_at	-2.10	-2.77	transmembrane and tetratricopeptide repeat containing 4	TMTC4
239682_at	-2.12	-2.46		
1564272_a_at	-2.14	-3.38	kelch domain containing 1	KLHDC1
211061_s_at	-2.15	-2.00	mannosyl (alpha-1,6-)-glycoprotein	MGAT2
230876_at	-2.16	-6.14		
205716_at	-2.36	-2.15	solute carrier family 25, member 40	SLC25A40
229128_s_at	-2.37	-2.61	acidic (leucine-rich) nuclear phosphoprotein 32 family, member E	ANP32E
242426_at	-2.38	-14.85	neuregulin 4	NRG4
200986_at	-2.42	-5.55	serpin peptidase inhibitor, clade G (C1 inhibitor), member 1, (angioedema, hereditary)	SERPING1
1555745_a_at	-2.45	-3.41	lysozyme (renal amyloidosis)	LYZ
1555337_a_at	-2.46	-3.80	zinc finger protein 317	ZNF317
210788_s_at	-2.50	-2.32	dehydrogenase/reductase (SDR family) member 7	DHRS7
1552792_at	-2.55	-2.24	suppressor of cytokine signaling 4	SOCS4
237233_at	-2.63	-2.18		
206710_s_at	-2.64	-2.18	erythrocyte membrane protein band 4.1-like 3	EPB41L3
1567457_at	-2.65	-8.42	ras-related C3 botulinum toxin substrate 1 (rho family, small GTP binding protein Rac1)	RAC1
211080_s_at	-2.67	-2.21	NIMA (never in mitosis gene a)-related kinase 2	NEK2
209683_at	-2.76	-3.48	family with sequence similarity 49, member A	FAM49A
237746_at	-2.77	-9.99	splicing factor, arginine/serine-rich 11	SFRS11
1555731_a_at	-2.79	-4.78	adaptor-related protein complex 1, sigma 3 subunit	AP1S3

214729_at	-2.94	-4.22	TWIST neighbor	TWISTNB
229713_at	-2.95	-2.71		
200796_s_at	-3.07	-9.95	myeloid cell leukemia sequence 1 (BCL2-related)	MCL1
222773_s_at	-3.29	-3.41	UDP-N-acetyl-alpha-D-galactosamine:polypeptide N-acetylgalactosaminyltransferase 12 (GalNAc-T12)	GALNT12
216915_s_at	-3.86	-2.96	protein tyrosine phosphatase, non-receptor type 12	PTPN12
1555340_x_at	-3.94	-8.31	RAP1A, member of RAS oncogene family	RAP1A
225150_s_at	-4.08	-5.31	rhotekin	RTKN
1555339_at	-4.78	-9.21	RAP1A, member of RAS oncogene family	RAP1A
238692_at	-5.60	-2.44	BTB (POZ) domain containing 11	BTBD11
AFFX-TrpnX-5_at	-5.86	-3.69		
209821_at	-18.18	-2.05	interleukin 33	IL33
214732_at	-22.22	-8.08	Sp1 transcription factor	SP1
