# Supplementary material for: In Vitro Transformation of Primary Human CD34+ Cells by AML Fusion Oncogenes: Early Gene Expression Profiling Reveals Possible Drug Target in AML
Source: PLoS One. 2010 Aug 27;5(8):e12464. doi: 10.1371/journal.pone.0012464 (PMC2929205; doi:10.1371/journal.pone.0012464)
Supplement: Table S3 — Genes deregulated by AML1-ETO 8 days after transduction. Primary human CD34+ cells were retrovirally transduced with either control MSCV-IRES-GFP vector or vector expressing AML1-ETO and sorted for GFP positivity. Total RNA was extracted 8 days after transduction and subjected to microarray analysis. Genes that showed up- or down-regulation by 2 fold or more in comparison to the control in 2 independent experiments (Exp.1 and Exp.2) were considered deregulated. (0.08 MB PDF) [file pone.0012464.s003.pdf]

Table S3. Genes deregulated by AML1-ETO at 8 d after transduction

Probe set ID	Fold Change		Gene Name	Gene Symbol
	Exp.1	Exp.2		
221169_s_at	35.17	5.07	histamine receptor H4	HRH4
214156_at	7.21	4.59	myosin VIIA and Rab interacting protein	MYRIP
205848_at	3.41	2.04	growth arrest-specific 2	GAS2
214974_x_at	2.91	2.33	chemokine (C-X-C motif) ligand 5	CXCL5
219255_x_at	2.89	2.26	interleukin 17 receptor B	IL17RB
242642_at	2.84	2.02	nudE nuclear distribution gene E homolog (A. nidulans)-like 1	NDEL1
205609_at	2.77	3.01	angiopoietin 1	ANGPT1
1558397_at	2.76	3.04		
1561139_at	2.69	2.26		
243767_at	2.57	2.07		
236237_at	2.32	2.40	KIAA1370	KIAA1370
242182_x_at	2.14	2.00		
201110_s_at	2.13	5.14	thrombospondin 1	THBS1
239045_at	2.06	2.50		
239861_at	2.06	2.22		
215810_x_at	2.03	2.25		
239693_at	2.01	2.42	sorting nexing 24	SNX24
236882_at	-2.01	-2.39		
233501_at	-2.02	-2.17		
214066_x_at	-2.12	-2.02	natriuretic peptide receptor B/guanylate cyclase B (atrionatriuretic peptide receptor B)	NPR2
224321_at	-2.18	-4.58	transmembrane protein with EGF-like and two follistatin-like domains 2	TMEFF2
241738_at	-2.23	-2.64		
200606_at	-2.28	-2.09	desmoplakin	DSP
232908_at	-2.28	-2.31	ATPase family, AAA domain containing 2B	ATAD2B
1553491_at	-2.29	-2.44	kinase suppressor of ras 2	KSR2
205976_at	-2.31	-3.28	FAST kinase domains 2	FASTKD2
217102_at	-2.33	-2.62		
244451_x_at	-2.35	-3.04	thymine-DNA glycosylase	TDG
204286_s_at	-2.37	-2.35	phorbol-12-myristate-13-acetate-induced protein 1	PMAIP1
230928_at	-2.59	-5.95		
217714_x_at	-2.68	-2.16	stathmin 1/oncoprotein 18	STMN1
244668_at	-3.22	-4.52		
215571_at	-3.46	-8.76		
1568856_at	-3.65	-3.12	neighbor of BRCA1 gene 1	NBR1
207665_at	-3.83	-2.74	ADAM metallopeptidase domain 21	ADAM21
1555339_at	-4.49	-5.17	RAP1A, member of RAS oncogene family	RAP1A
1555340_x_at	-4.61	-6.19	RAP1A, member of RAS oncogene family	RAP1A
1552797_s_at	-4.68	-5.60	prominin 2	PROM2
243573_at	-5.42	-3.05		
