# Supplementary material for: In Vitro Transformation of Primary Human CD34+ Cells by AML Fusion Oncogenes: Early Gene Expression Profiling Reveals Possible Drug Target in AML
Source: PLoS One. 2010 Aug 27;5(8):e12464. doi: 10.1371/journal.pone.0012464 (PMC2929205; doi:10.1371/journal.pone.0012464)
Supplement: Table S4 — Genes deregulated by PML-RARA 6 h after transfection. Primary human CD34+ cells were nucleofected with either control pTracer-CMV/Bsd vector or vector expressing PML-RARA and sorted for GFP positivity. Total RNA was extracted 6 h after nucleofection and subjected to microarray analysis. Genes that showed up- or down-regulation by 2 fold or more in comparison to the control in 2 independent experiments (Exp.1 and Exp.2) were considered deregulated. (0.18 MB PDF) [file pone.0012464.s004.pdf]

Table S4. Genes deregulated by PML-RARA at 6 h after transfection

Probe set ID	Fold Change		Gene Name	Gene Symbol
	Exp.1	Exp.2		
211605_s_at	310.86	183.07	retinoic acid receptor, alpha	RARA
1568673_s_at	146.41	4.59	ELL associated factor 2	EAF2
210439_at	127.54	11.74	inducible T-cell co-stimulator	ICOS
213435_at	37.42	4.00	SATB family member 2	SATB2
1556700_a_at	17.40	25.25		
216300_x_at	16.35	6.62	retinoic acid receptor, alpha	RARA
233011_at	14.88	2.58	annexin A1	ANXA1
1560135_at	11.95	2.32		
201289_at	10.59	3.23	cysteine-rich, angiogenic inducer, 61	CYR61
235976_at	10.31	3.58	SLIT and NTRK-like family, member 6	SLITRK6
236300_at	9.35	2.69		
204555_s_at	8.36	2.63	protein phosphatase 1, regulatory (inhibitor) subunit 3D	PPP1R3D
220030_at	8.29	5.43	serine/threonine/tyrosine kinase 1	STYK1
240161_s_at	8.20	2.80	cell division cycle 20 homolog B (S. cerevisiae)	CDC20B
226534_at	7.42	7.19	KIT ligand	KITLG
214467_at	6.94	3.58	G protein-coupled receptor 65	GPR65
210119_at	6.77	29.27	potassium inwardly-rectifying channel, subfamily J, member 15	KCNJ15
235944_at	6.55	6.48	hemicentin 1	HMCN1
216279_at	6.27	3.13	zinc finger protein 460	ZNF460
206157_at	6.17	4.70	pentraxin-related gene, rapidly induced by IL-1 beta	PTX3
219628_at	6.06	3.39	zinc finger, matrin type 3	ZMAT3
212850_s_at	5.91	2.28	low density lipoprotein receptor-related protein 4	LRP4
214079_at	5.84	2.93	dehydrogenase/reductase (SDR family) member 2	DHRS2
236281_x_at	5.82	2.54	5-hydroxytryptamine (serotonin) receptor 7 (adenylate cyclase-coupled)	HTR7
212611_at	5.65	4.17		
239825_at	5.57	2.42	activating transcription factor 6	ATF6
221696_s_at	5.46	3.59	serine/threonine/tyrosine kinase 1	STYK1
226446_at	5.44	2.52	hairy and enhancer of split 6 (Drosophila)	HES6
222088_s_at	5.25	2.60	solute carrier family 2 (facilitated glucose transporter), member 3	SLC2A3
209189_at	5.09	4.03	v-fos FBJ murine osteosarcoma viral oncogene homolog	FOS
228315_at	5.06	3.20		
202388_at	4.94	3.96	regulator of G-protein signalling 2, 24kDa	RGS2
213418_at	4.76	2.04	heat shock 70kDa protein 6 (HSP70B')	HSPA6

207120_at	4.72	3.08	zinc finger protein 667	ZNF667
202551_s_at	4.64	2.72	cysteine rich transmembrane BMP regulator 1 (chordin-like)	CRIM1
201939_at	4.51	3.28	polo-like kinase 2 (Drosophila)	PLK2
202497_x_at	4.49	3.68	solute carrier family 2 (facilitated glucose transporter), member 3	SLC2A3
232481_s_at	4.46	5.39	SLIT and NTRK-like family, member 6	SLITRK6
211812_s_at	4.45	2.36	beta-1,3-N-acetylgalactosaminyltransferase 1 (globoside blood group)	B3GALNT1
225725_at	4.45	3.94		
241929_at	4.43	3.17		
234303_s_at	4.40	2.34	G protein-coupled receptor 85	GPR85
209906_at	4.18	3.37	complement component 3a receptor 1	C3AR1
226837_at	4.16	3.23	sprouty-related, EVH1 domain containing 1	SPRED1
217503_at	4.14	2.23		
225056_at	4.13	2.45	signal-induced proliferation-associated 1 like 2	SIPA1L2
223131_s_at	4.12	3.16	tripartite motif-containing 8	TRIM8
225806_at	4.11	4.41	jub, ajuba homolog (Xenopus laevis)	JUB
202609_at	4.05	4.36	epidermal growth factor receptor pathway substrate 8	EPS8
241420_at	4.01	13.65		
209598_at	4.00	3.63	paraneoplastic antigen MA2	PNMA2
205386_s_at	3.93	2.79	Mdm2, transformed 3T3 cell double minute 2, p53 binding protein (mouse)	MDM2
1554742_at	3.93	3.43	PMS1 postmeiotic segregation increased 1 (S. cerevisiae)	PMS1
1562621_at	3.89	2.48		
206114_at	3.87	2.43	EPH receptor A4	EPHA4
235144_at	3.85	3.12		
205227_at	3.83	2.18	interleukin 1 receptor accessory protein	IL1RAP
214913_at	3.80	2.73	ADAM metallopeptidase with thrombospondin type 1 motif, 3	ADAMTS3
206239_s_at	3.73	3.11	serine peptidase inhibitor, Kazal type 1	SPINK1
216236_s_at	3.72	3.75	solute carrier family 2 (facilitated glucose transporter), member 3	SLC2A3
212104_s_at	3.63	3.20	RNA binding motif protein 9	RBM9
205514_at	3.56	3.31	zinc finger protein 415	ZNF415
202498_s_at	3.56	3.11	solute carrier family 2 (facilitated glucose transporter), member 3	SLC2A3
205020_s_at	3.52	2.19	ADP-ribosylation factor-like 4A	ARL4A
205249_at	3.51	4.26	early growth response 2 (Krox-20 homolog, Drosophila)	EGR2
239203_at	3.49	2.38		
230708_at	3.49	3.12	prickle homolog 1 (Drosophila)	PRICKLE1

218987_at	3.49	2.53	activating transcription factor 7 interacting protein	ATF7IP
227221_at	3.47	3.65		
202391_at	3.46	3.48	brain abundant, membrane attached signal protein 1	BASP1
244334_at	3.43	3.10	translocation associated membrane protein 1-like 1	TRAM1L1
202499_s_at	3.43	2.74	solute carrier family 2 (facilitated glucose transporter), member 3	SLC2A3
203032_s_at	3.36	4.46	fumarate hydratase	FH
238729_x_at	3.29	3.88		
205214_at	3.29	2.67	serine/threonine kinase 17b (apoptosis-inducing)	STK17B
1553276_at	3.29	42.02	zinc finger protein 560	ZNF560
205239_at	3.28	3.11	amphiregulin (schwannoma-derived growth factor)	AREG
1554997_a_at	3.27	3.26	prostaglandin-endoperoxide synthase 2 (prostaglandin G/H synthase and cyclooxygenase)	PTGS2
212188_at	3.27	2.41	potassium channel tetramerisation domain containing 12	KCTD12
204037_at	3.25	2.41	endothelial differentiation, lysophosphatidic acid G-protein-coupled receptor, 2	EDG2
209433_s_at	3.24	2.55	phosphoribosyl pyrophosphate amidotransferase	PPAT
232278_s_at	3.22	4.59	DEP domain containing 1	DEPDC1
235205_at	3.20	2.02		
236429_at	3.20	2.24		
225065_x_at	3.18	2.07	chromosome 17 open reading frame 45	C17orf45
206463_s_at	3.17	2.11	dehydrogenase/reductase (SDR family) member 2	DHRS2
203439_s_at	3.17	2.12	stanniocalcin 2	STC2
228285_at	3.15	3.00	tudor domain containing 9	TDRD9
229360_at	3.15	2.15	suppressor of hairy wing homolog 2 (Drosophila)	SUHW2
220295_x_at	3.14	4.76	DEP domain containing 1	DEPDC1
1555370_a_at	3.10	2.19	calmodulin binding transcription activator 1	CAMTA1
204827_s_at	3.09	2.55	cyclin F	CCNF
206085_s_at	3.09	2.49	cystathionase (cystathionine gamma-lyase)	CTH
223529_at	3.06	3.01	synaptotagmin IV	SYT4
201579_at	3.04	2.56	FAT tumor suppressor homolog 1 (Drosophila)	FAT
239455_at	3.02	3.38		
214745_at	3.02	2.91	phospholipase C, eta 1	PLCH1
243198_at	3.01	3.43	testis expressed sequence 9	TEX9
232291_at	3.00	2.28	microRNA host gene (non-protein coding) 1	MIRH1

201150_s_at	2.98	2.51	TIMP metallopeptidase inhibitor 3 (Sorsby fundus dystrophy, pseudoinflammatory)	TIMP3
204748_at	2.96	2.51	prostaglandin-endoperoxide synthase 2 (prostaglandin G/H synthase and cyclooxygenase)	PTGS2
237732_at	2.96	3.11		
244640_at	2.88	3.77		
1559950_at	2.87	5.19		
243861_at	2.87	2.14		
202668_at	2.83	3.67	ephrin-B2	EFNB2
1569191_at	2.83	2.15		
238935_at	2.83	2.29	ribosomal protein S27-like	RPS27L
240288_at	2.82	3.75	potassium channel regulator	KCNRG
242197_x_at	2.81	3.50	CD36 molecule (thrombospondin receptor)	CD36
229331_at	2.81	2.37	spermatogenesis associated 18 homolog (rat)	SPATA18
221994_at	2.80	2.11	PDZ and LIM domain 5	PDLIM5
222608_s_at	2.79	2.29	anillin, actin binding protein	ANLN
222162_s_at	2.78	2.33	ADAM metallopeptidase with thrombospondin type 1 motif, 1	ADAMTS1
212233_at	2.76	3.09		
223809_at	2.75	2.45	regulator of G-protein signalling 18	RGS18
1554536_at	2.74	2.48	dihydropyrimidine dehydrogenase	DPYD
243149_at	2.73	2.01		
219377_at	2.73	7.98	family with sequence similarity 59, member A	FAM59A
230542_at	2.72	2.94	zinc finger protein 597	ZNF597
238677_at	2.71	2.17	WD repeat domain 36	WDR36
228523_at	2.71	9.89	nanos homolog 1 (Drosophila)	NANOS1
206648_at	2.70	2.13	zinc finger protein 571	ZNF571
219737_s_at	2.70	2.61	protocadherin 9	PCDH9
210258_at	2.68	3.74	regulator of G-protein signalling 13	RGS13
222958_s_at	2.67	3.49	DEP domain containing 1	DEPDC1
223651_x_at	2.65	2.15	cell division cycle 23 homolog (S. cerevisiae)	CDC23
210942_s_at	2.64	2.40	ST3 beta-galactoside alpha-2,3-sialyltransferase 6	ST3GAL6
218726_at	2.64	2.06		
208670_s_at	2.64	2.61	EP300 interacting inhibitor of differentiation 1	EID1
220369_at	2.63	2.95	SMEK homolog 1, suppressor of mek1 (Dictyostelium)	SMEK1
223195_s_at	2.62	2.18	sestrin 2	SESN2
201333_s_at	2.62	2.26	Rho guanine nucleotide exchange factor (GEF) 12	ARHGEF12
205229_s_at	2.62	2.15	coagulation factor C homolog, cochlin (Limulus polyphemus)	COCH
214146_s_at	2.62	26.92	pro-platelet basic protein (chemokine (C-X-C motif) ligand 7)	PPBP

206411_s_at	2.62	2.17	v-abl Abelson murine leukemia viral oncogene homolog 2 (arg, Abelson-related gene)	ABL2
214318_s_at	2.61	2.55	furry homolog (Drosophila)	FRY
1555120_at	2.59	3.08	CD96 molecule	CD96
228433_at	2.58	2.80		
202988_s_at	2.57	3.53	regulator of G-protein signalling 1	RGS1
222771_s_at	2.57	2.86	myelin expression factor 2	MYEF2
244788_at	2.56	3.40		
233587_s_at	2.56	2.48	signal-induced proliferation-associated 1 like 2	SIPA1L2
201730_s_at	2.56	2.61	translocated promoter region (to activated MET oncogene)	TPR
242116_x_at	2.55	2.71	ankyrin repeat domain 17	ANKRD17
240465_at	2.54	9.32		
204321_at	2.53	2.22	neogenin homolog 1 (chicken)	NEO1
216350_s_at	2.53	2.36	zinc finger protein 10	ZNF10
238756_at	2.53	3.53		
204774_at	2.52	2.45	ecotropic viral integration site 2A	EVI2A
1569366_a_at	2.51	2.47	zinc finger protein 569	ZNF569
1560609_at	2.49	4.66	crystallin, zeta (quinone reductase)-like 1	CRYZL1
204602_at	2.49	2.05	dickkopf homolog 1 (Xenopus laevis)	DKK1
1569107_s_at	2.49	2.09	zinc finger protein 642	ZNF642
1554743_x_at	2.49	3.07	PMS1 postmeiotic segregation increased 1 (S. cerevisiae)	PMS1
235874_at	2.48	4.00	protease, serine, 35	PRSS35
207528_s_at	2.48	2.23	solute carrier family 7, (cationic amino acid transporter, y+ system) member 11	SLC7A11
204038_s_at	2.47	3.21	endothelial differentiation, lysophosphatidic acid G-protein-coupled receptor, 2	EDG2
1554309_at	2.47	2.14	eukaryotic translation initiation factor 4 gamma, 3	EIF4G3
206935_at	2.46	3.73	protocadherin 8	PCDH8
216834_at	2.45	2.92	regulator of G-protein signalling 1	RGS1
235121_at	2.44	2.22	zinc finger protein 542	ZNF542
225476_at	2.44	3.03	HLA-B associated transcript 4	BAT4
1558622_a_at	2.44	2.05	zinc finger protein 548	ZNF548
242427_at	2.43	2.31	WW domain containing adaptor with coiled-coil	WAC
214639_s_at	2.43	2.92	homeobox A1	HOXA1
228033_at	2.43	2.26	E2F transcription factor 7	E2F7
216574_s_at	2.42	2.27		
243242_at	2.42	2.05		
219201_s_at	2.42	2.30	twisted gastrulation homolog 1 (Drosophila)	TWSG1
206314_at	2.42	2.28	zinc finger protein 167	ZNF167
1553220_at	2.41	2.63	amyotrophic lateral sclerosis 2 (juvenile) chromosome region, candidate 13	ALS2CR13

1557029_at	2.40	7.23		
205659_at	2.39	3.49	histone deacetylase 9	HDAC9
228948_at	2.39	2.25	EPH receptor A4	EPHA4
230387_at	2.39	2.06		
1558409_at	2.38	2.19		
235069_at	2.38	5.56	TatD DNase domain containing 3	TATDN3
234970_at	2.38	2.02	membrane targeting (tandem) C2 domain containing 1	MTAC2D1
204517_at	2.38	2.21	peptidylprolyl isomerase C (cyclophilin C)	PPIC
207219_at	2.38	3.09	zinc finger protein 643	ZNF643
215615_x_at	2.37	2.30		
1553120_at	2.36	2.40	claspin homolog (Xenopus laevis)	CLSPN
210233_at	2.34	2.34	interleukin 1 receptor accessory protein	IL1RAP
230205_at	2.34	2.06	zinc finger protein 561	ZNF561
1554101_a_at	2.33	2.21	transmembrane and tetratricopeptide repeat containing 4	TMTC4
227306_at	2.32	2.20		
1554621_at	2.32	2.02	diacylglycerol kinase, epsilon 64kDa	DGKE
204962_s_at	2.32	2.41	centromere protein A	CENPA
243054_at	2.32	3.00	zinc finger, MYND domain containing 11	ZMYND11
227197_at	2.31	4.52		
207505_at	2.31	2.30	protein kinase, cGMP-dependent, type II	PRKG2
211756_at	2.30	2.09	parathyroid hormone-like hormone	PTHLH
228915_at	2.30	2.34	dachshund homolog 1 (Drosophila)	DACH1
206331_at	2.29	2.12	calcitonin receptor-like	CALCRL
244887_at	2.29	2.44	regulator of G-protein signalling 13	RGS13
204529_s_at	2.29	2.19		
238962_at	2.28	2.13		
239343_at	2.28	2.07		
209212_s_at	2.28	3.65	Kruppel-like factor 5 (intestinal)	KLF5
217127_at	2.28	2.13	cystathionase (cystathionine gamma-lyase)	CTH
206835_at	2.27	4.90	statherin	STATH
237491_at	2.26	3.14	myosin, heavy chain 10, non-muscle	MYH10
206907_at	2.26	4.05	tumor necrosis factor (ligand) superfamily, member 9	TNFSF9
1558592_at	2.25	2.36		
215506_s_at	2.25	2.32	DIRAS family, GTP-binding RAS-like 3	DIRAS3
204457_s_at	2.24	3.87	growth arrest-specific 1	GAS1
227801_at	2.24	2.94	tripartite motif-containing 59	TRIM59
1554242_a_at	2.24	2.64	coagulation factor C homolog, cochlin (Limulus polyphemus)	COCH
228824_s_at	2.23	4.12	leukotriene B4 12-hydroxydehydrogenase	LTB4DH
232295_at	2.22	7.42	G elongation factor, mitochondrial 1	GFM1
240141_at	2.22	2.01	PAP associated domain containing 4	PAPD4
207813_s_at	2.22	2.55	ferredoxin reductase	FDXR

203913_s_at	2.22	2.36	hydroxyprostaglandin dehydrogenase 15-(NAD)	HPGD
223005_s_at	2.20	2.05	chromosome 9 open reading frame 5	C9orf5
225481_at	2.19	2.21	FERM domain containing 6	FRMD6
219427_at	2.19	4.64	FAT tumor suppressor homolog 4 (Drosophila)	FAT4
238724_at	2.18	2.07	2,3-bisphosphoglycerate mutase	BPGM
229715_at	2.18	5.00		
227099_s_at	2.18	2.18		
213933_at	2.18	2.63	prostaglandin E receptor 3 (subtype EP3)	PTGER3
213355_at	2.17	2.38	ST3 beta-galactoside alpha-2,3-sialyltransferase 6	ST3GAL6
1558014_s_at	2.17	2.80	male sterility domain containing 2	MLSTD2
223669_at	2.17	2.91	hemogen	HEMGN
1553269_at	2.16	2.52	zinc finger protein 718	ZNF718
239957_at	2.16	2.27	SET domain containing 5	SETD5
220688_s_at	2.16	2.02	mRNA turnover 4 homolog (S. cerevisiae)	MRTO4
200730_s_at	2.15	2.31	protein tyrosine phosphatase type IVA, member 1	PTP4A1
209301_at	2.15	2.43	carbonic anhydrase II	CA2
222385_x_at	2.15	2.26	Sec61 alpha 1 subunit (S. cerevisiae)	SEC61A1
235846_at	2.15	2.64		
205018_s_at	2.15	3.30	muscleblind-like 2 (Drosophila)	MBNL2
207768_at	2.14	2.06	early growth response 4	EGR4
244454_at	2.14	2.18	heterogeneous nuclear ribonucleoprotein H3 (2H9)	HNRPH3
225600_at	2.14	2.25		
1553593_a_at	2.14	21.64	T-cell acute lymphocytic leukemia 2	TAL2
225687_at	2.14	2.31	family with sequence similarity 83, member D	FAM83D
206145_at	2.13	2.91	Rh-associated glycoprotein	RHAG
239494_at	2.12	4.25		
241583_x_at	2.12	5.40	synaptotagmin I	SYT1
215307_at	2.12	2.28	zinc finger protein 529	ZNF529
210757_x_at	2.11	2.22	disabled homolog 2, mitogen-responsive phosphoprotein (Drosophila)	DAB2
204525_at	2.10	2.19	PHD finger protein 14	PHF14
226884_at	2.10	3.80	leucine rich repeat neuronal 1	LRRN1
211734_s_at	2.10	2.08	Fc fragment of IgE, high affinity I, receptor for; alpha polypeptide	FCER1A
1565898_at	2.09	2.27	methyltransferase 5 domain containing 1	METT5D1
239002_at	2.09	2.82	asp (abnormal spindle) homolog, microcephaly associated (Drosophila)	ASPM
219717_at	2.07	2.22	chromosome 4 open reading frame 30	C4orf30
204298_s_at	2.07	4.12	lysyl oxidase	LOX
221078_s_at	2.07	2.02	KIAA1212	KIAA1212
230005_at	2.07	2.42		

238578_at	2.07	2.36	transmembrane protein 182	TMEM182
211742_s_at	2.07	2.61	ecotropic viral integration site 2B	EVI2B
224802_at	2.06	2.26	Nedd4 family interacting protein 2	NDFIP2
236565_s_at	2.05	2.22	La ribonucleoprotein domain family, member 6	LARP6
1553132_a_at	2.04	2.03	membrane targeting (tandem) C2 domain containing 1	MTAC2D1
219148_at	2.04	2.32	PDZ binding kinase	PBK
238623_at	2.04	2.16		
218469_at	2.04	2.47	gremlin 1, cysteine knot superfamily, homolog (Xenopus laevis)	GREM1
206207_at	2.04	2.39	Charcot-Leyden crystal protein	CLC
218458_at	2.03	2.14	germ cell-less homolog 1 (Drosophila)	GMCL1
1556404_a_at	2.03	2.71		
205235_s_at	2.03	2.15	M-phase phosphoprotein 1	MPHOSPH1
230134_s_at	2.02	2.20		
228144_at	2.02	2.24	zinc finger protein 300	ZNF300
204135_at	2.01	2.18	filamin A interacting protein 1-like	FILIP1L
215446_s_at	2.01	2.30	lysyl oxidase	LOX
232063_x_at	2.00	2.21	phenylalanine-tRNA synthetase-like, beta subunit	FARSLB
201873_s_at	2.00	2.02	ATP-binding cassette, sub-family E (OABP), member 1	ABCE1
232454_at	2.00	2.22		
1557701_s_at	2.00	2.18	polymerase (DNA directed), eta	POLH
235276_at	-2.00	-2.29	epithelial stromal interaction 1 (breast)	EPSTI1
225935_at	-2.01	-2.32		
202437_s_at	-2.01	-3.20	cytochrome P450, family 1, subfamily B, polypeptide 1	CYP1B1
1552343_s_at	-2.01	-2.02	phosphodiesterase 7A	PDE7A
218665_at	-2.01	-2.91	frizzled homolog 4 (Drosophila)	FZD4
228891_at	-2.01	-2.00	chromosome 9 open reading frame 164	C9orf164
235626_at	-2.01	-2.29	calcium/calmodulin-dependent protein kinase ID	CAMK1D
224833_at	-2.02	-2.32	v-ets erythroblastosis virus E26 oncogene homolog 1 (avian)	ETS1
202435_s_at	-2.02	-3.35		
243658_at	-2.03	-3.49	farnesyl-diphosphate farnesyltransferase 1	FDFT1
228450_at	-2.04	-2.31	pleckstrin homology domain containing, family A member 7	PLEKHA7
209193_at	-2.04	-2.23	pim-1 oncogene	PIM1
230741_at	-2.04	-2.46		
232068_s_at	-2.04	-6.42	toll-like receptor 4	TLR4
224560_at	-2.04	-2.28	TIMP metallopeptidase inhibitor 2	TIMP2
237086_at	-2.05	-2.80	forkhead box A1	FOXA1
217762_s_at	-2.05	-2.67	RAB31, member RAS oncogene family	RAB31

232382_s_at	-2.05	-2.50	protein-L-isoaspartate (D-aspartate) O-methyltransferase domain containing 1	PCMTD1
213348_at	-2.05	-2.05	cyclin-dependent kinase inhibitor 1C (p57, Kip2)	CDKN1C
226034_at	-2.05	-2.42		
227396_at	-2.05	-4.00		
1562271_x_at	-2.06	-2.33	Rho guanine nucleotide exchange factor (GEF) 7	ARHGEF7
1565974_at	-2.06	-3.10		
211842_s_at	-2.06	-2.25	solute carrier family 24 (sodium/potassium/calcium exchanger), member 1	SLC24A1
1560117_at	-2.06	-2.28	abhydrolase domain containing 1	ABHD1
1556067_a_at	-2.07	-3.04	jumonji domain containing 3	JMJD3
208820_at	-2.07	-2.22	PTK2 protein tyrosine kinase 2	PTK2
222303_at	-2.07	-2.31		
1564338_at	-2.08	-2.12		
211433_x_at	-2.08	-2.22	KIAA1539	KIAA1539
231902_at	-2.08	-2.81		
238653_at	-2.08	-2.48		
239453_at	-2.08	-2.10	formin binding protein 1	FNBP1
226551_at	-2.08	-2.54	receptor (TNFRSF)-interacting serine-threonine kinase 1	RIPK1
229810_at	-2.08	-2.47		
230999_at	-2.09	-2.56		
228718_at	-2.09	-4.28	zinc finger protein 44	ZNF44
227855_at	-2.10	-2.27	zinc finger protein 219	ZNF219
204858_s_at	-2.11	-2.77	endothelial cell growth factor 1 (platelet-derived)	ECGF1
226474_at	-2.11	-2.11	NLR family, CARD domain containing 5	NLRC5
229396_at	-2.11	-2.45	ovo-like 1(Drosophila)	OVOL1
205569_at	-2.11	-2.29	lysosomal-associated membrane protein 3	LAMP3
201005_at	-2.12	-4.24	CD9 molecule	CD9
212355_at	-2.12	-2.40	KIAA0323	KIAA0323
220144_s_at	-2.12	-2.06	ankyrin repeat domain 5	ANKRD5
218284_at	-2.12	-2.12	SMAD family member 3	SMAD3
1557036_at	-2.12	-2.09	zinc finger and BTB domain containing 1	ZBTB1
228046_at	-2.13	-3.77		
242794_at	-2.13	-2.37	mastermind-like 3 (Drosophila)	MAML3
202856_s_at	-2.14	-2.51	solute carrier family 16, member 3 (monocarboxylic acid transporter 4)	SLC16A3
209941_at	-2.14	-2.81	receptor (TNFRSF)-interacting serine-threonine kinase 1	RIPK1
215462_at	-2.14	-3.22		
213725_x_at	-2.14	-2.08	xylosyltransferase I	XYLT1
212120_at	-2.15	-2.63	ras homolog gene family, member Q	RHOQ
242109_at	-2.15	-2.61	synaptotagmin-like 3	SYTL3
221653_x_at	-2.15	-2.50	apolipoprotein L, 2	APOL2

203713_s_at	-2.16	-2.52	lethal giant larvae homolog 2 (Drosophila)	LLGL2
224832_at	-2.16	-2.97	dual specificity phosphatase 16	DUSP16
225789_at	-2.16	-3.32	centaurin, gamma 3	CENTG3
229504_at	-2.16	-2.40		
239058_at	-2.16	-2.60		
243173_at	-2.16	-6.25	calcium binding protein 7	CABP7
226681_at	-2.17	-4.60	ubiquitin-conjugating enzyme E2H (UBC8 homolog, yeast)	UBE2H
229250_at	-2.17	-2.07	two pore segment channel 2	TPCN2
227697_at	-2.18	-2.10	suppressor of cytokine signaling 3	SOCS3
211986_at	-2.18	-4.06	AHNAK nucleoprotein (desmoyokin)	AHNAK
202172_at	-2.19	-2.58	vascular endothelial zinc finger 1	VEZF1
240166_x_at	-2.19	-2.33	RNA (guanine-9-) methyltransferase domain containing 3	RG9MTD3
214888_at	-2.19	-2.96	calpain 2, (m/II) large subunit	CAPN2
227410_at	-2.19	-2.68	family with sequence similarity 43, member A	FAM43A
209829_at	-2.19	-2.02	chromosome 6 open reading frame 32	C6orf32
226101_at	-2.20	-2.82	protein kinase C, epsilon	PRKCE
240482_at	-2.21	-2.24	histone deacetylase 3	HDAC3
201126_s_at	-2.21	-2.11	mannosyl (alpha-1,3-)-glycoprotein beta-1,2-N-acetylglucosaminyltransferase	MGAT1
213038_at	-2.22	-2.23	IBR domain containing 3	IBRDC3
236231_at	-2.23	-3.95		
202357_s_at	-2.23	-2.43	complement factor B	CFB
209341_s_at	-2.23	-2.40	inhibitor of kappa light polypeptide gene enhancer in B-cells, kinase beta	IKBKB
225663_at	-2.24	-2.04	acyl-Coenzyme A binding domain containing 5	ACBD5
220174_at	-2.24	-2.71	leucine rich repeat containing 8 family, member E	LRRC8E
1568623_a_at	-2.24	-3.51	solute carrier family 35, member E4	SLC35E4
219383_at	-2.24	-2.74		
228610_at	-2.24	-2.47	transmembrane 9 superfamily member 3	TM9SF3
207629_s_at	-2.25	-2.11	rho/rac guanine nucleotide exchange factor (GEF) 2	ARHGEF2
241178_at	-2.25	-2.95	EGF-containing fibulin-like extracellular matrix protein 1	EFEMP1
213976_at	-2.25	-2.07	CDKN1A interacting zinc finger protein 1	CIZ1
226075_at	-2.26	-2.34	splA/ryanodine receptor domain and SOCS box containing 1	SPSB1
210123_s_at	-2.26	-2.53	cholinergic receptor, nicotinic, alpha 7	CHRNA7
203879_at	-2.27	-2.95	phosphoinositide-3-kinase, catalytic, delta polypeptide	PIK3CD
1554702_at	-2.27	-2.05	voltage gated channel like 1	VGCNL1
226715_at	-2.27	-3.20	forkhead box K1	FOXK1

238797_at	-2.27	-2.16	tripartite motif-containing 11	TRIM11
218764_at	-2.27	-2.09	protein kinase C, eta	PRKCH
213839_at	-2.28	-3.25		
203037_s_at	-2.28	-2.37	metastasis suppressor 1	MTSS1
236161_at	-2.29	-2.34		
206672_at	-2.29	-2.31	aquaporin 2 (collecting duct)	AQP2
234624_at	-2.30	-2.25		
227038_at	-2.30	-2.38		
239448_at	-2.30	-2.32		
240363_at	-2.30	-2.41	ankyrin 1, erythrocytic	ANK1
218093_s_at	-2.30	-2.41	ankyrin repeat domain 10	ANKRD10
226440_at	-2.30	-2.89	dual specificity phosphatase 22	DUSP22
1566764_at	-2.31	-3.61		
55081_at	-2.31	-2.34	MICAL-like 1	MICALL1
233606_at	-2.31	-2.10		
226576_at	-2.31	-2.75	Rho GTPase activating protein 26	ARHGAP26
227341_at	-2.31	-2.26	chromosome 10 open reading frame 30	C10orf30
210162_s_at	-2.31	-2.85	nuclear factor of activated T-cells, cytoplasmic, calcineurin-dependent 1	NFATC1
226040_at	-2.32	-3.14		
227346_at	-2.32	-2.17	IKAROS family zinc finger 1 (Ikaros)	IKZF1
201373_at	-2.32	-4.36	plectin 1, intermediate filament binding protein 500kDa	PLEC1
208933_s_at	-2.32	-2.10		
226219_at	-2.32	-2.49	Rho GTPase activating protein 30	ARHGAP30
214724_at	-2.32	-3.31	DIX domain containing 1	DIXDC1
228008_at	-2.32	-2.85		
229841_at	-2.33	-3.46	eukaryotic translation initiation factor 2C, 2	EIF2C2
237516_at	-2.33	-2.15		
1555781_at	-2.33	-2.24	PQ loop repeat containing 2	PQLC2
227049_at	-2.34	-2.22		
228528_at	-2.34	-3.09		
210853_at	-2.34	-2.33	sodium channel, voltage-gated, type XI, alpha subunit	SCN11A
227193_at	-2.34	-2.72		
204436_at	-2.34	-2.32	pleckstrin homology domain containing, family Q member 1	PLEKHQ1
209508_x_at	-2.35	-3.60	CASP8 and FADD-like apoptosis regulator	CFLAR
227539_at	-2.35	-2.86		
207499_x_at	-2.36	-2.38	unc-45 homolog A (C. elegans)	UNC45A
224780_at	-2.37	-2.34	RNA binding motif protein 17	RBM17
231769_at	-2.37	-2.30	F-box protein 6	FBXO6
229253_at	-2.37	-2.06	thioesterase superfamily member 4	THEM4
242739_at	-2.37	-3.13	chromosome 6 open reading frame 201	C6orf201
222061_at	-2.37	-2.05	CD58 molecule	CD58
209163_at	-2.38	-2.42	cytochrome b-561	CYB561
210357_s_at	-2.38	-2.94	spermine oxidase	SMOX
206618_at	-2.38	-2.46	interleukin 18 receptor 1	IL18R1

200704_at	-2.38	-2.22	lipopolysaccharide-induced TNF factor	LITAF
243626_at	-2.39	-2.79		
207133_x_at	-2.39	-2.23	alpha-kinase 1	ALPK1
228812_at	-2.39	-2.12		
1556429_a_at	-2.39	-2.51	WD repeat domain 67	WDR67
213191_at	-2.39	-3.16	toll-like receptor adaptor molecule 1	TICAM1
230728_at	-2.40	-2.29		
213182_x_at	-2.40	-2.21	cyclin-dependent kinase inhibitor 1C (p57, Kip2)	CDKN1C
223782_s_at	-2.40	-19.48	tubulointerstitial nephritis antigen	TINAG
202531_at	-2.40	-2.49	interferon regulatory factor 1	IRF1
214808_at	-2.40	-2.75		
217851_s_at	-2.40	-2.16	slowmo homolog 2 (Drosophila)	SLMO2
1557145_at	-2.41	-2.65	serine/threonine kinase 38	STK38
234177_at	-2.42	-3.43		
235670_at	-2.42	-2.24		
228869_at	-2.42	-2.66		
241353_s_at	-2.42	-3.37		
1555705_a_at	-2.42	-2.21	CKLF-like MARVEL transmembrane domain containing 3	CMTM3
224912_at	-2.43	-2.05	tetratricopeptide repeat domain 7A	TTC7A
237114_at	-2.43	-2.94	trafficking protein particle complex 3	TRAPPC3
210136_at	-2.43	-2.93	myelin basic protein	MBP
234332_at	-2.43	-2.14	negative regulator of ubiquitin-like proteins 1	NUB1
238853_at	-2.44	-2.30		
226991_at	-2.45	-4.45	nuclear factor of activated T-cells, cytoplasmic, calcineurin-dependent 2	NFATC2
216462_at	-2.47	-3.86		
225390_s_at	-2.47	-2.98	Kruppel-like factor 13	KLF13
201642_at	-2.47	-2.78	interferon gamma receptor 2 (interferon gamma transducer 1)	IFNGR2
212717_at	-2.47	-2.35	pleckstrin homology domain containing, family M (with RUN domain) member 1	PLEKHM1
220494_s_at	-2.48	-2.29		
204364_s_at	-2.48	-2.28	receptor accessory protein 1	REEP1
233622_x_at	-2.49	-3.98		
228728_at	-2.49	-2.07		
209710_at	-2.49	-2.48	GATA binding protein 2	GATA2
1564276_at	-2.49	-2.12		
224566_at	-2.49	-3.05		
217730_at	-2.49	-3.58	transmembrane BAX inhibitor motif containing 1	TMBIM1
209164_s_at	-2.49	-2.73	cytochrome b-561	CYB561
222139_at	-2.49	-3.02		
230499_at	-2.50	-4.66		
225368_at	-2.50	-2.42	homeodomain interacting protein kinase 2	HIPK2
226783_at	-2.50	-2.48	heterogeneous nuclear ribonucleoprotein A/B	HNRPAB
212895_s_at	-2.51	-2.50	active BCR-related gene	ABR

232081_at	-2.51	-3.27		
225116_at	-2.51	-2.13	homeodomain interacting protein kinase 2	HIPK2
226959_at	-2.52	-2.67		
220246_at	-2.52	-4.17	calcium/calmodulin-dependent protein kinase ID	CAMK1D
220727_at	-2.52	-2.13	potassium channel, subfamily K, member 10	KCNK10
235688_s_at	-2.52	-2.77	TNF receptor-associated factor 4	TRAF4
204613_at	-2.53	-3.14	phospholipase C, gamma 2 (phosphatidylinositol-specific)	PLCG2
224029_x_at	-2.54	-2.47	sodium channel, voltage-gated, type XI, alpha subunit	SCN11A
235252_at	-2.55	-2.35	kinase suppressor of ras 1	KSR1
207907_at	-2.55	-3.32	tumor necrosis factor (ligand) superfamily, member 14	TNFSF14
219364_at	-2.55	-3.01		
202308_at	-2.56	-2.24	sterol regulatory element binding transcription factor 1	SREBF1
238642_at	-2.56	-2.70	ankyrin repeat domain 13 family, member D	ANKRD13D
1552582_at	-2.56	-2.71	ATP-binding cassette, sub-family C (CFTR/MRP), member 13	ABCC13
223228_at	-2.56	-2.40	leucine zipper, down-regulated in cancer 1-like	LDOC1L
228909_at	-2.56	-3.31	chromosome 21 open reading frame 86	C21orf86
236982_at	-2.57	-2.15		
215555_at	-2.57	-8.70		
242324_x_at	-2.57	-2.83	collagen and calcium binding EGF domains 1	CCBE1
225602_at	-2.58	-2.22	chromosome 9 open reading frame 19	C9orf19
202417_at	-2.58	-2.97	kelch-like ECH-associated protein 1	KEAP1
243748_at	-2.59	-2.80	eukaryotic translation initiation factor 4E family member 3	EIF4E3
238767_at	-2.59	-2.08		
1557260_a_at	-2.59	-3.29	zinc finger protein 382	ZNF382
202082_s_at	-2.60	-2.90	SEC14-like 1 (S. cerevisiae)	SEC14L1
202111_at	-2.60	-2.56	solute carrier family 4, anion exchanger, member 2 (erythrocyte membrane protein band 3-like 1)	SLC4A2
211267_at	-2.60	-2.49	homeobox, ES cell expressed 1	HESX1
219911_s_at	-2.60	-2.73	solute carrier organic anion transporter family, member 4A1	SLCO4A1
237244_at	-2.61	-31.57		
202794_at	-2.61	-3.48	inositol polyphosphate-1-phosphatase	INPP1
1559763_at	-2.61	-2.31	zinc finger CCCH-type containing 12C	ZC3H12C
218902_at	-2.61	-3.10	Notch homolog 1, translocation-associated (Drosophila)	NOTCH1

204653_at	-2.62	-2.44	transcription factor AP-2 alpha (activating enhancer binding protein 2 alpha)	TFAP2A
211302_s_at	-2.62	-2.04	phosphodiesterase 4B, cAMP-specific (phosphodiesterase E4 dunce homolog, Drosophila)	PDE4B
218921_at	-2.62	-2.20	single immunoglobulin and toll-interleukin 1 receptor (TIR) domain	SIGIRR
227233_at	-2.63	-2.99	tetraspanin 2	TSPAN2
235657_at	-2.63	-3.26		
207986_x_at	-2.63	-5.32	cytochrome b-561	CYB561
238082_at	-2.63	-7.76		
202083_s_at	-2.63	-2.36	SEC14-like 1 (S. cerevisiae)	SEC14L1
203708_at	-2.64	-2.66	phosphodiesterase 4B, cAMP-specific (phosphodiesterase E4 dunce homolog, Drosophila)	PDE4B
212543_at	-2.64	-2.92	absent in melanoma 1	AIM1
203556_at	-2.65	-2.49	zinc fingers and homeoboxes 2	ZHX2
215068_s_at	-2.66	-2.37	F-box and leucine-rich repeat protein 18	FBXL18
57715_at	-2.66	-2.56	family with sequence similarity 26, member B	FAM26B
212274_at	-2.66	-3.93	lipin 1	LPIN1
239797_at	-2.67	-2.81		
227458_at	-2.67	-2.03		
1557553_at	-2.68	-3.88	protein phosphatase 1, regulatory (inhibitor) subunit 12B	PPP1R12B
209409_at	-2.68	-2.15	growth factor receptor-bound protein 10	GRB10
236191_at	-2.68	-2.15	CD38 molecule	CD38
1558938_at	-2.68	-2.58		
201471_s_at	-2.68	-2.39	sequestosome 1	SQSTM1
201460_at	-2.68	-2.15	mitogen-activated protein kinase-activated protein kinase 2	MAPKAPK2
233748_x_at	-2.69	-2.31	protein kinase, AMP-activated, gamma 2 non-catalytic subunit	PRKAG2
233630_at	-2.69	-4.04	CDP-diacylglycerol synthase (phosphatidate cytidylyltransferase) 2	CDS2
243972_at	-2.69	-2.20		
1562550_at	-2.69	-2.28		
227792_at	-2.70	-2.96		
225632_s_at	-2.71	-2.27	RAB43, member RAS oncogene family	RAB43
204088_at	-2.71	-2.94	purinergic receptor P2X, ligand-gated ion channel, 4	P2RX4
243846_x_at	-2.71	-2.48		
218844_at	-2.71	-2.20		
202191_s_at	-2.71	-2.52	growth arrest-specific 7	GAS7
208485_x_at	-2.72	-3.07	CASP8 and FADD-like apoptosis regulator	CFLAR
225133_at	-2.72	-2.18		
235860_at	-2.72	-2.38		

221551_x_at	-2.72	-3.12	ST6 (alpha-N-acetyl-neuraminyl-2,3-beta-galactosyl-1,3)-N-acetylgalactosaminide alpha-2,6-sialyltran	ST6GALNAC4
1570054_at	-2.73	-2.06		
227367_at	-2.73	-3.13		
214255_at	-2.73	-2.06	ATPase, Class V, type 10A	ATP10A
216509_x_at	-2.74	-3.15	myeloid/lymphoid or mixed-lineage leukemia (trithorax homolog, Drosophila); translocated to, 10	MLLT10
224925_at	-2.74	-11.15		
227329_at	-2.74	-2.45	zinc finger and BTB domain containing 46	ZBTB46
1553043_a_at	-2.75	-2.69	CD300 molecule-like family member f	CD300LF
40420_at	-2.76	-2.58	serine/threonine kinase 10	STK10
217200_x_at	-2.76	-3.02	cytochrome b-561	CYB561
238505_at	-2.76	-2.68	ADP-ribosylarginine hydrolase	ADPRH
203518_at	-2.77	-2.09	lysosomal trafficking regulator	LYST
210609_s_at	-2.77	-2.50	tumor protein p53 inducible protein 3	TP53I3
236295_s_at	-2.77	-2.18	NLR family, CARD domain containing 3	NLRC3
227087_at	-2.77	-2.24		
221012_s_at	-2.78	-2.78	tripartite motif-containing 8	TRIM8
202887_s_at	-2.78	-5.59	DNA-damage-inducible transcript 4	DDIT4
205965_at	-2.78	-2.09	basic leucine zipper transcription factor, ATF-like	BATF
243894_at	-2.78	-3.12	solute carrier family 41, member 2	SLC41A2
227999_at	-2.79	-3.61	PWWP domain containing 2	PWWP2
1554176_a_at	-2.79	-2.74	chromosome 3 open reading frame 33	C3orf33
225305_at	-2.79	-2.55	solute carrier family 25, member 29	SLC25A29
229687_s_at	-2.79	-2.30		
229691_at	-2.79	-4.70		
201566_x_at	-2.80	-2.28	inhibitor of DNA binding 2, dominant negative helix-loop-helix protein	ID2
214780_s_at	-2.80	-2.14	myosin IXB	MYO9B
218266_s_at	-2.80	-2.75	frequenin homolog (Drosophila)	FREQ
215369_at	-2.80	-2.59		
215521_at	-2.81	-2.51	polyhomeotic homolog 3 (Drosophila)	PHC3
208683_at	-2.81	-3.07	calpain 2, (m/II) large subunit	CAPN2
237753_at	-2.81	-2.31		
224533_s_at	-2.82	-3.89	interferon, alpha-inducible protein 6	IFI6
232303_at	-2.82	-2.53	zinc finger protein 608	ZNF608
212356_at	-2.83	-3.38	KIAA0323	KIAA0323
224940_s_at	-2.83	-3.03	pregnancy-associated plasma protein A, pappalysin 1	PAPPA
221223_x_at	-2.84	-2.22	cytokine inducible SH2-containing protein	CISH

225641_at	-2.84	-3.30	MADS box transcription enhancer factor 2, polypeptide D (myocyte enhancer factor 2D)	MEF2D
228605_at	-2.84	-2.38		
210873_x_at	-2.84	-2.43	apolipoprotein B mRNA editing enzyme, catalytic polypeptide-like 3A	APOBEC3A
210564_x_at	-2.85	-5.29	CASP8 and FADD-like apoptosis regulator	CFLAR
203839_s_at	-2.86	-2.24	tyrosine kinase, non-receptor, 2	TNK2
216109_at	-2.86	-2.28	thyroid hormone receptor associated protein 2	THRAP2
207643_s_at	-2.87	-3.17	tumor necrosis factor receptor superfamily, member 1A	TNFRSF1A
230369_at	-2.88	-2.95	G protein-coupled receptor 161	GPR161
222062_at	-2.88	-2.16	interleukin 27 receptor, alpha	IL27RA
218927_s_at	-2.88	-2.10	carbohydrate (chondroitin 4) sulfotransferase 12	CHST12
203148_s_at	-2.89	-2.61	tripartite motif-containing 14	TRIM14
242060_x_at	-2.89	-2.14	PHD finger protein 11	PHF11
235221_at	-2.89	-3.26	cerebellin 3 precursor	CBLN3
207859_s_at	-2.90	-10.17	cholinergic receptor, nicotinic, beta 3	CHRNB3
1561879_at	-2.90	-6.48		
218330_s_at	-2.91	-2.32	neuron navigator 2	NAV2
243604_at	-2.92	-2.80		
226143_at	-2.94	-2.71	retinoic acid induced 1	RAI1
222343_at	-2.95	-2.23	BCL2-like 11 (apoptosis facilitator)	BCL2L11
228167_at	-2.95	-2.69		
205013_s_at	-2.95	-8.64	adenosine A2a receptor	ADORA2A
239272_at	-2.96	-3.86	matrix metallopeptidase 28	MMP28
225764_at	-2.96	-2.13	ets variant gene 6 (TEL oncogene)	ETV6
210845_s_at	-2.96	-2.58	plasminogen activator, urokinase receptor	PLAUR
1559747_at	-2.97	-2.29	KIAA1840	KIAA1840
228762_at	-2.97	-7.21	LFNG O-fucosylpeptide 3-beta-N-acetylglucosaminyltransferase	LFNG
220035_at	-2.97	-2.93	nucleoporin 210kDa	NUP210
225262_at	-2.98	-5.43	FOS-like antigen 2	FOSL2
202192_s_at	-2.98	-2.25	growth arrest-specific 7	GAS7
232708_at	-2.99	-2.96	galactose-1-phosphate uridylyltransferase	GALT
208890_s_at	-2.99	-2.66	plexin B2	PLXNB2
227811_at	-2.99	-2.57	FYVE, RhoGEF and PH domain containing 3	FGD3
1557049_at	-2.99	-2.25		
223132_s_at	-3.00	-2.28	tripartite motif-containing 8	TRIM8
1552609_s_at	-3.00	-2.34	interleukin 28B (interferon, lambda 3)	IL28B
239629_at	-3.00	-2.89	CASP8 and FADD-like apoptosis regulator	CFLAR
228188_at	-3.00	-6.37	FOS-like antigen 2	FOSL2
211113_s_at	-3.00	-2.04	ATP-binding cassette, sub-family G (WHITE), member 1	ABCG1

224733_at	-3.00	-2.37	CKLF-like MARVEL transmembrane domain containing 3	CMTM3
202510_s_at	-3.01	-2.17	tumor necrosis factor, alpha-induced protein 2	TNFAIP2
223443_s_at	-3.01	-2.09		
236285_at	-3.01	-2.31		
221530_s_at	-3.01	-2.71	basic helix-loop-helix domain containing, class B, 3	BHLHB3
225763_at	-3.01	-2.00	RCSD domain containing 1	RCSD1
223437_at	-3.02	-3.19	peroxisome proliferator-activated receptor alpha	PPARA
224261_at	-3.03	-2.34		
202628_s_at	-3.03	-4.57	serpin peptidase inhibitor, clade E (nexin, plasminogen activator inhibitor type 1), member 1	SERPINE1
226663_at	-3.03	-2.40		
230810_at	-3.03	-2.29	jumonji domain containing 4	JMJD4
202627_s_at	-3.03	-3.26	serpin peptidase inhibitor, clade E (nexin, plasminogen activator inhibitor type 1), member 1	SERPINE1
224570_s_at	-3.04	-3.07		
219563_at	-3.04	-2.85	chromosome 14 open reading frame 139	C14orf139
215183_at	-3.04	-4.61		
217995_at	-3.05	-2.49	sulfide quinone reductase-like (yeast)	SQRDL
218880_at	-3.06	-2.98	FOS-like antigen 2	FOSL2
37005_at	-3.07	-2.76	neuroblastoma, suppression of tumorigenicity 1	NBL1
225909_at	-3.07	-3.17	zinc finger protein 775	ZNF775
225569_at	-3.07	-2.39	eukaryotic translation initiation factor 2C, 2	EIF2C2
1564672_at	-3.08	-2.06		
207996_s_at	-3.08	-3.04	chromosome 18 open reading frame 1	C18orf1
230917_at	-3.09	-2.54		
228442_at	-3.11	-3.31		
243915_at	-3.12	-2.02		
204683_at	-3.12	-2.64	intercellular adhesion molecule 2	ICAM2
219229_at	-3.13	-2.36	solute carrier organic anion transporter family, member 3A1	SLCO3A1
205841_at	-3.13	-3.23	Janus kinase 2 (a protein tyrosine kinase)	JAK2
1569346_a_at	-3.13	-2.34		
226062_x_at	-3.14	-2.07	family with sequence similarity 63, member A	FAM63A
217591_at	-3.14	-2.26		
223377_x_at	-3.15	-2.50	cytokine inducible SH2-containing protein	CISH
203370_s_at	-3.15	-2.80	PDZ and LIM domain 7 (enigma)	PDLIM7
228770_at	-3.16	-2.61	G protein-coupled receptor 146	GPR146

207426_s_at	-3.18	-3.23	tumor necrosis factor (ligand) superfamily, member 4 (tax-transcriptionally activated glycoprotein 1	TNFSF4
1556332_at	-3.18	-15.62		
212099_at	-3.20	-2.96	ras homolog gene family, member B	RHOB
235938_at	-3.20	-3.93		
242740_at	-3.21	-2.03		
221755_at	-3.21	-2.31	EH domain binding protein 1-like 1	EHBP1L1
230875_s_at	-3.21	-2.63	ATPase, Class VI, type 11A	ATP11A
232077_s_at	-3.22	-2.34		
222877_at	-3.23	-2.06		
242358_at	-3.23	-2.31		
230925_at	-3.24	-2.15	amyloid beta (A4) precursor protein-binding, family B, member 1 interacting protein	APBB1IP
226679_at	-3.24	-4.08	solute carrier family 26, member 11	SLC26A11
243356_at	-3.25	-2.67	family with sequence similarity 7, member A1	FAM7A1
204401_at	-3.27	-2.87	potassium intermediate/small conductance calcium-activated channel, subfamily N, member 4	KCNN4
1405_i_at	-3.28	-2.81	chemokine (C-C motif) ligand 5	CCL5
222154_s_at	-3.29	-2.08		
202071_at	-3.30	-4.08	syndecan 4 (amphiglycan, ryudocan)	SDC4
212276_at	-3.30	-3.30	lipin 1	LPIN1
230383_x_at	-3.30	-2.57		
211862_x_at	-3.31	-2.95	CASP8 and FADD-like apoptosis regulator	CFLAR
206600_s_at	-3.31	-2.71	solute carrier family 16, member 5 (monocarboxylic acid transporter 6)	SLC16A5
228226_s_at	-3.31	-4.69	zinc finger protein 775	ZNF775
221865_at	-3.32	-3.25	chromosome 9 open reading frame 91	C9orf91
226554_at	-3.32	-3.23	zinc finger and BTB domain containing 7A	ZBTB7A
243601_at	-3.33	-2.94		
238086_at	-3.33	-3.39		
208343_s_at	-3.33	-12.83	nuclear receptor subfamily 5, group A, member 2	NR5A2
225227_at	-3.34	-2.53		
227232_at	-3.34	-2.75	Enah/Vasp-like	EVL
219648_at	-3.34	-2.81	melanoregulin	MREG
200706_s_at	-3.34	-2.00	lipopolysaccharide-induced TNF factor	LITAF
212660_at	-3.36	-4.33	PHD finger protein 15	PHF15
217497_at	-3.36	-3.64	endothelial cell growth factor 1 (platelet-derived)	ECGF1
204236_at	-3.37	-2.20	Friend leukemia virus integration 1	FLI1
209939_x_at	-3.37	-3.18	CASP8 and FADD-like apoptosis regulator	CFLAR
203773_x_at	-3.38	-2.87	biliverdin reductase A	BLVRA
219680_at	-3.38	-2.10	NLR family member X1	NLRX1

218845_at	-3.38	-3.29	dual specificity phosphatase 22	DUSP22
214486_x_at	-3.39	-2.31	CASP8 and FADD-like apoptosis regulator	CFLAR
235352_at	-3.39	-2.74		
211105_s_at	-3.40	-3.16	nuclear factor of activated T-cells, cytoplasmic, calcineurin-dependent 1	NFATC1
217677_at	-3.42	-3.85	pleckstrin homology domain containing, family A (phosphoinositide binding specific) member 2	PLEKHA2
240526_at	-3.44	-2.82	ATPase, Class VI, type 11A	ATP11A
244689_at	-3.44	-4.93	peroxisome proliferator-activated receptor alpha	PPARA
223182_s_at	-3.45	-2.29	1-acylglycerol-3-phosphate O-acyltransferase 3	AGPAT3
244018_at	-3.45	-2.36		
229908_s_at	-3.46	-3.28		
232951_at	-3.47	-2.38		
209546_s_at	-3.47	-2.21	apolipoprotein L, 1	APOL1
1555759_a_at	-3.48	-2.04	chemokine (C-C motif) ligand 5	CCL5
219041_s_at	-3.48	-3.00	replication initiator 1	REPIN1
213212_x_at	-3.48	-3.41		
238678_at	-3.51	-2.27		
218812_s_at	-3.52	-4.87	transmembrane protein 142B	TMEM142B
220897_at	-3.52	-4.48		
203234_at	-3.53	-4.81	uridine phosphorylase 1	UPP1
204655_at	-3.54	-2.71	chemokine (C-C motif) ligand 5	CCL5
225407_at	-3.54	-3.31	myelin basic protein	MBP
212817_at	-3.56	-4.30	DnaJ (Hsp40) homolog, subfamily B, member 5	DNAJB5
232858_at	-3.56	-2.16		
240824_at	-3.57	-3.05	oligonucleotide/oligosaccharide-binding fold containing 1	OBFC1
203741_s_at	-3.57	-3.70	adenylate cyclase 7	ADCY7
1559584_a_at	-3.57	-2.53	chromosome 16 open reading frame 54	C16orf54
219278_at	-3.58	-3.28	mitogen-activated protein kinase kinase kinase 6	MAP3K6
224571_at	-3.58	-4.00	interferon regulatory factor 2 binding protein 2	IRF2BP2
241310_at	-3.58	-2.27		
225347_at	-3.58	-8.52	ADP-ribosylation factor-like 8A	ARL8A
203047_at	-3.58	-3.21	serine/threonine kinase 10	STK10
215078_at	-3.58	-3.41	superoxide dismutase 2, mitochondrial	SOD2
239364_at	-3.60	-3.06	ets variant gene 6 (TEL oncogene)	ETV6
235306_at	-3.62	-2.87	GTPase, IMAP family member 8	GIMAP8
224923_at	-3.62	-2.15	tetratricopeptide repeat domain 7A	TTC7A
205192_at	-3.64	-2.40	mitogen-activated protein kinase kinase kinase 14	MAP3K14
231963_at	-3.65	-3.43		
212119_at	-3.66	-2.50	ras homolog gene family, member Q	RHOQ
239474_at	-3.66	-2.29		

228675_at	-3.67	-2.40		
237772_at	-3.67	-2.50		
226530_at	-3.68	-2.79	Bcl2 modifying factor	BMF
204446_s_at	-3.69	-4.51	arachidonate 5-lipoxygenase	ALOX5
226055_at	-3.69	-3.60	arrestin domain containing 2	ARRDC2
225618_at	-3.69	-2.10	Rho GTPase activating protein 27	ARHGAP27
220377_at	-3.70	-3.28	family with sequence similarity 30, member A	FAM30A
210044_s_at	-3.74	-2.50	lymphoblastic leukemia derived sequence 1	LYL1
1560745_at	-3.74	-2.95		
235299_at	-3.74	-2.95		
227645_at	-3.75	-2.68	phosphoinositide-3-kinase, regulatory subunit 5, p101	PIK3R5
232682_at	-3.76	-2.33	peroxisomal trans-2-enoyl-CoA reductase	PECR
221234_s_at	-3.77	-3.42	BTB and CNC homology 1, basic leucine zipper transcription factor 2	BACH2
210563_x_at	-3.77	-2.84	CASP8 and FADD-like apoptosis regulator	CFLAR
223798_at	-3.78	-3.51	solute carrier family 41, member 2	SLC41A2
202910_s_at	-3.79	-4.72	CD97 molecule	CD97
231779_at	-3.80	-5.20	interleukin-1 receptor-associated kinase 2	IRAK2
240730_at	-3.80	-2.56		
232279_at	-3.81	-4.55	PHD finger protein 15	PHF15
219727_at	-3.82	-2.30	dual oxidase 2	DUOX2
214696_at	-3.83	-2.88		
219257_s_at	-3.85	-2.36	sphingosine kinase 1	SPHK1
205027_s_at	-3.88	-2.06	mitogen-activated protein kinase kinase kinase 8	MAP3K8
213367_at	-3.89	-2.68		
226764_at	-3.89	-2.11		
242790_at	-3.89	-2.03		
240949_x_at	-3.89	-2.79		
219647_at	-3.90	-11.62	popeye domain containing 2	POPDC2
219441_s_at	-3.90	-2.72	leucine-rich repeat kinase 1	LRRK1
233961_at	-3.90	-2.59		
227984_at	-3.90	-3.29		
215602_at	-3.92	-2.09	FYVE, RhoGEF and PH domain containing 2	FGD2
211316_x_at	-3.92	-4.15	CASP8 and FADD-like apoptosis regulator	CFLAR
243641_at	-3.94	-2.90		
1562938_at	-3.95	-3.19		
50221_at	-3.96	-2.69	transcription factor EB	TFEB
221752_at	-3.97	-3.33	slingshot homolog 1 (Drosophila)	SSH1
218723_s_at	-3.98	-2.66		
203823_at	-4.00	-4.87	regulator of G-protein signalling 3	RGS3
203851_at	-4.02	-2.85	insulin-like growth factor binding protein 6	IGFBP6
224565_at	-4.02	-2.78		

209050_s_at	-4.03	-2.78	ral guanine nucleotide dissociation stimulator	RALGDS
231699_at	-4.03	-2.58	nuclear factor of kappa light polypeptide gene enhancer in B-cells inhibitor, alpha	NFKBIA
1569872_a_at	-4.03	-2.45		
201473_at	-4.05	-2.22	jun B proto-oncogene	JUNB
213620_s_at	-4.07	-3.05	intercellular adhesion molecule 2	ICAM2
217529_at	-4.07	-4.80		
220578_at	-4.11	-5.14	ADAMTS-like 4	ADAMTSL4
226382_at	-4.11	-3.18		
229441_at	-4.12	-2.31	protease, serine, 23	PRSS23
212828_at	-4.14	-5.79	synaptojanin 2	SYNJ2
237448_at	-4.16	-4.32		
228258_at	-4.18	-2.67	TBC1 domain family, member 10C	TBC1D10C
227260_at	-4.19	-2.36		
201945_at	-4.21	-3.65	furin (paired basic amino acid cleaving enzyme)	FURIN
219975_x_at	-4.24	-11.16	oleoyl-ACP hydrolase	OLAH
221565_s_at	-4.24	-4.51	family with sequence similarity 26, member B	FAM26B
241506_at	-4.25	-4.73	latrophilin 3	LPHN3
204567_s_at	-4.26	-3.66	ATP-binding cassette, sub-family G (WHITE), member 1	ABCG1
221680_s_at	-4.26	-4.17	ets variant gene 7 (TEL2 oncogene)	ETV7
1558426_x_at	-4.26	-2.65	transmembrane protein 142B	TMEM142B
223158_s_at	-4.27	-2.40	NIMA (never in mitosis gene a)-related kinase 6	NEK6
223961_s_at	-4.28	-2.74	cytokine inducible SH2-containing protein	CISH
209498_at	-4.30	-2.11	carcinoembryonic antigen-related cell adhesion molecule 1 (biliary glycoprotein)	CEACAM1
231175_at	-4.31	-2.40	chromosome 6 open reading frame 65	C6orf65
219722_s_at	-4.34	-2.19	glycerophosphodiester phosphodiesterase domain containing 3	GDPD3
231406_at	-4.37	-4.91		
214012_at	-4.37	-2.23		
220326_s_at	-4.39	-2.81		
224701_at	-4.40	-2.17	poly (ADP-ribose) polymerase family, member 14	PARP14
209959_at	-4.40	-4.08	nuclear receptor subfamily 4, group A, member 3	NR4A3
201422_at	-4.41	-2.40	interferon, gamma-inducible protein 30	IFI30
238823_at	-4.43	-2.41	formin-like 3	FMNL3
221016_s_at	-4.44	-3.41	transcription factor 7-like 1 (T-cell specific, HMG-box)	TCF7L1
1569257_at	-4.47	-2.82	formin-like 1	FMNL1
219313_at	-4.49	-2.69	GRAM domain containing 1C	GRAMD1C
212122_at	-4.49	-2.72	ras homolog gene family, member Q	RHOQ

218368_s_at	-4.51	-4.05	tumor necrosis factor receptor superfamily, member 12A	TNFRSF12A
242811_x_at	-4.53	-2.01		
226189_at	-4.55	-2.60	integrin, beta 8	ITGB8
205081_at	-4.56	-4.32	cysteine-rich protein 1 (intestinal)	CRIP1
1555103_s_at	-4.57	-11.29	fibroblast growth factor 7 (keratinocyte growth factor)	FGF7
1559172_at	-4.59	-6.72		
1560517_s_at	-4.62	-51.29		
204963_at	-4.64	-2.34	sarcospan (Kras oncogene-associated gene)	SSPN
1560128_x_at	-4.65	-2.16		
58780_s_at	-4.67	-3.51		
1556072_at	-4.68	-4.12	chromosome 22 open reading frame 37	C22orf37
208978_at	-4.71	-7.98	cysteine-rich protein 2	CRIP2
209930_s_at	-4.76	-2.61	nuclear factor (erythroid-derived 2), 45kDa	NFE2
214329_x_at	-4.78	-3.48	tumor necrosis factor (ligand) superfamily, member 10	TNFSF10
229354_at	-4.80	-5.40	aryl-hydrocarbon receptor repressor	AHRR
238595_at	-4.80	-3.46		
205419_at	-4.80	-2.39	Epstein-Barr virus induced gene 2 (lymphocyte-specific G protein-coupled receptor)	EBI2
202687_s_at	-4.81	-2.89	tumor necrosis factor (ligand) superfamily, member 10	TNFSF10
238725_at	-4.81	-3.49		
230034_x_at	-4.82	-2.34	mitochondrial ribosomal protein L41	MRPL41
226673_at	-4.85	-2.31	SH2 domain containing 3C	SH2D3C
225293_at	-4.85	-3.98	collagen, type XXVII, alpha 1	COL27A1
219202_at	-4.90	-3.38	rhomboid 5 homolog 2 (Drosophila)	RHBDF2
207160_at	-4.90	-3.82	interleukin 12A (natural killer cell stimulatory factor 1, cytotoxic lymphocyte maturation factor 1,	IL12A
228885_at	-4.91	-7.03	ribosomal protein L24	RPL24
1556385_at	-4.92	-2.01		
221155_x_at	-4.92	-2.03		
217650_x_at	-4.93	-2.06	ST3 beta-galactoside alpha-2,3-sialyltransferase 2	ST3GAL2
1553204_at	-4.93	-4.45		
237802_at	-4.94	-2.02	XK, Kell blood group complex subunit-related family, member 4	XKR4
230563_at	-4.94	-8.75	RasGEF domain family, member 1A	RASGEF1A
1565628_at	-4.95	-4.58		
221753_at	-4.97	-3.59	slingshot homolog 1 (Drosophila)	SSH1
223159_s_at	-5.01	-3.97	NIMA (never in mitosis gene a)-related kinase 6	NEK6
204912_at	-5.06	-4.33	interleukin 10 receptor, alpha	IL10RA
201502_s_at	-5.07	-3.96	nuclear factor of kappa light polypeptide gene enhancer in B-cells inhibitor, alpha	NFKBIA
232322_x_at	-5.08	-3.03	START domain containing 10	STARD10

35626_at	-5.10	-5.20	N-sulfoglucosamine sulfohydrolase (sulfamidase)	SGSH
203726_s_at	-5.11	-3.33	laminin, alpha 3	LAMA3
1553706_at	-5.17	-8.52	HtrA serine peptidase 4	HTRA4
205463_s_at	-5.20	-4.54	platelet-derived growth factor alpha polypeptide	PDGFA
242755_at	-5.22	-2.07	SFRS protein kinase 2	SRPK2
230913_at	-5.30	-5.41		
203320_at	-5.30	-3.19	SH2B adaptor protein 3	SH2B3
238044_at	-5.31	-4.38		
209278_s_at	-5.34	-3.19	tissue factor pathway inhibitor 2	TFPI2
202688_at	-5.34	-3.58	tumor necrosis factor (ligand) superfamily, member 10	TNFSF10
219878_s_at	-5.34	-3.17	Kruppel-like factor 13	KLF13
241205_at	-5.35	-2.61	BMP2 inducible kinase	BMP2K
219183_s_at	-5.41	-2.51	pleckstrin homology, Sec7 and coiled-coil domains 4	PSCD4
238354_x_at	-5.42	-3.21		
217853_at	-5.46	-5.83	tensin 3	TNS3
1557749_at	-5.47	-2.25	EH domain binding protein 1-like 1	EHBP1L1
221087_s_at	-5.48	-7.01	apolipoprotein L, 3	APOL3
1561346_at	-5.48	-2.61		
206729_at	-5.55	-3.32	tumor necrosis factor receptor superfamily, member 8	TNFRSF8
222291_at	-5.56	-2.45		
1569150_x_at	-5.59	-5.07	PDZ and LIM domain 7 (enigma)	PDLIM7
207196_s_at	-5.60	-6.00	TNFAIP3 interacting protein 1	TNIP1
204794_at	-5.67	-4.33	dual specificity phosphatase 2	DUSP2
244580_at	-5.73	-2.67		
224569_s_at	-5.73	-2.67	interferon regulatory factor 2 binding protein 2	IRF2BP2
205180_s_at	-5.77	-3.58	ADAM metallopeptidase domain 8	ADAM8
205921_s_at	-5.78	-23.77	solute carrier family 6 (neurotransmitter transporter, taurine), member 6	SLC6A6
223438_s_at	-5.82	-3.04	peroxisome proliferator-activated receptor alpha	PPARA
210538_s_at	-5.87	-3.47	baculoviral IAP repeat-containing 3	BIRC3
241824_at	-5.89	-2.48	FOS-like antigen 2	FOSL2
238327_at	-5.91	-2.48		
230170_at	-5.92	-4.60	oncostatin M	OSM
218532_s_at	-5.97	-3.42		
210029_at	-6.03	-4.45	indoleamine-pyrrole 2,3 dioxygenase	INDO
201170_s_at	-6.06	-4.36	basic helix-loop-helix domain containing, class B, 2	BHLHB2
234989_at	-6.07	-5.04		
209722_s_at	-6.10	-4.15	serpin peptidase inhibitor, clade B (ovalbumin), member 9	SERPINB9
237016_at	-6.13	-2.50	chromosome 6 open reading frame 128	C6orf128
204198_s_at	-6.19	-4.87	runt-related transcription factor 3	RUNX3

225136_at	-6.19	-4.51	pleckstrin homology domain containing, family A (phosphoinositide binding specific) member 2	PLEKHA2
205681_at	-6.21	-2.67	BCL2-related protein A1	BCL2A1
204197_s_at	-6.27	-4.67	runt-related transcription factor 3	RUNX3
243934_at	-6.30	-2.31		
204205_at	-6.35	-3.94	apolipoprotein B mRNA editing enzyme, catalytic polypeptide-like 3G	APOBEC3G
207375_s_at	-6.36	-5.64	interleukin 15 receptor, alpha	IL15RA
224762_at	-6.40	-2.63	serine incorporator 2	SERINC2
219256_s_at	-6.49	-4.65	SH3 domain and tetratricopeptide repeats 1	SH3TC1
1563357_at	-6.54	-6.27		
228042_at	-6.62	-5.05	ADP-ribosylarginine hydrolase	ADPRH
227062_at	-6.65	-5.66		
1556950_s_at	-6.73	-2.65	serpin peptidase inhibitor, clade B (ovalbumin), member 6	SERPINB6
60471_at	-6.81	-3.19	Ras and Rab interactor 3	RIN3
209374_s_at	-6.81	-3.73	immunoglobulin heavy constant mu	IGHM
235116_at	-6.81	-2.75	TNF receptor-associated factor 1	TRAF1
222996_s_at	-6.83	-5.23	CXXC finger 5	CXXC5
225214_at	-6.94	-4.25		
223484_at	-6.94	-3.74	chromosome 15 open reading frame 48	C15orf48
213590_at	-6.98	-3.58	solute carrier family 16, member 5 (monocarboxylic acid transporter 6)	SLC16A5
224516_s_at	-6.98	-5.74	CXXC finger 5	CXXC5
212124_at	-6.99	-4.81	zinc finger, MIZ-type containing 1	ZMIZ1
219457_s_at	-7.01	-4.67	Ras and Rab interactor 3	RIN3
220118_at	-7.03	-3.26	zinc finger and BTB domain containing 32	ZBTB32
215595_x_at	-7.07	-2.76		
233853_at	-7.08	-2.56		
205069_s_at	-7.08	-2.37	Rho GTPase activating protein 26	ARHGAP26
205242_at	-7.11	-2.35	chemokine (C-X-C motif) ligand 13 (B-cell chemoattractant)	CXCL13
240232_at	-7.25	-13.00	chromosome 3 open reading frame 1	C3orf1
210271_at	-7.30	-6.99	neurogenic differentiation 2	NEUROD2
236127_at	-7.31	-2.17		
209262_s_at	-7.36	-3.89	nuclear receptor subfamily 2, group F, member 6	NR2F6
239213_at	-7.45	-7.00	serpin peptidase inhibitor, clade B (ovalbumin), member 1	SERPINB1
207176_s_at	-7.51	-9.22	CD80 molecule	CD80
230405_at	-7.71	-6.11		
201189_s_at	-7.77	-2.09	inositol 1,4,5-triphosphate receptor, type 3	ITPR3
212827_at	-7.79	-7.05	immunoglobulin heavy constant mu	IGHM
223887_at	-7.86	-7.28	G protein-coupled receptor 132	GPR132
236401_at	-7.88	-2.14	amiloride binding protein 1 (amine oxidase (copper-containing))	ABP1

233955_x_at	-7.94	-5.51	CXXC finger 5	CXXC5
205398_s_at	-7.98	-5.58	SMAD family member 3	SMAD3
226056_at	-8.04	-2.89		
211786_at	-8.09	-2.72	tumor necrosis factor receptor superfamily, member 9	TNFRSF9
211317_s_at	-8.12	-3.79	CASP8 and FADD-like apoptosis regulator	CFLAR
233056_x_at	-8.12	-2.41	discs, large (Drosophila) homolog-associated protein 4	DLGAP4
229830_at	-8.24	-4.37		
223514_at	-8.33	-15.22	caspase recruitment domain family, member 11	CARD11
234455_at	-8.59	-12.15	zinc finger protein 1 homolog (mouse)	ZFP1
1557056_at	-8.59	-18.71		
244654_at	-8.65	-6.14	myosin IG	MYO1G
33304_at	-8.81	-5.45	interferon stimulated exonuclease gene 20kDa	ISG20
202638_s_at	-8.87	-3.99	intercellular adhesion molecule 1 (CD54), human rhinovirus receptor	ICAM1
238013_at	-8.89	-4.95	pleckstrin homology domain containing, family A (phosphoinositide binding specific) member 2	PLEKHA2
243489_at	-9.11	-2.85		
242468_at	-9.13	-3.09		
209723_at	-9.27	-7.55	serpin peptidase inhibitor, clade B (ovalbumin), member 9	SERPINB9
232593_at	-9.45	-9.74		
204698_at	-9.81	-5.77	interferon stimulated exonuclease gene 20kDa	ISG20
232424_at	-9.90	-42.23	PR domain containing 16	PRDM16
207361_at	-9.90	-4.74	HMG-box transcription factor 1	HBP1
244342_at	-9.94	-2.13		
243869_at	-10.09	-3.67		
203045_at	-10.26	-5.81	ninjurin 1	NINJ1
208087_s_at	-10.38	-4.86	Z-DNA binding protein 1	ZBP1
240182_at	-10.47	-4.27		
206723_s_at	-10.56	-4.52	endothelial differentiation, lysophosphatidic acid G-protein-coupled receptor, 4	EDG4
225239_at	-10.71	-6.58		
214868_at	-10.77	-5.97	piwi-like 1 (Drosophila)	PIWIL1
239744_at	-10.84	-8.85		
1555689_at	-10.89	-8.28	CD80 molecule	CD80
228754_at	-11.03	-7.27	solute carrier family 6 (neurotransmitter transporter, taurine), member 6	SLC6A6
221477_s_at	-11.06	-2.83	superoxide dismutase 2, mitochondrial	SOD2
228432_at	-11.89	-8.07	RAB3A interacting protein (rabin3)	RAB3IP
206337_at	-12.20	-7.13	chemokine (C-C motif) receptor 7	CCR7
204908_s_at	-12.83	-6.10	B-cell CLL/lymphoma 3	BCL3

242814_at	-12.94	-6.30	serpin peptidase inhibitor, clade B (ovalbumin), member 9	SERPINB9
235380_at	-13.21	-2.95		
1569149_at	-13.37	-7.17	PDZ and LIM domain 7 (enigma)	PDLIM7
227347_x_at	-13.83	-4.52	hairy and enhancer of split 4 (Drosophila)	HES4
220091_at	-14.07	-18.02	solute carrier family 2 (facilitated glucose transporter), member 6	SLC2A6
206924_at	-15.29	-6.62	interleukin 11	IL11
211343_s_at	-15.49	-16.61	collagen, type XIII, alpha 1	COL13A1
243065_at	-15.65	-2.09		
241097_at	-15.95	-2.41	ras homolog gene family, member A	RHOA
205599_at	-17.05	-7.65	TNF receptor-associated factor 1	TRAF1
205799_s_at	-18.26	-2.02	solute carrier family 3 (cystine, dibasic and neutral amino acid transporters, activator of cystine,	SLC3A1
226913_s_at	-18.92	-53.38	SRY (sex determining region Y)-box 8	SOX8
235427_at	-19.90	-4.19		
1554519_at	-19.96	-9.28	CD80 molecule	CD80
1557717_at	-26.43	-3.92		
207010_at	-32.30	-4.94	gamma-aminobutyric acid (GABA) A receptor, beta 1	GABRB1
234247_at	-44.30	-3.50		
1564121_at	-45.52	-2.26		
206341_at	-94.94	-13.14	interleukin 2 receptor, alpha	IL2RA
215671_at	-103.17	-3.97	phosphodiesterase 4B, cAMP-specific (phosphodiesterase E4 dunce homolog, Drosophila)	PDE4B
