# Supplementary material for: In Vitro Transformation of Primary Human CD34+ Cells by AML Fusion Oncogenes: Early Gene Expression Profiling Reveals Possible Drug Target in AML
Source: PLoS One. 2010 Aug 27;5(8):e12464. doi: 10.1371/journal.pone.0012464 (PMC2929205; doi:10.1371/journal.pone.0012464)
Supplement: Table S5 — Genes deregulated by PML-RARA 3 days after transduction. Primary human CD34+ cells were retrovirally transduced with either control MSCV-IRES-GFP vector or vector expressing PML-RARA and sorted for GFP positivity. Total RNA was extracted 3 days after transduction and subjected to microarray analysis. Genes that showed up- or down-regulation by 2 fold or more in comparison to the control in 2 independent experiments (Exp.1 and Exp.2) were considered deregulated. (0.08 MB PDF) [file pone.0012464.s005.pdf]

Table S5. Genes deregulated by PML-RARA at 3 d after transduction

Probe set ID	Fold Change		Gene Name	Gene Symbol
	Exp.1	Exp.2		
1553428_at	48.53	67.51		
241147_at	25.35	2.19		
227503_at	13.63	2.52		
244557_at	11.54	8.83		
206785_s_at	10.40	13.01	killer cell lectin-like receptor subfamily C, member 2	KLRC2
233577_at	10.19	3.70	chromosome 18 open reading frame 57	C18orf57
228431_at	9.66	3.40		
211349_at	8.39	4.73	solute carrier family 15 (oligopeptide transporter), member 1	SLC15A1
202222_s_at	7.71	3.27	desmin	DES
216444_at	6.43	24.31		
1556817_a_at	5.81	2.23		
206655_s_at	5.24	2.43	glycoprotein Ib (platelet), beta polypeptide	GP1BB
237515_at	4.98	3.50	transmembrane protein 56	TMEM56
241030_at	4.65	3.29	fibrous sheath interacting protein 1	FSIP1
240979_at	4.55	2.33		
206773_at	4.46	2.42	lymphocyte antigen 6 complex, locus H	LY6H
239913_at	3.86	5.89	solute carrier family 10 (sodium/bile acid cotransporter family), member 4	SLC10A4
1562189_at	3.84	24.69	collagen, type XIV, alpha 1 (undulin)	COL14A1
238060_s_at	3.82	3.35	beta-1,4-N-acetyl-galactosaminyl transferase 4	B4GALNT4
1560455_at	3.81	2.16		
201131_s_at	3.47	2.75	cadherin 1, type 1, E-cadherin (epithelial)	CDH1
241777_x_at	3.43	2.64	adaptor protein, phosphotyrosine interaction, PH domain and leucine zipper containing 2	APPL2
211361_s_at	3.39	12.11	serpin peptidase inhibitor, clade B (ovalbumin), member 13	SERPINB13
1561191_at	3.38	5.63		
217274_x_at	3.37	2.98	myosin, light chain 4, alkali; atrial, embryonic	MYL4
1555488_at	3.37	4.72		
242579_at	3.11	2.09		
1562579_at	3.10	2.27		
236071_at	2.97	9.00		
1559529_at	2.92	2.84	PTK2 protein tyrosine kinase 2	PTK2
1554143_a_at	2.87	2.00	SGT1, suppressor of G2 allele of SKP1 like 1 (S. cerevisiae)	SUGT1L1
239606_at	2.86	2.02		
216764_at	2.74	4.39		
204321_at	2.74	2.72	neogenin homolog 1 (chicken)	NEO1
203038_at	2.74	5.00	protein tyrosine phosphatase, receptor type, K	PTPRK
205950_s_at	2.66	2.20	carbonic anhydrase I	CA1
1561056_a_at	2.61	2.76		

1561571_at	2.57	2.18		
243095_at	2.56	3.03		
234632_x_at	2.49	2.62		
243428_at	2.48	2.32	KCNQ1 overlapping transcript 1	KCNQ1OT1
209795_at	2.46	2.06	CD69 molecule	CD69
204416_x_at	2.43	3.03	apolipoprotein C-I	APOC1
241184_x_at	2.42	2.49	zinc finger protein 407	ZNF407
1556105_at	2.41	3.23		
233321_x_at	2.40	2.68		
224894_at	2.38	2.93	Yes-associated protein 1, 65kDa	YAP1
230688_at	2.31	2.45		
243012_at	2.29	4.23		
230560_at	2.27	11.12	syntaxin binding protein 6 (amisyn)	STXBP6
1557286_at	2.26	2.64		
207901_at	2.26	6.17	interleukin 12B (natural killer cell stimulatory factor 2, cytotoxic lymphocyte maturation factor 2,	IL12B
1557403_s_at	2.25	6.57		
221541_at	2.25	2.06	cysteine-rich secretory protein LCCL domain containing 2	CRISPLD2
219672_at	2.24	2.31	erythroid associated factor	ERAF
238353_at	2.23	2.14	RAS-like, family 11, member A	RASL11A
1552658_a_at	2.22	2.89	neuron navigator 3	NAV3
204217_s_at	2.19	8.80	reticulon 2	RTN2
235906_at	2.19	2.19	integrin-linked kinase	ILK
1569294_at	2.19	3.19	ring finger protein 187	RNF187
1562527_at	2.18	2.07		
1561141_at	2.18	27.47		
240542_at	2.15	2.08	microtubule-associated protein 4	MAP4
206306_at	2.13	4.03	ryanodine receptor 3	RYR3
214366_s_at	2.13	2.21	arachidonate 5-lipoxygenase	ALOX5
214278_s_at	2.11	9.32	NDRG family member 2	NDRG2
214984_at	2.11	3.54		
1570639_at	2.11	2.63		
215284_at	2.11	2.90		
1558606_s_at	2.09	7.89		
1562214_at	2.09	4.32		
1570033_at	2.08	3.61	WD repeat domain, phosphoinositide interacting 2	WIPI2
215306_at	2.06	4.76		
1561453_at	2.05	4.04		
219983_at	2.03	6.35	HRAS-like suppressor	HRASLS
233853_at	2.03	2.07		
240751_at	2.03	2.31	ubiquitin specific peptidase 8	USP8
223644_s_at	2.01	2.51	crystallin, gamma S	CRYGS
235300_x_at	-2.02	-2.18	ring finger and CHY zinc finger domain containing 1	RCHY1
1553285_s_at	-2.06	-2.40	RAD9 homolog B (S. cerevisiae)	RAD9B
223698_at	-2.06	-2.38	solute carrier family 25, member 36	SLC25A36
1562836_at	-2.08	-2.24		
222283_at	-2.14	-2.43	zinc finger protein 480	ZNF480
235502_at	-2.17	-2.36		

239192_at	-2.25	-3.13	par-3 partitioning defective 3 homolog B (C. elegans)	PARD3B
232959_at	-2.26	-2.23		
222943_at	-2.31	-2.96	glucosidase, beta, acid 3 (cytosolic)	GBA3
232484_at	-2.32	-2.08		
243952_at	-2.37	-2.57		
1556282_at	-2.46	-2.06	FGFR1 oncogene partner 2	FGFR1OP2
240391_at	-2.46	-2.31	NADH dehydrogenase (ubiquinone) 1 beta subcomplex, 2, 8kDa	NDUFB2
215995_x_at	-2.79	-2.65		
230834_at	-3.17	-4.05		
230055_at	-3.18	-2.17	chromosome 6 open reading frame 148	C6orf148
1565150_at	-3.32	-2.48		
1555340_x_at	-4.52	-2.52	RAP1A, member of RAS oncogene family	RAP1A
1555339_at	-5.01	-2.85	RAP1A, member of RAS oncogene family	RAP1A
1556325_at	-5.11	-2.04	filamin A interacting protein 1	FILIP1
1558177_at	-5.87	-3.07	chromosome 14 open reading frame 83	C14orf83
205879_x_at	-6.06	-2.02	ret proto-oncogene (multiple endocrine neoplasia and medullary thyroid carcinoma 1, Hirschsprung dis	RET
1557169_x_at	-6.43	-2.36	HLA complex group 11	HCG11
241781_at	-8.55	-2.00	chromosome 9 open reading frame 41	C9orf41
223884_at	-9.36	-2.14	opticin	OPTC
1558786_at	-10.81	-2.40		
230876_at	-10.85	-62.50		
