# Supplementary material for: In Vitro Transformation of Primary Human CD34+ Cells by AML Fusion Oncogenes: Early Gene Expression Profiling Reveals Possible Drug Target in AML
Source: PLoS One. 2010 Aug 27;5(8):e12464. doi: 10.1371/journal.pone.0012464 (PMC2929205; doi:10.1371/journal.pone.0012464)
Supplement: Table S6 — Genes deregulated by PML-RARA 8 days after transduction. Primary human CD34+ cells were retrovirally transduced with either control MSCV-IRES-GFP vector or vector expressing PML-RARA and sorted for GFP positivity. Total RNA was extracted 8 days after transduction and subjected to microarray analysis. Genes that showed up- or down-regulation by 2 fold or more in comparison to the control in 2 independent experiments (Exp.1 and Exp.2) were considered deregulated. (0.08 MB PDF) [file pone.0012464.s006.pdf]

Table S6. Genes deregulated by PML-RARA at 8 d after transduction

Probe set ID	Fold Change		Gene Name	Gene Symbol
	Exp.1	Exp.2		
221169_s_at	16.05	4.09	histamine receptor H4	HRH4
215507_x_at	10.29	3.44	RAB22A, member RAS oncogene family	RAB22A
206291_at	6.68	2.86	neurotensin	NTS
1564438_at	3.75	6.66		
224459_at	3.73	2.61	L-2-hydroxyglutarate dehydrogenase	L2HGDH
215674_at	3.48	2.10		
208712_at	2.89	5.23	cyclin D1	CCND1
213348_at	2.88	2.52	cyclin-dependent kinase inhibitor 1C (p57, Kip2)	CDKN1C
233945_at	2.86	2.21	UDP-glucose ceramide glucosyltransferase-like 2	UGCGL2
1552908_at	2.67	3.65	chromosome 1 open reading frame 150	C1orf150
201110_s_at	2.55	2.83	thrombospondin 1	THBS1
223805_at	2.50	2.84	oxysterol binding protein-like 6	OSBPL6
236845_at	2.44	2.44		
209881_s_at	2.28	2.26	linker for activation of T cells	LAT
215810_x_at	2.25	3.44		
211696_x_at	2.18	3.49	hemoglobin, beta	HBB
235062_at	2.17	6.59	PIH1 domain containing 2	PIH1D2
206306_at	2.14	2.56	ryanodine receptor 3	RYR3
201150_s_at	2.07	3.75	TIMP metallopeptidase inhibitor 3 (Sorsby fundus dystrophy, pseudoinflammatory)	TIMP3
205609_at	2.05	2.08	angiopoietin 1	ANGPT1
216191_s_at	2.02	2.04	T cell receptor alpha locus	TRA@
239617_at	2.02	5.28		
237034_at	2.02	2.51		
223958_s_at	2.01	2.39	dynein, axonemal, light chain 1	DNAL1
1558019_at	-2.00	-2.49		
1569827_at	-2.01	-2.37	ATG7 autophagy related 7 homolog (S. cerevisiae)	ATG7
1554229_at	-2.01	-2.17		
227842_at	-2.02	-8.25	RAB30, member RAS oncogene family	RAB30
1555024_at	-2.03	-2.23	ADAM metallopeptidase domain 22	ADAM22
214428_x_at	-2.03	-4.43	complement component 4A (Rodgers blood group)	C4A
234611_at	-2.04	-3.94		
243324_x_at	-2.04	-12.50		
207665_at	-2.05	-3.35	ADAM metallopeptidase domain 21	ADAM21
204813_at	-2.06	-2.16	mitogen-activated protein kinase 10	MAPK10
1568720_at	-2.07	-2.21	zinc finger protein 506	ZNF506
237673_at	-2.10	-2.41		
238586_at	-2.11	-3.18	tyrosine kinase, non-receptor, 2	TNK2
242199_at	-2.13	-2.73		
1556936_at	-2.13	-2.40		
1560552_a_at	-2.14	-2.30		
243934_at	-2.14	-2.47		
1555289_at	-2.18	-3.92	leucine zipper transcription regulator 2	LZTR2

204195_s_at	-2.20	-2.46	PBX/knotted 1 homeobox 1	PKNOX1
1562908_at	-2.20	-2.16		
1553523_at	-2.21	-4.11	NLR family, pyrin domain containing 14	NLRP14
241992_at	-2.21	-2.08		
211774_s_at	-2.29	-2.36	methylmalonic aciduria (cobalamin deficiency) cblC type, with homocystinuria	MMACHC
1555340_x_at	-2.29	-3.46	RAP1A, member of RAS oncogene family	RAP1A
1556175_at	-2.34	-4.16		
1558964_at	-2.36	-4.27	FAT tumor suppressor homolog 3 (Drosophila)	FAT3
1561604_at	-2.42	-2.00		
1557584_at	-2.45	-2.01		
1555339_at	-2.45	-2.13	RAP1A, member of RAS oncogene family	RAP1A
211106_at	-2.48	-2.86	suppressor of Ty 3 homolog (S. cerevisiae)	SUPT3H
244475_at	-2.50	-3.47		
234548_at	-2.52	-3.39		
1567031_at	-2.53	-4.72	zinc finger protein 160	ZNF160
1560264_at	-2.54	-2.47		
234239_at	-2.61	-2.35		
241555_at	-2.63	-4.61		
AFFX-HUMRGE/M	-2.65	-2.50		
233282_at	-2.65	-3.53		
244504_x_at	-2.66	-3.77	ADP-ribosylation factor 1	ARF1
217102_at	-2.67	-5.95		
1561207_at	-2.69	-2.37		
205590_at	-2.78	-6.26	RAS guanyl releasing protein 1 (calcium and DAG-regulated)	RASGRP1
1569786_at	-2.79	-3.25		
204665_at	-2.80	-3.00		
241700_at	-2.87	-3.36	zinc finger homeodomain 4	ZFHX4
226487_at	-2.87	-9.43	chromosome 12 open reading frame 34	C12orf34
217206_at	-2.93	-2.64		
212730_at	-2.93	-3.03	desmuslin	DMN
1569539_at	-2.96	-2.05		
217579_x_at	-3.23	-3.32		
203868_s_at	-3.28	-9.41	vascular cell adhesion molecule 1	VCAM1
220771_at	-3.37	-2.45		
1564765_at	-3.59	-3.23		
1563118_at	-3.67	-2.60		
1557057_a_at	-3.87	-3.05		
231367_s_at	-3.94	-2.19		
1566087_at	-4.15	-2.88		
1552991_at	-4.43	-15.36	olfactory receptor, family 5, subfamily P, member 2	OR5P2
222301_at	-4.51	-2.70	chromosome 1 open reading frame 61	C1orf61
213877_x_at	-4.75	-2.77	transcription elongation factor B (SIII), polypeptide 2 (18kDa, elongin B)	TCEB2
1556801_at	-4.85	-2.10		
232060_at	-4.89	-3.82		
216408_at	-5.11	-31.11	olfactory receptor, family 2, subfamily B, member 2	OR2B2
241636_x_at	-5.17	-2.34		
222720_x_at	-5.31	-3.60	chromosome 1 open reading frame 27	C1orf27

1553298_at	-5.50	-74.57	chromosome 17 open reading frame 77	C17orf77
233683_at	-5.51	-5.71		
224050_s_at	-6.23	-2.98		
206415_at	-7.72	-2.03	tolloid-like 1	TLL1
237675_at	-7.84	-3.17		
1561679_at	-10.02	-2.13		
1555103_s_at	-16.66	-2.41	fibroblast growth factor 7 (keratinocyte growth factor)	FGF7
214967_at	-18.97	-16.37		
1561362_at	-22.80	-3.73		
238392_at	-52.24	-3.34	translocation associated membrane protein 2	TRAM2
