# Supplementary material for: In Vitro Transformation of Primary Human CD34+ Cells by AML Fusion Oncogenes: Early Gene Expression Profiling Reveals Possible Drug Target in AML
Source: PLoS One. 2010 Aug 27;5(8):e12464. doi: 10.1371/journal.pone.0012464 (PMC2929205; doi:10.1371/journal.pone.0012464)
Supplement: Table S7 — Genes deregulated by MLL-AF9 6 h after transfection. Primary human CD34+ cells were nucleofected with either control pTracer-CMV/Bsd vector or vector expressing MLL-AF9 and sorted for GFP positivity. Total RNA was extracted 6 h after nucleofection and subjected to microarray analysis. Genes that showed up- or down-regulation by 2 fold or more in comparison to the control in 2 independent experiments (Exp.1 and Exp.2) were considered deregulated. (0.08 MB PDF) [file pone.0012464.s007.pdf]

Table S7. Genes deregulated by MLL-AF9 at 6 h after transfection

Probe set ID	Fold Change		Gene Name	Gene Symbol
	Exp.1	Exp.2		
1565436_s_at	14.85	73.56	myeloid/lymphoid or mixed-lineage leukemia (trithorax homolog, Drosophila)	MLL
229935_s_at	12.11	138.20	myeloid/lymphoid or mixed-lineage leukemia (trithorax homolog, Drosophila)	MLL
212079_s_at	7.82	4.08	myeloid/lymphoid or mixed-lineage leukemia (trithorax homolog, Drosophila)	MLL
212078_s_at	4.07	6.11	myeloid/lymphoid or mixed-lineage leukemia (trithorax homolog, Drosophila)	MLL
201416_at	3.66	5.01	SRY (sex determining region Y)-box 4	SOX4
212188_at	3.62	2.28	potassium channel tetramerisation domain containing 12	KCTD12
244021_at	3.30	3.47		
225806_at	3.29	2.02	jub, ajuba homolog (Xenopus laevis)	JUB
214534_at	3.21	10.83	histone cluster 1, H1b	HIST1H1B
242054_s_at	3.18	3.53		
206510_at	3.12	3.96	sine oculis homeobox homolog 2 (Drosophila)	SIX2
214537_at	3.10	4.43	histone cluster 1, H1d	HIST1H1D
226534_at	3.08	2.74	KIT ligand	KITLG
204802_at	3.06	4.81	Ras-related associated with diabetes	RRAD
209398_at	3.04	10.04	histone cluster 1, H1c	HIST1H1C
227546_x_at	2.95	5.95	cyclin L2	CCNL2
219727_at	2.91	2.18	dual oxidase 2	DUOX2
1553808_a_at	2.91	2.61	NK2 transcription factor related, locus 3 (Drosophila)	NKX2-3
219534_x_at	2.80	4.42	cyclin-dependent kinase inhibitor 1C (p57, Kip2)	CDKN1C
202668_at	2.78	2.37	ephrin-B2	EFNB2
228706_s_at	2.76	2.31	claudin 23	CLDN23
213577_at	2.74	3.71	squalene epoxidase	SQLE
213183_s_at	2.74	2.93	cyclin-dependent kinase inhibitor 1C (p57, Kip2)	CDKN1C
209291_at	2.70	2.14	inhibitor of DNA binding 4, dominant negative helix-loop-helix protein	ID4
204525_at	2.64	2.20	PHD finger protein 14	PHF14
226499_at	2.64	2.02		
235765_at	2.63	2.39	transducin-like enhancer of split 4 (E(sp1) homolog, Drosophila)	TLE4
241826_x_at	2.59	2.34		
213418_at	2.57	3.10	heat shock 70kDa protein 6 (HSP70B')	HSPA6
236668_at	2.57	2.55		
213182_x_at	2.54	3.81	cyclin-dependent kinase inhibitor 1C (p57, Kip2)	CDKN1C
209348_s_at	2.54	3.73	v-maf musculoaponeurotic fibrosarcoma oncogene homolog (avian)	MAF
235761_at	2.53	3.72		
233164_x_at	2.45	2.47	rhomboid domain containing 1	RHBDD1

228614_at	2.43	3.31		
1556579_s_at	2.36	2.22	immunoglobulin superfamily, member 10	IGSF10
219295_s_at	2.35	2.06	procollagen C-endopeptidase enhancer 2	PCOLCE2
222164_at	2.34	2.09		
227522_at	2.26	2.23	carboxymethylenebutenolidase homolog (Pseudomonas)	CMBL
203887_s_at	2.26	2.40	thrombomodulin	THBD
207688_s_at	2.20	3.07	inhibin, beta C	INHBC
217728_at	2.19	2.98	S100 calcium binding protein A6	S100A6
204659_s_at	2.18	3.06	growth factor, augmenter of liver regeneration (ERV1 homolog, S. cerevisiae)	GFER
203186_s_at	2.17	3.55	S100 calcium binding protein A4	S100A4
1552649_a_at	2.17	2.18	ring finger and FYVE-like domain containing 1	RFFL
1554742_at	2.15	2.21	PMS1 postmeiotic segregation increased 1 (S. cerevisiae)	PMS1
240533_at	2.15	2.03		
238935_at	2.14	3.31	ribosomal protein S27-like	RPS27L
225730_s_at	2.13	2.44	THUMP domain containing 3	THUMPD3
206669_at	2.13	2.54	glutamate decarboxylase 1 (brain, 67kDa)	GAD1
240044_x_at	2.12	2.83	trinucleotide repeat containing 6B	TNRC6B
1556194_a_at	2.10	3.91		
208506_at	2.09	2.44	histone cluster 1, H3f	HIST1H3F
203705_s_at	2.08	2.49	frizzled homolog 7 (Drosophila)	FZD7
1552794_a_at	2.08	2.12	zinc finger protein 547	ZNF547
203178_at	2.08	2.56	glycine amidinotransferase (L-arginine:glycine amidinotransferase)	GATM
221705_s_at	2.08	2.22		
203894_at	2.07	2.13	tubulin, gamma 2	TUBG2
203176_s_at	2.06	2.51	transcription factor A, mitochondrial	TFAM
207813_s_at	2.05	2.21	ferredoxin reductase	FDXR
213638_at	2.05	4.98	phosphatase and actin regulator 1	PHACTR1
218384_at	2.05	3.36	calcium regulated heat stable protein 1, 24kDa	CARHSP1
1558842_at	2.04	2.60		
201730_s_at	2.04	2.38	translocated promoter region (to activated MET oncogene)	TPR
227197_at	2.03	2.48		
207857_at	2.03	2.52	leukocyte immunoglobulin-like receptor, subfamily A (with TM domain), member 2	LILRA2
233852_at	2.02	2.50	polymerase (DNA directed), eta	POLH
236428_at	2.01	2.01		
221043_at	2.01	2.02		
213479_at	2.00	2.12	neuronal pentraxin II	NPTX2
242237_at	-2.01	-3.23	THO complex 7 homolog (Drosophila)	THOC7
1555884_at	-2.01	-2.22	proteasome (prosome, macropain) 26S subunit, non-ATPase, 6	PSMD6
214020_x_at	-2.01	-3.05	integrin, beta 5	ITGB5
224762_at	-2.02	-3.66	serine incorporator 2	SERINC2
214132_at	-2.03	-2.27	ATP synthase, H+ transporting, mitochondrial F1 complex, gamma polypeptide 1	ATP5C1

206031_s_at	-2.03	-2.05	ubiquitin specific peptidase 5 (isopeptidase T)	USP5
235884_at	-2.04	-2.65		
237579_at	-2.04	-2.72		
236122_at	-2.04	-2.21		
239759_at	-2.05	-6.27		
232355_at	-2.06	-2.75		
236419_at	-2.10	-2.02	transforming growth factor, beta receptor II (70/80kDa)	TGFBR2
238658_at	-2.11	-2.33		
240783_at	-2.12	-2.65		
1561488_at	-2.13	-2.58		
1558561_at	-2.16	-2.50	histocompatibility (minor) 13	HM13
207216_at	-2.16	-3.84	tumor necrosis factor (ligand) superfamily, member 8	TNFSF8
211338_at	-2.17	-3.11	interferon, alpha 2	IFNA2
238414_at	-2.18	-7.35		
1568768_s_at	-2.19	-5.69	brain and reproductive organ-expressed (TNFRSF1A modulator)	BRE
210362_x_at	-2.19	-2.70	promyelocytic leukemia	PML
1556761_at	-2.25	-2.13		
231954_at	-2.25	-2.15		
211354_s_at	-2.25	-4.81	leptin receptor	LEPR
218850_s_at	-2.32	-2.66	LIM domains containing 1	LIMD1
203357_s_at	-2.34	-2.28	calpain 7	CAPN7
222139_at	-2.35	-2.13		
210190_at	-2.37	-6.05	syntaxin 11	STX11
231958_at	-2.38	-2.01	chromosome 3 open reading frame 31	C3orf31
233622_x_at	-2.42	-2.40		
239381_at	-2.43	-2.34	kallikrein-related peptidase 7	KLK7
224533_s_at	-2.44	-10.87	interferon, alpha-inducible protein 6	IFI6
233784_at	-2.47	-3.13		
240703_s_at	-2.48	-5.04		
232593_at	-2.49	-2.05		
217388_s_at	-2.50	-2.89	kynureninase (L-kynurenine hydrolase)	KYNU
1557649_at	-2.51	-2.18	UDP-galactose-4-epimerase	GALE
211862_x_at	-2.51	-2.22	CASP8 and FADD-like apoptosis regulator	CFLAR
225239_at	-2.55	-2.44		
234989_at	-2.57	-3.71		
243748_at	-2.62	-3.94	eukaryotic translation initiation factor 4E family member 3	EIF4E3
243929_at	-2.66	-2.32	zinc finger protein 533	ZNF533
210029_at	-2.70	-2.81	indoleamine-pyrrole 2,3 dioxygenase	INDO
242299_at	-2.72	-2.18		
202357_s_at	-2.82	-3.13	complement factor B	CFB
234455_at	-2.84	-23.60	zinc finger protein 1 homolog (mouse)	ZFP1
204667_at	-2.84	-2.27	forkhead box A1	FOXA1
224106_at	-2.85	-2.67		
1558759_s_at	-2.91	-2.03	cytoplasmic linker associated protein 2	CLASP2
239712_at	-3.27	-3.50	chromosome 9 open reading frame 93	C9orf93
205242_at	-3.45	-2.57	chemokine (C-X-C motif) ligand 13 (B-cell chemoattractant)	CXCL13
237244_at	-3.54	-9.08		

240088_at	-3.55	-5.29	phosphodiesterase 5A, cGMP-specific	PDE5A
207859_s_at	-3.66	-4.85	cholinergic receptor, nicotinic, beta 3	CHRNB3
1560349_at	-3.71	-56.89		
234082_at	-3.87	-2.46		
227062_at	-3.99	-4.60		
1555103_s_at	-4.06	-12.37	fibroblast growth factor 7 (keratinocyte growth factor)	FGF7
241740_at	-4.38	-3.19	cAMP responsive element modulator	CREM
235704_at	-5.58	-7.45	DAZ associated protein 2	DAZAP2
227851_s_at	-5.62	-9.61		
1555199_at	-5.79	-3.58	golgi SNAP receptor complex member 1	GOSR1
229592_at	-9.20	-27.21		
221404_at	-11.23	-3.07	interleukin 1 family, member 6 (epsilon)	IL1F6
240658_at	-12.40	-5.16		
