# Supplementary material for: In Vitro Transformation of Primary Human CD34+ Cells by AML Fusion Oncogenes: Early Gene Expression Profiling Reveals Possible Drug Target in AML
Source: PLoS One. 2010 Aug 27;5(8):e12464. doi: 10.1371/journal.pone.0012464 (PMC2929205; doi:10.1371/journal.pone.0012464)
Supplement: Table S8 — Genes deregulated by MLL-AF9 3 days after transduction. Primary human CD34+ cells were retrovirally transduced with either control MSCV-IRES-GFP vector or vector expressing MLL-AF9 and sorted for GFP positivity. Total RNA was extracted 3 days after transduction and subjected to microarray analysis. Genes that showed up- or down-regulation by 2 fold or more in comparison to the control in 2 independent experiments (Exp.1 and Exp.2) were considered deregulated. (0.11 MB PDF) [file pone.0012464.s008.pdf]

Table S8. Genes deregulated by MLL-AF9 at 3 d after transduction

Probe set ID	Fold Change		Gene Name	Gene Symbol
	Exp.1	Exp.2		
1565875_at	39.18	2.28	nucleoporin 153kDa	NUP153
217145_at	22.17	2.09	immunoglobulin kappa constant	IGKC
1565436_s_at	17.64	8.34	myeloid/lymphoid or mixed-lineage leukemia (trithorax homolog, Drosophila)	MLL
216155_at	15.95	2.16		
211602_s_at	15.42	7.76	transient receptor potential cation channel, subfamily C, member 1	TRPC1
216067_at	15.35	2.60		
223812_at	13.16	14.33		
212079_s_at	13.15	6.77	myeloid/lymphoid or mixed-lineage leukemia (trithorax homolog, Drosophila)	MLL
212078_s_at	11.32	7.19	myeloid/lymphoid or mixed-lineage leukemia (trithorax homolog, Drosophila)	MLL
244557_at	10.47	9.66		
207447_s_at	9.73	3.81	mannosyl (alpha-1,3-)-glycoprotein beta-1,4-N-acetylglucosaminyltransferase, isozyme C (putative)	MGAT4C
237310_at	9.38	2.31	exostoses (multiple) 1	EXT1
206655_s_at	8.96	2.84	glycoprotein Ib (platelet), beta polypeptide	GP1BB
1563467_at	8.51	2.72		
240775_at	8.34	2.05	zinc finger protein 407	ZNF407
240135_x_at	8.12	3.61	TIMP metallopeptidase inhibitor 3 (Sorsby fundus dystrophy, pseudoinflammatory)	TIMP3
1559949_at	8.03	2.24	trichorhinophalangeal syndrome I	TRPS1
231598_x_at	7.04	5.59		
1561264_at	6.92	2.10		
242868_at	6.79	2.91	endothelial PAS domain protein 1	EPAS1
231734_at	6.62	7.20	retinol binding protein 2, cellular	RBP2
1564028_s_at	6.35	2.07		
210230_at	6.15	2.61		
234618_at	5.99	2.06		
214295_at	5.59	2.39		
230167_at	5.47	2.80	ADAM metallopeptidase with thrombospondin type 1 motif, 14	ADAMTS14
1561192_at	5.37	3.60		
239945_at	5.16	2.06		
240979_at	4.92	2.20		
1563452_at	4.87	2.04		
1554933_at	4.84	2.45	PC4 and SFRS1 interacting protein 1	PSIP1
243216_x_at	4.82	3.45	ubiquitin specific peptidase 40	USP40
239492_at	4.81	2.80	SEC14-like 4 (S. cerevisiae)	SEC14L4

229151_at	4.70	7.29	solute carrier family 14 (urea transporter), member 1 (Kidd blood group)	SLC14A1
243715_at	4.63	3.01		
237216_at	4.62	2.44	GTPase activating Rap/RanGAP domain-like 1	GARNL1
241030_at	4.62	2.97	fibrous sheath interacting protein 1	FSIP1
1556983_a_at	4.59	2.54		
215206_at	4.58	2.37		
241184_x_at	4.53	3.77	zinc finger protein 407	ZNF407
213553_x_at	4.52	2.26	apolipoprotein C-I	APOC1
201693_s_at	4.48	2.21	early growth response 1	EGR1
1566043_at	4.32	2.10		
243262_at	4.30	2.14	SET and MYND domain containing 3	SMYD3
219737_s_at	4.30	6.24	protocadherin 9	PCDH9
1562235_s_at	4.26	2.09		
1555014_x_at	4.21	2.77		
206937_at	4.16	2.15	spectrin, alpha, erythrocytic 1 (elliptocytosis 2)	SPTA1
1556425_a_at	4.10	2.11		
242622_x_at	4.00	2.01	phosphatase and tensin homolog (mutated in multiple advanced cancers 1)	PTEN
235592_at	3.95	2.25		
237614_at	3.92	2.20		
214294_at	3.73	2.61		
233004_x_at	3.71	2.01		
1569660_at	3.68	2.20		
232748_at	3.66	4.75	pregnancy-associated plasma protein A, pappalysin 1	PAPPA
1569067_at	3.64	2.30		
226716_at	3.63	2.34	proline rich 12	PRR12
239832_at	3.63	2.10		
231597_x_at	3.59	5.29		
234056_at	3.52	2.29		
241837_at	3.50	2.18	AT rich interactive domain 5B (MRF1-like)	ARID5B
238919_at	3.48	5.56		
233853_at	3.47	2.02		
242172_at	3.43	2.32	Meis1, myeloid ecotropic viral integration site 1 homolog (mouse)	MEIS1
1559376_at	3.41	2.57	chromosome 1 open reading frame 203	C1orf203
1555372_at	3.38	2.02	BCL2-like 11 (apoptosis facilitator)	BCL2L11
244104_at	3.38	2.58	mannosyl (beta-1,4-)-glycoprotein beta-1,4-N-acetylglucosaminyltransferase	MGAT3
240602_at	3.37	2.30	HBS1-like (S. cerevisiae)	HBS1L
230728_at	3.36	2.05		
240965_at	3.35	2.14	anaphase promoting complex subunit 10	ANAPC10
235133_at	3.34	2.32		
1561759_at	3.33	3.03		
217071_s_at	3.28	7.52	5,10-methylenetetrahydrofolate reductase (NADPH)	MTHFR
239856_at	3.26	2.79		
243122_at	3.25	2.14		

242733_at	3.25	2.52		
233202_at	3.24	3.33	contactin associated protein-like 3	CNTNAP3
229540_at	3.23	2.01	recombining binding protein suppressor of hairless (Drosophila)	RBPSUH
232896_at	3.23	2.01	erbb2 interacting protein	ERBB2IP
241154_x_at	3.23	3.02		
1570290_at	3.23	2.46		
206281_at	3.15	2.34	adenylate cyclase activating polypeptide 1 (pituitary)	ADCYAP1
237515_at	3.14	2.84	transmembrane protein 56	TMEM56
242572_at	3.14	2.52		
237953_at	3.14	3.93	dipeptidyl-peptidase 4 (CD26, adenosine deaminase complexing protein 2)	DPP4
205262_at	3.12	2.14	potassium voltage-gated channel, subfamily H (eag-related), member 2	KCNH2
215246_at	3.10	2.86	La ribonucleoprotein domain family, member 7	LARP7
231585_at	3.09	2.95	vacuolar protein sorting 13 homolog A (S. cerevisiae)	VPS13A
201556_s_at	3.09	2.48	vesicle-associated membrane protein 2 (synaptobrevin 2)	VAMP2
215306_at	3.09	2.45		
206306_at	3.09	3.55	ryanodine receptor 3	RYR3
1562283_at	3.08	2.69		
221225_at	3.07	2.33	dephospho-CoA kinase domain containing	DCAKD
1559697_a_at	3.06	2.61		
221538_s_at	3.06	2.36	plexin A1	PLXNA1
241855_s_at	3.06	2.82	cullin 3	CUL3
226806_s_at	3.05	2.32		
239971_at	3.05	2.31	chromosome 16 open reading frame 45	C16orf45
236742_at	3.04	2.23		
242719_at	3.03	3.31		
210714_at	3.02	2.31	R3H domain containing 1	R3HDM1
236630_at	3.02	3.21	aquaporin 2 (collecting duct)	AQP2
244026_at	3.01	2.17	elongation factor, RNA polymerase II, 2	ELL2
236892_s_at	3.00	3.53		
1570639_at	2.99	2.33		
1555547_at	2.97	2.59		
220118_at	2.97	2.43	zinc finger and BTB domain containing 32	ZBTB32
1555284_at	2.96	2.02	amyotrophic lateral sclerosis 2 (juvenile)	ALS2
204416_x_at	2.95	2.38	apolipoprotein C-I	APOC1
219727_at	2.92	5.92	dual oxidase 2	DUOX2
219672_at	2.90	2.64	erythroid associated factor	ERAF
222108_at	2.90	5.97	adhesion molecule with Ig-like domain 2	AMIGO2
244227_at	2.90	2.11	synaptotagmin VI	SYT6
234199_at	2.90	2.13		
216813_at	2.89	2.26		
233321_x_at	2.88	4.54		
235380_at	2.87	2.04		
244865_at	2.87	2.54	HCLS1 associated protein X-1	HAX1

228414_at	2.84	2.14	potassium large conductance calcium-activated channel, subfamily M, alpha member 1	KCNMA1
243720_at	2.84	2.34		
1560418_at	2.82	3.77	chromosome 6 open reading frame 182	C6orf182
227654_at	2.82	2.81	chromosome 20 open reading frame 175	C20orf175
204141_at	2.74	2.49	tubulin, beta 2A	TUBB2A
1552658_a_at	2.73	2.51	neuron navigator 3	NAV3
241430_at	2.73	2.72	chromosome 2 open reading frame 51	C2orf51
202421_at	2.70	3.80	immunoglobulin superfamily, member 3	IGSF3
1567101_at	2.69	2.04		
240254_at	2.68	2.01	TRAF2 and NCK interacting kinase	TNIK
232117_at	2.68	2.02		
242783_at	2.66	2.04		
237982_at	2.65	2.01		
229631_at	2.62	2.35	dynein heavy chain domain 1	DNHD1
228128_x_at	2.61	3.52	pregnancy-associated plasma protein A, pappalysin 1	PAPPA
202729_s_at	2.61	2.10	latent transforming growth factor beta binding protein 1	LTBP1
231880_at	2.60	2.08	family with sequence similarity 40, member B	FAM40B
1570169_at	2.60	2.15	CUB and Sushi multiple domains 2	CSMD2
218950_at	2.60	2.43	centaurin, delta 3	CENTD3
214414_x_at	2.59	2.53	hemoglobin, alpha 1	HBA1
227877_at	2.57	2.44		
204748_at	2.56	3.00	prostaglandin-endoperoxide synthase 2 (prostaglandin G/H synthase and cyclooxygenase)	PTGS2
1569813_at	2.56	2.31	striatin, calmodulin binding protein	STRN
231616_at	2.54	3.00	glycophorin A (MNS blood group)	GYPA
1559276_at	2.54	2.88		
1564438_at	2.53	2.07		
221419_s_at	2.53	2.58		
242142_at	2.52	3.75		
201059_at	2.50	2.08	cortactin	CTTN
228904_at	2.50	2.20	homeobox B3	HOXB3
1557394_at	2.49	2.40		
221071_at	2.49	2.27		
236990_at	2.49	5.88		
241679_at	2.48	2.22	A kinase (PRKA) anchor protein (gravin) 12	AKAP12
1566868_at	2.48	2.02		
239723_at	2.47	2.09		
200999_s_at	2.45	2.20	cytoskeleton-associated protein 4	CKAP4
215635_at	2.45	2.53		
214366_s_at	2.44	2.63	arachidonate 5-lipoxygenase	ALOX5
1569263_at	2.44	6.06		
203216_s_at	2.44	2.72	myosin VI	MYO6
229954_at	2.44	2.38		
240456_at	2.42	2.17		
240175_at	2.42	2.30		
215587_x_at	2.41	2.11		

1555144_at	2.40	2.43		
205857_at	2.38	2.39	solute carrier family 18 (vesicular monoamine), member 2	SLC18A2
218456_at	2.38	2.01	C1q domain containing 1	C1QDC1
217414_x_at	2.36	3.25	hemoglobin, alpha 2	HBA2
239850_at	2.36	2.75		
1557520_a_at	2.35	2.67	transmembrane protein 59	TMEM59
237165_at	2.35	2.07	gelsolin (amyloidosis, Finnish type)	GSN
234968_at	2.35	2.09	DENN/MADD domain containing 4C	DENND4C
240520_at	2.34	3.44	proline rich 8	PRR8
1566424_at	2.29	4.26		
1559037_a_at	2.29	3.80		
233152_x_at	2.29	2.06		
236719_at	2.29	16.71		
239913_at	2.28	7.84	solute carrier family 10 (sodium/bile acid cotransporter family), member 4	SLC10A4
242227_at	2.28	3.12		
240696_at	2.27	3.08		
44783_s_at	2.27	2.83	hairy/enhancer-of-split related with YRPW motif 1	HEY1
223644_s_at	2.26	2.10	crystallin, gamma S	CRYGS
1552825_at	2.25	3.81	zinc finger protein 396	ZNF396
1565877_at	2.25	5.80		
205729_at	2.24	2.06	oncostatin M receptor	OSMR
1570122_at	2.24	3.18		
241851_x_at	2.24	3.25		
1559172_at	2.23	3.09		
1557357_at	2.23	2.48		
220957_at	2.22	2.25	cutaneous T-cell lymphoma-associated antigen 1	CTAGE1
235864_at	2.22	3.19	ATPase, H+ transporting, lysosomal 34kDa, V1 subunit D	ATP6V1D
207067_s_at	2.21	2.64	histidine decarboxylase	HDC
211745_x_at	2.19	3.32	hemoglobin, alpha 2	HBA2
1556798_a_at	2.18	2.36		
242197_x_at	2.18	2.47	CD36 molecule (thrombospondin receptor)	CD36
204217_s_at	2.17	9.47	reticulon 2	RTN2
240768_x_at	2.17	2.26		
216956_s_at	2.16	2.28	integrin, alpha 2b (platelet glycoprotein IIb of IIb/IIIa complex, antigen CD41)	ITGA2B
1552522_at	2.16	16.53	tigger transposable element derived 4	TIGD4
225767_at	2.16	2.66		
209458_x_at	2.15	3.11	hemoglobin, alpha 1	HBA1
232158_x_at	2.15	2.10	NIPA-like domain containing 1	NPAL1
229854_at	2.15	2.47	obscurin, cytoskeletal calmodulin and titin-interacting RhoGEF	OBSCN
1559105_at	2.15	7.81		
237181_at	2.15	2.87	protein phosphatase 2, regulatory subunit B', gamma isoform	PPP2R5C
210215_at	2.15	2.43	transferrin receptor 2	TFR2
211699_x_at	2.14	3.12	hemoglobin, alpha 1	HBA1
1569882_at	2.14	14.12		

238301_at	2.12	3.02		
228309_at	2.12	2.03	chromosome 20 open reading frame 3	C20orf3
217619_x_at	2.12	2.29		
218033_s_at	2.12	2.24	stannin	SNN
209926_at	2.11	2.54	MADS box transcription enhancer factor 2, polypeptide B (myocyte enhancer factor 2B)	MEF2B
214920_at	2.11	2.66	thrombospondin, type I, domain containing 7A	THSD7A
208557_at	2.11	2.14	homeobox A6	HOXA6
230252_at	2.10	2.56	G protein-coupled receptor 92	GPR92
211123_at	2.10	3.74	solute carrier family 5 (sodium iodide symporter), member 5	SLC5A5
1556359_at	2.10	2.61	chromosome 6 open reading frame 89	C6orf89
215907_at	2.09	2.19		
216248_s_at	2.08	2.37	nuclear receptor subfamily 4, group A, member 2	NR4A2
209716_at	2.08	2.05	colony stimulating factor 1 (macrophage)	CSF1
244697_at	2.08	2.28	zinc finger and BTB domain containing 16	ZBTB16
234428_at	2.07	16.00		
1560706_at	2.07	2.19		
236338_at	2.07	2.99	insulin receptor substrate 2	IRS2
205266_at	2.07	2.14	leukemia inhibitory factor (cholinergic differentiation factor)	LIF
215439_x_at	2.07	2.00	synaptopodin 2	SYNPO2
229057_at	2.06	7.11	sodium channel, voltage-gated, type II, alpha subunit	SCN2A
202478_at	2.05	2.13	tribbles homolog 2 (Drosophila)	TRIB2
240450_at	2.04	2.16		
204018_x_at	2.03	2.55	hemoglobin, alpha 1	HBA1
226211_at	2.03	3.01	maternally expressed 3	MEG3
1563469_at	2.01	2.07		
1558969_a_at	2.01	2.50	ribosomal protein L32 pseudogene 3	RPL32P3
202175_at	2.01	2.26		
1563884_at	2.01	3.54		
211005_at	2.00	2.40	linker for activation of T cells	LAT
1556942_at	2.00	2.71		
1555505_a_at	2.00	2.26	tyrosinase (oculocutaneous albinism IA)	TYR
225271_at	2.00	2.25	transmembrane protein 63B	TMEM63B
1570257_x_at	-2.01	-2.59		
238790_at	-2.01	-2.96		
214995_s_at	-2.02	-2.04	apolipoprotein B mRNA editing enzyme, catalytic polypeptide-like 3F	APOBEC3F
211631_x_at	-2.03	-8.75	UDP-Gal:betaGlcNAc beta 1,4-galactosyltransferase, polypeptide 1	B4GALT1
213887_s_at	-2.06	-2.41	polymerase (RNA) II (DNA directed) polypeptide E, 25kDa	POLR2E
1564962_at	-2.06	-23.03	zinc finger protein 92	ZNF92
243619_at	-2.07	-5.06	FGFR1 oncogene partner 2	FGFR1OP2
209034_at	-2.07	-2.46	proline-rich nuclear receptor coactivator 1	PNRC1

207079_s_at	-2.07	-2.17	mediator of RNA polymerase II transcription, subunit 6 homolog (S. cerevisiae)	MED6
226748_at	-2.08	-2.01	LysM, putative peptidoglycan-binding, domain containing 2	LYSMD2
238483_at	-2.08	-2.09		
225468_at	-2.09	-2.20		
202912_at	-2.10	-2.37	adrenomedullin	ADM
1554341_a_at	-2.10	-3.74		
201226_at	-2.11	-2.35	NADH dehydrogenase (ubiquinone) 1 beta subcomplex, 8, 19kDa	NDUFB8
242755_at	-2.11	-3.87	SFRS protein kinase 2	SRPK2
219093_at	-2.11	-2.41	phosphotyrosine interaction domain containing 1	PID1
220051_at	-2.11	-6.10	protease, serine, 21 (testisin)	PRSS21
223698_at	-2.12	-2.05	solute carrier family 25, member 36	SLC25A36
202810_at	-2.13	-2.12	developmentally regulated GTP binding protein 1	DRG1
215646_s_at	-2.14	-2.55	chondroitin sulfate proteoglycan 2 (versican)	CSPG2
226220_at	-2.14	-3.45	methyltransferase like 9	METTL9
224918_x_at	-2.15	-2.20	microsomal glutathione S-transferase 1	MGST1
201506_at	-2.15	-2.12	transforming growth factor, beta-induced, 68kDa	TGFBI
239465_at	-2.16	-2.03		
235321_at	-2.16	-2.07		
230715_at	-2.17	-2.22		
201669_s_at	-2.18	-2.50	myristoylated alanine-rich protein kinase C substrate	MARCKS
244413_at	-2.18	-2.37		
243299_at	-2.19	-3.08		
224387_at	-2.19	-3.62	COMM domain containing 5	COMMD5
201324_at	-2.21	-2.13	epithelial membrane protein 1	EMP1
202968_s_at	-2.22	-2.42	dual-specificity tyrosine-(Y)-phosphorylation regulated kinase 2	DYRK2
236350_at	-2.24	-2.76		
241733_at	-2.25	-2.14	chromosome 18 open reading frame 54	C18orf54
208140_s_at	-2.27	-6.98	leucine rich repeat containing 48	LRRC48
1553108_at	-2.29	-2.39	chromosome 5 open reading frame 24	C5orf24
205419_at	-2.30	-3.73	Epstein-Barr virus induced gene 2 (lymphocyte-specific G protein-coupled receptor)	EBI2
1557167_at	-2.35	-4.06	HLA complex group 11	HCG11
236365_at	-2.36	-2.15	alpha-methylacyl-CoA racemase	AMACR
234512_x_at	-2.36	-2.02		
227839_at	-2.36	-3.33	methyl-CpG binding domain protein 5	MBD5
212595_s_at	-2.37	-2.38	DAZ associated protein 2	DAZAP2
211571_s_at	-2.38	-2.85	chondroitin sulfate proteoglycan 2 (versican)	CSPG2
222773_s_at	-2.38	-3.26	UDP-N-acetyl-alpha-D-galactosamine:polypeptide N-acetylgalactosaminyltransferase 12 (GalNAc-T12)	GALNT12

231736_x_at	-2.40	-2.82	microsomal glutathione S-transferase 1	MGST1
214959_s_at	-2.42	-2.02	apoptosis inhibitor 5	API5
224356_x_at	-2.44	-2.10	membrane-spanning 4-domains, subfamily A, member 6A	MS4A6A
1555829_at	-2.45	-2.93	family with sequence similarity 62 (C2 domain containing) member B	FAM62B
205023_at	-2.45	-2.33	RAD51 homolog (RecA homolog, E. coli) (S. cerevisiae)	RAD51
227265_at	-2.47	-2.80	fibrinogen-like 2	FGL2
204834_at	-2.48	-2.02	fibrinogen-like 2	FGL2
202593_s_at	-2.48	-2.21		
201971_s_at	-2.48	-2.10	ATPase, H+ transporting, lysosomal 70kDa, V1 subunit A	ATP6V1A
1557169_x_at	-2.49	-6.53	HLA complex group 11	HCG11
1552481_s_at	-2.50	-2.30	mannosidase, alpha, class 1A, member 2	MAN1A2
201619_at	-2.50	-2.11	peroxiredoxin 3	PRDX3
221786_at	-2.50	-2.24	chromosome 6 open reading frame 120	C6orf120
228855_at	-2.51	-5.57	nudix (nucleoside diphosphate linked moiety X)-type motif 7	NUDT7
218036_x_at	-2.51	-2.31	NMD3 homolog (S. cerevisiae)	NMD3
222559_s_at	-2.51	-3.09		
222071_s_at	-2.53	-3.44	solute carrier organic anion transporter family, member 4C1	SLCO4C1
236035_at	-2.53	-2.98		
233421_s_at	-2.53	-4.52	nucleoporin 133kDa	NUP133
225070_at	-2.55	-3.10	nuclear undecaprenyl pyrophosphate synthase 1 homolog (S. cerevisiae)	NUS1
206978_at	-2.57	-2.24	chemokine (C-C motif) receptor 2	CCR2
202978_s_at	-2.58	-2.02	CREB/ATF bZIP transcription factor	CREBZF
243529_at	-2.58	-2.27	methionine-tRNA synthetase 2 (mitochondrial)	MARS2
233465_at	-2.60	-29.44		
238469_at	-2.60	-2.17		
233011_at	-2.60	-7.05	annexin A1	ANXA1
238010_at	-2.60	-2.14	chromosome 1 open reading frame 174	C1orf174
225647_s_at	-2.61	-2.35	cathepsin C	CTSC
202586_at	-2.66	-4.02	polymerase (RNA) II (DNA directed) polypeptide L, 7.6kDa	POLR2L
212418_at	-2.70	-2.10	E74-like factor 1 (ets domain transcription factor)	ELF1
218498_s_at	-2.73	-2.38	ERO1-like (S. cerevisiae)	ERO1L
1556325_at	-2.73	-5.16	filamin A interacting protein 1	FILIP1
219553_at	-2.73	-2.17	non-metastatic cells 7, protein expressed in (nucleoside-diphosphate kinase)	NME7
230834_at	-2.75	-23.88		
209269_s_at	-2.77	-2.29		
1569539_at	-2.77	-2.93		
225580_at	-2.80	-2.71	mitochondrial ribosomal protein L50	MRPL50
224560_at	-2.81	-2.12	TIMP metallopeptidase inhibitor 2	TIMP2
219014_at	-2.83	-3.10	placenta-specific 8	PLAC8
229083_at	-2.83	-2.04		
1557905_s_at	-2.83	-2.41	CD44 molecule (Indian blood group)	CD44

219797_at	-2.84	-2.71	mannosyl (alpha-1,3-)-glycoprotein beta-1,4-N-acetylglucosaminyltransferase, isozyme A	MGAT4A
220550_at	-2.84	-4.18	F-box protein 4	FBXO4
220477_s_at	-2.84	-2.03	chromosome 20 open reading frame 30	C20orf30
219837_s_at	-2.85	-2.00	cytokine-like 1	CYTL1
239170_at	-2.87	-2.07		
1568673_s_at	-2.88	-7.50	ELL associated factor 2	EAF2
226142_at	-2.88	-2.11	GLI pathogenesis-related 1 (glioma)	GLIPR1
215397_x_at	-2.89	-2.46		
211061_s_at	-2.92	-2.48	mannosyl (alpha-1,6-)-glycoprotein beta-1,2-N-acetylglucosaminyltransferase	MGAT2
209645_s_at	-2.92	-4.51	aldehyde dehydrogenase 1 family, member B1	ALDH1B1
244171_at	-2.94	-13.07	muskelin 1, intracellular mediator containing kelch motifs	MKLN1
221695_s_at	-2.94	-2.47	mitogen-activated protein kinase kinase kinase 2	MAP3K2
214336_s_at	-2.95	-2.11	coatomer protein complex, subunit alpha	COPA
214277_at	-3.00	-3.61	COX11 homolog, cytochrome c oxidase assembly protein (yeast)	COX11
218172_s_at	-3.00	-2.14	Der1-like domain family, member 1	DERL1
203888_at	-3.04	-4.06	thrombomodulin	THBD
204472_at	-3.04	-2.51	GTP binding protein overexpressed in skeletal muscle	GEM
211189_x_at	-3.08	-2.58	CD84 molecule	CD84
238562_at	-3.08	-2.37	chromosome 8 open reading frame 53	C8orf53
244716_x_at	-3.11	-2.00	transmembrane and immunoglobulin domain containing 2	TMIGD2
212560_at	-3.12	-2.17	sortilin-related receptor, L(DLR class) A repeats-containing	SORL1
205841_at	-3.21	-2.40	Janus kinase 2 (a protein tyrosine kinase)	JAK2
205048_s_at	-3.24	-2.29	phosphoserine phosphatase	PSPH
201554_x_at	-3.31	-2.24	glycogenin 1	GYG1
200986_at	-3.36	-11.70	serpin peptidase inhibitor, clade G (C1 inhibitor), member 1, (angioedema, hereditary)	SERPING1
1562387_at	-3.40	-2.30		
213747_at	-3.41	-9.42	antizyme inhibitor 1	AZIN1
208894_at	-3.43	-3.20	major histocompatibility complex, class II, DR alpha	HLA-DRA
202917_s_at	-3.43	-3.32	S100 calcium binding protein A8	S100A8
1555639_a_at	-3.49	-2.14	RNA binding motif protein 14	RBM14
201309_x_at	-3.51	-2.06	chromosome 5 open reading frame 13	C5orf13
200769_s_at	-3.52	-2.80	methionine adenosyltransferase II, alpha	MAT2A
1565162_s_at	-3.61	-2.49	microsomal glutathione S-transferase 1	MGST1
209406_at	-3.62	-2.08	BCL2-associated athanogene 2	BAG2
228485_s_at	-3.68	-2.31	solute carrier family 44, member 1	SLC44A1
210788_s_at	-3.72	-2.29	dehydrogenase/reductase (SDR family) member 7	DHRS7
231152_at	-3.81	-3.12		
243495_s_at	-3.88	-2.75		
225408_at	-3.92	-5.25	myelin basic protein	MBP

223501_at	-4.10	-2.22		
200796_s_at	-4.12	-20.18	myeloid cell leukemia sequence 1 (BCL2-related)	MCL1
235202_x_at	-4.14	-2.51		
211363_s_at	-4.49	-3.17	methylthioadenosine phosphorylase	MTAP
233878_s_at	-4.51	-2.27	5'-3' exoribonuclease 2	XRN2
238443_at	-4.52	-3.32		
229128_s_at	-4.65	-2.21	acidic (leucine-rich) nuclear phosphoprotein 32 family, member E	ANP32E
216591_s_at	-4.69	-2.46	succinate dehydrogenase complex, subunit C, integral membrane protein, 15kDa	SDHC
223750_s_at	-5.17	-6.79	toll-like receptor 10	TLR10
209683_at	-5.33	-2.18	family with sequence similarity 49, member A	FAM49A
206512_at	-5.41	-2.78	zinc finger (CCCH type), RNA-binding motif and serine/arginine rich 1	ZRSR1
206710_s_at	-5.78	-2.81	erythrocyte membrane protein band 4.1-like 3	EPB41L3
241245_at	-5.91	-6.83	splicing factor, arginine/serine-rich 4	SFRS4
213872_at	-6.03	-2.10	chromosome 6 open reading frame 62	C6orf62
230659_at	-6.08	-2.38		
235118_at	-6.56	-13.81		
215784_at	-6.89	-3.49	CD1e molecule	CD1E
1558177_at	-7.38	-23.26	chromosome 14 open reading frame 83	C14orf83
231628_s_at	-7.66	-5.35		
1555745_a_at	-7.92	-2.81	lysozyme (renal amyloidosis)	LYZ
242426_at	-8.35	-3.91	neuregulin 4	NRG4
1555340_x_at	-11.80	-12.43	RAP1A, member of RAS oncogene family	RAP1A
212667_at	-12.10	-3.58	secreted protein, acidic, cysteine-rich (osteonectin)	SPARC
1555339_at	-12.29	-12.35	RAP1A, member of RAS oncogene family	RAP1A
240161_s_at	-13.98	-6.03	cell division cycle 20 homolog B (S. cerevisiae)	CDC20B
236459_at	-14.73	-6.39	protein kinase C, epsilon	PRKCE
