# Supplementary material for: In Vitro Transformation of Primary Human CD34+ Cells by AML Fusion Oncogenes: Early Gene Expression Profiling Reveals Possible Drug Target in AML
Source: PLoS One. 2010 Aug 27;5(8):e12464. doi: 10.1371/journal.pone.0012464 (PMC2929205; doi:10.1371/journal.pone.0012464)
Supplement: Table S9 — Genes deregulated by MLL-AF9 8 days after transduction. Primary human CD34+ cells were retrovirally transduced with either control MSCV-IRES-GFP vector or vector expressing MLL-AF9 and sorted for GFP positivity. Total RNA was extracted 8 days after transduction and subjected to microarray analysis. Genes that showed up- or down-regulation by 2 fold or more in comparison to the control in 2 independent experiments (Exp.1 and Exp.2) were considered deregulated. (0.10 MB PDF) [file pone.0012464.s009.pdf]

Table S9. Genes deregulated by MLL-AF9 at 8 d after transduction

Probe set ID	Fold Change Exp.1	Fold Change Exp.2	Gene Name	Gene Symbol
243489_at	27.22	8.16		
219737_s_at	22.00	5.50	protocadherin 9	PCDH9
235955_at	21.38	2.09	MARVEL domain containing 2	MARVELD2
AFFX-HUMRGE/M10098_5_at	12.75	8.89		
1554619_at	12.58	14.21		
1565436_s_at	10.41	4.99	myeloid/lymphoid or mixed-lineage leukemia (trithorax homolog, Drosophila)	MLL
212079_s_at	10.06	3.98	myeloid/lymphoid or mixed-lineage leukemia (trithorax homolog, Drosophila)	MLL
228904_at	9.66	2.90	homeobox B3	HOXB3
226677_at	9.46	15.62	zinc finger protein 521	ZNF521
213844_at	8.12	3.62	homeobox A5	HOXA5
230237_at	7.83	27.44		
235521_at	7.54	3.29	homeobox A3	HOXA3
222330_at	7.44	4.33		
212078_s_at	7.12	3.71	myeloid/lymphoid or mixed-lineage leukemia (trithorax homolog, Drosophila)	MLL
244508_at	6.77	2.79	septin 7	7-Sep
215674_at	6.63	2.07		
214414_x_at	6.35	5.37	hemoglobin, alpha 1	HBA1
229706_at	5.74	2.24	transcription elongation regulator 1	TCERG1
217414_x_at	5.15	5.60	hemoglobin, alpha 2	HBA2
205857_at	5.06	2.63	solute carrier family 18 (vesicular monoamine), member 2	SLC18A2
215296_at	4.93	3.64	CDC42 binding protein kinase alpha (DMPK-like)	CDC42BPA
209881_s_at	4.76	3.02	linker for activation of T cells	LAT
240768_x_at	4.61	2.04		
213348_at	4.53	3.56	cyclin-dependent kinase inhibitor 1C (p57, Kip2)	CDKN1C
210230_at	4.36	2.02		
242172_at	4.29	2.65	Meis1, myeloid ecotropic viral integration site 1 homolog (mouse)	MEIS1
241184_x_at	4.17	3.38	zinc finger protein 407	ZNF407
206834_at	4.15	9.71	hemoglobin, delta	HBD
206145_at	3.90	6.00	Rh-associated glycoprotein	RHAG
205608_s_at	3.90	2.05	angiopoietin 1	ANGPT1
231599_x_at	3.84	5.23	D4, zinc and double PHD fingers family 1	DPF1
205767_at	3.82	4.37	epiregulin	EREG
237009_at	3.80	2.26	CD69 molecule	CD69
236738_at	3.79	3.38		
205609_at	3.77	6.60	angiopoietin 1	ANGPT1
209905_at	3.69	2.39	homeobox A9	HOXA9
237502_at	3.69	3.48	cardiolipin synthase 1	CRLS1
203373_at	3.67	3.53	suppressor of cytokine signaling 2	SOCS2

206067_s_at	3.65	2.66	Wilms tumor 1	WT1
203372_s_at	3.62	3.44	suppressor of cytokine signaling 2	SOCS2
204720_s_at	3.61	5.08	DnaJ (Hsp40) homolog, subfamily C, member 6	DNAJC6
1553328_a_at	3.54	2.83	solute carrier family 18 (vesicular monoamine), member 2	SLC18A2
209458_x_at	3.51	4.05	hemoglobin, alpha 1	HBA1
211748_x_at	3.47	3.56	prostaglandin D2 synthase 21kDa (brain)	PTGDS
226189_at	3.44	4.08	integrin, beta 8	ITGB8
1570251_at	3.40	2.06	HECT domain containing 1	HECTD1
235086_at	3.39	6.85	thrombospondin 1	THBS1
244443_at	3.39	3.38	chromodomain helicase DNA binding protein 2	CHD2
238831_at	3.25	2.84		
214146_s_at	3.24	3.30	pro-platelet basic protein (chemokine (C-X-C motif) ligand 7)	PPBP
207911_s_at	3.24	2.03	transglutaminase 5	TGM5
235753_at	3.24	3.41	homeobox A7	HOXA7
213515_x_at	3.17	4.81	hemoglobin, gamma G	HBG2
213894_at	3.16	2.83	thrombospondin, type I, domain containing 7A	THSD7A
201110_s_at	3.15	4.50	thrombospondin 1	THBS1
208711_s_at	3.14	6.73	cyclin D1	CCND1
239167_at	3.07	2.03		
1552908_at	3.05	3.96	chromosome 1 open reading frame 150	C1orf150
205848_at	3.02	3.13	growth arrest-specific 2	GAS2
236645_at	3.02	2.99	HMG-box transcription factor 1	HBP1
221024_s_at	2.99	2.87	solute carrier family 2 (facilitated glucose transporter), member 10	SLC2A10
206026_s_at	2.98	2.61	tumor necrosis factor, alpha-induced protein 6	TNFAIP6
211745_x_at	2.93	4.98	hemoglobin, alpha 2	HBA2
236237_at	2.92	2.80	KIAA1370	KIAA1370
206954_at	2.91	2.21	Wilms tumor upstream neighbor 1	WIT1
237354_at	2.90	14.98		
1563453_at	2.87	2.80		
228708_at	2.86	4.01		
201418_s_at	2.86	2.06	SRY (sex determining region Y)-box 4	SOX4
204419_x_at	2.81	5.60	hemoglobin, gamma G	HBG2
241099_at	2.81	2.98	elongation protein 4 homolog (S. cerevisiae)	ELP4
211699_x_at	2.81	3.81	hemoglobin, alpha 1	HBA1
228250_at	2.81	2.56	folliculin interacting protein 1	FNIP1
206847_s_at	2.80	3.15	homeobox A7	HOXA7
208719_s_at	2.77	2.18	DEAD (Asp-Glu-Ala-Asp) box polypeptide 17	DDX17
207125_at	2.76	2.25	zinc finger protein 225	ZNF225
204018_x_at	2.73	5.44	hemoglobin, alpha 1	HBA1
204779_s_at	2.73	2.60	homeobox B7	HOXB7
207480_s_at	2.71	2.11	Meis1, myeloid ecotropic viral integration site 1 homolog 2 (mouse)	MEIS2
210504_at	2.67	2.36	Kruppel-like factor 1 (erythroid)	KLF1
204141_at	2.63	3.66	tubulin, beta 2A	TUBB2A

208712_at	2.61	5.95	cyclin D1	CCND1
213927_at	2.61	3.90	mitogen-activated protein kinase kinase kinase 9	MAP3K9
216459_x_at	2.60	2.14		
201150_s_at	2.59	3.24	TIMP metallopeptidase inhibitor 3 (Sorsby fundus dystrophy, pseudoinflammatory)	TIMP3
244181_at	2.58	3.28	phosphoinositide-3-kinase, regulatory subunit 1 (p85 alpha)	PIK3R1
222341_x_at	2.57	2.14		
236023_at	2.52	2.69		
204304_s_at	2.51	2.44	prominin 1	PROM1
217867_x_at	2.49	2.07	beta-site APP-cleaving enzyme 2	BACE2
225115_at	2.49	2.26	homeodomain interacting protein kinase 2	HIPK2
220234_at	2.48	2.18	carbonic anhydrase VIII	CA8
207526_s_at	2.47	2.26	interleukin 1 receptor-like 1	IL1RL1
202806_at	2.47	2.03	drebrin 1	DBN1
226234_at	2.45	4.49		
233007_at	2.44	2.07		
223079_s_at	2.44	2.06	glutaminase	GLS
210358_x_at	2.43	2.17	GATA binding protein 2	GATA2
236546_at	2.41	2.35		
1561763_at	2.40	2.91		
236757_at	2.39	2.09		
204256_at	2.37	3.29	ELOVL family member 6, elongation of long chain fatty acids (FEN1/Elo2, SUR4/Elo3-like, yeast)	ELOVL6
227051_at	2.34	5.40		
201655_s_at	2.33	2.31	heparan sulfate proteoglycan 2 (perlecan)	HSPG2
217025_s_at	2.30	2.38	drebrin 1	DBN1
228749_at	2.29	2.31		
214020_x_at	2.29	2.65	integrin, beta 5	ITGB5
222446_s_at	2.29	2.01	beta-site APP-cleaving enzyme 2	BACE2
207329_at	2.28	2.10	matrix metallopeptidase 8 (neutrophil collagenase)	MMP8
223766_at	2.28	2.22		
244267_at	2.27	3.60	special AT-rich sequence binding protein 1 (binds to nuclear matrix/scaffold-associating DNA's)	SATB1
209710_at	2.27	2.17	GATA binding protein 2	GATA2
236484_at	2.27	2.08		
228915_at	2.27	3.75	dachshund homolog 1 (Drosophila)	DACH1
1558202_at	2.26	4.60		
235852_at	2.24	2.97	stonin 2	STON2
227155_at	2.23	2.75		
228696_at	2.21	2.24	solute carrier family 45, member 3	SLC45A3
242248_at	2.21	2.04	phosphorylase kinase, beta	PHKB
225308_s_at	2.21	2.25	tetratricopeptide repeat, ankyrin repeat and coiled-coil containing 1	TANC1
1562947_x_at	2.20	2.42		
240773_at	2.19	2.54		
205472_s_at	2.19	3.80	dachshund homolog 1 (Drosophila)	DACH1
211828_s_at	2.18	2.15	TRAF2 and NCK interacting kinase	TNIK
241298_x_at	2.18	2.38		

241402_at	2.18	2.26	tRNA splicing endonuclease 54 homolog (S. cerevisiae)	TSEN54
231798_at	2.17	2.67	noggin	NOG
216860_s_at	2.16	2.13	growth differentiation factor 11	GDF11
242232_at	2.13	2.14	SLIT-ROBO Rho GTPase activating protein 2	SRGAP2
226751_at	2.13	2.23	chromosome 2 open reading frame 32	C2orf32
241453_at	2.12	6.33	PTK2 protein tyrosine kinase 2	PTK2
1558887_at	2.11	2.50		
204416_x_at	2.10	22.39	apolipoprotein C-I	APOC1
233912_x_at	2.08	2.46		
1552430_at	2.08	13.25	WD repeat domain 17	WDR17
242903_at	2.07	2.19	interferon gamma receptor 1	IFNGR1
234306_s_at	2.07	2.65	SLAM family member 7	SLAMF7
1565951_s_at	2.06	2.82	choroideremia-like (Rab escort protein 2)	CHML
227740_at	2.06	2.20	U2AF homology motif (UHM) kinase 1	UHMK1
243618_s_at	2.05	2.08		
206283_s_at	2.05	2.96	T-cell acute lymphocytic leukemia 1	TAL1
243361_at	2.04	2.38	splicing factor, arginine/serine-rich 12	SFRS12
233191_at	2.04	3.10	RUN and FYVE domain containing 2	RUFY2
204187_at	2.03	2.42	guanosine monophosphate reductase	GMPR
1565877_at	2.02	21.31		
238569_at	-2.01	-9.08	gamma-aminobutyric acid (GABA) B receptor, 1	GABBR1
243370_at	-2.01	-2.04	GPI-anchored membrane protein 1	GPIAP1
237891_at	-2.02	-27.88	Mdm2, transformed 3T3 cell double minute 2, p53 binding protein (mouse)	MDM2
1562537_at	-2.03	-3.60	Fc fragment of IgE, high affinity I, receptor for; alpha polypeptide	FCER1A
205403_at	-2.04	-2.56	interleukin 1 receptor, type II	IL1R2
238579_at	-2.04	-8.89	chromosome 9 open reading frame 85	C9orf85
242445_at	-2.07	-3.06	FYVE, RhoGEF and PH domain containing 4	FGD4
211106_at	-2.08	-2.05	suppressor of Ty 3 homolog (S. cerevisiae)	SUPT3H
221643_s_at	-2.09	-2.51	arginine-glutamic acid dipeptide (RE) repeats	RERE
241188_at	-2.10	-2.67		
236310_at	-2.12	-2.08		
1561155_at	-2.14	-2.28		
226425_at	-2.18	-2.28	CAP-GLY domain containing linker protein family, member 4	CLIP4
235685_at	-2.19	-2.30		
230324_at	-2.19	-2.13	nuclear receptor coactivator 2	NCOA2
1556338_at	-2.19	-2.21		
1558603_at	-2.19	-3.29	plasminogen-like B2	PLGLB2
212963_at	-2.19	-2.13	TM2 domain containing 1	TM2D1
219383_at	-2.28	-2.14		
243196_s_at	-2.31	-2.20	TRAF-type zinc finger domain containing 1	TRAFD1
214647_s_at	-2.34	-2.94	hemochromatosis	HFE
206818_s_at	-2.34	-2.41	cyclin M2	CNNM2
1556339_a_at	-2.36	-2.17		
242755_at	-2.42	-27.37	SFRS protein kinase 2	SRPK2

240338_at	-2.43	-2.66		
209396_s_at	-2.46	-3.76	chitinase 3-like 1 (cartilage glycoprotein-39)	CHI3L1
1559376_at	-2.47	-3.13	chromosome 1 open reading frame 203	C1orf203
213849_s_at	-2.49	-2.01	protein phosphatase 2 (formerly 2A), regulatory subunit B, beta isoform	PPP2R2B
233239_at	-2.49	-3.13		
43934_at	-2.53	-2.39	G protein-coupled receptor 137	GPR137
1561726_s_at	-2.56	-2.94		
242726_at	-2.56	-2.16		
241732_at	-2.60	-2.09	CDK5 regulatory subunit associated protein 1-like 1	CDKAL1
240191_at	-2.60	-2.45	zinc finger protein 543	ZNF543
241321_at	-2.62	-2.06	ankyrin repeat domain 23	ANKRD23
225987_at	-2.62	-2.57	STEAP family member 4	STEAP4
244710_at	-2.68	-2.91	leucine-rich repeats and guanylate kinase domain containing	LRGUK
233455_at	-2.71	-3.14		
231193_s_at	-2.81	-2.65		
203457_at	-2.88	-2.05	syntaxin 7	STX7
242654_at	-2.94	-3.41	Fanconi anemia, complementation group C	FANCC
1569827_at	-2.94	-2.10	ATG7 autophagy related 7 homolog (S. cerevisiae)	ATG7
243515_at	-3.01	-2.39		
234210_x_at	-3.01	-2.23	ARP2 actin-related protein 2 homolog (yeast)	ACTR2
207826_s_at	-3.02	-3.18	inhibitor of DNA binding 3, dominant negative helix-loop-helix protein	ID3
224321_at	-3.03	-62.44	transmembrane protein with EGF-like and two follistatin-like domains 2	TMEFF2
220399_at	-3.06	-2.08		
218850_s_at	-3.31	-2.75	LIM domains containing 1	LIMD1
238871_at	-3.44	-28.38	myeloid/lymphoid or mixed-lineage leukemia (trithorax homolog, Drosophila); translocated to, 4	MLLT4
221419_s_at	-3.67	-3.42		
214453_s_at	-3.75	-3.35	interferon-induced protein 44	IFI44
243185_at	-3.81	-3.26		
220266_s_at	-4.13	-2.03	Kruppel-like factor 4 (gut)	KLF4
233207_at	-4.27	-2.51	disrupted in schizophrenia 1	DISC1
203549_s_at	-4.53	-2.25	lipoprotein lipase	LPL
209863_s_at	-5.70	-2.08	tumor protein p73-like	TP73L
238320_at	-5.76	-2.91		
228376_at	-6.02	-2.32	glycoprotein, alpha-galactosyltransferase 1	GGTA1
223595_at	-6.53	-3.28	transmembrane protein 133	TMEM133
223889_at	-7.44	-2.11		
1555340_x_at	-7.64	-12.10	RAP1A, member of RAS oncogene family	RAP1A
1570176_at	-7.74	-2.40		
238379_x_at	-11.95	-6.35		
1555339_at	-12.12	-6.62	RAP1A, member of RAS oncogene family	RAP1A
