# Supplementary material for: In Vitro Transformation of Primary Human CD34+ Cells by AML Fusion Oncogenes: Early Gene Expression Profiling Reveals Possible Drug Target in AML
Source: PLoS One. 2010 Aug 27;5(8):e12464. doi: 10.1371/journal.pone.0012464 (PMC2929205; doi:10.1371/journal.pone.0012464)
Supplement: Table S10 — Genes deregulated by NUP98-HOXA9 6 h after transfection. Primary human CD34+ cells were nucleofected with either control pTracer-CMV/Bsd vector or vector expressing NUP98-HOXA9 and sorted for GFP positivity. Total RNA was extracted 6 h after nucleofection and subjected to microarray analysis. Genes that showed up- or down-regulation by 2 fold or more in comparison to the control in 2 independent experiments (Exp.1 and Exp.2) were considered deregulated. (0.11 MB PDF) [file pone.0012464.s010.pdf]

Table S10. Genes deregulated by NUP98-HOXA9 at 6 h after transfection

Probe set ID	Fold Change Exp.1	Fold Change Exp.2	Gene Name	Gene Symbol
235885_at	281.72	4.51		
1562270_at	47.05	2.58	Rho guanine nucleotide exchange factor (GEF) 7	ARHGEF7
204988_at	25.79	2.36	fibrinogen beta chain	FGB
227725_at	25.49	3.01	ST6 (alpha-N-acetyl-neuraminyl-2,3-beta-galactosyl-1,3)-N-acetylgalactosaminide alpha-2,6-sialyltran	ST6GALNAC1
207147_at	25.03	37.98	distal-less homeobox 2	DLX2
1556609_at	21.75	10.09		
207729_at	20.25	4.06	cadherin 9, type 2 (T1-cadherin)	CDH9
229057_at	18.41	3.17	sodium channel, voltage-gated, type II, alpha subunit	SCN2A
219106_s_at	14.01	4.88	kelch repeat and BTB (POZ) domain containing 10	KBTBD10
243792_x_at	12.88	2.63	protein tyrosine phosphatase, non-receptor type 13 (APO-1/CD95 (Fas)-associated phosphatase)	PTPN13
240462_at	12.61	7.93		
1560483_at	12.46	4.81		
1555586_at	12.22	2.13	family with sequence similarity 71, member B	FAM71B
1554712_a_at	11.70	3.37	glycine-N-acyltransferase-like 2	GLYATL2
220784_s_at	11.46	7.94	urotensin 2	UTS2
202551_s_at	10.44	3.90	cysteine rich transmembrane BMP regulator 1 (chordin-like)	CRIM1
206366_x_at	9.63	3.32	chemokine (C motif) ligand 1	XCL1
212611_at	8.92	3.61		
225481_at	8.51	3.10	FERM domain containing 6	FRMD6
1563512_at	7.86	2.43	nitric oxide synthase 1 (neuronal) adaptor protein	NOS1AP
204753_s_at	7.73	3.54	hepatic leukemia factor	HLF
235740_at	6.35	2.05		
204755_x_at	5.86	4.02	hepatic leukemia factor	HLF
244146_at	5.54	12.20	DTW domain containing 1	DTWD1
219295_s_at	5.48	3.78	procollagen C-endopeptidase enhancer 2	PCOLCE2
225464_at	5.29	2.50	FERM domain containing 6	FRMD6
226534_at	5.27	5.18	KIT ligand	KITLG
223204_at	4.97	2.81	chromosome 4 open reading frame 18	C4orf18
239512_at	4.96	2.59	splicing factor, arginine/serine-rich 4	SFRS4
230708_at	4.81	3.03	prickle homolog 1 (Drosophila)	PRICKLE1
236738_at	4.63	3.51		
229147_at	4.54	2.41		
1560957_at	4.52	6.48		
205844_at	4.46	3.07	vanin 1	VNN1
220122_at	4.38	2.73	multiple C2 domains, transmembrane 1	MCTP1

211138_s_at	4.36	3.68	kynurenine 3-monooxygenase (kynurenine 3-hydroxylase)	KMO
1555536_at	4.36	3.12	anthrax toxin receptor 2	ANTXR2
219049_at	4.28	4.54		
222153_at	4.17	2.39	myelin expression factor 2	MYEF2
212230_at	3.96	3.23	phosphatidic acid phosphatase type 2B	PPAP2B
241736_at	3.94	2.53	F-box and WD repeat domain containing 2	FBXW2
222717_at	3.93	3.23	serum deprivation response (phosphatidylserine binding protein)	SDPR
244021_at	3.90	3.18		
211812_s_at	3.86	2.40	beta-1,3-N-acetylgalactosaminyltransferase 1 (globoside blood group)	B3GALNT1
205306_x_at	3.81	3.33	kynurenine 3-monooxygenase (kynurenine 3-hydroxylase)	KMO
234994_at	3.81	3.10	KIAA1913	KIAA1913
239710_at	3.75	2.50		
224202_at	3.65	2.28	suppressor of fused homolog (Drosophila)	SUFU
202609_at	3.64	2.91	epidermal growth factor receptor pathway substrate 8	EPS8
201243_s_at	3.60	2.24	ATPase, Na+/K+ transporting, beta 1 polypeptide	ATP1B1
221884_at	3.56	2.44	ecotropic viral integration site 1	EVI1
212224_at	3.52	2.83	aldehyde dehydrogenase 1 family, member A1	ALDH1A1
228821_at	3.44	5.55	ST6 beta-galactosamide alpha-2,6-sialyltranferase 2	ST6GAL2
218711_s_at	3.40	3.90	serum deprivation response (phosphatidylserine binding protein)	SDPR
226420_at	3.39	2.22	ecotropic viral integration site 1	EVI1
206504_at	3.39	16.19	cytochrome P450, family 24, subfamily A, polypeptide 1	CYP24A1
223557_s_at	3.39	2.63	transmembrane protein with EGF-like and two follistatin-like domains 2	TMEFF2
235521_at	3.38	2.79	homeobox A3	HOXA3
201739_at	3.38	2.18	serum/glucocorticoid regulated kinase	SGK
204260_at	3.37	3.64	chromogranin B (secretogranin 1)	CHGB
226435_at	3.30	2.70	papilin, proteoglycan-like sulfated glycoprotein	PAPLN
242654_at	3.27	2.64	Fanconi anemia, complementation group C	FANCC
1559214_at	3.25	2.81		
234547_at	3.19	7.92		
214085_x_at	3.17	2.39	KRR1, small subunit (SSU) processome component, homolog (yeast)	KRR1
224856_at	3.13	2.41	FK506 binding protein 5	FKBP5
1554242_a_at	3.12	2.13	coagulation factor C homolog, cochlin (Limulus polyphemus)	COCH
204221_x_at	3.09	3.55	GLI pathogenesis-related 1 (glioma)	GLIPR1
1554783_s_at	3.06	2.60	rho/rac guanine nucleotide exchange factor (GEF) 2	ARHGEF2

1565602_at	3.02	7.22		
242828_at	3.01	2.71	fidgetin	FIGN
1566482_at	3.01	2.76		
236893_at	3.00	2.73		
1558766_at	3.00	3.22		
228977_at	2.97	2.29		
239455_at	2.96	2.41		
209193_at	2.92	2.21	pim-1 oncogene	PIM1
216279_at	2.91	2.08	zinc finger protein 460	ZNF460
211431_s_at	2.90	2.42	TYRO3 protein tyrosine kinase	TYRO3
227195_at	2.89	3.30	zinc finger protein 503	ZNF503
233876_at	2.88	2.69		
235069_at	2.87	4.94	TatD DNase domain containing 3	TATDN3
214596_at	2.86	2.01		
1556209_at	2.84	3.18	C-type lectin domain family 2, member B	CLEC2B
228915_at	2.83	2.45	dachshund homolog 1 (Drosophila)	DACH1
239999_at	2.82	2.66	chromosome 21 open reading frame 34	C21orf34
211742_s_at	2.81	2.27	ecotropic viral integration site 2B	EVI2B
219377_at	2.80	8.20	family with sequence similarity 59, member A	FAM59A
204222_s_at	2.79	4.08	GLI pathogenesis-related 1 (glioma)	GLIPR1
201295_s_at	2.74	2.30	WD repeat and SOCS box-containing 1	WSB1
243055_at	2.73	2.15		
219615_s_at	2.71	2.58	potassium channel, subfamily K, member 5	KCNK5
227210_at	2.70	2.01		
201418_s_at	2.69	2.33	SRY (sex determining region Y)-box 4	SOX4
209795_at	2.68	2.27	CD69 molecule	CD69
217599_s_at	2.68	2.16	MyoD family inhibitor domain containing	MDFIC
236892_s_at	2.66	2.59		
1554449_at	2.63	2.20	mesoderm induction early response 1, family member 3	MIER3
230493_at	2.60	2.15	transmembrane protein 46	TMEM46
204201_s_at	2.60	2.39	protein tyrosine phosphatase, non-receptor type 13 (APO-1/CD95 (Fas)-associated phosphatase)	PTPN13
210145_at	2.59	2.07	phospholipase A2, group IVA (cytosolic, calcium-dependent)	PLA2G4A
211402_x_at	2.57	2.36	nuclear receptor subfamily 6, group A, member 1	NR6A1
1569672_at	2.56	2.70		
220169_at	2.55	2.84	transmembrane protein 156	TMEM156
205746_s_at	2.53	2.13	ADAM metallopeptidase domain 17 (tumor necrosis factor, alpha, converting enzyme)	ADAM17
238756_at	2.53	2.16		
226142_at	2.51	2.60	GLI pathogenesis-related 1 (glioma)	GLIPR1
1557759_at	2.48	2.02		
1553120_at	2.47	2.46	claspin homolog (Xenopus laevis)	CLSPN
237741_at	2.43	2.29	solute carrier family 25, member 36	SLC25A36
212328_at	2.42	2.02		
219737_s_at	2.39	2.21	protocadherin 9	PCDH9
233024_at	2.39	2.24	RAB18, member RAS oncogene family	RAB18

236655_at	2.38	2.31	tumor protein D52	TPD52
232478_at	2.38	2.49		
203549_s_at	2.38	2.73	lipoprotein lipase	LPL
212253_x_at	2.36	5.57	dystonin	DST
1569312_at	2.36	2.07		
1558014_s_at	2.35	2.14	male sterility domain containing 2	MLSTD2
235846_at	2.34	3.29		
205518_s_at	2.34	2.87	cytidine monophosphate-N-acetylneuraminic acid hydroxylase (CMP-N-acetylneuraminate monooxygenase)	CMAH
206674_at	2.32	2.13	fms-related tyrosine kinase 3	FLT3
204082_at	2.31	2.47	pre-B-cell leukemia homeobox 3	PBX3
1559902_at	2.29	2.17	megakaryoblastic leukemia (translocation) 1	MKL1
219941_at	2.27	2.07	transmembrane protein 19	TMEM19
1556568_a_at	2.27	2.26		
238919_at	2.26	2.78		
233011_at	2.25	3.79	annexin A1	ANXA1
238863_x_at	2.23	2.49		
232295_at	2.23	8.51	G elongation factor, mitochondrial 1	GFM1
1559059_s_at	2.22	2.76	zinc finger protein 611	ZNF611
244726_at	2.22	2.71		
228708_at	2.21	2.09		
204517_at	2.19	2.07	peptidylprolyl isomerase C (cyclophilin C)	PPIC
238224_at	2.19	2.04		
1552752_a_at	2.17	2.76	cell adhesion molecule 2	CADM2
234278_at	2.16	5.13	epidermal growth factor receptor pathway substrate 15	EPS15
227484_at	2.15	3.62		
210571_s_at	2.14	2.62	cytidine monophosphate-N-acetylneuraminic acid hydroxylase (CMP-N-acetylneuraminate monooxygenase)	CMAH
241209_at	2.12	4.67	IQ motif and WD repeats 1	IQWD1
1557238_s_at	2.12	2.02		
239429_at	2.12	2.61		
233490_at	2.10	15.84	dynactin 4 (p62)	DCTN4
236435_at	2.10	2.01	zinc finger protein 292	ZNF292
206999_at	2.09	2.18	interleukin 12 receptor, beta 2	IL12RB2
226136_at	2.07	2.84		
233498_at	2.07	2.00	v-erb-a erythroblastic leukemia viral oncogene homolog 4 (avian)	ERBB4
207505_at	2.06	2.52	protein kinase, cGMP-dependent, type II	PRKG2
212820_at	2.06	2.03	Dmx-like 2	DMXL2
212822_at	2.04	3.35	HEG homolog 1 (zebrafish)	HEG1
213844_at	2.02	2.88	homeobox A5	HOXA5
210803_at	2.02	2.98	thioredoxin reductase 2	TXNRD2
1554524_a_at	2.00	3.81	olfactomedin 3	OLFM3
219191_s_at	-2.01	-2.07	bridging integrator 2	BIN2
227651_at	-2.02	-2.13	BTB (POZ) domain containing 14B	BTBD14B

204567_s_at	-2.03	-2.24	ATP-binding cassette, sub-family G (WHITE), member 1	ABCG1
230790_x_at	-2.03	-2.00	checkpoint suppressor 1	CHES1
201645_at	-2.05	-2.50	tenascin C (hexabrachion)	TNC
1563229_at	-2.05	-11.14	deleted in lymphocytic leukemia, 2	DLEU2
209651_at	-2.05	-2.33	transforming growth factor beta 1 induced transcript 1	TGFB1I1
231500_s_at	-2.06	-3.88	bolA homolog 2 (E. coli)	BOLA2
202949_s_at	-2.07	-2.27	four and a half LIM domains 2	FHL2
204014_at	-2.08	-3.28	dual specificity phosphatase 4	DUSP4
226034_at	-2.11	-2.57		
222941_at	-2.11	-2.50		
219648_at	-2.12	-2.01	melanoregulin	MREG
203505_at	-2.12	-2.23	ATP-binding cassette, sub-family A (ABC1), member 1	ABCA1
236861_at	-2.14	-2.19	THO complex 7 homolog (Drosophila)	THOC7
231732_at	-2.15	-2.83	sphingomyelin phosphodiesterase 3, neutral membrane (neutral sphingomyelinase II)	SMPD3
201041_s_at	-2.15	-2.32	dual specificity phosphatase 1	DUSP1
244569_at	-2.18	-2.27		
227855_at	-2.19	-2.26	zinc finger protein 219	ZNF219
242358_at	-2.20	-2.84		
203068_at	-2.20	-2.13	kelch-like 21 (Drosophila)	KLHL21
1568752_s_at	-2.21	-2.82	regulator of G-protein signalling 13	RGS13
205239_at	-2.24	-2.22	amphiregulin (schwannoma-derived growth factor)	AREG
202510_s_at	-2.25	-2.15	tumor necrosis factor, alpha-induced protein 2	TNFAIP2
1562850_at	-2.26	-2.31		
243004_at	-2.26	-2.26		
1555103_s_at	-2.27	-3.44	fibroblast growth factor 7 (keratinocyte growth factor)	FGF7
226084_at	-2.28	-3.33	microtubule-associated protein 1B	MAP1B
1556393_at	-2.28	-2.79	zinc finger protein 451	ZNF451
206683_at	-2.28	-2.28	zinc finger protein 165	ZNF165
218330_s_at	-2.29	-2.15	neuron navigator 2	NAV2
205242_at	-2.29	-15.45	chemokine (C-X-C motif) ligand 13 (B-cell chemoattractant)	CXCL13
227200_at	-2.31	-2.24		
242999_at	-2.32	-3.08	Rho guanine nucleotide exchange factor (GEF) 7	ARHGEF7
205567_at	-2.34	-3.13	carbohydrate (keratan sulfate Gal-6) sulfotransferase 1	CHST1
237753_at	-2.35	-2.39		
211889_x_at	-2.41	-3.60	carcinoembryonic antigen-related cell adhesion molecule 1 (biliary glycoprotein)	CEACAM1
220148_at	-2.41	-2.17	aldehyde dehydrogenase 8 family, member A1	ALDH8A1
239983_at	-2.41	-2.10	solute carrier family 30 (zinc transporter), member 8	SLC30A8
1563357_at	-2.42	-2.04		

1567997_x_at	-2.43	-2.55		
210845_s_at	-2.44	-2.02	plasminogen activator, urokinase receptor	PLAUR
209803_s_at	-2.46	-2.34	pleckstrin homology-like domain, family A, member 2	PHLDA2
212705_x_at	-2.46	-4.29	patatin-like phospholipase domain containing 2	PNPLA2
215034_s_at	-2.46	-2.73	transmembrane 4 L six family member 1	TM4SF1
1556685_at	-2.47	-26.00		
234455_at	-2.49	-2.56	zinc finger protein 1 homolog (mouse)	ZFP1
1554631_at	-2.50	-2.30	ataxia telangiectasia mutated (includes complementation groups A, C and D)	ATM
228728_at	-2.51	-2.61		
237411_at	-2.52	-18.50	ADAM metallopeptidase with thrombospondin type 1 motif, 6	ADAMTS6
223963_s_at	-2.53	-2.25	insulin-like growth factor 2 mRNA binding protein 2	IGF2BP2
1568623_a_at	-2.54	-2.78	solute carrier family 35, member E4	SLC35E4
1560495_at	-2.59	-3.19		
202638_s_at	-2.61	-2.70	intercellular adhesion molecule 1 (CD54), human rhinovirus receptor	ICAM1
206341_at	-2.63	-2.44	interleukin 2 receptor, alpha	IL2RA
222336_at	-2.64	-2.02	chromosome 4 open reading frame 34	C4orf34
244007_at	-2.68	-2.29		
230727_at	-2.69	-2.02	polycomb group ring finger 2	PCGF2
236043_at	-2.69	-3.76		
226560_at	-2.70	-2.63		
205931_s_at	-2.73	-2.25	cAMP responsive element binding protein 5	CREB5
237114_at	-2.73	-2.90	trafficking protein particle complex 3	TRAPPC3
204446_s_at	-2.73	-2.04	arachidonate 5-lipoxygenase	ALOX5
237597_at	-2.74	-2.08		
240232_at	-2.80	-2.11	chromosome 3 open reading frame 1	C3orf1
235079_at	-2.80	-2.21		
204015_s_at	-2.82	-3.47	dual specificity phosphatase 4	DUSP4
206805_at	-2.82	-2.32	sema domain, immunoglobulin domain (Ig), short basic domain, secreted, (semaphorin) 3A	SEMA3A
220897_at	-2.82	-2.24		
205283_at	-2.82	-2.15	Fukuyama type congenital muscular dystrophy (fukutin)	FCMD
205599_at	-2.84	-2.30	TNF receptor-associated factor 1	TRAF1
204420_at	-2.85	-2.46	FOS-like antigen 1	FOSL1
205534_at	-2.88	-2.25	BH-protocadherin (brain-heart)	PCDH7
224045_x_at	-2.89	-13.67	chromosome 18 open reading frame 2	C18orf2
1563303_at	-2.89	-2.16		
209959_at	-2.92	-2.32	nuclear receptor subfamily 4, group A, member 3	NR4A3
238371_s_at	-2.93	-8.13		
1564331_at	-2.97	-3.51		
202627_s_at	-3.06	-2.17	serpin peptidase inhibitor, clade E (nexin, plasminogen activator inhibitor type 1), member 1	SERPINE1

210822_at	-3.08	-2.45		
1405_i_at	-3.10	-2.52	chemokine (C-C motif) ligand 5	CCL5
216177_at	-3.12	-3.28		
204655_at	-3.13	-2.29	chemokine (C-C motif) ligand 5	CCL5
230346_x_at	-3.13	-2.14		
207216_at	-3.15	-2.57	tumor necrosis factor (ligand) superfamily, member 8	TNFSF8
1568713_a_at	-3.15	-6.07	TBC1 (tre-2/USP6, BUB2, cdc16) domain family, member 1	TBC1D1
212099_at	-3.16	-2.16	ras homolog gene family, member B	RHOB
214222_at	-3.17	-2.05	dynein, axonemal, heavy chain 7	DNAH7
237516_at	-3.18	-3.07		
1560370_x_at	-3.19	-3.45	ankylosis, progressive homolog (mouse)	ANKH
204698_at	-3.21	-2.05	interferon stimulated exonuclease gene 20kDa	ISG20
244429_at	-3.25	-2.41		
206510_at	-3.30	-2.06	sine oculis homeobox homolog 2 (Drosophila)	SIX2
216248_s_at	-3.34	-2.38	nuclear receptor subfamily 4, group A, member 2	NR4A2
215646_s_at	-3.41	-4.28	chondroitin sulfate proteoglycan 2 (versican)	CSPG2
231779_at	-3.42	-2.55	interleukin-1 receptor-associated kinase 2	IRAK2
210874_s_at	-3.44	-2.02	N-acetyltransferase 6	NAT6
1559701_s_at	-3.46	-2.64	N-deacetylase/N-sulfotransferase (heparan glucosaminyl) 2	NDST2
239058_at	-3.48	-2.74		
204621_s_at	-3.48	-2.57	nuclear receptor subfamily 4, group A, member 2	NR4A2
205002_at	-3.49	-6.42	AT hook, DNA binding motif, containing 1	AHDC1
1555486_a_at	-3.50	-2.13		
240698_s_at	-3.52	-2.67		
232593_at	-3.56	-2.52		
213182_x_at	-3.58	-4.21	cyclin-dependent kinase inhibitor 1C (p57, Kip2)	CDKN1C
1569872_a_at	-3.62	-2.61		
204622_x_at	-3.64	-2.61	nuclear receptor subfamily 4, group A, member 2	NR4A2
226913_s_at	-3.65	-3.44	SRY (sex determining region Y)-box 8	SOX8
204912_at	-3.71	-2.06	interleukin 10 receptor, alpha	IL10RA
217619_x_at	-4.15	-3.37		
207176_s_at	-4.19	-2.91	CD80 molecule	CD80
203726_s_at	-4.51	-2.03	laminin, alpha 3	LAMA3
1552917_at	-4.52	-3.99	interleukin 29 (interferon, lambda 1)	IL29
201566_x_at	-4.56	-2.88	inhibitor of DNA binding 2, dominant negative helix-loop-helix protein	ID2
1553151_at	-4.65	-2.76	ATPase, H+ transporting, lysosomal 38kDa, V0 subunit d2	ATP6V0D2
203665_at	-4.66	-3.08	heme oxygenase (decycling) 1	HMOX1
232322_x_at	-4.71	-2.07	START domain containing 10	STARD10
1570622_at	-4.72	-2.16		
226487_at	-4.91	-2.01	chromosome 12 open reading frame 34	C12orf34

235001_at	-4.92	-11.15		
1555689_at	-4.98	-2.71	CD80 molecule	CD80
242814_at	-5.05	-2.00	serpin peptidase inhibitor, clade B (ovalbumin), member 9	SERPINB9
206408_at	-5.05	-3.19	leucine rich repeat transmembrane neuronal 2	LRRTM2
239759_at	-5.17	-3.91		
1554519_at	-5.29	-2.49	CD80 molecule	CD80
218717_s_at	-5.33	-2.45	leprecan-like 1	LEPREL1
207113_s_at	-5.45	-2.31	tumor necrosis factor (TNF superfamily, member 2)	TNF
229830_at	-5.57	-2.90		
206942_s_at	-5.60	-4.48	pro-melanin-concentrating hormone	PMCH
203961_at	-5.60	-3.47	nebulette	NEBL
1569916_at	-5.91	-5.77		
223484_at	-5.92	-3.68	chromosome 15 open reading frame 48	C15orf48
218723_s_at	-6.08	-2.09		
206552_s_at	-6.36	-2.44	tachykinin, precursor 1 (substance K, substance P, neurokinin 1, neurokinin 2, neuromedin L, neuroki	TAC1
235735_at	-6.46	-3.60		
230232_at	-6.55	-2.20		
204103_at	-6.70	-2.38	chemokine (C-C motif) ligand 4	CCL4
204421_s_at	-6.74	-3.16	fibroblast growth factor 2 (basic)	FGF2
205463_s_at	-6.89	-3.01	platelet-derived growth factor alpha polypeptide	PDGFA
229495_at	-8.36	-3.83	aminoacylase 1-like 2	ACY1L2
241567_at	-9.17	-3.22	nucleolar protein 4	NOL4
237448_at	-9.33	-3.06		
224393_s_at	-10.73	-6.86	cat eye syndrome chromosome region, candidate 6	CECR6
228362_s_at	-18.12	-2.03		
226681_at	-21.47	-2.05	ubiquitin-conjugating enzyme E2H (UBC8 homolog, yeast)	UBE2H
206426_at	-23.43	-27.26	melan-A	MLANA
