# Supplementary material for: In Vitro Transformation of Primary Human CD34+ Cells by AML Fusion Oncogenes: Early Gene Expression Profiling Reveals Possible Drug Target in AML
Source: PLoS One. 2010 Aug 27;5(8):e12464. doi: 10.1371/journal.pone.0012464 (PMC2929205; doi:10.1371/journal.pone.0012464)
Supplement: Table S11 — Genes deregulated by NUP98-HOXA9 3 days after transduction. Primary human CD34+ cells were retrovirally transduced with either control MSCV-IRES-GFP vector or vector expressing NUP98-HOXA9 and sorted for GFP positivity. Total RNA was extracted 3 days after transduction and subjected to microarray analysis. Genes that showed up- or down-regulation by 2 fold or more in comparison to the control in 2 independent experiments (Exp.1 and Exp.2) were considered deregulated. (0.05 MB PDF) [file pone.0012464.s011.pdf]

Table S11. Genes deregulated by NUP98-HOXA9 at 3 d after transduction

Probe set ID	Fold Change		Gene Name	Gene Symbol
	Exp.1	Exp.2		
206367_at	124.34	348.47	renin	REN
204439_at	89.34	1189.73	interferon-induced protein 44-like	IFI44L
1563072_at	56.70	28.33		
237058_x_at	46.75	17.16	solute carrier family 6 (neurotransmitter transporter, GABA), member 13	SLC6A13
203153_at	45.48	41.71	interferon-induced protein with tetratricopeptide repeats 1	IFIT1
1567575_at	44.49	5.06		
223595_at	35.37	4.84	transmembrane protein 133	TMEM133
207836_s_at	34.36	6.68	RNA binding protein with multiple splicing	RBPMS
202086_at	32.86	40.05	myxovirus (influenza virus) resistance 1, interferon-inducible protein p78 (mouse)	MX1
236893_at	31.14	5.72		
206382_s_at	30.32	3.97	brain-derived neurotrophic factor	BDNF
206442_at	29.84	134.14	semenogelin I	SEMG1
204533_at	29.68	20.89	chemokine (C-X-C motif) ligand 10	CXCL10
206987_x_at	28.17	44.28	fibroblast growth factor 18	FGF18
223838_at	27.19	2.42	testis specific, 10	TSGA10
223652_at	24.70	2.27	arsenic (+3 oxidation state) methyltransferase	AS3MT
215301_at	23.96	16.15		
1555123_at	19.03	2.37	ST6 beta-galactosamide alpha-2,6-sialyltranferase 2	ST6GAL2
215571_at	16.67	4.31		
213578_at	16.67	3.30	bone morphogenetic protein receptor, type IA	BMPR1A
211361_s_at	15.81	3.13	serpin peptidase inhibitor, clade B (ovalbumin), member 13	SERPINB13
235617_x_at	15.29	2.91		
214569_at	15.09	15.53	interferon, alpha 5	IFNA5
232155_at	13.85	3.68	KIAA1618	KIAA1618
207837_at	13.80	5.55	RNA binding protein with multiple splicing	RBPMS
220237_at	12.34	2.93	ATG3 autophagy related 3 homolog (S. cerevisiae)	ATG3
1553300_a_at	12.31	4.30	diacylglycerol kinase, eta	DGKH
229057_at	12.15	3.59	sodium channel, voltage-gated, type II, alpha subunit	SCN2A
226757_at	12.08	9.52	interferon-induced protein with tetratricopeptide repeats 2	IFIT2
202011_at	11.78	2.01	tight junction protein 1 (zona occludens 1)	TJP1
207596_at	11.65	2.00		
234563_at	11.56	3.60		
220784_s_at	11.35	8.04	urotensin 2	UTS2
244503_at	11.35	5.98	brain-derived neurotrophic factor	BDNF
229450_at	11.31	10.08	interferon-induced protein with tetratricopeptide repeats 3	IFIT3
239979_at	11.24	10.87	epithelial stromal interaction 1 (breast)	EPSTI1
205278_at	10.73	14.54	glutamate decarboxylase 1 (brain, 67kDa)	GAD1

236892_s_at	10.64	8.32		
1558606_s_at	9.93	3.33		
218400_at	9.77	10.77	2'-5'-oligoadenylate synthetase 3, 100kDa	OAS3
213435_at	9.63	2.57	SATB family member 2	SATB2
205483_s_at	9.63	10.55	ISG15 ubiquitin-like modifier	ISG15
1555617_x_at	9.44	2.00		
214059_at	9.43	6.84	interferon-induced protein 44	IFI44
207901_at	9.09	4.21	interleukin 12B (natural killer cell stimulatory factor 2, cytotoxic lymphocyte maturation factor 2,	IL12B
1562103_at	8.95	2.40	Janus kinase 1 (a protein tyrosine kinase)	JAK1
241897_at	8.89	96.56	RNA binding protein with multiple splicing	RBPMS
235276_at	8.74	10.74	epithelial stromal interaction 1 (breast)	EPSTI1
219737_s_at	8.32	3.81	protocadherin 9	PCDH9
205984_at	8.30	12.96	corticotropin releasing hormone binding protein	CRHBP
204747_at	8.30	9.23	interferon-induced protein with tetratricopeptide repeats 3	IFIT3
204994_at	8.26	8.29	myxovirus (influenza virus) resistance 2 (mouse)	MX2
202869_at	7.98	36.76	2',5'-oligoadenylate synthetase 1, 40/46kDa	OAS1
1554712_a_at	7.92	2.16	glycine-N-acyltransferase-like 2	GLYATL2
208436_s_at	7.89	7.63	interferon regulatory factor 7	IRF7
211029_x_at	7.81	36.04	fibroblast growth factor 18	FGF18
226279_at	6.98	4.67	protease, serine, 23	PRSS23
1560615_a_at	6.88	2.12		
231382_at	6.87	18.71	fibroblast growth factor 18	FGF18
202350_s_at	6.76	2.99	matrilin 2	MATN2
213197_at	6.75	2.21	astrotactin 1	ASTN1
238919_at	6.72	3.51		
219211_at	6.58	6.30	ubiquitin specific peptidase 18	USP18
203595_s_at	6.56	4.07		
211597_s_at	6.48	4.75		
228607_at	6.43	6.34	2'-5'-oligoadenylate synthetase 2, 69/71kDa	OAS2
227195_at	6.35	6.11	zinc finger protein 503	ZNF503
229441_at	6.12	34.94	protease, serine, 23	PRSS23
209795_at	5.93	5.98	CD69 molecule	CD69
209487_at	5.89	6.99	RNA binding protein with multiple splicing	RBPMS
214453_s_at	5.85	8.73	interferon-induced protein 44	IFI44
206655_s_at	5.81	13.91	glycoprotein Ib (platelet), beta polypeptide	GP1BB
242873_at	5.78	6.14		
1560318_at	5.78	2.92	Rho GTPase activating protein 29	ARHGAP29
233321_x_at	5.78	2.29		
205749_at	5.65	5.41	cytochrome P450, family 1, subfamily A, polypeptide 1	CYP1A1
233888_s_at	5.65	2.02	SLIT-ROBO Rho GTPase activating protein 1	SRGAP1
205694_at	5.61	8.91	tyrosinase-related protein 1	TYRP1
235643_at	5.60	6.01	sterile alpha motif domain containing 9-like	SAMD9L
242002_at	5.52	2.51	T-cell lymphoma breakpoint associated target 1	TCBA1
228507_at	5.50	4.42		
217380_s_at	5.42	3.48		

237261_at	5.41	2.29		
210831_s_at	5.39	3.55	prostaglandin E receptor 3 (subtype EP3)	PTGER3
229151_at	5.36	5.35	solute carrier family 14 (urea transporter), member 1 (Kidd blood group)	SLC14A1
242234_at	5.28	4.93		
205552_s_at	5.17	8.02	2',5'-oligoadenylate synthetase 1, 40/46kDa	OAS1
228617_at	5.15	8.74		
242625_at	4.92	3.60	radical S-adenosyl methionine domain containing 2	RSAD2
242719_at	4.90	2.54		
1560734_at	4.88	2.38	olfactory receptor, family 4, subfamily N, member 4	OR4N4
1563854_s_at	4.84	2.80		
235737_at	4.80	4.22		
206385_s_at	4.75	4.01	ankyrin 3, node of Ranvier (ankyrin G)	ANK3
212850_s_at	4.70	2.35	low density lipoprotein receptor-related protein 4	LRP4
235521_at	4.70	4.38	homeobox A3	HOXA3
203789_s_at	4.61	2.22	sema domain, immunoglobulin domain (Ig), short basic domain, secreted, (semaphorin) 3C	SEMA3C
226603_at	4.54	4.65	sterile alpha motif domain containing 9-like	SAMD9L
233944_at	4.47	6.16		
239956_at	4.42	3.04		
230036_at	4.39	4.18	sterile alpha motif domain containing 9-like	SAMD9L
1563467_at	4.36	11.53		
209859_at	4.29	2.25	tripartite motif-containing 9	TRIM9
232979_at	4.28	5.42		
213358_at	4.23	2.13	KIAA0802	KIAA0802
205660_at	4.17	5.58	2'-5'-oligoadenylate synthetase-like	OASL
1556209_at	4.16	3.06	C-type lectin domain family 2, member B	CLEC2B
1554319_at	4.14	2.70	ribosomal protein S6 kinase, 90kDa, polypeptide 5	RPS6KA5
214043_at	4.13	2.87	protein tyrosine phosphatase, receptor type, D	PTPRD
235885_at	4.13	31.03		
214596_at	4.12	3.10		
206133_at	4.07	6.63		
229638_at	4.06	32.21	iroquois homeobox protein 3	IRX3
205844_at	4.06	6.74	vanin 1	VNN1
227609_at	4.06	5.85	epithelial stromal interaction 1 (breast)	EPSTI1
222793_at	4.06	2.28	DEAD (Asp-Glu-Ala-Asp) box polypeptide 58	DDX58
205513_at	4.03	5.67	transcobalamin I (vitamin B12 binding protein, R binder family)	TCN1
242321_at	3.99	2.09		
234640_x_at	3.99	2.07		
1562214_at	3.98	4.73		
205572_at	3.98	2.65	angiopoietin 2	ANGPT2
228121_at	3.94	2.10		
213797_at	3.93	3.36	radical S-adenosyl methionine domain containing 2	RSAD2
204753_s_at	3.91	2.28	hepatic leukemia factor	HLF

226189_at	3.88	2.63	integrin, beta 8	ITGB8
243271_at	3.88	4.50		
1562255_at	3.84	5.54	synaptotagmin-like 3	SYTL3
213844_at	3.81	3.73	homeobox A5	HOXA5
224349_at	3.81	4.14		
233927_at	3.81	2.70		
228531_at	3.81	2.97	sterile alpha motif domain containing 9	SAMD9
1565602_at	3.79	10.77		
214933_at	3.78	2.82	calcium channel, voltage-dependent, P/Q type, alpha 1A subunit	CACNA1A
227826_s_at	3.73	4.25		
239959_x_at	3.71	4.31	phosphodiesterase 3B, cGMP-inhibited	PDE3B
208557_at	3.71	5.17	homeobox A6	HOXA6
231947_at	3.70	2.11	myc target 1	MYCT1
237009_at	3.67	2.89	CD69 molecule	CD69
223220_s_at	3.64	3.09	poly (ADP-ribose) polymerase family, member 9	PARP9
219049_at	3.63	2.27		
240539_at	3.58	4.26	autism susceptibility candidate 2	AUTS2
242172_at	3.55	2.86	Meis1, myeloid ecotropic viral integration site 1 homolog (mouse)	MEIS1
203882_at	3.54	3.85	interferon-stimulated transcription factor 3, gamma 48kDa	ISGF3G
1552736_a_at	3.51	2.43	neuropilin (NRP) and tolloid (TLL)-like 1	NETO1
212224_at	3.47	2.59	aldehyde dehydrogenase 1 family, member A1	ALDH1A1
220697_at	3.45	2.73		
231578_at	3.44	2.40	guanylate binding protein 1, interferon-inducible, 67kDa	GBP1
220483_s_at	3.44	2.59	ring finger protein 19	RNF19
1555929_s_at	3.43	2.85		
218943_s_at	3.39	4.37	DEAD (Asp-Glu-Ala-Asp) box polypeptide 58	DDX58
231911_at	3.38	6.41	KIAA1189	KIAA1189
238921_at	3.36	2.28		
226207_at	3.35	2.37		
1562583_s_at	3.34	2.03		
202820_at	3.31	3.44	aryl hydrocarbon receptor	AHR
211267_at	3.29	2.32	homeobox, ES cell expressed 1	HESX1
231577_s_at	3.29	2.92	guanylate binding protein 1, interferon-inducible, 67kDa	GBP1
223179_at	3.28	2.90	yippee-like 3 (Drosophila)	YPEL3
241837_at	3.26	3.10	AT rich interactive domain 5B (MRF1-like)	ARID5B
44783_s_at	3.26	2.30	hairy/enhancer-of-split related with YRPW motif 1	HEY1
206289_at	3.25	3.34	homeobox A4	HOXA4
242907_at	3.24	2.58		
212820_at	3.23	2.99	Dmx-like 2	DMXL2
234284_at	3.23	3.24	guanine nucleotide binding protein (G protein), gamma 8	GNG8
204972_at	3.21	8.57	2'-5'-oligoadenylate synthetase 2, 69/71kDa	OAS2
204748_at	3.21	2.71	prostaglandin-endoperoxide synthase 2 (prostaglandin G/H synthase and cyclooxygenase)	PTGS2

204755_x_at	3.20	2.97	hepatic leukemia factor	HLF
206586_at	3.17	2.03	cannabinoid receptor 2 (macrophage)	CNR2
1557286_at	3.16	3.53		
227679_at	3.14	2.69		
206932_at	3.12	2.81	cholesterol 25-hydroxylase	CH25H
230000_at	3.12	3.96	ring finger protein 213	RNF213
227484_at	3.11	4.24		
202180_s_at	3.11	2.18	major vault protein	MVP
231880_at	3.09	2.76	family with sequence similarity 40, member B	FAM40B
211488_s_at	3.06	2.36	integrin, beta 8	ITGB8
202708_s_at	3.05	2.06	histone cluster 2, H2be	HIST2H2BE
219352_at	3.04	3.75	hect domain and RLD 6	HERC6
233430_at	3.03	2.14	TBC1 domain family, member 22B	TBC1D22B
212097_at	2.99	5.89	caveolin 1, caveolae protein, 22kDa	CAV1
219606_at	2.98	2.31	PHD finger protein 20-like 1	PHF20L1
235074_at	2.98	2.66	sprouty-related, EVH1 domain containing 1	SPRED1
231476_at	2.96	2.15		
207489_at	2.95	2.32		
214984_at	2.94	2.11		
209905_at	2.91	2.55	homeobox A9	HOXA9
1555392_at	2.91	2.29		
216460_at	2.90	4.54		
225929_s_at	2.89	3.43	ring finger protein 213	RNF213
229631_at	2.89	4.06	dynein heavy chain domain 1	DNHD1
1561690_at	2.88	2.68		
1557520_a_at	2.88	2.85	transmembrane protein 59	TMEM59
233880_at	2.88	3.26	ring finger protein 213	RNF213
206806_at	2.88	2.76	diacylglycerol kinase, iota	DGKI
216956_s_at	2.87	2.91	integrin, alpha 2b (platelet glycoprotein IIb of IIb/IIIa complex, antigen CD41)	ITGA2B
228708_at	2.87	2.68		
241497_at	2.85	3.76		
206828_at	2.84	2.53	TXK tyrosine kinase	TXK
231132_at	2.83	2.20		
229350_x_at	2.83	2.02	poly (ADP-ribose) polymerase family, member 10	PARP10
224403_at	2.83	4.17	Fc receptor-like 4	FCRL4
1564077_at	2.82	5.15		
210145_at	2.82	2.16	phospholipase A2, group IVA (cytosolic, calcium-dependent)	PLA2G4A
243576_at	2.81	2.05		
32128_at	2.79	4.29	chemokine (C-C motif) ligand 18 (pulmonary and activation-regulated)	CCL18
205547_s_at	2.78	2.79	transgelin	TAGLN
228285_at	2.78	3.57	tudor domain containing 9	TDRD9
214651_s_at	2.77	2.07	homeobox A9	HOXA9
214776_x_at	2.77	2.13	xylulokinase homolog (H. influenzae)	XYLB
201601_x_at	2.77	2.61	interferon induced transmembrane protein 1 (9 27)	IFITM1
1561642_at	2.76	12.41		
220315_at	2.76	2.01	poly (ADP-ribose) polymerase family, member 11	PARP11

233072_at	2.76	5.47	netrin G2	NTNG2
203020_at	2.76	2.19	RAB GTPase activating protein 1-like	RABGAP1L
206446_s_at	2.75	2.51	elastase 1, pancreatic	ELA1
241421_at	2.74	2.55		
219230_at	2.72	4.17	transmembrane protein 100	TMEM100
201242_s_at	2.72	2.84	ATPase, Na+/K+ transporting, beta 1 polypeptide	ATP1B1
1553705_a_at	2.71	3.93	cholinergic receptor, muscarinic 3	CHRM3
202458_at	2.71	2.41	protease, serine, 23	PRSS23
238595_at	2.71	2.68		
232653_at	2.70	2.41	trichorhinophalangeal syndrome I	TRPS1
201744_s_at	2.70	2.13	lumican	LUM
1552752_a_at	2.70	2.05	cell adhesion molecule 2	CADM2
231956_at	2.68	2.79	KIAA1618	KIAA1618
241777_x_at	2.68	5.08	adaptor protein, phosphotyrosine interaction, PH domain and leucine zipper containing 2	APPL2
242721_at	2.68	3.16	autism susceptibility candidate 2	AUTS2
202145_at	2.68	3.61	lymphocyte antigen 6 complex, locus E	LY6E
218429_s_at	2.67	2.30		
209732_at	2.67	3.65	C-type lectin domain family 2, member B	CLEC2B
204082_at	2.66	2.40	pre-B-cell leukemia homeobox 3	PBX3
238297_at	2.65	3.96	phosphatase and actin regulator 1	PHACTR1
223980_s_at	2.63	4.05	SP110 nuclear body protein	SP110
229309_at	2.63	3.02		
228904_at	2.63	3.02	homeobox B3	HOXB3
1556359_at	2.62	2.44	chromosome 6 open reading frame 89	C6orf89
1559633_a_at	2.62	2.42	cholinergic receptor, muscarinic 3	CHRM3
53720_at	2.62	2.46		
244764_at	2.62	2.12		
235157_at	2.62	4.39		
212950_at	2.62	2.80	G protein-coupled receptor 116	GPR116
1560483_at	2.61	2.54		
210029_at	2.61	5.17	indoleamine-pyrrole 2,3 dioxygenase	INDO
1560449_at	2.60	2.36		
219691_at	2.60	2.01	sterile alpha motif domain containing 9	SAMD9
231854_at	2.60	2.57		
1566446_at	2.60	2.57		
240602_at	2.59	2.91	HBS1-like (S. cerevisiae)	HBS1L
200872_at	2.59	2.16	S100 calcium binding protein A10	S100A10
241951_at	2.59	2.02		
244658_at	2.59	2.45		
232787_at	2.58	3.27		
216278_at	2.57	3.01		
214375_at	2.57	2.61	PTPRF interacting protein, binding protein 1 (liprin beta 1)	PPFIBP1
241508_at	2.57	2.65	ankyrin repeat domain 12	ANKRD12
224701_at	2.57	2.73	poly (ADP-ribose) polymerase family, member 14	PARP14
205081_at	2.56	2.33	cysteine-rich protein 1 (intestinal)	CRIP1
228152_s_at	2.56	3.13		
200923_at	2.56	2.42	lectin, galactoside-binding, soluble, 3 binding protein	LGALS3BP

243474_at	2.55	2.52		
1556713_at	2.55	43.63		
1563461_at	2.55	3.27		
205991_s_at	2.54	2.06	paired related homeobox 1	PRRX1
219452_at	2.54	2.99	dipeptidase 2	DPEP2
202269_x_at	2.53	3.06	guanylate binding protein 1, interferon-inducible, 67kDa	GBP1
242732_at	2.53	2.37	metastasis suppressor 1	MTSS1
218675_at	2.53	2.13	solute carrier family 22 (organic cation transporter), member 17	SLC22A17
234276_at	2.51	3.33		
219534_x_at	2.51	3.23	cyclin-dependent kinase inhibitor 1C (p57, Kip2)	CDKN1C
213338_at	2.51	2.20	transmembrane protein 158	TMEM158
1554840_at	2.50	2.32		
218986_s_at	2.50	3.11		
232449_at	2.49	3.23	beta-carotene dioxygenase 2	BCDO2
213348_at	2.49	2.32	cyclin-dependent kinase inhibitor 1C (p57, Kip2)	CDKN1C
226756_at	2.47	2.62		
202241_at	2.46	2.42	tribbles homolog 1 (Drosophila)	TRIB1
204547_at	2.46	2.33	RAB40B, member RAS oncogene family	RAB40B
204633_s_at	2.45	2.37	ribosomal protein S6 kinase, 90kDa, polypeptide 5	RPS6KA5
240400_at	2.45	2.54		
216439_at	2.44	9.79	tyrosine kinase, non-receptor, 2	TNK2
236453_at	2.44	2.01		
204044_at	2.44	2.27	quinolinate phosphoribosyltransferase (nicotinate-nucleotide pyrophosphorylase (carboxylating))	QPRT
1569652_at	2.44	3.37	myeloid/lymphoid or mixed-lineage leukemia (trithorax homolog, Drosophila); translocated to, 3	MLLT3
1557826_at	2.43	2.08		
244104_at	2.43	2.21	mannosyl (beta-1,4-)-glycoprotein beta-1,4-N-acetylglucosaminyltransferase	MGAT3
226716_at	2.42	2.25	proline rich 12	PRR12
241030_at	2.41	5.90	fibrous sheath interacting protein 1	FSIP1
209374_s_at	2.41	2.87	immunoglobulin heavy constant mu	IGHM
1563469_at	2.41	2.82		
1563611_at	2.40	2.32		
1555967_at	2.39	3.95		
234190_at	2.38	2.47		
239600_at	2.37	3.19		
44790_s_at	2.37	2.00	chromosome 13 open reading frame 18	C13orf18
204529_s_at	2.36	2.36		
216887_s_at	2.36	2.47	LIM domain binding 3	LDB3
244625_at	2.35	3.32	arginine-glutamic acid dipeptide (RE) repeats	RERE
1563589_at	2.35	17.63		
220504_at	2.34	8.02	keratocan	KERA
215321_at	2.34	2.28		
203461_at	2.33	2.06	chromodomain helicase DNA binding protein 2	CHD2

1556105_at	2.32	2.02		
243160_at	2.31	7.93		
241184_x_at	2.31	3.84	zinc finger protein 407	ZNF407
1553204_at	2.30	2.64		
205942_s_at	2.30	2.22	acyl-CoA synthetase medium-chain family member 3	ACSM3
209761_s_at	2.30	2.72	SP110 nuclear body protein	SP110
1563802_at	2.29	5.47		
240254_at	2.29	2.19	TRAF2 and NCK interacting kinase	TNIK
218543_s_at	2.29	2.36	poly (ADP-ribose) polymerase family, member 12	PARP12
244695_at	2.29	2.09		
210262_at	2.29	3.40	cysteine-rich secretory protein 2	CRISP2
212274_at	2.28	2.03	lipin 1	LPIN1
206553_at	2.28	2.41	2'-5'-oligoadenylate synthetase 2, 69/71kDa	OAS2
221943_x_at	2.27	2.27	ribosomal protein L38	RPL38
207437_at	2.27	2.75	neuro-oncological ventral antigen 1	NOVA1
225636_at	2.27	2.43	signal transducer and activator of transcription 2, 113kDa	STAT2
242414_at	2.27	2.20		
239162_at	2.27	2.86		
208392_x_at	2.27	2.68	SP110 nuclear body protein	SP110
232504_at	2.27	3.72		
213058_at	2.27	3.03	tetratricopeptide repeat domain 28	TTC28
212327_at	2.26	8.46		
1554601_at	2.26	4.82	T-cell lymphoma breakpoint associated target 1	TCBA1
203788_s_at	2.26	2.16	sema domain, immunoglobulin domain (Ig), short basic domain, secreted, (semaphorin) 3C	SEMA3C
242864_at	2.24	2.59	zinc finger protein 554	ZNF554
244471_x_at	2.24	2.67	pannexin 2	PANX2
1553271_at	2.24	3.55	DIP2 disco-interacting protein 2 homolog B (Drosophila)	DIP2B
204635_at	2.24	2.22	ribosomal protein S6 kinase, 90kDa, polypeptide 5	RPS6KA5
207788_s_at	2.23	2.78	sorbin and SH3 domain containing 3	SORBS3
239808_at	2.23	2.43		
244414_at	2.23	3.20	mastermind-like 2 (Drosophila)	MAML2
237632_at	2.23	2.31	hect (homologous to the E6-AP (UBE3A) carboxyl terminus) domain and RCC1 (CHC1)-like domain (RLD) 1	HERC1
212827_at	2.23	2.57	immunoglobulin heavy constant mu	IGHM
1555370_a_at	2.21	2.92	calmodulin binding transcription activator 1	CAMTA1
242357_x_at	2.20	2.62		
1555014_x_at	2.20	3.90		
231697_s_at	2.20	4.51	transmembrane protein 49	TMEM49
219501_at	2.19	5.34		
204917_s_at	2.19	2.62	myeloid/lymphoid or mixed-lineage leukemia (trithorax homolog, Drosophila); translocated to, 3	MLLT3
219863_at	2.19	2.23	hect domain and RLD 5	HERC5
213294_at	2.19	2.18		

1557046_x_at	2.18	2.22		
34408_at	2.18	2.04	reticulon 2	RTN2
242598_at	2.18	2.13	src kinase associated phosphoprotein 2	SKAP2
238076_at	2.18	2.01		
240656_at	2.18	2.52	signal-induced proliferation-associated 1 like 1	SIPA1L1
236385_at	2.17	2.35		
244359_s_at	2.17	2.31		
227807_at	2.17	3.31	poly (ADP-ribose) polymerase family, member 9	PARP9
232695_at	2.17	2.86	kinesin family member 6	KIF6
224321_at	2.17	2.86	transmembrane protein with EGF-like and two follistatin-like domains 2	TMEFF2
225415_at	2.17	2.04	deltex 3-like (Drosophila)	DTX3L
209969_s_at	2.16	3.80	signal transducer and activator of transcription 1, 91kDa	STAT1
215761_at	2.16	2.88	Dmx-like 2	DMXL2
215288_at	2.16	8.24	transient receptor potential cation channel, subfamily C, member 2	TRPC2
229540_at	2.16	2.34	recombining binding protein suppressor of hairless (Drosophila)	RBPSUH
217614_at	2.15	2.52		
228547_at	2.15	2.11	neurexin 1	NRXN1
207017_at	2.15	3.81	RAB27B, member RAS oncogene family	RAB27B
1569225_a_at	2.14	2.01	sex comb on midleg-like 4 (Drosophila)	SCML4
1560492_at	2.14	2.05		
232375_at	2.13	3.14		
232601_at	2.13	2.21		
237988_at	2.12	2.49	eukaryotic translation initiation factor 1B	EIF1B
242171_at	2.12	4.22		
242310_at	2.12	2.22		
208012_x_at	2.11	2.02	SP110 nuclear body protein	SP110
238327_at	2.11	2.97		
238732_at	2.11	2.58	collagen, type XXIV, alpha 1	COL24A1
1554996_at	2.11	3.67	zinc finger protein 479	ZNF479
225109_at	2.11	3.44	2-oxoglutarate and iron-dependent oxygenase domain containing 1	OGFOD1
37170_at	2.11	2.31	BMP2 inducible kinase	BMP2K
202748_at	2.10	2.24	guanylate binding protein 2, interferon-inducible	GBP2
214022_s_at	2.10	3.08	interferon induced transmembrane protein 1 (9 27)	IFITM1
1554933_at	2.10	2.59	PC4 and SFRS1 interacting protein 1	PSIP1
224558_s_at	2.09	2.36	metastasis associated lung adenocarcinoma transcript 1 (non-coding RNA)	MALAT1
242579_at	2.09	6.42		
212599_at	2.08	2.36	autism susceptibility candidate 2	AUTS2
212254_s_at	2.08	2.11	dystonin	DST
212013_at	2.08	2.23	peroxidasin homolog (Drosophila)	PXDN
209210_s_at	2.08	2.25	pleckstrin homology domain containing, family C (with FERM domain) member 1	PLEKHC1
201641_at	2.08	2.63	bone marrow stromal cell antigen 2	BST2
1553894_at	2.07	2.23	coiled-coil domain containing 122	CCDC122

1568736_s_at	2.07	4.60		
204187_at	2.07	2.60	guanosine monophosphate reductase	GMPR
243585_at	2.06	2.85	ATPase type 13A5	ATP13A5
234046_at	2.06	2.14		
204237_at	2.06	3.29	GULP, engulfment adaptor PTB domain containing 1	GULP1
242488_at	2.06	2.79		
241099_at	2.06	3.05	elongation protein 4 homolog (S. cerevisiae)	ELP4
216129_at	2.05	2.76	ATPase, Class II, type 9A	ATP9A
243262_at	2.05	2.50	SET and MYND domain containing 3	SMYD3
1557585_at	2.05	2.29	ATPase, H+ transporting, lysosomal 50/57kDa, V1 subunit H	ATP6V1H
244480_at	2.05	2.43		
1559916_a_at	2.04	2.04		
203476_at	2.04	2.56	trophoblast glycoprotein	TPBG
235601_at	2.04	2.03		
210832_x_at	2.04	2.15	prostaglandin E receptor 3 (subtype EP3)	PTGER3
209417_s_at	2.04	2.93	interferon-induced protein 35	IFI35
231616_at	2.04	3.58	glycophorin A (MNS blood group)	GYPA
243428_at	2.04	3.92	KCNQ1 overlapping transcript 1	KCNQ1OT1
216858_x_at	2.03	2.49		
206310_at	2.03	2.63	serine peptidase inhibitor, Kazal type 2 (acrosin-trypsin inhibitor)	SPINK2
231644_at	2.03	2.38		
213261_at	2.03	2.31		
1554866_at	2.03	2.12	transmembrane protein 135	TMEM135
237485_at	2.03	6.89		
239130_at	2.03	2.62		
1556682_s_at	2.02	8.51		
210163_at	2.02	2.24	chemokine (C-X-C motif) ligand 11	CXCL11
1565073_at	2.02	4.33		
201556_s_at	2.02	2.70	vesicle-associated membrane protein 2 (synaptobrevin 2)	VAMP2
244265_at	2.02	2.63	arginine-glutamic acid dipeptide (RE) repeats	RERE
234522_at	2.01	2.66		
220028_at	2.01	2.01	activin A receptor, type IIB	ACVR2B
244612_at	2.01	2.08		
205681_at	2.01	2.45	BCL2-related protein A1	BCL2A1
1561181_at	2.00	3.83		
203888_at	-16.36	-2.13	thrombomodulin	THBD
1555339_at	-12.79	-8.68	RAP1A, member of RAS oncogene family	RAP1A
1555340_x_at	-12.79	-7.94	RAP1A, member of RAS oncogene family	RAP1A
226446_at	-6.20	-2.18	hairy and enhancer of split 6 (Drosophila)	HES6
211298_s_at	-6.18	-5.46	albumin	ALB
220285_at	-3.16	-2.25	chromosome 9 open reading frame 77	C9orf77
1555745_a_at	-3.15	-2.36	lysozyme (renal amyloidosis)	LYZ
224387_at	-2.98	-2.33	COMM domain containing 5	COMMD5
208950_s_at	-2.57	-2.12	aldehyde dehydrogenase 7 family, member A1	ALDH7A1
219837_s_at	-2.49	-2.37	cytokine-like 1	CYTL1
200796_s_at	-2.48	-2.69	myeloid cell leukemia sequence 1 (BCL2-related)	MCL1
238469_at	-2.44	-2.14		
230503_at	-2.43	-3.14	sterile alpha motif domain containing 4A	SAMD4A

238448_at	-2.43	-2.38	mitochondrial ribosomal protein L19	MRPL19
218156_s_at	-2.38	-2.08	TSR1, 20S rRNA accumulation, homolog (S. cerevisiae)	TSR1
1555337_a_at	-2.37	-2.23	zinc finger protein 317	ZNF317
214974_x_at	-2.34	-2.85	chemokine (C-X-C motif) ligand 5	CXCL5
211450_s_at	-2.33	-2.03	mutS homolog 6 (E. coli)	MSH6
205159_at	-2.24	-2.50	colony stimulating factor 2 receptor, beta, low-affinity (granulocyte-macrophage)	CSF2RB
203936_s_at	-2.23	-2.39	matrix metallopeptidase 9 (gelatinase B, 92kDa gelatinase, 92kDa type IV collagenase)	MMP9
210325_at	-2.22	-2.10	CD1a molecule	CD1A
226218_at	-2.22	-2.05		
225400_at	-2.21	-2.01	chromosome 1 open reading frame 19	C1orf19
217552_x_at	-2.20	-2.25	complement component (3b/4b) receptor 1 (Knops blood group)	CR1
201330_at	-2.12	-2.93	arginyl-tRNA synthetase	RARS
200604_s_at	-2.12	-2.18	protein kinase, cAMP-dependent, regulatory, type I, alpha (tissue specific extinguisher 1)	PRKAR1A
204419_x_at	-2.06	-2.91	hemoglobin, gamma G	HBG2
214732_at	-2.04	-2.49	Sp1 transcription factor	SP1
