# Supplementary material for: In Vitro Transformation of Primary Human CD34+ Cells by AML Fusion Oncogenes: Early Gene Expression Profiling Reveals Possible Drug Target in AML
Source: PLoS One. 2010 Aug 27;5(8):e12464. doi: 10.1371/journal.pone.0012464 (PMC2929205; doi:10.1371/journal.pone.0012464)
Supplement: Table S12 — Genes deregulated by NUP98-HOXA9 8 days after transduction. Primary human CD34+ cells were retrovirally transduced with either control MSCV-IRES-GFP vector or vector expressing NUP98-HOXA9 and sorted for GFP positivity. Total RNA was extracted 8 days after transduction and subjected to microarray analysis. Genes that showed up- or down-regulation by 2 fold or more in comparison to the control in 2 independent experiments (Exp.1 and Exp.2) were considered deregulated. (0.11 MB PDF) [file pone.0012464.s012.pdf]

**Table S12.** Genes deregulated by NUP98-HOXA9 at 8 d after transduction

| Probe set ID | Fold Change Exp.1 | Fold Change Exp.2 | Gene Name | Gene Symbol |
|---|---|---|---|---|
| 237058_x_at | 70.15 | 7.23 | solute carrier family 6 (neurotransmitter transporter, GABA), member 13 | SLC6A13 |
| 212097_at | 23.27 | 3.73 | caveolin 1, caveolae protein, 22kDa | CAV1 |
| 226103_at | 18.51 | 8.20 | nexilin (F actin binding protein) | NEXN |
| 229450_at | 13.75 | 8.98 | interferon-induced protein with tetratricopeptide repeats 3 | IFIT3 |
| 203153_at | 13.01 | 7.23 | interferon-induced protein with tetratricopeptide repeats 1 | IFIT1 |
| 202086_at | 12.91 | 9.95 | myxovirus (influenza virus) resistance 1, interferon inducible protein p78 (mouse) | MX1 |
| 204747_at | 12.57 | 6.49 | interferon-induced protein with tetratricopeptide repeats 3 | IFIT3 |
| 202411_at | 11.93 | 11.74 | interferon, alpha-inducible protein 27 | IFI27 |
| 206310_at | 10.86 | 4.06 | serine peptidase inhibitor, Kazal type 2 (acrosin-trypsin inhibitor) | SPINK2 |
| 242625_at | 10.73 | 8.27 | radical S-adenosyl methionine domain containing 2 | RSAD2 |
| 210029_at | 10.47 | 4.16 | indoleamine-pyrrole 2,3 dioxygenase | INDO |
| 209757_s_at | 10.16 | 10.57 | v-myc myelocytomatosis viral related oncogene, neuroblastoma derived (avian) | MYCN |
| 212364_at | 9.97 | 2.21 | myosin IB | MYO1B |
| 218400_at | 8.71 | 4.59 | 2'-5'-oligoadenylate synthetase 3, 100kDa | OAS3 |
| 214453_s_at | 8.32 | 5.44 | interferon-induced protein 44 | IFI44 |
| 202145_at | 8.08 | 5.81 | lymphocyte antigen 6 complex, locus E | LY6E |
| 205483_s_at | 7.56 | 5.11 | ISG15 ubiquitin-like modifier | ISG15 |
| 209487_at | 7.52 | 9.16 | RNA binding protein with multiple splicing | RBPMS |
| 1556499_s_at | 7.22 | 21.13 | collagen, type I, alpha 1 | COL1A1 |
| 213844_at | 6.77 | 5.56 | homeobox A5 | HOXA5 |
| 204972_at | 6.26 | 5.79 | 2'-5'-oligoadenylate synthetase 2, 69/71kDa | OAS2 |
| 213797_at | 5.81 | 8.05 | radical S-adenosyl methionine domain containing 2 | RSAD2 |
| 235276_at | 5.74 | 5.38 | epithelial stromal interaction 1 (breast) | EPSTI1 |
| 235521_at | 5.57 | 4.63 | homeobox A3 | HOXA3 |
| 214059_at | 5.57 | 3.95 | interferon-induced protein 44 | IFI44 |
| 204994_at | 5.26 | 2.51 | myxovirus (influenza virus) resistance 2 (mouse) | MX2 |
| 235504_at | 5.12 | 3.27 | gremlin 2, cysteine knot superfamily, homolog (Xenopus laevis) | GREM2 |
| 204533_at | 5.11 | 3.36 | chemokine (C-X-C motif) ligand 10 | CXCL10 |
| 200923_at | 5.09 | 16.55 | lectin, galactoside-binding, soluble, 3 binding protein | LGALS3BP |
| 203595_s_at | 4.96 | 2.45 | | |
| 228617_at | 4.81 | 3.49 | | |
| 226757_at | 4.73 | 4.34 | interferon-induced protein with tetratricopeptide repeats 2 | IFIT2 |
| 218986_s_at | 4.67 | 3.54 | | |
| 205660_at | 4.65 | 2.49 | 2'-5'-oligoadenylate synthetase-like | OASL |

| | | | | |
|---|---|---|---|---|
| 235753_at | 4.63 | 3.98 | homeobox A7 | HOXA7 |
| 212489_at | 4.63 | 4.28 | collagen, type V, alpha 1 | COL5A1 |
| 241684_at | 4.60 | 4.02 | | |
| 219863_at | 4.54 | 2.29 | hect domain and RLD 5 | HERC5 |
| 207911_s_at | 4.32 | 3.17 | transglutaminase 5 | TGM5 |
| 218943_s_at | 4.23 | 2.07 | DEAD (Asp-Glu-Ala-Asp) box polypeptide 58 | DDX58 |
| 205609_at | 4.20 | 7.51 | angiopoietin 1 | ANGPT1 |
| 228904_at | 4.08 | 2.33 | homeobox B3 | HOXB3 |
| 205608_s_at | 4.02 | 2.43 | angiopoietin 1 | ANGPT1 |
| 235065_at | 4.00 | 9.08 | | |
| 227609_at | 3.94 | 2.44 | epithelial stromal interaction 1 (breast) | EPSTI1 |
| 228708_at | 3.83 | 3.83 | | |
| 215818_at | 3.79 | 2.36 | nudix (nucleoside diphosphate linked moiety X)-type motif 7 | NUDT7 |
| 201601_x_at | 3.78 | 3.02 | interferon induced transmembrane protein 1 (9-27) | IFITM1 |
| 209969_s_at | 3.64 | 2.66 | signal transducer and activator of transcription 1, 91kDa | STAT1 |
| 202869_at | 3.57 | 2.88 | 2',5'-oligoadenylate synthetase 1, 40/46kDa | OAS1 |
| 230218_at | 3.49 | 3.88 | | |
| 239979_at | 3.34 | 3.33 | epithelial stromal interaction 1 (breast) | EPSTI1 |
| 229309_at | 3.33 | 4.17 | | |
| 201110_s_at | 3.33 | 2.26 | thrombospondin 1 | THBS1 |
| 227195_at | 3.33 | 3.05 | zinc finger protein 503 | ZNF503 |
| 206133_at | 3.32 | 4.00 | | |
| 226603_at | 3.28 | 2.34 | sterile alpha motif domain containing 9-like | SAMD9L |
| 214022_s_at | 3.23 | 2.60 | interferon induced transmembrane protein 1 (9-27) | IFITM1 |
| 208613_s_at | 3.16 | 13.87 | filamin B, beta (actin binding protein 278) | FLNB |
| 217139_at | 3.14 | 2.86 | voltage-dependent anion channel 1 pseudogene | VDAC1P |
| 222717_at | 3.04 | 2.03 | serum deprivation response (phosphatidylserine binding protein) | SDPR |
| 219684_at | 2.99 | 2.42 | receptor (chemosensory) transporter protein 4 | RTP4 |
| 204187_at | 2.99 | 2.22 | guanosine monophosphate reductase | GMPR |
| 200897_s_at | 2.91 | 2.97 | palladin, cytoskeletal associated protein | PALLD |
| 228607_at | 2.86 | 4.28 | 2'-5'-oligoadenylate synthetase 2, 69/71kDa | OAS2 |
| 235643_at | 2.77 | 2.59 | sterile alpha motif domain containing 9-like | SAMD9L |
| 226702_at | 2.74 | 2.57 | | |
| 200907_s_at | 2.72 | 3.08 | palladin, cytoskeletal associated protein | PALLD |
| 243343_at | 2.72 | 2.63 | | |
| 243541_at | 2.71 | 15.05 | interleukin 31 receptor A | IL31RA |
| AFFX-HUMISGF3A/M97935_5_at | 2.71 | 2.41 | signal transducer and activator of transcription 1, 91kDa | STAT1 |
| AFFX-HUMISGF3A/M97935_MB_at | 2.71 | 2.44 | signal transducer and activator of transcription 1, 91kDa | STAT1 |
| 234393_at | 2.68 | 3.89 | histone deacetylase 9 | HDAC9 |
| 208436_s_at | 2.67 | 2.24 | interferon regulatory factor 7 | IRF7 |
| 204720_s_at | 2.66 | 3.13 | DnaJ (Hsp40) homolog, subfamily C, member 6 | DNAJC6 |
| 218758_s_at | 2.64 | 14.82 | | |
| 226034_at | 2.64 | 2.69 | | |
| 214651_s_at | 2.61 | 2.77 | homeobox A9 | HOXA9 |
| 210279_at | 2.53 | 2.61 | G protein-coupled receptor 18 | GPR18 |
| 230803_s_at | 2.53 | 2.78 | Rho GTPase activating protein 24 | ARHGAP2 |

| | | | | |
|---|---|---|---|---|
| 205552_s_at | 2.53 | 3.85 | 2',5'-oligoadenylate synthetase 1, 40/46kDa | OAS1 |
| 214999_s_at | 2.52 | 2.11 | RAB11 family interacting protein 3 (class II) | RAB11FIP 3 |
| 209905_at | 2.51 | 3.43 | homeobox A9 | HOXA9 |
| 1556682_s_at | 2.51 | 2.10 | | |
| 200887_s_at | 2.49 | 2.42 | signal transducer and activator of transcription 1, 91kDa | STAT1 |
| 206847_s_at | 2.48 | 3.73 | homeobox A7 | HOXA7 |
| 1558871_at | 2.40 | 2.79 | | |
| 231798_at | 2.37 | 4.27 | noggin | NOG |
| 220358_at | 2.35 | 3.78 | | |
| 1556747_a_at | 2.30 | 2.41 | | |
| 204015_s_at | 2.28 | 2.55 | dual specificity phosphatase 4 | DUSP4 |
| 213982_s_at | 2.27 | 2.08 | RAB GTPase activating protein 1-like | RABGAP1 L |
| 227915_at | 2.24 | 3.60 | ankyrin repeat and SOCS box-containing 2 | ASB2 |
| 201416_at | 2.21 | 2.70 | SRY (sex determining region Y)-box 4 | SOX4 |
| 200906_s_at | 2.20 | 2.88 | palladin, cytoskeletal associated protein | PALLD |
| 210796_x_at | 2.19 | 2.00 | sialic acid binding Ig-like lectin 6 | SIGLEC6 |
| 225929_s_at | 2.19 | 2.33 | ring finger protein 213 | RNF213 |
| 204256_at | 2.15 | 2.08 | ELOVL family member 6, elongation of long chain fatty acids (FEN1/Elo2, SUR4/Elo3-like, yeast) | ELOVL6 |
| 213294_at | 2.14 | 2.05 | | |
| 1557535_at | 2.13 | 2.59 | palladin, cytoskeletal associated protein | PALLD |
| 231577_s_at | 2.13 | 2.22 | guanylate binding protein 1, interferon-inducible, 67kDa | GBP1 |
| 234306_s_at | 2.05 | 2.15 | SLAM family member 7 | SLAMF7 |
| 239723_at | 2.05 | 3.04 | | |
| 215554_at | -2.60 | -40.92 | glycosylphosphatidylinositol specific phospholipase D1 | GPLD1 |
| 214632_at | -2.01 | -32.43 | neuropilin 2 | NRP2 |
| 1561362_at | -2.30 | -27.49 | | |
| 236220_at | -2.99 | -22.76 | | |
| 228170_at | -4.12 | -17.68 | oligodendrocyte transcription factor 1 | OLIG1 |
| 1555340_x_at | -14.94 | -15.70 | RAP1A, member of RAS oncogene family | RAP1A |
| 1562742_at | -2.18 | -15.59 | | |
| 1568856_at | -2.07 | -12.41 | neighbor of BRCA1 gene 1 | NBR1 |
| 1569782_at | -6.30 | -12.24 | | |
| 214421_x_at | -3.17 | -11.93 | cytochrome P450, family 2, subfamily C, polypeptide 9 | CYP2C9 |
| 1555339_at | -13.69 | -10.85 | RAP1A, member of RAS oncogene family | RAP1A |
| 242199_at | -10.50 | -9.24 | | |
| 232335_at | -2.60 | -8.99 | | |
| 241781_at | -2.93 | -8.90 | chromosome 9 open reading frame 41 | C9orf41 |
| 236197_at | -3.01 | -8.59 | | |
| 1555103_s_at | -59.82 | -8.42 | fibroblast growth factor 7 (keratinocyte growth factor) | FGF7 |
| 1553208_s_at | -2.01 | -8.03 | ADP-ribosylation factor-like 10 | ARL10 |
| 243095_at | -2.54 | -7.89 | | |
| 207651_at | -4.10 | -7.81 | G protein-coupled receptor 171 | GPR171 |
| 244504_x_at | -3.26 | -7.33 | ADP-ribosylation factor 1 | ARF1 |
| 207500_at | -2.39 | -6.95 | caspase 5, apoptosis-related cysteine peptidase | CASP5 |

| | | | | |
|---|---|---|---|---|
| 203868_s_at | -2.15 | -6.58 | vascular cell adhesion molecule 1 | VCAM1 |
| 236304_at | -6.37 | -6.50 | | |
| 240334_at | -16.19 | -6.04 | leucine rich repeat and fibronectin type III domain containing 5 | LRFN5 |
| 206676_at | -2.84 | -6.03 | carcinoembryonic antigen-related cell adhesion molecule 8 | CEACAM8 |
| 233821_at | -2.47 | -5.63 | RAB32, member RAS oncogene family | RAB32 |
| 235756_at | -4.68 | -5.51 | sterile alpha motif domain containing 4A | SAMD4A |
| 1553859_at | -4.17 | -4.78 | tryptophan hydroxylase 1 (tryptophan 5-monooxygenase) | TPH1 |
| 203381_s_at | -2.46 | -4.59 | apolipoprotein E | APOE |
| 217353_at | -2.18 | -4.40 | phenylalanine-tRNA synthetase 2 (mitochondrial) | FARS2 |
| 229228_at | -2.19 | -4.27 | cAMP responsive element binding protein 5 | CREB5 |
| 1553080_at | -4.44 | -4.27 | casein alpha s2-like A | CSN1S2A |
| 225987_at | -4.14 | -4.14 | STEAP family member 4 | STEAP4 |
| 212698_s_at | -2.34 | -4.11 | septin 10 | 10-Sep |
| 1566108_at | -3.52 | -4.07 | myoneurin | MYNN |
| 206363_at | -2.64 | -4.04 | v-maf musculoaponeurotic fibrosarcoma oncogene homolog (avian) | MAF |
| 33197_at | -2.50 | -4.03 | myosin VIIA | MYO7A |
| 209369_at | -2.46 | -3.99 | annexin A3 | ANXA3 |
| 228376_at | -7.43 | -3.99 | glycoprotein, alpha-galactosyltransferase 1 | GGTA1 |
| 206186_at | -2.09 | -3.89 | membrane protein, palmitoylated 3 (MAGUK p55 subfamily member 3) | MPP3 |
| 221464_at | -2.24 | -3.69 | olfactory receptor, family 1, subfamily D, member 2 | OR1D2 |
| 235212_at | -2.61 | -3.67 | chromosome 14 open reading frame 102 | C14orf102 |
| 205831_at | -2.08 | -3.65 | CD2 molecule | CD2 |
| 1569264_at | -2.52 | -3.36 | | |
| 242811_x_at | -2.76 | -3.35 | | |
| 237496_at | -2.79 | -3.31 | 3'-phosphoadenosine 5'-phosphosulfate synthase 2 | PAPSS2 |
| 222301_at | -2.73 | -3.24 | chromosome 1 open reading frame 61 | C1orf61 |
| 1559950_at | -2.64 | -3.22 | | |
| 235380_at | -2.57 | -3.18 | | |
| 224276_at | -2.10 | -3.18 | zinc finger protein 33A | ZNF33A |
| 220338_at | -2.34 | -3.17 | Ral GEF with PH domain and SH3 binding motif 2 | RALGPS2 |
| 226218_at | -2.11 | -3.17 | | |
| 1564760_at | -2.36 | -3.16 | | |
| 1569786_at | -3.87 | -3.15 | | |
| 231067_s_at | -2.24 | -3.14 | A kinase (PRKA) anchor protein (gravin) 12 | AKAP12 |
| 214967_at | -7.48 | -3.14 | | |
| 206371_at | -2.60 | -3.13 | folate receptor 3 (gamma) | FOLR3 |
| 215468_at | -2.55 | -3.12 | | |
| 215306_at | -2.54 | -3.11 | | |
| 205557_at | -2.99 | -3.09 | bactericidal/permeability-increasing protein | BPI |
| 215463_at | -3.27 | -3.08 | olfactory receptor, family 7, subfamily E, member 24 | OR7E24 |
| 201427_s_at | -6.41 | -3.07 | selenoprotein P, plasma, 1 | SEPP1 |
| 200785_s_at | -2.08 | -3.06 | low density lipoprotein-related protein 1 (alpha-2-macroglobulin receptor) | LRP1 |
| 1570354_s_at | -2.21 | -3.05 | zinc finger protein 169 | ZNF169 |
| 210832_x_at | -2.79 | -3.03 | prostaglandin E receptor 3 (subtype EP3) | PTGER3 |

| | | | | |
|---|---|---|---|---|
| 243485_at | -2.51 | -3.01 | | |
| 203936_s_at | -3.08 | -3.01 | matrix metallopeptidase 9 (gelatinase B, 92kDa gelatinase, 92kDa type IV collagenase) | MMP9 |
| 202948_at | -3.42 | -3.00 | interleukin 1 receptor, type I | IL1R1 |
| 218232_at | -2.74 | -2.99 | complement component 1, q subcomponent, A chain | C1QA |
| 201348_at | -2.07 | -2.98 | glutathione peroxidase 3 (plasma) | GPX3 |
| 222877_at | -2.14 | -2.93 | | |
| 1555024_at | -3.58 | -2.92 | ADAM metallopeptidase domain 22 | ADAM22 |
| 220428_at | -3.07 | -2.92 | CD207 molecule, langerin | CD207 |
| 206749_at | -2.23 | -2.91 | CD1b molecule | CD1B |
| 242814_at | -2.97 | -2.91 | serpin peptidase inhibitor, clade B (ovalbumin), member 9 | SERPINB9 |
| 220811_at | -5.84 | -2.91 | proteoglycan 3 | PRG3 |
| 240137_at | -2.82 | -2.90 | | |
| 220880_at | -2.66 | -2.85 | | |
| 221584_s_at | -5.32 | -2.83 | potassium large conductance calcium-activated channel, subfamily M, alpha member 1 | KCNMA1 |
| 214366_s_at | -2.04 | -2.83 | arachidonate 5-lipoxygenase | ALOX5 |
| 225353_s_at | -2.46 | -2.82 | complement component 1, q subcomponent, C chain | C1QC |
| 202283_at | -2.04 | -2.81 | serpin peptidase inhibitor, clade F (alpha-2 antiplasmin, pigment epithelium derived factor), member | SERPINF1 |
| 220963_s_at | -3.32 | -2.73 | chromosome 1 open reading frame 89 | C1orf89 |
| 1558692_at | -2.12 | -2.70 | chromosome 1 open reading frame 85 | C1orf85 |
| 204580_at | -2.33 | -2.68 | matrix metallopeptidase 12 (macrophage elastase) | MMP12 |
| 233282_at | -5.87 | -2.66 | | |
| 203290_at | -2.54 | -2.65 | major histocompatibility complex, class II, DQ alpha 1 | HLA-DQA1 |
| 207113_s_at | -2.04 | -2.63 | tumor necrosis factor (TNF superfamily, member 2) | TNF |
| 213974_at | -2.55 | -2.63 | ADAMTS-like 3 | ADAMTSL3 |
| 236199_at | -2.26 | -2.62 | arachidonate 5-lipoxygenase | ALOX5 |
| 237865_x_at | -4.02 | -2.62 | | |
| 201280_s_at | -2.02 | -2.60 | disabled homolog 2, mitogen-responsive phosphoprotein (Drosophila) | DAB2 |
| 213832_at | -2.05 | -2.58 | | |
| 202953_at | -2.52 | -2.58 | complement component 1, q subcomponent, B chain | C1QB |
| 1555214_a_at | -2.83 | -2.57 | C-type lectin domain family 7, member A | CLEC7A |
| 206729_at | -2.02 | -2.55 | tumor necrosis factor receptor superfamily, membe 8 | TNFRSF8 |
| 227189_at | -2.25 | -2.54 | copine V | CPNE5 |
| 204446_s_at | -2.38 | -2.53 | arachidonate 5-lipoxygenase | ALOX5 |
| 205729_at | -2.90 | -2.51 | oncostatin M receptor | OSMR |
| 201141_at | -2.52 | -2.51 | glycoprotein (transmembrane) nmb | GPNMB |
| 238281_at | -2.25 | -2.48 | RNA binding motif, single stranded interacting protein 1 | RBMS1 |
| 1556175_at | -3.06 | -2.47 | | |
| 228610_at | -3.07 | -2.46 | transmembrane 9 superfamily member 3 | TM9SF3 |
| 234783_at | -2.30 | -2.46 | | |

| | | | | |
|---|---|---|---|---|
| 211734_s_at | -3.11 | -2.44 | Fc fragment of IgE, high affinity I, receptor for; alpha polypeptide | FCER1A |
| 214627_at | -2.58 | -2.44 | eosinophil peroxidase | EPX |
| 205249_at | -2.12 | -2.42 | early growth response 2 (Krox-20 homolog, Drosophila) | EGR2 |
| 202286_s_at | -3.61 | -2.42 | tumor-associated calcium signal transducer 2 | TACSTD2 |
| 223937_at | -2.20 | -2.42 | forkhead box P1 | FOXP1 |
| 243882_at | -3.21 | -2.41 | | |
| 203305_at | -2.11 | -2.41 | coagulation factor XIII, A1 polypeptide | F13A1 |
| 1564640_at | -2.11 | -2.40 | MAX gene associated | MGA |
| 209395_at | -2.07 | -2.38 | chitinase 3-like 1 (cartilage glycoprotein-39) | CHI3L1 |
| 1566696_at | -3.56 | -2.37 | | |
| 201278_at | -2.21 | -2.35 | disabled homolog 2, mitogen-responsive phosphoprotein (Drosophila) | DAB2 |
| 213249_at | -5.33 | -2.35 | F-box and leucine-rich repeat protein 7 | FBXL7 |
| 203473_at | -2.45 | -2.34 | solute carrier organic anion transporter family, member 2B1 | SLCO2B1 |
| 214523_at | -2.69 | -2.34 | CCAAT/enhancer binding protein (C/EBP), epsilon | CEBPE |
| 201212_at | -2.14 | -2.34 | legumain | LGMN |
| 217340_at | -2.17 | -2.33 | leucine rich repeat containing 16 | LRRC16 |
| 1567031_at | -2.07 | -2.30 | zinc finger protein 160 | ZNF160 |
| 1562537_at | -4.01 | -2.30 | Fc fragment of IgE, high affinity I, receptor for; alpha polypeptide | FCER1A |
| 230503_at | -2.43 | -2.30 | sterile alpha motif domain containing 4A | SAMD4A |
| 204445_s_at | -4.56 | -2.30 | arachidonate 5-lipoxygenase | ALOX5 |
| 1570490_at | -2.61 | -2.29 | | |
| 1552991_at | -2.03 | -2.29 | olfactory receptor, family 5, subfamily P, member 2 | OR5P2 |
| 226878_at | -2.23 | -2.28 | major histocompatibility complex, class II, DO alpha | HLA-DOA |
| 215508_at | -2.54 | -2.27 | BUB1 budding uninhibited by benzimidazoles 1 homolog (yeast) | BUB1 |
| 221210_s_at | -2.10 | -2.23 | N-acetylneuraminate pyruvate lyase (dihydrodipicolinate synthase) | NPL |
| 215447_at | -2.02 | -2.23 | tissue factor pathway inhibitor (lipoprotein-associated coagulation inhibitor) | TFPI |
| 1559766_at | -3.67 | -2.23 | | |
| 231366_at | -3.03 | -2.22 | | |
| 228080_at | -3.40 | -2.22 | layilin | LAYN |
| 1558745_at | -2.18 | -2.22 | | |
| 1553708_at | -2.36 | -2.22 | | |
| 221307_at | -4.29 | -2.17 | Kv channel interacting protein 1 | KCNIP1 |
| 1560792_at | -2.34 | -2.16 | | |
| 229242_at | -2.48 | -2.16 | | |
| 228298_at | -2.56 | -2.15 | family with sequence similarity 113, member B | FAM113B |
| 233501_at | -2.22 | -2.15 | | |
| 208488_s_at | -2.06 | -2.14 | complement component (3b/4b) receptor 1 (Knops blood group) | CR1 |
| 237675_at | -5.07 | -2.14 | | |
| 1564765_at | -3.74 | -2.14 | | |
| 210375_at | -2.10 | -2.13 | prostaglandin E receptor 3 (subtype EP3) | PTGER3 |
| 244114_x_at | -2.38 | -2.13 | | |

| | | | | |
|---|---|---|---|---|
| 1554018_at | -3.22 | -2.12 | glycoprotein (transmembrane) nmb | GPNMB |
| 240440_at | -3.25 | -2.11 | N-acetylneuraminate pyruvate lyase (dihydrodipicolinate synthase) | NPL |
| 210690_at | -4.53 | -2.09 | killer cell lectin-like receptor subfamily C, member 4 | KLRC4 |
| 244840_x_at | -3.08 | -2.09 | | |
| 226281_at | -3.04 | -2.07 | delta/notch-like EGF repeat containing | DNER |
| 226436_at | -2.08 | -2.06 | Ras association (RalGDS/AF-6) domain family 4 | RASSF4 |
| 1561316_at | -9.72 | -2.05 | gamma-aminobutyric acid (GABA) A receptor, beta 3 | GABRB3 |
| 1558969_a_at | -2.32 | -2.05 | ribosomal protein L32 pseudogene 3 | RPL32P3 |
| 215459_at | -2.19 | -2.04 | Tax1 (human T-cell leukemia virus type I) binding protein 3 | TAX1BP3 |
| 213221_s_at | -2.08 | -2.02 | SNF1-like kinase 2 | SNF1LK2 |
| 201131_s_at | -7.60 | -2.01 | cadherin 1, type 1, E-cadherin (epithelial) | CDH1 |
| 232858_at | -2.37 | -2.00 | | |
