# Supplementary material for: In Vitro Transformation of Primary Human CD34+ Cells by AML Fusion Oncogenes: Early Gene Expression Profiling Reveals Possible Drug Target in AML
Source: PLoS One. 2010 Aug 27;5(8):e12464. doi: 10.1371/journal.pone.0012464 (PMC2929205; doi:10.1371/journal.pone.0012464)
Supplement: Table S13 — Genes deregulated by AML1-ETO 6 hours after transfection. Primary human CD34+ cells were nucleofected with either control pTracer-CMV/Bsd vector or vector expressing AML1-ETO and sorted for GFP positivity. Total RNA was extracted 6 h after nucleofection and subjected to microarray analysis. Microarray data were analyzed by SAM as described in Materials and Methods. Significantly deregulated genes are listed and the false discovery rate (FDR) is shown. (0.79 MB PDF) [file pone.0012464.s013.pdf]

Table S13. Genes deregulated by AML1-ETO at 6 h detected by SAM FDR = 0.17%

Probe set ID	Fold Change	Gene Name	Gene Symbol
216831_s_at	4348.75	runt-related transcription factor 1; translocated to, 1 (cyclin D-related)	RUNX1T1
1566749_at	78.12		
220005_at	76.50	purinergic receptor P2Y, G-protein coupled, 13	P2RY13
205943_at	76.49	tryptophan 2,3-dioxygenase	TDO2
207623_at	71.28	ATP-binding cassette, sub-family F (GCN20), member 2	ABCF2
235355_at	53.95		
228202_at	46.94	phospholamban	PLN
211181_x_at	42.53	runt-related transcription factor 1 (acute myeloid leukemia 1; aml1 oncogene)	RUNX1
1563053_at	42.11		
204939_s_at	36.84	phospholamban	PLN
1556700_a_at	35.33		
235885_at	34.13		
204938_s_at	31.13	phospholamban	PLN
223395_at	29.72	ABI gene family, member 3 (NESH) binding protein	ABI3BP
1553851_at	28.40	Spi-C transcription factor (Spi-1/PU.1 related)	SPIC
219936_s_at	27.44	G protein-coupled receptor 87	GPR87
221805_at	23.20	neurofilament, light polypeptide 68kDa	NEFL
239947_at	22.18		
1556609_at	21.50		
1557429_s_at	21.28	galactokinase 2	GALK2
220247_at	20.93	cancer susceptibility candidate 5	CASC5
231879_at	20.25	collagen, type XII, alpha 1	COL12A1
237169_at	20.24		
226534_at	19.39	KIT ligand	KITLG
208182_x_at	19.05	interferon, alpha 14	IFNA14
231032_at	18.93		
242204_at	18.78	WAP four-disulfide core domain 5	WFDC5
1552367_a_at	18.72	scinderin	SCIN
1553747_at	18.57		
241579_at	18.51		
206030_at	17.67	aspartoacylase (Canavan disease)	ASPA
214079_at	17.57	dehydrogenase/reductase (SDR family) member 2	DHRS2
243401_at	17.07	formin-like 2	FMNL2
243644_at	16.83		
219106_s_at	16.78	kelch repeat and BTB (POZ) domain containing 10	KBTBD10
240317_at	16.20	protocadherin beta 4	PCDHB4
206381_at	16.18	sodium channel, voltage-gated, type II, alpha subunit	SCN2A
214782_at	15.81	cortactin	CTTN
1568673_s_at	15.80	ELL associated factor 2	EAF2
240512_x_at	15.60	potassium channel tetramerisation domain containing 4	KCTD4
231218_at	15.55		

205513_at	15.48	transcobalamin I (vitamin B12 binding protein, R binder family)	TCN1
242661_x_at	15.19	amyotrophic lateral sclerosis 2 (juvenile) chromosome region, candidate 12	ALS2CR12
1569582_at	15.15		
228547_at	14.77	neurexin 1	NRXN1
238454_at	14.51	zinc finger protein 540	ZNF540
241510_at	14.51		
1560012_at	14.49		
1570031_at	14.24		
237837_at	13.67		
1558502_s_at	13.53	dynamin 3	DNM3
223279_s_at	13.36	uveal autoantigen with coiled-coil domains and ankyrin repeats	UACA
210650_s_at	13.29	piccolo (presynaptic cytomatrix protein)	PCLO
229057_at	13.17	sodium channel, voltage-gated, type II, alpha subunit	SCN2A
204959_at	13.06	myeloid cell nuclear differentiation antigen	MNDA
201939_at	12.51	polo-like kinase 2 (Drosophila)	PLK2
209960_at	12.48	hepatocyte growth factor (hepapoietin A; scatter factor)	HGF
227006_at	12.18	protein phosphatase 1, regulatory (inhibitor) subunit 14A	PPP1R14A
204940_at	12.02	phospholamban	PLN
220301_at	11.99	coiled-coil domain containing 102B	CCDC102B
206254_at	11.83	epidermal growth factor (beta-urogastrone)	EGF
1560676_at	11.70		
211298_s_at	11.61	albumin	ALB
244537_at	11.49	huntingtin interacting protein 2	HIP2
233021_at	11.47		
244009_at	11.27	calcium modulating ligand	CAMLG
239203_at	11.13		
242164_s_at	11.06	leucine-rich repeats and immunoglobulin-like domains 2	LRIG2
211620_x_at	11.03	runt-related transcription factor 1 (acute myeloid leukemia 1; aml1 oncogene)	RUNX1
210119_at	11.01	potassium inwardly-rectifying channel, subfamily J, member 15	KCNJ15
1568711_a_at	10.93		
220311_at	10.92	N-6 adenine-specific DNA methyltransferase 1 (putative)	N6AMT1
231898_x_at	10.80	SOX2 overlapping transcript (non-coding RNA)	SOX2OT
205844_at	10.73	vanin 1	VNN1
244334_at	10.72	translocation associated membrane protein 1-like 1	TRAM1L1
1553402_a_at	10.69	hemochromatosis	HFE
203129_s_at	10.68	kinesin family member 5C	KIF5C
205932_s_at	10.66	msh homeobox 1	MSX1
223962_at	10.66	tetratricopeptide repeat domain 29	TTC29
215388_s_at	10.62	complement factor H-related 1	CFHR1
219872_at	10.57	chromosome 4 open reading frame 18	C4orf18
234847_at	10.56		
235944_at	10.55	hemicentin 1	HMCN1

243060_at	10.45		
1561707_at	10.32		
239864_at	10.19		
230876_at	10.04		
219773_at	10.01	NADPH oxidase 4	NOX4
235490_at	9.89	transmembrane protein 107	TMEM107
205922_at	9.86	vanin 2	VNN2
244436_at	9.82		
207817_at	9.77	interferon, omega 1	IFNW1
1560746_at	9.75	chromosome 1 open reading frame 118	C1orf118
212865_s_at	9.57	collagen, type XIV, alpha 1 (undulin)	COL14A1
207813_s_at	9.57	ferredoxin reductase	FDXR
1567612_at	9.50		
240890_at	9.48		
206463_s_at	9.40	dehydrogenase/reductase (SDR family) member 2	DHRS2
1552957_at	9.23		
1554946_at	9.16		
207517_at	9.16	laminin, gamma 2	LAMC2
243602_at	9.12		
1561200_at	9.10		
201506_at	9.03	transforming growth factor, beta-induced, 68kDa	TGFBI
1562942_at	9.00		
222456_s_at	8.96	LIM domain and actin binding 1	LIMA1
205651_x_at	8.91	Rap guanine nucleotide exchange factor (GEF) 4	RAPGEF4
227819_at	8.84	leucine-rich repeat-containing G protein-coupled receptor 6	LGR6
1553645_at	8.83		
237304_at	8.73	synaptonemal complex central element protein 2	SYCE2
207729_at	8.71	cadherin 9, type 2 (T1-cadherin)	CDH9
203413_at	8.62	NEL-like 2 (chicken)	NELL2
244678_at	8.61	vacuolar protein sorting 13 homolog A (S. cerevisiae)	VPS13A
235825_at	8.57		
227839_at	8.46	methyl-CpG binding domain protein 5	MBD5
223838_at	8.40	testis specific, 10	TSGA10
240413_at	8.39	interferon, gamma-inducible protein 16	IFI16
1554648_a_at	8.31	dual oxidase maturation factor 1	DUOXA1
204273_at	8.29	endothelin receptor type B	EDNRB
238280_at	8.28		
214930_at	8.27	SLIT and NTRK-like family, member 5	SLITRK5
241890_at	8.20		
1562270_at	8.19	Rho guanine nucleotide exchange factor (GEF) 7	ARHGEF7
235063_at	8.14	chromosome 20 open reading frame 196	C20orf196
211832_s_at	8.11	Mdm2, transformed 3T3 cell double minute 2, p53 binding protein (mouse)	MDM2
244146_at	8.08	DTW domain containing 1	DTWD1
217589_at	8.07	RAB40A, member RAS oncogene family	RAB40A
242328_at	8.07	RAB3C, member RAS oncogene family	RAB3C
1558390_at	8.06	zinc finger protein 599	ZNF599
1552783_at	8.05	zinc finger protein 417	ZNF417
241269_at	8.05		

206155_at	7.98	ATP-binding cassette, sub-family C (CFTR/MRP), member 2	ABCC2
207932_at	7.98	interferon, alpha 8	IFNA8
232847_at	7.84	sal-like 3 (Drosophila)	SALL3
204464_s_at	7.67	endothelin receptor type A	EDNRA
205867_at	7.66	protein tyrosine phosphatase, non-receptor type 11 (Noonan syndrome 1)	PTPN11
232868_at	7.65	chromosome 9 open reading frame 11	C9orf11
1553293_at	7.64	MAS-related GPR, member X3	MRGPRX3
229517_at	7.61	protein tyrosine phosphatase domain containing 1	PTPDC1
210920_x_at	7.58	EMI domain containing 2	EMID2
1561657_at	7.58		
220623_s_at	7.57	testis specific, 10	TSGA10
235342_at	7.52	sparc/osteonectin, cwcv and kazal-like domains proteoglycan (testican) 3	SPOCK3
1565614_at	7.40	zinc finger protein 337	ZNF337
244259_s_at	7.39		
223204_at	7.38	chromosome 4 open reading frame 18	C4orf18
219973_at	7.35	arylsulfatase family, member J	ARSJ
243320_at	7.35	chromosome 14 open reading frame 161	C14orf161
238126_at	7.34		
225242_s_at	7.27	coiled-coil domain containing 80	CCDC80
210368_at	7.26	protocadherin gamma subfamily A, 8	PCDHGA8
1563179_at	7.24		
206504_at	7.24	cytochrome P450, family 24, subfamily A, polypeptide 1	CYP24A1
229006_at	7.23		
209841_s_at	7.22	leucine rich repeat neuronal 3	LRRN3
208484_at	7.22	histone cluster 1, H1a	HIST1H1A
219962_at	7.21	angiotensin I converting enzyme (peptidyl-dipeptidase A) 2	ACE2
223557_s_at	7.17	transmembrane protein with EGF-like and two follistatin-like domains 2	TMEFF2
209693_at	7.15	astrotactin 2	ASTN2
239319_at	7.13		
206591_at	7.11	recombination activating gene 1	RAG1
1557733_a_at	7.11		
207712_at	7.11	B melanoma antigen	BAGE
211538_s_at	7.09	heat shock 70kDa protein 2	HSPA2
1555346_at	7.08	cell division cycle 20 homolog B (S. cerevisiae)	CDC20B
232881_at	7.08		
203811_s_at	7.06	DnaJ (Hsp40) homolog, subfamily B, member 4	DNAJB4
1563310_a_at	7.03		
243581_at	6.97		
214073_at	6.93	cortactin	CTTN
1552694_at	6.92	solute carrier family 2 (facilitated glucose transporter), member 13	SLC2A13
235709_at	6.90	growth arrest-specific 2 like 3	GAS2L3
1561965_at	6.89		
206515_at	6.88	cytochrome P450, family 4, subfamily F, polypeptide 3	CYP4F3
230506_at	6.87	chromosome 6 open reading frame 164	C6orf164

236847_at	6.86	chromosome 19 open reading frame 18	C19orf18
1561223_at	6.85		
231391_at	6.84	cortexin 3	CTXN3
1561154_at	6.82		
227791_at	6.81	solute carrier family 9 (sodium/hydrogen exchanger), member 9	SLC9A9
230147_at	6.81	coagulation factor II (thrombin) receptor-like 2	F2RL2
236300_at	6.80		
230169_at	6.80	THAP domain containing 6	THAP6
224520_s_at	6.79	bestrophin 3	BEST3
219148_at	6.78	PDZ binding kinase	PBK
219532_at	6.78	elongation of very long chain fatty acids (FEN1/Elo2, SUR4/Elo3, yeast)-like 4	ELOVL4
231709_x_at	6.77		
223735_at	6.76	ADP-ribosylation factor-like 6	ARL6
227662_at	6.76	synaptopodin 2	SYNPO2
206655_s_at	6.75	glycoprotein Ib (platelet), beta polypeptide	GP1BB
234374_at	6.74		
206721_at	6.71	chromosome 1 open reading frame 114	C1orf114
239825_at	6.70	activating transcription factor 6	ATF6
242093_at	6.70	synaptotagmin-like 5	SYTL5
224020_at	6.70		
1560483_at	6.63		
209465_x_at	6.61	pleiotrophin (heparin binding growth factor 8, neurite growth-promoting factor 1)	PTN
233238_s_at	6.60		
215300_s_at	6.59	flavin containing monooxygenase 5	FMO5
224109_at	6.57		
1556812_a_at	6.54		
232956_at	6.54		
206726_at	6.54		
220115_s_at	6.52	cadherin 10, type 2 (T2-cadherin)	CDH10
243056_at	6.50	chromosome 12 open reading frame 60	C12orf60
203038_at	6.47	protein tyrosine phosphatase, receptor type, K	PTPRK
212624_s_at	6.47	chimerin (chimaerin) 1	CHN1
1558135_at	6.45	TAF11 RNA polymerase II, TATA box binding protein (TBP)-associated factor, 28kDa	TAF11
206331_at	6.45	calcitonin receptor-like	CALCRL
230130_at	6.43	slit homolog 2 (Drosophila)	SLIT2
206291_at	6.40	neurotensin	NTS
1557991_at	6.38	methyltransferase like 6	METTL6
243273_at	6.38		
1556817_a_at	6.36		
211182_x_at	6.34	runt-related transcription factor 1 (acute myeloid leukemia 1; aml1 oncogene)	RUNX1
240161_s_at	6.32	cell division cycle 20 homolog B (S. cerevisiae)	CDC20B
240351_at	6.31		
1559591_s_at	6.31	choline dehydrogenase	CHDH
1568615_a_at	6.30		
238844_s_at	6.28	nephronophthisis 1 (juvenile)	NPHP1
216456_at	6.28		
213423_x_at	6.27	tumor suppressor candidate 3	TUSC3
219873_at	6.27	collectin sub-family member 11	COLEC11

207202_s_at	6.24	nuclear receptor subfamily 1, group I, member 2	NR1I2
208173_at	6.23	interferon, beta 1, fibroblast	IFNB1
1552554_a_at	6.23	NLR family, CARD domain containing 4	NLRC4
241491_at	6.22		
243272_at	6.21		
238720_at	6.19	oligodendrocyte myelin glycoprotein	OMG
204894_s_at	6.18	amine oxidase, copper containing 3 (vascular adhesion protein 1)	AOC3
206932_at	6.16	cholesterol 25-hydroxylase	CH25H
233871_at	6.16		
243847_at	6.14		
230957_at	6.14		
240395_at	6.13		
244119_at	6.13		
1563460_at	6.13		
215796_at	6.11	T cell receptor alpha locus	TRA@
239202_at	6.07		
222376_at	6.07		
231581_at	6.06		
210121_at	6.06	UDP-Gal:betaGlcNAc beta 1,3-galactosyltransferase, polypeptide 2	B3GALT2
231400_s_at	6.05	translocase of inner mitochondrial membrane 22 homolog (yeast)	TIMM22
211341_at	6.05	POU domain, class 4, transcription factor 1	POU4F1
1556867_at	6.02		
243542_at	6.01		
209840_s_at	5.99	leucine rich repeat neuronal 3	LRRN3
244734_at	5.99		
1553506_at	5.99	carboxypeptidase O	CPO
221200_at	5.98		
219738_s_at	5.97	protocadherin 9	PCDH9
1570284_x_at	5.96		
237058_x_at	5.94	solute carrier family 6 (neurotransmitter transporter, GABA), member 13	SLC6A13
219274_at	5.94	tetraspanin 12	TSPAN12
206864_s_at	5.93	harakiri, BCL2 interacting protein (contains only BH3 domain)	HRK
1554706_at	5.90	olfactory receptor, family 2, subfamily L, member 13	OR2L13
216239_at	5.89	Bardet-Biedl syndrome 9	BBS9
1555112_a_at	5.88	chromosome 1 open reading frame 114	C1orf114
1554018_at	5.83	glycoprotein (transmembrane) nmb	GPNMB
235675_at	5.76	dihydrofolate reductase-like 1	DHFRL1
237891_at	5.75	Mdm2, transformed 3T3 cell double minute 2, p53 binding protein (mouse)	MDM2
206914_at	5.74	cytotoxic and regulatory T cell molecule	CRTAM
1565602_at	5.73		
238578_at	5.72	transmembrane protein 182	TMEM182
219655_at	5.71	chromosome 7 open reading frame 10	C7orf10
231260_at	5.70		
207315_at	5.68	CD226 molecule	CD226
203810_at	5.66	DnaJ (Hsp40) homolog, subfamily B, member 4	DNAJB4
230327_at	5.64		

205698_s_at	5.64	mitogen-activated protein kinase kinase 6	MAP2K6
226103_at	5.63	nexilin (F actin binding protein)	NEXN
244257_at	5.63	transmembrane protein 104	TMEM104
243222_at	5.62		
216344_at	5.62	nephronophthisis 4	NPHP4
234363_at	5.61	olfactory receptor, family 2, subfamily F, member 1#olfactory receptor, family 6, subfamily B, member 1#olfactory receptor, family 2, subfamily F, member 2	OR2F1#OR6B 1#OR2F2
230665_at	5.59		
232695_at	5.59	kinesin family member 6	KIF6
237070_at	5.58	transient receptor potential cation channel, subfamily M, member 1	TRPM1
210815_s_at	5.55	calcitonin receptor-like	CALCRL
1558365_at	5.54		
206361_at	5.53	G protein-coupled receptor 44	GPR44
220436_at	5.52	contactin associated protein-like 3B	CNTNAP3B
206214_at	5.50	phospholipase A2, group VII (platelet-activating factor acetylhydrolase, plasma)	PLA2G7
1561125_at	5.49	methylenetetrahydrofolate dehydrogenase (NADP+ dependent) 1-like	MTHFD1L
229065_at	5.48	solute carrier family 35, member F3	SLC35F3
234641_at	5.47		
239043_at	5.45	zinc finger protein 404	ZNF404
212104_s_at	5.45	RNA binding motif protein 9	RBM9
233591_at	5.43		
210127_at	5.43	RAB6B, member RAS oncogene family	RAB6B
208375_at	5.42	interferon, alpha 1	IFNA1
212224_at	5.42	aldehyde dehydrogenase 1 family, member A1	ALDH1A1
238756_at	5.42		
220497_at	5.41	zinc finger protein 214	ZNF214
1563495_at	5.40	solute carrier family 9, member 11	SLC9A11
231217_at	5.38		
242792_at	5.36	nuclear factor I/B	NFIB
219737_s_at	5.35	protocadherin 9	PCDH9
234393_at	5.35	histone deacetylase 9	HDAC9
210182_at	5.35	cortistatin	CORT
237540_at	5.34		
205856_at	5.34	solute carrier family 14 (urea transporter), member 1 (Kidd blood group)	SLC14A1
1562190_at	5.34		
208121_s_at	5.33	protein tyrosine phosphatase, receptor type, O	PTPRO
242601_at	5.31		
238228_at	5.31	component of oligomeric golgi complex 3	COG3
211104_s_at	5.30	myosin VIIA	MYO7A
1556158_at	5.27		
1559322_at	5.26		
233876_at	5.26		
1561997_at	5.25		
205618_at	5.24	proline rich Gla (G-carboxyglutamic acid) 1	PRRG1
229292_at	5.23	erythrocyte membrane protein band 4.1 like 5	EPB41L5
217452_s_at	5.23	UDP-Gal:betaGlcNAc beta 1,3-galactosyltransferase, polypeptide 2	B3GALT2

238488_at	5.22	importin 11	IPO11
208183_at	5.22	tachykinin receptor 3	TACR3
229360_at	5.21	suppressor of hairy wing homolog 2 (Drosophila)	SUHW2
1555412_at	5.19	F-box and leucine-rich repeat protein 21	FBXL21
1569571_at	5.19	lipase-like, ab-hydrolase domain containing 1	LIPL1
211192_s_at	5.18	CD84 molecule	CD84
1555677_s_at	5.16	structural maintenance of chromosomes 1A	SMC1A
1552521_a_at	5.16	transmembrane protein 74	TMEM74
232549_at	5.15	RNA binding motif protein 11	RBM11
238919_at	5.15		
240073_at	5.14	male-specific lethal 2-like 1 (Drosophila)	MSL2L1
215228_at	5.13	nescient helix loop helix 2	NHLH2
224322_at	5.12	AT rich interactive domain 4B (RBP1-like)	ARID4B
228680_at	5.12	kinesin family member 3A	KIF3A
205352_at	5.12	serpin peptidase inhibitor, clade I (neuroserpin), member 1	SERPINI1
236711_at	5.12	Zic family member 4	ZIC4
244571_s_at	5.11	tetratricopeptide repeat domain 12	TTC12
226467_at	5.11	transmembrane and coiled-coil domains 7	TMCO7
213820_s_at	5.11	START domain containing 5	STARD5
233314_at	5.10	phosphatase and tensin homolog (mutated in multiple advanced cancers 1)	PTEN
1557765_at	5.10		
211909_x_at	5.09	prostaglandin E receptor 3 (subtype EP3)	PTGER3
240823_at	5.08		
206204_at	5.08	growth factor receptor-bound protein 14	GRB14
1567078_x_at	5.08		
235952_at	5.08		
229964_at	5.07	chromosome 9 open reading frame 152	C9orf152
200974_at	5.06	actin, alpha 2, smooth muscle, aorta	ACTA2
1561887_at	5.05		
231131_at	5.05		
230708_at	5.04	prickle homolog 1 (Drosophila)	PRICKLE1
204708_at	5.04	mitogen-activated protein kinase 4	MAPK4
243483_at	5.02	transient receptor potential cation channel, subfamily M, member 8	TRPM8
1561763_at	5.01		
1559950_at	5.00		
231738_at	4.99	protocadherin beta 7	PCDHB7
232506_s_at	4.99	chromosome 15 open reading frame 41	C15orf41
230307_at	4.97	solute carrier family 25 (mitochondrial oxodicarboxylate carrier), member 21	SLC25A21
1564471_at	4.96		
240465_at	4.96		
223799_at	4.93	KIAA1826	KIAA1826
231138_at	4.93		
229778_at	4.93	chromosome 12 open reading frame 39	C12orf39
226436_at	4.92	Ras association (RalGDS/AF-6) domain family 4	RASSF4
1568807_a_at	4.91		
1554744_at	4.91		
215072_x_at	4.90	chromosome 10 open reading frame 137	C10orf137
205894_at	4.90	arylsulfatase E (chondrodysplasia punctata 1)	ARSE
215318_at	4.90		

1553655_at	4.89	cell division cycle 20 homolog B (S. cerevisiae)	CDC20B
204555_s_at	4.88	protein phosphatase 1, regulatory (inhibitor) subunit 3D	PPP1R3D
220330_s_at	4.88	SAM domain, SH3 domain and nuclear localization signals 1	SAMSN1
208498_s_at	4.87	amylase, alpha 1A; salivary	AMY1A
233295_at	4.87		
228964_at	4.84	PR domain containing 1, with ZNF domain	PRDM1
222184_at	4.84		
203821_at	4.83	heparin-binding EGF-like growth factor	HBEGF
202668_at	4.82	ephrin-B2	EFNB2
243712_at	4.80	X (inactive)-specific transcript	XIST
237663_at	4.79		
215376_at	4.78		
227336_at	4.78	deltex homolog 1 (Drosophila)	DTX1
241805_at	4.78	gamma-aminobutyric acid (GABA) A receptor, gamma 1	GABRG1
232271_at	4.76	hepatocyte nuclear factor 4, gamma	HNF4G
210118_s_at	4.76	interleukin 1, alpha	IL1A
1560258_a_at	4.76		
206249_at	4.75	mitogen-activated protein kinase kinase kinase 13	MAP3K13
1565925_at	4.73		
237657_at	4.73	PHD finger protein 21B	PHF21B
244290_at	4.72		
234799_at	4.72	adenosine deaminase, RNA-specific, B1 (RED1 homolog rat)	ADARB1
243610_at	4.70	chromosome 9 open reading frame 135	C9orf135
232829_at	4.70	olfactory receptor, family 52, subfamily K, member 3 pseudogene	OR52K3P
242247_at	4.70	methyltransferase 5 domain containing 1	METT5D1
214146_s_at	4.69	pro-platelet basic protein (chemokine (C-X-C motif) ligand 7)	PPBP
224362_at	4.68		
1562326_at	4.68		
228290_at	4.68	chromosome 20 open reading frame 19	C20orf19
230032_at	4.67		
236087_at	4.67	actin binding LIM protein family, member 2	ABLIM2
234611_at	4.66		
1561650_s_at	4.64		
230450_at	4.63		
204796_at	4.62	echinoderm microtubule associated protein like 1	EML1
1561663_at	4.62		
222717_at	4.61	serum deprivation response (phosphatidylserine binding protein)	SDPR
1556521_a_at	4.61		
235846_at	4.61		
217428_s_at	4.60	collagen, type X, alpha 1(Schmid metaphyseal chondrodysplasia)	COL10A1
205046_at	4.60	centromere protein E, 312kDa	CENPE
207653_at	4.59	forkhead box D2	FOXD2
212915_at	4.59	PDZ domain containing RING finger 3	PDZRN3
1569403_at	4.58		

241399_at	4.57	family with sequence similarity 19 (chemokine (C-C motif)-like), member A2	FAM19A2
234532_at	4.56		
201610_at	4.56	isoprenylcysteine carboxyl methyltransferase	ICMT
227197_at	4.54		
1556123_a_at	4.54		
219786_at	4.53	metallothionein-like 5, testis-specific (tesmin)	MTL5
1556287_a_at	4.53		
242178_at	4.52	lipase, member I	LIPI
1564706_s_at	4.52	glutaminase 2 (liver, mitochondrial)	GLS2
236442_at	4.51	D4, zinc and double PHD fingers, family 3	DPF3
213169_at	4.51		
218769_s_at	4.50	ankyrin repeat, family A (RFXANK-like), 2	ANKRA2
215422_at	4.50		
208577_at	4.49	histone cluster 1, H3c	HIST1H3C
1561113_at	4.49		
228946_at	4.49	inturned planar cell polarity effector homolog (Drosophila)	INTU
211819_s_at	4.49	sorbin and SH3 domain containing 1	SORBS1
206417_at	4.49	cyclic nucleotide gated channel alpha 1	CNGA1
240841_at	4.49	insulinoma-associated 2	INSM2
233163_at	4.48		
230804_at	4.48	chromosome 6 open reading frame 194	C6orf194
230520_at	4.48	androgen-induced 1	AIG1
228598_at	4.47	dipeptidyl-peptidase 10	DPP10
226966_at	4.46	PRP40 pre-mRNA processing factor 40 homolog B (S. cerevisiae)	PRPF40B
207495_at	4.46	RAB28, member RAS oncogene family	RAB28
244786_at	4.45	small nucleolar RNA host gene (non-protein coding) 10	SNHG10
1557029_at	4.44		
1554524_a_at	4.43	olfactomedin 3	OLFM3
1556695_a_at	4.41		
209030_s_at	4.41	cell adhesion molecule 1	CADM1
208300_at	4.41	protein tyrosine phosphatase, receptor type, H	PTPRH
231119_at	4.40	replication factor C (activator 1) 3, 38kDa	RFC3
237127_at	4.39		
232125_at	4.39		
233553_at	4.38		
1569599_at	4.38	SAM domain, SH3 domain and nuclear localization signals 1	SAMSN1
1569607_s_at	4.38		
1552309_a_at	4.37	nexilin (F actin binding protein)	NEXN
205100_at	4.37	glutamine-fructose-6-phosphate transaminase 2	GFPT2
231190_at	4.37		
213558_at	4.37	piccolo (presynaptic cytomatrix protein)	PCLO
204602_at	4.36	dickkopf homolog 1 (Xenopus laevis)	DKK1
1553355_at	4.34		
213623_at	4.32	kinesin family member 3A	KIF3A
1552837_at	4.31	intestinal cell (MAK-like) kinase	ICK

215841_at	4.31	guanylate cyclase activator 1A (retina)#guanylate cyclase activator 1B (retina)#mitochondrial ribosomal protein S10#transcriptional regulating factor 1	GUCA1A#GUCA1B#MRPS10#TRERF1
234382_x_at	4.31		
204149_s_at	4.31	glutathione S-transferase M4	GSTM4
237578_at	4.31		
213929_at	4.30		
242638_at	4.30		
1558193_at	4.29		
213901_x_at	4.29	RNA binding motif protein 9	RBM9
225548_at	4.29	shroom family member 3	SHROOM3
1558107_at	4.27	chromosome 20 open reading frame 80	C20orf80
215551_at	4.26	estrogen receptor 1	ESR1
225283_at	4.25	arrestin domain containing 4	ARRDC4
210755_at	4.25	hepatocyte growth factor (hepapoietin A; scatter factor)	HGF
241823_at	4.24		
219361_s_at	4.23	interferon stimulated exonuclease gene 20kDa-like 1	ISG20L1
1558383_at	4.23		
238450_at	4.22	6-phosphofructo-2-kinase/fructose-2,6-biphosphatase 2	PFKFB2
203998_s_at	4.22	synaptotagmin I	SYT1
235154_at	4.22	TAF3 RNA polymerase II, TATA box binding protein (TBP)-associated factor, 140kDa	TAF3
1553772_at	4.21	glycerol kinase 5 (putative)	GK5
202274_at	4.21	actin, gamma 2, smooth muscle, enteric	ACTG2
235431_s_at	4.20	pellino homolog 3 (Drosophila)	PELI3
232295_at	4.19	G elongation factor, mitochondrial 1	GFM1
232146_at	4.19	NADH dehydrogenase (ubiquinone) 1, subcomplex unknown, 1, 6kDa	NDUFC1
243057_at	4.18		
244176_at	4.18		
203889_at	4.18	secretogranin V (7B2 protein)	SCG5
220369_at	4.16	SMEK homolog 1, suppressor of mek1 (Dictyostelium)	SMEK1
233490_at	4.16	dynactin 4 (p62)	DCTN4
217678_at	4.15	solute carrier family 7, (cationic amino acid transporter, y+ system) member 11	SLC7A11
235320_at	4.15	ADP-ribosylation factor-like 6	ARL6
230789_at	4.15	suppressor of hairy wing homolog 2 (Drosophila)	SUHW2
228034_x_at	4.15	alkB, alkylation repair homolog 5 (E. coli)	ALKBH5
227939_s_at	4.15		
230451_at	4.15	chromosome 1 open reading frame 136	C1orf136
236548_at	4.14		
207267_s_at	4.13	Down syndrome critical region gene 6	DSCR6
232988_at	4.13	KIAA0182	KIAA0182
206091_at	4.13	matrilin 3	MATN3
1558501_at	4.13	dynamin 3	DNM3
223879_s_at	4.13	oxidation resistance 1	OXR1

243655_x_at	4.12	SWI/SNF related, matrix associated, actin dependent regulator of chromatin, subfamily a, member 4	SMARCA4
227488_at	4.11		
210387_at	4.10	histone cluster 1, H2bg	HIST1H2BG
207523_at	4.10	chromosome 6 open reading frame 10	C6orf10
240436_at	4.10		
241420_at	4.10		
1569948_at	4.10		
238867_at	4.10	transmembrane protein 182	TMEM182
214148_at	4.10		
239002_at	4.09	asp (abnormal spindle) homolog, microcephaly associated (Drosophila)	ASPM
221239_s_at	4.09	Fc receptor-like 2	FCRL2
205514_at	4.09	zinc finger protein 415	ZNF415
226733_at	4.09	6-phosphofructo-2-kinase/fructose-2,6-biphosphatase 2	PFKFB2
226528_at	4.09	metaxin 3	MTX3
234996_at	4.08		
220631_at	4.08	O-sialoglycoprotein endopeptidase-like 1	OSGEPL1
221296_at	4.08	tectorin alpha	TECTA
210932_s_at	4.08	ring finger protein (C3H2C3 type) 6	RNF6
241772_at	4.07		
1555263_at	4.07		
238962_at	4.07		
1556090_at	4.06		
216856_s_at	4.06	deleted in lymphocytic leukemia, 2	DLEU2
231091_x_at	4.05		
1552971_at	4.05	sarcoglycan zeta	SGCZ
1552705_at	4.05	dual specificity phosphatase 19	DUSP19
1561219_x_at	4.05		
236504_x_at	4.04	chromosome 6 open reading frame 52	C6orf52
1556238_at	4.04		
1566600_at	4.03		
234597_at	4.03		
229147_at	4.03		
1568627_at	4.03	SMEK homolog 2, suppressor of mek1 (Dictyostelium)	SMEK2
220803_at	4.03	STAM binding protein-like 1	STAMBPL1
240019_at	4.01	neural precursor cell expressed, developmentally down-regulated 9	NEDD9
233259_at	4.01	coiled-coil domain containing 48	CCDC48
244404_at	4.01		
232751_at	4.01	Sec23 homolog B (S. cerevisiae)#polymerase (RNA) III (DNA directed) polypeptide F, 39 kDa#retinoblastoma binding protein 9#chromosome 20 open reading frame 12#ribosomal protein S19 pseudogene 1#ribosomal protein L21 pseudogene 3#glucosaminyl (N-acetyl) transferase 1, core 2 (beta-1,6-N-acetylglucosaminyltransferase) pseudogene	SEC23B#POLR3F#RBBP9#C20orf12#RPS19P1#RPL21P3#GCNT1P
228850_s_at	4.01	slit homolog 2 (Drosophila)	SLIT2

209211_at	4.00	Kruppel-like factor 5 (intestinal)	KLF5
1562342_at	3.99		
1565131_x_at	3.99		
237894_at	3.99	chromosome 3 open reading frame 22	C3orf22
1552365_at	3.99	scinderin	SCIN
211560_s_at	3.98	aminolevulinate, delta-, synthase 2 (sideroblastic/hypochromic anemia)	ALAS2
243586_at	3.98	phosphodiesterase 4D, cAMP-specific (phosphodiesterase E3 dunce homolog, Drosophila)	PDE4D
235764_at	3.97		
231438_x_at	3.96		
1559587_at	3.96	symplekin	SYMPK
237485_at	3.96		
1569147_at	3.95		
1557021_s_at	3.95		
206716_at	3.95	uromodulin (uromucoid, Tamm-Horsfall glycoprotein)	UMOD
233452_at	3.94		
241340_at	3.94		
239710_at	3.94		
229975_at	3.92		
240287_at	3.92		
208344_x_at	3.92	interferon, alpha 13	IFNA13
220719_at	3.92		
1562387_at	3.91		
214189_s_at	3.91	golgi associated, gamma adaptin ear containing, ARF binding protein 2	GGA2
234628_at	3.91		
1555003_at	3.91	retinoblastoma-like 1 (p107)	RBL1
232523_at	3.90	multiple EGF-like-domains 10	MEGF10
237681_at	3.90		
220047_at	3.89	sirtuin (silent mating type information regulation 2 homolog) 4 (S. cerevisiae)	SIRT4
225420_at	3.89	glycerol-3-phosphate acyltransferase, mitochondrial	GPAM
233731_at	3.89		
236634_at	3.88	chromosome 8 open reading frame 48	C8orf48
226341_at	3.87		
1566256_s_at	3.86		
241437_s_at	3.86	EP400 N-terminal like	EP400NL
232517_s_at	3.85	eukaryotic translation elongation factor 1 alpha 2#potassium voltage-gated channel, KQT-like subfamily, member 2#potassium voltage-gated channel, KQT-like subfamily, member 2#PTK6 protein tyrosine kinase 6#src-related kinase lacking C-terminal regulatory tyrosine and N-terminal myristylation sites#chromosome 20 open reading frame 195#chromosome 20 open reading frame 149#null	EEF1A2#KCNQ2#KCNQ2#PTK6#SRMS#C20orf195#C20orf149#null
244424_at	3.85		
1555471_a_at	3.84	formin 2	FMN2
241149_at	3.83		

1559481_at	3.83	cysteine-rich hydrophobic domain 1	CHIC1
244705_at	3.82		
1554161_at	3.81	solute carrier family 25, member 27	SLC25A27
230539_at	3.81		
222245_s_at	3.80	fer-1-like 4 (C. elegans)	FER1L4
208529_at	3.80		
227306_at	3.80		
233549_at	3.80	phosphodiesterase 1A, calmodulin-dependent	PDE1A
235638_at	3.79	Ras association (RalGDS/AF-6) domain family 6	RASSF6
234657_at	3.79		
244171_at	3.79	muskelin 1, intracellular mediator containing kelch motifs	MKLN1
244021_at	3.79		
236495_at	3.79		
206978_at	3.78	chemokine (C-C motif) receptor 2	CCR2
231342_at	3.78		
223854_at	3.78	protocadherin beta 10	PCDHB10
229072_at	3.78		
227842_at	3.78	RAB30, member RAS oncogene family	RAB30
231513_at	3.78	potassium inwardly-rectifying channel, subfamily J, member 2	KCNJ2
206727_at	3.78	complement component 9	C9
1568589_at	3.77		
37547_at	3.76	Bardet-Biedl syndrome 9	BBS9
243525_at	3.76		
219895_at	3.76	family with sequence similarity 70, member A	FAM70A
214034_at	3.76		
241298_x_at	3.75		
1564274_at	3.75	chromosome 9 open reading frame 47	C9orf47
232604_at	3.75	zinc finger protein 541	ZNF541
1553024_at	3.75		
1566047_at	3.74	DEAH (Asp-Glu-Ala-His) box polypeptide 29	DHX29
206271_at	3.74	toll-like receptor 3	TLR3
1553674_at	3.74	leucine rich repeat containing 44	LRRC44
220145_at	3.74	microtubule-associated protein 9	MAP9
216350_s_at	3.74	zinc finger protein 10	ZNF10
1558280_s_at	3.74		
241541_at	3.73	mindbomb homolog 2 (Drosophila)	MIB2
1553296_at	3.73	G protein-coupled receptor 128	GPR128
236076_at	3.73		
216748_at	3.72	pyrin and HIN domain family, member 1	PYHIN1
237845_at	3.72		
203304_at	3.71	BMP and activin membrane-bound inhibitor homolog (Xenopus laevis)	BAMBI
236862_at	3.70	golgi associated PDZ and coiled-coil motif containing	GOPC
222953_at	3.70	G protein-coupled receptor 83	GPR83
232316_at	3.70	chromosome 14 open reading frame 174	C14orf174
1552826_at	3.69	solute carrier family 26, member 7	SLC26A7
222915_s_at	3.69	B-cell scaffold protein with ankyrin repeats 1	BANK1
204325_s_at	3.69	neurofibromin 1 (neurofibromatosis, von Recklinghausen disease, Watson disease)	NF1
214169_at	3.68	unc-84 homolog A (C. elegans)	UNC84A

242557_at	3.68	chromosome 6 open reading frame 12	C6orf12
240389_at	3.68	transient receptor potential cation channel, subfamily M, member 6	TRPM6
1569183_a_at	3.68	choroideremia (Rab escort protein 1)	CHM
233031_at	3.68	zinc finger homeobox 1b	ZFHX1B
203377_s_at	3.68	cell division cycle 40 homolog (S. cerevisiae)	CDC40
1553646_at	3.68	chromosome X open reading frame 43	CXorf43
239944_at	3.68		
232071_at	3.68	chromosome 2 open reading frame 3	C2orf3
231585_at	3.67	vacuolar protein sorting 13 homolog A (S. cerevisiae)	VPS13A
225687_at	3.67	family with sequence similarity 83, member D	FAM83D
216737_at	3.67		
1554328_at	3.67	syntaxin binding protein 4	STXBP4
1556735_at	3.66		
228602_at	3.66	sarcoglycan, delta (35kDa dystrophin-associated glycoprotein)	SGCD
1560814_a_at	3.66	coiled-coil domain containing 32	CCDC32
214900_at	3.66	alpha-2-glycoprotein 1, zinc-binding#zinc finger with KRAB and SCAN domains 1	AZGP1#ZKSCAN1
1563512_at	3.66	nitric oxide synthase 1 (neuronal) adaptor protein	NOS1AP
1570048_at	3.66	DPH4, JJJ3 homolog (S. cerevisiae)	DPH4
237695_at	3.66		
1557132_at	3.66	WD repeat domain 17	WDR17
210763_x_at	3.66	natural cytotoxicity triggering receptor 3	NCR3
208261_x_at	3.66	interferon, alpha 10	IFNA10
215578_at	3.66		
244165_at	3.65	chromosome 10 open reading frame 18	C10orf18
1569728_at	3.63		
1558452_at	3.63	transmembrane protein 144	TMEM144
1553954_at	3.63	asparagine-linked glycosylation 14 homolog (S. cerevisiae)	ALG14
227963_at	3.63		
1561828_at	3.63		
1559036_at	3.62		
209782_s_at	3.62	D site of albumin promoter (albumin D-box) binding protein	DBP
241880_x_at	3.61		
242433_at	3.61	zinc finger and BTB domain containing 11	ZBTB11
237269_at	3.60		
205433_at	3.60	butyrylcholinesterase	BCHE
230374_at	3.59		
222434_at	3.59	enabled homolog (Drosophila)	ENAH
1558945_s_at	3.58	calcium channel, voltage-dependent, P/Q type, alpha 1A subunit	CACNA1A
1558474_at	3.58		
201667_at	3.58	gap junction protein, alpha 1, 43kDa	GJA1
201733_at	3.58	chloride channel 3	CLCN3
1552445_a_at	3.57	extraembryonic, spermatogenesis, homeobox 1 homolog (mouse)	ESX1
223691_at	3.57	regulator of G-protein signalling 22	RGS22
219038_at	3.57	MORC family CW-type zinc finger 4	MORC4
235528_at	3.57	guanylate cyclase activator 1B (retina)	GUCA1B

236990_at	3.57		
237563_s_at	3.56		
226390_at	3.56	START domain containing 4, sterol regulated	STARD4
37117_at	3.56	PHD finger protein 21B	PHF21B
223672_at	3.56	SH3-domain GRB2-like (endophilin) interacting protein 1	SGIP1
1570119_at	3.56	androgen-induced proliferation inhibitor	APRIN
1560017_at	3.55	transmembrane and tetratricopeptide repeat containing 3	TMTC3
243352_at	3.55	alpha-kinase 1	ALPK1
226301_at	3.55	chromosome 6 open reading frame 192	C6orf192
1558391_s_at	3.54	zinc finger protein 599	ZNF599
231947_at	3.54	myc target 1	MYCT1
241026_at	3.54	ADAM metallopeptidase domain 12 (meltrin alpha)	ADAM12
240065_at	3.54	family with sequence similarity 81, member B	FAM81B
240865_at	3.53		
239370_at	3.53		
214534_at	3.53	histone cluster 1, H1b	HIST1H1B
228863_at	3.53	protocadherin 17	PCDH17
243936_x_at	3.53		
220351_at	3.52	chemokine (C-C motif) receptor-like 1	CCRL1
224724_at	3.52	sulfatase 2	SULF2
237670_at	3.51		
243930_x_at	3.51		
228347_at	3.51	sine oculis homeobox homolog 1 (Drosophila)	SIX1
218647_s_at	3.51	yrdC domain containing (E. coli)	YRDC
220813_at	3.50	cysteinyl leukotriene receptor 2	CYSLTR2
206648_at	3.49	zinc finger protein 571	ZNF571
239363_at	3.49		
241703_at	3.49		
242721_at	3.49	autism susceptibility candidate 2	AUTS2
220659_s_at	3.48	chromosome 7 open reading frame 43	C7orf43
241547_at	3.48		
232063_x_at	3.48	phenylalanine-tRNA synthetase-like, beta subunit	FARSLB
1565065_at	3.48	orofacial cleft 1 candidate 1	OFCC1
1557818_x_at	3.48		
231042_s_at	3.48		
242767_at	3.47	LIM and cysteine-rich domains 1	LMCD1
1564466_at	3.47		
235947_at	3.47		
206830_at	3.46	solute carrier family 4, sodium bicarbonate transporter-like, member 10	SLC4A10
244238_at	3.46		
240884_at	3.46	coiled-coil domain containing 14	CCDC14
216279_at	3.45	zinc finger protein 460	ZNF460
203854_at	3.44	complement factor I	CFI
216104_at	3.44		
209921_at	3.44	solute carrier family 7, (cationic amino acid transporter, y+ system) member 11	SLC7A11
227786_at	3.44	thyroid hormone receptor associated protein 6	THRAP6
1552427_at	3.44	zinc finger protein 485	ZNF485
227332_at	3.44		

1555638_a_at	3.43	SAM domain, SH3 domain and nuclear localization signals 1	SAMSN1
235608_at	3.43		
233011_at	3.43	annexin A1	ANXA1
1569953_at	3.43		
232385_x_at	3.42		
215779_s_at	3.42	histone cluster 1, H2bg	HIST1H2BG
239422_at	3.42	glypican 2 (cerebroglycan)	GPC2
231050_at	3.42	HRAS-like suppressor family, member 5	HRASLS5
223611_s_at	3.42	ligand of numb-protein X 1	LNX1
239995_at	3.42		
239199_at	3.42		
223834_at	3.41	CD274 molecule	CD274
201791_s_at	3.41	7-dehydrocholesterol reductase	DHCR7
210821_x_at	3.41	centromere protein A	CENPA
234274_at	3.41		
227765_at	3.41		
224175_s_at	3.41	tripartite motif-containing 34	TRIM34
239481_at	3.41		
231166_at	3.41		
232238_at	3.41	asp (abnormal spindle) homolog, microcephaly associated (Drosophila)	ASPM
226339_at	3.40	TruB pseudouridine (psi) synthase homolog 1 (E. coli)	TRUB1
1559756_at	3.40		
1554963_at	3.40	chromosome 6 open reading frame 192	C6orf192
229271_x_at	3.40	collagen, type XI, alpha 1	COL11A1
1552961_at	3.40		
221594_at	3.40		
218711_s_at	3.39	serum deprivation response (phosphatidylserine binding protein)	SDPR
201289_at	3.39	cysteine-rich, angiogenic inducer, 61	CYR61
1558010_s_at	3.39	solute carrier family 1 (glial high affinity glutamate transporter), member 2	SLC1A2
207103_at	3.38	potassium voltage-gated channel, Shal-related subfamily, member 2	KCND2
221051_s_at	3.38	integrin beta 1 binding protein 3	ITGB1BP3
1557283_a_at	3.37	zinc finger protein 519	ZNF519
207016_s_at	3.37	aldehyde dehydrogenase 1 family, member A2	ALDH1A2
214769_at	3.36		
231269_at	3.36	activating signal cointegrator 1 complex subunit 3	ASCC3
215342_s_at	3.36	RAB GTPase activating protein 1-like	RABGAP1L
219793_at	3.36	sorting nexin 16	SNX16
222213_x_at	3.36		
221587_s_at	3.35	chromosome 19 open reading frame 24	C19orf24
235155_at	3.35	3-hydroxybutyrate dehydrogenase, type 2	BDH2
238956_at	3.35		
1561148_at	3.35		
235119_at	3.35	TAF3 RNA polymerase II, TATA box binding protein (TBP)-associated factor, 140kDa	TAF3
219003_s_at	3.35	mannosidase, endo-alpha	MANEA
240000_at	3.34		
216070_at	3.34		

210852_s_at	3.33	aminoadipate-semialdehyde synthase	AASS
240319_at	3.33		
1556842_at	3.33		
228146_at	3.32	chromosome 17 open reading frame 51	C17orf51
222237_s_at	3.32	zinc finger protein 228#zinc finger protein 229#zinc finger protein 235#zinc finger protein 235	ZNF228#ZNF229#ZNF235#ZNF235
1559128_at	3.32	hydroxysteroid dehydrogenase like 2	HSDL2
238944_at	3.32	zinc finger protein 404	ZNF404
220343_at	3.32	phosphodiesterase 7B	PDE7B
236550_s_at	3.32	zinc finger protein 311	ZNF311
242470_at	3.32	EP300 interacting inhibitor of differentiation 2B	EID2B
206263_at	3.32	flavin containing monooxygenase 4	FMO4
215407_s_at	3.31	astrotactin 2	ASTN2
236278_at	3.31		
204962_s_at	3.31	centromere protein A	CENPA
242255_at	3.30	WD repeat domain 37	WDR37
1556778_at	3.30		
1556935_at	3.30		
1560924_at	3.30		
206765_at	3.30	potassium inwardly-rectifying channel, subfamily J, member 2	KCNJ2
1563794_s_at	3.30	intraflagellar transport 122 homolog (Chlamydomonas)	IFT122
229960_at	3.30	mitogen-activated protein kinase kinase kinase 6	MAP3K6
228158_at	3.29		
221136_at	3.29	growth differentiation factor 2	GDF2
244006_at	3.29		
1556122_at	3.28		
231726_at	3.28	protocadherin beta 14	PCDHB14
202779_s_at	3.28	ubiquitin-conjugating enzyme E2S	UBE2S
223413_s_at	3.28		
1559103_s_at	3.28		
1560048_at	3.28		
211737_x_at	3.27	pleiotrophin (heparin binding growth factor 8, neurite growth-promoting factor 1)	PTN
232985_s_at	3.27	developmental pluripotency associated 4	DPPA4
209135_at	3.27	aspartate beta-hydroxylase	ASPH
224802_at	3.27	Nedd4 family interacting protein 2	NDFIP2
207723_s_at	3.27	killer cell lectin-like receptor subfamily C, member 3	KLRC3
1560222_at	3.27		
204454_at	3.27	leucine zipper, down-regulated in cancer 1	LDOC1
1561143_at	3.26		
201150_s_at	3.26	TIMP metallopeptidase inhibitor 3 (Sorsby fundus dystrophy, pseudoinflammatory)	TIMP3
242128_at	3.26	orthodenticle homolog 2 (Drosophila)	OTX2
223651_x_at	3.26	cell division cycle 23 homolog (S. cerevisiae)	CDC23
204167_at	3.26	biotinidase	BTD
1560197_at	3.25	chromosome 8 open reading frame 70	C8orf70
1560020_at	3.25	DnaJ (Hsp40) homolog, subfamily C, member 13	DNAJC13
221287_at	3.25	ribonuclease L (2',5'-oligoisoadenylate synthetase-dependent)	RNASEL

204682_at	3.25	latent transforming growth factor beta binding protein 2	LTBP2
235461_at	3.25	KIAA1546	KIAA1546
220169_at	3.25	transmembrane protein 156	TMEM156
228507_at	3.24		
1568941_a_at	3.24		
241328_at	3.24	zinc finger, matrin type 1	ZMAT1
227176_at	3.23	solute carrier family 2 (facilitated glucose transporter), member 13	SLC2A13
230399_at	3.23		
233082_at	3.23	zinc finger protein 630	ZNF630
244888_at	3.22		
242206_at	3.22		
117_at	3.22		
235310_at	3.22	germinal center expressed transcript 2	GCET2
230781_at	3.22		
228660_x_at	3.22	sema domain, immunoglobulin domain (Ig), transmembrane domain (TM) and short cytoplasmic domain, (se	SEMA4F
1558466_at	3.22		
219544_at	3.21	chromosome 13 open reading frame 34	C13orf34
239975_at	3.21		
209310_s_at	3.21	caspase 4, apoptosis-related cysteine peptidase	CASP4
1564259_at	3.21		
228097_at	3.20	myosin regulatory light chain interacting protein	MYLIP
213418_at	3.20	heat shock 70kDa protein 6 (HSP70B')	HSPA6
243087_at	3.20	WD repeat domain 63	WDR63
229989_at	3.20	asparagine-linked glycosylation 9 homolog (S. cerevisiae, alpha- 1,2-mannosyltransferase)	ALG9
215402_at	3.19	amyloid beta precursor protein (cytoplasmic tail) binding protein 2	APPBP2
244027_at	3.19	ATP-binding cassette, sub-family B (MDR/TAP), member 7	ABCB7
229869_at	3.19		
209109_s_at	3.19	tetraspanin 6	TSPAN6
232257_s_at	3.19		
223582_at	3.19	G protein-coupled receptor 98	GPR98
1567912_s_at	3.19		
238466_at	3.19		
223541_at	3.19	hyaluronan synthase 3	HAS3
229400_at	3.19	homeobox D10	HOXD10
223758_s_at	3.18	general transcription factor IIH, polypeptide 2, 44kDa	GTF2H2
205551_at	3.18	synaptic vesicle glycoprotein 2B	SV2B
237750_at	3.18	X-prolyl aminopeptidase (aminopeptidase P) 3, putative	XPNPEP3
225462_at	3.18	transmembrane protein 128	TMEM128
223412_at	3.18	kelch repeat and BTB (POZ) domain containing 7	KBTBD7
1562621_at	3.18		
233133_at	3.18		
243909_x_at	3.17	glucuronidase, beta-like 2	GUSBL2
222262_s_at	3.17	ethanolamine kinase 1	ETNK1
220920_at	3.17	ATPase, Class V, type 10B	ATP10B

209335_at	3.17	decorin	DCN
225023_at	3.17	golgi associated PDZ and coiled-coil motif containing	GOPC
205486_at	3.17	testis-specific kinase 2	TESK2
234155_at	3.17		
1553607_at	3.16	chromosome 21 open reading frame 109	C21orf109
221184_at	3.16		
213124_at	3.16	zinc finger protein 473	ZNF473
231690_at	3.15		
229285_at	3.15	ribonuclease L (2',5'-oligoisoadenylate synthetase-dependent)	RNASEL
219397_at	3.15	coenzyme Q10 homolog B (S. cerevisiae)	COQ10B
235406_x_at	3.15		
239890_s_at	3.15	chromosome 13 open reading frame 21	C13orf21
1554345_a_at	3.15	zinc finger, H2C2 domain containing	ZH2C2
229374_at	3.14	EPH receptor A4	EPHA4
238504_at	3.14	chromosome 6 open reading frame 57	C6orf57
1553335_x_at	3.14		
230763_at	3.14	spermatogenesis associated 17	SPATA17
205229_s_at	3.13	coagulation factor C homolog, cochlin (Limulus polyphemus)	COCH
239768_x_at	3.13		
235771_at	3.13		
218755_at	3.13	kinesin family member 20A	KIF20A
243160_at	3.13		
243194_at	3.13	zinc finger protein 551	ZNF551
1553181_at	3.13	DEAD (Asp-Glu-Ala-Asp) box polypeptide 31	DDX31
237213_at	3.13		
243300_at	3.13		
206369_s_at	3.12	phosphoinositide-3-kinase, catalytic, gamma polypeptide	PIK3CG
228996_at	3.12	ring finger and CCCH-type zinc finger domains 1	RC3H1
206702_at	3.12	TEK tyrosine kinase, endothelial (venous malformations, multiple cutaneous and mucosal)	TEK
244634_at	3.12	family with sequence similarity 53, member B	FAM53B
1562267_s_at	3.11	zinc finger protein 564	ZNF564
217818_s_at	3.11	actin related protein 2/3 complex, subunit 4, 20kDa	ARPC4
231332_at	3.11		
231112_at	3.11	small nuclear ribonucleoprotein polypeptide E	SNRPE
221595_at	3.11		
226252_at	3.11		
242872_at	3.11	citron (rho-interacting, serine/threonine kinase 21)	CIT
1569235_a_at	3.11		
219947_at	3.11	C-type lectin domain family 4, member A	CLEC4A
227362_at	3.10	SLC2A4 regulator	SLC2A4RG
207115_x_at	3.10	mbt domain containing 1	MBTD1
243188_at	3.10	zinc finger protein 283	ZNF283
1557845_at	3.10		
234295_at	3.10	debranching enzyme homolog 1 (S. cerevisiae)	DBR1
213853_at	3.10	DPH4, JJJ3 homolog (S. cerevisiae)	DPH4
238634_x_at	3.09		

232247_at	3.09	zinc finger protein 502	ZNF502
239444_at	3.09		
225578_at	3.09		
222857_s_at	3.09	potassium large conductance calcium-activated channel, subfamily M, beta member 4	KCNMB4
238757_at	3.09	DBF4 homolog B (S. cerevisiae)	DBF4B
206828_at	3.09	TXK tyrosine kinase	TXK
239556_at	3.09		
1554242_a_at	3.08	coagulation factor C homolog, cochlin (Limulus polyphemus)	COCH
209182_s_at	3.08	chromosome 10 open reading frame 10	C10orf10
220004_at	3.08	DEAD (Asp-Glu-Ala-Asp) box polypeptide 43	DDX43
1559821_at	3.08		
1552430_at	3.08	WD repeat domain 17	WDR17
219377_at	3.08	family with sequence similarity 59, member A	FAM59A
221027_s_at	3.08	phospholipase A2, group XIIA	PLA2G12A
209795_at	3.08	CD69 molecule	CD69
236182_at	3.08		
223558_at	3.07		
1554964_x_at	3.07	chromosome 6 open reading frame 192	C6orf192
1556749_at	3.07		
225424_at	3.07	glycerol-3-phosphate acyltransferase, mitochondrial	GPAM
238168_at	3.07	transmembrane 4 L six family member 1	TM4SF1
208425_s_at	3.07	tetratricopeptide repeat, ankyrin repeat and coiled-coil containing 2	TANC2
232760_at	3.07	testis expressed sequence 15	TEX15
1568787_at	3.07		
213156_at	3.07		
204290_s_at	3.07	aldehyde dehydrogenase 6 family, member A1	ALDH6A1
226375_at	3.06		
233071_at	3.06	karyopherin alpha 5 (importin alpha 6)#chromosome 6 open reading frame 113#radial spokehead-like 3	KPNA5#C6orf113#RSHL3
238935_at	3.06	ribosomal protein S27-like	RPS27L
209915_s_at	3.06	neurexin 1	NRXN1
239169_at	3.06	RAD52 motif 1	RDM1
204271_s_at	3.06	endothelin receptor type B	EDNRB
227546_x_at	3.06	cyclin L2	CCNL2
236650_at	3.06		
206512_at	3.06	zinc finger (CCCH type), RNA-binding motif and serine/arginine rich 1	ZRSR1
243371_at	3.06		
206682_at	3.06	C-type lectin domain family 10, member A	CLEC10A
238773_at	3.05	methyltransferase 5 domain containing 1	METT5D1
235498_at	3.05	leucine rich repeat containing 44	LRRC44
230581_at	3.05		
230626_at	3.05	tetraspanin 12	TSPAN12
238264_at	3.05	NMD3 homolog (S. cerevisiae)	NMD3
203589_s_at	3.05	transcription factor Dp-2 (E2F dimerization partner 2)	TFDP2
237706_at	3.05	syntaxin binding protein 4	STXBP4
230294_at	3.04		

1569385_s_at	3.04	KIAA1546	KIAA1546
213032_at	3.04		
1557383_a_at	3.04		
219239_s_at	3.04	zinc finger protein 654	ZNF654
242626_at	3.04		
226752_at	3.03	transmembrane protein 157	TMEM157
229591_at	3.03	low density lipoprotein receptor-related protein 5	LRP5
205699_at	3.03	mitogen-activated protein kinase kinase 6	MAP2K6
1560853_x_at	3.03		
216009_at	3.03	solute carrier family 39 (zinc transporter), member 9	SLC39A9
1554742_at	3.03	PMS1 postmeiotic segregation increased 1 (S. cerevisiae)	PMS1
1553208_s_at	3.03	ADP-ribosylation factor-like 10	ARL10
1553321_a_at	3.03	sulfotransferase family, cytosolic, 1C, member 2	SULT1C2
216468_s_at	3.03	zinc finger protein 90#zinc finger protein 682	ZNF90#ZNF682
236512_at	3.02	sestrin 1	SESN1
202411_at	3.02	interferon, alpha-inducible protein 27	IFI27
235737_at	3.02		
223249_at	3.02	claudin 12	CLDN12
209463_s_at	3.02	TAF12 RNA polymerase II, TATA box binding protein (TBP)-associated factor, 20kDa	TAF12
244784_at	3.02	DEAH (Asp-Glu-Ala-Asp/His) box polypeptide 57	DHX57
203549_s_at	3.02	lipoprotein lipase	LPL
227214_at	3.01	golgi associated PDZ and coiled-coil motif containing	GOPC
215944_at	3.01		
213081_at	3.01	zinc finger and BTB domain containing 22	ZBTB22
222939_s_at	3.01	solute carrier family 16, member 10 (aromatic amino acid transporter)	SLC16A10
227121_at	3.01		
242409_at	3.01		
1569987_at	3.01		
1562815_at	3.01		
227082_at	3.00		
210762_s_at	3.00	deleted in liver cancer 1	DLC1
228941_at	3.00		
222351_at	3.00	protein phosphatase 2 (formerly 2A), regulatory subunit A, beta isoform	PPP2R1B
232778_at	3.00		
1562947_x_at	3.00		
239587_at	3.00		
206314_at	3.00	zinc finger protein 167	ZNF167
210548_at	3.00	chemokine (C-C motif) ligand 23	CCL23
228235_at	3.00		
232737_s_at	3.00	olfactory receptor, family 2, subfamily A, member 4	OR2A4
217127_at	3.00	cystathionase (cystathionine gamma-lyase)	CTH
204697_s_at	2.99	chromogranin A (parathyroid secretory protein 1)	CHGA
235606_at	2.99		
214213_x_at	2.99	lamin A/C	LMNA
1562712_at	2.99		

219587_at	2.99	tetratricopeptide repeat domain 12	TTC12
209293_x_at	2.99	inhibitor of DNA binding 4, dominant negative helix-loop-helix protein	ID4
217580_x_at	2.99		
232574_at	2.99	xylosyltransferase I	XYLT1
241184_x_at	2.99	zinc finger protein 407	ZNF407
231055_at	2.99		
212720_at	2.98	poly(A) polymerase alpha	PAPOLA
218940_at	2.98	chromosome 14 open reading frame 138	C14orf138
235757_at	2.97	chromosome 10 open reading frame 18	C10orf18
243231_at	2.97		
1554418_s_at	2.97	sparc/osteonectin, cwcv and kazal-like domains proteoglycan (testican) 3	SPOCK3
210669_at	2.97	transcription factor AP-2 alpha (activating enhancer binding protein 2 alpha)	TFAP2A
233241_at	2.97	chromosome 20 open reading frame 19	C20orf19
242828_at	2.96	fidgetin	FIGN
219810_at	2.96	valosin containing protein (p97)/p47 complex interacting protein 1	VCPIP1
1553415_at	2.96	solute carrier family 17 (sodium-dependent inorganic phosphate cotransporter), member 8	SLC17A8
1553301_a_at	2.96	transmembrane protein 182	TMEM182
222581_at	2.96	xenotropic and polytropic retrovirus receptor	XPR1
238289_at	2.96		
244752_at	2.95	zinc finger protein 438	ZNF438
220549_at	2.95	RAD54 homolog B (S. cerevisiae)	RAD54B
242035_at	2.95		
225689_at	2.95	chromosome 3 open reading frame 39	C3orf39
206300_s_at	2.95	parathyroid hormone-like hormone	PTHLH
209146_at	2.95	sterol-C4-methyl oxidase-like	SC4MOL
236613_at	2.95	RNA binding motif protein 25	RBM25
238304_at	2.94	dipeptidyl-peptidase 6	DPP6
1570001_at	2.94	CASP8 associated protein 2	CASP8AP2
219287_at	2.94	potassium large conductance calcium-activated channel, subfamily M, beta member 4	KCNMB4
1556325_at	2.94	filamin A interacting protein 1	FILIP1
1559515_at	2.94		
236517_at	2.94	multiple EGF-like-domains 10	MEGF10
232875_at	2.94		
216061_x_at	2.93	platelet-derived growth factor beta polypeptide (simian sarcoma viral (v-sis) oncogene homolog)	PDGFB
204979_s_at	2.93	SH3 domain binding glutamic acid-rich protein	SH3BGR
220129_at	2.93	spermatogenesis and oogenesis specific basic helix-loop-helix 2	SOHLH2
233367_at	2.93		
240268_at	2.93		
227628_at	2.93		
205001_s_at	2.93	DEAD (Asp-Glu-Ala-Asp) box polypeptide 3, Y-linked	DDX3Y
231249_at	2.92		
1556558_s_at	2.92		
209300_s_at	2.92	NECAP endocytosis associated 1	NECAP1
215307_at	2.92	zinc finger protein 529	ZNF529

229970_at	2.92		
233075_at	2.92	hect domain and RLD 2 pseudogene 7	HERC2P7
208186_s_at	2.92	lipase, hormone-sensitive	LIPE
1562259_at	2.91	testis expressed sequence 9	TEX9
206026_s_at	2.91	tumor necrosis factor, alpha-induced protein 6	TNFAIP6
204807_at	2.91	transmembrane protein 5	TMEM5
202973_x_at	2.91	family with sequence similarity 13, member A1	FAM13A1
233839_at	2.91		
221988_at	2.91	cofactor required for Sp1 transcriptional activation, subunit 7, 70kDa	CRSP7
229363_at	2.90		
207808_s_at	2.90	protein S (alpha)	PROS1
217242_at	2.90	zinc finger protein 154	ZNF154
1557720_s_at	2.90		
219750_at	2.90	transmembrane protein 144	TMEM144
1559108_at	2.90		
237065_s_at	2.90		
239479_x_at	2.90		
1569816_at	2.90		
220121_at	2.90	lines homolog 1 (Drosophila)	LINS1
223373_s_at	2.90	phospholipase A2, group XIIA	PLA2G12A
1554014_at	2.90	chromodomain helicase DNA binding protein 2	CHD2
237745_at	2.90		
238353_at	2.90	RAS-like, family 11, member A	RASL11A
209967_s_at	2.90	cAMP responsive element modulator	CREM
241014_at	2.90		
207029_at	2.89	KIT ligand	KITLG
237440_at	2.89		
239252_at	2.89		
209189_at	2.89	v-fos FBJ murine osteosarcoma viral oncogene homolog	FOS
238831_at	2.89		
1555598_a_at	2.89	dual specificity phosphatase 19	DUSP19
225914_s_at	2.89	calcium binding protein 39-like	CAB39L
238250_at	2.89		
220306_at	2.89	family with sequence similarity 46, member C	FAM46C
218197_s_at	2.88	oxidation resistance 1	OXR1
1556209_at	2.88	C-type lectin domain family 2, member B	CLEC2B
226661_at	2.88	cell division cycle associated 2	CDCA2
242924_at	2.88	phosphatidylinositol transfer protein, beta	PITPNB
204159_at	2.88	cyclin-dependent kinase inhibitor 2C (p18, inhibits CDK4)	CDKN2C
214542_x_at	2.88	histone cluster 1, H2ai	HIST1H2AI
1552497_a_at	2.88	SLAM family member 6	SLAMF6
243312_at	2.87	zinc finger protein 588	ZNF588
241905_at	2.87	phosphoinositide-3-kinase, class 2, alpha polypeptide	PIK3C2A
204135_at	2.87	filamin A interacting protein 1-like	FILIP1L
204712_at	2.87	WNT inhibitory factor 1	WIF1
209427_at	2.87	smoothelin	SMTN
240105_at	2.87	chromosome 21 open reading frame 66	C21orf66
230491_at	2.87		
213158_at	2.87		

235134_at	2.87		
235447_at	2.87	TruB pseudouridine (psi) synthase homolog 1 (E. coli)	TRUB1
244086_at	2.87	arylacetamide deacetylase-like 1	AADACL1
1567079_at	2.86	ceroid-lipofuscinosis, neuronal 6, late infantile, variant	CLN6
240855_at	2.86	spectrin, beta, erythrocytic (includes spherocytosis, clinical type I)	SPTB
1568987_at	2.86		
239025_at	2.86		
242667_at	2.86		
216001_at	2.86	PRAME family member 1#PRAME family member 12	PRAMEF1#PRAMEF12
205443_at	2.86	small nuclear RNA activating complex, polypeptide 1, 43kDa	SNAPC1
203502_at	2.86	2,3-bisphosphoglycerate mutase	BPGM
228636_at	2.85	basic helix-loop-helix domain containing, class B, 5	BHLHB5
1569191_at	2.85		
237756_at	2.85	kelch-like 23 (Drosophila)	KLHL23
1562611_at	2.85		
217188_s_at	2.85	basic leucine zipper transcription factor, ATF-like#chromosome 14 open reading frame 1#tubulin tyrosine ligase-like family, member 5#chromosome 14 open reading frame 58#chromosome 14 open reading frame 58#ribosomal protein S24 pseudogene 2	BATF#C14orf1#TTLL5#C14orf58#C14orf58#RPS24P2
223764_x_at	2.85	nipsnap homolog 3B (C. elegans)	NIPSNAP3B
243293_at	2.85		
229751_s_at	2.85	pseudouridylate synthase 7 homolog (S. cerevisiae)-like	PUS7L
202094_at	2.85	baculoviral IAP repeat-containing 5 (survivin)	BIRC5
1553122_s_at	2.85	RB-associated KRAB zinc finger	RBAK
1560480_at	2.84		
203478_at	2.84	NADH dehydrogenase (ubiquinone) 1, subcomplex unknown, 1, 6kDa	NDUFC1
235158_at	2.84		
232038_at	2.84	chromosome 6 open reading frame 170	C6orf170
237301_at	2.84		
230431_at	2.84		
207446_at	2.83	toll-like receptor 6	TLR6
222317_at	2.83	phosphodiesterase 3B, cGMP-inhibited	PDE3B
222798_at	2.83	phosphotriesterase related	PTER
209433_s_at	2.83	phosphoribosyl pyrophosphate amidotransferase	PPAT
237538_at	2.83	radical S-adenosyl methionine domain containing 2	RSAD2
232764_at	2.83		
227222_at	2.83	F-box protein 10	FBXO10
1558809_s_at	2.83		
228233_at	2.83	FRAS1 related extracellular matrix 1	FREM1
242513_x_at	2.82	KIAA2018	KIAA2018
1555217_at	2.82	ubiquitin-conjugating enzyme E2W (putative)	UBE2W
236174_at	2.82		

244549_at	2.82	fasciculation and elongation protein zeta 2 (zygin II)	FEZ2
231840_x_at	2.82	Lyrm7 homolog (mouse)	LYRM7
222248_s_at	2.82	musashi homolog 1 (Drosophila)#phospholipase A2, group IB (pancreas)#sirtuin (silent mating type information regulation 2 homolog) 4 (S. cerevisiae)	MSI1#PLA2G1B#SIRT4
229631_at	2.82	dynein heavy chain domain 1	DNHD1
228090_at	2.82	nicotinamide nucleotide adenylyltransferase 3	NMNAT3
227941_at	2.82		
243216_x_at	2.82	ubiquitin specific peptidase 40	USP40
211445_x_at	2.81	nascent-polypeptide-associated complex alpha polypeptide pseudogene 1	NACAP1
233981_at	2.81		
222513_s_at	2.81	sorbin and SH3 domain containing 1	SORBS1
238158_at	2.81	meiosis expressed gene 1 homolog (mouse)	MEIG1
207565_s_at	2.80	major histocompatibility complex, class I-related	MR1
233677_at	2.80		
230968_at	2.80		
219078_at	2.79	G patch domain containing 2	GPATCH2
203159_at	2.79	glutaminase	GLS
240974_at	2.79		
240800_x_at	2.79		
239896_at	2.79		
1568720_at	2.79	zinc finger protein 506	ZNF506
224019_at	2.79	stearoyl-CoA desaturase 5	SCD5
227443_at	2.79	chromosome 9 open reading frame 150	C9orf150
212107_s_at	2.78	DEAH (Asp-Glu-Ala-His) box polypeptide 9	DHX9
219829_at	2.78	integrin beta 1 binding protein (melusin) 2	ITGB1BP2
1553710_at	2.78		
243796_at	2.78		
237147_at	2.78		
241546_at	2.78	spermatogenesis associated 5	SPATA5
204569_at	2.78	intestinal cell (MAK-like) kinase	ICK
207142_at	2.78	potassium inwardly-rectifying channel, subfamily J, member 3	KCNJ3
225608_at	2.78		
239311_at	2.78		
212077_at	2.77	caldesmon 1	CALD1
244505_at	2.77		
1558699_a_at	2.77	HERPUD family member 2	HERPUD2
242900_at	2.77		
1554966_a_at	2.77	filamin A interacting protein 1-like	FILIP1L
234074_at	2.77		
1561652_at	2.77	beclin 1 (coiled-coil, myosin-like BCL2 interacting protein)	BECN1
220242_x_at	2.77	zinc finger protein 701	ZNF701
1553634_a_at	2.77		
1552970_s_at	2.77	zinc finger, MYM-type 6	ZMYM6
243512_x_at	2.77	IMP2 inner mitochondrial membrane peptidase-like (S. cerevisiae)	IMMP2L
216496_s_at	2.77		
240997_at	2.76		

224448_s_at	2.76	chromosome 6 open reading frame 125	C6orf125
244391_at	2.76	tRNA splicing endonuclease 2 homolog (S. cerevisiae)	TSEN2
232795_at	2.76		
1566700_at	2.76	vaccinia related kinase 3	VRK3
218597_s_at	2.76	zinc finger, CDGSH-type domain 1	ZCD1
238946_at	2.76		
218346_s_at	2.75	sestrin 1	SESN1
244557_at	2.75		
203157_s_at	2.75	glutaminase	GLS
207228_at	2.75	protein kinase, cAMP-dependent, catalytic, gamma	PRKACG
37860_at	2.75	zinc finger protein 337	ZNF337
219826_at	2.75	zinc finger protein 419	ZNF419
1554329_x_at	2.75	syntaxin binding protein 4	STXBP4
231822_at	2.75	CTTNBP2 N-terminal like	CTTNBP2NL
1553801_a_at	2.74	chromosome 14 open reading frame 126	C14orf126
231789_at	2.74	protocadherin beta 15	PCDHB15
231577_s_at	2.74	guanylate binding protein 1, interferon-inducible, 67kDa	GBP1
225851_at	2.74	farnesyltransferase, CAAX box, beta	FNTB
238379_x_at	2.74		
234371_at	2.74	keratin 18 pseudogene 1#tripartite motif-containing 27#olfactory receptor, family 2, subfamily AD, member 1 pseudogene#zinc finger protein 311	KRT18P1#TRIM27#OR2AD1P#ZNF311
237168_at	2.74		
223614_at	2.74	chromosome 8 open reading frame 57	C8orf57
1569544_at	2.73		
235512_at	2.73	cyclin-dependent kinase-like 1 (CDC2-related kinase)	CDKL1
218967_s_at	2.73	phosphotriesterase related	PTER
1562089_at	2.73	glycine-N-acyltransferase-like 1	GLYATL1
1558409_at	2.73		
234475_x_at	2.73	cholecystokinin B receptor	CCKBR
1556350_a_at	2.73	eukaryotic translation initiation factor 4A, isoform 2	EIF4A2
214734_at	2.73	exophilin 5	EXPH5
236529_at	2.73	scavenger receptor cysteine rich domain containing, group B (4 domains)	SRCRB4D
222553_x_at	2.73	oxidation resistance 1	OXR1
223680_at	2.72	zinc finger protein 607	ZNF607
225290_at	2.72	ethanolamine kinase 1	ETNK1
217383_at	2.72	phosphoglycerate kinase 1	PGK1
227458_at	2.72		
210199_at	2.72	crystallin, alpha A	CRYAA
236226_at	2.72	B and T lymphocyte associated	BTLA
239870_at	2.72	spermatogenesis associated, serine-rich 1	SPATS1
216215_s_at	2.72	RNA binding motif protein 9#apolipoprotein L, 5#mitochondrial ribosomal protein S16 pseudogene 3	RBM9#APOL5#MRPS16P3
204107_at	2.72	nuclear transcription factor Y, alpha	NFYA
216801_at	2.72	ciliary rootlet coiled-coil, rootletin	CROCC

1557786_s_at	2.71	cysteine-rich hydrophobic domain 1	CHIC1
1554547_at	2.71	family with sequence similarity 13, member C1	FAM13C1
230507_at	2.71	ataxin 1	ATXN1
240622_at	2.71	progestin and adipoQ receptor family member III	PAQR3
202609_at	2.71	epidermal growth factor receptor pathway substrate 8	EPS8
236568_at	2.71		
218192_at	2.70	inositol hexaphosphate kinase 2	IHPK2
235976_at	2.70	SLIT and NTRK-like family, member 6	SLITRK6
214588_s_at	2.70	UDP-N-acetyl-alpha-D-galactosamine:polypeptide N-acetylgalactosaminyltransferase 10 (GalNAc-T10)	GALNT10
209551_at	2.70	Yip1 domain family, member 4	YIPF4
218285_s_at	2.70	3-hydroxybutyrate dehydrogenase, type 2	BDH2
234531_at	2.70		
218853_s_at	2.70	motile sperm domain containing 1	MOSPD1
213934_s_at	2.70	zinc finger protein 23 (KOX 16)	ZNF23
235026_at	2.70		
1555125_at	2.69	chromosome 21 open reading frame 66	C21orf66
230216_at	2.69	chromosome 12 open reading frame 51	C12orf51
223850_at	2.69	DnaJ (Hsp40) homolog, subfamily A, member 2	DNAJA2
1564468_at	2.69		
1559975_at	2.69	B-cell translocation gene 1, anti-proliferative	BTG1
37577_at	2.69	Rho GTPase activating protein 19	ARHGAP19
214093_s_at	2.69	far upstream element (FUSE) binding protein 1	FUBP1
207505_at	2.69	protein kinase, cGMP-dependent, type II	PRKG2
226600_at	2.69	transmembrane and tetratricopeptide repeat containing 3	TMTC3
225847_at	2.69	arylacetamide deacetylase-like 1	AADACL1
1562026_at	2.69	WNK lysine deficient protein kinase 2	WNK2
218477_at	2.68	transmembrane protein 14A	TMEM14A
210432_s_at	2.68	sodium channel, voltage-gated, type III, alpha subunit	SCN3A
1560171_at	2.68	SNF2 histone linker PHD RING helicase	SHPRH
1554741_s_at	2.68		
221927_s_at	2.68	abhydrolase domain containing 11	ABHD11
237109_at	2.68	target of myb1-like 2 (chicken)	TOM1L2
221025_x_at	2.68	pseudouridylate synthase 7 homolog (S. cerevisiae)-like	PUS7L
202469_s_at	2.68	cleavage and polyadenylation specific factor 6, 68kDa	CPSF6
227289_at	2.68	protocadherin 17	PCDH17
242418_at	2.68		
210697_at	2.68	zinc finger protein 257	ZNF257
236958_at	2.67		
209927_s_at	2.67	chromosome 1 open reading frame 77	C1orf77
206011_at	2.67	caspase 1, apoptosis-related cysteine peptidase (interleukin 1, beta, convertase)	CASP1
205988_at	2.67	CD84 molecule	CD84
220679_s_at	2.67	cadherin 7, type 2	CDH7

213468_at	2.67	excision repair cross-complementing rodent repair deficiency, complementation group 2 (xeroderma pig	ERCC2
227787_s_at	2.67	thyroid hormone receptor associated protein 6	THRAP6
231838_at	2.67	chromosome 20 open reading frame 119	C20orf119
1552695_a_at	2.67	solute carrier family 2 (facilitated glucose transporter), member 13	SLC2A13
1558518_at	2.67	mitogen-activated protein kinase kinase kinase 7 interacting protein 3	MAP3K7IP3
238729_x_at	2.67		
220148_at	2.67	aldehyde dehydrogenase 8 family, member A1	ALDH8A1
1558856_at	2.66	DMRT-like family A2	DMRTA2
1554447_at	2.66		
219017_at	2.66	ethanolamine kinase 1	ETNK1
233025_at	2.66	PDZ domain containing 2	PDZD2
214050_at	2.66	CGG triplet repeat binding protein 1	CGGBP1
1557430_at	2.66		
204764_at	2.66	farnesyltransferase, CAAX box, beta	FNTB
1569608_x_at	2.66		
205938_at	2.66	protein phosphatase 1E (PP2C domain containing)	PPM1E
213424_at	2.66		
238708_at	2.65		
222128_at	2.65	NOL1/NOP2/Sun domain family, member 6	NSUN6
229075_at	2.65		
228692_at	2.65		
201732_s_at	2.65	chloride channel 3	CLCN3
204920_at	2.65	carbamoyl-phosphate synthetase 1, mitochondrial	CPS1
244049_at	2.64		
1553269_at	2.64	zinc finger protein 718	ZNF718
222457_s_at	2.64	LIM domain and actin binding 1	LIMA1
243184_at	2.64	tight junction protein 1 (zona occludens 1)	TJP1
226271_at	2.64	ganglioside-induced differentiation-associated protein 1	GDAP1
230351_at	2.64		
1552708_a_at	2.64	dual specificity phosphatase 19	DUSP19
212973_at	2.64	ribose 5-phosphate isomerase A (ribose 5-phosphate epimerase)	RPIA
214117_s_at	2.64	biotinidase	BTD
236551_at	2.64	zinc finger protein 311	ZNF311
228855_at	2.63	nudix (nucleoside diphosphate linked moiety X)-type motif 7	NUDT7
221845_s_at	2.63	ClpB caseinolytic peptidase B homolog (E. coli)	CLPB
203903_s_at	2.63	hephaestin	HEPH
226811_at	2.63	family with sequence similarity 46, member C	FAM46C
243190_at	2.63		
228057_at	2.63	DNA-damage-inducible transcript 4-like	DDIT4L
231270_at	2.63	carbonic anhydrase XIII	CA13
232787_at	2.63		
209897_s_at	2.63	slit homolog 2 (Drosophila)	SLIT2
1569114_at	2.63		
220269_at	2.63		

210073_at	2.63	ST8 alpha-N-acetyl-neuraminide alpha-2,8-sialyltransferase 1	ST8SIA1
210166_at	2.63	toll-like receptor 5	TLR5
1552735_at	2.62	protocadherin gamma subfamily A, 4	PCDHGA4
238623_at	2.62		
210945_at	2.62	collagen, type IV, alpha 6	COL4A6
1563475_s_at	2.62		
205662_at	2.62		
240612_at	2.62	solute carrier family 12 (potassium/chloride transporters), member 8	SLC12A8
233675_s_at	2.62		
206023_at	2.62	neuromedin U	NMU
202146_at	2.62	interferon-related developmental regulator 1	IFRD1
205357_s_at	2.62	angiotensin II receptor, type 1	AGTR1
221577_x_at	2.62	growth differentiation factor 15	GDF15
240239_at	2.61	zinc finger protein 566	ZNF566
231725_at	2.61	protocadherin beta 2	PCDHB2
209914_s_at	2.61	neurexin 1	NRXN1
204433_s_at	2.61	spermatogenesis associated 2	SPATA2
237153_at	2.61		
1552320_a_at	2.61	coiled-coil domain containing 65	CCDC65
228967_at	2.61		
221104_s_at	2.61	nipsnap homolog 3B (C. elegans)	NIPSNAP3B
233430_at	2.61	TBC1 domain family, member 22B	TBC1D22B
1554015_a_at	2.61	chromodomain helicase DNA binding protein 2	CHD2
215074_at	2.61	myosin IB	MYO1B
218546_at	2.61	chromosome 1 open reading frame 115	C1orf115
226800_at	2.61	phosphoglucomutase 1#null	PGM1#null
229298_at	2.61		
1565685_at	2.60		
230869_at	2.60		
208477_at	2.60	potassium voltage-gated channel, Shaw-related subfamily, member 1	KCNC1
206085_s_at	2.60	cystathionase (cystathionine gamma-lyase)	CTH
205011_at	2.60	loss of heterozygosity, 11, chromosomal region 2, gene A	LOH11CR2A
220586_at	2.60	chromodomain helicase DNA binding protein 9	CHD9
232785_at	2.60	retrotransposon gag domain containing 1	RGAG1
232600_at	2.60	ankyrin repeat domain 42	ANKRD42
207046_at	2.60	histone cluster 2, H4a	HIST2H4A
235215_at	2.60		
232090_at	2.60		
239343_at	2.60		
1564053_a_at	2.60	YTH domain family, member 3	YTHDF3
226181_at	2.59	tubulin, epsilon 1	TUBE1
228915_at	2.59	dachshund homolog 1 (Drosophila)	DACH1
1561948_at	2.59	coenzyme Q10 homolog B (S. cerevisiae)	COQ10B
242996_at	2.59	mitochondrial translational release factor 1	MTRF1
228345_at	2.59	cysteine-rich hydrophobic domain 1	CHIC1
239093_at	2.59	chromosome 10 open reading frame 65	C10orf65
222682_s_at	2.59	chromosome 5 open reading frame 3	C5orf3
220353_at	2.59	family with sequence similarity 86, member C	FAM86C
231976_at	2.59	lines homolog 1 (Drosophila)	LINS1

220195_at	2.59	methyl-CpG binding domain protein 5	MBD5
223462_at	2.59	transmembrane protein 175	TMEM175
1556706_at	2.59		
232481_s_at	2.59	SLIT and NTRK-like family, member 6	SLITRK6
211721_s_at	2.59	zinc finger protein 551	ZNF551
239091_at	2.59		
205868_s_at	2.58	protein tyrosine phosphatase, non-receptor type 11 (Noonan syndrome 1)	PTPN11
213709_at	2.58	basic helix-loop-helix domain containing, class B, 9	BHLHB9
239762_at	2.58		
209212_s_at	2.58	Kruppel-like factor 5 (intestinal)	KLF5
244240_at	2.58		
240141_at	2.58	PAP associated domain containing 4	PAPD4
1553527_at	2.58	NLR family, pyrin domain containing 9	NLRP9
243925_at	2.58		
235343_at	2.58		
236018_at	2.58	leucine carboxyl methyltransferase 2	LCMT2
229976_at	2.58	chromosome 9 open reading frame 18	C9orf18
1559009_at	2.58		
214748_at	2.58		
234992_x_at	2.57	epithelial cell transforming sequence 2 oncogene	ECT2
219915_s_at	2.57	solute carrier family 16, member 10 (aromatic amino acid transporter)	SLC16A10
241345_at	2.57		
233110_s_at	2.57	BCL2-like 12 (proline rich)	BCL2L12
1554831_x_at	2.57	amyotrophic lateral sclerosis 2 (juvenile) chromosome region, candidate 11	ALS2CR11
213218_at	2.57	zinc finger protein 187	ZNF187
223515_s_at	2.57	coenzyme Q3 homolog, methyltransferase (S. cerevisiae)	COQ3
242579_at	2.57		
206497_at	2.57	chromosome 7 open reading frame 44	C7orf44
222337_at	2.57	oxysterol binding protein-like 9	OSBPL9
223570_at	2.57	MCM10 minichromosome maintenance deficient 10 (S. cerevisiae)	MCM10
1559971_at	2.56	BSD domain containing 1	BSDC1
238256_at	2.56		
242819_at	2.56	BET1 homolog (S. cerevisiae)	BET1
237649_at	2.56	COP9 constitutive photomorphogenic homolog subunit 4 (Arabidopsis)	COPS4
1561453_at	2.56		
235962_at	2.56		
208229_at	2.56	fibroblast growth factor receptor 2 (bacteria-expressed kinase, keratinocyte growth factor receptor,	FGFR2
239916_at	2.56	WD repeat domain 16	WDR16
209839_at	2.56	dynamin 3	DNM3
1556061_at	2.56	ribonuclease P/MRP 30kDa subunit	RPP30
226065_at	2.56	prickle homolog 1 (Drosophila)	PRICKLE1
1562163_at	2.56		
219458_s_at	2.56	NOL1/NOP2/Sun domain family, member 3	NSUN3

219918_s_at	2.56	asp (abnormal spindle) homolog, microcephaly associated (Drosophila)	ASPM
1555872_a_at	2.56		
202269_x_at	2.55	guanylate binding protein 1, interferon-inducible, 67kDa	GBP1
217536_x_at	2.55		
209451_at	2.55	TRAF family member-associated NFKB activator	TANK
219467_at	2.55	zinc finger, H2C2 domain containing	ZH2C2
219343_at	2.55	cell division cycle 37 homolog (S. cerevisiae)-like 1	CDC37L1
1563614_at	2.55	Mdm2, transformed 3T3 cell double minute 2, p53 binding protein (mouse) binding protein, 104kDa	MTBP
205656_at	2.55	protocadherin 17	PCDH17
218616_at	2.55	integrator complex subunit 12	INTS12
207417_s_at	2.55	zinc finger protein 177	ZNF177
210223_s_at	2.55	major histocompatibility complex, class I-related	MR1
204641_at	2.55	NIMA (never in mitosis gene a)-related kinase 2	NEK2
238705_at	2.55		
238525_at	2.55		
241771_at	2.55	RIMS binding protein 2	RIMBP2
238063_at	2.55		
239400_at	2.55		
1556239_a_at	2.54		
234761_at	2.54		
218403_at	2.54	TP53 regulated inhibitor of apoptosis 1	TRIAP1
205934_at	2.54	phospholipase C-like 1	PLCL1
213959_s_at	2.54		
214120_at	2.54	ret finger protein-like 1 antisense	RFPL1S
205018_s_at	2.54	muscleblind-like 2 (Drosophila)	MBNL2
221054_s_at	2.54	T-cell leukemia/lymphoma 6	TCL6
226945_at	2.54	rhomboid domain containing 1	RHBDD1
210224_at	2.54	major histocompatibility complex, class I-related	MR1
230624_at	2.54	solute carrier family 25, member 27	SLC25A27
217552_x_at	2.54	complement component (3b/4b) receptor 1 (Knops blood group)	CR1
203725_at	2.54	growth arrest and DNA-damage-inducible, alpha	GADD45A
206615_s_at	2.53	ADAM metallopeptidase domain 22	ADAM22
233840_at	2.53		
202019_s_at	2.53	LanC lantibiotic synthetase component C-like 1 (bacterial)	LANCL1
1570125_at	2.53		
237246_at	2.53		
204108_at	2.53	nuclear transcription factor Y, alpha#apolipoprotein B mRNA editing enzyme, catalytic polypeptide-like 2#chromosome 6 open reading frame 130#benzodiazapine receptor (peripheral)-like 1#unc-5 homolog C (C. elegans)-like	NFYA#APOBEC2#C6orf130#BZRPL1#UNC5CL
222947_at	2.53	zinc finger protein 224	ZNF224
228281_at	2.53		
231817_at	2.52	ubiquitin specific peptidase 53	USP53
227739_at	2.52		

236305_at	2.52	Rieske (Fe-S) domain containing	RFESD
228184_at	2.52	dispatched homolog 1 (Drosophila)	DISP1
1569250_at	2.52	zinc finger protein 333	ZNF333
201790_s_at	2.52	7-dehydrocholesterol reductase	DHCR7
221291_at	2.52	UL16 binding protein 2	ULBP2
202058_s_at	2.52	karyopherin alpha 1 (importin alpha 5)	KPNA1
209740_s_at	2.52	patatin-like phospholipase domain containing 4	PNPLA4
229532_at	2.52	zinc finger protein 502	ZNF502
1555261_at	2.52		
214277_at	2.52	COX11 homolog, cytochrome c oxidase assembly protein (yeast)	COX11
243038_at	2.52	RNA binding motif protein 43	RBM43
224452_s_at	2.52		
219650_at	2.52		
243548_x_at	2.51		
1569714_at	2.51	zinc finger, FYVE domain containing 20	ZFYVE20
203882_at	2.51	interferon-stimulated transcription factor 3, gamma 48kDa	ISGF3G
240344_x_at	2.51	Lyrm7 homolog (mouse)	LYRM7
226443_at	2.51	family with sequence similarity 122A	FAM122A
241672_at	2.51		
213805_at	2.51		
1559902_at	2.51	megakaryoblastic leukemia (translocation) 1	MKL1
205493_s_at	2.51	dihydropyrimidinase-like 4	DPYSL4
209108_at	2.51	tetraspanin 6	TSPAN6
235387_at	2.51	glutathione S-transferase, C-terminal domain containing	GSTCD
221102_s_at	2.51	transient receptor potential cation channel, subfamily M, member 6	TRPM6
1562484_at	2.51		
234405_s_at	2.50	RNA U, small nuclear RNA export adaptor (phosphorylation regulated)	RNUXA
209681_at	2.50	solute carrier family 19 (thiamine transporter), member 2	SLC19A2
221640_s_at	2.50	leucine-rich repeats and death domain containing	LRDD
212806_at	2.50		
1555758_a_at	2.50	cyclin-dependent kinase inhibitor 3 (CDK2-associated dual specificity phosphatase)	CDKN3
235102_x_at	2.50	GRB2-related adaptor protein	GRAP
214598_at	2.50	claudin 8	CLDN8
207231_at	2.50		
201847_at	2.50	lipase A, lysosomal acid, cholesterol esterase (Wolman disease)	LIPA
243252_at	2.50		
230393_at	2.50		
229220_x_at	2.49	nucleolar protein with MIF4G domain 1	NOM1
235866_at	2.49		
223511_at	2.49	chromosome 1 open reading frame 124	C1orf124
1557838_at	2.49		
1562458_at	2.49	ubiquitin-conjugating enzyme E2W (putative)	UBE2W
231301_at	2.49	nucleoporin 54kDa	NUP54
226790_at	2.49	MORN repeat containing 2	MORN2
1558256_at	2.49		

1554743_x_at	2.49	PMS1 postmeiotic segregation increased 1 (S. cerevisiae)	PMS1
228841_at	2.49		
235453_at	2.49		
220511_s_at	2.48	deleted in liver cancer 1	DLC1
201679_at	2.48		
228390_at	2.48		
232144_at	2.48	pre-B-cell leukemia homeobox 1	PBX1
227267_at	2.48		
226094_at	2.48	phosphoinositide-3-kinase, class 2, alpha polypeptide	PIK3C2A
1556195_a_at	2.48		
232321_at	2.48	mucin 17, cell surface associated	MUC17
214061_at	2.48	WD repeat domain 67	WDR67
210304_at	2.48	phosphodiesterase 6B, cGMP-specific, rod, beta (congenital stationary night blindness 3, autosomal d	PDE6B
232641_at	2.48	zinc finger protein 596#olfactory receptor, family 4, subfamily F, member 21#null	ZNF596#OR4F 21#null
209595_at	2.48	general transcription factor IIF, polypeptide 2, 30kDa	GTF2F2
219951_s_at	2.48	chromosome 20 open reading frame 12	C20orf12
224521_s_at	2.48	coiled-coil domain containing 77	CCDC77
235428_at	2.48		
213701_at	2.48	chromosome 12 open reading frame 29	C12orf29
205347_s_at	2.48	thymosin-like 8	TMSL8
233990_at	2.48		
230281_at	2.48	chromosome 16 open reading frame 46	C16orf46
233127_at	2.48		
1564190_x_at	2.48	zinc finger protein 519	ZNF519
222500_at	2.47	peptidylprolyl isomerase (cyclophilin)-like 1	PPIL1
228717_at	2.47		
1569251_a_at	2.47	zinc finger protein 333	ZNF333
237577_at	2.47	PEST proteolytic signal containing nuclear protein	PCNP
1553702_at	2.47	zinc finger protein 697	ZNF697
212738_at	2.47	Rho GTPase activating protein 19	ARHGAP19
231356_at	2.47	myelin basic protein	MBP
244294_at	2.47		
219875_s_at	2.47	chromosome 1 open reading frame 121	C1orf121
223792_at	2.47	zinc finger protein 2	ZNF2
205694_at	2.46	tyrosinase-related protein 1	TYRP1
229018_at	2.46	chromosome 12 open reading frame 26	C12orf26
210471_s_at	2.46	potassium voltage-gated channel, shaker-related subfamily, beta member 1	KCNAB1
235824_at	2.46		
235456_at	2.46		
219309_at	2.46		
203762_s_at	2.46	dynein, cytoplasmic 2, light intermediate chain 1	DYNC2LI1
217564_s_at	2.46	carbamoyl-phosphate synthetase 1, mitochondrial	CPS1
238510_at	2.46	zinc finger protein 720	ZNF720

225066_at	2.46	protein phosphatase 2, regulatory subunit B, delta isoform	PPP2R2D
209375_at	2.46	xeroderma pigmentosum, complementation group C	XPC
235425_at	2.46	shugoshin-like 2 (S. pombe)	SGOL2
1552579_a_at	2.46	ADAM metallopeptidase domain 21	ADAM21
232980_at	2.45		
215672_s_at	2.45		
225867_at	2.45	vasorin	VASN
1557455_s_at	2.45	motile sperm domain containing 1	MOSPD1
229402_at	2.45	sterile alpha motif domain containing 13	SAMD13
203763_at	2.45	dynein, cytoplasmic 2, light intermediate chain 1	DYNC2LI1
232127_at	2.45	chloride channel 5 (nephrolithiasis 2, X-linked, Dent disease)	CLCN5
236311_at	2.45	loss of heterozygosity, 12, chromosomal region 2	LOH12CR2
237783_at	2.45	PLAC8-like 1	PLAC8L1
1556062_at	2.45	ribonuclease P/MRP 30kDa subunit	RPP30
235021_at	2.45	KIAA2026	KIAA2026
1553983_at	2.45		
207056_s_at	2.45	solute carrier family 4, sodium bicarbonate cotransporter, member 8	SLC4A8
206829_x_at	2.45	zinc finger protein 430	ZNF430
231578_at	2.45	guanylate binding protein 1, interferon-inducible, 67kDa	GBP1
1560570_a_at	2.45		
210820_x_at	2.45	coenzyme Q7 homolog, ubiquinone (yeast)	COQ7
204523_at	2.45	zinc finger protein 140	ZNF140
1564231_at	2.45	intraflagellar transport 80 homolog (Chlamydomonas)	IFT80
231850_x_at	2.45	KIAA1712	KIAA1712
235117_at	2.44	ChaC, cation transport regulator homolog 2 (E. coli)	CHAC2
209466_x_at	2.44	pleiotrophin (heparin binding growth factor 8, neurite growth-promoting factor 1)	PTN
215657_at	2.44		
244763_at	2.44	mitochondrial translational release factor 1	MTRF1
1561417_x_at	2.44		
202124_s_at	2.44	trafficking protein, kinesin binding 2	TRAK2
211080_s_at	2.44	NIMA (never in mitosis gene a)-related kinase 2	NEK2
222765_x_at	2.44	ESF1, nucleolar pre-rRNA processing protein, homolog (S. cerevisiae)#chromosome 20 open reading frame 7	ESF1#C20orf7
1566646_at	2.44		
225915_at	2.44	cytidine and dCMP deaminase domain containing 1#calcium binding protein 39-like	CDADC1#CAB39L
216317_x_at	2.44	Rh blood group, CcEe antigens	RHCE
235308_at	2.44	zinc finger and BTB domain containing 20	ZBTB20
230697_at	2.44	Bardet-Biedl syndrome 5	BBS5
233811_at	2.44	Ras and Rab interactor 2	RIN2
226483_at	2.44	transmembrane protein 68	TMEM68
226588_at	2.44		
219294_at	2.44	centromere protein Q	CENPQ
236623_at	2.44	chromosome 1 open reading frame 203	C1orf203

206280_at	2.44	cadherin 18, type 2	CDH18
1553405_a_at	2.44	CUB and Sushi multiple domains 1	CSMD1
228655_at	2.44		
235696_at	2.43		
214349_at	2.43		
214251_s_at	2.43	nuclear mitotic apparatus protein 1	NUMA1
232331_at	2.43		
1553694_a_at	2.43	phosphoinositide-3-kinase, class 2, alpha polypeptide	PIK3C2A
201707_at	2.43	peroxisomal biogenesis factor 19	PEX19
32042_at	2.43	cytosolic ovarian carcinoma antigen 1	COVA1
201666_at	2.43	TIMP metallopeptidase inhibitor 1	TIMP1
232679_at	2.43		
235761_at	2.43		
229440_at	2.43		
230040_at	2.43	ADAM metallopeptidase with thrombospondin type 1 motif, 18	ADAMTS18
223397_s_at	2.43	nuclear import 7 homolog (S. cerevisiae)	NIP7
210124_x_at	2.43	sema domain, immunoglobulin domain (Ig), transmembrane domain (TM) and short cytoplasmic domain, (se	SEMA4F
232841_at	2.43		
45653_at	2.43	potassium channel tetramerisation domain containing 13	KCTD13
218954_s_at	2.43	BRF2, subunit of RNA polymerase III transcription initiation factor, BRF1-like	BRF2
214537_at	2.43	histone cluster 1, H1d	HIST1H1D
213452_at	2.42	zinc finger protein 184	ZNF184
207598_x_at	2.42	X-ray repair complementing defective repair in Chinese hamster cells 2	XRCC2
243815_at	2.42	piggyBac transposable element derived 4	PGBD4
226346_at	2.42		
237247_at	2.42	ubiquitin specific peptidase 51	USP51
232656_at	2.42		
204804_at	2.42	tripartite motif-containing 21	TRIM21
230083_at	2.42		
244743_x_at	2.42	zinc finger protein 138	ZNF138
242251_at	2.42		
231472_at	2.42	F-box protein 15	FBXO15
215220_s_at	2.42	translocated promoter region (to activated MET oncogene)	TPR
205070_at	2.42	inhibitor of growth family, member 3	ING3
239188_at	2.42	protein phosphatase 2 (formerly 2A), regulatory subunit B'', gamma	PPP2R3C
224828_at	2.42	cytoplasmic polyadenylation element binding protein 4	CPEB4
206613_s_at	2.42	TATA box binding protein (TBP)-associated factor, RNA polymerase I, A, 48kDa	TAF1A
235064_s_at	2.42	chromosome 20 open reading frame 196	C20orf196
1558673_s_at	2.41	zinc finger protein 77	ZNF77
205260_s_at	2.41	acylphosphatase 1, erythrocyte (common) type	ACYP1
1552933_at	2.41	chromosome 1 open reading frame 62	C1orf62
1553395_a_at	2.41	CD200 receptor 1	CD200R1

220321_s_at	2.41	coiled-coil domain containing 121	CCDC121
237465_at	2.41	ubiquitin specific peptidase 53	USP53
1558372_at	2.41		
239835_at	2.41	kelch repeat and BTB (POZ) domain containing 8	KBTBD8
235653_s_at	2.41	THAP domain containing 6	THAP6
212113_at	2.41		
231546_at	2.41	scavenger receptor class A, member 5 (putative)	SCARA5
226890_at	2.41	WD repeat domain 35	WDR35
230391_at	2.41		
238475_at	2.41		
239960_x_at	2.41	Lyrm7 homolog (mouse)	LYRM7
226195_at	2.41	chromosome 14 open reading frame 179	C14orf179
206957_at	2.41	alanine-glyoxylate aminotransferase (oxalosis I; hyperoxaluria I; glycolicaciduria; serine-pyruvate	AGXT
240793_at	2.41	titin	TTN
230862_at	2.41	ribophorin I	RPN1
225028_at	2.41		
223229_at	2.40	ubiquitin-conjugating enzyme E2T (putative)	UBE2T
238952_x_at	2.40		
223784_at	2.40	transmembrane protein 27	TMEM27
226537_at	2.40	histidine triad nucleotide binding protein 3	HINT3
244631_at	2.40		
207402_at	2.40	zinc finger protein 132	ZNF132
218784_s_at	2.40	chromosome 6 open reading frame 64	C6orf64
1552370_at	2.40	chromosome 4 open reading frame 33	C4orf33
243982_at	2.40	kelch-like 28 (Drosophila)	KLHL28
225834_at	2.40		
213115_at	2.40	collagen, type IV, alpha 6#proteasome (prosome, macropain) 26S subunit, non-ATPase, 10#ATG4 autophagy related 4 homolog A (S. cerevisiae)#V-set and immunoglobulin domain containing 1	COL4A6#PSMD10#ATG4A#VSIG1
235493_at	2.40		
1557192_at	2.40	chromosome 1 open reading frame 136	C1orf136
202742_s_at	2.39	protein kinase, cAMP-dependent, catalytic, beta	PRKACB
203801_at	2.39		
208115_x_at	2.39	chromosome 10 open reading frame 137	C10orf137
235727_at	2.39	kelch-like 28 (Drosophila)	KLHL28
210680_s_at	2.39	mannan-binding lectin serine peptidase 1 (C4/C2 activating component of Ra-reactive factor)	MASP1
1553328_a_at	2.39	solute carrier family 18 (vesicular monoamine), member 2	SLC18A2
217321_x_at	2.39	ataxin 3	ATXN3
211734_s_at	2.39	Fc fragment of IgE, high affinity I, receptor for; alpha polypeptide	FCER1A
221596_s_at	2.39		
213704_at	2.39	Rab geranylgeranyltransferase, beta subunit	RABGGTB
1569706_at	2.39	myb-like, SWIRM and MPN domains 1	MYSM1
235839_at	2.39		
217047_s_at	2.39	family with sequence similarity 13, member A1	FAM13A1
1570039_at	2.39		
1553810_a_at	2.39	KIAA1524	KIAA1524
219019_at	2.39	leucine-rich repeats and death domain containing	LRDD

206493_at	2.38	integrin, alpha 2b (platelet glycoprotein IIb of IIb/IIIa complex, antigen CD41)	ITGA2B
1559240_at	2.38		
1556763_at	2.38		
239047_at	2.38	family with sequence similarity 122C	FAM122C
207528_s_at	2.38	solute carrier family 7, (cationic amino acid transporter, y+ system) member 11	SLC7A11
1557701_s_at	2.38	polymerase (DNA directed), eta	POLH
224831_at	2.38	cytoplasmic polyadenylation element binding protein 4	CPEB4
200895_s_at	2.38	FK506 binding protein 4, 59kDa	FKBP4
221043_at	2.38		
1562209_at	2.38	WD repeat domain 21B	WDR21B
216956_s_at	2.38	integrin, alpha 2b (platelet glycoprotein IIb of IIb/IIIa complex, antigen CD41)	ITGA2B
230177_at	2.38		
228766_at	2.38	CD36 molecule (thrombospondin receptor)	CD36
223629_at	2.38	protocadherin beta 5	PCDHB5
230324_at	2.38	nuclear receptor coactivator 2	NCOA2
211468_s_at	2.38	RecQ protein-like 5	RECQL5
205383_s_at	2.38	zinc finger and BTB domain containing 20	ZBTB20
214913_at	2.38	ADAM metallopeptidase with thrombospondin type 1 motif, 3	ADAMTS3
213122_at	2.38	TSPY-like 5	TSPYL5
222809_x_at	2.38	chromosome 14 open reading frame 65	C14orf65
233852_at	2.37	polymerase (DNA directed), eta	POLH
238920_at	2.37		
219756_s_at	2.37	premature ovarian failure, 1B	POF1B
49452_at	2.37	acetyl-Coenzyme A carboxylase beta	ACACB
222885_at	2.37	endomucin	EMCN
235554_x_at	2.37	chromosome 4 open reading frame 28	C4orf28
1555350_at	2.37	periphilin 1	PPHLN1
1557948_at	2.37	pleckstrin homology-like domain, family B, member 3	PHLDB3
228430_at	2.37		
234465_at	2.37	essential meiotic endonuclease 1 homolog 1 (S. pombe)	EME1
207183_at	2.37	G protein-coupled receptor 19	GPR19
1557745_at	2.37		
242116_x_at	2.37	ankyrin repeat domain 17	ANKRD17
232278_s_at	2.37	DEP domain containing 1	DEPDC1
218739_at	2.37	abhydrolase domain containing 5	ABHD5
227559_at	2.37		
215597_x_at	2.37	MYST histone acetyltransferase (monocytic leukemia) 4	MYST4
232024_at	2.37	GTPase, IMAP family member 2	GIMAP2
228971_at	2.37		
1554588_a_at	2.37	tetratricopeptide repeat domain 30B	TTC30B
240230_s_at	2.37		
242293_at	2.37	inhibitor of growth family, member 3	ING3
230329_s_at	2.36	nudix (nucleoside diphosphate linked moiety X)-type motif 6	NUDT6
227438_at	2.36	alpha-kinase 1	ALPK1

1564757_a_at	2.36		
233940_at	2.36		
219617_at	2.36	chromosome 2 open reading frame 34	C2orf34
243055_at	2.36		
206789_s_at	2.36	POU domain, class 2, transcription factor 1	POU2F1
218999_at	2.36	transmembrane protein 140	TMEM140
1555090_x_at	2.36	transmembrane protein 182	TMEM182
1558348_at	2.36		
229107_at	2.36		
242431_at	2.36	ariadne homolog, ubiquitin-conjugating enzyme E2 binding protein, 1 (Drosophila)	ARIH1
238843_at	2.36	nephronophthisis 1 (juvenile)	NPHP1
204753_s_at	2.36	hepatic leukemia factor	HLF
237236_x_at	2.36		
243153_at	2.36	CDK5 regulatory subunit associated protein 2	CDK5RAP2
235571_at	2.36		
230021_at	2.36	chromosome 15 open reading frame 42	C15orf42
220230_s_at	2.36	cytochrome b5 reductase 2	CYB5R2
220324_at	2.36	chromosome 6 open reading frame 155	C6orf155
1554321_a_at	2.36	NFS1 nitrogen fixation 1 homolog (S. cerevisiae)	NFS1
244641_at	2.36	chromosome 7 open reading frame 30	C7orf30
242458_at	2.35	Ral GEF with PH domain and SH3 binding motif 2	RALGPS2
214414_x_at	2.35	hemoglobin, alpha 1	HBA1
241106_at	2.35	serologically defined colon cancer antigen 10	SDCCAG10
1556725_a_at	2.35		
201138_s_at	2.35	Sjogren syndrome antigen B (autoantigen La)	SSB
243943_x_at	2.35	chromosome 6 open reading frame 52	C6orf52
244762_at	2.35	Down syndrome critical region gene 3	DSCR3
232398_at	2.35		
240158_at	2.35		
236097_at	2.35		
229582_at	2.35	chromosome 18 open reading frame 37	C18orf37
1558512_at	2.35		
1558686_at	2.35		
220117_at	2.35	zinc finger protein 659	ZNF659
1564876_s_at	2.35	forkhead box P2	FOXP2
209547_s_at	2.35	splicing factor 4	SF4
218987_at	2.35	activating transcription factor 7 interacting protein	ATF7IP
235346_at	2.35	FUN14 domain containing 1	FUNDC1
224453_s_at	2.35	ethanolamine kinase 1	ETNK1
212253_x_at	2.35	dystonin	DST
208670_s_at	2.35	EP300 interacting inhibitor of differentiation 1	EID1
232013_at	2.35	chromosome 9 open reading frame 102	C9orf102
227273_at	2.35		
208922_s_at	2.34	nuclear RNA export factor 1	NXF1
236910_at	2.34	mitochondrial ribosomal protein L39	MRPL39
219980_at	2.34	chromosome 4 open reading frame 29	C4orf29
219628_at	2.34	zinc finger, matrin type 3	ZMAT3
205716_at	2.34	solute carrier family 25, member 40	SLC25A40
204511_at	2.34	FERM, RhoGEF and pleckstrin domain protein 2	FARP2
226446_at	2.34	hairy and enhancer of split 6 (Drosophila)	HES6
236619_at	2.34		

1553120_at	2.34	claspin homolog (Xenopus laevis)	CLSPN
207064_s_at	2.34	amine oxidase, copper containing 2 (retina-specific)	AOC2
236646_at	2.34	chromosome 12 open reading frame 59	C12orf59
205964_at	2.34	zinc finger protein 426	ZNF426
218538_s_at	2.34	MRS2-like, magnesium homeostasis factor (S. cerevisiae)	MRS2L
242533_at	2.34	transmembrane protein 112	TMEM112
220168_at	2.34	cancer susceptibility candidate 1	CASC1
219822_at	2.34	mitochondrial translational release factor 1	MTRF1
215350_at	2.34	spectrin repeat containing, nuclear envelope 1	SYNE1
239936_at	2.34	deleted in lymphocytic leukemia, 2	DLEU2
240247_at	2.34	family with sequence similarity 7, member A2	FAM7A2
238490_at	2.34	KIAA2026	KIAA2026
237746_at	2.34	splicing factor, arginine/serine-rich 11	SFRS11
1558969_a_at	2.33	ribosomal protein L32 pseudogene 3	RPL32P3
221786_at	2.33	chromosome 6 open reading frame 120	C6orf120
242650_at	2.33		
225725_at	2.33		
233376_at	2.33		
223478_at	2.33	translocase of inner mitochondrial membrane 8 homolog B (yeast)	TIMM8B
212121_at	2.33	chromosome 10 open reading frame 61	C10orf61
207691_x_at	2.33	ectonucleoside triphosphate diphosphohydrolase 1	ENTPD1
222573_s_at	2.33	salvador homolog 1 (Drosophila)	SAV1
211279_at	2.33	nuclear respiratory factor 1	NRF1
200664_s_at	2.33	DnaJ (Hsp40) homolog, subfamily B, member 1	DNAJB1
222962_s_at	2.33	MCM10 minichromosome maintenance deficient 10 (S. cerevisiae)	MCM10
233372_at	2.33		
203213_at	2.33	cell division cycle 2, G1 to S and G2 to M	CDC2
1570130_at	2.33	spermatogenesis associated, serine-rich 2	SPATS2
208900_s_at	2.33	topoisomerase (DNA) I	TOP1
210462_at	2.33	basic leucine zipper nuclear factor 1 (JEM-1)	BLZF1
216039_at	2.33	postmeiotic segregation increased 2-like 1	PMS2L1
221705_s_at	2.33		
1569190_at	2.33	sodium channel and clathrin linker 1	SCLT1
204008_at	2.33	dynein, axonemal, light chain 4	DNAL4
232272_at	2.32	zinc finger protein 624	ZNF624
229784_at	2.32		
220716_at	2.32		
237083_at	2.32		
1554915_a_at	2.32		
1561187_at	2.32		
241053_at	2.32		
1561685_a_at	2.32		
227337_at	2.32	ankyrin repeat domain 37	ANKRD37
234464_s_at	2.32	essential meiotic endonuclease 1 homolog 1 (S. pombe)	EME1
214760_at	2.32	zinc finger protein 337	ZNF337
222642_s_at	2.32	transmembrane protein 33	TMEM33
208527_x_at	2.32	histone cluster 1, H2be	HIST1H2BE

202705_at	2.32	cyclin B2	CCNB2
227127_at	2.32	transmembrane protein 110	TMEM110
207045_at	2.32	coiled-coil domain containing 132	CCDC132
1570596_at	2.32		
1559982_s_at	2.32	aldo-keto reductase family 1, member C-like 2	AKR1CL2
222886_at	2.32		
1563693_at	2.32		
226787_at	2.31	zinc finger protein 18	ZNF18
219651_at	2.31	developmental pluripotency associated 4	DPPA4
213933_at	2.31	prostaglandin E receptor 3 (subtype EP3)	PTGER3
209183_s_at	2.31	chromosome 10 open reading frame 10	C10orf10
1564002_a_at	2.31	chromosome 6 open reading frame 199	C6orf199
238825_at	2.31	acidic repeat containing	ACRC
213234_at	2.31	KIAA1467	KIAA1467
238964_at	2.31		
1563858_at	2.31		
206383_s_at	2.31	GTPase activating protein (SH3 domain) binding protein 2	G3BP2
238529_at	2.31		
228147_at	2.31		
226749_at	2.31	mitochondrial ribosomal protein S9	MRPS9
244502_at	2.31		
227172_at	2.31	transmembrane protein 116	TMEM116
1569600_at	2.31	deleted in lymphocytic leukemia, 2	DLEU2
210597_x_at	2.31	proline-rich protein BstNI subfamily 1	PRB1
239701_at	2.31		
1561364_at	2.31		
222396_at	2.31	hematological and neurological expressed 1	HN1
213679_at	2.31	tetratricopeptide repeat domain 30A	TTC30A
244546_at	2.31	cytochrome c, somatic	CYCS
225981_at	2.30	chromosome 17 open reading frame 28	C17orf28
1561114_a_at	2.30	DEP domain containing 4	DEPDC4
212824_at	2.30	far upstream element (FUSE) binding protein 3	FUBP3
223458_at	2.30	seizure related 6 homolog (mouse)-like 2	SEZ6L2
1558906_a_at	2.30		
203947_at	2.30	cleavage stimulation factor, 3' pre-RNA, subunit 3, 77kDa	CSTF3
228033_at	2.30	E2F transcription factor 7	E2F7
219844_at	2.30	chromosome 10 open reading frame 118	C10orf118
232163_at	2.30	WD repeat domain 19	WDR19
1554509_a_at	2.30	chromosome 10 open reading frame 97	C10orf97
222170_at	2.30		
242248_at	2.30	phosphorylase kinase, beta	PHKB
203176_s_at	2.30	transcription factor A, mitochondrial	TFAM
1564215_at	2.30		
207630_s_at	2.30	cAMP responsive element modulator	CREM
1553192_at	2.30	zinc finger protein 441	ZNF441
226250_at	2.30		
228431_at	2.30		
227223_at	2.30	RNA binding motif protein 39	RBM39
238614_x_at	2.30	zinc finger protein 430	ZNF430
218516_s_at	2.30	inositol monophosphatase domain containing 1	IMPAD1
208066_s_at	2.30	general transcription factor IIB	GTF2B

215132_at	2.30		
210976_s_at	2.30	phosphofructokinase, muscle	PFKM
232585_at	2.29	tousled-like kinase 2	TLK2
244075_at	2.29	hydroxysteroid (17-beta) dehydrogenase 7 pseudogene 2	HSD17B7P2
238057_at	2.29	ubiquitin specific peptidase 45	USP45
238453_at	2.29	fibroblast growth factor binding protein 3	FGFBP3
1555900_at	2.29		
1552711_a_at	2.29	cytochrome b5 domain containing 1	CYB5D1
226196_s_at	2.29	chromosome 14 open reading frame 179	C14orf179
1554256_a_at	2.29	pecanex-like 2 (Drosophila)	PCNXL2
228433_at	2.29		
1556347_at	2.29		
224822_at	2.29	deleted in liver cancer 1	DLC1
238732_at	2.29	collagen, type XXIV, alpha 1	COL24A1
235785_at	2.29		
218043_s_at	2.29	5-azacytidine induced 2	AZI2
217597_x_at	2.29	RAB40B, member RAS oncogene family	RAB40B
1558740_s_at	2.29		
203610_s_at	2.29	tripartite motif-containing 38	TRIM38
205649_s_at	2.29	fibrinogen alpha chain	FGA
209185_s_at	2.29	insulin receptor substrate 2	IRS2
1554518_at	2.29	glutathione S-transferase, C-terminal domain containing	GSTCD
235017_s_at	2.29		
229338_at	2.29		
213930_at	2.29		
228003_at	2.29	RAB30, member RAS oncogene family	RAB30
231774_at	2.29	Kv channel interacting protein 3, calsenilin	KCNIP3
220844_at	2.28	transcription elongation factor B polypeptide 3B (elongin A2)	TCEB3B
239819_at	2.28		
226747_at	2.28	KIAA1344	KIAA1344
215820_x_at	2.28	sorting nexin 13	SNX13
243041_s_at	2.28	RNA binding motif, single stranded interacting protein	RBMS3
1554029_a_at	2.28	KIAA0372	KIAA0372
239265_at	2.28	transmembrane protein 20	TMEM20
208392_x_at	2.28	SP110 nuclear body protein	SP110
204643_s_at	2.28	cytosolic ovarian carcinoma antigen 1	COVA1
218680_x_at	2.28		
236897_at	2.28		
232449_at	2.28	beta-carotene dioxygenase 2	BCDO2
217396_at	2.28		
1552684_a_at	2.28	SUMO/sentrin specific peptidase family member 8	SENP8
227601_at	2.28		
210050_at	2.28	triosephosphate isomerase 1	TPI1
239346_at	2.27		
243064_at	2.27		
223181_at	2.27	chromosome 18 open reading frame 55	C18orf55
1568658_at	2.27		
207850_at	2.27	chemokine (C-X-C motif) ligand 3	CXCL3

228305_at	2.27	zinc finger protein 565	ZNF565
219177_at	2.27	brix domain containing 2	BXDC2
219345_at	2.27	bolA homolog 1 (E. coli)	BOLA1
201243_s_at	2.27	ATPase, Na+/K+ transporting, beta 1 polypeptide	ATP1B1
1557985_s_at	2.27	centrosomal protein 78kDa	CEP78
204133_at	2.27	RRP9, small subunit (SSU) processome component, homolog (yeast)	RRP9
218135_at	2.27	ERGIC and golgi 2	ERGIC2
1555384_a_at	2.27	La ribonucleoprotein domain family, member 4	LARP4
208671_at	2.26	serine incorporator 1	SERINC1
236546_at	2.26		
213761_at	2.26	Mdm4, transformed 3T3 cell double minute 1, p53 binding protein (mouse)	MDM1
1560806_at	2.26		
229659_s_at	2.26	polymeric immunoglobulin receptor	PIGR
203145_at	2.26	sperm associated antigen 5	SPAG5
202884_s_at	2.26	protein phosphatase 2 (formerly 2A), regulatory subunit A, beta isoform	PPP2R1B
209739_s_at	2.26	patatin-like phospholipase domain containing 4	PNPLA4
228208_x_at	2.26	zinc finger protein 354C	ZNF354C
210215_at	2.26	transferrin receptor 2	TFR2
1563841_at	2.26		
239802_at	2.26		
203011_at	2.26	inositol(myo)-1(or 4)-monophosphatase 1	IMPA1
202095_s_at	2.26	baculoviral IAP repeat-containing 5 (survivin)	BIRC5
236217_at	2.26	solute carrier family 31 (copper transporters), member 1	SLC31A1
219154_at	2.26	ras homolog gene family, member F (in filopodia)	RHOF
1559186_at	2.26	protein kinase, X-linked, pseudogene 1	PRKXP1
223613_at	2.26	ubiquinol-cytochrome c reductase, 6.4kDa subunit	UQCR
238783_at	2.26	transmembrane protein 161B	TMEM161B
238647_at	2.26	chromosome 14 open reading frame 28	C14orf28
212936_at	2.26	chromosome 5 open reading frame 21	C5orf21
240769_at	2.26		
230578_at	2.25		
1566472_s_at	2.25		
231653_at	2.25	coiled-coil domain containing 129	CCDC129
239592_at	2.25		
206910_x_at	2.25	complement factor H-related 2	CFHR2
230095_at	2.25	thioredoxin-like 2	TXNL2
223124_s_at	2.25	chromosome 1 open reading frame 128	C1orf128
214829_at	2.25	aminoadipate-semialdehyde synthase	AASS
224474_x_at	2.25	SMEK homolog 2, suppressor of mek1 (Dictyostelium)	SMEK2
238565_at	2.25		
205063_at	2.25	survival of motor neuron protein interacting protein 1	SIP1
242070_at	2.25		
218513_at	2.25		
223036_at	2.25	phenylalanine-tRNA synthetase-like, beta subunit	FARSLB
239466_at	2.25		
203913_s_at	2.25	hydroxyprostaglandin dehydrogenase 15-(NAD)	HPGD

207551_s_at	2.25	male-specific lethal 3-like 1 (Drosophila)	MSL3L1
233204_at	2.25		
1552306_at	2.25	asparagine-linked glycosylation 10 homolog (yeast, alpha-1,2-glucosyltransferase)	ALG10
224723_x_at	2.25		
215239_x_at	2.25	zinc finger protein 273	ZNF273
244265_at	2.24	arginine-glutamic acid dipeptide (RE) repeats	RERE
204886_at	2.24	polo-like kinase 4 (Drosophila)	PLK4
219838_at	2.24	tetratricopeptide repeat domain 23	TTC23
232692_at	2.24	tudor domain containing 6	TDRD6
202893_at	2.24	unc-13 homolog B (C. elegans)	UNC13B
212009_s_at	2.24	stress-induced-phosphoprotein 1 (Hsp70/Hsp90-organizing protein)	STIP1
222729_at	2.24		
213599_at	2.24	Opa interacting protein 5	OIP5
227091_at	2.24		
219728_at	2.24	myotilin	MYOT
232018_at	2.24	leukocyte receptor cluster (LRC) member 1	LENG1
239406_at	2.24	zinc finger protein 193	ZNF193
232462_s_at	2.24		
204525_at	2.24	PHD finger protein 14	PHF14
43427_at	2.24	acetyl-Coenzyme A carboxylase beta	ACACB
203321_s_at	2.24	zinc finger protein 508	ZNF508
220843_s_at	2.24	WD repeats and SOF1 domain containing	WDSOF1
204438_at	2.24	mannose receptor, C type 1	MRC1
222956_at	2.24	fidgetin	FIGN
238491_at	2.23		
1557120_at	2.23	eukaryotic translation elongation factor 1 alpha 1	EEF1A1
231950_at	2.23	zinc finger protein 658	ZNF658
244455_at	2.23	potassium channel, subfamily T, member 2	KCNT2
1558605_at	2.23		
227022_at	2.23	glucosamine-6-phosphate deaminase 2	GNPDA2
206175_x_at	2.23	zinc finger protein 222	ZNF222
1553633_s_at	2.23		
200868_s_at	2.23	zinc finger protein 313	ZNF313
223434_at	2.23	guanylate binding protein 3	GBP3
202733_at	2.23	procollagen-proline, 2-oxoglutarate 4-dioxygenase (proline 4-hydroxylase), alpha polypeptide II	P4HA2
218185_s_at	2.23	armadillo repeat containing 1	ARMC1
1563112_at	2.23	family with sequence similarity 120A opposite strand	FAM120AOS
1559461_at	2.23	centrosomal protein 72kDa	CEP72
229260_at	2.23	chromosome 5 open reading frame 15	C5orf15
244359_s_at	2.23		
221928_at	2.23	acetyl-Coenzyme A carboxylase beta	ACACB
214204_at	2.23	PARK2 co-regulated	PACRG
222827_s_at	2.23	kelch repeat and BTB (POZ) domain containing 10	KBTBD10
212597_s_at	2.23	high-mobility group protein 2-like 1	HMG2L1
225769_at	2.23	component of oligomeric golgi complex 6	COG6
204409_s_at	2.23	eukaryotic translation initiation factor 1A, Y-linked	EIF1AY
226069_at	2.23	prickle homolog 1 (Drosophila)	PRICKLE1

211251_x_at	2.23	nuclear transcription factor Y, gamma	NFYC
204240_s_at	2.23	structural maintenance of chromosomes 2	SMC2
234304_s_at	2.22	importin 11	IPO11
1552400_a_at	2.22	chromosome 15 open reading frame 27	C15orf27
221669_s_at	2.22	acyl-Coenzyme A dehydrogenase family, member 8	ACAD8
242333_at	2.22		
236957_at	2.22	cell division cycle associated 2	CDCA2
209666_s_at	2.22	conserved helix-loop-helix ubiquitous kinase	CHUK
221915_s_at	2.22	RAN binding protein 1	RANBP1
223644_s_at	2.22	crystallin, gamma S	CRYGS
216103_at	2.22	acyl-CoA thioesterase 11	ACOT11
232696_at	2.22		
204871_at	2.22	mitochondrial transcription termination factor	MTERF
206025_s_at	2.22	tumor necrosis factor, alpha-induced protein 6	TNFAIP6
218470_at	2.22	tyrosyl-tRNA synthetase 2 (mitochondrial)	YARS2
204011_at	2.22	sprouty homolog 2 (Drosophila)	SPRY2
202270_at	2.22	guanylate binding protein 1, interferon-inducible, 67kDa	GBP1
238914_at	2.22		
1562988_at	2.22		
235541_at	2.22	LAS1-like (S. cerevisiae)	LAS1L
230657_at	2.22		
1553512_at	2.22	homeobox C12	HOXC12
232228_at	2.22	zinc finger protein 530	ZNF530
221696_s_at	2.22	serine/threonine/tyrosine kinase 1	STYK1
202577_s_at	2.22	DEAD (Asp-Glu-Ala-As) box polypeptide 19A	DDX19A
221201_s_at	2.22	zinc finger protein 155	ZNF155
211095_at	2.22	neurofibromin 1 (neurofibromatosis, von Recklinghausen disease, Watson disease)	NF1
210559_s_at	2.21	cell division cycle 2, G1 to S and G2 to M	CDC2
1559922_at	2.21		
219446_at	2.21	resistance to inhibitors of cholinesterase 8 homolog B (C. elegans)	RIC8B
216775_at	2.21	ubiquitin specific peptidase 53	USP53
222890_at	2.21	coiled-coil domain containing 113	CCDC113
202581_at	2.21	heat shock 70kDa protein 1B	HSPA1B
203711_s_at	2.21	3-hydroxyisobutyryl-Coenzyme A hydrolase	HIBCH
203158_s_at	2.21	glutaminase	GLS
218859_s_at	2.21	ESF1, nucleolar pre-rRNA processing protein, homolog (S. cerevisiae)	ESF1
234995_at	2.21	coiled-coil domain containing 52	CCDC52
221588_x_at	2.21	aldehyde dehydrogenase 6 family, member A1	ALDH6A1
49077_at	2.21	protein phosphatase methylesterase 1	PPME1
234095_at	2.21		
235069_at	2.21	TatD DNase domain containing 3	TATDN3
222805_at	2.21	mannosidase, endo-alpha	MANEA
209075_s_at	2.21	iron-sulfur cluster scaffold homolog (E. coli)	ISCU
242037_at	2.21	aspartate beta-hydroxylase	ASPH
224678_at	2.21	KIAA1219	KIAA1219
206637_at	2.21	purinergic receptor P2Y, G-protein coupled, 14	P2RY14
217050_at	2.21		

227812_at	2.21	tumor necrosis factor receptor superfamily, member 19	TNFRSF19
1556224_a_at	2.21		
231031_at	2.20		
1556602_at	2.20	solute carrier family 19 (thiamine transporter), member 2	SLC19A2
1559477_s_at	2.20	Meis1, myeloid ecotropic viral integration site 1 homolog (mouse)	MEIS1
232429_at	2.20		
220473_s_at	2.20	zinc finger, CCHC domain containing 4	ZCCHC4
214668_at	2.20		
228201_at	2.20	ADP-ribosylation factor-like 13B	ARL13B
232686_at	2.20	sialic acid binding Ig-like lectin, pseudogene 3	SIGLECP3
232139_s_at	2.20	KIAA1919	KIAA1919
205776_at	2.20	flavin containing monooxygenase 5	FMO5
1569318_at	2.20		
201242_s_at	2.20	ATPase, Na+/K+ transporting, beta 1 polypeptide	ATP1B1
232242_at	2.20		
221220_s_at	2.20	SCY1-like 2 (S. cerevisiae)	SCYL2
241115_at	2.20	KIAA1467	KIAA1467
237864_at	2.20		
219455_at	2.20		
240768_x_at	2.20		
230254_at	2.20		
231846_at	2.20	FAD-dependent oxidoreductase domain containing 2	FOXRED2
223576_at	2.20	chromosome 6 open reading frame 203	C6orf203
214929_s_at	2.20	KIAA1109	KIAA1109
231939_s_at	2.20	B double prime 1, subunit of RNA polymerase III transcription initiation factor IIIB	BDP1
211398_at	2.20	fibroblast growth factor receptor 2 (bacteria-expressed kinase, keratinocyte growth factor receptor,	FGFR2
235697_at	2.20		
239482_x_at	2.20	zinc finger protein 708	ZNF708
216786_at	2.20		
208042_at	2.19	angiogenic factor with G patch and FHA domains 1	AGGF1
221962_s_at	2.19	ubiquitin-conjugating enzyme E2H (UBC8 homolog, yeast)	UBE2H
204109_s_at	2.19	nuclear transcription factor Y, alpha	NFYA
217010_s_at	2.19	cell division cycle 25 homolog C (S. pombe)	CDC25C
232559_at	2.19		
228486_at	2.19	solute carrier family 44, member 1	SLC44A1
1557278_s_at	2.19	transportin 1	TNPO1
226803_at	2.19	chromatin modifying protein 4C	CHMP4C
238971_at	2.19		
225855_at	2.19	erythrocyte membrane protein band 4.1 like 5	EPB41L5
211760_s_at	2.19	vesicle-associated membrane protein 4	VAMP4
228050_at	2.19	UTP15, U3 small nucleolar ribonucleoprotein, homolog (S. cerevisiae)	UTP15
217960_s_at	2.19	translocase of outer mitochondrial membrane 22 homolog (yeast)	TOMM22

220237_at	2.19	ATG3 autophagy related 3 homolog (S. cerevisiae)	ATG3
221750_at	2.19	3-hydroxy-3-methylglutaryl-Coenzyme A synthase 1 (soluble)	HMGCS1
227856_at	2.19	chromosome 4 open reading frame 32	C4orf32
215624_at	2.19	tuberous sclerosis 2	TSC2
223825_at	2.19	KIAA1432	KIAA1432
1555046_at	2.18	centromere protein I	CENPI
203404_at	2.18	armadillo repeat containing, X-linked 2	ARMCX2
242562_at	2.18	DPH4, JJJ3 homolog (S. cerevisiae)	DPH4
214862_x_at	2.18		
220969_s_at	2.18		
210867_at	2.18	CCR4-NOT transcription complex, subunit 4	CNOT4
203265_s_at	2.18	mitogen-activated protein kinase kinase 4	MAP2K4
236766_at	2.18	chromosome 8 open reading frame 38	C8orf38
1555166_a_at	2.18	zinc finger protein 396	ZNF396
235423_at	2.18		
219479_at	2.18	KDEL (Lys-Asp-Glu-Leu) containing 1	KDELC1
1557300_s_at	2.18		
225062_at	2.18		
1554193_s_at	2.18	mannosidase, endo-alpha	MANEA
220234_at	2.18	carbonic anhydrase VIII	CA8
218196_at	2.18	osteopetrosis associated transmembrane protein 1	OSTM1
220474_at	2.18	solute carrier family 25 (mitochondrial oxodicarboxylate carrier), member 21	SLC25A21
242134_at	2.18		
58367_s_at	2.18	zinc finger protein 419	ZNF419
205978_at	2.18	klotho	KL
221492_s_at	2.18	ATG3 autophagy related 3 homolog (S. cerevisiae)	ATG3
223538_at	2.18	small EDRK-rich factor 1A (telomeric)	SERF1A
1569495_at	2.18	sodium channel and clathrin linker 1	SCLT1
1566340_at	2.18	small nucleolar RNA, C/D box 8	SNORD8
226432_at	2.18	ethanolamine kinase 1	ETNK1
224734_at	2.17	high-mobility group box 1	HMGB1
219494_at	2.17	RAD54 homolog B (S. cerevisiae)	RAD54B
217593_at	2.17	zinc finger protein 447	ZNF447
205420_at	2.17	peroxisomal biogenesis factor 7	PEX7
207304_at	2.17	zinc finger protein 45	ZNF45
218717_s_at	2.17	leprecan-like 1	LEPREL1
211204_at	2.17	malic enzyme 1, NADP(+)-dependent, cytosolic	ME1
239151_at	2.17	centaurin, gamma-like family, member 6	CTGLF6
236074_at	2.17		
206207_at	2.17	Charcot-Leyden crystal protein	CLC
1556794_at	2.17		
202768_at	2.17	FBJ murine osteosarcoma viral oncogene homolog B	FOSB
212815_at	2.17	activating signal cointegrator 1 complex subunit 3	ASCC3
215128_at	2.17		
240700_at	2.17		
203214_x_at	2.17	cell division cycle 2, G1 to S and G2 to M	CDC2

208124_s_at	2.17	sema domain, immunoglobulin domain (Ig), transmembrane domain (TM) and short cytoplasmic domain, (se	SEMA4F
227084_at	2.17	dystrobrevin, alpha	DTNA
209746_s_at	2.17	coenzyme Q7 homolog, ubiquinone (yeast)	COQ7
201214_s_at	2.17	protein phosphatase 1, regulatory (inhibitor) subunit 7	PPP1R7
229586_at	2.17	chromodomain helicase DNA binding protein 9	CHD9
235572_at	2.17	spindle pole body component 24 homolog (S. cerevisiae)	SPBC24
215905_s_at	2.17	WD repeat domain 57 (U5 snRNP specific)	WDR57
1568834_s_at	2.17	coiled-coil domain containing 90B	CCDC90B
225392_at	2.17	G elongation factor, mitochondrial 2	GFM2
222130_s_at	2.17	FtsJ homolog 2 (E. coli)	FTSJ2
239494_at	2.17		
225640_at	2.17		
1558254_s_at	2.17	SFRS protein kinase 2	SRPK2
222958_s_at	2.17	DEP domain containing 1	DEPDC1
231542_at	2.16		
218373_at	2.16	AKT interacting protein	AKTIP
239760_at	2.16		
225956_at	2.16		
228029_at	2.16	ATP-binding cassette, sub-family A (ABC1), member 11 (pseudogene)	ABCA11
239298_at	2.16		
235218_x_at	2.16	THAP domain containing 6	THAP6
205130_at	2.16	renal tumor antigen	RAGE
242628_at	2.16	killer cell lectin-like receptor subfamily B, member 1	KLRB1
1557399_at	2.16		
1554476_x_at	2.16	zinc finger protein 808	ZNF808
219472_at	2.16	centromere protein O	CENPO
214901_at	2.16	zinc finger protein 8	ZNF8
219427_at	2.16	FAT tumor suppressor homolog 4 (Drosophila)	FAT4
228824_s_at	2.16	leukotriene B4 12-hydroxydehydrogenase	LTB4DH
229415_at	2.16	cytochrome c, somatic	CYCS
1561528_at	2.16		
202305_s_at	2.16	fasciculation and elongation protein zeta 2 (zygin II)	FEZ2
203894_at	2.16	tubulin, gamma 2	TUBG2
1556306_at	2.16		
218360_at	2.16	RAB22A, member RAS oncogene family	RAB22A
204392_at	2.16	calcium/calmodulin-dependent protein kinase I	CAMK1
240868_at	2.16	CTTNBP2 N-terminal like	CTTNBP2NL
205624_at	2.16	carboxypeptidase A3 (mast cell)	CPA3
212510_at	2.16	glycerol-3-phosphate dehydrogenase 1-like	GPD1L
208744_x_at	2.16	heat shock 105kDa/110kDa protein 1	HSPH1
228809_at	2.16		
206240_s_at	2.15	zinc finger protein 136	ZNF136
240061_at	2.15		
1569808_at	2.15		
232273_at	2.15		
1560720_at	2.15	MAP kinase interacting serine/threonine kinase 1	MKNK1

241741_at	2.15	cardiolipin synthase 1	CRLS1
235621_at	2.15		
235700_at	2.15		
207717_s_at	2.15	plakophilin 2	PKP2
221817_at	2.15	dolichyl pyrophosphate phosphatase 1	DOLPP1
242838_at	2.15	MAP6 domain containing 1	MAP6D1
242711_x_at	2.15	Fanconi anemia, complementation group M	FANCM
1553652_a_at	2.15	chromosome 18 open reading frame 54	C18orf54
219253_at	2.15	transmembrane protein 185B	TMEM185B
223401_at	2.15	chromosome 17 open reading frame 48	C17orf48
205739_x_at	2.15	zinc finger protein 588	ZNF588
207746_at	2.15	polymerase (DNA directed), theta	POLQ
205427_at	2.15	zinc finger protein 354A	ZNF354A
216594_x_at	2.15	aldo-keto reductase family 1, member C1 (dihydrodiol dehydrogenase 1; 20-alpha (3-alpha)-hydroxyster	AKR1C1
1554606_at	2.15	coiled-coil domain containing 100	CCDC100
1553625_at	2.15	family with sequence similarity 98, member B	FAM98B
226948_at	2.15	rhomboid domain containing 1	RHBDD1
205386_s_at	2.15	Mdm2, transformed 3T3 cell double minute 2, p53 binding protein (mouse)	MDM2
239677_at	2.15		
203712_at	2.15	KIAA0020	KIAA0020
202825_at	2.14	solute carrier family 25 (mitochondrial carrier; adenine nucleotide translocator), member 4	SLC25A4
223337_at	2.14	serologically defined colon cancer antigen 10	SDCCAG10
226558_at	2.14		
1569652_at	2.14	myeloid/lymphoid or mixed-lineage leukemia (trithorax homolog, Drosophila); translocated to, 3	MLLT3
1559161_at	2.14		
227805_at	2.14		
1557826_at	2.14		
221821_s_at	2.14	chromosome 12 open reading frame 41	C12orf41
230151_at	2.14	chromosome 13 open reading frame 1	C13orf1
213800_at	2.14	complement factor H	CFH
222879_s_at	2.14	polymerase (DNA directed), eta	POLH
1570578_at	2.14		
227215_at	2.14	golgi associated PDZ and coiled-coil motif containing	GOPC
228974_at	2.14		
206357_at	2.14	optic atrophy 3 (autosomal recessive, with chorea and spastic paraplegia)	OPA3
206353_at	2.14	cytochrome c oxidase subunit VIa polypeptide 2	COX6A2
1556744_a_at	2.14		
225161_at	2.14	G elongation factor, mitochondrial 1	GFM1
219531_at	2.14	centrosomal protein 72kDa	CEP72
233776_at	2.14	protein tyrosine phosphatase, receptor type, A	PTPRA
204971_at	2.13	cystatin A (stefin A)	CSTA
213695_at	2.13	paraoxonase 3	PON3
242107_x_at	2.13		
220081_x_at	2.13	hydroxysteroid (17-beta) dehydrogenase 7	HSD17B7
236198_at	2.13		
1568620_at	2.13	cysteine sulfinic acid decarboxylase	CSAD

216555_at	2.13	chromosome 22 open reading frame 30	C22orf30
235717_at	2.13	zinc finger protein 229	ZNF229
230652_at	2.13	v-raf murine sarcoma 3611 viral oncogene homolog	ARAF
207480_s_at	2.13	Meis1, myeloid ecotropic viral integration site 1 homolog 2 (mouse)	MEIS2
228990_at	2.13	chromosome 1 open reading frame 79	C1orf79
208896_at	2.13	DEAD (Asp-Glu-Ala-Asp) box polypeptide 18	DDX18
1553708_at	2.13		
232964_at	2.13	Williams Beuren syndrome chromosome region 19	WBSCR19
214943_s_at	2.13	RNA binding motif protein 34	RBM34
228515_at	2.13		
1569478_s_at	2.13		
1555491_a_at	2.13		
225521_at	2.13	anaphase promoting complex subunit 7	ANAPC7
221248_s_at	2.13	Wolf-Hirschhorn syndrome candidate 1-like 1	WHSC1L1
215170_s_at	2.13	centrosomal protein 152kDa	CEP152
1555439_at	2.13	general transcription factor IIIC, polypeptide 3, 102kDa	GTF3C3
221308_at	2.13	fibroblast growth factor receptor substrate 2	FRS2
201735_s_at	2.13	chloride channel 3	CLCN3
211767_at	2.13	GINS complex subunit 4 (Sld5 homolog)	GINS4
200832_s_at	2.13	stearoyl-CoA desaturase (delta-9-desaturase)	SCD
205052_at	2.13	AU RNA binding protein/enoyl-Coenzyme A hydratase	AUH
238481_at	2.13	matrix Gla protein	MGP
214440_at	2.13	N-acetyltransferase 1 (arylamine N-acetyltransferase)	NAT1
235660_at	2.13		
222851_at	2.13	zinc finger protein 654	ZNF654
1554636_at	2.13		
219306_at	2.13	kinesin family member 15	KIF15
1555793_a_at	2.13	zinc finger protein 545	ZNF545
213596_at	2.13	caspase 4, apoptosis-related cysteine peptidase	CASP4
244401_at	2.13	chromosome 6 open reading frame 152	C6orf152
219662_at	2.12	chromosome 2 open reading frame 49	C2orf49
210334_x_at	2.12	baculoviral IAP repeat-containing 5 (survivin)	BIRC5
238937_at	2.12	zinc finger protein 420	ZNF420
203930_s_at	2.12	microtubule-associated protein tau	MAPT
228328_at	2.12	kelch-like 28 (Drosophila)	KLHL28
214816_x_at	2.12	chromosome 19 open reading frame 40	C19orf40
226000_at	2.12	CTTNBP2 N-terminal like	CTTNBP2NL
1555135_at	2.12		
243790_at	2.12	zinc finger protein 585A	ZNF585A
1553111_a_at	2.12	kelch repeat and BTB (POZ) domain containing 6	KBTBD6
242719_at	2.12		
227418_at	2.12	KIAA1826	KIAA1826
228095_at	2.12	PHD finger protein 14	PHF14
226510_at	2.12	HEAT repeat containing 5A	HEATR5A
233020_at	2.12		
224370_s_at	2.12	calcyphosine 2	CAPS2
1552794_a_at	2.12	zinc finger protein 547	ZNF547

233564_s_at	2.12	cytidine and dCMP deaminase domain containing 1#calcium binding protein 39-like	CDADC1#CAB 39L
1554409_at	2.12	Williams-Beuren syndrome chromosome region 16	WBSCR16
214364_at	2.12	MTERF domain containing 2	MTERFD2
227547_at	2.12		
230165_at	2.12	shugoshin-like 2 (S. pombe)	SGOL2
1564802_at	2.12		
244828_x_at	2.12		
1555874_x_at	2.12		
233190_at	2.12		
219258_at	2.12	TIMELESS interacting protein	TIPIN
1554067_at	2.11		
1552628_a_at	2.11	HERPUD family member 2	HERPUD2
1555495_a_at	2.11	serologically defined colon cancer antigen 10	SDCCAG10
243476_at	2.11		
222748_s_at	2.11	thioredoxin-like 4B	TXNL4B
223846_at	2.11	5-azacytidine induced 2	AZI2
1569792_a_at	2.11		
207697_x_at	2.11	leukocyte immunoglobulin-like receptor, subfamily B (with TM and ITIM domains), member 2	LILRB2
220206_at	2.11	zinc finger, MYM-type 1	ZMYM1
211374_x_at	2.11		
221210_s_at	2.11	N-acetylneuraminate pyruvate lyase (dihydrodipicolinate synthase)	NPL
221158_at	2.11	chromosome 21 open reading frame 66	C21orf66
209180_at	2.11	Rab geranylgeranyltransferase, beta subunit	RABGGTB
201828_x_at	2.11	family with sequence similarity 127, member A	FAM127A
1559044_at	2.11	exosome component 1	EXOSC1
1555487_a_at	2.11	ARP3 actin-related protein 3 homolog B (yeast)	ACTR3B
241745_at	2.11		
224447_s_at	2.11	chromosome 17 open reading frame 37	C17orf37
232826_at	2.11		
209531_at	2.11	glutathione transferase zeta 1 (maleylacetoacetate isomerase)	GSTZ1
212812_at	2.11		
227967_at	2.11	tubulin, gamma complex associated protein 5	TUBGCP5
216459_x_at	2.11		
222498_at	2.11	5-azacytidine induced 2	AZI2
229603_at	2.11	Bardet-Biedl syndrome 12	BBS12
209714_s_at	2.11	cyclin-dependent kinase inhibitor 3 (CDK2-associated dual specificity phosphatase)	CDKN3
238704_at	2.11		
235125_x_at	2.11		
209555_s_at	2.11	CD36 molecule (thrombospondin receptor)	CD36
241757_x_at	2.11	dynein, cytoplasmic 2, light intermediate chain 1	DYNC2LI1
240996_at	2.11		
210465_s_at	2.11	small nuclear RNA activating complex, polypeptide 3, 50kDa	SNAPC3
232053_x_at	2.11	rhomboid domain containing 2	RHBDD2
223180_s_at	2.10	chromosome 18 open reading frame 55	C18orf55
241527_at	2.10		
1556789_a_at	2.10		

239646_at	2.10		
1562440_at	2.10	mitogen-activated protein kinase kinase kinase 13	MAP3K13
1557174_a_at	2.10		
206077_at	2.10	Kell blood group, metallo-endopeptidase	KEL
237651_x_at	2.10		
229672_at	2.10	chromosome 20 open reading frame 44	C20orf44
243565_at	2.10		
223890_at	2.10		
221182_at	2.10	chromosome 1 open reading frame 129	C1orf129
235360_at	2.10		
206295_at	2.10	interleukin 18 (interferon-gamma-inducing factor)	IL18
243519_at	2.10	ubiquitin protein ligase E3C	UBE3C
1554234_at	2.10	katanin p60 subunit A-like 2	KATNAL2
1552988_at	2.10	chromosome 11 open reading frame 65	C11orf65
226349_at	2.10	chromosome 12 open reading frame 45	C12orf45
241731_x_at	2.10	zinc finger protein 440	ZNF440
206022_at	2.10	Norrie disease (pseudoglioma)	NDP
219667_s_at	2.10	B-cell scaffold protein with ankyrin repeats 1	BANK1
223997_at	2.10	folliculin interacting protein 1	FNIP1
227741_at	2.10	protein tyrosine phosphatase-like (proline instead of catalytic arginine), member b	PTPLB
220295_x_at	2.10	DEP domain containing 1	DEPDC1
202007_at	2.10	nidogen 1	NID1
205135_s_at	2.10	nuclear fragile X mental retardation protein interacting protein 1 pseudogene	NUFIP1P
234929_s_at	2.10	spermatogenesis associated 7	SPATA7
222227_at	2.10	zinc finger protein 236	ZNF236
219603_s_at	2.10	zinc finger protein 226	ZNF226
215512_at	2.09	membrane-associated ring finger (C3HC4) 6	6-Mar
221586_s_at	2.09	E2F transcription factor 5, p130-binding	E2F5
238821_at	2.09	cleavage stimulation factor, 3' pre-RNA, subunit 2, 64kDa	CSTF2
227170_at	2.09		
1555074_a_at	2.09	potassium voltage-gated channel, subfamily H (eag-related), member 5	KCNH5
211743_s_at	2.09	proteoglycan 2, bone marrow (natural killer cell activator, eosinophil granule major basic protein)	PRG2
219288_at	2.09	chromosome 3 open reading frame 14	C3orf14
235165_at	2.09	par-6 partitioning defective 6 homolog beta (C. elegans)	PARD6B
228697_at	2.09	histidine triad nucleotide binding protein 3	HINT3
1553561_at	2.09	taste receptor, type 2, member 50	TAS2R50
237493_at	2.09	interleukin 22 receptor, alpha 2	IL22RA2
1559214_at	2.09		
241689_at	2.09		
65585_at	2.09	family with sequence similarity 86, member C	FAM86C
226533_at	2.09	histidine triad nucleotide binding protein 3	HINT3
244797_at	2.09		
222766_at	2.09	polymerase (RNA) III (DNA directed) polypeptide K, 12.3 kDa	POLR3K
1557357_at	2.09		

222938_x_at	2.09	ectonucleotide pyrophosphatase/phosphodiesterase 3	ENPP3
212882_at	2.09	kelch-like 18 (Drosophila)	KLHL18
1554665_at	2.09	zinc finger protein 587	ZNF587
204834_at	2.09	fibrinogen-like 2	FGL2
225371_at	2.09	GLE1 RNA export mediator-like (yeast)	GLE1L
207606_s_at	2.09	Rho GTPase activating protein 12	ARHGAP12
1570243_at	2.09		
220606_s_at	2.09	chromosome 17 open reading frame 48	C17orf48
217612_at	2.09	translocase of inner mitochondrial membrane 50 homolog (S. cerevisiae)	TIMM50
221606_s_at	2.08	nucleosomal binding protein 1	NSBP1
208268_at	2.08	ADAM metallopeptidase domain 28	ADAM28
243631_at	2.08		
241859_at	2.08		
210395_x_at	2.08	myosin, light chain 4, alkali; atrial, embryonic	MYL4
225046_at	2.08		
224860_at	2.08	chromosome 9 open reading frame 123	C9orf123
221591_s_at	2.08	family with sequence similarity 64, member A	FAM64A
1555351_s_at	2.08	periphilin 1	PPHLN1
209279_s_at	2.08	NAD(P) dependent steroid dehydrogenase-like	NSDHL
238548_at	2.08		
244043_at	2.08	transcription factor Dp-2 (E2F dimerization partner 2)	TFDP2
1554341_a_at	2.08		
241262_at	2.08		
235829_at	2.08		
202823_at	2.08	transcription elongation factor B (SIII), polypeptide 1 (15kDa, elongin C)	TCEB1
229888_at	2.08	chromosome 12 open reading frame 60	C12orf60
214670_at	2.08	zinc finger with KRAB and SCAN domains 1	ZKSCAN1
212674_s_at	2.08	DEAH (Asp-Glu-Ala-His) box polypeptide 30	DHX30
238553_at	2.08	poly (ADP-ribose) glycohydrolase	PARG
203403_s_at	2.08	ring finger protein (C3H2C3 type) 6	RNF6
242564_at	2.08		
1552682_a_at	2.08	cancer susceptibility candidate 5	CASC5
1560007_at	2.08		
227482_at	2.08	aarF domain containing kinase 1	ADCK1
205085_at	2.08	origin recognition complex, subunit 1-like (yeast)	ORC1L
215093_at	2.08	melanoma antigen family A, 2#null#melanoma antigen family A, 2B	MAGEA2#null#MAGEA2B
227686_at	2.08	oxidoreductase NAD-binding domain containing 1	OXNAD1
235550_at	2.08	microtubule-associated protein 9	MAP9
234268_at	2.08	solute carrier family 2 (facilitated glucose transporter), member 13	SLC2A13
230129_at	2.08	chromosome 10 open reading frame 89	C10orf89
214751_at	2.08	zinc finger protein 468	ZNF468
212530_at	2.08	NIMA (never in mitosis gene a)-related kinase 7	NEK7
218866_s_at	2.08	polymerase (RNA) III (DNA directed) polypeptide K, 12.3 kDa	POLR3K
230721_at	2.08	chromosome 16 open reading frame 52	C16orf52
1562289_at	2.08		
1555745_a_at	2.08	lysozyme (renal amyloidosis)	LYZ

211686_s_at	2.07	RNA binding motif protein 13	RBM13
216538_at	2.07		
1553220_at	2.07	amyotrophic lateral sclerosis 2 (juvenile) chromosome region, candidate 13	ALS2CR13
240896_at	2.07		
200666_s_at	2.07	DnaJ (Hsp40) homolog, subfamily B, member 1	DNAJB1
1566780_at	2.07		
237081_at	2.07		
233193_x_at	2.07	integrator complex subunit 4	INTS4
223289_s_at	2.07	ubiquitin specific peptidase 38	USP38
227813_at	2.07	THAP domain containing 6	THAP6
229621_x_at	2.07	early B-cell factor 3	EBF3
230795_at	2.07		
244352_at	2.07		
1554768_a_at	2.07	MAD2 mitotic arrest deficient-like 1 (yeast)	MAD2L1
215527_at	2.07	KH domain containing, RNA binding, signal transduction associated 2	KHDRBS2
229282_at	2.07	GATA binding protein 6	GATA6
235901_at	2.07		
208741_at	2.07	Sin3A-associated protein, 18kDa	SAP18
238841_at	2.07	protein tyrosine phosphatase domain containing 1	PTPDC1
216479_at	2.07	ribosomal protein L21 pseudogene 2	RPL21P2
212229_s_at	2.07	F-box protein 21	FBXO21
211086_x_at	2.07	NIMA (never in mitosis gene a)-related kinase 1	NEK1
205857_at	2.07	solute carrier family 18 (vesicular monoamine), member 2	SLC18A2
203764_at	2.07	discs, large homolog 7 (Drosophila)	DLG7
222995_s_at	2.06	rhomboid domain containing 2	RHBDD2
213647_at	2.06	DNA2 DNA replication helicase 2-like (yeast)	DNA2L
1562607_at	2.06		
242818_x_at	2.06		
220278_at	2.06	jumonji domain containing 2D	JMJD2D
238982_at	2.06	density-regulated protein	DENR
236133_x_at	2.06	zinc finger protein 254	ZNF254
241156_at	2.06		
226610_at	2.06	proline rich 6	PRR6
228093_at	2.06	zinc finger protein 599	ZNF599
239725_at	2.06		
224484_s_at	2.06	breast cancer metastasis-suppressor 1-like	BRMS1L
214314_s_at	2.06	eukaryotic translation initiation factor 5B	EIF5B
1562428_at	2.06		
1559059_s_at	2.06	zinc finger protein 611	ZNF611
229189_s_at	2.06		
227624_at	2.06	KIAA1546	KIAA1546
219023_at	2.06	chromosome 4 open reading frame 16	C4orf16
238913_at	2.06		
1552660_a_at	2.06	chromosome 5 open reading frame 22	C5orf22
222604_at	2.06	general transcription factor IIIC, polypeptide 3, 102kDa	GTF3C3
211074_at	2.06	folate receptor 1 (adult)	FOLR1
202726_at	2.06	ligase I, DNA, ATP-dependent	LIG1
231855_at	2.06	KIAA1524	KIAA1524

216716_at	2.06	ABO blood group (transferase A, alpha 1-3-N-acetylgalactosaminyltransferase; transferase B, alpha 1-	ABO
211371_at	2.06	mitogen-activated protein kinase kinase 5	MAP2K5
1552928_s_at	2.06	mitogen-activated protein kinase kinase kinase 7 interacting protein 3	MAP3K7IP3
203277_at	2.06	DNA fragmentation factor, 45kDa, alpha polypeptide	DFFA
208895_s_at	2.06	DEAD (Asp-Glu-Ala-Asp) box polypeptide 18	DDX18
202539_s_at	2.06	3-hydroxy-3-methylglutaryl-Coenzyme A reductase	HMGCR
241252_at	2.06	establishment of cohesion 1 homolog 2 (S. cerevisiae)	ESCO2
213199_at	2.06		
204566_at	2.06	protein phosphatase 1D magnesium-dependent, delta isoform	PPM1D
243500_at	2.06	CAS1 domain containing 1	CASD1
204848_x_at	2.06	hemoglobin, gamma A	HBG1
209770_at	2.06	butyrophilin, subfamily 3, member A1	BTN3A1
205667_at	2.06	Werner syndrome	WRN
201059_at	2.06	cortactin	CTTN
205822_s_at	2.06	3-hydroxy-3-methylglutaryl-Coenzyme A synthase 1 (soluble)	HMGCS1
1561918_at	2.06		
203577_at	2.06	general transcription factor IIH, polypeptide 4, 52kDa	GTF2H4
215983_s_at	2.06	UBX domain containing 6	UBXD6
228835_at	2.06		
214507_s_at	2.06	exosome component 2	EXOSC2
226402_at	2.05	cytochrome P450, family 2, subfamily U, polypeptide 1	CYP2U1
229143_at	2.05	CCR4-NOT transcription complex, subunit 3	CNOT3
214472_at	2.05	histone cluster 1, H3d	HIST1H3D
218264_at	2.05	BRCA2 and CDKN1A interacting protein	BCCIP
1563130_a_at	2.05		
220900_at	2.05		
1569058_at	2.05	general transcription factor IIIC, polypeptide 3, 102kDa	GTF3C3
218111_s_at	2.05	cytidine monophosphate N-acetylneuraminic acid synthetase	CMAS
227201_at	2.05		
204171_at	2.05	ribosomal protein S6 kinase, 70kDa, polypeptide 1	RPS6KB1
232323_s_at	2.05	tetratricopeptide repeat domain 17	TTC17
1553901_x_at	2.05	zinc finger protein 486	ZNF486
242242_at	2.05	ubiquitin specific peptidase 6 (Tre-2 oncogene)	USP6
205133_s_at	2.05	heat shock 10kDa protein 1 (chaperonin 10)	HSPE1
210896_s_at	2.05	aspartate beta-hydroxylase	ASPH
205003_at	2.05	dedicator of cytokinesis 4	DOCK4
204732_s_at	2.05	tripartite motif-containing 23	TRIM23
1562698_x_at	2.05		
228646_at	2.05	protein phosphatase 1, regulatory (inhibitor) subunit 1C	PPP1R1C

202093_s_at	2.05	Paf1, RNA polymerase II associated factor, homolog (S. cerevisiae)	PAF1
227922_x_at	2.05		
225737_s_at	2.05	F-box protein 22	FBXO22
211548_s_at	2.05	hydroxyprostaglandin dehydrogenase 15-(NAD)	HPGD
242362_at	2.05	cullin 3	CUL3
215483_at	2.05	A kinase (PRKA) anchor protein (yotiao) 9	AKAP9
218932_at	2.05	chromosome 1 open reading frame 181	C1orf181
1559523_at	2.05		
225158_at	2.05	G elongation factor, mitochondrial 1	GFM1
232023_at	2.05	transmembrane protein 67	TMEM67
202557_at	2.05	stress 70 protein chaperone, microsome-associated, 60kDa	STCH
224467_s_at	2.05	programmed cell death 2-like	PDCD2L
218642_s_at	2.05	coiled-coil-helix-coiled-coil-helix domain containing 7	CHCHD7
226943_at	2.04		
220020_at	2.04	X-prolyl aminopeptidase (aminopeptidase P) 3, putative	XPNPEP3
232044_at	2.04	retinoblastoma binding protein 6	RBBP6
226549_at	2.04	SH3-binding domain kinase 1	SBK1
204504_s_at	2.04	HIRA interacting protein 3	HIRIP3
230443_at	2.04	NHP2 non-histone chromosome protein 2-like 1 (S. cerevisiae)	NHP2L1
212216_at	2.04	prolyl endopeptidase-like	PREPL
218897_at	2.04	transmembrane protein 177	TMEM177
1556545_at	2.04		
204649_at	2.04	trophinin associated protein (tastin)	TROAP
242154_x_at	2.04	peptidylprolyl isomerase (cyclophilin)-like 5	PPIL5
224799_at	2.04	Nedd4 family interacting protein 2	NDFIP2
233746_x_at	2.04		
205741_s_at	2.04	dystrobrevin, alpha	DTNA
226060_at	2.04	RFT1 homolog (S. cerevisiae)	RFT1
228859_at	2.04		
222474_s_at	2.04	translocase of outer mitochondrial membrane 22 homolog (yeast)	TOMM22
211574_s_at	2.04	CD46 molecule, complement regulatory protein	CD46
1560760_s_at	2.04		
208478_s_at	2.04	BCL2-associated X protein	BAX
234902_s_at	2.04	zinc finger protein 134#zinc finger protein 211#zinc finger protein 416#zinc finger protein 550#zinc finger protein 549#zinc finger protein interacting with K protein 1 homolog (mouse)#zinc finger protein 530	ZNF134#ZNF211#ZNF416#ZNF550#ZNF549#ZIK1#ZNF530
237803_x_at	2.04	zinc finger and BTB domain containing 20	ZBTB20
242577_at	2.04		
244225_x_at	2.04	lamin A/C	LMNA
227414_at	2.04	rhomboid domain containing 1	RHBDD1
229516_at	2.04		
206494_s_at	2.04	integrin, alpha 2b (platelet glycoprotein IIb of IIb/IIIa complex, antigen CD41)	ITGA2B
238822_at	2.04		

221227_x_at	2.04	coenzyme Q3 homolog, methyltransferase (S. cerevisiae)	COQ3
212384_at	2.04	HLA-B associated transcript 1	BAT1
209917_s_at	2.04	TP53 activated protein 1	TP53AP1
217892_s_at	2.03	LIM domain and actin binding 1	LIMA1
239253_at	2.03		
241379_at	2.03	prokineticin receptor 1	PROKR1
236672_at	2.03		
212800_at	2.03	syntaxin 6	STX6
232014_at	2.03	zinc finger protein 30	ZNF30
211792_s_at	2.03	cyclin-dependent kinase inhibitor 2C (p18, inhibits CDK4)	CDKN2C
220397_at	2.03	Mdm4, transformed 3T3 cell double minute 1, p53 binding protein (mouse)	MDM1
228378_at	2.03	chromosome 12 open reading frame 29	C12orf29
213220_at	2.03		
201734_at	2.03		
223559_s_at	2.03	chromosome 9 open reading frame 80	C9orf80
227857_at	2.03	chromosome 20 open reading frame 3	C20orf3
235610_at	2.03	alkB, alkylation repair homolog 8 (E. coli)	ALKBH8
213392_at	2.03	IQ motif containing K	IQCK
203565_s_at	2.03	menage a trois homolog 1, cyclin H assembly factor (Xenopus laevis)	MNAT1
1558002_at	2.03	serine/threonine kinase receptor associated protein	STRAP
1557472_a_at	2.03		
232296_s_at	2.03	G elongation factor, mitochondrial 1	GFM1
211781_x_at	2.03		
221536_s_at	2.03	large subunit GTPase 1 homolog (S. cerevisiae)	LSG1
220488_s_at	2.03	breast carcinoma amplified sequence 3	BCAS3
226608_at	2.03		
235432_at	2.03	nephronophthisis 3 (adolescent)	NPHP3
219596_at	2.03	THAP domain containing 10	THAP10
206172_at	2.03	interleukin 13 receptor, alpha 2	IL13RA2
223539_s_at	2.03	small EDRK-rich factor 1A (telomeric)	SERF1A
206114_at	2.03	EPH receptor A4	EPHA4
215242_at	2.03	phosphatidylinositol glycan anchor biosynthesis, class C	PIGC
242669_at	2.03	ubiquitin-fold modifier 1	UFM1
209900_s_at	2.03	solute carrier family 16, member 1 (monocarboxylic acid transporter 1)	SLC16A1
234491_s_at	2.03	salvador homolog 1 (Drosophila)	SAV1
1557410_at	2.03		
229793_at	2.03		
208813_at	2.03	glutamic-oxaloacetic transaminase 1, soluble (aspartate aminotransferase 1)	GOT1
212225_at	2.03	eukaryotic translation initiation factor 1	EIF1
242673_at	2.03	ubiquitin protein ligase E3C	UBE3C
209365_s_at	2.02	extracellular matrix protein 1	ECM1
230777_s_at	2.02	PR domain containing 15	PRDM15
214484_s_at	2.02	opioid receptor, sigma 1	OPRS1
230355_at	2.02		

227428_at	2.02	GA binding protein transcription factor, alpha subunit 60kDa	GABPA
243937_x_at	2.02	centaurin, gamma-like family, member 2	CTGLF2
231173_at	2.02		
229907_at	2.02		
228040_at	2.02		
219443_at	2.02	taspase, threonine aspartase, 1	TASP1
215172_at	2.02	protein tyrosine phosphatase, non-receptor type 20B#protein tyrosine phosphatase, non-receptor type 20A#protein tyrosine phosphatase, non-receptor type 20A	PTPN20B#PTPN20A#PTPN20A
227207_x_at	2.02	zinc finger protein 213	ZNF213
238953_at	2.02		
1560774_at	2.02		
1554671_a_at	2.02	serine/arginine repetitive matrix 2	SRRM2
244551_at	2.02		
238004_at	2.02	piggyBac transposable element derived 2	PGBD2
225917_at	2.02		
64432_at	2.02	chromosome 12 open reading frame 47	C12orf47
211114_x_at	2.02	survival of motor neuron protein interacting protein 1	SIP1
1556690_s_at	2.02	myeloid zinc finger 1	MZF1
231376_at	2.02	uridine phosphorylase 2	UPP2
217665_at	2.02		
242585_at	2.02		
1552634_a_at	2.02	zinc finger protein 101	ZNF101
1563840_at	2.02	elongation factor Tu GTP binding domain containing 1	EFTUD1
232028_at	2.02	zinc finger protein 678	ZNF678
201323_at	2.02	EBNA1 binding protein 2	EBNA1BP2
237051_at	2.02	solute carrier family 30 (zinc transporter), member 9	SLC30A9
209366_x_at	2.02	cytochrome b5 type A (microsomal)	CYB5A
223069_s_at	2.01	echinoderm microtubule associated protein like 4	EML4
228084_at	2.01		
229835_s_at	2.01	slowmo homolog 2 (Drosophila)	SLMO2
1556284_at	2.01	pyrophosphatase (inorganic) 2	PPA2
206446_s_at	2.01	elastase 1, pancreatic	ELA1
203567_s_at	2.01	tripartite motif-containing 38	TRIM38
1554885_a_at	2.01	primase, polypeptide 2A, 58kDa	PRIM2A
238866_at	2.01		
240290_at	2.01		
235203_at	2.01		
1564467_at	2.01		
1554364_at	2.01	protein phosphatase 2, regulatory subunit B', gamma isoform	PPP2R5C
211423_s_at	2.01	sterol-C5-desaturase (ERG3 delta-5-desaturase homolog, S. cerevisiae)-like	SC5DL
215310_at	2.01	adenomatosis polyposis coli	APC
223275_at	2.01	protein arginine methyltransferase 6	PRMT6
231285_at	2.01	STT3, subunit of the oligosaccharyltransferase complex, homolog B (S. cerevisiae)	STT3B
1562336_at	2.01	cancer susceptibility candidate 2	CASC2

1554883_a_at	2.01	excision repair cross-complementing rodent repair deficiency, complementation group 8	ERCC8
218397_at	2.01	Fanconi anemia, complementation group L	FANCL
218838_s_at	2.01	tetratricopeptide repeat domain 31	TTC31
207622_s_at	2.01	ATP-binding cassette, sub-family F (GCN20), member 2	ABCF2
235953_at	2.01	zinc finger protein 610	ZNF610
230596_at	2.01		
242330_at	2.01		
52285_f_at	2.01	centrosomal protein 76kDa	CEP76
212596_s_at	2.01	high-mobility group protein 2-like 1	HMG2L1
1552519_at	2.01	activin A receptor, type IC	ACVR1C
206650_at	2.01	IQ motif containing C	IQCC
223298_s_at	2.01	5'-nucleotidase, cytosolic III	NT5C3
237005_at	2.01		
223898_at	2.01	zinc finger protein 670	ZNF670
1557363_a_at	2.01	pleckstrin homology domain interacting protein	PHIP
220077_at	2.01	coiled-coil domain containing 134	CCDC134
220123_at	2.01	solute carrier family 35, member F5	SLC35F5
225695_at	2.01	chromosome 2 open reading frame 18	C2orf18
203008_x_at	2.01	thioredoxin domain containing 9	TXNDC9
1554973_a_at	2.01	zinc finger and BTB domain containing 26	ZBTB26
234947_s_at	2.01	chromosome 10 open reading frame 84	C10orf84
222792_s_at	2.01	coiled-coil domain containing 59	CCDC59
232235_at	2.01	dermatan sulfate epimerase-like	DSEL
223766_at	2.01		
210568_s_at	2.01	RecQ protein-like (DNA helicase Q1-like)	RECQL
208079_s_at	2.01	aurora kinase A	AURKA
1557217_a_at	2.00	Fanconi anemia, complementation group B	FANCB
205218_at	2.00	polymerase (RNA) III (DNA directed) polypeptide F, 39 kDa	POLR3F
203039_s_at	2.00	NADH dehydrogenase (ubiquinone) Fe-S protein 1, 75kDa (NADH-coenzyme Q reductase)	NDUFS1
203846_at	2.00	tripartite motif-containing 32	TRIM32
223527_s_at	2.00	cytidine and dCMP deaminase domain containing 1#calcium binding protein 39-like	CDADC1#CAB39L
227072_at	2.00	rotatin	RTTN
1560204_at	2.00	5'-nucleotidase domain containing 4	NT5DC4
236999_at	2.00	peptidylprolyl isomerase domain and WD repeat containing 1	PPWD1
200862_at	2.00	24-dehydrocholesterol reductase	DHCR24
204389_at	2.00	monoamine oxidase A	MAOA
239452_at	2.00		
1558739_at	2.00		
1558651_at	2.00		
201516_at	2.00	spermidine synthase	SRM
1554145_a_at	2.00	coiled-coil domain containing 128	CCDC128
203899_s_at	2.00		
205901_at	2.00	prepronociceptin	PNOC
241454_at	2.00		
210188_at	2.00	GA binding protein transcription factor, alpha subunit 60kDa	GABPA
208025_s_at	0.50	high mobility group AT-hook 2	HMGA2

210733_at	0.50	translocation associated membrane protein 1	TRAM1
221073_s_at	0.50	nucleotide-binding oligomerization domain containing 1	NOD1
1559517_a_at	0.50	spire homolog 1 (Drosophila)	SPIRE1
209994_s_at	0.50	ATP-binding cassette, sub-family B (MDR/TAP), member 1	ABCB1
216862_s_at	0.50	mature T-cell proliferation 1	MTCP1
240540_at	0.50	sortilin-related VPS10 domain containing receptor 2	SORCS2
222139_at	0.50		
242058_at	0.50		
209306_s_at	0.50		
232370_at	0.50		
239585_at	0.50		
224761_at	0.50	guanine nucleotide binding protein (G protein), alpha 13	GNA13
227379_at	0.50	membrane bound O-acyltransferase domain containing 1	MBOAT1
243134_at	0.50	chromodomain helicase DNA binding protein 2	CHD2
238767_at	0.50		
231041_at	0.50	polymerase (RNA) I polypeptide E, 53kDa	POLR1E
224598_at	0.50	mannosyl (alpha-1,3-)-glycoprotein beta-1,4-N-acetylglucosaminyltransferase, isozyme B	MGAT4B
212547_at	0.50		
228299_at	0.50	potassium channel tetramerisation domain containing 20	KCTD20
222820_at	0.50	trinucleotide repeat containing 6C	TNRC6C
201203_s_at	0.50	ribosome binding protein 1 homolog 180kDa (dog)	RRBP1
201206_s_at	0.50	ribosome binding protein 1 homolog 180kDa (dog)	RRBP1
214787_at	0.50	DENN/MADD domain containing 4A	DENND4A
200906_s_at	0.50	palladin, cytoskeletal associated protein	PALLD
202552_s_at	0.50	cysteine rich transmembrane BMP regulator 1 (chordin-like)	CRIM1
212970_at	0.50		
223513_at	0.50	centromere protein J	CENPJ
230292_at	0.50		
239175_at	0.50		
229115_at	0.50	dynein, cytoplasmic 1, heavy chain 1	DYNC1H1
212106_at	0.50	UBX domain containing 8	UBXD8
1554239_s_at	0.50	zinc binding alcohol dehydrogenase, domain containing 2	ZADH2
218079_s_at	0.50	zinc finger protein 403	ZNF403
210706_s_at	0.50	ring finger protein 24	RNF24
215646_s_at	0.50	chondroitin sulfate proteoglycan 2 (versican)	CSPG2
212599_at	0.50	autism susceptibility candidate 2	AUTS2
239056_at	0.50	SEC22 vesicle trafficking protein homolog C (S. cerevisiae)	SEC22C
208873_s_at	0.50	receptor accessory protein 5	REEP5
229607_at	0.50		
217497_at	0.50	endothelial cell growth factor 1 (platelet-derived)	ECGF1
222981_s_at	0.49	RAB10, member RAS oncogene family	RAB10

1561144_at	0.49		
226865_at	0.49		
222119_s_at	0.49	F-box protein 11	FBXO11
226302_at	0.49	ATPase, Class I, type 8B, member 1	ATP8B1
200945_s_at	0.49	SEC31 homolog A (S. cerevisiae)	SEC31A
205429_s_at	0.49	membrane protein, palmitoylated 6 (MAGUK p55 subfamily member 6)	MPP6
225582_at	0.49	KIAA1754	KIAA1754
226121_at	0.49	dehydrogenase/reductase (SDR family) member 13	DHRS13
204538_x_at	0.49	nuclear pore complex interacting protein	NPIP
211137_s_at	0.49	ATPase, Ca++ transporting, type 2C, member 1	ATP2C1
203579_s_at	0.49	solute carrier family 7 (cationic amino acid transporter, y+ system), member 6	SLC7A6
228805_at	0.49	chromosome 5 open reading frame 25	C5orf25
210946_at	0.49	phosphatidic acid phosphatase type 2A	PPAP2A
244158_at	0.49		
238043_at	0.49		
227481_at	0.49	CNKSR family member 3	CNKSR3
238858_at	0.49		
227080_at	0.49	zinc finger protein 697	ZNF697
1559078_at	0.49	B-cell CLL/lymphoma 11A (zinc finger protein)	BCL11A
222473_s_at	0.49	erbb2 interacting protein	ERBB2IP
212976_at	0.49		
226099_at	0.49	elongation factor, RNA polymerase II, 2	ELL2
226819_at	0.49		
202117_at	0.49	Rho GTPase activating protein 1	ARHGAP1
227169_at	0.49	DnaJ (Hsp40) homolog, subfamily C, member 18	DNAJC18
243161_x_at	0.49	zinc finger protein 42 homolog (mouse)	ZFP42
243_g_at	0.49	microtubule-associated protein 4	MAP4
203989_x_at	0.49	coagulation factor II (thrombin) receptor	F2R
242457_at	0.49		
225856_at	0.49		
205601_s_at	0.49	homeobox B5	HOXB5
203156_at	0.49	A kinase (PRKA) anchor protein 11	AKAP11
225293_at	0.49	collagen, type XXVII, alpha 1	COL27A1
221501_x_at	0.49		
203416_at	0.49	CD53 molecule	CD53
221335_x_at	0.49		
242358_at	0.49		
1561767_at	0.49		
227280_s_at	0.49		
236094_at	0.49	transcription factor 7-like 2 (T-cell specific, HMG-box)	TCF7L2
34726_at	0.49	calcium channel, voltage-dependent, beta 3 subunit	CACNB3
241458_at	0.49		
204786_s_at	0.49	interferon (alpha, beta and omega) receptor 2	IFNAR2
201661_s_at	0.49	acyl-CoA synthetase long-chain family member 3	ACSL3
200629_at	0.49	tryptophanyl-tRNA synthetase	WARS
209941_at	0.49	receptor (TNFRSF)-interacting serine-threonine kinase 1	RIPK1
235339_at	0.49	SET domain, bifurcated 2	SETDB2

210276_s_at	0.49	TRIO and F-actin binding protein	TRIOBP
232909_s_at	0.49	bromodomain PHD finger transcription factor	BPTF
200796_s_at	0.49	myeloid cell leukemia sequence 1 (BCL2-related)	MCL1
201866_s_at	0.49	nuclear receptor subfamily 3, group C, member 1 (glucocorticoid receptor)	NR3C1
209878_s_at	0.49	v-rel reticuloendotheliosis viral oncogene homolog A, nuclear factor of kappa light polypeptide gene	RELA
227937_at	0.49		
1562719_at	0.49		
226395_at	0.49		
239682_at	0.49		
41577_at	0.49	protein phosphatase 1, regulatory (inhibitor) subunit 16B	PPP1R16B
235061_at	0.49	protein phosphatase 1K (PP2C domain containing)	PPM1K
204491_at	0.49	phosphodiesterase 4D, cAMP-specific (phosphodiesterase E3 dunce homolog, Drosophila)	PDE4D
200761_s_at	0.49	ADP-ribosylation-like factor 6 interacting protein 5	ARL6IP5
216216_at	0.49	slit homolog 3 (Drosophila)	SLIT3
214870_x_at	0.49	nuclear pore complex interacting protein	NPIP
212760_at	0.49	ubiquitin protein ligase E3 component n-recognin 2	UBR2
1561683_at	0.49		
212765_at	0.49	calmodulin regulated spectrin-associated protein 1-like 1	CAMSAP1L1
230183_at	0.49		
244804_at	0.49	sequestosome 1	SQSTM1
211842_s_at	0.49	solute carrier family 24 (sodium/potassium/calcium exchanger), member 1	SLC24A1
212696_s_at	0.49	ring finger protein 4	RNF4
217966_s_at	0.49	family with sequence similarity 129, member A	FAM129A
217941_s_at	0.49	erbb2 interacting protein	ERBB2IP
222395_s_at	0.49	ubiquitin-conjugating enzyme E2Z (putative)	UBE2Z
237195_at	0.49		
1570108_at	0.49		
212056_at	0.49	KIAA0182	KIAA0182
218704_at	0.49	ring finger protein 43	RNF43
225630_at	0.49		
212856_at	0.49		
227649_s_at	0.49	SLIT-ROBO Rho GTPase activating protein 2	SRGAP2
241963_at	0.49	zinc finger protein 704	ZNF704
1568609_s_at	0.49		
240960_at	0.49		
228415_at	0.49		
239170_at	0.49		
231869_at	0.49	zinc finger protein 451	ZNF451
1554153_a_at	0.49	PHD finger protein 21A	PHF21A
240824_at	0.49	oligonucleotide/oligosaccharide-binding fold containing 1	OBFC1
1564776_at	0.49	leukocyte receptor cluster (LRC) member 10	LENG10

202743_at	0.48	phosphoinositide-3-kinase, regulatory subunit 3 (p55, gamma)	PIK3R3
201054_at	0.48	heterogeneous nuclear ribonucleoprotein A0	HNRPA0
238797_at	0.48	tripartite motif-containing 11	TRIM11
232529_at	0.48	Sp3 transcription factor	SP3
214054_at	0.48	docking protein 2, 56kDa	DOK2
238480_at	0.48	chromosome 18 open reading frame 50	C18orf50
212665_at	0.48	TCDD-inducible poly(ADP-ribose) polymerase	TIPARP
1560189_at	0.48		
215084_s_at	0.48	deiodinase, iodothyronine, type I#chromosome 1 open reading frame 41#chromosome 1 open reading frame 41#Yip1 domain family, member 1#leucine rich repeat containing 42	DIO1#C1orf41#C1orf41#YIPF1#LRRC42
209515_s_at	0.48	RAB27A, member RAS oncogene family	RAB27A
214030_at	0.48		
238086_at	0.48		
218423_x_at	0.48	vacuolar protein sorting 54 homolog (S. cerevisiae)	VPS54
224788_at	0.48	ADP-ribosylation factor 6	ARF6
221081_s_at	0.48	DENN/MADD domain containing 2D	DENND2D
213610_s_at	0.48	kelch-like 23 (Drosophila)	KLHL23
224866_at	0.48	male sterility domain containing 2	MLSTD2
231873_at	0.48	bone morphogenetic protein receptor, type II (serine/threonine kinase)	BMPR2
218854_at	0.48	dermatan sulfate epimerase	DSE
205781_at	0.48	chromosome 16 open reading frame 7	C16orf7
222001_x_at	0.48		
217989_at	0.48	hydroxysteroid (17-beta) dehydrogenase 11	HSD17B11
238005_s_at	0.48		
225732_at	0.48		
228826_at	0.48		
224839_s_at	0.48	glutamic pyruvate transaminase (alanine aminotransferase) 2	GPT2
202761_s_at	0.48	spectrin repeat containing, nuclear envelope 2	SYNE2
214651_s_at	0.48	homeobox A9	HOXA9
227298_at	0.48		
221194_s_at	0.48		
208855_s_at	0.48	serine/threonine kinase 24 (STE20 homolog, yeast)	STK24
206839_at	0.48	chromosome 22 open reading frame 31	C22orf31
230219_at	0.48	nudE nuclear distribution gene E homolog 1 (A. nidulans)	NDE1
212762_s_at	0.48	transcription factor 7-like 2 (T-cell specific, HMG-box)	TCF7L2
241773_at	0.48		
202949_s_at	0.48	four and a half LIM domains 2	FHL2
229268_at	0.48	family with sequence similarity 105, member B	FAM105B
1552381_at	0.48		
222557_at	0.48	tumor necrosis factor receptor superfamily, member 6b, decoy#glucocorticoid modulatory element binding protein 2#stathmin-like 3#regulator of telomere elongation helicase 1	TNFRSF6B#GMEB2#STMN3#RTEL1
218459_at	0.48	torsin family 3, member A	TOR3A

235555_at	0.48		
227000_at	0.48	chromosome 7 open reading frame 41	C7orf41
208883_at	0.48	E3 ubiquitin protein ligase, HECT domain containing, 1	EDD1
224681_at	0.48	guanine nucleotide binding protein (G protein) alpha 12	GNA12
217783_s_at	0.48	yippee-like 5 (Drosophila)	YPEL5
235292_at	0.48		
227268_at	0.48		
1559942_at	0.48	MyoD family inhibitor domain containing	MDFIC
222142_at	0.48	cylindromatosis (turban tumor syndrome)	CYLD
213689_x_at	0.48	ribosomal protein L5	RPL5
218266_s_at	0.48	frequenin homolog (Drosophila)	FREQ
1557064_s_at	0.48	heparan-alpha-glucosaminide N-acetyltransferase	HGSNAT
201540_at	0.48	four and a half LIM domains 1	FHL1
218913_s_at	0.48	GEM interacting protein	GMIP
216614_at	0.48		
1556321_a_at	0.48		
240621_at	0.48	ataxia telangiectasia mutated (includes complementation groups A, C and D)	ATM
225814_at	0.48	5'-3' exoribonuclease 1	XRN1
1557347_at	0.48	microcephaly, primary autosomal recessive 1	MCPH1
243173_at	0.48	calcium binding protein 7	CABP7
204642_at	0.48	endothelial differentiation, sphingolipid G-protein-coupled receptor, 1	EDG1
232469_x_at	0.48	chromosome 1 open reading frame 191	C1orf191
203875_at	0.48	SWI/SNF related, matrix associated, actin dependent regulator of chromatin, subfamily a, member 1	SMARCA1
226272_at	0.48		
202181_at	0.48	KIAA0247	KIAA0247
244703_x_at	0.48	importin 9	IPO9
218614_at	0.48	chromosome 12 open reading frame 35	C12orf35
224560_at	0.48	TIMP metallopeptidase inhibitor 2	TIMP2
221858_at	0.48	TBC1 domain family, member 12	TBC1D12
239834_at	0.48		
227060_at	0.48	tumor necrosis factor receptor superfamily, member 19-like	TNFRSF19L
205105_at	0.48	mannosidase, alpha, class 2A, member 1	MAN2A1
203102_s_at	0.48	mannosyl (alpha-1,6-)-glycoprotein beta-1,2-N-acetylglucosaminyltransferase	MGAT2
203005_at	0.48	lymphotoxin beta receptor (TNFR superfamily, member 3)	LTBR
226607_at	0.48	chromosome 20 open reading frame 194	C20orf194
217376_at	0.48	signal-regulatory protein gamma	SIRPG
227554_at	0.48		
201643_x_at	0.48	jumonji domain containing 1B	JMJD1B
206593_s_at	0.48	surfeit 5	SURF5
237112_at	0.48	family with sequence similarity 102, member B	FAM102B
220015_at	0.48	castor zinc finger 1	CASZ1
230706_s_at	0.48	calcium/calmodulin-dependent protein kinase II inhibitor 2	CAMK2N2

227260_at	0.48		
226774_at	0.48	family with sequence similarity 120B	FAM120B
1564301_a_at	0.48	RPA interacting protein	RPAIN
238690_at	0.48		
207651_at	0.48	G protein-coupled receptor 171	GPR171
203297_s_at	0.48	jumonji, AT rich interactive domain 2	JARID2
214746_s_at	0.48	zinc finger protein 467	ZNF467
209676_at	0.48	tissue factor pathway inhibitor (lipoprotein-associated coagulation inhibitor)	TFPI
212146_at	0.48	pleckstrin homology domain containing, family M (with RUN domain) member 2	PLEKHM2
201976_s_at	0.48	myosin X	MYO10
205247_at	0.48	Notch homolog 4 (Drosophila)	NOTCH4
208132_x_at	0.48	HLA-B associated transcript 2	BAT2
214947_at	0.48		
218802_at	0.48	coiled-coil domain containing 109B	CCDC109B
205854_at	0.48	tubby like protein 3	TULP3
203016_s_at	0.47	synovial sarcoma, X breakpoint 2 interacting protein	SSX2IP
215046_at	0.47		
235349_at	0.47	family with sequence similarity 82, member A	FAM82A
233951_at	0.47		
213198_at	0.47	activin A receptor, type IB	ACVR1B
220137_at	0.47		
225135_at	0.47	SIN3 homolog A, transcription regulator (yeast)	SIN3A
224470_at	0.47	SEC22 vesicle trafficking protein homolog C (S. cerevisiae)	SEC22C
218399_s_at	0.47	cell division cycle associated 4	CDCA4
225391_at	0.47		
235938_at	0.47		
213351_s_at	0.47	transmembrane and coiled-coil domain family 1	TMCC1
204451_at	0.47	frizzled homolog 1 (Drosophila)	FZD1
224335_s_at	0.47	beta-site APP-cleaving enzyme 1	BACE1
210176_at	0.47	toll-like receptor 1	TLR1
239277_at	0.47		
224927_at	0.47	KIAA1949	KIAA1949
217196_s_at	0.47	calmodulin regulated spectrin-associated protein 1-like 1	CAMSAP1L1
238787_at	0.47		
229657_at	0.47	thyroid hormone receptor, beta (erythroblastic leukemia viral (v-erb-a) oncogene homolog 2, avian)	THRB
204970_s_at	0.47	v-maf musculoaponeurotic fibrosarcoma oncogene homolog G (avian)	MAFG
229027_at	0.47		
219208_at	0.47	F-box protein 11	FBXO11
1557261_at	0.47	WAS protein homology region 2 domain containing 1-like 1	WHDC1L1
211121_s_at	0.47	docking protein 1, 62kDa (downstream of tyrosine kinase 1)	DOK1
228618_at	0.47		
227384_s_at	0.47	family with sequence similarity 91, member A2	FAM91A2

230707_at	0.47	sortilin-related receptor, L(DLR class) A repeats-containing	SORL1
201302_at	0.47	annexin A4	ANXA4
238006_at	0.47		
219191_s_at	0.47	bridging integrator 2	BIN2
219629_at	0.47	family with sequence similarity 118, member A	FAM118A
213340_s_at	0.47		
237459_at	0.47	PCTAIRE protein kinase 2	PCTK2
215235_at	0.47	spectrin, alpha, non-erythrocytic 1 (alpha-fodrin)	SPTAN1
204137_at	0.47	G protein-coupled receptor 137B	GPR137B
225095_at	0.47	serine palmitoyltransferase, long chain base subunit 2	SPTLC2
225929_s_at	0.47	ring finger protein 213	RNF213
200940_s_at	0.47	arginine-glutamic acid dipeptide (RE) repeats	RERE
212873_at	0.47	histocompatibility (minor) HA-1	HMHA1
202208_s_at	0.47	ADP-ribosylation factor-like 4C	ARL4C
211075_s_at	0.47	CD47 molecule	CD47
209524_at	0.47		
236273_at	0.47	neuroblastoma breakpoint family, member 1	NBPF1
224106_at	0.47		
219501_at	0.47		
1561651_s_at	0.47	SCL/TAL1 interrupting locus	STIL
215087_at	0.47	chromosome 15 open reading frame 39	C15orf39
224956_at	0.47	nuclear fragile X mental retardation protein interacting protein 2	NUFIP2
236537_at	0.47		
209034_at	0.47	proline-rich nuclear receptor coactivator 1	PNRC1
206293_at	0.47	sulfotransferase family, cytosolic, 2A, dehydroepiandrosterone (DHEA)-preferring, member 1	SULT2A1
210878_s_at	0.47	jumonji domain containing 1B	JMJD1B
204391_x_at	0.47	tripartite motif-containing 24	TRIM24
220992_s_at	0.47	chromosome 1 open reading frame 25	C1orf25
201887_at	0.47	interleukin 13 receptor, alpha 1	IL13RA1
214844_s_at	0.47	docking protein 5	DOK5
1558679_at	0.47		
224318_s_at	0.47		
208071_s_at	0.47	leukocyte-associated immunoglobulin-like receptor 1	LAIR1
222566_at	0.47		
206432_at	0.47	hyaluronan synthase 2	HAS2
212573_at	0.47	endonuclease domain containing 1	ENDOD1
222494_at	0.47	checkpoint suppressor 1	CHES1
224697_at	0.47	WD repeat domain 22	WDR22
224910_at	0.47	calcium regulated heat stable protein 1, 24kDa	CARHSP1
49485_at	0.47	PR domain containing 4	PRDM4
220713_at	0.47		
225225_at	0.47	keratin associated protein 4-7	KRTAP4-7
213471_at	0.47	nephronophthisis 4	NPHP4
221737_at	0.47	guanine nucleotide binding protein (G protein) alpha 12	GNA12
217967_s_at	0.47	family with sequence similarity 129, member A	FAM129A
213301_x_at	0.47	tripartite motif-containing 24	TRIM24

229908_s_at	0.47		
230131_x_at	0.47	arylsulfatase D	ARSD
230328_at	0.47	RRN3 RNA polymerase I transcription factor homolog (S. cerevisiae)	RRN3
203332_s_at	0.47	inositol polyphosphate-5-phosphatase, 145kDa	INPP5D
226753_at	0.47	family with sequence similarity 76, member B	FAM76B
217875_s_at	0.47	transmembrane, prostate androgen induced RNA	TMEPAI
201037_at	0.47	phosphofructokinase, platelet	PFKP
225525_at	0.47		
209530_at	0.47	calcium channel, voltage-dependent, beta 3 subunit	CACNB3
213983_s_at	0.47		
202766_s_at	0.47	fibrillin 1	FBN1
235595_at	0.47	rho/rac guanine nucleotide exchange factor (GEF) 2	ARHGEF2
209361_s_at	0.47	poly(rC) binding protein 4	PCBP4
226032_at	0.47	caspase 2, apoptosis-related cysteine peptidase (neural precursor cell expressed, developmentally do	CASP2
235948_at	0.47	family with sequence similarity 80, member A	FAM80A
229713_at	0.47		
204474_at	0.47	zinc finger protein 142	ZNF142
1558837_a_at	0.47		
213792_s_at	0.47		
224045_x_at	0.47	chromosome 18 open reading frame 2	C18orf2
201888_s_at	0.47	interleukin 13 receptor, alpha 1	IL13RA1
205684_s_at	0.47	DENN/MADD domain containing 4C	DENND4C
238484_s_at	0.47		
231109_at	0.47	CUG triplet repeat, RNA binding protein 2	CUGBP2
213168_at	0.47	Sp3 transcription factor	SP3
200047_s_at	0.46	YY1 transcription factor	YY1
222482_at	0.46		
219147_s_at	0.46	chromosome 9 open reading frame 95	C9orf95
206972_s_at	0.46	G protein-coupled receptor 161	GPR161
232020_at	0.46	SMAD specific E3 ubiquitin protein ligase 2	SMURF2
219634_at	0.46	carbohydrate (chondroitin 4) sulfotransferase 11	CHST11
203912_s_at	0.46	deoxyribonuclease I-like 1	DNASE1L1
218198_at	0.46	DEAH (Asp-Glu-Ala-His) box polypeptide 32	DHX32
205567_at	0.46	carbohydrate (keratan sulfate Gal-6) sulfotransferase 1	CHST1
225820_at	0.46	PHD finger protein 17	PHF17
1562741_at	0.46	UBX domain containing 2	UBXD2
212200_at	0.46	KIAA0692	KIAA0692
218684_at	0.46	leucine rich repeat containing 8 family, member D	LRRC8D
226264_at	0.46	sushi domain containing 1	SUSD1
229204_at	0.46	heterochromatin protein 1, binding protein 3	HP1BP3
1557480_a_at	0.46	dysferlin interacting protein 1 (toonin)	DYSFIP1
209150_s_at	0.46	transmembrane 9 superfamily member 1	TM9SF1
1558949_at	0.46		
240458_at	0.46	inositol 1,4,5-triphosphate receptor, type 2	ITPR2
227804_at	0.46	TLC domain containing 1	TLCD1
227256_at	0.46	ubiquitin specific peptidase 31	USP31
212838_at	0.46	dynamin binding protein	DNMBP

1559401_a_at	0.46		
229539_at	0.46		
221413_at	0.46	potassium voltage-gated channel, shaker-related subfamily, beta member 3	KCNAB3
242034_at	0.46	F-box and leucine-rich repeat protein 17	FBXL17
203077_s_at	0.46	SMAD family member 2	SMAD2
205330_at	0.46	meningioma (disrupted in balanced translocation) 1	MN1
228487_s_at	0.46	ras responsive element binding protein 1	RREB1
209514_s_at	0.46	RAB27A, member RAS oncogene family	RAB27A
219437_s_at	0.46	ankyrin repeat domain 11	ANKRD11
212435_at	0.46	tripartite motif-containing 33	TRIM33
228124_at	0.46	abhydrolase domain containing 12	ABHD12
211026_s_at	0.46	monoglyceride lipase	MGLL
236191_at	0.46	CD38 molecule	CD38
226117_at	0.46		
209098_s_at	0.46	jagged 1 (Alagille syndrome)	JAG1
224705_s_at	0.46	trinucleotide repeat containing 6A	TNRC6A
241683_at	0.46	HECT domain containing 1	HECTD1
227312_at	0.46	syntrophin, beta 2 (dystrophin-associated protein A1, 59kDa, basic component 2)	SNTB2
201810_s_at	0.46	SH3-domain binding protein 5 (BTK-associated)	SH3BP5
1560559_at	0.46		
201975_at	0.46	CAP-GLY domain containing linker protein 1	CLIP1
218444_at	0.46	asparagine-linked glycosylation 12 homolog (S. cerevisiae, alpha-1,6-mannosyltransferase)	ALG12
223382_s_at	0.46	zinc and ring finger 1	ZNRF1
217824_at	0.46	ubiquitin-conjugating enzyme E2, J1 (UBC6 homolog, yeast)	UBE2J1
203019_x_at	0.46	synovial sarcoma, X breakpoint 2 interacting protein	SSX2IP
1559425_at	0.46		
236545_at	0.46		
211316_x_at	0.46	CASP8 and FADD-like apoptosis regulator	CFLAR
223184_s_at	0.46	1-acylglycerol-3-phosphate O-acyltransferase 3	AGPAT3
213306_at	0.46	multiple PDZ domain protein	MPDZ
243928_s_at	0.46	ATP-binding cassette, sub-family C (CFTR/MRP), member 4	ABCC4
220870_at	0.46		
226884_at	0.46	leucine rich repeat neuronal 1	LRRN1
232431_at	0.46		
235171_at	0.46		
216528_at	0.46		
214557_at	0.46	pituitary tumor-transforming 2	PTTG2
228837_at	0.46		
241025_at	0.46	UTP6, small subunit (SSU) processome component, homolog (yeast)	UTP6
237019_at	0.46		
226068_at	0.46	spleen tyrosine kinase	SYK
218517_at	0.46	PHD finger protein 17	PHF17
202197_at	0.46	myotubularin related protein 3	MTMR3
215775_at	0.46	thrombospondin 1	THBS1
226142_at	0.46	GLI pathogenesis-related 1 (glioma)	GLIPR1

224451_x_at	0.46	Rho GTPase activating protein 9	ARHGAP9
226179_at	0.46	solute carrier family 25, member 37	SLC25A37
91703_at	0.46	EH domain binding protein 1-like 1	EHBP1L1
220770_s_at	0.46		
203617_x_at	0.46	ELK1, member of ETS oncogene family	ELK1
1565886_at	0.46	transient receptor potential cation channel, subfamily M, member 7	TRPM7
203837_at	0.46	mitogen-activated protein kinase kinase kinase 5	MAP3K5
226384_at	0.46	phosphatidic acid phosphatase type 2 domain containing 1B	PPAPDC1B
212249_at	0.46	phosphoinositide-3-kinase, regulatory subunit 1 (p85 alpha)	PIK3R1
215595_x_at	0.46		
214881_s_at	0.46	upstream binding transcription factor, RNA polymerase I	UBTF
212796_s_at	0.46	TBC1 domain family, member 2B	TBC1D2B
240038_at	0.46		
235690_at	0.46	zinc finger protein 594	ZNF594
217996_at	0.46	pleckstrin homology-like domain, family A, member 1	PHLDA1
203151_at	0.46	microtubule-associated protein 1A	MAP1A
202692_s_at	0.46	upstream binding transcription factor, RNA polymerase I	UBTF
1564224_x_at	0.46		
201995_at	0.46	exostoses (multiple) 1	EXT1
225936_at	0.46	EP300 interacting inhibitor of differentiation 2	EID2
220199_s_at	0.46	chromosome 1 open reading frame 80	C1orf80
217953_at	0.46	PHD finger protein 3#null	PHF3#null
218543_s_at	0.46	poly (ADP-ribose) polymerase family, member 12	PARP12
238044_at	0.46		
1559172_at	0.46		
212758_s_at	0.46	transcription factor 8 (represses interleukin 2 expression)	TCF8
213844_at	0.46	homeobox A5	HOXA5
227180_at	0.46	ELOVL family member 7, elongation of long chain fatty acids (yeast)	ELOVL7
202510_s_at	0.46	tumor necrosis factor, alpha-induced protein 2	TNFAIP2
213069_at	0.45	HEG homolog 1 (zebrafish)	HEG1
244774_at	0.45		
211864_s_at	0.45	fer-1-like 3, myoferlin (C. elegans)	FER1L3
201549_x_at	0.45	jumonji, AT rich interactive domain 1B	JARID1B
226206_at	0.45	v-maf musculoaponeurotic fibrosarcoma oncogene homolog K (avian)	MAFK
209526_s_at	0.45		
222957_at	0.45	sialidase 4	NEU4
239208_s_at	0.45		
231964_at	0.45		
203411_s_at	0.45	lamin A/C	LMNA
226981_at	0.45	myeloid/lymphoid or mixed-lineage leukemia (trithorax homolog, Drosophila)	MLL
219497_s_at	0.45	B-cell CLL/lymphoma 11A (zinc finger protein)	BCL11A
228402_at	0.45	zinc finger, BED-type containing 3	ZBED3
219648_at	0.45	melanoregulin	MREG

239680_at	0.45		
219727_at	0.45	dual oxidase 2	DUOX2
210298_x_at	0.45	four and a half LIM domains 1	FHL1
212519_at	0.45	ubiquitin-conjugating enzyme E2E 1 (UBC4/5 homolog, yeast)	UBE2E1
204187_at	0.45	guanosine monophosphate reductase	GMPR
217904_s_at	0.45	beta-site APP-cleaving enzyme 1	BACE1
212471_at	0.45	KIAA0241	KIAA0241
236922_at	0.45		
242088_at	0.45	kelch-like 24 (Drosophila)	KLHL24
235500_at	0.45	heterogeneous nuclear ribonucleoprotein C (C1/C2)	HNRPC
1554274_a_at	0.45	slingshot homolog 1 (Drosophila)	SSH1
214163_at	0.45	chromosome 1 open reading frame 41	C1orf41
226183_at	0.45		
220668_s_at	0.45	DNA (cytosine-5-)-methyltransferase 3 beta	DNMT3B
217894_at	0.45	potassium channel tetramerisation domain containing 3	KCTD3
212117_at	0.45	ras homolog gene family, member Q	RHOQ
228685_at	0.45		
224204_x_at	0.45	aryl hydrocarbon receptor nuclear translocator-like 2	ARNTL2
229814_at	0.45		
219441_s_at	0.45	leucine-rich repeat kinase 1	LRRK1
51158_at	0.45		
229862_x_at	0.45	zinc finger and BTB domain containing 45	ZBTB45
226763_at	0.45	SEC14 and spectrin domains 1	SESTD1
211982_x_at	0.45	exportin 6	XPO6
235179_at	0.45	zinc finger protein 641	ZNF641
219880_at	0.45		
212268_at	0.45	serpin peptidase inhibitor, clade B (ovalbumin), member 1	SERPINB1
201751_at	0.45	Josephin domain containing 1	JOSD1
243948_at	0.45		
212476_at	0.45	centaurin, beta 2	CENTB2
218631_at	0.45	arginine vasopressin-induced 1	AVPI1
241910_x_at	0.45		
229437_at	0.45		
203623_at	0.45	plexin A3	PLXNA3
233616_at	0.45		
218414_s_at	0.45	nudE nuclear distribution gene E homolog 1 (A. nidulans)	NDE1
209561_at	0.45	thrombospondin 3	THBS3
227156_at	0.45	trinucleotide repeat containing 8	TNRC8
1557609_s_at	0.45	TBC1 domain family, member 12	TBC1D12
227095_at	0.45		
234734_s_at	0.45	trinucleotide repeat containing 6A	TNRC6A
217682_at	0.45		
218037_at	0.45	chromosome 2 open reading frame 17	C2orf17
217826_s_at	0.45	ubiquitin-conjugating enzyme E2, J1 (UBC6 homolog, yeast)	UBE2J1
202419_at	0.45	follicular lymphoma variant translocation 1	FVT1
230206_at	0.45	dedicator of cytokinesis 5	DOCK5

209285_s_at	0.45	chromosome 3 open reading frame 63	C3orf63
226979_at	0.45	mitogen-activated protein kinase kinase kinase 2	MAP3K2
209210_s_at	0.45	pleckstrin homology domain containing, family C (with FERM domain) member 1	PLEKHC1
231312_at	0.45		
239408_at	0.45	cerebral cavernous malformation 2	CCM2
203167_at	0.45	TIMP metallopeptidase inhibitor 2	TIMP2
200989_at	0.45	hypoxia-inducible factor 1, alpha subunit (basic helix-loop-helix transcription factor)	HIF1A
221485_at	0.45	UDP-Gal:betaGlcNAc beta 1,4-galactosyltransferase, polypeptide 5	B4GALT5
1560156_at	0.45		
225641_at	0.45	MADS box transcription enhancer factor 2, polypeptide D (myocyte enhancer factor 2D)	MEF2D
209905_at	0.45	homeobox A9	HOXA9
210078_s_at	0.45	potassium voltage-gated channel, shaker-related subfamily, beta member 1	KCNAB1
219335_at	0.45	armadillo repeat containing, X-linked 5	ARMCX5
202871_at	0.45	TNF receptor-associated factor 4	TRAF4
1557726_at	0.45	armadillo repeat containing, X-linked 4	ARMCX4
226801_s_at	0.45		
204254_s_at	0.45	vitamin D (1,25- dihydroxyvitamin D3) receptor	VDR
224701_at	0.45	poly (ADP-ribose) polymerase family, member 14	PARP14
1567539_at	0.45	sperm associated antigen 10	SPAG10
232129_s_at	0.45	leucine zipper, putative tumor suppressor 2	LZTS2
213693_s_at	0.45	mucin 1, cell surface associated	MUC1
203366_at	0.45	polymerase (DNA directed), gamma	POLG
1569142_at	0.45	tripartite motif-containing 13	TRIM13
227420_at	0.45	tumor necrosis factor, alpha-induced protein 8-like 1	TNFAIP8L1
201080_at	0.45	phosphatidylinositol-4-phosphate 5-kinase, type II, beta	PIP5K2B
214780_s_at	0.45	myosin IXB	MYO9B
1559077_at	0.45	ABI gene family, member 3 (NESH) binding protein	ABI3BP
1566901_at	0.45		
207837_at	0.45	RNA binding protein with multiple splicing	RBPMS
214743_at	0.45	cut-like 1, CCAAT displacement protein (Drosophila)	CUTL1
200899_s_at	0.45	meningioma expressed antigen 5 (hyaluronidase)	MGEA5
204963_at	0.45	sarcospan (Kras oncogene-associated gene)	SSPN
232647_at	0.45		
212708_at	0.45		
225816_at	0.45	PHD finger protein 17	PHF17
201925_s_at	0.45	CD55 molecule, decay accelerating factor for complement (Cromer blood group)	CD55
210405_x_at	0.45	tumor necrosis factor receptor superfamily, member 10b	TNFRSF10B
228314_at	0.45		
203139_at	0.45	death-associated protein kinase 1	DAPK1
210786_s_at	0.45	Friend leukemia virus integration 1	FLI1
203075_at	0.45	SMAD family member 2	SMAD2

211626_x_at	0.45	v-ets erythroblastosis virus E26 oncogene homolog (avian)	ERG
202431_s_at	0.45	v-myc myelocytomatosis viral oncogene homolog (avian)	MYC
211702_s_at	0.45	ubiquitin specific peptidase 32	USP32
212325_at	0.45		
212437_at	0.44	cell division cycle 25 homolog B (S. pombe)#centromere protein B, 80kDa#sialic acid binding Ig-like lectin 1, sialoadhesin#chromosome 20 open reading frame 28#chromosome 20 open reading frame 27#chromosome 20 open reading frame 29#null#ADAM metallopeptidase domain 33#heat shock 70kD protein 12B	CDC25B#CENPB#SIGLEC1#C20orf28#C20orf27#C20orf29#null#ADAM33#HSPA12B
226651_at	0.44		
216746_at	0.44		
225909_at	0.44	zinc finger protein 775	ZNF775
204413_at	0.44	TNF receptor-associated factor 2	TRAF2
209605_at	0.44	thiosulfate sulfurtransferase (rhodanese)	TST
211547_s_at	0.44	platelet-activating factor acetylhydrolase, isoform Ib, alpha subunit 45kDa	PAFAH1B1
214940_s_at	0.44	Smg-6 homolog, nonsense mediated mRNA decay factor (C. elegans)	SMG6
228006_at	0.44		
212463_at	0.44	CD59 molecule, complement regulatory protein	CD59
228955_at	0.44		
210201_x_at	0.44	bridging integrator 1	BIN1
217371_s_at	0.44	interleukin 15	IL15
225597_at	0.44	solute carrier family 45, member 4	SLC45A4
217910_x_at	0.44	MAX-like protein X	MLX
214196_s_at	0.44	tripeptidyl peptidase I	TPP1
213012_at	0.44	neural precursor cell expressed, developmentally down-regulated 4	NEDD4
225129_at	0.44	copine II	CPNE2
212722_s_at	0.44	phosphatidylserine receptor	PTDSR
225503_at	0.44	dehydrogenase/reductase (SDR family) X-linked	DHRSX
226985_at	0.44	FYVE, RhoGEF and PH domain containing 5	FGD5
228497_at	0.44	solute carrier family 22 (organic cation transporter), member 15	SLC22A15
1563245_at	0.44		
244313_at	0.44	complement component (3b/4b) receptor 1 (Knops blood group)	CR1
239272_at	0.44	matrix metallopeptidase 28	MMP28
224800_at	0.44	WD repeat and FYVE domain containing 1	WDFY1
205402_x_at	0.44	protease, serine, 2 (trypsin 2)	PRSS2
203505_at	0.44	ATP-binding cassette, sub-family A (ABC1), member 1	ABCA1
229013_at	0.44		
228410_at	0.44	GRB2-associated binding protein 3	GAB3
227985_at	0.44		
212977_at	0.44	chemokine (C-X-C motif) receptor 7	CXCR7

225472_at	0.44	allograft inflammatory factor 1#chloride intracellular channel 1#casein kinase 2, beta polypeptide#lymphotoxin alpha (TNF superfamily, member 1)#lymphotoxin beta (TNF superfamily, member 3)#mutS homolog 5 (E. coli)#mutS homolog 5 (E. coli)#tumor necrosis factor (TNF superfamily, member 2)#HLA-B associated transcript 2#HLA-B associated transcript 2#HLA-B associated transcript 3#HLA-B associated transcript 3#HLA-B associated transcript 4#HLA-B associated transcript 5#leukocyte specific transcript 1#dimethylarginine dimethylaminohydrolase 2#apolipoprotein M#chromosome 6 open reading frame 47#lymphocyte antigen 6 complex, locus G5B#lymphocyte antigen 6 complex, locus G6D#lymphocyte antigen 6 complex, locus G6E#chromosome 6 open reading frame 25#lymphocyte antigen 6 complex, locus G6C#lymphocyte antigen 6 complex, locus G5C#natural cytotoxicity triggering receptor 3#chromosome 6 open reading frame 21	AIF1#CLIC1#CSNK2B#LTA#LTB#MSH5#MSH5#TNF#BAT2#BAT2#BAT3#BAT3#BAT4#BAT5#LST1#DDAH2#APOM#C6orf47#LY6G5B#LY6G6D#LY6G6E#C6orf25#LY6G6C#LY6G5C#NCR3#C6orf21
203420_at	0.44	family with sequence similarity 8, member A1	FAM8A1
214806_at	0.44	bicaudal D homolog 1 (Drosophila)	BICD1
1560514_at	0.44		
227684_at	0.44		
202378_s_at	0.44	leptin receptor overlapping transcript	LEPROT
205926_at	0.44	interleukin 27 receptor, alpha	IL27RA
209651_at	0.44	transforming growth factor beta 1 induced transcript 1	TGFB1I1
1557450_s_at	0.44		
203100_s_at	0.44	chromodomain protein, Y-like	CDYL
227687_at	0.44	hydrolethalus syndrome 1	HYLS1
45687_at	0.44	proline rich 14	PRR14
213188_s_at	0.44	MYC induced nuclear antigen	MINA
1558212_at	0.44		
226686_at	0.44	zinc finger, CDGSH-type domain 2	ZCD2
202466_at	0.44	polymerase (DNA directed) sigma	POLS
242468_at	0.44		
208178_x_at	0.44	triple functional domain (PTPRF interacting)	TRIO
204346_s_at	0.44	Ras association (RalGDS/AF-6) domain family 1	RASSF1
228909_at	0.44	chromosome 21 open reading frame 86	C21orf86
236607_at	0.44		
238516_at	0.44	bone morphogenetic protein receptor, type II (serine/threonine kinase)	BMPR2
203217_s_at	0.44	ST3 beta-galactoside alpha-2,3-sialyltransferase 5	ST3GAL5
225288_at	0.44		
212288_at	0.44	formin binding protein 1	FNBP1
228550_at	0.44	reticulon 4 receptor	RTN4R
201669_s_at	0.44	myristoylated alanine-rich protein kinase C substrate	MARCKS

208944_at	0.44	transforming growth factor, beta receptor II (70/80kDa)	TGFBR2
225567_at	0.44		
201660_at	0.44		
204481_at	0.44	bromodomain and PHD finger containing, 1	BRPF1
236385_at	0.44		
237602_at	0.44		
203713_s_at	0.44	lethal giant larvae homolog 2 (Drosophila)	LLGL2
201063_at	0.44	reticulocalbin 1, EF-hand calcium binding domain	RCN1
209122_at	0.44	adipose differentiation-related protein	ADFP
241896_at	0.44	microtubule-actin crosslinking factor 1	MACF1
236975_at	0.44		
212860_at	0.44	zinc finger, DHHC-type containing 18	ZDHHC18
212184_s_at	0.44	mitogen-activated protein kinase kinase kinase 7 interacting protein 2	MAP3K7IP2
210233_at	0.44	interleukin 1 receptor accessory protein	IL1RAP
222877_at	0.44		
228027_at	0.44		
212465_at	0.44	SET domain containing 3	SETD3
212319_at	0.44	RUN and TBC1 domain containing 1	RUTBC1
212233_at	0.44		
235658_at	0.44		
230998_at	0.44		
212377_s_at	0.44	Notch homolog 2 (Drosophila)	NOTCH2
211250_s_at	0.44	SH3-domain binding protein 2	SH3BP2
232181_at	0.44		
239719_at	0.44	CD109 molecule	CD109
202445_s_at	0.44	Notch homolog 2 (Drosophila)	NOTCH2
202637_s_at	0.44	intercellular adhesion molecule 1 (CD54), human rhinovirus receptor	ICAM1
1555845_at	0.44		
239450_at	0.44		
212759_s_at	0.44	transcription factor 7-like 2 (T-cell specific, HMG-box)	TCF7L2
244783_at	0.44	YY1 associated factor 2	YAF2
212602_at	0.44	WD repeat and FYVE domain containing 3	WDFY3
232918_at	0.44		
207466_at	0.44	galanin	GAL
229064_s_at	0.44	Down syndrome critical region gene 1-like 2	DSCR1L2
1557316_at	0.44		
226018_at	0.44	chromosome 7 open reading frame 41	C7orf41
210205_at	0.44	UDP-Gal:betaGlcNAc beta 1,3-galactosyltransferase, polypeptide 4	B3GALT4
240134_at	0.44		
212952_at	0.44	calreticulin	CALR
242488_at	0.44		
202955_s_at	0.44	ADP-ribosylation factor guanine nucleotide-exchange factor 1(brefeldin A-inhibited)	ARFGEF1
206649_s_at	0.44	transcription factor binding to IGHM enhancer 3	TFE3
91617_at	0.44	DiGeorge syndrome critical region gene 8	DGCR8
235174_s_at	0.44		
237768_x_at	0.44		
1560874_at	0.44		

36129_at	0.44	RUN and TBC1 domain containing 1	RUTBC1
203819_s_at	0.44	insulin-like growth factor 2 mRNA binding protein 3	IGF2BP3
206347_at	0.44	pyruvate dehydrogenase kinase, isozyme 3	PDK3
238890_at	0.44		
219620_x_at	0.44		
200083_at	0.44	ubiquitin specific peptidase 22	USP22
234021_at	0.44	echinoderm microtubule associated protein like 2	EML2
209811_at	0.44	caspase 2, apoptosis-related cysteine peptidase (neural precursor cell expressed, developmentally do	CASP2
228854_at	0.44		
208087_s_at	0.44	Z-DNA binding protein 1	ZBP1
227539_at	0.44		
227066_at	0.44	MOB1, Mps One Binder kinase activator-like 2C (yeast)	MOBKL2C
219821_s_at	0.44	glucose-fructose oxidoreductase domain containing 1	GFOD1
243671_at	0.44		
209149_s_at	0.44	transmembrane 9 superfamily member 1	TM9SF1
235052_at	0.44	zinc finger protein 792	ZNF792
1565627_a_at	0.44		
201959_s_at	0.43	MYC binding protein 2	MYCBP2
210846_x_at	0.43	tripartite motif-containing 14	TRIM14
225533_at	0.43	PHD finger protein 19	PHF19
225701_at	0.43	AT-hook transcription factor	AKNA
200760_s_at	0.43	ADP-ribosylation-like factor 6 interacting protein 5	ARL6IP5
221698_s_at	0.43	C-type lectin domain family 7, member A	CLEC7A
242256_x_at	0.43		
229623_at	0.43		
210873_x_at	0.43	apolipoprotein B mRNA editing enzyme, catalytic polypeptide-like 3A	APOBEC3A
226743_at	0.43	schlafen family member 11	SLFN11
219484_at	0.43	host cell factor C2	HCFC2
211089_s_at	0.43	NIMA (never in mitosis gene a)-related kinase 3	NEK3
396_f_at	0.43	erythropoietin receptor	EPOR
201494_at	0.43	prolylcarboxypeptidase (angiotensinase C)	PRCP
212428_at	0.43	KIAA0368	KIAA0368
228231_at	0.43		
207319_s_at	0.43	cell division cycle 2-like 5 (cholinesterase-related cell division controller)	CDC2L5
237106_at	0.43	solute carrier family 11 (proton-coupled divalent metal ion transporters), member 2	SLC11A2
201195_s_at	0.43	solute carrier family 7 (cationic amino acid transporter, y+ system), member 5	SLC7A5
1563772_a_at	0.43	laminin, alpha 3	LAMA3
215245_x_at	0.43	fragile X mental retardation 1	FMR1
200897_s_at	0.43	palladin, cytoskeletal associated protein	PALLD
220252_x_at	0.43	chromosome X open reading frame 21	CXorf21
224162_s_at	0.43	F-box protein 31	FBXO31
218669_at	0.43	RAP2C, member of RAS oncogene family	RAP2C
202365_at	0.43		
226629_at	0.43	solute carrier family 43, member 2	SLC43A2

236261_at	0.43		
232997_at	0.43		
214221_at	0.43	Alstrom syndrome 1	ALMS1
218819_at	0.43	integrator complex subunit 6	INTS6
227679_at	0.43		
209667_at	0.43	carboxylesterase 2 (intestine, liver)	CES2
212474_at	0.43	KIAA0241	KIAA0241
228565_at	0.43		
240115_at	0.43		
227119_at	0.43	CCR4-NOT transcription complex, subunit 6-like	CNOT6L
231815_at	0.43	PHD finger protein 12	PHF12
218878_s_at	0.43	sirtuin (silent mating type information regulation 2 homolog) 1 (S. cerevisiae)	SIRT1
212761_at	0.43	transcription factor 7-like 2 (T-cell specific, HMG-box)	TCF7L2
227125_at	0.43		
221511_x_at	0.43	cell cycle progression 1	CCPG1
225564_at	0.43	spermatogenesis associated 13	SPATA13
219381_at	0.43		
225325_at	0.43		
243561_at	0.43		
211202_s_at	0.43	jumonji, AT rich interactive domain 1B	JARID1B
204040_at	0.43	ring finger protein 144	RNF144
204049_s_at	0.43	phosphatase and actin regulator 2	PHACTR2
41387_r_at	0.43	jumonji domain containing 3	JMJD3
207339_s_at	0.43	lymphotoxin beta (TNF superfamily, member 3)	LTB
227177_at	0.43	coronin, actin binding protein, 2A	CORO2A
236164_at	0.43		
211077_s_at	0.43	tousled-like kinase 1	TLK1
224813_at	0.43	Wiskott-Aldrich syndrome-like	WASL
224046_s_at	0.43	phosphodiesterase 7A	PDE7A
202051_s_at	0.43	zinc finger, MYM-type 4	ZMYM4
205269_at	0.43	lymphocyte cytosolic protein 2 (SH2 domain containing leukocyte protein of 76kDa)	LCP2
210007_s_at	0.43	glycerol-3-phosphate dehydrogenase 2 (mitochondrial)	GPD2
236810_at	0.43		
214356_s_at	0.43		
201580_s_at	0.43	thioredoxin domain containing 13	TXNDC13
212730_at	0.43	desmuslin	DMN
219155_at	0.43	phosphatidylinositol transfer protein, cytoplasmic 1	PITPNC1
205099_s_at	0.43	chemokine (C-C motif) receptor 1	CCR1
217734_s_at	0.43	WD repeat domain 6	WDR6
229984_at	0.43		
214596_at	0.43		
225764_at	0.43	ets variant gene 6 (TEL oncogene)	ETV6
204048_s_at	0.43	phosphatase and actin regulator 2	PHACTR2
212472_at	0.43	microtubule associated monoxygenase, calponin and LIM domain containing 2	MICAL2
222668_at	0.43	potassium channel tetramerisation domain containing 15	KCTD15
64418_at	0.43		

219282_s_at	0.43	transient receptor potential cation channel, subfamily V, member 2	TRPV2
227692_at	0.43	guanine nucleotide binding protein (G protein), alpha inhibiting activity polypeptide 1	GNAI1
210361_s_at	0.43	E74-like factor 2 (ets domain transcription factor)	ELF2
235177_at	0.43	family with sequence similarity 119, member A	FAM119A
229344_x_at	0.43	family with sequence similarity 80, member B	FAM80B
202923_s_at	0.43	glutamate-cysteine ligase, catalytic subunit	GCLC
205389_s_at	0.43	ankyrin 1, erythrocytic	ANK1
221806_s_at	0.43	SET domain containing 5	SETD5
209091_s_at	0.43	SH3-domain GRB2-like endophilin B1	SH3GLB1
208623_s_at	0.43	villin 2 (ezrin)	VIL2
213147_at	0.43	homeobox A10	HOXA10
1552664_at	0.43	folliculin	FLCN
227087_at	0.43		
219020_at	0.43	HCLS1 binding protein 3	HS1BP3
203977_at	0.43	tafazzin (cardiomyopathy, dilated 3A (X-linked); endocardial fibroelastosis 2; Barth syndrome)	TAZ
236114_at	0.43		
215446_s_at	0.43	lysyl oxidase	LOX
212322_at	0.43	sphingosine-1-phosphate lyase 1	SGPL1
225411_at	0.43		
209540_at	0.43	insulin-like growth factor 1 (somatomedin C)	IGF1
224906_at	0.43	transmembrane protein 16F	TMEM16F
206034_at	0.43	serpin peptidase inhibitor, clade B (ovalbumin), member 8	SERPINB8
242551_at	0.43	chromosome 18 open reading frame 1	C18orf1
214708_at	0.43	syntrophin, beta 1 (dystrophin-associated protein A1, 59kDa, basic component 1)	SNTB1
226668_at	0.43	WD repeat, sterile alpha motif and U-box domain containing 1	WDSUB1
206039_at	0.43	RAB33A, member RAS oncogene family	RAB33A
211421_s_at	0.43	ret proto-oncogene (multiple endocrine neoplasia and medullary thyroid carcinoma 1, Hirschsprung dis	RET
210870_s_at	0.43	epilepsy, progressive myoclonus type 2A, Lafora disease (laforin)	EPM2A
209724_s_at	0.43	zinc finger protein 161 homolog (mouse)	ZFP161
224215_s_at	0.43	delta-like 1 (Drosophila)	DLL1
239609_s_at	0.43	1-acylglycerol-3-phosphate O-acyltransferase 7 (lysophosphatidic acid acyltransferase, eta)	AGPAT7
225707_at	0.43	ADP-ribosylation-like factor 6 interacting protein 6	ARL6IP6
212956_at	0.43	TBC1 domain family, member 9 (with GRAM domain)	TBC1D9
229256_at	0.42	phosphoglucomutase 2-like 1	PGM2L1
225897_at	0.42		
226725_at	0.42		
230389_at	0.42	formin binding protein 1	FNBP1
239884_at	0.42	Ca2+-dependent secretion activator	CADPS
210101_x_at	0.42	SH3-domain GRB2-like endophilin B1	SH3GLB1
222294_s_at	0.42	RAB27A, member RAS oncogene family	RAB27A
203018_s_at	0.42	synovial sarcoma, X breakpoint 2 interacting protein	SSX2IP

221524_s_at	0.42	Ras-related GTP binding D	RRAGD
212201_at	0.42	KIAA0692	KIAA0692
38398_at	0.42	MAP-kinase activating death domain	MADD
227545_at	0.42		
201460_at	0.42	mitogen-activated protein kinase-activated protein kinase 2	MAPKAPK2
206238_s_at	0.42	YY1 associated factor 2	YAF2
242999_at	0.42	Rho guanine nucleotide exchange factor (GEF) 7	ARHGEF7
203003_at	0.42	MADS box transcription enhancer factor 2, polypeptide D (myocyte enhancer factor 2D)	MEF2D
1561061_at	0.42		
203578_s_at	0.42	transient receptor potential cation channel, subfamily V, member 6	TRPV6
1558522_at	0.42		
219696_at	0.42		
204923_at	0.42	X-prolyl aminopeptidase (aminopeptidase P) 2, membrane-bound#chromosome X open reading frame 9	XPNPEP2#CXorf9
235971_at	0.42		
226368_at	0.42	carbohydrate (chondroitin 4) sulfotransferase 11	CHST11
222156_x_at	0.42	cell cycle progression 1	CCPG1
211208_s_at	0.42	calcium/calmodulin-dependent serine protein kinase (MAGUK family)	CASK
226468_at	0.42		
229394_s_at	0.42	glucocorticoid receptor DNA binding factor 1	GRLF1
212057_at	0.42	KIAA0182	KIAA0182
1552625_a_at	0.42	tRNA nucleotidyl transferase, CCA-adding, 1	TRNT1
242834_at	0.42		
208322_s_at	0.42	ST3 beta-galactoside alpha-2,3-sialyltransferase 1	ST3GAL1
218176_at	0.42	melanoma antigen family F, 1	MAGEF1
232000_at	0.42	chromosome 9 open reading frame 52	C9orf52
233559_s_at	0.42	WD repeat and FYVE domain containing 1	WDFY1
223590_at	0.42	zinc finger protein 700	ZNF700
228588_s_at	0.42	ubiquitin-conjugating enzyme E2B (RAD6 homolog)	UBE2B
221903_s_at	0.42	cylindromatosis (turban tumor syndrome)	CYLD
208540_x_at	0.42	S100 calcium binding protein A11	S100A11
217750_s_at	0.42	ubiquitin-conjugating enzyme E2Z (putative)	UBE2Z
228073_at	0.42	N-acetylneuraminic acid phosphatase	NANP
236346_at	0.42	zinc finger protein 83	ZNF83
208998_at	0.42	uncoupling protein 2 (mitochondrial, proton carrier)	UCP2
210401_at	0.42	purinergic receptor P2X, ligand-gated ion channel, 1	P2RX1
218668_s_at	0.42	RAP2C, member of RAS oncogene family	RAP2C
1561439_at	0.42		
227344_at	0.42	IKAROS family zinc finger 1 (Ikaros)	IKZF1
223805_at	0.42	oxysterol binding protein-like 6	OSBPL6
212380_at	0.42	KIAA0082	KIAA0082
208275_x_at	0.42	undifferentiated embryonic cell transcription factor 1	UTF1
202047_s_at	0.42	chromobox homolog 6	CBX6

202443_x_at	0.42	Notch homolog 2 (Drosophila)	NOTCH2
211577_s_at	0.42	insulin-like growth factor 1 (somatomedin C)	IGF1
214808_at	0.42		
241925_x_at	0.42		
212070_at	0.42	G protein-coupled receptor 56	GPR56
230966_at	0.42	nucleoporin 62kDa	NUP62
209001_s_at	0.42	anaphase promoting complex subunit 13	ANAPC13
1557411_s_at	0.42	solute carrier family 25, member 43	SLC25A43
225490_at	0.42	AT rich interactive domain 2 (ARID, RFX-like)	ARID2
221261_x_at	0.42	melanoma antigen family D, 4	MAGED4
231829_at	0.42		
232001_at	0.42		
205098_at	0.42	chemokine (C-C motif) receptor 1	CCR1
225277_at	0.42	solute carrier family 39 (zinc transporter), member 13	SLC39A13
212770_at	0.42	transducin-like enhancer of split 3 (E(sp1) homolog, Drosophila)	TLE3
228914_at	0.42		
203313_s_at	0.42	TGFB-induced factor homeobox 1	TGIF1
202417_at	0.42	kelch-like ECH-associated protein 1	KEAP1
202968_s_at	0.42	dual-specificity tyrosine-(Y)-phosphorylation regulated kinase 2	DYRK2
227319_at	0.42		
225115_at	0.42	homeodomain interacting protein kinase 2	HIPK2
231165_at	0.42	DDHD domain containing 1	DDHD1
202382_s_at	0.42	glucosamine-6-phosphate deaminase 1	GNPDA1
241383_at	0.42		
230943_at	0.42		
224995_at	0.42	spire homolog 1 (Drosophila)	SPIRE1
218323_at	0.42	ras homolog gene family, member T1	RHOT1
220974_x_at	0.42	sideroflexin 3	SFXN3
206052_s_at	0.42	stem-loop (histone) binding protein	SLBP
206748_s_at	0.42	sperm associated antigen 9	SPAG9
240088_at	0.42	phosphodiesterase 5A, cGMP-specific	PDE5A
208749_x_at	0.42	flotillin 1	FLOT1
220449_at	0.42		
224649_x_at	0.42	chromosome 10 open reading frame 9	C10orf9
204497_at	0.42	adenylate cyclase 9	ADCY9
240549_at	0.42		
213562_s_at	0.42	squalene epoxidase	SQLE
222859_s_at	0.42	dual adaptor of phosphotyrosine and 3-phosphoinositides	DAPP1
218818_at	0.42	four and a half LIM domains 3	FHL3
214696_at	0.42		
221256_s_at	0.42	haloacid dehalogenase-like hydrolase domain containing 3	HDHD3
240498_at	0.42	ets variant gene 6 (TEL oncogene)	ETV6
228937_at	0.42	chromosome 13 open reading frame 31	C13orf31
224938_at	0.42		
205210_at	0.42	transforming growth factor, beta receptor associated protein 1	TGFBRAP1
239580_at	0.42		
204713_s_at	0.42	coagulation factor V (proaccelerin, labile factor)	F5

235295_at	0.42		
204452_s_at	0.42	frizzled homolog 1 (Drosophila)	FZD1
212620_at	0.42	zinc finger protein 609	ZNF609
241402_at	0.42	tRNA splicing endonuclease 54 homolog (S. cerevisiae)	TSEN54
1560184_at	0.42	transmembrane and coiled-coil domains 5	TMCO5
225735_at	0.42	ankyrin repeat domain 50	ANKRD50
223093_at	0.42	ankylosis, progressive homolog (mouse)	ANKH
45749_at	0.42	family with sequence similarity 65, member A	FAM65A
236035_at	0.42		
209701_at	0.42	calpastatin	CAST
236369_at	0.42		
207037_at	0.42	tumor necrosis factor receptor superfamily, member 11a, NFKB activator	TNFRSF11A
221222_s_at	0.42	chromosome 1 open reading frame 56	C1orf56
202958_at	0.42	protein tyrosine phosphatase, non-receptor type 9	PTPN9
203734_at	0.42	forkhead box J2	FOXJ2
202880_s_at	0.42	pleckstrin homology, Sec7 and coiled-coil domains 1(cytohesin 1)	PSCD1
224807_at	0.42	GRAM domain containing 1A	GRAMD1A
209933_s_at	0.42	CD300a molecule	CD300A
212948_at	0.42	calmodulin binding transcription activator 2	CAMTA2
234968_at	0.42	DENN/MADD domain containing 4C	DENND4C
212564_at	0.42	potassium channel tetramerisation domain containing 2	KCTD2
226251_at	0.41	additional sex combs like 2 (Drosophila)	ASXL2
202928_s_at	0.41	PHD finger protein 1	PHF1
212711_at	0.41	calmodulin regulated spectrin-associated protein 1	CAMSAP1
227167_s_at	0.41		
239474_at	0.41		
204683_at	0.41	intercellular adhesion molecule 2	ICAM2
226885_at	0.41		
227143_s_at	0.41	BH3 interacting domain death agonist	BID
234366_x_at	0.41	immunoglobulin lambda locus	IGL@
207872_s_at	0.41	leukocyte immunoglobulin-like receptor, subfamily A (with TM domain), member 1	LILRA1
212425_at	0.41	secretory carrier membrane protein 1	SCAMP1
244377_at	0.41	solute carrier family 1 (glutamate/neutral amino acid transporter), member 4	SLC1A4
222793_at	0.41	DEAD (Asp-Glu-Ala-Asp) box polypeptide 58	DDX58
210875_s_at	0.41	transcription factor 8 (represses interleukin 2 expression)	TCF8
226197_at	0.41		
235256_s_at	0.41	galactose mutarotase (aldose 1-epimerase)	GALM
213309_at	0.41	phospholipase C-like 2	PLCL2
236591_at	0.41		
224190_x_at	0.41	nucleotide-binding oligomerization domain containing 1	NOD1
203298_s_at	0.41	jumonji, AT rich interactive domain 2	JARID2
210252_s_at	0.41	MAP-kinase activating death domain	MADD
202545_at	0.41	protein kinase C, delta	PRKCD

232884_s_at	0.41		
234974_at	0.41	galactose mutarotase (aldose 1-epimerase)	GALM
212285_s_at	0.41	agrin	AGRIN
228869_at	0.41		
1569521_s_at	0.41		
200090_at	0.41	farnesyltransferase, CAAX box, alpha	FNTA
226512_at	0.41		
209457_at	0.41	dual specificity phosphatase 5	DUSP5
1556730_at	0.41		
1568592_at	0.41	tripartite motif-containing 69	TRIM69
203471_s_at	0.41	pleckstrin	PLEK
203372_s_at	0.41	suppressor of cytokine signaling 2	SOCS2
228954_at	0.41	LysM, putative peptidoglycan-binding, domain containing 4	LYSMD4
218032_at	0.41	stannin	SNN
224889_at	0.41	forkhead box O3A	FOXO3A
1556950_s_at	0.41	serpin peptidase inhibitor, clade B (ovalbumin), member 6	SERPINB6
206157_at	0.41	pentraxin-related gene, rapidly induced by IL-1 beta	PTX3
204226_at	0.41	staufen, RNA binding protein, homolog 2 (Drosophila)	STAU2
203044_at	0.41	carbohydrate (chondroitin) synthase 1	CHSY1
218705_s_at	0.41	sorting nexing 24	SNX24
223028_s_at	0.41	sorting nexin 9	SNX9
205251_at	0.41	period homolog 2 (Drosophila)	PER2
214319_at	0.41	furry homolog (Drosophila)	FRY
205038_at	0.41	IKAROS family zinc finger 1 (Ikaros)	IKZF1
219777_at	0.41	GTPase, IMAP family member 6	GIMAP6
224834_at	0.41	ubiquitin domain containing 2	UBTD2
200919_at	0.41	polyhomeotic homolog 2 (Drosophila)	PHC2
236600_at	0.41	spastic paraplegia 20, spartin (Troyer syndrome)	SPG20
203853_s_at	0.41	GRB2-associated binding protein 2	GAB2
213326_at	0.41	vesicle-associated membrane protein 1 (synaptobrevin 1)	VAMP1
234489_at	0.41		
211825_s_at	0.41	Ewing sarcoma breakpoint region 1	EWSR1
231996_at	0.41		
213138_at	0.41	AT rich interactive domain 5A (MRF1-like)	ARID5A
228494_at	0.41	protein phosphatase 1, regulatory (inhibitor) subunit 9A	PPP1R9A
200632_s_at	0.41	N-myc downstream regulated gene 1	NDRG1
1553147_at	0.41	RAN binding protein 3-like	RANBP3L
226440_at	0.41	dual specificity phosphatase 22	DUSP22
217901_at	0.41	desmoglein 2	DSG2
225351_at	0.41	family with sequence similarity 45, member A	FAM45A
217844_at	0.41	CTD (carboxy-terminal domain, RNA polymerase II, polypeptide A) small phosphatase 1	CTDSP1
209341_s_at	0.41	inhibitor of kappa light polypeptide gene enhancer in B-cells, kinase beta	IKBKB
226912_at	0.41	zinc finger, DHHC-type containing 23	ZDHHC23
210249_s_at	0.41	nuclear receptor coactivator 1	NCOA1
223627_at	0.41	ring finger and KH domain containing 3	RKHD3

1562850_at	0.41		
222777_s_at	0.41	Wolf-Hirschhorn syndrome candidate 1	WHSC1
243475_at	0.41	Cas-Br-M (murine) ecotropic retroviral transforming sequence	CBL
226872_at	0.41	regulatory factor X, 2 (influences HLA class II expression)	RFX2
207610_s_at	0.41	egf-like module containing, mucin-like, hormone receptor-like 2	EMR2
201490_s_at	0.41	peptidylprolyl isomerase F (cyclophilin F)	PPIF
222343_at	0.41	BCL2-like 11 (apoptosis facilitator)	BCL2L11
202239_at	0.41	poly (ADP-ribose) polymerase family, member 4	PARP4
203693_s_at	0.41	E2F transcription factor 3	E2F3
243444_at	0.41		
221064_s_at	0.41	chromosome 16 open reading frame 28	C16orf28
201647_s_at	0.41	scavenger receptor class B, member 2	SCARB2
239449_at	0.41	ankylosis, progressive homolog (mouse)	ANKH
222471_s_at	0.41	potassium channel modulatory factor 1	KCMF1
209105_at	0.41		
244458_at	0.41	zinc finger protein 592	ZNF592
211593_s_at	0.41	microtubule associated serine/threonine kinase 2	MAST2
243116_at	0.41	phosphatidylinositol-4-phosphate 5-kinase-like 1	PIP5KL1
213579_s_at	0.41	E1A binding protein p300	EP300
202315_s_at	0.41	breakpoint cluster region	BCR
235657_at	0.41		
222196_at	0.41		
221676_s_at	0.41	coronin, actin binding protein, 1C	CORO1C
244389_at	0.41		
214933_at	0.41	calcium channel, voltage-dependent, P/Q type, alpha 1A subunit	CACNA1A
209863_s_at	0.41	tumor protein p73-like	TP73L
244058_at	0.41	chromosome 10 open reading frame 72	C10orf72
203552_at	0.40	mitogen-activated protein kinase kinase kinase kinase 5	MAP4K5
234138_at	0.40		
207738_s_at	0.40	NCK-associated protein 1	NCKAP1
200984_s_at	0.40	CD59 molecule, complement regulatory protein	CD59
203795_s_at	0.40	B-cell CLL/lymphoma 7A	BCL7A
210644_s_at	0.40	leukocyte-associated immunoglobulin-like receptor 1	LAIR1
225060_at	0.40	low density lipoprotein receptor-related protein 11	LRP11
219797_at	0.40	mannosyl (alpha-1,3-)-glycoprotein beta-1,4-N-acetylglucosaminyltransferase, isozyme A	MGAT4A
228595_at	0.40		
227401_at	0.40	interleukin 17D	IL17D
221870_at	0.40	EH-domain containing 2	EHD2
204206_at	0.40	MAX binding protein	MNT
210951_x_at	0.40	RAB27A, member RAS oncogene family	RAB27A
204411_at	0.40	kinesin family member 21B	KIF21B
234099_at	0.40		
204639_at	0.40	adenosine deaminase	ADA
244773_at	0.40		

226122_at	0.40	methylenetetrahydrofolate dehydrogenase (NADP+ dependent) 1-like#pleckstrin homology domain containing, family G (with RhoGef domain) member 1	MTHFD1L#PLEKHG1
212097_at	0.40	caveolin 1, caveolae protein, 22kDa	CAV1
243431_at	0.40	BTB (POZ) domain containing 14A	BTBD14A
226495_at	0.40		
217977_at	0.40	selenoprotein X, 1	SEPX1
231175_at	0.40	chromosome 6 open reading frame 65	C6orf65
218759_at	0.40	dishevelled, dsh homolog 2 (Drosophila)	DVL2
224580_at	0.40	solute carrier family 38, member 1	SLC38A1
202101_s_at	0.40	v-ral simian leukemia viral oncogene homolog B (ras related; GTP binding protein)	RALB
212267_at	0.40	wings apart-like homolog (Drosophila)	WAPAL
1555294_a_at	0.40	ELKS/RAB6-interacting/CAST family member 1	ERC1
217234_s_at	0.40	villin 2 (ezrin)	VIL2
212607_at	0.40	v-akt murine thymoma viral oncogene homolog 3 (protein kinase B, gamma)	AKT3
201220_x_at	0.40	C-terminal binding protein 2	CTBP2
244461_at	0.40	sperm antigen with calponin homology and coiled-coil domains 1	SPECC1
219045_at	0.40	ras homolog gene family, member F (in filopodia)	RHOF
230860_at	0.40		
204078_at	0.40		
210139_s_at	0.40	peripheral myelin protein 22	PMP22
213258_at	0.40	tissue factor pathway inhibitor (lipoprotein-associated coagulation inhibitor)	TFPI
1555888_at	0.40	E3 ubiquitin protein ligase, HECT domain containing, 1	EDD1
201960_s_at	0.40	MYC binding protein 2	MYCBP2
214964_at	0.40		
209426_s_at	0.40	alpha-methylacyl-CoA racemase	AMACR
210664_s_at	0.40	tissue factor pathway inhibitor (lipoprotein-associated coagulation inhibitor)	TFPI
225585_at	0.40	RAP2A, member of RAS oncogene family	RAP2A
221087_s_at	0.40	apolipoprotein L, 3	APOL3
241984_at	0.40	checkpoint suppressor 1	CHES1
205503_at	0.40	protein tyrosine phosphatase, non-receptor type 14	PTPN14
221477_s_at	0.40	superoxide dismutase 2, mitochondrial	SOD2
236140_at	0.40	glutamate-cysteine ligase, modifier subunit	GCLM
203820_s_at	0.40	insulin-like growth factor 2 mRNA binding protein 3	IGF2BP3
225446_at	0.40	bromodomain and WD repeat domain containing 1	BRWD1
207614_s_at	0.40	cullin 1	CUL1
221221_s_at	0.40	kelch-like 3 (Drosophila)	KLHL3
226447_at	0.40	ash1 (absent, small, or homeotic)-like (Drosophila)	ASH1L
226974_at	0.40		
243469_at	0.40		
216569_at	0.40	fatty acid binding protein 3, pseudogene 2	FABP3P2
1568943_at	0.40	inositol polyphosphate-5-phosphatase, 145kDa	INPP5D

243539_at	0.40		
201952_at	0.40	activated leukocyte cell adhesion molecule	ALCAM
226568_at	0.40	family with sequence similarity 102, member B	FAM102B
218091_at	0.40	HIV-1 Rev binding protein	HRB
207318_s_at	0.40	cell division cycle 2-like 5 (cholinesterase-related cell division controller)	CDC2L5
201749_at	0.40	endothelin converting enzyme 1	ECE1
1556471_at	0.40	sex comb on midleg-like 4 (Drosophila)	SCML4
1556624_at	0.40		
227020_at	0.40	yippee-like 2 (Drosophila)	YPEL2
213386_at	0.40		
228063_s_at	0.40	nucleosome assembly protein 1-like 5	NAP1L5
226832_at	0.40		
220809_at	0.40		
215285_s_at	0.40	putative homeodomain transcription factor 1	PHTF1
226150_at	0.40	phosphatidic acid phosphatase type 2 domain containing 1B	PPAPDC1B
201548_s_at	0.40	jumonji, AT rich interactive domain 1B	JARID1B
210044_s_at	0.40	lymphoblastic leukemia derived sequence 1	LYL1
222715_s_at	0.40	AP1 gamma subunit binding protein 1	AP1GBP1
212486_s_at	0.40		
213725_x_at	0.40	xylosyltransferase I	XYLT1
221935_s_at	0.40	chromosome 3 open reading frame 64	C3orf64
212086_x_at	0.40	lamin A/C	LMNA
226773_at	0.40		
209735_at	0.40	ATP-binding cassette, sub-family G (WHITE), member 2	ABCG2
212646_at	0.40	raftlin, lipid raft linker 1	RFTN1
201594_s_at	0.40	protein phosphatase 4, regulatory subunit 1	PPP4R1
233539_at	0.40		
234801_s_at	0.40	acyl-CoA synthetase short-chain family member 1	ACSS1
201489_at	0.40	peptidylprolyl isomerase F (cyclophilin F)	PPIF
242887_at	0.40		
230894_s_at	0.39		
45297_at	0.39	EH-domain containing 2	EHD2
218363_at	0.39	exonuclease 3'-5' domain-like 2	EXDL2
225447_at	0.39	glycerol-3-phosphate dehydrogenase 2 (mitochondrial)	GPD2
226909_at	0.39		
212239_at	0.39	phosphoinositide-3-kinase, regulatory subunit 1 (p85 alpha)	PIK3R1
207904_s_at	0.39	leucyl/cystinyl aminopeptidase	LNPEP
228677_s_at	0.39		
207467_x_at	0.39	calpastatin	CAST
1556839_s_at	0.39	spectrin, beta, non-erythrocytic 5	SPTBN5
1559203_s_at	0.39	v-Ki-ras2 Kirsten rat sarcoma viral oncogene homolog	KRAS
221704_s_at	0.39	vacuolar protein sorting 37 homolog B (S. cerevisiae)	VPS37B
202922_at	0.39	glutamate-cysteine ligase, catalytic subunit	GCLC
201471_s_at	0.39	sequestosome 1	SQSTM1

212448_at	0.39	neural precursor cell expressed, developmentally down-regulated 4-like	NEDD4L
226314_at	0.39	dermatan 4 sulfotransferase 1	D4ST1
229342_at	0.39		
224558_s_at	0.39	metastasis associated lung adenocarcinoma transcript 1 (non-coding RNA)	MALAT1
225231_at	0.39	Cas-Br-M (murine) ecotropic retroviral transforming sequence	CBL
202211_at	0.39	ADP-ribosylation factor GTPase activating protein 3	ARFGAP3
204194_at	0.39	BTB and CNC homology 1, basic leucine zipper transcription factor 1	BACH1
205088_at	0.39	chromosome X open reading frame 6	CXorf6
218659_at	0.39	additional sex combs like 2 (Drosophila)	ASXL2
219498_s_at	0.39	B-cell CLL/lymphoma 11A (zinc finger protein)	BCL11A
229264_at	0.39		
244689_at	0.39	peroxisome proliferator-activated receptor alpha	PPARA
219394_at	0.39	phosphatidylglycerophosphate synthase 1	PGS1
218700_s_at	0.39	RAB7, member RAS oncogene family-like 1	RAB7L1
239956_at	0.39		
205110_s_at	0.39	fibroblast growth factor 13	FGF13
221349_at	0.39	pre-B lymphocyte gene 1	VPREB1
213700_s_at	0.39		
240978_at	0.39		
209281_s_at	0.39	ATPase, Ca++ transporting, plasma membrane 1	ATP2B1
1559361_at	0.39		
216260_at	0.39	Dicer1, Dcr-1 homolog (Drosophila)	DICER1
235233_s_at	0.39		
223085_at	0.39	ring finger protein 19	RNF19
243826_at	0.39		
229575_at	0.39		
225005_at	0.39	PHD finger protein 13	PHF13
229242_at	0.39		
216033_s_at	0.39	FYN oncogene related to SRC, FGR, YES	FYN
218344_s_at	0.39	REST corepressor 3	RCOR3
226830_x_at	0.39	chromodomain helicase DNA binding protein 2	CHD2
212295_s_at	0.39	solute carrier family 7 (cationic amino acid transporter, y+ system), member 1	SLC7A1
200985_s_at	0.39	CD59 molecule, complement regulatory protein	CD59
228977_at	0.39		
229551_x_at	0.39		
1568838_at	0.39		
208608_s_at	0.39	syntrophin, beta 1 (dystrophin-associated protein A1, 59kDa, basic component 1)	SNTB1
210210_at	0.39	myelin protein zero-like 1	MPZL1
202193_at	0.39	LIM domain kinase 2	LIMK2
209271_at	0.39	bromodomain PHD finger transcription factor	BPTF
212689_s_at	0.39	jumonji domain containing 1A	JMJD1A
202369_s_at	0.39	translocation associated membrane protein 2	TRAM2
227134_at	0.39	synaptotagmin-like 1	SYTL1
239629_at	0.39	CASP8 and FADD-like apoptosis regulator	CFLAR
202377_at	0.39		
226284_at	0.39	zinc finger and BTB domain containing 2	ZBTB2

211540_s_at	0.39	retinoblastoma 1 (including osteosarcoma)	RB1
225798_at	0.39	JAZF zinc finger 1	JAZF1
1558801_at	0.39		
223377_x_at	0.39	cytokine inducible SH2-containing protein	CISH
209688_s_at	0.39	coiled-coil domain containing 93	CCDC93
204236_at	0.39	Friend leukemia virus integration 1	FLI1
209110_s_at	0.39	ral guanine nucleotide dissociation stimulator-like 2	RGL2
1555789_s_at	0.39	PHD finger protein 23	PHF23
206478_at	0.39	KIAA0125	KIAA0125
224685_at	0.39	myeloid/lymphoid or mixed-lineage leukemia (trithorax homolog, Drosophila); translocated to, 4	MLLT4
207761_s_at	0.39	methyltransferase like 7A	METTL7A
226297_at	0.39	homeodomain interacting protein kinase 3	HIPK3
226551_at	0.39	receptor (TNFRSF)-interacting serine-threonine kinase 1	RIPK1
221695_s_at	0.39	mitogen-activated protein kinase kinase kinase 2	MAP3K2
1553768_a_at	0.39	discoidin, CUB and LCCL domain containing 1	DCBLD1
1552651_a_at	0.39	ring finger and FYVE-like domain containing 1	RFFL
212518_at	0.39	phosphatidylinositol-4-phosphate 5-kinase, type I, gamma	PIP5K1C
218327_s_at	0.39	synaptosomal-associated protein, 29kDa	SNAP29
224912_at	0.39	tetratricopeptide repeat domain 7A	TTC7A
1567703_at	0.39		
227930_at	0.39	eukaryotic translation initiation factor 2C, 4	EIF2C4
201598_s_at	0.39	inositol polyphosphate phosphatase-like 1	INPPL1
226364_at	0.39	huntingtin interacting protein 1	HIP1
228587_at	0.39	family with sequence similarity 83, member G	FAM83G
225935_at	0.39		
1555153_s_at	0.39	FCH domain only 2	FCHO2
215111_s_at	0.39	TSC22 domain family, member 1	TSC22D1
226538_at	0.39	mannosidase, alpha, class 2A, member 1	MAN2A1
220305_at	0.39		
1555854_at	0.39		
227647_at	0.39	potassium voltage-gated channel, Isk-related family, member 3	KCNE3
234023_s_at	0.39	centromere protein J	CENPJ
221905_at	0.39	cylindromatosis (turban tumor syndrome)	CYLD
1555852_at	0.39	proteasome (prosome, macropain) subunit, beta type, 9 (large multifunctional peptidase 2)	PSMB9
237370_at	0.39		
243528_at	0.39		
215047_at	0.39	tripartite motif-containing 58	TRIM58
202956_at	0.39	ADP-ribosylation factor guanine nucleotide-exchange factor 1(brefeldin A-inhibited)	ARFGEF1
217419_x_at	0.39	agrin	AGRIN
244008_at	0.39		
221983_at	0.39	chromosome 2 open reading frame 17	C2orf17
213518_at	0.39	protein kinase C, iota	PRKCI
225478_at	0.39	malignant fibrous histiocytoma amplified sequence 1	MFHAS1
222392_x_at	0.39	PERP, TP53 apoptosis effector	PERP
205510_s_at	0.39		

212468_at	0.39	sperm associated antigen 9	SPAG9
213142_x_at	0.39		
223081_at	0.38	PHD finger protein 23	PHF23
211433_x_at	0.38	KIAA1539	KIAA1539
236728_at	0.38	leucyl/cystinyl aminopeptidase	LNPEP
231579_s_at	0.38	TIMP metallopeptidase inhibitor 2	TIMP2
242268_at	0.38	CUG triplet repeat, RNA binding protein 2	CUGBP2
1558748_at	0.38		
217437_s_at	0.38	transforming, acidic coiled-coil containing protein 1	TACC1
215739_s_at	0.38	tubulin, gamma complex associated protein 3	TUBGCP3
226765_at	0.38	spectrin, beta, non-erythrocytic 1	SPTBN1
225197_at	0.38	chromosome 16 open reading frame 72	C16orf72
225360_at	0.38	TraB domain containing	TRABD
204304_s_at	0.38	prominin 1	PROM1
225970_at	0.38	DDHD domain containing 1	DDHD1
202800_at	0.38	solute carrier family 1 (glial high affinity glutamate transporter), member 3	SLC1A3
206710_s_at	0.38	erythrocyte membrane protein band 4.1-like 3	EPB41L3
1556429_a_at	0.38	WD repeat domain 67	WDR67
244154_at	0.38		
203110_at	0.38	PTK2B protein tyrosine kinase 2 beta	PTK2B
225755_at	0.38	kelch domain containing 8B	KLHDC8B
206341_at	0.38	interleukin 2 receptor, alpha	IL2RA
216159_s_at	0.38		
1557049_at	0.38		
223580_at	0.38	splA/ryanodine receptor domain and SOCS box containing 2	SPSB2
235875_at	0.38		
230248_x_at	0.38		
201851_at	0.38	SH3-domain GRB2-like 1	SH3GL1
244653_at	0.38	SET domain containing (lysine methyltransferase) 7	SETD7
206437_at	0.38	endothelial differentiation, lysophosphatidic acid G-protein-coupled receptor, 6	EDG6
228123_s_at	0.38	abhydrolase domain containing 12	ABHD12
207978_s_at	0.38	nuclear receptor subfamily 4, group A, member 3	NR4A3
242292_at	0.38		
202688_at	0.38	tumor necrosis factor (ligand) superfamily, member 10	TNFSF10
225791_at	0.38	ubiquitin-conjugating enzyme E2F (putative)	UBE2F
221867_at	0.38		
209710_at	0.38	GATA binding protein 2	GATA2
217315_s_at	0.38	kallikrein-related peptidase 13	KLK13
244757_at	0.38	cytochrome P450, family 2, subfamily R, polypeptide 1	CYP2R1
223944_at	0.38	NLR family, pyrin domain containing 12	NLRP12
219905_at	0.38	erythroblast membrane-associated protein (Scianna blood group)	ERMAP
1552516_a_at	0.38	homeodomain interacting protein kinase 1	HIPK1
208485_x_at	0.38	CASP8 and FADD-like apoptosis regulator	CFLAR
224939_at	0.38		
1558426_x_at	0.38	transmembrane protein 142B	TMEM142B

201215_at	0.38	plastin 3 (T isoform)	PLS3
213262_at	0.38	spastic ataxia of Charlevoix-Saguenay (sacsin)	SACS
227590_at	0.38		
212912_at	0.38	ribosomal protein S6 kinase, 90kDa, polypeptide 2	RPS6KA2
225711_at	0.38	ADP-ribosylation-like factor 6 interacting protein 6	ARL6IP6
1555705_a_at	0.38	CKLF-like MARVEL transmembrane domain containing 3	CMTM3
225959_s_at	0.38	zinc and ring finger 1	ZNRF1
210544_s_at	0.38	aldehyde dehydrogenase 3 family, member A2	ALDH3A2
224641_at	0.38	forty-two-three domain containing 1	FYTTD1
212274_at	0.38	lipin 1	LPIN1
204249_s_at	0.38	LIM domain only 2 (rhombotin-like 1)	LMO2
32128_at	0.38	chemokine (C-C motif) ligand 18 (pulmonary and activation-regulated)	CCL18
202221_s_at	0.38	E1A binding protein p300	EP300
233880_at	0.38	ring finger protein 213	RNF213
211763_s_at	0.38	ubiquitin-conjugating enzyme E2B (RAD6 homolog)	UBE2B
218871_x_at	0.38		
224924_at	0.38	tetratricopeptide repeat domain 7A	TTC7A
226638_at	0.38	Rho GTPase activating protein 23	ARHGAP23
225347_at	0.38	ADP-ribosylation factor-like 8A	ARL8A
205549_at	0.38	Purkinje cell protein 4	PCP4
204549_at	0.38	inhibitor of kappa light polypeptide gene enhancer in B-cells, kinase epsilon	IKBKE
204671_s_at	0.38	ankyrin repeat domain 6	ANKRD6
219183_s_at	0.38	pleckstrin homology, Sec7 and coiled-coil domains 4	PSCD4
217680_x_at	0.38		
225140_at	0.38	Kruppel-like factor 3 (basic)	KLF3
235024_at	0.38	PHD finger protein 17	PHF17
221920_s_at	0.38	solute carrier family 25, member 37	SLC25A37
210835_s_at	0.38	C-terminal binding protein 2	CTBP2
223227_at	0.38	Bardet-Biedl syndrome 2	BBS2
233589_x_at	0.38		
221551_x_at	0.38	ST6 (alpha-N-acetyl-neuraminyl-2,3-beta-galactosyl-1,3)-N-acetylgalactosaminide alpha-2,6-sialyltran	ST6GALNAC4
204667_at	0.38	forkhead box A1	FOXA1
224336_s_at	0.38	dual specificity phosphatase 16	DUSP16
203470_s_at	0.38	pleckstrin	PLEK
205290_s_at	0.38	bone morphogenetic protein 2	BMP2
227761_at	0.38	myosin VA (heavy chain 12, myoxin)	MYO5A
212308_at	0.38	cytoplasmic linker associated protein 2	CLASP2
202694_at	0.38	serine/threonine kinase 17a (apoptosis-inducing)	STK17A
209250_at	0.38	degenerative spermatocyte homolog 1, lipid desaturase (Drosophila)	DEGS1
244578_at	0.38	lymphocyte cytosolic protein 2 (SH2 domain containing leukocyte protein of 76kDa)	LCP2
226789_at	0.38		
204619_s_at	0.38	chondroitin sulfate proteoglycan 2 (versican)	CSPG2

204928_s_at	0.38	solute carrier family 10 (sodium/bile acid cotransporter family), member 3	SLC10A3
202082_s_at	0.38	SEC14-like 1 (S. cerevisiae)	SEC14L1
219892_at	0.38	transmembrane 6 superfamily member 1	TM6SF1
226689_at	0.38	zinc finger, CDGSH-type domain 2	ZCD2
226810_at	0.38		
211671_s_at	0.38	nuclear receptor subfamily 3, group C, member 1 (glucocorticoid receptor)	NR3C1
229507_at	0.38	chromosome 3 open reading frame 54	C3orf54
240481_at	0.38	TRAF3 interacting protein 3	TRAF3IP3
201126_s_at	0.38	mannosyl (alpha-1,3-)-glycoprotein beta-1,2-N-acetylglucosaminyltransferase	MGAT1
219636_s_at	0.38	armadillo repeat containing 9	ARMC9
229017_s_at	0.38	receptor interacting protein kinase 5	RIPK5
206348_s_at	0.38	pyruvate dehydrogenase kinase, isozyme 3	PDK3
225118_at	0.38	SET domain containing (lysine methyltransferase) 8	SETD8
1558496_at	0.38		
218815_s_at	0.38	transmembrane protein 51	TMEM51
215486_at	0.38	phosphoribosyl pyrophosphate synthetase 1-like 1	PRPS1L1
204527_at	0.38	myosin VA (heavy chain 12, myoxin)	MYO5A
219150_s_at	0.38	centaurin, alpha 1	CENTA1
226125_at	0.38		
229968_at	0.38		
1559051_s_at	0.38	chromosome 6 open reading frame 150	C6orf150
238940_at	0.38	Kruppel-like factor 12	KLF12
219316_s_at	0.38	chromosome 14 open reading frame 58	C14orf58
230263_s_at	0.38	dedicator of cytokinesis 5	DOCK5
216268_s_at	0.38	jagged 1 (Alagille syndrome)	JAG1
242157_at	0.38	chromodomain helicase DNA binding protein 9	CHD9
229492_at	0.38	vang-like 1 (van gogh, Drosophila)	VANGL1
226542_at	0.38		
242366_at	0.37		
1554414_a_at	0.37	oxidative stress induced growth inhibitor family member 2	OSGIN2
239434_at	0.37		
226299_at	0.37	protein kinase N3	PKN3
218456_at	0.37	C1q domain containing 1	C1QDC1
228226_s_at	0.37	zinc finger protein 775	ZNF775
224783_at	0.37	family with sequence similarity 100, member B	FAM100B
237332_at	0.37		
208092_s_at	0.37	family with sequence similarity 49, member A	FAM49A
203378_at	0.37	PCF11, cleavage and polyadenylation factor subunit, homolog (S. cerevisiae)	PCF11
220035_at	0.37	nucleoporin 210kDa	NUP210
225999_at	0.37	family with sequence similarity 80, member B	FAM80B
236192_at	0.37		
215716_s_at	0.37	ATPase, Ca++ transporting, plasma membrane 1	ATP2B1
1557285_at	0.37		
222221_x_at	0.37	EH-domain containing 1	EHD1
207798_s_at	0.37	ataxin 2-like	ATXN2L

235163_at	0.37	MOB1, Mps One Binder kinase activator-like 2A (yeast)	MOBKL2A
219471_at	0.37	chromosome 13 open reading frame 18	C13orf18
242946_at	0.37	CD53 molecule	CD53
208875_s_at	0.37	p21 (CDKN1A)-activated kinase 2	PAK2
226982_at	0.37	elongation factor, RNA polymerase II, 2	ELL2
200816_s_at	0.37	platelet-activating factor acetylhydrolase, isoform Ib, alpha subunit 45kDa	PAFAH1B1
208854_s_at	0.37	serine/threonine kinase 24 (STE20 homolog, yeast)	STK24
240405_at	0.37		
225091_at	0.37	SRY (sex determining region Y)-box 12#tribbles homolog 3 (Drosophila)#neurensin 2#zinc finger, CCHC domain containing 3#zinc finger, CCHC domain containing 3#chromosome 20 open reading frame 96#null	SOX12#TRIB3#NRSN2#ZCCHC3#ZCCHC3#C20orf96#null
1560495_at	0.37		
227531_at	0.37		
222780_s_at	0.37	brain and acute leukemia, cytoplasmic	BAALC
203881_s_at	0.37	dystrophin (muscular dystrophy, Duchenne and Becker types)	DMD
213624_at	0.37	sphingomyelin phosphodiesterase, acid-like 3A	SMPDL3A
222233_s_at	0.37	DNA cross-link repair 1C (PSO2 homolog, S. cerevisiae)	DCLRE1C
242790_at	0.37		
204803_s_at	0.37	Ras-related associated with diabetes	RRAD
210365_at	0.37	runt-related transcription factor 1 (acute myeloid leukemia 1; aml1 oncogene)	RUNX1
226738_at	0.37	WD repeat domain 81	WDR81
225234_at	0.37	Cas-Br-M (murine) ecotropic retroviral transforming sequence	CBL
226040_at	0.37		
222891_s_at	0.37	B-cell CLL/lymphoma 11A (zinc finger protein)	BCL11A
221984_s_at	0.37	chromosome 2 open reading frame 17	C2orf17
206148_at	0.37	interleukin 3 receptor, alpha (low affinity)	IL3RA
228088_at	0.37		
229167_at	0.37		
211776_s_at	0.37	erythrocyte membrane protein band 4.1-like 3	EPB41L3
217432_s_at	0.37	iduronate 2-sulfatase (Hunter syndrome)	IDS
235384_at	0.37		
226372_at	0.37	carbohydrate (chondroitin 4) sulfotransferase 11	CHST11
204047_s_at	0.37	phosphatase and actin regulator 2	PHACTR2
214486_x_at	0.37	CASP8 and FADD-like apoptosis regulator	CFLAR
213116_at	0.37	NIMA (never in mitosis gene a)-related kinase 3	NEK3
214657_s_at	0.37		
226475_at	0.37	family with sequence similarity 118, member A	FAM118A
217226_s_at	0.37	sideroflexin 3	SFXN3
1554612_at	0.37	KIAA0226	KIAA0226
1554821_a_at	0.37	zinc finger, BED-type containing 1	ZBED1
227014_at	0.37	aspartate beta-hydroxylase domain containing 2	ASPHD2
243395_at	0.37		
214606_at	0.37	tetraspanin 2	TSPAN2
204131_s_at	0.37	forkhead box O3A	FOXO3A

217897_at	0.37	FXYD domain containing ion transport regulator 6	FXYD6
201581_at	0.37	thioredoxin domain containing 13	TXNDC13
202084_s_at	0.37	SEC14-like 1 (S. cerevisiae)	SEC14L1
201319_at	0.37		
1556589_at	0.37		
231244_at	0.37	CAS1 domain containing 1	CASD1
230424_at	0.37	chromosome 5 open reading frame 13	C5orf13
205013_s_at	0.37	adenosine A2a receptor	ADORA2A
229872_s_at	0.37		
213327_s_at	0.37	ubiquitin specific peptidase 12	USP12
201431_s_at	0.37	dihydropyrimidinase-like 3	DPYSL3
1570259_at	0.37	LIM and senescent cell antigen-like domains 1	LIMS1
228708_at	0.37		
224374_s_at	0.37	elastin microfibril interfacer 2	EMILIN2
225306_s_at	0.37	solute carrier family 25, member 29	SLC25A29
200813_s_at	0.37	platelet-activating factor acetylhydrolase, isoform Ib, alpha subunit 45kDa	PAFAH1B1
1552343_s_at	0.37	phosphodiesterase 7A	PDE7A
203822_s_at	0.37	E74-like factor 2 (ets domain transcription factor)	ELF2
236961_at	0.37		
204702_s_at	0.37	nuclear factor (erythroid-derived 2)-like 3	NFE2L3
228037_at	0.37		
201883_s_at	0.37	UDP-Gal:betaGlcNAc beta 1,4-galactosyltransferase, polypeptide 1	B4GALT1
222858_s_at	0.37	dual adaptor of phosphotyrosine and 3-phosphoinositides	DAPP1
226977_at	0.37		
203017_s_at	0.37	synovial sarcoma, X breakpoint 2 interacting protein	SSX2IP
238948_at	0.37	transmembrane 9 superfamily member 1	TM9SF1
205921_s_at	0.36	solute carrier family 6 (neurotransmitter transporter, taurine), member 6	SLC6A6
202284_s_at	0.36	cyclin-dependent kinase inhibitor 1A (p21, Cip1)	CDKN1A
203869_at	0.36	ubiquitin specific peptidase 46	USP46
1557905_s_at	0.36	CD44 molecule (Indian blood group)	CD44
202734_at	0.36	thyroid hormone receptor interactor 10	TRIP10
217995_at	0.36	sulfide quinone reductase-like (yeast)	SQRDL
206874_s_at	0.36	collagen, type XVII, alpha 1#STE20-like kinase (yeast)	COL17A1#SLK
226869_at	0.36		
227279_at	0.36	transcription elongation factor A (SII)-like 3	TCEAL3
224469_s_at	0.36	chromosome 14 open reading frame 151	C14orf151
212252_at	0.36	calcium/calmodulin-dependent protein kinase kinase 2, beta	CAMKK2
226875_at	0.36	dedicator of cytokinesis 11	DOCK11
232350_x_at	0.36	G protein-coupled receptor 161	GPR161
205139_s_at	0.36	uronyl-2-sulfotransferase	UST
226474_at	0.36	NLR family, CARD domain containing 5	NLRC5
201353_s_at	0.36	bromodomain adjacent to zinc finger domain, 2A	BAZ2A
227440_at	0.36	ankyrin repeat and sterile alpha motif domain containing 1B	ANKS1B
226485_at	0.36		
212314_at	0.36		

203704_s_at	0.36	ras responsive element binding protein 1	RREB1
218332_at	0.36	brain expressed, X-linked 1	BEX1
241567_at	0.36	nucleolar protein 4	NOL4
230604_at	0.36		
210841_s_at	0.36	neuropilin 2	NRP2
1555781_at	0.36	PQ loop repeat containing 2	PQLC2
564_at	0.36	guanine nucleotide binding protein (G protein), alpha 11 (Gq class)	GNA11
205594_at	0.36	zinc finger protein 652	ZNF652
205349_at	0.36	guanine nucleotide binding protein (G protein), alpha 15 (Gq class)	GNA15
213954_at	0.36		
205705_at	0.36	ankyrin repeat domain 26	ANKRD26
209039_x_at	0.36	EH-domain containing 1	EHD1
231884_at	0.36	centrobin, centrosomal BRCA2 interacting protein	CNTROB
239364_at	0.36	ets variant gene 6 (TEL oncogene)	ETV6
225018_at	0.36	spire homolog 1 (Drosophila)	SPIRE1
210360_s_at	0.36	metastasis suppressor 1	MTSS1
224747_at	0.36	ubiquitin-conjugating enzyme E2Q (putative) 2	UBE2Q2
229295_at	0.36		
212355_at	0.36	KIAA0323	KIAA0323
222108_at	0.36	adhesion molecule with Ig-like domain 2	AMIGO2
227406_at	0.36	GA binding protein transcription factor, beta subunit 2	GABPB2
244082_at	0.36		
231899_at	0.36	zinc finger CCCH-type containing 12C	ZC3H12C
203870_at	0.36	ubiquitin specific peptidase 46	USP46
203373_at	0.36	suppressor of cytokine signaling 2	SOCS2
232615_at	0.36		
202259_s_at	0.36		
203385_at	0.36	diacylglycerol kinase, alpha 80kDa	DGKA
222802_at	0.36	endothelin 1	EDN1
202370_s_at	0.36	core-binding factor, beta subunit	CBFB
225048_at	0.36	PHD finger protein 10	PHF10
227013_at	0.36	LATS, large tumor suppressor, homolog 2 (Drosophila)	LATS2
204163_at	0.36	elastin microfibril interfacer 1	EMILIN1
226205_at	0.36	ankyrin repeat domain 13 family, member D	ANKRD13D
210895_s_at	0.36	CD86 molecule	CD86
206181_at	0.36	signaling lymphocytic activation molecule family member 1	SLAMF1
229429_x_at	0.36		
213639_s_at	0.36	zinc finger protein 500	ZNF500
203445_s_at	0.36	CTD (carboxy-terminal domain, RNA polymerase II, polypeptide A) small phosphatase 2	CTDSP2
204994_at	0.36	myxovirus (influenza virus) resistance 2 (mouse)	MX2
213940_s_at	0.36	formin binding protein 1	FNBP1
227954_at	0.36		
226215_s_at	0.36	F-box and leucine-rich repeat protein 10	FBXL10
210754_s_at	0.36	v-yes-1 Yamaguchi sarcoma viral related oncogene homolog	LYN
200628_s_at	0.36	tryptophanyl-tRNA synthetase	WARS
210174_at	0.36	nuclear receptor subfamily 5, group A, member 2	NR5A2

203887_s_at	0.36	thrombomodulin	THBD
244520_at	0.36		
202724_s_at	0.36	forkhead box O1A (rhabdomyosarcoma)	FOXO1A
235146_at	0.36		
209325_s_at	0.36	regulator of G-protein signalling 16	RGS16
204866_at	0.36	PHD finger protein 16	PHF16
204714_s_at	0.36	coagulation factor V (proaccelerin, labile factor)	F5
61297_at	0.36	CASK interacting protein 2	CASKIN2
210745_at	0.36	one cut domain, family member 1	ONECUT1
210105_s_at	0.36	FYN oncogene related to SRC, FGR, YES	FYN
1559964_at	0.36		
225658_at	0.36		
201849_at	0.36	BCL2/adenovirus E1B 19kDa interacting protein 3	BNIP3
223046_at	0.36	glyceronephosphate O-acyltransferase#egl nine homolog 1 (C. elegans)#chromosome 1 open reading frame 124#exocyst complex component 8	GNPAT#EGLN1#C1orf124#EXOC8
222746_s_at	0.36	B-box and SPRY domain containing	BSPRY
228991_at	0.36		
235478_at	0.36	DNA cross-link repair 1C (PSO2 homolog, S. cerevisiae)	DCLRE1C
220557_s_at	0.36	phosphofurin acidic cluster sorting protein 1	PACS1
208763_s_at	0.36	TSC22 domain family, member 3	TSC22D3
222079_at	0.36		
244229_at	0.36	parvin, gamma	PARVG
219637_at	0.36	armadillo repeat containing 9	ARMC9
235521_at	0.36	homeobox A3	HOXA3
227728_at	0.36		
205409_at	0.36	FOS-like antigen 2	FOSL2
203796_s_at	0.36	B-cell CLL/lymphoma 7A	BCL7A
212589_at	0.36	related RAS viral (r-ras) oncogene homolog 2	RRAS2
224465_s_at	0.36	within bgcn homolog (Drosophila)	WIBG
223380_s_at	0.36	LATS, large tumor suppressor, homolog 2 (Drosophila)	LATS2
228570_at	0.36	BTB (POZ) domain containing 11	BTBD11
200660_at	0.36	S100 calcium binding protein A11	S100A11
201965_s_at	0.36	senataxin	SETX
220313_at	0.36	G protein-coupled receptor 88	GPR88
41386_i_at	0.35	jumonji domain containing 3	JMJD3
211019_s_at	0.35	lanosterol synthase (2,3-oxidosqualene-lanosterol cyclase)	LSS
233952_s_at	0.35	zinc finger protein 295	ZNF295
222923_s_at	0.35	potassium voltage-gated channel, Isk-related family, member 3	KCNE3
237161_at	0.35		
209420_s_at	0.35	sphingomyelin phosphodiesterase 1, acid lysosomal (acid sphingomyelinase)	SMPD1
202350_s_at	0.35	matrilin 2	MATN2
218175_at	0.35	coiled-coil domain containing 92	CCDC92
229741_at	0.35		
222529_at	0.35	solute carrier family 25, member 37	SLC25A37
230961_at	0.35		

207102_at	0.35	aldo-keto reductase family 1, member D1 (delta 4-3-ketosteroid-5-beta-reductase)	AKR1D1
226212_s_at	0.35		
237999_at	0.35	zinc finger, DHHC-type containing 13	ZDHHC13
1566647_s_at	0.35		
212609_s_at	0.35	v-akt murine thymoma viral oncogene homolog 3 (protein kinase B, gamma)	AKT3
1560743_a_at	0.35		
222627_at	0.35	vacuolar protein sorting 54 homolog (S. cerevisiae)	VPS54
228193_s_at	0.35		
214916_x_at	0.35	interleukin 8	IL8
235430_at	0.35	chromosome 14 open reading frame 43	C14orf43
212586_at	0.35	calpastatin	CAST
243931_at	0.35	CD58 molecule	CD58
225937_at	0.35		
214449_s_at	0.35	ras homolog gene family, member Q	RHOQ
232937_at	0.35		
202860_at	0.35	DENN/MADD domain containing 4B	DENND4B
212651_at	0.35	Rho-related BTB domain containing 1	RHOBTB1
226901_at	0.35	chromosome 17 open reading frame 58	C17orf58
226676_at	0.35	zinc finger protein 521	ZNF521
1557632_at	0.35		
1563224_at	0.35		
205193_at	0.35	v-maf musculoaponeurotic fibrosarcoma oncogene homolog F (avian)	MAFF
1561153_at	0.35		
226392_at	0.35		
224473_x_at	0.35	leucine zipper, putative tumor suppressor 2	LZTS2
1566720_at	0.35		
230820_at	0.35	SMAD specific E3 ubiquitin protein ligase 2	SMURF2
240363_at	0.35	ankyrin 1, erythrocytic	ANK1
221799_at	0.35		
238622_at	0.35	RAP2B, member of RAS oncogene family	RAP2B
209678_s_at	0.35	protein kinase C, iota	PRKCI
205841_at	0.35	Janus kinase 2 (a protein tyrosine kinase)	JAK2
214505_s_at	0.35	four and a half LIM domains 1	FHL1
240372_at	0.35		
231274_s_at	0.35	solute carrier family 25, member 37	SLC25A37
209286_at	0.35	CDC42 effector protein (Rho GTPase binding) 3	CDC42EP3
1558613_at	0.35		
222303_at	0.35		
235766_x_at	0.35	RAB27A, member RAS oncogene family	RAB27A
211862_x_at	0.35	CASP8 and FADD-like apoptosis regulator	CFLAR
209272_at	0.35	NGFI-A binding protein 1 (EGR1 binding protein 1)	NAB1
217730_at	0.35	transmembrane BAX inhibitor motif containing 1	TMBIM1
241803_s_at	0.35		
1553647_at	0.35	chromodomain protein, Y-like 2	CDYL2
230546_at	0.35	vasohibin 1	VASH1
232690_at	0.35		
212473_s_at	0.35	microtubule associated monoxygenase, calponin and LIM domain containing 2	MICAL2

229897_at	0.35	zinc finger protein 641	ZNF641
205887_x_at	0.35	mutS homolog 3 (E. coli)	MSH3
224765_at	0.35		
204993_at	0.35	guanine nucleotide binding protein (G protein), alpha z polypeptide	GNAZ
244592_at	0.35	Friend leukemia virus integration 1	FLI1
206060_s_at	0.35	protein tyrosine phosphatase, non-receptor type 22 (lymphoid)	PTPN22
215188_at	0.35	serine/threonine kinase 24 (STE20 homolog, yeast)	STK24
238649_at	0.35	phosphatidylinositol transfer protein, cytoplasmic 1	PITPNC1
227811_at	0.35	FYVE, RhoGEF and PH domain containing 3	FGD3
209875_s_at	0.35	secreted phosphoprotein 1 (osteopontin, bone sialoprotein I, early T-lymphocyte activation 1)	SPP1
204484_at	0.35	phosphoinositide-3-kinase, class 2, beta polypeptide	PIK3C2B
202100_at	0.35	v-ral simian leukemia viral oncogene homolog B (ras related; GTP binding protein)	RALB
204021_s_at	0.35	purine-rich element binding protein A	PURA
222810_s_at	0.35	RAS protein activator like 2	RASAL2
223358_s_at	0.35		
214617_at	0.35	perforin 1 (pore forming protein)	PRF1
207992_s_at	0.35	adenosine monophosphate deaminase (isoform E)	AMPD3
223398_at	0.35	chromosome 9 open reading frame 89	C9orf89
236892_s_at	0.35		
203729_at	0.35	epithelial membrane protein 3	EMP3
223325_at	0.35	thioredoxin domain containing 11	TXNDC11
226796_at	0.35		
223125_s_at	0.35	chromosome 1 open reading frame 21	C1orf21
1570165_at	0.35		
239979_at	0.35	epithelial stromal interaction 1 (breast)	EPSTI1
236199_at	0.35	arachidonate 5-lipoxygenase	ALOX5
232080_at	0.35	HECT, C2 and WW domain containing E3 ubiquitin protein ligase 2	HECW2
1559377_at	0.35		
213620_s_at	0.35	intercellular adhesion molecule 2	ICAM2
1556474_a_at	0.35		
219365_s_at	0.35	CaM kinase-like vesicle-associated	CAMKV
201328_at	0.35	v-ets erythroblastosis virus E26 oncogene homolog 2 (avian)	ETS2
226715_at	0.35	forkhead box K1	FOXK1
1557458_s_at	0.35	Src homology 2 domain containing adaptor protein B	SHB
205391_x_at	0.35	ankyrin 1, erythrocytic	ANK1
209392_at	0.35	ectonucleotide pyrophosphatase/phosphodiesterase 2 (autotaxin)	ENPP2
224891_at	0.35	forkhead box O3A	FOXO3A
219190_s_at	0.35	eukaryotic translation initiation factor 2C, 4	EIF2C4
210305_at	0.35	phosphodiesterase 4D interacting protein (myomegalin)	PDE4DIP

214632_at	0.35	neuropilin 2	NRP2
218066_at	0.35	solute carrier family 12 (potassium/chloride transporters), member 7	SLC12A7
215630_at	0.35		
241087_at	0.35		
207949_s_at	0.35	islet cell autoantigen 1, 69kDa	ICA1
205541_s_at	0.35	G1 to S phase transition 2	GSPT2
227232_at	0.35	Enah/Vasp-like	EVL
213968_at	0.35	tetraspanin 5	TSPAN5
209106_at	0.35	nuclear receptor coactivator 1	NCOA1
218927_s_at	0.34	carbohydrate (chondroitin 4) sulfotransferase 12	CHST12
221245_s_at	0.34	frizzled homolog 5 (Drosophila)	FZD5
218626_at	0.34	eukaryotic translation initiation factor 4E nuclear import factor 1	EIF4ENIF1
209487_at	0.34	RNA binding protein with multiple splicing	RBPMS
227106_at	0.34		
230972_at	0.34	ankyrin repeat domain 9	ANKRD9
224958_at	0.34	nuclear fragile X mental retardation protein interacting protein 2	NUFIP2
203827_at	0.34	WD repeat domain, phosphoinositide interacting 1	WIPI1
208816_x_at	0.34	annexin A2 pseudogene 2	ANXA2P2
206411_s_at	0.34	v-abl Abelson murine leukemia viral oncogene homolog 2 (arg, Abelson-related gene)	ABL2
228503_at	0.34		
201861_s_at	0.34	leucine rich repeat (in FLII) interacting protein 1	LRRFIP1
1557394_at	0.34		
225653_at	0.34		
221011_s_at	0.34	limb bud and heart development homolog (mouse)	LBH
212818_s_at	0.34	ankyrin repeat and SOCS box-containing 1	ASB1
241873_at	0.34		
225355_at	0.34		
230543_at	0.34	ubiquitin specific peptidase 9, X-linked	USP9X
207275_s_at	0.34	acyl-CoA synthetase long-chain family member 1	ACSL1
210874_s_at	0.34	N-acetyltransferase 6	NAT6
1553043_a_at	0.34	CD300 molecule-like family member f	CD300LF
239246_at	0.34	FERM, RhoGEF (ARHGEF) and pleckstrin domain protein 1 (chondrocyte-derived)	FARP1
211596_s_at	0.34	leucine-rich repeats and immunoglobulin-like domains 1	LRIG1
212764_at	0.34		
213293_s_at	0.34	tripartite motif-containing 22	TRIM22
201354_s_at	0.34	bromodomain adjacent to zinc finger domain, 2A	BAZ2A
40359_at	0.34	Ras association (RalGDS/AF-6) domain family 7	RASSF7
218798_at	0.34		
230748_at	0.34		
241857_at	0.34		
231403_at	0.34	triple functional domain (PTPRF interacting)	TRIO
209488_s_at	0.34	RNA binding protein with multiple splicing	RBPMS
1558217_at	0.34	schlafen family member 13	SLFN13
223586_at	0.34	aryl hydrocarbon receptor nuclear translocator-like 2	ARNTL2

201811_x_at	0.34	SH3-domain binding protein 5 (BTK-associated)	SH3BP5
202626_s_at	0.34	v-yes-1 Yamaguchi sarcoma viral related oncogene homolog	LYN
226545_at	0.34	CD109 molecule	CD109
204268_at	0.34	S100 calcium binding protein A2	S100A2
235086_at	0.34	thrombospondin 1	THBS1
209305_s_at	0.34	growth arrest and DNA-damage-inducible, beta	GADD45B
218627_at	0.34		
218641_at	0.34		
241692_at	0.34		
218041_x_at	0.34	solute carrier family 38, member 2	SLC38A2
203600_s_at	0.34	chromosome 4 open reading frame 8	C4orf8
205345_at	0.34	BRCA1 associated RING domain 1	BARD1
205270_s_at	0.34	lymphocyte cytosolic protein 2 (SH2 domain containing leukocyte protein of 76kDa)	LCP2
203542_s_at	0.34	Kruppel-like factor 9	KLF9
227408_s_at	0.34	sorting nexin 25	SNX25
238917_s_at	0.34		
200911_s_at	0.34	transforming, acidic coiled-coil containing protein 1	TACC1
211061_s_at	0.34	mannosyl (alpha-1,6-)-glycoprotein beta-1,2-N-acetylglucosaminyltransferase	MGAT2
1555058_a_at	0.34	lysophosphatidylglycerol acyltransferase 1	LPGAT1
202804_at	0.34	ATP-binding cassette, sub-family C (CFTR/MRP), member 1	ABCC1
225116_at	0.34	homeodomain interacting protein kinase 2	HIPK2
227897_at	0.34	RAP2B, member of RAS oncogene family	RAP2B
204542_at	0.34	ST6 (alpha-N-acetyl-neuraminyl-2,3-beta-galactosyl-1,3)-N-acetylgalactosaminide alpha-2,6-sialyltran	ST6GALNAC2
231907_at	0.34		
228706_s_at	0.34	claudin 23	CLDN23
221523_s_at	0.34	ankyrin repeat domain 6#ubiquitin-conjugating enzyme E2, J1 (UBC6 homolog, yeast)#Ras-related GTP binding D	ANKRD6#UBE2J1#RRAGD
242586_at	0.34	fibronectin type III and SPRY domain containing 1-like	FSD1L
210123_s_at	0.34	cholinergic receptor, nicotinic, alpha 7	CHRNA7
235841_at	0.34		
235109_at	0.34		
1558342_x_at	0.34	DIX domain containing 1	DIXDC1
227851_s_at	0.34		
226721_at	0.34	dpy-19-like 4 (C. elegans)	DPY19L4
225768_at	0.34	nuclear receptor subfamily 1, group D, member 2	NR1D2
1552733_at	0.34	kelch domain containing 1	KLHDC1
202847_at	0.34	phosphoenolpyruvate carboxykinase 2 (mitochondrial)	PCK2
224481_s_at	0.34	HECT domain containing 1	HECTD1
209920_at	0.34	bone morphogenetic protein receptor, type II (serine/threonine kinase)	BMPR2
236471_at	0.34	nuclear factor (erythroid-derived 2)-like 3	NFE2L3
208908_s_at	0.34	calpastatin	CAST
229900_at	0.34	CD109 molecule	CD109

213888_s_at	0.34	adenosine A2b receptor pseudogene#hydroxysteroid (11-beta) dehydrogenase 1#interferon regulatory factor 6#chromosome 1 open reading frame 107#TRAF3 interacting protein 3#chromosome 1 open reading frame 74	ADORA2BP#HSD11B1#IRF6#C1orf107#TRAF3IP3#C1orf74
232039_at	0.34	KIAA1383	KIAA1383
244286_at	0.34	formin binding protein 1	FNBP1
216925_s_at	0.34	T-cell acute lymphocytic leukemia 1	TAL1
212063_at	0.34	CD44 molecule (Indian blood group)	CD44
228702_at	0.34		
223216_x_at	0.34	zinc finger protein 395	ZNF395
1561677_at	0.34	chromosome 20 open reading frame 133	C20orf133
210191_s_at	0.34	putative homeodomain transcription factor 1	PHTF1
227489_at	0.34	SMAD specific E3 ubiquitin protein ligase 2	SMURF2
229274_at	0.34	GNAS complex locus	GNAS
242277_at	0.34		
1555978_s_at	0.34		
223276_at	0.34		
203962_s_at	0.34	nebulette	NEBL
218648_at	0.34	CREB regulated transcription coactivator 3	CRTC3
232309_at	0.33		
1556761_at	0.33		
206589_at	0.33	growth factor independent 1	GFI1
208018_s_at	0.33	hemopoietic cell kinase	HCK
201566_x_at	0.33	inhibitor of DNA binding 2, dominant negative helix-loop-helix protein	ID2
205540_s_at	0.33	Ras-related GTP binding B	RRAGB
224091_at	0.33		
209750_at	0.33	nuclear receptor subfamily 1, group D, member 2	NR1D2
226441_at	0.33		
223967_at	0.33	angiopoietin-like 6	ANGPTL6
225486_at	0.33	AT rich interactive domain 2 (ARID, RFX-like)	ARID2
222116_s_at	0.33	TBC1 domain family, member 16	TBC1D16
206510_at	0.33	sine oculis homeobox homolog 2 (Drosophila)	SIX2
219039_at	0.33	sema domain, immunoglobulin domain (Ig), transmembrane domain (TM) and short cytoplasmic domain, (se	SEMA4C
203278_s_at	0.33	PHD finger protein 21A	PHF21A
236126_at	0.33	activin A receptor, type IIB	ACVR2B
242794_at	0.33	mastermind-like 3 (Drosophila)	MAML3
206176_at	0.33	bone morphogenetic protein 6	BMP6
231697_s_at	0.33	transmembrane protein 49	TMEM49
238001_at	0.33	potassium channel tetramerisation domain containing 6	KCTD6
222982_x_at	0.33	solute carrier family 38, member 2	SLC38A2
225965_at	0.33	DDHD domain containing 1	DDHD1
201100_s_at	0.33	ubiquitin specific peptidase 9, X-linked	USP9X
203925_at	0.33	glutamate-cysteine ligase, modifier subunit	GCLM
1569525_s_at	0.33		
1562587_at	0.33		
231484_at	0.33		

201670_s_at	0.33	myristoylated alanine-rich protein kinase C substrate	MARCKS
210839_s_at	0.33	ectonucleotide pyrophosphatase/phosphodiesterase 2 (autotaxin)	ENPP2
233167_at	0.33		
238015_at	0.33		
1552258_at	0.33		
219279_at	0.33	dedicator of cytokinesis 10	DOCK10
202449_s_at	0.33	retinoid X receptor, alpha	RXRA
224880_at	0.33	v-ral simian leukemia viral oncogene homolog A (ras related)	RALA
205801_s_at	0.33	RAS guanyl releasing protein 3 (calcium and DAG-regulated)	RASGRP3
221223_x_at	0.33	cytokine inducible SH2-containing protein	CISH
203369_x_at	0.33	PDZ and LIM domain 7 (enigma)	PDLIM7
201862_s_at	0.33	leucine rich repeat (in FLII) interacting protein 1	LRRFIP1
225789_at	0.33	centaurin, gamma 3	CENTG3
213375_s_at	0.33		
210504_at	0.33	Kruppel-like factor 1 (erythroid)	KLF1
244023_at	0.33	spleen tyrosine kinase	SYK
212276_at	0.33	lipin 1	LPIN1
220416_at	0.33	ATPase, Class I, type 8B, member 4	ATP8B4
218301_at	0.33	arginyl aminopeptidase (aminopeptidase B)-like 1	RNPEPL1
218262_at	0.33	required for meiotic nuclear division 5 homolog B (S. cerevisiae)	RMND5B
226677_at	0.33	zinc finger protein 521	ZNF521
228032_s_at	0.33		
237943_at	0.33	transmembrane and coiled-coil domain family 1	TMCC1
1556147_at	0.33		
227626_at	0.33	progestin and adipoQ receptor family member VIII	PAQR8
218845_at	0.33	dual specificity phosphatase 22	DUSP22
242054_s_at	0.33		
225527_at	0.33	CCAAT/enhancer binding protein (C/EBP), gamma	CEBPG
209627_s_at	0.33	oxysterol binding protein-like 3	OSBPL3
210039_s_at	0.33	protein kinase C, theta	PRKCQ
1556590_s_at	0.33		
239448_at	0.33		
216321_s_at	0.33	nuclear receptor subfamily 3, group C, member 1 (glucocorticoid receptor)	NR3C1
239442_at	0.33	centrosomal protein 68kDa	CEP68
201865_x_at	0.33		
1553155_x_at	0.33	ATPase, H+ transporting, lysosomal 38kDa, V0 subunit d2	ATP6V0D2
203773_x_at	0.33	biliverdin reductase A	BLVRA
1562894_at	0.33		
223852_s_at	0.33	serine/threonine kinase 40	STK40
204927_at	0.33	Ras association (RalGDS/AF-6) domain family 7	RASSF7
228975_at	0.33	Sp6 transcription factor	SP6
206513_at	0.33	absent in melanoma 2	AIM2
204520_x_at	0.33	bromodomain containing 1	BRD1

202111_at	0.33	solute carrier family 4, anion exchanger, member 2 (erythrocyte membrane protein band 3-like 1)	SLC4A2
201099_at	0.33	ubiquitin specific peptidase 9, X-linked	USP9X
206090_s_at	0.33	disrupted in schizophrenia 1	DISC1
1565599_at	0.33		
209370_s_at	0.33	SH3-domain binding protein 2	SH3BP2
228008_at	0.33		
1553349_at	0.33	AT rich interactive domain 2 (ARID, RFX-like)	ARID2
235334_at	0.33	ST6 (alpha-N-acetyl-neuraminyl-2,3-beta-galactosyl-1,3)-N-acetylgalactosaminide alpha-2,6-sialyltran	ST6GALNAC3
225954_s_at	0.33	midnolin	MIDN
239763_at	0.33		
224458_at	0.33	chromosome 9 open reading frame 125	C9orf125
222258_s_at	0.33	SH3-domain binding protein 4	SH3BP4
1557303_at	0.33	5', 3'-nucleotidase, cytosolic	NT5C
235221_at	0.33	cerebellin 3 precursor	CBLN3
218229_s_at	0.33	pogo transposable element with KRAB domain	POGK
1558143_a_at	0.33	BCL2-like 11 (apoptosis facilitator)	BCL2L11
209410_s_at	0.33	growth factor receptor-bound protein 10	GRB10
1559766_at	0.33		
209716_at	0.33	colony stimulating factor 1 (macrophage)	CSF1
221830_at	0.33	RAP2A, member of RAS oncogene family	RAP2A
217889_s_at	0.33	cytochrome b reductase 1	CYBRD1
213503_x_at	0.33	annexin A2	ANXA2
225827_at	0.33	eukaryotic translation initiation factor 2C, 2	EIF2C2
221746_at	0.33	ubiquitin-like 4A	UBL4A
222173_s_at	0.33	TBC1 domain family, member 2	TBC1D2
231795_at	0.33		
225752_at	0.33	non imprinted in Prader-Willi/Angelman syndrome 1	NIPA1
226560_at	0.33		
213153_at	0.33	SET domain containing 1B	SETD1B
226609_at	0.32	discoidin, CUB and LCCL domain containing 1	DCBLD1
200836_s_at	0.32	microtubule-associated protein 4	MAP4
229218_at	0.32	collagen, type I, alpha 2	COL1A2
202988_s_at	0.32	regulator of G-protein signalling 1	RGS1
210916_s_at	0.32	CD44 molecule (Indian blood group)	CD44
1554600_s_at	0.32	lamin A/C	LMNA
227346_at	0.32	IKAROS family zinc finger 1 (Ikaros)	IKZF1
212655_at	0.32	zinc finger, CCHC domain containing 14	ZCCHC14
213836_s_at	0.32	WD repeat domain, phosphoinositide interacting 1	WIPI1
227463_at	0.32		
212119_at	0.32	ras homolog gene family, member Q	RHOQ
200952_s_at	0.32	cyclin D2	CCND2
242523_at	0.32		
212014_x_at	0.32	CD44 molecule (Indian blood group)	CD44
212895_s_at	0.32	active BCR-related gene	ABR
242612_at	0.32		
225946_at	0.32	Ras association (RalGDS/AF-6) domain family 8	RASSF8
227502_at	0.32	KIAA1147	KIAA1147
230085_at	0.32	phosphate cytidylyltransferase 1, choline, beta	PCYT1B

229781_at	0.32		
243601_at	0.32		
207836_s_at	0.32	RNA binding protein with multiple splicing	RBPMS
229299_at	0.32	chromosome 5 open reading frame 33	C5orf33
1570408_at	0.32		
234437_at	0.32		
238684_at	0.32		
202460_s_at	0.32	lipin 2	LPIN2
203940_s_at	0.32	vasohibin 1	VASH1
1555912_at	0.32	ST7 overlapping transcript 1 (antisense non-coding RNA)	ST7OT1
205248_at	0.32	dopey family member 2	DOPEY2
204774_at	0.32	ecotropic viral integration site 2A	EVI2A
217655_at	0.32	FXYD domain containing ion transport regulator 5	FXYD5
219695_at	0.32	sphingomyelin phosphodiesterase 3, neutral membrane (neutral sphingomyelinase II)	SMPD3
202625_at	0.32	v-yes-1 Yamaguchi sarcoma viral related oncogene homolog	LYN
232752_at	0.32		
1560717_at	0.32		
210933_s_at	0.32	fascin homolog 1, actin-bundling protein (Strongylocentrotus purpuratus)	FSCN1
202173_s_at	0.32	vascular endothelial zinc finger 1	VEZF1
55093_at	0.32		
215248_at	0.32		
225924_at	0.32		
204494_s_at	0.32	chromosome 15 open reading frame 39	C15orf39
238013_at	0.32	pleckstrin homology domain containing, family A (phosphoinositide binding specific) member 2	PLEKHA2
235388_at	0.32	chromodomain helicase DNA binding protein 9	CHD9
235753_at	0.32	homeobox A7	HOXA7
210094_s_at	0.32	par-3 partitioning defective 3 homolog (C. elegans)	PARD3
1552841_s_at	0.32	BCL2-like 14 (apoptosis facilitator)	BCL2L14
233650_at	0.32	centrosomal protein 63kDa	CEP63
225738_at	0.32	Rap guanine nucleotide exchange factor (GEF) 1	RAPGEF1
203726_s_at	0.32	laminin, alpha 3	LAMA3
1559745_at	0.32		
241314_at	0.32		
235230_at	0.32		
202455_at	0.32	histone deacetylase 5	HDAC5
237692_at	0.32	thyroid adenoma associated	THADA
217943_s_at	0.32	MAP7 domain containing 1	MAP7D1
236769_at	0.32		
225913_at	0.32		
1569003_at	0.32	transmembrane protein 49	TMEM49
210162_s_at	0.32	nuclear factor of activated T-cells, cytoplasmic, calcineurin-dependent 1	NFATC1
235513_at	0.32		
242202_at	0.32		
225235_at	0.32	tetraspanin 17	TSPAN17
228240_at	0.32		

225530_at	0.32	MOB1, Mps One Binder kinase activator-like 2A (yeast)	MOBKL2A
235857_at	0.32	potassium channel tetramerisation domain containing 11	KCTD11
202617_s_at	0.32	methyl CpG binding protein 2 (Rett syndrome)	MECP2
215427_s_at	0.32	zinc finger, CCHC domain containing 14	ZCCHC14
203397_s_at	0.32	UDP-N-acetyl-alpha-D-galactosamine:polypeptide N-acetylgalactosaminyltransferase 3 (GalNAc-T3)	GALNT3
200706_s_at	0.32	lipopolysaccharide-induced TNF factor	LITAF
207051_at	0.32	solute carrier family 17 (sodium phosphate), member 4	SLC17A4
212989_at	0.32	transmembrane protein 23	TMEM23
221123_x_at	0.32	zinc finger protein 395	ZNF395
214663_at	0.32	receptor interacting protein kinase 5	RIPK5
209559_at	0.32	huntingtin interacting protein 1 related	HIP1R
223366_at	0.32		
227341_at	0.32	chromosome 10 open reading frame 30	C10orf30
221748_s_at	0.32	tensin 1	TNS1
232279_at	0.32	PHD finger protein 15	PHF15
227049_at	0.32		
217762_s_at	0.32	RAB31, member RAS oncogene family	RAB31
237387_at	0.32	actin related protein 2/3 complex, subunit 5, 16kDa	ARPC5
222491_at	0.32	heparan-alpha-glucosaminide N-acetyltransferase	HGSNAT
208056_s_at	0.32	core-binding factor, runt domain, alpha subunit 2; translocated to, 3	CBFA2T3
209604_s_at	0.32	GATA binding protein 3	GATA3
202606_s_at	0.32	tousled-like kinase 1	TLK1
210427_x_at	0.32	annexin A2	ANXA2
229785_at	0.32	KRIT1, ankyrin repeat containing	KRIT1
222716_s_at	0.32	sorting nexing 24	SNX24
220615_s_at	0.32	male sterility domain containing 1	MLSTD1
201250_s_at	0.32	solute carrier family 2 (facilitated glucose transporter), member 1	SLC2A1
226158_at	0.32	kelch-like 24 (Drosophila)	KLHL24
221286_s_at	0.32		
230999_at	0.32		
221865_at	0.32	chromosome 9 open reading frame 91	C9orf91
244808_at	0.32	GRAM domain containing 1A	GRAMD1A
223658_at	0.32	potassium channel, subfamily K, member 6	KCNK6
221937_at	0.32		
212830_at	0.32	multiple EGF-like-domains 9	MEGF9
209835_x_at	0.32	CD44 molecule (Indian blood group)	CD44
41113_at	0.32	zinc finger protein 500	ZNF500
229101_at	0.31		
209757_s_at	0.31	v-myc myelocytomatosis viral related oncogene, neuroblastoma derived (avian)	MYCN
234395_at	0.31	olfactory receptor, family 4, subfamily A, member 1 pseudogene	OR4A1P
226104_at	0.31		
226135_at	0.31	chromosome 6 open reading frame 107	C6orf107

207735_at	0.31	ring finger protein 125	RNF125
225632_s_at	0.31	RAB43, member RAS oncogene family	RAB43
203233_at	0.31	interleukin 4 receptor	IL4R
209099_x_at	0.31	jagged 1 (Alagille syndrome)	JAG1
1568752_s_at	0.31	regulator of G-protein signalling 13	RGS13
237175_at	0.31		
232682_at	0.31	peroxisomal trans-2-enoyl-CoA reductase	PECR
236286_at	0.31		
201557_at	0.31	vesicle-associated membrane protein 2 (synaptobrevin 2)	VAMP2
37005_at	0.31	neuroblastoma, suppression of tumorigenicity 1	NBL1
219722_s_at	0.31	glycerophosphodiester phosphodiesterase domain containing 3	GDPD3
239072_at	0.31		
226023_at	0.31	mitogen-activated protein kinase kinase 7	MAP2K7
222821_s_at	0.31	gem (nuclear organelle) associated protein 7	GEMIN7
216027_at	0.31	thioredoxin domain containing 13	TXNDC13
1552639_at	0.31	kelch domain containing 7B	KLHDC7B
212115_at	0.31	hematological and neurological expressed 1-like	HN1L
212921_at	0.31	SET and MYND domain containing 2	SMYD2
203094_at	0.31	MAD2L1 binding protein	MAD2L1BP
203188_at	0.31	UDP-GlcNAc:betaGal beta-1,3-N-acetylglucosaminyltransferase 1	B3GNT1
224352_s_at	0.31	cofilin 2 (muscle)	CFL2
228551_at	0.31		
209414_at	0.31	fizzy/cell division cycle 20 related 1 (Drosophila)	FZR1
203482_at	0.31	chromosome 10 open reading frame 6#progressive external ophthalmoplegia 1#sema domain, immunoglobulin domain (Ig), transmembrane domain (TM) and short cytoplasmic domain, (se#PDZ domain containing 7#Kazal-type serine peptidase inhibitor domain 1#Kazal-type serine peptidase inhibitor domain 1#sideroflexin 3#leucine zipper, putative tumor suppressor 2#mitochondrial ribosomal protein L43#microRNA 608	C10orf6#PEO1#SEMA4G#PDZD7#KAZALD1#KAZALD1#SFXN3#LZTS2#MRPL43#MIRN608
205965_at	0.31	basic leucine zipper transcription factor, ATF-like	BATF
213590_at	0.31	solute carrier family 16, member 5 (monocarboxylic acid transporter 6)	SLC16A5
241824_at	0.31	FOS-like antigen 2	FOSL2
216867_s_at	0.31	platelet-derived growth factor alpha polypeptide	PDGFA
209542_x_at	0.31	insulin-like growth factor 1 (somatomedin C)	IGF1
1555962_at	0.31	UDP-GlcNAc:betaGal beta-1,3-N-acetylglucosaminyltransferase 7	B3GNT7
225128_at	0.31	KDEL (Lys-Asp-Glu-Leu) containing 2	KDELC2
203553_s_at	0.31	mitogen-activated protein kinase kinase kinase kinase 5	MAP4K5
1554453_at	0.31	heterogeneous nuclear ribonucleoprotein L-like	HNRPLL
238469_at	0.31		
229393_at	0.31	l(3)mbt-like 3 (Drosophila)	L3MBTL3
208622_s_at	0.31		
214179_s_at	0.31	nuclear factor (erythroid-derived 2)-like 1	NFE2L1
217763_s_at	0.31	RAB31, member RAS oncogene family	RAB31

226143_at	0.31	retinoic acid induced 1	RAI1
227067_x_at	0.31	neuroblastoma breakpoint family, member 1	NBPF1
243613_at	0.31		
242076_at	0.31	zinc finger, CCHC domain containing 7	ZCCHC7
204502_at	0.31	SAM domain and HD domain 1	SAMHD1
210214_s_at	0.31	bone morphogenetic protein receptor, type II (serine/threonine kinase)	BMPR2
228959_at	0.31		
1558517_s_at	0.31		
212120_at	0.31	ras homolog gene family, member Q	RHOQ
217200_x_at	0.31	cytochrome b-561	CYB561
224704_at	0.31	trinucleotide repeat containing 6A	TNRC6A
241353_s_at	0.31		
236831_at	0.31	coiled-coil domain containing 50	CCDC50
200759_x_at	0.31	nuclear factor (erythroid-derived 2)-like 1	NFE2L1
1568783_at	0.31	splicing factor, arginine/serine-rich 12	SFRS12
210018_x_at	0.31	mucosa associated lymphoid tissue lymphoma translocation gene 1	MALT1
202459_s_at	0.31	lipin 2	LPIN2
203476_at	0.31	trophoblast glycoprotein	TPBG
202081_at	0.31	immediate early response 2	IER2
212677_s_at	0.31	centrosomal protein 68kDa	CEP68
212457_at	0.31	transcription factor binding to IGHM enhancer 3	TFE3
225606_at	0.31	BCL2-like 11 (apoptosis facilitator)	BCL2L11
205101_at	0.31	class II, major histocompatibility complex, transactivator	CIITA
213329_at	0.31	SLIT-ROBO Rho GTPase activating protein 2	SRGAP2
223385_at	0.31	cytochrome P450, family 2, subfamily S, polypeptide 1	CYP2S1
210072_at	0.31	chemokine (C-C motif) ligand 19	CCL19
1556216_s_at	0.31		
209309_at	0.31	alpha-2-glycoprotein 1, zinc-binding	AZGP1
226430_at	0.31		
204715_at	0.31	pannexin 1	PANX1
231313_at	0.31	leucine rich repeat containing 8 family, member B	LRRC8B
228980_at	0.31	ring finger and FYVE-like domain containing 1	RFFL
220095_at	0.31	chromosome 9 open reading frame 39	C9orf39
242338_at	0.31	transmembrane protein 64	TMEM64
209574_s_at	0.31	chromosome 18 open reading frame 1	C18orf1
229943_at	0.31	tripartite motif-containing 13	TRIM13
202969_at	0.31		
238597_at	0.31		
207882_at	0.31		
210512_s_at	0.31	vascular endothelial growth factor A	VEGFA
205479_s_at	0.31	plasminogen activator, urokinase	PLAU
213191_at	0.31	toll-like receptor adaptor molecule 1	TICAM1
228065_at	0.30	B-cell CLL/lymphoma 9-like	BCL9L
201848_s_at	0.30	BCL2/adenovirus E1B 19kDa interacting protein 3	BNIP3
230834_at	0.30		
221235_s_at	0.30		
208309_s_at	0.30	mucosa associated lymphoid tissue lymphoma translocation gene 1	MALT1

215473_at	0.30		
231049_at	0.30	LIM domain only 2 (rhombotin-like 1)	LMO2
219330_at	0.30	vang-like 1 (van gogh, Drosophila)	VANGL1
204621_s_at	0.30	nuclear receptor subfamily 4, group A, member 2	NR4A2
228083_at	0.30	calcium channel, voltage-dependent, alpha 2/delta subunit 4	CACNA2D4
226003_at	0.30	kinesin family member 21A	KIF21A
210999_s_at	0.30	growth factor receptor-bound protein 10	GRB10
211343_s_at	0.30	collagen, type XIII, alpha 1	COL13A1
230003_at	0.30		
230466_s_at	0.30		
225978_at	0.30		
235529_x_at	0.30		
223190_s_at	0.30	myeloid/lymphoid or mixed-lineage leukemia 5 (trithorax homolog, Drosophila)	MLL5
206674_at	0.30	fms-related tyrosine kinase 3	FLT3
225785_at	0.30	receptor accessory protein 3	REEP3
215460_x_at	0.30	bromodomain containing 1	BRD1
1566551_at	0.30		
213419_at	0.30	amyloid beta (A4) precursor protein-binding, family B, member 2 (Fe65-like)	APBB2
227632_at	0.30	TBC1 domain family, member 24	TBC1D24
1553743_at	0.30	family with sequence similarity 119, member A	FAM119A
227383_at	0.30	family with sequence similarity 91, member A2	FAM91A2
60084_at	0.30	cylindromatosis (turban tumor syndrome)	CYLD
228062_at	0.30	nucleosome assembly protein 1-like 5	NAP1L5
229997_at	0.30	vang-like 1 (van gogh, Drosophila)	VANGL1
222101_s_at	0.30	dachsous 1 (Drosophila)	DCHS1
204020_at	0.30	purine-rich element binding protein A	PURA
218486_at	0.30	Kruppel-like factor 11	KLF11
201963_at	0.30	acyl-CoA synthetase long-chain family member 1	ACSL1
228436_at	0.30	potassium voltage-gated channel, Shaw-related subfamily, member 4	KCNC4
201651_s_at	0.30	protein kinase C and casein kinase substrate in neurons 2	PACSIN2
209307_at	0.30		
217999_s_at	0.30	pleckstrin homology-like domain, family A, member 1	PHLDA1
201590_x_at	0.30	annexin A2	ANXA2
1552667_a_at	0.30	SH2 domain containing 3C	SH2D3C
202651_at	0.30	lysophosphatidylglycerol acyltransferase 1	LPGAT1
201218_at	0.30	C-terminal binding protein 2	CTBP2
219202_at	0.30	rhomboid 5 homolog 2 (Drosophila)	RHBDF2
236937_at	0.30	vacuolar protein sorting 8 homolog (S. cerevisiae)	VPS8
228258_at	0.30	TBC1 domain family, member 10C	TBC1D10C
221526_x_at	0.30	par-3 partitioning defective 3 homolog (C. elegans)	PARD3
230127_at	0.30		
201234_at	0.30	integrin-linked kinase	ILK
211685_s_at	0.30	neurocalcin delta	NCALD
240449_at	0.30	zinc finger protein 341	ZNF341
213112_s_at	0.30	sequestosome 1	SQSTM1
223264_at	0.30	mesoderm development candidate 1	MESDC1

226016_at	0.30	CD47 molecule	CD47
224572_s_at	0.30	interferon regulatory factor 2 binding protein 2	IRF2BP2
205453_at	0.30	homeobox B2	HOXB2
218928_s_at	0.30	solute carrier family 37 (glycerol-3-phosphate transporter), member 1	SLC37A1
204562_at	0.30	interferon regulatory factor 4	IRF4
235079_at	0.30		
210665_at	0.30	tissue factor pathway inhibitor (lipoprotein-associated coagulation inhibitor)	TFPI
235072_s_at	0.30		
203659_s_at	0.30	tripartite motif-containing 13	TRIM13
205366_s_at	0.30	homeobox B6	HOXB6
206904_at	0.30	matrilin 1, cartilage matrix protein	MATN1
201426_s_at	0.30	vimentin	VIM
212372_at	0.30	myosin, heavy chain 10, non-muscle	MYH10
205899_at	0.30	cyclin A1	CCNA1
240574_at	0.30		
212845_at	0.30	sterile alpha motif domain containing 4A	SAMD4A
230561_s_at	0.30		
212791_at	0.30		
213391_at	0.30	dpy-19-like 4 (C. elegans)	DPY19L4
235542_at	0.30		
238575_at	0.30	oxysterol binding protein-like 6	OSBPL6
204032_at	0.30	breast cancer anti-estrogen resistance 3	BCAR3
226473_at	0.30	chromobox homolog 2 (Pc class homolog, Drosophila)	CBX2
239376_at	0.30		
226867_at	0.30	DENN/MADD domain containing 4C	DENND4C
212062_at	0.30	ATPase, Class II, type 9A	ATP9A
230815_at	0.30		
204951_at	0.30	ras homolog gene family, member H	RHOH
225629_s_at	0.30	zinc finger and BTB domain containing 4	ZBTB4
241701_at	0.29		
242419_at	0.29		
221778_at	0.29		
216942_s_at	0.29	CD58 molecule	CD58
226959_at	0.29		
229312_s_at	0.29	G kinase anchoring protein 1	GKAP1
226795_at	0.29		
244271_at	0.29		
227470_at	0.29	zinc finger protein 553	ZNF553
233849_s_at	0.29	Rho GTPase activating protein 5	ARHGAP5
226946_at	0.29	chromosome 5 open reading frame 33	C5orf33
229307_at	0.29	ankyrin repeat domain 28	ANKRD28
221122_at	0.29	HRAS-like suppressor 2	HRASLS2
215053_at	0.29		
226771_at	0.29	ATPase, Class I, type 8B, member 2	ATP8B2
210971_s_at	0.29	aryl hydrocarbon receptor nuclear translocator-like	ARNTL
233863_at	0.29	castor zinc finger 1	CASZ1
209193_at	0.29	pim-1 oncogene	PIM1
223674_s_at	0.29	CDC42 small effector 1	CDC42SE1
220924_s_at	0.29	solute carrier family 38, member 2	SLC38A2

216250_s_at	0.29	leupaxin	LPXN
207160_at	0.29	interleukin 12A (natural killer cell stimulatory factor 1, cytotoxic lymphocyte maturation factor 1,	IL12A
218902_at	0.29	Notch homolog 1, translocation-associated (Drosophila)	NOTCH1
201430_s_at	0.29	dihydropyrimidinase-like 3	DPYSL3
202971_s_at	0.29	dual-specificity tyrosine-(Y)-phosphorylation regulated kinase 2	DYRK2
202083_s_at	0.29	SEC14-like 1 (S. cerevisiae)	SEC14L1
224762_at	0.29	serine incorporator 2	SERINC2
242284_at	0.29		
225142_at	0.29		
241881_at	0.29	olfactory receptor, family 2, subfamily W, member 3	OR2W3
1554544_a_at	0.29	myelin basic protein	MBP
204490_s_at	0.29	CD44 molecule (Indian blood group)	CD44
240393_at	0.29	amino-terminal enhancer of split	AES
222235_s_at	0.29		
235777_at	0.29	ankyrin repeat domain 44	ANKRD44
215275_at	0.29	TRAF3 interacting protein 3	TRAF3IP3
215127_s_at	0.29	RNA binding motif, single stranded interacting protein 1	RBMS1
227749_at	0.29		
209288_s_at	0.29	CDC42 effector protein (Rho GTPase binding) 3	CDC42EP3
224733_at	0.29	CKLF-like MARVEL transmembrane domain containing 3	CMTM3
225539_at	0.29	chromosome 21 open reading frame 25#zinc finger protein 295#zinc finger protein 295#PR domain containing 15	C21orf25#ZNF295#ZNF295#PRDM15
1555920_at	0.29	chromobox homolog 3 (HP1 gamma homolog, Drosophila)	CBX3
1553644_at	0.29	chromosome 14 open reading frame 49	C14orf49
228935_at	0.29	solute carrier family 4, sodium bicarbonate cotransporter, member 8	SLC4A8
231866_at	0.29	leucyl/cystinyl aminopeptidase	LNPEP
1569346_a_at	0.29		
210954_s_at	0.29	TSC22 domain family, member 2	TSC22D2
39582_at	0.29	cylindromatosis (turban tumor syndrome)	CYLD
206099_at	0.29	protein kinase C, eta	PRKCH
209060_x_at	0.29	nuclear receptor coactivator 3	NCOA3
242820_at	0.29		
211031_s_at	0.29	CAP-GLY domain containing linker protein 2	CLIP2
213310_at	0.29		
241016_at	0.29	cullin 3	CUL3
244447_at	0.29		
210170_at	0.29	PDZ and LIM domain 3	PDLIM3
226554_at	0.29	zinc finger and BTB domain containing 7A	ZBTB7A
231295_at	0.29	malic enzyme 3, NADP(+)-dependent, mitochondrial	ME3
209062_x_at	0.29	nuclear receptor coactivator 3	NCOA3
235427_at	0.29		
1555009_a_at	0.29	synaptojanin 2	SYNJ2

231202_at	0.29		
236539_at	0.29	protein tyrosine phosphatase, non-receptor type 22 (lymphoid)	PTPN22
224827_at	0.29	ubiquitin domain containing 2	UBTD2
1559545_at	0.29	small nuclear ribonucleoprotein polypeptide N	SNRPN
212122_at	0.29	ras homolog gene family, member Q	RHOQ
222918_at	0.29	RAB9B, member RAS oncogene family	RAB9B
223092_at	0.29	ankylosis, progressive homolog (mouse)	ANKH
235844_at	0.29	putative homeodomain transcription factor 1	PHTF1
209012_at	0.29	triple functional domain (PTPRF interacting)	TRIO
223189_x_at	0.29	myeloid/lymphoid or mixed-lineage leukemia 5 (trithorax homolog, Drosophila)	MLL5
1563357_at	0.29		
207303_at	0.28	phosphodiesterase 1C, calmodulin-dependent 70kDa	PDE1C
225339_at	0.28		
211317_s_at	0.28	CASP8 and FADD-like apoptosis regulator	CFLAR
226793_at	0.28		
204222_s_at	0.28	GLI pathogenesis-related 1 (glioma)	GLIPR1
228257_at	0.28	ankyrin repeat domain 52	ANKRD52
214888_at	0.28	calpain 2, (m/II) large subunit	CAPN2
207700_s_at	0.28	nuclear receptor coactivator 3	NCOA3
228904_at	0.28	homeobox B3	HOXB3
208890_s_at	0.28	plexin B2	PLXNB2
239982_at	0.28		
205569_at	0.28	lysosomal-associated membrane protein 3	LAMP3
205702_at	0.28	putative homeodomain transcription factor 1	PHTF1
204980_at	0.28	clock homolog (mouse)	CLOCK
1554690_a_at	0.28	transforming, acidic coiled-coil containing protein 1	TACC1
238642_at	0.28	ankyrin repeat domain 13 family, member D	ANKRD13D
211105_s_at	0.28	nuclear factor of activated T-cells, cytoplasmic, calcineurin-dependent 1	NFATC1
222560_at	0.28	LanC lantibiotic synthetase component C-like 2 (bacterial)	LANCL2
212561_at	0.28	RAB6 interacting protein 1	RAB6IP1
238451_at	0.28	membrane protein, palmitoylated 7 (MAGUK p55 subfamily member 7)	MPP7
231699_at	0.28	nuclear factor of kappa light polypeptide gene enhancer in B-cells inhibitor, alpha	NFKBIA
207996_s_at	0.28	chromosome 18 open reading frame 1	C18orf1
241492_at	0.28		
229739_s_at	0.28	family with sequence similarity 116, member B	FAM116B
225436_at	0.28		
214104_at	0.28	G protein-coupled receptor 161	GPR161
203543_s_at	0.28	Kruppel-like factor 9	KLF9
223915_at	0.28	BCL6 co-repressor	BCOR
229495_at	0.28	aminoacylase 1-like 2	ACY1L2
244492_at	0.28		
209050_s_at	0.28	ral guanine nucleotide dissociation stimulator	RALGDS
218149_s_at	0.28	zinc finger protein 395	ZNF395
212398_at	0.28	radixin	RDX
1557749_at	0.28	EH domain binding protein 1-like 1	EHBP1L1

235996_at	0.28	Ras association (RalGDS/AF-6) domain family 8	RASSF8
201454_s_at	0.28	aminopeptidase puromycin sensitive	NPEPPS
236889_at	0.28	vacuolar protein sorting 37 homolog B (S. cerevisiae)	VPS37B
212641_at	0.28	human immunodeficiency virus type I enhancer binding protein 2	HIVEP2
219520_s_at	0.28	WWC family member 3	WWC3
226112_at	0.28	sarcoglycan, beta (43kDa dystrophin-associated glycoprotein)	SGCB
218157_x_at	0.28	CDC42 small effector 1	CDC42SE1
232224_at	0.28	mannan-binding lectin serine peptidase 1 (C4/C2 activating component of Ra-reactive factor)	MASP1
221680_s_at	0.28	ets variant gene 7 (TEL2 oncogene)	ETV7
1552696_at	0.28	non imprinted in Prader-Willi/Angelman syndrome 1	NIPA1
204469_at	0.28	protein tyrosine phosphatase, receptor-type, Z polypeptide 1	PTPRZ1
202335_s_at	0.28	ubiquitin-conjugating enzyme E2B (RAD6 homolog)	UBE2B
221864_at	0.28		
228833_s_at	0.28		
240316_at	0.28	chromosome 9 open reading frame 57	C9orf57
212356_at	0.28	KIAA0323	KIAA0323
227283_at	0.28		
210038_at	0.28	protein kinase C, theta#null#null	PRKCQ#null#null
209949_at	0.28	neutrophil cytosolic factor 2 (65kDa, chronic granulomatous disease, autosomal 2)	NCF2
204512_at	0.28	human immunodeficiency virus type I enhancer binding protein 1	HIVEP1
218263_s_at	0.28	zinc finger, BED-type containing 5	ZBED5
205107_s_at	0.28	ephrin-A4	EFNA4
236685_at	0.28		
218792_s_at	0.28	B-box and SPRY domain containing	BSPRY
206847_s_at	0.28	homeobox A7	HOXA7
211962_s_at	0.28	zinc finger protein 36, C3H type-like 1	ZFP36L1
207375_s_at	0.28	interleukin 15 receptor, alpha	IL15RA
203921_at	0.28	carbohydrate (N-acetylglucosamine-6-O) sulfotransferase 2	CHST2
213295_at	0.28	cylindromatosis (turban tumor syndrome)	CYLD
203955_at	0.28	KIAA0649	KIAA0649
226497_s_at	0.28		
230688_at	0.28		
205896_at	0.28	solute carrier family 22 (organic cation transporter), member 4	SLC22A4
226219_at	0.28	Rho GTPase activating protein 30	ARHGAP30
227099_s_at	0.28		
225602_at	0.28	chromosome 9 open reading frame 19	C9orf19
225144_at	0.28	bone morphogenetic protein receptor, type II (serine/threonine kinase)	BMPR2
224466_s_at	0.27	v-maf musculoaponeurotic fibrosarcoma oncogene homolog G (avian)	MAFG
225532_at	0.27	Cdk5 and Abl enzyme substrate 1	CABLES1

222061_at	0.27	CD58 molecule	CD58
201645_at	0.27	tenascin C (hexabrachion)	TNC
217132_at	0.27		
1563392_at	0.27		
201005_at	0.27	CD9 molecule	CD9
224909_s_at	0.27		
230092_at	0.27	UBX domain containing 3	UBXD3
228173_at	0.27	GNAS complex locus	GNAS
1557036_at	0.27	zinc finger and BTB domain containing 1	ZBTB1
1559204_x_at	0.27	v-Ki-ras2 Kirsten rat sarcoma viral oncogene homolog	KRAS
225604_s_at	0.27	chromosome 9 open reading frame 19	C9orf19
225386_s_at	0.27	heterogeneous nuclear ribonucleoprotein L-like	HNRPLL
223437_at	0.27	peroxisome proliferator-activated receptor alpha	PPARA
212930_at	0.27	ATPase, Ca++ transporting, plasma membrane 1	ATP2B1
204489_s_at	0.27	CD44 molecule (Indian blood group)	CD44
207971_s_at	0.27	centrosomal protein 68kDa	CEP68
206586_at	0.27	cannabinoid receptor 2 (macrophage)	CNR2
202807_s_at	0.27	target of myb1 (chicken)	TOM1
204440_at	0.27	CD83 molecule	CD83
212475_at	0.27	KIAA0241	KIAA0241
219911_s_at	0.27	solute carrier organic anion transporter family, member 4A1	SLCO4A1
203823_at	0.27	regulator of G-protein signalling 3	RGS3
227999_at	0.27	PWWP domain containing 2	PWWP2
210758_at	0.27	PC4 and SFRS1 interacting protein 1	PSIP1
216180_s_at	0.27	synaptojanin 2	SYNJ2
1557585_at	0.27	ATPase, H+ transporting, lysosomal 50/57kDa, V1 subunit H	ATP6V1H
226074_at	0.27	protein phosphatase 1M (PP2C domain containing)	PPM1M
229004_at	0.27		
228594_at	0.27	chromosome 5 open reading frame 33	C5orf33
1569335_a_at	0.27	stimulated by retinoic acid gene 6 homolog (mouse)	STRA6
226164_x_at	0.27	family with sequence similarity 80, member B	FAM80B
36564_at	0.27	IBR domain containing 3	IBRDC3
201646_at	0.27	scavenger receptor class B, member 2	SCARB2
213690_s_at	0.27		
224739_at	0.27	pim-3 oncogene	PIM3
209295_at	0.27	tumor necrosis factor receptor superfamily, member 10b	TNFRSF10B
227036_at	0.27		
224534_at	0.27	kringle containing transmembrane protein 1	KREMEN1
224833_at	0.27	v-ets erythroblastosis virus E26 oncogene homolog 1 (avian)	ETS1
1555977_at	0.27		
227112_at	0.27	transmembrane and coiled-coil domain family 1	TMCC1
205278_at	0.27	glutamate decarboxylase 1 (brain, 67kDa)	GAD1
228549_at	0.27	transmembrane protein 63A	TMEM63A
204631_at	0.27	myosin, heavy chain 2, skeletal muscle, adult	MYH2
1557527_at	0.27		
209909_s_at	0.27	transforming growth factor, beta 2	TGFB2

226265_at	0.27	glutamine and serine rich 1	QSER1
224998_at	0.27	CKLF-like MARVEL transmembrane domain containing 4	CMTM4
221957_at	0.27	pyruvate dehydrogenase kinase, isozyme 3	PDK3
226155_at	0.27	KIAA1600	KIAA1600
223182_s_at	0.27	1-acylglycerol-3-phosphate O-acyltransferase 3	AGPAT3
228220_at	0.27	FCH domain only 2	FCHO2
213010_at	0.27	protein kinase C, delta binding protein	PRKCDBP
227261_at	0.27	Kruppel-like factor 12	KLF12
203098_at	0.27	chromodomain protein, Y-like	CDYL
211719_x_at	0.27	fibronectin 1	FN1
231769_at	0.27	F-box protein 6	FBXO6
212445_s_at	0.27	neural precursor cell expressed, developmentally down-regulated 4-like	NEDD4L
241627_x_at	0.27		
226932_at	0.27		
1555777_at	0.27	periostin, osteoblast specific factor	POSTN
240744_at	0.27	carboxypeptidase A5	CPA5
235362_at	0.27		
225368_at	0.27	homeodomain interacting protein kinase 2	HIPK2
1570409_x_at	0.27		
224480_s_at	0.27		
236559_at	0.27	tyrosine 3-monooxygenase/tryptophan 5-monooxygenase activation protein, eta polypeptide	YWHAH
237026_at	0.27	sorbin and SH3 domain containing 1	SORBS1
226039_at	0.27	mannosyl (alpha-1,3-)-glycoprotein beta-1,4-N-acetylglucosaminyltransferase, isozyme A	MGAT4A
222537_s_at	0.27	CDC42 small effector 1	CDC42SE1
1569736_at	0.27		
232155_at	0.27	KIAA1618	KIAA1618
214438_at	0.27	H2.0-like homeobox 1 (Drosophila)	HLX1
1558340_at	0.27	DIX domain containing 1	DIXDC1
239336_at	0.27	thrombospondin 1	THBS1
244035_at	0.27		
243869_at	0.27		
212675_s_at	0.27	centrosomal protein 68kDa	CEP68
241581_at	0.27		
225416_at	0.27	ring finger protein 12	RNF12
1563507_at	0.27		
230179_at	0.27		
203514_at	0.27	mitogen-activated protein kinase kinase kinase 3	MAP3K3
230086_at	0.27	formin binding protein 1	FNBP1
212441_at	0.27		
227775_at	0.27	bruno-like 6, RNA binding protein (Drosophila)	BRUNOL6
1559050_at	0.27	HLA complex group 27	HCG27
231688_at	0.27		
1559584_a_at	0.27	chromosome 16 open reading frame 54	C16orf54
223161_at	0.27	KIAA1147	KIAA1147
206675_s_at	0.27	SKI-like oncogene	SKIL
207426_s_at	0.27	tumor necrosis factor (ligand) superfamily, member 4 (tax-transcriptionally activated glycoprotein 1	TNFSF4

210871_x_at	0.26	synovial sarcoma, X breakpoint 2 interacting protein	SSX2IP
1553765_a_at	0.26	kelch-like 32 (Drosophila)	KLHL32
1569104_a_at	0.26		
209164_s_at	0.26	cytochrome b-561	CYB561
241819_at	0.26	tumor necrosis factor (ligand) superfamily, member 8	TNFSF8
242230_at	0.26	ataxin 1	ATXN1
244790_at	0.26	mature T-cell proliferation 1	MTCP1
227228_s_at	0.26	KIAA1509	KIAA1509
228343_at	0.26	POU domain, class 2, transcription factor 2	POU2F2
209606_at	0.26	pleckstrin homology, Sec7 and coiled-coil domains, binding protein	PSCDBP
235048_at	0.26		
213541_s_at	0.26	v-ets erythroblastosis virus E26 oncogene homolog (avian)	ERG
229435_at	0.26	GLIS family zinc finger 3	GLIS3
209868_s_at	0.26	RNA binding motif, single stranded interacting protein 1	RBMS1
225266_at	0.26	zinc finger protein 652	ZNF652
221753_at	0.26	slingshot homolog 1 (Drosophila)	SSH1
1559882_at	0.26	SAM domain and HD domain 1	SAMHD1
1552564_at	0.26	nudix (nucleoside diphosphate linked moiety X)-type motif 9 pseudogene 1	NUDT9P1
235961_at	0.26	G protein-coupled receptor 161	GPR161
232081_at	0.26		
229670_at	0.26		
223164_at	0.26	cerebral cavernous malformation 2	CCM2
211489_at	0.26	adrenergic, alpha-1A-, receptor	ADRA1A
243356_at	0.26	family with sequence similarity 7, member A1	FAM7A1
228899_at	0.26		
214679_x_at	0.26	guanine nucleotide binding protein (G protein), alpha 11 (Gq class)	GNA11
225269_s_at	0.26	RNA binding motif, single stranded interacting protein 1	RBMS1
242738_s_at	0.26		
1557158_s_at	0.26	myeloid/lymphoid or mixed-lineage leukemia 3	MLL3
209061_at	0.26	nuclear receptor coactivator 3	NCOA3
219229_at	0.26	solute carrier organic anion transporter family, member 3A1	SLCO3A1
207629_s_at	0.26	rho/rac guanine nucleotide exchange factor (GEF) 2	ARHGEF2
244536_at	0.26	tumor protein p53 binding protein, 2	TP53BP2
231029_at	0.26	coagulation factor V (proaccelerin, labile factor)	F5
227844_at	0.26	formin-like 3	FMNL3
209973_at	0.26	nuclear factor of kappa light polypeptide gene enhancer in B-cells inhibitor-like 1	NFKBIL1
206687_s_at	0.26	protein tyrosine phosphatase, non-receptor type 6	PTPN6
211113_s_at	0.26	ATP-binding cassette, sub-family G (WHITE), member 1	ABCG1
239779_at	0.26		
235012_at	0.26		

238725_at	0.26		
227599_at	0.26	chromosome 3 open reading frame 59	C3orf59
228843_at	0.26		
231963_at	0.26		
221718_s_at	0.26	A kinase (PRKA) anchor protein 13	AKAP13
1557882_at	0.26		
226961_at	0.26	proline rich 15	PRR15
1560241_at	0.26		
232979_at	0.26		
231858_x_at	0.26		
236701_at	0.26	GSG1-like	GSG1L
235142_at	0.26	zinc finger and BTB domain containing 8	ZBTB8
204205_at	0.26	apolipoprotein B mRNA editing enzyme, catalytic polypeptide-like 3G	APOBEC3G
204618_s_at	0.26	GA binding protein transcription factor, beta subunit 2	GABPB2
218421_at	0.26	ceramide kinase	CERK
234987_at	0.26		
225150_s_at	0.26	rhotekin	RTKN
214515_at	0.26	olfactory receptor, family 1, subfamily E, member 1	OR1E1
201000_at	0.26	alanyl-tRNA synthetase	AARS
201578_at	0.26	podocalyxin-like	PODXL
1555355_a_at	0.26	v-ets erythroblastosis virus E26 oncogene homolog 1 (avian)	ETS1
203627_at	0.26	insulin-like growth factor 1 receptor	IGF1R
200704_at	0.26	lipopolysaccharide-induced TNF factor	LITAF
225232_at	0.26	myotubularin related protein 12	MTMR12
235627_at	0.26	profilin family, member 4	PFN4
1555963_x_at	0.26	UDP-GlcNAc:betaGal beta-1,3-N-acetylglucosaminyltransferase 7	B3GNT7
235276_at	0.26	epithelial stromal interaction 1 (breast)	EPSTI1
212733_at	0.26	KIAA0226	KIAA0226
242168_at	0.26	NADH dehydrogenase (ubiquinone) Fe-S protein 7, 20kDa (NADH-coenzyme Q reductase)	NDUFS7
203185_at	0.26	Ras association (RalGDS/AF-6) domain family 2	RASSF2
243767_at	0.26		
1565628_at	0.26		
239744_at	0.26		
235252_at	0.26	kinase suppressor of ras 1	KSR1
240432_x_at	0.26		
1570224_at	0.26		
1565900_at	0.26	methyltransferase 5 domain containing 1	METT5D1
210655_s_at	0.26	forkhead box O3A	FOXO3A
238638_at	0.26	solute carrier family 37 (glycerol-3-phosphate transporter), member 2	SLC37A2
208111_at	0.26	arginine vasopressin receptor 2 (nephrogenic diabetes insipidus)	AVPR2
238077_at	0.26	potassium channel tetramerisation domain containing 6	KCTD6
1559159_at	0.26	centrosomal protein 68kDa	CEP68
212327_at	0.26		
225385_s_at	0.26	heterogeneous nuclear ribonucleoprotein L-like	HNRPLL

205469_s_at	0.26	interferon regulatory factor 5	IRF5
233310_at	0.26		
225628_s_at	0.26	myeloid/lymphoid or mixed-lineage leukemia (trithorax homolog, Drosophila); translocated to, 6	MLLT6
239023_at	0.26		
223158_s_at	0.26	NIMA (never in mitosis gene a)-related kinase 6	NEK6
201389_at	0.25	integrin, alpha 5 (fibronectin receptor, alpha polypeptide)	ITGA5
1552295_a_at	0.25	solute carrier family 39 (zinc transporter), member 13	SLC39A13
211457_at	0.25	GABA(A) receptors associated protein like 3	GABARAPL3
1563386_at	0.25		
214446_at	0.25	elongation factor, RNA polymerase II, 2	ELL2
1552301_a_at	0.25	coronin 6	CORO6
228557_at	0.25	l(3)mbt-like 4 (Drosophila)	L3MBTL4
224115_at	0.25		
225997_at	0.25	MOB1, Mps One Binder kinase activator-like 1A (yeast)	MOBKL1A
225739_at	0.25	RAB11 family interacting protein 4 (class II)	RAB11FIP4
202006_at	0.25	protein tyrosine phosphatase, non-receptor type 12	PTPN12
1566766_a_at	0.25		
219491_at	0.25	leucine rich repeat and fibronectin type III domain containing 4	LRFN4
207075_at	0.25	NLR family, pyrin domain containing 3	NLRP3
212458_at	0.25	sprouty-related, EVH1 domain containing 2	SPRED2
217997_at	0.25	pleckstrin homology-like domain, family A, member 1	PHLDA1
218880_at	0.25	FOS-like antigen 2	FOSL2
241075_at	0.25		
203574_at	0.25	nuclear factor, interleukin 3 regulated	NFIL3
1569140_at	0.25	ubiquitin protein ligase E3 component n-recognin 2	UBR2
204622_x_at	0.25	nuclear receptor subfamily 4, group A, member 2	NR4A2
234204_at	0.25		
220787_at	0.25		
220377_at	0.25	family with sequence similarity 30, member A	FAM30A
204103_at	0.25	chemokine (C-C motif) ligand 4	CCL4
236749_at	0.25	MAX binding protein	MNT
225330_at	0.25	insulin-like growth factor 1 receptor	IGF1R
204684_at	0.25	neuronal pentraxin I	NPTX1
229740_at	0.25		
202723_s_at	0.25	forkhead box O1A (rhabdomyosarcoma)	FOXO1A
1554290_at	0.25	hect domain and RLD 3	HERC3
210001_s_at	0.25	suppressor of cytokine signaling 1	SOCS1
223681_s_at	0.25	InaD-like (Drosophila)	INADL
216248_s_at	0.25	nuclear receptor subfamily 4, group A, member 2	NR4A2
204342_at	0.25	solute carrier family 25 (mitochondrial carrier; phosphate carrier), member 24	SLC25A24
216766_at	0.25		
1555015_a_at	0.25	zinc finger protein 398	ZNF398
211352_s_at	0.25	nuclear receptor coactivator 3	NCOA3
221779_at	0.25	MICAL-like 1	MICALL1

211178_s_at	0.25	proline-serine-threonine phosphatase interacting protein 1	PSTPIP1
212543_at	0.25	absent in melanoma 1	AIM1
218237_s_at	0.25	solute carrier family 38, member 1	SLC38A1
214724_at	0.25	DIX domain containing 1	DIXDC1
237444_at	0.25		
201109_s_at	0.25	thrombospondin 1	THBS1
227290_at	0.25		
236125_at	0.25		
244470_at	0.25	ring finger protein 12	RNF12
226682_at	0.25		
231875_at	0.25	kinesin family member 21A	KIF21A
225545_at	0.25	eukaryotic elongation factor-2 kinase	EEF2K
212817_at	0.25	DnaJ (Hsp40) homolog, subfamily B, member 5	DNAJB5
225627_s_at	0.25	cache domain containing 1	CACHD1
215447_at	0.25	tissue factor pathway inhibitor (lipoprotein-associated coagulation inhibitor)	TFPI
242258_at	0.24		
212735_at	0.24	KIAA0226	KIAA0226
202638_s_at	0.24	intercellular adhesion molecule 1 (CD54), human rhinovirus receptor	ICAM1
236893_at	0.24		
213146_at	0.24	jumonji domain containing 3	JMJD3
229452_at	0.24	transmembrane protein 88	TMEM88
203043_at	0.24	zinc finger, BED-type containing 1	ZBED1
206943_at	0.24	transforming growth factor, beta receptor I (activin A receptor type II-like kinase, 53kDa)	TGFBR1
240294_at	0.24	homeodomain interacting protein kinase 2	HIPK2
225133_at	0.24		
1557644_at	0.24		
209360_s_at	0.24	runt-related transcription factor 1 (acute myeloid leukemia 1; aml1 oncogene)	RUNX1
213219_at	0.24	adenylate cyclase 2 (brain)	ADCY2
242098_at	0.24		
1552372_at	0.24	chromosome 4 open reading frame 33	C4orf33
217538_at	0.24	RUN and TBC1 domain containing 1	RUTBC1
229120_s_at	0.24	CDC42 small effector 1	CDC42SE1
238983_at	0.24	NOL1/NOP2/Sun domain family, member 7	NSUN7
225557_at	0.24	AXIN1 up-regulated 1	AXUD1
226673_at	0.24	SH2 domain containing 3C	SH2D3C
243641_at	0.24		
230047_at	0.24		
228647_at	0.24		
218694_at	0.24	armadillo repeat containing, X-linked 1	ARMCX1
227514_at	0.24		
216979_at	0.24	nuclear receptor subfamily 4, group A, member 3	NR4A3
206173_x_at	0.24	GA binding protein transcription factor, beta subunit 2	GABPB2
239678_at	0.24		
218017_s_at	0.24	heparan-alpha-glucosaminide N-acetyltransferase	HGSNAT
226509_at	0.24	zinc finger protein 641	ZNF641
223103_at	0.24	START domain containing 10	STARD10

201798_s_at	0.24	fer-1-like 3, myoferlin (C. elegans)	FER1L3
202756_s_at	0.24	glypican 1	GPC1
207986_x_at	0.24	cytochrome b-561	CYB561
210357_s_at	0.24	spermine oxidase	SMOX
209402_s_at	0.24	solute carrier family 12 (potassium/chloride transporters), member 4	SLC12A4
36711_at	0.24	phospholipase A2, group VI (cytosolic, calcium-independent)#v-maf musculoaponeurotic fibrosarcoma oncogene homolog F (avian)#chromosome 22 open reading frame 5	PLA2G6#MAF F#C22orf5
227867_at	0.24		
204679_at	0.24	potassium channel, subfamily K, member 1	KCNK1
212859_x_at	0.24	metallothionein 1E (functional)	MT1E
201331_s_at	0.24	signal transducer and activator of transcription 6, interleukin-4 induced	STAT6
232424_at	0.24	PR domain containing 16	PRDM16
237739_at	0.24		
224579_at	0.24	solute carrier family 38, member 1	SLC38A1
225176_at	0.24		
215078_at	0.24	superoxide dismutase 2, mitochondrial	SOD2
227618_at	0.24		
205346_at	0.24	ST3 beta-galactoside alpha-2,3-sialyltransferase 2	ST3GAL2
33304_at	0.24	interferon stimulated exonuclease gene 20kDa	ISG20
1558836_at	0.24		
228450_at	0.24	pleckstrin homology domain containing, family A member 7	PLEKHA7
227534_at	0.24	chromosome 9 open reading frame 21	C9orf21
229397_s_at	0.24	glucocorticoid receptor DNA binding factor 1	GRLF1
201462_at	0.24	secernin 1	SCRN1
214482_at	0.24	zinc finger and BTB domain containing 25	ZBTB25
226685_at	0.24	syntrophin, beta 2 (dystrophin-associated protein A1, 59kDa, basic component 2)	SNTB2
226188_at	0.24		
211407_at	0.24	NADH dehydrogenase (ubiquinone) 1 beta subcomplex, 7, 18kDa	NDUFB7
227948_at	0.24	FYVE, RhoGEF and PH domain containing 4	FGD4
240194_at	0.24		
239455_at	0.24		
225262_at	0.24	FOS-like antigen 2	FOSL2
236630_at	0.24	aquaporin 2 (collecting duct)	AQP2
232504_at	0.24		
222605_at	0.24	REST corepressor 3	RCOR3
1560692_at	0.24		
1562169_at	0.24		
218898_at	0.24	family with sequence similarity 57, member A	FAM57A
225685_at	0.24		
1561210_at	0.24		
60471_at	0.24	Ras and Rab interactor 3	RIN3
1562916_at	0.24		
237244_at	0.24		
203231_s_at	0.24	ataxin 1	ATXN1
1561892_at	0.24	zinc finger, MYM-type 6	ZMYM6

230733_at	0.24		
209939_x_at	0.24	CASP8 and FADD-like apoptosis regulator	CFLAR
204495_s_at	0.24	chromosome 15 open reading frame 39	C15orf39
236874_at	0.23		
229390_at	0.23		
222154_s_at	0.23		
235479_at	0.23	cytoplasmic polyadenylation element binding protein 2	CPEB2
204215_at	0.23	chromosome 7 open reading frame 23	C7orf23
203317_at	0.23	pleckstrin and Sec7 domain containing 4	PSD4
219898_at	0.23	G protein-coupled receptor 85	GPR85
219457_s_at	0.23	Ras and Rab interactor 3	RIN3
243797_at	0.23	serine/threonine kinase 17b (apoptosis-inducing)	STK17B
226460_at	0.23		
212647_at	0.23	related RAS viral (r-ras) oncogene homolog	RRAS
219317_at	0.23	polymerase (DNA directed) iota	POLI
204088_at	0.23	purinergic receptor P2X, ligand-gated ion channel, 4	P2RX4
224650_at	0.23	mal, T-cell differentiation protein 2	MAL2
226322_at	0.23	transmembrane and tetratricopeptide repeat containing 1	TMTC1
230440_at	0.23	zinc finger protein 469	ZNF469
40420_at	0.23	serine/threonine kinase 10	STK10
210845_s_at	0.23	plasminogen activator, urokinase receptor	PLAUR
211778_s_at	0.23	ovo-like 2 (Drosophila)	OVOL2
230718_at	0.23	heat shock transcription factor family member 5	HSF5
205935_at	0.23	forkhead box F1	FOXF1
225265_at	0.23	RNA binding motif, single stranded interacting protein 1	RBMS1
212642_s_at	0.23	human immunodeficiency virus type I enhancer binding protein 2	HIVEP2
1560823_at	0.23		
209011_at	0.23	triple functional domain (PTPRF interacting)	TRIO
223514_at	0.23	caspase recruitment domain family, member 11	CARD11
203695_s_at	0.23	deafness, autosomal dominant 5	DFNA5
223162_s_at	0.23	KIAA1147	KIAA1147
1554593_s_at	0.23	solute carrier family 1 (high affinity aspartate/glutamate transporter), member 6	SLC1A6
214618_at	0.23	CASP8 and FADD-like apoptosis regulator	CFLAR
1556332_at	0.23		
222463_s_at	0.23	beta-site APP-cleaving enzyme 1	BACE1
214352_s_at	0.23	v-Ki-ras2 Kirsten rat sarcoma viral oncogene homolog	KRAS
225562_at	0.23	RAS p21 protein activator 3	RASA3
243396_at	0.23		
212959_s_at	0.23	N-acetylglucosamine-1-phosphate transferase, alpha and beta subunits	GNPTAB
238992_at	0.23	polymerase (DNA directed) iota	POLI
202762_at	0.23	Rho-associated, coiled-coil containing protein kinase 2	ROCK2
235264_at	0.23		
213766_x_at	0.23	guanine nucleotide binding protein (G protein), alpha 11 (Gq class)	GNA11

212820_at	0.23	Dmx-like 2	DMXL2
203037_s_at	0.23	metastasis suppressor 1	MTSS1
236436_at	0.23	solute carrier family 25, member 45	SLC25A45
65438_at	0.23	KIAA1609	KIAA1609
217628_at	0.23	chloride intracellular channel 5	CLIC5
209722_s_at	0.23	serpin peptidase inhibitor, clade B (ovalbumin), member 9	SERPINB9
204436_at	0.23	pleckstrin homology domain containing, family Q member 1	PLEKHQ1
235094_at	0.23		
206600_s_at	0.23	solute carrier family 16, member 5 (monocarboxylic acid transporter 6)	SLC16A5
229134_at	0.23	vang-like 1 (van gogh, Drosophila)	VANGL1
243495_s_at	0.23		
201964_at	0.23	senataxin	SETX
201367_s_at	0.23	zinc finger protein 36, C3H type-like 2	ZFP36L2
229336_at	0.23		
206961_s_at	0.23	Trf (TATA binding protein-related factor)-proximal homolog (Drosophila)	TRFP
206669_at	0.23	glutamate decarboxylase 1 (brain, 67kDa)	GAD1
201110_s_at	0.23	thrombospondin 1	THBS1
221755_at	0.23	EH domain binding protein 1-like 1	EHBP1L1
204689_at	0.23	homeobox, hematopoietically expressed	HHEX
212660_at	0.23	PHD finger protein 15	PHF15
209541_at	0.23		
228242_at	0.23		
205192_at	0.23	mitogen-activated protein kinase kinase kinase 14	MAP3K14
203490_at	0.23	E74-like factor 4 (ets domain transcription factor)	ELF4
209304_x_at	0.23	growth arrest and DNA-damage-inducible, beta	GADD45B
235964_x_at	0.23	chromosome 20 open reading frame 118	C20orf118
210563_x_at	0.23	CASP8 and FADD-like apoptosis regulator	CFLAR
1557305_at	0.23	transforming, acidic coiled-coil containing protein 1	TACC1
214994_at	0.23	apolipoprotein B mRNA editing enzyme, catalytic polypeptide-like 3F	APOBEC3F
211067_s_at	0.23	growth arrest-specific 7	GAS7
201300_s_at	0.23	prion protein (p27-30) (Creutzfeldt-Jakob disease, Gerstmann-Strausler-Scheinker syndrome, fatal fam	PRNP
223132_s_at	0.23	tripartite motif-containing 8	TRIM8
204009_s_at	0.23	v-Ki-ras2 Kirsten rat sarcoma viral oncogene homolog	KRAS
212688_at	0.23	phosphoinositide-3-kinase, catalytic, beta polypeptide	PIK3CB
238790_at	0.22		
1553071_a_at	0.22	myozenin 3	MYOZ3
209435_s_at	0.22	rho/rac guanine nucleotide exchange factor (GEF) 2	ARHGEF2
207129_at	0.22	carbonic anhydrase VB, mitochondrial	CA5B
1553151_at	0.22	ATPase, H+ transporting, lysosomal 38kDa, V0 subunit d2	ATP6V0D2
238722_x_at	0.22		

225763_at	0.22	RCSD domain containing 1	RCSD1
224983_at	0.22	scavenger receptor class B, member 2	SCARB2
227107_at	0.22		
207643_s_at	0.22	tumor necrosis factor receptor superfamily, member 1A	TNFRSF1A
225922_at	0.22		
205051_s_at	0.22	v-kit Hardy-Zuckerman 4 feline sarcoma viral oncogene homolog	KIT
227361_at	0.22	heparan sulfate (glucosamine) 3-O-sulfotransferase 3B1	HS3ST3B1
241889_at	0.22		
1556465_at	0.22		
203331_s_at	0.22	inositol polyphosphate-5-phosphatase, 145kDa	INPP5D
213150_at	0.22	homeobox A10	HOXA10
238029_s_at	0.22	solute carrier family 16, member 14 (monocarboxylic acid transporter 14)	SLC16A14
219278_at	0.22	mitogen-activated protein kinase kinase kinase 6	MAP3K6
237569_at	0.22		
232480_at	0.22		
229686_at	0.22	purinergic receptor P2Y, G-protein coupled, 8	P2RY8
1559810_at	0.22		
210258_at	0.22	regulator of G-protein signalling 13	RGS13
238846_at	0.22	tumor necrosis factor receptor superfamily, member 11a, NFKB activator	TNFRSF11A
210041_s_at	0.22	phosphoglucomutase 3	PGM3
240533_at	0.22		
224917_at	0.22	transmembrane protein 49	TMEM49
204166_at	0.22	strawberry notch homolog 2 (Drosophila)	SBNO2
238909_at	0.22	S100 calcium binding protein A10	S100A10
201368_at	0.22	zinc finger protein 36, C3H type-like 2	ZFP36L2
206672_at	0.22	aquaporin 2 (collecting duct)	AQP2
243541_at	0.22	interleukin 31 receptor A	IL31RA
1556682_s_at	0.22		
209446_s_at	0.22	chromosome 7 open reading frame 44	C7orf44
203274_at	0.22	coagulation factor VIII-associated (intronic transcript) 1	F8A1
204063_s_at	0.22	unc-51-like kinase 2 (C. elegans)	ULK2
236729_at	0.22	caspase 3, apoptosis-related cysteine peptidase	CASP3
236293_at	0.22		
203879_at	0.22	phosphoinositide-3-kinase, catalytic, delta polypeptide	PIK3CD
237516_at	0.22		
214377_s_at	0.22	chymotrypsin-like	CTRL
57715_at	0.22	family with sequence similarity 26, member B	FAM26B
204794_at	0.22	dual specificity phosphatase 2	DUSP2
222838_at	0.22	CD48 molecule#lymphocyte antigen 9#signaling lymphocytic activation molecule family member 1#SLAM family member 7	CD48#LY9#SLAMF1#SLAMF7
230741_at	0.22		
1566764_at	0.22		
1568638_a_at	0.22	indoleamine-pyrrole 2,3 dioxygenase-like 1	INDOL1
204672_s_at	0.22	ankyrin repeat domain 6	ANKRD6
209946_at	0.22	vascular endothelial growth factor C	VEGFC

226641_at	0.22		
226100_at	0.22	myeloid/lymphoid or mixed-lineage leukemia 5 (trithorax homolog, Drosophila)	MLL5
1564338_at	0.22		
204472_at	0.22	GTP binding protein overexpressed in skeletal muscle	GEM
201631_s_at	0.22	immediate early response 3	IER3
226691_at	0.22		
208303_s_at	0.22	cytokine receptor-like factor 2	CRLF2
226893_at	0.22		
212774_at	0.22	zinc finger protein 238	ZNF238
238327_at	0.22		
239953_at	0.22		
226202_at	0.22	zinc finger protein 398	ZNF398
238778_at	0.22	membrane protein, palmitoylated 7 (MAGUK p55 subfamily member 7)	MPP7
205511_at	0.22		
218810_at	0.21	zinc finger CCCH-type containing 12A	ZC3H12A
202171_at	0.21	vascular endothelial zinc finger 1	VEZF1
236454_at	0.21	ring finger protein 212	RNF212
1552266_at	0.21	ADAM metallopeptidase domain 32	ADAM32
228714_at	0.21		
229841_at	0.21	eukaryotic translation initiation factor 2C, 2	EIF2C2
1555332_at	0.21	tryptophan hydroxylase 2	TPH2
1570021_at	0.21		
235899_at	0.21	carbonic anhydrase XIII	CA13
210785_s_at	0.21	chromosome 1 open reading frame 38	C1orf38
222062_at	0.21	interleukin 27 receptor, alpha	IL27RA
218029_at	0.21	family with sequence similarity 65, member A	FAM65A
216764_at	0.21		
229733_s_at	0.21		
239213_at	0.21	serpin peptidase inhibitor, clade B (ovalbumin), member 1	SERPINB1
204038_s_at	0.21	endothelial differentiation, lysophosphatidic acid G-protein-coupled receptor, 2	EDG2
225569_at	0.21	eukaryotic translation initiation factor 2C, 2	EIF2C2
203006_at	0.21	inositol polyphosphate-5-phosphatase, 40kDa	INPP5A
239058_at	0.21		
223027_at	0.21	sorting nexin 9	SNX9
1569703_a_at	0.21		
226333_at	0.21		
202074_s_at	0.21	optineurin	OPTN
227410_at	0.21	family with sequence similarity 43, member A	FAM43A
217635_s_at	0.21	polymerase (DNA directed), gamma	POLG
209403_at	0.21	TBC1 domain family, member 3	TBC1D3
218699_at	0.21	RAB7, member RAS oncogene family-like 1	RAB7L1
209124_at	0.21	myeloid differentiation primary response gene (88)	MYD88
239752_at	0.21		
226240_at	0.21		
204036_at	0.21	endothelial differentiation, lysophosphatidic acid G-protein-coupled receptor, 2	EDG2
218651_s_at	0.21	La ribonucleoprotein domain family, member 6	LARP6

217529_at	0.21		
202206_at	0.21	ADP-ribosylation factor-like 4C	ARL4C
237819_at	0.21	cAMP responsive element binding protein 3-like 2	CREB3L2
1555311_at	0.21		
232539_at	0.21		
223322_at	0.21	Ras association (RalGDS/AF-6) domain family 5	RASSF5
226939_at	0.21	cytoplasmic polyadenylation element binding protein 2	CPEB2
221571_at	0.21	TNF receptor-associated factor 3	TRAF3
222496_s_at	0.21		
211924_s_at	0.21	plasminogen activator, urokinase receptor	PLAUR
206636_at	0.21	RAS p21 protein activator 2	RASA2
1554866_at	0.21	transmembrane protein 135	TMEM135
236104_at	0.21		
219257_s_at	0.21	sphingosine kinase 1	SPHK1
1555847_a_at	0.21		
230050_at	0.21	BTB (POZ) domain containing 14A	BTBD14A
235670_at	0.21		
236295_s_at	0.21	NLR family, CARD domain containing 3	NLRC3
235306_at	0.21	GTPase, IMAP family member 8	GIMAP8
235458_at	0.21	hepatitis A virus cellular receptor 2	HAVCR2
242635_s_at	0.21		
229090_at	0.21		
211744_s_at	0.21	CD58 molecule	CD58
209764_at	0.21	activating transcription factor 4 (tax-responsive enhancer element B67)#mannosyl (beta-1,4-)-glycoprotein beta-1,4-N-acetylglucosaminyltransferase#calcium channel, voltage-dependent, alpha 1I subunit#Smith-Magenis syndrome chromosome region, candidate 7-like#Smith-Magenis syndrome chromosome region, candidate 7-like#ribosomal protein S19 binding protein 1	ATF4#MGAT3#CACNA1I#SMCR7L#SMCR7L#RPS19BP1
206283_s_at	0.21	T-cell acute lymphocytic leukemia 1	TAL1
1559889_at	0.21		
238432_at	0.21		
203324_s_at	0.21	caveolin 2	CAV2
217936_at	0.21	Rho GTPase activating protein 5	ARHGAP5
230154_at	0.21		
229373_at	0.21		
203839_s_at	0.21	tyrosine kinase, non-receptor, 2	TNK2
227609_at	0.21	epithelial stromal interaction 1 (breast)	EPSTI1
230052_s_at	0.21		
230276_at	0.21	family with sequence similarity 49, member A	FAM49A
207124_s_at	0.21	guanine nucleotide binding protein (G protein), beta 5	GNB5
224793_s_at	0.21	transforming growth factor, beta receptor I (activin A receptor type II-like kinase, 53kDa)	TGFBR1
223963_s_at	0.21	insulin-like growth factor 2 mRNA binding protein 2	IGF2BP2
240232_at	0.21	chromosome 3 open reading frame 1	C3orf1
212811_x_at	0.21	solute carrier family 1 (glutamate/neutral amino acid transporter), member 4	SLC1A4

226978_at	0.21	peroxisome proliferator-activated receptor alpha	PPARA
207091_at	0.21	purinergic receptor P2X, ligand-gated ion channel, 7	P2RX7
226075_at	0.21	splA/ryanodine receptor domain and SOCS box containing 1	SPSB1
1556941_a_at	0.21		
234668_at	0.21		
225328_at	0.21		
221878_at	0.21		
242250_at	0.21		
233261_at	0.21	early B-cell factor 1	EBF1
50221_at	0.21	transcription factor EB	TFEB
237753_at	0.21		
230170_at	0.21	oncostatin M	OSM
226490_at	0.21	NHS-like 1	NHSL1
219454_at	0.21	EGF-like-domain, multiple 6	EGFL6
216834_at	0.21	regulator of G-protein signalling 1	RGS1
225273_at	0.20	WWC family member 3	WWC3
204698_at	0.20	interferon stimulated exonuclease gene 20kDa	ISG20
200999_s_at	0.20	cytoskeleton-associated protein 4	CKAP4
235527_at	0.20		
229876_at	0.20	phosphorylase kinase, alpha 1 (muscle)	PHKA1
1559064_at	0.20	nucleoporin 153kDa	NUP153
207266_x_at	0.20	RNA binding motif, single stranded interacting protein 1	RBMS1
1555729_a_at	0.20	CD209 molecule	CD209
219041_s_at	0.20	replication initiator 1	REPIN1
218368_s_at	0.20	tumor necrosis factor receptor superfamily, member 12A	TNFRSF12A
224764_at	0.20	Rho GTPase activating protein 21	ARHGAP21
233636_at	0.20	microRNA host gene (non-protein coding) 1	MIRH1
200758_s_at	0.20	nuclear factor (erythroid-derived 2)-like 1	NFE2L1
224925_at	0.20		
212810_s_at	0.20	solute carrier family 1 (glutamate/neutral amino acid transporter), member 4	SLC1A4
211030_s_at	0.20	solute carrier family 6 (neurotransmitter transporter, taurine), member 6	SLC6A6
204790_at	0.20	SMAD family member 7	SMAD7
202192_s_at	0.20	growth arrest-specific 7	GAS7
210136_at	0.20	myelin basic protein	MBP
216598_s_at	0.20	chemokine (C-C motif) ligand 2	CCL2
204352_at	0.20	TNF receptor-associated factor 5	TRAF5
227388_at	0.20	tumor suppressor candidate 1	TUSC1
209824_s_at	0.20	aryl hydrocarbon receptor nuclear translocator-like	ARNTL
208047_s_at	0.20	NGFI-A binding protein 1 (EGR1 binding protein 1)	NAB1
214082_at	0.20	carbonic anhydrase VB, mitochondrial	CA5B
224923_at	0.20	tetratricopeptide repeat domain 7A	TTC7A
230482_at	0.20	ST6 (alpha-N-acetyl-neuraminyl-2,3-beta-galactosyl-1,3)-N-acetylgalactosaminide alpha-2,6-sialyltran	ST6GALNAC5
232017_at	0.20	tight junction protein 2 (zona occludens 2)	TJP2

1553153_at	0.20	ATPase, H+ transporting, lysosomal 38kDa, V0 subunit d2	ATP6V0D2
218812_s_at	0.20	transmembrane protein 142B	TMEM142B
1553232_at	0.20	family with sequence similarity 82, member A	FAM82A
201169_s_at	0.20	basic helix-loop-helix domain containing, class B, 2	BHLHB2
209828_s_at	0.20	interleukin 16 (lymphocyte chemoattractant factor)	IL16
225803_at	0.20	F-box protein 32	FBXO32
209409_at	0.20	growth factor receptor-bound protein 10	GRB10
241879_at	0.20		
230786_at	0.20		
201626_at	0.20	insulin induced gene 1	INSIG1
235421_at	0.20		
202565_s_at	0.20	supervillin	SVIL
226423_at	0.20	progestin and adipoQ receptor family member VIII	PAQR8
205440_s_at	0.20	neuropeptide Y receptor Y1	NPY1R
204401_at	0.20	potassium intermediate/small conductance calcium-activated channel, subfamily N, member 4	KCNN4
212419_at	0.20	chromosome 10 open reading frame 56	C10orf56
229391_s_at	0.20		
52975_at	0.20	family with sequence similarity 125, member B	FAM125B
31874_at	0.20	growth arrest-specific 2 like 1	GAS2L1
210271_at	0.20	neurogenic differentiation 2	NEUROD2
209163_at	0.20	cytochrome b-561	CYB561
224832_at	0.20	dual specificity phosphatase 16	DUSP16
202628_s_at	0.20	serpin peptidase inhibitor, clade E (nexin, plasminogen activator inhibitor type 1), member 1	SERPINE1
209610_s_at	0.20	solute carrier family 1 (glutamate/neutral amino acid transporter), member 4	SLC1A4
235626_at	0.20	calcium/calmodulin-dependent protein kinase ID	CAMK1D
230563_at	0.20	RasGEF domain family, member 1A	RASGEF1A
212099_at	0.20	ras homolog gene family, member B	RHOB
1561864_at	0.20		
202172_at	0.19	vascular endothelial zinc finger 1	VEZF1
222449_at	0.19	phosphoenolpyruvate carboxykinase 1 (soluble)#transmembrane, prostate androgen induced RNA#Z-DNA binding protein 1#CCCTC-binding factor (zinc finger protein)-like	PCK1#TMEPAI #ZBP1#CTCFL
242229_at	0.19		
215933_s_at	0.19	homeobox, hematopoietically expressed	HHEX
1569150_x_at	0.19	PDZ and LIM domain 7 (enigma)	PDLIM7
219456_s_at	0.19	Ras and Rab interactor 3	RIN3
244053_at	0.19	ATP-binding cassette, sub-family C (CFTR/MRP), member 4	ABCC4
230053_at	0.19		
1569872_a_at	0.19		
219806_s_at	0.19	chromosome 11 open reading frame 75	C11orf75
242917_at	0.19	RasGEF domain family, member 1A	RASGEF1A
228791_at	0.19	chromosome 15 open reading frame 38	C15orf38

244481_at	0.19	solute carrier family 25 (mitochondrial carrier; phosphate carrier), member 24	SLC25A24
208683_at	0.19	calpain 2, (m/II) large subunit	CAPN2
215431_at	0.19	syntrophin, beta 1 (dystrophin-associated protein A1, 59kDa, basic component 1)	SNTB1
224565_at	0.19		
202085_at	0.19	tight junction protein 2 (zona occludens 2)	TJP2
206209_s_at	0.19	carbonic anhydrase IV	CA4
230104_s_at	0.19		
212397_at	0.19	radixin	RDX
203748_x_at	0.19	RNA binding motif, single stranded interacting protein 1	RBMS1
213038_at	0.19	IBR domain containing 3	IBRDC3
1556064_at	0.19		
203760_s_at	0.19	Src-like-adaptor	SLA
1563346_at	0.19		
203851_at	0.19	insulin-like growth factor binding protein 6	IGFBP6
203741_s_at	0.19	adenylate cyclase 7	ADCY7
207571_x_at	0.19	chromosome 1 open reading frame 38	C1orf38
203754_s_at	0.19	BRF1 homolog, subunit of RNA polymerase III transcription initiation factor IIIB (S. cerevisiae)	BRF1
228724_at	0.19		
221866_at	0.19	MyoD family inhibitor#transcription factor EB	MDFI#TFEB
209573_s_at	0.19	chromosome 18 open reading frame 1	C18orf1
200782_at	0.19	annexin A5	ANXA5
201860_s_at	0.19	plasminogen activator, tissue	PLAT
212923_s_at	0.19	chromosome 6 open reading frame 145	C6orf145
1563629_a_at	0.19		
204802_at	0.19	Ras-related associated with diabetes	RRAD
1555938_x_at	0.19	vimentin	VIM
218964_at	0.19	AT rich interactive domain 3B (BRIGHT-like)	ARID3B
221752_at	0.19	slingshot homolog 1 (Drosophila)	SSH1
229157_at	0.19		
238861_at	0.19		
1570622_at	0.19		
214671_s_at	0.19	active BCR-related gene	ABR
224566_at	0.19		
222453_at	0.19	cytochrome b reductase 1	CYBRD1
241218_at	0.19		
228426_at	0.19	C-type lectin domain family 2, member D	CLEC2D
209723_at	0.19	serpin peptidase inhibitor, clade B (ovalbumin), member 9	SERPINB9
225971_at	0.19		
204058_at	0.19	malic enzyme 1, NADP(+)-dependent, cytosolic#phosphoglucomutase 3#RWD domain containing 2	ME1#PGM3#RWDD2
233627_at	0.18		
201627_s_at	0.18	insulin induced gene 1	INSIG1
225305_at	0.18	solute carrier family 25, member 29	SLC25A29
207113_s_at	0.18	tumor necrosis factor (TNF superfamily, member 2)	TNF
229396_at	0.18	ovo-like 1(Drosophila)	OVOL1

216509_x_at	0.18	myeloid/lymphoid or mixed-lineage leukemia (trithorax homolog, Drosophila); translocated to, 10	MLLT10
235344_at	0.18		
224570_s_at	0.18		
202342_s_at	0.18	tripartite motif-containing 2	TRIM2
207574_s_at	0.18	growth arrest and DNA-damage-inducible, beta	GADD45B
232077_s_at	0.18		
212292_at	0.18	solute carrier family 7 (cationic amino acid transporter, y+ system), member 1	SLC7A1
218532_s_at	0.18		
230192_at	0.18	tripartite motif-containing 13	TRIM13
1561252_at	0.18		
204912_at	0.18	interleukin 10 receptor, alpha	IL10RA
1569569_x_at	0.18		
205032_at	0.18	integrin, alpha 2 (CD49B, alpha 2 subunit of VLA-2 receptor)	ITGA2
201531_at	0.18	zinc finger protein 36, C3H type, homolog (mouse)	ZFP36
206618_at	0.18	interleukin 18 receptor 1	IL18R1
219344_at	0.18	solute carrier family 29 (nucleoside transporters), member 3	SLC29A3
218764_at	0.18	protein kinase C, eta	PRKCH
212124_at	0.18	zinc finger, MIZ-type containing 1	ZMIZ1
55081_at	0.18	MICAL-like 1	MICALL1
233227_at	0.18	KIAA1109	KIAA1109
1553031_at	0.18	G protein-coupled receptor 115	GPR115
31845_at	0.18	E74-like factor 4 (ets domain transcription factor)	ELF4
229543_at	0.18		
1555486_a_at	0.18		
210117_at	0.18	sperm associated antigen 1	SPAG1
205945_at	0.18	interleukin 6 receptor	IL6R
212068_s_at	0.18	KIAA0515	KIAA0515
205158_at	0.18	ribonuclease, RNase A family, 4	RNASE4
201455_s_at	0.18	aminopeptidase puromycin sensitive	NPEPPS
222942_s_at	0.18	T-cell lymphoma invasion and metastasis 2	TIAM2
1565755_at	0.18		
213868_s_at	0.18	dehydrogenase/reductase (SDR family) member 7	DHRS7
230805_at	0.18		
217591_at	0.18		
229776_at	0.18	solute carrier organic anion transporter family, member 3A1	SLCO3A1
236280_at	0.18		
229687_s_at	0.18		
221451_s_at	0.18	olfactory receptor, family 2, subfamily W, member 1	OR2W1
203521_s_at	0.18	zinc finger protein 318	ZNF318
238692_at	0.18	BTB (POZ) domain containing 11	BTBD11
226991_at	0.18	nuclear factor of activated T-cells, cytoplasmic, calcineurin-dependent 2	NFATC2
224663_s_at	0.17	cofilin 2 (muscle)	CFL2

235518_at	0.17	solute carrier family 8 (sodium/calcium exchanger), member 1	SLC8A1
222597_at	0.17	synaptosomal-associated protein, 29kDa	SNAP29
1554844_at	0.17	eyes absent homolog 3 (Drosophila)	EYA3
214321_at	0.17	nephroblastoma overexpressed gene	NOV
228442_at	0.17		
1569257_at	0.17	formin-like 1	FMNL1
212364_at	0.17	myosin IB	MYO1B
203665_at	0.17	heme oxygenase (decycling) 1	HMOX1
46665_at	0.17	sema domain, immunoglobulin domain (Ig), transmembrane domain (TM) and short cytoplasmic domain, (se	SEMA4C
224989_at	0.17		
229033_s_at	0.17	melanoma associated antigen (mutated) 1	MUM1
223950_s_at	0.17	FLYWCH-type zinc finger 1	FLYWCH1
206337_at	0.17	chemokine (C-C motif) receptor 7	CCR7
227792_at	0.17		
220118_at	0.17	zinc finger and BTB domain containing 32	ZBTB32
223159_s_at	0.17	NIMA (never in mitosis gene a)-related kinase 6	NEK6
230252_at	0.17	G protein-coupled receptor 92	GPR92
233362_at	0.17	zinc finger protein 341	ZNF341
241416_at	0.17		
201642_at	0.17	interferon gamma receptor 2 (interferon gamma transducer 1)	IFNGR2
224569_s_at	0.17	interferon regulatory factor 2 binding protein 2	IRF2BP2
229188_s_at	0.17	zinc and ring finger 2	ZNRF2
1565863_at	0.17		
207196_s_at	0.17	TNFAIP3 interacting protein 1	TNIP1
211667_x_at	0.17		
219383_at	0.17		
234986_at	0.17		
241900_at	0.17		
234455_at	0.17	zinc finger protein 1 homolog (mouse)	ZFP1
1562644_at	0.17	methylenetetrahydrofolate dehydrogenase (NADP+ dependent) 2-like	MTHFD2L
235382_at	0.17		
205173_x_at	0.17	CD58 molecule	CD58
219680_at	0.17	NLR family member X1	NLRX1
203047_at	0.17	serine/threonine kinase 10	STK10
209959_at	0.17	nuclear receptor subfamily 4, group A, member 3	NR4A3
228325_at	0.17		
202437_s_at	0.17	cytochrome P450, family 1, subfamily B, polypeptide 1	CYP1B1
212993_at	0.17		
221658_s_at	0.17	interleukin 21 receptor	IL21R
231283_at	0.17	mannosyl (alpha-1,3-)-glycoprotein beta-1,4-N-acetylglucosaminyltransferase, isozyme A	MGAT4A
202627_s_at	0.17	serpin peptidase inhibitor, clade E (nexin, plasminogen activator inhibitor type 1), member 1	SERPINE1
205081_at	0.17	cysteine-rich protein 1 (intestinal)	CRIP1
231406_at	0.17		
201473_at	0.17	jun B proto-oncogene	JUNB

205590_at	0.17	RAS guanyl releasing protein 1 (calcium and DAG-regulated)	RASGRP1
228471_at	0.17	ankyrin repeat domain 44	ANKRD44
209925_at	0.17	occludin	OCLN
236826_at	0.17	chromosome 9 open reading frame 52	C9orf52
212464_s_at	0.17	fibronectin 1	FN1
233771_at	0.17	triple functional domain (PTPRF interacting)	TRIO
202191_s_at	0.17	growth arrest-specific 7	GAS7
203234_at	0.17	uridine phosphorylase 1	UPP1
206552_s_at	0.16	tachykinin, precursor 1 (substance K, substance P, neurokinin 1, neurokinin 2, neuromedin L, neuroki	TAC1
243242_at	0.16		
218978_s_at	0.16	solute carrier family 25, member 37	SLC25A37
1556487_a_at	0.16	chromosome 3 open reading frame 15	C3orf15
203761_at	0.16	Src-like-adaptor	SLA
222842_at	0.16	eukaryotic translation initiation factor 2C, 4	EIF2C4
243124_at	0.16		
1562780_at	0.16		
235299_at	0.16		
227582_at	0.16		
225618_at	0.16	Rho GTPase activating protein 27	ARHGAP27
1552986_at	0.16		
227367_at	0.16		
228754_at	0.16	solute carrier family 6 (neurotransmitter transporter, taurine), member 6	SLC6A6
226504_at	0.16	family with sequence similarity 109, member B	FAM109B
1556423_at	0.16	vasohibin 1	VASH1
220326_s_at	0.16		
1557233_at	0.16		
204446_s_at	0.16	arachidonate 5-lipoxygenase	ALOX5
1552972_at	0.16		
215617_at	0.16		
209930_s_at	0.16	nuclear factor (erythroid-derived 2), 45kDa	NFE2
1568619_s_at	0.16		
1563725_at	0.16	zinc finger protein 583	ZNF583
220985_s_at	0.16	ring finger protein 170	RNF170
236248_x_at	0.16		
225136_at	0.16	pleckstrin homology domain containing, family A (phosphoinositide binding specific) member 2	PLEKHA2
230980_x_at	0.16		
203131_at	0.16	platelet-derived growth factor receptor, alpha polypeptide	PDGFRA
205027_s_at	0.16	mitogen-activated protein kinase kinase kinase 8	MAP3K8
213222_at	0.16	phospholipase C, beta 1 (phosphoinositide-specific)	PLCB1
203845_at	0.16	p300/CBP-associated factor	PCAF
212108_at	0.16	UBX domain containing 8	UBXD8
227236_at	0.16	tetraspanin 2	TSPAN2
226041_at	0.16		
226055_at	0.16	arrestin domain containing 2	ARRDC2

227015_at	0.16	tuftelin interacting protein 11#aspartate beta-hydroxylase domain containing 2#Hermansky-Pudlak syndrome 4#null#null	TFIP11#ASPHD2#HPS4#null#null
223520_s_at	0.16	kinesin family member 13A	KIF13A
222162_s_at	0.16	ADAM metallopeptidase with thrombospondin type 1 motif, 1	ADAMTS1
217650_x_at	0.16	ST3 beta-galactoside alpha-2,3-sialyltransferase 2	ST3GAL2
226438_at	0.16		
1554592_a_at	0.16	solute carrier family 1 (high affinity aspartate/glutamate transporter), member 6	SLC1A6
227391_x_at	0.16	leucine rich repeat (in FLII) interacting protein 1	LRRFIP1
205120_s_at	0.16	sarcoglycan, beta (43kDa dystrophin-associated glycoprotein)	SGCB
202435_s_at	0.15		
213361_at	0.15	tudor domain containing 7	TDRD7
232607_at	0.15		
228145_s_at	0.15	zinc finger protein 398	ZNF398
202073_at	0.15	optineurin	OPTN
221788_at	0.15		
234994_at	0.15	KIAA1913	KIAA1913
1559149_at	0.15		
218723_s_at	0.15		
228394_at	0.15	serine/threonine kinase 10	STK10
239156_at	0.15		
211748_x_at	0.15	prostaglandin D2 synthase 21kDa (brain)	PTGDS
224571_at	0.15	interferon regulatory factor 2 binding protein 2	IRF2BP2
223179_at	0.15	yippee-like 3 (Drosophila)	YPEL3
213376_at	0.15	zinc finger and BTB domain containing 1	ZBTB1
229354_at	0.15	aryl-hydrocarbon receptor repressor	AHRR
201809_s_at	0.15	endoglin (Osler-Rendu-Weber syndrome 1)	ENG
228923_at	0.15	S100 calcium binding protein A6	S100A6
240783_at	0.15		
232573_at	0.15		
226084_at	0.15	microtubule-associated protein 1B	MAP1B
236285_at	0.15		
242321_at	0.15		
227984_at	0.15		
223767_at	0.15	G protein-coupled receptor 84	GPR84
223468_s_at	0.15	RGM domain family, member A	RGMA
212618_at	0.15	zinc finger protein 609	ZNF609
221790_s_at	0.15	low density lipoprotein receptor adaptor protein 1	LDLRAP1
1562403_a_at	0.15	solute carrier family 8 (sodium-calcium exchanger), member 3	SLC8A3
227131_at	0.15	mitogen-activated protein kinase kinase kinase 3	MAP3K3
225407_at	0.15	myelin basic protein	MBP
229728_at	0.15		
229914_at	0.15		
224990_at	0.15	chromosome 4 open reading frame 34	C4orf34
202393_s_at	0.15	Kruppel-like factor 10	KLF10
205180_s_at	0.15	ADAM metallopeptidase domain 8	ADAM8
235534_at	0.15		

234514_at	0.15	eukaryotic translation elongation factor 1 alpha 2#potassium voltage-gated channel, KQT-like subfamily, member 2#potassium voltage-gated channel, KQT-like subfamily, member 2#PTK6 protein tyrosine kinase 6#src-related kinase lacking C-terminal regulatory tyrosine and N-terminal myristylation sites#chromosome 20 open reading frame 195#chromosome 20 open reading frame 149#null	EEF1A2#KCNQ2#KCNQ2#PTK6#SRMS#C20orf195#C20orf149#null
242445_at	0.15	FYVE, RhoGEF and PH domain containing 4	FGD4
1553787_at	0.15	chromosome 11 open reading frame 45	C11orf45
209374_s_at	0.15	immunoglobulin heavy constant mu	IGHM
1556486_at	0.15		
243711_at	0.15	dimethylarginine dimethylaminohydrolase 1	DDAH1
205279_s_at	0.15	glycine receptor, beta	GLRB
228042_at	0.15	ADP-ribosylarginine hydrolase	ADPRH
213172_at	0.15	tetratricopeptide repeat domain 9	TTC9
200872_at	0.15	S100 calcium binding protein A10	S100A10
217849_s_at	0.15	CDC42 binding protein kinase beta (DMPK-like)	CDC42BPB
241396_at	0.15	neural precursor cell expressed, developmentally down-regulated 4-like	NEDD4L
215945_s_at	0.14	tripartite motif-containing 2	TRIM2
210689_at	0.14	claudin 14	CLDN14
226056_at	0.14		
202913_at	0.14	Rho guanine nucleotide exchange factor (GEF) 11	ARHGEF11
201945_at	0.14	furin (paired basic amino acid cleaving enzyme)	FURIN
226382_at	0.14		
210941_at	0.14	BH-protocadherin (brain-heart)	PCDH7
228054_at	0.14	transmembrane protein 44	TMEM44
228188_at	0.14	FOS-like antigen 2	FOSL2
227314_at	0.14	integrin, alpha 2 (CD49B, alpha 2 subunit of VLA-2 receptor)	ITGA2
204475_at	0.14	matrix metallopeptidase 1 (interstitial collagenase)	MMP1
219159_s_at	0.14	SLAM family member 7	SLAMF7
211434_s_at	0.14	chemokine (C-C motif) receptor-like 2	CCRL2
225980_at	0.14	chromosome 14 open reading frame 43	C14orf43
227439_at	0.14	ankyrin repeat and sterile alpha motif domain containing 1B	ANKS1B
1559035_a_at	0.14	aryl hydrocarbon receptor	AHR
201189_s_at	0.14	inositol 1,4,5-triphosphate receptor, type 3	ITPR3
1552334_at	0.14	TRIO and F-actin binding protein	TRIOBP
231779_at	0.14	interleukin-1 receptor-associated kinase 2	IRAK2
216740_at	0.14		
237597_at	0.14		
227233_at	0.14	tetraspanin 2	TSPAN2
237417_at	0.14	regulator of chromosome condensation (RCC1) and BTB (POZ) domain containing protein 1	RCBTB1
203232_s_at	0.14	ataxin 1	ATXN1
1553558_at	0.14	taste receptor, type 2, member 41	TAS2R41
223228_at	0.14	leucine zipper, down-regulated in cancer 1-like	LDOC1L

240850_at	0.14	dual-specificity tyrosine-(Y)-phosphorylation regulated kinase 1A	DYRK1A
235199_at	0.14	ring finger protein 125	RNF125
235916_at	0.14	yippee-like 4 (Drosophila)	YPEL4
201332_s_at	0.14	signal transducer and activator of transcription 6, interleukin-4 induced	STAT6
204858_s_at	0.14	endothelial cell growth factor 1 (platelet-derived)	ECGF1
236553_at	0.14		
207765_s_at	0.14	KIAA1539	KIAA1539
207216_at	0.14	tumor necrosis factor (ligand) superfamily, member 8	TNFSF8
225214_at	0.14		
218404_at	0.14	sorting nexin 10	SNX10
244533_at	0.14		
220494_s_at	0.14		
201373_at	0.14	plectin 1, intermediate filament binding protein 500kDa	PLEC1
1555689_at	0.14	CD80 molecule	CD80
227923_at	0.14	SH3 and multiple ankyrin repeat domains 3	SHANK3
227697_at	0.14	suppressor of cytokine signaling 3	SOCS3
203320_at	0.14	SH2B adaptor protein 3	SH2B3
237105_at	0.13		
202616_s_at	0.13	methyl CpG binding protein 2 (Rett syndrome)	MECP2
225251_at	0.13	RAB24, member RAS oncogene family	RAB24
201369_s_at	0.13	zinc finger protein 36, C3H type-like 2	ZFP36L2
221534_at	0.13	chromosome 11 open reading frame 68	C11orf68
211241_at	0.13	annexin A2 pseudogene 3	ANXA2P3
216322_at	0.13	CD58 molecule	CD58
204198_s_at	0.13	runt-related transcription factor 3	RUNX3
230499_at	0.13		
237987_x_at	0.13	carbamoyl-phosphate synthetase 1, mitochondrial	CPS1
204420_at	0.13	FOS-like antigen 1	FOSL1
228889_at	0.13	chromosome 14 open reading frame 128	C14orf128
244654_at	0.13	myosin IG	MYO1G
227513_s_at	0.13	leucine rich repeat (in FLII) interacting protein 1	LRRFIP1
232725_s_at	0.13	membrane-spanning 4-domains, subfamily A, member 6A	MS4A6A
202357_s_at	0.13	complement factor B	CFB
221012_s_at	0.13	tripartite motif-containing 8	TRIM8
203927_at	0.13	nuclear factor of kappa light polypeptide gene enhancer in B-cells inhibitor, epsilon	NFKBIE
226249_at	0.13	sorting nexin family member 30	SNX30
225162_at	0.13		
208446_s_at	0.13	zinc finger, FYVE domain containing 9	ZFYVE9
204400_at	0.13	embryonal Fyn-associated substrate	EFS
230369_at	0.13	G protein-coupled receptor 161	GPR161
1559405_a_at	0.13	transient receptor potential cation channel, subfamily V, member 6	TRPV6
205466_s_at	0.13	heparan sulfate (glucosamine) 3-O-sulfotransferase 1	HS3ST1
220300_at	0.13	regulator of G-protein signalling 3	RGS3
227544_at	0.13	chromosome 14 open reading frame 83	C14orf83

241274_at	0.13		
211272_s_at	0.13	diacylglycerol kinase, alpha 80kDa	DGKA
218284_at	0.13	SMAD family member 3	SMAD3
213280_at	0.13	GTPase activating Rap/RanGAP domain-like 4	GARNL4
229691_at	0.13		
58780_s_at	0.13		
219701_at	0.13	tropomodulin 2 (neuronal)	TMOD2
217206_at	0.13		
213385_at	0.13	chimerin (chimaerin) 2	CHN2
223340_at	0.13	spastic paraplegia 3A (autosomal dominant)	SPG3A
35626_at	0.13	N-sulfoglucosamine sulfohydrolase (sulfamidase)	SGSH
240828_at	0.13		
204422_s_at	0.13	fibroblast growth factor 2 (basic)	FGF2
212606_at	0.12	WD repeat and FYVE domain containing 3	WDFY3
231732_at	0.12	sphingomyelin phosphodiesterase 3, neutral membrane (neutral sphingomyelinase II)	SMPD3
205242_at	0.12	chemokine (C-X-C motif) ligand 13 (B-cell chemoattractant)	CXCL13
1552757_s_at	0.12	chromosome 9 open reading frame 66	C9orf66
215818_at	0.12	nudix (nucleoside diphosphate linked moiety X)-type motif 7	NUDT7
228707_at	0.12	claudin 23	CLDN23
204942_s_at	0.12	aldehyde dehydrogenase 3 family, member B2	ALDH3B2
212617_at	0.12	zinc finger protein 609	ZNF609
200998_s_at	0.12	cytoskeleton-associated protein 4	CKAP4
210017_at	0.12	mucosa associated lymphoid tissue lymphoma translocation gene 1	MALT1
241762_at	0.12	F-box protein 32	FBXO32
235106_at	0.12	mastermind-like 2 (Drosophila)	MAML2
202436_s_at	0.12	cytochrome P450, family 1, subfamily B, polypeptide 1	CYP1B1
229972_at	0.12		
242316_at	0.12		
204197_s_at	0.12	runt-related transcription factor 3	RUNX3
1559117_at	0.12		
228362_s_at	0.12		
208343_s_at	0.12	nuclear receptor subfamily 5, group A, member 2	NR5A2
202052_s_at	0.12	retinoic acid induced 14	RAI14
223472_at	0.12	Wolf-Hirschhorn syndrome candidate 1	WHSC1
1553894_at	0.12	coiled-coil domain containing 122	CCDC122
219049_at	0.12		
1561553_at	0.12		
225227_at	0.12		
1564676_a_at	0.12		
205579_at	0.12	histamine receptor H1	HRH1
202071_at	0.12	syndecan 4 (amphiglycan, ryudocan)	SDC4
229011_at	0.12		
243204_at	0.12		
203556_at	0.12	zinc fingers and homeoboxes 2	ZHX2
226748_at	0.12	LysM, putative peptidoglycan-binding, domain containing 2	LYSMD2
222996_s_at	0.12	CXXC finger 5	CXXC5
202887_s_at	0.12	DNA-damage-inducible transcript 4	DDIT4

204567_s_at	0.12	ATP-binding cassette, sub-family G (WHITE), member 1	ABCG1
1558869_at	0.12	A kinase (PRKA) anchor protein 6	AKAP6
226679_at	0.12	solute carrier family 26, member 11	SLC26A11
1555133_at	0.11	family with sequence similarity 9, member A	FAM9A
225612_s_at	0.11	UDP-GlcNAc:betaGal beta-1,3-N-acetylglucosaminyltransferase 5	B3GNT5
218223_s_at	0.11	pleckstrin homology domain containing, family O member 1	PLEKHO1
1554519_at	0.11	CD80 molecule	CD80
206924_at	0.11	interleukin 11	IL11
206127_at	0.11	ELK3, ETS-domain protein (SRF accessory protein 2)	ELK3
235422_at	0.11		
227052_at	0.11		
221195_at	0.11		
219256_s_at	0.11	SH3 domain and tetratricopeptide repeats 1	SH3TC1
217853_at	0.11	tensin 3	TNS3
214255_at	0.11	ATPase, Class V, type 10A	ATP10A
205532_s_at	0.11	cadherin 6, type 2, K-cadherin (fetal kidney)	CDH6
227062_at	0.11		
210770_s_at	0.11	calcium channel, voltage-dependent, P/Q type, alpha 1A subunit	CACNA1A
221773_at	0.11	ELK3, ETS-domain protein (SRF accessory protein 2)	ELK3
1552422_at	0.11	chromosome 10 open reading frame 25	C10orf25
202970_at	0.11		
241789_at	0.11		
229934_at	0.11		
225390_s_at	0.11	Kruppel-like factor 13	KLF13
228620_at	0.11		
206756_at	0.11	carbohydrate (N-acetylglucosamine 6-O) sulfotransferase 7	CHST7
232322_x_at	0.11	START domain containing 10	STARD10
231397_at	0.11		
223311_s_at	0.11	metastasis associated 1 family, member 3	MTA3
216222_s_at	0.11	myosin X	MYO10
226722_at	0.11	family with sequence similarity 20, member C	FAM20C
233955_x_at	0.11	CXXC finger 5	CXXC5
210587_at	0.11	inhibin, beta E	INHBE
217155_at	0.11	ribosomal protein L34 pseudogene 2	RPL34P2
228762_at	0.11	LFNG O-fucosylpeptide 3-beta-N-acetylglucosaminyltransferase	LFNG
206251_s_at	0.11	arginine vasopressin receptor 1A	AVPR1A
224223_s_at	0.11	phosphodiesterase 11A	PDE11A
1554835_a_at	0.11	UDP-GlcNAc:betaGal beta-1,3-N-acetylglucosaminyltransferase 5	B3GNT5
213148_at	0.11		
225224_at	0.11	chromosome 20 open reading frame 112#additional sex combs like 1 (Drosophila)	C20orf112#ASXL1
243592_at	0.10	REV1 homolog (S. cerevisiae)	REV1
205442_at	0.10	microfibrillar-associated protein 3-like	MFAP3L
225239_at	0.10		

219881_s_at	0.10		
239307_at	0.10	myosin, heavy chain 11, smooth muscle	MYH11
1555435_at	0.10	AF4/FMR2 family, member 4	AFF4
223655_at	0.10	CD163 molecule-like 1	CD163L1
207176_s_at	0.10	CD80 molecule	CD80
223916_s_at	0.10	BCL6 co-repressor	BCOR
236899_at	0.10		
230643_at	0.10	wingless-type MMTV integration site family, member 9A	WNT9A
230615_at	0.10	dual oxidase maturation factor 2	DUOXA2
241966_at	0.10		
211161_s_at	0.10	collagen, type III, alpha 1 (Ehlers-Danlos syndrome type IV, autosomal dominant)	COL3A1
225033_at	0.10		
221016_s_at	0.10	transcription factor 7-like 1 (T-cell specific, HMG-box)	TCF7L1
212828_at	0.10	synaptojanin 2	SYNJ2
221234_s_at	0.10	BTB and CNC homology 1, basic leucine zipper transcription factor 2	BACH2
223474_at	0.10	chromosome 14 open reading frame 4	C14orf4
232423_at	0.10	arylsulfatase D	ARSD
227396_at	0.10		
242814_at	0.10	serpin peptidase inhibitor, clade B (ovalbumin), member 9	SERPINB9
212827_at	0.10	immunoglobulin heavy constant mu	IGHM
216529_at	0.09		
226282_at	0.09		
227038_at	0.09		
213555_at	0.09	malic enzyme 1, NADP(+)-dependent, cytosolic#phosphoglucomutase 3#RWD domain containing 2	ME1#PGM3#RWDD2
232693_s_at	0.09	zinc finger protein 395	ZNF395
224516_s_at	0.09	CXXC finger 5	CXXC5
206670_s_at	0.09	glutamate decarboxylase 1 (brain, 67kDa)	GAD1
231296_at	0.09		
211986_at	0.09	AHNAK nucleoprotein (desmoyokin)	AHNAK
201502_s_at	0.09	nuclear factor of kappa light polypeptide gene enhancer in B-cells inhibitor, alpha	NFKBIA
203723_at	0.09	inositol 1,4,5-trisphosphate 3-kinase B	ITPKB
240182_at	0.09		
213839_at	0.09		
1562107_at	0.09		
202464_s_at	0.09	6-phosphofructo-2-kinase/fructose-2,6-biphosphatase 3	PFKFB3
1569673_at	0.09		
1569149_at	0.09	PDZ and LIM domain 7 (enigma)	PDLIM7
240118_at	0.09		
202910_s_at	0.09	CD97 molecule	CD97
203961_at	0.09	nebulette	NEBL
216439_at	0.09	tyrosine kinase, non-receptor, 2	TNK2
226955_at	0.09		
219563_at	0.09	chromosome 14 open reading frame 139	C14orf139
226189_at	0.09	integrin, beta 8	ITGB8

236465_at	0.09	ring finger protein 175	RNF175
203068_at	0.09	kelch-like 21 (Drosophila)	KLHL21
219433_at	0.09	BCL6 co-repressor	BCOR
227478_at	0.09	SET binding protein 1	SETBP1
243894_at	0.08	solute carrier family 41, member 2	SLC41A2
235116_at	0.08	TNF receptor-associated factor 1	TRAF1
206599_at	0.08	solute carrier family 16, member 5 (monocarboxylic acid transporter 6)	SLC16A5
220998_s_at	0.08	unc-93 homolog B1 (C. elegans)	UNC93B1
216971_s_at	0.08	plectin 1, intermediate filament binding protein 500kDa	PLEC1
1564107_at	0.08		
205599_at	0.08	TNF receptor-associated factor 1	TRAF1
220246_at	0.08	calcium/calmodulin-dependent protein kinase ID	CAMK1D
201324_at	0.08	epithelial membrane protein 1	EMP1
239846_at	0.08		
202948_at	0.08	interleukin 1 receptor, type I	IL1R1
229016_s_at	0.08	transcriptional regulating factor 1	TRERF1
228770_at	0.08	G protein-coupled receptor 146	GPR146
234989_at	0.08		
243386_at	0.08		
223484_at	0.08	chromosome 15 open reading frame 48	C15orf48
216566_at	0.08		
202403_s_at	0.08	collagen, type I, alpha 2	COL1A2
227329_at	0.08	zinc finger and BTB domain containing 46	ZBTB46
217091_at	0.08		
223961_s_at	0.08	cytokine inducible SH2-containing protein	CISH
211571_s_at	0.08	chondroitin sulfate proteoglycan 2 (versican)	CSPG2
1561042_at	0.08	integrin, beta 1 (fibronectin receptor, beta polypeptide, antigen CD29 includes MDF2, MSK12)	ITGB1
230913_at	0.08		
224746_at	0.08	KIAA1522	KIAA1522
231798_at	0.07	noggin	NOG
1556067_a_at	0.07	jumonji domain containing 3	JMJD3
210612_s_at	0.07	synaptojanin 2	SYNJ2
209262_s_at	0.07	nuclear receptor subfamily 2, group F, member 6	NR2F6
227645_at	0.07	phosphoinositide-3-kinase, regulatory subunit 5, p101	PIK3R5
1557963_at	0.07	CDC42 binding protein kinase beta (DMPK-like)	CDC42BPB
221843_s_at	0.07	KIAA1609	KIAA1609
1562514_at	0.07		
201170_s_at	0.07	basic helix-loop-helix domain containing, class B, 2	BHLHB2
235874_at	0.07	protease, serine, 35	PRSS35
1553962_s_at	0.07	ras homolog gene family, member B	RHOB
209683_at	0.07	family with sequence similarity 49, member A	FAM49A
226530_at	0.07	Bcl2 modifying factor	BMF
210029_at	0.07	indoleamine-pyrrole 2,3 dioxygenase	INDO
202207_at	0.07	ADP-ribosylation factor-like 4C	ARL4C
202015_x_at	0.07	methionyl aminopeptidase 2	METAP2

222450_at	0.07	phosphoenolpyruvate carboxykinase 1 (soluble)#transmembrane, prostate androgen induced RNA#Z-DNA binding protein 1#CCCTC-binding factor (zinc finger protein)-like	PCK1#TMEPAI #ZBP1#CTCFL
205463_s_at	0.07	platelet-derived growth factor alpha polypeptide	PDGFA
1556066_at	0.07	jumonji domain containing 3	JMJD3
207826_s_at	0.07	inhibitor of DNA binding 3, dominant negative helix-loop-helix protein	ID3
201325_s_at	0.06	epithelial membrane protein 1	EMP1
228263_at	0.06	GRP1 (general receptor for phosphoinositides 1)-associated scaffold protein	GRASP
1559653_at	0.06		
202404_s_at	0.06	collagen, type I, alpha 2	COL1A2
207272_at	0.06	zinc finger protein 80	ZNF80
1568777_at	0.06		
235111_at	0.06		
238600_at	0.06	janus kinase and microtubule interacting protein 1	JAKMIP1
1561096_at	0.06		
1563135_at	0.06		
221565_s_at	0.06	family with sequence similarity 26, member B	FAM26B
231665_at	0.06	arginase, liver	ARG1
207907_at	0.06	tumor necrosis factor (ligand) superfamily, member 14	TNFSF14
244079_at	0.06		
227571_at	0.06		
205669_at	0.06	neural cell adhesion molecule 2	NCAM2
209277_at	0.06	tissue factor pathway inhibitor 2	TFPI2
204428_s_at	0.06	lecithin-cholesterol acyltransferase	LCAT
224393_s_at	0.06	cat eye syndrome chromosome region, candidate 6	CECR6
213895_at	0.05	epithelial membrane protein 1	EMP1
215294_s_at	0.05	SWI/SNF related, matrix associated, actin dependent regulator of chromatin, subfamily a, member 1	SMARCA1
214046_at	0.05		
229830_at	0.05		
223798_at	0.05	solute carrier family 41, member 2	SLC41A2
227347_x_at	0.05	hairy and enhancer of split 4 (Drosophila)	HES4
204908_s_at	0.05	B-cell CLL/lymphoma 3	BCL3
209278_s_at	0.05	tissue factor pathway inhibitor 2	TFPI2
1558387_at	0.05		
219115_s_at	0.05	interleukin 20 receptor, alpha	IL20RA
207072_at	0.05	interleukin 18 receptor accessory protein	IL18RAP
219878_s_at	0.05	Kruppel-like factor 13	KLF13
208010_s_at	0.05	protein tyrosine phosphatase, non-receptor type 22 (lymphoid)	PTPN22
221530_s_at	0.05	basic helix-loop-helix domain containing, class B, 3	BHLHB3
235735_at	0.05		
205398_s_at	0.05	SMAD family member 3	SMAD3
230493_at	0.04	transmembrane protein 46	TMEM46
210538_s_at	0.04	baculoviral IAP repeat-containing 3	BIRC3

232151_at	0.04		
225615_at	0.04		
239605_x_at	0.04		
205419_at	0.04	Epstein-Barr virus induced gene 2 (lymphocyte-specific G protein-coupled receptor)	EBI2
235359_at	0.04	leucine rich repeat containing 33	LRRC33
223887_at	0.04	G protein-coupled receptor 132	GPR132
228885_at	0.04	ribosomal protein L24	RPL24
223566_s_at	0.03	BCL6 co-repressor	BCOR
219584_at	0.03	phospholipase A1 member A	PLA1A
203045_at	0.03	ninjurin 1	NINJ1
208978_at	0.02	cysteine-rich protein 2	CRIP2
1568623_a_at	0.02	solute carrier family 35, member E4	SLC35E4
226913_s_at	0.02	SRY (sex determining region Y)-box 8	SOX8
240463_at	0.02		
242963_at	0.01		
206486_at	0.01	lymphocyte-activation gene 3	LAG3
