# Supplementary material for: In Vitro Transformation of Primary Human CD34+ Cells by AML Fusion Oncogenes: Early Gene Expression Profiling Reveals Possible Drug Target in AML
Source: PLoS One. 2010 Aug 27;5(8):e12464. doi: 10.1371/journal.pone.0012464 (PMC2929205; doi:10.1371/journal.pone.0012464)
Supplement: Table S14 — Genes deregulated by AML1-ETO 3 days after transduction. Primary human CD34+ cells were retrovirally transduced with either control MSCV-IRES-GFP vector or vector expressing AML1-ETO and sorted for GFP positivity. Total RNA was extracted 3 days after transduction and subjected to microarray analysis. Microarray data were analyzed by SAM as described in Materials and Methods. Significantly deregulated genes are listed and the false discovery rate (FDR) is shown. (0.06 MB PDF) [file pone.0012464.s014.pdf]

Table S14. Genes deregulated by AML1-ETO at 3 d detected by SAM

FDR = 6.68%

Probe set ID	Fold Change	Gene Name	Gene Symbol
243520_x_at	54.37	ADAM metallopeptidase domain 30	ADAM30
1564580_at	30.97		
1557057_a_at	30.13		
204888_s_at	26.91	neuralized homolog (Drosophila)	NEURL
1553428_at	24.54		
242882_at	24.16	isoprenylcysteine carboxyl methyltransferase	ICMT
206460_at	19.81	adherens junction associated protein 1	AJAP1
215301_at	19.73		
224533_s_at	16.70	interferon, alpha-inducible protein 6	IFI6
217875_s_at	16.28	transmembrane, prostate androgen induced RNA	TMEPAI
241067_at	16.24		
1564083_at	15.80		
233145_at	15.55	CUB and Sushi multiple domains 2	CSMD2
1559664_at	15.45		
237391_at	15.42		
1563087_at	15.25		
234702_x_at	14.81	cystic fibrosis transmembrane conductance regulator (ATP-binding cassette sub-family C, member 7)	CFTR
231699_at	14.62	nuclear factor of kappa light polypeptide gene enhancer in B-cells inhibitor, alpha	NFKBIA
1566947_at	14.34		
229546_at	14.25		
214247_s_at	14.22	dickkopf homolog 3 (Xenopus laevis)	DKK3
220530_at	14.14		
238932_at	13.96		
241316_at	13.19	trinucleotide repeat containing 6A	TNRC6A
237835_at	13.13		
224405_at	12.55	Fc receptor-like 5	FCRL5
205635_at	11.54	kalirin, RhoGEF kinase	KALRN
243541_at	11.24	interleukin 31 receptor A	IL31RA
230757_at	10.84		
215325_x_at	10.51	ATP synthase,	ATP5D
232037_at	10.29	putative neuronal cell adhesion molecule	PUNC
222910_s_at	10.29	peroxisomal biogenesis factor 5-like	PEX5L
1554371_at	10.22	polycystic kidney disease 1-like 2	PKD1L2
222153_at	10.14	myelin expression factor 2	MYEF2
244557_at	9.94		
242344_at	9.88	gamma-aminobutyric acid (GABA) A receptor, beta 2	GABRB2
205749_at	9.82	cytochrome P450, family 1, subfamily A, polypeptide 1	CYP1A1
216307_at	9.74	diacylglycerol kinase, beta 90kDa	DGKB
231156_at	9.73		
203187_at	8.93	dedicator of cytokinesis 1	DOCK1
206772_at	8.91	parathyroid hormone receptor 2	PTHR2

1553822_at	8.83	receptor (chemosensory) transporter protein 1	RTP1
206785_s_at	8.62	killer cell lectin-like receptor subfamily C, member 2	KLRC2
210888_s_at	8.56	inter-alpha (globulin) inhibitor H1	ITIH1
1558587_at	8.25		
234504_at	8.06	bactericidal/permeability-increasing protein-like 1#bactericidal/permeability-increasing protein-like 3#sperm associated antigen 4-like#chromosome 20 open reading frame 186#chromosome 20 open reading frame 185	BPIL1#BPIL3#SPAG4L#C20orf186#C20orf185
233978_at	7.66	protein tyrosine phosphatase, receptor type, E	PTPRE
233858_at	7.08		
220851_at	6.85		
240933_at	6.78	electron-transfer-flavoprotein, beta polypeptide	ETFB
204684_at	6.37	neuronal pentraxin I	NPTX1
215712_s_at	6.19	insulin-like growth factor binding protein, acid labile subunit	IGFALS
236870_at	5.95	IQ motif containing F3	IQCF3
241249_at	5.95		
1568896_at	5.88	syntaxin binding protein 5-like	STXBP5L
227376_at	5.75	GLI-Kruppel family member GLI3 (Greig cephalopolysyndactyly syndrome)	GLI3
1562311_at	5.65		
1555719_a_at	5.60	chromosome 3 open reading frame 15	C3orf15
205561_at	5.54	potassium channel tetramerisation domain containing 17	KCTD17
1561090_at	5.43		
234765_at	5.23		
243892_at	5.14		
242522_at	4.76		
217380_s_at	4.71		
237986_at	4.45		
243712_at	4.41	X (inactive)-specific transcript	XIST
207951_at	4.27	casein beta	CSN2
229832_x_at	4.24	SH3 domain and tetratricopeptide repeats 1	SH3TC1
237295_at	4.21		
240863_at	4.20	cytochrome P450, family 19, subfamily A, polypeptide 1	CYP19A1
1553123_at	3.35	WD repeat domain 62	WDR62
1558982_at	3.34		
242719_at	3.29		
227188_at	3.27	chromosome 21 open reading frame 63	C21orf63
239956_at	3.27		
228438_at	3.17		
1563854_s_at	3.15		
244455_at	3.11	potassium channel, subfamily T, member 2	KCNT2
217473_x_at	3.08	solute carrier family 11 (proton-coupled divalent metal ion transporters), member 1#CTD (carboxy-terminal domain, RNA polymerase II, polypeptide A) small phosphatase 1	SLC11A1#CTDSP1
336_at	2.99	thromboxane A2 receptor	TBXA2R
216192_at	2.93	fatty acid binding protein 7, brain	FABP7
241831_at	2.93	zinc finger protein 614	ZNF614

231270_at	2.89	carbonic anhydrase XIII	CA13
226637_at	2.88	ubiquitin-conjugating enzyme E2H (UBC8 homolog, yeast)	UBE2H
1564887_at	2.79		
204932_at	2.76	tumor necrosis factor receptor superfamily, member 11b (osteoprotegerin)	TNFRSF11B
242873_at	2.75		
238103_at	2.69		
203827_at	2.69	WD repeat domain, phosphoinositide interacting 1	WIPI1
1561247_at	2.65		
224497_x_at	2.65	hydroxysteroid (17-beta) dehydrogenase 14	HSD17B14
227140_at	2.62		
205872_x_at	2.61	phosphodiesterase 4D interacting protein (myomegalin)	PDE4DIP
205699_at	2.55	mitogen-activated protein kinase kinase 6	MAP2K6
211343_s_at	2.54	collagen, type XIII, alpha 1	COL13A1
205205_at	2.33	v-rel reticuloendotheliosis viral oncogene homolog B, nuclear factor of kappa light polypeptide gene	RELB
239572_at	2.33	gap junction protein, alpha 3, 46kDa	GJA3
226756_at	2.27		
237932_at	2.25		
237770_at	2.24		
212104_s_at	2.16	RNA binding motif protein 9	RBM9
1556898_at	2.14		
244359_s_at	2.11		
238093_at	2.11		
206025_s_at	2.10	tumor necrosis factor, alpha-induced protein 6	TNFAIP6
242447_at	2.08		
1562337_at	2.08	olfactory receptor, family 7, subfamily D, member 2	OR7D2
232886_at	0.20		
241868_at	0.02		
