# Supplementary material for: In Vitro Transformation of Primary Human CD34+ Cells by AML Fusion Oncogenes: Early Gene Expression Profiling Reveals Possible Drug Target in AML
Source: PLoS One. 2010 Aug 27;5(8):e12464. doi: 10.1371/journal.pone.0012464 (PMC2929205; doi:10.1371/journal.pone.0012464)
Supplement: Table S15 — Genes deregulated by AML1-ETO 8 days after transduction. Primary human CD34+ cells were retrovirally transduced with either control MSCV-IRES-GFP vector or vector expressing AML1-ETO and sorted for GFP positivity. Total RNA was extracted 8 days after transduction and subjected to microarray analysis. Microarray data were analyzed by SAM as described in Materials and Methods. Significantly deregulated genes are listed and the false discovery rate (FDR) is shown. (0.15 MB PDF) [file pone.0012464.s015.pdf]

Table S15. Genes deregulated by AML1-ETO at 8 d detected by SAM FDR = 9.88%

Probe set ID	Fold Change	Gene Name	Gene Symbol
240915_at	27.16	netrin 2-like (chicken)	NTN2L
1555454_at	24.37	lipopolysaccharide-induced TNF factor	LITAF
205749_at	16.67	cytochrome P450, family 1, subfamily A, polypeptide 1	CYP1A1
242513_x_at	16.41	KIAA2018	KIAA2018
232976_at	15.39	Prader-Willi syndrome chromosome region 1	PWCR1
1554524_a_at	15.03	olfactomedin 3	OLFM3
1569341_at	14.75	polymerase (DNA directed), eta	POLH
221169_s_at	13.34	histamine receptor H4	HRH4
224354_at	12.37		
240036_at	12.26	SEC14-like 1 (S. cerevisiae)	SEC14L1
239298_at	11.02		
230671_at	11.00		
239860_at	10.94		
1570300_at	10.73		
215514_at	10.70		
1556166_x_at	10.60		
1562431_x_at	9.48		
231048_at	8.99		
336_at	8.81	thromboxane A2 receptor	TBXA2R
1566995_at	8.70		
1559287_at	8.59		
1567244_at	8.41	olfactory receptor, family 5, subfamily J, member 2	OR5J2
243268_at	8.20	chromosome 11 open reading frame 38	C11orf38
1564438_at	8.05		
234583_at	7.90	cell adhesion molecule with homology to L1CAM (close homolog of L1)	CHL1
1555273_at	7.78		
213319_s_at	7.73	cold shock domain protein A	CSDA
227984_at	7.67		
1561418_at	7.62		
228877_at	7.61	erythropoietin receptor	EPOR
240681_at	7.50		
230237_at	7.44		
217085_at	7.35		
222950_at	7.29	NIPA-like domain containing 2	NPAL2
228368_at	7.24	Rho GTPase activating protein 20	ARHGAP20
1561663_at	7.13		
223652_at	7.10	arsenic (+3 oxidation state) methyltransferase	AS3MT
1561658_at	6.71		
243694_at	6.69	G protein-coupled receptor 125	GPR125
1567859_at	6.63		
240921_at	6.57		
1558400_x_at	6.51	ankyrin repeat domain 24	ANKRD24
1561244_at	6.40		
237297_at	6.34	family with sequence similarity 53, member B	FAM53B
244885_at	6.33		
222066_at	6.09	erythrocyte membrane protein band 4.1-like 1	EPB41L1

230552_at	6.05		
1565616_at	5.96		
207990_x_at	5.94	acrosomal vesicle protein 1	ACRV1
242964_at	5.94		
1564841_at	5.91		
214156_at	5.75	myosin VIIA and Rab interacting protein	MYRIP
236258_at	5.73	chromosome 20 open reading frame 151	C20orf151
238269_at	5.70	F-box and leucine-rich repeat protein 7	FBXL7
244081_at	5.69		
232928_at	5.68		
230658_at	5.56	solute carrier family 7 (cationic amino acid transporter, y+ system), member 2	SLC7A2
1553915_at	5.56	chromosome 10 open reading frame 126	C10orf126
208245_at	5.53	RAB9, member RAS oncogene family, pseudogene 1	RAB9P1
1560305_x_at	5.50		
226103_at	5.47	nexilin (F actin binding protein)	NEXN
234187_at	5.42		
235362_at	5.30		
239150_at	5.08		
233863_at	4.90	castor zinc finger 1	CASZ1
216675_at	4.89		
205765_at	4.88	cytochrome P450, family 3, subfamily A, polypeptide 5	CYP3A5
1566295_at	4.77	family with sequence similarity 118, member B	FAM118B
1559889_at	4.71		
232368_at	4.57	BET3 like (S. cerevisiae)	BET3L
212198_s_at	0.50	transmembrane 9 superfamily protein member 4	TM9SF4
222097_at	0.50		
229247_at	0.50		
235840_at	0.50		
236588_at	0.50	craniofacial development protein 1	CFDP1
226122_at	0.50	methylenetetrahydrofolate dehydrogenase (NADP+ dependent) 1-like#pleckstrin homology domain containing, family G (with RhoGef domain) member 1	MTHFD1L#PLEKHG1
208604_s_at	0.50	homeobox A3	HOXA3
1562069_at	0.50		
210690_at	0.50	killer cell lectin-like receptor subfamily C, member 4	KLRC4
239959_x_at	0.50	phosphodiesterase 3B, cGMP-inhibited	PDE3B
220464_at	0.49	MCF.2 cell line derived transforming sequence-like	MCF2L
234571_at	0.49		
239984_at	0.49	sodium channel, voltage-gated, type VII, alpha	SCN7A
236527_at	0.49		
243179_at	0.49		
1558894_a_at	0.49	coiled-coil domain containing 67	CCDC67
228503_at	0.49		
216279_at	0.49	zinc finger protein 460	ZNF460
237700_at	0.49		
1556110_at	0.49		
217445_s_at	0.49	phosphoribosylglycinamide formyltransferase, phosphoribosylglycinamide synthetase, phosphoribosylami	GART

243017_at	0.49		
236945_at	0.49	chromosome 9 open reading frame 93	C9orf93
235756_at	0.49	sterile alpha motif domain containing 4A	SAMD4A
235212_at	0.49	chromosome 14 open reading frame 102	C14orf102
1556763_at	0.49		
1558742_at	0.49		
1559790_at	0.49		
1553024_at	0.49		
237702_at	0.49		
243661_at	0.49	zinc finger protein 273	ZNF273
1564662_at	0.49		
233061_at	0.49	hepatocyte nuclear factor 4, alpha#ganglioside-induced differentiation-associated protein 1-like 1#chromosome 20 open reading frame 142#R3H domain containing-like	HNF4A#GDAP1L1#C20orf142#R3HDML
220103_s_at	0.49	mitochondrial ribosomal protein S18C	MRPS18C
233740_at	0.49		
240712_s_at	0.49		
211032_at	0.49	COBL-like 1	COBLL1
237861_at	0.48		
1554453_at	0.48	heterogeneous nuclear ribonucleoprotein L-like	HNRPLL
214066_x_at	0.48	natriuretic peptide receptor B/guanylate cyclase B (atrionatriuretic peptide receptor B)	NPR2
1568720_at	0.48	zinc finger protein 506	ZNF506
243348_at	0.48	WD repeat and FYVE domain containing 1	WDFY1
222168_at	0.48		
242387_at	0.48	chromosome 8 open reading frame 42	C8orf42
228716_at	0.48	thyroid hormone receptor, beta (erythroblastic leukemia viral (v-erb-a) oncogene homolog 2, avian)	THRB
238713_at	0.48		
208261_x_at	0.48	interferon, alpha 10	IFNA10
204974_at	0.48	RAB3A, member RAS oncogene family	RAB3A
1563118_at	0.48		
207425_s_at	0.48	septin 9	9-Sep
1555217_at	0.48	ubiquitin-conjugating enzyme E2W (putative)	UBE2W
240963_x_at	0.48	plexin domain containing 1	PLXDC1
216582_at	0.48	zinc finger protein 204#protease, serine, 16 (thymus)#null#POM121 membrane glycoprotein-like 2 (rat)	ZNF204#PRSS16#null#POM121L2
1565840_at	0.48	PR domain containing 4	PRDM4
213756_s_at	0.48	heat shock transcription factor 1	HSF1
232749_at	0.48		
206648_at	0.48	zinc finger protein 571	ZNF571
222094_at	0.48	sulfotransferase family, cytosolic, 1A, phenol-preferring, member 4	SULT1A4
1553428_at	0.48		
214245_at	0.48	ribosomal protein S14	RPS14
243707_at	0.48		
1558466_at	0.48		
1554280_a_at	0.47	chromosome 9 open reading frame 43	C9orf43
208592_s_at	0.47	CD1e molecule	CD1E
1557644_at	0.47		
1568783_at	0.47	splicing factor, arginine/serine-rich 12	SFRS12

210234_at	0.47	glutamate receptor, metabotropic 4	GRM4
239153_at	0.47		
216073_at	0.47		
244504_x_at	0.47	ADP-ribosylation factor 1	ARF1
238801_at	0.47	RNA binding motif protein 33	RBM33
238221_at	0.47		
230685_at	0.47		
243918_at	0.47		
1563884_at	0.47		
227660_at	0.47	anthrax toxin receptor 1	ANTXR1
244503_at	0.47	brain-derived neurotrophic factor	BDNF
206222_at	0.47	tumor necrosis factor receptor superfamily, member 10c, decoy without an intracellular domain	TNFRSF10C
244624_at	0.47	ribosomal protein S27a	RPS27A
219095_at	0.47	phospholipase A2, group IVB (cytosolic)	PLA2G4B
221464_at	0.47	olfactory receptor, family 1, subfamily D, member 2	OR1D2
1560264_at	0.47		
242747_at	0.47		
233294_at	0.47		
1553193_at	0.47	zinc finger protein 441	ZNF441
1552732_at	0.47	actin-binding Rho activating protein	ABRA
230402_at	0.47	BCL2-like 1#forkhead-like 18 (Drosophila)#TPX2, microtubule-associated, homolog (Xenopus laevis)#chromosome 20 open reading frame 57#myosin light chain kinase 2, skeletal muscle#dual specificity phosphatase 15#tubulin tyrosine ligase-like family, member 9	BCL2L1#FKHL18#TPX2#C20orf57#MYLK2#DUSP15#TTLL9
223371_s_at	0.47	DnaJ (Hsp40) homolog, subfamily C, member 4	DNAJC4
226058_at	0.47		
203750_s_at	0.46	retinoic acid receptor, alpha	RARA
223614_at	0.46	chromosome 8 open reading frame 57	C8orf57
1570229_at	0.46		
1553834_at	0.46		
213424_at	0.46		
219928_s_at	0.46	calcium binding tyrosine-(Y)-phosphorylation regulated (fibrousheathin 2)	CABYR
232060_at	0.46		
229518_at	0.46	family with sequence similarity 46, member B	FAM46B
220965_s_at	0.46	radial spokehead-like 1	RSHL1
1559507_at	0.46		
1559762_at	0.46		
234196_at	0.46		
206573_at	0.46	potassium voltage-gated channel, KQT-like subfamily, member 3	KCNQ3
206339_at	0.46	CART prepropeptide	CARTPT
239827_at	0.46		
205782_at	0.46	fibroblast growth factor 7 (keratinocyte growth factor)	FGF7
244242_at	0.46		
241634_at	0.46		
207001_x_at	0.46	TSC22 domain family, member 3	TSC22D3
233932_at	0.46		
221031_s_at	0.46	apolipoprotein L domain containing 1	APOLD1

200606_at	0.46	desmoplakin	DSP
230611_at	0.46	synaptophysin-like 2	SYPL2
237696_at	0.46		
242212_at	0.46	regulator of G-protein signalling 16	RGS16
220787_at	0.46		
229159_at	0.46	thrombospondin, type I, domain containing 7A	THSD7A
236882_at	0.46		
226764_at	0.46		
209672_s_at	0.46		
227289_at	0.45	protocadherin 17	PCDH17
229546_at	0.45		
231860_at	0.45	bromodomain and WD repeat domain containing 1	BRWD1
1552734_at	0.45		
203901_at	0.45	mitogen-activated protein kinase kinase kinase 7 interacting protein 1	MAP3K7IP1
220474_at	0.45	solute carrier family 25 (mitochondrial oxodicarboxylate carrier), member 21	SLC25A21
244819_x_at	0.45		
234136_at	0.45		
216325_x_at	0.45	tumor necrosis factor receptor superfamily, member 6b, decoy#ADP-ribosylation factor related protein 1#stathmin-like 3#regulator of telomere elongation helicase 1	TNFRSF6B#ARFRP1#STMN3#RTEL1
207283_at	0.45		
1569855_at	0.45		
1558540_s_at	0.45		
210479_s_at	0.45	RAR-related orphan receptor A	RORA
211493_x_at	0.45	dystrobrevin, alpha	DTNA
1569072_s_at	0.45	ATP-binding cassette, sub-family B (MDR/TAP), member 5	ABCB5
206563_s_at	0.45	opiate receptor-like 1	OPRL1
241193_at	0.45	v-ets erythroblastosis virus E26 oncogene homolog 2 (avian)	ETS2
1554041_at	0.45	chromosome 20 open reading frame 141	C20orf141
220182_at	0.45	solute carrier family 25 (mitochondrial carrier; phosphate carrier), member 23	SLC25A23
1569349_at	0.45	chromosome 11 open reading frame 30	C11orf30
239914_at	0.45		
243658_at	0.45	farnesyl-diphosphate farnesyltransferase 1	FDFT1
241542_at	0.45	SRY (sex determining region Y)-box 6	SOX6
222259_s_at	0.45	RAE1 RNA export 1 homolog (S. pombe)#SPO11 meiotic protein covalently bound to DSB homolog (S. cerevisiae)	RAE1#SPO11
1566607_at	0.45		
216989_at	0.45	sperm adhesion molecule 1 (PH-20 hyaluronidase, zona pellucida binding)	SPAM1
1555867_at	0.45		
1570398_at	0.45		
235881_at	0.45	formin-like 2	FMNL2
239006_at	0.45	solute carrier family 26, member 7	SLC26A7
241719_at	0.45		
1557843_at	0.45		
1554649_at	0.44		

1555073_at	0.44		
1570169_at	0.44	CUB and Sushi multiple domains 2	CSMD2
205898_at	0.44	chemokine (C-X3-C motif) receptor 1	CX3CR1
220546_at	0.44		
1556462_a_at	0.44		
233649_at	0.44	katanin p60 subunit A-like 2	KATNAL2
229657_at	0.44	thyroid hormone receptor, beta (erythroblastic leukemia viral (v-erb-a) oncogene homolog 2, avian)	THRB
205772_s_at	0.44	A kinase (PRKA) anchor protein 7	AKAP7
1557354_at	0.44	son of sevenless homolog 1 (Drosophila)	SOS1
228890_at	0.44	atonal homolog 8 (Drosophila)	ATOH8
230810_at	0.44	jumonji domain containing 4	JMJD4
213436_at	0.44	cannabinoid receptor 1 (brain)	CNR1
242270_at	0.44		
233548_at	0.44		
1552770_s_at	0.44	zinc finger protein 563	ZNF563
208394_x_at	0.44	endothelial cell-specific molecule 1	ESM1
1562878_at	0.44		
1562399_at	0.44		
234048_s_at	0.44	KIAA1632	KIAA1632
1552943_at	0.44	gamma-aminobutyric acid (GABA) A receptor, gamma 1	GABRG1
222079_at	0.44		
207470_at	0.44		
235453_at	0.44		
232908_at	0.44	ATPase family, AAA domain containing 2B	ATAD2B
1566108_at	0.44	myoneurin	MYNN
1555123_at	0.44	ST6 beta-galactosamide alpha-2,6-sialyltranferase 2	ST6GAL2
1559634_at	0.43	cholinergic receptor, muscarinic 3	CHRM3
211775_x_at	0.43		
227842_at	0.43	RAB30, member RAS oncogene family	RAB30
238868_at	0.43	uveal autoantigen with coiled-coil domains and ankyrin repeats	UACA
1558651_at	0.43		
232276_at	0.43	heparan sulfate 6-O-sulfotransferase 3	HS6ST3
230503_at	0.43	sterile alpha motif domain containing 4A	SAMD4A
215118_s_at	0.43	immunoglobulin heavy constant alpha 1	IGHA1
1561450_at	0.43		
236863_at	0.43	chromosome 17 open reading frame 67	C17orf67
237187_at	0.43		
1561915_at	0.43		
217120_s_at	0.43	cofactor required for Sp1 transcriptional activation, subunit 2, 150kDa	CRSP2
238182_at	0.43		
240180_at	0.43		
223690_at	0.43	latent transforming growth factor beta binding protein 2	LTBP2
231756_at	0.43	zona pellucida glycoprotein 4	ZP4
239587_at	0.43		
207926_at	0.43	glycoprotein V (platelet)	GP5
1553553_at	0.43	taste receptor, type 2, member 39	TAS2R39
1552649_a_at	0.42	ring finger and FYVE-like domain containing 1	RFFL
237016_at	0.42	chromosome 6 open reading frame 128	C6orf128

233831_at	0.42		
204286_s_at	0.42	phorbol-12-myristate-13-acetate-induced protein 1	PMAIP1
240728_at	0.42	phospholipase C, beta 4	PLCB4
1553491_at	0.42	kinase suppressor of ras 2	KSR2
224940_s_at	0.42	pregnancy-associated plasma protein A, pappalysin 1	PAPPA
222534_s_at	0.42	chromosome 14 open reading frame 173	C14orf173
213406_at	0.42	WD repeat and SOCS box-containing 1	WSB1
217128_s_at	0.42	calcium/calmodulin-dependent protein kinase IG	CAMK1G
211171_s_at	0.42	phosphodiesterase 10A	PDE10A
242188_at	0.42	protein tyrosine phosphatase, receptor type, G	PTPRG
1558308_at	0.42		
241035_s_at	0.42		
217714_x_at	0.42	stathmin 1/oncoprotein 18	STMN1
234134_at	0.41		
231031_at	0.41		
1553166_at	0.41	cadherin-like 24	CDH24
1564392_at	0.41	chromosome 21 open reading frame 131	C21orf131
1554804_a_at	0.41	claudin 19	CLDN19
230450_at	0.41		
241738_at	0.41		
244787_at	0.41		
1556331_a_at	0.41		
228003_at	0.41	RAB30, member RAS oncogene family	RAB30
240878_at	0.41	fibroblast growth factor 11	FGF11
234682_at	0.41	BTB (POZ) domain containing 9	BTBD9
239351_at	0.41	FK506 binding protein 3, 25kDa	FKBP3
230523_at	0.41		
1556421_at	0.41		
211812_s_at	0.41	beta-1,3-N-acetylgalactosaminyltransferase 1 (globoside blood group)	B3GALNT1
230500_at	0.41	phosphodiesterase 7A	PDE7A
214009_at	0.41	male-specific lethal 3-like 1 (Drosophila)	MSL3L1
1557617_at	0.41		
1565662_at	0.41	mucin 6, oligomeric mucus/gel-forming	MUC6
1552414_at	0.41	WAP four-disulfide core domain 9	WFDC9
211756_at	0.41	parathyroid hormone-like hormone	PTHLH
206374_at	0.40	dual specificity phosphatase 8	DUSP8
217102_at	0.40		
216981_x_at	0.40	sialophorin (leukosialin, CD43)	SPN

234819_at	0.40	T cell receptor alpha locus#T cell receptor delta variable 1#T cell receptor alpha variable 36/delta variable 7#T cell receptor alpha variable 35#T cell receptor alpha variable 34#T cell receptor alpha variable 33#T cell receptor alpha variable 32#T cell receptor alpha variable 31#T cell receptor alpha variable 30#T cell receptor alpha variable 29/delta variable 5#T cell receptor alpha variable 28#T cell receptor alpha variable 27#T cell receptor alpha variable 26-2#T cell receptor alpha variable 26-1#T cell receptor alpha variable 25#T cell receptor alpha variable 24#T cell receptor alpha variable 23/delta variable 6#T cell receptor alpha variable 22#T cell receptor alpha variable 21#T cell receptor alpha variable 20#T cell receptor alpha variable 19#T cell receptor alpha variable 18#T cell receptor alpha variable 17#T cell receptor alpha variable 8-7	TRA@#TRDV1#TRAV36DV7#TRAV35#TRAV34#TRAV33#TRAV32#TRAV31#TRAV30#TRAV29DV5#TRAV28#TRAV27#TRAV26-2#TRAV26-1#TRAV25#TRAV24#TRAV23DV6#TRAV22#TRAV21#TRAV20#TRAV19#TRAV18#TRAV17#TRAV8-7
233130_at	0.40		
216712_at	0.40	transmembrane protein 132A	TMEM132A
1564039_at	0.40	zinc finger protein 390	ZNF390
234853_s_at	0.40	YWHAQ pseudogene 2	YWHAQP2
205048_s_at	0.40	phosphoserine phosphatase	PSPH
206545_at	0.40	CD28 molecule	CD28
241898_at	0.40		
237675_at	0.40		
1557374_at	0.40	ATP-binding cassette, sub-family C (CFTR/MRP), member 9	ABCC9
239738_at	0.40	dachshund homolog 2 (Drosophila)	DACH2
215302_at	0.40		
1562116_at	0.40		
224507_s_at	0.40		
216898_s_at	0.40	collagen, type IV, alpha 3 (Goodpasture antigen)	COL4A3
242004_x_at	0.40		
224126_at	0.40	solute carrier family 10 (sodium/bile acid cotransporter family), member 7	SLC10A7
243517_at	0.40		
1554417_s_at	0.40	anterior pharynx defective 1 homolog A (C. elegans)	APH1A
238368_at	0.40		
240501_at	0.40		
1554436_a_at	0.40	regenerating islet-derived family, member 4	REG4
210787_s_at	0.40	calcium/calmodulin-dependent protein kinase kinase 2, beta	CAMKK2
201510_at	0.39	E74-like factor 3 (ets domain transcription factor, epithelial-specific)	ELF3
1556725_a_at	0.39		
1554372_at	0.39		
238348_x_at	0.39		
239438_at	0.39	Rap guanine nucleotide exchange factor (GEF) 6	RAPGEF6
241804_at	0.39		
207095_at	0.39	solute carrier family 10 (sodium/bile acid cotransporter family), member 2	SLC10A2

215569_at	0.39	general transcription factor II, i	GTF2I
244076_at	0.39		
242199_at	0.39		
1561407_at	0.39	centaurin, delta 1	CENTD1
211356_x_at	0.39	leptin receptor	LEPR
237393_at	0.39		
233600_at	0.39		
1554324_s_at	0.39	dynein, cytoplasmic 2, light intermediate chain 1	DYNC2LI1
1566440_at	0.39		
204719_at	0.39	ATP-binding cassette, sub-family A (ABC1), member 8	ABCA8
1555912_at	0.39	ST7 overlapping transcript 1 (antisense non-coding RNA)	ST7OT1
229054_at	0.39		
223817_at	0.39	leucine-rich repeats and IQ motif containing 1	LRRIQ1
212353_at	0.39	sulfatase 1	SULF1
231366_at	0.39		
207651_at	0.39	G protein-coupled receptor 171	GPR171
207246_at	0.38	zinc finger protein, Y-linked	ZFY
1563061_at	0.38		
230507_at	0.38	ataxin 1	ATXN1
239546_at	0.38		
231683_at	0.38	glycine-N-acyltransferase	GLYAT
235099_at	0.38	CKLF-like MARVEL transmembrane domain containing 8	CMTM8
240184_at	0.38		
230981_at	0.38	cation channel, sperm associated 3	CATSPER3
211599_x_at	0.38	met proto-oncogene (hepatocyte growth factor receptor)	MET
216090_x_at	0.38		
1561242_at	0.38		
236262_at	0.38	multimerin 2	MMRN2
203187_at	0.38	dedicator of cytokinesis 1	DOCK1
1556469_s_at	0.38		
1558964_at	0.38	FAT tumor suppressor homolog 3 (Drosophila)	FAT3
1570289_at	0.38		
244269_at	0.37		
244451_x_at	0.37	thymine-DNA glycosylase	TDG
243211_at	0.37		
209552_at	0.37	paired box gene 8	PAX8
233927_at	0.37		
1552599_at	0.37	peroxisomal, testis specific 1	PXT1
215801_at	0.37		
228833_s_at	0.37		
209946_at	0.37	vascular endothelial growth factor C	VEGFC
217319_x_at	0.37	SCL/TAL1 interrupting locus#T-cell acute lymphocytic leukemia 1#PDZK1 interacting protein 1#cytochrome P450, family 4, subfamily Z, polypeptide 1#cytochrome P450, family 4, subfamily A, polypeptide 22	STIL#TAL1#PDZK1IP1#CYP4Z1#CYP4A22

215536_at	0.37	major histocompatibility complex, class II, DM alpha#major histocompatibility complex, class II, DM beta#major histocompatibility complex, class II, DO beta#major histocompatibility complex, class II, DQ beta 2#protein phosphatase 1, regulatory (inhibitor) subunit 2 pseudogene 1#proteasome (prosome, macropain) subunit, beta type, 8 (large multifunctional peptidase 7)#proteasome (prosome, macropain) subunit, beta type, 9 (large multifunctional peptidase 2)#transporter 1, ATP-binding cassette, sub-family B (MDR/TAP)#transporter 2, ATP-binding cassette, sub-family B (MDR/TAP)#major histocompatibility complex, class I, Z (pseudogene)	HLA-DMA#HLA-DMB#HLA-DOB#HLA-DQB2#PPP1R2P1#PSMB8#PSMB9#TAP1#TAP2#HLA-Z
204856_at	0.37	UDP-GlcNAc:betaGal beta-1,3-N-acetylglucosaminyltransferase 3	B3GNT3
1559393_at	0.37	aldehyde dehydrogenase 1 family, member L2	ALDH1L2
1553797_a_at	0.37		
239286_at	0.37		
205100_at	0.37	glutamine-fructose-6-phosphate transaminase 2	GFPT2
238272_at	0.37	thiopurine S-methyltransferase	TPMT
234330_at	0.37		
1560404_a_at	0.37	ATP binding domain 4	ATPBD4
1568876_a_at	0.37		
233915_at	0.37		
216992_s_at	0.37	glutamate receptor, metabotropic 8	GRM8
1560733_at	0.37		
228472_at	0.37		
216004_s_at	0.37	crystallin, alpha A#PBX/knotted 1 homeobox 1#U2 small nuclear RNA auxiliary factor 1	CRYAA#PKNOX1#U2AF1
1568590_at	0.37	ADP-ribosylation factor-like 3	ARL3
215053_at	0.37		
244336_at	0.37		
211331_x_at	0.37	hemochromatosis	HFE
1561652_at	0.37	beclin 1 (coiled-coil, myosin-like BCL2 interacting protein)	BECN1
239429_at	0.36		
234103_at	0.36		
1561882_at	0.36	synaptotagmin-like 3	SYTL3
1557832_at	0.36		
1556520_at	0.36		
205976_at	0.36	FAST kinase domains 2	FASTKD2
214935_at	0.36	nucleoporin 62kDa	NUP62
237488_at	0.36	heterogeneous nuclear ribonucleoprotein C (C1/C2)	HNRPC
1559159_at	0.36	centrosomal protein 68kDa	CEP68
235732_at	0.36		
242168_at	0.36	NADH dehydrogenase (ubiquinone) Fe-S protein 7, 20kDa (NADH-coenzyme Q reductase)	NDUFS7
217239_x_at	0.36		
202709_at	0.36	fibromodulin	FMOD
230485_at	0.36		
231060_at	0.36	heterogeneous nuclear ribonucleoprotein A/B	HNRPAB
233845_at	0.36		

1555095_at	0.36	chromosome 6 open reading frame 123	C6orf123
235326_at	0.36		
233433_at	0.36		
1564627_at	0.36	DnaJ (Hsp40) homolog, subfamily C, member 13	DNAJC13
229133_s_at	0.36	zinc finger protein 397	ZNF397
233777_at	0.36		
240189_at	0.36		
237566_at	0.36		
1554816_at	0.35	astrotactin 2	ASTN2
1559293_x_at	0.35	chromosome 9 open reading frame 14	C9orf14
224234_at	0.35		
215928_at	0.35		
220118_at	0.35	zinc finger and BTB domain containing 32	ZBTB32
233716_at	0.35		
231583_at	0.35	keratin 74	KRT74
223185_s_at	0.35	basic helix-loop-helix domain containing, class B, 3	BHLHB3
1557775_a_at	0.35		
241405_at	0.35		
207822_at	0.35	fibroblast growth factor receptor 1 (fms-related tyrosine kinase 2, Pfeiffer syndrome)	FGFR1
210643_at	0.35	tumor necrosis factor (ligand) superfamily, member 11	TNFSF11
216040_x_at	0.35		
1561606_at	0.35		
208274_at	0.35	oculomedin	OCLM
1557758_at	0.35		
208426_x_at	0.35	killer cell immunoglobulin-like receptor, two domains, long cytoplasmic tail, 4	KIR2DL4
244370_at	0.35		
235293_at	0.35		
1553523_at	0.35	NLR family, pyrin domain containing 14	NLRP14
244596_at	0.35		
237669_at	0.35	protein tyrosine phosphatase domain containing 1	PTPDC1
233585_at	0.35	sidekick homolog 2 (chicken)	SDK2
1557194_a_at	0.35		
1555734_x_at	0.34	adaptor-related protein complex 1, sigma 3 subunit	AP1S3
223612_s_at	0.34	ligand of numb-protein X 1	LNX1
1556111_s_at	0.34		
1553134_s_at	0.34	chromosome 9 open reading frame 72	C9orf72
228203_at	0.34	UDP-GlcNAc:betaGal beta-1,3-N-acetylglucosaminyltransferase 1	B3GNT1
211106_at	0.34	suppressor of Ty 3 homolog (S. cerevisiae)	SUPT3H
234939_s_at	0.34	PHD finger protein 12	PHF12
220589_s_at	0.34	integrin alpha FG-GAP repeat containing 2	ITFG2
230366_at	0.34		
222219_s_at	0.34	transducin-like enhancer of split 2 (E(sp1) homolog, Drosophila)#transducin-like enhancer of split 6 (E(sp1) homolog, Drosophila)	TLE2#TLE6
1555401_at	0.34	spermatogenesis and oogenesis specific basic helix-loop-helix 2	SOHLH2
218687_s_at	0.34	mucin 13, cell surface associated	MUC13
214572_s_at	0.34	insulin-like 3 (Leydig cell)	INSL3
233282_at	0.34		

241994_at	0.34	xanthine dehydrogenase	XDH
215770_at	0.34	olfactory receptor, family 7, subfamily E, member 2 pseudogene	OR7E2P
216749_at	0.33		
227318_at	0.33		
1562789_at	0.33	zinc finger protein 229	ZNF229
209589_s_at	0.33	EPH receptor B2	EPHB2
239218_at	0.33		
1563827_at	0.33		
231992_x_at	0.33		
1561303_at	0.33		
225147_at	0.33	pleckstrin homology, Sec7 and coiled-coil domains 3	PSCD3
1559568_at	0.33		
237648_x_at	0.33		
1553564_at	0.33	chromosome 20 open reading frame 133	C20orf133
233127_at	0.33		
236827_at	0.33		
229048_at	0.33		
243161_x_at	0.33	zinc finger protein 42 homolog (mouse)	ZFP42
206082_at	0.33	HLA complex P5	HCP5
210029_at	0.33	indoleamine-pyrrole 2,3 dioxygenase	INDO
227657_at	0.33	ring finger protein 150	RNF150
219949_at	0.33	leucine rich repeat containing 2	LRRC2
220310_at	0.32	tubulin, alpha-like 3	TUBAL3
239945_at	0.32		
232263_at	0.32	solute carrier family 6, member 15	SLC6A15
214732_at	0.32	Sp1 transcription factor	SP1
224590_at	0.32	X (inactive)-specific transcript	XIST
224514_x_at	0.32	interleukin 17 receptor C	IL17RC
207705_s_at	0.32		
209639_s_at	0.32	regulator of G-protein signalling 12	RGS12
221207_s_at	0.32	neurobeachin	NBEA
1559833_at	0.32	Rho guanine nucleotide exchange factor (GEF) 12	ARHGEF12
231327_at	0.32		
1564630_at	0.32	endothelin 1	EDN1
1553534_at	0.32	NLR family, pyrin domain containing 10	NLRP10
221309_at	0.32	RNA binding motif protein 17	RBM17
1556488_s_at	0.32	chromosome 3 open reading frame 15	C3orf15
217245_at	0.32		
220769_s_at	0.32	WD repeat domain 78	WDR78
230666_at	0.32		
207963_at	0.32	chromosome 6 open reading frame 54	C6orf54
221442_at	0.32	melanocortin 3 receptor	MC3R
1552976_at	0.32	DnaJ (Hsp40) related, subfamily B, member 13	DNAJB13
225968_at	0.32	prickle homolog 2 (Drosophila)	PRICKLE2
215368_at	0.32	nebulin	NEB
1569672_at	0.32		
224321_at	0.32	transmembrane protein with EGF-like and two follistatin-like domains 2	TMEFF2
1556813_at	0.32		
244865_at	0.32	HCLS1 associated protein X-1	HAX1
1563539_at	0.32		
230143_at	0.31	ring finger protein 165	RNF165

232321_at	0.31	mucin 17, cell surface associated	MUC17
1554122_a_at	0.31	hydroxysteroid (17-beta) dehydrogenase 12	HSD17B12
230726_at	0.31	mitochondrial ribosomal protein L38	MRPL38
234448_at	0.31		
240536_at	0.31		
234236_at	0.31		
221372_s_at	0.31	purinergic receptor P2X, ligand-gated ion channel, 2	P2RX2
220908_at	0.31	coiled-coil domain containing 33	CCDC33
235549_at	0.31	IBR domain containing 2	IBRDC2
240919_at	0.31		
215916_at	0.31	misshapen-like kinase 1 (zebrafish)	MINK1
217369_at	0.31	immunoglobulin heavy constant gamma 1 (G1m marker)	IGHG1
216387_x_at	0.31	olfactory receptor, family 7, subfamily E, member 111 pseudogene	OR7E111P
207665_at	0.31	ADAM metallopeptidase domain 21	ADAM21
230933_at	0.31	destrin (actin depolymerizing factor)	DSTN
1553208_s_at	0.31	ADP-ribosylation factor-like 10	ARL10
1559073_at	0.31	jumonji domain containing 2B	JMJD2B
204663_at	0.31	malic enzyme 3, NADP(+)-dependent, mitochondrial	ME3
233837_at	0.31	gamma-glutamyltransferase 1	GGT1
231654_s_at	0.31		
1553562_at	0.31	CD8b molecule	CD8B
227321_at	0.30		
1564323_at	0.30		
1563753_at	0.30		
240820_at	0.30		
1556697_at	0.30	GPRIN family member 3	GPRIN3
243174_at	0.30		
220880_at	0.30		
240469_at	0.30		
222927_s_at	0.30	complexin 3	CPLX3
234174_at	0.30		
238803_at	0.30	HECT domain containing 2	HECTD2
216131_at	0.30	FERM domain containing 4B	FRMD4B
1568856_at	0.30	neighbor of BRCA1 gene 1	NBR1
1564338_at	0.30		
1554302_s_at	0.30		
231046_at	0.30		
1557117_at	0.29		
209016_s_at	0.29	keratin 7	KRT7
231242_at	0.29	basic helix-loop-helix domain containing, class B, 3	BHLHB3
1553666_at	0.29	coiled-coil domain containing 34	CCDC34
243886_at	0.29		
211819_s_at	0.29	sorbin and SH3 domain containing 1	SORBS1
228102_at	0.29	neuropilin 2	NRP2
208352_x_at	0.29	ankyrin 1, erythrocytic	ANK1
1566249_at	0.29		
1553887_at	0.29		
1557120_at	0.29	eukaryotic translation elongation factor 1 alpha 1	EEF1A1
1568448_at	0.29		
210833_at	0.29	prostaglandin E receptor 3 (subtype EP3)	PTGER3
211359_s_at	0.29	opioid receptor, mu 1	OPRM1

221312_at	0.29	glucagon-like peptide 2 receptor	GLP2R
1559672_a_at	0.29	chromosome 9 open reading frame 93	C9orf93
215417_at	0.29	exocyst complex component 6B	EXOC6B
240334_at	0.29	leucine rich repeat and fibronectin type III domain containing 5	LRFN5
220324_at	0.29	chromosome 6 open reading frame 155	C6orf155
211826_s_at	0.29	myeloid/lymphoid or mixed-lineage leukemia (trithorax homolog, Drosophila)	MLL
1554170_a_at	0.28		
1560369_at	0.28	ankylosis, progressive homolog (mouse)	ANKH
1569809_at	0.28		
1569724_at	0.28		
230432_at	0.28		
1569783_at	0.28		
203798_s_at	0.28	visinin-like 1	VSNL1
237973_at	0.28		
1555068_at	0.28	WNK lysine deficient protein kinase 1	WNK1
229912_at	0.28	sidekick homolog 1 (chicken)	SDK1
1554850_at	0.28		
1559513_a_at	0.28	Fanconi anemia, complementation group C	FANCC
222783_s_at	0.28	SPARC related modular calcium binding 1	SMOC1
229942_at	0.28	basonuclin 2	BNC2
1559640_at	0.28	ankyrin-repeat and fibronectin type III domain containing 1	ANKFN1
221321_s_at	0.27	Kv channel interacting protein 2	KCNIP2
216408_at	0.27	olfactory receptor, family 2, subfamily B, member 2	OR2B2
1561448_at	0.27		
236925_at	0.27		
236304_at	0.27		
1561417_x_at	0.27		
203562_at	0.27	fasciculation and elongation protein zeta 1 (zygin I)	FEZ1
1567457_at	0.27	ras-related C3 botulinum toxin substrate 1 (rho family, small GTP binding protein Rac1)	RAC1
201905_s_at	0.27	CTD (carboxy-terminal domain, RNA polymerase II, polypeptide A) small phosphatase-like	CTDSPL
242326_at	0.27	collagen, type XXII, alpha 1	COL22A1
241044_x_at	0.27		
208145_at	0.27		
233162_at	0.27		
1569417_at	0.27		
238971_at	0.27		
230271_at	0.27	one cut domain, family member 2	ONECUT2
239650_at	0.27		
229349_at	0.26	lin-28 homolog B (C. elegans)	LIN28B
1561232_at	0.26		
1568638_a_at	0.26	indoleamine-pyrrole 2,3 dioxygenase-like 1	INDOL1
207692_s_at	0.26	aggrecan 1 (chondroitin sulfate proteoglycan 1, large aggregating proteoglycan, antigen identified b	AGC1
217696_at	0.26	fucosyltransferase 7 (alpha (1,3) fucosyltransferase)	FUT7
240022_at	0.26	chromosome 19 open reading frame 7	C19orf7
1552991_at	0.26	olfactory receptor, family 5, subfamily P, member 2	OR5P2
244668_at	0.26		
1564131_a_at	0.26		

242018_at	0.26	son of sevenless homolog 1 (Drosophila)	SOS1
235501_at	0.26		
241733_at	0.26	chromosome 18 open reading frame 54	C18orf54
228074_at	0.26		
1569218_at	0.26		
239502_at	0.26		
238753_at	0.26	frequenin homolog (Drosophila)	FREQ
219685_at	0.26	transmembrane protein 35	TMEM35
1553799_at	0.26	chromosome 15 open reading frame 33	C15orf33
214677_x_at	0.26	immunoglobulin lambda joining 3	IGLJ3
204653_at	0.26	transcription factor AP-2 alpha (activating enhancer binding protein 2 alpha)	TFAP2A
210366_at	0.26	solute carrier organic anion transporter family, member 1B1	SLCO1B1
1553734_at	0.26	adenylate kinase 7	AK7
210989_at	0.25	laminin, alpha 4	LAMA4
230928_at	0.25		
206344_at	0.25	paraoxonase 1	PON1
1559665_at	0.25		
238577_s_at	0.25		
232190_x_at	0.25		
208460_at	0.25	gap junction protein, alpha 7, 45kDa	GJA7
1558881_at	0.25		
240018_at	0.25		
237862_at	0.25		
210662_at	0.25	kynureninase (L-kynurenine hydrolase)	KYNU
1553859_at	0.25	tryptophan hydroxylase 1 (tryptophan 5-monooxygenase)	TPH1
206324_s_at	0.25	death-associated protein kinase 2	DAPK2
235379_at	0.25		
1554288_at	0.25	KIAA1600	KIAA1600
1570536_at	0.25		
242478_at	0.25		
220672_at	0.25	KIAA1622	KIAA1622
1552540_s_at	0.25	IQ motif containing D	IQCD
1557584_at	0.25		
220596_at	0.25	G patch domain containing 4	GPATCH4
243573_at	0.25		
232163_at	0.24	WD repeat domain 19	WDR19
241963_at	0.24	zinc finger protein 704	ZNF704
233312_at	0.24		
242697_at	0.24	zinc finger protein 540	ZNF540

234655_at	0.24	annexin A2 pseudogene 2#T cell receptor beta variable 21/OR9-2#T cell receptor beta variable 24/OR9-2#T cell receptor beta variable 20/OR9-2#T cell receptor beta variable orphans on chromosome 9#T cell receptor beta variable 23/OR9-2#T cell receptor beta variable 22/OR9-2#null#suppressor of G2 allele of SKP1 pseudogene (S. cerevisiae)#ankyrin repeat domain 18B	ANXA2P2#TRBV21OR9-2#TRBV24OR9-2#TRBV20OR9-2#TRBVOR9@#TRBV23OR9-2#TRBV22OR9-2#null#SUGT1P#ANKRD18B
242822_at	0.24		
1564949_at	0.24		
235763_at	0.24	solute carrier family 44, member 5	SLC44A5
217533_x_at	0.24		
215115_x_at	0.24	neurotrophic tyrosine kinase, receptor, type 3	NTRK3
1564525_at	0.24		
240201_at	0.24		
234004_at	0.24	tetratricopeptide repeat domain 28	TTC28
203868_s_at	0.23	vascular cell adhesion molecule 1	VCAM1
215073_s_at	0.23	nuclear receptor subfamily 2, group F, member 2	NR2F2
1553422_s_at	0.23		
229495_at	0.23	aminoacylase 1-like 2	ACY1L2
206597_at	0.23	neural retina leucine zipper	NRL
1560630_at	0.23		
230346_x_at	0.23		
219795_at	0.23	solute carrier family 6 (amino acid transporter), member 14	SLC6A14
1562459_at	0.23		
241909_at	0.23	tankyrase, TRF1-interacting ankyrin-related ADP-ribose polymerase 2	TNKS2
1559526_at	0.23		
206434_at	0.23	sparc/osteonectin, cwcv and kazal-like domains proteoglycan (testican) 3	SPOCK3
236071_at	0.23		
233073_at	0.23		
1563135_at	0.23		

234013_at	0.23	T cell receptor alpha locus#T cell receptor alpha variable 6#T cell receptor alpha variable 16#T cell receptor alpha variable 15#T cell receptor alpha variable 14/delta variable 4#T cell receptor alpha variable 13-2#T cell receptor alpha variable 13-1#T cell receptor alpha variable 12-3#T cell receptor alpha variable 12-2#T cell receptor alpha variable 12-1#T cell receptor alpha variable 11#T cell receptor alpha variable 10#T cell receptor alpha variable 9-2#T cell receptor alpha variable 9-1#T cell receptor alpha variable 8-6#T cell receptor alpha variable 8-5#T cell receptor alpha variable 8-4#T cell receptor alpha variable 8-3#T cell receptor alpha variable 8-2#T cell receptor alpha variable 8-1#T cell receptor alpha variable 7#T cell receptor alpha variable 5#null	TRA@#TRAV6#TRAV16#TRAV15#TRAV14DV4#TRAV13-2#TRAV13-1#TRAV12-3#TRAV12-2#TRAV12-1#TRAV11#TRAV10#TRAV9-2#TRAV9-1#TRAV8-6#TRAV8-5#TRAV8-4#TRAV8-3#TRAV8-2#TRAV8-1#TRAV7#TRAV5#null
236197_at	0.23		
236570_at	0.23	zinc finger protein 366	ZNF366
242107_x_at	0.23		
244493_at	0.23	G protein-coupled receptor 22	GPR22
233424_at	0.23		
220639_at	0.23	transmembrane 4 L six family member 20	TM4SF20
243841_at	0.23	spectrin repeat containing, nuclear envelope 2	SYNE2
1566937_at	0.23		
237730_at	0.22		
205699_at	0.22	mitogen-activated protein kinase kinase 6	MAP2K6
1556325_at	0.22	filamin A interacting protein 1	FILIP1
216290_x_at	0.22		
1552604_at	0.22	chromosome 21 open reading frame 74	C21orf74
207498_s_at	0.22	cytochrome P450, family 2, subfamily D, polypeptide 6	CYP2D6
238385_at	0.22	chromosome 6 open reading frame 58	C6orf58
224045_x_at	0.22	chromosome 18 open reading frame 2	C18orf2
227885_at	0.22		
1560990_a_at	0.22		
209631_s_at	0.22	G protein-coupled receptor 37 (endothelin receptor type B-like)	GPR37
207580_at	0.22	melanoma antigen family B, 4	MAGEB4
243911_at	0.22		
1561289_at	0.22		
240354_at	0.22	chromosome 12 open reading frame 54	C12orf54
1569624_at	0.22		
1556362_at	0.22		
235565_at	0.22	zinc finger protein 425	ZNF425
234015_at	0.22		
216084_at	0.21	chromosome 9 open reading frame 144	C9orf144
207478_at	0.21		
232694_at	0.21	zinc finger protein 395	ZNF395
237411_at	0.21	ADAM metallopeptidase with thrombospondin type 1 motif, 6	ADAMTS6

243130_at	0.21		
1556156_at	0.21	estrogen-related receptor beta	ESRRB
242464_at	0.21		
1560854_s_at	0.21	zinc finger protein 588	ZNF588
242849_at	0.21	SAM and SH3 domain containing 1	SASH1
242623_x_at	0.21		
208508_s_at	0.21	olfactory receptor, family 2, subfamily J, member 2	OR2J2
1554781_at	0.21		
216192_at	0.21	fatty acid binding protein 7, brain	FABP7
223861_at	0.21	HORMA domain containing 1	HORMAD1
1555339_at	0.21	RAP1A, member of RAS oncogene family	RAP1A
223805_at	0.21	oxysterol binding protein-like 6	OSBPL6
238492_at	0.21		
227088_at	0.21	phosphodiesterase 5A, cGMP-specific	PDE5A
239612_at	0.21		
1568805_at	0.21	nuclear receptor coactivator 7	NCOA7
1559252_a_at	0.20	chromosome 20 open reading frame 29	C20orf29
218468_s_at	0.20	gremlin 1, cysteine knot superfamily, homolog (Xenopus laevis)	GREM1
1562860_at	0.20		
1563465_at	0.20	polycystic kidney disease 1 like 1	PKD1L1
238111_at	0.20	serologically defined colon cancer antigen 3	SDCCAG3
234690_at	0.20		
207723_s_at	0.20	killer cell lectin-like receptor subfamily C, member 3	KLRC3
217017_at	0.20	oxysterol binding protein-like 10	OSBPL10
238282_at	0.20		
206930_at	0.20	glycine-N-acyltransferase	GLYAT
224103_at	0.20		
1560570_a_at	0.20		
230923_at	0.20	family with sequence similarity 19 (chemokine (C-C motif)-like), member A1	FAM19A1
233838_at	0.20		
1552797_s_at	0.20	prominin 2	PROM2
221546_at	0.19	PRP18 pre-mRNA processing factor 18 homolog (S. cerevisiae)	PRPF18
209821_at	0.19	interleukin 33	IL33
206727_at	0.19	complement component 9	C9
1570038_at	0.19	zinc finger protein 718	ZNF718
232246_at	0.19		
1555385_at	0.19	beta-1,4-N-acetyl-galactosaminyl transferase 1	B4GALNT1
242719_at	0.19		
234108_at	0.19	taste receptor, type 2, member 45	TAS2R45
211608_at	0.19		
240330_at	0.19		
215039_at	0.19		
1559462_at	0.19		
235704_at	0.19	DAZ associated protein 2	DAZAP2
241149_at	0.19		
1555340_x_at	0.19	RAP1A, member of RAS oncogene family	RAP1A
237395_at	0.19	cytochrome P450, family 4, subfamily Z, polypeptide 1	CYP4Z1
1562876_s_at	0.19		
1557821_at	0.19		

214889_at	0.19		
213664_at	0.19	solute carrier family 1 (neuronal/epithelial high affinity glutamate transporter, system Xag), membe	SLC1A1
236627_at	0.19		
239697_x_at	0.19		
233821_at	0.19	RAB32, member RAS oncogene family	RAB32
1552765_x_at	0.19	transmembrane protein 67	TMEM67
217551_at	0.19		
220773_s_at	0.18	gephyrin	GPHN
240927_at	0.18		
205343_at	0.18	sulfotransferase family, cytosolic, 1C, member 1	SULT1C1
224081_at	0.18		
224022_x_at	0.18	wingless-type MMTV integration site family, member 16	WNT16
215571_at	0.18		
215282_at	0.18	anaphase promoting complex subunit 13	ANAPC13
214967_at	0.18		
1556487_a_at	0.18	chromosome 3 open reading frame 15	C3orf15
240844_at	0.18		
1560286_s_at	0.18		
58916_at	0.18	potassium channel tetramerisation domain containing 14	KCTD14
240720_at	0.18		
229857_s_at	0.18		
237920_at	0.18	synaptonemal complex protein 2	SYCP2
234271_at	0.17	otopetrin 2	OTOP2
232611_at	0.17		
1552919_at	0.17	chromosome 4 open reading frame 36	C4orf36
230419_at	0.17		
1554929_at	0.17		
229667_s_at	0.17	homeobox B8	HOXB8
1554500_a_at	0.17	regulator of G-protein signalling 7	RGS7
237099_at	0.17	chromosome 20 open reading frame 70	C20orf70
214837_at	0.17	albumin	ALB
216516_at	0.17		
204437_s_at	0.17	folate receptor 1 (adult)	FOLR1
242680_at	0.17		
1560111_at	0.17		
241195_at	0.17		
236740_at	0.17		
204525_at	0.16	PHD finger protein 14	PHF14
232268_at	0.16		
237690_at	0.16	G protein-coupled receptor 115	GPR115
1555925_at	0.16		
1559376_at	0.16	chromosome 1 open reading frame 203	C1orf203
1555752_at	0.16		
216163_at	0.16		
206211_at	0.16	selectin E (endothelial adhesion molecule 1)	SELE
241257_at	0.16		
1557793_at	0.16	family with sequence similarity 62 (C2 domain containing), member C	FAM62C
1558682_at	0.16	high mobility group AT-hook 2	HMGA2
243998_at	0.16	keratin 222 pseudogene	KRT222P

220550_at	0.16	F-box protein 4	FBXO4
1553792_at	0.15	KIAA1109	KIAA1109
241653_x_at	0.15		
236651_at	0.15	kalirin, RhoGEF kinase	KALRN
1561713_at	0.15		
1560684_x_at	0.15	B-cell CLL/lymphoma 8	BCL8
1560424_at	0.15		
1566740_at	0.15	phospholipase C, epsilon 1	PLCE1
215755_at	0.15		
1554027_a_at	0.15	solute carrier family 4, sodium bicarbonate cotransporter, member 4	SLC4A4
224030_s_at	0.15		
239814_at	0.15		
1570490_at	0.15		
229386_at	0.15	inhibitor of DNA binding 4, dominant negative helix-loop-helix protein	ID4
232777_s_at	0.15	chromosome 6 open reading frame 118	C6orf118
1568931_at	0.15		
216420_at	0.15	TAR DNA binding protein-like	TARDBPL
1569780_at	0.15		
1560142_at	0.15	glutamate receptor, ionotropic, kainate 2	GRIK2
244558_at	0.15		
1552463_at	0.15	serpin peptidase inhibitor, clade B (ovalbumin), member 11	SERPINB11
1562406_at	0.15		
211161_s_at	0.14	collagen, type III, alpha 1 (Ehlers-Danlos syndrome type IV, autosomal dominant)	COL3A1
237602_at	0.14		
242102_at	0.14		
230249_at	0.14	KH domain containing, RNA binding, signal transduction associated 3	KHDRBS3
1555074_a_at	0.14	potassium voltage-gated channel, subfamily H (eag-related), member 5	KCNH5
216856_s_at	0.14	deleted in lymphocytic leukemia, 2	DLEU2
1553796_at	0.14		
230254_at	0.14		
217578_at	0.14		
203684_s_at	0.14	B-cell CLL/lymphoma 2	BCL2
1558494_at	0.14	cadherin 12, type 2 (N-cadherin 2)	CDH12
243990_at	0.14		
230718_at	0.13	heat shock transcription factor family member 5	HSF5
1560078_at	0.13	laminin, alpha 3	LAMA3
214605_x_at	0.13	G protein-coupled receptor 1	GPR1
1554492_at	0.13	thyroid adenoma associated	THADA
1557398_at	0.13		
217287_s_at	0.13	transient receptor potential cation channel, subfamily C, member 6	TRPC6
1558867_at	0.13	dermatan sulfate epimerase	DSE
223918_at	0.13	acyl-CoA synthetase long-chain family member 6	ACSL6
1552791_a_at	0.13	triadin	TRDN
1552858_at	0.13	melanoma antigen family B, 6	MAGEB6
229960_at	0.13	mitogen-activated protein kinase kinase kinase 6	MAP3K6
1556521_a_at	0.13		

210292_s_at	0.13	protocadherin 11 X-linked	PCDH11X
222249_at	0.12		
240085_at	0.12		
237770_at	0.12		
238441_at	0.12		
240556_at	0.12	decorin	DCN
1561271_at	0.12		
240448_at	0.12	KIAA0802	KIAA0802
237619_at	0.12	chromosome 6 open reading frame 146	C6orf146
221874_at	0.12	KIAA1324	KIAA1324
237622_at	0.12		
1569858_at	0.12		
237351_at	0.12		
238392_at	0.12	translocation associated membrane protein 2	TRAM2
231351_at	0.11		
1558977_at	0.11		
1561877_at	0.11		
221023_s_at	0.11	potassium voltage-gated channel, subfamily H (eag-related), member 6	KCNH6
237831_x_at	0.11	methylmalonic aciduria (cobalamin deficiency) cblA type	MMAA
1562991_at	0.11	zinc finger protein 292	ZNF292
240904_at	0.11		
202995_s_at	0.10	fibulin 1	FBLN1
239436_at	0.10	cysteine and histidine-rich domain (CHORD)-containing 1	CHORDC1
1562736_at	0.10	LIM homeobox 9	LHX9
234090_at	0.10		
209869_at	0.10	adrenergic, alpha-2A-, receptor	ADRA2A
1559839_at	0.09	T-box 18	TBX18
244098_at	0.09	ADAM metallopeptidase with thrombospondin type 1 motif, 3	ADAMTS3
240474_x_at	0.09		
1555492_a_at	0.09	bestrophin 3	BEST3
237667_at	0.09		
1565228_s_at	0.08	albumin	ALB
1553811_at	0.08		
1569406_at	0.07		
242322_at	0.07		
238372_s_at	0.07		
231580_at	0.07		
234184_at	0.07		
1562477_at	0.07	early B-cell factor 2	EBF2
1562879_at	0.06		
239072_at	0.05		
224024_at	0.03	endoplasmic reticulum-golgi intermediate compartment (ERGIC) 1	ERGIC1
1568919_at	0.03		
241868_at	0.02		
