# Supplementary material for: In Vitro Transformation of Primary Human CD34+ Cells by AML Fusion Oncogenes: Early Gene Expression Profiling Reveals Possible Drug Target in AML
Source: PLoS One. 2010 Aug 27;5(8):e12464. doi: 10.1371/journal.pone.0012464 (PMC2929205; doi:10.1371/journal.pone.0012464)
Supplement: Table S16 — Genes deregulated by PML-RARA 6 h after transfection. Primary human CD34+ cells were nucleofected with either control pTracer-CMV/Bsd vector or vector expressing PML-RARA and sorted for GFP positivity. Total RNA was extracted 6 h after nucleofection and subjected to microarray analysis. Microarray data were analyzed by SAM as described in Materials and Methods. Significantly deregulated genes are listed and the false discovery rate (FDR) is shown. (0.40 MB PDF) [file pone.0012464.s016.pdf]

Table S16. Genes deregulated by PML-RARA at 6 h detected by SAM FDR = 0.62%

Probe set ID	Fold Change	Gene Name	Gene Symbol
211605_s_at	238.57	retinoic acid receptor, alpha	RARA
1563207_at	42.96	chromosome 3 open reading frame 65	C3orf65
210439_at	38.68	inducible T-cell co-stimulator	ICOS
1568673_s_at	26.13	ELL associated factor 2	EAF2
241579_at	21.09		
1556700_a_at	20.82		
205709_s_at	19.99	CDP-diacylglycerol synthase (phosphatidate cytidylyltransferase) 1	CDS1
243644_at	19.97		
231560_at	16.21	leucine rich repeat containing 34	LRRC34
201744_s_at	14.39	lumican	LUM
210119_at	14.10	potassium inwardly-rectifying channel, subfamily J, member 15	KCNJ15
1556609_at	13.49		
214930_at	13.29	SLIT and NTRK-like family, member 5	SLITRK5
235147_at	12.94		
1556786_at	12.34	phosphodiesterase 5A, cGMP-specific	PDE5A
204719_at	12.31	ATP-binding cassette, sub-family A (ABC1), member 8	ABCA8
213435_at	12.21	SATB family member 2	SATB2
1553276_at	11.72	zinc finger protein 560	ZNF560
1552806_a_at	11.02	sialic acid binding Ig-like lectin 10	SIGLEC10
219196_at	10.74	secretogranin III	SCG3
216300_x_at	10.40	retinoic acid receptor, alpha	RARA
223642_at	10.37	Zic family member 2 (odd-paired homolog, Drosophila)	ZIC2
222355_at	10.31		
224545_at	10.28		
244537_at	10.26	huntingtin interacting protein 2	HIP2
207147_at	10.14	distal-less homeobox 2	DLX2
214887_at	10.01		
234623_x_at	9.97		
223279_s_at	9.86	uveal autoantigen with coiled-coil domains and ankyrin repeats	UACA
207099_s_at	9.77	choroideremia (Rab escort protein 1)	CHM
237123_x_at	9.68	kelch-like 9 (Drosophila)	KLHL9
1564787_at	9.26		
227911_at	8.69	Rho GTPase activating protein 28	ARHGAP28
1561961_at	8.56		
214146_s_at	8.40	pro-platelet basic protein (chemokine (C-X-C motif) ligand 7)	PPBP
241038_at	8.31		
208142_at	8.30	family with sequence similarity 12, member A	FAM12A
1558135_at	8.20	TAF11 RNA polymerase II, TATA box binding protein (TBP)-associated factor, 28kDa	TAF11
220845_at	8.18	acyl-Coenzyme A oxidase-like	ACOXL
230368_at	8.01	glycogen synthase kinase 3 alpha	GSK3A
242721_at	7.76	autism susceptibility candidate 2	AUTS2

244853_at	7.47		
241420_at	7.40		
218541_s_at	7.40	chromosome 8 open reading frame 4	C8orf4
226534_at	7.30	KIT ligand	KITLG
241805_at	7.28	gamma-aminobutyric acid (GABA) A receptor, gamma 1	GABRG1
223739_at	7.19	peptidyl arginine deiminase, type I	PADI1
236774_at	7.00		
1557838_at	6.88		
1553593_a_at	6.79	T-cell acute lymphocytic leukemia 2	TAL2
1561200_at	6.79		
243727_at	6.74	copine VIII	CPNE8
220030_at	6.71	serine/threonine/tyrosine kinase 1	STYK1
239155_at	6.57		
1556810_a_at	6.56		
235944_at	6.52	hemicentin 1	HMCN1
1557321_a_at	6.52	calpain 14	CAPN14
220146_at	6.47	toll-like receptor 7	TLR7
238280_at	6.41		
231025_at	6.38		
219522_at	6.36	four jointed box 1 (Drosophila)	FJX1
206023_at	6.28	neuromedin U	NMU
240186_at	6.24		
233011_at	6.20	annexin A1	ANXA1
1559462_at	6.19		
1557535_at	6.17	palladin, cytoskeletal associated protein	PALLD
1562754_at	6.11		
235976_at	6.07	SLIT and NTRK-like family, member 6	SLITRK6
211819_s_at	6.02	sorbin and SH3 domain containing 1	SORBS1
221805_at	5.99	neurofilament, light polypeptide 68kDa	NEFL
230804_at	5.94	chromosome 6 open reading frame 194	C6orf194
235431_s_at	5.91	pellino homolog 3 (Drosophila)	PELI3
201289_at	5.85	cysteine-rich, angiogenic inducer, 61	CYR61
234167_at	5.84	MKI67 (FHA domain) interacting nucleolar phosphoprotein	MKI67IP
1569459_a_at	5.83		
207864_at	5.78	sodium channel, voltage-gated, type VII, alpha	SCN7A
1557348_at	5.76		
211832_s_at	5.75	Mdm2, transformed 3T3 cell double minute 2, p53 binding protein (mouse)	MDM2
216433_s_at	5.71	PR domain containing 2, with ZNF domain	PRDM2
1557807_a_at	5.71		
214828_s_at	5.70		
238018_at	5.66		
205104_at	5.62	syntaphilin	SNPH
219414_at	5.62	calsyntenin 2	CLSTN2
233591_at	5.60		
219874_at	5.53	solute carrier family 12 (potassium/chloride transporters), member 8	SLC12A8
1562805_at	5.52		
229485_x_at	5.47		
222202_at	5.45		

229683_s_at	5.42	potassium channel tetramerisation domain containing 15	KCTD15
206591_at	5.42	recombination activating gene 1	RAG1
241890_at	5.41		
206157_at	5.38	pentraxin-related gene, rapidly induced by IL-1 beta	PTX3
231581_at	5.34		
1557346_a_at	5.30		
1560135_at	5.26		
1562270_at	5.21	Rho guanine nucleotide exchange factor (GEF) 7	ARHGEF7
228523_at	5.18	nanos homolog 1 (Drosophila)	NANOS1
1565614_at	5.17	zinc finger protein 337	ZNF337
204359_at	5.17	fibronectin leucine rich transmembrane protein 2	FLRT2
206179_s_at	5.16		
233744_at	5.14	RAS-like, estrogen-regulated, growth inhibitor	RERG
206300_s_at	5.14	parathyroid hormone-like hormone	PTHLH
227487_s_at	5.09	serpin peptidase inhibitor, clade E (nexin, plasminogen activator inhibitor type 1), member 2	SERPINE2
207689_at	5.06	T-box 10	TBX10
241232_x_at	5.05		
220158_at	5.05	lectin, galactoside-binding, soluble, 14	LGALS14
235490_at	5.04	transmembrane protein 107	TMEM107
214368_at	5.04	RAS guanyl releasing protein 2 (calcium and DAG-regulated)	RASGRP2
216856_s_at	5.04	deleted in lymphocytic leukemia, 2	DLEU2
236300_at	5.02		
1561010_a_at	5.01	monoamine oxidase B	MAOB
1558882_at	4.99		
214467_at	4.99	G protein-coupled receptor 65	GPR65
221594_at	4.99		
1554831_x_at	4.98	amyotrophic lateral sclerosis 2 (juvenile) chromosome region, candidate 11	ALS2CR11
1570038_at	4.96	zinc finger protein 718	ZNF718
206366_x_at	4.92	chemokine (C motif) ligand 1	XCL1
232481_s_at	4.90	SLIT and NTRK-like family, member 6	SLITRK6
240465_at	4.86		
212611_at	4.85		
1563260_at	4.85	chromosome 9 open reading frame 107	C9orf107
233485_at	4.82		
227006_at	4.82	protein phosphatase 1, regulatory (inhibitor) subunit 14A	PPP1R14A
244785_at	4.81		
240161_s_at	4.79	cell division cycle 20 homolog B (S. cerevisiae)	CDC20B
238661_at	4.77		
205867_at	4.77	protein tyrosine phosphatase, non-receptor type 11 (Noonan syndrome 1)	PTPN11
1560305_x_at	4.77		
237385_at	4.75		
206665_s_at	4.71	BCL2-like 1	BCL2L1

204555_s_at	4.69	protein phosphatase 1, regulatory (inhibitor) subunit 3D	PPP1R3D
205303_at	4.69	potassium inwardly-rectifying channel, subfamily J, member 8	KCNJ8
235297_at	4.69	CUG triplet repeat, RNA binding protein 1	CUGBP1
219377_at	4.66	family with sequence similarity 59, member A	FAM59A
229387_at	4.66	leucine-rich repeats and calponin homology (CH) domain containing 3	LRCH3
240073_at	4.64	male-specific lethal 2-like 1 (Drosophila)	MSL2L1
216239_at	4.63	Bardet-Biedl syndrome 9	BBS9
203697_at	4.63	frizzled-related protein	FRZB
1561965_at	4.61		
1562106_at	4.58		
205618_at	4.57	proline rich Gla (G-carboxyglutamic acid) 1	PRRG1
215586_at	4.56		
217580_x_at	4.56		
201506_at	4.56	transforming growth factor, beta-induced, 68kDa	TGFBI
219628_at	4.54	zinc finger, matrin type 3	ZMAT3
209189_at	4.53	v-fos FBJ murine osteosarcoma viral oncogene homolog	FOS
1558365_at	4.52		
215551_at	4.51	estrogen receptor 1	ESR1
228507_at	4.50		
209603_at	4.50	GATA binding protein 3	GATA3
213496_at	4.49		
1564007_at	4.49		
1556187_at	4.49		
203038_at	4.45	protein tyrosine phosphatase, receptor type, K	PTPRK
1560494_a_at	4.45	CPX chromosome region, candidate 1	CPXCR1
1569023_a_at	4.44		
216279_at	4.43	zinc finger protein 460	ZNF460
202388_at	4.42	regulator of G-protein signalling 2, 24kDa	RGS2
221696_s_at	4.42	serine/threonine/tyrosine kinase 1	STYK1
1561136_at	4.41	glycophorin E	GYPE
1552826_at	4.38	solute carrier family 26, member 7	SLC26A7
205789_at	4.36	CD1d molecule	CD1D
215351_at	4.33	RNA terminal phosphate cyclase domain 1	RTCD1
217633_at	4.33	URB1 ribosome biogenesis 1 homolog (S. cerevisiae)	URB1
241643_at	4.33	tousled-like kinase 1	TLK1
235652_at	4.32		
239936_at	4.32	deleted in lymphocytic leukemia, 2	DLEU2
202274_at	4.32	actin, gamma 2, smooth muscle, enteric	ACTG2
1552698_at	4.30		
207517_at	4.28	laminin, gamma 2	LAMC2
204557_s_at	4.28	DAZ interacting protein 1	DZIP1

232751_at	4.27	Sec23 homolog B (S. cerevisiae)#polymerase (RNA) III (DNA directed) polypeptide F, 39 kDa#retinoblastoma binding protein 9#chromosome 20 open reading frame 12#ribosomal protein S19 pseudogene 1#ribosomal protein L21 pseudogene 3#glucosaminyl (N-acetyl) transferase 1, core 2 (beta-1,6-N-acetylglucosaminyltransferase) pseudogene	SEC23B#POLR3F#RBBP9#C20orf12#RPS19P1#RPL21P3#GCNT1P
225806_at	4.26	jub, ajuba homolog (Xenopus laevis)	JUB
1561615_s_at	4.26	solute carrier family 8 (sodium/calcium exchanger), member 1	SLC8A1
1554492_at	4.23	thyroid adenoma associated	THADA
208416_s_at	4.21	spectrin, beta, erythrocytic (includes spherocytosis, clinical type I)	SPTB
202609_at	4.20	epidermal growth factor receptor pathway substrate 8	EPS8
1565681_s_at	4.20	DIP2 disco-interacting protein 2 homolog C (Drosophila)	DIP2C
244071_at	4.20		
222456_s_at	4.20	LIM domain and actin binding 1	LIMA1
1567612_at	4.19		
217400_at	4.19	proliferating cell nuclear antigen pseudogene	PCNAP
225725_at	4.19		
228964_at	4.18	PR domain containing 1, with ZNF domain	PRDM1
1557029_at	4.17		
231131_at	4.16		
244583_at	4.15		
1565881_at	4.15		
214079_at	4.14	dehydrogenase/reductase (SDR family) member 2	DHRS2
217382_at	4.14		
234713_x_at	4.13		
241491_at	4.12		
227629_at	4.12	prolactin receptor	PRLR
1556876_s_at	4.11		
1561125_at	4.11	methylenetetrahydrofolate dehydrogenase (NADP+ dependent) 1-like	MTHFD1L
233295_at	4.11		
210113_s_at	4.10	NLR family, pyrin domain containing 1	NLRP1
227705_at	4.09	transcription elongation factor A (SII)-like 7	TCEAL7
1566881_at	4.09		
1560483_at	4.08		
240202_x_at	4.08	MAP/microtubule affinity-regulating kinase 1	MARK1
237603_at	4.07	chromosome 1 open reading frame 100	C1orf100
202497_x_at	4.07	solute carrier family 2 (facilitated glucose transporter), member 3	SLC2A3
232295_at	4.06	G elongation factor, mitochondrial 1	GFM1
1558466_at	4.05		
233031_at	4.05	zinc finger homeobox 1b	ZFHX1B
1561663_at	4.05		
215072_x_at	4.04	chromosome 10 open reading frame 137	C10orf137
206655_s_at	4.04	glycoprotein Ib (platelet), beta polypeptide	GP1BB

232864_s_at	4.03	AF4/FMR2 family, member 4	AFF4
233314_at	4.03	phosphatase and tensin homolog (mutated in multiple advanced cancers 1)	PTEN
228315_at	4.02		
206346_at	4.02	prolactin receptor	PRLR
1561083_at	4.00		
224965_at	3.98	guanine nucleotide binding protein (G protein), gamma 2	GNG2
230520_at	3.98	androgen-induced 1	AIG1
1554649_at	3.97		
1570470_at	3.95	chromosome 14 open reading frame 161	C14orf161
244678_at	3.93	vacuolar protein sorting 13 homolog A (S. cerevisiae)	VPS13A
219815_at	3.90	galactose-3-O-sulfotransferase 4	GAL3ST4
206933_s_at	3.90	hexose-6-phosphate dehydrogenase (glucose 1-dehydrogenase)	H6PD
221421_s_at	3.89	ADAM metallopeptidase with thrombospondin type 1 motif, 12	ADAMTS12
210127_at	3.89	RAB6B, member RAS oncogene family	RAB6B
244146_at	3.89	DTW domain containing 1	DTWD1
205536_at	3.88	vav 2 oncogene	VAV2
231696_x_at	3.88	transmembrane protein 50B	TMEM50B
205249_at	3.87	early growth response 2 (Krox-20 homolog, Drosophila)	EGR2
203032_s_at	3.87	fumarate hydratase	FH
1556777_a_at	3.87		
220295_x_at	3.87	DEP domain containing 1	DEPDC1
238264_at	3.87	NMD3 homolog (S. cerevisiae)	NMD3
1559950_at	3.86		
1557672_s_at	3.86		
1570153_at	3.85	spermatogenesis and oogenesis specific basic helix-loop-helix 2	SOHLH2
228027_at	3.85		
241483_at	3.85		
201939_at	3.85	polo-like kinase 2 (Drosophila)	PLK2
232278_s_at	3.85	DEP domain containing 1	DEPDC1
236281_x_at	3.84	5-hydroxytryptamine (serotonin) receptor 7 (adenylate cyclase-coupled)	HTR7
227623_at	3.84		
241235_at	3.83		
242513_x_at	3.83	KIAA2018	KIAA2018
1569213_at	3.82		
226498_at	3.82		
209598_at	3.81	paraneoplastic antigen MA2	PNMA2
207120_at	3.81	zinc finger protein 667	ZNF667
1554534_at	3.80	dihydropyrimidine dehydrogenase	DPYD
1563000_at	3.79		
237007_at	3.79	potassium voltage-gated channel, Shab-related subfamily, member 2	KCNB2
228584_at	3.79	sarcoglycan, beta (43kDa dystrophin-associated glycoprotein)	SGCB
209906_at	3.76	complement component 3a receptor 1	C3AR1
211371_at	3.75	mitogen-activated protein kinase kinase 5	MAP2K5

241929_at	3.75		
1561425_a_at	3.75	zinc finger protein 568	ZNF568
1553326_at	3.74	relaxin/insulin-like family peptide receptor 2	RXFP2
227803_at	3.74	ectonucleotide pyrophosphatase/phosphodiesterase 5 (putative function)	ENPP5
232868_at	3.74	chromosome 9 open reading frame 11	C9orf11
234034_at	3.74		
216236_s_at	3.73	solute carrier family 2 (facilitated glucose transporter), member 3	SLC2A3
219736_at	3.72	tripartite motif-containing 36	TRIM36
1561002_at	3.71		
226446_at	3.70	hairy and enhancer of split 6 (Drosophila)	HES6
235395_at	3.70	SEC63 homolog (S. cerevisiae)	SEC63
222088_s_at	3.69	solute carrier family 2 (facilitated glucose transporter), member 3	SLC2A3
203404_at	3.69	armadillo repeat containing, X-linked 2	ARMCX2
203821_at	3.69	heparin-binding EGF-like growth factor	HBEGF
1554742_at	3.67	PMS1 postmeiotic segregation increased 1 (S. cerevisiae)	PMS1
239825_at	3.67	activating transcription factor 6	ATF6
1558324_a_at	3.67		
212850_s_at	3.67	low density lipoprotein receptor-related protein 4	LRP4
226837_at	3.66	sprouty-related, EVH1 domain containing 1	SPRED1
211143_x_at	3.65	nuclear receptor subfamily 4, group A, member 1	NR4A1
235069_at	3.64	TatD DNase domain containing 3	TATDN3
1558846_at	3.63	pancreatic lipase-related protein 3	PNLIPRP3
205112_at	3.63	phospholipase C, epsilon 1	PLCE1
242680_at	3.63		
1554328_at	3.62	syntaxin binding protein 4	STXBP4
223131_s_at	3.61	tripartite motif-containing 8	TRIM8
244439_at	3.60	sprouty-related, EVH1 domain containing 1	SPRED1
1569054_at	3.60	solute carrier family 1 (glial high affinity glutamate transporter), member 3	SLC1A3
1556037_s_at	3.59	hedgehog interacting protein	HHIP
205937_at	3.59	cell growth regulator with EF-hand domain 1	CGREF1
232716_at	3.58		
230130_at	3.58	slit homolog 2 (Drosophila)	SLIT2
238729_x_at	3.57		
206960_at	3.57	G protein-coupled receptor 23	GPR23
236504_x_at	3.56	chromosome 6 open reading frame 52	C6orf52
227221_at	3.56		
233584_at	3.56	chromosome 20 open reading frame 62	C20orf62
202551_s_at	3.55	cysteine rich transmembrane BMP regulator 1 (chordin-like)	CRIM1
225188_at	3.55	Ras association (RalGDS/AF-6) and pleckstrin homology domains 1	RAPH1
1563115_at	3.54		
234669_x_at	3.54		
214038_at	3.51	chemokine (C-C motif) ligand 8	CCL8

221697_at	3.51	microtubule-associated protein 1 light chain 3 gamma	MAP1LC3C
1560225_at	3.51	cannabinoid receptor 1 (brain)	CNR1
207068_at	3.50	zinc finger protein 37 homolog (mouse)	ZFP37
1562458_at	3.49	ubiquitin-conjugating enzyme E2W (putative)	UBE2W
215139_at	3.49	Rho guanine nucleotide exchange factor (GEF) 10	ARHGEF10
207729_at	3.48	cadherin 9, type 2 (T1-cadherin)	CDH9
224202_at	3.48	suppressor of fused homolog (Drosophila)	SUFU
1559980_at	3.47	zinc finger protein 7	ZNF7
235144_at	3.47		
238752_at	3.47		
202391_at	3.47	brain abundant, membrane attached signal protein 1	BASP1
1555677_s_at	3.47	structural maintenance of chromosomes 1A	SMC1A
234767_at	3.47	microRNA 622	MIRN622
207052_at	3.45	hepatitis A virus cellular receptor 1	HAVCR1
1570593_at	3.45		
215592_at	3.45		
231050_at	3.44	HRAS-like suppressor family, member 5	HRASLS5
205514_at	3.43	zinc finger protein 415	ZNF415
237070_at	3.43	transient receptor potential cation channel, subfamily M, member 1	TRPM1
210304_at	3.43	phosphodiesterase 6B, cGMP-specific, rod, beta (congenital stationary night blindness 3, autosomal d	PDE6B
231138_at	3.43		
244257_at	3.42	transmembrane protein 104	TMEM104
228823_at	3.42		
223614_at	3.41	chromosome 8 open reading frame 57	C8orf57
216344_at	3.41	nephronophthisis 4	NPHP4
206239_s_at	3.41	serine peptidase inhibitor, Kazal type 1	SPINK1
1560609_at	3.41	crystallin, zeta (quinone reductase)-like 1	CRYZL1
212104_s_at	3.41	RNA binding motif protein 9	RBM9
235562_at	3.41		
216178_x_at	3.40	integrin, beta 1 (fibronectin receptor, beta polypeptide, antigen CD29 includes MDF2, MSK12)	ITGB1
241583_x_at	3.39	synaptotagmin I	SYT1
236423_at	3.38		
1555811_at	3.37	Rho GDP dissociation inhibitor (GDI) beta	ARHGDIB
1554867_a_at	3.37	proline rich 16	PRR16
237168_at	3.37		
237894_at	3.37	chromosome 3 open reading frame 22	C3orf22
204363_at	3.36	coagulation factor III (thromboplastin, tissue factor)	F3
237502_at	3.34	cardiolipin synthase 1	CRLS1
219912_s_at	3.34	ectonucleotide pyrophosphatase/phosphodiesterase 3	ENPP3
206417_at	3.34	cyclic nucleotide gated channel alpha 1	CNGA1
37117_at	3.34	PHD finger protein 21B	PHF21B
206835_at	3.33	statherin	STATH

244586_x_at	3.33	par-3 partitioning defective 3 homolog B (C. elegans)	PARD3B
202498_s_at	3.33	solute carrier family 2 (facilitated glucose transporter), member 3	SLC2A3
1560994_x_at	3.33		
206268_at	3.32	left-right determination factor 1	LEFTY1
205386_s_at	3.31	Mdm2, transformed 3T3 cell double minute 2, p53 binding protein (mouse)	MDM2
1557513_a_at	3.31		
229715_at	3.30		
230708_at	3.30	prickle homolog 1 (Drosophila)	PRICKLE1
239396_at	3.30		
244640_at	3.30		
211560_s_at	3.30	aminolevulinate, delta-, synthase 2 (sideroblastic/hypochromic anemia)	ALAS2
239657_x_at	3.30		
243655_x_at	3.30	SWI/SNF related, matrix associated, actin dependent regulator of chromatin, subfamily a, member 4	SMARCA4
243581_at	3.29		
233876_at	3.29		
1555929_s_at	3.28		
222337_at	3.28	oxysterol binding protein-like 9	OSBPL9
219841_at	3.28	activation-induced cytidine deaminase	AICDA
1561113_at	3.28		
220330_s_at	3.27	SAM domain, SH3 domain and nuclear localization signals 1	SAMSN1
1554997_a_at	3.27	prostaglandin-endoperoxide synthase 2 (prostaglandin G/H synthase and cyclooxygenase)	PTGS2
204959_at	3.26	myeloid cell nuclear differentiation antigen	MNDA
244334_at	3.26	translocation associated membrane protein 1-like 1	TRAM1L1
240288_at	3.26	potassium channel regulator	KCNRG
1554699_at	3.26	l(3)mbt-like 4 (Drosophila)	L3MBTL4
1562032_at	3.26		
242085_at	3.25	chromosome 2 open reading frame 18	C2orf18
230249_at	3.25	KH domain containing, RNA binding, signal transduction associated 3	KHDRBS3
232552_at	3.24	dishevelled associated activator of morphogenesis 1	DAAM1
221279_at	3.24	ganglioside-induced differentiation-associated protein 1	GDAP1
211812_s_at	3.24	beta-1,3-N-acetylgalactosaminyltransferase 1 (globoside blood group)	B3GALNT1
217513_at	3.23	chromosome 17 open reading frame 60	C17orf60
234532_at	3.23		
227197_at	3.23		
202668_at	3.22	ephrin-B2	EFNB2
214913_at	3.22	ADAM metallopeptidase with thrombospondin type 1 motif, 3	ADAMTS3
243198_at	3.21	testis expressed sequence 9	TEX9
229292_at	3.21	erythrocyte membrane protein band 4.1 like 5	EPB41L5

207228_at	3.21	protein kinase, cAMP-dependent, catalytic, gamma	PRKACG
234303_s_at	3.20	G protein-coupled receptor 85	GPR85
223962_at	3.20	tetratricopeptide repeat domain 29	TTC29
209802_at	3.20	pleckstrin homology-like domain, family A, member 2	PHLDA2
239455_at	3.20		
230506_at	3.20	chromosome 6 open reading frame 164	C6orf164
228368_at	3.19	Rho GTPase activating protein 20	ARHGAP20
218559_s_at	3.19	v-maf musculoaponeurotic fibrosarcoma oncogene homolog B (avian)	MAFB
212107_s_at	3.19	DEAH (Asp-Glu-Ala-His) box polypeptide 9	DHX9
205239_at	3.19	amphiregulin (schwannoma-derived growth factor)	AREG
219427_at	3.19	FAT tumor suppressor homolog 4 (Drosophila)	FAT4
230046_at	3.18		
225056_at	3.18	signal-induced proliferation-associated 1 like 2	SIPA1L2
210258_at	3.16	regulator of G-protein signalling 13	RGS13
204681_s_at	3.16	Rap guanine nucleotide exchange factor (GEF) 5	RAPGEF5
1563179_at	3.15		
235874_at	3.15	protease, serine, 35	PRSS35
242197_x_at	3.14	CD36 molecule (thrombospondin receptor)	CD36
1565602_at	3.13		
1566605_at	3.13	testis expressed sequence 9	TEX9
210193_at	3.13	myelin-associated oligodendrocyte basic protein	MOBP
213418_at	3.11	heat shock 70kDa protein 6 (HSP70B')	HSPA6
220659_s_at	3.10	chromosome 7 open reading frame 43	C7orf43
1562621_at	3.10		
210697_at	3.10	zinc finger protein 257	ZNF257
232665_x_at	3.10	SMAD specific E3 ubiquitin protein ligase 1	SMURF1
232574_at	3.09	xylosyltransferase I	XYLT1
232774_x_at	3.08	zinc finger protein 134#zinc finger protein 211#zinc finger protein 416#zinc finger protein 550#zinc finger protein 549#zinc finger protein interacting with K protein 1 homolog (mouse)#zinc finger protein 530	ZNF134#ZNF211#ZNF416#ZNF550#ZNF549#ZIK1#ZNF530
225981_at	3.08	chromosome 17 open reading frame 28	C17orf28
228285_at	3.08	tudor domain containing 9	TDRD9
1557733_a_at	3.08		
210755_at	3.08	hepatocyte growth factor (hepapoietin A; scatter factor)	HGF
206114_at	3.07	EPH receptor A4	EPHA4
202499_s_at	3.07	solute carrier family 2 (facilitated glucose transporter), member 3	SLC2A3
210368_at	3.06	protocadherin gamma subfamily A, 8	PCDHGA8
206291_at	3.06	neurotensin	NTS
231083_at	3.05	ets variant gene 5 (ets-related molecule)	ETV5
222958_s_at	3.05	DEP domain containing 1	DEPDC1
210765_at	3.05	CSE1 chromosome segregation 1-like (yeast)	CSE1L
117_at	3.05		
237268_at	3.04	Down syndrome cell adhesion molecule	DSCAM

204793_at	3.04	G protein-coupled receptor associated sorting protein 1	GPRASP1
223529_at	3.04	synaptotagmin IV	SYT4
209348_s_at	3.04	v-maf musculoaponeurotic fibrosarcoma oncogene homolog (avian)	MAF
243814_at	3.04	zinc finger, MYND-type containing 8	ZMYND8
217503_at	3.03		
1562625_at	3.03	FRY-like	FRYL
228824_s_at	3.03	leukotriene B4 12-hydroxydehydrogenase	LTB4DH
237732_at	3.03		
206935_at	3.03	protocadherin 8	PCDH8
1557326_at	3.02		
206907_at	3.02	tumor necrosis factor (ligand) superfamily, member 9	TNFSF9
214213_x_at	3.02	lamin A/C	LMNA
238932_at	3.01		
239394_at	3.01	solute carrier family 6 (neurotransmitter transporter, noradrenalin), member 2	SLC6A2
220667_at	3.01		
237663_at	3.01		
202988_s_at	3.01	regulator of G-protein signalling 1	RGS1
230307_at	3.00	solute carrier family 25 (mitochondrial oxodicarboxylate carrier), member 21	SLC25A21
221187_s_at	3.00	fuzzy homolog (Drosophila)	FUZ
239494_at	3.00		
239847_at	3.00		
1563460_at	2.99		
211538_s_at	2.99	heat shock 70kDa protein 2	HSPA2
240000_at	2.99		
1569061_at	2.99	IQ motif containing GTPase activating protein 3	IQGAP3
238353_at	2.99	RAS-like, family 11, member A	RASL11A
1562712_at	2.99		
238756_at	2.99		
216828_at	2.98	chromosome 20 open reading frame 80#null	C20orf80#null
210932_s_at	2.98	ring finger protein (C3H2C3 type) 6	RNF6
218987_at	2.97	activating transcription factor 7 interacting protein	ATF7IP
236656_s_at	2.97		
1554987_at	2.97	golgi autoantigen, golgin subfamily a, 3	GOLGA3
214745_at	2.96	phospholipase C, eta 1	PLCH1
205214_at	2.96	serine/threonine kinase 17b (apoptosis-inducing)	STK17B
221587_s_at	2.96	chromosome 19 open reading frame 24	C19orf24
244788_at	2.95		
206488_s_at	2.94	CD36 molecule (thrombospondin receptor)	CD36
204457_s_at	2.94	growth arrest-specific 1	GAS1
244176_at	2.94		
204298_s_at	2.92	lysyl oxidase	LOX
212233_at	2.92		
1552579_a_at	2.91	ADAM metallopeptidase domain 21	ADAM21
231658_x_at	2.91	ribosomal protein L36	RPL36
228235_at	2.90		

209030_s_at	2.90	cell adhesion molecule 1	CADM1
217081_at	2.89	gamma-aminobutyric acid (GABA) B receptor, 1#olfactory receptor, family 2, subfamily H, member 2#olfactory receptor, family 2, subfamily H, member 2#ubiquitin D#SMT3 suppressor of mif two 3 homolog 2 (S. cerevisiae) pseudogene	GABBR1#OR2H2#OR2H2#UBD#SUMO2P
205659_at	2.89	histone deacetylase 9	HDAC9
208401_s_at	2.89	glucagon-like peptide 1 receptor	GLP1R
205227_at	2.89	interleukin 1 receptor accessory protein	IL1RAP
239203_at	2.89		
209212_s_at	2.88	Kruppel-like factor 5 (intestinal)	KLF5
230781_at	2.88		
240359_at	2.88	chromosome 20 open reading frame 71	C20orf71
226187_at	2.88		
207881_at	2.88		
236859_at	2.87	runt-related transcription factor 2	RUNX2
217157_x_at	2.87		
232604_at	2.87	zinc finger protein 541	ZNF541
209433_s_at	2.87	phosphoribosyl pyrophosphate amidotransferase	PPAT
239456_at	2.87	glucocorticoid receptor DNA binding factor 1	GRLF1
242206_at	2.86		
210471_s_at	2.86	potassium voltage-gated channel, shaker-related subfamily, beta member 1	KCNAB1
1567856_x_at	2.85		
241184_x_at	2.85	zinc finger protein 407	ZNF407
1553661_a_at	2.85	HUS1 checkpoint homolog b (S. pombe)	HUS1B
1553674_at	2.85	leucine rich repeat containing 44	LRRC44
1560011_at	2.85	prostate stem cell antigen	PSCA
1557818_x_at	2.84		
1554310_a_at	2.83	eukaryotic translation initiation factor 4 gamma, 3	EIF4G3
230542_at	2.83	zinc finger protein 597	ZNF597
226884_at	2.83	leucine rich repeat neuronal 1	LRRN1
239669_at	2.83	histone cluster 1, H3d	HIST1H3D
1555120_at	2.82	CD96 molecule	CD96
229869_at	2.82		
204038_s_at	2.82	endothelial differentiation, lysophosphatidic acid G-protein-coupled receptor, 2	EDG2
217678_at	2.82	solute carrier family 7, (cationic amino acid transporter, y+ system) member 11	SLC7A11
238844_s_at	2.82	nephronophthisis 1 (juvenile)	NPHP1
1561431_at	2.82		
225664_at	2.81	collagen, type XII, alpha 1	COL12A1
235638_at	2.81	Ras association (RalGDS/AF-6) domain family 6	RASSF6
212188_at	2.81	potassium channel tetramerisation domain containing 12	KCTD12
204827_s_at	2.80	cyclin F	CCNF
243982_at	2.80	kelch-like 28 (Drosophila)	KLHL28
227034_at	2.80	ankyrin repeat domain 57	ANKRD57

204037_at	2.80	endothelial differentiation, lysophosphatidic acid G-protein-coupled receptor, 2	EDG2
210267_at	2.80	NIPA-like domain containing 3	NPAL3
1556827_at	2.80		
240179_at	2.79		
220166_at	2.79	cyclin M1	CNNM1
230863_at	2.79	low density lipoprotein-related protein 2	LRP2
201579_at	2.79	FAT tumor suppressor homolog 1 (Drosophila)	FAT
209555_s_at	2.79	CD36 molecule (thrombospondin receptor)	CD36
220369_at	2.79	SMEK homolog 1, suppressor of mek1 (Dictyostelium)	SMEK1
227842_at	2.79	RAB30, member RAS oncogene family	RAB30
204086_at	2.78	preferentially expressed antigen in melanoma	PRAME
205020_s_at	2.78	ADP-ribosylation factor-like 4A	ARL4A
221161_at	2.78	achaete-scute complex homolog 3 (Drosophila)	ASCL3
206085_s_at	2.77	cystathionase (cystathionine gamma-lyase)	CTH
213943_at	2.76	twist homolog 1 (acrocephalosyndactyly 3; Saethre-Chotzen syndrome) (Drosophila)	TWIST1
1554743_x_at	2.76	PMS1 postmeiotic segregation increased 1 (S. cerevisiae)	PMS1
1553666_at	2.75	coiled-coil domain containing 34	CCDC34
1569254_s_at	2.75	integrator complex subunit 4	INTS4
240982_at	2.74	cysteinyl-tRNA synthetase	CARS
238452_at	2.74	Fc receptor-like B	FCRLB
225028_at	2.74		
208300_at	2.74	protein tyrosine phosphatase, receptor type, H	PTPRH
1557217_a_at	2.74	Fanconi anemia, complementation group B	FANCB
201150_s_at	2.73	TIMP metallopeptidase inhibitor 3 (Sorsby fundus dystrophy, pseudoinflammatory)	TIMP3
236933_at	2.73		
224197_s_at	2.73	C1q and tumor necrosis factor related protein 1	C1QTNF1
1560853_x_at	2.73		
243272_at	2.73		
204748_at	2.73	prostaglandin-endoperoxide synthase 2 (prostaglandin G/H synthase and cyclooxygenase)	PTGS2
225476_at	2.72	HLA-B associated transcript 4	BAT4
228449_at	2.72	chromosome 22 open reading frame 27	C22orf27
227127_at	2.72	transmembrane protein 110	TMEM110
233481_at	2.72		
222771_s_at	2.71	myelin expression factor 2	MYEF2
207219_at	2.71	zinc finger protein 643	ZNF643
205488_at	2.71	granzyme A (granzyme 1, cytotoxic T-lymphocyte-associated serine esterase 3)	GZMA
232127_at	2.71	chloride channel 5 (nephrolithiasis 2, X-linked, Dent disease)	CLCN5
213325_at	2.71	poliovirus receptor-related 3	PVRL3
224095_at	2.70		
1556090_at	2.70		
205001_s_at	2.70	DEAD (Asp-Glu-Ala-Asp) box polypeptide 3, Y-linked	DDX3Y
1561143_at	2.69		

1555638_a_at	2.69	SAM domain, SH3 domain and nuclear localization signals 1	SAMSN1
228433_at	2.69		
224384_s_at	2.68	ring finger protein 17	RNF17
236429_at	2.68		
216834_at	2.67	regulator of G-protein signalling 1	RGS1
242123_at	2.67	progestin and adipoQ receptor family member VII	PAQR7
214639_s_at	2.67	homeobox A1	HOXA1
205018_s_at	2.66	muscleblind-like 2 (Drosophila)	MBNL2
1555623_at	2.66		
220473_s_at	2.66	zinc finger, CCHC domain containing 4	ZCCHC4
218959_at	2.66	homeobox C10	HOXC10
237491_at	2.66	myosin, heavy chain 10, non-muscle	MYH10
219270_at	2.66	ChaC, cation transport regulator homolog 1 (E. coli)	CHAC1
1562894_at	2.65		
219737_s_at	2.65	protocadherin 9	PCDH9
1555318_at	2.65	hypoxia inducible factor 3, alpha subunit	HIF3A
242843_at	2.65	brevican	BCAN
1559059_s_at	2.64	zinc finger protein 611	ZNF611
244044_at	2.64		
210362_x_at	2.64	promyelocytic leukemia	PML
243054_at	2.64	zinc finger, MYND domain containing 11	ZMYND11
1566267_at	2.63		
228636_at	2.63	basic helix-loop-helix domain containing, class B, 5	BHLHB5
242116_x_at	2.63	ankyrin repeat domain 17	ANKRD17
236948_x_at	2.63	splicing factor, arginine/serine-rich 11	SFRS11
224950_at	2.63	prostaglandin F2 receptor negative regulator	PTGFRN
208670_s_at	2.63	EP300 interacting inhibitor of differentiation 1	EID1
225354_s_at	2.63	SH3 domain binding glutamic acid-rich protein like 2	SH3BGRL2
232291_at	2.62	microRNA host gene (non-protein coding) 1	MIRH1
234478_at	2.61		
236650_at	2.61		
1554536_at	2.61	dihydropyrimidine dehydrogenase	DPYD
1555370_a_at	2.61	calmodulin binding transcription activator 1	CAMTA1
229360_at	2.60	suppressor of hairy wing homolog 2 (Drosophila)	SUHW2
209473_at	2.60		
223809_at	2.60	regulator of G-protein signalling 18	RGS18
240694_at	2.60		
219829_at	2.60	integrin beta 1 binding protein (melusin) 2	ITGB1BP2
203520_s_at	2.59	zinc finger protein 318	ZNF318
203439_s_at	2.59	stanniocalcin 2	STC2
206463_s_at	2.59	dehydrogenase/reductase (SDR family) member 2	DHRS2
243847_at	2.59		
216133_at	2.59	T cell receptor alpha locus	TRA@
201730_s_at	2.58	translocated promoter region (to activated MET oncogene)	TPR
214318_s_at	2.58	furry homolog (Drosophila)	FRY

201739_at	2.58	serum/glucocorticoid regulated kinase	SGK
229331_at	2.58	spermatogenesis associated 18 homolog (rat)	SPATA18
216468_s_at	2.58	zinc finger protein 90#zinc finger protein 682	ZNF90#ZNF682
1563512_at	2.57	nitric oxide synthase 1 (neuronal) adaptor protein	NOS1AP
228146_at	2.57	chromosome 17 open reading frame 51	C17orf51
220054_at	2.57	interleukin 23, alpha subunit p19	IL23A
1554333_at	2.57	DnaJ (Hsp40) homolog, subfamily A, member 4	DNAJA4
1552870_s_at	2.57	chromosome 1 open reading frame 125	C1orf125
227801_at	2.57	tripartite motif-containing 59	TRIM59
225065_x_at	2.56	chromosome 17 open reading frame 45	C17orf45
242247_at	2.56	methyltransferase 5 domain containing 1	METT5D1
238919_at	2.56		
217606_at	2.56		
1552732_at	2.55	actin-binding Rho activating protein	ABRA
238935_at	2.55	ribosomal protein S27-like	RPS27L
222162_s_at	2.55	ADAM metallopeptidase with thrombospondin type 1 motif, 1	ADAMTS1
238304_at	2.55	dipeptidyl-peptidase 6	DPP6
237278_x_at	2.54	ATPase, Ca++ transporting, type 2C, member 1	ATP2C1
235205_at	2.54		
1561086_at	2.54		
1562244_at	2.54		
240597_at	2.54		
217196_s_at	2.53	calmodulin regulated spectrin-associated protein 1-like 1	CAMSAP1L1
230846_at	2.53		
222608_s_at	2.53	anillin, actin binding protein	ANLN
227488_at	2.53		
210942_s_at	2.52	ST3 beta-galactoside alpha-2,3-sialyltransferase 6	ST3GAL6
233587_s_at	2.52	signal-induced proliferation-associated 1 like 2	SIPA1L2
1553220_at	2.52	amyotrophic lateral sclerosis 2 (juvenile) chromosome region, candidate 13	ALS2CR13
233514_x_at	2.52	testis expressed sequence 11	TEX11
223669_at	2.51	hemogen	HEMGN
1564463_at	2.51		
229591_at	2.51	low density lipoprotein receptor-related protein 5	LRP5
232517_s_at	2.51	eukaryotic translation elongation factor 1 alpha 2#potassium voltage-gated channel, KQT-like subfamily, member 2#potassium voltage-gated channel, KQT-like subfamily, member 2#PTK6 protein tyrosine kinase 6#src-related kinase lacking C-terminal regulatory tyrosine and N-terminal myristylation sites#chromosome 20 open reading frame 195#chromosome 20 open reading frame 149#null	EEF1A2#KCNQ2#KCNQ2#PTK6#SRMS#C20orf195#C20orf149#null
208425_s_at	2.51	tetratricopeptide repeat, ankyrin repeat and coiled-coil containing 2	TANC2
233397_at	2.50		

217743_s_at	2.50	transmembrane protein 30A	TMEM30A
213268_at	2.50	period homolog 3 (Drosophila)#vesicle-associated membrane protein 3 (cellubrevin)#urotensin 2#calmodulin binding transcription activator 1	PER3#VAMP3#UTS2#CAMTA1
202444_s_at	2.50	ER lipid raft associated 1	ERLIN1
209209_s_at	2.50	pleckstrin homology domain containing, family C (with FERM domain) member 1	PLEKHC1
207156_at	2.50	histone cluster 1, H2ag	HIST1H2AG
214320_x_at	2.49	cytochrome P450, family 2, subfamily A, polypeptide 7	CYP2A7
206145_at	2.49	Rh-associated glycoprotein	RHAG
223758_s_at	2.49	general transcription factor IIH, polypeptide 2, 44kDa	GTF2H2
234946_at	2.49	ectonucleoside triphosphate diphosphohydrolase 6 (putative function)	ENTPD6
244195_at	2.49	tubby like protein 4	TULP4
1569366_a_at	2.49	zinc finger protein 569	ZNF569
233592_at	2.49	annexin A2 pseudogene 2#T cell receptor beta variable 21/OR9-2#T cell receptor beta variable 24/OR9-2#T cell receptor beta variable 20/OR9-2#T cell receptor beta variable orphans on chromosome 9#T cell receptor beta variable 23/OR9-2#T cell receptor beta variable 22/OR9-2#null#suppressor of G2 allele of SKP1 pseudogene (S. cerevisiae)#ankyrin repeat domain 18B	ANXA2P2#TRBV21OR9-2#TRBV24OR9-2#TRBV20OR9-2#TRBVOR9@#TRBV23OR9-2#TRBV22OR9-2#null#SUGT1P#ANKRD18B
226769_at	2.48		
204774_at	2.48	ecotropic viral integration site 2A	EVI2A
205809_s_at	2.48	Wiskott-Aldrich syndrome-like	WASL
243861_at	2.48		
204697_s_at	2.47	chromogranin A (parathyroid secretory protein 1)	CHGA
1555225_at	2.47	chromosome 1 open reading frame 43	C1orf43
204107_at	2.47	nuclear transcription factor Y, alpha	NFYA
1569191_at	2.47		
1558014_s_at	2.47	male sterility domain containing 2	MLSTD2
217242_at	2.46	zinc finger protein 154	ZNF154
208261_x_at	2.46	interferon, alpha 10	IFNA10
223799_at	2.46	KIAA1826	KIAA1826
237755_s_at	2.46	WD repeat domain 16	WDR16
231885_at	2.46		
1553859_at	2.46	tryptophan hydroxylase 1 (tryptophan 5-monooxygenase)	TPH1
1564757_a_at	2.45		
226818_at	2.45		
225599_s_at	2.45		
220254_at	2.45	low density lipoprotein-related protein 12	LRP12
202779_s_at	2.45	ubiquitin-conjugating enzyme E2S	UBE2S
202340_x_at	2.45	nuclear receptor subfamily 4, group A, member 1	NR4A1
233827_s_at	2.44	suppressor of Ty 16 homolog (S. cerevisiae)	SUPT16H

216350_s_at	2.44	zinc finger protein 10	ZNF10
230493_at	2.44	transmembrane protein 46	TMEM46
219669_at	2.44	CD177 molecule	CD177
1562484_at	2.44		
242070_at	2.44		
217259_at	2.44		
231947_at	2.43	myc target 1	MYCT1
201333_s_at	2.43	Rho guanine nucleotide exchange factor (GEF) 12	ARHGEF12
238705_at	2.43		
1554242_a_at	2.43	coagulation factor C homolog, cochlin (Limulus polyphemus)	COCH
221994_at	2.43	PDZ and LIM domain 5	PDLIM5
238677_at	2.43	WD repeat domain 36	WDR36
228602_at	2.43	sarcoglycan, delta (35kDa dystrophin-associated glycoprotein)	SGCD
239002_at	2.43	asp (abnormal spindle) homolog, microcephaly associated (Drosophila)	ASPM
213825_at	2.42	oligodendrocyte lineage transcription factor 2	OLIG2
232980_at	2.41		
206105_at	2.41	AF4/FMR2 family, member 2	AFF2
234055_s_at	2.41	NTF2-like export factor 1#N-ethylmaleimide-sensitive factor attachment protein, beta#GDNF-inducible zinc finger protein 1#cystatin-like 1#cystatin 11	NXT1#NAPB#GZF1#CSTL1#CST11
231479_at	2.41	tetratricopeptide repeat domain 33	TTC33
1569183_a_at	2.41	choroideremia (Rab escort protein 1)	CHM
230149_at	2.40		
1559982_s_at	2.40	aldo-keto reductase family 1, member C-like 2	AKR1CL2
206648_at	2.40	zinc finger protein 571	ZNF571
213933_at	2.39	prostaglandin E receptor 3 (subtype EP3)	PTGER3
223195_s_at	2.39	sestrin 2	SESN2
204389_at	2.39	monoamine oxidase A	MAOA
1558292_s_at	2.39	phosphatidylinositol glycan anchor biosynthesis, class W	PIGW
223651_x_at	2.39	cell division cycle 23 homolog (S. cerevisiae)	CDC23
206411_s_at	2.38	v-abl Abelson murine leukemia viral oncogene homolog 2 (arg, Abelson-related gene)	ABL2
235846_at	2.38		
207813_s_at	2.38	ferredoxin reductase	FDXR
1553120_at	2.38	claspin homolog (Xenopus laevis)	CLSPN
205289_at	2.38	bone morphogenetic protein 2	BMP2
219890_at	2.38	C-type lectin domain family 5, member A	CLEC5A
210762_s_at	2.38	deleted in liver cancer 1	DLC1
204321_at	2.37	neogenin homolog 1 (chicken)	NEO1
242427_at	2.37	WW domain containing adaptor with coiled-coil	WAC
205229_s_at	2.37	coagulation factor C homolog, cochlin (Limulus polyphemus)	COCH
219585_at	2.37	coiled-coil domain containing 28B	CCDC28B
239959_x_at	2.37	phosphodiesterase 3B, cGMP-inhibited	PDE3B
236548_at	2.37		
217597_x_at	2.37	RAB40B, member RAS oncogene family	RAB40B
1556435_at	2.37		

216870_x_at	2.36	deleted in lymphocytic leukemia, 2	DLEU2
244887_at	2.36	regulator of G-protein signalling 13	RGS13
209795_at	2.36	CD69 molecule	CD69
204962_s_at	2.36	centromere protein A	CENPA
229374_at	2.36	EPH receptor A4	EPHA4
219201_s_at	2.36	twisted gastrulation homolog 1 (Drosophila)	TWSG1
219412_at	2.36	RAB38, member RAS oncogene family	RAB38
206765_at	2.36	potassium inwardly-rectifying channel, subfamily J, member 2	KCNJ2
1556558_s_at	2.36		
1556128_a_at	2.35	Ras protein-specific guanine nucleotide-releasing factor 2	RASGRF2
207528_s_at	2.35	solute carrier family 7, (cationic amino acid transporter, y+ system) member 11	SLC7A11
207808_s_at	2.35	protein S (alpha)	PROS1
222317_at	2.35	phosphodiesterase 3B, cGMP-inhibited	PDE3B
1562073_at	2.35		
228033_at	2.35	E2F transcription factor 7	E2F7
206314_at	2.34	zinc finger protein 167	ZNF167
216574_s_at	2.34		
1556404_a_at	2.34		
235296_at	2.34	eukaryotic translation initiation factor 5A2	EIF5A2
243149_at	2.34		
1553883_at	2.34	zinc finger protein 99	ZNF99
210233_at	2.34	interleukin 1 receptor accessory protein	IL1RAP
214190_x_at	2.34	golgi associated, gamma adaptin ear containing, ARF binding protein 2	GGA2
215629_s_at	2.34	deleted in lymphocytic leukemia 2-like	DLEU2L
234274_at	2.34		
1553269_at	2.34	zinc finger protein 718	ZNF718
235636_at	2.34		
207115_x_at	2.34	mbt domain containing 1	MBTD1
215615_x_at	2.34		
209211_at	2.33	Kruppel-like factor 5 (intestinal)	KLF5
218726_at	2.33		
1564468_at	2.33		
235121_at	2.33	zinc finger protein 542	ZNF542
205017_s_at	2.33	muscleblind-like 2 (Drosophila)	MBNL2
1553037_a_at	2.33	synapsin II	SYN2
1560258_a_at	2.32		
1566165_at	2.32		
209841_s_at	2.32	leucine rich repeat neuronal 3	LRRN3
211742_s_at	2.32	ecotropic viral integration site 2B	EVI2B
223196_s_at	2.32	sestrin 2	SESN2
228915_at	2.32	dachshund homolog 1 (Drosophila)	DACH1
228948_at	2.32	EPH receptor A4	EPHA4
236613_at	2.32	RNA binding motif protein 25	RBM25
204783_at	2.32	myeloid leukemia factor 1	MLF1
240357_at	2.32		
1556409_a_at	2.32		
237950_s_at	2.31		
221618_s_at	2.31	TAF9B RNA polymerase II, TATA box binding protein (TBP)-associated factor, 31kDa	TAF9B

207234_at	2.31	regulatory factor X, 3 (influences HLA class II expression)	RFX3
238803_at	2.31	HECT domain containing 2	HECTD2
1564970_at	2.31	SET domain, bifurcated 2	SETDB2
244021_at	2.31		
204042_at	2.31	WAS protein family, member 3	WASF3
208227_x_at	2.31	ADAM metallopeptidase domain 22	ADAM22
229996_s_at	2.31	polycomb group ring finger 5	PCGF5
1558592_at	2.30		
230450_at	2.30		
228063_s_at	2.30	nucleosome assembly protein 1-like 5	NAP1L5
207505_at	2.30	protein kinase, cGMP-dependent, type II	PRKG2
1555594_a_at	2.30	muscleblind-like (Drosophila)	MBNL1
1554309_at	2.30	eukaryotic translation initiation factor 4 gamma, 3	EIF4G3
240720_at	2.30		
203894_at	2.29	tubulin, gamma 2	TUBG2
1569791_at	2.29	serine/threonine kinase 4	STK4
204517_at	2.29	peptidylprolyl isomerase C (cyclophilin C)	PPIC
1560257_at	2.29		
203913_s_at	2.29	hydroxyprostaglandin dehydrogenase 15-(NAD)	HPGD
1558409_at	2.29		
209301_at	2.29	carbonic anhydrase II	CA2
1554999_at	2.29	RasGEF domain family, member 1B	RASGEF1B
1559523_at	2.28		
210867_at	2.28	CCR4-NOT transcription complex, subunit 4	CNOT4
215506_s_at	2.28	DIRAS family, GTP-binding RAS-like 3	DIRAS3
203184_at	2.28	fibrillin 2 (congenital contractural arachnodactyly)	FBN2
1569107_s_at	2.28	zinc finger protein 642	ZNF642
238160_at	2.28	acyl-CoA thioesterase 12	ACOT12
242218_at	2.27		
213355_at	2.27	ST3 beta-galactoside alpha-2,3-sialyltransferase 6	ST3GAL6
1555046_at	2.27	centromere protein I	CENPI
232247_at	2.27	zinc finger protein 502	ZNF502
220707_s_at	2.27	FAD-dependent oxidoreductase domain containing 2	FOXRED2
1554101_a_at	2.27	transmembrane and tetratricopeptide repeat containing 4	TMTC4
244861_at	2.27	zinc finger protein 527	ZNF527
215674_at	2.27		
210462_at	2.27	basic leucine zipper nuclear factor 1 (JEM-1)	BLZF1
228370_at	2.26	small nuclear ribonucleoprotein polypeptide N	SNRPN
222227_at	2.26	zinc finger protein 236	ZNF236
223593_at	2.26	aminoadipate aminotransferase	AADAT
211700_s_at	2.26	trophinin	TRO
220311_at	2.26	N-6 adenine-specific DNA methyltransferase 1 (putative)	N6AMT1
239169_at	2.26	RAD52 motif 1	RDM1
1560006_a_at	2.26		
222902_s_at	2.26	chromosome 1 open reading frame 176	C1orf176

204602_at	2.26	dickkopf homolog 1 (Xenopus laevis)	DKK1
223343_at	2.26	membrane-spanning 4-domains, subfamily A, member 7	MS4A7
227306_at	2.26		
204388_s_at	2.26	monoamine oxidase A	MAOA
206937_at	2.26	spectrin, alpha, erythrocytic 1 (elliptocytosis 2)	SPTA1
228717_at	2.26		
225578_at	2.25		
224048_at	2.25	ubiquitin specific peptidase 44	USP44
238779_at	2.25	DCP2 decapping enzyme homolog (S. cerevisiae)	DCP2
201167_x_at	2.25	Rho GDP dissociation inhibitor (GDI) alpha	ARHGDIA
218469_at	2.24	gremlin 1, cysteine knot superfamily, homolog (Xenopus laevis)	GREM1
238435_at	2.24		
223392_s_at	2.24	teashirt family zinc finger 3	TSHZ3
230005_at	2.24		
204529_s_at	2.24		
1555441_at	2.24	ubiquitin-activating enzyme E1-like 2	UBE1L2
209921_at	2.24	solute carrier family 7, (cationic amino acid transporter, y+ system) member 11	SLC7A11
214762_at	2.24	ATPase, H+ transporting, lysosomal 13kDa, V1 subunit G2	ATP6V1G2
206175_x_at	2.24	zinc finger protein 222	ZNF222
241060_x_at	2.24	tetraspanin 5	TSPAN5
206526_at	2.23	RIB43A domain with coiled-coils 2	RIBC2
1558622_a_at	2.23	zinc finger protein 548	ZNF548
200730_s_at	2.23	protein tyrosine phosphatase type IVA, member 1	PTP4A1
233071_at	2.23	karyopherin alpha 5 (importin alpha 6)#chromosome 6 open reading frame 113#radial spokehead-like 3	KPNA5#C6orf113 #RSHL3
218793_s_at	2.23	sex comb on midleg-like 1 (Drosophila)	SCML1
243242_at	2.23		
229260_at	2.23	chromosome 5 open reading frame 15	C5orf15
240417_at	2.22		
225687_at	2.22	family with sequence similarity 83, member D	FAM83D
1554966_a_at	2.22	filamin A interacting protein 1-like	FILIP1L
230387_at	2.22		
1564610_at	2.22		
237538_at	2.22	radical S-adenosyl methionine domain containing 2	RSAD2
210033_s_at	2.22	sperm associated antigen 6	SPAG6
239957_at	2.22	SET domain containing 5	SETD5
242384_at	2.22		
222809_x_at	2.21	chromosome 14 open reading frame 65	C14orf65
207495_at	2.21	RAB28, member RAS oncogene family	RAB28
238578_at	2.21	transmembrane protein 182	TMEM182
238962_at	2.21		
215597_x_at	2.21	MYST histone acetyltransferase (monocytic leukemia) 4	MYST4
206331_at	2.21	calcitonin receptor-like	CALCRL
206569_at	2.21	interleukin 24	IL24

207402_at	2.21	zinc finger protein 132	ZNF132
222385_x_at	2.21	Sec61 alpha 1 subunit (S. cerevisiae)	SEC61A1
205950_s_at	2.21	carbonic anhydrase I	CA1
225737_s_at	2.21	F-box protein 22	FBXO22
217127_at	2.20	cystathionase (cystathionine gamma-lyase)	CTH
225481_at	2.20	FERM domain containing 6	FRMD6
206207_at	2.20	Charcot-Leyden crystal protein	CLC
235085_at	2.20		
242931_at	2.20		
1554329_x_at	2.20	syntaxin binding protein 4	STXBP4
242330_at	2.20		
1555758_a_at	2.20	cyclin-dependent kinase inhibitor 3 (CDK2-associated dual specificity phosphatase)	CDKN3
217558_at	2.20	cytochrome P450, family 2, subfamily C, polypeptide 9	CYP2C9
243427_at	2.20		
206566_at	2.20	solute carrier family 7 (cationic amino acid transporter, y+ system), member 1	SLC7A1
230205_at	2.20	zinc finger protein 561	ZNF561
1567022_at	2.20	olfactory receptor, family 5, subfamily AK, member 4 pseudogene	OR5AK4P
215307_at	2.20	zinc finger protein 529	ZNF529
206079_at	2.20	choroideremia-like (Rab escort protein 2)	CHML
226649_at	2.19	pantothenate kinase 1	PANK1
242138_at	2.19	distal-less homeobox 1	DLX1
225600_at	2.19		
211756_at	2.19	parathyroid hormone-like hormone	PTHLH
1564691_at	2.19		
234970_at	2.19	membrane targeting (tandem) C2 domain containing 1	MTAC2D1
240952_at	2.19		
1559108_at	2.19		
231102_at	2.19	carnitine O-octanoyltransferase	CROT
208115_x_at	2.18	chromosome 10 open reading frame 137	C10orf137
242522_at	2.18		
1552740_at	2.18	chromosome 2 open reading frame 15	C2orf15
213283_s_at	2.18	sal-like 2 (Drosophila)	SALL2
204507_s_at	2.18	protein phosphatase 3 (formerly 2B), regulatory subunit B, alpha isoform	PPP3R1
233538_s_at	2.18	cytochrome b-245, beta polypeptide (chronic granulomatous disease)	CYBB
227099_s_at	2.18		
232630_at	2.18	APAF1 interacting protein	APIP
1565898_at	2.18	methyltransferase 5 domain containing 1	METT5D1
212127_at	2.18	Ran GTPase activating protein 1	RANGAP1
220253_s_at	2.18	low density lipoprotein-related protein 12	LRP12
237565_at	2.18	GRIP and coiled-coil domain containing 2	GCC2
222224_at	2.18	nascent-polypeptide-associated complex alpha polypeptide-like	NACAL
219148_at	2.18	PDZ binding kinase	PBK
227443_at	2.18	chromosome 9 open reading frame 150	C9orf150
239343_at	2.18		
205625_s_at	2.17	calbindin 1, 28kDa	CALB1

204749_at	2.17	nucleosome assembly protein 1-like 3	NAP1L3
223680_at	2.17	zinc finger protein 607	ZNF607
211548_s_at	2.17	hydroxyprostaglandin dehydrogenase 15-(NAD)	HPGD
214319_at	2.17	furry homolog (Drosophila)	FRY
1554621_at	2.17	diacylglycerol kinase, epsilon 64kDa	DGKE
210757_x_at	2.16	disabled homolog 2, mitogen-responsive phosphoprotein (Drosophila)	DAB2
205655_at	2.16	Mdm4, transformed 3T3 cell double minute 4, p53 binding protein (mouse)	MDM4
224802_at	2.16	Nedd4 family interacting protein 2	NDFIP2
244454_at	2.16	heterogeneous nuclear ribonucleoprotein H3 (2H9)	HNRPH3
235761_at	2.16		
1553192_at	2.15	zinc finger protein 441	ZNF441
231846_at	2.15	FAD-dependent oxidoreductase domain containing 2	FOXRED2
220236_at	2.15		
215446_s_at	2.15	lysyl oxidase	LOX
219717_at	2.15	chromosome 4 open reading frame 30	C4orf30
204525_at	2.15	PHD finger protein 14	PHF14
204071_s_at	2.15	topoisomerase I binding, arginine/serine-rich	TOPORS
205411_at	2.15	serine/threonine kinase 4	STK4
1556244_s_at	2.15		
238073_at	2.15	ELAV (embryonic lethal, abnormal vision, Drosophila)-like 4 (Hu antigen D)	ELAVL4
236635_at	2.14	zinc finger protein 667	ZNF667
1558111_at	2.14	muscleblind-like (Drosophila)	MBNL1
1557636_a_at	2.14		
244822_at	2.14	phosphoribosylglycinamide formyltransferase, phosphoribosylglycinamide synthetase, phosphoribosylami	GART
213067_at	2.13	myosin, heavy chain 10, non-muscle	MYH10
207597_at	2.13	ADAM metallopeptidase domain 18	ADAM18
236565_s_at	2.13	La ribonucleoprotein domain family, member 6	LARP6
218413_s_at	2.13	zinc finger protein 639	ZNF639
240447_at	2.13		
1553328_a_at	2.13	solute carrier family 18 (vesicular monoamine), member 2	SLC18A2
243312_at	2.13	zinc finger protein 588	ZNF588
238624_at	2.13		
214534_at	2.13	histone cluster 1, H1b	HIST1H1B
228144_at	2.13	zinc finger protein 300	ZNF300
241399_at	2.13	family with sequence similarity 19 (chemokine (C-C motif)-like), member A2	FAM19A2
241565_at	2.13	collagen, type IV, alpha 4	COL4A4
243326_at	2.13	AXIN1 up-regulated 1	AXUD1
238724_at	2.12	2,3-bisphosphoglycerate mutase	BPGM
220306_at	2.12	family with sequence similarity 46, member C	FAM46C
1558842_at	2.12		
214847_s_at	2.12	G-protein signalling modulator 3 (AGS3-like, C. elegans)	GPSM3
223005_s_at	2.12	chromosome 9 open reading frame 5	C9orf5

1553585_a_at	2.12	melanoma antigen family A, 5	MAGEA5
207722_s_at	2.12	BTB (POZ) domain containing 2	BTBD2
228654_at	2.12		
1564471_at	2.12		
235202_x_at	2.11		
223936_s_at	2.11	forkhead box P1	FOXP1
205352_at	2.11	serpin peptidase inhibitor, clade I (neuroserpin), member 1	SERPINI1
238488_at	2.11	importin 11	IPO11
229347_at	2.11		
242628_at	2.11	killer cell lectin-like receptor subfamily B, member 1	KLRB1
225270_at	2.11	neogenin homolog 1 (chicken)	NEO1
240141_at	2.11	PAP associated domain containing 4	PAPD4
230134_s_at	2.11		
203549_s_at	2.11	lipoprotein lipase	LPL
232454_at	2.11		
209737_at	2.11	membrane associated guanylate kinase, WW and PDZ domain containing 2	MAGI2
51158_at	2.11		
210821_x_at	2.11	centromere protein A	CENPA
232063_x_at	2.11	phenylalanine-tRNA synthetase-like, beta subunit	FARSLB
219875_s_at	2.10	chromosome 1 open reading frame 121	C1orf121
239481_at	2.10		
241888_at	2.10		
207768_at	2.10	early growth response 4	EGR4
209135_at	2.10	aspartate beta-hydroxylase	ASPH
1562921_at	2.10		
226733_at	2.10	6-phosphofructo-2-kinase/fructose-2,6-biphosphatase 2	PFKFB2
238623_at	2.10		
204135_at	2.10	filamin A interacting protein 1-like	FILIP1L
1556361_s_at	2.10	ankyrin repeat domain 13C	ANKRD13C
1560694_at	2.09	SFT2 domain containing 1	SFT2D1
243755_at	2.09	prolactin receptor	PRLR
209815_at	2.09	patched homolog 1 (Drosophila)	PTCH1
223249_at	2.09	claudin 12	CLDN12
211734_s_at	2.09	Fc fragment of IgE, high affinity I, receptor for; alpha polypeptide	FCER1A
205235_s_at	2.09	M-phase phosphoprotein 1	MPHOSPH1
210997_at	2.09	hepatocyte growth factor (hepapoietin A; scatter factor)	HGF
243222_at	2.09		
218458_at	2.09	germ cell-less homolog 1 (Drosophila)	GMCL1
1557701_s_at	2.09	polymerase (DNA directed), eta	POLH
1554015_a_at	2.09	chromodomain helicase DNA binding protein 2	CHD2
223413_s_at	2.08		
220688_s_at	2.08	mRNA turnover 4 homolog (S. cerevisiae)	MRTO4
212009_s_at	2.08	stress-induced-phosphoprotein 1 (Hsp70/Hsp90- organizing protein)	STIP1

216553_x_at	2.08	solute carrier family 23 (nucleobase transporters), member 2#chromosome 20 open reading frame 30	SLC23A2#C20orf 30
206862_at	2.08	zinc finger protein 254	ZNF254
219638_at	2.08	F-box protein 22	FBXO22
230574_at	2.08		
1563061_at	2.08		
204523_at	2.08	zinc finger protein 140	ZNF140
235780_at	2.07	protein kinase, cAMP-dependent, catalytic, beta	PRKACB
235044_at	2.07	cysteine/tyrosine-rich 1	CYYR1
225287_s_at	2.07	transmembrane protein 55B	TMEM55B
211549_s_at	2.07	hydroxyprostaglandin dehydrogenase 15-(NAD)	HPGD
1554445_at	2.07	zinc finger protein 85	ZNF85
240615_at	2.07		
227653_at	2.07	TRM5 tRNA methyltransferase 5 homolog (S. cerevisiae)	TRMT5
233914_s_at	2.07	SET binding factor 2	SBF2
227226_at	2.07	chromosome 6 open reading frame 117	C6orf117
215018_at	2.07	KIAA1731	KIAA1731
204612_at	2.07	protein kinase (cAMP-dependent, catalytic) inhibitor alpha	PKIA
210102_at	2.07	loss of heterozygosity, 11, chromosomal region 2, gene A	LOH11CR2A
230294_at	2.07		
224397_s_at	2.07	transmembrane and tetratricopeptide repeat containing 1	TMTC1
204211_x_at	2.07	eukaryotic translation initiation factor 2-alpha kinase 2	EIF2AK2
1562259_at	2.07	testis expressed sequence 9	TEX9
211810_s_at	2.07	galactosylceramidase	GALC
215563_s_at	2.07	macrophage stimulating, pseudogene 9	MSTP9
1554020_at	2.07	bicaudal D homolog 1 (Drosophila)	BICD1
241495_at	2.06	cyclin L1	CCNL1
204971_at	2.06	cystatin A (stefin A)	CSTA
240622_at	2.06	progestin and adipoQ receptor family member III	PAQR3
209615_s_at	2.06	p21/Cdc42/Rac1-activated kinase 1 (STE20 homolog, yeast)	PAK1
200756_x_at	2.06	calumenin	CALU
205570_at	2.06	phosphatidylinositol-4-phosphate 5-kinase, type II, alpha	PIP5K2A
201872_s_at	2.06	ATP-binding cassette, sub-family E (OABP), member 1	ABCE1
209287_s_at	2.06	CDC42 effector protein (Rho GTPase binding) 3	CDC42EP3
203967_at	2.06	cell division cycle 6 homolog (S. cerevisiae)	CDC6
213139_at	2.06	snail homolog 2 (Drosophila)	SNAI2
219403_s_at	2.06	heparanase	HPSE
229604_at	2.06	cytidine monophosphate-N-acetylneuraminic acid hydroxylase (CMP-N-acetylneuraminate monooxygenase)	CMAH

222093_s_at	2.06	zinc finger, HIT type 4	ZNHIT4
231791_at	2.06		
1557363_a_at	2.06	pleckstrin homology domain interacting protein	PHIP
216070_at	2.06		
206721_at	2.06	chromosome 1 open reading frame 114	C1orf114
1552908_at	2.06	chromosome 1 open reading frame 150	C1orf150
227741_at	2.05	protein tyrosine phosphatase-like (proline instead of catalytic arginine), member b	PTPLB
232369_at	2.05		
241912_at	2.05		
206914_at	2.05	cytotoxic and regulatory T cell molecule	CRTAM
216449_x_at	2.05	heat shock protein 90kDa beta (Grp94), member 1	HSP90B1
207334_s_at	2.05	transforming growth factor, beta receptor II (70/80kDa)	TGFBR2
206704_at	2.05	chloride channel 5 (nephrolithiasis 2, X-linked, Dent disease)	CLCN5
215635_at	2.05		
1560369_at	2.05	ankylosis, progressive homolog (mouse)	ANKH
238720_at	2.05	oligodendrocyte myelin glycoprotein	OMG
230626_at	2.05	tetraspanin 12	TSPAN12
241703_at	2.05		
203418_at	2.04	cyclin A2	CCNA2
222071_s_at	2.04	solute carrier organic anion transporter family, member 4C1	SLCO4C1
228708_at	2.04		
221078_s_at	2.04	KIAA1212	KIAA1212
1555068_at	2.04	WNK lysine deficient protein kinase 1	WNK1
226499_at	2.04		
226192_at	2.04		
213262_at	2.04	spastic ataxia of Charlevoix-Saguenay (sacsin)	SACS
205934_at	2.04	phospholipase C-like 1	PLCL1
204454_at	2.04	leucine zipper, down-regulated in cancer 1	LDOC1
240158_at	2.04		
214295_at	2.04		
221224_s_at	2.04	dephospho-CoA kinase domain containing	DCAKD
1553132_a_at	2.04	membrane targeting (tandem) C2 domain containing 1	MTAC2D1
221625_at	2.04	zinc finger protein 506	ZNF506
240259_at	2.04	fibronectin leucine rich transmembrane protein 2	FLRT2
1555943_at	2.04	phosphoglycerate mutase family member 5	PGAM5
230718_at	2.04	heat shock transcription factor family member 5	HSF5
1561361_at	2.03	zinc finger protein 660	ZNF660
207781_s_at	2.03	zinc finger protein 711	ZNF711
212945_s_at	2.03	MAX gene associated	MGA
1560020_at	2.03	DnaJ (Hsp40) homolog, subfamily C, member 13	DNAJC13
206552_s_at	2.03	tachykinin, precursor 1 (substance K, substance P, neurokinin 1, neurokinin 2, neuromedin L, neuroki	TAC1
228208_x_at	2.03	zinc finger protein 354C	ZNF354C

223735_at	2.03	ADP-ribosylation factor-like 6	ARL6
238490_at	2.03	KIAA2026	KIAA2026
230351_at	2.03		
232371_at	2.02		
206142_at	2.02	zinc finger protein 135	ZNF135
1554794_a_at	2.02	ubiquitin protein ligase E3C	UBE3C
242719_at	2.02		
211172_x_at	2.02	A kinase (PRKA) anchor protein 7	AKAP7
1561473_at	2.02		
206353_at	2.02	cytochrome c oxidase subunit VIa polypeptide 2	COX6A2
203811_s_at	2.02	DnaJ (Hsp40) homolog, subfamily B, member 4	DNAJB4
215708_s_at	2.02	primase, polypeptide 2A, 58kDa	PRIM2A
232333_at	2.02		
228863_at	2.02	protocadherin 17	PCDH17
225834_at	2.02		
239001_at	2.02	microsomal glutathione S-transferase 1	MGST1
1554606_at	2.02	coiled-coil domain containing 100	CCDC100
217240_at	2.02	signal-regulatory protein beta 1#signal-regulatory protein delta	SIRPB1#SIRPD
228766_at	2.02	CD36 molecule (thrombospondin receptor)	CD36
244370_at	2.02		
209822_s_at	2.01	very low density lipoprotein receptor	VLDLR
201873_s_at	2.01	ATP-binding cassette, sub-family E (OABP), member 1	ABCE1
206521_s_at	2.01	general transcription factor IIA, 1, 19/37kDa	GTF2A1
207030_s_at	2.01	cysteine and glycine-rich protein 2	CSRP2
1569669_at	2.01	forkhead box R2	FOXR2
234902_s_at	2.01	zinc finger protein 134#zinc finger protein 211#zinc finger protein 416#zinc finger protein 550#zinc finger protein 549#zinc finger protein interacting with K protein 1 homolog (mouse)#zinc finger protein 530	ZNF134#ZNF211#ZNF416#ZNF550#ZNF549#ZIK1#ZNF530
201197_at	2.01	adenosylmethionine decarboxylase 1	AMD1
242045_at	2.01		
201243_s_at	2.01	ATPase, Na+/K+ transporting, beta 1 polypeptide	ATP1B1
219544_at	2.01	chromosome 13 open reading frame 34	C13orf34
232138_at	2.01	muscleblind-like 2 (Drosophila)	MBNL2
208368_s_at	2.01	breast cancer 2, early onset	BRCA2
224063_at	2.01	neurolysin (metallopeptidase M3 family)	NLN
205046_at	2.01	centromere protein E, 312kDa	CENPE
217777_s_at	2.01	protein tyrosine phosphatase-like A domain containing 1	PTPLAD1
229533_x_at	2.00	zinc finger protein 680	ZNF680
207526_s_at	2.00	interleukin 1 receptor-like 1	IL1RL1
207057_at	2.00	solute carrier family 16, member 7 (monocarboxylic acid transporter 2)	SLC16A7
239778_x_at	0.50	calpain 7	CAPN7
242282_at	0.50	zinc finger protein, multitype 1	ZFPM1
239583_x_at	0.50	placenta-specific 7	PLAC7
212468_at	0.50	sperm associated antigen 9	SPAG9

225339_at	0.50		
229776_at	0.50	solute carrier organic anion transporter family, member 3A1	SLCO3A1
1564028_s_at	0.50		
214922_at	0.50	zinc finger protein 484	ZNF484
209039_x_at	0.50	EH-domain containing 1	EHD1
228891_at	0.50	chromosome 9 open reading frame 164	C9orf164
227344_at	0.50	IKAROS family zinc finger 1 (Ikaros)	IKZF1
223963_s_at	0.50	insulin-like growth factor 2 mRNA binding protein 2	IGF2BP2
212547_at	0.50		
1555486_a_at	0.50		
205241_at	0.50	SCO cytochrome oxidase deficient homolog 2 (yeast)	SCO2
203825_at	0.50	bromodomain containing 3	BRD3
244082_at	0.50		
201999_s_at	0.50	dynein, light chain, Tctex-type 1	DYNLT1
226771_at	0.50	ATPase, Class I, type 8B, member 2	ATP8B2
228139_at	0.50	receptor-interacting serine-threonine kinase 3	RIPK3
217628_at	0.50	chloride intracellular channel 5	CLIC5
1555034_at	0.50	clarin 1	CLRN1
213572_s_at	0.50	serpin peptidase inhibitor, clade B (ovalbumin), member 1	SERPINB1
226158_at	0.50	kelch-like 24 (Drosophila)	KLHL24
215188_at	0.50	serine/threonine kinase 24 (STE20 homolog, yeast)	STK24
203505_at	0.50	ATP-binding cassette, sub-family A (ABC1), member 1	ABCA1
233353_at	0.50		
201324_at	0.50	epithelial membrane protein 1	EMP1
223983_s_at	0.50	chromosome 19 open reading frame 12	C19orf12
237208_at	0.50	WD repeat domain 61	WDR61
242364_x_at	0.50		
1566515_at	0.49	CWF19-like 2, cell cycle control (S. pombe)	CWF19L2
242986_at	0.49		
214594_x_at	0.49	ATPase, Class I, type 8B, member 1	ATP8B1
201455_s_at	0.49	aminopeptidase puromycin sensitive	NPEPPS
214657_s_at	0.49		
238896_at	0.49		
1563076_x_at	0.49		
214964_at	0.49		
201448_at	0.49	TIA1 cytotoxic granule-associated RNA binding protein	TIA1
218501_at	0.49	Rho guanine nucleotide exchange factor (GEF) 3	ARHGEF3
219429_at	0.49	fatty acid 2-hydroxylase	FA2H
214918_at	0.49	heterogeneous nuclear ribonucleoprotein M	HNRPM
207536_s_at	0.49	tumor necrosis factor receptor superfamily, member 9	TNFRSF9
204420_at	0.49	FOS-like antigen 1	FOSL1
204413_at	0.49	TNF receptor-associated factor 2	TRAF2
232037_at	0.49	putative neuronal cell adhesion molecule	PUNC
242897_at	0.49		

223407_at	0.49	chromosome 16 open reading frame 48	C16orf48
233730_at	0.49	KIAA1411	KIAA1411
1569668_at	0.49		
208981_at	0.49	platelet/endothelial cell adhesion molecule (CD31 antigen)	PECAM1
211744_s_at	0.49	CD58 molecule	CD58
1562799_at	0.49		
229958_at	0.49	chromosome 8 open reading frame 61	C8orf61
213348_at	0.49	cyclin-dependent kinase inhibitor 1C (p57, Kip2)	CDKN1C
1562107_at	0.49		
226961_at	0.49	proline rich 15	PRR15
238649_at	0.49	phosphatidylinositol transfer protein, cytoplasmic 1	PITPNC1
1568803_at	0.49		
224761_at	0.49	guanine nucleotide binding protein (G protein), alpha 13	GNA13
231876_at	0.49	tripartite motif-containing 56	TRIM56
244703_x_at	0.49	importin 9	IPO9
222073_at	0.49	collagen, type IV, alpha 3 (Goodpasture antigen)	COL4A3
218862_at	0.49	ankyrin repeat and SOCS box-containing 13	ASB13
225968_at	0.49	prickle homolog 2 (Drosophila)	PRICKLE2
237829_at	0.49		
226796_at	0.49		
1557570_a_at	0.49		
238189_at	0.49	SIN3 homolog A, transcription regulator (yeast)	SIN3A
205379_at	0.49	carbonyl reductase 3	CBR3
242797_x_at	0.49	zinc finger RNA binding protein	ZFR
242768_at	0.49		
226372_at	0.49	carbohydrate (chondroitin 4) sulfotransferase 11	CHST11
202409_at	0.48	insulin-like growth factor 2 (somatomedin A)	IGF2
1562935_at	0.48		
235421_at	0.48		
227036_at	0.48		
227000_at	0.48	chromosome 7 open reading frame 41	C7orf41
217738_at	0.48	pre-B-cell colony enhancing factor 1	PBEF1
1554710_at	0.48	potassium large conductance calcium-activated channel, subfamily M, beta member 1	KCNMB1
212445_s_at	0.48	neural precursor cell expressed, developmentally down-regulated 4-like	NEDD4L
212623_at	0.48	transmembrane protein 41B	TMEM41B
224983_at	0.48	scavenger receptor class B, member 2	SCARB2
1561042_at	0.48	integrin, beta 1 (fibronectin receptor, beta polypeptide, antigen CD29 includes MDF2, MSK12)	ITGB1
240766_at	0.48	interleukin 23, alpha subunit p19	IL23A
243216_x_at	0.48	ubiquitin specific peptidase 40	USP40
212457_at	0.48	transcription factor binding to IGHM enhancer 3	TFE3
204066_s_at	0.48	centaurin, gamma 2	CENTG2

238130_at	0.48	nuclear factor of activated T-cells, cytoplasmic, calcineurin-dependent 2 interacting protein	NFATC2IP
204639_at	0.48	adenosine deaminase	ADA
1560118_at	0.48		
236892_s_at	0.48		
203728_at	0.48	BCL2-antagonist/killer 1	BAK1
242558_at	0.48		
230152_at	0.48	WD repeat domain 52	WDR52
207407_x_at	0.48	cytochrome P450, family 4, subfamily A, polypeptide 11	CYP4A11
217335_at	0.48		
232363_at	0.48		
242657_at	0.48	insulin-like growth factor binding protein 4	IGFBP4
235735_at	0.48		
209354_at	0.48	tumor necrosis factor receptor superfamily, member 14 (herpesvirus entry mediator)	TNFRSF14
232752_at	0.48		
239642_at	0.48		
219340_s_at	0.48	ceroid-lipofuscinosis, neuronal 8 (epilepsy, progressive with mental retardation)	CLN8
226504_at	0.48	family with sequence similarity 109, member B	FAM109B
224215_s_at	0.48	delta-like 1 (Drosophila)	DLL1
220144_s_at	0.48	ankyrin repeat domain 5	ANKRD5
1566243_at	0.48		
237794_at	0.48		
239453_at	0.48	formin binding protein 1	FNBP1
212912_at	0.48	ribosomal protein S6 kinase, 90kDa, polypeptide 2	RPS6KA2
207481_at	0.48		
212753_at	0.48	polycomb group ring finger 3	PCGF3
221773_at	0.48	ELK3, ETS-domain protein (SRF accessory protein 2)	ELK3
226855_at	0.48		
211864_s_at	0.48	fer-1-like 3, myoferlin (C. elegans)	FER1L3
202171_at	0.48	vascular endothelial zinc finger 1	VEZF1
225615_at	0.48		
202880_s_at	0.48	pleckstrin homology, Sec7 and coiled-coil domains 1(cytohesin 1)	PSCD1
212285_s_at	0.48	agrin	AGRIN
205781_at	0.48	chromosome 16 open reading frame 7	C16orf7
240906_at	0.48	mitochondrial ribosomal protein S36	MRPS36
1559814_at	0.48		
207987_s_at	0.48	gonadotropin-releasing hormone 1 (luteinizing-releasing hormone)	GNRH1
1568592_at	0.48	tripartite motif-containing 69	TRIM69
204262_s_at	0.48	presenilin 2 (Alzheimer disease 4)	PSEN2
1557575_at	0.48		
233952_s_at	0.48	zinc finger protein 295	ZNF295
241505_at	0.48		
226104_at	0.48		
219209_at	0.48	interferon induced with helicase C domain 1	IFIH1
209360_s_at	0.47	runt-related transcription factor 1 (acute myeloid leukemia 1; aml1 oncogene)	RUNX1

1557036_at	0.47	zinc finger and BTB domain containing 1	ZBTB1
212117_at	0.47	ras homolog gene family, member Q	RHOQ
201850_at	0.47	capping protein (actin filament), gelsolin-like	CAPG
225306_s_at	0.47	solute carrier family 25, member 29	SLC25A29
226474_at	0.47	NLR family, CARD domain containing 5	NLRC5
209829_at	0.47	chromosome 6 open reading frame 32	C6orf32
209640_at	0.47	promyelocytic leukemia	PML
231906_at	0.47	homeobox D8	HOXD8
208466_at	0.47	RAB3D, member RAS oncogene family	RAB3D
227211_at	0.47	PHD finger protein 19	PHF19
209488_s_at	0.47	RNA binding protein with multiple splicing	RBPMS
213725_x_at	0.47	xylosyltransferase I	XYLT1
225216_at	0.47	chromosome X open reading frame 39	CXorf39
235497_at	0.47		
1558748_at	0.47		
204070_at	0.47	retinoic acid receptor responder (tazarotene induced) 3	RARRES3
1560625_s_at	0.47		
230120_s_at	0.47		
232151_at	0.47		
210486_at	0.47	ankyrin repeat and MYND domain containing 1	ANKMY1
212948_at	0.47	calmodulin binding transcription activator 2	CAMTA2
209236_at	0.47	solute carrier family 23 (nucleobase transporters), member 2	SLC23A2
229250_at	0.47	two pore segment channel 2	TPCN2
233118_at	0.47		
241742_at	0.47	PML-RARA regulated adaptor molecule 1	PRAM1
1555014_x_at	0.47		
1563088_a_at	0.47		
240728_at	0.47	phospholipase C, beta 4	PLCB4
1563611_at	0.47		
218421_at	0.47	ceramide kinase	CERK
218284_at	0.47	SMAD family member 3	SMAD3
215131_at	0.47	IQ motif containing K#null	IQCK#null
219451_at	0.47	methionine sulfoxide reductase B2	MSRB2
213629_x_at	0.47	metallothionein 1J (pseudogene)	MT1JP
204594_s_at	0.47	Smith-Magenis syndrome chromosome region, candidate 7-like	SMCR7L
214104_at	0.47	G protein-coupled receptor 161	GPR161
230980_x_at	0.47		
230722_at	0.47	basonuclin 2	BNC2
221556_at	0.47	CDC14 cell division cycle 14 homolog B (S. cerevisiae)	CDC14B
217456_x_at	0.47	major histocompatibility complex, class I, E	HLA-E
1557478_at	0.47		
209193_at	0.47	pim-1 oncogene	PIM1
217767_at	0.47	complement component 3	C3
225663_at	0.47	acyl-Coenzyme A binding domain containing 5	ACBD5
1554835_a_at	0.47	UDP-GlcNAc:betaGal beta-1,3-N-acetylglucosaminyltransferase 5	B3GNT5
202800_at	0.47	solute carrier family 1 (glial high affinity glutamate transporter), member 3	SLC1A3
1564331_at	0.47		

215232_at	0.47		
242082_at	0.47	methylmalonic aciduria (cobalamin deficiency) cblB type	MMAB
206148_at	0.47	interleukin 3 receptor, alpha (low affinity)	IL3RA
207765_s_at	0.47	KIAA1539	KIAA1539
1559287_at	0.47		
225980_at	0.47	chromosome 14 open reading frame 43	C14orf43
237337_at	0.47		
36564_at	0.47	IBR domain containing 3	IBRDC3
232466_at	0.47	cullin 4A	CUL4A
223184_s_at	0.47	1-acylglycerol-3-phosphate O-acyltransferase 3	AGPAT3
227697_at	0.47	suppressor of cytokine signaling 3	SOCS3
211250_s_at	0.47	SH3-domain binding protein 2	SH3BP2
235276_at	0.47	epithelial stromal interaction 1 (breast)	EPSTI1
208820_at	0.47	PTK2 protein tyrosine kinase 2	PTK2
243161_x_at	0.47	zinc finger protein 42 homolog (mouse)	ZFP42
207582_at	0.47	protein (peptidylprolyl cis/trans isomerase) NIMA-interacting 1-like	PIN1L
1564444_at	0.47		
239593_at	0.47		
235108_at	0.47		
238084_at	0.47	polycomb group ring finger 3	PCGF3
1570505_at	0.47	ATP-binding cassette, sub-family B (MDR/TAP), member 4	ABCB4
235626_at	0.47	calcium/calmodulin-dependent protein kinase ID	CAMK1D
211433_x_at	0.47	KIAA1539	KIAA1539
242229_at	0.47		
215604_x_at	0.47		
211731_x_at	0.46	synovial sarcoma, X breakpoint 3	SSX3
211842_s_at	0.46	solute carrier family 24 (sodium/potassium/calcium exchanger), member 1	SLC24A1
221816_s_at	0.46	PHD finger protein 11	PHF11
217164_at	0.46		
232488_at	0.46	alanine-glyoxylate aminotransferase 2-like 2	AGXT2L2
224560_at	0.46	TIMP metallopeptidase inhibitor 2	TIMP2
39582_at	0.46	cylindromatosis (turban tumor syndrome)	CYLD
222838_at	0.46	CD48 molecule#lymphocyte antigen 9#signaling lymphocytic activation molecule family member 1#SLAM family member 7	CD48#LY9#SLAMF1#SLAMF7
225935_at	0.46		
216745_x_at	0.46		
242454_at	0.46		
201126_s_at	0.46	mannosyl (alpha-1,3-)-glycoprotein beta-1,2-N-acetylglucosaminyltransferase	MGAT1
213976_at	0.46	CDKN1A interacting zinc finger protein 1	CIZ1
203635_at	0.46	Down syndrome critical region gene 3	DSCR3
205668_at	0.46	lymphocyte antigen 75	LY75
217487_x_at	0.46	folate hydrolase (prostate-specific membrane antigen) 1	FOLH1

210017_at	0.46	mucosa associated lymphoid tissue lymphoma translocation gene 1	MALT1
211883_x_at	0.46	carcinoembryonic antigen-related cell adhesion molecule 1 (biliary glycoprotein)	CEACAM1
224833_at	0.46	v-ets erythroblastosis virus E26 oncogene homolog 1 (avian)	ETS1
210201_x_at	0.46	bridging integrator 1	BIN1
223615_at	0.46	ABI gene family, member 3	ABI3
219401_at	0.46	xylosyltransferase II	XYLT2
217518_at	0.46	fer-1-like 3, myoferlin (C. elegans)	FER1L3
1560117_at	0.46	abhydrolase domain containing 1	ABHD1
244608_at	0.46		
220547_s_at	0.46	family with sequence similarity 35, member A	FAM35A
228450_at	0.46	pleckstrin homology domain containing, family A member 7	PLEKHA7
224336_s_at	0.46	dual specificity phosphatase 16	DUSP16
203827_at	0.46	WD repeat domain, phosphoinositide interacting 1	WIPI1
1560184_at	0.46	transmembrane and coiled-coil domains 5	TMCO5
219454_at	0.46	EGF-like-domain, multiple 6	EGFL6
244204_at	0.46	proline rich 3	PRR3
1570566_at	0.46		
241738_at	0.46		
207629_s_at	0.46	rho/rac guanine nucleotide exchange factor (GEF) 2	ARHGEF2
219352_at	0.46	hect domain and RLD 6	HERC6
226516_at	0.46	chromosome 19 open reading frame 28	C19orf28
227855_at	0.46	zinc finger protein 219	ZNF219
226326_at	0.46	polycomb group ring finger 5	PCGF5
218764_at	0.46	protein kinase C, eta	PRKCH
239842_x_at	0.46		
215122_at	0.46	T-box 6	TBX6
227695_at	0.46	glycine-N-acyltransferase-like 1	GLYATL1
1562271_x_at	0.46	Rho guanine nucleotide exchange factor (GEF) 7	ARHGEF7
236455_at	0.46		
208902_s_at	0.46	ribosomal protein S28	RPS28
222303_at	0.46		
203391_at	0.46	FK506 binding protein 2, 13kDa	FKBP2
1556942_at	0.46		
209764_at	0.46	activating transcription factor 4 (tax-responsive enhancer element B67)#mannosyl (beta-1,4-)-glycoprotein beta-1,4-N-acetylglucosaminyltransferase#calcium channel, voltage-dependent, alpha 1I subunit#Smith-Magenis syndrome chromosome region, candidate 7-like#Smith-Magenis syndrome chromosome region, candidate 7-like#ribosomal protein S19 binding protein 1	ATF4#MGAT3#CACNA1I#SMCR7L#SMCR7L#RPS19BP1
213834_at	0.46	IQ motif and Sec7 domain 3	IQSEC3
225140_at	0.46	Kruppel-like factor 3 (basic)	KLF3

208438_s_at	0.46	Gardner-Rasheed feline sarcoma viral (v-fgr) oncogene homolog	FGR
1568706_s_at	0.46	advillin	AVIL
240723_at	0.46	Enah/Vasp-like	EVL
244868_at	0.46	zinc fingers and homeoboxes 2	ZHX2
228054_at	0.46	transmembrane protein 44	TMEM44
1561450_at	0.46		
217739_s_at	0.46	pre-B-cell colony enhancing factor 1	PBEF1
205569_at	0.45	lysosomal-associated membrane protein 3	LAMP3
232161_x_at	0.45		
233606_at	0.45		
241616_at	0.45		
203754_s_at	0.45	BRF1 homolog, subunit of RNA polymerase III transcription initiation factor IIIB (S. cerevisiae)	BRF1
239242_at	0.45		
229390_at	0.45		
204684_at	0.45	neuronal pentraxin I	NPTX1
233702_x_at	0.45		
224405_at	0.45	Fc receptor-like 5	FCRL5
229660_at	0.45	chromosome 16 open reading frame 55	C16orf55
219150_s_at	0.45	centaurin, alpha 1	CENTA1
205842_s_at	0.45	Janus kinase 2 (a protein tyrosine kinase)	JAK2
208933_s_at	0.45		
1567009_at	0.45		
237083_at	0.45		
236708_at	0.45	capping protein (actin filament) muscle Z-line, beta	CAPZB
222061_at	0.45	CD58 molecule	CD58
223179_at	0.45	yippee-like 3 (Drosophila)	YPEL3
204923_at	0.45	X-prolyl aminopeptidase (aminopeptidase P) 2, membrane-bound#chromosome X open reading frame 9	XPNPEP2#CXorf9
211434_s_at	0.45	chemokine (C-C motif) receptor-like 2	CCRL2
228454_at	0.45	ligand dependent nuclear receptor corepressor	LCOR
229253_at	0.45	thioesterase superfamily member 4	THEM4
229222_at	0.45		
233438_at	0.45		
238797_at	0.45	tripartite motif-containing 11	TRIM11
216286_at	0.45	UDP-Gal:betaGlcNAc beta 1,4-galactosyltransferase, polypeptide 6	B4GALT6
210001_s_at	0.45	suppressor of cytokine signaling 1	SOCS1
241633_x_at	0.45		
1555167_s_at	0.45	pre-B-cell colony enhancing factor 1	PBEF1
1568638_a_at	0.45	indoleamine-pyrrole 2,3 dioxygenase-like 1	INDOL1
210671_x_at	0.45	mitogen-activated protein kinase 8	MAPK8
222482_at	0.45		
213895_at	0.45	epithelial membrane protein 1	EMP1
229391_s_at	0.45		
216583_x_at	0.45	homeobox A1#homeobox A2#homeobox A2#homeobox A3#null#null	HOXA1#HOXA2#HOXA2#HOXA3#null#null
1558830_at	0.45		
240482_at	0.45	histone deacetylase 3	HDAC3

217991_x_at	0.45	single stranded DNA binding protein 3	SSBP3
234297_at	0.45	programmed cell death 6	PDCD6
227513_s_at	0.45	leucine rich repeat (in FLII) interacting protein 1	LRRFIP1
226441_at	0.45		
225286_at	0.45	arylsulfatase D	ARSD
201368_at	0.45	zinc finger protein 36, C3H type-like 2	ZFP36L2
213038_at	0.45	IBR domain containing 3	IBRDC3
1570210_x_at	0.45	SAPS domain family, member 2	SAPS2
215858_at	0.45		
217371_s_at	0.45	interleukin 15	IL15
236630_at	0.45	aquaporin 2 (collecting duct)	AQP2
225605_at	0.45	tumor protein p53 inducible protein 13	TP53I13
224912_at	0.45	tetratricopeptide repeat domain 7A	TTC7A
226034_at	0.45		
217679_x_at	0.45		
239100_x_at	0.45		
227113_at	0.45	alcohol dehydrogenase, iron containing, 1	ADHFE1
236750_at	0.45		
213966_at	0.45	high-mobility group 20B	HMG20B
235889_at	0.45		
223950_s_at	0.45	FLYWCH-type zinc finger 1	FLYWCH1
1563524_a_at	0.45	chromosome 14 open reading frame 85	C14orf85
230629_s_at	0.45	E1A binding protein p400	EP400
231697_s_at	0.45	transmembrane protein 49	TMEM49
1557331_at	0.45	polymerase (RNA) I polypeptide B, 128kDa	POLR1B
233330_s_at	0.45		
237516_at	0.45		
203633_at	0.45	carnitine palmitoyltransferase 1A (liver)	CPT1A
230741_at	0.45		
219550_at	0.45	roundabout, axon guidance receptor, homolog 3 (Drosophila)	ROBO3
227346_at	0.45	IKAROS family zinc finger 1 (Ikaros)	IKZF1
244327_at	0.45		
222861_x_at	0.45	F-box protein 2	FBXO2
1566607_at	0.45		
1561130_at	0.45	chromosome 12 open reading frame 51	C12orf51
240561_at	0.45		
215974_at	0.45		
203149_at	0.45	poliovirus receptor-related 2 (herpesvirus entry mediator B)	PVRL2
242794_at	0.45	mastermind-like 3 (Drosophila)	MAML3
243689_s_at	0.44		
204549_at	0.44	inhibitor of kappa light polypeptide gene enhancer in B-cells, kinase epsilon	IKBKE
230333_at	0.44		
219999_at	0.44	mannosidase, alpha, class 2A, member 2	MAN2A2
1557293_at	0.44		
228812_at	0.44		
225628_s_at	0.44	myeloid/lymphoid or mixed-lineage leukemia (trithorax homolog, Drosophila); translocated to, 6	MLLT6
212355_at	0.44	KIAA0323	KIAA0323

225597_at	0.44	solute carrier family 45, member 4	SLC45A4
243613_at	0.44		
224772_at	0.44	neuron navigator 1	NAV1
240166_x_at	0.44	RNA (guanine-9-) methyltransferase domain containing 3	RG9MTD3
232861_at	0.44		
232382_s_at	0.44	protein-L-isoaspartate (D-aspartate) O-methyltransferase domain containing 1	PCMTD1
229072_at	0.44		
235285_at	0.44		
229202_at	0.44		
226016_at	0.44	CD47 molecule	CD47
1554594_at	0.44		
229810_at	0.44		
233600_at	0.44		
210544_s_at	0.44	aldehyde dehydrogenase 3 family, member A2	ALDH3A2
228728_at	0.44		
241421_at	0.44		
231049_at	0.44	LIM domain only 2 (rhombotin-like 1)	LMO2
240744_at	0.44	carboxypeptidase A5	CPA5
234624_at	0.44		
238653_at	0.44		
229396_at	0.44	ovo-like 1(Drosophila)	OVOL1
211667_x_at	0.44		
1558340_at	0.44	DIX domain containing 1	DIXDC1
216123_x_at	0.44		
227049_at	0.44		
229504_at	0.44		
1569345_at	0.44		
217851_s_at	0.44	slowmo homolog 2 (Drosophila)	SLMO2
234332_at	0.44	negative regulator of ubiquitin-like proteins 1	NUB1
216971_s_at	0.44	plectin 1, intermediate filament binding protein 500kDa	PLEC1
241879_at	0.44		
1555781_at	0.44	PQ loop repeat containing 2	PQLC2
1558342_x_at	0.44	DIX domain containing 1	DIXDC1
227341_at	0.44	chromosome 10 open reading frame 30	C10orf30
1569898_a_at	0.44		
217643_x_at	0.44		
222860_s_at	0.44	platelet derived growth factor D	PDGFD
236586_at	0.44		
236476_at	0.44		
202084_s_at	0.44	SEC14-like 1 (S. cerevisiae)	SEC14L1
1566772_at	0.44		
224962_at	0.44		
221205_at	0.44		
215704_at	0.44	filaggrin#null	FLG#null
234594_at	0.44	chromosome 14 open reading frame 85	C14orf85
1556590_s_at	0.44		
205469_s_at	0.44	interferon regulatory factor 5	IRF5
1563519_at	0.44		
1564276_at	0.43		

226075_at	0.43	splA/ryanodine receptor domain and SOCS box containing 1	SPSB1
200704_at	0.43	lipopolysaccharide-induced TNF factor	LITAF
226551_at	0.43	receptor (TNFRSF)-interacting serine-threonine kinase 1	RIPK1
206672_at	0.43	aquaporin 2 (collecting duct)	AQP2
1553248_at	0.43	coiled-coil domain containing 57	CCDC57
235157_at	0.43		
1560855_at	0.43		
225277_at	0.43	solute carrier family 39 (zinc transporter), member 13	SLC39A13
211302_s_at	0.43	phosphodiesterase 4B, cAMP-specific (phosphodiesterase E4 dunce homolog, Drosophila)	PDE4B
207133_x_at	0.43	alpha-kinase 1	ALPK1
239448_at	0.43		
1566541_at	0.43		
232792_at	0.43	tripartite motif-containing 69	TRIM69
225116_at	0.43	homeodomain interacting protein kinase 2	HIPK2
216604_s_at	0.43	solute carrier family 7 (cationic amino acid transporter, y+ system), member 8	SLC7A8
230999_at	0.43		
209341_s_at	0.43	inhibitor of kappa light polypeptide gene enhancer in B-cells, kinase beta	IKBKB
218627_at	0.43		
209217_s_at	0.43	WD repeat domain 45	WDR45
232947_at	0.43		
1555705_a_at	0.43	CKLF-like MARVEL transmembrane domain containing 3	CMTM3
221779_at	0.43	MICAL-like 1	MICALL1
236161_at	0.43		
215553_x_at	0.43		
221653_x_at	0.43	apolipoprotein L, 2	APOL2
202856_s_at	0.43	solute carrier family 16, member 3 (monocarboxylic acid transporter 4)	SLC16A3
220727_at	0.43	potassium channel, subfamily K, member 10	KCNK10
238767_at	0.43		
202145_at	0.43	lymphocyte antigen 6 complex, locus E	LY6E
223314_at	0.43	nucleoporin like 1	NUPL1
207303_at	0.43	phosphodiesterase 1C, calmodulin-dependent 70kDa	PDE1C
213010_at	0.43	protein kinase C, delta binding protein	PRKCDBP
221103_s_at	0.43	WD repeat domain 52	WDR52
55081_at	0.43	MICAL-like 1	MICALL1
216859_x_at	0.43		
242275_at	0.43		
216180_s_at	0.43	synaptojanin 2	SYNJ2
210512_s_at	0.43	vascular endothelial growth factor A	VEGFA
233863_at	0.43	castor zinc finger 1	CASZ1
229543_at	0.43		
1555881_s_at	0.43	leucine zipper, putative tumor suppressor 2	LZTS2
213988_s_at	0.43	spermidine/spermine N1-acetyltransferase 1	SAT1
202545_at	0.43	protein kinase C, delta	PRKCD

203037_s_at	0.43	metastasis suppressor 1	MTSS1
235670_at	0.43		
202357_s_at	0.43	complement factor B	CFB
227458_at	0.43		
1554690_a_at	0.43	transforming, acidic coiled-coil containing protein 1	TACC1
204436_at	0.43	pleckstrin homology domain containing, family Q member 1	PLEKHQ1
206510_at	0.43	sine oculis homeobox homolog 2 (Drosophila)	SIX2
203713_s_at	0.43	lethal giant larvae homolog 2 (Drosophila)	LLGL2
233771_at	0.43	triple functional domain (PTPRF interacting)	TRIO
225598_at	0.43	solute carrier family 45, member 4	SLC45A4
210853_at	0.43	sodium channel, voltage-gated, type XI, alpha subunit	SCN11A
231769_at	0.43	F-box protein 6	FBXO6
1561673_at	0.43		
217762_s_at	0.43	RAB31, member RAS oncogene family	RAB31
227038_at	0.43		
221176_x_at	0.43	Williams-Beuren syndrome chromosome region 23	WBSCR23
208018_s_at	0.43	hemopoietic cell kinase	HCK
244503_at	0.43	brain-derived neurotrophic factor	BDNF
221748_s_at	0.43	tensin 1	TNS1
230728_at	0.43		
1556222_at	0.43		
209949_at	0.43	neutrophil cytosolic factor 2 (65kDa, chronic granulomatous disease, autosomal 2)	NCF2
242157_at	0.43	chromodomain helicase DNA binding protein 9	CHD9
220639_at	0.43	transmembrane 4 L six family member 20	TM4SF20
206478_at	0.43	KIAA0125	KIAA0125
230757_at	0.43		
240257_at	0.43	synaptojanin 2	SYNJ2
236982_at	0.43		
1570007_at	0.43	leucine rich repeat containing 8 family, member C	LRRC8C
218093_s_at	0.42	ankyrin repeat domain 10	ANKRD10
224780_at	0.42	RNA binding motif protein 17	RBM17
228610_at	0.42	transmembrane 9 superfamily member 3	TM9SF3
240363_at	0.42	ankyrin 1, erythrocytic	ANK1
223607_x_at	0.42	zinc finger, SWIM-type containing 1	ZSWIM1
222363_at	0.42		
208056_s_at	0.42	core-binding factor, runt domain, alpha subunit 2; translocated to, 3	CBFA2T3
232031_s_at	0.42	KIAA1632	KIAA1632
235629_at	0.42		
238853_at	0.42		
1555847_a_at	0.42		
214643_x_at	0.42	bridging integrator 1	BIN1
242109_at	0.42	synaptotagmin-like 3	SYTL3
212272_at	0.42	lipin 1	LPIN1
201169_s_at	0.42	basic helix-loop-helix domain containing, class B, 2	BHLHB2
214255_at	0.42	ATPase, Class V, type 10A	ATP10A

227749_at	0.42		
216189_at	0.42		
1558354_s_at	0.42		
207499_x_at	0.42	unc-45 homolog A (C. elegans)	UNC45A
239749_at	0.42	Fas (TNFRSF6) associated factor 1	FAF1
228362_s_at	0.42		
239058_at	0.42		
212120_at	0.42	ras homolog gene family, member Q	RHOQ
210064_s_at	0.42	uroplakin 1B	UPK1B
204928_s_at	0.42	solute carrier family 10 (sodium/bile acid cotransporter family), member 3	SLC10A3
202172_at	0.42	vascular endothelial zinc finger 1	VEZF1
230502_s_at	0.42		
219045_at	0.42	ras homolog gene family, member F (in filopodia)	RHOF
234503_at	0.42		
204364_s_at	0.42	receptor accessory protein 1	REEP1
235961_at	0.42	G protein-coupled receptor 161	GPR161
226738_at	0.42	WD repeat domain 81	WDR81
225883_at	0.42	ATG16 autophagy related 16-like 2 (S. cerevisiae)	ATG16L2
220494_s_at	0.42		
212268_at	0.42	serpin peptidase inhibitor, clade B (ovalbumin), member 1	SERPINB1
226824_at	0.42	carboxypeptidase X (M14 family), member 2	CPXM2
202393_s_at	0.42	Kruppel-like factor 10	KLF10
244140_at	0.42	Wolf-Hirschhorn syndrome candidate 1	WHSC1
219520_s_at	0.42	WWC family member 3	WWC3
210123_s_at	0.42	cholinergic receptor, nicotinic, alpha 7	CHRNA7
225602_at	0.42	chromosome 9 open reading frame 19	C9orf19
225858_s_at	0.42	baculoviral IAP repeat-containing 4	BIRC4
202308_at	0.42	sterol regulatory element binding transcription factor 1	SREBF1
237086_at	0.42	forkhead box A1	FOXA1
237999_at	0.42	zinc finger, DHHC-type containing 13	ZDHHC13
1566528_at	0.42		
211682_x_at	0.42	UDP glucuronosyltransferase 2 family, polypeptide B28	UGT2B28
224013_s_at	0.42	SRY (sex determining region Y)-box 7	SOX7
234258_at	0.42		
209409_at	0.42	growth factor receptor-bound protein 10	GRB10
221746_at	0.42	ubiquitin-like 4A	UBL4A
236191_at	0.42	CD38 molecule	CD38
209163_at	0.42	cytochrome b-561	CYB561
203518_at	0.42	lysosomal trafficking regulator	LYST
218921_at	0.42	single immunoglobulin and toll-interleukin 1 receptor (TIR) domain	SIGIRR
202804_at	0.42	ATP-binding cassette, sub-family C (CFTR/MRP), member 1	ABCC1
201460_at	0.42	mitogen-activated protein kinase-activated protein kinase 2	MAPKAPK2
226219_at	0.42	Rho GTPase activating protein 30	ARHGAP30
201749_at	0.42	endothelin converting enzyme 1	ECE1

243539_at	0.42		
205965_at	0.42	basic leucine zipper transcription factor, ATF-like	BATF
227228_s_at	0.42	KIAA1509	KIAA1509
243262_at	0.42	SET and MYND domain containing 3	SMYD3
212717_at	0.42	pleckstrin homology domain containing, family M (with RUN domain) member 1	PLEKHM1
225606_at	0.41	BCL2-like 11 (apoptosis facilitator)	BCL2L11
238423_at	0.41	synaptotagmin-like 3	SYTL3
219794_at	0.41	vacuolar protein sorting 53 homolog (S. cerevisiae)	VPS53
227391_x_at	0.41	leucine rich repeat (in FLII) interacting protein 1	LRRFIP1
231902_at	0.41		
204858_s_at	0.41	endothelial cell growth factor 1 (platelet-derived)	ECGF1
242710_at	0.41		
1556211_a_at	0.41		
227410_at	0.41	family with sequence similarity 43, member A	FAM43A
241635_at	0.41		
218665_at	0.41	frizzled homolog 4 (Drosophila)	FZD4
205692_s_at	0.41	CD38 molecule	CD38
232622_at	0.41		
206618_at	0.41	interleukin 18 receptor 1	IL18R1
211089_s_at	0.41	NIMA (never in mitosis gene a)-related kinase 3	NEK3
229670_at	0.41		
241819_at	0.41	tumor necrosis factor (ligand) superfamily, member 8	TNFSF8
224572_s_at	0.41	interferon regulatory factor 2 binding protein 2	IRF2BP2
201809_s_at	0.41	endoglin (Osler-Rendu-Weber syndrome 1)	ENG
239672_at	0.41		
243972_at	0.41		
213215_at	0.41		
1552489_s_at	0.41	membrane protein, palmitoylated 4 (MAGUK p55 subfamily member 4)	MPP4
1562403_a_at	0.41	solute carrier family 8 (sodium-calcium exchanger), member 3	SLC8A3
203147_s_at	0.41	tripartite motif-containing 14	TRIM14
1562255_at	0.41	synaptotagmin-like 3	SYTL3
215179_x_at	0.41	placental growth factor, vascular endothelial growth factor-related protein	PGF
225133_at	0.41		
1559436_x_at	0.41		
1560770_at	0.41	poly(A) binding protein, cytoplasmic 1	PABPC1
225539_at	0.41	chromosome 21 open reading frame 25#zinc finger protein 295#zinc finger protein 295#PR domain containing 15	C21orf25#ZNF295#ZNF295#PRDM15
202531_at	0.41	interferon regulatory factor 1	IRF1
217604_at	0.41		
229336_at	0.41		
214780_s_at	0.41	myosin IXB	MYO9B
235252_at	0.41	kinase suppressor of ras 1	KSR1

218844_at	0.41		
1566342_at	0.41	superoxide dismutase 2, mitochondrial	SOD2
1556429_a_at	0.41	WD repeat domain 67	WDR67
229164_s_at	0.41	ankyrin repeat and BTB (POZ) domain containing 1	ABTB1
1566656_a_at	0.41		
213888_s_at	0.41	adenosine A2b receptor pseudogene#hydroxysteroid (11-beta) dehydrogenase 1#interferon regulatory factor 6#chromosome 1 open reading frame 107#TRAF3 interacting protein 3#chromosome 1 open reading frame 74	ADORA2BP#HSD11B1#IRF6#C1orf107#TRAF3IP3#C1orf74
61297_at	0.41	CASK interacting protein 2	CASKIN2
209941_at	0.41	receptor (TNFRSF)-interacting serine-threonine kinase 1	RIPK1
225763_at	0.41	RCSD domain containing 1	RCSD1
1559763_at	0.41	zinc finger CCCH-type containing 12C	ZC3H12C
225368_at	0.41	homeodomain interacting protein kinase 2	HIPK2
1553847_a_at	0.41	spermatid associated	SPERT
236295_s_at	0.41	NLR family, CARD domain containing 3	NLRC3
234043_at	0.41		
216216_at	0.41	slit homolog 3 (Drosophila)	SLIT3
218927_s_at	0.41	carbohydrate (chondroitin 4) sulfotransferase 12	CHST12
220174_at	0.41	leucine rich repeat containing 8 family, member E	LRRC8E
1562091_at	0.41		
242578_x_at	0.41	solute carrier family 22 (extraneuronal monoamine transporter), member 3	SLC22A3
214987_at	0.41		
202237_at	0.41	nicotinamide N-methyltransferase	NNMT
230131_x_at	0.41	arylsulfatase D	ARSD
1552283_s_at	0.41	zinc finger, DHHC-type containing 11	ZDHHC11
1557755_at	0.41	chromosome 14 open reading frame 145	C14orf145
206286_s_at	0.40	teratocarcinoma-derived growth factor 1	TDGF1
1568619_s_at	0.40		
218523_at	0.40		
202133_at	0.40	WW domain containing transcription regulator 1	WWTR1
1557316_at	0.40		
226279_at	0.40	protease, serine, 23	PRSS23
211113_s_at	0.40	ATP-binding cassette, sub-family G (WHITE), member 1	ABCG1
206687_s_at	0.40	protein tyrosine phosphatase, non-receptor type 6	PTPN6
234019_at	0.40		
229011_at	0.40		
225632_s_at	0.40	RAB43, member RAS oncogene family	RAB43
1562550_at	0.40		
221443_x_at	0.40	prolactin releasing hormone	PRLH
1563533_at	0.40	glutamate decarboxylase-like 1	GADL1
223228_at	0.40	leucine zipper, down-regulated in cancer 1-like	LDOC1L
219383_at	0.40		

1557731_at	0.40		
209710_at	0.40	GATA binding protein 2	GATA2
244005_at	0.40	glypican 5	GPC5
232485_at	0.40	RUN domain containing 2A	RUNDC2A
228981_at	0.40	transmembrane protein 169	TMEM169
210612_s_at	0.40	synaptojanin 2	SYNJ2
242732_at	0.40	metastasis suppressor 1	MTSS1
214996_at	0.40		
242060_x_at	0.40	PHD finger protein 11	PHF11
233748_x_at	0.40	protein kinase, AMP-activated, gamma 2 non-catalytic subunit	PRKAG2
204667_at	0.40	forkhead box A1	FOXA1
238999_at	0.40		
226101_at	0.40	protein kinase C, epsilon	PRKCE
202083_s_at	0.40	SEC14-like 1 (S. cerevisiae)	SEC14L1
222062_at	0.40	interleukin 27 receptor, alpha	IL27RA
1562278_at	0.40	dynein, axonemal, heavy chain 1	DNAH1
220853_at	0.40	glycosyltransferase-like domain containing 1	GTDC1
226783_at	0.40	heterogeneous nuclear ribonucleoprotein A/B	HNRPAB
227087_at	0.40		
231858_x_at	0.40		
224029_x_at	0.40	sodium channel, voltage-gated, type XI, alpha subunit	SCN11A
239653_at	0.40		
234330_at	0.40		
212895_s_at	0.40	active BCR-related gene	ABR
223443_s_at	0.40		
1556067_a_at	0.40	jumonji domain containing 3	JMJD3
237161_at	0.40		
215068_s_at	0.40	F-box and leucine-rich repeat protein 18	FBXL18
221223_x_at	0.40	cytokine inducible SH2-containing protein	CISH
243915_at	0.40		
225764_at	0.40	ets variant gene 6 (TEL oncogene)	ETV6
231187_at	0.40	solute carrier family 28 (sodium-coupled nucleoside transporter), member 1	SLC28A1
235437_at	0.40		
1568785_a_at	0.40		
1564672_at	0.40		
226576_at	0.40	Rho GTPase activating protein 26	ARHGAP26
1553684_at	0.40	peptidylprolyl isomerase (cyclophilin)-like 6	PPIL6
201566_x_at	0.40	inhibitor of DNA binding 2, dominant negative helix-loop-helix protein	ID2
227193_at	0.40		
201332_s_at	0.40	signal transducer and activator of transcription 6, interleukin-4 induced	STAT6
225330_at	0.40	insulin-like growth factor 1 receptor	IGF1R
232365_at	0.40	seven in absentia homolog 1 (Drosophila)	SIAH1
1565974_at	0.40		
204653_at	0.40	transcription factor AP-2 alpha (activating enhancer binding protein 2 alpha)	TFAP2A
1557145_at	0.40	serine/threonine kinase 38	STK38
243117_at	0.40		
224832_at	0.40	dual specificity phosphatase 16	DUSP16

201471_s_at	0.40	sequestosome 1	SQSTM1
203839_s_at	0.40	tyrosine kinase, non-receptor, 2	TNK2
238432_at	0.39		
229687_s_at	0.39		
202437_s_at	0.39	cytochrome P450, family 1, subfamily B, polypeptide 1	CYP1B1
230104_s_at	0.39		
207638_at	0.39	protease, serine, 7 (enterokinase)	PRSS7
228869_at	0.39		
230706_s_at	0.39	calcium/calmodulin-dependent protein kinase II inhibitor 2	CAMK2N2
242287_at	0.39	CAP-GLY domain containing linker protein 1	CLIP1
226716_at	0.39	proline rich 12	PRR12
227167_s_at	0.39		
244790_at	0.39	mature T-cell proliferation 1	MTCP1
214888_at	0.39	calpain 2, (m/II) large subunit	CAPN2
204272_at	0.39	lectin, galactoside-binding, soluble, 4 (galectin 4)	LGALS4
235860_at	0.39		
1568904_at	0.39		
212993_at	0.39		
233932_at	0.39		
211267_at	0.39	homeobox, ES cell expressed 1	HESX1
242795_at	0.39	heterogeneous nuclear ribonucleoprotein A0	HNRPA0
226062_x_at	0.39	family with sequence similarity 63, member A	FAM63A
230657_at	0.39		
237753_at	0.39		
244655_at	0.39		
211617_at	0.39	aldolase A, fructose-bisphosphate pseudogene 2	ALDOAP2
244037_at	0.39		
242740_at	0.39		
216109_at	0.39	thyroid hormone receptor associated protein 2	THRAP2
202510_s_at	0.39	tumor necrosis factor, alpha-induced protein 2	TNFAIP2
210846_x_at	0.39	tripartite motif-containing 14	TRIM14
210770_s_at	0.39	calcium channel, voltage-dependent, P/Q type, alpha 1A subunit	CACNA1A
202436_s_at	0.39	cytochrome P450, family 1, subfamily B, polypeptide 1	CYP1B1
231913_s_at	0.39	BRCA1/BRCA2-containing complex, subunit 3	BRCC3
222343_at	0.39	BCL2-like 11 (apoptosis facilitator)	BCL2L11
244723_at	0.39		
203556_at	0.39	zinc fingers and homeoboxes 2	ZHX2
210162_s_at	0.39	nuclear factor of activated T-cells, cytoplasmic, calcineurin-dependent 1	NFATC1
234346_x_at	0.39		
214808_at	0.39		
228008_at	0.39		
236199_at	0.39	arachidonate 5-lipoxygenase	ALOX5
223853_at	0.39	blood vessel epicardial substance	BVES
241178_at	0.39	EGF-containing fibulin-like extracellular matrix protein 1	EFEMP1
243626_at	0.39		

205074_at	0.39	solute carrier family 22 (organic cation transporter), member 5	SLC22A5
222877_at	0.39		
226440_at	0.39	dual specificity phosphatase 22	DUSP22
202111_at	0.39	solute carrier family 4, anion exchanger, member 2 (erythrocyte membrane protein band 3-like 1)	SLC4A2
240604_at	0.39	exonuclease domain containing 1	EXOD1
203879_at	0.39	phosphoinositide-3-kinase, catalytic, delta polypeptide	PIK3CD
200706_s_at	0.39	lipopolysaccharide-induced TNF factor	LITAF
202192_s_at	0.39	growth arrest-specific 7	GAS7
233817_at	0.39		
225859_at	0.39	baculoviral IAP repeat-containing 4	BIRC4
227329_at	0.39	zinc finger and BTB domain containing 46	ZBTB46
243846_x_at	0.39		
236432_at	0.39		
226959_at	0.39		
205184_at	0.39	guanine nucleotide binding protein (G protein), gamma 4	GNG4
227539_at	0.39		
230276_at	0.39	family with sequence similarity 49, member A	FAM49A
218330_s_at	0.39	neuron navigator 2	NAV2
1557049_at	0.39		
1562432_at	0.38		
202435_s_at	0.38		
228605_at	0.38		
243474_at	0.38		
1556402_at	0.38		
209277_at	0.38	tissue factor pathway inhibitor 2	TFPI2
209164_s_at	0.38	cytochrome b-561	CYB561
236385_at	0.38		
1559747_at	0.38	KIAA1840	KIAA1840
57715_at	0.38	family with sequence similarity 26, member B	FAM26B
233276_at	0.38	F-box and leucine-rich repeat protein 7	FBXL7
215123_at	0.38		
222154_s_at	0.38		
1563332_at	0.38		
223132_s_at	0.38	tripartite motif-containing 8	TRIM8
202191_s_at	0.38	growth arrest-specific 7	GAS7
1558738_at	0.38	nucleolar protein 3 (apoptosis repressor with CARD domain)	NOL3
201642_at	0.38	interferon gamma receptor 2 (interferon gamma transducer 1)	IFNGR2
237452_at	0.38	collagen, type XXVII, alpha 1	COL27A1
241346_at	0.38	Rho GTPase activating protein 30	ARHGAP30
215462_at	0.38		
207978_s_at	0.38	nuclear receptor subfamily 4, group A, member 3	NR4A3
210873_x_at	0.38	apolipoprotein B mRNA editing enzyme, catalytic polypeptide-like 3A	APOBEC3A
209216_at	0.38	WD repeat domain 45	WDR45
231136_at	0.38		

238642_at	0.38	ankyrin repeat domain 13 family, member D	ANKRD13D
210609_s_at	0.38	tumor protein p53 inducible protein 3	TP53I3
1558938_at	0.38		
234762_x_at	0.38	neurolysin (metallopeptidase M3 family)	NLN
215978_x_at	0.38		
230810_at	0.38	jumonji domain containing 4	JMJD4
213873_at	0.38	discoidin, CUB and LCCL domain containing 2	DCBLD2
236285_at	0.38		
1552582_at	0.38	ATP-binding cassette, sub-family C (CFTR/MRP), member 13	ABCC13
230925_at	0.38	amyloid beta (A4) precursor protein-binding, family B, member 1 interacting protein	APBB1IP
235688_s_at	0.38	TNF receptor-associated factor 4	TRAF4
214933_at	0.38	calcium channel, voltage-dependent, P/Q type, alpha 1A subunit	CACNA1A
206738_at	0.38	apolipoprotein C-IV	APOC4
1552609_s_at	0.38	interleukin 28B (interferon, lambda 3)	IL28B
1568981_at	0.38		
210357_s_at	0.38	spermine oxidase	SMOX
215056_at	0.38		
203708_at	0.38	phosphodiesterase 4B, cAMP-specific (phosphodiesterase E4 dunce homolog, Drosophila)	PDE4B
215801_at	0.38		
236291_at	0.38	retinol dehydrogenase 5 (11-cis/9-cis)	RDH5
209716_at	0.38	colony stimulating factor 1 (macrophage)	CSF1
1564200_at	0.38		
215521_at	0.38	polyhomeotic homolog 3 (Drosophila)	PHC3
206144_at	0.38	membrane associated guanylate kinase, WW and PDZ domain containing 1	MAGI1
1555759_a_at	0.38	chemokine (C-C motif) ligand 5	CCL5
204682_at	0.38	latent transforming growth factor beta binding protein 2	LTBP2
243658_at	0.38	farnesyl-diphosphate farnesyltransferase 1	FDFT1
1566787_at	0.38		
224261_at	0.38		
217591_at	0.38		
219680_at	0.38	NLR family member X1	NLRX1
225305_at	0.38	solute carrier family 25, member 29	SLC25A29
224733_at	0.38	CKLF-like MARVEL transmembrane domain containing 3	CMTM3
219911_s_at	0.37	solute carrier organic anion transporter family, member 4A1	SLCO4A1
210136_at	0.37	myelin basic protein	MBP
242237_at	0.37	THO complex 7 homolog (Drosophila)	THOC7
40420_at	0.37	serine/threonine kinase 10	STK10
1570176_at	0.37		
227514_at	0.37		
237114_at	0.37	trafficking protein particle complex 3	TRAPPC3
232303_at	0.37	zinc finger protein 608	ZNF608
228707_at	0.37	claudin 23	CLDN23
225789_at	0.37	centaurin, gamma 3	CENTG3
1564475_s_at	0.37		

228528_at	0.37		
215369_at	0.37		
243748_at	0.37	eukaryotic translation initiation factor 4E family member 3	EIF4E3
236465_at	0.37	ring finger protein 175	RNF175
226663_at	0.37		
226040_at	0.37		
242324_x_at	0.37	collagen and calcium binding EGF domains 1	CCBE1
226715_at	0.37	forkhead box K1	FOXK1
210786_s_at	0.37	Friend leukemia virus integration 1	FLI1
211182_x_at	0.37	runt-related transcription factor 1 (acute myeloid leukemia 1; aml1 oncogene)	RUNX1
203927_at	0.37	nuclear factor of kappa light polypeptide gene enhancer in B-cells inhibitor, epsilon	NFKBIE
220998_s_at	0.37	unc-93 homolog B1 (C. elegans)	UNC93B1
1569346_a_at	0.37		
220460_at	0.37	solute carrier organic anion transporter family, member 1C1	SLCO1C1
232807_at	0.37	chromosome 3 open reading frame 40	C3orf40
225569_at	0.37	eukaryotic translation initiation factor 2C, 2	EIF2C2
223398_at	0.37	chromosome 9 open reading frame 89	C9orf89
225390_s_at	0.37	Kruppel-like factor 13	KLF13
1564567_at	0.37		
219229_at	0.37	solute carrier organic anion transporter family, member 3A1	SLCO3A1
1553043_a_at	0.37	CD300 molecule-like family member f	CD300LF
204236_at	0.37	Friend leukemia virus integration 1	FLI1
238505_at	0.37	ADP-ribosylarginine hydrolase	ADPRH
228240_at	0.37		
221755_at	0.37	EH domain binding protein 1-like 1	EHBP1L1
213839_at	0.37		
240536_at	0.37		
232806_s_at	0.37	chromosome 3 open reading frame 40	C3orf40
240283_at	0.37		
242739_at	0.37	chromosome 6 open reading frame 201	C6orf201
242358_at	0.37		
1570627_at	0.37	transcription elongation factor B (SIII), polypeptide 3 (110kDa, elongin A)	TCEB3
239797_at	0.37		
1556685_at	0.36		
237669_at	0.36	protein tyrosine phosphatase domain containing 1	PTPDC1
202082_s_at	0.36	SEC14-like 1 (S. cerevisiae)	SEC14L1
222139_at	0.36		
203148_s_at	0.36	tripartite motif-containing 14	TRIM14
232077_s_at	0.36		
224836_at	0.36	nuclear receptor coactivator 6#tumor protein p53 inducible nuclear protein 2	NCOA6#TP53INP2
213191_at	0.36	toll-like receptor adaptor molecule 1	TICAM1
217995_at	0.36	sulfide quinone reductase-like (yeast)	SQRDL
224566_at	0.36		
228394_at	0.36	serine/threonine kinase 10	STK10
228193_s_at	0.36		

223566_s_at	0.36	BCL6 co-repressor	BCOR
1557400_at	0.36		
1554176_a_at	0.36	chromosome 3 open reading frame 33	C3orf33
210845_s_at	0.36	plasminogen activator, urokinase receptor	PLAUR
202417_at	0.36	kelch-like ECH-associated protein 1	KEAP1
219364_at	0.36		
241951_at	0.36		
227811_at	0.36	FYVE, RhoGEF and PH domain containing 3	FGD3
209546_s_at	0.36	apolipoprotein L, 1	APOL1
214724_at	0.36	DIX domain containing 1	DIXDC1
231433_at	0.36		
218266_s_at	0.36	frequenin homolog (Drosophila)	FREQ
232858_at	0.36		
1559882_at	0.36	SAM domain and HD domain 1	SAMHD1
221012_s_at	0.36	tripartite motif-containing 8	TRIM8
212543_at	0.36	absent in melanoma 1	AIM1
242181_at	0.36		
218983_at	0.36	complement component 1, r subcomponent-like	C1RL
225618_at	0.36	Rho GTPase activating protein 27	ARHGAP27
220178_at	0.36	chromosome 19 open reading frame 28	C19orf28
213837_at	0.36	serum/glucocorticoid regulated kinase 2#l(3)mbt-like (Drosophila)#intraflagellar transport 52 homolog (Chlamydomonas)	SGK2#L3MBTL#IFT52
224923_at	0.36	tetratricopeptide repeat domain 7A	TTC7A
207051_at	0.36	solute carrier family 17 (sodium phosphate), member 4	SLC17A4
223915_at	0.36	BCL6 co-repressor	BCOR
243858_at	0.36	steroid sulfatase (microsomal), arylsulfatase C, isozyme S	STS
206032_at	0.36	desmocollin 3	DSC3
230917_at	0.36		
214486_x_at	0.36	CASP8 and FADD-like apoptosis regulator	CFLAR
227233_at	0.36	tetraspanin 2	TSPAN2
229375_at	0.36		
1569270_at	0.36		
1568623_a_at	0.36	solute carrier family 35, member E4	SLC35E4
223377_x_at	0.36	cytokine inducible SH2-containing protein	CISH
238989_at	0.36	C1GALT1-specific chaperone 1	C1GALT1C1
223182_s_at	0.36	1-acylglycerol-3-phosphate O-acyltransferase 3	AGPAT3
242790_at	0.36		
1554400_at	0.36	t-complex-associated-testis-expressed 3	TCTE3
228167_at	0.36		
204613_at	0.36	phospholipase C, gamma 2 (phosphatidylinositol-specific)	PLCG2
208890_s_at	0.35	plexin B2	PLXNB2
234811_at	0.35	centromere protein N	CENPN
204088_at	0.35	purinergic receptor P2X, ligand-gated ion channel, 4	P2RX4
243783_at	0.35		
226143_at	0.35	retinoic acid induced 1	RAI1
238678_at	0.35		

242949_x_at	0.35		
205027_s_at	0.35	mitogen-activated protein kinase kinase kinase 8	MAP3K8
227792_at	0.35		
1559035_a_at	0.35	aryl hydrocarbon receptor	AHR
228046_at	0.35		
229841_at	0.35	eukaryotic translation initiation factor 2C, 2	EIF2C2
1561767_at	0.35		
204679_at	0.35	potassium channel, subfamily K, member 1	KCNK1
1568802_at	0.35		
237367_x_at	0.35	CASP8 and FADD-like apoptosis regulator	CFLAR
203287_at	0.35	ladinin 1	LAD1
1563725_at	0.35	zinc finger protein 583	ZNF583
229499_at	0.35	calpain 13	CAPN13
218902_at	0.35	Notch homolog 1, translocation-associated (Drosophila)	NOTCH1
241310_at	0.35		
1554274_a_at	0.35	slingshot homolog 1 (Drosophila)	SSH1
239611_at	0.35		
244018_at	0.35		
243604_at	0.35		
221530_s_at	0.35	basic helix-loop-helix domain containing, class B, 3	BHLHB3
241353_s_at	0.35		
231880_at	0.35	family with sequence similarity 40, member B	FAM40B
232253_at	0.35		
215602_at	0.35	FYVE, RhoGEF and PH domain containing 2	FGD2
226764_at	0.35		
227396_at	0.35		
232081_at	0.35		
240847_at	0.35	reelin	RELN
228770_at	0.35	G protein-coupled receptor 146	GPR146
232951_at	0.35		
204683_at	0.35	intercellular adhesion molecule 2	ICAM2
1555560_at	0.35	UDP-glucose ceramide glucosyltransferase-like 2	UGCGL2
36499_at	0.35	cadherin, EGF LAG seven-pass G-type receptor 2 (flamingo homolog, Drosophila)	CELSR2
234177_at	0.35		
221064_s_at	0.35	chromosome 16 open reading frame 28	C16orf28
1554825_at	0.35		
220660_at	0.35	chromosome 9 open reading frame 27	C9orf27
232088_x_at	0.35		
1561210_at	0.35		
1566764_at	0.35		
242077_x_at	0.35	chromosome 6 open reading frame 150	C6orf150
217200_x_at	0.35	cytochrome b-561	CYB561
208485_x_at	0.35	CASP8 and FADD-like apoptosis regulator	CFLAR
1558906_a_at	0.35		
1566551_at	0.35		
239474_at	0.35		

212875_s_at	0.35	chromosome 21 open reading frame 25#zinc finger protein 295#zinc finger protein 295#PR domain containing 15	C21orf25#ZNF295#ZNF295#PRDM15
241484_x_at	0.35		
230875_s_at	0.34	ATPase, Class VI, type 11A	ATP11A
216516_at	0.34		
227571_at	0.34		
225227_at	0.34		
240935_at	0.34	transcription factor 2, hepatic; LF-B3; variant hepatic nuclear factor	TCF2
207907_at	0.34	tumor necrosis factor (ligand) superfamily, member 14	TNFSF14
209508_x_at	0.34	CASP8 and FADD-like apoptosis regulator	CFLAR
230369_at	0.34	G protein-coupled receptor 161	GPR161
221062_at	0.34	heparan sulfate (glucosamine) 3-O-sulfotransferase 3B1	HS3ST3B1
37005_at	0.34	neuroblastoma, suppression of tumorigenicity 1	NBL1
228909_at	0.34	chromosome 21 open reading frame 86	C21orf86
221551_x_at	0.34	ST6 (alpha-N-acetyl-neuraminyl-2,3-beta-galactosyl-1,3)-N-acetylgalactosaminide alpha-2,6-sialyltran	ST6GALNAC4
230383_x_at	0.34		
1569416_at	0.34		
1557260_a_at	0.34	zinc finger protein 382	ZNF382
227367_at	0.34		
242316_at	0.34		
243034_at	0.34		
219365_s_at	0.34	CaM kinase-like vesicle-associated	CAMKV
235657_at	0.34		
243147_x_at	0.34		
224940_s_at	0.34	pregnancy-associated plasma protein A, pappalysin 1	PAPPA
1560493_a_at	0.34	CPX chromosome region, candidate 1	CPXCR1
216509_x_at	0.34	myeloid/lymphoid or mixed-lineage leukemia (trithorax homolog, Drosophila); translocated to, 10	MLLT10
208683_at	0.34	calpain 2, (m/II) large subunit	CAPN2
212503_s_at	0.34	DIP2 disco-interacting protein 2 homolog C (Drosophila)	DIP2C
211489_at	0.34	adrenergic, alpha-1A-, receptor	ADRA1A
219563_at	0.34	chromosome 14 open reading frame 139	C14orf139
1552301_a_at	0.34	coronin 6	CORO6
243356_at	0.34	family with sequence similarity 7, member A1	FAM7A1
228621_at	0.34	hemochromatosis type 2 (juvenile)	HFE2
239629_at	0.34	CASP8 and FADD-like apoptosis regulator	CFLAR
243894_at	0.34	solute carrier family 41, member 2	SLC41A2
1570213_at	0.34		
220035_at	0.34	nucleoporin 210kDa	NUP210
205192_at	0.34	mitogen-activated protein kinase kinase kinase 14	MAP3K14
225273_at	0.34	WWC family member 3	WWC3
208849_at	0.34		

203507_at	0.34	CD68 molecule	CD68
237637_at	0.34		
232682_at	0.34	peroxisomal trans-2-enoyl-CoA reductase	PECR
230278_at	0.34		
219727_at	0.34	dual oxidase 2	DUOX2
236231_at	0.34		
228675_at	0.34		
203370_s_at	0.34	PDZ and LIM domain 7 (enigma)	PDLIM7
232708_at	0.34	galactose-1-phosphate uridylyltransferase	GALT
211986_at	0.34	AHNAK nucleoprotein (desmoyokin)	AHNAK
1564449_at	0.34		
233009_at	0.33		
210584_s_at	0.33	polymerase (DNA-directed), delta interacting protein 3	POLDIP3
208111_at	0.33	arginine vasopressin receptor 2 (nephrogenic diabetes insipidus)	AVPR2
217730_at	0.33	transmembrane BAX inhibitor motif containing 1	TMBIM1
206600_s_at	0.33	solute carrier family 16, member 5 (monocarboxylic acid transporter 6)	SLC16A5
228718_at	0.33	zinc finger protein 44	ZNF44
201005_at	0.33	CD9 molecule	CD9
215818_at	0.33	nudix (nucleoside diphosphate linked moiety X)-type motif 7	NUDT7
240329_at	0.33		
228755_at	0.33	PERQ amino acid rich, with GYF domain 1	PERQ1
221081_s_at	0.33	DENN/MADD domain containing 2D	DENND2D
201473_at	0.33	jun B proto-oncogene	JUNB
1556414_at	0.33	chromosome 21 open reading frame 71	C21orf71
1559584_a_at	0.33	chromosome 16 open reading frame 54	C16orf54
209498_at	0.33	carcinoembryonic antigen-related cell adhesion molecule 1 (biliary glycoprotein)	CEACAM1
205896_at	0.33	solute carrier family 22 (organic cation transporter), member 4	SLC22A4
1565825_at	0.33	lipoma HMGIC fusion partner	LHFP
202794_at	0.33	inositol polyphosphate-1-phosphatase	INPP1
219257_s_at	0.33	sphingosine kinase 1	SPHK1
222899_at	0.33	integrin, alpha 11	ITGA11
242811_x_at	0.33		
207643_s_at	0.33	tumor necrosis factor receptor superfamily, member 1A	TNFRSF1A
218880_at	0.33	FOS-like antigen 2	FOSL2
212119_at	0.33	ras homolog gene family, member Q	RHOQ
205640_at	0.33	aldehyde dehydrogenase 3 family, member B1	ALDH3B1
237772_at	0.33		
233325_at	0.33	solute carrier family 35, member D2	SLC35D2
227232_at	0.33	Enah/Vasp-like	EVL
235382_at	0.33		
229242_at	0.33		
1557541_at	0.33	chromosome 9 open reading frame 122	C9orf122
1405_i_at	0.33	chemokine (C-C motif) ligand 5	CCL5
225288_at	0.33		
216284_at	0.33		

227331_at	0.33		
235352_at	0.33		
224570_s_at	0.33		
210044_s_at	0.33	lymphoblastic leukemia derived sequence 1	LYL1
207996_s_at	0.33	chromosome 18 open reading frame 1	C18orf1
225641_at	0.33	MADS box transcription enhancer factor 2, polypeptide D (myocyte enhancer factor 2D)	MEF2D
219648_at	0.33	melanoregulin	MREG
235996_at	0.33	Ras association (RalGDS/AF-6) domain family 8	RASSF8
204401_at	0.33	potassium intermediate/small conductance calcium-activated channel, subfamily N, member 4	KCNN4
230894_s_at	0.33		
1559420_x_at	0.33	calcium channel, voltage-dependent, beta 2 subunit	CACNB2
235221_at	0.33	cerebellin 3 precursor	CBLN3
238097_at	0.33		
232928_at	0.33		
219039_at	0.33	sema domain, immunoglobulin domain (Ig), transmembrane domain (TM) and short cytoplasmic domain, (se	SEMA4C
212099_at	0.33	ras homolog gene family, member B	RHOB
232526_at	0.32	inositol 1,4,5-trisphosphate 3-kinase B	ITPKB
212002_at	0.32	chromosome 1 open reading frame 144	C1orf144
229726_at	0.32	GRB2-related adaptor protein	GRAP
206169_x_at	0.32	zinc finger CCCH-type containing 7B	ZC3H7B
236522_at	0.32	nuclear factor I/A	NFIA
219722_s_at	0.32	glycerophosphodiester phosphodiesterase domain containing 3	GDPD3
216462_at	0.32		
229441_at	0.32	protease, serine, 23	PRSS23
224701_at	0.32	poly (ADP-ribose) polymerase family, member 14	PARP14
212356_at	0.32	KIAA0323	KIAA0323
204655_at	0.32	chemokine (C-C motif) ligand 5	CCL5
231006_at	0.32	spermatogenesis associated 8	SPATA8
213148_at	0.32		
204163_at	0.32	elastin microfibril interfacer 1	EMILIN1
223437_at	0.32	peroxisome proliferator-activated receptor alpha	PPARA
203773_x_at	0.32	biliverdin reductase A	BLVRA
240526_at	0.32	ATPase, Class VI, type 11A	ATP11A
1553828_at	0.32	family with sequence similarity 55, member A	FAM55A
240730_at	0.32		
234958_at	0.32		
239965_at	0.32		
225909_at	0.32	zinc finger protein 775	ZNF775
211862_x_at	0.32	CASP8 and FADD-like apoptosis regulator	CFLAR
234423_x_at	0.32		
214012_at	0.32		
243601_at	0.32		
1566645_at	0.32		

244307_s_at	0.32		
215473_at	0.32		
1560763_at	0.32		
233622_x_at	0.32		
1570146_at	0.32		
227260_at	0.32		
1569872_a_at	0.32		
1556385_at	0.32		
202627_s_at	0.32	serpin peptidase inhibitor, clade E (nexin, plasminogen activator inhibitor type 1), member 1	SERPINE1
230466_s_at	0.32		
237802_at	0.32	XK, Kell blood group complex subunit-related family, member 4	XKR4
221155_x_at	0.32		
226681_at	0.32	ubiquitin-conjugating enzyme E2H (UBC8 homolog, yeast)	UBE2H
239963_at	0.32		
1557411_s_at	0.32	solute carrier family 25, member 43	SLC25A43
1560128_x_at	0.32		
227645_at	0.32	phosphoinositide-3-kinase, regulatory subunit 5, p101	PIK3R5
219433_at	0.32	BCL6 co-repressor	BCOR
215800_at	0.32	dual oxidase 1	DUOX1
227999_at	0.32	PWWP domain containing 2	PWWP2
239984_at	0.31	sodium channel, voltage-gated, type VII, alpha	SCN7A
1565728_at	0.31		
221917_s_at	0.31	G-rich RNA sequence binding factor 1	GRSF1
201373_at	0.31	plectin 1, intermediate filament binding protein 500kDa	PLEC1
205841_at	0.31	Janus kinase 2 (a protein tyrosine kinase)	JAK2
233961_at	0.31		
212538_at	0.31	dedicator of cytokinesis 9	DOCK9
234372_at	0.31		
217650_x_at	0.31	ST3 beta-galactoside alpha-2,3-sialyltransferase 2	ST3GAL2
241869_at	0.31	apolipoprotein L, 6	APOL6
229125_at	0.31	ankyrin repeat domain 38	ANKRD38
216174_at	0.31		
227759_at	0.31	proprotein convertase subtilisin/kexin type 9	PCSK9
223158_s_at	0.31	NIMA (never in mitosis gene a)-related kinase 6	NEK6
216746_at	0.31		
207426_s_at	0.31	tumor necrosis factor (ligand) superfamily, member 4 (tax-transcriptionally activated glycoprotein 1	TNFSF4
222842_at	0.31	eukaryotic translation initiation factor 2C, 4	EIF2C4
226530_at	0.31	Bcl2 modifying factor	BMF
234020_x_at	0.31		
228442_at	0.31		
1556182_x_at	0.31	DR1-associated protein 1 (negative cofactor 2 alpha)	DRAP1
231175_at	0.31	chromosome 6 open reading frame 65	C6orf65

1558139_at	0.31		
225328_at	0.31		
1557553_at	0.31	protein phosphatase 1, regulatory (inhibitor) subunit 12B	PPP1R12B
235306_at	0.31	GTPase, IMAP family member 8	GIMAP8
215609_at	0.31		
231699_at	0.31	nuclear factor of kappa light polypeptide gene enhancer in B-cells inhibitor, alpha	NFKBIA
213367_at	0.31		
235814_at	0.31		
1554844_at	0.31	eyes absent homolog 3 (Drosophila)	EYA3
219041_s_at	0.31	replication initiator 1	REPIN1
212274_at	0.31	lipin 1	LPIN1
220246_at	0.31	calcium/calmodulin-dependent protein kinase ID	CAMK1D
218723_s_at	0.31		
219441_s_at	0.31	leucine-rich repeat kinase 1	LRRK1
201422_at	0.31	interferon, gamma-inducible protein 30	IFI30
237676_at	0.31		
201369_s_at	0.31	zinc finger protein 36, C3H type-like 2	ZFP36L2
211818_s_at	0.31	phosphodiesterase 4C, cAMP-specific (phosphodiesterase E1 dunce homolog, Drosophila)	PDE4C
207092_at	0.31	leptin (obesity homolog, mouse)	LEP
50221_at	0.31	transcription factor EB	TFEB
223863_at	0.31		
238823_at	0.31	formin-like 3	FMNL3
210563_x_at	0.31	CASP8 and FADD-like apoptosis regulator	CFLAR
205092_x_at	0.31	zinc finger and BTB domain containing 1	ZBTB1
211105_s_at	0.31	nuclear factor of activated T-cells, cytoplasmic, calcineurin-dependent 1	NFATC1
209939_x_at	0.31	CASP8 and FADD-like apoptosis regulator	CFLAR
238032_at	0.31		
224801_at	0.31	Nedd4 family interacting protein 2	NDFIP2
226554_at	0.31	zinc finger and BTB domain containing 7A	ZBTB7A
221866_at	0.31	MyoD family inhibitor#transcription factor EB	MDFI#TFEB
242755_at	0.30	SFRS protein kinase 2	SRPK2
221865_at	0.30	chromosome 9 open reading frame 91	C9orf91
233630_at	0.30	CDP-diacylglycerol synthase (phosphatidate cytidylyltransferase) 2	CDS2
226991_at	0.30	nuclear factor of activated T-cells, cytoplasmic, calcineurin-dependent 2	NFATC2
240949_x_at	0.30		
204963_at	0.30	sarcospan (Kras oncogene-associated gene)	SSPN
212276_at	0.30	lipin 1	LPIN1
240824_at	0.30	oligonucleotide/oligosaccharide-binding fold containing 1	OBFC1
239428_at	0.30	RAB1A, member RAS oncogene family	RAB1A
224533_s_at	0.30	interferon, alpha-inducible protein 6	IFI6
239364_at	0.30	ets variant gene 6 (TEL oncogene)	ETV6
1560745_at	0.30		
214696_at	0.30		
235299_at	0.30		

207711_at	0.30	chromosome 20 open reading frame 117	C20orf117
223103_at	0.30	START domain containing 10	STARD10
220002_at	0.30	kinesin family member 26B	KIF26B
243186_at	0.30	amyotrophic lateral sclerosis 2 (juvenile) chromosome region, candidate 13	ALS2CR13
218845_at	0.30	dual specificity phosphatase 22	DUSP22
226932_at	0.30		
211627_x_at	0.30	estrogen receptor 1	ESR1
209050_s_at	0.30	ral guanine nucleotide dissociation stimulator	RALGDS
228258_at	0.30	TBC1 domain family, member 10C	TBC1D10C
231303_at	0.30	chromosome 21 open reading frame 42	C21orf42
224565_at	0.30		
226673_at	0.30	SH2 domain containing 3C	SH2D3C
224499_s_at	0.30	activation-induced cytidine deaminase	AICDA
238086_at	0.30		
1558426_x_at	0.30	transmembrane protein 142B	TMEM142B
230034_x_at	0.30	mitochondrial ribosomal protein L41	MRPL41
241482_at	0.30		
238316_at	0.30	zinc finger protein 567	ZNF567
229908_s_at	0.30		
214705_at	0.30	InaD-like (Drosophila)	INADL
228037_at	0.30		
1569849_at	0.30		
221391_at	0.30	taste receptor, type 2, member 14	TAS2R14
1561121_at	0.30		
239272_at	0.30	matrix metallopeptidase 28	MMP28
243641_at	0.30		
1555394_at	0.30	phosphatidylinositol glycan anchor biosynthesis, class K	PIGK
1561205_at	0.30		
1568812_at	0.30		
203851_at	0.30	insulin-like growth factor binding protein 6	IGFBP6
205419_at	0.30	Epstein-Barr virus induced gene 2 (lymphocyte-specific G protein-coupled receptor)	EBI2
203047_at	0.29	serine/threonine kinase 10	STK10
1555305_at	0.29	forkhead box J2	FOXJ2
228923_at	0.29	S100 calcium binding protein A6	S100A6
1556645_s_at	0.29		
238415_at	0.29		
230499_at	0.29		
1555291_at	0.29	transient receptor potential cation channel, subfamily V, member 3	TRPV3
214297_at	0.29	chondroitin sulfate proteoglycan 4 (melanoma-associated)	CSPG4
1561433_at	0.29		
223961_s_at	0.29	cytokine inducible SH2-containing protein	CISH
225407_at	0.29	myelin basic protein	MBP
219278_at	0.29	mitogen-activated protein kinase kinase kinase 6	MAP3K6
217449_at	0.29		
237072_at	0.29	signal-induced proliferation-associated 1 like 2	SIPA1L2
203961_at	0.29	nebulette	NEBL
1552623_at	0.29	hematopoietic SH2 domain containing	HSH2D

226189_at	0.29	integrin, beta 8	ITGB8
240988_x_at	0.29		
213212_x_at	0.29		
237090_at	0.29		
229188_s_at	0.29	zinc and ring finger 2	ZNRF2
213909_at	0.29	leucine rich repeat containing 15	LRRC15
219313_at	0.29	GRAM domain containing 1C	GRAMD1C
231244_at	0.29	CAS1 domain containing 1	CASD1
220377_at	0.29	family with sequence similarity 30, member A	FAM30A
203490_at	0.29	E74-like factor 4 (ets domain transcription factor)	ELF4
210373_at	0.29	tumor suppressor candidate 4	TUSC4
212122_at	0.29	ras homolog gene family, member Q	RHOQ
239067_s_at	0.29	pannexin 2	PANX2
215078_at	0.29	superoxide dismutase 2, mitochondrial	SOD2
217497_at	0.29	endothelial cell growth factor 1 (platelet-derived)	ECGF1
1557749_at	0.29	EH domain binding protein 1-like 1	EHBP1L1
220870_at	0.28		
237407_at	0.28	HCLS1 binding protein 3	HS1BP3
220326_s_at	0.28		
1558170_at	0.28		
230823_at	0.28		
213620_s_at	0.28	intercellular adhesion molecule 2	ICAM2
209930_s_at	0.28	nuclear factor (erythroid-derived 2), 45kDa	NFE2
231963_at	0.28		
1569001_at	0.28	bone morphogenetic protein 1	BMP1
230582_at	0.28	headcase homolog (Drosophila)	HECA
231688_at	0.28		
235938_at	0.28		
1560798_at	0.28	nuclear receptor coactivator 1	NCOA1
1569257_at	0.28	formin-like 1	FMNL1
1560995_s_at	0.28		
1562938_at	0.28		
207489_at	0.28		
206218_at	0.28	melanoma antigen family B, 2	MAGEB2
227372_s_at	0.28	BAI1-associated protein 2-like 1	BAIAP2L1
227984_at	0.28		
221234_s_at	0.28	BTB and CNC homology 1, basic leucine zipper transcription factor 2	BACH2

215797_at	0.28	T cell receptor alpha locus#T cell receptor alpha variable 6#T cell receptor alpha variable 16#T cell receptor alpha variable 15#T cell receptor alpha variable 14/delta variable 4#T cell receptor alpha variable 13-2#T cell receptor alpha variable 13-1#T cell receptor alpha variable 12-3#T cell receptor alpha variable 12-2#T cell receptor alpha variable 12-1#T cell receptor alpha variable 11#T cell receptor alpha variable 10#T cell receptor alpha variable 9-2#T cell receptor alpha variable 9-1#T cell receptor alpha variable 8-6#T cell receptor alpha variable 8-5#T cell receptor alpha variable 8-4#T cell receptor alpha variable 8-3#T cell receptor alpha variable 8-2#T cell receptor alpha variable 8-1#T cell receptor alpha variable 7#T cell receptor alpha variable 5#null	TRA@#TRAV6#TRAV16#TRAV15#TRAV14DV4#TRAV13-2#TRAV13-1#TRAV12-3#TRAV12-2#TRAV12-1#TRAV11#TRAV10#TRAV9-2#TRAV9-1#TRAV8-6#TRAV8-5#TRAV8-4#TRAV8-3#TRAV8-2#TRAV8-1#TRAV7#TRAV5#null
1559889_at	0.28		
1566958_at	0.28		
220809_at	0.28		
1569810_at	0.28		
229972_at	0.28		
226382_at	0.28		
232068_s_at	0.28	toll-like receptor 4	TLR4
1563529_at	0.28	hydrocephalus inducing homolog 2 (mouse)	HYDIN2
229691_at	0.28		
1563036_at	0.28		
1554108_at	0.28		
217677_at	0.28	pleckstrin homology domain containing, family A (phosphoinositide binding specific) member 2	PLEKHA2
226679_at	0.28	solute carrier family 26, member 11	SLC26A11
203741_s_at	0.28	adenylate cyclase 7	ADCY7
220445_s_at	0.27	CSAG family, member 2	CSAG2
221752_at	0.27	slingshot homolog 1 (Drosophila)	SSH1
231598_x_at	0.27		
223798_at	0.27	solute carrier family 41, member 2	SLC41A2
227648_at	0.27	chromosome 22 open reading frame 32	C22orf32
226055_at	0.27	arrestin domain containing 2	ARRDC2
1564676_a_at	0.27		
228137_s_at	0.27	protein phosphatase 2 (formerly 2A), regulatory subunit B, gamma isoform	PPP2R2C
206121_at	0.27	adenosine monophosphate deaminase 1 (isoform M)	AMPD1
202071_at	0.27	syndecan 4 (amphiglycan, ryudocan)	SDC4
243173_at	0.27	calcium binding protein 7	CABP7
1568644_at	0.27	zinc finger protein 208	ZNF208
219183_s_at	0.27	pleckstrin homology, Sec7 and coiled-coil domains 4	PSCD4
225163_at	0.27	FERM domain containing 4A	FRMD4A
222291_at	0.27		
241581_at	0.27		

237305_at	0.27		
241673_x_at	0.27		
239738_at	0.27	dachshund homolog 2 (Drosophila)	DACH2
202628_s_at	0.27	serpin peptidase inhibitor, clade E (nexin, plasminogen activator inhibitor type 1), member 1	SERPINE1
202687_s_at	0.27	tumor necrosis factor (ligand) superfamily, member 10	TNFSF10
241205_at	0.27	BMP2 inducible kinase	BMP2K
206354_at	0.27	solute carrier organic anion transporter family, member 1B3	SLCO1B3
207986_x_at	0.27	cytochrome b-561	CYB561
215183_at	0.27		
233008_at	0.27		
240133_x_at	0.27		
1561346_at	0.26		
224571_at	0.26	interferon regulatory factor 2 binding protein 2	IRF2BP2
231597_x_at	0.26		
1559812_at	0.26		
210745_at	0.26	one cut domain, family member 1	ONECUT1
210221_at	0.26	cholinergic receptor, nicotinic, alpha 3	CHRNA3
242631_x_at	0.26	deleted in liver cancer 1	DLC1
243934_at	0.26		
212660_at	0.26	PHD finger protein 15	PHF15
1559807_at	0.26		
241824_at	0.26	FOS-like antigen 2	FOSL2
1554964_x_at	0.26	chromosome 6 open reading frame 192	C6orf192
238327_at	0.26		
1562780_at	0.26		
1562122_at	0.26		
1564097_at	0.26		
1554790_at	0.26	zinc finger protein 31	ZNF31
238405_at	0.26		
231733_at	0.26		
244079_at	0.26		
206545_at	0.26	CD28 molecule	CD28
1563389_at	0.26		
1559077_at	0.26	ABI gene family, member 3 (NESH) binding protein	ABI3BP
227779_at	0.26		
210564_x_at	0.26	CASP8 and FADD-like apoptosis regulator	CFLAR
236360_at	0.26		
1560974_s_at	0.26	nitric oxide synthase 1 (neuronal)	NOS1
221016_s_at	0.26	transcription factor 7-like 1 (T-cell specific, HMG-box)	TCF7L1
1561870_at	0.26		
1562644_at	0.26	methylenetetrahydrofolate dehydrogenase (NADP+ dependent) 2-like	MTHFD2L
215231_at	0.26	amiloride binding protein 1 (amine oxidase (copper-containing))	ABP1
1554954_at	0.26	chromosome 21 open reading frame 90	C21orf90
242718_at	0.26		
224569_s_at	0.26	interferon regulatory factor 2 binding protein 2	IRF2BP2

244580_at	0.26		
212817_at	0.26	DnaJ (Hsp40) homolog, subfamily B, member 5	DNAJB5
237016_at	0.26	chromosome 6 open reading frame 128	C6orf128
221392_at	0.26	taste receptor, type 2, member 4	TAS2R4
201945_at	0.26	furin (paired basic amino acid cleaving enzyme)	FURIN
232322_x_at	0.25	START domain containing 10	STARD10
238204_at	0.25	leiomodin 1 (smooth muscle)	LMOD1
209683_at	0.25	family with sequence similarity 49, member A	FAM49A
228226_s_at	0.25	zinc finger protein 775	ZNF775
202887_s_at	0.25	DNA-damage-inducible transcript 4	DDIT4
204567_s_at	0.25	ATP-binding cassette, sub-family G (WHITE), member 1	ABCG1
234770_at	0.25	olfactory receptor, family 51, subfamily A, member 1 pseudogene	OR51A1P
244761_at	0.25		
220897_at	0.25		
243880_at	0.25	golgi SNAP receptor complex member 2	GOSR2
236127_at	0.25		
213120_at	0.25		
1560201_at	0.25	zinc finger protein 713	ZNF713
231473_at	0.25		
225262_at	0.25	FOS-like antigen 2	FOSL2
237124_at	0.25		
201189_s_at	0.25	inositol 1,4,5-triphosphate receptor, type 3	ITPR3
211316_x_at	0.25	CASP8 and FADD-like apoptosis regulator	CFLAR
208217_at	0.25	gamma-aminobutyric acid (GABA) receptor, rho 2	GABRR2
214110_s_at	0.25		
58780_s_at	0.25		
233083_at	0.25	methylenetetrahydrofolate dehydrogenase (NADP+ dependent) 2-like	MTHFD2L
208337_s_at	0.25	nuclear receptor subfamily 5, group A, member 2	NR5A2
219202_at	0.25	rhomboid 5 homolog 2 (Drosophila)	RHBDF2
1556903_at	0.25		
205681_at	0.25	BCL2-related protein A1	BCL2A1
238595_at	0.25		
214329_x_at	0.25	tumor necrosis factor (ligand) superfamily, member 10	TNFSF10
204446_s_at	0.25	arachidonate 5-lipoxygenase	ALOX5
241132_at	0.24		
205242_at	0.24	chemokine (C-X-C motif) ligand 13 (B-cell chemoattractant)	CXCL13
203369_x_at	0.24	PDZ and LIM domain 7 (enigma)	PDLIM7
205069_s_at	0.24	Rho GTPase activating protein 26	ARHGAP26
238725_at	0.24		
224762_at	0.24	serine incorporator 2	SERINC2
236401_at	0.24	amiloride binding protein 1 (amine oxidase (copper-containing))	ABP1
203320_at	0.24	SH2B adaptor protein 3	SH2B3
219878_s_at	0.24	Kruppel-like factor 13	KLF13

244689_at	0.24	peroxisome proliferator-activated receptor alpha	PPARA
203234_at	0.24	uridine phosphorylase 1	UPP1
209278_s_at	0.24	tissue factor pathway inhibitor 2	TFPI2
203726_s_at	0.24	laminin, alpha 3	LAMA3
1566720_at	0.24		
218812_s_at	0.24	transmembrane protein 142B	TMEM142B
233446_at	0.24	one cut domain, family member 2	ONECUT2
230828_at	0.24	GRAM domain containing 2	GRAMD2
217563_at	0.24	clock homolog (mouse)	CLOCK
238221_at	0.24		
232279_at	0.24	PHD finger protein 15	PHF15
238354_x_at	0.24		
227440_at	0.24	ankyrin repeat and sterile alpha motif domain containing 1B	ANKS1B
237187_at	0.24		
241699_at	0.24		
205101_at	0.24	class II, major histocompatibility complex, transactivator	CIITA
235672_at	0.24	microtubule-associated protein 6	MAP6
1555485_s_at	0.24		
233634_at	0.24	MARVEL domain containing 3	MARVELD3
223438_s_at	0.24	peroxisome proliferator-activated receptor alpha	PPARA
221680_s_at	0.24	ets variant gene 7 (TEL2 oncogene)	ETV7
234561_at	0.24	solute carrier family 2 (facilitated glucose transporter), member 13	SLC2A13
218029_at	0.24	family with sequence similarity 65, member A	FAM65A
1556950_s_at	0.24	serpin peptidase inhibitor, clade B (ovalbumin), member 6	SERPINB6
221753_at	0.24	slingshot homolog 1 (Drosophila)	SSH1
1561463_at	0.24		
202910_s_at	0.24	CD97 molecule	CD97
222560_at	0.24	LanC lantibiotic synthetase component C-like 2 (bacterial)	LANCL2
209959_at	0.24	nuclear receptor subfamily 4, group A, member 3	NR4A3
237448_at	0.24		
233853_at	0.23		
1552607_at	0.23	chromosome X open reading frame 52	CXorf52
1569560_at	0.23	HIR histone cell cycle regulation defective homolog A (S. cerevisiae)	HIRA
218368_s_at	0.23	tumor necrosis factor receptor superfamily, member 12A	TNFRSF12A
220872_at	0.23		
206729_at	0.23	tumor necrosis factor receptor superfamily, member 8	TNFRSF8
242481_at	0.23		
220752_at	0.23		
1555133_at	0.23	family with sequence similarity 9, member A	FAM9A
234666_at	0.23		
216444_at	0.23		

207160_at	0.23	interleukin 12A (natural killer cell stimulatory factor 1, cytotoxic lymphocyte maturation factor 1,	IL12A
221118_at	0.23	polycystic kidney disease 2-like 2	PKD2L2
235116_at	0.23	TNF receptor-associated factor 1	TRAF1
1561879_at	0.23		
1560153_at	0.23	Fraser syndrome 1	FRAS1
222253_s_at	0.23		
1560034_a_at	0.23		
243654_at	0.23		
221565_s_at	0.23	family with sequence similarity 26, member B	FAM26B
1563728_at	0.23	chromosome 9 open reading frame 14	C9orf14
202688_at	0.23	tumor necrosis factor (ligand) superfamily, member 10	TNFSF10
228188_at	0.23	FOS-like antigen 2	FOSL2
242096_at	0.23		
235658_at	0.23		
1569580_a_at	0.23		
206486_at	0.23	lymphocyte-activation gene 3	LAG3
225293_at	0.23	collagen, type XXVII, alpha 1	COL27A1
206961_s_at	0.23	Trf (TATA binding protein-related factor)-proximal homolog (Drosophila)	TRFP
1556072_at	0.23	chromosome 22 open reading frame 37	C22orf37
1553558_at	0.23	taste receptor, type 2, member 41	TAS2R41
1560692_at	0.23		
1556839_s_at	0.23	spectrin, beta, non-erythrocytic 5	SPTBN5
220944_at	0.23	peptidoglycan recognition protein 4	PGLYRP4
203823_at	0.23	regulator of G-protein signalling 3	RGS3
236187_s_at	0.23	peptidylprolyl isomerase (cyclophilin)-like 6	PPIL6
1563708_at	0.23	sideroflexin 5	SFXN5
217529_at	0.23		
215595_x_at	0.23		
240118_at	0.23		
233056_x_at	0.23	discs, large (Drosophila) homolog-associated protein 4	DLGAP4
243592_at	0.23	REV1 homolog (S. cerevisiae)	REV1
205081_at	0.23	cysteine-rich protein 1 (intestinal)	CRIP1
231779_at	0.23	interleukin-1 receptor-associated kinase 2	IRAK2
242419_at	0.23		
232480_at	0.22		
238472_at	0.22	F-box protein 9	FBXO9
223159_s_at	0.22	NIMA (never in mitosis gene a)-related kinase 6	NEK6
1566698_at	0.22		
201502_s_at	0.22	nuclear factor of kappa light polypeptide gene enhancer in B-cells inhibitor, alpha	NFKBIA
241506_at	0.22	latrophilin 3	LPHN3
237718_at	0.22	eukaryotic translation initiation factor 4E	EIF4E
1562169_at	0.22		
241203_at	0.22		
210538_s_at	0.22	baculoviral IAP repeat-containing 3	BIRC3
238082_at	0.22		
218532_s_at	0.22		

242152_at	0.22		
1566964_at	0.22		
215761_at	0.22	Dmx-like 2	DMXL2
203844_at	0.22	von Hippel-Lindau tumor suppressor	VHL
205180_s_at	0.22	ADAM metallopeptidase domain 8	ADAM8
206756_at	0.22	carbohydrate (N-acetylglucosamine 6-O) sulfotransferase 7	CHST7
204926_at	0.22	inhibin, beta A (activin A, activin AB alpha polypeptide)	INHBA
1564386_at	0.22	thioredoxin domain containing 8	TXNDC8
1562398_at	0.22		
31845_at	0.22	E74-like factor 4 (ets domain transcription factor)	ELF4
238186_at	0.22		
244342_at	0.22		
220578_at	0.22	ADAMTS-like 4	ADAMTSL4
233202_at	0.22	contactin associated protein-like 3	CNTNAP3
228762_at	0.22	LFNG O-fucosylpeptide 3-beta-N-acetylglucosaminyltransferase	LFNG
231406_at	0.22		
238320_at	0.22		
1564469_at	0.21	leiomodin 3 (fetal)	LMOD3
60471_at	0.21	Ras and Rab interactor 3	RIN3
220269_at	0.21		
204912_at	0.21	interleukin 10 receptor, alpha	IL10RA
1553204_at	0.21		
211786_at	0.21	tumor necrosis factor receptor superfamily, member 9	TNFRSF9
242734_x_at	0.21	galactose-1-phosphate uridylyltransferase	GALT
1569978_x_at	0.21		
216529_at	0.21		
215555_at	0.21		
1568012_at	0.21	CAP-GLY domain containing linker protein 1	CLIP1
214947_at	0.21		
230211_at	0.21		
211361_s_at	0.21	serpin peptidase inhibitor, clade B (ovalbumin), member 13	SERPINB13
1565628_at	0.21		
1568713_a_at	0.21	TBC1 (tre-2/USP6, BUB2, cdc16) domain family, member 1	TBC1D1
208303_s_at	0.21	cytokine receptor-like factor 2	CRLF2
230615_at	0.21	dual oxidase maturation factor 2	DUOXA2
1561370_at	0.21		
1553962_s_at	0.21	ras homolog gene family, member B	RHOB
1569640_s_at	0.21		
1566094_at	0.21		
220118_at	0.21	zinc finger and BTB domain containing 32	ZBTB32
205927_s_at	0.21	cathepsin E	CTSE
1552675_at	0.21	DnaJ (Hsp40) homolog, subfamily B, member 7	DNAJB7
226056_at	0.21		
238044_at	0.21		
232039_at	0.21	KIAA1383	KIAA1383

1566768_at	0.21		
223618_at	0.21	formin 2	FMN2
221684_s_at	0.21	nyctalopin	NYX
204428_s_at	0.21	lecithin-cholesterol acyltransferase	LCAT
205463_s_at	0.21	platelet-derived growth factor alpha polypeptide	PDGFA
1553470_at	0.21	dynein, axonemal, heavy chain like 1	DNAHL1
243756_at	0.21	thrombospondin, type I, domain containing 7A	THSD7A
206599_at	0.20	solute carrier family 16, member 5 (monocarboxylic acid transporter 6)	SLC16A5
221126_at	0.20		
210724_at	0.20	egf-like module containing, mucin-like, hormone receptor-like 3	EMR3
212828_at	0.20	synaptojanin 2	SYNJ2
244773_at	0.20		
229701_at	0.20	DnaJ (Hsp40) homolog, subfamily C, member 9	DNAJC9
238307_at	0.20		
204400_at	0.20	embryonal Fyn-associated substrate	EFS
240950_s_at	0.20		
215475_at	0.20		
231296_at	0.20		
235106_at	0.20	mastermind-like 2 (Drosophila)	MAML2
1555009_a_at	0.20	synaptojanin 2	SYNJ2
204794_at	0.20	dual specificity phosphatase 2	DUSP2
229934_at	0.20		
233755_at	0.20		
1552876_at	0.20	chromosome 21 open reading frame 89	C21orf89
236209_at	0.20		
213590_at	0.20	solute carrier family 16, member 5 (monocarboxylic acid transporter 6)	SLC16A5
204205_at	0.20	apolipoprotein B mRNA editing enzyme, catalytic polypeptide-like 3G	APOBEC3G
237134_at	0.20		
1561985_at	0.20	chromosome 14 open reading frame 39	C14orf39
231265_at	0.20	cytochrome c oxidase subunit VIIb2	COX7B2
243287_s_at	0.20		
209722_s_at	0.20	serpin peptidase inhibitor, clade B (ovalbumin), member 9	SERPINB9
234030_at	0.20		
209374_s_at	0.20	immunoglobulin heavy constant mu	IGHM
236105_at	0.20		
234389_x_at	0.20		
205013_s_at	0.20	adenosine A2a receptor	ADORA2A
213001_at	0.20	angiopoietin-like 2	ANGPTL2
240130_at	0.20		
208387_s_at	0.20	matrix metallopeptidase 24 (membrane-inserted)	MMP24
229354_at	0.20	aryl-hydrocarbon receptor repressor	AHRR
223484_at	0.20	chromosome 15 open reading frame 48	C15orf48
243489_at	0.20		
218353_at	0.20	regulator of G-protein signalling 5	RGS5
219514_at	0.19	angiopoietin-like 2	ANGPTL2

201170_s_at	0.19	basic helix-loop-helix domain containing, class B, 2	BHLHB2
242202_at	0.19		
35626_at	0.19	N-sulfoglucosamine sulfohydrolase (sulfamidase)	SGSH
231734_at	0.19	retinol binding protein 2, cellular	RBP2
237987_x_at	0.19	carbamoyl-phosphate synthetase 1, mitochondrial	CPS1
1562514_at	0.19		
241256_at	0.19		
210029_at	0.19	indoleamine-pyrrole 2,3 dioxygenase	INDO
232423_at	0.19	arylsulfatase D	ARSD
242344_at	0.19	gamma-aminobutyric acid (GABA) A receptor, beta 2	GABRB2
239962_at	0.19		
240026_x_at	0.19		
230170_at	0.19	oncostatin M	OSM
1556941_a_at	0.19		
205440_s_at	0.19	neuropeptide Y receptor Y1	NPY1R
234317_s_at	0.19	storkhead box 2	STOX2
225136_at	0.19	pleckstrin homology domain containing, family A (phosphoinositide binding specific) member 2	PLEKHA2
206866_at	0.19	cadherin 4, type 1, R-cadherin (retinal)	CDH4
244637_at	0.19		
215760_s_at	0.19	glutathione peroxidase 4 (phospholipid hydroperoxidase)#strawberry notch homolog 2 (Drosophila)	GPX4#SBNO2
242468_at	0.19		
1569150_x_at	0.19	PDZ and LIM domain 7 (enigma)	PDLIM7
1560881_a_at	0.19	chromosome 21 open reading frame 22	C21orf22
220682_s_at	0.19	kelch-like 5 (Drosophila)	KLHL5
209262_s_at	0.19	nuclear receptor subfamily 2, group F, member 6	NR2F6
230913_at	0.19		
236443_at	0.19		
207102_at	0.19	aldo-keto reductase family 1, member D1 (delta 4-3-ketosteroid-5-beta-reductase)	AKR1D1
242228_at	0.19		
219584_at	0.18	phospholipase A1 member A	PLA1A
1560813_at	0.18		
204197_s_at	0.18	runt-related transcription factor 3	RUNX3
207859_s_at	0.18	cholinergic receptor, nicotinic, beta 3	CHRNB3
225214_at	0.18		
1552895_a_at	0.18	chromosome 21 open reading frame 99	C21orf99
231881_at	0.18		
240825_at	0.18		
1565755_at	0.18		
220084_at	0.18	chromosome 14 open reading frame 105	C14orf105
237156_at	0.18	IQ motif and Sec7 domain 1	IQSEC1
217236_x_at	0.18	immunoglobulin heavy constant gamma 1 (G1m marker)	IGHG1
204198_s_at	0.18	runt-related transcription factor 3	RUNX3

240995_at	0.18	zinc finger protein 211	ZNF211
234655_at	0.18	annexin A2 pseudogene 2#T cell receptor beta variable 21/OR9-2#T cell receptor beta variable 24/OR9-2#T cell receptor beta variable 20/OR9-2#T cell receptor beta variable orphans on chromosome 9#T cell receptor beta variable 23/OR9-2#T cell receptor beta variable 22/OR9-2#null#suppressor of G2 allele of SKP1 pseudogene (S. cerevisiae)#ankyrin repeat domain 18B	ANXA2P2#TRBV21OR9-2#TRBV24OR9-2#TRBV20OR9-2#TRBVOR9@#TRBV23OR9-2#TRBV22OR9-2#null#SUGT1P#ANKRD18B
219256_s_at	0.18	SH3 domain and tetratricopeptide repeats 1	SH3TC1
1554866_at	0.18	transmembrane protein 135	TMEM135
243004_at	0.18		
224925_at	0.18		
225347_at	0.18	ADP-ribosylation factor-like 8A	ARL8A
234989_at	0.18		
240714_at	0.18		
239698_at	0.18		
211317_s_at	0.18	CASP8 and FADD-like apoptosis regulator	CFLAR
1559172_at	0.18		
233818_at	0.18	zinc finger protein 294	ZNF294
239846_at	0.18		
239423_at	0.18		
1556153_s_at	0.18	nuclear factor of kappa light polypeptide gene enhancer in B-cells inhibitor, zeta	NFKBIZ
221477_s_at	0.18	superoxide dismutase 2, mitochondrial	SOD2
243146_at	0.18	adrenergic, alpha-1A-, receptor	ADRA1A
1558406_a_at	0.18		
217853_at	0.18	tensin 3	TNS3
204268_at	0.18	S100 calcium binding protein A2	S100A2
241274_at	0.18		
201131_s_at	0.18	cadherin 1, type 1, E-cadherin (epithelial)	CDH1
236253_at	0.18	zinc finger protein 546	ZNF546
1564077_at	0.18		
219457_s_at	0.17	Ras and Rab interactor 3	RIN3
243065_at	0.17		
1560241_at	0.17		
235001_at	0.17		
228042_at	0.17	ADP-ribosylarginine hydrolase	ADPRH
212124_at	0.17	zinc finger, MIZ-type containing 1	ZMIZ1
207196_s_at	0.17	TNFAIP3 interacting protein 1	TNIP1
1559394_a_at	0.17		
243335_at	0.17	coiled-coil domain containing 109A	CCDC109A
1553372_at	0.17		
225224_at	0.17	chromosome 20 open reading frame 112#additional sex combs like 1 (Drosophila)	C20orf112#ASXL1
242360_at	0.17	LIM domain binding 2	LDB2
227463_at	0.17		
228885_at	0.17	ribosomal protein L24	RPL24
241605_at	0.17	TruB pseudouridine (psi) synthase homolog 1 (E. coli)	TRUB1
1556304_s_at	0.17		

233441_at	0.17	one cut domain, family member 2	ONECUT2
215550_at	0.17	SLIT-ROBO Rho GTPase activating protein 3	SRGAP3
202638_s_at	0.17	intercellular adhesion molecule 1 (CD54), human rhinovirus receptor	ICAM1
1570177_at	0.17		
222996_s_at	0.17	CXXC finger 5	CXXC5
207375_s_at	0.17	interleukin 15 receptor, alpha	IL15RA
241631_at	0.17		
1562916_at	0.17		
229830_at	0.17		
205399_at	0.17	doublecortin and CaM kinase-like 1	DCAMKL1
244849_at	0.17	sema domain, immunoglobulin domain (Ig), short basic domain, secreted, (semaphorin) 3A	SEMA3A
219668_at	0.17	ganglioside-induced differentiation-associated protein 1-like 1	GDAP1L1
206264_at	0.17	glycosylphosphatidylinositol specific phospholipase D1	GPLD1
1566602_at	0.17	RNA pseudouridylate synthase domain containing 3	RPUSD3
202637_s_at	0.17	intercellular adhesion molecule 1 (CD54), human rhinovirus receptor	ICAM1
237117_at	0.17		
216771_at	0.17		
227925_at	0.16		
205799_s_at	0.16	solute carrier family 3 (cystine, dibasic and neutral amino acid transporters, activator of cystine,	SLC3A1
243869_at	0.16		
237928_at	0.16		
233267_at	0.16	selenium binding protein 1	SELENBP1
208978_at	0.16	cysteine-rich protein 2	CRIP2
227062_at	0.16		
240316_at	0.16	chromosome 9 open reading frame 57	C9orf57
207229_at	0.16	killer cell lectin-like receptor subfamily A, member 1	KLRA1
1570409_x_at	0.16		
221087_s_at	0.16	apolipoprotein L, 3	APOL3
241097_at	0.16	ras homolog gene family, member A	RHOA
238361_s_at	0.16		
207706_at	0.16	Usher syndrome 2A (autosomal recessive, mild)	USH2A
235380_at	0.16		
1570128_at	0.16	DEAD (Asp-Glu-Ala-As) box polypeptide 19A	DDX19A
216408_at	0.16	olfactory receptor, family 2, subfamily B, member 2	OR2B2
215481_s_at	0.16	peroxisomal biogenesis factor 5	PEX5
1563016_at	0.16		
240502_at	0.16		
215642_at	0.16		
223751_x_at	0.16	toll-like receptor 10	TLR10
216439_at	0.16	tyrosine kinase, non-receptor, 2	TNK2
1565132_at	0.16	RNA binding motif protein, Y-linked, family 3, member A pseudogene	RBMY3AP

224516_s_at	0.16	CXXC finger 5	CXXC5
234458_at	0.16	alanyl (membrane) aminopeptidase (aminopeptidase N, aminopeptidase M, microsomal aminopeptidase, CD1	ANPEP
1560175_at	0.16	protein phosphatase 4, regulatory subunit 1-like	PPP4R1L
1563357_at	0.16		
1559450_at	0.16		
203798_s_at	0.16	visinin-like 1	VSNL1
1558867_at	0.16	dermatan sulfate epimerase	DSE
1560652_at	0.15		
219508_at	0.15	glucosaminyl (N-acetyl) transferase 3, mucin type	GCNT3
1563110_at	0.15		
208343_s_at	0.15	nuclear receptor subfamily 5, group A, member 2	NR5A2
1568970_at	0.15	ADAM metallopeptidase domain 18	ADAM18
230628_at	0.15	E1A binding protein p400	EP400
230563_at	0.15	RasGEF domain family, member 1A	RASGEF1A
1565637_at	0.15		
233955_x_at	0.15	CXXC finger 5	CXXC5
223681_s_at	0.15	InaD-like (Drosophila)	INADL
1553706_at	0.15	HtrA serine peptidase 4	HTRA4
238013_at	0.15	pleckstrin homology domain containing, family A (phosphoinositide binding specific) member 2	PLEKHA2
216528_at	0.15		
1556985_at	0.15		
205398_s_at	0.15	SMAD family member 3	SMAD3
240182_at	0.15		
219647_at	0.15	popeye domain containing 2	POPDC2
230966_at	0.15	nucleoporin 62kDa	NUP62
241087_at	0.15		
1556624_at	0.15		
1555353_at	0.15	low density lipoprotein-related protein 1 (alpha-2-macroglobulin receptor)	LRP1
1557620_a_at	0.15	coiled-coil domain containing 38	CCDC38
243386_at	0.15		
234230_at	0.15		
239854_at	0.15		
207361_at	0.15	HMG-box transcription factor 1	HBP1
223782_s_at	0.15	tubulointerstitial nephritis antigen	TINAG
230405_at	0.15		
215610_at	0.15		
219975_x_at	0.15	oleoyl-ACP hydrolase	OLAH
206723_s_at	0.14	endothelial differentiation, lysophosphatidic acid G-protein-coupled receptor, 4	EDG4
33304_at	0.14	interferon stimulated exonuclease gene 20kDa	ISG20
242098_at	0.14		
237692_at	0.14	thyroid adenoma associated	THADA
1556332_at	0.14		
208087_s_at	0.14	Z-DNA binding protein 1	ZBP1
1560637_at	0.14		

206251_s_at	0.14	arginine vasopressin receptor 1A	AVPR1A
210271_at	0.14	neurogenic differentiation 2	NEUROD2
211207_s_at	0.14	acyl-CoA synthetase long-chain family member 6	ACSL6
1555103_s_at	0.14	fibroblast growth factor 7 (keratinocyte growth factor)	FGF7
219935_at	0.14	ADAM metallopeptidase with thrombospondin type 1 motif, 5 (aggrecanase-2)	ADAMTS5
239213_at	0.14	serpin peptidase inhibitor, clade B (ovalbumin), member 1	SERPINB1
234196_at	0.14		
244654_at	0.14	myosin IG	MYO1G
1553776_at	0.14	ubiquitin-conjugating enzyme E2U (putative)	UBE2U
209687_at	0.14	chemokine (C-X-C motif) ligand 12 (stromal cell-derived factor 1)	CXCL12
234844_at	0.14	zinc finger protein 407	ZNF407
213280_at	0.13	GTPase activating Rap/RanGAP domain-like 4	GARNL4
212827_at	0.13	immunoglobulin heavy constant mu	IGHM
232742_at	0.13		
1553418_a_at	0.13	contactin associated protein-like 5	CNTNAP5
206293_at	0.13	sulfotransferase family, cytosolic, 2A, dehydroepiandrosterone (DHEA)-preferring, member 1	SULT2A1
210689_at	0.13	claudin 14	CLDN14
204698_at	0.13	interferon stimulated exonuclease gene 20kDa	ISG20
210170_at	0.13	PDZ and LIM domain 3	PDLIM3
214799_at	0.13	neurofascin homolog (chicken)	NFASC
223887_at	0.13	G protein-coupled receptor 132	GPR132
1557211_a_at	0.13	chromosome 14 open reading frame 86	C14orf86
235886_at	0.13		
206048_at	0.13	ovo-like 2 (Drosophila)	OVOL2
205971_s_at	0.13	chymotrypsinogen B1	CTRB1
1557740_a_at	0.13		
215448_at	0.13		
203045_at	0.13	ninjurin 1	NINJ1
219671_at	0.13	hippocalcin like 4	HPCAL4
209402_s_at	0.13	solute carrier family 12 (potassium/chloride transporters), member 4	SLC12A4
1566428_at	0.13		
205549_at	0.13	Purkinje cell protein 4	PCP4
234219_at	0.13		
234384_at	0.13		
227347_x_at	0.13	hairy and enhancer of split 4 (Drosophila)	HES4
243422_at	0.13		
1565820_x_at	0.13	phosphatase and actin regulator 4	PHACTR4
244308_at	0.13		
1563845_at	0.12		
214868_at	0.12	piwi-like 1 (Drosophila)	PIWIL1
217091_at	0.12		
1569981_at	0.12		
238048_at	0.12	cytoplasmic linker associated protein 2	CLASP2
1558578_a_at	0.12	solute carrier family 13 (sodium/sulfate symporters), member 4	SLC13A4

1569716_at	0.12		
207176_s_at	0.12	CD80 molecule	CD80
241536_at	0.12		
209723_at	0.12	serpin peptidase inhibitor, clade B (ovalbumin), member 9	SERPINB9
225239_at	0.12		
237135_at	0.12		
243015_at	0.12	cytochrome P450, family 3, subfamily A, polypeptide 5	CYP3A5
1562055_at	0.12		
230248_x_at	0.12		
1554438_at	0.12	KIAA1217	KIAA1217
240936_at	0.12	major facilitator superfamily domain containing 1	MFSD1
204469_at	0.12	protein tyrosine phosphatase, receptor-type, Z polypeptide 1	PTPRZ1
215350_at	0.11	spectrin repeat containing, nuclear envelope 1	SYNE1
224091_at	0.11		
1556166_x_at	0.11		
204908_s_at	0.11	B-cell CLL/lymphoma 3	BCL3
228754_at	0.11	solute carrier family 6 (neurotransmitter transporter, taurine), member 6	SLC6A6
242814_at	0.11	serpin peptidase inhibitor, clade B (ovalbumin), member 9	SERPINB9
237244_at	0.11		
235427_at	0.11		
1555055_at	0.11	KIAA0241	KIAA0241
227867_at	0.11		
210072_at	0.11	chemokine (C-C motif) ligand 19	CCL19
241025_at	0.11	UTP6, small subunit (SSU) processome component, homolog (yeast)	UTP6
241032_at	0.11	ankyrin repeat domain 40	ANKRD40
206337_at	0.11	chemokine (C-C motif) receptor 7	CCR7
241562_x_at	0.11		
241861_at	0.11	synaptonemal complex protein 3	SYCP3
1555689_at	0.11	CD80 molecule	CD80
232593_at	0.10		
240232_at	0.10	chromosome 3 open reading frame 1	C3orf1
228432_at	0.10	RAB3A interacting protein (rabin3)	RAB3IP
239744_at	0.10		
1569149_at	0.10	PDZ and LIM domain 7 (enigma)	PDLIM7
1558387_at	0.10		
1564807_at	0.10		
1569569_x_at	0.10		
231247_s_at	0.10		
242623_x_at	0.10		
232607_at	0.10		
206924_at	0.10	interleukin 11	IL11
1564121_at	0.10		
1557717_at	0.10		
234739_at	0.10		
234455_at	0.10	zinc finger protein 1 homolog (mouse)	ZFP1

210078_s_at	0.10	potassium voltage-gated channel, shaker-related subfamily, beta member 1	KCNAB1
216811_at	0.10		
1554816_at	0.10	astrotactin 2	ASTN2
1561864_at	0.09		
243204_at	0.09		
243275_at	0.09	bone morphogenetic protein receptor, type IA	BMPR1A
242604_at	0.09		
223514_at	0.09	caspase recruitment domain family, member 11	CARD11
243588_at	0.09	FERM, RhoGEF (ARHGEF) and pleckstrin domain protein 1 (chondrocyte-derived)	FARP1
205599_at	0.09	TNF receptor-associated factor 1	TRAF1
1568931_at	0.09		
244556_at	0.09	lymphocyte cytosolic protein 2 (SH2 domain containing leukocyte protein of 76kDa)	LCP2
220817_at	0.09	transient receptor potential cation channel, subfamily C, member 4	TRPC4
242170_at	0.09		
205921_s_at	0.09	solute carrier family 6 (neurotransmitter transporter, taurine), member 6	SLC6A6
241760_x_at	0.09		
233419_at	0.08		
1559206_at	0.08	presenilin 1 (Alzheimer disease 3)	PSEN1
230594_at	0.08		
1560174_at	0.08	sperm associated antigen 16	SPAG16
1570250_at	0.08		
204166_at	0.08	strawberry notch homolog 2 (Drosophila)	SBNO2
1566647_s_at	0.08		
237417_at	0.08	regulator of chromosome condensation (RCC1) and BTB (POZ) domain containing protein 1	RCBTB1
234247_at	0.08		
1570015_at	0.08		
207010_at	0.08	gamma-aminobutyric acid (GABA) A receptor, beta 1	GABRB1
1557056_at	0.08		
207272_at	0.08	zinc finger protein 80	ZNF80
240463_at	0.08		
1563133_at	0.08		
227618_at	0.07		
237290_at	0.07		
1554519_at	0.07	CD80 molecule	CD80
232262_at	0.07		
211030_s_at	0.07	solute carrier family 6 (neurotransmitter transporter, taurine), member 6	SLC6A6
216369_at	0.07		
238236_at	0.07		
240330_at	0.07		
1560517_s_at	0.06		
1566902_at	0.06		
220091_at	0.06	solute carrier family 2 (facilitated glucose transporter), member 6	SLC2A6

211343_s_at	0.06	collagen, type XIII, alpha 1	COL13A1
1563223_a_at	0.06	centromere protein I	CENPI
1558496_at	0.06		
1552673_at	0.06	regulatory factor X domain containing 1	RFXDC1
215671_at	0.05	phosphodiesterase 4B, cAMP-specific (phosphodiesterase E4 dunce homolog, Drosophila)	PDE4B
232424_at	0.05	PR domain containing 16	PRDM16
1568882_at	0.05	leucine rich repeat containing 51	LRRC51
226978_at	0.05	peroxisome proliferator-activated receptor alpha	PPARA
242800_at	0.04	Nance-Horan syndrome (congenital cataracts and dental anomalies)	NHS
1564405_at	0.04		
226913_s_at	0.03	SRY (sex determining region Y)-box 8	SOX8
234271_at	0.03	otopetrin 2	OTOP2
206341_at	0.03	interleukin 2 receptor, alpha	IL2RA
