# Supplementary material for: In Vitro Transformation of Primary Human CD34+ Cells by AML Fusion Oncogenes: Early Gene Expression Profiling Reveals Possible Drug Target in AML
Source: PLoS One. 2010 Aug 27;5(8):e12464. doi: 10.1371/journal.pone.0012464 (PMC2929205; doi:10.1371/journal.pone.0012464)
Supplement: Table S17 — Genes deregulated by PML-RARA 3 days after transduction. Primary human CD34+ cells were retrovirally transduced with either control MSCV-IRES-GFP vector or vector expressing PML-RARA and sorted for GFP positivity. Total RNA was extracted 3 days after transduction and subjected to microarray analysis. Microarray data were analyzed by SAM as described in Materials and Methods. Significantly deregulated genes are listed and the false discovery rate (FDR) is shown. (0.14 MB PDF) [file pone.0012464.s017.pdf]

Table S17. Genes deregulated by PML-RARA at 3 d detected by SAM FDR = 10.3%

Probe set ID	Fold Change	Gene Name	Gene Symbol
1553428_at	57.84		
1564580_at	33.60		
242524_at	24.15	cerebellin 4 precursor	CBLN4
233096_at	23.64	KIAA1109	KIAA1109
1556026_at	21.77	iduronate 2-sulfatase (Hunter syndrome)	IDS
1556158_at	18.44		
241888_at	18.12		
215216_at	17.36	vacuolar protein sorting 16 homolog (S. cerevisiae)	VPS16
204688_at	16.93	sarcoglycan, epsilon	SGCE
234655_at	16.11	annexin A2 pseudogene 2	ANXA2P2
204103_at	15.47	chemokine (C-C motif) ligand 4	CCL4
234702_x_at	15.26	cystic fibrosis transmembrane conductance regulator (ATP-binding cassette sub-family C, member 7)	CFTR
1560390_s_at	14.09		
237206_at	13.38	myocardin	MYOCD
1560419_at	13.35	chromodomain helicase DNA binding protein 4	CHD4
244314_at	13.10		
1569831_at	13.06		
244169_x_at	12.99		
216444_at	12.51		
206785_s_at	11.61	killer cell lectin-like receptor subfamily C, member 2	KLRC2
230228_at	11.33		
214954_at	11.21	sushi domain containing 5	SUSD5
239738_at	11.02	dachshund homolog 2 (Drosophila)	DACH2
220870_at	10.78		
226612_at	10.70		
241316_at	10.69	trinucleotide repeat containing 6A	TNRC6A
1562805_at	10.56		
205278_at	10.31	glutamate decarboxylase 1 (brain, 67kDa)	GAD1
238385_at	10.12	chromosome 6 open reading frame 58	C6orf58
211912_at	10.09	c-mer proto-oncogene tyrosine kinase	MERTK
244557_at	10.09		
1552904_at	9.76	neuropilin (NRP) and tolloid (TLL)-like 1	NETO1
1562189_at	9.73	collagen, type XIV, alpha 1 (undulin)	COL14A1
1559252_a_at	9.56	chromosome 20 open reading frame 29	C20orf29
221365_at	9.52	motilin receptor	MLNR
1553212_at	9.49	keratin 78	KRT78
231385_at	9.45	developmental pluripotency associated 3	DPPA3
1561090_at	9.45		
238751_at	9.21		
1562742_at	9.20		
230757_at	9.14		
211560_s_at	9.11	aminolevulinate, delta-, synthase 2 (sideroblastic/hypochromic anemia)	ALAS2
229288_at	9.08		
232853_at	8.89		
217177_s_at	8.86		

233965_at	8.64		
215796_at	8.62	T cell receptor alpha locus	TRA@
208233_at	8.58	podoplanin	PDPN
242005_at	8.58		
1553834_at	8.46		
1562400_at	8.39		
1555722_at	8.38		
234662_at	8.28	actin	ACTGP3
233160_at	8.26	defensin, beta 126#defensin, beta 127#defensin, beta 129#defensin, beta 125#defensin, beta 128	DEFB126#DEFB127#DEFB129#DEFB125#DEFB128
223595_at	8.25	transmembrane protein 133	TMEM133
1552393_at	8.20	ENTH domain containing 1	ENTHD1
215937_at	8.18	prostaglandin D2 receptor (DP)	PTGDR
217048_at	8.15	SHC (Src homology 2 domain containing) transforming protein 1 pseudogene 1	SHC1P1
1555166_a_at	8.10	zinc finger protein 396	ZNF396
1562953_s_at	8.04	chromosome 4 open reading frame 12	C4orf12
234636_at	8.03		
232113_at	7.98		
244602_at	7.91		
1562453_at	7.91		
240876_x_at	7.82	chromosome 15 open reading frame 43	C15orf43
1558672_at	7.75		
236357_at	7.75		
1561141_at	7.72		
219655_at	7.68	chromosome 7 open reading frame 10	C7orf10
216625_at	7.58		
215810_x_at	7.57		
1552858_at	7.48	melanoma antigen family B, 6	MAGEB6
1556019_at	7.47		
209956_s_at	7.47	calcium/calmodulin-dependent protein kinase (CaM kinase) II beta	CAMK2B
223812_at	7.46		
241147_at	7.41		
1554252_a_at	7.41	LAG1 homolog, ceramide synthase 3 (S. cerevisiae)	LASS3
244888_at	7.39		
241806_at	7.32		
1556231_a_at	7.32		
242899_at	7.31		
206346_at	7.29	prolactin receptor	PRLR
215653_at	7.26		
1563906_at	7.19		
207694_at	7.15	POU domain, class 3, transcription factor 4	POU3F4
224052_at	7.13	heat shock transcription factor, Y-linked 1	HSFY1
1558322_a_at	7.11	progestin and adipoQ receptor family member IX	PAQR9
1553607_at	7.04	chromosome 21 open reading frame 109	C21orf109
1569525_s_at	6.96		
203324_s_at	6.96	caveolin 2	CAV2
243932_at	6.94		
1560570_a_at	6.72		
240726_at	6.70		

204667_at	6.68	forkhead box A1	FOXA1
1561110_at	6.67		
215240_at	6.67	integrin, beta 3 (platelet glycoprotein IIIa, antigen CD61)	ITGB3
1559849_at	6.65	zinc finger protein 605	ZNF605
1562916_at	6.58		
220893_at	6.54		
244119_at	6.53		
242709_s_at	6.50		
205893_at	6.48	neuroligin 1	NLGN1
220476_s_at	6.47	chromosome 1 open reading frame 183	C1orf183
211361_s_at	6.40	serpin peptidase inhibitor, clade B (ovalbumin), member 13	SERPINB13
211525_s_at	6.39	glycoprotein V (platelet)	GP5
1567288_at	6.35	olfactory receptor, family 5, subfamily K, member 1	OR5K1
1562751_at	6.34		
204517_at	6.32	peptidylprolyl isomerase C (cyclophilin C)	PPIC
211349_at	6.30	solute carrier family 15 (oligopeptide transporter), member 1	SLC15A1
210138_at	6.28	regulator of G-protein signalling 20	RGS20
1566860_at	6.28		
239506_s_at	6.25		
235801_at	6.23		
205567_at	6.19	carbohydrate (keratan sulfate Gal-6) sulfotransferase 1	CHST1
1553579_a_at	6.15	sperm associated antigen 11	SPAG11
1555489_at	6.15		
233494_at	6.14	v-erb-a erythroblastic leukemia viral oncogene homolog 4 (avian)	ERBB4
233577_at	6.14	chromosome 18 open reading frame 57	C18orf57
1565346_a_at	6.13	ATPase, Na+/K+ transporting, alpha 4 polypeptide	ATP1A4
210661_at	6.12	glycine receptor, alpha 3	GLRA3
1562932_at	6.10		
207214_at	6.09	serine peptidase inhibitor, Kazal type 4	SPINK4
244639_at	6.02	transmembrane BAX inhibitor motif containing 4	TMBIM4
1558971_at	6.01	chromosome 6 open reading frame 190	C6orf190
1561517_at	5.99	sarcospan (Kras oncogene-associated gene)	SSPN
215928_at	5.98		
234752_x_at	5.96	dystrophin (muscular dystrophy, Duchenne and Becker types)	DMD
1567223_at	5.93	high mobility group AT-hook 2	HMGA2
215738_at	5.86		
232384_s_at	5.85		
227503_at	5.84		
242999_at	5.84	Rho guanine nucleotide exchange factor (GEF) 7	ARHGEF7
234369_at	5.80	zinc finger protein 596#olfactory receptor, family 4, subfamily F, member 21#null	ZNF596#OR4F21#null
1564642_at	5.80	runt-related transcription factor 1; translocated to, 1 (cyclin D-related)	RUNX1T1
228431_at	5.74		
229302_at	5.67	transmembrane protein 178	TMEM178
236810_at	5.65		
1559542_a_at	5.64		

1556232_at	5.63	kinesin family member 6	KIF6
236894_at	5.62		
1553645_at	5.57		
220148_at	5.55	aldehyde dehydrogenase 8 family, member A1	ALDH8A1
241592_at	5.55		
234313_at	5.54	nuclear receptor co-repressor 1	NCOR1
241291_at	5.52		
240466_at	5.46		
1562094_at	5.46		
234686_at	5.44	annexin A2 pseudogene 2	ANXA2P2
1555519_at	5.36		
1567376_at	5.36	dynein, axonemal, heavy chain 1	DNAH1
1561422_at	5.35		
1563572_at	5.33		
233126_s_at	5.32	oleoyl-ACP hydrolase	OLAH
1563427_at	5.31		
240293_at	5.31		
240750_at	5.30	chromosome 7 open reading frame 44	C7orf44
234892_at	5.30		
222162_s_at	5.28	ADAM metallopeptidase with thrombospondin type 1 motif, 1	ADAMTS1
222877_at	5.28		
1563743_at	5.27	chromosome 1 open reading frame 180	C1orf180
212094_at	5.25	paternally expressed 10	PEG10
208508_s_at	5.24	olfactory receptor, family 2, subfamily J, member 2	OR2J2
242715_at	5.24		
241218_at	5.23		
219895_at	5.22	family with sequence similarity 70, member A	FAM70A
1561564_at	5.21		
204273_at	5.19	endothelin receptor type B	EDNRB
205691_at	5.19	synaptogyrin 3	SYNGR3
236071_at	5.17		
203874_s_at	5.12	SWI/SNF related, matrix associated, actin dependent regulator of chromatin, subfamily a, member 1	SMARCA1
219436_s_at	5.10	endomucin	EMCN
240968_at	5.10		
1562313_at	5.09	BCL6 co-repressor-like 2	BCORL2
1569112_at	5.08	solute carrier family 44, member 5	SLC44A5
239199_at	5.07		
1553071_a_at	5.07	myozenin 3	MYOZ3
1570506_at	5.05		
205990_s_at	5.05	wingless-type MMTV integration site family, member 5A	WNT5A
224278_at	5.04	chromosome 2 open reading frame 14	C2orf14
1563465_at	5.04	polycystic kidney disease 1 like 1	PKD1L1
230560_at	5.03	syntaxin binding protein 6 (amisyn)	STXBP6
202222_s_at	5.02	desmin	DES
206022_at	5.01	Norrie disease (pseudoglioma)	NDP
235754_at	5.01	hemochromatosis	HFE
215034_s_at	4.98	transmembrane 4 L six family member 1	TM4SF1
1557030_at	4.98	GRB2-associated binding protein 1	GAB1

207406_at	4.98	cytochrome P450, family 7, subfamily A, polypeptide 1	CYP7A1
1554772_at	4.98	chromosome 17 open reading frame 57	C17orf57
211161_s_at	4.97	collagen, type III, alpha 1 (Ehlers-Danlos syndrome type IV, autosomal dominant)	COL3A1
223798_at	4.95	solute carrier family 41, member 2	SLC41A2
233261_at	4.92	early B-cell factor 1	EBF1
1569192_at	4.91		
211820_x_at	4.89	glycophorin A (MNS blood group)	GYPA
227189_at	4.83	copine V	CPNE5
235153_at	4.83	ring finger protein 183	RNF183
243542_at	4.82		
1561468_at	4.80		
241396_at	4.79	neural precursor cell expressed, developmentally down-regulated 4-like	NEDD4L
206772_at	4.78	parathyroid hormone receptor 2	PTHR2
230066_at	4.78	sorting nexin 25	SNX25
239913_at	4.77	solute carrier family 10 (sodium/bile acid cotransporter family), member 4	SLC10A4
239156_at	4.76		
1569858_at	4.75		
1566606_a_at	4.73	testis expressed sequence 9	TEX9
231351_at	4.72		
236719_at	4.71		
1558406_a_at	4.69		
1565693_at	4.69	deoxythymidylate kinase (thymidylate kinase)	DTYMK
220064_at	4.69	tetratricopeptide repeat domain 21B	TTC21B
1565748_at	4.68		
1554436_a_at	4.67	regenerating islet-derived family, member 4	REG4
222063_s_at	4.67	CDP-diacylglycerol synthase (phosphatidate cytidylyltransferase) 1	CDS1
238467_at	4.67		
219250_s_at	4.65	fibronectin leucine rich transmembrane protein 3	FLRT3
241646_s_at	4.65		
243034_at	4.64		
243015_at	4.63	cytochrome P450, family 3, subfamily A, polypeptide 5	CYP3A5
1565588_at	4.62		
210390_s_at	4.62	chemokine (C-C motif) ligand 15	CCL15
238203_at	4.61		
234765_at	4.61		
236556_s_at	4.59	LON peptidase N-terminal domain and ring finger 1	LONRF1
221672_s_at	4.59		
1557346_a_at	4.57		
1561136_at	4.55	glycophorin E	GYPE
238914_at	4.54		
240548_at	4.54		
237494_at	4.53		
233277_at	4.53	deleted in lymphocytic leukemia, 7	DLEU7
237721_s_at	4.53	ankyrin repeat and SOCS box-containing 4	ASB4
1555025_at	4.52	transmembrane protein 26	TMEM26
1560762_at	4.51		
242249_at	4.51		

237732_at	4.50		
233502_at	4.48		
220237_at	4.47	ATG3 autophagy related 3 homolog (S. cerevisiae)	ATG3
1570158_at	4.47		
233302_at	4.46		
244600_at	4.46		
239330_at	4.44		
240571_at	4.44		
214278_s_at	4.44	NDRG family member 2	NDRG2
1552368_at	4.42	CCCTC-binding factor (zinc finger protein)-like	CTCFL
221319_at	4.41	protocadherin beta 8	PCDHB8
229374_at	4.41	EPH receptor A4	EPHA4
206227_at	4.39	cartilage intermediate layer protein, nucleotide pyrophosphohydrolase	CILP
207452_s_at	4.39	contactin 5	CNTN5
204217_s_at	4.39	reticulon 2	RTN2
206617_s_at	4.37	renin binding protein	RENBP
206561_s_at	4.37	aldo-keto reductase family 1, member B10 (aldose reductase)	AKR1B10
240863_at	4.36	cytochrome P450, family 19, subfamily A, polypeptide 1	CYP19A1
243408_at	4.36		
1561191_at	4.36		
205365_at	4.35	homeobox B6	HOXB6
221448_s_at	4.35	testis expressed sequence 15	TEX15
1566995_at	4.34		
242006_at	4.34	chromosome 6 open reading frame 152	C6orf152
1560832_at	4.31		
223838_at	4.31	testis specific, 10	TSGA10
1556578_a_at	4.31		
211494_s_at	4.30	solute carrier family 4, sodium bicarbonate cotransporter, member 4	SLC4A4
237160_at	4.28	coiled-coil domain containing 83	CCDC83
223652_at	4.28	arsenic (+3 oxidation state) methyltransferase	AS3MT
1552477_a_at	4.27	interferon regulatory factor 6	IRF6
244806_at	4.25		
1562686_at	4.24		
221126_at	4.23		
224231_at	4.22		
217194_at	4.22	RAS protein activator like 2	RASAL2
233984_at	4.21		
1559970_at	4.21		
207596_at	4.21		
207780_at	4.20	cylicin, basic protein of sperm head cytoskeleton 2	CYLC2
1563103_at	4.19		
232988_at	4.18	KIAA0182	KIAA0182
234480_at	4.17		
237515_at	4.17	transmembrane protein 56	TMEM56
203066_at	4.17		
1561606_at	4.16		
241767_at	4.14		
241005_at	4.12		
234428_at	4.12		

1561846_s_at	4.11		
1558987_at	4.10		
1552939_at	4.09	angiopoietin 1	ANGPT1
1554744_at	4.08		
1554710_at	4.08	potassium large conductance calcium-activated channel, subfamily M, beta member 1	KCNMB1
228875_at	4.07	chromosome 6 open reading frame 189	C6orf189
1558606_s_at	4.06		
1561000_at	4.05		
233008_at	4.04		
1554474_a_at	4.04	monooxygenase, DBH-like 1	MOXD1
230288_at	4.04	fibroblast growth factor 14	FGF14
1570090_at	4.03		
229151_at	4.02	solute carrier family 14 (urea transporter), member 1 (Kidd blood group)	SLC14A1
242275_at	4.02		
1553944_at	4.00	melanoma inhibitory activity 2	MIA2
1555488_at	3.99		
1569882_at	3.99		
205862_at	3.98		
211479_s_at	3.98	5-hydroxytryptamine (serotonin) receptor 2C	HTR2C
209227_at	3.98		
203399_x_at	3.96	pregnancy specific beta-1-glycoprotein 3	PSG3
206160_at	3.96	apolipoprotein B mRNA editing enzyme, catalytic polypeptide-like 2	APOBEC2
1555755_at	3.96		
229310_at	3.96	kelch-like 29 (Drosophila)	KLHL29
207678_s_at	3.95	SRY (sex determining region Y)-box 30	SOX30
214633_at	3.94	SRY (sex determining region Y)-box 3	SOX3
1559433_at	3.94		
1556420_s_at	3.92	yippee-like 2 (Drosophila)	YPEL2
1558867_at	3.92	dermatan sulfate epimerase	DSE
244762_at	3.92	Down syndrome critical region gene 3	DSCR3
241030_at	3.91	fibrous sheath interacting protein 1	FSIP1
221451_s_at	3.90	olfactory receptor, family 2, subfamily W, member 1	OR2W1
238073_at	3.90	ELAV (embryonic lethal, abnormal vision, Drosophila)-like 4 (Hu antigen D)	ELAVL4
1562949_at	3.88		
1553471_at	3.88	acyl-malonyl condensing enzyme 1	AMAC1
1557845_at	3.87		
215598_at	3.86	tetratricopeptide repeat domain 12	TTC12
1563360_at	3.86		
233343_at	3.86	defensin, beta 126	DEFB126
1557403_s_at	3.85		
1560493_a_at	3.85	CPX chromosome region, candidate 1	CPXCR1
237248_at	3.84	phosphodiesterase 11A	PDE11A
1563333_at	3.84		
226470_at	3.83	gamma-glutamyltransferase-like 3#	GGTL3
206591_at	3.83	recombination activating gene 1	RAG1
233121_at	3.82		
222078_at	3.82		
238447_at	3.82	RNA binding motif, single stranded interacting protein	RBMS3

207916_at	3.81		
234108_at	3.81	taste receptor, type 2, member 45	TAS2R45
238402_s_at	3.80		
230496_at	3.80	family with sequence similarity 123A	FAM123A
1552713_a_at	3.80	solute carrier family 4, anion exchanger, member 1 (erythrocyte membrane protein band 3, Diego blood	SLC4A1
237883_at	3.79		
1566093_at	3.79	Rho guanine nucleotide exchange factor (GEF) 12	ARHGEF12
1553793_a_at	3.78	KIAA1109	KIAA1109
238242_at	3.77		
226863_at	3.75		
219471_at	3.74	chromosome 13 open reading frame 18	C13orf18
207901_at	3.73	interleukin 12B (natural killer cell stimulatory factor 2, cytotoxic lymphocyte maturation factor 2,	IL12B
207705_s_at	3.72		
1555123_at	3.72	ST6 beta-galactosamide alpha-2,6-sialyltranferase 2	ST6GAL2
1553207_at	3.72	ADP-ribosylation factor-like 10	ARL10
1564022_at	3.72	zinc finger protein 804B	ZNF804B
241098_at	3.71	C-type lectin domain family 7, member A	CLEC7A
244467_at	3.70		
206094_x_at	3.70	UDP glucuronosyltransferase 1 family, polypeptide A6	UGT1A6
243401_at	3.70	formin-like 2	FMNL2
203038_at	3.70	protein tyrosine phosphatase, receptor type, K	PTPRK
217423_at	3.69		
231560_at	3.68	leucine rich repeat containing 34	LRRC34
242496_at	3.68		
1556876_s_at	3.66		
222904_s_at	3.66	transmembrane channel-like 5	TMC5
211645_x_at	3.66		
232592_at	3.66		
243057_at	3.65		
220357_s_at	3.65	serum/glucocorticoid regulated kinase 2	SGK2
208446_s_at	3.65	zinc finger, FYVE domain containing 9	ZFYVE9
1560503_a_at	3.64		
1562653_at	3.63		
239984_at	3.63	sodium channel, voltage-gated, type VII, alpha	SCN7A
229638_at	3.63	iroquois homeobox protein 3	IRX3
227475_at	3.62	forkhead box Q1	FOXQ1
1563121_at	3.62		
213268_at	3.61	period homolog 3 (Drosophila)	PER3
1565563_at	3.61		
243275_at	3.60	bone morphogenetic protein receptor, type IA	BMPR1A
1556817_a_at	3.60		
244891_x_at	3.60		
241180_at	3.60		
219983_at	3.59	HRAS-like suppressor	HRASLS
211436_at	3.58		
241096_at	3.58		
213909_at	3.58	leucine rich repeat containing 15	LRRC15
240904_at	3.58		
238060_s_at	3.58	beta-1,4-N-acetyl-galactosaminyl transferase 4	B4GALNT4

206655_s_at	3.57	glycoprotein Ib (platelet), beta polypeptide	GP1BB
1562384_at	3.57		
1555617_x_at	3.57		
219783_at	3.57	chromosome 2 open reading frame 18	C2orf18
1561545_at	3.57		
214407_x_at	3.56	glycophorin B (MNS blood group)	GYPB
1558565_at	3.56		
1564466_at	3.55		
222513_s_at	3.55	sorbin and SH3 domain containing 1	SORBS1
227209_at	3.53	contactin 1	CNTN1
210091_s_at	3.52	dystrobrevin, alpha	DTNA
1566202_at	3.51		
207445_s_at	3.51	chemokine (C-C motif) receptor 9	CCR9
219937_at	3.50	thyrotropin-releasing hormone degrading enzyme	TRHDE
227317_at	3.49	LIM and cysteine-rich domains 1	LMCD1
202011_at	3.49	tight junction protein 1 (zona occludens 1)	TJP1
1562863_at	3.48		
213578_at	3.47	bone morphogenetic protein receptor, type IA	BMPR1A
216764_at	3.47		
1558148_x_at	3.45		
219850_s_at	3.45	ets homologous factor	EHF
205806_at	3.43	retinal outer segment membrane protein 1	ROM1
206769_at	3.42	thymosin, beta 4, Y-linked	TMSB4Y
231128_at	3.42		
238634_x_at	3.42		
213435_at	3.41	SATB family member 2	SATB2
1558813_at	3.41	transmembrane emp24 protein transport domain containing 5	TMED5
206222_at	3.41	tumor necrosis factor receptor superfamily, member 10c, decoy without an intracellular domain	TNFRSF10C
1562273_at	3.40	cyclic nucleotide gated channel alpha 4	CNGA4
1553920_at	3.40	chromosome 9 open reading frame 84	C9orf84
239121_at	3.40	PTK2 protein tyrosine kinase 2	PTK2
213791_at	3.40	proenkephalin	PENK
241387_at	3.39		
231303_at	3.39	chromosome 21 open reading frame 42	C21orf42
240933_at	3.38	electron-transfer-flavoprotein, beta polypeptide	ETFB
207447_s_at	3.38	mannosyl (alpha-1,3-)-glycoprotein beta-1,4-N-acetylglucosaminyltransferase, isozyme C (putative)	MGAT4C
242841_at	3.37		
1562568_at	3.37		
1558425_x_at	3.37		
1561450_at	3.37		
1556700_a_at	3.37		
1561856_at	3.37		
222899_at	3.35	integrin, alpha 11	ITGA11
242351_at	3.35		
1561280_at	3.35		
240898_at	3.35	sperm associated antigen 16	SPAG16
240497_at	3.35		
219935_at	3.34	ADAM metallopeptidase with thrombospondin type 1 motif, 5 (aggrecanase-2)	ADAMTS5
242178_at	3.33	lipase, member I	LIPI

239945_at	3.32		
1558796_a_at	3.32		
237770_at	3.32		
222184_at	3.31		
236939_at	3.31	protein tyrosine phosphatase-like A domain containing 2	PTPLAD2
228426_at	3.31	C-type lectin domain family 2, member D	CLEC2D
213776_at	3.30		
211753_s_at	3.30	relaxin 1	RLN1
220562_at	3.30	cytochrome P450, family 2, subfamily W, polypeptide 1	CYP2W1
206773_at	3.29	lymphocyte antigen 6 complex, locus H	LY6H
242845_at	3.28		
207123_s_at	3.28	matrilin 4	MATN4
1564241_at	3.28	ATPase, Na+/K+ transporting, alpha 4 polypeptide	ATP1A4
206344_at	3.27	paraoxonase 1	PON1
209594_x_at	3.27	pregnancy specific beta-1-glycoprotein 9	PSG9
1554957_at	3.26		
1562103_at	3.26	Janus kinase 1 (a protein tyrosine kinase)	JAK1
1554303_at	3.26	histamine N-methyltransferase	HNMT
1553828_at	3.25	family with sequence similarity 55, member A	FAM55A
240979_at	3.25		
241794_at	3.25	ARP6 actin-related protein 6 homolog (yeast)	ACTR6
1553454_at	3.25	repetin	RPTN
222219_s_at	3.25	transducin-like enhancer of split 2 (E(sp1) homolog, Drosophila)#transducin-like enhancer of split 6 (E(sp1) homolog, Drosophila)	TLE2#TLE6
231181_at	3.24		
206626_x_at	3.24	synovial sarcoma, X breakpoint 1	SSX1
216368_s_at	3.23	collagen, type IV, alpha 3 (Goodpasture antigen)	COL4A3
241474_at	3.23		
244393_x_at	3.22		
214598_at	3.21	claudin 8	CLDN8
235004_at	3.21		
231911_at	3.20	KIAA1189	KIAA1189
236442_at	3.20	D4, zinc and double PHD fingers, family 3	DPF3
239623_at	3.19		
218326_s_at	3.18	leucine-rich repeat-containing G protein-coupled receptor 4	LGR4
217321_x_at	3.18	ataxin 3	ATXN3
211602_s_at	3.18	transient receptor potential cation channel, subfamily C, member 1	TRPC1
230074_s_at	3.18		
244837_at	3.17		
230845_at	3.17		
217274_x_at	3.17	myosin, light chain 4, alkali; atrial, embryonic	MYL4
227376_at	3.17	GLI-Kruppel family member GLI3 (Greig cephalopolysyndactyly syndrome)	GLI3
219962_at	3.17	angiotensin I converting enzyme (peptidyl-dipeptidase A) 2	ACE2
224245_at	3.16	inhibitor of growth family, X-linked, pseudogene	INGX
220872_at	3.16		
230539_at	3.16		

230863_at	3.16	low density lipoprotein-related protein 2	LRP2
239292_at	3.16		
210469_at	3.16	discs, large homolog 5 (Drosophila)	DLG5
1557456_a_at	3.14		
229964_at	3.14	chromosome 9 open reading frame 152	C9orf152
237566_at	3.14		
233234_at	3.13	potassium channel tetramerisation domain containing 16	KCTD16
215306_at	3.13		
213613_s_at	3.12	NAD kinase	NADK
241943_at	3.12		
206526_at	3.12	RIB43A domain with coiled-coils 2	RIBC2
204932_at	3.11	tumor necrosis factor receptor superfamily, member 11b (osteoprotegerin)	TNFRSF11B
236705_at	3.11		
1553484_at	3.11		
233561_at	3.11	SH3 domain and tetratricopeptide repeats 2	SH3TC2
243012_at	3.11		
227997_at	3.11	interleukin 17 receptor D	IL17RD
204029_at	3.10	cadherin, EGF LAG seven-pass G-type receptor 2 (flamingo homolog, Drosophila)	CELSR2
201131_s_at	3.09	cadherin 1, type 1, E-cadherin (epithelial)	CDH1
217376_at	3.08	signal-regulatory protein gamma	SIRPG
241612_at	3.08		
232799_at	3.08		
237225_at	3.08		
1562639_at	3.07	kinesin family member 6	KIF6
215520_at	3.07	pygopus homolog 1 (Drosophila)	PYGO1
217386_at	3.07	mitochondrial ribosomal protein S11 pseudogene 1	MRPS11P1
225568_at	3.06	transmembrane protein 141	TMEM141
1560615_a_at	3.06		
208250_s_at	3.06	deleted in malignant brain tumors 1	DMBT1
211102_s_at	3.06	leukocyte immunoglobulin-like receptor, subfamily A (with TM domain), member 2	LILRA2
208536_s_at	3.06	BCL2-like 11 (apoptosis facilitator)	BCL2L11
242215_at	3.05	glutamate receptor, ionotropic, kainate 4	GRIK4
1569941_at	3.05		
242081_at	3.04		
211906_s_at	3.04	serpin peptidase inhibitor, clade B (ovalbumin), member 4	SERPINB4
220436_at	3.04	contactin associated protein-like 3B	CNTNAP3B
224490_s_at	3.04	KIAA1267	KIAA1267
242093_at	3.04	synaptotagmin-like 5	SYTL5
220991_s_at	3.03	ring finger protein 32	RNF32
216657_at	3.03	ataxin 3	ATXN3
244608_at	3.02		
217220_at	3.02		
235503_at	3.02	ankyrin repeat and SOCS box-containing 5	ASB5
244213_at	3.02		
242104_at	3.01		
228438_at	3.01		
241777_x_at	3.01	adaptor protein, phosphotyrosine interaction, PH domain and leucine zipper containing 2	APPL2

227174_at	3.01	WD repeat domain 72	WDR72
224193_s_at	3.00	Fc receptor-like 2	FCRL2
1562214_at	3.00		
233170_at	3.00		
213183_s_at	3.00	cyclin-dependent kinase inhibitor 1C (p57, Kip2)	CDKN1C
220564_at	2.99	chromosome 10 open reading frame 59	C10orf59
1552791_a_at	2.98	triadin	TRDN
1563173_at	2.98		
238235_at	2.97		
1563725_at	2.96	zinc finger protein 583	ZNF583
231142_at	2.96		
231594_at	2.96		
224965_at	2.95	guanine nucleotide binding protein (G protein), gamma 2	GNG2
1568882_at	2.95	leucine rich repeat containing 51	LRRC51
1559991_s_at	2.95		
1567380_at	2.94		
214639_s_at	2.94	homeobox A1	HOXA1
232851_at	2.94	F-box protein 3	FBXO3
206306_at	2.93	ryanodine receptor 3	RYR3
239015_at	2.93		
241898_at	2.93		
233192_s_at	2.93	RUN and FYVE domain containing 2	RUFY2
236990_at	2.93		
227662_at	2.92	synaptopodin 2	SYNPO2
237252_at	2.92	thrombomodulin	THBD
216557_x_at	2.92	interferon, alpha-inducible protein 6	IFI6
217465_at	2.92	NCK-associated protein 1	NCKAP1
236936_at	2.91		
1566598_at	2.90		
1565657_at	2.90	CKLF-like MARVEL transmembrane domain containing 6	CMTM6
1569761_x_at	2.89		
204940_at	2.89	phospholamban	PLN
217033_x_at	2.89	neurotrophic tyrosine kinase, receptor, type 3	NTRK3
1560318_at	2.89	Rho GTPase activating protein 29	ARHGAP29
234340_at	2.89		
222549_at	2.88	claudin 1	CLDN1
1561453_at	2.88		
1559529_at	2.88	PTK2 protein tyrosine kinase 2	PTK2
1559928_at	2.87	pregnancy-associated plasma protein A, pappalysin 1	PAPPA
1560455_at	2.87		
229047_at	2.87	pleckstrin homology domain containing, family B (evectins) member 1	PLEKHB1
213112_s_at	2.86	sequestosome 1	SQSTM1
216590_at	2.86	guanine nucleotide binding protein, alpha transducing 3	GNAT3
206937_at	2.86	spectrin, alpha, erythrocytic 1 (elliptocytosis 2)	SPTA1
241672_at	2.86		
52651_at	2.85	collagen, type VIII, alpha 2	COL8A2
207222_at	2.85	phospholipase A2, group X	PLA2G10
235874_at	2.85	protease, serine, 35	PRSS35

206165_s_at	2.84	chloride channel, calcium activated, family member 2	CLCA2
242815_x_at	2.83		
1569892_at	2.83		
1554302_s_at	2.83		
242843_at	2.82	brevican	BCAN
234174_at	2.82		
1568745_at	2.81		
241834_at	2.81		
227850_x_at	2.81	CDC42 effector protein (Rho GTPase binding) 5	CDC42EP5
1562288_at	2.80		
238755_at	2.80		
217380_s_at	2.80		
231813_s_at	2.79	family with sequence similarity 104, member A	FAM104A
1559105_at	2.79		
240025_x_at	2.79		
1556105_at	2.79		
206794_at	2.79	v-erb-a erythroblastic leukemia viral oncogene homolog 4 (avian)	ERBB4
243095_at	2.78		
230249_at	2.78	KH domain containing, RNA binding, signal transduction associated 3	KHDRBS3
218785_s_at	2.78	RAB, member RAS oncogene family-like 5	RABL5
1569637_at	2.78	zinc finger protein 100	ZNF100
241088_at	2.77		
1557672_s_at	2.77		
236912_at	2.76		
208196_x_at	2.76	nuclear factor of activated T-cells, cytoplasmic, calcineurin-dependent 1	NFATC1
210304_at	2.76	phosphodiesterase 6B, cGMP-specific, rod, beta (congenital stationary night blindness 3, autosomal d	PDE6B
234794_at	2.75		
1553466_at	2.75	chromosome X open reading frame 59	CXorf59
1561207_at	2.75		
206863_x_at	2.75	harakiri, BCL2 interacting protein (contains only BH3 domain)	HRK
1570033_at	2.74	WD repeat domain, phosphoinositide interacting 2	WIPI2
240135_x_at	2.74	TIMP metallopeptidase inhibitor 3 (Sorsby fundus dystrophy, pseudoinflammatory)	TIMP3
206808_at	2.74	heterogeneous nuclear ribonucleoprotein A3 pseudogene 1	HNRPA3P1
1562102_at	2.74	aldo-keto reductase family 1, member C2 (dihydrodiol dehydrogenase 2; bile acid binding protein; 3-a	AKR1C2
214984_at	2.74		
205880_at	2.73	protein kinase D1	PRKD1
204321_at	2.73	neogenin homolog 1 (chicken)	NEO1
237542_at	2.72		
207267_s_at	2.72	Down syndrome critical region gene 6	DSCR6
204416_x_at	2.72	apolipoprotein C-I	APOC1
1552785_at	2.72	zinc finger protein 781	ZNF781
229423_at	2.71		

244112_x_at	2.70		
204509_at	2.70	carbonic anhydrase XII	CA12
241760_x_at	2.70		
241469_at	2.70	HFM1, ATP-dependent DNA helicase homolog (S. cerevisiae)	HFM1
238182_at	2.70		
235916_at	2.69	yippee-like 4 (Drosophila)	YPEL4
1558847_at	2.69		
210273_at	2.69	BH-protocadherin (brain-heart)	PCDH7
1561056_a_at	2.69		
208425_s_at	2.68	tetratricopeptide repeat, ankyrin repeat and coiled-coil containing 2	TANC2
220640_at	2.68	casein kinase 1, gamma 1	CSNK1G1
1559591_s_at	2.68	choline dehydrogenase	CHDH
231658_x_at	2.68	ribosomal protein L36	RPL36
1553066_at	2.68	trace amine associated receptor 9	TAAR9
234652_at	2.67		
1567181_x_at	2.67		
224454_at	2.67	ethanolamine kinase 1	ETNK1
211616_s_at	2.67	5-hydroxytryptamine (serotonin) receptor 2A	HTR2A
239856_at	2.67		
213087_s_at	2.66	eukaryotic translation elongation factor 1 delta (guanine nucleotide exchange protein)	EEF1D
241532_at	2.66	required for meiotic nuclear division 5 homolog A (S. cerevisiae)	RMND5A
237645_at	2.66		
242868_at	2.65	endothelial PAS domain protein 1	EPAS1
1562579_at	2.65		
237485_at	2.65		
239890_s_at	2.65	chromosome 13 open reading frame 21	C13orf21
208035_at	2.64	glutamate receptor, metabotropic 6	GRM6
224894_at	2.64	Yes-associated protein 1, 65kDa	YAP1
1569294_at	2.64	ring finger protein 187	RNF187
207938_at	2.64	peptidase inhibitor 15	PI15
1562669_at	2.64		
1553561_at	2.63	taste receptor, type 2, member 50	TAS2R50
207951_at	2.63	casein beta	CSN2
237918_at	2.63		
215228_at	2.62	nescient helix loop helix 2	NHLH2
217574_at	2.62	cadherin 8, type 2	CDH8
1556170_at	2.62		
220822_at	2.62		
229631_at	2.62	dynein heavy chain domain 1	DNHD1
208027_s_at	2.62	tolloid-like 2	TLL2
218279_s_at	2.61	histone cluster 2, H2aa3#histone cluster 2, H2aa4#histone cluster 2, H2aa4	HIST2H2AA3#HIST2H2AA4#HIST2H2AA4
1558673_s_at	2.61	zinc finger protein 77	ZNF77
1564767_at	2.61		
206615_s_at	2.60	ADAM metallopeptidase domain 22	ADAM22
234145_at	2.60	pre-B-cell leukemia homeobox 3	PBX3
228187_at	2.60	growth arrest-specific 1	GAS1
213839_at	2.60		

1558645_at	2.59	mindbomb homolog 1 (Drosophila)	MIB1
214985_at	2.59		
220278_at	2.59	jumonji domain containing 2D	JMJD2D
239492_at	2.59	SEC14-like 4 (S. cerevisiae)	SEC14L4
205120_s_at	2.59	sarcoglycan, beta (43kDa dystrophin-associated glycoprotein)	SGCB
220595_at	2.59	PDZ domain containing RING finger 4	PDZRN4
1563467_at	2.58		
1565900_at	2.58	methyltransferase 5 domain containing 1	METT5D1
1557232_at	2.58		
242148_at	2.58	B-cell CLL/lymphoma 6, member B (zinc finger protein)	BCL6B
1559889_at	2.58		
241287_x_at	2.57	chromosome 4 open reading frame 15	C4orf15
236305_at	2.57	Rieske (Fe-S) domain containing	RFESD
1570289_at	2.57		
1557769_at	2.57	chimerin (chimaerin) 2	CHN2
206932_at	2.56	cholesterol 25-hydroxylase	CH25H
231985_at	2.56	microtubule associated monoxygenase, calponin and LIM domain containing 3	MICAL3
1556801_at	2.56		
241225_at	2.56		
234632_x_at	2.56		
242579_at	2.55		
224031_at	2.55	serine/threonine kinase 17a (apoptosis-inducing)	STK17A
229452_at	2.54	transmembrane protein 88	TMEM88
233321_x_at	2.54		
217471_at	2.54		
209108_at	2.53	tetraspanin 6	TSPAN6
219232_s_at	2.53	egl nine homolog 3 (C. elegans)	EGLN3
1552658_a_at	2.53	neuron navigator 3	NAV3
241410_at	2.53		
1552639_at	2.53	kelch domain containing 7B	KLHDC7B
240217_s_at	2.53		
1563884_at	2.53		
240488_at	2.52		
244175_at	2.52		
217687_at	2.52	adenylate cyclase 2 (brain)	ADCY2
237295_at	2.52		
210724_at	2.52	egf-like module containing, mucin-like, hormone receptor-like 3	EMR3
1559129_a_at	2.51		
1560348_at	2.51		
235920_at	2.50		
222153_at	2.50	myelin expression factor 2	MYEF2
234475_x_at	2.50	cholecystokinin B receptor	CCKBR
212703_at	2.50	talin 2	TLN2
233227_at	2.50	KIAA1109	KIAA1109
205626_s_at	2.50	calbindin 1, 28kDa	CALB1
241175_at	2.49		
214417_s_at	2.49	fetuin B	FETUB
208396_s_at	2.49	phosphodiesterase 1A, calmodulin-dependent	PDE1A
1563511_at	2.49		

208394_x_at	2.49	endothelial cell-specific molecule 1	ESM1
1554875_at	2.48	chromosome 2 open reading frame 34	C2orf34
222381_at	2.48		
234970_at	2.48	membrane targeting (tandem) C2 domain containing 1	MTAC2D1
1558760_at	2.47		
216828_at	2.47	chromosome 20 open reading frame 80#null	C20orf80#null
220149_at	2.47		
207256_at	2.47	mannose-binding lectin (protein C) 2, soluble (opsonic defect)	MBL2
215284_at	2.47		
227059_at	2.46	glypican 6	GPC6
219604_s_at	2.46	zinc finger protein 3	ZNF3
240445_at	2.46	protocadherin beta 14	PCDHB14
220497_at	2.46	zinc finger protein 214	ZNF214
234744_x_at	2.46		
241184_x_at	2.45	zinc finger protein 407	ZNF407
205825_at	2.45	proprotein convertase subtilisin/kexin type 1	PCSK1
221409_at	2.45	olfactory receptor, family 2, subfamily S, member 2	OR2S2
223863_at	2.45		
211327_x_at	2.45	hemochromatosis	HFE
220150_s_at	2.45	chromosome 6 open reading frame 60	C6orf60
1559890_a_at	2.45	abl-interactor 1	ABI1
1557286_at	2.44		
214509_at	2.44	histone cluster 1, H3i	HIST1H3I
226858_at	2.44	casein kinase 1, epsilon	CSNK1E
236498_s_at	2.44	chromosome 1 open reading frame 86	C1orf86
229186_s_at	2.43	zinc finger protein 64 homolog (mouse)	ZFP64
236886_at	2.43		
233327_at	2.43	chromosome 6 open reading frame 157	C6orf157
239370_at	2.43		
216774_at	2.43		
238283_at	2.43		
1553060_at	2.43	protein serine kinase H2	PSKH2
215560_x_at	2.43	mitochondrial translational release factor 1-like	MTRF1L
232316_at	2.43	chromosome 14 open reading frame 174	C14orf174
220873_at	2.42		
204261_s_at	2.42	presenilin 2 (Alzheimer disease 4)	PSEN2
233202_at	2.42	contactin associated protein-like 3	CNTNAP3
240095_at	2.42		
205950_s_at	2.42	carbonic anhydrase I	CA1
229242_at	2.42		
230887_at	2.41	CDC14 cell division cycle 14 homolog B (S. cerevisiae)	CDC14B
1569040_s_at	2.41		
239444_at	2.40		
1562306_at	2.40		
1553842_at	2.40	chromosome X open reading frame 20	CXorf20
222108_at	2.40	adhesion molecule with Ig-like domain 2	AMIGO2
232804_at	2.40		
208259_x_at	2.40	interferon, alpha 7	IFNA7
213843_x_at	2.40	solute carrier family 6 (neurotransmitter transporter, creatine), member 8	SLC6A8

1554143_a_at	2.40	SGT1, suppressor of G2 allele of SKP1 like 1 (S. cerevisiae)	SUGT1L1
243428_at	2.39	KCNQ1 overlapping transcript 1	KCNQ1OT1
231070_at	2.39	iodotyrosine deiodinase	IYD
243715_at	2.39		
230346_x_at	2.39		
239956_at	2.39		
230988_at	2.39		
235301_at	2.38	KIAA1324-like	KIAA1324L
234688_x_at	2.38	centrobin, centrosomal BRCA2 interacting protein	CNTROB
244388_at	2.38	wingless-type MMTV integration site family, member 5A	WNT5A
230688_at	2.37		
1559948_at	2.37		
1555516_at	2.37	forkhead box P2	FOXP2
241434_at	2.37		
211182_x_at	2.37	runt-related transcription factor 1 (acute myeloid leukemia 1; aml1 oncogene)	RUNX1
230113_at	2.37		
241244_at	2.37		
1561571_at	2.36		
210876_at	2.36	annexin A2 pseudogene 1	ANXA2P1
1553493_a_at	2.36	L-threonine dehydrogenase	TDH
1561148_at	2.36		
1570639_at	2.36		
207401_at	2.35	prospero-related homeobox 1	PROX1
213832_at	2.35		
211745_x_at	2.35	hemoglobin, alpha 2	HBA2
202312_s_at	2.35	collagen, type I, alpha 1	COL1A1
1562301_at	2.34	chromosome 8 open reading frame 34	C8orf34
233351_at	2.34		
228718_at	2.34	zinc finger protein 44	ZNF44
1562656_at	2.34		
244885_at	2.33		
1557712_x_at	2.33		
213492_at	2.33	collagen, type II, alpha 1 (primary osteoarthritis, spondyloepiphyseal dysplasia, congenital)	COL2A1
240110_at	2.33		
1559063_at	2.33		
242280_x_at	2.33	cytoplasmic polyadenylation element binding protein 4	CPEB4
1569856_at	2.32	tripeptidyl peptidase II	TPP2
235619_at	2.32		
220957_at	2.32	cutaneous T-cell lymphoma-associated antigen 1	CTAGE1
225488_at	2.32	Der1-like domain family, member 1	DERL1
237460_x_at	2.32		
222142_at	2.32	cylindromatosis (turban tumor syndrome)	CYLD
233338_at	2.32		
236034_at	2.31		
214341_at	2.31	adaptor-related protein complex 1, gamma 2 subunit	AP1G2
1562888_at	2.31	galactosidase, beta 1 like 3	GLB1L3
214913_at	2.31	ADAM metallopeptidase with thrombospondin type 1 motif, 3	ADAMTS3

1558905_at	2.31		
220551_at	2.31	solute carrier family 17 (sodium-dependent inorganic phosphate cotransporter), member 6	SLC17A6
207601_at	2.30	sulfotransferase family, cytosolic, 1B, member 1	SULT1B1
221461_at	2.30	taste receptor, type 2, member 9	TAS2R9
241694_at	2.30	polycystic kidney and hepatic disease 1 (autosomal recessive)	PKHD1
209458_x_at	2.30	hemoglobin, alpha 1	HBA1
230662_at	2.29	ring finger protein 187	RNF187
218665_at	2.29	frizzled homolog 4 (Drosophila)	FZD4
205132_at	2.29	actin, alpha, cardiac muscle 1	ACTC1
220096_at	2.29	ribonuclease T2	RNASET2
243474_at	2.29		
1555988_a_at	2.29		
242629_at	2.28		
217414_x_at	2.28	hemoglobin, alpha 2	HBA2
243489_at	2.28		
208711_s_at	2.28	cyclin D1	CCND1
219672_at	2.28	erythroid associated factor	ERAF
233373_at	2.28		
1564306_at	2.27		
240725_at	2.27		
204141_at	2.27	tubulin, beta 2A	TUBB2A
240133_x_at	2.26		
1566665_at	2.26		
1567166_at	2.26		
1562850_at	2.26		
209116_x_at	2.26	hemoglobin, beta	HBB
216369_at	2.25		
215473_at	2.25		
1570231_at	2.25	LATS, large tumor suppressor, homolog 1 (Drosophila)	LATS1
226713_at	2.25	coiled-coil domain containing 50	CCDC50
209795_at	2.25	CD69 molecule	CD69
1564215_at	2.25		
205699_at	2.25	mitogen-activated protein kinase kinase 6	MAP2K6
242719_at	2.25		
238564_at	2.25	KIAA1946	KIAA1946
222350_at	2.25		
230958_s_at	2.24		
223644_s_at	2.24	crystallin, gamma S	CRYGS
205564_at	2.24	P antigen family, member 4 (prostate associated)	PAGE4
1569263_at	2.24		
205268_s_at	2.24	adducin 2 (beta)	ADD2
244104_at	2.24	mannosyl (beta-1,4-)-glycoprotein beta-1,4-N-acetylglucosaminyltransferase	MGAT3
208263_at	2.24		
1553123_at	2.24	WD repeat domain 62	WDR62
217232_x_at	2.23	hemoglobin, beta	HBB
1554582_a_at	2.23		
234640_x_at	2.23		
219450_at	2.23	chromosome 4 open reading frame 19	C4orf19
213836_s_at	2.23	WD repeat domain, phosphoinositide interacting 1	WIPI1

1554996_at	2.23	zinc finger protein 479	ZNF479
1552318_at	2.23	GTPase, IMAP family member 1	GIMAP1
1554816_at	2.22	astrotactin 2	ASTN2
237932_at	2.22		
239870_at	2.22	spermatogenesis associated, serine-rich 1	SPATS1
241929_at	2.22		
1556879_at	2.21		
226111_s_at	2.21	zinc finger protein 385	ZNF385
211699_x_at	2.21	hemoglobin, alpha 1	HBA1
215518_at	2.20	syntaxin binding protein 5-like	STXBP5L
1558640_a_at	2.20		
224548_at	2.20	hairy and enhancer of split 7 (Drosophila)	HES7
241969_at	2.20	AT rich interactive domain 5B (MRF1-like)	ARID5B
221128_at	2.20	ADAM metallopeptidase domain 19 (meltrin beta)	ADAM19
221605_s_at	2.20	pipecolic acid oxidase	PIPOX
227809_at	2.20	zinc finger CCCH-type containing 6	ZC3H6
1562086_at	2.20		
205318_at	2.19	kinesin family member 5A	KIF5A
204018_x_at	2.19	hemoglobin, alpha 1	HBA1
1563283_at	2.19		
235906_at	2.19	integrin-linked kinase	ILK
224362_at	2.19		
238353_at	2.18	RAS-like, family 11, member A	RASL11A
243530_at	2.18	cleavage and polyadenylation specific factor 6, 68kDa	CPSF6
1560070_at	2.18		
226701_at	2.17	gap junction protein, alpha 5, 40kDa	GJA5
222377_at	2.17	T-box 10	TBX10
213486_at	2.17		
214366_s_at	2.17	arachidonate 5-lipoxygenase	ALOX5
239359_at	2.17		
1553849_at	2.17	coiled-coil domain containing 26	CCDC26
202478_at	2.17	tribbles homolog 2 (Drosophila)	TRIB2
231050_at	2.16	HRAS-like suppressor family, member 5	HRASLS5
200999_s_at	2.16	cytoskeleton-associated protein 4	CKAP4
214701_s_at	2.16	fibronectin 1	FN1
219550_at	2.15	roundabout, axon guidance receptor, homolog 3 (Drosophila)	ROBO3
221541_at	2.15	cysteine-rich secretory protein LCCL domain containing 2	CRISPLD2
1564344_at	2.15	ATPase, Class II, type 9B	ATP9B
211696_x_at	2.15	hemoglobin, beta	HBB
228821_at	2.15	ST6 beta-galactosamide alpha-2,6-sialyltranferase 2	ST6GAL2
40665_at	2.14	flavin containing monooxygenase 3	FMO3
219441_s_at	2.14	leucine-rich repeat kinase 1	LRRK1
232742_at	2.14		
229057_at	2.13	sodium channel, voltage-gated, type II, alpha subunit	SCN2A
236129_at	2.13	UDP-N-acetyl-alpha-D-galactosamine:polypeptide N-acetylgalactosaminyltransferase 5 (GalNAc-T5)	GALNT5
206698_at	2.13	X-linked Kx blood group (McLeod syndrome)	XK

213343_s_at	2.12	glycerophosphodiester phosphodiesterase domain containing 5	GDPD5
230093_at	2.11	testis specific A2 homolog (mouse)	TSGA2
209469_at	2.11	glycoprotein M6A	GPM6A
232270_at	2.11	chromosome 9 open reading frame 3	C9orf3
236577_at	2.11		
223665_at	2.10		
1564097_at	2.10		
220666_at	2.09		
201839_s_at	2.09	tumor-associated calcium signal transducer 1	TACSTD1
1565130_at	2.09		
208486_at	2.09	dopamine receptor D5	DRD5
208546_x_at	2.08	histone cluster 1, H2bh	HIST1H2BH
243638_at	2.07	quaking homolog, KH domain RNA binding (mouse)	QKI
205389_s_at	2.07	ankyrin 1, erythrocytic	ANK1
243689_s_at	2.06		
211570_s_at	2.06	receptor-associated protein of the synapse, 43kD	RAPSN
218656_s_at	2.06	lipoma HMGIC fusion partner	LHFP
220471_s_at	2.06	myc target 1	MYCT1
1558202_at	2.05		
209555_s_at	2.05	CD36 molecule (thrombospondin receptor)	CD36
1554295_x_at	2.05	tau tubulin kinase 2	TTBK2
205307_s_at	2.05	kynurenine 3-monooxygenase (kynurenine 3-hydroxylase)	KMO
210504_at	2.05	Kruppel-like factor 1 (erythroid)	KLF1
208366_at	2.04	protocadherin 11 X-linked	PCDH11X
1554997_a_at	2.04	prostaglandin-endoperoxide synthase 2 (prostaglandin G/H synthase and cyclooxygenase)	PTGS2
211585_at	2.04	nuclear protein, ataxia-telangiectasia locus	NPAT
231219_at	2.03	chemokine-like factor	CKLF
204748_at	2.03	prostaglandin-endoperoxide synthase 2 (prostaglandin G/H synthase and cyclooxygenase)	PTGS2
203827_at	2.03	WD repeat domain, phosphoinositide interacting 1	WIPI1
1569551_at	2.02		
241773_at	2.02		
1557675_at	2.02	v-raf-1 murine leukemia viral oncogene homolog 1	RAF1
202555_s_at	2.01	myosin, light chain kinase	MYLK
204105_s_at	2.01	neuronal cell adhesion molecule	NRCAM
238632_at	2.01		
217744_s_at	2.00	PERP, TP53 apoptosis effector	PERP
