# Supplementary material for: In Vitro Transformation of Primary Human CD34+ Cells by AML Fusion Oncogenes: Early Gene Expression Profiling Reveals Possible Drug Target in AML
Source: PLoS One. 2010 Aug 27;5(8):e12464. doi: 10.1371/journal.pone.0012464 (PMC2929205; doi:10.1371/journal.pone.0012464)
Supplement: Table S18 — Genes deregulated by PML-RARA 8 days after transduction. Primary human CD34+ cells were retrovirally transduced with either control MSCV-IRES-GFP vector or vector expressing PML-RARA and sorted for GFP positivity. Total RNA was extracted 8 days after transduction and subjected to microarray analysis. Microarray data were analyzed by SAM as described in Materials and Methods. Significantly deregulated genes are listed and the false discovery rate (FDR) is shown. (0.19 MB PDF) [file pone.0012464.s018.pdf]

Table S18. Genes deregulated by PML-RARA at 8 d detected by SAM FDR = 7.81%

Probe set ID	Fold Change	Gene Name	Gene Symbol
1559545_at	54.93	small nuclear ribonucleoprotein polypeptide N	SNRPN
224354_at	26.43		
1562431_x_at	19.60		
1568785_a_at	18.03		
1553470_at	14.83	dynein, axonemal, heavy chain like 1	DNAHL1
230671_at	12.85		
1555801_s_at	12.78	zinc finger protein 533	ZNF533
213591_at	11.59	aldehyde dehydrogenase 7 family, member A1	ALDH7A1
220107_s_at	11.18	chromosome 14 open reading frame 140	C14orf140
1565563_at	11.12		
206489_s_at	10.69	discs, large (Drosophila) homolog-associated protein 1	DLGAP1
1561222_at	10.44		
214023_x_at	10.04	tubulin, beta 2B	TUBB2B
1559287_at	9.84		
215796_at	9.14	T cell receptor alpha locus	TRA@
233793_at	9.09		
1560225_at	8.71	cannabinoid receptor 1 (brain)	CNR1
237634_at	8.69		
1553868_a_at	8.36	chromosome 5 open reading frame 36	C5orf36
221169_s_at	8.10	histamine receptor H4	HRH4
1566995_at	8.10		
203394_s_at	7.88	hairy and enhancer of split 1, (Drosophila)	HES1
231063_at	7.87		
224237_at	7.86		
241656_at	7.75		
220280_s_at	7.65	ankyrin repeat and MYND domain containing 1	ANKMY1
1555273_at	7.57		
203878_s_at	6.94	matrix metallopeptidase 11 (stromelysin 3)	MMP11
217085_at	6.93		
1569453_a_at	6.85		
238575_at	6.83	oxysterol binding protein-like 6	OSBPL6
220878_at	6.79		
207437_at	6.70	neuro-oncological ventral antigen 1	NOVA1
236339_at	6.69	protein phosphatase 1 (formerly 2C)-like	PPM1L
222869_s_at	6.69	elaC homolog 1 (E. coli)	ELAC1
214209_s_at	6.63	ATP-binding cassette, sub-family B (MDR/TAP), member 9	ABCB9
222950_at	6.62	NIPA-like domain containing 2	NPAL2
1556166_x_at	6.59		
237520_x_at	6.44		
212589_at	6.35	related RAS viral (r-ras) oncogene homolog 2	RRAS2
206145_at	6.14	Rh-associated glycoprotein	RHAG
1567056_at	6.11	olfactory receptor, family 8, subfamily G, member 2	OR8G2
1552372_at	6.10	chromosome 4 open reading frame 33	C4orf33
215507_x_at	5.96	RAB22A, member RAS oncogene family	RAB22A
1567280_at	5.94		

1561566_at	5.93		
232122_s_at	5.88	ventricular zone expressed PH domain homolog 1 (zebrafish)	VEPH1
205540_s_at	5.85	Ras-related GTP binding B	RRAGB
244657_at	5.85	glucosidase, beta, acid 3 (cytosolic)	GBA3
220850_at	5.82	MORC family CW-type zinc finger 1	MORC1
1557871_at	5.81		
1553804_a_at	5.71	chromosome 17 open reading frame 46	C17orf46
209498_at	5.67	carcinoembryonic antigen-related cell adhesion molecule 1 (biliary glycoprotein)	CEACAM1
214156_at	5.53	myosin VIIA and Rab interacting protein	MYRIP
228321_s_at	5.51	N-acetyltransferase 12	NAT12
1561418_at	5.49		
231174_s_at	5.44		
242591_at	5.40	dynein, axonemal, heavy chain 1	DNAH1
220205_at	5.25	transmembrane phosphatase with tensin homology	TPTE
211567_at	5.21		
206446_s_at	5.20	elastase 1, pancreatic	ELA1
1570412_at	5.13		
241086_at	5.09		
1552430_at	5.08	WD repeat domain 17	WDR17
210163_at	5.08	chemokine (C-X-C motif) ligand 11	CXCL11
217539_at	5.04	chromosome 18 open reading frame 25	C18orf25
1564438_at	5.00		
227703_s_at	5.00	sushi-repeat-containing protein, X-linked 2#synaptotagmin-like 4 (granuphilin-a)	SRPX2#SYTL4
240036_at	4.98	SEC14-like 1 (S. cerevisiae)	SEC14L1
239492_at	4.95	SEC14-like 4 (S. cerevisiae)	SEC14L4
203666_at	4.89	chemokine (C-X-C motif) ligand 12 (stromal cell-derived factor 1)	CXCL12
228877_at	4.86	erythropoietin receptor	EPOR
223890_at	4.84		
1553472_at	4.81		
220030_at	4.78	serine/threonine/tyrosine kinase 1	STYK1
216200_at	4.76	pleckstrin homology domain containing, family M (with RUN domain) member 1	PLEKHM1
1564950_at	4.75		
216812_at	4.73		
243947_s_at	4.72		
216633_s_at	4.72	phospholipase C, eta 1	PLCH1
203903_s_at	4.69	hephaestin	HEPH
1555003_at	4.68	retinoblastoma-like 1 (p107)	RBL1
237175_at	4.67		
235608_at	4.66		
230545_at	4.60		
234855_at	4.58		
234142_at	4.57		
214940_s_at	4.56	Smg-6 homolog, nonsense mediated mRNA decay factor (C. elegans)	SMG6
1559419_at	4.51	calcium channel, voltage-dependent, beta 2 subunit	CACNB2
234379_at	4.51	fms-related tyrosine kinase 4	FLT4

1567100_at	4.50		
238625_at	4.44	chromosome 1 open reading frame 168	C1orf168
229385_s_at	4.44	placenta-specific 2	PLAC2
214212_x_at	4.43	pleckstrin homology domain containing, family C (with FERM domain) member 1	PLEKHC1
239595_at	4.41	glutathione peroxidase 2 (gastrointestinal)	GPX2
206291_at	4.38	neurotensin	NTS
223588_at	4.35	THAP domain containing, apoptosis associated protein 2	THAP2
1555868_at	4.34		
233755_at	4.34		
234022_at	4.30		
224241_s_at	4.28		
233786_at	4.27		
1562368_at	4.26		
233791_at	4.25		
226847_at	4.24	follistatin	FST
1561870_at	4.22		
208711_s_at	4.21	cyclin D1	CCND1
209772_s_at	4.19	CD24 molecule	CD24
237937_x_at	4.19	syntrophin, gamma 1	SNTG1
230498_at	4.18	melanin-concentrating hormone receptor 1	MCHR1
1569176_at	4.17	transmembrane protease, serine 12	TMPRSS12
219423_x_at	4.16	tumor necrosis factor receptor superfamily, member 25	TNFRSF25
210600_s_at	4.15	G protein-coupled receptor kinase 4	GRK4
244436_at	4.14		
1553508_at	4.13	myelodysplastic syndrome 2 translocation associated	MDS2
218630_at	4.12	Meckel syndrome, type 1	MKS1
230431_at	4.08		
1559069_at	4.05		
207741_x_at	4.04	tryptase beta 2	TPSB2
1567703_at	4.04		
217680_x_at	4.04		
1553013_at	4.03	contactin associated protein-like 5	CNTNAP5
243022_at	4.01		
232368_at	4.01	BET3 like (S. cerevisiae)	BET3L
232355_at	3.99		
206301_at	3.99	tec protein tyrosine kinase	TEC
1555704_at	3.98	CKLF-like MARVEL transmembrane domain containing 3	CMTM3
244745_at	3.98	RAS-like, estrogen-regulated, growth inhibitor	RERG
214159_at	3.96	phospholipase C, epsilon 1	PLCE1
216549_s_at	3.93	ring finger protein 8#TBC1 domain family, member 22B	RNF8#TBC1D22B
228044_at	3.93	chromosome 13 open reading frame 21	C13orf21
209736_at	3.92	SRY (sex determining region Y)-box 13	SOX13
215896_at	3.92		
1563719_a_at	3.91		
235854_x_at	3.90	Rho-associated, coiled-coil containing protein kinase 1	ROCK1
241236_at	3.90		

208712_at	3.89	cyclin D1	CCND1
1561255_at	3.88		
1562850_at	3.88		
221577_x_at	3.88	growth differentiation factor 15	GDF15
1559975_at	3.87	B-cell translocation gene 1, anti-proliferative	BTG1
203395_s_at	3.87	hairy and enhancer of split 1, (Drosophila)	HES1
237988_at	3.86	eukaryotic translation initiation factor 1B	EIF1B
203998_s_at	3.85	synaptotagmin I	SYT1
224398_at	3.85		
240153_at	3.82		
1561997_at	3.82		
204292_x_at	3.81	serine/threonine kinase 11	STK11
241362_at	3.81	chromosome 20 open reading frame 117	C20orf117
232830_at	3.81		
239786_at	3.79		
235062_at	3.78	PIH1 domain containing 2	PIH1D2
239051_at	3.78		
235801_at	3.77		
1552580_at	3.77		
222281_s_at	3.76		
220021_at	3.76	transmembrane channel-like 7	TMC7
1552698_at	3.74		
239696_at	3.73		
1562820_at	3.73		
218758_s_at	3.72		
240953_at	3.70		
219895_at	3.66	family with sequence similarity 70, member A	FAM70A
235318_at	3.65	fibrillin 1	FBN1
1562443_at	3.64	chromosome 6 open reading frame 213	C6orf213
1557506_a_at	3.64		
206048_at	3.63	ovo-like 2 (Drosophila)	OVOL2
203441_s_at	3.63	cadherin 2, type 1, N-cadherin (neuronal)	CDH2
228554_at	3.61		
204078_at	3.59		
1560745_at	3.59		
205391_x_at	3.55	ankyrin 1, erythrocytic	ANK1
230664_at	3.54		
237354_at	3.53		
237058_x_at	3.52	solute carrier family 6 (neurotransmitter transporter, GABA), member 13	SLC6A13
239209_at	3.52		
241089_at	3.52		
1557044_at	3.51		
225966_at	3.51		
242152_at	3.50		
208366_at	3.47	protocadherin 11 X-linked	PCDH11X
232397_at	3.47	HECT, C2 and WW domain containing E3 ubiquitin protein ligase 2	HECW2
225739_at	3.46	RAB11 family interacting protein 4 (class II)	RAB11FIP4
1563241_at	3.46		
1557826_at	3.45		
204422_s_at	3.44	fibroblast growth factor 2 (basic)	FGF2

224941_at	3.43	pregnancy-associated plasma protein A, pappalysin 1	PAPPA
1560255_at	3.42	chromosome 10 open reading frame 31	C10orf31
231599_x_at	3.42	D4, zinc and double PHD fingers family 1	DPF1
1556771_a_at	3.41		
228109_at	3.41	Ras protein-specific guanine nucleotide-releasing factor 2	RASGRF2
221118_at	3.40	polycystic kidney disease 2-like 2	PKD2L2
238049_at	3.38		
219908_at	3.37	dickkopf homolog 2 (Xenopus laevis)	DKK2
235144_at	3.35		
1561133_at	3.35		
238060_s_at	3.35	beta-1,4-N-acetyl-galactosaminyl transferase 4	B4GALNT4
240921_at	3.35		
1562698_x_at	3.33		
241088_at	3.33		
237416_at	3.32		
239666_at	3.31	pygopus homolog 2 (Drosophila)	PYGO2
219932_at	3.30	solute carrier family 27 (fatty acid transporter), member 6	SLC27A6
230683_at	3.29		
229151_at	3.29	solute carrier family 14 (urea transporter), member 1 (Kidd blood group)	SLC14A1
233146_at	3.28	kinase non-catalytic C-lobe domain (KIND) containing 1	KNDC1
233673_at	3.28		
241311_at	3.27	suppression of tumorigenicity 18 (breast carcinoma) (zinc finger protein)	ST18
1561384_a_at	3.27		
239617_at	3.27		
215296_at	3.26	CDC42 binding protein kinase alpha (DMPK-like)	CDC42BPA
217320_at	3.26		
204066_s_at	3.26	centaurin, gamma 2	CENTG2
220758_s_at	3.25	roundabout homolog 4, magic roundabout (Drosophila)	ROBO4
243637_at	3.24	Fanconi anemia, complementation group C	FANCC
1568488_at	3.24	galactosyltransferase activator	GTA
244148_at	3.24		
1570299_at	3.23		
201467_s_at	3.22	NAD(P)H dehydrogenase, quinone 1	NQO1
229659_s_at	3.21	polymeric immunoglobulin receptor	PIGR
243972_at	3.20		
242882_at	3.20	isoprenylcysteine carboxyl methyltransferase	ICMT
242037_at	3.19	aspartate beta-hydroxylase	ASPH
210084_x_at	3.19	tryptase alpha/beta 1	TPSAB1
1557486_at	3.19		
205110_s_at	3.18	fibroblast growth factor 13	FGF13
239913_at	3.17	solute carrier family 10 (sodium/bile acid cotransporter family), member 4	SLC10A4
1553232_at	3.17	family with sequence similarity 82, member A	FAM82A
240484_at	3.17		
208613_s_at	3.17	filamin B, beta (actin binding protein 278)	FLNB

242082_at	3.14	methylmalonic aciduria (cobalamin deficiency) cblB type	MMAB
240841_at	3.13	insulinoma-associated 2	INSM2
222287_at	3.13	triadin	TRDN
1552908_at	3.12	chromosome 1 open reading frame 150	C1orf150
224459_at	3.12	L-2-hydroxyglutarate dehydrogenase	L2HGDH
231515_at	3.12		
224942_at	3.11	pregnancy-associated plasma protein A, pappalysin 1	PAPPA
243858_at	3.11	steroid sulfatase (microsomal), arylsulfatase C, isozyme S	STS
219606_at	3.10	PHD finger protein 20-like 1	PHF20L1
227348_at	3.08	prolyl-tRNA synthetase (mitochondrial)(putative)	PARS2
204416_x_at	3.08	apolipoprotein C-I	APOC1
1555970_at	3.08	F-box protein 28	FBXO28
1560525_at	3.06		
201147_s_at	3.06	TIMP metallopeptidase inhibitor 3 (Sorsby fundus dystrophy, pseudoinflammatory)	TIMP3
216353_s_at	3.05	chromosome 20 open reading frame 133#ring finger protein 11B, pseudogene	C20orf133#RNF11B
208161_s_at	3.05	ATP-binding cassette, sub-family C (CFTR/MRP), member 3	ABCC3
240293_at	3.05		
216759_at	3.04	HRAS-like suppressor 2	HRASLS2
219888_at	3.02	sperm associated antigen 4	SPAG4
218186_at	3.02	RAB25, member RAS oncogene family	RAB25
1555110_a_at	3.01	kelch-like 3 (Drosophila)	KLHL3
1563513_at	3.00	synaptotagmin-like 4 (granuphilin-a)	SYTL4
208455_at	3.00	poliovirus receptor-related 1 (herpesvirus entry mediator C; nectin)	PVRL1
1553747_at	3.00		
240321_at	2.99		
205103_at	2.98	chromosome 1 open reading frame 61	C1orf61
202404_s_at	2.96	collagen, type I, alpha 2	COL1A2
215241_at	2.95	transmembrane protein 16C	TMEM16C
227475_at	2.94	forkhead box Q1	FOXQ1
1568449_at	2.94		
1556941_a_at	2.93		
242203_at	2.92		
233116_at	2.91	zinc finger protein 92 homolog (mouse)	ZFP92
1561469_at	2.91		
1556608_a_at	2.91	EH-domain containing 4	EHD4
1560279_a_at	2.90		
221756_at	2.90		
229818_at	2.89	SV2 related protein homolog (rat)	SVOP
209469_at	2.88	glycoprotein M6A	GPM6A
211021_s_at	2.88	regulator of G-protein signalling 14	RGS14
1553288_a_at	2.87		
218523_at	2.87		
221168_at	2.87	PR domain containing 13	PRDM13
227959_at	2.86		
236150_at	2.86		
233934_at	2.85		

1558945_s_at	2.85	calcium channel, voltage-dependent, P/Q type, alpha 1A subunit	CACNA1A
236698_at	2.84		
239165_at	2.84		
1560803_at	2.84	dynein, axonemal, heavy chain 3	DNAH3
207566_at	2.83	major histocompatibility complex, class I-related	MR1
210760_x_at	2.83	thyroid hormone receptor interactor 11	TRIP11
238277_at	2.83	tankyrase, TRF1-interacting ankyrin-related ADP-ribose polymerase	TNKS
1561398_at	2.83		
239187_at	2.81	chromosome 4 open reading frame 30	C4orf30
231127_at	2.81	RAP1B, member of RAS oncogene family	RAP1B
234209_at	2.80		
233689_at	2.79		
201150_s_at	2.79	TIMP metallopeptidase inhibitor 3 (Sorsby fundus dystrophy, pseudoinflammatory)	TIMP3
225254_at	2.78	coiled-coil domain containing 97	CCDC97
220001_at	2.78	peptidyl arginine deiminase, type IV	PADI4
215810_x_at	2.78		
209116_x_at	2.78	hemoglobin, beta	HBB
213953_at	2.77	keratin 20	KRT20
211696_x_at	2.76	hemoglobin, beta	HBB
207950_s_at	2.75	ankyrin 3, node of Ranvier (ankyrin G)	ANK3
214038_at	2.74	chemokine (C-C motif) ligand 8	CCL8
228504_at	2.74		
234231_at	2.73		
233857_s_at	2.73	ankyrin repeat and SOCS box-containing 2	ASB2
240078_at	2.72	splicing factor, arginine/serine-rich 8 (suppressor-of-white-apricot homolog, Drosophila)	SFRS8
239552_at	2.72		
209776_s_at	2.72	solute carrier family 19 (folate transporter), member 1	SLC19A1
1558475_at	2.72		
1552570_at	2.70	chromosome 21 open reading frame 29	C21orf29
215674_at	2.70		
239956_at	2.70		
207254_at	2.70	solute carrier family 15 (oligopeptide transporter), member 1	SLC15A1
224056_at	2.70	guanine nucleotide binding protein (G protein), alpha 13	GNA13
213348_at	2.69	cyclin-dependent kinase inhibitor 1C (p57, Kip2)	CDKN1C
233054_at	2.69	CCR4-NOT transcription complex, subunit 2	CNOT2
225308_s_at	2.69	tetratricopeptide repeat, ankyrin repeat and coiled-coil containing 1	TANC1
201110_s_at	2.69	thrombospondin 1	THBS1
206938_at	2.69	steroid-5-alpha-reductase, alpha polypeptide 2 (3-oxo-5 alpha-steroid delta 4-dehydrogenase alpha 2)	SRD5A2
1556627_at	2.68	dystrophin related protein 2	DRP2
215671_at	2.67	phosphodiesterase 4B, cAMP-specific (phosphodiesterase E4 dunce homolog, Drosophila)	PDE4B
233527_at	2.67		

1557924_s_at	2.67	alkaline phosphatase, liver/bone/kidney	ALPL
216016_at	2.67	NLR family, pyrin domain containing 3	NLRP3
227362_at	2.67	SLC2A4 regulator	SLC2A4RG
223805_at	2.66	oxysterol binding protein-like 6	OSBPL6
211791_s_at	2.66	potassium voltage-gated channel, shaker-related subfamily, beta member 2	KCNAB2
240107_at	2.63		
244620_at	2.62		
1563659_at	2.62	hect domain and RLD 6	HERC6
241618_at	2.61		
214053_at	2.61	v-erb-a erythroblastic leukemia viral oncogene homolog 4 (avian)	ERBB4
213228_at	2.60	phosphodiesterase 8B	PDE8B
238133_at	2.60		
236714_at	2.60		
214203_s_at	2.60	proline dehydrogenase (oxidase) 1	PRODH
242088_at	2.60	kelch-like 24 (Drosophila)	KLHL24
237794_at	2.59		
222776_at	2.58		
224365_s_at	2.58	tigger transposable element derived 7	TIGD7
1554619_at	2.58		
1561016_at	2.57		
216470_x_at	2.57	protease, serine, 2 (trypsin 2)	PRSS2
206385_s_at	2.56	ankyrin 3, node of Ranvier (ankyrin G)	ANK3
1558202_at	2.56		
216220_s_at	2.55	adenosine A1 receptor	ADORA1
213343_s_at	2.55	glycerophosphodiester phosphodiesterase domain containing 5	GDPD5
232930_at	2.54	dedicator of cytokinesis 1	DOCK1
233606_at	2.54		
205492_s_at	2.53	dihydropyrimidinase-like 4	DPYSL4
207125_at	2.53	zinc finger protein 225	ZNF225
230803_s_at	2.53	Rho GTPase activating protein 24	ARHGAP24
237291_at	2.52		
205463_s_at	2.52	platelet-derived growth factor alpha polypeptide	PDGFA
1556695_a_at	2.52		
231320_at	2.52	TBC1 domain family, member 25	TBC1D25
235108_at	2.52		
233945_at	2.51	UDP-glucose ceramide glucosyltransferase-like 2	UGCGL2
217263_x_at	2.51	runt-related transcription factor 1 (acute myeloid leukemia 1; aml1 oncogene)	RUNX1
241319_at	2.51	exocyst complex component 6	EXOC6
238413_at	2.49	cerebellar degeneration-related protein 2, 62kDa	CDR2
241844_x_at	2.49	transmembrane protein 156	TMEM156
217192_s_at	2.49	PR domain containing 1, with ZNF domain#ATG5 autophagy related 5 homolog (S. cerevisiae)	PRDM1#ATG5
1560226_at	2.48		
224058_s_at	2.48	hydroxysteroid (17-beta) dehydrogenase 7 pseudogene 2	HSD17B7P2
244307_s_at	2.48		
222456_s_at	2.47	LIM domain and actin binding 1	LIMA1
1561454_at	2.47		
236266_at	2.46		

231124_x_at	2.46	lymphocyte antigen 9	LY9
219522_at	2.46	four jointed box 1 (Drosophila)	FJX1
243460_at	2.46		
207714_s_at	2.45	serpin peptidase inhibitor, clade H (heat shock protein 47), member 1, (collagen binding protein 1)	SERPINH1
242348_at	2.45	family with sequence similarity 19 (chemokine (C-C motif)-like), member A4	FAM19A4
236845_at	2.44		
241935_at	2.44	shroom family member 1	SHROOM1
1559520_at	2.43	glycophorin A (MNS blood group)	GYPA
236521_at	2.42		
243136_at	2.41		
1552592_at	2.40	matrix metallopeptidase 21	MMP21
211722_s_at	2.40	histone deacetylase 6	HDAC6
243942_at	2.40		
210446_at	2.40	GATA binding protein 1 (globin transcription factor 1)	GATA1
206805_at	2.39	sema domain, immunoglobulin domain (Ig), short basic domain, secreted, (semaphorin) 3A	SEMA3A
209848_s_at	2.39	silver homolog (mouse)	SILV
205389_s_at	2.38	ankyrin 1, erythrocytic	ANK1
228924_s_at	2.37	ubiquitin-conjugating enzyme E2, J1 (UBC6 homolog, yeast)	UBE2J1
230095_at	2.37	thioredoxin-like 2	TXNL2
243322_at	2.37		
201904_s_at	2.35	CTD (carboxy-terminal domain, RNA polymerase II, polypeptide A) small phosphatase-like	CTDSPL
234475_x_at	2.35	cholecystokinin B receptor	CCKBR
1562282_at	2.34	zinc finger protein 568	ZNF568
206306_at	2.34	ryanodine receptor 3	RYR3
1570145_at	2.34	ubiquitin carboxyl-terminal hydrolase L5	UCHL5
208608_s_at	2.34	syntrophin, beta 1 (dystrophin-associated protein A1, 59kDa, basic component 1)	SNTB1
1562625_at	2.34	FRY-like	FRYL
230834_at	2.33		
1555865_at	2.33		
1554074_s_at	2.31	schlafen-like 1	SLFNL1
1569941_at	2.31		
201655_s_at	2.31	heparan sulfate proteoglycan 2 (perlecan)	HSPG2
228705_at	2.31	calpain 12	CAPN12
220422_at	2.30	ubiquilin 3	UBQLN3
1559510_at	2.30	basic helix-loop-helix domain containing, class B, 9	BHLHB9
1558711_at	2.30	family with sequence similarity 13, member A1 opposite strand	FAM13A1OS
235885_at	2.30		
202585_s_at	2.29	nuclear transcription factor, X-box binding 1	NFX1
1570301_at	2.28		
230383_x_at	2.28		
216532_x_at	2.28	PRP4 pre-mRNA processing factor 4 homolog B (yeast)#chromosome 6 open reading frame 50	PRPF4B#C6orf50
217141_at	2.28	BTB (POZ) domain containing 7	BTBD7

1561038_at	2.27	zinc finger protein 81	ZNF81
204731_at	2.27	transforming growth factor, beta receptor III (betaglycan, 300kDa)	TGFBR3
209881_s_at	2.27	linker for activation of T cells	LAT
226234_at	2.27		
223784_at	2.26	transmembrane protein 27	TMEM27
227584_at	2.26		
204310_s_at	2.26	natriuretic peptide receptor B/guanylate cyclase B (atrionatriuretic peptide receptor B)	NPR2
204660_at	2.26	growth factor, augmenter of liver regeneration (ERV1 homolog, S. cerevisiae)	GFER
1559806_at	2.25		
1567288_at	2.25	olfactory receptor, family 5, subfamily K, member 1	OR5K1
237034_at	2.25		
210534_s_at	2.25		
217232_x_at	2.24	hemoglobin, beta	HBB
209604_s_at	2.24	GATA binding protein 3	GATA3
1557818_x_at	2.24		
227192_at	2.23	chromosome 16 open reading frame 53	C16orf53
1570181_a_at	2.23		
211413_s_at	2.23	peptidyl arginine deiminase, type IV	PADI4
209803_s_at	2.23	pleckstrin homology-like domain, family A, member 2	PHLDA2
238748_at	2.22		
204848_x_at	2.22	hemoglobin, gamma A	HBG1
239288_at	2.22	TRAF2 and NCK interacting kinase	TNIK
1570257_x_at	2.21		
211959_at	2.21	insulin-like growth factor binding protein 5	IGFBP5
1555677_s_at	2.21	structural maintenance of chromosomes 1A	SMC1A
231356_at	2.21	myelin basic protein	MBP
232334_at	2.21	neurexophilin 2	NXPH2
232187_at	2.20	palmdelphin	PALMD
214010_s_at	2.20	ATPase, Class II, type 9B	ATP9B
223958_s_at	2.19	dynein, axonemal, light chain 1	DNAL1
1561918_at	2.19		
214955_at	2.18	transmembrane protease, serine 6	TMPRSS6
204250_s_at	2.18	centrosomal protein 164kDa	CEP164
1557540_at	2.18		
243723_at	2.18		
235268_at	2.18		
219672_at	2.17	erythroid associated factor	ERAF
1568903_at	2.17		
238322_s_at	2.15	TEA domain family member 2	TEAD2
215656_at	2.15	lectin, mannose-binding 2	LMAN2
1555166_a_at	2.14	zinc finger protein 396	ZNF396
207134_x_at	2.14	tryptase beta 2	TPSB2
220334_at	2.14	regulator of G-protein signalling 17	RGS17
214546_s_at	2.14	purinergic receptor P2Y, G-protein coupled, 11	P2RY11
227429_at	2.13	EF-hand calcium binding domain 4A	EFCAB4A
215382_x_at	2.13	tryptase alpha/beta 1	TPSAB1
232029_at	2.13		
229629_at	2.13		

208790_s_at	2.13	polymerase I and transcript release factor	PTRF
203280_at	2.12	scaffold attachment factor B2	SAFB2
219927_at	2.11	FCF1 small subunit (SSU) processome component homolog (S. cerevisiae)	FCF1
203662_s_at	2.11	tropomodulin 1	TMOD1
219901_at	2.11	FYVE, RhoGEF and PH domain containing 6	FGD6
204505_s_at	2.11	erythrocyte membrane protein band 4.9 (dematin)	EPB49
1566295_at	2.10	family with sequence similarity 118, member B	FAM118B
206283_s_at	2.10	T-cell acute lymphocytic leukemia 1	TAL1
228449_at	2.09	chromosome 22 open reading frame 27	C22orf27
213515_x_at	2.09	hemoglobin, gamma G	HBG2
1562342_at	2.08		
214414_x_at	2.08	hemoglobin, alpha 1	HBA1
216753_at	2.08		
210164_at	2.07	granzyme B (granzyme 2, cytotoxic T-lymphocyte-associated serine esterase 1)	GZMB
230850_at	2.07		
1567334_at	2.07		
205609_at	2.07	angiopoietin 1	ANGPT1
209361_s_at	2.07	poly(rC) binding protein 4	PCBP4
1562316_at	2.07		
224110_at	2.07		
220366_at	2.07	epididymal sperm binding protein 1	ELSPBP1
236118_at	2.06		
243260_x_at	2.06		
1566763_at	2.06		
1561339_at	2.05		
221225_at	2.05	dephospho-CoA kinase domain containing	DCAKD
220190_s_at	2.05	stonin 1	STON1
231609_at	2.05	chromosome 10 open reading frame 82	C10orf82
220234_at	2.05	carbonic anhydrase VIII	CA8
1567247_at	2.04	olfactory receptor, family 5, subfamily H, member 1	OR5H1
209031_at	2.04	cell adhesion molecule 1	CADM1
212755_at	2.04	MON2 homolog (S. cerevisiae)	MON2
204419_x_at	2.04	hemoglobin, gamma G	HBG2
228285_at	2.03	tudor domain containing 9	TDRD9
216191_s_at	2.03	T cell receptor alpha locus	TRA@
1566938_at	2.03		
220530_at	2.03		
205513_at	2.03	transcobalamin I (vitamin B12 binding protein, R binder family)	TCN1
1561037_a_at	2.02		
214950_at	2.01	interleukin 9 receptor	IL9R
205962_at	2.01	p21 (CDKN1A)-activated kinase 2	PAK2
1569371_at	2.01	leucine rich repeat containing 59	LRRC59
243983_at	0.50		
1569776_at	0.50		
243656_at	0.50		
234042_at	0.50	taste receptor, type 2, member 45	TAS2R45
218850_s_at	0.50	LIM domains containing 1	LIMD1
243387_at	0.50	mesoderm posterior 1 homolog (mouse)	MESP1
1569624_at	0.49		

242811_x_at	0.49		
208230_s_at	0.49	neuregulin 1	NRG1
223918_at	0.49	acyl-CoA synthetase long-chain family member 6	ACSL6
232196_at	0.49	chromosome 21 open reading frame 13	C21orf13
217016_x_at	0.49		
238871_at	0.49	myeloid/lymphoid or mixed-lineage leukemia (trithorax homolog, Drosophila); translocated to, 4	MLLT4
231110_at	0.49		
213942_at	0.49	multiple EGF-like-domains 6	MEGF6
228917_at	0.49		
203170_at	0.49	KIAA0409	KIAA0409
219701_at	0.49	tropomodulin 2 (neuronal)	TMOD2
203769_s_at	0.49	steroid sulfatase (microsomal), arylsulfatase C, isozyme S	STS
1562263_at	0.49	lysyl oxidase-like 2	LOXL2
222835_at	0.49	thrombospondin, type I, domain containing 4	THSD4
1553354_a_at	0.49		
1554934_at	0.49	regulator of chromosome condensation (RCC1) and BTB (POZ) domain containing protein 1	RCBTB1
234757_at	0.49		
204932_at	0.49	tumor necrosis factor receptor superfamily, member 11b (osteoprotegerin)	TNFRSF11B
1552414_at	0.49	WAP four-disulfide core domain 9	WFDC9
1563103_at	0.49		
224104_at	0.48		
206229_x_at	0.48	paired box gene 2	PAX2
1565795_at	0.48	dual oxidase 1	DUOX1
216540_at	0.48	T cell receptor alpha locus	TRA@
235212_at	0.48	chromosome 14 open reading frame 102	C14orf102
230084_at	0.48	solute carrier family 30 (zinc transporter), member 2	SLC30A2
241945_at	0.48		
1554744_at	0.48		
217353_at	0.48	phenylalanine-tRNA synthetase 2 (mitochondrial)	FARS2
1569690_at	0.48	coiled-coil domain containing 36	CCDC36
217296_at	0.48	killer cell immunoglobulin-like receptor, three domains, long cytoplasmic tail, 1	KIR3DL1
1562399_at	0.48		
220787_at	0.48		
1556528_at	0.48		
243452_at	0.48		
1554229_at	0.48		
1560282_at	0.48		
232690_at	0.48		
210870_s_at	0.48	epilepsy, progressive myoclonus type 2A, Lafora disease (laforin)	EPM2A
244171_at	0.48	muskelin 1, intracellular mediator containing kelch motifs	MKLN1
244190_at	0.48	THAP domain containing 5	THAP5
212963_at	0.47	TM2 domain containing 1	TM2D1
226809_at	0.47	chromosome 2 open reading frame 14	C2orf14
204813_at	0.47	mitogen-activated protein kinase 10	MAPK10
239395_at	0.47		

1566696_at	0.47		
244328_x_at	0.47	platelet-activating factor acetylhydrolase, isoform Ib, alpha subunit 45kDa	PAFAH1B1
1556486_at	0.47		
243064_at	0.47		
206161_s_at	0.47	synaptotagmin V	SYT5
218591_s_at	0.47	nucleolar protein 10	NOL10
235881_at	0.47	formin-like 2	FMNL2
204574_s_at	0.47	matrix metallopeptidase 19	MMP19
203134_at	0.47	phosphatidylinositol binding clathrin assembly protein	PICALM
1555024_at	0.47	ADAM metallopeptidase domain 22	ADAM22
1569316_at	0.47		
220897_at	0.47		
220033_at	0.47		
243571_at	0.47		
215956_at	0.47		
1568720_at	0.47	zinc finger protein 506	ZNF506
1554294_s_at	0.47	tau tubulin kinase 2	TTBK2
220399_at	0.47		
241992_at	0.47		
1556469_s_at	0.47		
230652_at	0.47	v-raf murine sarcoma 3611 viral oncogene homolog	ARAF
208048_at	0.46	tachykinin receptor 1	TACR1
1558494_at	0.46	cadherin 12, type 2 (N-cadherin 2)	CDH12
214691_x_at	0.46	family with sequence similarity 63, member B	FAM63B
206261_at	0.46	zinc finger protein 239	ZNF239
214294_at	0.46		
228074_at	0.46		
206101_at	0.46	extracellular matrix protein 2, female organ and adipocyte specific	ECM2
1557117_at	0.46		
241951_at	0.46		
205815_at	0.46	regenerating islet-derived 3 alpha	REG3A
1556462_a_at	0.46		
230979_at	0.46		
241653_x_at	0.46		
226122_at	0.46	methylenetetrahydrofolate dehydrogenase (NADP+ dependent) 1-like#pleckstrin homology domain containing, family G (with RhoGef domain) member 1	MTHFD1L#PLEKHG1
210195_s_at	0.46	pregnancy specific beta-1-glycoprotein 1	PSG1
1562908_at	0.46		
239556_at	0.46		
243417_at	0.46		
1570176_at	0.46		
1564257_at	0.46		
216350_s_at	0.46	zinc finger protein 10	ZNF10
236805_at	0.46	chromosome 9 open reading frame 96	C9orf96
210952_at	0.46	adaptor-related protein complex 4, sigma 1 subunit	AP4S1

1569827_at	0.46	ATG7 autophagy related 7 homolog (S. cerevisiae)	ATG7
1565825_at	0.46	lipoma HMGIC fusion partner	LHFP
1555210_at	0.46	DTW domain containing 1	DTWD1
237831_x_at	0.46	methylmalonic aciduria (cobalamin deficiency) cblA type	MMAA
1559568_at	0.46		
220158_at	0.46	lectin, galactoside-binding, soluble, 14	LGALS14
228632_at	0.46		
1566725_at	0.46		
1560416_at	0.46	dynein, axonemal, heavy chain 11	DNAH11
227771_at	0.46	leukemia inhibitory factor receptor alpha	LIFR
224103_at	0.46		
237339_at	0.45		
1559222_at	0.45		
234490_at	0.45		
1561604_at	0.45		
1568448_at	0.45		
240495_at	0.45	A kinase (PRKA) anchor protein 1	AKAP1
216967_at	0.45	growth associated protein 43	GAP43
207323_s_at	0.45	myelin basic protein	MBP
241197_at	0.45		
205116_at	0.45	laminin, alpha 2 (merosin, congenital muscular dystrophy)	LAMA2
228436_at	0.45	potassium voltage-gated channel, Shaw-related subfamily, member 4	KCNC4
1556903_at	0.45		
1554805_at	0.45	claudin 19	CLDN19
244558_at	0.45		
215798_at	0.45	aldehyde dehydrogenase 1 family, member L1	ALDH1L1
208394_x_at	0.45	endothelial cell-specific molecule 1	ESM1
241149_at	0.45		
212464_s_at	0.45	fibronectin 1	FN1
1560552_a_at	0.45		
243719_at	0.45	serine/threonine kinase 19	STK19
201006_at	0.45	peroxiredoxin 2	PRDX2
1552971_at	0.45	sarcoglycan zeta	SGCZ
1558020_at	0.45	ERGIC and golgi 3	ERGIC3
237745_at	0.45		
1564960_at	0.45	keratin associated protein 7-1	KRTAP7-1
1553364_at	0.45	patatin-like phospholipase domain containing 1	PNPLA1
1561361_at	0.45	zinc finger protein 660	ZNF660
1553296_at	0.45	G protein-coupled receptor 128	GPR128
1558019_at	0.45		
1570169_at	0.45	CUB and Sushi multiple domains 2	CSMD2
1556235_at	0.45		
240461_at	0.45	bicaudal D homolog 1 (Drosophila)	BICD1
238569_at	0.45	gamma-aminobutyric acid (GABA) B receptor, 1	GABBR1
230952_at	0.45		
1561254_at	0.44		
237673_at	0.44		
224235_at	0.44		
1563524_a_at	0.44	chromosome 14 open reading frame 85	C14orf85

219962_at	0.44	angiotensin I converting enzyme (peptidyl-dipeptidase A) 2	ACE2
214762_at	0.44	ATPase, H+ transporting, lysosomal 13kDa, V1 subunit G2	ATP6V1G2
1569834_at	0.44		
1556936_at	0.44		
211736_at	0.44	Sp2 transcription factor	SP2
1569998_at	0.44	monocyte to macrophage differentiation-associated 2	MMD2
244097_at	0.44	complement component (3d/Epstein Barr virus) receptor 2	CR2
1570490_at	0.44		
1559672_a_at	0.44	chromosome 9 open reading frame 93	C9orf93
223661_at	0.44		
1567457_at	0.44	ras-related C3 botulinum toxin substrate 1 (rho family, small GTP binding protein Rac1)	RAC1
1560493_a_at	0.44	CPX chromosome region, candidate 1	CPXCR1
1555339_at	0.44	RAP1A, member of RAS oncogene family	RAP1A
1555734_x_at	0.44	adaptor-related protein complex 1, sigma 3 subunit	AP1S3
1553373_at	0.44	WD repeat domain 64	WDR64
212942_s_at	0.44	KIAA1199	KIAA1199
1564974_at	0.44	keratin associated protein 8-1	KRTAP8-1
1559950_at	0.44		
221703_at	0.44	BRCA1 interacting protein C-terminal helicase 1	BRIP1
1562267_s_at	0.43	zinc finger protein 564	ZNF564
243934_at	0.43		
210200_at	0.43	WW domain containing E3 ubiquitin protein ligase 2	WWP2
236528_at	0.43		
234581_at	0.43		
1563800_at	0.43		
210495_x_at	0.43	fibronectin 1	FN1
1552816_at	0.43	chromosome 9 open reading frame 121	C9orf121
237365_at	0.43		
1554043_a_at	0.43		
1562168_at	0.43		
211774_s_at	0.43	methylmalonic aciduria (cobalamin deficiency) cblC type, with homocystinuria	MMACHC
204195_s_at	0.43	PBX/knotted 1 homeobox 1	PKNOX1
220545_s_at	0.43		
208404_x_at	0.43	potassium inwardly-rectifying channel, subfamily J, member 5	KCNJ5
1552540_s_at	0.43	IQ motif containing D	IQCD
217569_x_at	0.43	calcium/calmodulin-dependent protein kinase (CaM kinase) II delta	CAMK2D
1552425_a_at	0.43	kelch-like 10 (Drosophila)	KLHL10
209863_s_at	0.42	tumor protein p73-like	TP73L
1553166_at	0.42	cadherin-like 24	CDH24
1562697_at	0.42		
242901_at	0.42		
218469_at	0.42	gremlin 1, cysteine knot superfamily, homolog (Xenopus laevis)	GREM1

235736_at	0.42		
223736_at	0.42	intraflagellar transport 81 homolog (Chlamydomonas)	IFT81
1562102_at	0.42	aldo-keto reductase family 1, member C2 (dihydrodiol dehydrogenase 2; bile acid binding protein; 3-a	AKR1C2
221307_at	0.42	Kv channel interacting protein 1	KCNIP1
1552735_at	0.42	protocadherin gamma subfamily A, 4	PCDHGA4
241490_s_at	0.42	piggyBac transposable element derived 2	PGBD2
242205_at	0.42		
1562903_at	0.42		
214862_x_at	0.42		
1562386_s_at	0.42	zinc finger protein 501	ZNF501
233053_at	0.41		
219454_at	0.41	EGF-like-domain, multiple 6	EGFL6
242199_at	0.41		
240979_at	0.41		
242841_at	0.41		
1553619_a_at	0.41	tripartite motif-containing 43	TRIM43
228610_at	0.41	transmembrane 9 superfamily member 3	TM9SF3
210234_at	0.41	glutamate receptor, metabotropic 4	GRM4
1569637_at	0.41	zinc finger protein 100	ZNF100
1556232_at	0.41	kinesin family member 6	KIF6
222676_at	0.41	BAI1-associated protein 2-like 1	BAIAP2L1
231244_at	0.41	CAS1 domain containing 1	CASD1
244596_at	0.41		
1554268_at	0.41	MORN repeat containing 1	MORN1
243783_at	0.41		
1558846_at	0.41	pancreatic lipase-related protein 3	PNLIPRP3
240536_at	0.41		
231095_at	0.41		
1561626_at	0.41		
243017_at	0.41		
214379_at	0.41	heterogeneous nuclear ribonucleoprotein D-like	HNRPDL
1564338_at	0.41		
1569539_at	0.41		
239012_at	0.41	IBR domain containing 2	IBRDC2
1552587_at	0.41	cyclic nucleotide binding domain containing 1	CNBD1
1555675_at	0.41		
205286_at	0.41	transcription factor AP-2 gamma (activating enhancer binding protein 2 gamma)	TFAP2C
220910_at	0.41	Fraser syndrome 1	FRAS1
1569380_a_at	0.40		
234239_at	0.40		
223876_at	0.40	spermatogenesis associated 16	SPATA16
1560854_s_at	0.40	zinc finger protein 588	ZNF588
234208_at	0.40		
240209_at	0.40	chromosome 16 open reading frame 78	C16orf78
238221_at	0.40		
233433_at	0.40		
1560264_at	0.40		
1569962_at	0.40		
233932_at	0.40		

1562953_s_at	0.40	chromosome 4 open reading frame 12	C4orf12
216989_at	0.40	sperm adhesion molecule 1 (PH-20 hyaluronidase, zona pellucida binding)	SPAM1
237783_at	0.40	PLAC8-like 1	PLAC8L1
217128_s_at	0.40	calcium/calmodulin-dependent protein kinase IG	CAMK1G
1556609_at	0.40		
204819_at	0.40	FYVE, RhoGEF and PH domain containing 1 (faciogenital dysplasia)	FGD1
1555752_at	0.39		
221405_at	0.39		
240492_at	0.39		
241799_x_at	0.39		
1562878_at	0.39		
239816_at	0.39	polymerase (DNA-directed), delta 3, accessory subunit	POLD3
221085_at	0.39	tumor necrosis factor (ligand) superfamily, member 15	TNFSF15
233111_at	0.39		
1564148_at	0.39		
206414_s_at	0.39	development and differentiation enhancing factor 2	DDEF2
223889_at	0.39		
234190_at	0.39		
230666_at	0.39		
214368_at	0.39	RAS guanyl releasing protein 2 (calcium and DAG-regulated)	RASGRP2
238586_at	0.39	tyrosine kinase, non-receptor, 2	TNK2
229349_at	0.39	lin-28 homolog B (C. elegans)	LIN28B
220769_s_at	0.39	WD repeat domain 78	WDR78
1559053_at	0.39		
233845_at	0.39		
1558640_a_at	0.39		
213613_s_at	0.38	NAD kinase	NADK
242700_at	0.38	paraoxonase 2	PON2
1558450_at	0.38	alpha-2-macroglobulin	A2M
228503_at	0.38		
217120_s_at	0.38	cofactor required for Sp1 transcriptional activation, subunit 2, 150kDa	CRSP2
1556047_s_at	0.38	melanoma antigen family E, 1	MAGEE1
227846_at	0.38	G protein-coupled receptor 176	GPR176
241329_s_at	0.38		
1568887_at	0.38		
207665_at	0.38	ADAM metallopeptidase domain 21	ADAM21
220245_at	0.38	solute carrier family 45, member 2	SLC45A2
224022_x_at	0.38	wingless-type MMTV integration site family, member 16	WNT16
206353_at	0.38	cytochrome c oxidase subunit VIa polypeptide 2	COX6A2
220483_s_at	0.38	ring finger protein 19	RNF19
232240_at	0.38	coiled-coil domain containing 35	CCDC35
239481_at	0.38		
219739_at	0.38	ring finger protein 186	RNF186
233156_at	0.38	ribonuclease H2, subunit B	RNASEH2B
224140_at	0.38	nasopharyngeal carcinoma, down-regulated 1	NPCDR1

238368_at	0.38		
211106_at	0.38	suppressor of Ty 3 homolog (S. cerevisiae)	SUPT3H
228935_at	0.37	solute carrier family 4, sodium bicarbonate cotransporter, member 8	SLC4A8
210118_s_at	0.37	interleukin 1, alpha	IL1A
236035_at	0.37		
227816_at	0.37		
1562313_at	0.37	BCL6 co-repressor-like 2	BCORL2
238581_at	0.37	guanylate binding protein 5	GBP5
240880_at	0.37		
241770_x_at	0.37	solute carrier family 22 (organic anion/cation transporter), member 9	SLC22A9
1552596_at	0.37	growth arrest-specific 2 like 2	GAS2L2
210537_s_at	0.37	transcriptional adaptor 2 (ADA2 homolog, yeast)-like	TADA2L
238492_at	0.37		
244609_at	0.37		
242876_at	0.37	v-akt murine thymoma viral oncogene homolog 3 (protein kinase B, gamma)	AKT3
215853_at	0.37		
244269_at	0.37		
244537_at	0.37	huntingtin interacting protein 2	HIP2
223614_at	0.37	chromosome 8 open reading frame 57	C8orf57
228884_at	0.37	leucine rich repeat containing 27	LRRC27
208280_at	0.37	CMT1A duplicated region transcript 1	CDRT1
243263_at	0.37	chromosome 17 open reading frame 55	C17orf55
220460_at	0.37	solute carrier organic anion transporter family, member 1C1	SLCO1C1
240162_at	0.37		
232748_at	0.37	pregnancy-associated plasma protein A, pappalysin 1	PAPPA
1568673_s_at	0.36	ELL associated factor 2	EAF2
230578_at	0.36		
240319_at	0.36		
244604_at	0.36		
241725_at	0.36	E2F transcription factor 7	E2F7
217206_at	0.36		
220580_at	0.36	bicaudal C homolog 1 (Drosophila)	BICC1
211819_s_at	0.36	sorbin and SH3 domain containing 1	SORBS1
220562_at	0.36	cytochrome P450, family 2, subfamily W, polypeptide 1	CYP2W1
240597_at	0.36		
234184_at	0.36		
238407_at	0.36	capping protein (actin filament) muscle Z-line, alpha 1	CAPZA1
233266_at	0.36		
1555340_x_at	0.36	RAP1A, member of RAS oncogene family	RAP1A
234101_at	0.36		
204721_s_at	0.36	DnaJ (Hsp40) homolog, subfamily C, member 6	DNAJC6
220440_at	0.35	lectin, galactoside-binding, soluble, 13 (galectin 13)	LGALS13
214081_at	0.35	plexin domain containing 1	PLXDC1
217239_x_at	0.35		

234611_at	0.35		
213675_at	0.35		
1562716_at	0.35		
240101_at	0.35		
217369_at	0.35	immunoglobulin heavy constant gamma 1 (G1m marker)	IGHG1
1564757_a_at	0.35		
220771_at	0.35		
207689_at	0.35	T-box 10	TBX10
234401_at	0.35	T cell receptor alpha locus#T cell receptor delta locus#T cell receptor delta variable 3#T cell receptor delta variable 2#T cell receptor delta joining 4#T cell receptor delta joining 3#T cell receptor delta joining 2#T cell receptor delta joining 1#T cell receptor delta diversity 3#T cell receptor delta diversity 2#T cell receptor delta diversity 1#T cell receptor delta constant#T cell receptor alpha variable 41#T cell receptor alpha variable 40#T cell receptor alpha variable 39#T cell receptor alpha variable 38-2/delta variable 8#T cell receptor alpha variable 38-1#T cell receptor alpha variable 37#T cell receptor alpha joining 61#T cell receptor alpha joining 60#T cell receptor alpha joining 59#T cell receptor alpha joining 58#T cell receptor alpha joining 57#T cell receptor alpha joining 56#T cell receptor alpha joining 55#T cell receptor alpha joining 54#T cell receptor alpha joining 53#T cell receptor alpha joining 52#T cell receptor alpha joining 51#T cell receptor alpha joining 50#T cell receptor alpha joining 49#T cell receptor alpha joining 48#T cell receptor alpha joining 47#T cell receptor alpha joining 46	TRA@#TRD@#TRDV3#TRDV2#TRDJ4#TRDJ3#TRDJ2#TRDJ1#TRDD3#TRDD2#TRDD1#TRDC#TRAV41#TRAV40#TRAV39#TRAV38-2DV8#TRAV38-1#TRAV37#TRAJ61#TRAJ60#TRAJ59#TRAJ58#TRAJ57#TRAJ56#TRAJ55#TRAJ54#TRAJ53#TRAJ52#TRAJ51#TRAJ50#TRAJ49#TRAJ48#TRAJ47#TRAJ46
242445_at	0.35	FYVE, RhoGEF and PH domain containing 4	FGD4
222927_s_at	0.35	complexin 3	CPLX3
215263_at	0.34	zinc finger, X-linked, duplicated A	ZXDA
204665_at	0.34		
236263_at	0.34	sonic hedgehog homolog (Drosophila)	SHH
1564854_at	0.34		
238282_at	0.34		
207466_at	0.34	galanin	GAL
238500_at	0.34	epithelial membrane protein 2	EMP2
239158_at	0.34		
211422_at	0.34	transient receptor potential cation channel, subfamily M, member 3	TRPM3
229260_at	0.34	chromosome 5 open reading frame 15	C5orf15
214105_at	0.34	suppressor of cytokine signaling 3	SOCS3
234548_at	0.34		
1555289_at	0.34	leucine zipper transcription regulator 2	LZTR2

216065_at	0.34	collagen, type XI, alpha 2#ring finger protein 1#ribosomal protein S18#retinoid X receptor, beta#vacuolar protein sorting 52 homolog (S. cerevisiae)#solute carrier family 39 (zinc transporter), member 7#hydroxysteroid (17-beta) dehydrogenase 8#UDP-Gal:betaGlcNAc beta 1,3-galactosyltransferase, polypeptide 4#WD repeat domain 46#null#major histocompatibility complex, class II, DP alpha 3 (pseudogene)	COL11A2#RING1#RPS18#RXRB#VPS52#SLC39A7#HSD17B8#B3GALT4#WDR46#null#HLA-DPA3
1570465_at	0.34		
215626_at	0.34		
1565838_at	0.34		
1557312_at	0.34	chromosome 12 open reading frame 61	C12orf61
240204_at	0.34	small nuclear ribonucleoprotein polypeptide N	SNRPN
231367_s_at	0.34		
205941_s_at	0.34	collagen, type X, alpha 1(Schmid metaphyseal chondrodysplasia)	COL10A1
218820_at	0.34	chromosome 14 open reading frame 132	C14orf132
239144_at	0.34	beta-1,3-glucuronyltransferase 2 (glucuronosyltransferase S)	B3GAT2
244475_at	0.34		
1561526_at	0.34		
230253_at	0.34	signal peptide, CUB domain, EGF-like 3	SCUBE3
217376_at	0.34	signal-regulatory protein gamma	SIRPG
235565_at	0.34	zinc finger protein 425	ZNF425
241644_at	0.34	bolA homolog 2 (E. coli)	BOLA2
238503_at	0.34		
1562789_at	0.34	zinc finger protein 229	ZNF229
211514_at	0.34	receptor interacting protein kinase 5	RIPK5
216135_at	0.34	IQ motif containing K	IQCK
238441_at	0.34		
212730_at	0.34	desmuslin	DMN
202289_s_at	0.33	transforming, acidic coiled-coil containing protein 2	TACC2
1556294_at	0.33		
1562923_at	0.33		
1553561_at	0.33	taste receptor, type 2, member 50	TAS2R50
210626_at	0.33	A kinase (PRKA) anchor protein 1	AKAP1
214428_x_at	0.33	complement component 4A (Rodgers blood group)	C4A
1562116_at	0.33		
1553523_at	0.33	NLR family, pyrin domain containing 14	NLRP14
234108_at	0.33	taste receptor, type 2, member 45	TAS2R45
1569786_at	0.33		
243211_at	0.33		
234094_x_at	0.33		
233833_at	0.33		
215770_at	0.33	olfactory receptor, family 7, subfamily E, member 2 pseudogene	OR7E2P
243590_at	0.33		
235732_at	0.33		
221442_at	0.33	melanocortin 3 receptor	MC3R
231385_at	0.33	developmental pluripotency associated 3	DPPA3

214523_at	0.33	CCAAT/enhancer binding protein (C/EBP), epsilon	CEBPE
205100_at	0.33	glutamine-fructose-6-phosphate transaminase 2	GFPT2
229352_at	0.33	NADPH oxidase, EF-hand calcium binding domain 5	NOX5
205699_at	0.33	mitogen-activated protein kinase kinase 6	MAP2K6
1556758_at	0.33		
239279_at	0.33		
221126_at	0.33		
233282_at	0.33		
209713_s_at	0.33	solute carrier family 35 (UDP-glucuronic acid/UDP-N-acetylgalactosamine dual transporter), member D1	SLC35D1
241292_at	0.33		
214245_at	0.33	ribosomal protein S14	RPS14
233187_s_at	0.33		
228933_at	0.33	Nance-Horan syndrome (congenital cataracts and dental anomalies)	NHS
224007_at	0.33	heat shock transcription factor, Y-linked 1	HSFY1
1559538_at	0.33		
237996_at	0.32		
1558977_at	0.32		
239984_at	0.32	sodium channel, voltage-gated, type VII, alpha	SCN7A
1563118_at	0.32		
210380_s_at	0.32	calcium channel, voltage-dependent, alpha 1G subunit	CACNA1G
241700_at	0.32	zinc finger homeodomain 4	ZFHX4
235938_at	0.32		
240487_at	0.32		
210138_at	0.32	regulator of G-protein signalling 20	RGS20
1566740_at	0.32	phospholipase C, epsilon 1	PLCE1
241994_at	0.32	xanthine dehydrogenase	XDH
1556175_at	0.32		
1556905_at	0.32	zinc finger protein 577	ZNF577
231586_at	0.32		
236933_at	0.32		
234123_at	0.32		
242913_at	0.32	chloride intracellular channel 6	CLIC6
239030_at	0.32	septin 9	9-Sep
241193_at	0.32	v-ets erythroblastosis virus E26 oncogene homolog 2 (avian)	ETS2
206248_at	0.32	protein kinase C, epsilon	PRKCE
244504_x_at	0.32	ADP-ribosylation factor 1	ARF1
244276_at	0.32	klotho beta	KLB
1558964_at	0.32	FAT tumor suppressor homolog 3 (Drosophila)	FAT3
217430_x_at	0.31	collagen, type I, alpha 1	COL1A1
242849_at	0.31	SAM and SH3 domain containing 1	SASH1
243658_at	0.31	farnesyl-diphosphate farnesyltransferase 1	FDFT1
1556801_at	0.31		
236130_at	0.31	small nucleolar RNA, H/ACA box 37	SNORA37
203798_s_at	0.31	visinin-like 1	VSNL1
237516_at	0.31		
1553797_a_at	0.31		
1554672_at	0.31	tetratricopeptide repeat domain 26	TTC26

222184_at	0.31		
239753_at	0.31		
1556488_s_at	0.31	chromosome 3 open reading frame 15	C3orf15
222219_s_at	0.31	transducin-like enhancer of split 2 (E(sp1) homolog, Drosophila)#transducin-like enhancer of split 6 (E(sp1) homolog, Drosophila)	TLE2#TLE6
1561232_at	0.31		
244503_at	0.31	brain-derived neurotrophic factor	BDNF
1564963_x_at	0.31	zinc finger protein 92	ZNF92
232752_at	0.31		
220186_s_at	0.31		
244404_at	0.31		
240696_at	0.31		
237043_at	0.31		
235617_x_at	0.31		
232193_at	0.31	glutathione S-transferase theta 1	GSTT1
217579_x_at	0.31		
239850_at	0.31		
240501_at	0.30		
239058_at	0.30		
234667_at	0.30		
220655_at	0.30	TNFAIP3 interacting protein 3	TNIP3
206538_at	0.30	muscle RAS oncogene homolog	MRAS
216466_at	0.30	neuron navigator 3	NAV3
1561213_at	0.30		
1563061_at	0.30		
240651_at	0.30		
1554957_at	0.30		
237099_at	0.30	chromosome 20 open reading frame 70	C20orf70
233488_at	0.30	ribonuclease, RNase A family, 7	RNASE7
241898_at	0.30		
240820_at	0.30		
1557557_at	0.30		
215445_x_at	0.30		
1556963_at	0.30		
241606_s_at	0.30	TruB pseudouridine (psi) synthase homolog 1 (E. coli)	TRUB1
242269_at	0.30		
234669_x_at	0.30		
207207_at	0.30		
216712_at	0.30	transmembrane protein 132A	TMEM132A
1559093_at	0.30		
215801_at	0.30		
1562388_at	0.30		
1567390_at	0.30		
1570163_at	0.30		
234090_at	0.30		
237284_at	0.30	DnaJ (Hsp40) homolog, subfamily B, member 8	DNAJB8
1558706_a_at	0.30	atonal homolog 8 (Drosophila)	ATOH8
1555123_at	0.29	ST6 beta-galactosamide alpha-2,6-sialyltranferase 2	ST6GAL2
1564765_at	0.29		
236762_at	0.29	ATPase, Class II, type 9B	ATP9B

243939_at	0.29		
239152_at	0.29	influenza virus NS1A binding protein	IVNS1ABP
228618_at	0.29		
1557057_a_at	0.29		
216133_at	0.29	T cell receptor alpha locus	TRA@
242299_at	0.29		
233043_at	0.29		
1567031_at	0.29	zinc finger protein 160	ZNF160
1566087_at	0.29		
1559462_at	0.29		
240720_at	0.29		
1560765_a_at	0.29	Rho GTPase activating protein 22	ARHGAP22
244143_at	0.29		
205343_at	0.29	sulfotransferase family, cytosolic, 1C, member 1	SULT1C1
211719_x_at	0.29	fibronectin 1	FN1
241636_x_at	0.29		
241555_at	0.29		
207505_at	0.29	protein kinase, cGMP-dependent, type II	PRKG2
222301_at	0.29	chromosome 1 open reading frame 61	C1orf61
1561114_a_at	0.29	DEP domain containing 4	DEPDC4
207201_s_at	0.29	solute carrier family 22 (organic cation transporter), member 1	SLC22A1
234095_at	0.29		
236311_at	0.28	loss of heterozygosity, 12, chromosomal region 2	LOH12CR2
217030_at	0.28		
233048_at	0.28	family with sequence similarity 35, member A	FAM35A
205485_at	0.28	ryanodine receptor 1 (skeletal)	RYR1
231327_at	0.28		
243551_at	0.28		
221023_s_at	0.28	potassium voltage-gated channel, subfamily H (eag-related), member 6	KCNH6
1570138_at	0.28		
1570534_a_at	0.28	zinc finger protein 483	ZNF483
214935_at	0.28	nucleoporin 62kDa	NUP62
1559656_a_at	0.28		
241060_x_at	0.28	tetraspanin 5	TSPAN5
1553024_at	0.28		
232477_at	0.28		
213877_x_at	0.28	transcription elongation factor B (SIII), polypeptide 2 (18kDa, elongin B)	TCEB2
240160_x_at	0.28		
212865_s_at	0.27	collagen, type XIV, alpha 1 (undulin)	COL14A1
237622_at	0.27		
1553504_at	0.27	MAS-related GPR, member X4	MRGPRX4
238318_at	0.27		
240565_at	0.27	zinc finger protein 28 homolog (mouse)	ZFP28
1566776_at	0.27	dynein, axonemal, heavy chain 1	DNAH1
207096_at	0.27	serum amyloid A4, constitutive	SAA4
237696_at	0.27		
1561179_s_at	0.27		
1569858_at	0.27		
227752_at	0.27	serine palmitoyltransferase, long chain base subunit 3	SPTLC3

1558537_x_at	0.27		
1555074_a_at	0.27	potassium voltage-gated channel, subfamily H (eag-related), member 5	KCNH5
202008_s_at	0.27	nidogen 1	NID1
215817_at	0.27	serpin peptidase inhibitor, clade B (ovalbumin), member 13	SERPINB13
241035_s_at	0.27		
214135_at	0.27	claudin 18	CLDN18
231046_at	0.27		
1562860_at	0.27		
220835_s_at	0.27	zinc finger protein 407	ZNF407
1560142_at	0.27	glutamate receptor, ionotropic, kainate 2	GRIK2
216774_at	0.26		
1560372_at	0.26		
237153_at	0.26		
205230_at	0.26	rabphilin 3A homolog (mouse)	RPH3A
238111_at	0.26	serologically defined colon cancer antigen 3	SDCCAG3
219945_at	0.26	DEAD (Asp-Glu-Ala-Asp) box polypeptide 25	DDX25
1558682_at	0.26	high mobility group AT-hook 2	HMGA2
1554809_at	0.26		
237069_s_at	0.26	transient receptor potential cation channel, subfamily M, member 1	TRPM1
205350_at	0.26	cellular retinoic acid binding protein 1	CRABP1
1556221_a_at	0.26		
230923_at	0.26	family with sequence similarity 19 (chemokine (C-C motif)-like), member A1	FAM19A1
235892_at	0.26		
211078_s_at	0.25	serine/threonine kinase 3 (STE20 homolog, yeast)	STK3
209951_s_at	0.25	mitogen-activated protein kinase kinase 7	MAP2K7
232640_at	0.25	COMM domain containing 5	COMMD5
1552919_at	0.25	chromosome 4 open reading frame 36	C4orf36
206415_at	0.25	tolloid-like 1	TLL1
242188_at	0.25	protein tyrosine phosphatase, receptor type, G	PTPRG
217102_at	0.25		
1561090_at	0.25		
242478_at	0.25		
231306_at	0.25	lysozyme-like 4	LYZL4
1555854_at	0.25		
219845_at	0.25	BarH-like homeobox 1	BARX1
226647_at	0.25	transmembrane protein 25	TMEM25
1557793_at	0.25	family with sequence similarity 62 (C2 domain containing), member C	FAM62C
201905_s_at	0.25	CTD (carboxy-terminal domain, RNA polymerase II, polypeptide A) small phosphatase-like	CTDSPL
1565801_at	0.24		
227842_at	0.24	RAB30, member RAS oncogene family	RAB30
237395_at	0.24	cytochrome P450, family 4, subfamily Z, polypeptide 1	CYP4Z1
220749_at	0.24	chromosome 10 open reading frame 68	C10orf68
229675_at	0.24	MYC induced nuclear antigen	MINA
1564007_at	0.24		
1569780_at	0.24		
214837_at	0.24	albumin	ALB

206948_at	0.24	sialidase 3 (membrane sialidase)	NEU3
244673_at	0.24	KIAA1841	KIAA1841
231031_at	0.24		
216073_at	0.24		
203873_at	0.24	SWI/SNF related, matrix associated, actin dependent regulator of chromatin, subfamily a, member 1	SMARCA1
1552906_at	0.24	fragile X mental retardation 1 neighbor	FMR1NB
221349_at	0.24	pre-B lymphocyte gene 1	VPREB1
243161_x_at	0.24	zinc finger protein 42 homolog (mouse)	ZFP42
203875_at	0.24	SWI/SNF related, matrix associated, actin dependent regulator of chromatin, subfamily a, member 1	SMARCA1
205590_at	0.24	RAS guanyl releasing protein 1 (calcium and DAG-regulated)	RASGRP1
219114_at	0.24	chromosome 3 open reading frame 18	C3orf18
1568634_a_at	0.24		
240085_at	0.24		
1564449_at	0.24		
1564630_at	0.24	endothelin 1	EDN1
236881_at	0.24		
1559163_at	0.24		
220908_at	0.24	coiled-coil domain containing 33	CCDC33
220826_at	0.24	chromosome 21 open reading frame 77	C21orf77
215783_s_at	0.24	alkaline phosphatase, liver/bone/kidney	ALPL
216341_s_at	0.24	gonadotropin-releasing hormone receptor	GNRHR
1561289_at	0.24		
244465_at	0.23		
233389_at	0.23	chromosome 20 open reading frame 26	C20orf26
1556730_at	0.23		
221309_at	0.23	RNA binding motif protein 17	RBM17
1561407_at	0.23	centaurin, delta 1	CENTD1
240201_at	0.23		
224050_s_at	0.23		
232060_at	0.23		
208430_s_at	0.23	dystrobrevin, alpha	DTNA
220639_at	0.23	transmembrane 4 L six family member 20	TM4SF20
1558387_at	0.23		
219831_at	0.23	cyclin-dependent kinase-like 3	CDKL3
1552400_a_at	0.23	chromosome 15 open reading frame 27	C15orf27
222720_x_at	0.23	chromosome 1 open reading frame 27	C1orf27
1557311_at	0.23		
1570160_at	0.23		
230378_at	0.23	secretoglobin, family 3A, member 1	SCGB3A1
205691_at	0.23	synaptogyrin 3	SYNGR3
234765_at	0.23		
219954_s_at	0.23	glucosidase, beta, acid 3 (cytosolic)	GBA3
1557775_a_at	0.23		
223185_s_at	0.23	basic helix-loop-helix domain containing, class B, 3	BHLHB3
206733_at	0.23	tubby like protein 2	TULP2
203498_at	0.23	Down syndrome critical region gene 1-like 1	DSCR1L1
1563038_at	0.22		

205772_s_at	0.22	A kinase (PRKA) anchor protein 7	AKAP7
223963_s_at	0.22	insulin-like growth factor 2 mRNA binding protein 2	IGF2BP2
241547_at	0.22		
208557_at	0.22	homeobox A6	HOXA6
1555912_at	0.22	ST7 overlapping transcript 1 (antisense non-coding RNA)	ST7OT1
1562879_at	0.22		
242880_at	0.22	voltage gated channel like 1	VGCNL1
1555925_at	0.22		
207703_at	0.22	neuroligin 4, Y-linked	NLGN4Y
241174_at	0.22	adaptor-related protein complex 4, epsilon 1 subunit	AP4E1
1560359_at	0.22	pelota homolog (Drosophila)	PELO
232694_at	0.22	zinc finger protein 395	ZNF395
213832_at	0.22		
1560049_at	0.22		
1563961_at	0.22	forkhead-associated (FHA) phosphopeptide binding domain 1	FHAD1
209016_s_at	0.22	keratin 7	KRT7
1561679_at	0.22		
242426_at	0.22	neuregulin 4	NRG4
206408_at	0.21	leucine rich repeat transmembrane neuronal 2	LRRTM2
209869_at	0.21	adrenergic, alpha-2A-, receptor	ADRA2A
206721_at	0.21	chromosome 1 open reading frame 114	C1orf114
237883_at	0.21		
205994_at	0.21	ELK4, ETS-domain protein (SRF accessory protein 1)	ELK4
231546_at	0.21	scavenger receptor class A, member 5 (putative)	SCARA5
236827_at	0.21		
222940_at	0.21	sulfotransferase family 1E, estrogen-preferring, member 1	SULT1E1
214320_x_at	0.21	cytochrome P450, family 2, subfamily A, polypeptide 7	CYP2A7
1569072_s_at	0.21	ATP-binding cassette, sub-family B (MDR/TAP), member 5	ABCB5
206344_at	0.21	paraoxonase 1	PON1
1554929_at	0.21		
215539_at	0.21		
242043_s_at	0.21		
243635_at	0.20		
1552564_at	0.20	nudix (nucleoside diphosphate linked moiety X)-type motif 9 pseudogene 1	NUDT9P1
220898_at	0.20		
1552715_a_at	0.20	relaxin/insulin-like family peptide receptor 1	RXFP1
238577_s_at	0.20		
221312_at	0.20	glucagon-like peptide 2 receptor	GLP2R
221609_s_at	0.20	wingless-type MMTV integration site family, member 6	WNT6
1559129_a_at	0.20		
1566251_at	0.20	SH3-domain GRB2-like pseudogene 1	SH3GLP1
1564469_at	0.20	leiomodin 3 (fetal)	LMOD3
1552765_x_at	0.20	transmembrane protein 67	TMEM67

237675_at	0.20		
227885_at	0.20		
206772_at	0.20	parathyroid hormone receptor 2	PTHR2
1569673_at	0.20		
217292_at	0.20		
243324_x_at	0.20		
237795_s_at	0.20		
240138_at	0.20		
232871_at	0.20		
229277_at	0.20		
216290_x_at	0.20		
242697_at	0.20	zinc finger protein 540	ZNF540
1561652_at	0.20	beclin 1 (coiled-coil, myosin-like BCL2 interacting protein)	BECN1
214557_at	0.19	pituitary tumor-transforming 2	PTTG2
1557724_a_at	0.19	FK506 binding protein 5	FKBP5
1563539_at	0.19		
226487_at	0.19	chromosome 12 open reading frame 34	C12orf34
205669_at	0.19	neural cell adhesion molecule 2	NCAM2
214956_at	0.19		
216529_at	0.19		
224083_s_at	0.19		
1568931_at	0.19		
1562775_at	0.19	nudix (nucleoside diphosphate linked moiety X)-type motif 12	NUDT12
207815_at	0.19	platelet factor 4 variant 1	PF4V1
215494_at	0.19		
1569405_at	0.18		
215871_at	0.18	phospholipase A2, group V#phospholipase A2, group IID#phospholipase A2, group IIF	PLA2G5#PLA2G2D#PLA2G2F
217505_at	0.18	kelch-like 23 (Drosophila)	KLHL23
223137_at	0.18	zinc finger, DHHC-type containing 4	ZDHHC4
220830_at	0.18	interphotoreceptor matrix proteoglycan 2	IMPG2
1566424_at	0.18		
217670_at	0.18	tetraspanin 4	TSPAN4
229656_s_at	0.18		
237458_at	0.18		
219685_at	0.18	transmembrane protein 35	TMEM35
1556026_at	0.18	iduronate 2-sulfatase (Hunter syndrome)	IDS
203868_s_at	0.18	vascular cell adhesion molecule 1	VCAM1
234534_at	0.18		
244032_at	0.18		
233683_at	0.18		
1563484_at	0.18		
203083_at	0.18	thrombospondin 2	THBS2
231654_s_at	0.18		
229247_at	0.18		
238372_s_at	0.18		
234896_at	0.18		
230419_at	0.18		
235834_at	0.17	caldesmon 1	CALD1
1561877_at	0.17		
1558308_at	0.17		

1565662_at	0.17	mucin 6, oligomeric mucus/gel-forming	MUC6
210309_at	0.17	RecQ protein-like 5	RECQL5
234789_at	0.17		
205501_at	0.17		
1568920_at	0.17		
215459_at	0.17	Tax1 (human T-cell leukemia virus type I) binding protein 3	TAX1BP3
217316_at	0.17	olfactory receptor, family 7, subfamily A, member 17#olfactory receptor, family 7, subfamily A, member 5#olfactory receptor, family 7, subfamily C, member 1#olfactory receptor, family 7, subfamily A, member 10	OR7A17#OR7A5#OR7C1#OR7A10
230663_at	0.17		
237920_at	0.17	synaptonemal complex protein 2	SYCP2
216214_at	0.17		
204519_s_at	0.17	plasma membrane proteolipid (plasmolipin)	PLLP
219393_s_at	0.17	v-akt murine thymoma viral oncogene homolog 3 (protein kinase B, gamma)	AKT3
239153_at	0.17		
237128_at	0.17		
1564392_at	0.17	chromosome 21 open reading frame 131	C21orf131
225571_at	0.17	leukemia inhibitory factor receptor alpha	LIFR
1561606_at	0.17		
1559283_a_at	0.16	canopy 1 homolog (zebrafish)	CNPY1
223529_at	0.16	synaptotagmin IV	SYT4
1564767_at	0.16		
243351_at	0.16		
1565228_s_at	0.16	albumin	ALB
204944_at	0.16	protein tyrosine phosphatase, receptor type, G	PTPRG
219795_at	0.16	solute carrier family 6 (amino acid transporter), member 14	SLC6A14
242107_x_at	0.16		
1566656_a_at	0.16		
1558867_at	0.16	dermatan sulfate epimerase	DSE
1555492_a_at	0.16	bestrophin 3	BEST3
1553534_at	0.16	NLR family, pyrin domain containing 10	NLRP10
229623_at	0.16		
242358_at	0.16		
238262_at	0.16	speedy homolog A (Drosophila)	SPDYA
1555103_s_at	0.16	fibroblast growth factor 7 (keratinocyte growth factor)	FGF7
216443_at	0.16		
1561540_at	0.16		
232771_at	0.16	serpin peptidase inhibitor, clade A (alpha-1 antiproteinase, antitrypsin), member 7	SERPINA7
213663_s_at	0.16		
229308_at	0.16		
1558163_at	0.15	peroxisome biogenesis factor 13	PEX13
241767_at	0.15		
222783_s_at	0.15	SPARC related modular calcium binding 1	SMOC1
229546_at	0.15		
225806_at	0.15	jub, ajuba homolog (Xenopus laevis)	JUB
230186_at	0.15	transmembrane protein 136	TMEM136

1561450_at	0.15		
1561281_a_at	0.15		
208389_s_at	0.15	solute carrier family 1 (glial high affinity glutamate transporter), member 2	SLC1A2
224213_at	0.15	chromosome 14 open reading frame 91	C14orf91
1570398_at	0.15		
1557017_at	0.14		
1564533_at	0.14		
1569730_at	0.14		
222168_at	0.14		
237648_x_at	0.14		
233705_at	0.13	protein kinase C and casein kinase substrate in neurons 2	PACSIN2
1561292_at	0.13		
234174_at	0.13		
235401_s_at	0.13	Fc receptor-like A	FCRLA
1553564_at	0.13	chromosome 20 open reading frame 133	C20orf133
220824_at	0.13		
1552858_at	0.13	melanoma antigen family B, 6	MAGEB6
1560819_a_at	0.13		
1560954_at	0.13		
214515_at	0.13	olfactory receptor, family 1, subfamily E, member 1	OR1E1
1561343_a_at	0.12		
224181_at	0.12	chromosome 18 open reading frame 2	C18orf2
227660_at	0.12	anthrax toxin receptor 1	ANTXR1
210292_s_at	0.12	protocadherin 11 X-linked	PCDH11X
1552991_at	0.12	olfactory receptor, family 5, subfamily P, member 2	OR5P2
1560251_at	0.12		
229505_at	0.12	nucleosome assembly protein 1-like 4	NAP1L4
213436_at	0.12	cannabinoid receptor 1 (brain)	CNR1
244624_at	0.12	ribosomal protein S27a	RPS27A
240330_at	0.12		
208508_s_at	0.11	olfactory receptor, family 2, subfamily J, member 2	OR2J2
1556069_s_at	0.11	hypoxia inducible factor 3, alpha subunit	HIF3A
235979_at	0.11	complement component 7	C7
1569934_at	0.11	DEP domain containing 2	DEPDC2
215118_s_at	0.11	immunoglobulin heavy constant alpha 1	IGHA1
1561362_at	0.11		
1558881_at	0.11		
1552604_at	0.11	chromosome 21 open reading frame 74	C21orf74
244645_at	0.10	collagen, type XIV, alpha 1 (undulin)	COL14A1
201510_at	0.10	E74-like factor 3 (ets domain transcription factor, epithelial-specific)	ELF3
233045_at	0.10		
231580_at	0.10		
239072_at	0.10		
1557398_at	0.10		
1565668_at	0.10		
1563933_a_at	0.10	phospholipase D family, member 5	PLD5
1555135_at	0.09		
242102_at	0.09		
206201_s_at	0.09	mesenchyme homeobox 2	MEOX2

244608_at	0.09		
1554288_at	0.09	KIAA1600	KIAA1600
241500_at	0.09		
216771_at	0.09		
1553811_at	0.08		
243998_at	0.08	keratin 222 pseudogene	KRT222P
1566176_at	0.08		
216408_at	0.08	olfactory receptor, family 2, subfamily B, member 2	OR2B2
1556706_at	0.08		
238392_at	0.08	translocation associated membrane protein 2	TRAM2
236740_at	0.07		
211032_at	0.07	COBL-like 1	COBLL1
1552745_at	0.06	solute carrier organic anion transporter family, member 6A1	SLCO6A1
1553887_at	0.06		
237770_at	0.06		
1555538_s_at	0.06	family with sequence similarity 9, member B	FAM9B
214967_at	0.06		
241487_at	0.06		
222259_s_at	0.05	RAE1 RNA export 1 homolog (S. pombe)#SPO11 meiotic protein covalently bound to DSB homolog (S. cerevisiae)	RAE1#SPO11
1555385_at	0.05	beta-1,4-N-acetyl-galactosaminyl transferase 1	B4GALNT1
1553298_at	0.05	chromosome 17 open reading frame 77	C17orf77
1562477_at	0.05	early B-cell factor 2	EBF2
216441_at	0.05		
1554492_at	0.04	thyroid adenoma associated	THADA
205433_at	0.04	butyrylcholinesterase	BCHE
224094_at	0.03		
234097_s_at	0.03	chromosome 6 open reading frame 12	C6orf12
219949_at	0.03	leucine rich repeat containing 2	LRRC2
