# Supplementary material for: In Vitro Transformation of Primary Human CD34+ Cells by AML Fusion Oncogenes: Early Gene Expression Profiling Reveals Possible Drug Target in AML
Source: PLoS One. 2010 Aug 27;5(8):e12464. doi: 10.1371/journal.pone.0012464 (PMC2929205; doi:10.1371/journal.pone.0012464)
Supplement: Table S19 — Genes deregulated by MLL-AF9 6 h after transfection. Primary human CD34+ cells were nucleofected with either control pTracer-CMV/Bsd vector or vector expressing MLL-AF9 and sorted for GFP positivity. Total RNA was extracted 6 h after nucleofection and subjected to microarray analysis. Microarray data were analyzed by SAM as described in Materials and Methods. Significantly deregulated genes are listed and the false discovery rate (FDR) is shown. (0.26 MB PDF) [file pone.0012464.s019.pdf]

Table S19. Genes deregulated by MLL-AF9 at 6 h detected by SAM

FDR = 4.06%

Gene ID	Fold Change	Gene Name	Gene Symbol
229935_s_at	40.91	myeloid/lymphoid or mixed-lineage leukemia (trithorax homolog, Drosophila)	MLL
1565436_s_at	33.05	myeloid/lymphoid or mixed-lineage leukemia (trithorax homolog, Drosophila)	MLL
243644_at	23.05		
1553228_at	18.56	coiled-coil domain containing 89	CCDC89
1553851_at	17.95	Spi-C transcription factor (Spi-1/PU.1 related)	SPIC
235183_at	16.21		
220785_at	15.97	urotensin 2	UTS2
243744_at	14.71		
211840_s_at	12.84	phosphodiesterase 4D, cAMP-specific (phosphodiesterase E3 dunce homolog, Drosophila)	PDE4D
1565877_at	12.80		
1564940_at	12.42		
242689_at	12.37	Ral GEF with PH domain and SH3 binding motif 1	RALGPS1
244009_at	12.01	calcium modulating ligand	CAMLG
231560_at	11.85	leucine rich repeat containing 34	LRRC34
206363_at	11.83	v-maf musculoaponeurotic fibrosarcoma oncogene homolog (avian)	MAF
1559268_at	11.62	ADAM metallopeptidase domain 23	ADAM23
228347_at	11.57	sine oculis homeobox homolog 1 (Drosophila)	SIX1
222202_at	11.48		
1558636_s_at	11.35	ADAM metallopeptidase with thrombospondin type 1 motif, 5 (aggrecanase-2)	ADAMTS5
1563833_at	11.24		
234847_at	11.19		
214614_at	10.82	homeobox HB9	HLXB9
1556609_at	10.69		
213435_at	10.43	SATB family member 2	SATB2
234289_x_at	10.34		
1568673_s_at	10.19	ELL associated factor 2	EAF2
223642_at	9.73	Zic family member 2 (odd-paired homolog, Drosophila)	ZIC2
236925_at	9.56		
239947_at	9.54		
227006_at	9.49	protein phosphatase 1, regulatory (inhibitor) subunit 14A	PPP1R14A
1560746_at	9.42	chromosome 1 open reading frame 118	C1orf118
236491_at	9.42	BCL2-like 10 (apoptosis facilitator)	BCL2L10
1569406_at	9.32		
234382_x_at	8.79		
1557343_at	8.67	dual specificity phosphatase 16	DUSP16
243378_at	8.65		
232108_at	8.41	secernin 3	SCRN3

214884_at	8.38	coagulation factor IX (plasma thromboplastic component, Christmas disease, hemophilia B)#MCF.2 cell line derived transforming sequence	F9#MCF2
220719_at	8.35		
235795_at	8.24	paired box gene 6 (aniridia, keratitis)	PAX6
224119_at	8.13		
223279_s_at	8.09	uveal autoantigen with coiled-coil domains and ankyrin repeats	UACA
230700_at	8.04	reticulon 4 receptor-like 1	RTN4RL1
1565614_at	7.99	zinc finger protein 337	ZNF337
1558565_at	7.94		
215423_at	7.93		
211131_s_at	7.93	ectodysplasin A	EDA
1553645_at	7.91		
1561053_at	7.88		
234517_at	7.87		
235490_at	7.76	transmembrane protein 107	TMEM107
240278_at	7.68	Ras association (RalGDS/AF-6) domain family 1	RASSF1
208182_x_at	7.66	interferon, alpha 14	IFNA14
206336_at	7.58	chemokine (C-X-C motif) ligand 6 (granulocyte chemotactic protein 2)	CXCL6
240196_at	7.54		
1561200_at	7.51		
221805_at	7.43	neurofilament, light polypeptide 68kDa	NEFL
210355_at	7.43	parathyroid hormone-like hormone	PTHLH
218559_s_at	7.37	v-maf musculoaponeurotic fibrosarcoma oncogene homolog B (avian)	MAFB
1558343_at	7.13		
1558882_at	7.11		
220705_s_at	7.08	ADAM metallopeptidase with thrombospondin type 1 motif, 7	ADAMTS7
231628_s_at	7.06		
1561938_at	7.05		
214146_s_at	6.99	pro-platelet basic protein (chemokine (C-X-C motif) ligand 7)	PPBP
235431_s_at	6.98	pellino homolog 3 (Drosophila)	PELI3
241890_at	6.93		
231793_s_at	6.92	calcium/calmodulin-dependent protein kinase (CaM kinase) II delta	CAMK2D
240965_at	6.91	anaphase promoting complex subunit 10	ANAPC10
210248_at	6.85	wingless-type MMTV integration site family, member 7A	WNT7A
210920_x_at	6.77	EMI domain containing 2	EMID2
244169_x_at	6.76		
1553317_s_at	6.61	G protein-coupled receptor 82	GPR82
244259_s_at	6.54		
237859_at	6.50	ankyrin 2, neuronal	ANK2
238269_at	6.48	F-box and leucine-rich repeat protein 7	FBXL7
1567527_at	6.43		
1570506_at	6.42		
242721_at	6.42	autism susceptibility candidate 2	AUTS2

1556155_at	6.40	ADP-ribosylation factor-like 8A	ARL8A
202712_s_at	6.23	creatine kinase, mitochondrial 1B	CKMT1B
1552783_at	6.21	zinc finger protein 417	ZNF417
236335_at	6.17		
1561154_at	6.14		
201843_s_at	6.13	EGF-containing fibulin-like extracellular matrix protein 1	EFEMP1
211896_s_at	6.08	decorin	DCN
240249_at	6.03	breast carcinoma amplified sequence 1	BCAS1
1557195_at	6.01		
243792_x_at	5.93	protein tyrosine phosphatase, non-receptor type 13 (APO-1/CD95 (Fas)-associated phosphatase)	PTPN13
216239_at	5.93	Bardet-Biedl syndrome 9	BBS9
214534_at	5.90	histone cluster 1, H1b	HIST1H1B
237102_at	5.90		
1559109_a_at	5.83		
241338_at	5.78		
217157_x_at	5.78		
215924_at	5.76		
223372_at	5.75	DnaJ (Hsp40) homolog, subfamily C, member 4	DNAJC4
217470_at	5.74		
1558020_at	5.73	ERGIC and golgi 3	ERGIC3
208711_s_at	5.72	cyclin D1	CCND1
1565131_x_at	5.69		
230665_at	5.69		
238901_at	5.68		
212079_s_at	5.64	myeloid/lymphoid or mixed-lineage leukemia (trithorax homolog, Drosophila)	MLL
230520_at	5.62	androgen-induced 1	AIG1
1562270_at	5.59	Rho guanine nucleotide exchange factor (GEF) 7	ARHGEF7
232654_s_at	5.57	UDP glucuronosyltransferase 1 family, polypeptide A6	UGT1A6
237701_at	5.54	chromosome 12 open reading frame 54	C12orf54
217247_at	5.54	vesicle-associated membrane protein 4#null#null	VAMP4#null#null
209398_at	5.53	histone cluster 1, H1c	HIST1H1C
1553613_s_at	5.51	forkhead box C1	FOXC1
244682_at	5.51	calmodulin regulated spectrin-associated protein 1	CAMSAP1
1559591_s_at	5.45	choline dehydrogenase	CHDH
233534_at	5.39	keratin associated protein 3-2	KRTAP3-2
1562990_at	5.36		
242622_x_at	5.33	phosphatase and tensin homolog (mutated in multiple advanced cancers 1)	PTEN
1558611_at	5.32	chromosome 17 open reading frame 69	C17orf69
217400_at	5.30	proliferating cell nuclear antigen pseudogene	PCNAP
1559402_a_at	5.30	chromosome 1 open reading frame 61	C1orf61
1564626_at	5.30		
1566600_at	5.26		
1563498_s_at	5.24	solute carrier family 25, member 45	SLC25A45

240573_at	5.24		
230368_at	5.23	glycogen synthase kinase 3 alpha	GSK3A
206140_at	5.20	LIM homeobox 2	LHX2
217500_at	5.16	TIA1 cytotoxic granule-associated RNA binding protein-like 1	TIAL1
233381_at	5.13	RUN and FYVE domain containing 1	RUFY1
220435_at	5.13	solute carrier family 30, member 10	SLC30A10
237540_at	5.11		
1554699_at	5.11	l(3)mbt-like 4 (Drosophila)	L3MBTL4
236433_at	5.08		
1554142_at	5.04	SGT1, suppressor of G2 allele of SKP1 like 1 (S. cerevisiae)	SUGT1L1
231709_x_at	5.00		
1561657_at	5.00		
230067_at	5.00	family with sequence similarity 124A	FAM124A
212078_s_at	4.99	myeloid/lymphoid or mixed-lineage leukemia (trithorax homolog, Drosophila)	MLL
236110_at	4.98		
207517_at	4.97	laminin, gamma 2	LAMC2
220062_s_at	4.93	melanoma antigen family C, 2	MAGEC2
1553772_at	4.88	glycerol kinase 5 (putative)	GK5
231091_x_at	4.87		
228219_s_at	4.86	ureidopropionase, beta	UPB1
1561125_at	4.84	methylenetetrahydrofolate dehydrogenase (NADP+ dependent) 1-like	MTHFD1L
223938_at	4.82	chromosome 1 open reading frame 49	C1orf49
209962_at	4.80	erythropoietin receptor	EPOR
215376_at	4.78		
206978_at	4.77	chemokine (C-C motif) receptor 2	CCR2
244729_at	4.77		
206655_s_at	4.76	glycoprotein Ib (platelet), beta polypeptide	GP1BB
206300_s_at	4.76	parathyroid hormone-like hormone	PTHLH
210239_at	4.76	iroquois homeobox protein 5	IRX5
228547_at	4.75	neurexin 1	NRXN1
229400_at	4.71	homeobox D10	HOXD10
1558280_s_at	4.70		
209782_s_at	4.69	D site of albumin promoter (albumin D-box) binding protein	DBP
205793_x_at	4.68	tyrosine kinase, non-receptor, 1	TNK1
236654_s_at	4.68		
1559950_at	4.67		
232760_at	4.65	testis expressed sequence 15	TEX15
219522_at	4.62	four jointed box 1 (Drosophila)	FJX1
1559292_s_at	4.60	chromosome 9 open reading frame 14	C9orf14
213831_at	4.60	major histocompatibility complex, class II, DQ alpha 1	HLA-DQA1
243486_at	4.58	staphylococcal nuclease and tudor domain containing 1	SND1
211192_s_at	4.57	CD84 molecule	CD84
1554831_x_at	4.57	amyotrophic lateral sclerosis 2 (juvenile) chromosome region, candidate 11	ALS2CR11
240977_at	4.55	leucine-rich repeats and calponin homology (CH) domain containing 1	LRCH1

229960_at	4.54	mitogen-activated protein kinase kinase kinase 6	MAP3K6
1569841_x_at	4.53		
1556873_at	4.52	ring finger and KH domain containing 2	RKHD2
240845_at	4.49		
204932_at	4.49	tumor necrosis factor receptor superfamily, member 11b (osteoprotegerin)	TNFRSF11B
210182_at	4.45	cortistatin	CORT
222456_s_at	4.45	LIM domain and actin binding 1	LIMA1
219377_at	4.42	family with sequence similarity 59, member A	FAM59A
240569_at	4.41	asialoglycoprotein receptor 2	ASGR2
1561083_at	4.40		
1562106_at	4.40		
1553293_at	4.39	MAS-related GPR, member X3	MRGPRX3
206864_s_at	4.38	harakiri, BCL2 interacting protein (contains only BH3 domain)	HRK
226446_at	4.37	hairy and enhancer of split 6 (Drosophila)	HES6
240409_at	4.36		
216759_at	4.35	HRAS-like suppressor 2	HRASLS2
1556334_s_at	4.34	desmocollin 1	DSC1
238392_at	4.34	translocation associated membrane protein 2	TRAM2
1561763_at	4.32		
1561090_at	4.32		
231647_s_at	4.32	Fc receptor-like 5	FCRL5
244572_at	4.32		
1556817_a_at	4.30		
214347_s_at	4.30	dopa decarboxylase (aromatic L-amino acid decarboxylase)	DDC
201416_at	4.28	SRY (sex determining region Y)-box 4	SOX4
214320_x_at	4.28	cytochrome P450, family 2, subfamily A, polypeptide 7	CYP2A7
241805_at	4.26	gamma-aminobutyric acid (GABA) A receptor, gamma 1	GABRG1
206258_at	4.25	ST8 alpha-N-acetyl-neuraminide alpha-2,8-sialyltransferase 5	ST8SIA5
238808_at	4.24		
224022_x_at	4.24	wingless-type MMTV integration site family, member 16	WNT16
229770_at	4.22	glycosyltransferase 1 domain containing 1	GLT1D1
233766_at	4.21		
208577_at	4.20	histone cluster 1, H3c	HIST1H3C
206179_s_at	4.20		
227546_x_at	4.19	cyclin L2	CCNL2
204422_s_at	4.18	fibroblast growth factor 2 (basic)	FGF2
237578_at	4.18		
234529_at	4.17		
224354_at	4.17		
1554161_at	4.17	solute carrier family 25, member 27	SLC25A27
229622_at	4.16		
242843_at	4.15	brevican	BCAN
1555298_a_at	4.15		
1565804_at	4.14		
228780_at	4.12		

217670_at	4.12	tetraspanin 4	TSPAN4
223449_at	4.12	sema domain, transmembrane domain (TM), and cytoplasmic domain, (semaphorin) 6A	SEMA6A
232663_s_at	4.10		
240042_at	4.09	fibrinogen C domain containing 1	FIBCD1
228170_at	4.08	oligodendrocyte transcription factor 1	OLIG1
242815_x_at	4.08		
241491_at	4.08		
1561136_at	4.07	glycophorin E	GYPE
240447_at	4.07		
230327_at	4.06		
208183_at	4.06	tachykinin receptor 3	TACR3
210304_at	4.05	phosphodiesterase 6B, cGMP-specific, rod, beta (congenital stationary night blindness 3, autosomal d	PDE6B
215878_at	4.04	integrin, beta 1 (fibronectin receptor, beta polypeptide, antigen CD29 includes MDF2, MSK12)	ITGB1
1556113_at	4.03		
243844_at	4.01		
239394_at	4.01	solute carrier family 6 (neurotransmitter transporter, noradrenalin), member 2	SLC6A2
205100_at	3.99	glutamine-fructose-6-phosphate transaminase 2	GFPT2
210366_at	3.98	solute carrier organic anion transporter family, member 1B1	SLCO1B1
219663_s_at	3.97	transmembrane protein 121	TMEM121
237168_at	3.95		
1570228_at	3.95	complement factor H-related 4	CFHR4
1568933_at	3.94		
206591_at	3.94	recombination activating gene 1	RAG1
1555273_at	3.94		
231488_at	3.93		
241507_x_at	3.92		
230456_at	3.91		
243847_at	3.91		
214763_at	3.91	acyl-CoA thioesterase 11	ACOT11
229591_at	3.90	low density lipoprotein receptor-related protein 5	LRP5
228034_x_at	3.89	alkB, alkylation repair homolog 5 (E. coli)	ALKBH5
229554_at	3.89		
1556938_a_at	3.88	dynein, light chain, LC8-type 1	DYNLL1
214605_x_at	3.87	G protein-coupled receptor 1	GPR1
235310_at	3.87	germinal center expressed transcript 2	GCET2
215925_s_at	3.86	CD72 molecule	CD72
244143_at	3.86		
210661_at	3.84	glycine receptor, alpha 3	GLRA3
204802_at	3.84	Ras-related associated with diabetes	RRAD
1570119_at	3.84	androgen-induced proliferation inhibitor	APRIN
201289_at	3.82	cysteine-rich, angiogenic inducer, 61	CYR61
216178_x_at	3.80	integrin, beta 1 (fibronectin receptor, beta polypeptide, antigen CD29 includes MDF2, MSK12)	ITGB1

235895_at	3.80		
207031_at	3.79	bagpipe homeobox homolog 1 (Drosophila)	BAPX1
207434_s_at	3.79	FXYD domain containing ion transport regulator 2	FXYD2
228103_s_at	3.79	neuropilin 2	NRP2
1556529_a_at	3.79		
1569719_at	3.78	BCL2-like 14 (apoptosis facilitator)	BCL2L14
1553428_at	3.77		
1552445_a_at	3.77	extraembryonic, spermatogenesis, homeobox 1 homolog (mouse)	ESX1
236504_x_at	3.76	chromosome 6 open reading frame 52	C6orf52
210040_at	3.76	solute carrier family 12, (potassium-chloride transporter) member 5	SLC12A5
205309_at	3.75	sphingomyelin phosphodiesterase, acid-like 3B	SMPDL3B
237663_at	3.75		
1563190_at	3.74		
210119_at	3.71	potassium inwardly-rectifying channel, subfamily J, member 15	KCNJ15
214537_at	3.71	histone cluster 1, H1d	HIST1H1D
1554769_at	3.70	zinc finger protein 785	ZNF785
241781_at	3.70	chromosome 9 open reading frame 41	C9orf41
215318_at	3.70		
244257_at	3.69	transmembrane protein 104	TMEM104
240900_at	3.69		
222925_at	3.68	doublecortin domain containing 2	DCDC2
243823_at	3.67		
1570384_at	3.67		
1552819_at	3.67	chromosome 21 open reading frame 86	C21orf86
1558466_at	3.67		
227034_at	3.66	ankyrin repeat domain 57	ANKRD57
206380_s_at	3.66	complement factor properdin	CFP
228414_at	3.66	potassium large conductance calcium-activated channel, subfamily M, alpha member 1	KCNMA1
216384_x_at	3.66	prothymosin, alpha pseudogene 4 (gene sequence 112)	PTMAP4
1553655_at	3.65	cell division cycle 20 homolog B (S. cerevisiae)	CDC20B
221587_s_at	3.65	chromosome 19 open reading frame 24	C19orf24
238168_at	3.63	transmembrane 4 L six family member 1	TM4SF1
1567612_at	3.63		
240000_at	3.61		
231655_x_at	3.61		
205649_s_at	3.61	fibrinogen alpha chain	FGA
226435_at	3.61	papilin, proteoglycan-like sulfated glycoprotein	PAPLN
212338_at	3.60	myosin ID	MYO1D
205894_at	3.60	arylsulfatase E (chondrodysplasia punctata 1)	ARSE
230804_at	3.59	chromosome 6 open reading frame 194	C6orf194
1558649_at	3.59		
238844_s_at	3.58	nephronophthisis 1 (juvenile)	NPHP1
1570153_at	3.58	spermatogenesis and oogenesis specific basic helix-loop-helix 2	SOHLH2
243418_at	3.58		

226225_at	3.57	mutated in colorectal cancers	MCC
229012_at	3.57	chromosome 9 open reading frame 24	C9orf24
238024_at	3.57	ornithine decarboxylase antizyme 2	OAZ2
227671_at	3.56	X (inactive)-specific transcript	XIST
1569112_at	3.54	solute carrier family 44, member 5	SLC44A5
215615_x_at	3.53		
204913_s_at	3.53	SRY (sex determining region Y)-box 11	SOX11
242789_at	3.52	phosphodiesterase 1A, calmodulin-dependent	PDE1A
222337_at	3.52	oxysterol binding protein-like 9	OSBPL9
210650_s_at	3.52	piccolo (presynaptic cytomatrix protein)	PCLO
1563691_at	3.52		
219534_x_at	3.52	cyclin-dependent kinase inhibitor 1C (p57, Kip2)	CDKN1C
206510_at	3.51	sine oculis homeobox homolog 2 (Drosophila)	SIX2
227629_at	3.51	prolactin receptor	PRLR
202274_at	3.50	actin, gamma 2, smooth muscle, enteric	ACTG2
231223_at	3.50	CUB and Sushi multiple domains 1	CSMD1
222181_at	3.50	CCR4-NOT transcription complex, subunit 2	CNOT2
230960_at	3.50		
243307_at	3.50		
1558999_x_at	3.46		
1556439_at	3.46		
216495_x_at	3.45	isovaleryl Coenzyme A dehydrogenase	IVD
242085_at	3.44	chromosome 2 open reading frame 18	C2orf18
209293_x_at	3.42	inhibitor of DNA binding 4, dominant negative helix-loop-helix protein	ID4
1563203_at	3.42		
243655_x_at	3.40	SWI/SNF related, matrix associated, actin dependent regulator of chromatin, subfamily a, member 4	SMARCA4
228066_at	3.40		
1562443_at	3.39	chromosome 6 open reading frame 213	C6orf213
244021_at	3.39		
1561965_at	3.39		
1555677_s_at	3.38	structural maintenance of chromosomes 1A	SMC1A
214038_at	3.37	chemokine (C-C motif) ligand 8	CCL8
243735_at	3.37		
244518_at	3.37		
230539_at	3.36		
242054_s_at	3.35		
244595_at	3.34		
233676_at	3.34		
231581_at	3.33		
1563963_at	3.32		
214554_at	3.32	histone cluster 1, H2al	HIST1H2AL
1561512_at	3.32		
1562947_x_at	3.32		
207962_at	3.31	calpain 11	CAPN11
235102_x_at	3.31	GRB2-related adaptor protein	GRAP
241042_at	3.30		
230655_at	3.30		
207267_s_at	3.29	Down syndrome critical region gene 6	DSCR6
224724_at	3.28	sulfatase 2	SULF2

243184_at	3.28	tight junction protein 1 (zona occludens 1)	TJP1
214542_x_at	3.27	histone cluster 1, H2ai	HIST1H2AI
1569334_at	3.27	stimulated by retinoic acid gene 6 homolog (mouse)	STRA6
244505_at	3.27		
239546_at	3.26		
235144_at	3.26		
223435_s_at	3.26	protocadherin alpha subfamily C, 2	PCDHAC2
204555_s_at	3.25	protein phosphatase 1, regulatory (inhibitor) subunit 3D	PPP1R3D
1567013_at	3.25	nuclear factor (erythroid-derived 2)-like 2	NFE2L2
237338_at	3.25	UDP-GlcNAc:betaGal beta-1,3-N-acetylglucosaminyltransferase 8	B3GNT8
230524_at	3.24	mannose phosphate isomerase	MPI
216089_at	3.24		
210412_at	3.24	glutamate receptor, ionotropic, N-methyl D-aspartate 2B	GRIN2B
206207_at	3.24	Charcot-Leyden crystal protein	CLC
1563173_at	3.23		
216475_at	3.22		
206957_at	3.21	alanine-glyoxylate aminotransferase (oxalosis I; hyperoxaluria I; glycolicaciduria; serine-pyruvate	AGXT
215185_at	3.21		
237979_at	3.20	EH-domain containing 4	EHD4
1563039_at	3.20		
213638_at	3.19	phosphatase and actin regulator 1	PHACTR1
1553181_at	3.19	DEAD (Asp-Glu-Ala-Asp) box polypeptide 31	DDX31
213577_at	3.19	squalene epoxidase	SQLE
1565685_at	3.18		
225188_at	3.17	Ras association (RalGDS/AF-6) and pleckstrin homology domains 1	RAPH1
232127_at	3.16	chloride channel 5 (nephrolithiasis 2, X-linked, Dent disease)	CLCN5
207105_s_at	3.16	phosphoinositide-3-kinase, regulatory subunit 2 (p85 beta)	PIK3R2
215779_s_at	3.15	histone cluster 1, H2bg	HIST1H2BG
216459_x_at	3.14		
1564259_at	3.13		
1563121_at	3.13		
1567060_at	3.12	olfactory receptor, family 8, subfamily G, member 1	OR8G1
1570388_a_at	3.11		
235266_at	3.11	ATPase family, AAA domain containing 2	ATAD2
213182_x_at	3.11	cyclin-dependent kinase inhibitor 1C (p57, Kip2)	CDKN1C
1562761_at	3.11	chromosome 9 open reading frame 95	C9orf95
233652_at	3.10		
242924_at	3.10	phosphatidylinositol transfer protein, beta	PITPNB
235343_at	3.10		
242680_at	3.10		
226559_at	3.09	immediate early response 5-like	IER5L

215686_x_at	3.09	transcription factor AP-2 beta (activating enhancer binding protein 2 beta)#transcription factor AP-2 delta (activating enhancer binding protein 2 delta)	TFAP2B#TFAP2D
205729_at	3.09	oncostatin M receptor	OSMR
206519_x_at	3.09	sialic acid binding Ig-like lectin 6	SIGLEC6
208186_s_at	3.09	lipase, hormone-sensitive	LIPE
209348_s_at	3.08	v-maf musculoaponeurotic fibrosarcoma oncogene homolog (avian)	MAF
241209_at	3.08	IQ motif and WD repeats 1	IQWD1
211538_s_at	3.08	heat shock 70kDa protein 2	HSPA2
238264_at	3.08	NMD3 homolog (S. cerevisiae)	NMD3
205937_at	3.07	cell growth regulator with EF-hand domain 1	CGREF1
230493_at	3.07	transmembrane protein 46	TMEM46
230402_at	3.07	BCL2-like 1#forkhead-like 18 (Drosophila)#TPX2, microtubule-associated, homolog (Xenopus laevis)#chromosome 20 open reading frame 57#myosin light chain kinase 2, skeletal muscle#dual specificity phosphatase 15#tubulin tyrosine ligase-like family, member 9	BCL2L1#FKHL18#TPX2#C20orf57#MYLK2#DUSP15#TTLL9
235761_at	3.07		
211980_at	3.07	collagen, type IV, alpha 1	COL4A1
243542_at	3.07		
1555144_at	3.06		
241149_at	3.06		
243755_at	3.06	prolactin receptor	PRLR
241744_x_at	3.05		
1564039_at	3.05	zinc finger protein 390	ZNF390
232202_at	3.05		
1560573_at	3.05		
217443_at	3.04		
239203_at	3.03		
211585_at	3.02	nuclear protein, ataxia-telangiectasia locus	NPAT
210738_s_at	3.02	solute carrier family 4, sodium bicarbonate cotransporter, member 4	SLC4A4
1554987_at	3.01	golgi autoantigen, golgin subfamily a, 3	GOLGA3
1563398_at	3.01		
233981_at	3.01		
208576_s_at	2.99	histone cluster 1, H3b	HIST1H3B
208527_x_at	2.99	histone cluster 1, H2be	HIST1H2BE
218468_s_at	2.99	gremlin 1, cysteine knot superfamily, homolog (Xenopus laevis)	GREM1
204787_at	2.98	V-set and immunoglobulin domain containing 4	VSIG4
237837_at	2.98		
227429_at	2.97	EF-hand calcium binding domain 4A	EFCAB4A
209598_at	2.97	paraneoplastic antigen MA2	PNMA2
1553674_at	2.96	leucine rich repeat containing 44	LRRC44
217560_at	2.96		
1554867_a_at	2.96	proline rich 16	PRR16
230863_at	2.96	low density lipoprotein-related protein 2	LRP2
234344_at	2.96	RAP2C, member of RAS oncogene family	RAP2C
236650_at	2.95		

231050_at	2.95	HRAS-like suppressor family, member 5	HRASLS5
1566700_at	2.95	vaccinia related kinase 3	VRK3
228523_at	2.94	nanos homolog 1 (Drosophila)	NANOS1
224448_s_at	2.93	chromosome 6 open reading frame 125	C6orf125
1558247_s_at	2.92		
204149_s_at	2.92	glutathione S-transferase M4	GSTM4
1560883_s_at	2.92		
223690_at	2.92	latent transforming growth factor beta binding protein 2	LTBP2
237784_at	2.92	SUB1 homolog (S. cerevisiae)	SUB1
1553661_a_at	2.91	HUS1 checkpoint homolog b (S. pombe)	HUS1B
226534_at	2.91	KIT ligand	KITLG
221577_x_at	2.91	growth differentiation factor 15	GDF15
230785_at	2.90		
235401_s_at	2.90	Fc receptor-like A	FCRLA
244385_at	2.90	jumonji domain containing 2C	JMJD2C
1555263_at	2.89		
217513_at	2.89	chromosome 17 open reading frame 60	C17orf60
209189_at	2.89	v-fos FBJ murine osteosarcoma viral oncogene homolog	FOS
229869_at	2.88		
203032_s_at	2.88	fumarate hydratase	FH
243814_at	2.87	zinc finger, MYND-type containing 8	ZMYND8
233791_at	2.87		
212188_at	2.87	potassium channel tetramerisation domain containing 12	KCTD12
1560108_at	2.87	negative regulator of ubiquitin-like proteins 1	NUB1
1556194_a_at	2.87		
224361_s_at	2.86	interleukin 17 receptor B	IL17RB
206622_at	2.86	thyrotropin-releasing hormone	TRH
208553_at	2.85	histone cluster 1, H1e	HIST1H1E
241674_s_at	2.85		
223131_s_at	2.85	tripartite motif-containing 8	TRIM8
236277_at	2.84		
228614_at	2.83		
213183_s_at	2.83	cyclin-dependent kinase inhibitor 1C (p57, Kip2)	CDKN1C
213418_at	2.82	heat shock 70kDa protein 6 (HSP70B')	HSPA6
210033_s_at	2.82	sperm associated antigen 6	SPAG6
1560320_a_at	2.81		
205293_x_at	2.81	BAI1-associated protein 2	BAIAP2
215349_at	2.80		
117_at	2.80		
206935_at	2.80	protocadherin 8	PCDH8
238379_x_at	2.79		
211124_s_at	2.79	KIT ligand	KITLG
244001_at	2.79	nucleosome assembly protein 1-like 4	NAP1L4
1561615_s_at	2.79	solute carrier family 8 (sodium/calcium exchanger), member 1	SLC8A1
227405_s_at	2.78	frizzled homolog 8 (Drosophila)	FZD8
203186_s_at	2.78	S100 calcium binding protein A4	S100A4
204431_at	2.78	transducin-like enhancer of split 2 (E(sp1) homolog, Drosophila)	TLE2

1562458_at	2.78	ubiquitin-conjugating enzyme E2W (putative)	UBE2W
222096_x_at	2.77		
242418_at	2.77		
220523_at	2.77	EF-hand domain (C-terminal) containing 2	EFHC2
227362_at	2.77	SLC2A4 regulator	SLC2A4RG
202986_at	2.76	aryl-hydrocarbon receptor nuclear translocator 2	ARNT2
209687_at	2.76	chemokine (C-X-C motif) ligand 12 (stromal cell-derived factor 1)	CXCL12
223467_at	2.76	RAS, dexamethasone-induced 1	RASD1
214384_s_at	2.76	dynactin 2 (p50)	DCTN2
1553808_a_at	2.76	NK2 transcription factor related, locus 3 (Drosophila)	NKX2-3
1562712_at	2.76		
1552694_at	2.75	solute carrier family 2 (facilitated glucose transporter), member 13	SLC2A13
210763_x_at	2.75	natural cytotoxicity triggering receptor 3	NCR3
1558858_at	2.74		
213260_at	2.74		
1563802_at	2.74		
1565337_at	2.73	dynein, axonemal, heavy chain 6	DNAH6
230542_at	2.73	zinc finger protein 597	ZNF597
1569136_at	2.73	mannosyl (alpha-1,3-)-glycoprotein beta-1,4-N-acetylglucosaminyltransferase, isozyme A	MGAT4A
238411_x_at	2.73		
229229_at	2.73	alanine-glyoxylate aminotransferase 2	AGXT2
234531_at	2.73		
218959_at	2.72	homeobox C10	HOXC10
204611_s_at	2.71	protein phosphatase 2, regulatory subunit B', beta isoform	PPP2R5B
1558452_at	2.71	transmembrane protein 144	TMEM144
209815_at	2.71	patched homolog 1 (Drosophila)	PTCH1
229189_s_at	2.70		
224972_at	2.70	chromosome 20 open reading frame 52	C20orf52
211445_x_at	2.69	nascent-polypeptide-associated complex alpha polypeptide pseudogene 1	NACAP1
233062_at	2.69		
1562621_at	2.69		
209419_at	2.69	THO complex 5	THOC5
236087_at	2.68	actin binding LIM protein family, member 2	ABLIM2
230084_at	2.68	solute carrier family 30 (zinc transporter), member 2	SLC30A2
1568739_at	2.67		
226346_at	2.67		
225441_x_at	2.67	LSM domain containing 1	LSMD1
205809_s_at	2.67	Wiskott-Aldrich syndrome-like	WASL
210992_x_at	2.66		
220096_at	2.66	ribonuclease T2	RNASET2
238935_at	2.66	ribosomal protein S27-like	RPS27L
202526_at	2.65	SMAD family member 4	SMAD4
238160_at	2.65	acyl-CoA thioesterase 12	ACOT12
1553561_at	2.65	taste receptor, type 2, member 50	TAS2R50
240800_x_at	2.65		

227292_at	2.65		
1554677_s_at	2.65	CKLF-like MARVEL transmembrane domain containing 4	CMTM4
234375_x_at	2.64		
243064_at	2.64		
206776_x_at	2.63	acrosomal vesicle protein 1	ACRV1
1559561_at	2.63	F-box protein, helicase, 18	FBXO18
228217_s_at	2.62		
218384_at	2.62	calcium regulated heat stable protein 1, 24kDa	CARHSP1
213719_s_at	2.62	SWI/SNF related, matrix associated, actin dependent regulator of chromatin, subfamily a, member 4	SMARCA4
214195_at	2.62	tripeptidyl peptidase I	TPP1
1559059_s_at	2.62	zinc finger protein 611	ZNF611
209492_x_at	2.62	ATP synthase, H+ transporting, mitochondrial F0 complex, subunit E	ATP5I
232090_at	2.61		
219537_x_at	2.61	delta-like 3 (Drosophila)	DLL3
1560755_at	2.61		
1565925_at	2.61		
236529_at	2.60	scavenger receptor cysteine rich domain containing, group B (4 domains)	SRCRB4D
1553335_x_at	2.60		
244205_at	2.60	aminolevulinate, delta-, synthase 2 (sideroblastic/hypochromic anemia)	ALAS2
206353_at	2.60	cytochrome c oxidase subunit VIa polypeptide 2	COX6A2
206105_at	2.60	AF4/FMR2 family, member 2	AFF2
1553265_at	2.60	solute carrier family 23 (nucleobase transporters), member 3	SLC23A3
207688_s_at	2.60	inhibin, beta C	INHBC
232706_s_at	2.60	TraB domain containing	TRABD
244885_at	2.60		
1556279_at	2.59		
228584_at	2.59	sarcoglycan, beta (43kDa dystrophin-associated glycoprotein)	SGCB
220551_at	2.59	solute carrier family 17 (sodium-dependent inorganic phosphate cotransporter), member 6	SLC17A6
244557_at	2.59		
208229_at	2.58	fibroblast growth factor receptor 2 (bacteria-expressed kinase, keratinocyte growth factor receptor,	FGFR2
204659_s_at	2.58	growth factor, augmenter of liver regeneration (ERV1 homolog, S. cerevisiae)	GFER
225806_at	2.58	jub, ajuba homolog (Xenopus laevis)	JUB
1560869_a_at	2.58		
241298_x_at	2.58		
204457_s_at	2.58	growth arrest-specific 1	GAS1
229117_s_at	2.57	jun D proto-oncogene	JUND
213174_at	2.57	tetratricopeptide repeat domain 9	TTC9
1561650_s_at	2.57		
202668_at	2.57	ephrin-B2	EFNB2
214677_x_at	2.56	immunoglobulin lambda joining 3	IGLJ3

212611_at	2.56		
236668_at	2.56		
210906_x_at	2.56	aquaporin 4	AQP4
228972_at	2.56		
1566272_at	2.56	GTPase activating Rap/RanGAP domain-like 1	GARNL1
211695_x_at	2.56	mucin 1, cell surface associated	MUC1
217728_at	2.56	S100 calcium binding protein A6	S100A6
208261_x_at	2.56	interferon, alpha 10	IFNA10
236897_at	2.56		
214330_at	2.55	ATP synthase mitochondrial F1 complex assembly factor 2	ATPAF2
234553_at	2.55		
222847_s_at	2.55	egl nine homolog 3 (C. elegans)	EGLN3
237563_s_at	2.55		
239488_at	2.55		
241736_at	2.54	F-box and WD repeat domain containing 2	FBXW2
226989_at	2.54	RGM domain family, member B	RGMB
210199_at	2.54	crystallin, alpha A	CRYAA
210809_s_at	2.54	periostin, osteoblast specific factor	POSTN
228824_s_at	2.54	leukotriene B4 12-hydroxydehydrogenase	LTB4DH
207881_at	2.54		
1558668_s_at	2.53	spermatogenesis associated 22	SPATA22
1558754_at	2.53	zinc finger protein 763	ZNF763
206950_at	2.53	sodium channel, voltage-gated, type IX, alpha subunit	SCN9A
243019_at	2.53		
228706_s_at	2.53	claudin 23	CLDN23
209807_s_at	2.52	nuclear factor I/X (CCAAT-binding transcription factor)	NFIX
219727_at	2.52	dual oxidase 2	DUOX2
209204_at	2.52	LIM domain only 4	LMO4
223860_at	2.52		
235350_at	2.51		
1557636_a_at	2.51		
214467_at	2.51	G protein-coupled receptor 65	GPR65
203973_s_at	2.51	CCAAT/enhancer binding protein (C/EBP), delta	CEBPD
1564463_at	2.51		
229116_at	2.51		
235765_at	2.50	transducin-like enhancer of split 4 (E(sp1) homolog, Drosophila)	TLE4
241328_at	2.50	zinc finger, matrin type 1	ZMAT1
225167_at	2.50	FERM domain containing 4A	FRMD4A
1556967_at	2.49	zinc finger, DHHC-type containing 14	ZDHHC14
225476_at	2.49	HLA-B associated transcript 4	BAT4
217171_at	2.49	sphingomyelin phosphodiesterase 1, acid lysosomal (acid sphingomyelinase)	SMPD1
200826_at	2.49	small nuclear ribonucleoprotein D2 polypeptide 16.5kDa	SNRPD2
1556683_x_at	2.49		
205208_at	2.49	aldehyde dehydrogenase 1 family, member L1	ALDH1L1
1554522_at	2.49	cyclin M2	CNNM2
239592_at	2.49		

1562815_at	2.48		
240855_at	2.48	spectrin, beta, erythrocytic (includes spherocytosis, clinical type I)	SPTB
204559_s_at	2.48	LSM7 homolog, U6 small nuclear RNA associated (S. cerevisiae)	LSM7
205932_s_at	2.48	msh homeobox 1	MSX1
1558831_x_at	2.47		
224547_at	2.47		
229715_at	2.47		
241184_x_at	2.47	zinc finger protein 407	ZNF407
228444_at	2.47		
230696_at	2.47		
239850_at	2.47		
238705_at	2.46		
226241_s_at	2.46	mitochondrial ribosomal protein L52	MRPL52
241826_x_at	2.46		
233164_x_at	2.46	rhomboid domain containing 1	RHBDD1
224774_s_at	2.46	neuron navigator 1	NAV1
232295_at	2.46	G elongation factor, mitochondrial 1	GFM1
211730_s_at	2.45	polymerase (RNA) II (DNA directed) polypeptide L, 7.6kDa	POLR2L
206538_at	2.45	muscle RAS oncogene homolog	MRAS
230519_at	2.45	family with sequence similarity 124A	FAM124A
240044_x_at	2.44	trinucleotide repeat containing 6B	TNRC6B
1559000_at	2.44	chromosome 10 open reading frame 108	C10orf108
1561468_at	2.43		
209030_s_at	2.43	cell adhesion molecule 1	CADM1
216421_at	2.43	v-myb myeloblastosis viral oncogene homolog (avian)-like 2#intraflagellar transport 52 homolog (Chlamydomonas)#ribosomal protein L27a pseudogene#family with sequence similarity 112, member A	MYBL2#IFT52 #RPL27AP#FA M112A
216380_x_at	2.42	calneuron 1	CALN1
211603_s_at	2.42	ets variant gene 4 (E1A enhancer binding protein, E1AF)	ETV4
233146_at	2.42	kinase non-catalytic C-lobe domain (KIND) containing 1	KNDC1
216894_x_at	2.42	cyclin-dependent kinase inhibitor 1C (p57, Kip2)	CDKN1C
238949_at	2.42		
219740_at	2.42	vasohibin 2	VASH2
231798_at	2.42	noggin	NOG
1552509_a_at	2.41	CD300 molecule-like family member g	CD300LG
204525_at	2.41	PHD finger protein 14	PHF14
221997_s_at	2.41	mitochondrial ribosomal protein L52	MRPL52
205436_s_at	2.41	H2A histone family, member X	H2AFX
231696_x_at	2.41	transmembrane protein 50B	TMEM50B
1552320_a_at	2.41	coiled-coil domain containing 65	CCDC65
225425_s_at	2.41	mitochondrial ribosomal protein L41	MRPL41
232864_s_at	2.41	AF4/FMR2 family, member 4	AFF4
237159_x_at	2.40	adaptor-related protein complex 1, sigma 3 subunit	AP1S3

209291_at	2.40	inhibitor of DNA binding 4, dominant negative helix-loop-helix protein	ID4
234127_at	2.40		
212107_s_at	2.40	DEAH (Asp-Glu-Ala-His) box polypeptide 9	DHX9
239936_at	2.40	deleted in lymphocytic leukemia, 2	DLEU2
1561369_at	2.40		
220369_at	2.40	SMEK homolog 1, suppressor of mek1 (Dictyostelium)	SMEK1
206307_s_at	2.40	forkhead box D1	FOXD1
1569578_at	2.39		
202528_at	2.39	UDP-galactose-4-epimerase	GALE
216191_s_at	2.38	T cell receptor alpha locus	TRA@
207064_s_at	2.38	amine oxidase, copper containing 2 (retina-specific)	AOC2
229690_at	2.38	family with sequence similarity 109, member A	FAM109A
233639_at	2.38	protein O-linked mannose beta1,2-N-acetylglucosaminyltransferase	POMGNT1
1554770_x_at	2.38	zinc finger protein 785	ZNF785
1565577_s_at	2.37		
218494_s_at	2.37	SLC2A4 regulator	SLC2A4RG
222295_x_at	2.37		
207335_x_at	2.37	ATP synthase, H+ transporting, mitochondrial F0 complex, subunit E	ATP5I
1561417_x_at	2.36		
235903_at	2.36	ankyrin repeat and sterile alpha motif domain containing 6	ANKS6
208579_x_at	2.36	H2B histone family, member S	H2BFS
219371_s_at	2.36	Kruppel-like factor 2 (lung)	KLF2
210267_at	2.36	NIPA-like domain containing 3	NPAL3
238353_at	2.35	RAS-like, family 11, member A	RASL11A
237440_at	2.35		
213825_at	2.35	oligodendrocyte lineage transcription factor 2	OLIG2
208490_x_at	2.35	histone cluster 1, H2bf	HIST1H2BF
203549_s_at	2.35	lipoprotein lipase	LPL
204379_s_at	2.35	fibroblast growth factor receptor 3 (achondroplasia, thanatophoric dwarfism)	FGFR3
230871_at	2.35	DEAH (Asp-Glu-Ala-His) box polypeptide 30	DHX30
235000_at	2.35		
1555303_at	2.34		
227443_at	2.34	chromosome 9 open reading frame 150	C9orf150
236270_at	2.34	nuclear factor of activated T-cells, cytoplasmic, calcineurin-dependent 4	NFATC4
234525_at	2.34		
241265_x_at	2.34		
1559266_s_at	2.33		
227725_at	2.33	ST6 (alpha-N-acetyl-neuraminyl-2,3-beta-galactosyl-1,3)-N-acetylgalactosaminide alpha-2,6-sialyltran	ST6GALNAC1
240622_at	2.33	progestin and adipoQ receptor family member III	PAQR3
222254_at	2.33	protein tyrosine phosphatase, non-receptor type 1#cytochrome c oxidase subunit VIc pseudogene 2	PTPN1#COX6CP2

203887_s_at	2.33	thrombomodulin	THBD
1570023_at	2.33		
244502_at	2.33		
214862_x_at	2.32		
206669_at	2.32	glutamate decarboxylase 1 (brain, 67kDa)	GAD1
205618_at	2.32	proline rich Gla (G-carboxyglutamic acid) 1	PRRG1
244467_at	2.32		
224785_at	2.32	family with sequence similarity 100, member B	FAM100B
209836_x_at	2.32	bolA homolog 2 (E. coli)	BOLA2
214213_x_at	2.32	lamin A/C	LMNA
211263_s_at	2.31	proprotein convertase subtilisin/kexin type 6	PCSK6
226499_at	2.31		
203178_at	2.31	glycine amidinotransferase (L-arginine:glycine amidinotransferase)	GATM
232380_at	2.30		
217128_s_at	2.30	calcium/calmodulin-dependent protein kinase IG	CAMK1G
1558842_at	2.30		
217700_at	2.30		
1553710_at	2.30		
201171_at	2.30	ATPase, H+ transporting, lysosomal 9kDa, V0 subunit e1	ATP6V0E1
1556579_s_at	2.29	immunoglobulin superfamily, member 10	IGSF10
234266_at	2.29		
211775_x_at	2.29		
204682_at	2.29	latent transforming growth factor beta binding protein 2	LTBP2
239114_at	2.29	secretion regulating guanine nucleotide exchange factor	SERGEF
1563614_at	2.29	Mdm2, transformed 3T3 cell double minute 2, p53 binding protein (mouse) binding protein, 104kDa	MTBP
218762_at	2.28	zinc finger protein 574	ZNF574
239002_at	2.28	asp (abnormal spindle) homolog, microcephaly associated (Drosophila)	ASPM
239913_at	2.28	solute carrier family 10 (sodium/bile acid cotransporter family), member 4	SLC10A4
228501_at	2.28	UDP-N-acetyl-alpha-D-galactosamine:polypeptide N-acetylgalactosaminyltransferase-like 2	GALNTL2
242835_s_at	2.28		
225730_s_at	2.28	THUMP domain containing 3	THUMPD3
230811_at	2.28	chromosome 16 open reading frame 55	C16orf55
203705_s_at	2.28	frizzled homolog 7 (Drosophila)	FZD7
203176_s_at	2.27	transcription factor A, mitochondrial	TFAM
235562_at	2.27		
232144_at	2.27	pre-B-cell leukemia homeobox 1	PBX1
239714_at	2.27	C1q and tumor necrosis factor related protein 3	C1QTNF3
241569_at	2.26		
211074_at	2.26	folate receptor 1 (adult)	FOLR1
207857_at	2.26	leukocyte immunoglobulin-like receptor, subfamily A (with TM domain), member 2	LILRA2

208506_at	2.26	histone cluster 1, H3f	HIST1H3F
1557987_at	2.26		
208301_at	2.26		
241114_s_at	2.26		
223779_at	2.26		
202343_x_at	2.25	cytochrome c oxidase subunit Vb	COX5B
236988_x_at	2.25	integrin, beta 2 (complement component 3 receptor 3 and 4 subunit)	ITGB2
227876_at	2.25		
240079_at	2.25	zinc finger protein 81	ZNF81
212105_s_at	2.25	DEAH (Asp-Glu-Ala-His) box polypeptide 9	DHX9
227522_at	2.25	carboxymethylenebutenolidase homolog (Pseudomonas)	CMBL
208583_x_at	2.25	histone cluster 1, H2aj	HIST1H2AJ
233673_at	2.25		
233852_at	2.24	polymerase (DNA directed), eta	POLH
1562894_at	2.24		
227197_at	2.24		
229972_at	2.24		
224563_at	2.24		
207056_s_at	2.24	solute carrier family 4, sodium bicarbonate cotransporter, member 8	SLC4A8
1559006_at	2.24		
243855_at	2.24	small nuclear ribonucleoprotein polypeptide N	SNRPN
1555489_at	2.24		
1554628_at	2.24	zinc finger protein 57	ZNF57
1563512_at	2.24	nitric oxide synthase 1 (neuronal) adaptor protein	NOS1AP
210050_at	2.24	triosephosphate isomerase 1	TPI1
1553192_at	2.24	zinc finger protein 441	ZNF441
215415_s_at	2.23	lysosomal trafficking regulator	LYST
1553120_at	2.23	claspin homolog (Xenopus laevis)	CLSPN
224937_at	2.23	prostaglandin F2 receptor negative regulator	PTGFRN
228049_x_at	2.22		
225867_at	2.22	vasorin	VASN
213139_at	2.22	snail homolog 2 (Drosophila)	SNAI2
205559_s_at	2.22	proprotein convertase subtilisin/kexin type 5	PCSK5
226388_at	2.22	transcription elongation factor A (SII), 3	TCEA3
1561016_at	2.22		
213311_s_at	2.22	transcription factor 25 (basic helix-loop-helix)	TCF25
1568596_a_at	2.22	trophinin associated protein (tastin)	TROAP
243440_at	2.21		
232695_at	2.21	kinesin family member 6	KIF6
213668_s_at	2.21	SRY (sex determining region Y)-box 4	SOX4
222164_at	2.21		
218906_x_at	2.21	kinesin light chain 2	KLC2
220453_at	2.21	PQ loop repeat containing 2	PQLC2
1566691_at	2.20		
235202_x_at	2.20		
244335_at	2.20		
205289_at	2.20	bone morphogenetic protein 2	BMP2
201392_s_at	2.20	insulin-like growth factor 2 receptor	IGF2R
215853_at	2.20		

201730_s_at	2.20	translocated promoter region (to activated MET oncogene)	TPR
204175_at	2.20	zinc finger protein 593	ZNF593
226374_at	2.20		
226014_at	2.20	eukaryotic translation initiation factor 3, subunit 5 epsilon, 47kDa	EIF3S5
242154_x_at	2.20	peptidylprolyl isomerase (cyclophilin)-like 5	PPIL5
227063_at	2.20	chromosome 17 open reading frame 61	C17orf61
233452_at	2.20		
219295_s_at	2.20	procollagen C-endopeptidase enhancer 2	PCOLCE2
204717_s_at	2.20	solute carrier family 29 (nucleoside transporters), member 2	SLC29A2
203190_at	2.20	NADH dehydrogenase (ubiquinone) Fe-S protein 8, 23kDa (NADH-coenzyme Q reductase)	NDUFS8
226845_s_at	2.20	myeloma overexpressed 2	MYEOV2
207046_at	2.20	histone cluster 2, H4a	HIST2H4A
224412_s_at	2.19	transient receptor potential cation channel, subfamily M, member 6	TRPM6
232684_at	2.19		
1559839_at	2.19	T-box 18	TBX18
1555120_at	2.19	CD96 molecule	CD96
214463_x_at	2.19	histone cluster 1, H4k	HIST1H4K
242564_at	2.18		
205774_at	2.18	coagulation factor XII (Hageman factor)	F12
1554742_at	2.18	PMS1 postmeiotic segregation increased 1 (S. cerevisiae)	PMS1
226899_at	2.18	unc-5 homolog B (C. elegans)	UNC5B
230169_at	2.18	THAP domain containing 6	THAP6
1552649_a_at	2.18	ring finger and FYVE-like domain containing 1	RFFL
205475_at	2.18		
234434_at	2.18		
1555444_a_at	2.17	protein phosphatase 1, regulatory (inhibitor) subunit 12B	PPP1R12B
240147_at	2.17		
1556404_a_at	2.17		
1558354_s_at	2.17		
200702_s_at	2.16	DEAD (Asp-Glu-Ala-Asp) box polypeptide 24	DDX24
208292_at	2.16	bone morphogenetic protein 10	BMP10
243740_at	2.16		
241416_at	2.16		
1554830_a_at	2.16	STEAP family member 3	STEAP3
205081_at	2.16	cysteine-rich protein 1 (intestinal)	CRIP1
207132_x_at	2.16	prefoldin subunit 5	PFDN5
202090_s_at	2.16	ubiquinol-cytochrome c reductase, 6.4kDa subunit	UQCR
202865_at	2.16	DnaJ (Hsp40) homolog, subfamily B, member 12	DNAJB12
1553037_a_at	2.16	synapsin II	SYN2
211299_s_at	2.16	flotillin 2	FLOT2
1555536_at	2.16	anthrax toxin receptor 2	ANTXR2
244265_at	2.16	arginine-glutamic acid dipeptide (RE) repeats	RERE
200772_x_at	2.16	prothymosin, alpha (gene sequence 28)	PTMA

206565_x_at	2.16		
220145_at	2.16	microtubule-associated protein 9	MAP9
201635_s_at	2.16	fragile X mental retardation, autosomal homolog 1	FXR1
1553107_s_at	2.15	chromosome 5 open reading frame 24	C5orf24
1569816_at	2.15		
214965_at	2.15	spermatogenesis associated 2-like	SPATA2L
232679_at	2.15		
237910_x_at	2.15	family with sequence similarity 92, member A1	FAM92A1
212009_s_at	2.15	stress-induced-phosphoprotein 1 (Hsp70/Hsp90-organizing protein)	STIP1
223927_at	2.15	protocadherin beta 9	PCDHB9
234472_at	2.15	UDP-N-acetyl-alpha-D-galactosamine:polypeptide N-acetylgalactosaminyltransferase 13 (GalNAc-T13)	GALNT13
221705_s_at	2.15		
224932_at	2.15	chromosome 22 open reading frame 16	C22orf16
222153_at	2.15	myelin expression factor 2	MYEF2
1554010_at	2.15	N-deacetylase/N-sulfotransferase (heparan glucosaminyl) 1	NDST1
217149_x_at	2.14	tyrosine kinase, non-receptor, 1	TNK1
207634_at	2.14	programmed cell death 1	PDCD1
1558123_at	2.14		
222396_at	2.14	hematological and neurological expressed 1	HN1
225870_s_at	2.14	trafficking protein particle complex 5	TRAPPC5
239046_at	2.14		
223614_at	2.14	chromosome 8 open reading frame 57	C8orf57
225703_at	2.14		
209258_s_at	2.14	structural maintenance of chromosomes 3	SMC3
233777_at	2.13		
207813_s_at	2.13	ferredoxin reductase	FDXR
220734_s_at	2.13		
239429_at	2.13		
211549_s_at	2.13	hydroxyprostaglandin dehydrogenase 15-(NAD)	HPGD
232096_x_at	2.13		
217014_s_at	2.13	alpha-2-glycoprotein 1, zinc-binding#zinc finger with KRAB and SCAN domains 1	AZGP1#ZKSCAN1
239066_at	2.13		
1557832_at	2.13		
225967_s_at	2.12		
231909_x_at	2.12	outer dense fiber of sperm tails 2-like	ODF2L
239896_at	2.12		
242246_x_at	2.12		
227273_at	2.12		
208202_s_at	2.12	PHD finger protein 15	PHF15
225552_x_at	2.11	cyclin L2	CCNL2
232516_x_at	2.11	YY1 associated protein 1	YY1AP1
1557818_x_at	2.11		
228776_at	2.11	gap junction protein, alpha 7, 45kDa	GJA7
228368_at	2.11	Rho GTPase activating protein 20	ARHGAP20
223397_s_at	2.11	nuclear import 7 homolog (S. cerevisiae)	NIP7

230218_at	2.11		
1553599_a_at	2.11	synaptonemal complex protein 3	SYCP3
1559399_s_at	2.11	zinc finger, CCHC domain containing 10	ZCCHC10
242931_at	2.11		
212113_at	2.11		
239176_at	2.11		
204115_at	2.11	guanine nucleotide binding protein (G protein), gamma 11	GNG11
1554903_at	2.10		
203894_at	2.10	tubulin, gamma 2	TUBG2
1552794_a_at	2.10	zinc finger protein 547	ZNF547
223584_s_at	2.10	kelch repeat and BTB (POZ) domain containing 2	KBTBD2
231666_at	2.10	paired box gene 3 (Waardenburg syndrome 1)	PAX3
41037_at	2.09	TEA domain family member 4	TEAD4
1556226_at	2.09		
237138_at	2.09		
240158_at	2.09		
225072_at	2.09	zinc finger, CCHC domain containing 3	ZCCHC3
240533_at	2.09		
218580_x_at	2.09	aurora kinase A interacting protein 1	AURKAIP1
220647_s_at	2.09	coiled-coil-helix-coiled-coil-helix domain containing 8	CHCHD8
1569490_at	2.08	fibronectin type III domain containing 3B	FNDC3B
229819_at	2.08	alpha-1-B glycoprotein	A1BG
227997_at	2.08	interleukin 17 receptor D	IL17RD
221594_at	2.08		
232737_s_at	2.08	olfactory receptor, family 2, subfamily A, member 4	OR2A4
216555_at	2.08	chromosome 22 open reading frame 30	C22orf30
230647_at	2.08	transmembrane protein 53	TMEM53
1560590_s_at	2.08		
1570301_at	2.08		
243185_at	2.08		
204432_at	2.07	SRY (sex determining region Y)-box 12	SOX12
212955_s_at	2.07	polymerase (RNA) II (DNA directed) polypeptide I, 14.5kDa	POLR2I
215058_at	2.07		
229002_at	2.07	family with sequence similarity 69, member B	FAM69B
207255_at	2.07	leptin receptor	LEPR
228971_at	2.07		
201242_s_at	2.07	ATPase, Na+/K+ transporting, beta 1 polypeptide	ATP1B1
1561113_at	2.07		
1564580_at	2.07		
227099_s_at	2.07		
241608_at	2.07		
1563241_at	2.06		
224447_s_at	2.06	chromosome 17 open reading frame 37	C17orf37
217240_at	2.06	signal-regulatory protein beta 1#signal-regulatory protein delta	SIRPB1#SIRPD
213479_at	2.06	neuronal pentraxin II	NPTX2

218205_s_at	2.06	MAP kinase interacting serine/threonine kinase 2	MKNK2
200085_s_at	2.06	transcription elongation factor B (SIII), polypeptide 2 (18kDa, elongin B)	TCEB2
229099_at	2.06		
228233_at	2.06	FRAS1 related extracellular matrix 1	FREM1
240230_s_at	2.06		
228868_x_at	2.06	chromatin licensing and DNA replication factor 1	CDT1
206435_at	2.06	beta-1,4-N-acetyl-galactosaminyl transferase 1	B4GALNT1
239957_at	2.05	SET domain containing 5	SETD5
1570320_at	2.05		
1555097_a_at	2.05	prostaglandin F receptor (FP)	PTGFR
220073_s_at	2.05	pleckstrin homology domain containing, family G (with RhoGef domain) member 6	PLEKHG6
241992_at	2.05		
229684_s_at	2.05	zinc finger protein 644	ZNF644
216762_at	2.05	ankyrin repeat domain 15	ANKRD15
1562023_at	2.05		
208180_s_at	2.05	histone cluster 1, H4h	HIST1H4H
237649_at	2.05	COP9 constitutive photomorphogenic homolog subunit 4 (Arabidopsis)	COPS4
227032_at	2.05	plexin A2	PLXNA2
1559656_a_at	2.05		
201150_s_at	2.05	TIMP metallopeptidase inhibitor 3 (Sorsby fundus dystrophy, pseudoinflammatory)	TIMP3
202366_at	2.05	acyl-Coenzyme A dehydrogenase, C-2 to C-3 short chain	ACADS
239252_at	2.05		
243539_at	2.04		
1562921_at	2.04		
205434_s_at	2.04	AP2 associated kinase 1	AAK1
230262_at	2.04	ST8 alpha-N-acetyl-neuraminide alpha-2,8-sialyltransferase 3	ST8SIA3
236611_at	2.04		
1560346_at	2.04		
215633_x_at	2.04	leukocyte specific transcript 1	LST1
243997_x_at	2.04		
1563166_at	2.03		
244788_at	2.03		
238953_at	2.03		
1558595_at	2.03		
232980_at	2.03		
224397_s_at	2.02	transmembrane and tetratricopeptide repeat containing 1	TMTC1
230177_at	2.02		
228905_at	2.02		
1565389_s_at	2.02	glutamate receptor, metabotropic 5	GRM5
219888_at	2.02	sperm associated antigen 4	SPAG4
211022_s_at	2.02	alpha thalassemia/mental retardation syndrome X-linked (RAD54 homolog, S. cerevisiae)	ATRX
239143_x_at	2.01	ring finger protein 138	RNF138

222809_x_at	2.01	chromosome 14 open reading frame 65	C14orf65
206910_x_at	2.01	complement factor H-related 2	CFHR2
1558686_at	2.01		
221043_at	2.01		
208975_s_at	2.01	karyopherin (importin) beta 1	KPNB1
227556_at	2.01	ATPase, Na+/K+ transporting, beta 1 polypeptide	ATP1B1
231676_s_at	2.01		
226561_at	2.01		
236428_at	2.01		
1565875_at	2.01	nucleoporin 153kDa	NUP153
242766_at	2.00		
237365_at	2.00		
228698_at	2.00	SRY (sex determining region Y)-box 7	SOX7
1556175_at	2.00		
214289_at	0.50	proteasome (prosome, macropain) subunit, beta type, 1	PSMB1
224115_at	0.50		
212272_at	0.50	lipin 1	LPIN1
212385_at	0.50		
207978_s_at	0.50	nuclear receptor subfamily 4, group A, member 3	NR4A3
1566958_at	0.50		
1560874_at	0.50		
209541_at	0.50		
239876_at	0.50		
219701_at	0.50	tropomodulin 2 (neuronal)	TMOD2
1570177_at	0.50		
234605_at	0.50	CDC14 cell division cycle 14 homolog B (S. cerevisiae)	CDC14B
230499_at	0.50		
208862_s_at	0.50	catenin (cadherin-associated protein), delta 1	CTNND1
225217_s_at	0.50	bromodomain and PHD finger containing, 3	BRPF3
209722_s_at	0.50	serpin peptidase inhibitor, clade B (ovalbumin), member 9	SERPINB9
232954_at	0.50		
206385_s_at	0.50	ankyrin 3, node of Ranvier (ankyrin G)	ANK3
205798_at	0.49	interleukin 7 receptor	IL7R
215034_s_at	0.49	transmembrane 4 L six family member 1	TM4SF1
213744_at	0.49	attractin-like 1	ATRNL1
232262_at	0.49		
208047_s_at	0.49	NGFI-A binding protein 1 (EGR1 binding protein 1)	NAB1
1560963_a_at	0.49		
233999_s_at	0.49	tetratricopeptide repeat domain 26	TTC26
1555815_a_at	0.49	l(3)mbt-like 2 (Drosophila)	L3MBTL2
219722_s_at	0.49	glycerophosphodiester phosphodiesterase domain containing 3	GDPD3
215071_s_at	0.49	histone cluster 1, H1e#histone cluster 1, H2bd#histone cluster 1, H2be#histone cluster 1, H2bc#histone cluster 1, H4d	HIST1H1E#HIST1H2BD#HIST1H2BE#HIST1H2BC#HIST1H4D

224829_at	0.49	cytoplasmic polyadenylation element binding protein 4	CPEB4
205599_at	0.49	TNF receptor-associated factor 1	TRAF1
229437_at	0.49		
230401_at	0.49		
1569057_s_at	0.49	melanoma inhibitory activity family, member 3	MIA3
209878_s_at	0.49	v-rel reticuloendotheliosis viral oncogene homolog A, nuclear factor of kappa light polypeptide gene	RELA
207535_s_at	0.49	nuclear factor of kappa light polypeptide gene enhancer in B-cells 2 (p49/p100)	NFKB2
242814_at	0.49	serpin peptidase inhibitor, clade B (ovalbumin), member 9	SERPINB9
1552917_at	0.49	interleukin 29 (interferon, lambda 1)	IL29
206031_s_at	0.49	ubiquitin specific peptidase 5 (isopeptidase T)	USP5
237758_at	0.49		
221087_s_at	0.49	apolipoprotein L, 3	APOL3
208428_at	0.49	transporter 2, ATP-binding cassette, sub-family B (MDR/TAP)	TAP2
210147_at	0.49	ADP-ribosyltransferase 3	ART3
1556656_at	0.49		
220853_at	0.49	glycosyltransferase-like domain containing 1	GTDC1
1553055_a_at	0.49	schlafen family member 5	SLFN5
1569021_at	0.49	phosphoinositide-3-kinase, class 2, alpha polypeptide	PIK3C2A
232785_at	0.49	retrotransposon gag domain containing 1	RGAG1
221023_s_at	0.49	potassium voltage-gated channel, subfamily H (eag-related), member 6	KCNH6
211559_s_at	0.49	cyclin G2	CCNG2
1565882_at	0.49		
236419_at	0.49	transforming growth factor, beta receptor II (70/80kDa)	TGFBR2
238695_s_at	0.49	RAB39B, member RAS oncogene family	RAB39B
236590_at	0.48	ankyrin repeat domain 46	ANKRD46
207229_at	0.48	killer cell lectin-like receptor subfamily A, member 1	KLRA1
237208_at	0.48	WD repeat domain 61	WDR61
224832_at	0.48	dual specificity phosphatase 16	DUSP16
216589_at	0.48	tumor necrosis factor receptor superfamily, member 1B#vacuolar protein sorting 13 homolog D (S. cerevisiae)#vacuolar protein sorting 13 homolog D (S. cerevisiae)	TNFRSF1B#VPS13D#VPS13D
232158_x_at	0.48	NIPA-like domain containing 1	NPAL1
1564362_x_at	0.48		
1558139_at	0.48		
210538_s_at	0.48	baculoviral IAP repeat-containing 3	BIRC3
1557158_s_at	0.48	myeloid/lymphoid or mixed-lineage leukemia 3	MLL3
1570415_at	0.48	DEAD (Asp-Glu-Ala-Asp) box polypeptide 52	DDX52
1557267_s_at	0.48		
240982_at	0.48	cysteinyl-tRNA synthetase	CARS
1552696_at	0.48	non imprinted in Prader-Willi/Angelman syndrome 1	NIPA1

209239_at	0.48	nuclear factor of kappa light polypeptide gene enhancer in B-cells 1 (p105)	NFKB1
206134_at	0.48	ADAM-like, decysin 1	ADAMDEC1
216349_at	0.48	A kinase (PRKA) anchor protein 11#diacylglycerol kinase, eta	AKAP11#DGKH
240766_at	0.48	interleukin 23, alpha subunit p19	IL23A
228263_at	0.48	GRP1 (general receptor for phosphoinositides 1)-associated scaffold protein	GRASP
1555996_s_at	0.48	eukaryotic translation initiation factor 4A, isoform 2	EIF4A2
1563497_at	0.48	ubiquitin specific peptidase 25	USP25
238581_at	0.48	guanylate binding protein 5	GBP5
214985_at	0.48		
209169_at	0.48	glycoprotein M6B	GPM6B
207079_s_at	0.48	mediator of RNA polymerase II transcription, subunit 6 homolog (S. cerevisiae)	MED6
202319_at	0.48	SUMO1/sentrin specific peptidase 6	SENP6
225163_at	0.48	FERM domain containing 4A	FRMD4A
202340_x_at	0.48	nuclear receptor subfamily 4, group A, member 1	NR4A1
230842_at	0.48	fibroblast growth factor receptor 2 (bacteria-expressed kinase, keratinocyte growth factor receptor,	FGFR2
226288_s_at	0.48	neuroligin 2	NLGN2
241853_at	0.48		
223876_at	0.48	spermatogenesis associated 16	SPATA16
243178_at	0.48		
237489_at	0.48		
1554659_at	0.48	NIMA (never in mitosis gene a)-related kinase 4	NEK4
233808_at	0.48		
207108_s_at	0.48	Nipped-B homolog (Drosophila)	NIPBL
240184_at	0.48		
214972_at	0.48	meningioma expressed antigen 5 (hyaluronidase)	MGEA5
1566638_at	0.48		
214543_x_at	0.47	quaking homolog, KH domain RNA binding (mouse)	QKI
1553071_a_at	0.47	myozenin 3	MYOZ3
1559002_at	0.47		
1555884_at	0.47	proteasome (prosome, macropain) 26S subunit, non-ATPase, 6	PSMD6
206898_at	0.47	cadherin 19, type 2	CDH19
239751_at	0.47		
243196_s_at	0.47	TRAF-type zinc finger domain containing 1	TRAFD1
228197_at	0.47	chromosome 1 open reading frame 86	C1orf86
1559544_s_at	0.47		
238597_at	0.47		
232242_at	0.47		
1560855_at	0.47		
1559964_at	0.47		
234164_at	0.47	muscleblind-like 3 (Drosophila)	MBNL3
236122_at	0.47		

222793_at	0.47	DEAD (Asp-Glu-Ala-Asp) box polypeptide 58	DDX58
209052_s_at	0.47	Wolf-Hirschhorn syndrome candidate 1	WHSC1
206748_s_at	0.47	sperm associated antigen 9	SPAG9
1564878_at	0.47		
237861_at	0.47		
223094_s_at	0.47	ankylosis, progressive homolog (mouse)	ANKH
224149_x_at	0.47	sarcolemma associated protein	SLMAP
1552724_at	0.47		
1557584_at	0.47		
226439_s_at	0.47	neurobeachin	NBEA
233750_s_at	0.47	ring finger protein 2#chromosome 1 open reading frame 25#family with sequence similarity 129, member A	RNF2#C1orf25 #FAM129A
237753_at	0.47		
230688_at	0.47		
214132_at	0.47	ATP synthase, H+ transporting, mitochondrial F1 complex, gamma polypeptide 1	ATP5C1
1560995_s_at	0.47		
204348_s_at	0.47	adenylate kinase 3-like 1	AK3L1
226564_at	0.47	ZFAT zinc finger 1	ZFAT1
236961_at	0.46		
235285_at	0.46		
221477_s_at	0.46	superoxide dismutase 2, mitochondrial	SOD2
233292_s_at	0.46	ankyrin repeat and KH domain containing 1	ANKHD1
236422_at	0.46		
216294_s_at	0.46	KIAA1109	KIAA1109
236749_at	0.46	MAX binding protein	MNT
205746_s_at	0.46	ADAM metallopeptidase domain 17 (tumor necrosis factor, alpha, converting enzyme)	ADAM17
214895_s_at	0.46	ADAM metallopeptidase domain 10	ADAM10
234206_at	0.46		
1561536_at	0.46		
236944_at	0.46		
205114_s_at	0.46	chemokine (C-C motif) ligand 3	CCL3
1557438_at	0.46		
1560865_a_at	0.46		
207588_at	0.46	myelin transcription factor 2	MYT2
236280_at	0.46		
236676_at	0.46	NudC domain containing 3	NUDCD3
231958_at	0.46	chromosome 3 open reading frame 31	C3orf31
1554410_a_at	0.46	Williams-Beuren syndrome chromosome region 16	WBSCR16
230826_at	0.46	monocyte to macrophage differentiation-associated 2	MMD2
1556761_at	0.46		
236397_at	0.46		
233322_at	0.46	CD9 molecule	CD9
211113_s_at	0.46	ATP-binding cassette, sub-family G (WHITE), member 1	ABCG1
1570033_at	0.46	WD repeat domain, phosphoinositide interacting 2	WIPI2

203708_at	0.46	phosphodiesterase 4B, cAMP-specific (phosphodiesterase E4 dunce homolog, Drosophila)	PDE4B
227130_s_at	0.45	transducin-like enhancer of split 1 (E(sp1) homolog, Drosophila)	TLE1
231954_at	0.45		
1562107_at	0.45		
1560836_at	0.45		
236781_at	0.45		
225628_s_at	0.45	myeloid/lymphoid or mixed-lineage leukemia (trithorax homolog, Drosophila); translocated to, 6	MLLT6
210511_s_at	0.45	inhibin, beta A (activin A, activin AB alpha polypeptide)	INHBA
215183_at	0.45		
216296_at	0.45	clathrin, light chain (Lca)	CLTA
220710_at	0.45	chromosome 15 open reading frame 28	C15orf28
217701_x_at	0.45	alpha thalassemia/mental retardation syndrome X-linked (RAD54 homolog, S. cerevisiae)	ATRX
238658_at	0.45		
242270_at	0.45		
210072_at	0.45	chemokine (C-C motif) ligand 19	CCL19
1563524_a_at	0.45	chromosome 14 open reading frame 85	C14orf85
1554774_at	0.45	MYC induced nuclear antigen	MINA
239292_at	0.45		
202928_s_at	0.45	PHD finger protein 1	PHF1
235110_at	0.45	HRAS-like suppressor 3	HRASLS3
229213_at	0.45		
223798_at	0.45	solute carrier family 41, member 2	SLC41A2
243375_at	0.45		
1555854_at	0.45		
1553856_s_at	0.45	purinergic receptor P2Y, G-protein coupled, 10	P2RY10
232360_at	0.45	ets homologous factor	EHF
1566183_at	0.45		
241266_at	0.45		
1555364_at	0.45		
1557758_at	0.45		
1554027_a_at	0.45	solute carrier family 4, sodium bicarbonate cotransporter, member 4	SLC4A4
1553759_at	0.45	minichromosome maintenance deficient domain containing 1	MCMDC1
236623_at	0.45	chromosome 1 open reading frame 203	C1orf203
222139_at	0.45		
230614_at	0.45		
216746_at	0.44		
207426_s_at	0.44	tumor necrosis factor (ligand) superfamily, member 4 (tax-transcriptionally activated glycoprotein 1	TNFSF4
232593_at	0.44		
204533_at	0.44	chemokine (C-X-C motif) ligand 10	CXCL10
201796_s_at	0.44	valyl-tRNA synthetase	VARS
215610_at	0.44		

215495_s_at	0.44	sterile alpha motif domain containing 4A	SAMD4A
213776_at	0.44		
242999_at	0.44	Rho guanine nucleotide exchange factor (GEF) 7	ARHGEF7
1560347_at	0.44		
240169_at	0.44		
235633_at	0.44		
233101_at	0.44	myotubularin related protein 9	MTMR9
242067_at	0.44		
208212_s_at	0.44	anaplastic lymphoma kinase (Ki-1)	ALK
228574_at	0.44	transmembrane and tetratricopeptide repeat containing 2	TMTC2
236029_at	0.44	FAT tumor suppressor homolog 3 (Drosophila)	FAT3
218297_at	0.44	chromosome 10 open reading frame 97	C10orf97
210170_at	0.44	PDZ and LIM domain 3	PDLIM3
239958_at	0.44		
204330_s_at	0.44	mitochondrial ribosomal protein S12	MRPS12
1569917_at	0.44		
1560342_at	0.44		
207010_at	0.44	gamma-aminobutyric acid (GABA) A receptor, beta 1	GABRB1
243062_at	0.43	folliculin	FLCN
1563612_at	0.43		
205440_s_at	0.43	neuropeptide Y receptor Y1	NPY1R
240232_at	0.43	chromosome 3 open reading frame 1	C3orf1
216971_s_at	0.43	plectin 1, intermediate filament binding protein 500kDa	PLEC1
1562341_at	0.43		
203357_s_at	0.43	calpain 7	CAPN7
233630_at	0.43	CDP-diacylglycerol synthase (phosphatidate cytidylyltransferase) 2	CDS2
1557450_s_at	0.43		
215189_at	0.43	keratin 86	KRT86
1564627_at	0.43	DnaJ (Hsp40) homolog, subfamily C, member 13	DNAJC13
221684_s_at	0.43	nyctalopin	NYX
222031_at	0.43		
214408_s_at	0.43	ret finger protein-like 3 antisense#ret finger protein-like 3#F-box protein 7#chromosome 22 open reading frame 28#bactericidal/permeability-increasing protein-like 2	RFPL3S#RFPL3#FBXO7#C22orf28#BPIL2
1555408_at	0.43	B melanoma antigen family, member 2	BAGE2
1561097_at	0.43		
219647_at	0.43	popeye domain containing 2	POPDC2
1558561_at	0.43	histocompatibility (minor) 13	HM13
242379_at	0.43	LON peptidase N-terminal domain and ring finger 1	LONRF1
230706_s_at	0.43	calcium/calmodulin-dependent protein kinase II inhibitor 2	CAMK2N2
239013_at	0.43	SEC22 vesicle trafficking protein homolog C (S. cerevisiae)	SEC22C
223311_s_at	0.43	metastasis associated 1 family, member 3	MTA3

235884_at	0.43		
215945_s_at	0.43	tripartite motif-containing 2	TRIM2
244542_at	0.43		
216516_at	0.43		
202287_s_at	0.43	tumor-associated calcium signal transducer 2	TACSTD2
1557649_at	0.43	UDP-galactose-4-epimerase	GALE
233203_at	0.43	ropporin, rhophilin associated protein 1	ROPN1
1561488_at	0.43		
1562226_at	0.43		
215377_at	0.43	C-terminal binding protein 2	CTBP2
209766_at	0.43	peroxiredoxin 3	PRDX3
219975_x_at	0.42	oleoyl-ACP hydrolase	OLAH
237579_at	0.42		
220977_x_at	0.42	erythrocyte membrane protein band 4.1 like 5	EPB41L5
239750_x_at	0.42	VAMP (vesicle-associated membrane protein)-associated protein A, 33kDa	VAPA
215761_at	0.42	Dmx-like 2	DMXL2
1555759_a_at	0.42	chemokine (C-C motif) ligand 5	CCL5
233556_at	0.42		
1559393_at	0.42	aldehyde dehydrogenase 1 family, member L2	ALDH1L2
211862_x_at	0.42	CASP8 and FADD-like apoptosis regulator	CFLAR
232599_at	0.42	exocyst complex component 6	EXOC6
212292_at	0.42	solute carrier family 7 (cationic amino acid transporter, y+ system), member 1	SLC7A1
208241_at	0.42	neuregulin 1	NRG1
204103_at	0.42	chemokine (C-C motif) ligand 4	CCL4
1555394_at	0.42	phosphatidylinositol glycan anchor biosynthesis, class K	PIGK
240783_at	0.42		
237436_at	0.42		
1556058_s_at	0.42	spen homolog, transcriptional regulator (Drosophila)	SPEN
1568752_s_at	0.42	regulator of G-protein signalling 13	RGS13
239052_at	0.42	heterogeneous nuclear ribonucleoprotein D (AU-rich element RNA binding protein 1, 37kDa)	HNRPD
232086_at	0.42		
220564_at	0.42	chromosome 10 open reading frame 59	C10orf59
219584_at	0.42	phospholipase A1 member A	PLA1A
232355_at	0.42		
232345_at	0.42	chromosome 18 open reading frame 8	C18orf8
1559094_at	0.42	F-box protein 9	FBXO9
1554907_a_at	0.42	hydrocephalus inducing homolog (mouse)	HYDIN
239381_at	0.42	kallikrein-related peptidase 7	KLK7
235042_at	0.42	cardiomyopathy associated 1	CMYA1
221126_at	0.42		
1560153_at	0.42	Fraser syndrome 1	FRAS1
238231_at	0.42	nuclear transcription factor Y, gamma	NFYC
233622_x_at	0.41		
242234_at	0.41		
238745_at	0.41		
224566_at	0.41		
238032_at	0.41		

222719_s_at	0.41	platelet derived growth factor C	PDGFC
1556783_a_at	0.41		
1557866_at	0.41	chromosome 9 open reading frame 117	C9orf117
1558759_s_at	0.41	cytoplasmic linker associated protein 2	CLASP2
1552773_at	0.41	C-type lectin domain family 4, member D	CLEC4D
206172_at	0.41	interleukin 13 receptor, alpha 2	IL13RA2
207361_at	0.41	HMG-box transcription factor 1	HBP1
211287_x_at	0.41	colony stimulating factor 2 receptor, alpha, low-affinity (granulocyte-macrophage)	CSF2RA
210362_x_at	0.41	promyelocytic leukemia	PML
238466_at	0.41		
219614_s_at	0.41	solute carrier family 6 (proline IMINO transporter), member 20	SLC6A20
241107_at	0.41	ubiquitin specific peptidase 32	USP32
222329_x_at	0.41		
242299_at	0.41		
1554614_a_at	0.41	polypyrimidine tract binding protein 2	PTBP2
220460_at	0.41	solute carrier organic anion transporter family, member 1C1	SLCO1C1
241819_at	0.41	tumor necrosis factor (ligand) superfamily, member 8	TNFSF8
237124_at	0.41		
239041_at	0.41	histone cluster 1, H2ak	HIST1H2AK
201747_s_at	0.41	scaffold attachment factor B	SAFB
1562528_at	0.41		
243592_at	0.41	REV1 homolog (S. cerevisiae)	REV1
205540_s_at	0.41	Ras-related GTP binding B	RRAGB
1558673_s_at	0.40	zinc finger protein 77	ZNF77
229219_s_at	0.40	N-terminal asparagine amidase	NTAN1
214020_x_at	0.40	integrin, beta 5	ITGB5
218850_s_at	0.40	LIM domains containing 1	LIMD1
243929_at	0.40	zinc finger protein 533	ZNF533
200628_s_at	0.40	tryptophanyl-tRNA synthetase	WARS
242174_at	0.40	zinc finger and BTB domain containing 10	ZBTB10
234871_at	0.40	G protein-coupled receptor 98	GPR98
238221_at	0.40		
225239_at	0.40		
213219_at	0.40	adenylate cyclase 2 (brain)	ADCY2
236873_at	0.40		
242906_at	0.40		
1568882_at	0.40	leucine rich repeat containing 51	LRRC51
205971_s_at	0.40	chymotrypsinogen B1	CTRB1
1556082_a_at	0.40		
1563596_at	0.40		
214671_s_at	0.40	active BCR-related gene	ABR
229072_at	0.40		
1558356_at	0.40	uveal autoantigen with coiled-coil domains and ankyrin repeats	UACA
1555870_at	0.40	ring finger protein 207	RNF207
227913_at	0.40	exosome component 3	EXOSC3
1561609_at	0.40		
220275_at	0.40	CUB and zona pellucida-like domains 1	CUZD1

237953_at	0.40	dipeptidyl-peptidase 4 (CD26, adenosine deaminase complexing protein 2)	DPP4
203389_at	0.40	kinesin family member 3C	KIF3C
221642_at	0.39	three prime repair exonuclease 1	TREX1
236094_at	0.39	transcription factor 7-like 2 (T-cell specific, HMG-box)	TCF7L2
204667_at	0.39	forkhead box A1	FOXA1
221489_s_at	0.39	sprouty homolog 4 (Drosophila)	SPRY4
242237_at	0.39	THO complex 7 homolog (Drosophila)	THOC7
220958_at	0.39	unc-51-like kinase 4 (C. elegans)	ULK4
215670_s_at	0.39	SCAN domain containing 2	SCAND2
237733_at	0.39		
228837_at	0.39		
216716_at	0.39	ABO blood group (transferase A, alpha 1-3-N-acetylgalactosaminyltransferase; transferase B, alpha 1-	ABO
211171_s_at	0.39	phosphodiesterase 10A	PDE10A
208263_at	0.39		
239285_at	0.39	jumonji domain containing 2C	JMJD2C
206685_at	0.39	HLA complex group 4	HCG4
1558641_at	0.39		
202285_s_at	0.39	tumor-associated calcium signal transducer 2	TACSTD2
227126_at	0.39		
1557731_at	0.39		
207963_at	0.39	chromosome 6 open reading frame 54	C6orf54
211338_at	0.38	interferon, alpha 2	IFNA2
227283_at	0.38		
243290_at	0.38	WW and C2 domain containing 1	WWC1
244858_at	0.38	TGFB-induced factor homeobox 1	TGIF1
244005_at	0.38	glypican 5	GPC5
207665_at	0.38	ADAM metallopeptidase domain 21	ADAM21
228981_at	0.38	transmembrane protein 169	TMEM169
210832_x_at	0.38	prostaglandin E receptor 3 (subtype EP3)	PTGER3
234403_at	0.38	ilvB (bacterial acetolactate synthase)-like#synapse defective 1, Rho GTPase, homolog 1 (C. elegans)#olfactory receptor, family 1, subfamily I, member 1#olfactory receptor, family 10, subfamily B, member 1 pseudogene	ILVBL#SYDE1#OR1I1#OR10B1P
222038_s_at	0.38	non-metastatic cells 1, protein (NM23A) expressed in	NME1
209677_at	0.38	protein kinase C, iota	PRKCI
1568887_at	0.38		
1557719_at	0.38	phosphatidylinositol-3-phosphate/phosphatidylinositol 5-kinase, type III	PIP5K3
204574_s_at	0.38	matrix metallopeptidase 19	MMP19
216472_at	0.38		
222470_s_at	0.38	chromosome 20 open reading frame 44	C20orf44
210918_at	0.38		
208337_s_at	0.38	nuclear receptor subfamily 5, group A, member 2	NR5A2
234384_at	0.38		

206738_at	0.37	apolipoprotein C-IV	APOC4
1563719_a_at	0.37		
244274_at	0.37		
220282_at	0.37	resistance to inhibitors of cholinesterase 3 homolog (C. elegans)	RIC3
1569813_at	0.37	striatin, calmodulin binding protein	STRN
240921_at	0.37		
206942_s_at	0.37	pro-melanin-concentrating hormone	PMCH
217388_s_at	0.37	kynureninase (L-kynurenine hydrolase)	KYNU
237943_at	0.37	transmembrane and coiled-coil domain family 1	TMCC1
1557583_at	0.37	suppression of tumorigenicity 18 (breast carcinoma) (zinc finger protein)	ST18
207671_s_at	0.37	bestrophin 1	BEST1
240267_at	0.37	synaptotagmin VI	SYT6
241619_at	0.37		
210544_s_at	0.37	aldehyde dehydrogenase 3 family, member A2	ALDH3A2
235616_at	0.37	teashirt family zinc finger 2	TSHZ2
234668_at	0.37		
224438_at	0.37		
241127_at	0.37	placenta-specific 8	PLAC8
224762_at	0.37	serine incorporator 2	SERINC2
1554840_at	0.37		
213419_at	0.37	amyloid beta (A4) precursor protein-binding, family B, member 2 (Fe65-like)	APBB2
208481_at	0.37	ankyrin repeat and SOCS box-containing 4	ASB4
243065_at	0.37		
1563133_at	0.37		
204942_s_at	0.37	aldehyde dehydrogenase 3 family, member B2	ALDH3B2
1559696_at	0.36		
211302_s_at	0.36	phosphodiesterase 4B, cAMP-specific (phosphodiesterase E4 dunce homolog, Drosophila)	PDE4B
1559053_at	0.36		
210029_at	0.36	indoleamine-pyrrole 2,3 dioxygenase	INDO
237387_at	0.36	actin related protein 2/3 complex, subunit 5, 16kDa	ARPC5
224106_at	0.36		
1560652_at	0.36		
244320_at	0.36	NHL repeat containing 2	NHLRC2
233784_at	0.36		
241256_at	0.36		
207593_at	0.36	ATP-binding cassette, sub-family G (WHITE), member 4	ABCG4
244058_at	0.36	chromosome 10 open reading frame 72	C10orf72
1564822_at	0.36		
1560881_a_at	0.36	chromosome 21 open reading frame 22	C21orf22
231435_at	0.36	chromosome 7 open reading frame 34	C7orf34
236409_at	0.35		
239525_at	0.35		
236861_at	0.35	THO complex 7 homolog (Drosophila)	THOC7
212448_at	0.35	neural precursor cell expressed, developmentally down-regulated 4-like	NEDD4L

227169_at	0.35	DnaJ (Hsp40) homolog, subfamily C, member 18	DNAJC18
240847_at	0.35	reelin	RELN
238048_at	0.35	cytoplasmic linker associated protein 2	CLASP2
238420_at	0.35		
1560382_at	0.35	GRB2-associated binding protein 1	GAB1
215213_at	0.35	nucleoporin 54kDa	NUP54
224272_at	0.35	Rac GTPase activating protein 1 pseudogene	RACGAP1P
238186_at	0.35		
207216_at	0.35	tumor necrosis factor (ligand) superfamily, member 8	TNFSF8
220863_at	0.35	major intrinsic protein of lens fiber	MIP
234458_at	0.35	alanyl (membrane) aminopeptidase (aminopeptidase N, aminopeptidase M, microsomal aminopeptidase, CD1	ANPEP
34063_at	0.35	RecQ protein-like 5	RECQL5
1558790_s_at	0.35	chromosome 8 open reading frame 77	C8orf77
243932_at	0.35		
230810_at	0.35	jumonji domain containing 4	JMJD4
1564807_at	0.35		
224140_at	0.34	nasopharyngeal carcinoma, down-regulated 1	NPCDR1
217133_x_at	0.34	cytochrome P450, family 2, subfamily B, polypeptide 6	CYP2B6
215658_at	0.34		
207706_at	0.34	Usher syndrome 2A (autosomal recessive, mild)	USH2A
1562028_at	0.34	cyclin D3	CCND3
1570541_s_at	0.34		
208181_at	0.34	histone cluster 1, H4h	HIST1H4H
224081_at	0.34		
212592_at	0.34		
232224_at	0.34	mannan-binding lectin serine peptidase 1 (C4/C2 activating component of Ra-reactive factor)	MASP1
238316_at	0.34	zinc finger protein 567	ZNF567
206839_at	0.34	chromosome 22 open reading frame 31	C22orf31
1565837_at	0.34		
216135_at	0.34	IQ motif containing K	IQCK
202357_s_at	0.34	complement factor B	CFB
242542_at	0.34	phosphatase and actin regulator 2	PHACTR2
205242_at	0.34	chemokine (C-X-C motif) ligand 13 (B-cell chemoattractant)	CXCL13
211514_at	0.34	receptor interacting protein kinase 5	RIPK5
1563226_at	0.33		
235321_at	0.33		
1557717_at	0.33		
207470_at	0.33		
205076_s_at	0.33	myotubularin related protein 11	MTMR11
222831_at	0.33	SAP30-like	SAP30L
215371_at	0.33	cofactor required for Sp1 transcriptional activation, subunit 8, 34kDa	CRSP8
233227_at	0.33	KIAA1109	KIAA1109
1569491_at	0.33		

211617_at	0.33	aldolase A, fructose-bisphosphate pseudogene 2	ALDOAP2
233154_at	0.33		
1558590_at	0.33	methyltransferase 11 domain containing 1	METT11D1
239434_at	0.33		
232480_at	0.33		
234091_at	0.33		
235485_at	0.33	WD repeat domain 44	WDR44
241597_at	0.33	arginine-glutamic acid dipeptide (RE) repeats	RERE
219671_at	0.33	hippocalcin like 4	HPCAL4
216765_at	0.33	mitogen-activated protein kinase kinase 5	MAP2K5
1554234_at	0.33	katanin p60 subunit A-like 2	KATNAL2
233770_at	0.33		
1557566_at	0.32		
222219_s_at	0.32	transducin-like enhancer of split 2 (E(sp1) homolog, Drosophila)#transducin-like enhancer of split 6 (E(sp1) homolog, Drosophila)	TLE2#TLE6
234082_at	0.32		
234989_at	0.32		
1570531_at	0.32		
1560676_at	0.32		
1560117_at	0.32	abhydrolase domain containing 1	ABHD1
235111_at	0.32		
234138_at	0.32		
232607_at	0.32		
233267_at	0.32	selenium binding protein 1	SELENBP1
228447_at	0.32	chromosome X and Y open reading frame 3	CXYorf3
206940_s_at	0.32	POU domain, class 4, transcription factor 1	POU4F1
218835_at	0.32	surfactant, pulmonary-associated protein A2	SFTPA2
216807_at	0.32	KIAA1751	KIAA1751
208077_at	0.32	chromosome 9 open reading frame 38	C9orf38
207991_x_at	0.31	acrosomal vesicle protein 1	ACRV1
230143_at	0.31	ring finger protein 165	RNF165
225299_at	0.31	myosin VB	MYO5B
1553147_at	0.31	RAN binding protein 3-like	RANBP3L
1552325_at	0.31	coiled-coil domain containing 11	CCDC11
243748_at	0.31	eukaryotic translation initiation factor 4E family member 3	EIF4E3
1553668_at	0.31	leucine-rich repeats and calponin homology (CH) domain containing 3	LRCH3
223618_at	0.31	formin 2	FMN2
232122_s_at	0.31	ventricular zone expressed PH domain homolog 1 (zebrafish)	VEPH1
206286_s_at	0.31	teratocarcinoma-derived growth factor 1	TDGF1
1558273_a_at	0.31		
211361_s_at	0.31	serpin peptidase inhibitor, clade B (ovalbumin), member 13	SERPINB13
208324_at	0.30	A kinase (PRKA) anchor protein 13	AKAP13
1570246_at	0.30		
211354_s_at	0.30	leptin receptor	LEPR
216465_at	0.30		

215236_s_at	0.30	phosphatidylinositol binding clathrin assembly protein	PICALM
1557742_a_at	0.30		
229004_at	0.30		
206858_s_at	0.30	homeobox C6	HOXC6
203473_at	0.30	solute carrier organic anion transporter family, member 2B1	SLCO2B1
229147_at	0.30		
236161_at	0.30		
238370_x_at	0.30		
221052_at	0.30	tudor and KH domain containing	TDRKH
236065_at	0.30		
240821_at	0.30		
217525_at	0.30	olfactomedin-like 1	OLFML1
239712_at	0.30	chromosome 9 open reading frame 93	C9orf93
1563386_at	0.30		
1553776_at	0.29	ubiquitin-conjugating enzyme E2U (putative)	UBE2U
236786_at	0.29		
211343_s_at	0.29	collagen, type XIII, alpha 1	COL13A1
211317_s_at	0.29	CASP8 and FADD-like apoptosis regulator	CFLAR
1568822_at	0.29	GTP binding protein 5 (putative)	GTPBP5
1558745_at	0.29		
235304_at	0.29	zinc finger protein 573	ZNF573
242791_at	0.29	F-box protein 3	FBXO3
1555516_at	0.29	forkhead box P2	FOXP2
1562169_at	0.29		
1556492_a_at	0.29		
1569525_s_at	0.29		
1568783_at	0.29	splicing factor, arginine/serine-rich 12	SFRS12
1569203_at	0.29	chemokine (C-X-C motif) ligand 2	CXCL2
233826_at	0.29		
240744_at	0.29	carboxypeptidase A5	CPA5
233627_at	0.28		
238790_at	0.28		
215704_at	0.28	filaggrin#null	FLG#null
1568768_s_at	0.28	brain and reproductive organ-expressed (TNFRSF1A modulator)	BRE
1565861_at	0.28		
232880_at	0.28		
1557765_at	0.28		
240703_s_at	0.28		
241699_at	0.28		
241732_at	0.28	CDK5 regulatory subunit associated protein 1-like 1	CDKAL1
216439_at	0.28	tyrosine kinase, non-receptor, 2	TNK2
223793_at	0.28	KIAA0515	KIAA0515
1555542_at	0.28		
239759_at	0.28		
1563913_at	0.28		
232222_at	0.28	chromosome 18 open reading frame 49	C18orf49
217315_s_at	0.28	kallikrein-related peptidase 13	KLK13
244698_at	0.28	CMT1A duplicated region transcript 4	CDRT4
238882_at	0.28		

237668_at	0.28		
238868_at	0.28	uveal autoantigen with coiled-coil domains and ankyrin repeats	UACA
1566602_at	0.28	RNA pseudouridylate synthase domain containing 3	RPUSD3
231683_at	0.28	glycine-N-acyltransferase	GLYAT
236586_at	0.27		
1566094_at	0.27		
238461_at	0.27	eukaryotic translation initiation factor 4E family member 3	EIF4E3
214627_at	0.27	eosinophil peroxidase	EPX
237808_at	0.27	ST7 overlapping transcript 2 (antisense non-coding RNA)	ST7OT2
1562514_at	0.27		
215282_at	0.27	anaphase promoting complex subunit 13	ANAPC13
207303_at	0.27	phosphodiesterase 1C, calmodulin-dependent 70kDa	PDE1C
229669_at	0.27		
241740_at	0.27	cAMP responsive element modulator	CREM
240329_at	0.27		
216043_x_at	0.27		
1555465_at	0.27	mucolipin 2	MCOLN2
234608_at	0.27	laminin, alpha 3	LAMA3
210190_at	0.26	syntaxin 11	STX11
213834_at	0.26	IQ motif and Sec7 domain 3	IQSEC3
1553558_at	0.26	taste receptor, type 2, member 41	TAS2R41
236810_at	0.26		
1555488_at	0.26		
217024_x_at	0.26	SEC14-like 2 (S. cerevisiae)#protein tyrosine phosphatase, non-receptor type substrate 1-like#null#SEC14-like 3 (S. cerevisiae)#SEC14-like 4 (S. cerevisiae)#syndecan 4 pseudogene#null	SEC14L2#PTPNS1L#null#SEC14L3#SEC14L4#SDC4P#null
1564405_at	0.26		
1556465_at	0.26		
1563591_at	0.26		
242724_x_at	0.26	nuclear receptor subfamily 6, group A, member 1	NR6A1
1563135_at	0.26		
1557099_at	0.26		
232457_at	0.25		
234165_at	0.25	prostaglandin D2 receptor (DP)	PTGDR
215298_at	0.25		
227721_at	0.25	C3 and PZP-like, alpha-2-macroglobulin domain containing 8	CPAMD8
236987_at	0.25	alpha-kinase 2	ALPK2
1562854_at	0.25		
238428_at	0.25	potassium inwardly-rectifying channel, subfamily J, member 15	KCNJ15
220545_s_at	0.25		
1569630_a_at	0.25	RUN and FYVE domain containing 2	RUFY2
1559412_at	0.25		
229222_at	0.25		

238414_at	0.25		
229701_at	0.25	DnaJ (Hsp40) homolog, subfamily C, member 9	DNAJC9
1555133_at	0.25	family with sequence similarity 9, member A	FAM9A
238097_at	0.25		
213148_at	0.25		
214489_at	0.25	follicle stimulating hormone, beta polypeptide	FSHB
1569289_at	0.25	basic, immunoglobulin-like variable motif containing	BIVM
216566_at	0.25		
207103_at	0.25	potassium voltage-gated channel, Shal-related subfamily, member 2	KCND2
205365_at	0.24	homeobox B6	HOXB6
1560241_at	0.24		
210078_s_at	0.24	potassium voltage-gated channel, shaker-related subfamily, beta member 1	KCNAB1
1560407_at	0.24	MAP/microtubule affinity-regulating kinase 1	MARK1
203700_s_at	0.24	deiodinase, iodothyronine, type II	DIO2
241760_x_at	0.24		
231485_at	0.24		
233352_at	0.24		
241676_x_at	0.24		
207859_s_at	0.24	cholinergic receptor, nicotinic, beta 3	CHRNB3
1560692_at	0.24		
1565608_at	0.24		
1555729_a_at	0.24	CD209 molecule	CD209
1562091_at	0.23		
240586_at	0.23		
227062_at	0.23		
1569780_at	0.23		
1552895_a_at	0.23	chromosome 21 open reading frame 99	C21orf99
1554400_at	0.23	t-complex-associated-testis-expressed 3	TCTE3
240088_at	0.23	phosphodiesterase 5A, cGMP-specific	PDE5A
204679_at	0.23	potassium channel, subfamily K, member 1	KCNK1
239904_at	0.23	phospholipase C, epsilon 1	PLCE1
234103_at	0.23		
234395_at	0.23	olfactory receptor, family 4, subfamily A, member 1 pseudogene	OR4A1P
220901_at	0.23	G protein-coupled receptor 157	GPR157
215639_at	0.23	SH2 domain containing 3C	SH2D3C
242481_at	0.23		
205893_at	0.23	neuroligin 1	NLGN1
228237_at	0.23	pappalysin 2	PAPPA2
208055_s_at	0.23	hect domain and RLD 4	HERC4
234019_at	0.23		
224241_s_at	0.22		
235201_at	0.22		
1555352_at	0.22	forkhead box P2	FOXP2
241784_x_at	0.22		
213756_s_at	0.22	heat shock transcription factor 1	HSF1
241789_at	0.22		
210724_at	0.22	egf-like module containing, mucin-like, hormone receptor-like 3	EMR3

205279_s_at	0.22	glycine receptor, beta	GLRB
220786_s_at	0.22	solute carrier family 38, member 4	SLC38A4
1555199_at	0.22	golgi SNAP receptor complex member 1	GOSR1
239962_at	0.22		
215475_at	0.22		
243146_at	0.22	adrenergic, alpha-1A-, receptor	ADRA1A
234042_at	0.22	taste receptor, type 2, member 45	TAS2R45
237998_at	0.22	chromosome 20 open reading frame 94	C20orf94
1561303_at	0.22		
1570338_at	0.22		
221638_s_at	0.22	syntaxin 16	STX16
239023_at	0.22		
205399_at	0.21	doublecortin and CaM kinase-like 1	DCAMKL1
219746_at	0.21	D4, zinc and double PHD fingers, family 3	DPF3
205815_at	0.21	regenerating islet-derived 3 alpha	REG3A
208366_at	0.21	protocadherin 11 X-linked	PCDH11X
215868_x_at	0.21		
205143_at	0.21	chondroitin sulfate proteoglycan 3 (neurocan)	CSPG3
1569736_at	0.21		
242623_x_at	0.21		
1559256_at	0.21	membrane associated guanylate kinase, WW and PDZ domain containing 1	MAGI1
1555717_at	0.21		
234134_at	0.21		
210745_at	0.21	one cut domain, family member 1	ONECUT1
239846_at	0.21		
214871_x_at	0.21		
1557126_a_at	0.21	phospholipase D1, phosphatidylcholine-specific	PLD1
1561638_at	0.21		
1554140_at	0.20	WD repeat domain 78	WDR78
1560222_at	0.20		
205549_at	0.20	Purkinje cell protein 4	PCP4
1564690_at	0.20		
1561877_at	0.20		
1557782_s_at	0.20	chromosome 21 open reading frame 49	C21orf49
1566862_at	0.20		
1565668_at	0.20		
1566046_at	0.20		
216813_at	0.20		
1569849_at	0.20		
244608_at	0.20		
233353_at	0.20		
231734_at	0.20	retinol binding protein 2, cellular	RBP2
235899_at	0.20	carbonic anhydrase XIII	CA13
213411_at	0.20		
215290_at	0.20		
220817_at	0.20	transient receptor potential cation channel, subfamily C, member 4	TRPC4
232039_at	0.20	KIAA1383	KIAA1383
1555011_at	0.19	zinc finger, FYVE domain containing 16	ZFYVE16
233419_at	0.19		
1557094_at	0.19		

224533_s_at	0.19	interferon, alpha-inducible protein 6	IFI6
206579_at	0.19	zinc finger protein 192	ZNF192
213968_at	0.19	tetraspanin 5	TSPAN5
1555671_at	0.19	islet cell autoantigen 1,69kDa-like	ICA1L
214569_at	0.19	interferon, alpha 5	IFNA5
234963_s_at	0.19	fatty acid 2-hydroxylase#mixed lineage kinase domain-like	FA2H#MLKL
214561_at	0.19	leukocyte immunoglobulin-like receptor pseudogene 2	LILRP2
224091_at	0.19		
1555456_at	0.19		
211842_s_at	0.19	solute carrier family 24 (sodium/potassium/calcium exchanger), member 1	SLC24A1
224499_s_at	0.19	activation-induced cytidine deaminase	AICDA
208266_at	0.19	chromosome 8 open reading frame 17	C8orf17
232885_at	0.19		
214631_at	0.19	zinc finger and BTB domain containing 33	ZBTB33
228325_at	0.18		
239428_at	0.18	RAB1A, member RAS oncogene family	RAB1A
238375_at	0.18		
1566337_x_at	0.18		
1559895_x_at	0.18		
207481_at	0.18		
236278_at	0.18		
1552258_at	0.18		
239956_at	0.18		
1561516_at	0.18		
237244_at	0.18		
234678_at	0.18	keratin associated protein 4-3	KRTAP4-3
1569569_x_at	0.18		
217206_at	0.17		
216811_at	0.17		
220660_at	0.17	chromosome 9 open reading frame 27	C9orf27
218978_s_at	0.17	solute carrier family 25, member 37	SLC25A37
211667_x_at	0.17		
233118_at	0.17		
222189_at	0.17		
214088_s_at	0.17	fucosyltransferase 3 (galactoside 3(4)-L-fucosyltransferase, Lewis blood group)	FUT3
221404_at	0.17	interleukin 1 family, member 6 (epsilon)	IL1F6
1554797_at	0.17	synaptotagmin XVI	SYT16
206543_at	0.17	SWI/SNF related, matrix associated, actin dependent regulator of chromatin, subfamily a, member 2	SMARCA2
1561474_at	0.17		
1556624_at	0.17		
238579_at	0.17	chromosome 9 open reading frame 85	C9orf85
234148_at	0.17		
231433_at	0.17		
235382_at	0.17		
220870_at	0.17		
1553194_at	0.16	neuronal growth regulator 1	NEGR1

210941_at	0.16	BH-protocadherin (brain-heart)	PCDH7
234852_at	0.16	T cell receptor alpha locus#T cell receptor delta variable 1#T cell receptor alpha variable 36/delta variable 7#T cell receptor alpha variable 35#T cell receptor alpha variable 34#T cell receptor alpha variable 33#T cell receptor alpha variable 32#T cell receptor alpha variable 31#T cell receptor alpha variable 30#T cell receptor alpha variable 29/delta variable 5#T cell receptor alpha variable 28#T cell receptor alpha variable 27#T cell receptor alpha variable 26-2#T cell receptor alpha variable 26-1#T cell receptor alpha variable 25#T cell receptor alpha variable 24#T cell receptor alpha variable 23/delta variable 6#T cell receptor alpha variable 22#T cell receptor alpha variable 21#T cell receptor alpha variable 20#T cell receptor alpha variable 19#T cell receptor alpha variable 18#T cell receptor alpha variable 17#T cell receptor alpha variable 8-7	TRA@#TRDV 1#TRAV36DV 7#TRAV35#TR AV34#TRAV33 #TRAV32#TRA V31#TRAV30# TRAV29DV5# TRAV28#TRA V27#TRAV26- 2#TRAV26- 1#TRAV25#TR AV24#TRAV23 DV6#TRAV22 #TRAV21#TRA V20#TRAV19# TRAV18#TRA V17#TRAV8-7
241025_at	0.16	UTP6, small subunit (SSU) processome component, homolog (yeast)	UTP6
234739_at	0.16		
237026_at	0.16	sorbin and SH3 domain containing 1	SORBS1
1557107_at	0.16		
243204_at	0.16		
1559035_a_at	0.16	aryl hydrocarbon receptor	AHR
215079_at	0.16		
1570250_at	0.16		
211489_at	0.16	adrenergic, alpha-1A-, receptor	ADRA1A
235704_at	0.16	DAZ associated protein 2	DAZAP2
1563519_at	0.16		
234271_at	0.15	otopetrin 2	OTOP2
233863_at	0.15	castor zinc finger 1	CASZ1
241830_at	0.15	chromosome 20 open reading frame 112	C20orf112
1562644_at	0.15	methylenetetrahydrofolate dehydrogenase (NADP+ dependent) 2-like	MTHFD2L
223427_s_at	0.15	erythrocyte membrane protein band 4.1 like 4B	EPB41L4B
209436_at	0.15	spondin 1, extracellular matrix protein	SPON1
233446_at	0.15	one cut domain, family member 2	ONECUT2
235654_at	0.15		
207928_s_at	0.15	glycine receptor, alpha 3	GLRA3
1559149_at	0.15		
231397_at	0.14		
1566551_at	0.14		
1563032_at	0.14		
231813_s_at	0.14	family with sequence similarity 104, member A	FAM104A
1555103_s_at	0.14	fibroblast growth factor 7 (keratinocyte growth factor)	FGF7
1560184_at	0.14	transmembrane and coiled-coil domains 5	TMCO5
227851_s_at	0.14		
1559889_at	0.13		

240321_at	0.13		
231789_at	0.13	protocadherin beta 15	PCDHB15
1555777_at	0.13	periostin, osteoblast specific factor	POSTN
1557280_s_at	0.13		
215646_s_at	0.13	chondroitin sulfate proteoglycan 2 (versican)	CSPG2
1554909_at	0.13	coiled-coil domain containing 36	CCDC36
216740_at	0.13		
243485_at	0.13		
239517_at	0.13		
240658_at	0.12		
219115_s_at	0.12	interleukin 20 receptor, alpha	IL20RA
221170_at	0.12	histamine receptor H4	HRH4
234455_at	0.12	zinc finger protein 1 homolog (mouse)	ZFP1
240026_x_at	0.12		
204631_at	0.12	myosin, heavy chain 2, skeletal muscle, adult	MYH2
240825_at	0.12		
231426_at	0.11		
234514_at	0.11	eukaryotic translation elongation factor 1 alpha 2#potassium voltage-gated channel, KQT-like subfamily, member 2#potassium voltage-gated channel, KQT-like subfamily, member 2#PTK6 protein tyrosine kinase 6#src-related kinase lacking C-terminal regulatory tyrosine and N-terminal myristylation sites#chromosome 20 open reading frame 195#chromosome 20 open reading frame 149#null	EEF1A2#KCNQ2#KCNQ2#PTK6#SRMS#C20orf195#C20orf149#null
243941_at	0.11		
232786_at	0.11	component of oligomeric golgi complex 6	COG6
1566515_at	0.11	CWF19-like 2, cell cycle control (S. pombe)	CWF19L2
214593_at	0.11	protein inhibitor of activated STAT, 2	PIAS2
1553418_a_at	0.11	contactin associated protein-like 5	CNTNAP5
207638_at	0.11	protease, serine, 7 (enterokinase)	PRSS7
215294_s_at	0.10	SWI/SNF related, matrix associated, actin dependent regulator of chromatin, subfamily a, member 1	SMARCA1
221451_s_at	0.10	olfactory receptor, family 2, subfamily W, member 1	OR2W1
232450_at	0.10		
226701_at	0.10	gap junction protein, alpha 5, 40kDa	GJA5
1563389_at	0.09		
204926_at	0.09	inhibin, beta A (activin A, activin AB alpha polypeptide)	INHBA
240828_at	0.09		
1561553_at	0.09		
217091_at	0.09		
1558867_at	0.08	dermatan sulfate epimerase	DSE
1552675_at	0.08	DnaJ (Hsp40) homolog, subfamily B, member 7	DNAJB7
241383_at	0.08		
231881_at	0.07		
1560773_at	0.07		
1560349_at	0.07		
221414_s_at	0.07	defensin, beta 126	DEFB126

229592_at	0.06		
1561864_at	0.06		
1559653_at	0.06		
205532_s_at	0.06	cadherin 6, type 2, K-cadherin (fetal kidney)	CDH6
1555731_a_at	0.06	adaptor-related protein complex 1, sigma 3 subunit	AP1S3
1568644_at	0.06	zinc finger protein 208	ZNF208
1559077_at	0.05	ABI gene family, member 3 (NESH) binding protein	ABI3BP
208394_x_at	0.04	endothelial cell-specific molecule 1	ESM1
1558387_at	0.02		
