# Supplementary material for: In Vitro Transformation of Primary Human CD34+ Cells by AML Fusion Oncogenes: Early Gene Expression Profiling Reveals Possible Drug Target in AML
Source: PLoS One. 2010 Aug 27;5(8):e12464. doi: 10.1371/journal.pone.0012464 (PMC2929205; doi:10.1371/journal.pone.0012464)
Supplement: Table S20 — Genes deregulated by MLL-AF9 3 days after transduction. Primary human CD34+ cells were retrovirally transduced with either control MSCV-IRES-GFP vector or vector expressing MLL-AF9 and sorted for GFP positivity. Total RNA was extracted 3 days after transduction and subjected to microarray analysis. Microarray data were analyzed by SAM as described in Materials and Methods. Significantly deregulated genes are listed and the false discovery rate (FDR) is shown. (0.35 MB PDF) [file pone.0012464.s020.pdf]

Table S20. Genes deregulated by MLL-AF9 at 3 d detected by SAM **FDR = 9.3%**

Probe set ID	Fold Change	Gene Name	Gene Symbol
206367_at	57.18	renin	REN
1570246_at	39.99		
1564580_at	39.93		
1557881_at	39.56	chromosome 10 open reading frame 44	C10orf44
233096_at	28.23	KIAA1109	KIAA1109
215301_at	27.23		
1566947_at	25.73		
242118_x_at	24.18		
1565742_at	24.00		
205278_at	23.93	glutamate decarboxylase 1 (brain, 67kDa)	GAD1
1569459_a_at	23.55		
1557057_a_at	22.92		
1555498_at	21.01		
1556026_at	20.52	iduronate 2-sulfatase (Hunter syndrome)	IDS
234655_at	20.34	annexin A2	ANXA2P2
1558392_at	19.93	spectrin repeat containing, nuclear envelope 2	SYNE2
243478_at	19.80		
237677_at	19.02	transmembrane protein 110	TMEM110
215924_at	17.22		
238868_at	16.64	uveal autoantigen with coiled-coil domains and ankyrin repeats	UACA
1560851_at	16.27	chromosome 10 open reading frame 136	C10orf136
214247_s_at	16.19	dickkopf homolog 3 (Xenopus laevis)	DKK3
242904_x_at	16.09		
244403_at	16.06	crumbs homolog 1 (Drosophila)	CRB1
240068_at	16.03	chromosome 21 open reading frame 130	C21orf130
223315_at	15.94	netrin 4	NTN4
241961_at	15.75		
1562400_at	15.72		
204888_s_at	15.54	neuralized homolog (Drosophila)	NEURL
229559_at	14.48		
211480_s_at	14.01	solute carrier organic anion transporter family, member 1A2	SLCO1A2
1557597_at	13.89		
223812_at	13.74		
241888_at	13.63		
243489_at	13.61		
229201_at	13.58		
1560570_a_at	13.29		
1561732_at	12.93		
231442_at	12.58	zona pellucida binding protein 2	ZPBP2
1558972_s_at	12.53	chromosome 6 open reading frame 190	C6orf190
207881_at	12.41		
1565436_s_at	12.13	myeloid/lymphoid or mixed-lineage leukemia (trithorax homolog, Drosophila)	MLL
220565_at	12.10	chemokine (C-C motif) receptor 10	CCR10
237795_s_at	11.95		
234669_x_at	11.55		

231385_at	11.38	developmental pluripotency associated 3	DPPA3
221659_s_at	11.32		
1553834_at	11.00		
211602_s_at	10.93	transient receptor potential cation channel, subfamily C, member 1	TRPC1
220435_at	10.84	solute carrier family 30, member 10	SLC30A10
242344_at	10.71	gamma-aminobutyric acid (GABA) A receptor, beta 2	GABRB2
207678_s_at	10.61	SRY (sex determining region Y)-box 30	SOX30
206786_at	10.54	histatin 3	HTN3
241218_at	10.53		
234930_at	10.43	chromosome 1 open reading frame 91	C1orf91
241147_at	10.37		
1570051_at	10.35	ring finger protein 144	RNF144
1564642_at	10.18	runt-related transcription factor 1; translocated to, 1 (cyclin D-related)	RUNX1T1
244557_at	10.05		
229288_at	9.96		
1561564_at	9.89		
1559930_at	9.85		
1563825_at	9.74		
1561113_at	9.73		
219302_s_at	9.70	contactin associated protein-like 2	CNTNAP2
1555580_at	9.67		
217177_s_at	9.52		
208145_at	9.50		
211436_at	9.47		
1565875_at	9.45	nucleoporin 153kDa	NUP153
212079_s_at	9.43	myeloid/lymphoid or mixed-lineage leukemia (trithorax homolog, Drosophila)	MLL
1561242_at	9.40		
205088_at	9.35	chromosome X open reading frame 6	CXorf6
1553830_s_at	9.12	melanoma antigen family A, 2	MAGEA2
1559550_s_at	9.11		
1557395_at	9.05	insulin-like growth factor binding protein 7	IGFBP7
212078_s_at	9.02	myeloid/lymphoid or mixed-lineage leukemia (trithorax homolog, Drosophila)	MLL
1563589_at	8.96		
234387_at	8.90	collagen, type IV, alpha 5 (Alport syndrome)#collagen, type IV, alpha 6	COL4A5#COL4A6
235146_at	8.88		
242856_at	8.87		
1563087_at	8.85		
241567_at	8.76	nucleolar protein 4	NOL4
225990_at	8.75	Boc homolog (mouse)	BOC
211560_s_at	8.75	aminolevulinate, delta-, synthase 2 (sideroblastic/hypochromic anemia)	ALAS2
217709_at	8.68	N-myristoyltransferase 2	NMT2
234823_at	8.55		
1555942_a_at	8.54		
234075_at	8.53		
231386_at	8.52		
213316_at	8.44	KIAA1462	KIAA1462
1570376_at	8.31		

228384_s_at	8.31	chromosome 10 open reading frame 33	C10orf33
239738_at	8.27	dachshund homolog 2 (Drosophila)	DACH2
237411_at	8.27	ADAM metallopeptidase with thrombospondin type 1 motif, 6	ADAMTS6
235523_at	8.25		
203889_at	8.21	secretogranin V (7B2 protein)	SCG5
1556232_at	8.13	kinesin family member 6	KIF6
225491_at	8.11	solute carrier family 1 (glial high affinity glutamate transporter), member 2	SLC1A2
233048_at	8.09	family with sequence similarity 35, member A	FAM35A
205816_at	8.07	integrin, beta 8	ITGB8
1562742_at	8.03		
243381_at	7.98		
211510_s_at	7.97	corticotropin releasing hormone receptor 2	CRHR2
215893_x_at	7.93		
242046_at	7.86	transmembrane protein 163	TMEM163
206953_s_at	7.83	latrophilin 2	LPHN2
228731_at	7.82		
233386_at	7.80		
214725_at	7.78		
207866_at	7.75	bone morphogenetic protein 8b (osteogenic protein 2)	BMP8B
1560528_at	7.74		
1561141_at	7.70		
210026_s_at	7.67	caspase recruitment domain family, member 10	CARD10
1560813_at	7.66		
214569_at	7.65	interferon, alpha 5	IFNA5
1561210_at	7.65		
210991_s_at	7.59	regulating synaptic membrane exocytosis 3	RIMS3
231430_at	7.36		
217194_at	7.30	RAS protein activator like 2	RASAL2
244036_at	7.29		
1558672_at	7.28		
215738_at	7.28		
210661_at	7.27	glycine receptor, alpha 3	GLRA3
215894_at	7.27	prostaglandin D2 receptor (DP)	PTGDR
1556713_at	7.26		
228560_at	7.23		
1566115_at	7.22		
220476_s_at	7.19	chromosome 1 open reading frame 183	C1orf183
224590_at	7.18	X (inactive)-specific transcript	XIST
230472_at	7.14	iroquois homeobox protein 1	IRX1
1564323_at	7.10		
241859_at	7.08		
1564307_a_at	7.08	alpha-2-macroglobulin-like 1	A2ML1
240293_at	7.02		
224022_x_at	6.99	wingless-type MMTV integration site family, member 16	WNT16
1563332_at	6.97		
1561985_at	6.96	chromosome 14 open reading frame 39	C14orf39
209230_s_at	6.94		
225168_at	6.92	FERM domain containing 4A	FRMD4A
231734_at	6.90	retinol binding protein 2, cellular	RBP2

234454_at	6.90	vasohibin 1#angel homolog 1 (Drosophila)#chromosome 14 open reading frame 166B#ribosomal protein L22 pseudogene 2	VASH1#ANGEL1#C14orf166B#RPL22P2
222033_s_at	6.83	fms-related tyrosine kinase 1 (vascular endothelial growth factor/vascular permeability factor recep	FLT1
224278_at	6.83	chromosome 2 open reading frame 14	C2orf14
217145_at	6.79	immunoglobulin kappa constant	IGKC
234892_at	6.78		
213578_at	6.70	bone morphogenetic protein receptor, type IA	BMPR1A
240726_at	6.64		
231467_at	6.62		
215240_at	6.62	integrin, beta 3 (platelet glycoprotein IIIa, antigen CD61)	ITGB3
1567287_at	6.57	olfactory receptor, family 5, subfamily K, member 1	OR5K1
1557609_s_at	6.56	TBC1 domain family, member 12	TBC1D12
205764_at	6.55		
215769_at	6.51		
215810_x_at	6.51		
238081_at	6.47		
1554964_x_at	6.44	chromosome 6 open reading frame 192	C6orf192
202712_s_at	6.42	creatine kinase, mitochondrial 1B	CKMT1B
205567_at	6.38	carbohydrate (keratan sulfate Gal-6) sulfotransferase 1	CHST1
233356_at	6.36		
233858_at	6.35		
235238_at	6.32	SHC (Src homology 2 domain containing) family, member 4	SHC4
237398_at	6.32		
216067_at	6.32		
1562168_at	6.27		
1561094_a_at	6.27		
231598_x_at	6.27		
243932_at	6.27		
211524_at	6.23	nuclear factor of kappa light polypeptide gene enhancer in B-cells 2 (p49/p100)	NFKB2
1562946_at	6.20		
236719_at	6.18		
227209_at	6.18	contactin 1	CNTN1
233984_at	6.15		
215034_s_at	6.14	transmembrane 4 L six family member 1	TM4SF1
1555382_at	6.13	premature ovarian failure, 1B	POF1B
207447_s_at	6.10	mannosyl (alpha-1,3-)-glycoprotein beta-1,4-N-acetylglucosaminyltransferase, isozyme C (putative)	MGAT4C
1555573_at	6.08	chromosome 10 open reading frame 93	C10orf93
240120_at	6.08	sorbin and SH3 domain containing 2	SORBS2
209793_at	6.06	glutamate receptor, ionotropic, AMPA 1	GRIA1
234448_at	6.04		
243015_at	6.04	cytochrome P450, family 3, subfamily A, polypeptide 5	CYP3A5
1557787_at	6.03		
217479_at	6.01		
1552522_at	5.98	tigger transposable element derived 4	TIGD4
1563128_at	5.95		
1565337_at	5.91	dynein, axonemal, heavy chain 6	DNAH6

210239_at	5.90	iroquois homeobox protein 5	IRX5
216155_at	5.87		
229151_at	5.85	solute carrier family 14 (urea transporter), member 1 (Kidd blood group)	SLC14A1
1560075_at	5.85	zinc finger protein 622	ZNF622
237634_at	5.85		
1561125_at	5.84	methylenetetrahydrofolate dehydrogenase (NADP+ dependent) 1-like	MTHFD1L
215598_at	5.76	tetratricopeptide repeat domain 12	TTC12
1561266_at	5.76		
234428_at	5.76		
1570009_at	5.72		
242249_at	5.70		
1558890_at	5.70		
1555519_at	5.64		
240512_x_at	5.64	potassium channel tetramerisation domain containing 4	KCTD4
207705_s_at	5.63		
229160_at	5.61	melanoma associated antigen (mutated) 1-like 1	MUM1L1
1569712_at	5.59		
203426_s_at	5.52	insulin-like growth factor binding protein 5	IGFBP5
243684_at	5.52	FAT tumor suppressor homolog 3 (Drosophila)	FAT3
234218_at	5.50		
226989_at	5.49	RGM domain family, member B	RGMB
1569882_at	5.49		
236357_at	5.45		
1555025_at	5.44	transmembrane protein 26	TMEM26
229441_at	5.43	protease, serine, 23	PRSS23
1566786_at	5.42		
240135_x_at	5.42	TIMP metallopeptidase inhibitor 3 (Sorsby fundus dystrophy, pseudoinflammatory)	TIMP3
235570_at	5.41		
223633_s_at	5.40	brevican	BCAN
216595_at	5.40		
208479_at	5.38	potassium voltage-gated channel, shaker-related subfamily, member 1 (episodic ataxia with myokymia)	KCNA1
210055_at	5.38	thyroid stimulating hormone receptor	TSHR
203066_at	5.38		
242522_at	5.36		
203324_s_at	5.35	caveolin 2	CAV2
229638_at	5.34	iroquois homeobox protein 3	IRX3
221451_s_at	5.30	olfactory receptor, family 2, subfamily W, member 1	OR2W1
206617_s_at	5.30	renin binding protein	RENBP
234369_at	5.28	zinc finger protein 596#olfactory receptor, family 4, subfamily F, member 21#null	ZNF596#OR4F21#null
1554994_at	5.28	recombination activating gene 1	RAG1
231128_at	5.28		
242257_at	5.27		
222513_s_at	5.26	sorbin and SH3 domain containing 1	SORBS1
1569659_at	5.24		
225981_at	5.22	chromosome 17 open reading frame 28	C17orf28
231424_at	5.21		
206189_at	5.20	unc-5 homolog C (C. elegans)	UNC5C

214425_at	5.18	alpha-1-microglobulin/bikunin precursor	AMBP
223595_at	5.18	transmembrane protein 133	TMEM133
219737_s_at	5.18	protocadherin 9	PCDH9
244300_at	5.17	latrophilin 3	LPHN3
1553328_a_at	5.17	solute carrier family 18 (vesicular monoamine), member 2	SLC18A2
1556944_at	5.17		
241970_at	5.17	poliovirus receptor-related 3	PVRL3
243831_at	5.16		
1554565_x_at	5.15		
233696_at	5.15		
232714_at	5.15		
204385_at	5.13	kynureninase (L-kynurenine hydrolase)	KYNU
240522_at	5.12	BAI1-associated protein 2-like 1	BAIAP2L1
1552939_at	5.12	angiopoietin 1	ANGPT1
1561846_s_at	5.12		
240582_x_at	5.08		
238914_at	5.07		
230730_at	5.07	sarcoglycan, delta (35kDa dystrophin-associated glycoprotein)	SGCD
207926_at	5.06	glycoprotein V (platelet)	GP5
206655_s_at	5.05	glycoprotein Ib (platelet), beta polypeptide	GP1BB
232638_at	5.03	collagen, type XX, alpha 1	COL20A1
221065_s_at	5.02	carbohydrate (N-acetylgalactosamine 4-0) sulfotransferase 8	CHST8
217081_at	5.00	gamma-aminobutyric acid (GABA) B receptor, 1#olfactory receptor, family 2, subfamily H, member 2#olfactory receptor, family 2, subfamily H, member 2#ubiquitin D#SMT3 suppressor of mif two 3 homolog 2 (S. cerevisiae) pseudogene	GABBR1#OR2H2#OR2H2#UBD#SUMO2P
228632_at	4.99		
217071_s_at	4.97	5,10-methylenetetrahydrofolate reductase (NADPH)	MTHFR
1562473_at	4.95		
243481_at	4.94	ras homolog gene family, member J	RHOJ
205932_s_at	4.94	msh homeobox 1	MSX1
1560373_a_at	4.93		
220418_at	4.92	ubiquitin associated and SH3 domain containing, A	UBASH3A
244853_at	4.91		
239544_at	4.91		
228875_at	4.91	chromosome 6 open reading frame 189	C6orf189
233008_at	4.90		
228657_at	4.89	kinesin family member 1B	KIF1B
1557745_at	4.89		
230074_s_at	4.88		
230665_at	4.88		
1559434_at	4.88		
237157_at	4.88		
243281_at	4.88		
213369_at	4.87	protocadherin 21	PCDH21
1565026_a_at	4.87	orofacial cleft 1 candidate 1	OFCC1
236810_at	4.85		
1569673_at	4.85		
208460_at	4.85	gap junction protein, alpha 7, 45kDa	GJA7

239483_at	4.82		
1563467_at	4.81		
244762_at	4.80	Down syndrome critical region gene 3	DSCR3
208232_x_at	4.78	neuregulin 1	NRG1
216827_at	4.78		
1565346_a_at	4.78	ATPase, Na+/K+ transporting, alpha 4 polypeptide	ATP1A4
1558987_at	4.77		
214593_at	4.77	protein inhibitor of activated STAT, 2	PIAS2
239668_at	4.76		
237232_at	4.76		
208542_x_at	4.76	zinc finger protein 208	ZNF208
1562690_at	4.74		
220503_at	4.73	solute carrier family 13 (sodium/sulfate symporters), member 1	SLC13A1
229262_at	4.73		
238966_at	4.73		
234835_at	4.72		
235754_at	4.70	hemochromatosis	HFE
226470_at	4.69	gamma-glutamyltransferase-like 3#nuclear receptor coactivator 6#acyl-CoA synthetase short-chain family member 2#high-mobility group (nonhistone chromosomal) protein 4-like	GGTL3#NCOA6#ACSS2#HMG4L
237109_at	4.68	target of myb1-like 2 (chicken)	TOM1L2
204136_at	4.68	collagen, type VII, alpha 1 (epidermolysis bullosa, dystrophic, dominant and recessive)	COL7A1
207033_at	4.66	gastric intrinsic factor (vitamin B synthesis)	GIF
1566763_at	4.66		
237310_at	4.65	exostoses (multiple) 1	EXT1
213662_at	4.64		
227475_at	4.63	forkhead box Q1	FOXQ1
1559450_at	4.63		
1562863_at	4.63		
206344_at	4.63	paraoxonase 1	PON1
234752_x_at	4.62	dystrophin (muscular dystrophy, Duchenne and Becker types)	DMD
223574_x_at	4.60	protein phosphatase 2 (formerly 2A), regulatory subunit B, gamma isoform	PPP2R2C
244399_at	4.60		
52837_at	4.60		
1565748_at	4.57		
234340_at	4.56		
207052_at	4.56	hepatitis A virus cellular receptor 1	HAVCR1
1565602_at	4.54		
1561062_a_at	4.54		
204217_s_at	4.54	reticulon 2	RTN2
238060_s_at	4.54	beta-1,4-N-acetyl-galactosaminyl transferase 4	B4GALNT4
1561966_at	4.52		
1561714_a_at	4.52	beta-1,3-N-acetylgalactosaminyltransferase 2	B3GALNT2
1562815_at	4.51		
1570090_at	4.51		
216256_at	4.50	glutamate receptor, metabotropic 8	GRM8
208304_at	4.50	chemokine (C-C motif) receptor 3	CCR3

234686_at	4.50	annexin A2 pseudogene 2#T cell receptor beta variable 21/OR9-2#T cell receptor beta variable 24/OR9-2#T cell receptor beta variable 20/OR9-2#T cell receptor beta variable orphans on chromosome 9#T cell receptor beta variable 23/OR9-2#T cell receptor beta variable 22/OR9-2#null#suppressor of G2 allele of SKP1 pseudogene (S. cerevisiae)#ankyrin repeat domain 18B	ANXA2P2#TRBV21OR9-2#TRBV24OR9-2#TRBV20OR9-2#TRBVOR9@#TRBV23OR9-2#TRBV22OR9-2#null#SUGT1P#ANKRD18B
244453_at	4.49	ankyrin repeat domain 53	ANKRD53
238379_x_at	4.49		
207267_s_at	4.48	Down syndrome critical region gene 6	DSCR6
237058_x_at	4.48	solute carrier family 6 (neurotransmitter transporter, GABA), member 13	SLC6A13
1563590_at	4.46		
242868_at	4.45	endothelial PAS domain protein 1	EPAS1
1560538_at	4.43		
203874_s_at	4.41	SWI/SNF related, matrix associated, actin dependent regulator of chromatin, subfamily a, member 1	SMARCA1
1557386_at	4.41		
1555617_x_at	4.41		
238919_at	4.40		
243066_at	4.40	N-acetylneuraminate pyruvate lyase (dihydrodipicolinate synthase)	NPL
1561192_at	4.40		
1554314_at	4.39	chromosome 6 open reading frame 141	C6orf141
228602_at	4.39	sarcoglycan, delta (35kDa dystrophin-associated glycoprotein)	SGCD
233261_at	4.39	early B-cell factor 1	EBF1
212353_at	4.39	sulfatase 1	SULF1
222247_at	4.38		
230841_at	4.37	chromosome 6 open reading frame 60	C6orf60
216898_s_at	4.36	collagen, type IV, alpha 3 (Goodpasture antigen)	COL4A3
1554474_a_at	4.36	monooxygenase, DBH-like 1	MOXD1
231597_x_at	4.36		
1563693_at	4.36		
1561431_at	4.33		
1561856_at	4.33		
203187_at	4.33	dedicator of cytokinesis 1	DOCK1
223866_at	4.32	armadillo repeat containing 2	ARMC2
204029_at	4.32	cadherin, EGF LAG seven-pass G-type receptor 2 (flamingo homolog, Drosophila)	CELSR2
222904_s_at	4.32	transmembrane channel-like 5	TMC5
1562514_at	4.32		
220562_at	4.29	cytochrome P450, family 2, subfamily W, polypeptide 1	CYP2W1
234213_at	4.28		
238073_at	4.28	ELAV (embryonic lethal, abnormal vision, Drosophila)-like 4 (Hu antigen D)	ELAVL4

205485_at	4.28	ryanodine receptor 1 (skeletal)	RYR1
1556630_at	4.28	cancer susceptibility candidate 2	CASC2
231911_at	4.28	KIAA1189	KIAA1189
236393_at	4.27		
206785_s_at	4.26	killer cell lectin-like receptor subfamily C, member 2	KLRC2
1559949_at	4.25	trichorhinophalangeal syndrome I	TRPS1
244806_at	4.24		
241387_at	4.23		
239913_at	4.23	solute carrier family 10 (sodium/bile acid cotransporter family), member 4	SLC10A4
1560503_a_at	4.22		
205439_at	4.21	glutathione S-transferase theta 2	GSTT2
202254_at	4.20	signal-induced proliferation-associated 1 like 1	SIPA1L1
1555471_a_at	4.20	formin 2	FMN2
203485_at	4.20	reticulon 1	RTN1
205561_at	4.20	potassium channel tetramerisation domain containing 17	KCTD17
1565588_at	4.19		
1565728_at	4.18		
232155_at	4.18	KIAA1618	KIAA1618
203425_s_at	4.17	insulin-like growth factor binding protein 5	IGFBP5
232748_at	4.17	pregnancy-associated plasma protein A, pappalysin 1	PAPPA
230402_at	4.16	BCL2-like 1#forkhead-like 18 (Drosophila)#TPX2, microtubule-associated, homolog (Xenopus laevis)#chromosome 20 open reading frame 57#myosin light chain kinase 2, skeletal muscle#dual specificity phosphatase 15#tubulin tyrosine ligase-like family, member 9	BCL2L1#FKHL18#TPX2#C20orf57#MYLK2#DUSP15#TTLL9
222108_at	4.16	adhesion molecule with Ig-like domain 2	AMIGO2
219727_at	4.16	dual oxidase 2	DUOX2
1562103_at	4.15	Janus kinase 1 (a protein tyrosine kinase)	JAK1
1569861_at	4.15	TNF receptor-associated factor 5	TRAF5
1558894_a_at	4.14	coiled-coil domain containing 67	CCDC67
242516_x_at	4.14		
240775_at	4.13	zinc finger protein 407	ZNF407
241184_x_at	4.13	zinc finger protein 407	ZNF407
205166_at	4.13	calpain 5	CAPN5
1553851_at	4.13	Spi-C transcription factor (Spi-1/PU.1 related)	SPIC
1560762_at	4.12		
217525_at	4.12	olfactomedin-like 1	OLFML1
1554710_at	4.11	potassium large conductance calcium-activated channel, subfamily M, beta member 1	KCNMB1
241305_at	4.10	kynureninase (L-kynurenine hydrolase)	KYNU
241829_at	4.10	family with sequence similarity 124A	FAM124A
1559105_at	4.09		
224113_at	4.09	caspase recruitment domain family, member 14	CARD14
215522_at	4.09	sortilin-related VPS10 domain containing receptor 3	SORCS3
242496_at	4.09		
243216_x_at	4.08	ubiquitin specific peptidase 40	USP40
241287_x_at	4.07	chromosome 4 open reading frame 15	C4orf15
220749_at	4.07	chromosome 10 open reading frame 68	C10orf68
202222_s_at	4.06	desmin	DES
1553197_at	4.06	WD repeat domain 21C	WDR21C

1556477_a_at	4.06		
1569941_at	4.06		
205935_at	4.05	forkhead box F1	FOXF1
206112_at	4.05	ankyrin repeat domain 7	ANKRD7
1561191_at	4.05		
232988_at	4.05	KIAA0182	KIAA0182
207445_s_at	4.04	chemokine (C-C motif) receptor 9	CCR9
206094_x_at	4.03	UDP glucuronosyltransferase 1 family, polypeptide A6	UGT1A6
243237_at	4.02		
1569054_at	4.02	solute carrier family 1 (glial high affinity glutamate transporter), member 3	SLC1A3
1569819_at	4.01		
210230_at	4.00		
203038_at	4.00	protein tyrosine phosphatase, receptor type, K	PTPRK
211736_at	4.00	Sp2 transcription factor	SP2
243814_at	3.99	zinc finger, MYND-type containing 8	ZMYND8
1552785_at	3.99	zinc finger protein 781	ZNF781
227842_at	3.99	RAB30, member RAS oncogene family	RAB30
240188_at	3.98	transforming growth factor, beta receptor III (betaglycan, 300kDa)	TGFBR3
204981_at	3.98	solute carrier family 22 (organic cation transporter), member 18	SLC22A18
1563671_at	3.98	lactation elevated 1	LACE1
207222_at	3.98	phospholipase A2, group X	PLA2G10
1568896_at	3.97	syntaxin binding protein 5-like	STXBP5L
221546_at	3.97	PRP18 pre-mRNA processing factor 18 homolog (S. cerevisiae)	PRPF18
230030_at	3.97	heparan sulfate 6-O-sulfotransferase 2	HS6ST2
241672_at	3.95		
244838_at	3.94		
1569466_at	3.94	chromosome 20 open reading frame 152	C20orf152
230167_at	3.92	ADAM metallopeptidase with thrombospondin type 1 motif, 14	ADAMTS14
234896_at	3.92		
204779_s_at	3.91	homeobox B7	HOXB7
213183_s_at	3.90	cyclin-dependent kinase inhibitor 1C (p57, Kip2)	CDKN1C
232853_at	3.88		
1569740_at	3.88		
237351_at	3.88		
219783_at	3.88	chromosome 2 open reading frame 18	C2orf18
209818_s_at	3.87	hyaluronan binding protein 4	HABP4
1564097_at	3.86		
1557466_at	3.86		
224965_at	3.86	guanine nucleotide binding protein (G protein), gamma 2	GNG2
237732_at	3.85		
1569263_at	3.85		
1552609_s_at	3.83	interleukin 28B (interferon, lambda 3)	IL28B
229049_at	3.82		
216001_at	3.82	PRAME family member 1#PRAME family member 12	PRAMEF1#PRAMEF12
229057_at	3.82	sodium channel, voltage-gated, type II, alpha subunit	SCN2A
236990_at	3.82		

1569525_s_at	3.82		
1554772_at	3.82	chromosome 17 open reading frame 57	C17orf57
1561264_at	3.81		
1563637_at	3.81		
218087_s_at	3.81	sorbin and SH3 domain containing 1	SORBS1
204797_s_at	3.81	echinoderm microtubule associated protein like 1	EML1
216077_s_at	3.80	serum/glucocorticoid regulated kinase 2	SGK2
205691_at	3.80	synaptogyrin 3	SYNGR3
218665_at	3.79	frizzled homolog 4 (Drosophila)	FZD4
213343_s_at	3.77	glycerophosphodiester phosphodiesterase domain containing 5	GDPD5
208849_at	3.76		
207892_at	3.76	CD40 ligand (TNF superfamily, member 5, hyper-IgM syndrome)	CD40LG
231098_at	3.75		
207596_at	3.74		
227289_at	3.74	protocadherin 17	PCDH17
243715_at	3.73		
235004_at	3.73		
1566598_at	3.72		
225270_at	3.72	neogenin homolog 1 (chicken)	NEO1
1556185_a_at	3.72		
217321_x_at	3.71	ataxin 3	ATXN3
1555235_s_at	3.71	IQ motif containing F3	IQCF3
219795_at	3.71	solute carrier family 6 (amino acid transporter), member 14	SLC6A14
236634_at	3.71	chromosome 8 open reading frame 48	C8orf48
231243_s_at	3.71	basic helix-loop-helix domain containing, class B, 3	BHLHB3
227439_at	3.70	ankyrin repeat and sterile alpha motif domain containing 1B	ANKS1B
233488_at	3.70	ribonuclease, RNase A family, 7	RNASE7
235955_at	3.70	MARVEL domain containing 2	MARVELD2
241030_at	3.70	fibrous sheath interacting protein 1	FSIP1
1558148_x_at	3.69		
207780_at	3.69	cylicin, basic protein of sperm head cytoskeleton 2	CYLC2
234039_at	3.68	tetratricopeptide repeat, ankyrin repeat and coiled-coil containing 1	TANC1
239492_at	3.67	SEC14-like 4 (S. cerevisiae)	SEC14L4
1553672_at	3.67	enabled homolog (Drosophila)	ENAH
1553613_s_at	3.66	forkhead box C1	FOXC1
214295_at	3.66		
216460_at	3.65		
214858_at	3.65		
206769_at	3.65	thymosin, beta 4, Y-linked	TMSB4Y
1561262_at	3.64		
239092_at	3.64	integrin, alpha 8	ITGA8
238402_s_at	3.64		
214218_s_at	3.63		
1564028_s_at	3.63		
242815_x_at	3.63		
215860_at	3.63	synaptotagmin XII	SYT12
231046_at	3.63		
208406_s_at	3.62	GRB2-related adaptor protein 2	GRAP2

40148_at	3.62	amyloid beta (A4) precursor protein-binding, family B, member 2 (Fe65-like)	APBB2
1562698_x_at	3.62		
233321_x_at	3.62		
1565877_at	3.61		
231181_at	3.61		
234765_at	3.61		
240366_at	3.60		
237522_at	3.60	Fas (TNF receptor superfamily, member 6)	FAS
207923_x_at	3.60	paired box gene 8	PAX8
1553828_at	3.60	family with sequence similarity 55, member A	FAM55A
220582_at	3.60		
215928_at	3.58		
1558247_s_at	3.58		
227472_at	3.57	chromosome 19 open reading frame 58	C19orf58
237252_at	3.57	thrombomodulin	THBD
231078_at	3.57		
1567576_at	3.57		
223801_s_at	3.55	apolipoprotein L, 4	APOL4
240497_at	3.54		
214994_at	3.53	apolipoprotein B mRNA editing enzyme, catalytic polypeptide-like 3F	APOBEC3F
243859_at	3.53		
239381_at	3.52	kallikrein-related peptidase 7	KLK7
238204_at	3.52	leiomodin 1 (smooth muscle)	LMOD1
227997_at	3.52	interleukin 17 receptor D	IL17RD
242999_at	3.52	Rho guanine nucleotide exchange factor (GEF) 7	ARHGEF7
207498_s_at	3.51	cytochrome P450, family 2, subfamily D, polypeptide 6	CYP2D6
237953_at	3.51	dipeptidyl-peptidase 4 (CD26, adenosine deaminase complexing protein 2)	DPP4
234618_at	3.51		
242182_x_at	3.51		
231813_s_at	3.50	family with sequence similarity 104, member A	FAM104A
1556876_s_at	3.50		
237460_x_at	3.50		
240401_at	3.49	apoptosis antagonizing transcription factor	AATF
207951_at	3.49	casein beta	CSN2
212344_at	3.48	sulfatase 1	SULF1
234472_at	3.48	UDP-N-acetyl-alpha-D-galactosamine:polypeptide N-acetylgalactosaminyltransferase 13 (GalNAc-T13)	GALNT13
1552477_a_at	3.48	interferon regulatory factor 6	IRF6
204179_at	3.48	myoglobin	MB
1558847_at	3.48		
1561207_at	3.48		
216522_at	3.47	olfactory receptor, family 2, subfamily B, member 6#olfactory receptor, family 2, subfamily W, member 6 pseudogene#olfactory receptor, family 2, subfamily W, member 4 pseudogene	OR2B6#OR2W6P#OR2W4P
209498_at	3.47	carcinoembryonic antigen-related cell adhesion molecule 1 (biliary glycoprotein)	CEACAM1
239371_at	3.47	forkhead box K2	FOXK2
216828_at	3.46	chromosome 20 open reading frame 80#null	C20orf80#null

235752_at	3.45		
224438_at	3.45		
239345_at	3.45	solute carrier family 19, member 3	SLC19A3
1554933_at	3.45	PC4 and SFRS1 interacting protein 1	PSIP1
231622_at	3.44	ankyrin repeat and SOCS box-containing 17	ASB17
241393_at	3.44		
1563854_s_at	3.43		
237896_at	3.43	nodal homolog (mouse)	NODAL
1560943_s_at	3.43	phosphogluconate dehydrogenase	PGD
235874_at	3.43	protease, serine, 35	PRSS35
227629_at	3.42	prolactin receptor	PRLR
1565862_a_at	3.42	tight junction protein 2 (zona occludens 2)	TJP2
1555014_x_at	3.42		
243291_at	3.41	forkhead box P1	FOXP1
1556983_a_at	3.41		
1557008_at	3.41		
221009_s_at	3.41	angiopoietin-like 4	ANGPTL4
227906_s_at	3.41		
242737_at	3.40		
1557403_s_at	3.38		
1563595_at	3.38	SLIT-ROBO Rho GTPase activating protein 3	SRGAP3
1560733_at	3.38		
206253_at	3.38	discs, large homolog 2, chapsyn-110 (Drosophila)	DLG2
241760_x_at	3.37		
239292_at	3.37		
227662_at	3.36	synaptopodin 2	SYNPO2
207284_s_at	3.36	aspartate beta-hydroxylase	ASPH
242252_at	3.36		
242843_at	3.36	brevican	BCAN
1566093_at	3.36	Rho guanine nucleotide exchange factor (GEF) 12	ARHGEF12
1557126_a_at	3.36	phospholipase D1, phosphatidylcholine-specific	PLD1
237216_at	3.36	GTPase activating Rap/RanGAP domain-like 1	GARNL1
1558179_at	3.35	ATP synthase, H+ transporting, mitochondrial F0 complex, subunit F2	ATP5J2
234768_at	3.35		
232868_at	3.34	chromosome 9 open reading frame 11	C9orf11
219738_s_at	3.33	protocadherin 9	PCDH9
1559663_at	3.33		
236792_at	3.33		
242824_at	3.32		
236556_s_at	3.32	LON peptidase N-terminal domain and ring finger 1	LONRF1
241209_at	3.32	IQ motif and WD repeats 1	IQWD1
204517_at	3.32	peptidylprolyl isomerase C (cyclophilin C)	PPIC
233412_x_at	3.32		
222378_at	3.32		
206306_at	3.31	ryanodine receptor 3	RYR3
216192_at	3.30	fatty acid binding protein 7, brain	FABP7
202011_at	3.30	tight junction protein 1 (zona occludens 1)	TJP1
1561969_at	3.30	zona pellucida-like domain containing 1	ZPLD1
223875_s_at	3.30	enhancer of polycomb homolog 1 (Drosophila)	EPC1
240979_at	3.30		
215206_at	3.29		
1562607_at	3.29		

1565819_at	3.29	phosphatase and actin regulator 4	PHACTR4
233202_at	3.29	contactin associated protein-like 3	CNTNAP3
238296_at	3.28	GLI pathogenesis-related 1 like 1	GLIPR1L1
244710_at	3.28	leucine-rich repeats and guanylate kinase domain containing	LRGUK
228335_at	3.28	claudin 11 (oligodendrocyte transmembrane protein)	CLDN11
243517_at	3.28		
241155_at	3.28	phosphatidylinositol-4-phosphate 5-kinase, type II, alpha	PIP5K2A
228636_at	3.27	basic helix-loop-helix domain containing, class B, 5	BHLHB5
234958_at	3.27		
1563805_a_at	3.27	family with sequence similarity 83, member C	FAM83C
1556700_a_at	3.26		
208280_at	3.26	CMT1A duplicated region transcript 1	CDRT1
1560418_at	3.26	chromosome 6 open reading frame 182	C6orf182
236892_s_at	3.26		
239945_at	3.26		
235442_at	3.26	chromosome X open reading frame 56	CXorf56
1558534_at	3.25		
206515_at	3.24	cytochrome P450, family 4, subfamily F, polypeptide 3	CYP4F3
214598_at	3.24	claudin 8	CLDN8
1554607_at	3.24	connector enhancer of kinase suppressor of Ras 2	CNKSR2
206691_s_at	3.24	protein disulfide isomerase family A, member 2	PDIA2
1570138_at	3.23		
1562389_at	3.21		
203666_at	3.21	chemokine (C-X-C motif) ligand 12 (stromal cell-derived factor 1)	CXCL12
202421_at	3.21	immunoglobulin superfamily, member 3	IGSF3
233108_at	3.20		
213553_x_at	3.19	apolipoprotein C-I	APOC1
240998_at	3.19		
232705_at	3.18	leucine rich repeat (in FLII) interacting protein 2	LRRFIP2
212327_at	3.18		
223137_at	3.18	zinc finger, DHHC-type containing 4	ZDHHC4
217505_at	3.18	kelch-like 23 (Drosophila)	KLHL23
1561759_at	3.18		
1566798_at	3.18		
1555970_at	3.18	F-box protein 28	FBXO28
240593_x_at	3.17	calcium/calmodulin-dependent protein kinase (CaM kinase) II delta	CAMK2D
232837_at	3.17	kinesin family member 13A	KIF13A
244608_at	3.17		
206032_at	3.17	desmocollin 3	DSC3
242719_at	3.17		
1562772_a_at	3.17	DAN domain family, member 5	DAND5
204856_at	3.16	UDP-GlcNAc:betaGal beta-1,3-N-acetylglucosaminyltransferase 3	B3GNT3
1566656_a_at	3.16		
216062_at	3.16		
222153_at	3.16	myelin expression factor 2	MYEF2
234009_at	3.16		
1560318_at	3.16	Rho GTPase activating protein 29	ARHGAP29
1562933_at	3.16		

1563452_at	3.16		
233170_at	3.15		
201693_s_at	3.15	early growth response 1	EGR1
237660_at	3.14		
240599_x_at	3.14		
1559529_at	3.14	PTK2 protein tyrosine kinase 2	PTK2
1563771_a_at	3.14		
210666_at	3.14	iduronate 2-sulfatase (Hunter syndrome)	IDS
217494_s_at	3.14	phosphatase and tensin homolog (mutated in multiple advanced cancers 1), pseudogene 1	PTENP1
233234_at	3.14	potassium channel tetramerisation domain containing 16	KCTD16
221605_s_at	3.13	pipecolic acid oxidase	PIPOX
242259_at	3.13	transmembrane protein 162	TMEM162
237739_at	3.13		
231180_at	3.13		
1566424_at	3.13		
241154_x_at	3.12		
240564_x_at	3.12	synuclein, alpha (non A4 component of amyloid precursor)	SNCA
214294_at	3.12		
1560863_a_at	3.12		
240211_at	3.12		
234010_at	3.11		
236630_at	3.11	aquaporin 2 (collecting duct)	AQP2
220148_at	3.11	aldehyde dehydrogenase 8 family, member A1	ALDH8A1
209612_s_at	3.10	alcohol dehydrogenase IB (class I), beta polypeptide	ADH1B
1560467_at	3.09	progestin and adipoQ receptor family member IX	PAQR9
237491_at	3.09	myosin, heavy chain 10, non-muscle	MYH10
221729_at	3.09		
222082_at	3.09	zinc finger and BTB domain containing 7A	ZBTB7A
1561232_at	3.09		
1569294_at	3.09	ring finger protein 187	RNF187
1553711_a_at	3.08		
240095_at	3.07		
243401_at	3.07	formin-like 2	FMNL2
1561424_at	3.07		
235590_at	3.07	chromosome 10 open reading frame 6	C10orf6
242142_at	3.07		
242889_x_at	3.07		
240706_at	3.06		
1559780_at	3.06		
1556794_at	3.06		
221076_at	3.05		
1560340_s_at	3.05		
233072_at	3.05	netrin G2	NTNG2
235847_at	3.04		
236272_at	3.04		
236065_at	3.04		
228128_x_at	3.03	pregnancy-associated plasma protein A, pappalysin 1	PAPPA
230372_at	3.03		
1566459_at	3.03		
243262_at	3.03	SET and MYND domain containing 3	SMYD3

241770_x_at	3.02	solute carrier family 22 (organic anion/cation transporter), member 9	SLC22A9
244175_at	3.02		
236746_at	3.02	UDP-N-acetyl-alpha-D-galactosamine:polypeptide N-acetylgalactosaminyltransferase 1 (GalNAc-T1)	GALNT1
1564157_at	3.02		
236066_at	3.02		
231585_at	3.02	vacuolar protein sorting 13 homolog A (S. cerevisiae)	VPS13A
239856_at	3.02		
1566043_at	3.01		
1561180_at	3.01		
1555394_at	3.01	phosphatidylinositol glycan anchor biosynthesis, class K	PIGK
217573_at	3.01	glutamate receptor, ionotropic, N-methyl D-aspartate 2C	GRIN2C
241281_at	3.00	armadillo repeat containing, X-linked 4	ARMCX4
236886_at	3.00		
234055_s_at	3.00	NTF2-like export factor 1#N-ethylmaleimide-sensitive factor attachment protein, beta#GDNF-inducible zinc finger protein 1#cystatin-like 1#cystatin 11	NXT1#NAPB#GZF1#CSTL1#CST11
1566242_at	3.00		
1562529_s_at	3.00		
1565693_at	3.00	deoxythymidylate kinase (thymidylate kinase)	DTYMK
229621_x_at	2.99	early B-cell factor 3	EBF3
206937_at	2.99	spectrin, alpha, erythrocytic 1 (elliptocytosis 2)	SPTA1
217654_at	2.99	CASP8 and FADD-like apoptosis regulator	CFLAR
242357_x_at	2.99		
1561065_at	2.99	ankyrin repeat domain 6	ANKRD6
231644_at	2.99		
236034_at	2.99		
237515_at	2.98	transmembrane protein 56	TMEM56
216955_at	2.98	TAF1 RNA polymerase II, TATA box binding protein (TBP)-associated factor, 250kDa	TAF1
1562235_s_at	2.98		
230856_at	2.98		
234585_at	2.98		
1566902_at	2.98		
204932_at	2.98	tumor necrosis factor receptor superfamily, member 11b (osteoprotegerin)	TNFRSF11B
235592_at	2.98		
1565801_at	2.97		
1559469_s_at	2.97	signal-induced proliferation-associated 1 like 2	SIPA1L2
215246_at	2.97	La ribonucleoprotein domain family, member 7	LARP7
1570289_at	2.97		
1560738_at	2.97		
239837_at	2.96	ADAM metallopeptidase domain 11	ADAM11
1559376_at	2.96	chromosome 1 open reading frame 203	C1orf203
1565639_a_at	2.96		
219318_x_at	2.95	mediator of RNA polymerase II transcription, subunit 31 homolog (S. cerevisiae)	MED31
244104_at	2.95	mannosyl (beta-1,4-)-glycoprotein beta-1,4-N-acetylglucosaminyltransferase	MGAT3
1559037_a_at	2.95		

206119_at	2.95	betaine-homocysteine methyltransferase	BHMT
220830_at	2.95	interphotoreceptor matrix proteoglycan 2	IMPG2
210108_at	2.95	calcium channel, voltage-dependent, L type, alpha 1D subunit	CACNA1D
239669_at	2.94	histone cluster 1, H3d	HIST1H3D
1556425_a_at	2.94		
210660_at	2.94	leukocyte immunoglobulin-like receptor, subfamily A (with TM domain), member 1	LILRA1
237614_at	2.94		
234709_at	2.94	calpain 13	CAPN13
241855_s_at	2.94	cullin 3	CUL3
235619_at	2.93		
1552825_at	2.93	zinc finger protein 396	ZNF396
1568615_a_at	2.93		
219693_at	2.93	1-acylglycerol-3-phosphate O-acyltransferase 4 (lysophosphatidic acid acyltransferase, delta)	AGPAT4
241787_at	2.92		
215102_at	2.92	dpy-19-like 1 pseudogene 1 (C. elegans)	DPY19L1P1
234389_x_at	2.92		
204442_x_at	2.92	latent transforming growth factor beta binding protein 4	LTBP4
226716_at	2.92	proline rich 12	PRR12
206164_at	2.91	chloride channel, calcium activated, family member 2	CLCA2
230989_s_at	2.91	testis-specific serine kinase 6	TSSK6
238390_at	2.90	G protein-coupled receptor 39	GPR39
231991_at	2.90	chromosome 20 open reading frame 160	C20orf160
1569067_at	2.90		
1570244_at	2.89		
215330_at	2.89		
230711_at	2.89		
237973_at	2.89		
215956_at	2.89		
242841_at	2.89		
220878_at	2.88		
241079_at	2.88	pumilio homolog 1 (Drosophila)	PUM1
207733_x_at	2.88	pregnancy specific beta-1-glycoprotein 9	PSG9
204721_s_at	2.88	DnaJ (Hsp40) homolog, subfamily C, member 6	DNAJC6
1567540_at	2.88	sperm associated antigen 10	SPAG10
244567_at	2.88		
1556770_a_at	2.88	F-box and leucine-rich repeat protein 13	FBXL13
215213_at	2.87	nucleoporin 54kDa	NUP54
242733_at	2.86		
1561218_s_at	2.86		
1553533_at	2.86	junctophilin 1	JPH1
236966_at	2.86	thioredoxin domain containing 6	TXNDC6
215137_at	2.86		
217465_at	2.86	NCK-associated protein 1	NCKAP1
208194_s_at	2.86	signal transducing adaptor molecule (SH3 domain and ITAM motif) 2	STAM2
1554766_s_at	2.86	Pvt1 oncogene homolog, MYC activator (mouse)	PVT1
234228_at	2.85		
244690_at	2.85		
231560_at	2.85	leucine rich repeat containing 34	LRRC34
215296_at	2.85	CDC42 binding protein kinase alpha (DMPK-like)	CDC42BPA

1552899_at	2.85		
1569660_at	2.85		
208353_x_at	2.84	ankyrin 1, erythrocytic	ANK1
232174_at	2.84		
217033_x_at	2.84	neurotrophic tyrosine kinase, receptor, type 3	NTRK3
240217_s_at	2.84		
240520_at	2.84	proline rich 8	PRR8
239600_at	2.84		
234056_at	2.84		
242622_x_at	2.83	phosphatase and tensin homolog (mutated in multiple advanced cancers 1)	PTEN
216981_x_at	2.83	sialophorin (leukosialin, CD43)	SPN
242172_at	2.83	Meis1, myeloid ecotropic viral integration site 1 homolog (mouse)	MEIS1
1559697_a_at	2.82		
237053_at	2.82	transmembrane protein 90A	TMEM90A
224237_at	2.82		
221902_at	2.82	G protein-coupled receptor 153	GPR153
204400_at	2.82	embryonal Fyn-associated substrate	EFS
1570290_at	2.82		
233373_at	2.82		
227654_at	2.82	chromosome 20 open reading frame 175	C20orf175
216034_at	2.82	suppressor of hairy wing homolog 1 (Drosophila)	SUHW1
219102_at	2.82	reticulocalbin 3, EF-hand calcium binding domain	RCN3
1553392_at	2.81	EF-hand calcium binding domain 3	EFCAB3
242572_at	2.81		
234231_at	2.81		
232528_at	2.81		
216657_at	2.80	ataxin 3	ATXN3
211123_at	2.80	solute carrier family 5 (sodium iodide symporter), member 5	SLC5A5
229592_at	2.80		
219606_at	2.80	PHD finger protein 20-like 1	PHF20L1
244494_at	2.79	zinc finger, DHHC-type containing 1	ZDHHC1
205880_at	2.79	protein kinase D1	PRKD1
237057_at	2.79		
1552975_x_at	2.79		
231363_at	2.79	late cornified envelope-like proline-rich 1	LELP1
1570412_at	2.79		
206297_at	2.79	chymotrypsin C (caldecrin)	CTRC
240602_at	2.79	HBS1-like (S. cerevisiae)	HBS1L
1569856_at	2.78	tripeptidyl peptidase II	TPP2
228405_at	2.78	rhophilin, Rho GTPase binding protein 1	RHPN1
1564209_at	2.78		
235133_at	2.78		
209604_s_at	2.78	GATA binding protein 3	GATA3
228833_s_at	2.78		
1553764_a_at	2.78	jub, ajuba homolog (Xenopus laevis)	JUB
1563750_at	2.78		
241769_at	2.78		
226279_at	2.78	protease, serine, 23	PRSS23
204748_at	2.78	prostaglandin-endoperoxide synthase 2 (prostaglandin G/H synthase and cyclooxygenase)	PTGS2

236442_at	2.77	D4, zinc and double PHD fingers, family 3	DPF3
217414_x_at	2.77	hemoglobin, alpha 2	HBA2
209327_s_at	2.77		
207536_s_at	2.77	tumor necrosis factor receptor superfamily, member 9	TNFRSF9
219672_at	2.77	erythroid associated factor	ERAF
236078_at	2.77		
201556_s_at	2.77	vesicle-associated membrane protein 2 (synaptobrevin 2)	VAMP2
203192_at	2.77	ATP-binding cassette, sub-family B (MDR/TAP), member 6	ABCB6
206773_at	2.77	lymphocyte antigen 6 complex, locus H	LY6H
241633_x_at	2.76		
243746_at	2.76	immunoglobulin mu binding protein 2	IGHMBP2
236884_at	2.76		
205626_s_at	2.76	calbindin 1, 28kDa	CALB1
231616_at	2.76	glycophorin A (MNS blood group)	GYPA
239832_at	2.76		
241837_at	2.76	AT rich interactive domain 5B (MRF1-like)	ARID5B
1565582_at	2.76		
216068_at	2.76		
206622_at	2.75	thyrotropin-releasing hormone	TRH
215306_at	2.75		
205390_s_at	2.75	ankyrin 1, erythrocytic	ANK1
230640_at	2.74		
206209_s_at	2.74	carbonic anhydrase IV	CA4
231020_at	2.74	aristaless related homeobox	ARX
235967_at	2.74		
216249_at	2.74	Pvt1 oncogene homolog, MYC activator (mouse)	PVT1
216108_at	2.74		
236091_at	2.74	high-mobility group box 2	HMGB2
233004_x_at	2.73		
235501_at	2.73		
52651_at	2.73	collagen, type VIII, alpha 2	COL8A2
1554003_at	2.73		
215589_at	2.73		
241430_at	2.73	chromosome 2 open reading frame 51	C2orf51
226207_at	2.72		
236498_s_at	2.72	chromosome 1 open reading frame 86	C1orf86
220726_at	2.72		
232822_x_at	2.72		
222853_at	2.72	fibronectin leucine rich transmembrane protein 3	FLRT3
205923_at	2.72	reelin	RELN
206281_at	2.72	adenylate cyclase activating polypeptide 1 (pituitary)	ADCYAP1
239623_at	2.72		
225474_at	2.71	membrane associated guanylate kinase, WW and PDZ domain containing 1	MAGI1
217272_s_at	2.71	serpin peptidase inhibitor, clade B (ovalbumin), member 13	SERPINB13
241777_x_at	2.71	adaptor protein, phosphotyrosine interaction, PH domain and leucine zipper containing 2	APPL2
1564665_at	2.70		
207206_s_at	2.70	arachidonate 12-lipoxygenase	ALOX12
1559276_at	2.70		

234889_at	2.70	SPANX family, member N3	SPANXN3
1557617_at	2.70		
244865_at	2.70	HCLS1 associated protein X-1	HAX1
232885_at	2.70		
1560475_at	2.70		
235538_at	2.70		
211745_x_at	2.70	hemoglobin, alpha 2	HBA2
236005_at	2.70	Kruppel-like factor 12	KLF12
241851_x_at	2.69		
240445_at	2.69	protocadherin beta 14	PCDHB14
221538_s_at	2.69	plexin A1	PLXNA1
220118_at	2.69	zinc finger and BTB domain containing 32	ZBTB32
230446_at	2.68		
222142_at	2.68	cylindromatosis (turban tumor syndrome)	CYLD
237950_s_at	2.68		
240458_at	2.68	inositol 1,4,5-triphosphate receptor, type 2	ITPR2
1563720_at	2.68		
204934_s_at	2.67	hepsin (transmembrane protease, serine 1)	HPN
240965_at	2.67	anaphase promoting complex subunit 10	ANAPC10
215611_at	2.67	transcription factor 12 (HTF4, helix-loop-helix transcription factors 4)	TCF12
1557823_s_at	2.67		
221225_at	2.67	dephospho-CoA kinase domain containing	DCAKD
205389_s_at	2.67	ankyrin 1, erythrocytic	ANK1
1570122_at	2.67		
242227_at	2.67		
1569477_at	2.67		
1563884_at	2.67		
1560455_at	2.66		
235864_at	2.66	ATPase, H+ transporting, lysosomal 34kDa, V1 subunit D	ATP6V1D
226806_s_at	2.66		
213260_at	2.66		
1556624_at	2.66		
220918_at	2.66	chromosome 21 open reading frame 96	C21orf96
219181_at	2.66	lipase, endothelial	LIPG
1558425_x_at	2.65		
239971_at	2.65	chromosome 16 open reading frame 45	C16orf45
242695_at	2.65		
204416_x_at	2.65	apolipoprotein C-I	APOC1
1569921_at	2.65		
244789_at	2.65	chromosome 10 open reading frame 122	C10orf122
241081_at	2.65	UDP-N-acetyl-alpha-D-galactosamine:polypeptide N-acetylgalactosaminyltransferase 10 (GalNAc-T10)	GALNT10
207659_s_at	2.65	myelin-associated oligodendrocyte basic protein	MOBP
204321_at	2.65	neogenin homolog 1 (chicken)	NEO1
233853_at	2.65		
1557211_a_at	2.65	chromosome 14 open reading frame 86	C14orf86
1558447_at	2.65		
1568749_at	2.65		
240696_at	2.64		
1570639_at	2.64		
239227_at	2.64	exostoses (multiple) 1	EXT1

210714_at	2.64	R3H domain containing 1	R3HDM1
243689_s_at	2.64		
214913_at	2.64	ADAM metallopeptidase with thrombospondin type 1 motif, 3	ADAMTS3
243122_at	2.64		
236114_at	2.64		
207671_s_at	2.63	bestrophin 1	BEST1
223959_at	2.63	dynein, axonemal, light chain 1	DNAL1
228303_at	2.63	UDP-N-acetyl-alpha-D-galactosamine:polypeptide N-acetylgalactosaminyltransferase 6 (GalNAc-T6)	GALNT6
237794_at	2.63		
213843_x_at	2.62	solute carrier family 6 (neurotransmitter transporter, creatine), member 8	SLC6A8
1559591_s_at	2.62	choline dehydrogenase	CHDH
230728_at	2.62		
1559172_at	2.62		
211028_s_at	2.62	ketohexokinase (fructokinase)	KHK
1552658_a_at	2.62	neuron navigator 3	NAV3
1568699_at	2.62	chromosome 14 open reading frame 179	C14orf179
1556903_at	2.62		
240393_at	2.62	amino-terminal enhancer of split	AES
213486_at	2.62		
233613_x_at	2.62	REX2, RNA exonuclease 2 homolog (S. cerevisiae)	REXO2
239167_at	2.62		
227360_at	2.62	retinol dehydrogenase 13 (all-trans/9-cis)	RDH13
233664_at	2.61	ADP-ribosylation factor-like 15	ARL15
240845_at	2.61		
204141_at	2.61	tubulin, beta 2A	TUBB2A
1555372_at	2.61	BCL2-like 11 (apoptosis facilitator)	BCL2L11
1561658_at	2.61		
216876_s_at	2.60	interleukin 17A	IL17A
1569238_a_at	2.60		
236742_at	2.60		
239264_at	2.60		
217617_at	2.60		
217484_at	2.60	complement component (3b/4b) receptor 1 (Knops blood group)	CR1
202350_s_at	2.60	matrilin 2	MATN2
232852_at	2.59		
231436_at	2.59		
209458_x_at	2.59	hemoglobin, alpha 1	HBA1
205262_at	2.59	potassium voltage-gated channel, subfamily H (eag-related), member 2	KCNH2
232017_at	2.59	tight junction protein 2 (zona occludens 2)	TJP2
1559077_at	2.59	ABI gene family, member 3 (NESH) binding protein	ABI3BP
205862_at	2.58		
239452_at	2.58		
224079_at	2.58	interleukin 17C	IL17C
211699_x_at	2.58	hemoglobin, alpha 1	HBA1
1557889_at	2.58		
216773_at	2.58		
1562650_at	2.58	FRY-like	FRYL
214522_x_at	2.58	histone cluster 1, H2ad	HIST1H2AD

206025_s_at	2.58	tumor necrosis factor, alpha-induced protein 6	TNFAIP6
203216_s_at	2.58	myosin VI	MYO6
222272_x_at	2.58	scinderin	SCIN
233921_s_at	2.58		
234174_at	2.58		
240561_at	2.58		
234652_at	2.57		
215510_at	2.57	ets variant gene 2	ETV2
243720_at	2.57		
205234_at	2.57	solute carrier family 16, member 4 (monocarboxylic acid transporter 5)	SLC16A4
1557818_x_at	2.57		
215811_at	2.57		
1562598_at	2.57		
222940_at	2.57	sulfotransferase family 1E, estrogen-preferring, member 1	SULT1E1
214414_x_at	2.56	hemoglobin, alpha 1	HBA1
244558_at	2.56		
214618_at	2.56	CASP8 and FADD-like apoptosis regulator	CFLAR
211809_x_at	2.56	collagen, type XIII, alpha 1	COL13A1
244026_at	2.56	elongation factor, RNA polymerase II, 2	ELL2
207472_at	2.56		
1555988_a_at	2.56		
1552509_a_at	2.56	CD300 molecule-like family member g	CD300LG
243161_x_at	2.55	zinc finger protein 42 homolog (mouse)	ZFP42
221419_s_at	2.55		
244530_at	2.55	RAN binding protein 3-like	RANBP3L
216813_at	2.55		
230591_at	2.55		
230594_at	2.55		
219388_at	2.55	grainyhead-like 2 (Drosophila)	GRHL2
1557312_at	2.55	chromosome 12 open reading frame 61	C12orf61
229540_at	2.55	recombining binding protein suppressor of hairless (Drosophila)	RBPSUH
207810_at	2.55	coagulation factor XIII, B polypeptide	F13B
232896_at	2.55	erbb2 interacting protein	ERBB2IP
210081_at	2.55	advanced glycosylation end product-specific receptor	AGER
220595_at	2.55	PDZ domain containing RING finger 4	PDZRN4
239850_at	2.54		
230249_at	2.54	KH domain containing, RNA binding, signal transduction associated 3	KHDRBS3
239041_at	2.54	histone cluster 1, H2ak	HIST1H2AK
240088_at	2.54	phosphodiesterase 5A, cGMP-specific	PDE5A
214366_s_at	2.54	arachidonate 5-lipoxygenase	ALOX5
44783_s_at	2.54	hairy/enhancer-of-split related with YRPW motif 1	HEY1
222696_at	2.54	axin 2 (conductin, axil)	AXIN2
242762_s_at	2.53	KIAA1946	KIAA1946
234970_at	2.53	membrane targeting (tandem) C2 domain containing 1	MTAC2D1
238301_at	2.53		
215473_at	2.53		
237456_at	2.53		
241834_at	2.53		
1563477_at	2.53		

1566665_at	2.53		
242686_at	2.52	START domain containing 13	STARD13
222970_at	2.52		
1561129_at	2.52		
1561676_at	2.52		
206035_at	2.52	v-rel reticuloendotheliosis viral oncogene homolog (avian)	REL
1553708_at	2.52		
229374_at	2.52	EPH receptor A4	EPHA4
234563_at	2.52		
243073_at	2.52		
241775_at	2.52		
218950_at	2.52	centaurin, delta 3	CENTD3
205146_x_at	2.51	amyloid beta (A4) precursor protein-binding, family A, member 3 (X11-like 2)	APBA3
241159_x_at	2.51	integrin alpha FG-GAP repeat containing 1	ITFG1
216563_at	2.51	ankyrin repeat domain 12	ANKRD12
1557520_a_at	2.51	transmembrane protein 59	TMEM59
228877_at	2.50	erythropoietin receptor	EPOR
227877_at	2.50		
208446_s_at	2.50	zinc finger, FYVE domain containing 9	ZFYVE9
203963_at	2.50	carbonic anhydrase XII	CA12
244369_at	2.50	chromosome 21 open reading frame 59	C21orf59
222166_at	2.50	chromosome 9 open reading frame 16	C9orf16
1569583_at	2.50	epiregulin	EREG
206618_at	2.50	interleukin 18 receptor 1	IL18R1
1562888_at	2.50	galactosidase, beta 1 like 3	GLB1L3
1561002_at	2.49		
244358_at	2.49		
241464_s_at	2.49		
215635_at	2.49		
234573_at	2.49		
236338_at	2.49	insulin receptor substrate 2	IRS2
1563453_at	2.49		
234199_at	2.48		
240395_at	2.48		
204694_at	2.48	alpha-fetoprotein	AFP
240927_at	2.48		
229631_at	2.48	dynein heavy chain domain 1	DNHD1
235895_at	2.48		
231985_at	2.48	microtubule associated monoxygenase, calponin and LIM domain containing 3	MICAL3
1570628_at	2.48		
1556672_a_at	2.48	RNA binding motif protein 6	RBM6
237181_at	2.48	protein phosphatase 2, regulatory subunit B', gamma isoform	PPP2R5C
244480_at	2.48		
1553994_at	2.48	5'-nucleotidase, ecto (CD73)	NT5E
222549_at	2.48	claudin 1	CLDN1
1556344_at	2.48		
1559763_at	2.48	zinc finger CCCH-type containing 12C	ZC3H12C
1559063_at	2.48		
244227_at	2.47	synaptotagmin VI	SYT6

213524_s_at	2.47	G0/G1switch 2	G0S2
221251_x_at	2.47	zinc finger, HIT type 4	ZNHIT4
217274_x_at	2.47	myosin, light chain 4, alkali; atrial, embryonic	MYL4
226211_at	2.47	maternally expressed 3	MEG3
241016_at	2.47	cullin 3	CUL3
231247_s_at	2.47		
1560728_at	2.47		
228414_at	2.47	potassium large conductance calcium-activated channel, subfamily M, alpha member 1	KCNMA1
244076_at	2.47		
239028_at	2.46	LY6/PLAUR domain containing 6	LYPD6
232370_at	2.46		
233313_at	2.46		
208352_x_at	2.46	ankyrin 1, erythrocytic	ANK1
234640_x_at	2.46		
1555502_at	2.46		
1562303_at	2.46	zinc finger protein 306	ZNF306
227188_at	2.46	chromosome 21 open reading frame 63	C21orf63
218934_s_at	2.45	heat shock 27kDa protein family, member 7 (cardiovascular)	HSPB7
243597_at	2.45	Fanconi anemia, complementation group B	FANCB
224894_at	2.45	Yes-associated protein 1, 65kDa	YAP1
239870_at	2.45	spermatogenesis associated, serine-rich 1	SPATS1
237600_at	2.45	myeloid/lymphoid or mixed-lineage leukemia (trithorax homolog, Drosophila); translocated to, 10	MLLT10
1555284_at	2.45	amyotrophic lateral sclerosis 2 (juvenile)	ALS2
206776_x_at	2.45	acrosomal vesicle protein 1	ACRV1
1557394_at	2.45		
216029_at	2.45		
209204_at	2.44	LIM domain only 4	LMO4
1562288_at	2.44		
1553826_a_at	2.44		
1556829_at	2.44	TCDD-inducible poly(ADP-ribose) polymerase	TIPARP
203907_s_at	2.44	IQ motif and Sec7 domain 1	IQSEC1
1555192_at	2.44	zinc finger protein 277 pseudogene	ZNF277P
207051_at	2.44	solute carrier family 17 (sodium phosphate), member 4	SLC17A4
244535_at	2.44		
1569813_at	2.44	striatin, calmodulin binding protein	STRN
238899_at	2.43	KIAA1267	KIAA1267
243114_at	2.43		
208481_at	2.43	ankyrin repeat and SOCS box-containing 4	ASB4
1554492_at	2.43	thyroid adenoma associated	THADA
243030_at	2.43	mitogen-activated protein kinase kinase kinase 1	MAP3K1
215083_at	2.43		
234609_at	2.42		
236130_at	2.42	small nucleolar RNA, H/ACA box 37	SNORA37
1555246_a_at	2.42	sodium channel, voltage-gated, type I, alpha subunit	SCN1A
232110_at	2.42		
216284_at	2.42		
1561247_at	2.42		
214639_s_at	2.42	homeobox A1	HOXA1
205366_s_at	2.42	homeobox B6	HOXB6
1565601_at	2.42	protocadherin 9	PCDH9

235380_at	2.42		
225442_at	2.42	discoidin domain receptor family, member 2	DDR2
211965_at	2.42	zinc finger protein 36, C3H type-like 1	ZFP36L1
226700_at	2.42	U2 small nuclear RNA auxiliary factor 1-like 4	U2AF1L4
231142_at	2.42		
1555144_at	2.42		
207067_s_at	2.42	histidine decarboxylase	HDC
229954_at	2.41		
242068_at	2.41	baculoviral IAP repeat-containing 6 (apollon)	BIRC6
1564344_at	2.41	ATPase, Class II, type 9B	ATP9B
1559979_at	2.41	SYF2 homolog, RNA splicing factor (S. cerevisiae)	SYF2
234420_at	2.41		
207401_at	2.40	prospero-related homeobox 1	PROX1
214407_x_at	2.40	glycophorin B (MNS blood group)	GYPB
1565873_at	2.40	KIAA1279	KIAA1279
232614_at	2.40		
202555_s_at	2.40	myosin, light chain kinase	MYLK
1560118_at	2.40		
238712_at	2.40		
225767_at	2.40		
240544_at	2.40	zinc finger, AN1-type domain 3	ZFAND3
238588_at	2.39		
1559895_x_at	2.39		
236919_at	2.39	chromosome 18 open reading frame 25	C18orf25
232449_at	2.39	beta-carotene dioxygenase 2	BCDO2
1555842_at	2.39		
240656_at	2.39	signal-induced proliferation-associated 1 like 1	SIPA1L1
1556828_at	2.39		
220623_s_at	2.39	testis specific, 10	TSGA10
234074_at	2.39		
216614_at	2.39		
201694_s_at	2.38	early growth response 1	EGR1
205857_at	2.38	solute carrier family 18 (vesicular monoamine), member 2	SLC18A2
1562898_at	2.38		
227193_at	2.38		
236947_at	2.38		
235721_at	2.38	deltex 3 homolog (Drosophila)	DTX3
1559975_at	2.38	B-cell translocation gene 1, anti-proliferative	BTG1
1553466_at	2.38	chromosome X open reading frame 59	CXorf59
229065_at	2.38	solute carrier family 35, member F3	SLC35F3
243442_x_at	2.38		
237336_at	2.38	adducin 2 (beta)	ADD2
1570032_at	2.38	adaptor-related protein complex 3, beta 2 subunit	AP3B2
227376_at	2.38	GLI-Kruppel family member GLI3 (Greig cephalopolysyndactyly syndrome)	GLI3
1559362_at	2.38		
221071_at	2.38		
206199_at	2.38	carcinoembryonic antigen-related cell adhesion molecule 7	CEACAM7
231070_at	2.38	iodotyrosine deiodinase	IYD
1559406_at	2.37	ankyrin repeat domain 18A	ANKRD18A
230932_at	2.37		

231083_at	2.37	ets variant gene 5 (ets-related molecule)	ETV5
237511_at	2.37	deaminase domain containing 1	DEADC1
204595_s_at	2.37		
214920_at	2.37	thrombospondin, type I, domain containing 7A	THSD7A
1558950_at	2.37		
1570169_at	2.37	CUB and Sushi multiple domains 2	CSMD2
219879_s_at	2.37	chromosome 17 open reading frame 53	C17orf53
230004_at	2.36	ubiquitin specific peptidase 24	USP24
233040_at	2.36	pleckstrin homology domain containing, family A member 5	PLEKHA5
242736_at	2.36	sorbin and SH3 domain containing 1	SORBS1
205132_at	2.36	actin, alpha, cardiac muscle 1	ACTC1
237733_at	2.36		
1556568_a_at	2.36		
244508_at	2.36	septin 7	7-Sep
215189_at	2.36	keratin 86	KRT86
243640_x_at	2.36	CDC14 cell division cycle 14 homolog A (S. cerevisiae)	CDC14A
240238_at	2.36	TBC1 domain family, member 22A	TBC1D22A
240175_at	2.36		
203442_x_at	2.36	echinoderm microtubule associated protein like 3	EML3
239872_at	2.35		
220663_at	2.35	interleukin 1 receptor accessory protein-like 1	IL1RAPL1
215616_s_at	2.35	jumonji domain containing 2B	JMJD2B
1558714_at	2.35		
203065_s_at	2.35	caveolin 1, caveolae protein, 22kDa	CAV1
232779_at	2.35		
241679_at	2.35	A kinase (PRKA) anchor protein (gravin) 12	AKAP12
1557357_at	2.35		
226858_at	2.35	casein kinase 1, epsilon	CSNK1E
242022_at	2.35	pre-B-cell leukemia homeobox 1	PBX1
237202_at	2.35	pyroglutamyl-peptidase I	PGPEP1
228904_at	2.35	homeobox B3	HOXB3
1556518_at	2.34		
1567101_at	2.34		
1556359_at	2.34	chromosome 6 open reading frame 89	C6orf89
202729_s_at	2.34	latent transforming growth factor beta binding protein 1	LTBP1
1565617_at	2.34		
236923_x_at	2.34		
225615_at	2.34		
1562821_a_at	2.34		
1557987_at	2.34		
1562673_at	2.33		
242783_at	2.33		
1556942_at	2.33		
243834_at	2.33	trinucleotide repeat containing 6A	TNRC6A
231880_at	2.33	family with sequence similarity 40, member B	FAM40B
214469_at	2.32	histone cluster 1, H2ae	HIST1H2AE
216386_at	2.32		
200999_s_at	2.32	cytoskeleton-associated protein 4	CKAP4
211447_s_at	2.32	phosphodiesterase 4A, cAMP-specific (phosphodiesterase E2 dunce homolog, Drosophila)	PDE4A
233333_x_at	2.32	advillin	AVIL
232117_at	2.32		

231050_at	2.32	HRAS-like suppressor family, member 5	HRASLS5
240254_at	2.32	TRAF2 and NCK interacting kinase	TNIK
238688_at	2.32	tropomyosin 1 (alpha)	TPM1
1552322_at	2.32	family with sequence similarity 122C	FAM122C
1560705_at	2.32		
216658_at	2.32		
1558540_s_at	2.32		
1553808_a_at	2.32	NK2 transcription factor related, locus 3 (Drosophila)	NKX2-3
216633_s_at	2.32	phospholipase C, eta 1	PLCH1
230252_at	2.32	G protein-coupled receptor 92	GPR92
205268_s_at	2.32	adducin 2 (beta)	ADD2
209926_at	2.32	MADS box transcription enhancer factor 2, polypeptide B (myocyte enhancer factor 2B)	MEF2B
200884_at	2.31	creatine kinase, brain	CKB
1554143_a_at	2.31	SGT1, suppressor of G2 allele of SKP1 like 1 (S. cerevisiae)	SUGT1L1
206927_s_at	2.31	guanylate cyclase 1, soluble, alpha 2	GUCY1A2
236455_at	2.31		
239868_at	2.31		
1553066_at	2.31	trace amine associated receptor 9	TAAR9
214600_at	2.31	TEA domain family member 1 (SV40 transcriptional enhancer factor)	TEAD1
242108_at	2.31		
1563724_at	2.31		
215607_x_at	2.31	SMEK homolog 1, suppressor of mek1 (Dictyostelium)	SMEK1
242373_at	2.31		
232516_x_at	2.31	YY1 associated protein 1	YY1AP1
237982_at	2.31		
241929_at	2.31		
238044_at	2.31		
1562901_at	2.31		
229854_at	2.30	obscurin, cytoskeletal calmodulin and titin-interacting RhoGEF	OBSCN
220872_at	2.30		
222110_at	2.30	SUMO1/sentrin specific peptidase 5	SENP5
222950_at	2.30	NIPA-like domain containing 2	NPAL2
210479_s_at	2.30	RAR-related orphan receptor A	RORA
1564333_a_at	2.30	prosaposin-like 1	PSAPL1
1561254_at	2.30		
234505_at	2.30		
1565756_a_at	2.29		
222351_at	2.29	protein phosphatase 2 (formerly 2A), regulatory subunit A, beta isoform	PPP2R1B
229192_s_at	2.29	tubulin folding cofactor D	TBCD
220833_at	2.29		
232504_at	2.29		
221261_x_at	2.29	melanoma antigen family D, 4	MAGED4
240456_at	2.29		
208019_at	2.29	zinc finger protein 157	ZNF157
1562656_at	2.29		
1564378_a_at	2.29		
1559753_at	2.29		
1563569_at	2.29		

207608_x_at	2.29	cytochrome P450, family 1, subfamily A, polypeptide 2	CYP1A2
216856_s_at	2.28	deleted in lymphocytic leukemia, 2	DLEU2
230237_at	2.28		
210215_at	2.28	transferrin receptor 2	TFR2
1553960_at	2.28	sorting nexin family member 21	SNX21
207977_s_at	2.28	dermatopontin	DPT
201059_at	2.28	cortactin	CTTN
243396_at	2.28		
243437_at	2.28	GRIP and coiled-coil domain containing 1	GCC1
205391_x_at	2.28	ankyrin 1, erythrocytic	ANK1
229964_at	2.28	chromosome 9 open reading frame 152	C9orf152
1561915_at	2.28		
226111_s_at	2.28	zinc finger protein 385	ZNF385
222357_at	2.28	zinc finger and BTB domain containing 20	ZBTB20
239723_at	2.28		
1569140_at	2.27	ubiquitin protein ligase E3 component n-recognin 2	UBR2
204018_x_at	2.27	hemoglobin, alpha 1	HBA1
1556739_at	2.27		
239435_x_at	2.27	shroom family member 1	SHROOM1
1556798_a_at	2.27		
31874_at	2.27	growth arrest-specific 2 like 1	GAS2L1
213338_at	2.27	transmembrane protein 158	TMEM158
219450_at	2.27	chromosome 4 open reading frame 19	C4orf19
243585_at	2.27	ATPase type 13A5	ATP13A5
1566146_x_at	2.27		
227820_at	2.27	TBC1 domain family, member 25	TBC1D25
1562282_at	2.27	zinc finger protein 568	ZNF568
1556783_a_at	2.26		
229107_at	2.26		
211106_at	2.26	suppressor of Ty 3 homolog (S. cerevisiae)	SUPT3H
231107_at	2.26		
220150_s_at	2.26	chromosome 6 open reading frame 60	C6orf60
213418_at	2.26	heat shock 70kDa protein 6 (HSP70B')	HSPA6
241723_at	2.26	IQ motif containing GTPase activating protein 2	IQGAP2
227174_at	2.26	WD repeat domain 72	WDR72
236255_at	2.26		
1566678_at	2.25	matrix metallopeptidase 2 (gelatinase A, 72kDa gelatinase, 72kDa type IV collagenase)	MMP2
213813_x_at	2.25	RNA binding protein S1, serine-rich domain	RNPS1
222341_x_at	2.25		
1561148_at	2.25		
1552829_at	2.25	transmembrane protein 23	TMEM23
203862_s_at	2.25	actinin, alpha 2	ACTN2
1556126_s_at	2.25	G patch domain containing 2	GPATCH2
210414_at	2.25	fibronectin leucine rich transmembrane protein 1	FLRT1
243509_at	2.25		
231818_x_at	2.25		
1560631_at	2.25	calcium binding and coiled-coil domain 2	CALCOCO2
1558969_a_at	2.24	ribosomal protein L32 pseudogene 3	RPL32P3
229622_at	2.24		

234617_at	2.24	ubiquilin 3#olfactory receptor, family 51, subfamily B, member 4#olfactory receptor, family 51, subfamily B, member 2#olfactory receptor, family 51, subfamily B, member 5#olfactory receptor, family 51, subfamily B, member 6#olfactory receptor, family 51, subfamily M, member 1#olfactory receptor, family 51, subfamily I, member 1#olfactory receptor, family 51, subfamily I, member 2#olfactory receptor, family 52, subfamily D, member 1	UBQLN3#OR51B4#OR51B2#OR51B5#OR51B6#OR51M1#OR51I1#OR51I2#OR52D1
243724_at	2.24		
1566868_at	2.24		
207466_at	2.24	galanin	GAL
232768_at	2.24	cyclin B2	CCNB2
243185_at	2.24		
232953_at	2.24	protein-L-isoaspartate (D-aspartate) O-methyltransferase domain containing 2#chromosome 20 open reading frame 69	PCMTD2#C20orf69
1554041_at	2.24	chromosome 20 open reading frame 141	C20orf141
217925_s_at	2.24	chromosome 6 open reading frame 106	C6orf106
224346_at	2.24	RNA binding protein S1, serine-rich domain	RNPS1
244147_at	2.23		
214950_at	2.23	interleukin 9 receptor	IL9R
220957_at	2.23	cutaneous T-cell lymphoma-associated antigen 1	CTAGE1
234502_at	2.23		
235916_at	2.23	yippee-like 4 (Drosophila)	YPEL4
237389_at	2.23		
239890_s_at	2.23	chromosome 13 open reading frame 21	C13orf21
230928_at	2.23		
1565911_at	2.22		
213358_at	2.22	KIAA0802	KIAA0802
227597_at	2.22	DIS3 mitotic control homolog (S. cerevisiae)-like 2	DIS3L2
216248_s_at	2.22	nuclear receptor subfamily 4, group A, member 2	NR4A2
233976_at	2.22		
216956_s_at	2.22	integrin, alpha 2b (platelet glycoprotein IIb of IIb/IIIa complex, antigen CD41)	ITGA2B
243395_at	2.22		
232690_at	2.22		
206521_s_at	2.22	general transcription factor IIA, 1, 19/37kDa	GTF2A1
230988_at	2.22		
1563933_a_at	2.22	phospholipase D family, member 5	PLD5
203868_s_at	2.22	vascular cell adhesion molecule 1	VCAM1
224104_at	2.22		
207087_x_at	2.22	ankyrin 1, erythrocytic	ANK1
239297_at	2.22		
1561059_a_at	2.22		
233203_at	2.22	ropporin, rhophilin associated protein 1	ROPN1
1557712_x_at	2.22		
240768_x_at	2.21		
1562862_at	2.21		
230092_at	2.21	UBX domain containing 3	UBXD3
234968_at	2.21	DENN/MADD domain containing 4C	DENND4C
227591_at	2.21	SH3-domain binding protein 5 (BTK-associated)	SH3BP5
227679_at	2.21		

219411_at	2.21	engulfment and cell motility 3	ELMO3
243691_at	2.20	DCN1, defective in cullin neddylation 1, domain containing 4 (S. cerevisiae)	DCUN1D4
1560049_at	2.20		
237165_at	2.20	gelsolin (amyloidosis, Finnish type)	GSN
1558691_a_at	2.20	dedicator of cytokinesis 4	DOCK4
217619_x_at	2.20		
243068_at	2.20		
233744_at	2.20	RAS-like, estrogen-regulated, growth inhibitor	RERG
242606_at	2.20		
244084_at	2.20	apoptosis-inducing factor, mitochondrion-associated, 3	AIFM3
215197_at	2.20		
203939_at	2.20	5'-nucleotidase, ecto (CD73)	NT5E
1559648_at	2.19		
207779_at	2.19		
229617_x_at	2.19	adaptor-related protein complex 2, alpha 1 subunit	AP2A1
242298_x_at	2.19		
239886_at	2.19		
234209_at	2.19		
211005_at	2.19	linker for activation of T cells	LAT
240216_at	2.19		
232968_at	2.19	fibronectin type III and ankyrin repeat domains 1	FANK1
219508_at	2.19	glucosaminyl (N-acetyl) transferase 3, mucin type	GCNT3
1554380_at	2.19	NIMA (never in mitosis gene a)- related kinase 11	NEK11
236427_at	2.19	WW domain containing oxidoreductase	WWOX
218456_at	2.19	C1q domain containing 1	C1QDC1
242216_at	2.18		
205294_at	2.18	BAI1-associated protein 2	BAIAP2
236385_at	2.18		
227243_s_at	2.18	early B-cell factor 3	EBF3
1562271_x_at	2.18	Rho guanine nucleotide exchange factor (GEF) 7	ARHGEF7
223644_s_at	2.18	crystallin, gamma S	CRYGS
244697_at	2.18	zinc finger and BTB domain containing 16	ZBTB16
237556_at	2.18		
206077_at	2.17	Kell blood group, metallo-endopeptidase	KEL
218033_s_at	2.17	stannin	SNN
205307_s_at	2.17	kynurenine 3-monooxygenase (kynurenine 3-hydroxylase)	KMO
242769_at	2.17		
230093_at	2.17	testis specific A2 homolog (mouse)	TSGA2
1566835_at	2.17		
224082_at	2.17		
220795_s_at	2.17		
220784_s_at	2.17	urotensin 2	UTS2
238889_at	2.17		
232288_at	2.17		
221558_s_at	2.17	lymphoid enhancer-binding factor 1	LEF1
243826_at	2.16		
243050_at	2.16	peripheral myelin protein 22	PMP22
241434_at	2.16		
230143_at	2.16	ring finger protein 165	RNF165
214349_at	2.16		
243919_at	2.16	SAFB-like, transcription modulator	SLTM

243756_at	2.16	thrombospondin, type I, domain containing 7A	THSD7A
216070_at	2.16		
234643_x_at	2.16		
210641_at	2.16	calpain 9	CAPN9
241737_x_at	2.16		
224024_at	2.16	endoplasmic reticulum-golgi intermediate compartment (ERGIC) 1	ERGIC1
1570230_at	2.16		
227140_at	2.16		
214464_at	2.16	CDC42 binding protein kinase alpha (DMPK-like)	CDC42BPA
216529_at	2.16		
215818_at	2.16	nudix (nucleoside diphosphate linked moiety X)-type motif 7	NUDT7
239664_at	2.15		
211343_s_at	2.15	collagen, type XIII, alpha 1	COL13A1
210515_at	2.15	transcription factor 1, hepatic; LF-B1, hepatic nuclear factor (HNF1), albumin proximal factor	TCF1
240446_at	2.15	mindbomb homolog 1 (Drosophila)	MIB1
242732_at	2.15	metastasis suppressor 1	MTSS1
205729_at	2.15	oncostatin M receptor	OSMR
233059_at	2.15		
1566599_at	2.15		
221386_at	2.15	olfactory receptor, family 3, subfamily A, member 2	OR3A2
217702_at	2.15	interleukin 27 receptor, alpha	IL27RA
238949_at	2.15		
1561879_at	2.15		
239551_at	2.15		
201839_s_at	2.15	tumor-associated calcium signal transducer 1	TACSTD1
236062_at	2.14		
233736_at	2.14	ubiquilin 3#olfactory receptor, family 51, subfamily B, member 4#olfactory receptor, family 51, subfamily B, member 2#olfactory receptor, family 51, subfamily B, member 5#olfactory receptor, family 51, subfamily B, member 6#olfactory receptor, family 51, subfamily M, member 1#olfactory receptor, family 51, subfamily I, member 1#olfactory receptor, family 51, subfamily I, member 2#olfactory receptor, family 52, subfamily D, member 1	UBQLN3#OR51B4#OR51B2#OR51B5#OR51B6#OR51M1#OR51I1#OR51I2#OR52D1
215999_at	2.14	CMT1A duplicated region transcript 1	CDRT1
210358_x_at	2.14	GATA binding protein 2	GATA2
220803_at	2.14	STAM binding protein-like 1	STAMBPL1
227306_at	2.14		
215907_at	2.14		
242564_at	2.14		
1566001_at	2.14		
244143_at	2.14		
1569344_a_at	2.14		
1562250_at	2.14		
1558862_at	2.14	neuroblastoma breakpoint family, member 5	NBPF5
37996_s_at	2.14	dystrophia myotonica-protein kinase#dystrophia myotonica-containing WD repeat motif	DMPK#DMWD
204446_s_at	2.13	arachidonate 5-lipoxygenase	ALOX5
221838_at	2.13	kelch-like 22 (Drosophila)	KLHL22

207005_s_at	2.13	B-cell CLL/lymphoma 2	BCL2
203461_at	2.13	chromodomain helicase DNA binding protein 2	CHD2
230008_at	2.13	thrombospondin, type I, domain containing 7A	THSD7A
230113_at	2.13		
1554558_at	2.13	WD repeat domain 22	WDR22
226205_at	2.13	ankyrin repeat domain 13 family, member D	ANKRD13D
1565898_at	2.13	methyltransferase 5 domain containing 1	METT5D1
244891_x_at	2.13		
202175_at	2.13		
1560706_at	2.13		
217636_at	2.13	polymerase (DNA directed), gamma	POLG
215816_at	2.13	immunoglobulin lambda-like polypeptide 2	IGLL2
222809_x_at	2.13	chromosome 14 open reading frame 65	C14orf65
1562413_at	2.13	chromosome 11 open reading frame 37	C11orf37
1555505_a_at	2.13	tyrosinase (oculocutaneous albinism IA)	TYR
221152_at	2.13	collagen, type VIII, alpha 1	COL8A1
214222_at	2.13	dynein, axonemal, heavy chain 7	DNAH7
1567166_at	2.13		
232158_x_at	2.13	NIPA-like domain containing 1	NPAL1
238460_at	2.12	family with sequence similarity 83, member A	FAM83A
227599_at	2.12	chromosome 3 open reading frame 59	C3orf59
235301_at	2.12	KIAA1324-like	KIAA1324L
1561942_x_at	2.12		
227096_at	2.12	Josephin domain containing 2	JOSD2
208557_at	2.12	homeobox A6	HOXA6
225271_at	2.12	transmembrane protein 63B	TMEM63B
229831_at	2.12	contactin 3 (plasmacytoma associated)	CNTN3
233295_at	2.12		
206467_x_at	2.12	tumor necrosis factor receptor superfamily, member 6b, decoy	TNFRSF6B
239982_at	2.12		
228252_at	2.12	PIF1 5'-to-3' DNA helicase homolog (S. cerevisiae)	PIF1
206738_at	2.12	apolipoprotein C-IV	APOC4
1562850_at	2.11		
1560865_a_at	2.11		
1553561_at	2.11	taste receptor, type 2, member 50	TAS2R50
232144_at	2.11	pre-B-cell leukemia homeobox 1	PBX1
212443_at	2.11	neurobeachin-like 2	NBEAL2
233210_at	2.11		
232530_at	2.11	phospholipase D1, phosphatidylcholine-specific	PLD1
242306_at	2.11	transmembrane protein 165	TMEM165
1565723_at	2.11	LSM domain containing 1	LSMD1
238413_at	2.11	cerebellar degeneration-related protein 2, 62kDa	CDR2
244162_at	2.11		
220601_at	2.11	chromosome 16 open reading frame 70	C16orf70
205347_s_at	2.11	thymosin-like 8	TMSL8
1555217_at	2.11	ubiquitin-conjugating enzyme E2W (putative)	UBE2W
242829_x_at	2.10	F-box and leucine-rich repeat protein 3	FBXL3
209352_s_at	2.10	SIN3 homolog B, transcription regulator (yeast)	SIN3B
1562484_at	2.10		
205266_at	2.10	leukemia inhibitory factor (cholinergic differentiation factor)	LIF

210036_s_at	2.10	potassium voltage-gated channel, subfamily H (eag-related), member 2	KCNH2
227400_at	2.10	nuclear factor I/X (CCAAT-binding transcription factor)	NFIX
240450_at	2.10		
244658_at	2.10		
243915_at	2.10		
201884_at	2.10	carcinoembryonic antigen-related cell adhesion molecule 5	CEACAM5
1558202_at	2.10		
231632_at	2.10		
212771_at	2.09	chromosome 10 open reading frame 38	C10orf38
202728_s_at	2.09	latent transforming growth factor beta binding protein 1	LTBP1
213777_s_at	2.09	CREB/ATF bZIP transcription factor	CREBZF
206432_at	2.09	hyaluronan synthase 2	HAS2
1570125_at	2.09		
210446_at	2.09	GATA binding protein 1 (globin transcription factor 1)	GATA1
202478_at	2.09	tribbles homolog 2 (Drosophila)	TRIB2
235884_at	2.09		
212392_s_at	2.09	phosphodiesterase 4D interacting protein (myomegalin)	PDE4DIP
219255_x_at	2.08	interleukin 17 receptor B	IL17RB
336_at	2.08	thromboxane A2 receptor	TBXA2R
243583_at	2.08	transcription factor 7-like 2 (T-cell specific, HMG-box)	TCF7L2
1560483_at	2.08		
231272_at	2.08		
236722_at	2.08		
213529_at	2.08	zinc finger protein 688	ZNF688
205198_s_at	2.08	ATPase, Cu++ transporting, alpha polypeptide (Menkes syndrome)	ATP7A
243968_x_at	2.08	Fc receptor-like 1	FCRL1
1554556_a_at	2.08	ATPase, Class VI, type 11B	ATP11B
209457_at	2.07	dual specificity phosphatase 5	DUSP5
1566039_a_at	2.07		
228309_at	2.07	chromosome 20 open reading frame 3	C20orf3
224274_at	2.07	chromosome 14 open reading frame 155	C14orf155
1570042_a_at	2.07	ADAM metallopeptidase domain 9 (meltrin gamma)	ADAM9
1568907_at	2.07		
205767_at	2.07	epiregulin	EREG
234284_at	2.07	guanine nucleotide binding protein (G protein), gamma 8	GNG8
231476_at	2.07		
205120_s_at	2.07	sarcoglycan, beta (43kDa dystrophin-associated glycoprotein)	SGCB
1565692_at	2.07		
211095_at	2.07	neurofibromin 1 (neurofibromatosis, von Recklinghausen disease, Watson disease)	NF1
209716_at	2.06	colony stimulating factor 1 (macrophage)	CSF1
229198_at	2.06	ubiquitin specific peptidase 35	USP35
217728_at	2.06	S100 calcium binding protein A6	S100A6
243559_at	2.06		
242730_at	2.06		
1553153_at	2.06	ATPase, H+ transporting, lysosomal 38kDa, V0 subunit d2	ATP6V0D2

239906_at	2.06		
207876_s_at	2.06	filamin C, gamma (actin binding protein 280)	FLNC
239441_at	2.06	zinc finger protein 780A	ZNF780A
207308_at	2.06	solute carrier organic anion transporter family, member 1A2	SLCO1A2
237916_at	2.06		
244302_at	2.06	eukaryotic translation initiation factor 4E family member 2	EIF4E2
216113_at	2.06	abl interactor 2	ABI2
228377_at	2.06	kelch-like 14 (Drosophila)	KLHL14
1562919_at	2.06	family with sequence similarity 45, member A	FAM45A
215098_at	2.05	retinoid X receptor, beta	RXRB
209469_at	2.05	glycoprotein M6A	GPM6A
203394_s_at	2.05	hairy and enhancer of split 1, (Drosophila)	HES1
217684_at	2.05	thymidylate synthetase	TYMS
206585_at	2.05	makorin, ring finger protein, 3	MKRN3
228854_at	2.05		
244868_at	2.05	zinc fingers and homeoboxes 2	ZHX2
231925_at	2.05		
237100_at	2.05	janus kinase and microtubule interacting protein 2	JAKMIP2
237566_at	2.05		
208886_at	2.05	H1 histone family, member 0	H1F0
209324_s_at	2.05	regulator of G-protein signalling 16	RGS16
206115_at	2.05	early growth response 3	EGR3
201655_s_at	2.05	heparan sulfate proteoglycan 2 (perlecan)	HSPG2
209079_x_at	2.04	protocadherin gamma subfamily A, 1	PCDHGA1
232444_at	2.04	chromosome 6 open reading frame 204	C6orf204
1563469_at	2.04		
215298_at	2.04		
1561363_a_at	2.04		
48031_r_at	2.04	chromosome 5 open reading frame 4	C5orf4
214395_x_at	2.04	eukaryotic translation elongation factor 1 delta (guanine nucleotide exchange protein)	EEF1D
1554816_at	2.04	astrotactin 2	ASTN2
215987_at	2.04	Rap guanine nucleotide exchange factor (GEF) 2	RAPGEF2
201508_at	2.04	insulin-like growth factor binding protein 4	IGFBP4
241614_at	2.04		
243617_at	2.04		
225451_at	2.04	GRIP1 associated protein 1	GRIPAP1
209795_at	2.04	CD69 molecule	CD69
1568936_a_at	2.04		
231949_at	2.04		
234150_at	2.04		
1565000_a_at	2.04	t-complex 11 (mouse)-like 2	TCP11L2
221176_x_at	2.04	Williams-Beuren syndrome chromosome region 23	WBSCR23
227899_at	2.04	vitrin	VIT
219474_at	2.03	chromosome 3 open reading frame 52	C3orf52
204151_x_at	2.03	aldo-keto reductase family 1, member C1 (dihydrodiol dehydrogenase 1; 20-alpha (3-alpha)-hydroxyster	AKR1C1
213109_at	2.03	TRAF2 and NCK interacting kinase	TNIK
215497_s_at	2.03	WD and tetratricopeptide repeats 1	WDTC1
1553323_a_at	2.03	cation channel, sperm associated 2	CATSPER2
235852_at	2.03	stonin 2	STON2

213600_at	2.03	signal-induced proliferation-associated 1 like 3	SIPA1L3
227155_at	2.03		
244695_at	2.03		
211481_at	2.03	solute carrier organic anion transporter family, member 1A2	SLCO1A2
1556201_at	2.03	ribonuclease T2	RNASET2
1554108_at	2.03		
216040_x_at	2.03		
1556879_at	2.03		
241951_at	2.03		
219186_at	2.02	zinc finger and BTB domain containing 7A	ZBTB7A
235421_at	2.02		
215321_at	2.02		
224466_s_at	2.02	v-maf musculoaponeurotic fibrosarcoma oncogene homolog G (avian)	MAFG
206675_s_at	2.02	SKI-like oncogene	SKIL
230170_at	2.02	oncostatin M	OSM
243451_at	2.02		
228910_at	2.02		
213744_at	2.02	attractin-like 1	ATRNL1
235074_at	2.02	sprouty-related, EVH1 domain containing 1	SPRED1
206283_s_at	2.02	T-cell acute lymphocytic leukemia 1	TAL1
217651_at	2.02		
1553123_at	2.02	WD repeat domain 62	WDR62
1560104_at	2.02		
218552_at	2.02	enoyl Coenzyme A hydratase domain containing 2	ECHDC2
214679_x_at	2.02	guanine nucleotide binding protein (G protein), alpha 11 (Gq class)	GNA11
1555392_at	2.02		
215054_at	2.02	erythropoietin receptor	EPOR
1565939_at	2.02	chromosome 5 open reading frame 22	C5orf22
243560_at	2.02		
1564932_at	2.01		
227208_at	2.01	coiled-coil domain containing 84	CCDC84
239130_at	2.01		
228199_at	2.01		
1569417_at	2.01		
1568745_at	2.01		
220115_s_at	2.01	cadherin 10, type 2 (T2-cadherin)	CDH10
1554987_at	2.01	golgi autoantigen, golgin subfamily a, 3	GOLGA3
239959_x_at	2.01	phosphodiesterase 3B, cGMP-inhibited	PDE3B
223729_at	2.01	cat eye syndrome chromosome region, candidate 2	CECR2
202807_s_at	2.01	target of myb1 (chicken)	TOM1
205315_s_at	2.01	syntrophin, beta 2 (dystrophin-associated protein A1, 59kDa, basic component 2)	SNTB2
226697_at	2.01	family with sequence similarity 114, member A1	FAM114A1
1554866_at	2.01	transmembrane protein 135	TMEM135
217937_s_at	2.01	histone deacetylase 7A	HDAC7A
228010_at	2.01	protein phosphatase 2 (formerly 2A), regulatory subunit B, gamma isoform	PPP2R2C
1554234_at	2.01	katanin p60 subunit A-like 2	KATNAL2
1556528_at	2.00		
207489_at	2.00		

212503_s_at	2.00	DIP2 disco-interacting protein 2 homolog C (Drosophila)	DIP2C
209442_x_at	2.00	ankyrin 3, node of Ranvier (ankyrin G)	ANK3
205683_x_at	2.00	tryptase alpha/beta 1	TPSAB1
207964_x_at	2.00	interferon, alpha 4	IFNA4
1563941_at	2.00		
207082_at	2.00	colony stimulating factor 1 (macrophage)	CSF1
1560051_at	2.00		
238959_at	0.50	La ribonucleoprotein domain family, member 4	LARP4
208910_s_at	0.50	complement component 1, q subcomponent binding protein	C1QBP
201923_at	0.50	peroxiredoxin 4	PRDX4
1557918_s_at	0.50	solute carrier family 16, member 1 (monocarboxylic acid transporter 1)	SLC16A1
1553192_at	0.50	zinc finger protein 441	ZNF441
223996_s_at	0.50	mitochondrial ribosomal protein L30	MRPL30
229606_at	0.50		
201627_s_at	0.50	insulin induced gene 1	INSIG1
211150_s_at	0.50	dihydrolipoamide S-acetyltransferase (E2 component of pyruvate dehydrogenase complex)	DLAT
212619_at	0.50		
200738_s_at	0.50	phosphoglycerate kinase 1	PGK1
205726_at	0.50	diaphanous homolog 2 (Drosophila)	DIAPH2
221592_at	0.50	ribosomal protein L31	RPL31
201566_x_at	0.50	inhibitor of DNA binding 2, dominant negative helix-loop-helix protein	ID2
222983_s_at	0.50	poly(A) binding protein interacting protein 2	PAIP2
210567_s_at	0.50	S-phase kinase-associated protein 2 (p45)	SKP2
239045_at	0.50		
242312_x_at	0.50		
203646_at	0.50	ferredoxin 1	FDX1
229367_s_at	0.50	GTPase, IMAP family member 6	GIMAP6
229309_at	0.50		
219979_s_at	0.50	chromosome 11 open reading frame 73	C11orf73
210754_s_at	0.50	v-yes-1 Yamaguchi sarcoma viral related oncogene homolog	LYN
242228_at	0.50		
214875_x_at	0.50	amyloid beta (A4) precursor-like protein 2	APLP2
243738_at	0.50	nicotinamide nucleotide adenylyltransferase 3	NMNAT3
237819_at	0.50	cAMP responsive element binding protein 3-like 2	CREB3L2
235088_at	0.50		
244814_at	0.50		
205822_s_at	0.50	3-hydroxy-3-methylglutaryl-Coenzyme A synthase 1 (soluble)	HMGCS1
222459_at	0.50	chromosome 1 open reading frame 108	C1orf108
217995_at	0.50	sulfide quinone reductase-like (yeast)	SQRDL
201330_at	0.50	arginyl-tRNA synthetase	RARS
219954_s_at	0.50	glucosidase, beta, acid 3 (cytosolic)	GBA3
236477_at	0.49	PRP40 pre-mRNA processing factor 40 homolog A (S. cerevisiae)	PRPF40A
219187_at	0.49	FK506 binding protein like	FKBPL
201670_s_at	0.49	myristoylated alanine-rich protein kinase C substrate	MARCKS
220721_at	0.49	zinc finger protein 614	ZNF614

211622_s_at	0.49	ADP-ribosylation factor 3	ARF3
204864_s_at	0.49	interleukin 6 signal transducer (gp130, oncostatin M receptor)	IL6ST
221212_x_at	0.49		
212415_at	0.49	septin 6	6-Sep
212671_s_at	0.49	major histocompatibility complex, class II, DQ alpha 1	HLA-DQA1
1554464_a_at	0.49	cartilage associated protein	CRTAP
214995_s_at	0.49	apolipoprotein B mRNA editing enzyme, catalytic polypeptide-like 3F	APOBEC3F
211744_s_at	0.49	CD58 molecule	CD58
201298_s_at	0.49	MOB1, Mps One Binder kinase activator-like 1B (yeast)	MOBK1B
205996_s_at	0.49	adenylate kinase 2	AK2
210506_at	0.49	fucosyltransferase 7 (alpha (1,3) fucosyltransferase)	FUT7
207269_at	0.49	defensin, alpha 4, corticostatin	DEFA4
1554883_a_at	0.49	excision repair cross-complementing rodent repair deficiency, complementation group 8	ERCC8
213789_at	0.49	TBC1 domain family, member 25	TBC1D25
218516_s_at	0.49	inositol monophosphatase domain containing 1	IMPAD1
227086_at	0.49		
231840_x_at	0.49	Lyrm7 homolog (mouse)	LYRM7
208310_s_at	0.49	follistatin-like 1	FSTL1
203922_s_at	0.49	cytochrome b-245, beta polypeptide (chronic granulomatous disease)	CYBB
224660_at	0.49	phosphatidylinositol glycan anchor biosynthesis, class Y	PIGY
218264_at	0.49	BRCA2 and CDKN1A interacting protein	BCCIP
202873_at	0.49	ATPase, H+ transporting, lysosomal 42kDa, V1 subunit C1	ATP6V1C1
235180_at	0.49	serine/threonine/tyrosine interacting protein	STYX
229846_s_at	0.49	mitogen-activated protein kinase associated protein 1	MAPKAP1
235341_at	0.49		
1557411_s_at	0.49	solute carrier family 25, member 43	SLC25A43
202672_s_at	0.49	activating transcription factor 3	ATF3
228239_at	0.49	chromosome 21 open reading frame 51	C21orf51
214666_x_at	0.49		
203087_s_at	0.49	kinesin heavy chain member 2A	KIF2A
216100_s_at	0.49	torsin A interacting protein 1	TOR1AIP1
210950_s_at	0.49	farnesyl-diphosphate farnesyltransferase 1	FDFT1
211367_s_at	0.49	caspase 1, apoptosis-related cysteine peptidase (interleukin 1, beta, convertase)	CASP1
226748_at	0.49	LysM, putative peptidoglycan-binding, domain containing 2	LYSMD2
216064_s_at	0.49	aspartylglucosaminidase	AGA
227514_at	0.49		
206127_at	0.49	ELK3, ETS-domain protein (SRF accessory protein 2)	ELK3
217477_at	0.49	phosphatidylinositol-4-phosphate 5-kinase, type I, beta	PIP5K1B
225759_x_at	0.49	calmin (calponin-like, transmembrane)	CLMN
204005_s_at	0.49	PRKC, apoptosis, WT1, regulator	PAWR
1552703_s_at	0.49		
236917_at	0.49	leucine rich repeat containing 34	LRRC34
217310_s_at	0.49	forkhead box J3	FOXJ3
211990_at	0.49	major histocompatibility complex, class II, DP alpha 1	HLA-DPA1

222501_s_at	0.49	replication initiator 1	REPIN1
212497_at	0.49	chromosome 14 open reading frame 32	C14orf32
224786_at	0.49	short coiled-coil protein	SCOC
240078_at	0.49	splicing factor, arginine/serine-rich 8 (suppressor-of-white-apricot homolog, Drosophila)	SFRS8
214581_x_at	0.49	tumor necrosis factor receptor superfamily, member 21	TNFRSF21
1555176_at	0.49		
208966_x_at	0.49	interferon, gamma-inducible protein 16	IFI16
218569_s_at	0.49	kelch repeat and BTB (POZ) domain containing 4	KBTBD4
1560533_at	0.49		
221220_s_at	0.48	SCY1-like 2 (S. cerevisiae)	SCYL2
211379_x_at	0.48	beta-1,3-N-acetylgalactosaminyltransferase 1 (globoside blood group)	B3GALNT1
208643_s_at	0.48	X-ray repair complementing defective repair in Chinese hamster cells 5 (double-strand-break rejoinin	XRCC5
225029_at	0.48		
201339_s_at	0.48	sterol carrier protein 2	SCP2
210371_s_at	0.48	retinoblastoma binding protein 4	RBBP4
203213_at	0.48	cell division cycle 2, G1 to S and G2 to M	CDC2
1558522_at	0.48		
239563_at	0.48		
203582_s_at	0.48	RAB4A, member RAS oncogene family	RAB4A
235924_at	0.48		
241623_at	0.48	protein tyrosine phosphatase, non-receptor type 2	PTPN2
209218_at	0.48	squalene epoxidase	SQLE
201656_at	0.48	integrin, alpha 6	ITGA6
222846_at	0.48	RAB8B, member RAS oncogene family	RAB8B
214754_at	0.48		
218025_s_at	0.48	peroxisomal D3,D2-enoyl-CoA isomerase	PECI
227089_at	0.48		
216521_s_at	0.48	BRCA1/BRCA2-containing complex, subunit 3	BRCC3
204429_s_at	0.48	solute carrier family 2 (facilitated glucose/fructose transporter), member 5	SLC2A5
224153_s_at	0.48		
1555705_a_at	0.48	CKLF-like MARVEL transmembrane domain containing 3	CMTM3
230854_at	0.48	breast cancer anti-estrogen resistance 4	BCAR4
219449_s_at	0.48	transmembrane protein 70	TMEM70
236132_at	0.48		
202139_at	0.48	aldo-keto reductase family 7, member A2 (aflatoxin aldehyde reductase)	AKR7A2
238483_at	0.48		
223698_at	0.48	solute carrier family 25, member 36	SLC25A36
217666_at	0.48		
211704_s_at	0.48	spindlin family, member 2B	SPIN2B
238320_at	0.48		
240277_at	0.48		
216532_x_at	0.48	PRP4 pre-mRNA processing factor 4 homolog B (yeast)#chromosome 6 open reading frame 50	PRPF4B#C6orf50
239465_at	0.48		
200973_s_at	0.48	tetraspanin 3	TSPAN3
216985_s_at	0.48	syntaxin 3	STX3
234953_x_at	0.48	zinc finger protein 19	ZNF19

201259_s_at	0.48	synaptophysin-like 1	SYPL1
200722_s_at	0.48	GPI-anchored membrane protein 1	GPIAP1
223318_s_at	0.48	alkB, alkylation repair homolog 7 (E. coli)	ALKBH7
210599_at	0.48	zinc finger protein 614	ZNF614
229689_s_at	0.48		
200988_s_at	0.48	proteasome (prosome, macropain) activator subunit 3 (PA28 gamma; Ki)	PSME3
201486_at	0.48	reticulocalbin 2, EF-hand calcium binding domain	RCN2
214895_s_at	0.48	ADAM metallopeptidase domain 10	ADAM10
234304_s_at	0.48	importin 11	IPO11
217114_at	0.48	activating signal cointegrator 1 complex subunit 3-like 1	ASCC3L1
1553316_at	0.48	G protein-coupled receptor 82	GPR82
219607_s_at	0.47	membrane-spanning 4-domains, subfamily A, member 4	MS4A4A
227787_s_at	0.47	thyroid hormone receptor associated protein 6	THRAP6
236591_at	0.47		
211864_s_at	0.47	fer-1-like 3, myoferlin (C. elegans)	FER1L3
1555797_a_at	0.47	actin related protein 2/3 complex, subunit 5, 16kDa	ARPC5
1561358_at	0.47	taxilin alpha	TXLNA
238506_at	0.47	leucine rich repeat containing 58	LRRC58
201476_s_at	0.47	ribonucleotide reductase M1 polypeptide	RRM1
235321_at	0.47		
241666_at	0.47	chromosome 3 open reading frame 23	C3orf23
227231_at	0.47		
211431_s_at	0.47	TYRO3 protein tyrosine kinase	TYRO3
1554451_s_at	0.47	DnaJ (Hsp40) homolog, subfamily C, member 14	DNAJC14
1564494_s_at	0.47	procollagen-proline, 2-oxoglutarate 4-dioxygenase (proline 4-hydroxylase), beta polypeptide	P4HB
208290_s_at	0.47	eukaryotic translation initiation factor 5	EIF5
207079_s_at	0.47	mediator of RNA polymerase II transcription, subunit 6 homolog (S. cerevisiae)	MED6
211563_s_at	0.47	chromosome 19 open reading frame 2	C19orf2
208852_s_at	0.47	calnexin	CANX
1558014_s_at	0.47	male sterility domain containing 2	MLSTD2
202810_at	0.47	developmentally regulated GTP binding protein 1	DRG1
223262_s_at	0.47	FGFR1 oncogene partner 2	FGFR1OP2
204452_s_at	0.47	frizzled homolog 1 (Drosophila)	FZD1
205978_at	0.47	klotho	KL
226134_s_at	0.47		
242577_at	0.47		
225796_at	0.47	PX domain containing serine/threonine kinase	PXK
1562771_at	0.47		
223879_s_at	0.47	oxidation resistance 1	OXR1
1554834_a_at	0.47	Ras association (RalGDS/AF-6) domain family 5	RASSF5
201504_s_at	0.47	translin	TSN
211088_s_at	0.47	polo-like kinase 4 (Drosophila)	PLK4
212956_at	0.47	TBC1 domain family, member 9 (with GRAM domain)	TBC1D9
210148_at	0.47	homeodomain interacting protein kinase 3	HIPK3
1555731_a_at	0.47	adaptor-related protein complex 1, sigma 3 subunit	AP1S3
207974_s_at	0.47	S-phase kinase-associated protein 1A (p19A)	SKP1A
209372_x_at	0.47	tubulin, beta 2A	TUBB2A

231101_at	0.47	protein phosphatase 2, regulatory subunit B', epsilon isoform	PPP2R5E
205227_at	0.47	interleukin 1 receptor accessory protein	IL1RAP
203923_s_at	0.47	cytochrome b-245, beta polypeptide (chronic granulomatous disease)	CYBB
236948_x_at	0.47	splicing factor, arginine/serine-rich 11	SFRS11
1552717_s_at	0.47	centrosomal protein 170kDa-like	CEP170L
1561042_at	0.47	integrin, beta 1 (fibronectin receptor, beta polypeptide, antigen CD29 includes MDF2, MSK12)	ITGB1
1552978_a_at	0.47	secretory carrier membrane protein 1	SCAMP1
219718_at	0.47		
211221_at	0.47	transcriptional adaptor 3 (NGG1 homolog, yeast)-like	TADA3L
201506_at	0.47	transforming growth factor, beta-induced, 68kDa	TGFBI
237706_at	0.47	syntaxin binding protein 4	STXBP4
204222_s_at	0.47	GLI pathogenesis-related 1 (glioma)	GLIPR1
204614_at	0.47	serpin peptidase inhibitor, clade B (ovalbumin), member 2	SERPINB2
1562012_at	0.47		
201520_s_at	0.47	G-rich RNA sequence binding factor 1	GRSF1
225468_at	0.47		
205466_s_at	0.47	heparan sulfate (glucosamine) 3-O-sulfotransferase 1	HS3ST1
216057_at	0.47	RAB3 GTPase activating protein subunit 2 (non-catalytic)	RAB3GAP2
222507_s_at	0.47	TMEM9 domain family, member B	TMEM9B
212514_x_at	0.47	DEAD (Asp-Glu-Ala-Asp) box polypeptide 3, X-linked	DDX3X
212681_at	0.47		
239006_at	0.47	solute carrier family 26, member 7	SLC26A7
209893_s_at	0.47	fucosyltransferase 4 (alpha (1,3) fucosyltransferase, myeloid-specific)	FUT4
228222_at	0.47	protein phosphatase 1, catalytic subunit, beta isoform	PPP1CB
211922_s_at	0.47	catalase	CAT
211404_s_at	0.47	amyloid beta (A4) precursor-like protein 2	APLP2
232079_s_at	0.46	poliovirus receptor-related 2 (herpesvirus entry mediator B)	PVRL2
222393_s_at	0.46	N-acetyltransferase 13	NAT13
208983_s_at	0.46	platelet/endothelial cell adhesion molecule (CD31 antigen)	PECAM1
241661_at	0.46	jumonji domain containing 1C	JMJD1C
202902_s_at	0.46	cathepsin S	CTSS
223860_at	0.46		
214575_s_at	0.46	azurocidin 1 (cationic antimicrobial protein 37)	AZU1
238539_at	0.46	Hermansky-Pudlak syndrome 3	HPS3
219312_s_at	0.46	zinc finger and BTB domain containing 10	ZBTB10
201162_at	0.46	insulin-like growth factor binding protein 7	IGFBP7
1568955_at	0.46	SLIT-ROBO Rho GTPase activating protein 2	SRGAP2
219969_at	0.46	chromosome X open reading frame 15	CXorf15
233591_at	0.46		
217019_at	0.46	ribosomal protein S4-like	RPS4L
1552660_a_at	0.46	chromosome 5 open reading frame 22	C5orf22
201324_at	0.46	epithelial membrane protein 1	EMP1
210350_x_at	0.46	inhibitor of growth family, member 1	ING1
218694_at	0.46	armadillo repeat containing, X-linked 1	ARMCX1
206113_s_at	0.46	RAB5A, member RAS oncogene family	RAB5A

224918_x_at	0.46	microsomal glutathione S-transferase 1	MGST1
1561215_at	0.46		
207431_s_at	0.46	degenerative spermatocyte homolog 1, lipid desaturase (Drosophila)	DEGS1
226136_at	0.46		
201538_s_at	0.46	dual specificity phosphatase 3 (vaccinia virus phosphatase VH1-related)	DUSP3
1555106_a_at	0.46	CTD (carboxy-terminal domain, RNA polymerase II, polypeptide A) small phosphatase like 2	CTDSPL2
225147_at	0.46	pleckstrin homology, Sec7 and coiled-coil domains 3	PSCD3
202823_at	0.46	transcription elongation factor B (SIII), polypeptide 1 (15kDa, elongin C)	TCEB1
229994_at	0.46		
206098_at	0.46	zinc finger and BTB domain containing 6	ZBTB6
234512_x_at	0.46		
232901_at	0.46	arginyl-tRNA synthetase-like	RARSL
200776_s_at	0.46		
228296_at	0.46	yippee-like 1 (Drosophila)	YPEL1
202437_s_at	0.46	cytochrome P450, family 1, subfamily B, polypeptide 1	CYP1B1
201942_s_at	0.46	carboxypeptidase D	CPD
230715_at	0.46		
239252_at	0.46		
241733_at	0.46	chromosome 18 open reading frame 54	C18orf54
1560028_at	0.46	chromosome 11 open reading frame 57	C11orf57
202143_s_at	0.45	COP9 constitutive photomorphogenic homolog subunit 8 (Arabidopsis)	COPS8
223551_at	0.45	protein kinase (cAMP-dependent, catalytic) inhibitor beta	PKIB
1565599_at	0.45		
205328_at	0.45	claudin 10	CLDN10
228204_at	0.45	proteasome (prosome, macropain) subunit, beta type, 4	PSMB4
1562984_at	0.45		
207426_s_at	0.45	tumor necrosis factor (ligand) superfamily, member 4 (tax-transcriptionally activated glycoprotein 1	TNFSF4
230422_at	0.45	formyl peptide receptor-like 2	FPRL2
219522_at	0.45	four jointed box 1 (Drosophila)	FJX1
214959_s_at	0.45	apoptosis inhibitor 5	API5
233849_s_at	0.45	Rho GTPase activating protein 5	ARHGAP5
1552312_a_at	0.45	microfibrillar-associated protein 3	MFAP3
1556285_s_at	0.45	pyrophosphatase (inorganic) 2	PPA2
203245_s_at	0.45		
239898_x_at	0.45	zinc finger protein 286A	ZNF286A
217245_at	0.45		
1568713_a_at	0.45	TBC1 (tre-2/USP6, BUB2, cdc16) domain family, member 1	TBC1D1
1555510_at	0.45	zinc finger protein 215	ZNF215
214513_s_at	0.45	cAMP responsive element binding protein 1	CREB1
217448_s_at	0.45		
1556609_at	0.45		
201226_at	0.45	NADH dehydrogenase (ubiquinone) 1 beta subcomplex, 8, 19kDa	NDUFB8

213887_s_at	0.45	polymerase (RNA) II (DNA directed) polypeptide E, 25kDa	POLR2E
207497_s_at	0.45	membrane-spanning 4-domains, subfamily A, member 2 (Fc fragment of IgE, high affinity I, receptor fo	MS4A2
202872_at	0.45	ATPase, H+ transporting, lysosomal 42kDa, V1 subunit C1	ATP6V1C1
222636_at	0.45	mediator of RNA polymerase II transcription, subunit 28 homolog (S. cerevisiae)	MED28
1554508_at	0.45	phosphoinositide-3-kinase adaptor protein 1	PIK3AP1
202912_at	0.45	adrenomedullin	ADM
206835_at	0.45	statherin	STATH
223328_at	0.45	armadillo repeat containing 10	ARMC10
213548_s_at	0.45	CDV3 homolog (mouse)	CDV3
217466_x_at	0.45		
231233_at	0.45		
204834_at	0.45	fibrinogen-like 2	FGL2
233097_x_at	0.45		
225065_x_at	0.45	chromosome 17 open reading frame 45	C17orf45
229787_s_at	0.45	O-linked N-acetylglucosamine (GlcNAc) transferase (UDP-N-acetylglucosamine:polypeptide-N-acetylgluco	OGT
1556058_s_at	0.45	spen homolog, transcriptional regulator (Drosophila)	SPEN
206369_s_at	0.44	phosphoinositide-3-kinase, catalytic, gamma polypeptide	PIK3CG
223751_x_at	0.44	toll-like receptor 10	TLR10
222731_at	0.44	zinc finger, DHHC-type containing 2	ZDHHC2
1558015_s_at	0.44	ARP2 actin-related protein 2 homolog (yeast)	ACTR2
236365_at	0.44	alpha-methylacyl-CoA racemase	AMACR
201946_s_at	0.44	chaperonin containing TCP1, subunit 2 (beta)	CCT2
219093_at	0.44	phosphotyrosine interaction domain containing 1	PID1
1562996_at	0.44		
1557486_at	0.44		
238816_at	0.44		
214203_s_at	0.44	proline dehydrogenase (oxidase) 1	PRODH
209034_at	0.44	proline-rich nuclear receptor coactivator 1	PNRC1
219947_at	0.44	C-type lectin domain family 4, member A	CLEC4A
233053_at	0.44		
224582_s_at	0.44		
238492_at	0.44		
224356_x_at	0.44	membrane-spanning 4-domains, subfamily A, member 6A	MS4A6A
214543_x_at	0.44	quaking homolog, KH domain RNA binding (mouse)	QKI
1555193_a_at	0.44	zinc finger protein 277 pseudogene	ZNF277P
231579_s_at	0.44	TIMP metallopeptidase inhibitor 2	TIMP2
231354_at	0.44		
211218_at	0.44		
223863_at	0.44		
200604_s_at	0.44	protein kinase, cAMP-dependent, regulatory, type I, alpha (tissue specific extinguisher 1)	PRKAR1A
244413_at	0.44		
1558937_s_at	0.44		
220494_s_at	0.44		
222504_s_at	0.44	COX4 neighbor	COX4NB
202877_s_at	0.44	CD93 molecule	CD93

243219_x_at	0.44		
237968_at	0.44	ADP-ribosylation factor-like 6 interacting protein 2	ARL6IP2
201151_s_at	0.44	muscleblind-like (Drosophila)	MBNL1
202978_s_at	0.44	CREB/ATF bZIP transcription factor	CREBZF
229905_at	0.44		
1570257_x_at	0.44		
235273_at	0.44	dyslexia susceptibility 1 candidate 1	DYX1C1
201971_s_at	0.44	ATPase, H+ transporting, lysosomal 70kDa, V1 subunit A	ATP6V1A
202257_s_at	0.44	CD2 (cytoplasmic tail) binding protein 2	CD2BP2
1557233_at	0.44		
231169_at	0.44		
1570253_a_at	0.44	Ras homolog enriched in brain like 1	RHEBL1
205638_at	0.44	brain-specific angiogenesis inhibitor 3	BAI3
201897_s_at	0.44	CDC28 protein kinase regulatory subunit 1B	CKS1B
217371_s_at	0.44	interleukin 15	IL15
1554462_a_at	0.44	DnaJ (Hsp40) homolog, subfamily B, member 9	DNAJB9
202691_at	0.44	small nuclear ribonucleoprotein D1 polypeptide 16kDa	SNRPD1
1568900_a_at	0.44	zinc finger protein 568	ZNF568
201619_at	0.44	peroxiredoxin 3	PRDX3
1553122_s_at	0.43	RB-associated KRAB zinc finger	RBAK
225422_at	0.43	cell division cycle 26 homolog (S. cerevisiae)	CDC26
212198_s_at	0.43	transmembrane 9 superfamily protein member 4	TM9SF4
203565_s_at	0.43	menage a trois homolog 1, cyclin H assembly factor (Xenopus laevis)	MNAT1
214698_at	0.43	ROD1 regulator of differentiation 1 (S. pombe)	ROD1
231094_s_at	0.43	methylenetetrahydrofolate dehydrogenase (NADP+ dependent) 1-like#pleckstrin homology domain containing, family G (with RhoGef domain) member 1	MTHFD1L#PLEKHG1
1555814_a_at	0.43	ras homolog gene family, member A	RHOA
239068_at	0.43		
1561609_at	0.43		
202968_s_at	0.43	dual-specificity tyrosine-(Y)-phosphorylation regulated kinase 2	DYRK2
211612_s_at	0.43	interleukin 13 receptor, alpha 1	IL13RA1
202533_s_at	0.43	dihydrofolate reductase	DHFR
217239_x_at	0.43		
37079_at	0.43		
200723_s_at	0.43	GPI-anchored membrane protein 1	GPIAP1
217915_s_at	0.43	chromosome 15 open reading frame 15	C15orf15
201742_x_at	0.43	splicing factor, arginine/serine-rich 1 (splicing factor 2, alternate splicing factor)	SFRS1
216871_at	0.43	zinc finger, DHHC-type containing 17	ZDHHC17
202444_s_at	0.43	ER lipid raft associated 1	ERLIN1
201669_s_at	0.43	myristoylated alanine-rich protein kinase C substrate	MARCKS
214341_at	0.43	adaptor-related protein complex 1, gamma 2 subunit	AP1G2
215646_s_at	0.43	chondroitin sulfate proteoglycan 2 (versican)	CSPG2
1553108_at	0.43	chromosome 5 open reading frame 24	C5orf24
202593_s_at	0.43		
1563840_at	0.43	elongation factor Tu GTP binding domain containing 1	EFTUD1
215286_s_at	0.43	putative homeodomain transcription factor 2	PHTF2
208520_at	0.43	olfactory receptor, family 10, subfamily H, member 3	OR10H3
217628_at	0.43	chloride intracellular channel 5	CLIC5

237061_at	0.42	zinc finger protein 347	ZNF347
1559069_at	0.42		
201325_s_at	0.42	epithelial membrane protein 1	EMP1
1556282_at	0.42	FGFR1 oncogene partner 2	FGFR1OP2
219076_s_at	0.42	peroxisomal membrane protein 2, 22kDa	PXMP2
222726_s_at	0.42	exocyst complex component 5	EXOC5
238010_at	0.42	chromosome 1 open reading frame 174	C1orf174
224311_s_at	0.42	calcium binding protein 39	CAB39
204221_x_at	0.42	GLI pathogenesis-related 1 (glioma)	GLIPR1
242727_at	0.42	ADP-ribosylation factor-like 5B	ARL5B
240327_at	0.42		
1569830_at	0.42	protein tyrosine phosphatase, receptor type, C	PTPRC
221786_at	0.42	chromosome 6 open reading frame 120	C6orf120
219326_s_at	0.42	UDP-GlcNAc:betaGal beta-1,3-N-acetylglucosaminyltransferase 2	B3GNT2
222725_s_at	0.42	palmdelphin	PALMD
220199_s_at	0.42	chromosome 1 open reading frame 80	C1orf80
238469_at	0.42		
236985_at	0.42		
1568836_at	0.42	CDC-like kinase 4	CLK4
216705_s_at	0.42	adenosine deaminase	ADA
1554577_a_at	0.42	proteasome (prosome, macropain) 26S subunit, non-ATPase, 10	PSMD10
212595_s_at	0.42	DAZ associated protein 2	DAZAP2
224470_at	0.42	SEC22 vesicle trafficking protein homolog C (S. cerevisiae)	SEC22C
201445_at	0.42	calponin 3, acidic	CNN3
1559374_at	0.42	ATPase, H+ transporting, lysosomal V0 subunit a1	ATP6V0A1
207686_s_at	0.42	caspase 8, apoptosis-related cysteine peptidase	CASP8
230265_at	0.42	sel-1 suppressor of lin-12-like (C. elegans)	SEL1L
212418_at	0.42	E74-like factor 1 (ets domain transcription factor)	ELF1
210933_s_at	0.42	fascin homolog 1, actin-bundling protein (Strongylocentrotus purpuratus)	FSCN1
1555728_a_at	0.42	membrane-spanning 4-domains, subfamily A, member 4	MS4A4A
214738_s_at	0.42	NIMA (never in mitosis gene a)- related kinase 9	NEK9
234607_at	0.42		
1558523_at	0.42	chromosome 6 open reading frame 60	C6orf60
235717_at	0.42	zinc finger protein 229	ZNF229
211016_x_at	0.42	heat shock 70kDa protein 4	HSPA4
228746_s_at	0.42		
1556413_a_at	0.42		
205023_at	0.42	RAD51 homolog (RecA homolog, E. coli) (S. cerevisiae)	RAD51
219837_s_at	0.42	cytokine-like 1	CYTL1
210935_s_at	0.42	WD repeat domain 1	WDR1
218778_x_at	0.42	EPS8-like 1	EPS8L1
211547_s_at	0.42	platelet-activating factor acetylhydrolase, isoform Ib, alpha subunit 45kDa	PAFAH1B1
1552481_s_at	0.42	mannosidase, alpha, class 1A, member 2	MAN1A2
1563531_at	0.42		
206978_at	0.42	chemokine (C-C motif) receptor 2	CCR2
220477_s_at	0.42	chromosome 20 open reading frame 30	C20orf30

1554482_a_at	0.42	SAR1 gene homolog B (S. cerevisiae)	SAR1B
239632_at	0.42		
229083_at	0.42		
218036_x_at	0.42	NMD3 homolog (S. cerevisiae)	NMD3
200737_at	0.42	phosphoglycerate kinase 1	PGK1
239080_at	0.42		
213856_at	0.41	CD47 molecule	CD47
1562457_at	0.41		
1561016_at	0.41		
202131_s_at	0.41	RIO kinase 3 (yeast)	RIOK3
222862_s_at	0.41	adenylate kinase 5	AK5
1554899_s_at	0.41	Fc fragment of IgE, high affinity I, receptor for; gamma polypeptide	FCER1G
243529_at	0.41	methionine-tRNA synthetase 2 (mitochondrial)	MARS2
213468_at	0.41	excision repair cross-complementing rodent repair deficiency, complementation group 2 (xeroderma pig	ERCC2
231093_at	0.41	Fc receptor-like 3	FCRL3
204374_s_at	0.41	galactokinase 1	GALK1
244186_at	0.41	chromosome 10 open reading frame 11	C10orf11
1555830_s_at	0.41	family with sequence similarity 62 (C2 domain containing) member B	FAM62B
219553_at	0.41	non-metastatic cells 7, protein expressed in (nucleoside-diphosphate kinase)	NME7
228977_at	0.41		
239170_at	0.41		
238790_at	0.41		
1554539_a_at	0.41	ras homolog gene family, member F (in filopodia)	RHOF
206631_at	0.41	prostaglandin E receptor 2 (subtype EP2), 53kDa	PTGER2
224560_at	0.41	TIMP metallopeptidase inhibitor 2	TIMP2
214539_at	0.41	serpin peptidase inhibitor, clade B (ovalbumin), member 10	SERPINB10
224798_s_at	0.41	chromosome 15 open reading frame 17	C15orf17
201120_s_at	0.41	progesterone receptor membrane component 1	PGRMC1
1570470_at	0.41	chromosome 14 open reading frame 161	C14orf161
244187_at	0.41		
212096_s_at	0.41	mitochondrial tumor suppressor 1	MTUS1
210074_at	0.41	cathepsin L2	CTSL2
214823_at	0.41	zinc finger protein 204	ZNF204
214945_at	0.41		
210982_s_at	0.41	major histocompatibility complex, class II, DR alpha	HLA-DRA
226142_at	0.41	GLI pathogenesis-related 1 (glioma)	GLIPR1
217125_at	0.40	ubiquitin B pseudogene 2	UBBP2
238493_at	0.40		
225647_s_at	0.40	cathepsin C	CTSC
228649_at	0.40	notum pectinacetylesterase homolog (Drosophila)	NOTUM
210801_at	0.40	DIM1 dimethyladenosine transferase 1-like (S. cerevisiae)	DIMT1L
205772_s_at	0.40	A kinase (PRKA) anchor protein 7	AKAP7
236350_at	0.40		
234993_at	0.40	abhydrolase domain containing 13	ABHD13
1567458_s_at	0.40	ras-related C3 botulinum toxin substrate 1 (rho family, small GTP binding protein Rac1)	RAC1
210663_s_at	0.40	kynureninase (L-kynurenine hydrolase)	KYNU

1554571_at	0.40	amyloid beta (A4) precursor protein-binding, family B, member 1 interacting protein	APBB1IP
244716_x_at	0.40	transmembrane and immunoglobulin domain containing 2	TMIGD2
1557910_at	0.40	heat shock protein 90kDa alpha (cytosolic), class B member 1	HSP90AB1
233856_at	0.40		
214336_s_at	0.40	coatomer protein complex, subunit alpha	COPA
213003_s_at	0.40	CCAAT/enhancer binding protein (C/EBP), delta	CEBPD
241636_x_at	0.40		
225661_at	0.40	interferon (alpha, beta and omega) receptor 1	IFNAR1
223758_s_at	0.40	general transcription factor IIH, polypeptide 2, 44kDa	GTF2H2
210154_at	0.40	malic enzyme 2, NAD(+)-dependent, mitochondrial	ME2
226341_at	0.40		
214658_at	0.40	toll-like receptor adaptor molecule 2	TICAM2
227198_at	0.40	AF4/FMR2 family, member 3	AFF3
215671_at	0.40	phosphodiesterase 4B, cAMP-specific (phosphodiesterase E4 dunce homolog, Drosophila)	PDE4B
237569_at	0.40		
205398_s_at	0.40	SMAD family member 3	SMAD3
1565563_at	0.40		
209269_s_at	0.40		
238448_at	0.40	mitochondrial ribosomal protein L19	MRPL19
202063_s_at	0.40	sel-1 suppressor of lin-12-like (C. elegans)	SEL1L
230951_at	0.40		
206667_s_at	0.40	secretory carrier membrane protein 1	SCAMP1
215250_at	0.40	transmembrane protein 111	TMEM111
213228_at	0.40	phosphodiesterase 8B	PDE8B
210748_at	0.40	DnaJ (Hsp40) homolog, subfamily C, member 8	DNAJC8
1555594_a_at	0.39	muscleblind-like (Drosophila)	MBNL1
218172_s_at	0.39	Der1-like domain family, member 1	DERL1
203248_at	0.39	zinc finger protein 24	ZNF24
209821_at	0.39	interleukin 33	IL33
1568822_at	0.39	GTP binding protein 5 (putative)	GTPBP5
211050_x_at	0.39		
218498_s_at	0.39	ERO1-like (S. cerevisiae)	ERO1L
230537_at	0.39		
1555326_a_at	0.39	ADAM metallopeptidase domain 9 (meltrin gamma)	ADAM9
244158_at	0.39		
237052_x_at	0.39	trinucleotide repeat containing 15	TNRC15
236932_s_at	0.39	GATA zinc finger domain containing 2A	GATAD2A
244653_at	0.39	SET domain containing (lysine methyltransferase) 7	SETD7
243809_at	0.39		
1553459_at	0.39		
1561523_at	0.39	coiled-coil domain containing 65	CCDC65
235268_at	0.39		
1559601_at	0.39	KIAA2018	KIAA2018
201653_at	0.39	cornichon homolog (Drosophila)	CNIH
225019_at	0.39	calcium/calmodulin-dependent protein kinase (CaM kinase) II delta	CAMK2D
215980_s_at	0.39	immunoglobulin mu binding protein 2	IGHMBP2
208351_s_at	0.39	mitogen-activated protein kinase 1	MAPK1
239354_at	0.39		

1554797_at	0.39	synaptotagmin XVI	SYT16
221650_s_at	0.39	mediator of RNA polymerase II transcription, subunit 18 homolog (S. cerevisiae)	MED18
236608_at	0.39	G protein-coupled receptor 113	GPR113
238561_s_at	0.39	chromosome 8 open reading frame 53	C8orf53
1555324_at	0.39	PTK7 protein tyrosine kinase 7	PTK7
215252_at	0.39		
220343_at	0.39	phosphodiesterase 7B	PDE7B
223423_at	0.39	G protein-coupled receptor 160	GPR160
1566764_at	0.39		
243299_at	0.39		
212560_at	0.38	sortilin-related receptor, L(DLR class) A repeats-containing	SORL1
231736_x_at	0.38	microsomal glutathione S-transferase 1	MGST1
211571_s_at	0.38	chondroitin sulfate proteoglycan 2 (versican)	CSPG2
206808_at	0.38	heterogeneous nuclear ribonucleoprotein A3 pseudogene 1	HNRPA3P1
237981_at	0.38	cardiomyopathy associated 5	CMYA5
243369_at	0.38	FRAS1 related extracellular matrix 1	FREM1
1557905_s_at	0.38	CD44 molecule (Indian blood group)	CD44
1559404_a_at	0.38		
243452_at	0.38		
228873_at	0.38	collagen, type XXII, alpha 1	COL22A1
227265_at	0.38	fibrinogen-like 2	FGL2
204128_s_at	0.38	replication factor C (activator 1) 3, 38kDa	RFC3
217445_s_at	0.38	phosphoribosylglycinamide formyltransferase, phosphoribosylglycinamide synthetase, phosphoribosylami	GART
215504_x_at	0.38		
210880_s_at	0.38	embryonal Fyn-associated substrate	EFS
1560758_at	0.38		
239760_at	0.38		
237305_at	0.38		
211812_s_at	0.38	beta-1,3-N-acetylgalactosaminyltransferase 1 (globoside blood group)	B3GALNT1
214971_s_at	0.38	ST6 beta-galactosamide alpha-2,6-sialyltranferase 1	ST6GAL1
231877_at	0.38	RNA (guanine-9-) methyltransferase domain containing 2	RG9MTD2
1554757_a_at	0.38	inositol polyphosphate-5-phosphatase, 40kDa	INPP5A
215397_x_at	0.38		
1553685_s_at	0.38	Sp1 transcription factor	SP1
207583_at	0.37	ATP-binding cassette, sub-family D (ALD), member 2	ABCD2
212598_at	0.37	WD repeat and FYVE domain containing 3	WDFY3
1555829_at	0.37	family with sequence similarity 62 (C2 domain containing) member B	FAM62B
1559052_s_at	0.37	p21 (CDKN1A)-activated kinase 2	PAK2
211776_s_at	0.37	erythrocyte membrane protein band 4.1-like 3	EPB41L3
205279_s_at	0.37	glycine receptor, beta	GLRB
201309_x_at	0.37	chromosome 5 open reading frame 13	C5orf13
237746_at	0.37	splicing factor, arginine/serine-rich 11	SFRS11
211061_s_at	0.37	mannosyl (alpha-1,6-)-glycoprotein beta-1,2-N-acetylglucosaminyltransferase	MGAT2
237233_at	0.37		

221695_s_at	0.37	mitogen-activated protein kinase kinase kinase 2	MAP3K2
238438_at	0.37	CCR4-NOT transcription complex, subunit 6-like	CNOT6L
238562_at	0.37	chromosome 8 open reading frame 53	C8orf53
1556236_at	0.37		
1564474_at	0.37		
218954_s_at	0.37	BRF2, subunit of RNA polymerase III transcription initiation factor, BRF1-like	BRF2
226220_at	0.37	methyltransferase like 9	METTL9
1555676_s_at	0.37	GS homeobox 1	GSH1
201554_x_at	0.37	glycogenin 1	GYG1
222922_at	0.37	potassium voltage-gated channel, Isk-related family, member 3	KCNE3
1564907_s_at	0.37	small nucleolar RNA host gene (non-protein coding) 4	SNHG4
205048_s_at	0.37	phosphoserine phosphatase	PSPH
243432_at	0.37		
242702_at	0.37	methylmalonic aciduria (cobalamin deficiency) cblA type	MMAA
211450_s_at	0.37	mutS homolog 6 (E. coli)	MSH6
1555639_a_at	0.37	RNA binding motif protein 14	RBM14
216467_s_at	0.37		
236596_at	0.37		
239468_at	0.37	mohawk homeobox	MKX
209406_at	0.36	BCL2-associated athanogene 2	BAG2
207794_at	0.36	chemokine (C-C motif) receptor 2	CCR2
236035_at	0.36		
203535_at	0.36	S100 calcium binding protein A9	S100A9
210176_at	0.36	toll-like receptor 1	TLR1
231816_s_at	0.36		
241868_at	0.36		
225580_at	0.36	mitochondrial ribosomal protein L50	MRPL50
205867_at	0.36	protein tyrosine phosphatase, non-receptor type 11 (Noonan syndrome 1)	PTPN11
204472_at	0.36	GTP binding protein overexpressed in skeletal muscle	GEM
216015_s_at	0.36	NLR family, pyrin domain containing 3	NLRP3
215602_at	0.36	FYVE, RhoGEF and PH domain containing 2	FGD2
241542_at	0.36	SRY (sex determining region Y)-box 6	SOX6
232661_s_at	0.36		
219797_at	0.36	mannosyl (alpha-1,3-)-glycoprotein beta-1,4-N-acetylglucosaminyltransferase, isozyme A	MGAT4A
205841_at	0.36	Janus kinase 2 (a protein tyrosine kinase)	JAK2
216428_x_at	0.36	killer cell immunoglobulin-like receptor, three domains, X1	KIR3DX1
234361_at	0.36	sirtuin (silent mating type information regulation 2 homolog) 6 (S. cerevisiae)#cAMP responsive element binding protein 3-like 3	SIRT6#CREB3L3
241444_at	0.36		
229045_at	0.36		
222559_s_at	0.36		
222773_s_at	0.36	UDP-N-acetyl-alpha-D-galactosamine:polypeptide N-acetylgalactosaminyltransferase 12 (GalNAc-T12)	GALNT12
1554768_a_at	0.36	MAD2 mitotic arrest deficient-like 1 (yeast)	MAD2L1
218748_s_at	0.36	exocyst complex component 5	EXOC5
1555780_a_at	0.36	Ras homolog enriched in brain	RHEB

216317_x_at	0.36	Rh blood group, CcEe antigens	RHCE
211190_x_at	0.36	CD84 molecule	CD84
1556123_a_at	0.36		
210433_at	0.36	protein O-fucosyltransferase 1	POFUT1
238705_at	0.36		
1562387_at	0.36		
227839_at	0.36	methyl-CpG binding domain protein 5	MBD5
1561292_at	0.36		
1554341_a_at	0.36		
228634_s_at	0.36	cold shock domain protein A	CSDA
224899_s_at	0.36		
216653_at	0.36	down-regulator of transcription 1, TBP-binding (negative cofactor 2)	DR1
225070_at	0.36	nuclear undecaprenyl pyrophosphate synthase 1 homolog (S. cerevisiae)	NUS1
224387_at	0.36	COMM domain containing 5	COMMD5
211189_x_at	0.36	CD84 molecule	CD84
1560581_at	0.36		
237327_at	0.35		
204196_x_at	0.35	PBX/knotted 1 homeobox 1	PKNOX1
241069_at	0.35	zinc finger and BTB domain containing 16	ZBTB16
239494_at	0.35		
1554242_a_at	0.35	coagulation factor C homolog, cochlin (Limulus polyphemus)	COCH
230907_at	0.35	G protein-coupled receptor, family C, group 5, member C	GPRC5C
202226_s_at	0.35	v-crk sarcoma virus CT10 oncogene homolog (avian)	CRK
1569539_at	0.35		
206902_s_at	0.35	endonuclease G-like 1	ENDOGL1
1568647_at	0.35		
242755_at	0.35	SFRS protein kinase 2	SRPK2
1565795_at	0.35	dual oxidase 1	DUOX1
224407_s_at	0.35		
207099_s_at	0.35	choroideremia (Rab escort protein 1)	CHM
229713_at	0.35		
209638_x_at	0.35	regulator of G-protein signalling 12	RGS12
244390_at	0.35		
205230_at	0.35	rabphilin 3A homolog (mouse)	RPH3A
241611_s_at	0.35	fibronectin type III domain containing 3A	FNDC3A
242179_s_at	0.35	tetraspanin 16	TSPAN16
1555463_a_at	0.35	chromodomain helicase DNA binding protein 6	CHD6
1558440_at	0.35		
241774_at	0.35		
1555702_a_at	0.35	ST3 beta-galactoside alpha-2,3-sialyltransferase 3	ST3GAL3
1570070_at	0.35	chromosome 20 open reading frame 160	C20orf160
1554923_at	0.34	ankyrin repeat and sterile alpha motif domain containing 6	ANKS6
1561926_at	0.34		
233120_at	0.34		
228485_s_at	0.34	solute carrier family 44, member 1	SLC44A1
222847_s_at	0.34	egl nine homolog 3 (C. elegans)	EGLN3
1554800_at	0.34	RAB39, member RAS oncogene family	RAB39
210788_s_at	0.34	dehydrogenase/reductase (SDR family) member 7	DHRS7

1552798_a_at	0.34	toll-like receptor 4	TLR4
205419_at	0.34	Epstein-Barr virus induced gene 2 (lymphocyte-specific G protein-coupled receptor)	EBI2
1562353_x_at	0.34		
236180_at	0.34		
239477_at	0.34	EF-hand domain family, member B	EFHB
237406_at	0.34		
214505_s_at	0.34	four and a half LIM domains 1	FHL1
1555097_a_at	0.34	prostaglandin F receptor (FP)	PTGFR
214340_at	0.34	arachidonate 12-lipoxygenase pseudogene 2	ALOX12P2
222071_s_at	0.34	solute carrier organic anion transporter family, member 4C1	SLCO4C1
242830_at	0.34		
238834_at	0.34		
219014_at	0.34	placenta-specific 8	PLAC8
1563524_a_at	0.34	chromosome 14 open reading frame 85	C14orf85
208463_at	0.34	gamma-aminobutyric acid (GABA) A receptor, alpha 4	GABRA4
239806_at	0.34		
213721_at	0.34	SRY (sex determining region Y)-box 2	SOX2
241266_at	0.34		
240298_at	0.34		
223341_s_at	0.33	short coiled-coil protein	SCOC
1565162_s_at	0.33	microsomal glutathione S-transferase 1	MGST1
238779_at	0.33	DCP2 decapping enzyme homolog (S. cerevisiae)	DCP2
232894_at	0.33	SEC14-like 2 (S. cerevisiae)	SEC14L2
1554424_at	0.33	FIP1 like 1 (S. cerevisiae)	FIP1L1
215459_at	0.33	Tax1 (human T-cell leukemia virus type I) binding protein 3	TAX1BP3
1560460_at	0.33		
223501_at	0.33		
201796_s_at	0.33	valyl-tRNA synthetase	VARS
1569905_at	0.33	hydroxysteroid (11-beta) dehydrogenase 1-like	HSD11B1L
241328_at	0.33	zinc finger, matrin type 1	ZMAT1
224148_at	0.33	FYN binding protein (FYB-120/130)	FYB
221111_at	0.33	interleukin 26	IL26
223732_at	0.33	solute carrier family 23 (nucleobase transporters), member 1	SLC23A1
225987_at	0.33	STEAP family member 4	STEAP4
1554679_a_at	0.33	lysosomal associated protein transmembrane 4 beta	LAPTM4B
234651_at	0.33		
1558595_at	0.33		
205224_at	0.33	surfeit 2	SURF2
222558_at	0.33		
238795_at	0.33	chromosome 10 open reading frame 18	C10orf18
231576_at	0.33		
1557167_at	0.32	HLA complex group 11	HCG11
1564075_a_at	0.32		
219872_at	0.32	chromosome 4 open reading frame 18	C4orf18
240587_x_at	0.32		
1565633_at	0.32		
208143_s_at	0.32	family with sequence similarity 12, member A	FAM12A
217356_s_at	0.32	phosphoglycerate kinase 1	PGK1
1563165_at	0.32		

1565879_at	0.32	solute carrier family 5 (sodium/glucose cotransporter), member 11	SLC5A11
242019_at	0.32	LAG1 homolog, ceramide synthase 6 (S. cerevisiae)	LASS6
200769_s_at	0.32	methionine adenosyltransferase II, alpha	MAT2A
203645_s_at	0.32	CD163 molecule	CD163
1556935_at	0.32		
203041_s_at	0.32	lysosomal-associated membrane protein 2	LAMP2
1555202_a_at	0.32		
240785_at	0.32		
1570176_at	0.32		
202638_s_at	0.32	intercellular adhesion molecule 1 (CD54), human rhinovirus receptor	ICAM1
1553562_at	0.32	CD8b molecule	CD8B
235899_at	0.32	carbonic anhydrase XIII	CA13
1559203_s_at	0.31	v-Ki-ras2 Kirsten rat sarcoma viral oncogene homolog	KRAS
236491_at	0.31	BCL2-like 10 (apoptosis facilitator)	BCL2L10
224105_x_at	0.31		
212592_at	0.31		
236565_s_at	0.31	La ribonucleoprotein domain family, member 6	LARP6
218584_at	0.31		
219927_at	0.31	FCF1 small subunit (SSU) processome component homolog (S. cerevisiae)	FCF1
214300_s_at	0.31	topoisomerase (DNA) III alpha	TOP3A
233878_s_at	0.31	5'-3' exoribonuclease 2	XRN2
229128_s_at	0.31	acidic (leucine-rich) nuclear phosphoprotein 32 family, member E	ANP32E
229327_s_at	0.31	v-maf musculoaponeurotic fibrosarcoma oncogene homolog (avian)	MAF
234360_at	0.31	family with sequence similarity 120C#WNK lysine deficient protein kinase 3	FAM120C#W NK3
206331_at	0.31	calcitonin receptor-like	CALCRL
237762_at	0.31	Rh-associated glycoprotein	RHAG
235202_x_at	0.31		
231217_at	0.31		
210872_x_at	0.31	growth arrest-specific 7	GAS7
202834_at	0.31	angiotensinogen (serpin peptidase inhibitor, clade A, member 8)	AGT
214669_x_at	0.31	major histocompatibility complex, class I, C	HLA-C
218368_s_at	0.31	tumor necrosis factor receptor superfamily, member 12A	TNFRSF12A
217294_s_at	0.31	enolase 1, (alpha)	ENO1
1561770_at	0.31		
243619_at	0.31	FGFR1 oncogene partner 2	FGFR1OP2
215619_at	0.31		
237508_at	0.31	cytoplasmic polyadenylation element binding protein 3	CPEB3
238486_at	0.31	fibroblast growth factor receptor substrate 2	FRS2
203004_s_at	0.31	MADS box transcription enhancer factor 2, polypeptide D (myocyte enhancer factor 2D)	MEF2D
211227_s_at	0.31	protocadherin 11 Y-linked	PCDH11Y
230220_at	0.31		
1558397_at	0.31		
233149_at	0.31		
242979_at	0.31		

215446_s_at	0.31	lysyl oxidase	LOX
243495_s_at	0.31		
210291_s_at	0.31	zinc finger protein 174	ZNF174
202586_at	0.31	polymerase (RNA) II (DNA directed) polypeptide L, 7.6kDa	POLR2L
226360_at	0.30	zinc and ring finger 3	ZNRF3
235447_at	0.30	TruB pseudouridine (psi) synthase homolog 1 (E. coli)	TRUB1
214277_at	0.30	COX11 homolog, cytochrome c oxidase assembly protein (yeast)	COX11
222915_s_at	0.30	B-cell scaffold protein with ankyrin repeats 1	BANK1
208894_at	0.30	major histocompatibility complex, class II, DR alpha	HLA-DRA
218790_s_at	0.30	trimethyllysine hydroxylase, epsilon	TMLHE
214588_s_at	0.30	UDP-N-acetyl-alpha-D-galactosamine:polypeptide N-acetylgalactosaminyltransferase 10 (GalNAc-T10)	GALNT10
208608_s_at	0.30	syntrophin, beta 1 (dystrophin-associated protein A1, 59kDa, basic component 1)	SNTB1
1563536_at	0.30		
210299_s_at	0.30	four and a half LIM domains 1	FHL1
1553266_at	0.30	CCR4-NOT transcription complex, subunit 6-like	CNOT6L
232751_at	0.30	Sec23 homolog B (S. cerevisiae)#polymerase (RNA) III (DNA directed) polypeptide F, 39 kDa#retinoblastoma binding protein 9#chromosome 20 open reading frame 12#ribosomal protein S19 pseudogene 1#ribosomal protein L21 pseudogene 3#glucosaminyl (N-acetyl) transferase 1, core 2 (beta-1,6-N-acetylglucosaminyltransferase) pseudogene	SEC23B#POLR3F#RBBP9#C20orf12#RPS19P1#RPL21P3#GCNT1P
209387_s_at	0.30	transmembrane 4 L six family member 1	TM4SF1
215787_at	0.30	actin, alpha 2, smooth muscle, aorta	ACTA2
210166_at	0.30	toll-like receptor 5	TLR5
202917_s_at	0.30	S100 calcium binding protein A8	S100A8
244007_at	0.30		
243727_at	0.30	copine VIII	CPNE8
233421_s_at	0.30	nucleoporin 133kDa	NUP133
226079_at	0.30		
227055_at	0.30	methyltransferase like 7B	METTL7B
243487_at	0.30	AF4/FMR2 family, member 4	AFF4
203072_at	0.30	myosin IE	MYO1E
228876_at	0.29	BAI1-associated protein 2-like 2	BAIAP2L2
216591_s_at	0.29	succinate dehydrogenase complex, subunit C, integral membrane protein, 15kDa	SDHC
230254_at	0.29		
1558428_at	0.29	amyotrophic lateral sclerosis 2 (juvenile) chromosome region, candidate 11	ALS2CR11
240332_at	0.29		
214037_s_at	0.29	coiled-coil domain containing 22	CCDC22
1563135_at	0.29		
209683_at	0.29	family with sequence similarity 49, member A	FAM49A
205188_s_at	0.29	SMAD family member 5	SMAD5
37547_at	0.29	Bardet-Biedl syndrome 9	BBS9
241345_at	0.29		
230365_at	0.29	lin-9 homolog (C. elegans)	LIN9
243858_at	0.29	steroid sulfatase (microsomal), arylsulfatase C, isozyme S	STS

220550_at	0.29	F-box protein 4	FBXO4
244584_at	0.29	chromosome 11 open reading frame 41	C11orf41
231152_at	0.29		
239160_at	0.29		
1567682_x_at	0.29	small nucleolar RNA, H/ACA box 74A	SNORA74A
230432_at	0.29		
216580_at	0.29		
232049_at	0.29		
239675_at	0.29		
203888_at	0.28	thrombomodulin	THBD
235395_at	0.28	SEC63 homolog (S. cerevisiae)	SEC63
224397_s_at	0.28	transmembrane and tetratricopeptide repeat containing 1	TMTC1
233791_at	0.28		
213872_at	0.28	chromosome 6 open reading frame 62	C6orf62
206522_at	0.28	maltase-glucoamylase (alpha-glucosidase)	MGAM
1559441_s_at	0.28		
1569624_at	0.28		
1555167_s_at	0.28	pre-B-cell colony enhancing factor 1	PBEF1
220541_at	0.28	matrix metallopeptidase 26	MMP26
238618_at	0.28	neurofibromin 2 (bilateral acoustic neuroma)	NF2
231382_at	0.28	fibroblast growth factor 18	FGF18
220051_at	0.28	protease, serine, 21 (testisin)	PRSS21
1566991_at	0.28	AT rich interactive domain 1B (SWI1-like)	ARID1B
203771_s_at	0.28	biliverdin reductase A	BLVRA
239380_at	0.28	chromosome 5 open reading frame 27	C5orf27
233023_at	0.28	armadillo repeat containing 10	ARMC10
236372_at	0.28		
234547_at	0.28		
209645_s_at	0.28	aldehyde dehydrogenase 1 family, member B1	ALDH1B1
1564688_a_at	0.28	RAD9 homolog B (S. cerevisiae)	RAD9B
1564158_a_at	0.27		
243789_at	0.27		
1555972_s_at	0.27	F-box protein 28	FBXO28
244041_at	0.27		
220324_at	0.27	chromosome 6 open reading frame 155	C6orf155
214942_at	0.27	RNA binding motif protein 34	RBM34
236345_at	0.27		
215672_s_at	0.27		
201539_s_at	0.27	four and a half LIM domains 1	FHL1
228855_at	0.27	nudix (nucleoside diphosphate linked moiety X)-type motif 7	NUDT7
241998_at	0.27		
240818_at	0.27		
216677_at	0.27	zinc finger protein 154	ZNF154
244719_at	0.27	NTF2-like export factor 1	NXT1
1556325_at	0.27	filamin A interacting protein 1	FILIP1
1555319_at	0.27	stabilin 1	STAB1
211363_s_at	0.27	methylthioadenosine phosphorylase	MTAP
1562222_at	0.27		
244009_at	0.26	calcium modulating ligand	CAMLG

210930_s_at	0.26	v-erb-b2 erythroblastic leukemia viral oncogene homolog 2, neuro/glioblastoma derived oncogene homol	ERBB2
230659_at	0.26		
207174_at	0.26	glypican 5	GPC5
244259_s_at	0.26		
240107_at	0.26		
206615_s_at	0.26	ADAM metallopeptidase domain 22	ADAM22
206553_at	0.26	2'-5'-oligoadenylate synthetase 2, 69/71kDa	OAS2
1553267_a_at	0.26	CCR4-NOT transcription complex, subunit 6-like	CNOT6L
219338_s_at	0.26	leucine rich repeat containing 49	LRRC49
214579_at	0.26	NIPA-like domain containing 3	NPAL3
1560520_at	0.26		
236138_at	0.26		
231167_at	0.26		
238443_at	0.26		
205388_at	0.26	troponin C type 2 (fast)	TNNC2
206512_at	0.26	zinc finger (CCCH type), RNA-binding motif and serine/arginine rich 1	ZRSR1
231353_at	0.26		
226719_at	0.26		
231041_at	0.26	polymerase (RNA) I polypeptide E, 53kDa	POLR1E
217369_at	0.26	immunoglobulin heavy constant gamma 1 (G1m marker)	IGHG1
238552_at	0.26	SFRS protein kinase 2	SRPK2
216290_x_at	0.26		
1565920_at	0.25		
1565840_at	0.25	PR domain containing 4	PRDM4
240730_at	0.25		
208075_s_at	0.25	chemokine (C-C motif) ligand 7	CCL7
208140_s_at	0.25	leucine rich repeat containing 48	LRRC48
235163_at	0.25	MOB1, Mps One Binder kinase activator-like 2A (yeast)	MOBKL2A
1555733_s_at	0.25	adaptor-related protein complex 1, sigma 3 subunit	AP1S3
219864_s_at	0.25	Down syndrome critical region gene 1-like 2	DSCR1L2
223938_at	0.25	chromosome 1 open reading frame 49	C1orf49
211087_x_at	0.25	mitogen-activated protein kinase 14	MAPK14
1557169_x_at	0.25	HLA complex group 11	HCG11
206710_s_at	0.25	erythrocyte membrane protein band 4.1-like 3	EPB41L3
220593_s_at	0.25	coiled-coil domain containing 40	CCDC40
215523_at	0.25	zinc finger protein 184#zinc finger protein 204#null	ZNF184#ZNF204#null
236586_at	0.25		
240405_at	0.24		
243062_at	0.24	folliculin	FLCN
1552381_at	0.24		
218824_at	0.24		
227460_at	0.24	chromosome 3 open reading frame 63	C3orf63
203101_s_at	0.24		
238461_at	0.24	eukaryotic translation initiation factor 4E family member 3	EIF4E3
227401_at	0.24	interleukin 17D	IL17D
223143_s_at	0.24	chromosome 6 open reading frame 166	C6orf166

242850_at	0.24		
1565495_at	0.24		
207651_at	0.24	G protein-coupled receptor 171	GPR171
210869_s_at	0.24	melanoma cell adhesion molecule	MCAM
231934_at	0.24	N-acetylneuraminic acid synthase (sialic acid synthase)	NANS
242288_s_at	0.24	elastin microfibril interfacer 2	EMILIN2
214975_s_at	0.24	myotubularin related protein 1	MTMR1
220817_at	0.24	transient receptor potential cation channel, subfamily C, member 4	TRPC4
211631_x_at	0.24	UDP-Gal:betaGlcNAc beta 1,4- galactosyltransferase, polypeptide 1	B4GALT1
207167_at	0.24	immunoglobulin superfamily, member 2	IGSF2
244407_at	0.24	cytochrome P450, family 39, subfamily A, polypeptide 1	CYP39A1
234205_at	0.23		
205712_at	0.23	protein tyrosine phosphatase, receptor type, D	PTPRD
244416_at	0.23	tetratricopeptide repeat domain 17	TTC17
233011_at	0.23	annexin A1	ANXA1
1562642_at	0.23		
203032_s_at	0.23	fumarate hydratase	FH
231842_at	0.23	KIAA1462	KIAA1462
226830_x_at	0.23	chromodomain helicase DNA binding protein 2	CHD2
205573_s_at	0.23	sorting nexin 7	SNX7
201741_x_at	0.23	splicing factor, arginine/serine-rich 1 (splicing factor 2, alternate splicing factor)	SFRS1
1555358_a_at	0.23	ectonucleoside triphosphate diphosphohydrolase 4	ENTPD4
1555434_a_at	0.23	solute carrier family 39 (zinc transporter), member 14	SLC39A14
1558463_s_at	0.23		
205095_s_at	0.23	ATPase, H+ transporting, lysosomal V0 subunit a1	ATP6V0A1
1566632_at	0.23		
225165_at	0.23	protein phosphatase 1, regulatory (inhibitor) subunit 1B (dopamine and cAMP regulated phosphoprotein	PPP1R1B
243577_at	0.22		
234500_at	0.22		
209504_s_at	0.22	pleckstrin homology domain containing, family B (evectins) member 1	PLEKHB1
203895_at	0.22	phospholipase C, beta 4	PLCB4
234136_at	0.22		
205320_at	0.22	adenomatosis polyposis coli 2	APC2
225408_at	0.22	myelin basic protein	MBP
242246_x_at	0.22		
210225_x_at	0.22	leukocyte immunoglobulin-like receptor, subfamily B (with TM and ITIM domains), member 3	LILRB3
202668_at	0.22	ephrin-B2	EFNB2
240551_at	0.22	zinc finger and BTB domain containing 45	ZBTB45
1568673_s_at	0.22	ELL associated factor 2	EAF2
1558867_at	0.22	dermatan sulfate epimerase	DSE
209170_s_at	0.21	glycoprotein M6B	GPM6B
204955_at	0.21	sushi-repeat-containing protein, X-linked	SRPX
1554612_at	0.21	KIAA0226	KIAA0226
243125_x_at	0.21		
1555745_a_at	0.21	lysozyme (renal amyloidosis)	LYZ

210298_x_at	0.21	four and a half LIM domains 1	FHL1
241682_at	0.21	kelch-like 23 (Drosophila)	KLHL23
1559640_at	0.21	ankyrin-repeat and fibronectin type III domain containing 1	ANKFN1
228461_at	0.21		
242474_s_at	0.21		
234011_at	0.21		
1553605_a_at	0.21	ATP-binding cassette, sub-family A (ABC1), member 13	ABCA13
1554327_a_at	0.21	calcium activated nucleotidase 1	CANT1
1556266_a_at	0.21		
220931_at	0.21		
214606_at	0.21	tetraspanin 2	TSPAN2
1563022_at	0.21		
241340_at	0.21		
232571_at	0.20		
215784_at	0.20	CD1e molecule	CD1E
238391_at	0.20	SMAD specific E3 ubiquitin protein ligase 2	SMURF2
1552987_a_at	0.20		
241174_at	0.20	adaptor-related protein complex 4, epsilon 1 subunit	AP4E1
237396_at	0.20		
211140_s_at	0.20	caspase 2, apoptosis-related cysteine peptidase (neural precursor cell expressed, developmentally do	CASP2
1553127_a_at	0.20	ring finger protein 168	RNF168
205071_x_at	0.20	X-ray repair complementing defective repair in Chinese hamster cells 4	XRCC4
1556492_a_at	0.20		
244030_at	0.20	serine/threonine/tyrosine interacting protein	STYX
240836_at	0.20	ribosomal protein L27	RPL27
212292_at	0.20	solute carrier family 7 (cationic amino acid transporter, y+ system), member 1	SLC7A1
227846_at	0.20	G protein-coupled receptor 176	GPR176
205116_at	0.20	laminin, alpha 2 (merosin, congenital muscular dystrophy)	LAMA2
1559530_at	0.19		
222295_x_at	0.19		
1555330_at	0.19	glutamate-cysteine ligase, catalytic subunit	GCLC
223891_at	0.19	potassium voltage-gated channel, KQT-like subfamily, member 5	KCNQ5
209189_at	0.19	v-fos FBJ murine osteosarcoma viral oncogene homolog	FOS
224080_at	0.19		
230465_at	0.19	heparan sulfate 2-O-sulfotransferase 1	HS2ST1
234126_at	0.19		
244839_at	0.18	titin	TTN
232971_at	0.18	RNA binding motif protein 15	RBM15
1562823_at	0.18		
214648_at	0.18		
1553798_a_at	0.18	F-box and leucine-rich repeat protein 13	FBXL13
206435_at	0.18	beta-1,4-N-acetyl-galactosaminyl transferase 1	B4GALNT1
237531_at	0.18	chromosome 2 open reading frame 3	C2orf3
232886_at	0.18		
1554468_s_at	0.18	mitochondrial ribosomal protein L38	MRPL38

229912_at	0.18	sidekick homolog 1 (chicken)	SDK1
238605_at	0.18		
1559986_at	0.18		
236882_at	0.18		
213747_at	0.18	antizyme inhibitor 1	AZIN1
242426_at	0.17	neuregulin 4	NRG4
237578_at	0.17		
213481_at	0.17	chromosome 1 open reading frame 77	C1orf77
214597_at	0.17	somatostatin receptor 2	SSTR2
225803_at	0.17	F-box protein 32	FBXO32
224225_s_at	0.17	ets variant gene 7 (TEL2 oncogene)	ETV7
214732_at	0.17	Sp1 transcription factor	SP1
223750_s_at	0.17	toll-like receptor 10	TLR10
221618_s_at	0.17	TAF9B RNA polymerase II, TATA box binding protein (TBP)-associated factor, 31kDa	TAF9B
235166_at	0.17		
206481_s_at	0.17	LIM domain binding 2	LDB2
244436_at	0.16		
207932_at	0.16	interferon, alpha 8	IFNA8
202005_at	0.16	suppression of tumorigenicity 14 (colon carcinoma)	ST14
234616_at	0.16		
1557257_at	0.16	B-cell CLL/lymphoma 10	BCL10
244171_at	0.16	muskelin 1, intracellular mediator containing kelch motifs	MKLN1
214105_at	0.16	suppressor of cytokine signaling 3	SOCS3
1554417_s_at	0.16	anterior pharynx defective 1 homolog A (C. elegans)	APH1A
223765_s_at	0.16	kelch repeat and BTB (POZ) domain containing 4	KBTBD4
229493_at	0.16		
1555018_at	0.16	olfactory receptor, family 2, subfamily C, member 3	OR2C3
241115_at	0.16	KIAA1467	KIAA1467
239364_at	0.16	ets variant gene 6 (TEL oncogene)	ETV6
200986_at	0.16	serpin peptidase inhibitor, clade G (C1 inhibitor), member 1, (angioedema, hereditary)	SERPING1
1564072_at	0.16	myosin, heavy chain 16	MYH16
238257_at	0.16	myeloid/lymphoid or mixed-lineage leukemia (trithorax homolog, Drosophila); translocated to, 10	MLLT10
241245_at	0.16	splicing factor, arginine/serine-rich 4	SFRS4
226717_at	0.16	leucine rich repeat containing 51	LRRC51
206911_at	0.16	tripartite motif-containing 25	TRIM25
231628_s_at	0.16		
227648_at	0.16	chromosome 22 open reading frame 32	C22orf32
239272_at	0.15	matrix metallopeptidase 28	MMP28
236549_x_at	0.15		
1556513_at	0.15	zinc finger protein 573	ZNF573
201467_s_at	0.15	NAD(P)H dehydrogenase, quinone 1	NQO1
216493_s_at	0.15	ribosomal protein S6 kinase, 90kDa, polypeptide 2	RPS6KA2
237602_at	0.15		
243294_at	0.15	zinc finger protein 780B	ZNF780B
212667_at	0.15	secreted protein, acidic, cysteine-rich (osteonectin)	SPARC
234904_x_at	0.15	ELAV (embryonic lethal, abnormal vision, Drosophila)-like 4 (Hu antigen D)	ELAVL4
1552367_a_at	0.15	scinderin	SCIN
233547_x_at	0.15	phosphodiesterase 1A, calmodulin-dependent	PDE1A

206841_at	0.15	phosphodiesterase 6H, cGMP-specific, cone, gamma	PDE6H
242331_x_at	0.15		
1564962_at	0.15	zinc finger protein 92	ZNF92
221724_s_at	0.14	C-type lectin domain family 4, member A	CLEC4A
236244_at	0.14	heterogeneous nuclear ribonucleoprotein U (scaffold attachment factor A)	HNRPU
213562_s_at	0.14	squalene epoxidase	SQLE
219026_s_at	0.14	RAS protein activator like 2	RASAL2
1561347_a_at	0.13		
236543_at	0.13		
229802_at	0.13		
202434_s_at	0.13	cytochrome P450, family 1, subfamily B, polypeptide 1	CYP1B1
210193_at	0.13	myelin-associated oligodendrocyte basic protein	MOBP
229331_at	0.13	spermatogenesis associated 18 homolog (rat)	SPATA18
239072_at	0.13		
216758_at	0.13		
1559606_at	0.13	guanylate binding protein family, member 6	GBP6
210690_at	0.13	killer cell lectin-like receptor subfamily C, member 4	KLRC4
203783_x_at	0.13	polymerase (RNA) mitochondrial (DNA directed)	POLRMT
217559_at	0.13	ribosomal protein L10-like	RPL10L
206091_at	0.13	matrilin 3	MATN3
217489_s_at	0.13	interleukin 6 receptor	IL6R
206292_s_at	0.12	sulfotransferase family, cytosolic, 2A, dehydroepiandrosterone (DHEA)-preferring, member 1	SULT2A1
241066_at	0.12	zinc finger protein 449	ZNF449
230834_at	0.12		
205719_s_at	0.12	phenylalanine hydroxylase	PAH
225946_at	0.12	Ras association (RalGDS/AF-6) domain family 8	RASSF8
238717_at	0.12		
239818_x_at	0.12	tribbles homolog 1 (Drosophila)	TRIB1
1554777_at	0.12	zinc finger protein 42 homolog (mouse)	ZFP42
216931_at	0.12		
239271_at	0.12	SMAD family member 2	SMAD2
242604_at	0.11		
233465_at	0.11		
205808_at	0.11	aspartate beta-hydroxylase	ASPH
1554793_at	0.11	ubiquitin protein ligase E3C	UBE3C
215219_at	0.11	dopey family member 2	DOPEY2
233697_at	0.11		
200796_s_at	0.11	myeloid cell leukemia sequence 1 (BCL2-related)	MCL1
224410_s_at	0.11	limb region 1 homolog (mouse)	LMBR1
240161_s_at	0.11	cell division cycle 20 homolog B (S. cerevisiae)	CDC20B
212806_at	0.11		
235118_at	0.11		
1553229_at	0.10	zinc finger protein 572	ZNF572
236459_at	0.10	protein kinase C, epsilon	PRKCE
1552389_at	0.10	chromosome 8 open reading frame 47	C8orf47
227618_at	0.10		
227868_at	0.10		
221352_at	0.10		
1570585_at	0.10		
242758_x_at	0.09		

215172_at	0.09	protein tyrosine phosphatase, non-receptor type 20B#protein tyrosine phosphatase, non-receptor type 20A#protein tyrosine phosphatase, non-receptor type 20A	PTPN20B#PTPN20A#PTPN20A
224134_at	0.08		
243974_at	0.08		
1555340_x_at	0.08	RAP1A, member of RAS oncogene family	RAP1A
1562301_at	0.08	chromosome 8 open reading frame 34	C8orf34
1555339_at	0.08	RAP1A, member of RAS oncogene family	RAP1A
231064_s_at	0.08	nucleoporin 50kDa	NUP50
1569755_at	0.08		
1558177_at	0.08	chromosome 14 open reading frame 83	C14orf83
1556873_at	0.07	ring finger and KH domain containing 2	RKHD2
210170_at	0.06	PDZ and LIM domain 3	PDLIM3
227148_at	0.06	pleckstrin homology domain containing, family H (with MyTH4 domain) member 2	PLEKHH2
235851_s_at	0.06		
216660_at	0.06	myosin VIIB	MYO7B
1556354_s_at	0.05	erythropoietin receptor	EPOR
239935_at	0.05	MAM domain containing 1	MAMDC1
