# Supplementary material for: In Vitro Transformation of Primary Human CD34+ Cells by AML Fusion Oncogenes: Early Gene Expression Profiling Reveals Possible Drug Target in AML
Source: PLoS One. 2010 Aug 27;5(8):e12464. doi: 10.1371/journal.pone.0012464 (PMC2929205; doi:10.1371/journal.pone.0012464)
Supplement: Table S21 — Genes deregulated by MLL-AF9 8 days after transduction. Primary human CD34+ cells were retrovirally transduced with either control MSCV-IRES-GFP vector or vector expressing MLL-AF9 and sorted for GFP positivity. Total RNA was extracted 8 days after transduction and subjected to microarray analysis. Microarray data were analyzed by SAM as described in Materials and Methods. Significantly deregulated genes are listed and the false discovery rate (FDR) is shown. (0.27 MB PDF) [file pone.0012464.s021.pdf]

Table S21. Genes deregulated by MLL-AF9 at 8 d detected by SAM **FDR = 6.84%**

Probe set ID	Fold Change	Gene Name	Gene Symbol
1553808_a_at	76.95	NK2 transcription factor related, locus 3 (Drosophila)	NKX2-3
237058_x_at	34.20	solute carrier family 6 (neurotransmitter transporter, GABA), member 13	SLC6A13
210239_at	30.76	iroquois homeobox protein 5	IRX5
240915_at	28.55	netrin 2-like (chicken)	NTN2L
1563040_s_at	25.04		
203215_s_at	21.43	myosin VI	MYO6
206367_at	19.87	renin	REN
237403_at	19.75	growth factor independent 1B (potential regulator of CDKN1A, translocated in CML)	GFI1B
1557285_at	18.83		
220512_at	17.65	deleted in liver cancer 1	DLC1
1557566_at	16.35		
243806_at	16.15		
1553868_a_at	16.12	chromosome 5 open reading frame 36	C5orf36
1553613_s_at	15.78	forkhead box C1	FOXC1
243489_at	14.90		
230237_at	14.66		
205932_s_at	14.07	msh homeobox 1	MSX1
232738_at	13.71		
1554619_at	13.36		
203394_s_at	13.14	hairy and enhancer of split 1, (Drosophila)	HES1
237951_at	13.08	phosphatidylinositol glycan anchor biosynthesis, class F	PIGF
202728_s_at	13.07	latent transforming growth factor beta binding protein 1	LTBP1
226677_at	12.15	zinc finger protein 521	ZNF521
1565407_at	12.05	LIM homeobox 9	LHX9
238390_at	11.92	G protein-coupled receptor 39	GPR39
239860_at	11.64		
208292_at	11.58	bone morphogenetic protein 10	BMP10
238919_at	11.54		
219737_s_at	11.00	protocadherin 9	PCDH9
1554524_a_at	10.85	olfactomedin 3	OLFM3
211701_s_at	10.61	trophinin	TRO
239710_at	10.03		
239712_at	9.34	chromosome 9 open reading frame 93	C9orf93
214023_x_at	9.32	tubulin, beta 2B	TUBB2B
230671_at	9.19		
219727_at	9.17	dual oxidase 2	DUOX2
216200_at	9.02	pleckstrin homology domain containing, family M (with RUN domain) member 1	PLEKHM1
1570412_at	8.79		
238460_at	8.76	family with sequence similarity 83, member A	FAM83A
214614_at	8.72	homeobox HB9	HLXB9
215796_at	8.64	T cell receptor alpha locus	TRA@
244885_at	8.57		

217385_at	8.54		
1562342_at	8.52		
236892_s_at	8.43		
212097_at	8.20	caveolin 1, caveolae protein, 22kDa	CAV1
219789_at	7.98	natriuretic peptide receptor C/guanylate cyclase C (atrionatriuretic peptide receptor C)	NPR3
220594_at	7.91	O-linked N-acetylglucosamine (GlcNAc) transferase (UDP-N-acetylglucosamine:polypeptide-N-acetylgluco	OGT
213183_s_at	7.86	cyclin-dependent kinase inhibitor 1C (p57, Kip2)	CDKN1C
206953_s_at	7.69	latrophilin 2	LPHN2
239492_at	7.48	SEC14-like 4 (S. cerevisiae)	SEC14L4
1564438_at	7.35		
1562245_a_at	7.34		
1565436_s_at	7.21	myeloid/lymphoid or mixed-lineage leukemia (trithorax homolog, Drosophila)	MLL
228848_at	7.08	ankyrin repeat and BTB (POZ) domain containing 1	ABTB1
214078_at	7.07		
234565_x_at	6.90		
220102_at	6.87	forkhead box L2	FOXL2
204416_x_at	6.86	apolipoprotein C-I	APOC1
1566438_at	6.80		
226470_at	6.79	gamma-glutamyltransferase-like 3#nuclear receptor coactivator 6#acyl-CoA synthetase short-chain family member 2#high-mobility group (nonhistone chromosomal) protein 4-like	GGTL3#NCOA6#ACSS2#HMG4L
233772_at	6.73		
235955_at	6.71	MARVEL domain containing 2	MARVELD2
236792_at	6.70		
1565834_a_at	6.65	ankyrin repeat domain 15	ANKRD15
242517_at	6.64	KISS1 receptor	KISS1R
228044_at	6.63	chromosome 13 open reading frame 21	C13orf21
237354_at	6.59		
205391_x_at	6.57	ankyrin 1, erythrocytic	ANK1
223652_at	6.55	arsenic (+3 oxidation state) methyltransferase	AS3MT
1565877_at	6.55		
223890_at	6.50		
221169_s_at	6.41	histamine receptor H4	HRH4
239786_at	6.40		
206834_at	6.35	hemoglobin, delta	HBD
212079_s_at	6.33	myeloid/lymphoid or mixed-lineage leukemia (trithorax homolog, Drosophila)	MLL
1556239_a_at	6.31		
213182_x_at	6.28	cyclin-dependent kinase inhibitor 1C (p57, Kip2)	CDKN1C
244403_at	6.18	crumbs homolog 1 (Drosophila)	CRB1
224202_at	6.17	suppressor of fused homolog (Drosophila)	SUFU
222281_s_at	6.15		
1560745_at	6.15		
1553710_at	6.14		
218758_s_at	6.01		
213458_at	6.01	KIAA0974	KIAA0974
1555235_s_at	5.90	IQ motif containing F3	IQCF3

211906_s_at	5.90	serpin peptidase inhibitor, clade B (ovalbumin), member 4	SERPINB4
214414_x_at	5.84	hemoglobin, alpha 1	HBA1
239913_at	5.82	solute carrier family 10 (sodium/bile acid cotransporter family), member 4	SLC10A4
204069_at	5.82	Meis1, myeloid ecotropic viral integration site 1 homolog (mouse)	MEIS1
226676_at	5.77	zinc finger protein 521	ZNF521
219780_at	5.76	zinc finger protein 771	ZNF771
1562431_x_at	5.71		
1555273_at	5.70		
222330_at	5.68		
1553931_at	5.66		
232370_at	5.65		
1561052_s_at	5.65		
215896_at	5.56		
1563329_s_at	5.50		
223642_at	5.44	Zic family member 2 (odd-paired homolog, Drosophila)	ZIC2
213844_at	5.42	homeobox A5	HOXA5
209757_s_at	5.41	v-myc myelocytomatosis viral related oncogene, neuroblastoma derived (avian)	MYCN
217414_x_at	5.37	hemoglobin, alpha 2	HBA2
336_at	5.35	thromboxane A2 receptor	TBXA2R
234409_at	5.30	zinc finger protein 354C	ZNF354C
228904_at	5.30	homeobox B3	HOXB3
220345_at	5.27	leucine rich repeat transmembrane neuronal 4	LRRTM4
1552430_at	5.25	WD repeat domain 17	WDR17
227654_at	5.24	chromosome 20 open reading frame 175	C20orf175
240420_at	5.24	arylacetamide deacetylase-like 2	AADACL2
206072_at	5.20	urocortin	UCN
207990_x_at	5.20	acrosomal vesicle protein 1	ACRV1
231947_at	5.17	myc target 1	MYCT1
240439_at	5.16		
1559889_at	5.16		
212078_s_at	5.14	myeloid/lymphoid or mixed-lineage leukemia (trithorax homolog, Drosophila)	MLL
233170_at	5.12		
237147_at	5.12		
228877_at	5.11	erythropoietin receptor	EPOR
204505_s_at	5.06	erythrocyte membrane protein band 4.9 (dematin)	EPB49
1558856_at	5.06	DMRT-like family A2	DMRTA2
206140_at	5.06	LIM homeobox 2	LHX2
215507_x_at	5.04	RAB22A, member RAS oncogene family	RAB22A
205609_at	4.98	angiopoietin 1	ANGPT1
235521_at	4.98	homeobox A3	HOXA3
229291_at	4.96	RNA binding motif protein 15	RBM15
219895_at	4.94	family with sequence similarity 70, member A	FAM70A
234142_at	4.93		
244844_at	4.93	MRS2-like, magnesium homeostasis factor (S. cerevisiae)	MRS2L
243755_at	4.92	prolactin receptor	PRLR
226103_at	4.89	nexilin (F actin binding protein)	NEXN

230288_at	4.87	fibroblast growth factor 14	FGF14
243637_at	4.87	Fanconi anemia, complementation group C	FANCC
234692_at	4.87		
244639_at	4.87	transmembrane BAX inhibitor motif containing 4	TMBIM4
206145_at	4.84	Rh-associated glycoprotein	RHAG
235086_at	4.82	thrombospondin 1	THBS1
226535_at	4.78	integrin, beta 6	ITGB6
244815_at	4.75	ubiquitin specific peptidase 36	USP36
1569044_at	4.73	CDC42 binding protein kinase gamma (DMPK-like)	CDC42BPG
1556695_a_at	4.72		
206281_at	4.72	adenylate cyclase activating polypeptide 1 (pituitary)	ADCYAP1
1555444_a_at	4.71	protein phosphatase 1, regulatory (inhibitor) subunit 12B	PPP1R12B
1552372_at	4.70	chromosome 4 open reading frame 33	C4orf33
241643_at	4.70	tousled-like kinase 1	TLK1
227475_at	4.65	forkhead box Q1	FOXQ1
235362_at	4.65		
209498_at	4.65	carcinoembryonic antigen-related cell adhesion molecule 1 (biliary glycoprotein)	CEACAM1
209108_at	4.62	tetraspanin 6	TSPAN6
243087_at	4.61	WD repeat domain 63	WDR63
227472_at	4.61	chromosome 19 open reading frame 58	C19orf58
208613_s_at	4.60	filamin B, beta (actin binding protein 278)	FLNB
208711_s_at	4.60	cyclin D1	CCND1
239121_at	4.57	PTK2 protein tyrosine kinase 2	PTK2
225381_at	4.57		
229999_at	4.56		
228621_at	4.51	hemochromatosis type 2 (juvenile)	HFE2
218630_at	4.50	Meckel syndrome, type 1	MKS1
228724_at	4.50		
212070_at	4.49	G protein-coupled receptor 56	GPR56
231599_x_at	4.48	D4, zinc and double PHD fingers family 1	DPF1
233960_s_at	4.48		
227190_at	4.48	transmembrane protein 37	TMEM37
235318_at	4.43	fibrillin 1	FBN1
209120_at	4.43	nuclear receptor subfamily 2, group F, member 2	NR2F2
221273_s_at	4.43	ring finger protein 208	RNF208
1559477_s_at	4.43	Meis1, myeloid ecotropic viral integration site 1 homolog (mouse)	MEIS1
214159_at	4.41	phospholipase C, epsilon 1	PLCE1
1562639_at	4.41	kinesin family member 6	KIF6
241499_at	4.38	zinc finger protein 621	ZNF621
230357_at	4.37		
244508_at	4.35	septin 7	7-Sep
204848_x_at	4.32	hemoglobin, gamma A	HBG1
234475_x_at	4.31	cholecystokinin B receptor	CCKBR
1563496_at	4.30		
1555659_a_at	4.29	triggering receptor expressed on myeloid cells-like 1	TREML1
1566576_at	4.29		
204720_s_at	4.29	DnaJ (Hsp40) homolog, subfamily C, member 6	DNAJC6
239248_at	4.27		

1569231_x_at	4.26		
241089_at	4.25		
215296_at	4.24	CDC42 binding protein kinase alpha (DMPK-like)	CDC42BPA
242021_at	4.23		
210446_at	4.22	GATA binding protein 1 (globin transcription factor 1)	GATA1
206251_s_at	4.21	arginine vasopressin receptor 1A	AVPR1A
243268_at	4.21	chromosome 11 open reading frame 38	C11orf38
228311_at	4.21	B-cell CLL/lymphoma 6, member B (zinc finger protein)	BCL6B
211113_s_at	4.19	ATP-binding cassette, sub-family G (WHITE), member 1	ABCG1
239109_at	4.19		
222755_s_at	4.16	chromodomain helicase DNA binding protein 7	CHD7
1556781_at	4.16		
239210_at	4.15		
240321_at	4.15		
236912_at	4.15		
243538_at	4.15		
1553882_at	4.15		
1561274_at	4.14		
1566606_a_at	4.14	testis expressed sequence 9	TEX9
225491_at	4.12	solute carrier family 1 (glial high affinity glutamate transporter), member 2	SLC1A2
240769_at	4.12		
229638_at	4.12	iroquois homeobox protein 3	IRX3
241780_at	4.11		
242152_at	4.10		
1564950_at	4.10		
220205_at	4.09	transmembrane phosphatase with tensin homology	TPTE
214156_at	4.09	myosin VIIA and Rab interacting protein	MYRIP
205767_at	4.09	epiregulin	EREG
215132_at	4.06		
243804_at	4.06	myotubularin related protein 7	MTMR7
1559975_at	4.06	B-cell translocation gene 1, anti-proliferative	BTG1
242785_at	4.04		
222950_at	4.04	NIPA-like domain containing 2	NPAL2
1562562_at	4.04		
237175_at	4.04		
231515_at	4.03		
1569277_at	4.03	zinc finger protein 91	ZNF91
1570578_at	4.02		
213348_at	4.02	cyclin-dependent kinase inhibitor 1C (p57, Kip2)	CDKN1C
1553260_s_at	4.00	amyotrophic lateral sclerosis 2 (juvenile) chromosome region, candidate 11	ALS2CR11
1561279_at	3.99		
204292_x_at	3.99	serine/threonine kinase 11	STK11
204419_x_at	3.97	hemoglobin, gamma G	HBG2
220803_at	3.97	STAM binding protein-like 1	STAMBPL1
208712_at	3.94	cyclin D1	CCND1
205390_s_at	3.94	ankyrin 1, erythrocytic	ANK1
237907_at	3.92	casein kinase 2, alpha 1 polypeptide	CSNK2A1
213515_x_at	3.91	hemoglobin, gamma G	HBG2

242203_at	3.90		
228274_at	3.89	serine dehydratase-like	SDSL
226084_at	3.89	microtubule-associated protein 1B	MAP1B
216016_at	3.88	NLR family, pyrin domain containing 3	NLRP3
211124_s_at	3.88	KIT ligand	KITLG
217708_x_at	3.88		
234231_at	3.87		
220030_at	3.87	serine/threonine/tyrosine kinase 1	STYK1
215810_x_at	3.87		
216189_at	3.86		
238049_at	3.86		
229592_at	3.86		
204018_x_at	3.85	hemoglobin, alpha 1	HBA1
224237_at	3.85		
243460_at	3.85		
234621_at	3.84		
227314_at	3.83	integrin, alpha 2 (CD49B, alpha 2 subunit of VLA-2 receptor)	ITGA2
237150_at	3.82		
211745_x_at	3.82	hemoglobin, alpha 2	HBA2
1557826_at	3.82		
219054_at	3.81	chromosome 5 open reading frame 23	C5orf23
1557080_s_at	3.81	integrin, beta-like 1 (with EGF-like repeat domains)	ITGBL1
215768_at	3.81		
240516_at	3.80		
209881_s_at	3.79	linker for activation of T cells	LAT
229151_at	3.78	solute carrier family 14 (urea transporter), member 1 (Kidd blood group)	SLC14A1
239552_at	3.78		
1553013_at	3.78	contactin associated protein-like 5	CNTNAP5
241362_at	3.77	chromosome 20 open reading frame 117	C20orf117
217630_at	3.77	angel homolog 2 (Drosophila)	ANGEL2
1561703_at	3.77		
209458_x_at	3.77	hemoglobin, alpha 1	HBA1
201110_s_at	3.77	thrombospondin 1	THBS1
206588_at	3.76	deleted in azoospermia-like	DAZL
207206_s_at	3.76	arachidonate 12-lipoxygenase	ALOX12
234014_at	3.76		
241184_x_at	3.75	zinc finger protein 407	ZNF407
226189_at	3.75	integrin, beta 8	ITGB8
205268_s_at	3.74	adducin 2 (beta)	ADD2
242088_at	3.74	kelch-like 24 (Drosophila)	KLHL24
243159_x_at	3.74	myosin X	MYO10
1562901_at	3.73		
244456_at	3.73	inositol 1,4,5-triphosphate receptor, type 2	ITPR2
201610_at	3.72	isoprenylcysteine carboxyl methyltransferase	ICMT
215674_at	3.70		
244502_at	3.70		
207273_at	3.69		
224322_at	3.68	AT rich interactive domain 4B (RBP1-like)	ARID4B
241453_at	3.66	PTK2 protein tyrosine kinase 2	PTK2
204136_at	3.66	collagen, type VII, alpha 1 (epidermolysis bullosa, dystrophic, dominant and recessive)	COL7A1

239595_at	3.65	glutathione peroxidase 2 (gastrointestinal)	GPX2
205857_at	3.65	solute carrier family 18 (vesicular monoamine), member 2	SLC18A2
1556404_a_at	3.65		
209670_at	3.64	T cell receptor alpha constant	TRAC
243947_s_at	3.63		
217624_at	3.63	PDGFA associated protein 1	PDAP1
213719_s_at	3.62	SWI/SNF related, matrix associated, actin dependent regulator of chromatin, subfamily a, member 4	SMARCA4
204541_at	3.62	SEC14-like 2 (S. cerevisiae)	SEC14L2
206927_s_at	3.61	guanylate cyclase 1, soluble, alpha 2	GUCY1A2
203373_at	3.60	suppressor of cytokine signaling 2	SOCS2
1562992_at	3.60		
229706_at	3.58	transcription elongation regulator 1	TCERG1
237502_at	3.58	cardiolipin synthase 1	CRLS1
236738_at	3.58		
223966_at	3.57		
242662_at	3.57	proprotein convertase subtilisin/kexin type 6	PCSK6
1563569_at	3.56		
1568593_a_at	3.56	nudix (nucleoside diphosphate linked moiety X)-type motif 16 pseudogene	NUDT16P
227051_at	3.55		
1562514_at	3.55		
238605_at	3.55		
217037_at	3.55		
203372_s_at	3.53	suppressor of cytokine signaling 2	SOCS2
206951_at	3.53	histone cluster 1, H4e	HIST1H4E
211748_x_at	3.51	prostaglandin D2 synthase 21kDa (brain)	PTGDS
1568366_at	3.51		
221175_at	3.51	chromosome 3 open reading frame 36	C3orf36
212364_at	3.50	myosin IB	MYO1B
203808_at	3.50	v-akt murine thymoma viral oncogene homolog 2	AKT2
210583_at	3.50	polymerase (DNA-directed), delta interacting protein 3	POLDIP3
239399_at	3.48		
1552908_at	3.48	chromosome 1 open reading frame 150	C1orf150
237322_at	3.47		
232574_at	3.45	xylosyltransferase I	XYLT1
216240_at	3.44	Pvt1 oncogene homolog, MYC activator (mouse)	PVT1
243896_at	3.42	chromosome 10 open reading frame 79	C10orf79
1552585_s_at	3.40		
206522_at	3.40	maltase-glucoamylase (alpha-glucosidase)	MGAM
233026_s_at	3.39	PDZ domain containing 2	PDZD2
228708_at	3.39		
229774_at	3.39	CXXC finger 4	CXXC4
244443_at	3.38	chromodomain helicase DNA binding protein 2	CHD2
207346_at	3.37	syntaxin 2	STX2
242172_at	3.37	Meis1, myeloid ecotropic viral integration site 1 homolog (mouse)	MEIS1
1566580_at	3.37		
230840_at	3.37		
234538_at	3.35		

217263_x_at	3.34	runt-related transcription factor 1 (acute myeloid leukemia 1; aml1 oncogene)	RUNX1
223395_at	3.34	ABI gene family, member 3 (NESH) binding protein	ABI3BP
236536_at	3.33	UDP-N-acetyl-alpha-D-galactosamine:polypeptide N-acetylgalactosaminyltransferase 13 (GalNAc-T13)	GALNT13
239956_at	3.33		
233610_at	3.33		
235057_at	3.33	itchy homolog E3 ubiquitin protein ligase (mouse)	ITCH
235753_at	3.32	homeobox A7	HOXA7
226234_at	3.32		
232257_s_at	3.31		
1561581_at	3.31		
221232_s_at	3.31	ankyrin repeat domain 2 (stretch responsive muscle)	ANKRD2
205103_at	3.30	chromosome 1 open reading frame 61	C1orf61
206165_s_at	3.30	chloride channel, calcium activated, family member 2	CLCA2
1564676_a_at	3.29		
1563659_at	3.29	hect domain and RLD 6	HERC6
209116_x_at	3.28	hemoglobin, beta	HBB
240116_at	3.28	AT rich interactive domain 1B (SWI1-like)	ARID1B
211699_x_at	3.27	hemoglobin, alpha 1	HBA1
214146_s_at	3.27	pro-platelet basic protein (chemokine (C-X-C motif) ligand 7)	PPBP
241758_at	3.26	nucleoporin 93kDa	NUP93
220851_at	3.26		
1559953_at	3.26	DEAD (Asp-Glu-Ala-Asp) box polypeptide 42	DDX42
229935_s_at	3.25	myeloid/lymphoid or mixed-lineage leukemia (trithorax homolog, Drosophila)	MLL
231534_at	3.25	cell division cycle 2, G1 to S and G2 to M	CDC2
235976_at	3.24	SLIT and NTRK-like family, member 6	SLITRK6
240268_at	3.24		
1555704_at	3.23	CKLF-like MARVEL transmembrane domain containing 3	CMTM3
1560219_at	3.23		
234492_at	3.23		
211696_x_at	3.23	hemoglobin, beta	HBB
1558202_at	3.22		
240351_at	3.21		
218523_at	3.21		
232851_at	3.21	F-box protein 3	FBXO3
1561141_at	3.20		
213927_at	3.19	mitogen-activated protein kinase kinase kinase 9	MAP3K9
219463_at	3.19	chromosome 20 open reading frame 103	C20orf103
1561002_at	3.19		
218745_x_at	3.19	transmembrane protein 161A	TMEM161A
208186_s_at	3.18	lipase, hormone-sensitive	LIPE
206307_s_at	3.17	forkhead box D1	FOXD1
1553328_a_at	3.17	solute carrier family 18 (vesicular monoamine), member 2	SLC18A2
233786_at	3.16		
221594_at	3.16		

1561460_at	3.16		
206346_at	3.16	prolactin receptor	PRLR
244147_at	3.15		
1560105_at	3.15	protein tyrosine phosphatase, receptor type, B	PTPRB
221863_at	3.15	mesoderm induction early response 1, family member 2	MIER2
244006_at	3.15		
230803_s_at	3.14	Rho GTPase activating protein 24	ARHGAP24
243089_at	3.14	Kruppel-like factor 12	KLF12
216814_at	3.14	SERPINE1 mRNA binding protein 1 pseudogene	SERBP1P
218484_at	3.13	NADH dehydrogenase (ubiquinone) 1 alpha subcomplex, 4-like 2	NDUFA4L2
223539_s_at	3.13	small EDRK-rich factor 1A (telomeric)	SERF1A
215818_at	3.13	nudix (nucleoside diphosphate linked moiety X)-type motif 7	NUDT7
240953_at	3.13		
233727_at	3.13		
236516_at	3.12	CXXC finger 5	CXXC5
202894_at	3.12	EPH receptor B4	EPHB4
202588_at	3.11	adenylate kinase 1	AK1
228923_at	3.11	S100 calcium binding protein A6	S100A6
206067_s_at	3.11	Wilms tumor 1	WT1
204141_at	3.10	tubulin, beta 2A	TUBB2A
230717_at	3.10	lipocalcin 12	LCN12
235184_at	3.09	AE binding protein 2	AEBP2
221942_s_at	3.09	guanylate cyclase 1, soluble, alpha 3	GUCY1A3
1561262_at	3.09		
242843_at	3.08	brevican	BCAN
205848_at	3.07	growth arrest-specific 2	GAS2
240768_x_at	3.06		
214995_s_at	3.06	apolipoprotein B mRNA editing enzyme, catalytic polypeptide-like 3F	APOBEC3F
229523_at	3.06		
208353_x_at	3.05	ankyrin 1, erythrocytic	ANK1
240211_at	3.04		
238831_at	3.04		
1553804_a_at	3.04	chromosome 17 open reading frame 46	C17orf46
228636_at	3.03	basic helix-loop-helix domain containing, class B, 5	BHLHB5
224058_s_at	3.02	hydroxysteroid (17-beta) dehydrogenase 7 pseudogene 2	HSD17B7P2
239645_at	3.02	cerebral cavernous malformation 2	CCM2
237252_at	3.02	thrombomodulin	THBD
244162_at	3.02		
226211_at	3.01	maternally expressed 3	MEG3
215241_at	3.01	transmembrane protein 16C	TMEM16C
216589_at	3.01	tumor necrosis factor receptor superfamily, member 1B#vacuolar protein sorting 13 homolog D (S. cerevisiae)#vacuolar protein sorting 13 homolog D (S. cerevisiae)	TNFRSF1B#VPS13D#VPS13D
236645_at	3.00	HMG-box transcription factor 1	HBP1
238301_at	3.00		
222110_at	3.00	SUMO1/sentrin specific peptidase 5	SENP5
213894_at	2.99	thrombospondin, type I, domain containing 7A	THSD7A

240394_at	2.99		
206622_at	2.97	thyrotropin-releasing hormone	TRH
209905_at	2.97	homeobox A9	HOXA9
210230_at	2.97		
209399_at	2.97	holocarboxylase synthetase (biotin-(proprionyl-Coenzyme A-carboxylase (ATP-hydrolysing)) ligase)	HLCS
206847_s_at	2.97	homeobox A7	HOXA7
1553175_s_at	2.96	phosphodiesterase 5A, cGMP-specific	PDE5A
1554319_at	2.96	ribosomal protein S6 kinase, 90kDa, polypeptide 5	RPS6KA5
1560284_at	2.96		
1553970_s_at	2.95	carboxyl ester lipase (bile salt-stimulated lipase)	CEL
1553394_a_at	2.94	transcription factor AP-2 beta (activating enhancer binding protein 2 beta)	TFAP2B
210226_at	2.94	nuclear receptor subfamily 4, group A, member 1	NR4A1
205935_at	2.94	forkhead box F1	FOXF1
232968_at	2.93	fibronectin type III and ankyrin repeat domains 1	FANK1
218466_at	2.93	TBC1 domain family, member 17	TBC1D17
1565861_at	2.93		
238336_s_at	2.93		
237009_at	2.93	CD69 molecule	CD69
221024_s_at	2.93	solute carrier family 2 (facilitated glucose transporter), member 10	SLC2A10
216703_at	2.92		
209818_s_at	2.92	hyaluronan binding protein 4	HABP4
207741_x_at	2.92	tryptase beta 2	TPSB2
207010_at	2.92	gamma-aminobutyric acid (GABA) A receptor, beta 1	GABRB1
217574_at	2.92	cadherin 8, type 2	CDH8
228915_at	2.92	dachshund homolog 1 (Drosophila)	DACH1
244181_at	2.91	phosphoinositide-3-kinase, regulatory subunit 1 (p85 alpha)	PIK3R1
232119_at	2.90	synaptopodin 2	SYNPO2
243868_at	2.90		
201150_s_at	2.90	TIMP metallopeptidase inhibitor 3 (Sorsby fundus dystrophy, pseudoinflammatory)	TIMP3
241099_at	2.89	elongation protein 4 homolog (S. cerevisiae)	ELP4
238100_at	2.89		
241844_x_at	2.89	transmembrane protein 156	TMEM156
205472_s_at	2.89	dachshund homolog 1 (Drosophila)	DACH1
222776_at	2.88		
1569274_at	2.88		
207087_x_at	2.87	ankyrin 1, erythrocytic	ANK1
234393_at	2.87	histone deacetylase 9	HDAC9
237633_at	2.86		
244267_at	2.86	special AT-rich sequence binding protein 1 (binds to nuclear matrix/scaffold-associating DNA's)	SATB1
236237_at	2.86	KIAA1370	KIAA1370
1562440_at	2.86	mitogen-activated protein kinase kinase kinase 13	MAP3K13
205386_s_at	2.85	Mdm2, transformed 3T3 cell double minute 2, p53 binding protein (mouse)	MDM2
232610_at	2.85	poly (ADP-ribose) polymerase family, member 14	PARP14
216793_x_at	2.85		

1562850_at	2.85		
233944_at	2.85		
220551_at	2.85	solute carrier family 17 (sodium-dependent inorganic phosphate cotransporter), member 6	SLC17A6
227503_at	2.84		
219106_s_at	2.84	kelch repeat and BTB (POZ) domain containing 10	KBTBD10
1563453_at	2.84		
221125_s_at	2.83	potassium large conductance calcium-activated channel, subfamily M beta member 3	KCNMB3
243744_at	2.83		
206306_at	2.83	ryanodine receptor 3	RYR3
1564907_s_at	2.83	small nucleolar RNA host gene (non-protein coding) 4	SNHG4
205608_s_at	2.83	angiopoietin 1	ANGPT1
230594_at	2.82		
237291_at	2.82		
236521_at	2.81		
236414_at	2.81		
226446_at	2.81	hairy and enhancer of split 6 (Drosophila)	HES6
243814_at	2.79	zinc finger, MYND-type containing 8	ZMYND8
207867_at	2.79	paired box gene 4	PAX4
204256_at	2.79	ELOVL family member 6, elongation of long chain fatty acids (FEN1/Elo2, SUR4/Elo3-like, yeast)	ELOVL6
213553_x_at	2.79	apolipoprotein C-I	APOC1
234009_at	2.79		
217680_x_at	2.79		
206026_s_at	2.79	tumor necrosis factor, alpha-induced protein 6	TNFAIP6
1559877_at	2.79		
215655_at	2.78	glutamate receptor, ionotropic, kainate 2	GRIK2
240175_at	2.78		
211925_s_at	2.78	phospholipase C, beta 1 (phosphoinositide-specific)	PLCB1
221577_x_at	2.78	growth differentiation factor 15	GDF15
222082_at	2.76	zinc finger and BTB domain containing 7A	ZBTB7A
1562983_at	2.76		
243136_at	2.76		
1564805_a_at	2.76	orofacial cleft 1 candidate 1	OFCC1
243582_at	2.76	SH3 domain containing ring finger 2	SH3RF2
236092_at	2.75		
1560590_s_at	2.75		
204628_s_at	2.74	integrin, beta 3 (platelet glycoprotein IIIa, antigen CD61)	ITGB3
241654_at	2.74		
213109_at	2.74	TRAF2 and NCK interacting kinase	TNIK
241840_at	2.74		
240866_at	2.73		
221008_s_at	2.73	alanine-glyoxylate aminotransferase 2-like 1	AGXT2L1
204945_at	2.73	protein tyrosine phosphatase, receptor type, N	PTPRN
216039_at	2.73	postmeiotic segregation increased 2-like 1	PMS2L1
1554648_a_at	2.72	dual oxidase maturation factor 1	DUOXA1
207134_x_at	2.72	tryptase beta 2	TPSB2
1555028_at	2.71	bromodomain containing 3	BRD3
1555487_a_at	2.71	ARP3 actin-related protein 3 homolog B (yeast)	ACTR3B
1559675_at	2.71		

214053_at	2.70	v-erb-a erythroblastic leukemia viral oncogene homolog 4 (avian)	ERBB4
223779_at	2.70		
238464_at	2.69	KIAA1641	KIAA1641
228250_at	2.68	folliculin interacting protein 1	FNIP1
1567101_at	2.68		
204017_at	2.68	KDEL (Lys-Asp-Glu-Leu) endoplasmic reticulum protein retention receptor 3	KDELR3
1555403_a_at	2.68	cadherin 19, type 2	CDH19
216894_x_at	2.67	cyclin-dependent kinase inhibitor 1C (p57, Kip2)	CDKN1C
212013_at	2.67	peroxidasin homolog (Drosophila)	PXDN
213742_at	2.67	splicing factor, arginine/serine-rich 11	SFRS11
215745_at	2.67		
239723_at	2.67		
239506_s_at	2.67		
209204_at	2.67	LIM domain only 4	LMO4
204779_s_at	2.66	homeobox B7	HOXB7
233054_at	2.65	CCR4-NOT transcription complex, subunit 2	CNOT2
1555136_at	2.65	FYVE, RhoGEF and PH domain containing 6	FGD6
1570251_at	2.65	HECT domain containing 1	HECTD1
208352_x_at	2.64	ankyrin 1, erythrocytic	ANK1
1561763_at	2.64		
214120_at	2.64	ret finger protein-like 1 antisense	RFPL1S
1553645_at	2.63		
242183_at	2.63		
236107_at	2.63	ubiquitin-conjugating enzyme E2Z (putative)	UBE2Z
220250_at	2.62	zinc finger protein 286A	ZNF286A
240735_at	2.62	CDC42 binding protein kinase alpha (DMPK-like)	CDC42BPA
220299_at	2.62	spermatogenesis associated 6	SPATA6
227392_at	2.62	nischarin	NISCH
208016_s_at	2.62	angiotensin II receptor, type 1	AGTR1
242603_x_at	2.62		
201147_s_at	2.61	TIMP metallopeptidase inhibitor 3 (Sorsby fundus dystrophy, pseudoinflammatory)	TIMP3
213343_s_at	2.61	glycerophosphodiester phosphodiesterase domain containing 5	GDPD5
235102_x_at	2.60	GRB2-related adaptor protein	GRAP
236023_at	2.60		
236161_at	2.59		
241640_at	2.59		
1559172_at	2.59		
203323_at	2.58	caveolin 2	CAV2
1562282_at	2.58	zinc finger protein 568	ZNF568
239282_at	2.58		
229003_x_at	2.58	family with sequence similarity 69, member B	FAM69B
241155_at	2.58	phosphatidylinositol-4-phosphate 5-kinase, type II, alpha	PIP5K2A
235852_at	2.58	stonin 2	STON2
236946_at	2.57	G protein-coupled receptor 75	GPR75
207911_s_at	2.56	transglutaminase 5	TGM5
238277_at	2.55	tankyrase, TRF1-interacting ankyrin-related ADP-ribose polymerase	TNKS
205389_s_at	2.55	ankyrin 1, erythrocytic	ANK1

1561864_at	2.54		
221187_s_at	2.54	fuzzy homolog (Drosophila)	FUZ
217544_at	2.54		
206954_at	2.54	Wilms tumor upstream neighbor 1	WIT1
225270_at	2.54	neogenin homolog 1 (chicken)	NEO1
237583_at	2.53		
215456_at	2.53		
237723_at	2.53	SMAD specific E3 ubiquitin protein ligase 1	SMURF1
217137_x_at	2.53		
218279_s_at	2.52	histone cluster 2, H2aa3#histone cluster 2, H2aa4#histone cluster 2, H2aa4	HIST2H2AA3# HIST2H2AA4# HIST2H2AA4
221251_x_at	2.52	zinc finger, HIT type 4	ZNHIT4
235149_at	2.52	phosphoglucomutase 2-like 1	PGM2L1
241809_at	2.52	chromosome 1 open reading frame 183	C1orf183
242508_at	2.52	KIAA2018	KIAA2018
233191_at	2.51	RUN and FYVE domain containing 2	RUFY2
210504_at	2.51	Kruppel-like factor 1 (erythroid)	KLF1
224189_x_at	2.51	ets homologous factor	EHF
1553105_s_at	2.51	desmoglein 2	DSG2
203817_at	2.51	guanylate cyclase 1, soluble, beta 3	GUCY1B3
213257_at	2.50	sterile alpha and TIR motif containing 1	SARM1
230319_at	2.50		
205195_at	2.50	adaptor-related protein complex 1, sigma 1 subunit	AP1S1
239167_at	2.50		
1562249_at	2.50		
207125_at	2.49	zinc finger protein 225	ZNF225
215802_at	2.49		
217232_x_at	2.49	hemoglobin, beta	HBB
242622_x_at	2.49	phosphatase and tensin homolog (mutated in multiple advanced cancers 1)	PTEN
239674_at	2.49	AT rich interactive domain 1B (SWI1-like)	ARID1B
213917_at	2.49		
239503_at	2.49		
241278_at	2.48	Fc receptor-like 3	FCRL3
213543_at	2.48		
214597_at	2.48	somatostatin receptor 2	SSTR2
211132_at	2.48	integrator complex subunit 3	INTS3
237402_at	2.48	glutathione reductase	GSR
210084_x_at	2.48	tryptase alpha/beta 1	TPSAB1
227155_at	2.48		
204304_s_at	2.47	prominin 1	PROM1
239596_at	2.47	solute carrier family 30 (zinc transporter), member 7	SLC30A7
205919_at	2.47	hemoglobin, epsilon 1	HBE1
242097_at	2.47		
211832_s_at	2.47	Mdm2, transformed 3T3 cell double minute 2, p53 binding protein (mouse)	MDM2
206283_s_at	2.46	T-cell acute lymphocytic leukemia 1	TAL1
204731_at	2.46	transforming growth factor, beta receptor III (betaglycan, 300kDa)	TGFBR3
214020_x_at	2.46	integrin, beta 5	ITGB5
235039_x_at	2.46	lin-9 homolog (C. elegans)	LIN9

1564315_at	2.46	chromosome 8 open reading frame 49	C8orf49
208719_s_at	2.46	DEAD (Asp-Glu-Ala-Asp) box polypeptide 17	DDX17
244027_at	2.46	ATP-binding cassette, sub-family B (MDR/TAP), member 7	ABCB7
231142_at	2.45		
232769_at	2.44		
232427_at	2.44	zinc finger protein 224	ZNF224
1559510_at	2.44	basic helix-loop-helix domain containing, class B, 9	BHLHB9
223840_s_at	2.44	spermatogenesis associated 9	SPATA9
231482_at	2.43		
201418_s_at	2.43	SRY (sex determining region Y)-box 4	SOX4
235541_at	2.43	LAS1-like (S. cerevisiae)	LAS1L
211079_s_at	2.42	dual-specificity tyrosine-(Y)-phosphorylation regulated kinase 1A	DYRK1A
241316_at	2.42	trinucleotide repeat containing 6A	TNRC6A
1555303_at	2.41		
1556999_at	2.41		
242982_x_at	2.41	integrin, beta 8	ITGB8
243381_at	2.41		
1565951_s_at	2.41	choroideremia-like (Rab escort protein 2)	CHML
232564_at	2.41	solute carrier family 9 (sodium/hydrogen exchanger), member 5	SLC9A5
231798_at	2.41	noggin	NOG
237784_at	2.41	SUB1 homolog (S. cerevisiae)	SUB1
238119_at	2.41		
1560119_at	2.41		
1558122_s_at	2.40		
236334_at	2.40		
239014_at	2.39	cell division cycle and apoptosis regulator 1	CCAR1
216179_x_at	2.39		
239955_at	2.39		
207480_s_at	2.39	Meis1, myeloid ecotropic viral integration site 1 homolog 2 (mouse)	MEIS2
1554303_at	2.39	histamine N-methyltransferase	HNMT
214871_x_at	2.39		
1563900_at	2.39	family with sequence similarity 83, member B	FAM83B
1552694_at	2.39	solute carrier family 2 (facilitated glucose transporter), member 13	SLC2A13
216191_s_at	2.38	T cell receptor alpha locus	TRA@
236546_at	2.38		
210703_at	2.38		
209361_s_at	2.37	poly(rC) binding protein 4	PCBP4
1562244_at	2.37		
210215_at	2.37	transferrin receptor 2	TFR2
225115_at	2.37	homeodomain interacting protein kinase 2	HIPK2
206937_at	2.37	spectrin, alpha, erythrocytic 1 (elliptocytosis 2)	SPTA1
214084_x_at	2.37	neutrophil cytosolic factor 1, (chronic granulomatous disease, autosomal 1)	NCF1
1566947_at	2.37		
243260_x_at	2.37		
233372_at	2.37		
235952_at	2.37		
220145_at	2.37	microtubule-associated protein 9	MAP9

1554631_at	2.36	ataxia telangiectasia mutated (includes complementation groups A, C and D)	ATM
241613_at	2.36		
231146_at	2.36	family with sequence similarity 24, member B	FAM24B
222108_at	2.36	adhesion molecule with Ig-like domain 2	AMIGO2
207526_s_at	2.36	interleukin 1 receptor-like 1	IL1RL1
240773_at	2.36		
216459_x_at	2.36		
216058_s_at	2.36	cytochrome P450, family 2, subfamily C, polypeptide 19	CYP2C19
242741_x_at	2.35	NOL1/NOP2/Sun domain family, member 4	NSUN4
240107_at	2.35		
210809_s_at	2.35	periostin, osteoblast specific factor	POSTN
220672_at	2.35	KIAA1622	KIAA1622
240520_at	2.35	proline rich 8	PRR8
237999_at	2.35	zinc finger, DHHC-type containing 13	ZDHHC13
237189_at	2.35		
244047_at	2.35		
213665_at	2.35	SRY (sex determining region Y)-box 4	SOX4
230220_at	2.35		
222341_x_at	2.35		
240965_at	2.34	anaphase promoting complex subunit 10	ANAPC10
207568_at	2.34	cholinergic receptor, nicotinic, alpha 6	CHRNA6
234306_s_at	2.34	SLAM family member 7	SLAMF7
217025_s_at	2.34	drebrin 1	DBN1
235062_at	2.34	PIH1 domain containing 2	PIH1D2
244153_at	2.34		
211005_at	2.33	linker for activation of T cells	LAT
1558523_at	2.33	chromosome 6 open reading frame 60	C6orf60
1563858_at	2.33		
214250_at	2.32	nuclear mitotic apparatus protein 1	NUMA1
220234_at	2.32	carbonic anhydrase VIII	CA8
210282_at	2.32	zinc finger, MYM-type 2	ZMYM2
240787_at	2.32		
1554963_at	2.32	chromosome 6 open reading frame 192	C6orf192
237839_at	2.32	ankyrin 3, node of Ranvier (ankyrin G)	ANK3
201655_s_at	2.32	heparan sulfate proteoglycan 2 (perlecan)	HSPG2
235147_at	2.32		
216470_x_at	2.31	protease, serine, 2 (trypsin 2)	PRSS2
227259_at	2.31	CD47 molecule	CD47
1562947_x_at	2.31		
212187_x_at	2.31	prostaglandin D2 synthase 21kDa (brain)	PTGDS
234074_at	2.31		
207415_at	2.30	phospholipase A2 receptor 1, 180kDa	PLA2R1
1559585_at	2.30		
228749_at	2.30		
220609_at	2.30		
1558887_at	2.30		
1556336_at	2.30	RNA binding motif protein, X-linked	RBMX
210358_x_at	2.29	GATA binding protein 2	GATA2
244696_at	2.29	AF4/FMR2 family, member 3	AFF3
208025_s_at	2.29	high mobility group AT-hook 2	HMGA2
223514_at	2.29	caspase recruitment domain family, member 11	CARD11

233121_at	2.29		
227877_at	2.29		
240927_at	2.29		
237988_at	2.28	eukaryotic translation initiation factor 1B	EIF1B
1555292_at	2.28	family with sequence similarity 40, member B	FAM40B
238706_at	2.28	PAP associated domain containing 4	PAPD4
241298_x_at	2.28		
239023_at	2.28		
219645_at	2.28	calsequestrin 1 (fast-twitch, skeletal muscle)	CASQ1
244026_at	2.28	elongation factor, RNA polymerase II, 2	ELL2
217867_x_at	2.27	beta-site APP-cleaving enzyme 2	BACE2
222956_at	2.27	fidgetin	FIGN
208151_x_at	2.27	DEAD (Asp-Glu-Ala-Asp) box polypeptide 17	DDX17
233912_x_at	2.26		
243138_at	2.26		
228755_at	2.25	PERQ amino acid rich, with GYF domain 1	PERQ1
207589_at	2.25	adrenergic, alpha-1B-, receptor	ADRA1B
233007_at	2.25		
223766_at	2.25		
222267_at	2.25		
1552792_at	2.25	suppressor of cytokine signaling 4	SOCS4
223079_s_at	2.24	glutaminase	GLS
230383_x_at	2.24		
222938_x_at	2.24	ectonucleotide pyrophosphatase/phosphodiesterase 3	ENPP3
208790_s_at	2.24	polymerase I and transcript release factor	PTRF
202806_at	2.24	drebrin 1	DBN1
236757_at	2.24		
217536_x_at	2.23		
237833_s_at	2.23	synuclein, alpha interacting protein (synphilin)	SNCAIP
240241_at	2.23	mitogen-activated protein kinase associated protein 1	MAPKAP1
215457_at	2.23		
228696_at	2.23	solute carrier family 45, member 3	SLC45A3
225308_s_at	2.23	tetratricopeptide repeat, ankyrin repeat and coiled-coil containing 1	TANC1
233879_at	2.23		
223567_at	2.23	sema domain, transmembrane domain (TM), and cytoplasmic domain, (semaphorin) 6B	SEMA6B
201083_s_at	2.22	BCL2-associated transcription factor 1	BCLAF1
215968_at	2.22		
241402_at	2.22	tRNA splicing endonuclease 54 homolog (S. cerevisiae)	TSEN54
214950_at	2.22	interleukin 9 receptor	IL9R
243715_at	2.22		
229139_at	2.22	junctophilin 1	JPH1
209710_at	2.22	GATA binding protein 2	GATA2
204187_at	2.22	guanosine monophosphate reductase	GMPR
235811_at	2.22	muscleblind-like (Drosophila)	MBNL1
1559441_s_at	2.22		
236284_at	2.22	KIAA0146	KIAA0146
1560821_at	2.21	Rho GTPase activating protein 22	ARHGAP22
238964_at	2.21		

220116_at	2.21	potassium intermediate/small conductance calcium-activated channel, subfamily N, member 2	KCNN2
241416_at	2.21		
241763_s_at	2.21	F-box protein 32	FBXO32
243361_at	2.21	splicing factor, arginine/serine-rich 12	SFRS12
235592_at	2.20		
212233_at	2.20		
230552_at	2.20		
239661_at	2.20		
235886_at	2.20		
231555_at	2.20		
1561731_at	2.20		
219368_at	2.20	nucleosome assembly protein 1-like 2	NAP1L2
235229_at	2.19		
225175_s_at	2.19	solute carrier family 44, member 2	SLC44A2
236966_at	2.19	thioredoxin domain containing 6	TXNDC6
215846_at	2.19		
207067_s_at	2.19	histidine decarboxylase	HDC
240626_at	2.19	chromosome 8 open reading frame 15	C8orf15
241336_at	2.19	ribosomal protein L32 pseudogene 3	RPL32P3
207329_at	2.19	matrix metallopeptidase 8 (neutrophil collagenase)	MMP8
243763_x_at	2.19		
239165_at	2.18		
240613_at	2.18		
226751_at	2.18	chromosome 2 open reading frame 32	C2orf32
238422_at	2.18		
225611_at	2.17	microtubule associated serine/threonine kinase family member 4	MAST4
236484_at	2.17		
234520_at	2.17	Friend leukemia virus integration 1	FLI1
218819_at	2.17	integrator complex subunit 6	INTS6
205990_s_at	2.17	wingless-type MMTV integration site family, member 5A	WNT5A
211828_s_at	2.16	TRAF2 and NCK interacting kinase	TNIK
222306_at	2.16		
1565563_at	2.16		
204684_at	2.16	neuronal pentraxin I	NPTX1
231320_at	2.16	TBC1 domain family, member 25	TBC1D25
222787_s_at	2.16	transmembrane protein 106B	TMEM106B
212629_s_at	2.16	protein kinase N2	PKN2
1563546_at	2.15		
1562332_at	2.15		
244140_at	2.15	Wolf-Hirschhorn syndrome candidate 1	WHSC1
1557066_at	2.15	LUC7-like (S. cerevisiae)	LUC7L
1562612_at	2.15		
203216_s_at	2.15	myosin VI	MYO6
206830_at	2.15	solute carrier family 4, sodium bicarbonate transporter-like, member 10	SLC4A10
1569856_at	2.15	tripeptidyl peptidase II	TPP2
216860_s_at	2.14	growth differentiation factor 11	GDF11
231925_at	2.14		
222446_s_at	2.14	beta-site APP-cleaving enzyme 2	BACE2
219524_s_at	2.14	chromosome 20 open reading frame 7	C20orf7

205303_at	2.14	potassium inwardly-rectifying channel, subfamily J, member 8	KCNJ8
1560813_at	2.14		
220358_at	2.14		
210986_s_at	2.14	tropomyosin 1 (alpha)	TPM1
218847_at	2.14	insulin-like growth factor 2 mRNA binding protein 2	IGF2BP2
238925_at	2.14	syntrophin, beta 2 (dystrophin-associated protein A1, 59kDa, basic component 2)	SNTB2
220295_x_at	2.13	DEP domain containing 1	DEPDC1
243878_at	2.13		
242232_at	2.13	SLIT-ROBO Rho GTPase activating protein 2	SRGAP2
210281_s_at	2.13	zinc finger, MYM-type 2	ZMYM2
208557_at	2.13	homeobox A6	HOXA6
242903_at	2.13	interferon gamma receptor 1	IFNGR1
206381_at	2.13	sodium channel, voltage-gated, type II, alpha subunit	SCN2A
227740_at	2.13	U2AF homology motif (UHM) kinase 1	UHMK1
242248_at	2.13	phosphorylase kinase, beta	PHKB
238889_at	2.13		
224114_at	2.12		
210001_s_at	2.12	suppressor of cytokine signaling 1	SOCS1
212372_at	2.12	myosin, heavy chain 10, non-muscle	MYH10
239224_at	2.12	F-box and leucine-rich repeat protein 20	FBXL20
236700_at	2.12		
207445_s_at	2.11	chemokine (C-C motif) receptor 9	CCR9
1558251_a_at	2.11	zinc finger protein 587	ZNF587
1552772_at	2.11	C-type lectin domain family 4, member D	CLEC4D
242638_at	2.11		
216063_at	2.11	hemoglobin, beta pseudogene 1	HBBP1
238882_at	2.11		
221558_s_at	2.11	lymphoid enhancer-binding factor 1	LEF1
1556771_a_at	2.10		
229517_at	2.10	protein tyrosine phosphatase domain containing 1	PTPDC1
204716_at	2.10	coiled-coil domain containing 6	CCDC6
1558711_at	2.10	family with sequence similarity 13, member A1 opposite strand	FAM13A1OS
220481_at	2.10	G protein-coupled receptor 75	GPR75
228558_at	2.10	chromosome 14 open reading frame 80	C14orf80
1558501_at	2.10	dynamin 3	DNM3
239788_at	2.10		
205466_s_at	2.10	heparan sulfate (glucosamine) 3-O-sulfotransferase 1	HS3ST1
230850_at	2.10		
234618_at	2.09		
1553972_a_at	2.09	cystathionine-beta-synthase	CBS
228303_at	2.09	UDP-N-acetyl-alpha-D-galactosamine:polypeptide N-acetylgalactosaminyltransferase 6 (GalNAc-T6)	GALNT6
206757_at	2.09	phosphodiesterase 5A, cGMP-specific	PDE5A
241642_x_at	2.09	tousled-like kinase 1	TLK1
210414_at	2.09	fibronectin leucine rich transmembrane protein 1	FLRT1
232029_at	2.09		
206025_s_at	2.09	tumor necrosis factor, alpha-induced protein 6	TNFAIP6
201508_at	2.09	insulin-like growth factor binding protein 4	IGFBP4

205646_s_at	2.09	paired box gene 6 (aniridia, keratitis)	PAX6
1552563_a_at	2.08	chromosome 8 open reading frame 6	C8orf6
1555772_a_at	2.08	cell division cycle 25 homolog A (S. pombe)	CDC25A
1557505_a_at	2.08		
216876_s_at	2.08	interleukin 17A	IL17A
225981_at	2.08	chromosome 17 open reading frame 28	C17orf28
206879_s_at	2.08	neuregulin 2	NRG2
240602_at	2.08	HBS1-like (S. cerevisiae)	HBS1L
235885_at	2.08		
240971_x_at	2.08	cullin 4A	CUL4A
221458_at	2.08	5-hydroxytryptamine (serotonin) receptor 1F	HTR1F
236313_at	2.08	cyclin-dependent kinase inhibitor 2B (p15, inhibits CDK4)	CDKN2B
224822_at	2.08	deleted in liver cancer 1	DLC1
208022_s_at	2.07	CDC14 cell division cycle 14 homolog B (S. cerevisiae)	CDC14B
203280_at	2.07	scaffold attachment factor B2	SAFB2
242285_at	2.07		
216093_at	2.07		
243909_x_at	2.07	glucuronidase, beta-like 2	GUSBL2
243618_s_at	2.07		
202175_at	2.07		
222924_at	2.06		
243589_at	2.06	KIAA1267	KIAA1267
207194_s_at	2.06	intercellular adhesion molecule 4 (Landsteiner-Wiener blood group)	ICAM4
1558387_at	2.06		
239614_x_at	2.06		
210630_s_at	2.06	RAD52 homolog (S. cerevisiae)	RAD52
1553798_a_at	2.06	F-box and leucine-rich repeat protein 13	FBXL13
238224_at	2.06		
213013_at	2.05	mitogen-activated protein kinase 8 interacting protein 1	MAPK8IP1
226610_at	2.05	proline rich 6	PRR6
239127_at	2.05		
1557818_x_at	2.05		
1570125_at	2.05		
236919_at	2.05	chromosome 18 open reading frame 25	C18orf25
206393_at	2.05	troponin I type 2 (skeletal, fast)	TNNI2
231698_at	2.05	UDP-glucose pyrophosphorylase 2	UGP2
238428_at	2.05	potassium inwardly-rectifying channel, subfamily J, member 15	KCNJ15
232461_at	2.04	Abelson helper integration site 1	AHI1
214954_at	2.04	sushi domain containing 5	SUSD5
1558871_at	2.04		
1567287_at	2.04	olfactory receptor, family 5, subfamily K, member 1	OR5K1
236159_x_at	2.04		
236974_at	2.04		
243756_at	2.03	thrombospondin, type I, domain containing 7A	THSD7A
236150_at	2.03		
231247_s_at	2.03		
230024_at	2.03		
223958_s_at	2.03	dynein, axonemal, light chain 1	DNAL1

211555_s_at	2.03	guanylate cyclase 1, soluble, beta 3	GUCY1B3
210517_s_at	2.03	A kinase (PRKA) anchor protein (gravin) 12	AKAP12
243458_at	2.03	tumor necrosis factor, alpha-induced protein 8	TNFAIP8
1554686_at	2.03	staufen, RNA binding protein, homolog 2 (Drosophila)	STAU2
1553789_a_at	2.03	chromosome 21 open reading frame 58	C21orf58
204316_at	2.03	regulator of G-protein signalling 10	RGS10
214716_at	2.02	BMP2 inducible kinase	BMP2K
1563958_at	2.02		
231611_at	2.02		
208129_x_at	2.02	runt-related transcription factor 1 (acute myeloid leukemia 1; aml1 oncogene)	RUNX1
244480_at	2.02		
240468_at	2.02		
232340_at	2.02		
214651_s_at	2.02	homeobox A9	HOXA9
231212_x_at	2.02	tyrosylprotein sulfotransferase 1	TPST1
239456_at	2.02	glucocorticoid receptor DNA binding factor 1	GRLF1
240624_x_at	2.01		
215382_x_at	2.01	tryptase alpha/beta 1	TPSAB1
1555761_x_at	2.01	RNA binding motif protein 15	RBM15
1559469_s_at	2.01	signal-induced proliferation-associated 1 like 2	SIPA1L2
241147_at	2.01		
223279_s_at	2.01	uveal autoantigen with coiled-coil domains and ankyrin repeats	UACA
1561121_at	2.01		
234036_x_at	2.01		
221088_s_at	2.01	protein phosphatase 1, regulatory (inhibitor) subunit 9A	PPP1R9A
1561712_at	2.01		
226461_at	2.00	homeobox B9	HOXB9
200952_s_at	2.00	cyclin D2	CCND2
242467_at	2.00	casein kinase 1, alpha 1	CSNK1A1
242747_at	0.50		
207580_at	0.50	melanoma antigen family B, 4	MAGEB4
1569380_a_at	0.50		
240454_at	0.50		
210889_s_at	0.50	Fc fragment of IgG, low affinity IIb, receptor (CD32)	FCGR2B
243261_at	0.50		
236358_at	0.50		
238207_at	0.50		
226912_at	0.50	zinc finger, DHHC-type containing 23	ZDHHC23
1556620_at	0.50		
214572_s_at	0.50	insulin-like 3 (Leydig cell)	INSL3
213120_at	0.50		
215532_x_at	0.50	zinc finger protein 492	ZNF492
223751_x_at	0.50	toll-like receptor 10	TLR10
230254_at	0.50		
235917_at	0.50		
226365_at	0.50		
231683_at	0.50	glycine-N-acyltransferase	GLYAT
237663_at	0.50		
231110_at	0.49		

213072_at	0.49	cysteine/histidine-rich 1	CYHR1
1558383_at	0.49		
232494_at	0.49	cytochrome P450, family 8, subfamily B, polypeptide 1	CYP8B1
1564963_x_at	0.49	zinc finger protein 92	ZNF92
243370_at	0.49	GPI-anchored membrane protein 1	GPIAP1
207341_at	0.49	proteinase 3 (serine proteinase, neutrophil, Wegener granulomatosis autoantigen)	PRTN3
238564_at	0.49	KIAA1946	KIAA1946
1552515_at	0.49	homeodomain interacting protein kinase 1	HIPK1
1563118_at	0.49		
238539_at	0.49	Hermansky-Pudlak syndrome 3	HPS3
1553134_s_at	0.49	chromosome 9 open reading frame 72	C9orf72
215298_at	0.49		
242703_at	0.49	methylcrotonoyl-Coenzyme A carboxylase 1 (alpha)	MCCC1
1556082_a_at	0.49		
238771_at	0.49	fibrosin 1	FBS1
229518_at	0.49	family with sequence similarity 46, member B	FAM46B
234217_at	0.49		
231296_at	0.49		
207096_at	0.49	serum amyloid A4, constitutive	SAA4
215118_s_at	0.49	immunoglobulin heavy constant alpha 1	IGHA1
1558867_at	0.49	dermatan sulfate epimerase	DSE
203950_s_at	0.49	chloride channel 6	CLCN6
202718_at	0.49	insulin-like growth factor binding protein 2, 36kDa	IGFBP2
209823_x_at	0.49	major histocompatibility complex, class II, DQ beta 1	HLA-DQB1
206668_s_at	0.49	secretory carrier membrane protein 1	SCAMP1
243996_at	0.49		
205100_at	0.49	glutamine-fructose-6-phosphate transaminase 2	GFPT2
216915_s_at	0.49	protein tyrosine phosphatase, non-receptor type 12	PTPN12
1568799_at	0.49		
203864_s_at	0.49	actinin, alpha 2	ACTN2
232026_at	0.49	hect domain and RLD 4	HERC4
233946_at	0.49		
242545_at	0.49	tubulin tyrosine ligase-like family, member 11	TTLL11
202948_at	0.49	interleukin 1 receptor, type I	IL1R1
241360_at	0.49	coiled-coil domain containing 15	CCDC15
206590_x_at	0.49	dopamine receptor D2	DRD2
236490_at	0.49		
221841_s_at	0.49	Kruppel-like factor 4 (gut)	KLF4
1561600_at	0.49		
211172_x_at	0.49	A kinase (PRKA) anchor protein 7	AKAP7
203811_s_at	0.49	DnaJ (Hsp40) homolog, subfamily B, member 4	DNAJB4
237128_at	0.49		
1559624_at	0.49	serine/threonine kinase 32A	STK32A
1569089_a_at	0.49		
223376_s_at	0.49	brain protein I3	BRI3
241517_at	0.49	development and differentiation enhancing factor 1	DDEF1
238332_at	0.49	ankyrin repeat domain 29	ANKRD29
211459_at	0.49		
200866_s_at	0.48	prosaposin (variant Gaucher disease and variant metachromatic leukodystrophy)	PSAP

211106_at	0.48	suppressor of Ty 3 homolog (S. cerevisiae)	SUPT3H
234581_at	0.48		
237032_x_at	0.48	signal-induced proliferation-associated 1 like 1	SIPA1L1
239612_at	0.48		
224813_at	0.48	Wiskott-Aldrich syndrome-like	WASL
214967_at	0.48		
233660_at	0.48	EH-domain containing 4	EHD4
1561673_at	0.48		
213675_at	0.48		
204567_s_at	0.48	ATP-binding cassette, sub-family G (WHITE), member 1	ABCG1
243296_at	0.48	pre-B-cell colony enhancing factor 1	PBEF1
216174_at	0.48		
221023_s_at	0.48	potassium voltage-gated channel, subfamily H (eag-related), member 6	KCNH6
1555319_at	0.48	stabilin 1	STAB1
211557_x_at	0.48	solute carrier organic anion transporter family, member 2B1	SLCO2B1
207205_at	0.48	carcinoembryonic antigen-related cell adhesion molecule 4	CEACAM4
1564306_at	0.48		
1556204_a_at	0.48		
1560369_at	0.48	ankylosis, progressive homolog (mouse)	ANKH
232749_at	0.48		
236465_at	0.48	ring finger protein 175	RNF175
213522_s_at	0.48	solute carrier family 16, member 3 (monocarboxylic acid transporter 4)	SLC16A3
218723_s_at	0.48		
221104_s_at	0.48	nipsnap homolog 3B (C. elegans)	NIPSNAP3B
228596_at	0.48	Rho guanine nucleotide exchange factor (GEF) 5	ARHGEF5
220338_at	0.48	Ral GEF with PH domain and SH3 binding motif 2	RALGPS2
207655_s_at	0.48	B-cell linker	BLNK
236882_at	0.48		
219505_at	0.48	cat eye syndrome chromosome region, candidate 1	CECR1
217503_at	0.48		
243906_at	0.48	phosphate cytidylyltransferase 1, choline, alpha	PCYT1A
228638_at	0.48	family with sequence similarity 76, member A	FAM76A
220173_at	0.48	chromosome 14 open reading frame 45	C14orf45
202150_s_at	0.48	neural precursor cell expressed, developmentally down-regulated 9	NEDD9
236310_at	0.48		
205879_x_at	0.48	ret proto-oncogene (multiple endocrine neoplasia and medullary thyroid carcinoma 1, Hirschsprung dis	RET
207686_s_at	0.48	caspase 8, apoptosis-related cysteine peptidase	CASP8
214272_at	0.48	cylindromatosis (turban tumor syndrome)	CYLD
205552_s_at	0.47	2',5'-oligoadenylate synthetase 1, 40/46kDa	OAS1
1564280_x_at	0.47		
1567628_at	0.47	CD74 molecule, major histocompatibility complex, class II invariant chain	CD74
233261_at	0.47	early B-cell factor 1	EBF1
1554027_a_at	0.47	solute carrier family 4, sodium bicarbonate cotransporter, member 4	SLC4A4

241963_at	0.47	zinc finger protein 704	ZNF704
202270_at	0.47	guanylate binding protein 1, interferon-inducible, 67kDa	GBP1
240077_at	0.47		
212998_x_at	0.47	major histocompatibility complex, class II, DQ beta 1	HLA-DQB1
215172_at	0.47	protein tyrosine phosphatase, non-receptor type 20B#protein tyrosine phosphatase, non-receptor type 20A#protein tyrosine phosphatase, non-receptor type 20A	PTPN20B#PTPN20A#PTPN20A
1558999_x_at	0.47		
212182_at	0.47	nudix (nucleoside diphosphate linked moiety X)-type motif 4	NUDT4
237122_at	0.47		
216568_x_at	0.47	aminolevulinate, delta-, synthase 2 (sideroblastic/hypochromic anemia)	ALAS2
205785_at	0.47	integrin, alpha M (complement component 3 receptor 3 subunit)	ITGAM
240022_at	0.47	chromosome 19 open reading frame 7	C19orf7
232188_at	0.47	A kinase (PRKA) anchor protein 13	AKAP13
1562069_at	0.47		
221185_s_at	0.47	IQ motif containing G	IQCG
1558365_at	0.47		
236777_at	0.47		
1553271_at	0.47	DIP2 disco-interacting protein 2 homolog B (Drosophila)	DIP2B
238780_s_at	0.47		
1561058_at	0.47		
234152_at	0.47		
1556465_at	0.47		
228568_at	0.47		
218368_s_at	0.47	tumor necrosis factor receptor superfamily, member 12A	TNFRSF12A
224235_at	0.47		
223721_s_at	0.47	DnaJ (Hsp40) homolog, subfamily C, member 12	DNAJC12
239441_at	0.47	zinc finger protein 780A	ZNF780A
1552604_at	0.47	chromosome 21 open reading frame 74	C21orf74
231971_at	0.47	Fanconi anemia, complementation group M	FANCM
229992_at	0.47		
233541_at	0.47		
1564039_at	0.47	zinc finger protein 390	ZNF390
228717_at	0.47		
242181_at	0.47		
235555_at	0.47		
220121_at	0.47	lines homolog 1 (Drosophila)	LINS1
223501_at	0.47		
233156_at	0.47	ribonuclease H2, subunit B	RNASEH2B
244576_at	0.47		
234571_at	0.47		
234892_at	0.47		
229362_at	0.47		
1565597_at	0.46		
241735_at	0.46		

239963_at	0.46		
230324_at	0.46	nuclear receptor coactivator 2	NCOA2
226322_at	0.46	transmembrane and tetratricopeptide repeat containing 1	TMTC1
211884_s_at	0.46	class II, major histocompatibility complex, transactivator	CIITA
1552734_at	0.46		
235673_at	0.46		
212963_at	0.46	TM2 domain containing 1	TM2D1
232158_x_at	0.46	NIPA-like domain containing 1	NPAL1
227140_at	0.46		
216002_at	0.46		
239936_at	0.46	deleted in lymphocytic leukemia, 2	DLEU2
205247_at	0.46	Notch homolog 4 (Drosophila)	NOTCH4
238931_at	0.46	zinc finger and SCAN domain containing 22	ZSCAN22
242270_at	0.46		
242959_at	0.46	KIAA1239	KIAA1239
243315_at	0.46		
206910_x_at	0.46	complement factor H-related 2	CFHR2
1558648_at	0.46	coiled-coil domain containing 32	CCDC32
222463_s_at	0.46	beta-site APP-cleaving enzyme 1	BACE1
224546_at	0.46		
224067_at	0.46		
217445_s_at	0.46	phosphoribosylglycinamide formyltransferase, phosphoribosylglycinamide synthetase, phosphoribosylami	GART
1561604_at	0.46		
207920_x_at	0.46	zinc finger protein, X-linked	ZFX
217420_s_at	0.46	polymerase (RNA) II (DNA directed) polypeptide A, 220kDa	POLR2A
214240_at	0.46	galanin	GAL
235687_at	0.46	zinc finger protein 626	ZNF626
208389_s_at	0.46	solute carrier family 1 (glial high affinity glutamate transporter), member 2	SLC1A2
235412_at	0.46	Rho guanine nucleotide exchange factor (GEF) 7	ARHGEF7
234476_at	0.46	dynein, axonemal, heavy chain 7	DNAH7
238581_at	0.46	guanylate binding protein 5	GBP5
231252_at	0.46		
219834_at	0.46	amyotrophic lateral sclerosis 2 (juvenile) chromosome region, candidate 8	ALS2CR8
242057_at	0.46		
220471_s_at	0.46	myc target 1	MYCT1
241879_at	0.46		
223332_x_at	0.46	ring finger protein 126	RNF126
1561760_s_at	0.46		
221874_at	0.46	KIAA1324	KIAA1324
1553060_at	0.45	protein serine kinase H2	PSKH2
243707_at	0.45		
239687_at	0.45	BTB (POZ) domain containing 12	BTBD12
243294_at	0.45	zinc finger protein 780B	ZNF780B
242474_s_at	0.45		
1556338_at	0.45		
205299_s_at	0.45	butyrophilin, subfamily 2, member A2	BTN2A2

1570163_at	0.45		
238881_at	0.45		
1556272_a_at	0.45		
1554676_at	0.45	proteoglycan 1, secretory granule	PRG1
219383_at	0.45		
216045_at	0.45		
209802_at	0.45	pleckstrin homology-like domain, family A, member 2	PHLDA2
1561155_at	0.45		
1558519_at	0.45		
215463_at	0.45	olfactory receptor, family 7, subfamily E, member 24	OR7E24
1556697_at	0.45	GPRIN family member 3	GPRIN3
1557311_at	0.45		
211329_x_at	0.45	hemochromatosis	HFE
239144_at	0.45	beta-1,3-glucuronyltransferase 2 (glucuronosyltransferase S)	B3GAT2
213697_at	0.45	homeodomain interacting protein kinase 3	HIPK3
216537_s_at	0.45	sialic acid binding Ig-like lectin 7	SIGLEC7
1553452_at	0.45	myosin IH	MYO1H
207689_at	0.45	T-box 10	TBX10
237952_at	0.45	cAMP responsive element binding protein 3-like 2	CREB3L2
218362_s_at	0.45	DIS3 mitotic control homolog (S. cerevisiae)	DIS3
226425_at	0.45	CAP-GLY domain containing linker protein family, member 4	CLIP4
210412_at	0.45	glutamate receptor, ionotropic, N-methyl D-aspartate 2B	GRIN2B
217637_at	0.45		
243851_at	0.45	RAB3 GTPase activating protein subunit 2 (non-catalytic)	RAB3GAP2
1569753_at	0.45		
235453_at	0.45		
208033_s_at	0.45	AT-binding transcription factor 1	ATBF1
213849_s_at	0.45	protein phosphatase 2 (formerly 2A), regulatory subunit B, beta isoform	PPP2R2B
222772_at	0.45	myelin expression factor 2	MYEF2
206563_s_at	0.45	opiate receptor-like 1	OPRL1
232579_at	0.45		
1566695_at	0.45		
221142_s_at	0.45	peroxisomal trans-2-enoyl-CoA reductase	PECR
220618_s_at	0.45	zinc finger, CW type with PWWP domain 1	ZCWPW1
235685_at	0.45		
238342_at	0.45		
214720_x_at	0.44	septin 10	10-Sep
1556730_at	0.44		
242188_at	0.44	protein tyrosine phosphatase, receptor type, G	PTPRG
243196_s_at	0.44	TRAF-type zinc finger domain containing 1	TRAFD1
1559222_at	0.44		
1554539_a_at	0.44	ras homolog gene family, member F (in filopodia)	RHOF
1565254_s_at	0.44	myeloid/lymphoid or mixed-lineage leukemia (trithorax homolog, Drosophila)	MLL
223918_at	0.44	acyl-CoA synthetase long-chain family member 6	ACSL6
1556527_a_at	0.44		

1556339_a_at	0.44		
233927_at	0.44		
1566656_a_at	0.44		
1553792_at	0.44	KIAA1109	KIAA1109
230333_at	0.44		
1555681_at	0.44		
222305_at	0.44	hexokinase 2	HK2
242728_at	0.44		
1565801_at	0.44		
215602_at	0.44	FYVE, RhoGEF and PH domain containing 2	FGD2
223835_x_at	0.44	orthopedia homolog (Drosophila)	OTP
216712_at	0.44	transmembrane protein 132A	TMEM132A
216411_s_at	0.44	galactokinase 2	GALK2
208868_s_at	0.44	GABA(A) receptor-associated protein like 1	GABARAPL1
210029_at	0.44	indoleamine-pyrrole 2,3 dioxygenase	INDO
214205_x_at	0.44	thioredoxin-like 2	TXNL2
216798_at	0.44	ribonuclease/angiogenin inhibitor 1#null	RNH1#null
205403_at	0.44	interleukin 1 receptor, type II	IL1R2
221643_s_at	0.44	arginine-glutamic acid dipeptide (RE) repeats	RERE
228791_at	0.44	chromosome 15 open reading frame 38	C15orf38
209289_at	0.44		
237571_at	0.44		
220494_s_at	0.44		
208404_x_at	0.44	potassium inwardly-rectifying channel, subfamily J, member 5	KCNJ5
1565228_s_at	0.44	albumin	ALB
229219_s_at	0.44	N-terminal asparagine amidase	NTAN1
216628_at	0.44		
244317_at	0.44	KIAA1324-like	KIAA1324L
202953_at	0.44	complement component 1, q subcomponent, B chain	C1QB
237183_at	0.44	UDP-N-acetyl-alpha-D-galactosamine:polypeptide N-acetylgalactosaminyltransferase 5 (GalNAc-T5)	GALNT5
1554508_at	0.44	phosphoinositide-3-kinase adaptor protein 1	PIK3AP1
240906_at	0.44	mitochondrial ribosomal protein S36	MRPS36
235218_x_at	0.44	THAP domain containing 6	THAP6
220464_at	0.44	MCF.2 cell line derived transforming sequence-like	MCF2L
222542_x_at	0.43	chaperone, ABC1 activity of bc1 complex homolog (S. pombe)	CABC1
235710_at	0.43	vacuolar protein sorting 53 homolog (S. cerevisiae)	VPS53
1555734_x_at	0.43	adaptor-related protein complex 1, sigma 3 subunit	AP1S3
1563465_at	0.43	polycystic kidney disease 1 like 1	PKD1L1
233321_x_at	0.43		
210992_x_at	0.43		
1559950_at	0.43		
1569934_at	0.43	DEP domain containing 2	DEPDC2
1552425_a_at	0.43	kelch-like 10 (Drosophila)	KLHL10
234174_at	0.43		
243220_at	0.43		
207283_at	0.43		
1560080_at	0.43	diaphanous homolog 1 (Drosophila)	DIAPH1
203854_at	0.43	complement factor I	CFI
242003_at	0.43	glutamate-rich 1	ERICH1

232523_at	0.43	multiple EGF-like-domains 10	MEGF10
215484_at	0.43		
241321_at	0.43	ankyrin repeat domain 23	ANKRD23
1555429_at	0.43		
230933_at	0.43	destrin (actin depolymerizing factor)	DSTN
244150_at	0.43	structural maintenance of chromosomes 1A	SMC1A
205501_at	0.43		
222802_at	0.43	endothelin 1	EDN1
1554453_at	0.43	heterogeneous nuclear ribonucleoprotein L-like	HNRPLL
235330_at	0.43	coiled-coil domain containing 117	CCDC117
241732_at	0.43	CDK5 regulatory subunit associated protein 1-like 1	CDKAL1
207001_x_at	0.43	TSC22 domain family, member 3	TSC22D3
1558670_at	0.43		
230466_s_at	0.43		
1561238_at	0.43	peroxisomal membrane protein 3, 35kDa (Zellweger syndrome)	PXMP3
236660_at	0.43		
242726_at	0.43		
228610_at	0.43	transmembrane 9 superfamily member 3	TM9SF3
1568673_s_at	0.43	ELL associated factor 2	EAF2
235163_at	0.42	MOB1, Mps One Binder kinase activator-like 2A (yeast)	MOBKL2A
244219_at	0.42		
1563884_at	0.42		
243042_at	0.42	family with sequence similarity 73, member A	FAM73A
228103_s_at	0.42	neuropilin 2	NRP2
206331_at	0.42	calcitonin receptor-like	CALCRL
233053_at	0.42		
239306_at	0.42		
240536_at	0.42		
232723_at	0.42		
241074_at	0.42	chromosome 12 open reading frame 32	C12orf32
207861_at	0.42	chemokine (C-C motif) ligand 22	CCL22
222245_s_at	0.42	fer-1-like 4 (C. elegans)	FER1L4
222720_x_at	0.42	chromosome 1 open reading frame 27	C1orf27
241188_at	0.42		
234094_x_at	0.42		
208027_s_at	0.42	tolloid-like 2	TLL2
233137_at	0.42		
234277_at	0.42	epidermal growth factor receptor pathway substrate 15	EPS15
1555898_at	0.42		
232480_at	0.42		
206818_s_at	0.42	cyclin M2	CNNM2
227862_at	0.42		
235285_at	0.42		
244730_x_at	0.42		
1552408_at	0.42	outer dense fiber of sperm tails 4	ODF4
1561289_at	0.42		
1556350_a_at	0.42	eukaryotic translation initiation factor 4A, isoform 2	EIF4A2
240495_at	0.42	A kinase (PRKA) anchor protein 1	AKAP1
1568874_at	0.42	nuclear receptor coactivator 6	NCOA6
222566_at	0.42		

235565_at	0.42	zinc finger protein 425	ZNF425
225016_at	0.42	adenomatosis polyposis coli down-regulated 1	APCDD1
211096_at	0.42	pre-B-cell leukemia homeobox 2	PBX2
214070_s_at	0.42	ATPase, Class V, type 10B	ATP10B
217353_at	0.42	phenylalanine-tRNA synthetase 2 (mitochondrial)	FARS2
219941_at	0.42	transmembrane protein 19	TMEM19
1554757_a_at	0.42	inositol polyphosphate-5-phosphatase, 40kDa	INPP5A
203134_at	0.42	phosphatidylinositol binding clathrin assembly protein	PICALM
213506_at	0.42	coagulation factor II (thrombin) receptor-like 1	F2RL1
237675_at	0.42		
1557418_at	0.41	acyl-CoA synthetase long-chain family member 4	ACSL4
232665_x_at	0.41	SMAD specific E3 ubiquitin protein ligase 1	SMURF1
220877_at	0.41		
1552919_at	0.41	chromosome 4 open reading frame 36	C4orf36
203757_s_at	0.41	carcinoembryonic antigen-related cell adhesion molecule 6 (non-specific cross reacting antigen)	CEACAM6
1555000_at	0.41		
229690_at	0.41	family with sequence similarity 109, member A	FAM109A
223881_at	0.41	islet cell autoantigen 1,69kDa-like	ICA1L
1556898_at	0.41		
203457_at	0.41	syntaxin 7	STX7
231756_at	0.41	zona pellucida glycoprotein 4	ZP4
1567079_at	0.41	ceroid-lipofuscinosis, neuronal 6, late infantile, variant	CLN6
239914_at	0.41		
1564238_a_at	0.41	WD repeat domain 49	WDR49
1556426_at	0.41	hexosaminidase A (alpha polypeptide)	HEXA
217696_at	0.41	fucosyltransferase 7 (alpha (1,3) fucosyltransferase)	FUT7
1557398_at	0.41		
237370_at	0.41		
1554559_at	0.41	G protein-coupled receptor 62	GPR62
233057_at	0.41		
217714_x_at	0.41	stathmin 1/oncoprotein 18	STMN1
236035_at	0.41		
43934_at	0.41	G protein-coupled receptor 137	GPR137
1559713_at	0.41		
210663_s_at	0.41	kynureninase (L-kynurenine hydrolase)	KYNU
219671_at	0.41	hippocalcin like 4	HPCAL4
1554575_a_at	0.40	3'(2'), 5'-bisphosphate nucleotidase 1	BPNT1
216446_at	0.40		
244329_at	0.40		
206921_at	0.40	GLE1 RNA export mediator-like (yeast)	GLE1L
207665_at	0.40	ADAM metallopeptidase domain 21	ADAM21
239560_at	0.40		
1554710_at	0.40	potassium large conductance calcium-activated channel, subfamily M, beta member 1	KCNMB1
1566108_at	0.40	myoneurin	MYNN
244334_at	0.40	translocation associated membrane protein 1-like 1	TRAM1L1
224340_at	0.40		
1569827_at	0.40	ATG7 autophagy related 7 homolog (S. cerevisiae)	ATG7
1559393_at	0.40	aldehyde dehydrogenase 1 family, member L2	ALDH1L2

229349_at	0.40	lin-28 homolog B (C. elegans)	LIN28B
243547_at	0.40		
221171_at	0.40		
241941_at	0.40		
226918_at	0.40	junctophilin 4	JPH4
226388_at	0.40	transcription elongation factor A (SII), 3	TCEA3
1555034_at	0.40	clarin 1	CLRN1
1558756_at	0.40		
239283_at	0.40	transmembrane emp24 protein transport domain containing 5	TMED5
237136_at	0.40		
234123_at	0.40		
203850_s_at	0.40	kinesin family member 1A	KIF1A
211305_x_at	0.40	Fc fragment of IgA, receptor for	FCAR
211842_s_at	0.40	solute carrier family 24 (sodium/potassium/calcium exchanger), member 1	SLC24A1
220692_at	0.40		
1556508_s_at	0.40		
216627_s_at	0.40	UDP-Gal:betaGlcNAc beta 1,4- galactosyltransferase, polypeptide 1	B4GALT1
231653_at	0.40	coiled-coil domain containing 129	CCDC129
207187_at	0.40	Janus kinase 3 (a protein tyrosine kinase, leukocyte)	JAK3
242445_at	0.40	FYVE, RhoGEF and PH domain containing 4	FGD4
215928_at	0.40		
1561531_at	0.40		
1561451_a_at	0.40		
217130_at	0.40	chromosome 9 open reading frame 33	C9orf33
220399_at	0.40		
239550_at	0.40	RAR-related orphan receptor A	RORA
240191_at	0.40	zinc finger protein 543	ZNF543
205404_at	0.39	hydroxysteroid (11-beta) dehydrogenase 1	HSD11B1
240338_at	0.39		
244030_at	0.39	serine/threonine/tyrosine interacting protein	STYX
244041_at	0.39		
235212_at	0.39	chromosome 14 open reading frame 102	C14orf102
235150_at	0.39		
240114_s_at	0.39	transmembrane protein 174	TMEM174
1561908_a_at	0.39		
237016_at	0.39	chromosome 6 open reading frame 128	C6orf128
230652_at	0.39	v-raf murine sarcoma 3611 viral oncogene homolog	ARAF
1563187_at	0.39		
1559634_at	0.39	cholinergic receptor, muscarinic 3	CHRM3
212464_s_at	0.39	fibronectin 1	FN1
229415_at	0.39	cytochrome c, somatic	CYCS
205729_at	0.39	oncostatin M receptor	OSMR
235442_at	0.39	chromosome X open reading frame 56	CXorf56
208460_at	0.39	gap junction protein, alpha 7, 45kDa	GJA7
242205_at	0.39		
227648_at	0.39	chromosome 22 open reading frame 32	C22orf32
1569128_at	0.39		
241500_at	0.39		
231981_at	0.39	prolactin receptor	PRLR

232905_at	0.39		
242491_at	0.39		
210077_s_at	0.39	splicing factor, arginine/serine-rich 5	SFRS5
234210_x_at	0.39	ARP2 actin-related protein 2 homolog (yeast)	ACTR2
238159_at	0.39	GATA zinc finger domain containing 2B	GATAD2B
209951_s_at	0.39	mitogen-activated protein kinase kinase 7	MAP2K7
1558905_at	0.39		
225987_at	0.39	STEAP family member 4	STEAP4
1566696_at	0.39		
211171_s_at	0.38	phosphodiesterase 10A	PDE10A
233316_at	0.38		
207207_at	0.38		
1570022_at	0.38		
1564640_at	0.38	MAX gene associated	MGA
221294_at	0.38	G protein-coupled receptor 21	GPR21
227034_at	0.38	ankyrin repeat domain 57	ANKRD57
211805_s_at	0.38	solute carrier family 8 (sodium/calcium exchanger), member 1	SLC8A1
214647_s_at	0.38	hemochromatosis	HFE
231710_at	0.38	calcyphosine	CAPS
241634_at	0.38		
241094_at	0.38		
1562988_at	0.38		
230505_at	0.38		
1566607_at	0.38		
214106_s_at	0.38	GDP-mannose 4,6-dehydratase	GMDS
206189_at	0.38	unc-5 homolog C (C. elegans)	UNC5C
1555431_a_at	0.38	interleukin 31 receptor A	IL31RA
207972_at	0.38	glycine receptor, alpha 1 (startle disease/hyperekplexia, stiff man syndrome)	GLRA1
219413_at	0.38	acyl-Coenzyme A binding domain containing 4	ACBD4
209598_at	0.38	paraneoplastic antigen MA2	PNMA2
229546_at	0.38		
239364_at	0.38	ets variant gene 6 (TEL oncogene)	ETV6
214081_at	0.38	plexin domain containing 1	PLXDC1
241898_at	0.38		
236400_at	0.38		
1569003_at	0.38	transmembrane protein 49	TMEM49
1556555_at	0.37		
216850_at	0.37	small nuclear ribonucleoprotein polypeptide N	SNRPN
213590_at	0.37	solute carrier family 16, member 5 (monocarboxylic acid transporter 6)	SLC16A5
228956_at	0.37	UDP glycosyltransferase 8 (UDP-galactose ceramide galactosyltransferase)	UGT8
228431_at	0.37		
222676_at	0.37	BAI1-associated protein 2-like 1	BAIAP2L1
234487_at	0.37		
233915_at	0.37		
241280_at	0.37	aldolase B, fructose-bisphosphate	ALDOB
243515_at	0.37		
1558603_at	0.37	plasminogen-like B2	PLGLB2
235629_at	0.37		
216040_x_at	0.37		

240319_at	0.37		
228836_at	0.37	solute carrier family 25, member 35	SLC25A35
37566_at	0.37	KIAA1045	KIAA1045
233218_at	0.37		
221085_at	0.37	tumor necrosis factor (ligand) superfamily, member 15	TNFSF15
212497_at	0.37	chromosome 14 open reading frame 32	C14orf32
229495_at	0.37	aminoacylase 1-like 2	ACY1L2
228715_at	0.37	zinc finger, CCHC domain containing 12	ZCCHC12
207901_at	0.37	interleukin 12B (natural killer cell stimulatory factor 2, cytotoxic lymphocyte maturation factor 2,	IL12B
1564231_at	0.37	intraflagellar transport 80 homolog (Chlamydomonas)	IFT80
1562537_at	0.37	Fc fragment of IgE, high affinity I, receptor for; alpha polypeptide	FCER1A
1560282_at	0.37		
1567023_at	0.37	olfactory receptor, family 5, subfamily AK, member 4 pseudogene	OR5AK4P
227376_at	0.37	GLI-Kruppel family member GLI3 (Greig cephalopolysyndactyly syndrome)	GLI3
214569_at	0.37	interferon, alpha 5	IFNA5
235251_at	0.37		
235636_at	0.37		
203548_s_at	0.37	lipoprotein lipase	LPL
221372_s_at	0.37	purinergic receptor P2X, ligand-gated ion channel, 2	P2RX2
211516_at	0.37	interleukin 5 receptor, alpha	IL5RA
231193_s_at	0.37		
202768_at	0.37	FBJ murine osteosarcoma viral oncogene homolog B	FOSB
1559826_a_at	0.37		
232263_at	0.37	solute carrier family 6, member 15	SLC6A15
225999_at	0.37	family with sequence similarity 80, member B	FAM80B
240085_at	0.37		
1561726_s_at	0.37		
1557589_a_at	0.36		
204972_at	0.36	2'-5'-oligoadenylate synthetase 2, 69/71kDa	OAS2
238272_at	0.36	thiopurine S-methyltransferase	TPMT
242299_at	0.36		
205328_at	0.36	claudin 10	CLDN10
223641_at	0.36	melanoma inhibitory activity family, member 3	MIA3
239533_at	0.36	G protein-coupled receptor 155	GPR155
1555364_at	0.36		
1561867_at	0.36		
233066_at	0.36		
1566700_at	0.36	vaccinia related kinase 3	VRK3
1554295_x_at	0.36	tau tubulin kinase 2	TTBK2
1559376_at	0.36	chromosome 1 open reading frame 203	C1orf203
1566294_at	0.36		
244710_at	0.36	leucine-rich repeats and guanylate kinase domain containing	LRGUK
227088_at	0.36	phosphodiesterase 5A, cGMP-specific	PDE5A
1568812_at	0.36		

240948_at	0.36		
233239_at	0.36		
1569624_at	0.36		
214775_at	0.36		
244134_at	0.36	3-oxoacid CoA transferase 1	OXCT1
231116_at	0.36		
1557190_at	0.36		
219685_at	0.36	transmembrane protein 35	TMEM35
230718_at	0.36	heat shock transcription factor family member 5	HSF5
215871_at	0.36	phospholipase A2, group V#phospholipase A2, group IID#phospholipase A2, group IIF	PLA2G5#PLA2G2D#PLA2G2F
217376_at	0.36	signal-regulatory protein gamma	SIRPG
233351_at	0.36		
241523_at	0.35		
221405_at	0.35		
1552991_at	0.35	olfactory receptor, family 5, subfamily P, member 2	OR5P2
233911_s_at	0.35	protein phosphatase 1H (PP2C domain containing)	PPM1H
215300_s_at	0.35	flavin containing monooxygenase 5	FMO5
201427_s_at	0.35	selenoprotein P, plasma, 1	SEPP1
220900_at	0.35		
233657_at	0.35	opsin 5	OPN5
240728_at	0.35	phospholipase C, beta 4	PLCB4
233234_at	0.35	potassium channel tetramerisation domain containing 16	KCTD16
207175_at	0.35	adiponectin, C1Q and collagen domain containing	ADIPOQ
235704_at	0.35	DAZ associated protein 2	DAZAP2
217245_at	0.35		
243390_at	0.35	transient receptor potential cation channel, subfamily M, member 3	TRPM3
240006_at	0.35		
232321_at	0.35	mucin 17, cell surface associated	MUC17
1563166_at	0.35		
211599_x_at	0.35	met proto-oncogene (hepatocyte growth factor receptor)	MET
1554413_s_at	0.35	RUN domain containing 2B	RUNDC2B
220266_s_at	0.35	Kruppel-like factor 4 (gut)	KLF4
233838_at	0.34		
213488_at	0.34	sushi, nidogen and EGF-like domains 1	SNED1
1556110_at	0.34		
233455_at	0.34		
238264_at	0.34	NMD3 homolog (S. cerevisiae)	NMD3
244269_at	0.34		
240820_at	0.34		
219845_at	0.34	BarH-like homeobox 1	BARX1
202286_s_at	0.34	tumor-associated calcium signal transducer 2	TACSTD2
237057_at	0.34		
227660_at	0.34	anthrax toxin receptor 1	ANTXR1
1569403_at	0.34		
207912_s_at	0.34	deleted in azoospermia 1	DAZ1
207229_at	0.34	killer cell lectin-like receptor subfamily A, member 1	KLRA1
233320_at	0.34	testicular cell adhesion molecule 1 homolog (mouse)	TCAM1

241149_at	0.34		
230503_at	0.34	sterile alpha motif domain containing 4A	SAMD4A
1569917_at	0.34		
1555401_at	0.34	spermatogenesis and oogenesis specific basic helix-loop-helix 2	SOHLH2
216084_at	0.34	chromosome 9 open reading frame 144	C9orf144
243635_at	0.34		
1556808_at	0.34		
1554305_at	0.34		
1553787_at	0.34	chromosome 11 open reading frame 45	C11orf45
224024_at	0.34	endoplasmic reticulum-golgi intermediate compartment (ERGIC) 1	ERGIC1
204856_at	0.34	UDP-GlcNAc:betaGal beta-1,3-N-acetylglucosaminyltransferase 3	B3GNT3
220067_at	0.34	spectrin, beta, non-erythrocytic 5	SPTBN5
222783_s_at	0.33	SPARC related modular calcium binding 1	SMOC1
236005_at	0.33	Kruppel-like factor 12	KLF12
1554175_at	0.33	CD300 molecule-like family member b	CD300LB
241016_at	0.33	cullin 3	CUL3
209583_s_at	0.33	CD200 molecule	CD200
209727_at	0.33	GM2 ganglioside activator	GM2A
218850_s_at	0.33	LIM domains containing 1	LIMD1
219950_s_at	0.33	T-cell lymphoma invasion and metastasis 2	TIAM2
1555471_a_at	0.33	formin 2	FMN2
236740_at	0.33		
209396_s_at	0.33	chitinase 3-like 1 (cartilage glycoprotein-39)	CHI3L1
234127_at	0.33		
1568805_at	0.33	nuclear receptor coactivator 7	NCOA7
220773_s_at	0.33	gephyrin	GPHN
1557519_at	0.33		
239759_at	0.32		
216837_at	0.32	EPH receptor A5	EPHA5
226069_at	0.32	prickle homolog 1 (Drosophila)	PRICKLE1
234168_at	0.32	TAF15 RNA polymerase II, TATA box binding protein (TBP)-associated factor, 68kDa	TAF15
238753_at	0.32	frequenin homolog (Drosophila)	FREQ
210748_at	0.32	DnaJ (Hsp40) homolog, subfamily C, member 8	DNAJC8
1552540_s_at	0.32	IQ motif containing D	IQCD
207826_s_at	0.32	inhibitor of DNA binding 3, dominant negative helix-loop-helix protein	ID3
202289_s_at	0.32	transforming, acidic coiled-coil containing protein 2	TACC2
1556362_at	0.32		
1553296_at	0.32	G protein-coupled receptor 128	GPR128
242634_at	0.32	GATA zinc finger domain containing 1	GATAD1
210479_s_at	0.32	RAR-related orphan receptor A	RORA
240101_at	0.32		
1557782_s_at	0.32	chromosome 21 open reading frame 49	C21orf49
241007_at	0.32		
219429_at	0.32	fatty acid 2-hydroxylase	FA2H
243493_at	0.32		
1552715_a_at	0.32	relaxin/insulin-like family peptide receptor 1	RXFP1
242654_at	0.32	Fanconi anemia, complementation group C	FANCC
220572_at	0.32		

229823_at	0.32		
240492_at	0.32		
231666_at	0.31	paired box gene 3 (Waardenburg syndrome 1)	PAX3
216695_s_at	0.31	tankyrase, TRF1-interacting ankyrin-related ADP-ribose polymerase	TNKS
208272_at	0.31	RAN binding protein 3	RANBP3
212957_s_at	0.31		
203549_s_at	0.31	lipoprotein lipase	LPL
217474_at	0.31		
1562937_at	0.31		
215101_s_at	0.31	chemokine (C-X-C motif) ligand 5	CXCL5
239593_at	0.31		
1561232_at	0.31		
1559252_a_at	0.31	chromosome 20 open reading frame 29	C20orf29
221397_at	0.31	taste receptor, type 2, member 10	TAS2R10
1562722_at	0.31		
237602_at	0.31		
234573_at	0.31		
1560630_at	0.31		
237905_at	0.31	keratin 25	KRT25
1570490_at	0.31		
244475_at	0.31		
1569439_at	0.31		
1556156_at	0.31	estrogen-related receptor beta	ESRRB
240405_at	0.31		
1562953_s_at	0.31	chromosome 4 open reading frame 12	C4orf12
201905_s_at	0.31	CTD (carboxy-terminal domain, RNA polymerase II, polypeptide A) small phosphatase-like	CTDSPL
233207_at	0.31	disrupted in schizophrenia 1	DISC1
241060_x_at	0.30	tetraspanin 5	TSPAN5
235156_at	0.30		
1560370_x_at	0.30	ankylosis, progressive homolog (mouse)	ANKH
1554672_at	0.30	tetratricopeptide repeat domain 26	TTC26
1555560_at	0.30	UDP-glucose ceramide glucosyltransferase-like 2	UGCGL2
242541_at	0.30	ATP-binding cassette, sub-family A (ABC1), member 9	ABCA9
233391_at	0.30	cadherin-like 26	CDH26
1561362_at	0.30		
227823_at	0.30	retrotransposon gag domain containing 4	RGAG4
1556175_at	0.30		
242102_at	0.30		
243990_at	0.30		
237320_at	0.30		
222299_x_at	0.30		
219962_at	0.30	angiotensin I converting enzyme (peptidyl-dipeptidase A) 2	ACE2
1552564_at	0.30	nudix (nucleoside diphosphate linked moiety X)-type motif 9 pseudogene 1	NUDT9P1
1562263_at	0.30	lysyl oxidase-like 2	LOXL2
240750_at	0.30	chromosome 7 open reading frame 44	C7orf44
211017_s_at	0.30	neurofibromin 2 (bilateral acoustic neuroma)	NF2
221131_at	0.30	alpha-1,4-N-acetylglucosaminyltransferase	A4GNT
1566860_at	0.30		

205719_s_at	0.30	phenylalanine hydroxylase	PAH
1568920_at	0.30		
1558452_at	0.30	transmembrane protein 144	TMEM144
214837_at	0.30	albumin	ALB
211756_at	0.30	parathyroid hormone-like hormone	PTHLH
234472_at	0.30	UDP-N-acetyl-alpha-D-galactosamine:polypeptide N-acetylgalactosaminyltransferase 13 (GalNAc-T13)	GALNT13
1569858_at	0.30		
242599_at	0.29	WW domain containing oxidoreductase	WWOX
243449_at	0.29	oxysterol binding protein 2	OSBP2
1569780_at	0.29		
238368_at	0.29		
1558336_at	0.29		
1553654_at	0.29	synaptotagmin XIV	SYT14
211055_s_at	0.29	inversin	INVS
1556019_at	0.29		
240334_at	0.29	leucine rich repeat and fibronectin type III domain containing 5	LRFN5
240661_at	0.29		
221304_at	0.29	UDP glucuronosyltransferase 1 family, polypeptide A8	UGT1A8
205772_s_at	0.29	A kinase (PRKA) anchor protein 7	AKAP7
217120_s_at	0.29	cofactor required for Sp1 transcriptional activation, subunit 2, 150kDa	CRSP2
239945_at	0.29		
1562918_at	0.29		
1556081_at	0.29		
233327_at	0.29	chromosome 6 open reading frame 157	C6orf157
237667_at	0.29		
209863_s_at	0.29	tumor protein p73-like	TP73L
1554474_a_at	0.29	monooxygenase, DBH-like 1	MOXD1
230507_at	0.29	ataxin 1	ATXN1
242322_at	0.29		
242426_at	0.29	neuregulin 4	NRG4
1556938_a_at	0.29	dynein, light chain, LC8-type 1	DYNLL1
211196_at	0.29	dihydrolipoamide branched chain transacylase E2	DBT
238111_at	0.29	serologically defined colon cancer antigen 3	SDCCAG3
1554391_at	0.29	TANK-binding kinase 1	TBK1
1558356_at	0.29	uveal autoantigen with coiled-coil domains and ankyrin repeats	UACA
1557233_at	0.29		
216441_at	0.29		
1564131_a_at	0.29		
243185_at	0.28		
220440_at	0.28	lectin, galactoside-binding, soluble, 13 (galectin 13)	LGALS13
207596_at	0.28		
233434_at	0.28		
221419_s_at	0.28		
238081_at	0.28		
214453_s_at	0.28	interferon-induced protein 44	IFI44
225299_at	0.28	myosin VB	MYO5B
214079_at	0.28	dehydrogenase/reductase (SDR family) member 2	DHRS2

1565806_at	0.28		
238262_at	0.28	speedy homolog A (Drosophila)	SPDYA
1564392_at	0.28	chromosome 21 open reading frame 131	C21orf131
241648_at	0.28	intersectin 2	ITSN2
233821_at	0.28	RAB32, member RAS oncogene family	RAB32
243161_x_at	0.28	zinc finger protein 42 homolog (mouse)	ZFP42
240660_at	0.28		
220311_at	0.28	N-6 adenine-specific DNA methyltransferase 1 (putative)	N6AMT1
1569783_at	0.28		
215282_at	0.28	anaphase promoting complex subunit 13	ANAPC13
217004_s_at	0.28	MCF.2 cell line derived transforming sequence	MCF2
236570_at	0.28	zinc finger protein 366	ZNF366
233222_at	0.28		
243193_at	0.28	glypican 3	GPC3
241069_at	0.28	zinc finger and BTB domain containing 16	ZBTB16
212353_at	0.27	sulfatase 1	SULF1
201213_at	0.27	protein phosphatase 1, regulatory (inhibitor) subunit 7	PPP1R7
214341_at	0.27	adaptor-related protein complex 1, gamma 2 subunit	AP1G2
231026_at	0.27	EF-hand domain (C-terminal) containing 1	EFHC1
227115_at	0.27		
213562_s_at	0.27	squalene epoxidase	SQLE
1554791_a_at	0.27		
214064_at	0.27	transferrin	TF
237069_s_at	0.27	transient receptor potential cation channel, subfamily M, member 1	TRPM1
242930_at	0.27	O-sialoglycoprotein endopeptidase	OSGEP
1566740_at	0.27	phospholipase C, epsilon 1	PLCE1
228074_at	0.27		
241552_at	0.27		
212865_s_at	0.27	collagen, type XIV, alpha 1 (undulin)	COL14A1
211267_at	0.27	homeobox, ES cell expressed 1	HESX1
242880_at	0.27	voltage gated channel like 1	VGCNL1
242228_at	0.27		
228376_at	0.27	glycoprotein, alpha-galactosyltransferase 1	GGTA1
242901_at	0.27		
1560684_x_at	0.27	B-cell CLL/lymphoma 8	BCL8
234194_at	0.27		
233118_at	0.27		
244097_at	0.27	complement component (3d/Epstein Barr virus) receptor 2	CR2
244091_at	0.27		
238503_at	0.27		
1557211_a_at	0.27	chromosome 14 open reading frame 86	C14orf86
203874_s_at	0.26	SWI/SNF related, matrix associated, actin dependent regulator of chromatin, subfamily a, member 1	SMARCA1
206785_s_at	0.26	killer cell lectin-like receptor subfamily C, member 2	KLRC2
206101_at	0.26	extracellular matrix protein 2, female organ and adipocyte specific	ECM2
37802_r_at	0.26	family with sequence similarity 63, member B	FAM63B

1557821_at	0.26		
217607_x_at	0.26	eukaryotic translation initiation factor 4 gamma, 2	EIF4G2
215756_at	0.26		
219701_at	0.26	tropomodulin 2 (neuronal)	TMOD2
237819_at	0.26	cAMP responsive element binding protein 3-like 2	CREB3L2
224997_x_at	0.26	H19, imprinted maternally expressed untranslated mRNA	H19
220639_at	0.26	transmembrane 4 L six family member 20	TM4SF20
214516_at	0.26	histone cluster 1, H4b	HIST1H4B
234013_at	0.26	T cell receptor alpha locus#T cell receptor alpha variable 6#T cell receptor alpha variable 16#T cell receptor alpha variable 15#T cell receptor alpha variable 14/delta variable 4#T cell receptor alpha variable 13-2#T cell receptor alpha variable 13-1#T cell receptor alpha variable 12-3#T cell receptor alpha variable 12-2#T cell receptor alpha variable 12-1#T cell receptor alpha variable 11#T cell receptor alpha variable 10#T cell receptor alpha variable 9-2#T cell receptor alpha variable 9-1#T cell receptor alpha variable 8-6#T cell receptor alpha variable 8-5#T cell receptor alpha variable 8-4#T cell receptor alpha variable 8-3#T cell receptor alpha variable 8-2#T cell receptor alpha variable 8-1#T cell receptor alpha variable 7#T cell receptor alpha variable 5#null	TRA@#TRAV6#TRAV16#TRAV15#TRAV14DV4#TRAV13-2#TRAV13-1#TRAV12-3#TRAV12-2#TRAV12-1#TRAV11#TRAV10#TRAV9-2#TRAV9-1#TRAV8-6#TRAV8-5#TRAV8-4#TRAV8-3#TRAV8-2#TRAV8-1#TRAV7#TRAV5#null
240220_at	0.26	engulfment and cell motility 2	ELMO2
234790_at	0.26		
215302_at	0.26		
1556521_a_at	0.26		
1565825_at	0.26	lipoma HMGIC fusion partner	LHFP
237061_at	0.26	zinc finger protein 347	ZNF347
236910_at	0.25	mitochondrial ribosomal protein L39	MRPL39
240090_at	0.25		
240171_at	0.25		
216442_x_at	0.25	fibronectin 1	FN1
223900_s_at	0.25		
208384_s_at	0.25	midline 2	MID2
202995_s_at	0.25	fibulin 1	FBLN1
1561624_at	0.25		
210234_at	0.25	glutamate receptor, metabotropic 4	GRM4
223889_at	0.25		
234138_at	0.25		
207737_at	0.25		
213664_at	0.25	solute carrier family 1 (neuronal/epithelial high affinity glutamate transporter, system Xag), membe	SLC1A1
227913_at	0.25	exosome component 3	EXOSC3
242481_at	0.25		
211719_x_at	0.25	fibronectin 1	FN1

1562102_at	0.25	aldo-keto reductase family 1, member C2 (dihydrodiol dehydrogenase 2; bile acid binding protein; 3-a	AKR1C2
234789_at	0.25		
210997_at	0.25	hepatocyte growth factor (hepapoietin A; scatter factor)	HGF
208446_s_at	0.25	zinc finger, FYVE domain containing 9	ZFYVE9
207958_at	0.25	UDP glucuronosyltransferase 2 family, polypeptide A1	UGT2A1
228767_at	0.25	ataxin 2-like	ATXN2L
1555098_a_at	0.25	calcium channel, voltage-dependent, beta 2 subunit	CACNB2
1553708_at	0.25		
214798_at	0.25	ATPase, Ca++ transporting, type 2C, member 2	ATP2C2
235979_at	0.25	complement component 7	C7
213942_at	0.25	multiple EGF-like-domains 6	MEGF6
1557241_a_at	0.25		
214605_x_at	0.25	G protein-coupled receptor 1	GPR1
211782_at	0.25	iduronate 2-sulfatase (Hunter syndrome)	IDS
1554907_a_at	0.25	hydrocephalus inducing homolog (mouse)	HYDIN
238320_at	0.24		
216327_s_at	0.24	sialic acid binding Ig-like lectin 8	SIGLEC8
237690_at	0.24	G protein-coupled receptor 115	GPR115
244067_x_at	0.24		
208588_at	0.24		
242302_at	0.24	androgen-induced proliferation inhibitor	APRIN
216247_at	0.24		
205799_s_at	0.24	solute carrier family 3 (cystine, dibasic and neutral amino acid transporters, activator of cystine,	SLC3A1
1569948_at	0.24		
234134_at	0.24		
242387_at	0.24	chromosome 8 open reading frame 42	C8orf42
229675_at	0.24	MYC induced nuclear antigen	MINA
244465_at	0.24		
242269_at	0.24		
1565776_at	0.24		
221978_at	0.24	major histocompatibility complex, class I, F	HLA-F
227197_at	0.24		
1553658_at	0.24		
238579_at	0.23	chromosome 9 open reading frame 85	C9orf85
238569_at	0.23	gamma-aminobutyric acid (GABA) B receptor, 1	GABBR1
1569782_at	0.23		
217487_x_at	0.23	folate hydrolase (prostate-specific membrane antigen) 1	FOLH1
241653_x_at	0.23		
243017_at	0.23		
232272_at	0.23	zinc finger protein 624	ZNF624
1570176_at	0.23		
226884_at	0.23	leucine rich repeat neuronal 1	LRRN1
232694_at	0.23	zinc finger protein 395	ZNF395
1563827_at	0.23		
211332_x_at	0.23	hemochromatosis	HFE
229386_at	0.23	inhibitor of DNA binding 4, dominant negative helix-loop-helix protein	ID4

242206_at	0.23		
1556963_at	0.23		
1552708_a_at	0.23	dual specificity phosphatase 19	DUSP19
1558052_at	0.23	transmembrane emp24 protein transport domain containing 4	TMED4
222470_s_at	0.22	chromosome 20 open reading frame 44	C20orf44
220824_at	0.22		
238355_at	0.22	RNA binding motif protein 39	RBM39
222927_s_at	0.22	complexin 3	CPLX3
210853_at	0.22	sodium channel, voltage-gated, type XI, alpha subunit	SCN11A
240501_at	0.22		
237103_at	0.22	v-src sarcoma (Schmidt-Ruppin A-2) viral oncogene homolog (avian)	SRC
238239_at	0.22	WD repeat domain 27	WDR27
1557078_at	0.22	schlafen family member 5	SLFN5
233048_at	0.22	family with sequence similarity 35, member A	FAM35A
1561676_at	0.22		
237171_at	0.22		
1553523_at	0.22	NLR family, pyrin domain containing 14	NLRP14
1553354_a_at	0.22		
1561037_a_at	0.22		
1564757_a_at	0.22		
220614_s_at	0.22	chromosome 6 open reading frame 103	C6orf103
224548_at	0.22	hairy and enhancer of split 7 (Drosophila)	HES7
223595_at	0.22	transmembrane protein 133	TMEM133
1556429_a_at	0.22	WD repeat domain 67	WDR67
1552401_a_at	0.22		
207425_s_at	0.22	septin 9	9-Sep
236940_at	0.21		
235732_at	0.21		
1559229_at	0.21		
234603_at	0.21		
240844_at	0.21		
243889_at	0.21	hypoxia inducible factor 3, alpha subunit	HIF3A
237957_at	0.21		
206218_at	0.21	melanoma antigen family B, 2	MAGEB2
1568876_a_at	0.21		
1569810_at	0.21		
1566087_at	0.21		
1557203_at	0.21		
1562477_at	0.21	early B-cell factor 2	EBF2
207491_at	0.21	monoacylglycerol O-acyltransferase 2	MOGAT2
208145_at	0.21		
221466_at	0.21	pyrimidinergic receptor P2Y, G-protein coupled, 4	P2RY4
225806_at	0.21	jub, ajuba homolog (Xenopus laevis)	JUB
216856_s_at	0.21	deleted in lymphocytic leukemia, 2	DLEU2
1560851_at	0.21	chromosome 10 open reading frame 136	C10orf136
207815_at	0.21	platelet factor 4 variant 1	PF4V1
1559833_at	0.21	Rho guanine nucleotide exchange factor (GEF) 12	ARHGEF12
1557079_at	0.21	integrin, beta-like 1 (with EGF-like repeat domains)	ITGBL1
221958_s_at	0.21	G protein-coupled receptor 177	GPR177

206222_at	0.21	tumor necrosis factor receptor superfamily, member 10c, decoy without an intracellular domain	TNFRSF10C
1560404_a_at	0.21	ATP binding domain 4	ATPBD4
232060_at	0.21		
1561281_a_at	0.20		
216133_at	0.20	T cell receptor alpha locus	TRA@
235379_at	0.20		
1570398_at	0.20		
232871_at	0.20		
1559929_at	0.20		
1558120_at	0.20	DEAD (Asp-Glu-Ala-Asp) box polypeptide 3, X-linked	DDX3X
241285_at	0.20	calmodulin binding transcription activator 1	CAMTA1
221312_at	0.20	glucagon-like peptide 2 receptor	GLP2R
230271_at	0.20	one cut domain, family member 2	ONECUT2
205343_at	0.20	sulfotransferase family, cytosolic, 1C, member 1	SULT1C1
1555171_at	0.20	ST3 beta-galactoside alpha-2,3-sialyltransferase 3	ST3GAL3
1562651_at	0.20	zinc finger and SCAN domain containing 1	ZSCAN1
1560416_at	0.20	dynein, axonemal, heavy chain 11	DNAH11
220141_at	0.20	chromosome 11 open reading frame 63	C11orf63
1553811_at	0.20		
1563386_at	0.20		
218692_at	0.20		
222184_at	0.20		
1569121_at	0.20	solute carrier family 25 (mitochondrial carrier; phosphate carrier), member 24	SLC25A24
223736_at	0.19	intraflagellar transport 81 homolog (Chlamydomonas)	IFT81
230039_at	0.19		
1556649_at	0.19		
215393_s_at	0.19	COBL-like 1	COBLL1
210118_s_at	0.19	interleukin 1, alpha	IL1A
201510_at	0.19	E74-like factor 3 (ets domain transcription factor, epithelial-specific)	ELF3
234447_at	0.19		
214345_at	0.19	EGF-like repeats and discoidin I-like domains 3	EDIL3
240201_at	0.19		
232849_at	0.19		
1555135_at	0.19		
238755_at	0.19		
211693_at	0.19	immunoglobulin heavy constant alpha 1	IGHA1
242815_x_at	0.19		
240945_at	0.19		
210864_x_at	0.19	hemochromatosis	HFE
1559129_a_at	0.19		
223760_s_at	0.19		
215459_at	0.19	Tax1 (human T-cell leukemia virus type I) binding protein 3	TAX1BP3
242112_at	0.19	LSM11, U7 small nuclear RNA associated	LSM11
211514_at	0.19	receptor interacting protein kinase 5	RIPK5
241238_at	0.19		
221464_at	0.19	olfactory receptor, family 1, subfamily D, member 2	OR1D2
224109_at	0.19		

240179_at	0.19		
217369_at	0.18	immunoglobulin heavy constant gamma 1 (G1m marker)	IGHG1
215445_x_at	0.18		
1569809_at	0.18		
1554268_at	0.18	MORN repeat containing 1	MORN1
239984_at	0.18	sodium channel, voltage-gated, type VII, alpha	SCN7A
239295_at	0.18		
217049_x_at	0.18	protocadherin 11 Y-linked	PCDH11Y
240506_at	0.18		
229048_at	0.18		
240795_at	0.18		
239278_at	0.18		
1553823_a_at	0.18	receptor (chemosensory) transporter protein 1	RTP1
202674_s_at	0.18	LIM domain 7	LMO7
220623_s_at	0.18	testis specific, 10	TSGA10
217239_x_at	0.18		
213747_at	0.18	antizyme inhibitor 1	AZIN1
232611_at	0.18		
1561148_at	0.18		
1563162_at	0.18		
212328_at	0.18		
241257_at	0.18		
228584_at	0.18	sarcoglycan, beta (43kDa dystrophin-associated glycoprotein)	SGCB
1555564_a_at	0.18	complement factor I	CFI
1561606_at	0.17		
1555867_at	0.17		
1561343_a_at	0.17		
1568012_at	0.17	CAP-GLY domain containing linker protein 1	CLIP1
242876_at	0.17	v-akt murine thymoma viral oncogene homolog 3 (protein kinase B, gamma)	AKT3
210495_x_at	0.17	fibronectin 1	FN1
244690_at	0.17		
1555095_at	0.17	chromosome 6 open reading frame 123	C6orf123
233406_at	0.17		
244276_at	0.17	klotho beta	KLB
240904_at	0.17		
240300_at	0.17	thymidine kinase 2, mitochondrial	TK2
240878_at	0.17	fibroblast growth factor 11	FGF11
1552899_at	0.16		
1555385_at	0.16	beta-1,4-N-acetyl-galactosaminyl transferase 1	B4GALNT1
234666_at	0.16		
228732_at	0.16		
221051_s_at	0.16	integrin beta 1 binding protein 3	ITGB1BP3
211249_at	0.16	G protein-coupled receptor 68	GPR68
207053_at	0.16	solute carrier family 8 (sodium/calcium exchanger), member 1	SLC8A1
221349_at	0.16	pre-B lymphocyte gene 1	VPREB1
213909_at	0.16	leucine rich repeat containing 15	LRRC15
1553834_at	0.16		
207322_at	0.16	intersectin 1 (SH3 domain protein)	ITSN1
1565637_at	0.16		

221321_s_at	0.15	Kv channel interacting protein 2	KCNIP2
1561363_a_at	0.15		
231566_at	0.15	chromosome 14 open reading frame 148	C14orf148
238392_at	0.15	translocation associated membrane protein 2	TRAM2
215252_at	0.15		
1558375_at	0.15	leucine rich repeat containing 38	LRRC38
1570536_at	0.15		
1553401_at	0.15	MAS-related GPR, member X1	MRGPRX1
1559665_at	0.15		
220830_at	0.15	interphotoreceptor matrix proteoglycan 2	IMPG2
1568826_at	0.15		
1556488_s_at	0.15	chromosome 3 open reading frame 15	C3orf15
237795_s_at	0.14		
1552658_a_at	0.14	neuron navigator 3	NAV3
231580_at	0.14		
233127_at	0.14		
1554571_at	0.14	amyloid beta (A4) precursor protein-binding, family B, member 1 interacting protein	APBB1IP
211608_at	0.14		
235715_at	0.14		
238489_at	0.14		
242697_at	0.14	zinc finger protein 540	ZNF540
236597_at	0.14	UDP glycosyltransferase 3 family, polypeptide A1	UGT3A1
220483_s_at	0.14	ring finger protein 19	RNF19
1556236_at	0.14		
231583_at	0.14	keratin 74	KRT74
220436_at	0.13	contactin associated protein-like 3B	CNTNAP3B
241868_at	0.13		
210366_at	0.13	solute carrier organic anion transporter family, member 1B1	SLCO1B1
241519_at	0.13	histone cluster 1, H2ba	HIST1H2BA
237891_at	0.13	Mdm2, transformed 3T3 cell double minute 2, p53 binding protein (mouse)	MDM2
1562879_at	0.13		
207300_s_at	0.13	coagulation factor VII (serum prothrombin conversion accelerator)	F7
205669_at	0.13	neural cell adhesion molecule 2	NCAM2
239292_at	0.13		
217319_x_at	0.13	SCL/TAL1 interrupting locus#T-cell acute lymphocytic leukemia 1#PDZK1 interacting protein 1#cytochrome P450, family 4, subfamily Z, polypeptide 1#cytochrome P450, family 4, subfamily A, polypeptide 22	STIL#TAL1#PDZK1IP1#CYP4Z1#CYP4A22
1556469_s_at	0.13		
205481_at	0.12	adenosine A1 receptor	ADORA1
1565898_at	0.12	methyltransferase 5 domain containing 1	METT5D1
242755_at	0.12	SFRS protein kinase 2	SRPK2
1561713_at	0.12		
221609_s_at	0.12	wingless-type MMTV integration site family, member 6	WNT6
243211_at	0.12		
1559971_at	0.12	BSD domain containing 1	BSDC1
1568590_at	0.12	ADP-ribosylation factor-like 3	ARL3

244388_at	0.12	wingless-type MMTV integration site family, member 5A	WNT5A
239827_at	0.12		
1561692_at	0.12		
227885_at	0.12		
1566937_at	0.12		
244645_at	0.12	collagen, type XIV, alpha 1 (undulin)	COL14A1
234548_at	0.12		
240958_at	0.12	unc-5 homolog C (C. elegans)	UNC5C
1561589_a_at	0.12	neurobeachin-like 1	NBEAL1
244369_at	0.12	chromosome 21 open reading frame 59	C21orf59
241831_at	0.12	zinc finger protein 614	ZNF614
238379_x_at	0.11		
216970_at	0.11	regulator of G-protein signalling 7	RGS7
220696_at	0.11		
1555339_at	0.11	RAP1A, member of RAS oncogene family	RAP1A
215626_at	0.11		
206433_s_at	0.11	sparc/osteonectin, cwcv and kazal-like domains proteoglycan (testican) 3	SPOCK3
219795_at	0.11	solute carrier family 6 (amino acid transporter), member 14	SLC6A14
1555340_x_at	0.10	RAP1A, member of RAS oncogene family	RAP1A
220921_at	0.10	sperm protein associated with the nucleus, X-linked, family member A1	SPANXA1
219454_at	0.10	EGF-like-domain, multiple 6	EGFL6
238871_at	0.10	myeloid/lymphoid or mixed-lineage leukemia (trithorax homolog, Drosophila); translocated to, 4	MLLT4
236129_at	0.10	UDP-N-acetyl-alpha-D-galactosamine:polypeptide N-acetylgalactosaminyltransferase 5 (GalNAc-T5)	GALNT5
1555492_a_at	0.10	bestrophin 3	BEST3
206201_s_at	0.10	mesenchyme homeobox 2	MEOX2
206211_at	0.10	selectin E (endothelial adhesion molecule 1)	SELE
1556903_at	0.10		
208280_at	0.09	CMT1A duplicated region transcript 1	CDRT1
241994_at	0.09	xanthine dehydrogenase	XDH
1552765_x_at	0.09	transmembrane protein 67	TMEM67
1563856_at	0.09	insulin activator factor (insulin control element-binding transcription factor)	INSAF
1554354_at	0.09	sialic acid acetylesterase	SIAE
219949_at	0.09	leucine rich repeat containing 2	LRRC2
228740_at	0.09		
242050_at	0.09	leucine rich repeat containing 7	LRRC7
234108_at	0.09	taste receptor, type 2, member 45	TAS2R45
201645_at	0.08	tenascin C (hexabrachion)	TNC
209335_at	0.08	decorin	DCN
1561411_at	0.08		
1552732_at	0.08	actin-binding Rho activating protein	ABRA
240266_at	0.07	integrator complex subunit 7	INTS7
224321_at	0.07	transmembrane protein with EGF-like and two follistatin-like domains 2	TMEFF2
241035_s_at	0.07		
1567853_at	0.07	zinc finger protein 28	ZNF28
1563121_at	0.07		

211161_s_at	0.07	collagen, type III, alpha 1 (Ehlers-Danlos syndrome type IV, autosomal dominant)	COL3A1
235401_s_at	0.06	Fc receptor-like A	FCRLA
207723_s_at	0.06	killer cell lectin-like receptor subfamily C, member 3	KLRC3
1559839_at	0.06	T-box 18	TBX18
233569_at	0.05		
238318_at	0.05		
1552747_a_at	0.05	chromosome 3 open reading frame 48	C3orf48
205433_at	0.05	butyrylcholinesterase	BCHE
1562459_at	0.04		
1554492_at	0.03	thyroid adenoma associated	THADA
234640_x_at	0.03		
240874_at	0.03		
233845_at	0.03		
212592_at	0.03		
1564533_at	0.03		
