# Supplementary material for: In Vitro Transformation of Primary Human CD34+ Cells by AML Fusion Oncogenes: Early Gene Expression Profiling Reveals Possible Drug Target in AML
Source: PLoS One. 2010 Aug 27;5(8):e12464. doi: 10.1371/journal.pone.0012464 (PMC2929205; doi:10.1371/journal.pone.0012464)
Supplement: Table S22 — Genes deregulated by NUP98-HOXA9 6 h after transfection. Primary human CD34+ cells were nucleofected with either control pTracer-CMV/Bsd vector or vector expressing NUP98-HOXA9 and sorted for GFP positivity. Total RNA was extracted 6 h after nucleofection and subjected to microarray analysis. Microarray data were analyzed by SAM as described in Materials and Methods. Significantly deregulated genes are listed and the false discovery rate (FDR) is shown. (0.30 MB PDF) [file pone.0012464.s022.pdf]

Table S22. Genes deregulated by NUP98-HOXA9 at 6 h detected by SAM

FDR = 3.81%

Probe set ID	Fold Change	Gene Name	Gene Symbol
235885_at	35.47		
207147_at	30.85	distal-less homeobox 2	DLX2
223918_at	24.52	acyl-CoA synthetase long-chain family member 6	ACSL6
235355_at	18.68		
209101_at	18.00	connective tissue growth factor	CTGF
214567_s_at	15.56	chemokine (C motif) ligand 2	XCL2
1556609_at	14.82		
1553645_at	12.38		
214725_at	11.91		
235652_at	11.78		
242221_at	11.47		
1552480_s_at	11.40	protein tyrosine phosphatase, receptor type, C	PTPRC
1562270_at	11.05	Rho guanine nucleotide exchange factor (GEF) 7	ARHGEF7
236797_at	10.65		
211819_s_at	10.52	sorbin and SH3 domain containing 1	SORBS1
240462_at	10.01		
237008_at	9.98		
205499_at	9.92	sushi-repeat-containing protein, X-linked 2	SRPX2
220784_s_at	9.54	urotensin 2	UTS2
1555643_s_at	9.34	leukocyte immunoglobulin-like receptor, subfamily A (with TM domain), member 5	LILRA5
1561067_at	9.21		
1562431_x_at	9.15		
207729_at	9.07	cadherin 9, type 2 (T1-cadherin)	CDH9
226907_at	8.84	protein phosphatase 1, regulatory (inhibitor) subunit 14C	PPP1R14C
227725_at	8.76	ST6 (alpha-N-acetyl-neuraminyl-2,3-beta-galactosyl-1,3)-N-acetylgalactosaminide alpha-2,6-sialyltran	ST6GALNAC1
204967_at	8.75	shroom family member 2	SHROOM2
240039_at	8.48		
1562942_at	8.40		
204259_at	8.29	matrix metallopeptidase 7 (matrilysin, uterine)	MMP7
219106_s_at	8.27	kelch repeat and BTB (POZ) domain containing 10	KBTBD10
240472_at	8.24		
229201_at	8.21		
244146_at	8.19	DTW domain containing 1	DTWD1
1565722_at	8.11		
242634_at	7.94	GATA zinc finger domain containing 1	GATAD1
233871_at	7.89		
231488_at	7.84		
234178_at	7.84		
204988_at	7.80	fibrinogen beta chain	FGB
234529_at	7.80		
207517_at	7.76	laminin, gamma 2	LAMC2
1560483_at	7.73		
230368_at	7.70	glycogen synthase kinase 3 alpha	GSK3A

1562628_at	7.68	keratin 40	KRT40
244853_at	7.68		
229057_at	7.64	sodium channel, voltage-gated, type II, alpha subunit	SCN2A
206978_at	7.62	chemokine (C-C motif) receptor 2	CCR2
219962_at	7.45	angiotensin I converting enzyme (peptidyl-dipeptidase A) 2	ACE2
1566868_at	7.42		
206504_at	7.41	cytochrome P450, family 24, subfamily A, polypeptide 1	CYP24A1
206336_at	7.39	chemokine (C-X-C motif) ligand 6 (granulocyte chemotactic protein 2)	CXCL6
244874_at	7.36		
238178_at	7.29		
1561125_at	7.10	methylenetetrahydrofolate dehydrogenase (NADP+ dependent) 1-like	MTHFD1L
224278_at	7.06	chromosome 2 open reading frame 14	C2orf14
233402_at	7.03	sorting nexing 24	SNX24
216290_x_at	6.92		
236121_at	6.81	olfactory receptor, family 51, subfamily E, member 2	OR51E2
234556_at	6.78		
232544_at	6.71		
1561238_at	6.58	peroxisomal membrane protein 3, 35kDa (Zellweger syndrome)	PXMP3
226498_at	6.56		
207447_s_at	6.49	mannosyl (alpha-1,3-)-glycoprotein beta-1,4-N-acetylglucosaminyltransferase, isozyme C (putative)	MGAT4C
1560994_x_at	6.48		
237010_at	6.41	STEAP family member 3	STEAP3
202551_s_at	6.38	cysteine rich transmembrane BMP regulator 1 (chordin-like)	CRIM1
242622_x_at	6.31	phosphatase and tensin homolog (mutated in multiple advanced cancers 1)	PTEN
216068_at	6.29		
1554712_a_at	6.28	glycine-N-acyltransferase-like 2	GLYATL2
207142_at	6.18	potassium inwardly-rectifying channel, subfamily J, member 3	KCNJ3
241191_at	6.18	TRAF family member-associated NFKB activator	TANK
1558882_at	6.18		
1561965_at	6.12		
216104_at	5.96		
240977_at	5.92	leucine-rich repeats and calponin homology (CH) domain containing 1	LRCH1
215494_at	5.89		
216737_at	5.83		
243792_x_at	5.81	protein tyrosine phosphatase, non-receptor type 13 (APO-1/CD95 (Fas)-associated phosphatase)	PTPN13
233490_at	5.77	dynactin 4 (p62)	DCTN4
241283_at	5.71		
1562876_s_at	5.68		

212611_at	5.67		
1556421_at	5.67		
239558_at	5.67		
1560011_at	5.66	prostate stem cell antigen	PSCA
206366_x_at	5.66	chemokine (C motif) ligand 1	XCL1
242367_at	5.65	solute carrier family 38, member 1	SLC38A1
1562316_at	5.64		
215139_at	5.60	Rho guanine nucleotide exchange factor (GEF) 10	ARHGEF10
230447_at	5.60		
240694_at	5.54		
1559591_s_at	5.52	choline dehydrogenase	CHDH
1567612_at	5.50		
1559949_at	5.46	trichorhinophalangeal syndrome I	TRPS1
239814_at	5.43		
1558649_at	5.43		
1560957_at	5.41		
227874_at	5.40		
1561324_at	5.36		
243847_at	5.36		
203821_at	5.33	heparin-binding EGF-like growth factor	HBEGF
1556521_a_at	5.31		
224073_at	5.28		
231439_at	5.26		
207145_at	5.24	growth differentiation factor 8	GDF8
242426_at	5.24	neuregulin 4	NRG4
204753_s_at	5.23	hepatic leukemia factor	HLF
204754_at	5.23	hepatic leukemia factor	HLF
226534_at	5.22	KIT ligand	KITLG
215262_at	5.20		
213496_at	5.19		
244705_at	5.16		
1554946_at	5.15		
1556351_at	5.15	hyperpolarization activated cyclic nucleotide-gated potassium channel 1	HCN1
225481_at	5.13	FERM domain containing 6	FRMD6
1555586_at	5.11	family with sequence similarity 71, member B	FAM71B
1561154_at	5.06		
1569023_a_at	5.05		
238645_at	5.03		
234547_at	5.02		
1560540_x_at	5.02		
1566295_at	5.00	family with sequence similarity 118, member B	FAM118B
1564402_at	4.95		
1555346_at	4.94	cell division cycle 20 homolog B (S. cerevisiae)	CDC20B
242721_at	4.94	autism susceptibility candidate 2	AUTS2
1556827_at	4.94		
224322_at	4.91	AT rich interactive domain 4B (RBP1-like)	ARID4B
1562619_at	4.90	thioredoxin domain containing 6	TXNDC6
1558466_at	4.87		
204755_x_at	4.85	hepatic leukemia factor	HLF
216433_s_at	4.84	PR domain containing 2, with ZNF domain	PRDM2
213580_at	4.83	CREB/ATF bZIP transcription factor	CREBZF

1562079_at	4.81		
204560_at	4.79	FK506 binding protein 5	FKBP5
223620_at	4.79	G protein-coupled receptor 34	GPR34
219377_at	4.79	family with sequence similarity 59, member A	FAM59A
220432_s_at	4.78	cytochrome P450, family 39, subfamily A, polypeptide 1	CYP39A1
1560320_a_at	4.78		
214284_s_at	4.75	fibroblast growth factor 18	FGF18
231709_x_at	4.73		
244259_s_at	4.71		
1568734_a_at	4.69	HCLS1 binding protein 3	HS1BP3
1558383_at	4.67		
1565602_at	4.67		
215233_at	4.67	phosphatidylserine receptor	PTDSR
201289_at	4.67	cysteine-rich, angiogenic inducer, 61	CYR61
214368_at	4.62	RAS guanyl releasing protein 2 (calcium and DAG-regulated)	RASGRP2
1556683_x_at	4.61		
208005_at	4.60	netrin 1	NTN1
1563260_at	4.60	chromosome 9 open reading frame 107	C9orf107
1553319_at	4.59	oxoglutarate (alpha-ketoglutarate) receptor 1	OXGR1
1564795_at	4.59		
231330_at	4.58		
1553772_at	4.58	glycerol kinase 5 (putative)	GK5
1561657_at	4.58		
238966_at	4.57		
1563188_at	4.57		
219295_s_at	4.55	procollagen C-endopeptidase enhancer 2	PCOLCE2
1556037_s_at	4.51	hedgehog interacting protein	HHIP
236794_at	4.45	C-terminal binding protein 2	CTBP2
1555811_at	4.42	Rho GDP dissociation inhibitor (GDI) beta	ARHGDIB
218793_s_at	4.41	sex comb on midleg-like 1 (Drosophila)	SCML1
219049_at	4.41		
1561264_at	4.39		
1563512_at	4.37	nitric oxide synthase 1 (neuronal) adaptor protein	NOS1AP
228821_at	4.37	ST6 beta-galactosamide alpha-2,6-sialyltranferase 2	ST6GAL2
205867_at	4.37	protein tyrosine phosphatase, non-receptor type 11 (Noonan syndrome 1)	PTPN11
232295_at	4.35	G elongation factor, mitochondrial 1	GFM1
243548_x_at	4.35		
241470_x_at	4.34		
244590_at	4.33		
1569809_at	4.30		
1562761_at	4.25	chromosome 9 open reading frame 95	C9orf95
239777_at	4.25		
1558124_at	4.22	NudC domain containing 2	NUDCD2
213474_at	4.22		
219773_at	4.20	NADPH oxidase 4	NOX4
231560_at	4.20	leucine rich repeat containing 34	LRRC34

243655_x_at	4.20	SWI/SNF related, matrix associated, actin dependent regulator of chromatin, subfamily a, member 4	SMARCA4
231155_at	4.19	defensin, beta 119	DEFB119
1561997_at	4.17		
1570371_a_at	4.16		
215551_at	4.15	estrogen receptor 1	ESR1
230119_at	4.14		
206232_s_at	4.13	UDP-Gal:betaGlcNAc beta 1,4-galactosyltransferase, polypeptide 6	B4GALT6
1561663_at	4.11		
224104_at	4.11		
1562579_at	4.09		
1555273_at	4.08		
243663_at	4.07		
206591_at	4.05	recombination activating gene 1	RAG1
236738_at	4.03		
224406_s_at	4.03	Fc receptor-like 5	FCRL5
232751_at	4.02	Sec23 homolog B (S. cerevisiae)#polymerase (RNA) III (DNA directed) polypeptide F, 39 kDa#retinoblastoma binding protein 9#chromosome 20 open reading frame 12#ribosomal protein S19 pseudogene 1#ribosomal protein L21 pseudogene 3#glucosaminyl (N-acetyl) transferase 1, core 2 (beta-1,6-N-acetylglucosaminyltransferase) pseudogene	SEC23B#POLR3F#RBBP9#C20orf12#RPS19P1#RPL21P3#GCNT1P
240573_at	4.02		
211138_s_at	4.01	kynurenine 3-monooxygenase (kynurenine 3-hydroxylase)	KMO
233145_at	4.00	CUB and Sushi multiple domains 2	CSMD2
211520_s_at	4.00	glutamate receptor, ionotropic, AMPA 1	GRIA1
223168_at	3.98	ferritin, heavy polypeptide-like 2#ras homolog gene family, member U	FTHL2#RHOU
241831_at	3.98	zinc finger protein 614	ZNF614
233534_at	3.97	keratin associated protein 3-2	KRTAP3-2
1561230_at	3.97		
204654_s_at	3.97	transcription factor AP-2 alpha (activating enhancer binding protein 2 alpha)	TFAP2A
238269_at	3.97	F-box and leucine-rich repeat protein 7	FBXL7
1557486_at	3.95		
203769_s_at	3.94	steroid sulfatase (microsomal), arylsulfatase C, isozyme S	STS
233549_at	3.93	phosphodiesterase 1A, calmodulin-dependent	PDE1A
1555025_at	3.93	transmembrane protein 26	TMEM26
1566624_at	3.90		
209335_at	3.88	decorin	DCN
206179_s_at	3.87		
1570506_at	3.86		
1557321_a_at	3.85	calpain 14	CAPN14
240202_x_at	3.85	MAP/microtubule affinity-regulating kinase 1	MARK1
1561650_s_at	3.85		
241491_at	3.84		

1555024_at	3.83	ADAM metallopeptidase domain 22	ADAM22
230708_at	3.81	prickle homolog 1 (Drosophila)	PRICKLE1
203144_s_at	3.79	KIAA0040	KIAA0040
1563027_at	3.79		
223395_at	3.77	ABI gene family, member 3 (NESH) binding protein	ABI3BP
212850_s_at	3.77	low density lipoprotein receptor-related protein 4	LRP4
204464_s_at	3.77	endothelin receptor type A	EDNRA
235069_at	3.77	TatD DNase domain containing 3	TATDN3
234320_at	3.76	CD244 molecule, natural killer cell receptor 2B4	CD244
1555277_a_at	3.76	solute carrier family 4, sodium bicarbonate cotransporter, member 5	SLC4A5
220803_at	3.75	STAM binding protein-like 1	STAMBPL1
1569948_at	3.75		
223204_at	3.73	chromosome 4 open reading frame 18	C4orf18
1560573_at	3.73		
214189_s_at	3.72	golgi associated, gamma adaptin ear containing, ARF binding protein 2	GGA2
204939_s_at	3.71	phospholamban	PLN
239150_at	3.71		
205844_at	3.71	vanin 1	VNN1
206388_at	3.70	phosphodiesterase 3A, cGMP-inhibited	PDE3A
1561002_at	3.69		
1556520_at	3.69		
230520_at	3.69	androgen-induced 1	AIG1
1555536_at	3.69	anthrax toxin receptor 2	ANTXR2
244257_at	3.68	transmembrane protein 104	TMEM104
228414_at	3.68	potassium large conductance calcium-activated channel, subfamily M, alpha member 1	KCNMA1
242924_at	3.67	phosphatidylinositol transfer protein, beta	PITPNB
237663_at	3.66		
240741_x_at	3.66		
209355_s_at	3.66	phosphatidic acid phosphatase type 2B	PPAP2B
1553655_at	3.65	cell division cycle 20 homolog B (S. cerevisiae)	CDC20B
1560225_at	3.65	cannabinoid receptor 1 (brain)	CNR1
218711_s_at	3.64	serum deprivation response (phosphatidylserine binding protein)	SDPR
228262_at	3.64		
225464_at	3.63	FERM domain containing 6	FRMD6
1552732_at	3.63	actin-binding Rho activating protein	ABRA
228290_at	3.63	chromosome 20 open reading frame 19	C20orf19
219876_s_at	3.63	golgi autoantigen, golgin subfamily a, 2-like 1	GOLGA2L1
212253_x_at	3.63	dystonin	DST
224175_s_at	3.62	tripartite motif-containing 34	TRIM34
214213_x_at	3.61	lamin A/C	LMNA
214010_s_at	3.61	ATPase, Class II, type 9B	ATP9B
235740_at	3.60		
239512_at	3.58	splicing factor, arginine/serine-rich 4	SFRS4
241788_x_at	3.58		
212230_at	3.58	phosphatidic acid phosphatase type 2B	PPAP2B
1555808_a_at	3.58	exonuclease 3'-5' domain-like 2	EXDL2
222717_at	3.56	serum deprivation response (phosphatidylserine binding protein)	SDPR

205306_x_at	3.56	kynurenine 3-monooxygenase (kynurenine 3-hydroxylase)	KMO
223279_s_at	3.56	uveal autoantigen with coiled-coil domains and ankyrin repeats	UACA
206983_at	3.53	chemokine (C-C motif) receptor 6	CCR6
238387_s_at	3.52		
244021_at	3.52		
1558411_at	3.52	chromosome 3 open reading frame 50	C3orf50
1561078_at	3.51		
220676_at	3.51	ADAM metallopeptidase with thrombospondin type 1 motif, 8	ADAMTS8
204260_at	3.50	chromogranin B (secretogranin 1)	CHGB
1554987_at	3.50	golgi autoantigen, golgin subfamily a, 3	GOLGA3
236587_at	3.50		
222925_at	3.48	doublecortin domain containing 2	DCDC2
1555551_at	3.46	serpin peptidase inhibitor, clade B (ovalbumin), member 5	SERPINB5
242792_at	3.46	nuclear factor I/B	NFIB
220122_at	3.46	multiple C2 domains, transmembrane 1	MCTP1
219786_at	3.45	metallothionein-like 5, testis-specific (tesmin)	MTL5
1569987_at	3.44		
1561313_at	3.44		
234994_at	3.43	KIAA1913	KIAA1913
224062_x_at	3.43	kallikrein-related peptidase 4	KLK4
1562217_at	3.42		
1556717_at	3.41		
221077_at	3.41	armadillo repeat containing 4	ARMC4
239370_at	3.41		
1563173_at	3.40		
234393_at	3.39	histone deacetylase 9	HDAC9
1569647_at	3.39		
215376_at	3.38		
204222_s_at	3.37	GLI pathogenesis-related 1 (glioma)	GLIPR1
1553674_at	3.37	leucine rich repeat containing 44	LRRC44
234822_at	3.37		
1559950_at	3.36		
207661_s_at	3.35	SH3 and PX domains 2A	SH3PXD2A
241612_at	3.35		
240334_at	3.35	leucine rich repeat and fibronectin type III domain containing 5	LRFN5
230046_at	3.34		
1558975_at	3.33	family with sequence similarity 100, member A	FAM100A
230327_at	3.33		
234278_at	3.33	epidermal growth factor receptor pathway substrate 15	EPS15
230507_at	3.33	ataxin 1	ATXN1
1554649_at	3.33		
1560475_at	3.32		
207672_at	3.31	regulatory factor X, 4 (influences HLA class II expression)	RFX4
204221_x_at	3.31	GLI pathogenesis-related 1 (glioma)	GLIPR1
229147_at	3.31		
229964_at	3.30	chromosome 9 open reading frame 152	C9orf152

1561763_at	3.29		
1563312_at	3.28		
230130_at	3.27	slit homolog 2 (Drosophila)	SLIT2
1559760_at	3.27		
202609_at	3.25	epidermal growth factor receptor pathway substrate 8	EPS8
1564066_at	3.25	chromosome 6 open reading frame 137	C6orf137
232847_at	3.25	sal-like 3 (Drosophila)	SALL3
227692_at	3.23	guanine nucleotide binding protein (G protein), alpha inhibiting activity polypeptide 1	GNAI1
232144_at	3.23	pre-B-cell leukemia homeobox 1	PBX1
220393_at	3.23	glutamate-ammonia ligase (glutamine synthetase) domain containing 1	GLULD1
223614_at	3.22	chromosome 8 open reading frame 57	C8orf57
1561003_at	3.22		
217428_s_at	3.21	collagen, type X, alpha 1(Schmid metaphyseal chondrodysplasia)	COL10A1
235638_at	3.20	Ras association (RalGDS/AF-6) domain family 6	RASSF6
1559877_at	3.18		
233553_at	3.18		
1557513_a_at	3.18		
205729_at	3.16	oncostatin M receptor	OSMR
215065_at	3.16	PHD finger protein 8	PHF8
242709_s_at	3.16		
222153_at	3.16	myelin expression factor 2	MYEF2
216196_at	3.15		
241736_at	3.15	F-box and WD repeat domain containing 2	FBXW2
235063_at	3.15	chromosome 20 open reading frame 196	C20orf196
212224_at	3.15	aldehyde dehydrogenase 1 family, member A1	ALDH1A1
240000_at	3.15		
241209_at	3.15	IQ motif and WD repeats 1	IQWD1
207673_at	3.15	nephrosis 1, congenital, Finnish type (nephrin)	NPHS1
1560806_at	3.13		
234905_at	3.13		
234768_at	3.12		
1558766_at	3.11		
205939_at	3.11	cytochrome P450, family 3, subfamily A, polypeptide 7	CYP3A7
1566527_at	3.11		
208297_s_at	3.10	ecotropic viral integration site 5	EVI5
242518_at	3.09		
227195_at	3.09	zinc finger protein 503	ZNF503
215993_at	3.08		
213920_at	3.08	cut-like 2 (Drosophila)	CUTL2
239847_at	3.07		
235521_at	3.07	homeobox A3	HOXA3
1556817_a_at	3.07		
207874_s_at	3.07	complement factor H-related 4	CFHR4
239710_at	3.06		
1553794_at	3.06	stomatin (EPB72)-like 3	STOML3
221010_s_at	3.05	sirtuin (silent mating type information regulation 2 homolog) 5 (S. cerevisiae)	SIRT5
229591_at	3.04	low density lipoprotein receptor-related protein 5	LRP5

211812_s_at	3.04	beta-1,3-N-acetylgalactosaminyltransferase 1 (globoside blood group)	B3GALNT1
1556735_at	3.04		
204792_s_at	3.04	intraflagellar transport 140 homolog (Chlamydomonas)	IFT140
1559214_at	3.02		
217049_x_at	3.01	protocadherin 11 Y-linked	PCDH11Y
233062_at	3.01		
228202_at	3.01	phospholamban	PLN
1558322_a_at	3.01	progestin and adipoQ receptor family member IX	PAQR9
1556209_at	3.00	C-type lectin domain family 2, member B	CLEC2B
207295_at	3.00	sodium channel, nonvoltage-gated 1, gamma	SCNN1G
205536_at	2.99	vav 2 oncogene	VAV2
202274_at	2.99	actin, gamma 2, smooth muscle, enteric	ACTG2
220146_at	2.99	toll-like receptor 7	TLR7
226435_at	2.98	papilin, proteoglycan-like sulfated glycoprotein	PAPLN
240012_at	2.98		
223557_s_at	2.98	transmembrane protein with EGF-like and two follistatin-like domains 2	TMEFF2
214772_at	2.98	chromosome 11 open reading frame 41	C11orf41
210080_x_at	2.98	elastase 3A, pancreatic	ELA3A
1556417_a_at	2.98		
237913_at	2.98		
205877_s_at	2.98	zinc finger CCCH-type containing 7B	ZC3H7B
1560863_a_at	2.98		
226837_at	2.97	sprouty-related, EVH1 domain containing 1	SPRED1
1565685_at	2.96		
1562484_at	2.96		
243184_at	2.95	tight junction protein 1 (zona occludens 1)	TJP1
1558611_at	2.95	chromosome 17 open reading frame 69	C17orf69
221884_at	2.95	ecotropic viral integration site 1	EVI1
234127_at	2.94		
241639_at	2.94		
1553883_at	2.94	zinc finger protein 99	ZNF99
242654_at	2.94	Fanconi anemia, complementation group C	FANCC
1561148_at	2.93		
1554492_at	2.92	thyroid adenoma associated	THADA
214251_s_at	2.92	nuclear mitotic apparatus protein 1	NUMA1
233011_at	2.92	annexin A1	ANXA1
244238_at	2.92		
204751_x_at	2.91	desmocollin 2	DSC2
223889_at	2.91		
220679_s_at	2.90	cadherin 7, type 2	CDH7
244408_at	2.90		
224202_at	2.89	suppressor of fused homolog (Drosophila)	SUFU
227671_at	2.89	X (inactive)-specific transcript	XIST
1557993_at	2.89		
1564007_at	2.88		
206682_at	2.88	C-type lectin domain family 10, member A	CLEC10A
1566482_at	2.88		
1554730_at	2.87	multiple C2 domains, transmembrane 1	MCTP1
1562766_at	2.87		

1562071_at	2.87		
1562712_at	2.86		
238217_at	2.86		
236893_at	2.86		
242828_at	2.86	fidgetin	FIGN
224397_s_at	2.84	transmembrane and tetratricopeptide repeat containing 1	TMTC1
241583_x_at	2.84	synaptotagmin I	SYT1
201243_s_at	2.84	ATPase, Na+/K+ transporting, beta 1 polypeptide	ATP1B1
1556777_a_at	2.83		
1560756_at	2.83		
1554783_s_at	2.82	rho/rac guanine nucleotide exchange factor (GEF) 2	ARHGEF2
1557724_a_at	2.82	FK506 binding protein 5	FKBP5
206105_at	2.82	AF4/FMR2 family, member 2	AFF2
238489_at	2.81		
217382_at	2.81		
229260_at	2.81	chromosome 5 open reading frame 15	C5orf15
1563170_at	2.80		
227484_at	2.79		
207881_at	2.79		
238705_at	2.78		
238844_s_at	2.78	nephronophthisis 1 (juvenile)	NPHP1
215477_at	2.78		
233876_at	2.78		
235846_at	2.78		
203032_s_at	2.77	fumarate hydratase	FH
210650_s_at	2.77	piccolo (presynaptic cytomatrix protein)	PCLO
1554524_a_at	2.76	olfactomedin 3	OLFM3
214085_x_at	2.75	KRR1, small subunit (SSU) processome component, homolog (yeast)	KRR1
1561453_at	2.75		
235701_at	2.75		
224856_at	2.75	FK506 binding protein 5	FKBP5
226420_at	2.75	ecotropic viral integration site 1	EVI1
239999_at	2.74	chromosome 21 open reading frame 34	C21orf34
214862_x_at	2.74		
1570352_at	2.73	ataxia telangiectasia mutated (includes complementation groups A, C and D)	ATM
203789_s_at	2.73	sema domain, immunoglobulin domain (Ig), short basic domain, secreted, (semaphorin) 3C	SEMA3C
1553691_at	2.73	beta-1,3-N-acetylgalactosaminyltransferase 2	B3GALNT2
244863_at	2.72		
201739_at	2.72	serum/glucocorticoid regulated kinase	SGK
234454_at	2.71	vasohibin 1#angel homolog 1 (Drosophila)#chromosome 14 open reading frame 166B#ribosomal protein L22 pseudogene 2	VASH1#ANGEL1#C14orf166B#RPL22P2
1568673_s_at	2.71	ELL associated factor 2	EAF2
1565681_s_at	2.70	DIP2 disco-interacting protein 2 homolog C (Drosophila)	DIP2C
232291_at	2.70	microRNA host gene (non-protein coding) 1	MIRH1

1570441_at	2.70	N-ethylmaleimide-sensitive factor attachment protein, beta	NAPB
239308_at	2.70	dual-specificity tyrosine-(Y)-phosphorylation regulated kinase 1A	DYRK1A
239870_at	2.69	spermatogenesis associated, serine-rich 1	SPATS1
220169_at	2.69	transmembrane protein 156	TMEM156
1556012_at	2.68	kelch domain containing 7A	KLHDC7A
240151_at	2.68		
240465_at	2.68		
221138_s_at	2.67		
222719_s_at	2.67	platelet derived growth factor C	PDGFC
220869_at	2.67	ubiquitin-activating enzyme E1-like 2	UBE1L2
239455_at	2.67		
240622_at	2.67	progestin and adipoQ receptor family member III	PAQR3
205255_x_at	2.66	transcription factor 7 (T-cell specific, HMG-box)	TCF7
230846_at	2.66		
239552_at	2.66		
208425_s_at	2.66	tetratricopeptide repeat, ankyrin repeat and coiled-coil containing 2	TANC2
1556325_at	2.65	filamin A interacting protein 1	FILIP1
211431_s_at	2.65	TYRO3 protein tyrosine kinase	TYRO3
227254_at	2.65		
226492_at	2.65	sema domain, transmembrane domain (TM), and cytoplasmic domain, (semaphorin) 6D	SEMA6D
219615_s_at	2.65	potassium channel, subfamily K, member 5	KCNK5
238461_at	2.64	eukaryotic translation initiation factor 4E family member 3	EIF4E3
207115_x_at	2.64	mbt domain containing 1	MBTD1
1564757_a_at	2.64		
201796_s_at	2.64	valyl-tRNA synthetase	VARS
235937_at	2.64		
241361_at	2.64		
212215_at	2.64	prolyl endopeptidase-like	PREPL
228915_at	2.63	dachshund homolog 1 (Drosophila)	DACH1
234055_s_at	2.63	NTF2-like export factor 1#N-ethylmaleimide-sensitive factor attachment protein, beta#GDNF-inducible zinc finger protein 1#cystatin-like 1#cystatin 11	NXT1#NAPB#GZF1#CSTL1#CST11
241020_at	2.63	membrane protein, palmitoylated 5 (MAGUK p55 subfamily member 5)	MPP5
1569672_at	2.63		
236892_s_at	2.62		
232559_at	2.62		
1562381_at	2.62		
220161_s_at	2.62	erythrocyte membrane protein band 4.1 like 4B	EPB41L4B
208227_x_at	2.62	ADAM metallopeptidase domain 22	ADAM22
204557_s_at	2.61	DAZ interacting protein 1	DZIP1
206194_at	2.61	homeobox C4	HOXC4
212822_at	2.61	HEG homolog 1 (zebrafish)	HEG1
214139_at	2.61	AT rich interactive domain 4B (RBP1-like)	ARID4B
240895_at	2.61		
1553527_at	2.61	NLR family, pyrin domain containing 9	NLRP9
228977_at	2.61		

1562621_at	2.61		
207691_x_at	2.60	ectonucleoside triphosphate diphosphohydrolase 1	ENTPD1
232841_at	2.60		
1569858_at	2.59		
205518_s_at	2.59	cytidine monophosphate-N-acetylneuraminic acid hydroxylase (CMP-N-acetylneuraminate monooxygenase)	CMAH
1564468_at	2.58		
227443_at	2.58	chromosome 9 open reading frame 150	C9orf150
1554242_a_at	2.58	coagulation factor C homolog, cochlin (Limulus polyphemus)	COCH
1566267_at	2.58		
233430_at	2.57	TBC1 domain family, member 22B	TBC1D22B
216072_at	2.56		
230775_s_at	2.56	spastic paraplegia 20, spartin (Troyer syndrome)	SPG20
223595_at	2.56	transmembrane protein 133	TMEM133
203854_at	2.56	complement factor I	CFI
1562342_at	2.55		
243247_at	2.55	coiled-coil domain containing 26	CCDC26
226142_at	2.55	GLI pathogenesis-related 1 (glioma)	GLIPR1
203549_s_at	2.55	lipoprotein lipase	LPL
242546_at	2.55		
204028_s_at	2.54	RAB GTPase activating protein 1	RABGAP1
210755_at	2.54	hepatocyte growth factor (hepapoietin A; scatter factor)	HGF
244772_at	2.54		
209193_at	2.54	pim-1 oncogene	PIM1
1556170_at	2.53		
202124_s_at	2.53	trafficking protein, kinesin binding 2	TRAK2
221355_at	2.53	cholinergic receptor, nicotinic, gamma	CHRNG
211742_s_at	2.53	ecotropic viral integration site 2B	EVI2B
1568903_at	2.52		
218413_s_at	2.52	zinc finger protein 639	ZNF639
216933_x_at	2.52	adenomatosis polyposis coli	APC
1570354_s_at	2.51	zinc finger protein 169	ZNF169
1562308_at	2.51		
1561143_at	2.51		
1557762_at	2.51		
1553335_x_at	2.51		
1555898_at	2.51		
238264_at	2.51	NMD3 homolog (S. cerevisiae)	NMD3
201295_s_at	2.51	WD repeat and SOCS box-containing 1	WSB1
238919_at	2.51		
201418_s_at	2.50	SRY (sex determining region Y)-box 4	SOX4
232788_at	2.49		
217560_at	2.49		
204201_s_at	2.49	protein tyrosine phosphatase, non-receptor type 13 (APO-1/CD95 (Fas)-associated phosphatase)	PTPN13
232427_at	2.49	zinc finger protein 224	ZNF224
220524_at	2.49		
1559059_s_at	2.48	zinc finger protein 611	ZNF611

241837_at	2.48	AT rich interactive domain 5B (MRF1-like)	ARID5B
231479_at	2.47	tetratricopeptide repeat domain 33	TTC33
1558440_at	2.47		
1559928_at	2.47	pregnancy-associated plasma protein A, pappalysin 1	PAPPA
223799_at	2.47	KIAA1826	KIAA1826
203998_s_at	2.47	synaptotagmin I	SYT1
209795_at	2.47	CD69 molecule	CD69
1552808_at	2.47	ADAMTS-like 1	ADAMTSL1
1553120_at	2.46	claspin homolog (Xenopus laevis)	CLSPN
214631_at	2.46	zinc finger and BTB domain containing 33	ZBTB33
211402_x_at	2.46	nuclear receptor subfamily 6, group A, member 1	NR6A1
203788_s_at	2.46	sema domain, immunoglobulin domain (Ig), short basic domain, secreted, (semaphorin) 3C	SEMA3C
206327_s_at	2.46	cadherin 15, M-cadherin (myotubule)	CDH15
216279_at	2.46	zinc finger protein 460	ZNF460
239343_at	2.45		
210803_at	2.45	thioredoxin reductase 2	TXNRD2
244726_at	2.45		
202036_s_at	2.45	secreted frizzled-related protein 1	SFRP1
1552752_a_at	2.45	cell adhesion molecule 2	CADM2
218342_s_at	2.44	KIAA1815	KIAA1815
242564_at	2.44		
1552393_at	2.44	ENTH domain containing 1	ENTHD1
211303_x_at	2.44		
220667_at	2.43		
232478_at	2.43		
226136_at	2.43		
216272_x_at	2.43	synapse defective 1, Rho GTPase, homolog 1 (C. elegans)	SYDE1
214295_at	2.43		
230092_at	2.42	UBX domain containing 3	UBXD3
215796_at	2.42	T cell receptor alpha locus	TRA@
1569600_at	2.42	deleted in lymphocytic leukemia, 2	DLEU2
243055_at	2.42		
222649_at	2.42	exportin 4	XPO4
236270_at	2.42	nuclear factor of activated T-cells, cytoplasmic, calcineurin-dependent 4	NFATC4
236300_at	2.42		
236453_at	2.42		
232687_at	2.42		
1558111_at	2.41	muscleblind-like (Drosophila)	MBNL1
218468_s_at	2.41	gremlin 1, cysteine knot superfamily, homolog (Xenopus laevis)	GREM1
217678_at	2.41	solute carrier family 7, (cationic amino acid transporter, y+ system) member 11	SLC7A11
213844_at	2.41	homeobox A5	HOXA5
209212_s_at	2.41	Kruppel-like factor 5 (intestinal)	KLF5
240927_at	2.41		
1566691_at	2.41		
209905_at	2.41	homeobox A9	HOXA9
1562984_at	2.41		

221823_at	2.41	chromosome 5 open reading frame 30	C5orf30
1556657_at	2.41		
1554449_at	2.41	mesoderm induction early response 1, family member 3	MIER3
217599_s_at	2.41	MyoD family inhibitor domain containing	MDFIC
233652_at	2.40		
232380_at	2.40		
232127_at	2.40	chloride channel 5 (nephrolithiasis 2, X-linked, Dent disease)	CLCN5
244265_at	2.40	arginine-glutamic acid dipeptide (RE) repeats	RERE
1552579_a_at	2.40	ADAM metallopeptidase domain 21	ADAM21
214596_at	2.40		
240147_at	2.40		
1569428_at	2.40	within bgcn homolog (Drosophila)	WIBG
233304_at	2.39		
204082_at	2.39	pre-B-cell leukemia homeobox 3	PBX3
244419_at	2.39	frizzled-related protein	FRZB
1562625_at	2.39	FRY-like	FRYL
231428_at	2.38		
244831_at	2.38		
1564640_at	2.38	MAX gene associated	MGA
228692_at	2.38		
239342_at	2.38		
242235_x_at	2.38	nardilysin (N-arginine dibasic convertase)	NRD1
1556558_s_at	2.37		
210571_s_at	2.37	cytidine monophosphate-N-acetylneuraminic acid hydroxylase (CMP-N-acetylneuraminate monooxygenase)	CMAH
221294_at	2.37	G protein-coupled receptor 21	GPR21
206800_at	2.36	5,10-methylenetetrahydrofolate reductase (NADPH)	MTHFR
1561113_at	2.36		
237979_at	2.36	EH-domain containing 4	EHD4
230493_at	2.36	transmembrane protein 46	TMEM46
237741_at	2.36	solute carrier family 25, member 36	SLC25A36
201918_at	2.36	solute carrier family 25, member 36	SLC25A36
1563265_at	2.36		
237609_at	2.36		
234067_at	2.36		
238863_x_at	2.35		
243022_at	2.35		
239429_at	2.35		
214867_at	2.35		
212942_s_at	2.35	KIAA1199	KIAA1199
236655_at	2.35	tumor protein D52	TPD52
214034_at	2.34		
1558017_s_at	2.34		
238756_at	2.33		
227210_at	2.33		
235074_at	2.33	sprouty-related, EVH1 domain containing 1	SPRED1
228118_x_at	2.33		
240061_at	2.33		
239043_at	2.33	zinc finger protein 404	ZNF404

1560222_at	2.33		
205746_s_at	2.33	ADAM metallopeptidase domain 17 (tumor necrosis factor, alpha, converting enzyme)	ADAM17
201654_s_at	2.32	heparan sulfate proteoglycan 2 (perlecan)	HSPG2
205716_at	2.32	solute carrier family 25, member 40	SLC25A40
239924_at	2.32		
177_at	2.32	phospholipase D1, phosphatidylcholine-specific	PLD1
210061_at	2.32	zinc finger protein 589	ZNF589
210145_at	2.32	phospholipase A2, group IVA (cytosolic, calcium-dependent)	PLA2G4A
242494_at	2.31	phospholipase C, beta 1 (phosphoinositide-specific)	PLCB1
240467_at	2.31		
225056_at	2.31	signal-induced proliferation-associated 1 like 2	SIPA1L2
202552_s_at	2.31	cysteine rich transmembrane BMP regulator 1 (chordin-like)	CRIM1
233024_at	2.31	RAB18, member RAS oncogene family	RAB18
228584_at	2.31	sarcoglycan, beta (43kDa dystrophin-associated glycoprotein)	SGCB
216992_s_at	2.31	glutamate receptor, metabotropic 8	GRM8
1558518_at	2.30	mitogen-activated protein kinase kinase kinase 7 interacting protein 3	MAP3K7IP3
240023_at	2.30		
1556697_at	2.30	GPRIN family member 3	GPRIN3
219737_s_at	2.30	protocadherin 9	PCDH9
240173_at	2.30		
239867_at	2.29		
209135_at	2.29	aspartate beta-hydroxylase	ASPH
242314_at	2.29	trinucleotide repeat containing 6C	TNRC6C
240161_s_at	2.29	cell division cycle 20 homolog B (S. cerevisiae)	CDC20B
222809_x_at	2.29	chromosome 14 open reading frame 65	C14orf65
235761_at	2.29		
219497_s_at	2.28	B-cell CLL/lymphoma 11A (zinc finger protein)	BCL11A
1558754_at	2.28	zinc finger protein 763	ZNF763
205547_s_at	2.28	transgelin	TAGLN
230095_at	2.28	thioredoxin-like 2	TXNL2
241304_at	2.28	phosphoinositide-3-kinase, class 3	PIK3C3
207505_at	2.28	protein kinase, cGMP-dependent, type II	PRKG2
1559914_at	2.28		
232760_at	2.27	testis expressed sequence 15	TEX15
231172_at	2.27	chromosome 9 open reading frame 117	C9orf117
238304_at	2.27	dipeptidyl-peptidase 6	DPP6
1556568_a_at	2.26		
1554213_at	2.26	Rho guanine nucleotide exchange factor (GEF) 10	ARHGEF10
212614_at	2.26	AT rich interactive domain 5B (MRF1-like)	ARID5B
1563467_at	2.26		
237864_at	2.26		
223672_at	2.26	SH3-domain GRB2-like (endophilin) interacting protein 1	SGIP1
238423_at	2.25	synaptotagmin-like 3	SYTL3
232656_at	2.25		
1558483_at	2.25	leucine rich repeat containing 27	LRRC27

244031_at	2.25	receptor accessory protein 5	REEP5
242384_at	2.25		
1555166_a_at	2.24	zinc finger protein 396	ZNF396
1557759_at	2.24		
1558014_s_at	2.24	male sterility domain containing 2	MLSTD2
1557029_at	2.24		
211675_s_at	2.24	MyoD family inhibitor domain containing	MDFIC
230324_at	2.24	nuclear receptor coactivator 2	NCOA2
241645_at	2.24		
1570588_at	2.23		
220557_s_at	2.23	phosphofurin acidic cluster sorting protein 1	PACS1
1559000_at	2.23	chromosome 10 open reading frame 108	C10orf108
211445_x_at	2.23	nascent-polypeptide-associated complex alpha polypeptide pseudogene 1	NACAP1
241164_at	2.23		
220267_at	2.23	keratin 24	KRT24
1559902_at	2.23	megakaryoblastic leukemia (translocation) 1	MKL1
233678_at	2.23	transcription factor 12 (HTF4, helix-loop-helix transcription factors 4)	TCF12
1569490_at	2.22	fibronectin type III domain containing 3B	FNDC3B
238297_at	2.22	phosphatase and actin regulator 1	PHACTR1
228647_at	2.22		
206674_at	2.22	fms-related tyrosine kinase 3	FLT3
243449_at	2.22	oxysterol binding protein 2	OSBP2
236884_at	2.22		
1566251_at	2.22	SH3-domain GRB2-like pseudogene 1	SH3GLP1
212328_at	2.21		
229070_at	2.21	chromosome 6 open reading frame 105	C6orf105
1569312_at	2.21		
223765_s_at	2.21	kelch repeat and BTB (POZ) domain containing 4	KBTBD4
241974_at	2.21		
226322_at	2.20	transmembrane and tetratricopeptide repeat containing 1	TMTC1
215920_s_at	2.20	ceroid-lipofuscinosis, neuronal 3, juvenile (Batten, Spielmeyer-Vogt disease)#eukaryotic translation initiation factor 3, subunit 8, 110kDa#eukaryotic translation initiation factor 3, subunit 8, 110kDa#null#null#null	CLN3#EIF3S8#EIF3S8#null#null#null
1559523_at	2.20		
211548_s_at	2.20	hydroxyprostaglandin dehydrogenase 15-(NAD)	HPGD
232371_at	2.20		
228444_at	2.20		
205472_s_at	2.20	dachshund homolog 1 (Drosophila)	DACH1
233839_at	2.20		
207033_at	2.19	gastric intrinsic factor (vitamin B synthesis)	GIF
217620_s_at	2.19	phosphoinositide-3-kinase, catalytic, beta polypeptide	PIK3CB
233575_s_at	2.19	transducin-like enhancer of split 4 (E(sp1) homolog, Drosophila)	TLE4
226811_at	2.19	family with sequence similarity 46, member C	FAM46C
220234_at	2.19	carbonic anhydrase VIII	CA8
1552827_s_at	2.19	solute carrier family 26, member 7	SLC26A7

237342_at	2.19	toll interacting protein	TOLLIP
215944_at	2.19		
230596_at	2.19		
222352_at	2.18		
216103_at	2.18	acyl-CoA thioesterase 11	ACOT11
208557_at	2.18	homeobox A6	HOXA6
1553326_at	2.18	relaxin/insulin-like family peptide receptor 2	RXFP2
201249_at	2.18	solute carrier family 2 (facilitated glucose transporter), member 1	SLC2A1
238744_at	2.18		
1563203_at	2.18		
227019_at	2.18		
1557772_at	2.17		
216167_at	2.17	leucine rich repeat neuronal 2	LRRN2
232653_at	2.17	trichorhinophalangeal syndrome I	TRPS1
219941_at	2.17	transmembrane protein 19	TMEM19
1563899_at	2.17	lactase-like	LCTL
244231_at	2.17		
1552809_at	2.16	regulatory factor X, 4 (influences HLA class II expression)	RFX4
244845_at	2.16		
227573_s_at	2.16	obscurin-like 1	OBSL1
239825_at	2.16	activating transcription factor 6	ATF6
230336_at	2.16		
232454_at	2.16		
205991_s_at	2.16	paired related homeobox 1	PRRX1
243537_at	2.15	trichoplein, keratin filament binding	TCHP
234303_s_at	2.15	G protein-coupled receptor 85	GPR85
1554474_a_at	2.15	monooxygenase, DBH-like 1	MOXD1
228708_at	2.15		
222573_s_at	2.15	salvador homolog 1 (Drosophila)	SAV1
1558755_x_at	2.15	zinc finger protein 763	ZNF763
1562089_at	2.15	glycine-N-acyltransferase-like 1	GLYATL1
225717_at	2.15	KIAA1715	KIAA1715
231947_at	2.15	myc target 1	MYCT1
232614_at	2.15		
201417_at	2.14	SRY (sex determining region Y)-box 4#null	SOX4#null
206999_at	2.14	interleukin 12 receptor, beta 2	IL12RB2
213313_at	2.14	RAB GTPase activating protein 1	RABGAP1
210119_at	2.14	potassium inwardly-rectifying channel, subfamily J, member 15	KCNJ15
240248_at	2.14		
209211_at	2.14	Kruppel-like factor 5 (intestinal)	KLF5
234472_at	2.13	UDP-N-acetyl-alpha-D-galactosamine:polypeptide N-acetylgalactosaminyltransferase 13 (GalNAc-T13)	GALNT13
204517_at	2.13	peptidylprolyl isomerase C (cyclophilin C)	PPIC
223936_s_at	2.13	forkhead box P1	FOXP1
1553622_a_at	2.13	fibrous sheath interacting protein 1	FSIP1
1556055_at	2.13		
207494_s_at	2.13	zinc finger protein 76 (expressed in testis)	ZNF76
240595_at	2.13		

243427_at	2.12		
243764_at	2.12	V-set and immunoglobulin domain containing 1	VSIG1
222772_at	2.12	myelin expression factor 2	MYEF2
204184_s_at	2.12	adrenergic, beta, receptor kinase 2	ADRBK2
211279_at	2.12	nuclear respiratory factor 1	NRF1
220489_s_at	2.12		
215058_at	2.12		
1554329_x_at	2.11	syntaxin binding protein 4	STXBP4
239567_at	2.11		
234709_at	2.11	calpain 13	CAPN13
1553138_a_at	2.11	ankyrin repeat domain 41	ANKRD41
213541_s_at	2.11	v-ets erythroblastosis virus E26 oncogene homolog (avian)	ERG
232098_at	2.11	dystonin	DST
238224_at	2.11		
223243_s_at	2.11	ER degradation enhancer, mannosidase alpha-like 3	EDEM3
203386_at	2.11	TBC1 domain family, member 4	TBC1D4
1555136_at	2.11	FYVE, RhoGEF and PH domain containing 6	FGD6
226782_at	2.11	solute carrier family 25, member 30	SLC25A30
229116_at	2.11		
234297_at	2.11	programmed cell death 6	PDCD6
1552971_at	2.11	sarcoglycan zeta	SGCZ
235551_at	2.10	WD repeat domain 4	WDR4
216368_s_at	2.10	collagen, type IV, alpha 3 (Goodpasture antigen)	COL4A3
222280_at	2.10	glyceraldehyde-3-phosphate dehydrogenase, spermatogenic	GAPDHS
213824_at	2.10	oligodendrocyte lineage transcription factor 2	OLIG2
1569941_at	2.10		
214651_s_at	2.10	homeobox A9	HOXA9
206022_at	2.10	Norrie disease (pseudoglioma)	NDP
1558028_x_at	2.10		
220586_at	2.10	chromodomain helicase DNA binding protein 9	CHD9
208261_x_at	2.10	interferon, alpha 10	IFNA10
207495_at	2.10	RAB28, member RAS oncogene family	RAB28
1564960_at	2.10	keratin associated protein 7-1	KRTAP7-1
1569527_at	2.10		
240745_at	2.10		
226069_at	2.09	prickle homolog 1 (Drosophila)	PRICKLE1
237009_at	2.09	CD69 molecule	CD69
243319_at	2.09	mediator of RNA polymerase II transcription, subunit 31 homolog (S. cerevisiae)	MED31
225721_at	2.09	synaptopodin 2	SYNPO2
205884_at	2.09	integrin, alpha 4 (antigen CD49D, alpha 4 subunit of VLA-4 receptor)	ITGA4
1555829_at	2.09	family with sequence similarity 62 (C2 domain containing) member B	FAM62B
1562458_at	2.09	ubiquitin-conjugating enzyme E2W (putative)	UBE2W
234953_x_at	2.09	zinc finger protein 19	ZNF19
234690_at	2.09		
1557737_s_at	2.09	natural killer-tumor recognition sequence	NKTR
213728_at	2.09		
215390_at	2.09		

1561389_at	2.09		
217657_at	2.08	B-cell receptor-associated protein 29	BCAP29
1556824_at	2.08		
1552794_a_at	2.08	zinc finger protein 547	ZNF547
1556128_a_at	2.08	Ras protein-specific guanine nucleotide-releasing factor 2	RASGRF2
209921_at	2.08	solute carrier family 7, (cationic amino acid transporter, y+ system) member 11	SLC7A11
211665_s_at	2.08	son of sevenless homolog 2 (Drosophila)	SOS2
214975_s_at	2.08	myotubularin related protein 1	MTMR1
1555120_at	2.08	CD96 molecule	CD96
209576_at	2.08	guanine nucleotide binding protein (G protein), alpha inhibiting activity polypeptide 1	GNAI1
242123_at	2.08	progestin and adipoQ receptor family member VII	PAQR7
228507_at	2.08		
205376_at	2.08	inositol polyphosphate-4-phosphatase, type II, 105kDa	INPP4B
238774_at	2.07	KIAA1267	KIAA1267
1555159_at	2.07	transmembrane protein 74	TMEM74
226733_at	2.07	6-phosphofructo-2-kinase/fructose-2,6-biphosphatase 2	PFKFB2
202236_s_at	2.07	solute carrier family 16, member 1 (monocarboxylic acid transporter 1)	SLC16A1
214932_at	2.07		
226367_at	2.07	jumonji, AT rich interactive domain 1A	JARID1A
243122_at	2.07		
1569854_at	2.07		
239893_at	2.07		
204156_at	2.07		
1555618_s_at	2.07	SUMO1 activating enzyme subunit 1	SAE1
1557238_s_at	2.07		
205941_s_at	2.06	collagen, type X, alpha 1(Schmid metaphyseal chondrodysplasia)	COL10A1
205806_at	2.06	retinal outer segment membrane protein 1	ROM1
214318_s_at	2.06	furry homolog (Drosophila)	FRY
1560141_at	2.06		
209839_at	2.06	dynamin 3	DNM3
225270_at	2.06	neogenin homolog 1 (chicken)	NEO1
216969_s_at	2.06	kinesin family member 22#MYC-associated zinc finger protein (purine-binding transcription factor)#null	KIF22#MAZ#null
1558581_at	2.06	metastasis associated 1	MTA1
214998_at	2.06		
236488_s_at	2.05		
1558391_s_at	2.05	zinc finger protein 599	ZNF599
236435_at	2.05	zinc finger protein 292	ZNF292
213094_at	2.05	G protein-coupled receptor 126	GPR126
239791_at	2.05		
1565821_at	2.05		
214945_at	2.05		
240793_at	2.05	titin	TTN

218865_at	2.05	MOCO sulphurase C-terminal domain containing 1	MOSC1
213079_at	2.05	TSR2, 20S rRNA accumulation, homolog (S. cerevisiae)	TSR2
229287_at	2.05	pecanex homolog (Drosophila)	PCNX
243397_at	2.05		
201917_s_at	2.05	solute carrier family 25, member 36	SLC25A36
216176_at	2.05		
236606_at	2.04		
223006_s_at	2.04	chromosome 9 open reading frame 5	C9orf5
201919_at	2.04	solute carrier family 25, member 36	SLC25A36
215288_at	2.04	transient receptor potential cation channel, subfamily C, member 2	TRPC2
212820_at	2.04	Dmx-like 2	DMXL2
202125_s_at	2.04	trafficking protein, kinesin binding 2	TRAK2
205229_s_at	2.04	coagulation factor C homolog, cochlin (Limulus polyphemus)	COCH
233383_at	2.04	TBC1 domain family, member 5	TBC1D5
228636_at	2.04	basic helix-loop-helix domain containing, class B, 5	BHLHB5
233303_at	2.04		
1569147_at	2.04		
233498_at	2.03	v-erb-a erythroblastic leukemia viral oncogene homolog 4 (avian)	ERBB4
212719_at	2.03	PH domain and leucine rich repeat protein phosphatase	PHLPP
217242_at	2.03	zinc finger protein 154	ZNF154
236047_at	2.03	chromosome 8 open reading frame 5	C8orf5
240367_at	2.03	chromodomain helicase DNA binding protein 9	CHD9
225854_x_at	2.03	interferon regulatory factor 2 binding protein 2	IRF2BP2
224970_at	2.03	nuclear factor I/A	NFIA
238729_x_at	2.03		
231658_x_at	2.02	ribosomal protein L36	RPL36
232921_at	2.02		
213413_at	2.02		
226505_x_at	2.02	ubiquitin specific peptidase 32	USP32
221696_s_at	2.02	serine/threonine/tyrosine kinase 1	STYK1
219628_at	2.02	zinc finger, matrin type 3	ZMAT3
224009_x_at	2.02	dehydrogenase/reductase (SDR family) member 9	DHRS9
1566165_at	2.02		
201656_at	2.02	integrin, alpha 6	ITGA6
243587_x_at	2.02		
206116_s_at	2.02	tropomyosin 1 (alpha)	TPM1
244550_at	2.02	transcription factor Dp-1	TFDP1
212945_s_at	2.02	MAX gene associated	MGA
244622_at	2.02	bromodomain and WD repeat domain containing 1	BRWD1
1555098_a_at	2.02	calcium channel, voltage-dependent, beta 2 subunit	CACNB2
206522_at	2.02	maltase-glucoamylase (alpha-glucosidase)	MGAM
221497_x_at	2.02	egl nine homolog 1 (C. elegans)	EGLN1

237586_at	2.02	epidermal growth factor receptor pathway substrate 15	EPS15
1560006_a_at	2.02		
229309_at	2.01		
215660_s_at	2.01		
204991_s_at	2.01	neurofibromin 2 (bilateral acoustic neuroma)	NF2
203915_at	2.01	chemokine (C-X-C motif) ligand 9	CXCL9
220843_s_at	2.01	WD repeats and SOF1 domain containing	WDSOF1
241147_at	2.01		
235011_at	2.01		
1555154_a_at	2.00	quaking homolog, KH domain RNA binding (mouse)	QKI
214130_s_at	2.00	phosphodiesterase 4D interacting protein (myomegalin)	PDE4DIP
222587_s_at	2.00	UDP-N-acetyl-alpha-D-galactosamine:polypeptide N-acetylgalactosaminyltransferase 7 (GalNAc-T7)	GALNT7
227884_at	2.00	TAF15 RNA polymerase II, TATA box binding protein (TBP)-associated factor, 68kDa	TAF15
1554742_at	2.00	PMS1 postmeiotic segregation increased 1 (S. cerevisiae)	PMS1
1558448_a_at	2.00		
212325_at	2.00		
205401_at	2.00	alkylglycerone phosphate synthase	AGPS
242301_at	2.00	cerebellin 2 precursor	CBLN2
238583_at	2.00	methionine sulfoxide reductase B3	MSRB3
1558019_at	1.99		
202770_s_at	1.99	cyclin G2	CCNG2
235829_at	1.99		
224952_at	1.99	tetratricopeptide repeat, ankyrin repeat and coiled-coil containing 2	TANC2
233614_at	1.99		
230650_at	1.99		
219750_at	1.99	transmembrane protein 144	TMEM144
236782_at	1.99	sterile alpha motif domain containing 3	SAMD3
1563226_at	1.99		
210867_at	1.99	CCR4-NOT transcription complex, subunit 4	CNOT4
1569472_s_at	1.99	tetratricopeptide repeat domain 3	TTC3
32128_at	1.98	chemokine (C-C motif) ligand 18 (pulmonary and activation-regulated)	CCL18
209635_at	1.98	adaptor-related protein complex 1, sigma 1 subunit	AP1S1
206574_s_at	1.98	protein tyrosine phosphatase type IVA, member 3	PTP4A3
1553315_at	1.98	schlafen-like 1	SLFNL1
244452_at	1.98	chromosome 2 open reading frame 30	C2orf30
242590_at	1.98		
228771_at	1.98	adrenergic, beta, receptor kinase 2	ADRBK2
233251_at	1.98	spermatid perinuclear RNA binding protein	STRBP
1552671_a_at	1.98	solute carrier family 9 (sodium/hydrogen exchanger), member 7	SLC9A7
232324_x_at	1.98		

222572_at	1.98	protein phosphatase 2C, magnesium-dependent, catalytic subunit	PPM2C
1555074_a_at	1.97	potassium voltage-gated channel, subfamily H (eag-related), member 5	KCNH5
222747_s_at	1.97	sex comb on midleg-like 1 (Drosophila)	SCML1
1569192_at	1.97		
1561181_at	1.97		
234276_at	1.97		
203811_s_at	1.97	DnaJ (Hsp40) homolog, subfamily B, member 4	DNAJB4
1564707_x_at	1.97	glutaminase 2 (liver, mitochondrial)	GLS2
1555775_a_at	1.97	zygote arrest 1	ZAR1
213069_at	1.97	HEG homolog 1 (zebrafish)	HEG1
228496_s_at	1.97	cysteine rich transmembrane BMP regulator 1 (chordin-like)	CRIM1
1557543_at	1.97		
236621_at	1.96		
206721_at	1.96	chromosome 1 open reading frame 114	C1orf114
202241_at	1.96	tribbles homolog 1 (Drosophila)	TRIB1
230610_at	1.96		
235684_s_at	1.96	sestrin 3	SESN3
231150_at	1.96		
218276_s_at	1.96	salvador homolog 1 (Drosophila)	SAV1
217056_at	1.96		
1566927_at	1.96	chromosome 21 open reading frame 104	C21orf104
215976_at	1.96		
229844_at	1.96		
234033_at	1.96	Rap guanine nucleotide exchange factor (GEF) 2	RAPGEF2
204529_s_at	1.96		
205307_s_at	1.95	kynurenine 3-monooxygenase (kynurenine 3-hydroxylase)	KMO
1555489_at	1.95		
224237_at	1.95		
227846_at	1.95	G protein-coupled receptor 176	GPR176
205352_at	1.95	serpin peptidase inhibitor, clade I (neuroserpin), member 1	SERPINI1
215200_x_at	1.95		
228190_at	1.95	ATG4 autophagy related 4 homolog C (S. cerevisiae)	ATG4C
215083_at	1.95		
1564970_at	1.95	SET domain, bifurcated 2	SETDB2
227197_at	1.95		
242673_at	1.94	ubiquitin protein ligase E3C	UBE3C
237578_at	1.94		
219721_at	1.94		
232007_at	1.94	1-acylglycerol-3-phosphate O-acyltransferase 5 (lysophosphatidic acid acyltransferase, epsilon)	AGPAT5
216550_x_at	1.93	ankyrin repeat domain 12	ANKRD12
223690_at	1.93	latent transforming growth factor beta binding protein 2	LTBP2
228686_at	1.93		
227754_at	1.93		
215626_at	1.93		

231981_at	1.93	prolactin receptor	PRLR
226497_s_at	1.93		
232270_at	1.92	chromosome 9 open reading frame 3	C9orf3
1569706_at	1.92	myb-like, SWIRM and MPN domains 1	MYSM1
1555594_a_at	1.92	muscleblind-like (Drosophila)	MBNL1
1569433_at	1.91		
1554328_at	1.91	syntaxin binding protein 4	STXBP4
217671_at	1.91		
1552970_s_at	1.91	zinc finger, MYM-type 6	ZMYM6
1558556_at	1.91	calcium/calmodulin-dependent protein kinase I	CAMK1
218692_at	1.90		
205934_at	1.90	phospholipase C-like 1	PLCL1
237882_at	1.89		
1553216_at	1.89	zinc finger protein 41	ZNF41
1559665_at	1.89		
239705_at	1.88		
213510_x_at	1.88		
215063_x_at	1.88	leucine rich repeat containing 40	LRRC40
212450_at	1.88		
212217_at	1.88	prolyl endopeptidase-like	PREPL
1556944_at	1.87		
219239_s_at	1.87	zinc finger protein 654	ZNF654
222727_s_at	1.87	solute carrier family 24 (sodium/potassium/calcium exchanger), member 6	SLC24A6
231852_at	1.87	three prime histone mRNA exonuclease 1	THEX1
1561615_s_at	1.86	solute carrier family 8 (sodium/calcium exchanger), member 1	SLC8A1
1568448_at	1.86		
211549_s_at	1.85	hydroxyprostaglandin dehydrogenase 15-(NAD)	HPGD
1555929_s_at	1.85		
238608_at	1.85		
215316_at	1.85		
208434_at	1.84	myelodysplasia syndrome 1	MDS1
1564215_at	1.84		
210815_s_at	1.84	calcitonin receptor-like	CALCRL
234979_at	1.84		
237845_at	1.82		
226778_at	1.78	chromosome 8 open reading frame 42	C8orf42
243995_at	1.78	protein prenyltransferase alpha subunit repeat containing 1	PTAR1
218959_at	1.77	homeobox C10	HOXC10
1553316_at	1.74	G protein-coupled receptor 82	GPR82
234491_s_at	1.74	salvador homolog 1 (Drosophila)	SAV1
237066_at	1.73		
1552391_at	1.71	chromosome 1 open reading frame 65	C1orf65
215294_s_at	1.71	SWI/SNF related, matrix associated, actin dependent regulator of chromatin, subfamily a, member 1	SMARCA1
1552830_at	1.65	F-box protein 39	FBXO39
204175_at	0.53	zinc finger protein 593	ZNF593
204684_at	0.53	neuronal pentraxin I	NPTX1

221023_s_at	0.53	potassium voltage-gated channel, subfamily H (eag-related), member 6	KCNH6
209160_at	0.53	aldo-keto reductase family 1, member C3 (3-alpha hydroxysteroid dehydrogenase, type II)	AKR1C3
221960_s_at	0.52	RAB2A, member RAS oncogene family	RAB2A
232785_at	0.52	retrotransposon gag domain containing 1	RGAG1
219316_s_at	0.52	chromosome 14 open reading frame 58	C14orf58
224052_at	0.52	heat shock transcription factor, Y-linked 1	HSFY1
230170_at	0.52	oncostatin M	OSM
234665_x_at	0.52	HERV-H LTR-associating 3	HHLA3
210873_x_at	0.52	apolipoprotein B mRNA editing enzyme, catalytic polypeptide-like 3A	APOBEC3A
205935_at	0.52	forkhead box F1	FOXF1
222173_s_at	0.52	TBC1 domain family, member 2	TBC1D2
218675_at	0.52	solute carrier family 22 (organic cation transporter), member 17	SLC22A17
1555019_at	0.52	protocadherin 21	PCDH21
226554_at	0.52	zinc finger and BTB domain containing 7A	ZBTB7A
241421_at	0.52		
229848_at	0.52	zinc finger protein 10	ZNF10
218226_s_at	0.52	NADH dehydrogenase (ubiquinone) 1 beta subcomplex, 4, 15kDa	NDUFB4
204319_s_at	0.52	regulator of G-protein signalling 10	RGS10
213619_at	0.52	heterogeneous nuclear ribonucleoprotein H1 (H)	HNRPH1
236667_at	0.52	ras homolog gene family, member F (in filopodia)	RHOF
1556107_at	0.52		
1562107_at	0.52		
237086_at	0.52	forkhead box A1	FOXA1
222996_s_at	0.51	CXXC finger 5	CXXC5
216322_at	0.51	CD58 molecule	CD58
231954_at	0.51		
208733_at	0.51	RAB2A, member RAS oncogene family	RAB2A
205590_at	0.51	RAS guanyl releasing protein 1 (calcium and DAG-regulated)	RASGRP1
220118_at	0.51	zinc finger and BTB domain containing 32	ZBTB32
229437_at	0.51		
202948_at	0.51	interleukin 1 receptor, type I	IL1R1
1562341_at	0.51		
238389_s_at	0.51		
229228_at	0.51	cAMP responsive element binding protein 5	CREB5
223681_s_at	0.51	InaD-like (Drosophila)	INADL
234403_at	0.51	ilvB (bacterial acetolactate synthase)-like#synapse defective 1, Rho GTPase, homolog 1 (C. elegans)#olfactory receptor, family 1, subfamily I, member 1#olfactory receptor, family 10, subfamily B, member 1 pseudogene	ILVBL#SYDE1#OR1I1#OR10B1P
225373_at	0.51	chromosome 10 open reading frame 54	C10orf54
219978_s_at	0.51	nucleolar and spindle associated protein 1	NUSAP1
204032_at	0.51	breast cancer anti-estrogen resistance 3	BCAR3
232880_at	0.51		
237702_at	0.51		

200036_s_at	0.51	ribosomal protein L10a	RPL10A
203927_at	0.51	nuclear factor of kappa light polypeptide gene enhancer in B-cells inhibitor, epsilon	NFKBIE
223398_at	0.51	chromosome 9 open reading frame 89	C9orf89
201995_at	0.51	exostoses (multiple) 1	EXT1
225214_at	0.51		
209917_s_at	0.51	TP53 activated protein 1	TP53AP1
209392_at	0.51	ectonucleotide pyrophosphatase/phosphodiesterase 2 (autotaxin)	ENPP2
209824_s_at	0.51	aryl hydrocarbon receptor nuclear translocator-like	ARNTL
204230_s_at	0.51	solute carrier family 17 (sodium-dependent inorganic phosphate cotransporter), member 7	SLC17A7
229455_at	0.51		
225751_at	0.51	RNA binding motif protein 17	RBM17
225102_at	0.51	monoglyceride lipase	MGLL
225339_at	0.51		
242451_x_at	0.51	ribosomal protein S19	RPS19
230288_at	0.51	fibroblast growth factor 14	FGF14
213348_at	0.51	cyclin-dependent kinase inhibitor 1C (p57, Kip2)	CDKN1C
1557233_at	0.51		
217419_x_at	0.51	agrin	AGRIN
207100_s_at	0.51	vesicle-associated membrane protein 1 (synaptobrevin 1)	VAMP1
213326_at	0.51	vesicle-associated membrane protein 1 (synaptobrevin 1)	VAMP1
1568768_s_at	0.51	brain and reproductive organ-expressed (TNFRSF1A modulator)	BRE
235174_s_at	0.51		
1553394_a_at	0.50	transcription factor AP-2 beta (activating enhancer binding protein 2 beta)	TFAP2B
216216_at	0.50	slit homolog 3 (Drosophila)	SLIT3
212845_at	0.50	sterile alpha motif domain containing 4A	SAMD4A
242949_x_at	0.50		
228398_at	0.50	SUMO/sentrin specific peptidase family member 8	SENP8
228832_at	0.50		
210162_s_at	0.50	nuclear factor of activated T-cells, cytoplasmic, calcineurin-dependent 1	NFATC1
209357_at	0.50	Cbp/p300-interacting transactivator, with Glu/Asp-rich carboxy-terminal domain, 2	CITED2
204070_at	0.50	retinoic acid receptor responder (tazarotene induced) 3	RARRES3
223377_x_at	0.50	cytokine inducible SH2-containing protein	CISH
206932_at	0.50	cholesterol 25-hydroxylase	CH25H
203085_s_at	0.50	transforming growth factor, beta 1 (Camurati-Engelmann disease)	TGFB1
235380_at	0.50		
243834_at	0.50	trinucleotide repeat containing 6A	TNRC6A
239744_at	0.50		
204212_at	0.50	acyl-CoA thioesterase 8	ACOT8
1553426_at	0.50		

216895_at	0.50	gamma-aminobutyric acid (GABA) A receptor, gamma 3	GABRG3
200017_at	0.50	ribosomal protein S27a	RPS27A
1562446_at	0.50		
233352_at	0.50		
215464_s_at	0.50	Tax1 (human T-cell leukemia virus type I) binding protein 3	TAX1BP3
218205_s_at	0.50	MAP kinase interacting serine/threonine kinase 2	MKNK2
201531_at	0.50	zinc finger protein 36, C3H type, homolog (mouse)	ZFP36
204745_x_at	0.50	metallothionein 1G	MT1G
238317_x_at	0.50	RNA binding motif, single stranded interacting protein 1	RBMS1
1569580_a_at	0.50		
1561247_at	0.50		
1554591_at	0.50		
218880_at	0.50	FOS-like antigen 2	FOSL2
1556306_at	0.50		
200920_s_at	0.50	B-cell translocation gene 1, anti-proliferative	BTG1
224030_s_at	0.50		
223502_s_at	0.50	tumor necrosis factor (ligand) superfamily, member 13b	TNFSF13B
216160_at	0.50		
1565628_at	0.50		
230790_x_at	0.50	checkpoint suppressor 1	CHES1
230598_at	0.50		
223526_at	0.50	chromosome 18 open reading frame 21	C18orf21
243756_at	0.50	thrombospondin, type I, domain containing 7A	THSD7A
218297_at	0.50	chromosome 10 open reading frame 97	C10orf97
218157_x_at	0.50	CDC42 small effector 1	CDC42SE1
215706_x_at	0.50	zyxin	ZYX
237161_at	0.49		
1562981_at	0.49	hemoglobin, beta	HBB
1560821_at	0.49	Rho GTPase activating protein 22	ARHGAP22
229143_at	0.49	CCR4-NOT transcription complex, subunit 3	CNOT3
208581_x_at	0.49	metallothionein 1X	MT1X
216509_x_at	0.49	myeloid/lymphoid or mixed-lineage leukemia (trithorax homolog, Drosophila); translocated to, 10	MLLT10
208540_x_at	0.49	S100 calcium binding protein A11	S100A11
233770_at	0.49		
239423_at	0.49		
1564134_at	0.49		
209544_at	0.49	receptor-interacting serine-threonine kinase 2	RIPK2
237083_at	0.49		
1565728_at	0.49		
236465_at	0.49	ring finger protein 175	RNF175
201403_s_at	0.49	microsomal glutathione S-transferase 3	MGST3
215810_x_at	0.49		
205249_at	0.49	early growth response 2 (Krox-20 homolog, Drosophila)	EGR2
242732_at	0.49	metastasis suppressor 1	MTSS1

1558834_s_at	0.49	chromosome 1 open reading frame 62	C1orf62
1558934_a_at	0.49		
221159_at	0.49		
242126_at	0.49		
221530_s_at	0.49	basic helix-loop-helix domain containing, class B, 3	BHLHB3
236085_at	0.49	calcyphosine-like	CAPSL
1555131_a_at	0.49	period homolog 3 (Drosophila)	PER3
1558404_at	0.49		
213281_at	0.49	jun oncogene	JUN
244637_at	0.49		
219191_s_at	0.49	bridging integrator 2	BIN2
227099_s_at	0.49		
221221_s_at	0.49	kelch-like 3 (Drosophila)	KLHL3
1566477_at	0.49		
215451_s_at	0.49	AF4/FMR2 family, member 1	AFF1
1552675_at	0.49	DnaJ (Hsp40) homolog, subfamily B, member 7	DNAJB7
216821_at	0.49	Sec61 beta subunit#asparagine-linked glycosylation 2 homolog (S. cerevisiae, alpha-1,3-mannosyltransferase)#keratin 8-like 1	SEC61B#ALG2#KRT8L1
219288_at	0.49	chromosome 3 open reading frame 14	C3orf14
239503_at	0.49		
215516_at	0.49	laminin, beta 4	LAMB4
206337_at	0.49	chemokine (C-C motif) receptor 7	CCR7
207597_at	0.49	ADAM metallopeptidase domain 18	ADAM18
230591_at	0.49		
228092_at	0.49	cAMP responsive element modulator	CREM
241353_s_at	0.49		
209774_x_at	0.49	chemokine (C-X-C motif) ligand 2	CXCL2
234871_at	0.49	G protein-coupled receptor 98	GPR98
217371_s_at	0.49	interleukin 15	IL15
239953_at	0.48		
219648_at	0.48	melanoregulin	MREG
1569194_at	0.48	zinc finger protein 789	ZNF789
207980_s_at	0.48	Cbp/p300-interacting transactivator, with Glu/Asp-rich carboxy-terminal domain, 2	CITED2
204059_s_at	0.48	malic enzyme 1, NADP(+)-dependent, cytosolic	ME1
238725_at	0.48		
1553275_s_at	0.48		
214121_x_at	0.48	PDZ and LIM domain 7 (enigma)	PDLIM7
239219_at	0.48	aurora kinase B	AURKB
204198_s_at	0.48	runt-related transcription factor 3	RUNX3
205032_at	0.48	integrin, alpha 2 (CD49B, alpha 2 subunit of VLA-2 receptor)	ITGA2
227621_at	0.48	Wilms tumor 1 associated protein	WTAP
210933_s_at	0.48	fascin homolog 1, actin-bundling protein (Strongylocentrotus purpuratus)	FSCN1
204066_s_at	0.48	centaurin, gamma 2	CENTG2
1558692_at	0.48	chromosome 1 open reading frame 85	C1orf85
227651_at	0.48	BTB (POZ) domain containing 14B	BTBD14B
209066_x_at	0.48	ubiquinol-cytochrome c reductase binding protein	UQCRB

218368_s_at	0.48	tumor necrosis factor receptor superfamily, member 12A	TNFRSF12A
206940_s_at	0.48	POU domain, class 4, transcription factor 1	POU4F1
229391_s_at	0.48		
1568882_at	0.48	leucine rich repeat containing 51	LRRC51
204789_at	0.48	formin-like 1	FMNL1
1552423_at	0.48	ets variant gene 3	ETV3
225612_s_at	0.48	UDP-GlcNAc:betaGal beta-1,3-N-acetylglucosaminyltransferase 5	B3GNT5
1566342_at	0.48	superoxide dismutase 2, mitochondrial	SOD2
1564010_at	0.48	calpastatin	CAST
231435_at	0.48	chromosome 7 open reading frame 34	C7orf34
1552381_at	0.48		
206461_x_at	0.48	metallothionein 1H	MT1H
204794_at	0.48	dual specificity phosphatase 2	DUSP2
1556666_a_at	0.48	tetratricopeptide repeat domain 6	TTC6
203708_at	0.48	phosphodiesterase 4B, cAMP-specific (phosphodiesterase E4 dunce homolog, Drosophila)	PDE4B
226382_at	0.48		
203749_s_at	0.48	retinoic acid receptor, alpha	RARA
1554710_at	0.48	potassium large conductance calcium-activated channel, subfamily M, beta member 1	KCNMB1
231610_at	0.48	tau tubulin kinase 2	TTBK2
222329_x_at	0.48		
202350_s_at	0.48	matrilin 2	MATN2
213298_at	0.48	nuclear factor I/C (CCAAT-binding transcription factor)	NFIC
211883_x_at	0.48	carcinoembryonic antigen-related cell adhesion molecule 1 (biliary glycoprotein)	CEACAM1
226530_at	0.48	Bcl2 modifying factor	BMF
230291_s_at	0.47		
234179_at	0.47		
244058_at	0.47	chromosome 10 open reading frame 72	C10orf72
236559_at	0.47	tyrosine 3-monooxygenase/tryptophan 5-monooxygenase activation protein, eta polypeptide	YWHAH
240847_at	0.47	reelin	RELN
201860_s_at	0.47	plasminogen activator, tissue	PLAT
1554274_a_at	0.47	slingshot homolog 1 (Drosophila)	SSH1
1561469_at	0.47		
204958_at	0.47	polo-like kinase 3 (Drosophila)	PLK3
209656_s_at	0.47	transmembrane protein 47	TMEM47
205226_at	0.47	platelet-derived growth factor receptor-like	PDGFRL
219456_s_at	0.47	Ras and Rab interactor 3	RIN3
232200_at	0.47	heat shock 70kDa protein 9 (mortalin)	HSPA9
208579_x_at	0.47	H2B histone family, member S	H2BFS
209963_s_at	0.47	erythropoietin receptor	EPOR
235156_at	0.47		
224533_s_at	0.47	interferon, alpha-inducible protein 6	IFI6
204567_s_at	0.47	ATP-binding cassette, sub-family G (WHITE), member 1	ABCG1
222139_at	0.47		

1569716_at	0.47		
236213_at	0.47		
238193_at	0.47		
1553418_a_at	0.47	contactin associated protein-like 5	CNTNAP5
236253_at	0.47	zinc finger protein 546	ZNF546
223961_s_at	0.47	cytokine inducible SH2-containing protein	CISH
1559807_at	0.47		
232081_at	0.47		
1559766_at	0.47		
231598_x_at	0.47		
205440_s_at	0.47	neuropeptide Y receptor Y1	NPY1R
243290_at	0.47	WW and C2 domain containing 1	WWC1
1554593_s_at	0.47	solute carrier family 1 (high affinity aspartate/glutamate transporter), member 6	SLC1A6
204667_at	0.47	forkhead box A1	FOXA1
1553380_at	0.47	poly (ADP-ribose) polymerase family, member 15	PARP15
207826_s_at	0.47	inhibitor of DNA binding 3, dominant negative helix-loop-helix protein	ID3
207882_at	0.47		
1564690_at	0.47		
1565735_at	0.46		
1553194_at	0.46	neuronal growth regulator 1	NEGR1
229934_at	0.46		
1565579_at	0.46		
45687_at	0.46	proline rich 14	PRR14
222067_x_at	0.46	histone cluster 1, H1e#histone cluster 1, H2bd#histone cluster 1, H2be#histone cluster 1, H2bc#histone cluster 1, H4d	HIST1H1E#HIST1H2BD#HIST1H2BE#HIST1H2BC#HIST1H4D
201565_s_at	0.46	inhibitor of DNA binding 2, dominant negative helix-loop-helix protein	ID2
239272_at	0.46	matrix metallopeptidase 28	MMP28
201502_s_at	0.46	nuclear factor of kappa light polypeptide gene enhancer in B-cells inhibitor, alpha	NFKBIA
229120_s_at	0.46	CDC42 small effector 1	CDC42SE1
202949_s_at	0.46	four and a half LIM domains 2	FHL2
243186_at	0.46	amyotrophic lateral sclerosis 2 (juvenile) chromosome region, candidate 13	ALS2CR13
215475_at	0.46		
1552301_a_at	0.46	coronin 6	CORO6
236861_at	0.46	THO complex 7 homolog (Drosophila)	THOC7
203068_at	0.46	kelch-like 21 (Drosophila)	KLHL21
216189_at	0.46		
237106_at	0.46	solute carrier family 11 (proton-coupled divalent metal ion transporters), member 2	SLC11A2
203505_at	0.46	ATP-binding cassette, sub-family A (ABC1), member 1	ABCA1
204166_at	0.46	strawberry notch homolog 2 (Drosophila)	SBNO2
201367_s_at	0.46	zinc finger protein 36, C3H type-like 2	ZFP36L2
219508_at	0.46	glucosaminyl (N-acetyl) transferase 3, mucin type	GCNT3

200660_at	0.46	S100 calcium binding protein A11	S100A11
235944_at	0.46	hemicentin 1	HMCN1
222762_x_at	0.46	LIM domains containing 1	LIMD1
201105_at	0.46	lectin, galactoside-binding, soluble, 1 (galectin 1)	LGALS1
1568785_a_at	0.46		
1564475_s_at	0.46		
217849_s_at	0.46	CDC42 binding protein kinase beta (DMPK-like)	CDC42BPB
200925_at	0.46	cytochrome c oxidase subunit VIa polypeptide 1	COX6A1
214469_at	0.46	histone cluster 1, H2ae	HIST1H2AE
228982_s_at	0.46	ubiquitin specific peptidase 42	USP42
240372_at	0.46		
221016_s_at	0.46	transcription factor 7-like 1 (T-cell specific, HMG-box)	TCF7L1
233040_at	0.46	pleckstrin homology domain containing, family A member 5	PLEKHA5
234437_at	0.46		
209651_at	0.46	transforming growth factor beta 1 induced transcript 1	TGFB1I1
223514_at	0.46	caspase recruitment domain family, member 11	CARD11
217729_s_at	0.46	amino-terminal enhancer of split	AES
241314_at	0.46		
212285_s_at	0.46	agrin	AGRIN
224027_at	0.46	chemokine (C-C motif) ligand 28	CCL28
233231_at	0.46	methylcrotonoyl-Coenzyme A carboxylase 2 (beta)	MCCC2
209498_at	0.46	carcinoembryonic antigen-related cell adhesion molecule 1 (biliary glycoprotein)	CEACAM1
227314_at	0.46	integrin, alpha 2 (CD49B, alpha 2 subunit of VLA-2 receptor)	ITGA2
204280_at	0.46	regulator of G-protein signalling 14	RGS14
242197_x_at	0.46	CD36 molecule (thrombospondin receptor)	CD36
210948_s_at	0.46	lymphoid enhancer-binding factor 1	LEF1
202510_s_at	0.45	tumor necrosis factor, alpha-induced protein 2	TNFAIP2
1557576_at	0.45		
214720_x_at	0.45	septin 10	10-Sep
33304_at	0.45	interferon stimulated exonuclease gene 20kDa	ISG20
240004_at	0.45		
208553_at	0.45	histone cluster 1, H1e	HIST1H1E
232556_at	0.45		
231244_at	0.45	CAS1 domain containing 1	CASD1
211658_at	0.45	peroxiredoxin 2	PRDX2
209949_at	0.45	neutrophil cytosolic factor 2 (65kDa, chronic granulomatous disease, autosomal 2)	NCF2
234844_at	0.45	zinc finger protein 407	ZNF407
214414_x_at	0.45	hemoglobin, alpha 1	HBA1
228791_at	0.45	chromosome 15 open reading frame 38	C15orf38
208937_s_at	0.45	inhibitor of DNA binding 1, dominant negative helix-loop-helix protein	ID1
212698_s_at	0.45	septin 10	10-Sep
218330_s_at	0.45	neuron navigator 2	NAV2
208092_s_at	0.45	family with sequence similarity 49, member A	FAM49A

1554592_a_at	0.45	solute carrier family 1 (high affinity aspartate/glutamate transporter), member 6	SLC1A6
1563357_at	0.45		
210845_s_at	0.45	plasminogen activator, urokinase receptor	PLAUR
209278_s_at	0.45	tissue factor pathway inhibitor 2	TFPI2
1561096_at	0.45		
209387_s_at	0.45	transmembrane 4 L six family member 1	TM4SF1
244569_at	0.45		
208446_s_at	0.45	zinc finger, FYVE domain containing 9	ZFYVE9
217962_at	0.45	nucleolar protein family A, member 3 (H/ACA small nucleolar RNPs)	NOLA3
224792_at	0.45	tankyrase 1 binding protein 1, 182kDa	TNKS1BP1
227855_at	0.45	zinc finger protein 219	ZNF219
213825_at	0.45	oligodendrocyte lineage transcription factor 2	OLIG2
1562591_a_at	0.45	orofacial cleft 1 candidate 1	OFCC1
205239_at	0.45	amphiregulin (schwannoma-derived growth factor)	AREG
201627_s_at	0.45	insulin induced gene 1	INSIG1
231040_at	0.45		
205681_at	0.45	BCL2-related protein A1	BCL2A1
201041_s_at	0.45	dual specificity phosphatase 1	DUSP1
222450_at	0.45	phosphoenolpyruvate carboxykinase 1 (soluble)#transmembrane, prostate androgen induced RNA#Z-DNA binding protein 1#CCCTC-binding factor (zinc finger protein)-like	PCK1#TMEPAI #ZBP1#CTCFL
240609_at	0.45		
1569917_at	0.45		
235739_at	0.45		
203760_s_at	0.45	Src-like-adaptor	SLA
239749_at	0.45	Fas (TNFRSF6) associated factor 1	FAF1
201369_s_at	0.45	zinc finger protein 36, C3H type-like 2	ZFP36L2
1559399_s_at	0.45	zinc finger, CCHC domain containing 10	ZCCHC10
237498_at	0.45	neural precursor cell expressed, developmentally down-regulated 4-like	NEDD4L
212657_s_at	0.44	interleukin 1 receptor antagonist	IL1RN
208077_at	0.44	chromosome 9 open reading frame 38	C9orf38
216680_s_at	0.44	EPH receptor B4	EPHB4
241159_x_at	0.44	integrin alpha FG-GAP repeat containing 1	ITFG1
201473_at	0.44	jun B proto-oncogene	JUNB
1561121_at	0.44		
1559394_a_at	0.44		
239983_at	0.44	solute carrier family 30 (zinc transporter), member 8	SLC30A8
1554844_at	0.44	eyes absent homolog 3 (Drosophila)	EYA3
226212_s_at	0.44		
211025_x_at	0.44	cytochrome c oxidase subunit Vb	COX5B
205330_at	0.44	meningioma (disrupted in balanced translocation) 1	MN1
203216_s_at	0.44	myosin VI	MYO6
230546_at	0.44	vasohibin 1	VASH1
237912_at	0.44	DnaJ (Hsp40) homolog, subfamily A, member 4	DNAJA4
236272_at	0.44		
243004_at	0.44		

201645_at	0.44	tenascin C (hexabrachion)	TNC
235082_at	0.44		
237981_at	0.44	cardiomyopathy associated 5	CMYA5
240850_at	0.44	dual-specificity tyrosine-(Y)-phosphorylation regulated kinase 1A	DYRK1A
233714_at	0.44	aryl hydrocarbon receptor nuclear translocator	ARNT
206839_at	0.44	chromosome 22 open reading frame 31	C22orf31
240267_at	0.44	synaptotagmin VI	SYT6
227200_at	0.44		
223402_at	0.44	dual specificity phosphatase 23	DUSP23
215642_at	0.44		
210504_at	0.44	Kruppel-like factor 1 (erythroid)	KLF1
206683_at	0.44	zinc finger protein 165	ZNF165
218280_x_at	0.44	histone cluster 2, H2aa3	HIST2H2AA3
1562850_at	0.44		
231887_s_at	0.44	KIAA1274	KIAA1274
220148_at	0.44	aldehyde dehydrogenase 8 family, member A1	ALDH8A1
231187_at	0.44	solute carrier family 28 (sodium-coupled nucleoside transporter), member 1	SLC28A1
243974_at	0.44		
232951_at	0.44		
231426_at	0.44		
222941_at	0.44		
216791_at	0.44	transmembrane protein 92	TMEM92
1558217_at	0.43	schlafen family member 13	SLFN13
237244_at	0.43		
1553373_at	0.43	WD repeat domain 64	WDR64
1566609_at	0.43		
211030_s_at	0.43	solute carrier family 6 (neurotransmitter transporter, taurine), member 6	SLC6A6
239522_at	0.43	interleukin 12 receptor, beta 1	IL12RB1
241889_at	0.43		
217340_at	0.43	leucine rich repeat containing 16	LRRC16
225288_at	0.43		
222336_at	0.43	chromosome 4 open reading frame 34	C4orf34
222877_at	0.43		
204908_s_at	0.43	B-cell CLL/lymphoma 3	BCL3
210044_s_at	0.43	lymphoblastic leukemia derived sequence 1	LYL1
215302_at	0.43		
218651_s_at	0.43	La ribonucleoprotein domain family, member 6	LARP6
244407_at	0.43	cytochrome P450, family 39, subfamily A, polypeptide 1	CYP39A1
1560637_at	0.43		
235163_at	0.43	MOB1, Mps One Binder kinase activator-like 2A (yeast)	MOBKL2A
222942_s_at	0.43	T-cell lymphoma invasion and metastasis 2	TIAM2
210483_at	0.43		
226034_at	0.43		
238464_at	0.43	KIAA1641	KIAA1641
230727_at	0.43	polycomb group ring finger 2	PCGF2
50221_at	0.43	transcription factor EB	TFEB
1560153_at	0.43	Fraser syndrome 1	FRAS1
207768_at	0.43	early growth response 4	EGR4

229543_at	0.43		
1557260_a_at	0.43	zinc finger protein 382	ZNF382
202149_at	0.43	neural precursor cell expressed, developmentally down-regulated 9#null#null	NEDD9#null#null
208217_at	0.43	gamma-aminobutyric acid (GABA) receptor, rho 2	GABRR2
212124_at	0.43	zinc finger, MIZ-type containing 1	ZMIZ1
226716_at	0.43	proline rich 12	PRR12
1558702_at	0.43	inversin	INVS
208472_at	0.43	IKAROS family zinc finger 4 (Eos)	IKZF4
214482_at	0.43	zinc finger and BTB domain containing 25	ZBTB25
210534_s_at	0.42		
1563299_at	0.42		
237480_at	0.42		
234565_x_at	0.42		
204446_s_at	0.42	arachidonate 5-lipoxygenase	ALOX5
234221_at	0.42	breast carcinoma amplified sequence 1	BCAS1
234020_x_at	0.42		
221035_s_at	0.42	testis expressed sequence 14	TEX14
237753_at	0.42		
238595_at	0.42		
238316_at	0.42	zinc finger protein 567	ZNF567
222529_at	0.42	solute carrier family 25, member 37	SLC25A37
1569525_s_at	0.42		
1563519_at	0.42		
243116_at	0.42	phosphatidylinositol-4-phosphate 5-kinase-like 1	PIP5KL1
211818_s_at	0.42	phosphodiesterase 4C, cAMP-specific (phosphodiesterase E1 dunce homolog, Drosophila)	PDE4C
234605_at	0.42	CDC14 cell division cycle 14 homolog B (S. cerevisiae)	CDC14B
215250_at	0.42	transmembrane protein 111	TMEM111
237884_x_at	0.42	transient receptor potential cation channel, subfamily M, member 7	TRPM7
214696_at	0.42		
226679_at	0.42	solute carrier family 26, member 11	SLC26A11
239958_at	0.42		
228618_at	0.42		
215801_at	0.42		
223963_s_at	0.42	insulin-like growth factor 2 mRNA binding protein 2	IGF2BP2
237597_at	0.42		
1570484_at	0.42		
239307_at	0.42	myosin, heavy chain 11, smooth muscle	MYH11
242481_at	0.42		
216746_at	0.42		
1554631_at	0.42	ataxia telangiectasia mutated (includes complementation groups A, C and D)	ATM
1555718_x_at	0.42		
209803_s_at	0.42	pleckstrin homology-like domain, family A, member 2	PHLDA2
241130_at	0.42		
1563900_at	0.42	family with sequence similarity 83, member B	FAM83B

242930_at	0.42	O-sialoglycoprotein endopeptidase	OSGEP
217767_at	0.42	complement component 3	C3
1557726_at	0.41	armadillo repeat containing, X-linked 4	ARMCX4
219159_s_at	0.41	SLAM family member 7	SLAMF7
209723_at	0.41	serpin peptidase inhibitor, clade B (ovalbumin), member 9	SERPINB9
204452_s_at	0.41	frizzled homolog 1 (Drosophila)	FZD1
216635_at	0.41		
210376_x_at	0.41	ELK1, member of ETS oncogene family	ELK1
1560023_x_at	0.41		
233865_at	0.41		
222031_at	0.41		
205180_s_at	0.41	ADAM metallopeptidase domain 8	ADAM8
210730_s_at	0.41	neuropeptide Y receptor Y2	NPY2R
1569960_at	0.41		
240232_at	0.41	chromosome 3 open reading frame 1	C3orf1
230052_s_at	0.41		
242385_at	0.41	RAR-related orphan receptor B	RORB
203851_at	0.41	insulin-like growth factor binding protein 6	IGFBP6
1564075_a_at	0.41		
227965_at	0.41		
216598_s_at	0.41	chemokine (C-C motif) ligand 2	CCL2
244507_at	0.41		
1555759_a_at	0.41	chemokine (C-C motif) ligand 5	CCL5
206172_at	0.41	interleukin 13 receptor, alpha 2	IL13RA2
236161_at	0.41		
241581_at	0.41		
1552876_at	0.41	chromosome 21 open reading frame 89	C21orf89
236065_at	0.41		
236525_at	0.41	F-box protein 36	FBXO36
233008_at	0.41		
1562278_at	0.41	dynein, axonemal, heavy chain 1	DNAH1
220091_at	0.41	solute carrier family 2 (facilitated glucose transporter), member 6	SLC2A6
224012_at	0.41	ankyrin repeat domain 20 family, member A1	ANKRD20A1
1553765_a_at	0.41	kelch-like 32 (Drosophila)	KLHL32
205283_at	0.41	Fukuyama type congenital muscular dystrophy (fukutin)	FCMD
241605_at	0.41	TruB pseudouridine (psi) synthase homolog 1 (E. coli)	TRUB1
243603_at	0.41		
231732_at	0.41	sphingomyelin phosphodiesterase 3, neutral membrane (neutral sphingomyelinase II)	SMPD3
241033_at	0.41		
241380_at	0.40		
240602_at	0.40	HBS1-like (S. cerevisiae)	HBS1L
1560823_at	0.40		
230943_at	0.40		
205931_s_at	0.40	cAMP responsive element binding protein 5	CREB5
228907_at	0.40		
244007_at	0.40		
208303_s_at	0.40	cytokine receptor-like factor 2	CRLF2
207928_s_at	0.40	glycine receptor, alpha 3	GLRA3

1558899_s_at	0.40		
235079_at	0.40		
219454_at	0.40	EGF-like-domain, multiple 6	EGFL6
1567997_x_at	0.40		
209253_at	0.40	sorbin and SH3 domain containing 3	SORBS3
216284_at	0.40		
204379_s_at	0.40	fibroblast growth factor receptor 3 (achondroplasia, thanatophoric dwarfism)	FGFR3
205114_s_at	0.40	chemokine (C-C motif) ligand 3	CCL3
242358_at	0.40		
1568752_s_at	0.40	regulator of G-protein signalling 13	RGS13
1563303_at	0.40		
216739_at	0.40		
204428_s_at	0.40	lecithin-cholesterol acyltransferase	LCAT
237417_at	0.40	regulator of chromosome condensation (RCC1) and BTB (POZ) domain containing protein 1	RCBTB1
238370_x_at	0.40		
220897_at	0.40		
231295_at	0.40	malic enzyme 3, NADP(+)-dependent, mitochondrial	ME3
207747_s_at	0.40	docking protein 4	DOK4
34210_at	0.40		
1560621_at	0.40	solute carrier family 14 (urea transporter), member 2	SLC14A2
1556393_at	0.40	zinc finger protein 451	ZNF451
240171_at	0.40		
209875_s_at	0.40	secreted phosphoprotein 1 (osteopontin, bone sialoprotein I, early T-lymphocyte activation 1)	SPP1
202485_s_at	0.40	methyl-CpG binding domain protein 2	MBD2
238499_at	0.40	solute carrier family 45, member 3	SLC45A3
234455_at	0.40	zinc finger protein 1 homolog (mouse)	ZFP1
216979_at	0.40	nuclear receptor subfamily 4, group A, member 3	NR4A3
204078_at	0.40		
206341_at	0.40	interleukin 2 receptor, alpha	IL2RA
241819_at	0.39	tumor necrosis factor (ligand) superfamily, member 8	TNFSF8
210512_s_at	0.39	vascular endothelial growth factor A	VEGFA
232725_s_at	0.39	membrane-spanning 4-domains, subfamily A, member 6A	MS4A6A
205534_at	0.39	BH-protocadherin (brain-heart)	PCDH7
214222_at	0.39	dynein, axonemal, heavy chain 7	DNAH7
229281_at	0.39		
209788_s_at	0.39		
227439_at	0.39	ankyrin repeat and sterile alpha motif domain containing 1B	ANKS1B
229118_at	0.39		
205599_at	0.39	TNF receptor-associated factor 1	TRAF1
206805_at	0.39	sema domain, immunoglobulin domain (Ig), short basic domain, secreted, (semaphorin) 3A	SEMA3A
228728_at	0.39		
1556814_a_at	0.39		
1569569_x_at	0.39		

230068_s_at	0.39	paternally expressed 3	PEG3
210072_at	0.39	chemokine (C-C motif) ligand 19	CCL19
204698_at	0.39	interferon stimulated exonuclease gene 20kDa	ISG20
37004_at	0.39	surfactant, pulmonary-associated protein B	SFTPB
230405_at	0.39		
1552266_at	0.39	ADAM metallopeptidase domain 32	ADAM32
227399_at	0.39	vestigial like 3 (Drosophila)	VGLL3
1569741_at	0.39		
228477_at	0.39		
235616_at	0.39	teashirt family zinc finger 2	TSHZ2
219172_at	0.39	ubiquitin domain containing 1	UBTD1
89977_at	0.39		
227478_at	0.39	SET binding protein 1	SETBP1
230931_at	0.39		
241395_at	0.39	nitrilase 1	NIT1
235942_at	0.39		
202627_s_at	0.39	serpin peptidase inhibitor, clade E (nexin, plasminogen activator inhibitor type 1), member 1	SERPINE1
239263_at	0.39		
240703_s_at	0.39		
233261_at	0.39	early B-cell factor 1	EBF1
1563225_a_at	0.39		
230346_x_at	0.39		
215034_s_at	0.39	transmembrane 4 L six family member 1	TM4SF1
1560449_at	0.39		
210031_at	0.38	CD247 molecule	CD247
233973_at	0.38	lysozyme-like 1	LYZL1
1560184_at	0.38	transmembrane and coiled-coil domains 5	TMCO5
209959_at	0.38	nuclear receptor subfamily 4, group A, member 3	NR4A3
206510_at	0.38	sine oculis homeobox homolog 2 (Drosophila)	SIX2
224972_at	0.38	chromosome 20 open reading frame 52	C20orf52
204014_at	0.38	dual specificity phosphatase 4	DUSP4
212099_at	0.38	ras homolog gene family, member B	RHOB
1568012_at	0.38	CAP-GLY domain containing linker protein 1	CLIP1
203798_s_at	0.38	visinin-like 1	VSNL1
1552582_at	0.38	ATP-binding cassette, sub-family C (CFTR/MRP), member 13	ABCC13
212003_at	0.38	chromosome 1 open reading frame 144	C1orf144
241037_at	0.38		
215510_at	0.38	ets variant gene 2	ETV2
210874_s_at	0.38	N-acetyltransferase 6	NAT6
210584_s_at	0.38	polymerase (DNA-directed), delta interacting protein 3	POLDIP3
203823_at	0.38	regulator of G-protein signalling 3	RGS3
210457_x_at	0.38	high mobility group AT-hook 1	HMGA1
242096_at	0.38		
202340_x_at	0.38	nuclear receptor subfamily 4, group A, member 1	NR4A1
207440_at	0.38	solute carrier family 35 (UDP-galactose transporter), member A2	SLC35A2
204420_at	0.38	FOS-like antigen 1	FOSL1

240050_s_at	0.38		
202638_s_at	0.38	intercellular adhesion molecule 1 (CD54), human rhinovirus receptor	ICAM1
1568623_a_at	0.38	solute carrier family 35, member E4	SLC35E4
214966_at	0.38	glutamate receptor, ionotropic, kainate 5	GRIK5
226560_at	0.38		
1562514_at	0.38		
240283_at	0.38		
1559827_at	0.37		
1566809_a_at	0.37		
242999_at	0.37	Rho guanine nucleotide exchange factor (GEF) 7	ARHGEF7
232918_at	0.37		
211617_at	0.37	aldolase A, fructose-bisphosphate pseudogene 2	ALDOAP2
204655_at	0.37	chemokine (C-C motif) ligand 5	CCL5
1553002_at	0.37	defensin, beta 105A	DEFB105A
211791_s_at	0.37	potassium voltage-gated channel, shaker-related subfamily, beta member 2	KCNAB2
1557347_at	0.37	microcephaly, primary autosomal recessive 1	MCPH1
228199_at	0.37		
221866_at	0.37	MyoD family inhibitor#transcription factor EB	MDFI#TFEB
243255_at	0.37	SRY (sex determining region Y)-box 6	SOX6
216490_x_at	0.37	olfactory receptor, family 2, subfamily B, member 6#olfactory receptor, family 2, subfamily W, member 6 pseudogene#olfactory receptor, family 2, subfamily W, member 4 pseudogene	OR2B6#OR2W6P#OR2W4P
242420_at	0.37	protein phosphatase 1, regulatory (inhibitor) subunit 9A	PPP1R9A
237637_at	0.37		
227759_at	0.37	proprotein convertase subtilisin/kexin type 9	PCSK9
205567_at	0.37	carbohydrate (keratan sulfate Gal-6) sulfotransferase 1	CHST1
1569892_at	0.37		
242316_at	0.37		
211627_x_at	0.37	estrogen receptor 1	ESR1
238834_at	0.37		
216575_at	0.37		
234561_at	0.37	solute carrier family 2 (facilitated glucose transporter), member 13	SLC2A13
212859_x_at	0.37	metallothionein 1E (functional)	MT1E
1558869_at	0.37	A kinase (PRKA) anchor protein 6	AKAP6
224999_at	0.37		
1561450_at	0.37		
1555486_a_at	0.37		
1552570_at	0.37	chromosome 21 open reading frame 29	C21orf29
205549_at	0.37	Purkinje cell protein 4	PCP4
233066_at	0.37		
208650_s_at	0.36	CD24 molecule	CD24
242971_at	0.36		
243666_at	0.36		
234500_at	0.36		
235527_at	0.36		

233584_at	0.36	chromosome 20 open reading frame 62	C20orf62
206486_at	0.36	lymphocyte-activation gene 3	LAG3
210822_at	0.36		
231323_at	0.36	proteasome (prosome, macropain) subunit, beta type, 2	PSMB2
226084_at	0.36	microtubule-associated protein 1B	MAP1B
243605_at	0.36		
204912_at	0.36	interleukin 10 receptor, alpha	IL10RA
241879_at	0.36		
219429_at	0.36	fatty acid 2-hydroxylase	FA2H
227984_at	0.36		
205400_at	0.36	Wiskott-Aldrich syndrome (eczema-thrombocytopenia)	WAS
1558867_at	0.36	dermatan sulfate epimerase	DSE
234166_at	0.36	ubiquitin protein ligase E3A (human papilloma virus E6-associated protein, Angelman syndrome)	UBE3A
214366_s_at	0.36	arachidonate 5-lipoxygenase	ALOX5
1556903_at	0.36		
1565661_x_at	0.36	fucosyltransferase 6 (alpha (1,3) fucosyltransferase)	FUT6
1555103_s_at	0.36	fibroblast growth factor 7 (keratinocyte growth factor)	FGF7
234655_at	0.36	annexin A2 pseudogene 2#T cell receptor beta variable 21/OR9-2#T cell receptor beta variable 24/OR9-2#T cell receptor beta variable 20/OR9-2#T cell receptor beta variable orphans on chromosome 9#T cell receptor beta variable 23/OR9-2#T cell receptor beta variable 22/OR9-2#null#suppressor of G2 allele of SKP1 pseudogene (S. cerevisiae)#ankyrin repeat domain 18B	ANXA2P2#TRBV21OR9-2#TRBV24OR9-2#TRBV20OR9-2#TRBVOR9@#TRBV23OR9-2#TRBV22OR9-2#null#SUGT1P#ANKRD18B
1405_i_at	0.36	chemokine (C-C motif) ligand 5	CCL5
244429_at	0.36		
232792_at	0.36	tripartite motif-containing 69	TRIM69
226985_at	0.36	FYVE, RhoGEF and PH domain containing 5	FGD5
242910_x_at	0.36		
238949_at	0.36		
237114_at	0.36	trafficking protein particle complex 3	TRAPPC3
1562413_at	0.36	chromosome 11 open reading frame 37	C11orf37
216248_s_at	0.35	nuclear receptor subfamily 4, group A, member 2	NR4A2
216894_x_at	0.35	cyclin-dependent kinase inhibitor 1C (p57, Kip2)	CDKN1C
219878_s_at	0.35	Kruppel-like factor 13	KLF13
231500_s_at	0.35	bolA homolog 2 (E. coli)	BOLA2
219457_s_at	0.35	Ras and Rab interactor 3	RIN3
205069_s_at	0.35	Rho GTPase activating protein 26	ARHGAP26
1566638_at	0.35		

210108_at	0.35	calcium channel, voltage-dependent, L type, alpha 1D subunit	CACNA1D
211489_at	0.35	adrenergic, alpha-1A-, receptor	ADRA1A
203394_s_at	0.35	hairy and enhancer of split 1, (Drosophila)	HES1
231881_at	0.35		
207216_at	0.35	tumor necrosis factor (ligand) superfamily, member 8	TNFSF8
220445_s_at	0.35	CSAG family, member 2	CSAG2
244364_at	0.35	myosin IIIA	MYO3A
209841_s_at	0.35	leucine rich repeat neuronal 3	LRRN3
1567361_at	0.35	brain-derived neurotrophic factor opposite strand	BDNFOS
215858_at	0.35		
1557585_at	0.35	ATPase, H+ transporting, lysosomal 50/57kDa, V1 subunit H	ATP6V1H
216164_at	0.35	leucine rich repeat neuronal 2	LRRN2
243929_at	0.35	zinc finger protein 533	ZNF533
213382_at	0.35	macrophage stimulating, pseudogene 2#neuroblastoma breakpoint family, member 1#neuroblastoma breakpoint family, member 1#neuroblastoma breakpoint family, member 1#neuroblastoma breakpoint family, member 1#null	MSTP2#NBPF1#NBPF1#NBPF1#NBPF1#null
1560495_at	0.35		
209617_s_at	0.35	catenin (cadherin-associated protein), delta 2 (neural plakophilin-related arm-repeat protein)	CTNND2
205143_at	0.35	chondroitin sulfate proteoglycan 3 (neurocan)	CSPG3
231363_at	0.35	late cornified envelope-like proline-rich 1	LELP1
220275_at	0.35	CUB and zona pellucida-like domains 1	CUZD1
1557107_at	0.35		
214408_s_at	0.35	ret finger protein-like 3 antisense#ret finger protein-like 3#F-box protein 7#chromosome 22 open reading frame 28#bactericidal/permeability-increasing protein-like 2	RFPL3S#RFPL3#FBXO7#C22orf28#BPIL2
60471_at	0.35	Ras and Rab interactor 3	RIN3
213690_s_at	0.34		
233693_at	0.34	chromosome 1 open reading frame 201	C1orf201
1553147_at	0.34	RAN binding protein 3-like	RANBP3L
220931_at	0.34		
203862_s_at	0.34	actinin, alpha 2	ACTN2
234019_at	0.34		
243592_at	0.34	REV1 homolog (S. cerevisiae)	REV1

234852_at	0.34	T cell receptor alpha locus#T cell receptor delta variable 1#T cell receptor alpha variable 36/delta variable 7#T cell receptor alpha variable 35#T cell receptor alpha variable 34#T cell receptor alpha variable 33#T cell receptor alpha variable 32#T cell receptor alpha variable 31#T cell receptor alpha variable 30#T cell receptor alpha variable 29/delta variable 5#T cell receptor alpha variable 28#T cell receptor alpha variable 27#T cell receptor alpha variable 26-2#T cell receptor alpha variable 26-1#T cell receptor alpha variable 25#T cell receptor alpha variable 24#T cell receptor alpha variable 23/delta variable 6#T cell receptor alpha variable 22#T cell receptor alpha variable 21#T cell receptor alpha variable 20#T cell receptor alpha variable 19#T cell receptor alpha variable 18#T cell receptor alpha variable 17#T cell receptor alpha variable 8-7	TRA@#TRDV1#TRAV36DV7#TRAV35#TRAV34#TRAV33#TRAV32#TRAV31#TRAV30#TRAV29DV5#TRAV28#TRAV27#TRAV26-2#TRAV26-1#TRAV25#TRAV24#TRAV23DV6#TRAV22#TRAV21#TRAV20#TRAV19#TRAV18#TRAV17#TRAV8-7
1559654_s_at	0.34		
1560559_at	0.34		
220639_at	0.34	transmembrane 4 L six family member 20	TM4SF20
204292_x_at	0.34	serine/threonine kinase 11	STK11
211889_x_at	0.34	carcinoembryonic antigen-related cell adhesion molecule 1 (biliary glycoprotein)	CEACAM1
214088_s_at	0.34	fucosyltransferase 3 (galactoside 3(4)-L-fucosyltransferase, Lewis blood group)	FUT3
208387_s_at	0.34	matrix metallopeptidase 24 (membrane-inserted)	MMP24
202339_at	0.34	symplekin	SYMPK
231779_at	0.34	interleukin-1 receptor-associated kinase 2	IRAK2
230190_at	0.34	Nedd4 family interacting protein 2	NDFIP2
210605_s_at	0.34	milk fat globule-EGF factor 8 protein	MFGE8
205033_s_at	0.34	defensin, alpha 1	DEFA1
228343_at	0.34	POU domain, class 2, transcription factor 2	POU2F2
1561639_at	0.34		
217650_x_at	0.33	ST3 beta-galactoside alpha-2,3-sialyltransferase 2	ST3GAL2
227727_at	0.33	MAS-related GPR, member F	MRGPRF
204621_s_at	0.33	nuclear receptor subfamily 4, group A, member 2	NR4A2
1566995_at	0.33		
205597_at	0.33	solute carrier family 44, member 4	SLC44A4
232593_at	0.33		
1559284_at	0.33		
1570289_at	0.33		
202237_at	0.33	nicotinamide N-methyltransferase	NNMT
208186_s_at	0.33	lipase, hormone-sensitive	LIPE
229821_at	0.33		
240144_at	0.33	deoxyribonuclease I	DNASE1
239351_at	0.33	FK506 binding protein 3, 25kDa	FKBP3
1557866_at	0.33	chromosome 9 open reading frame 117	C9orf117
1561864_at	0.33		

1559701_s_at	0.33	N-deacetylase/N-sulfotransferase (heparan glucosaminyl) 2	NDST2
203726_s_at	0.33	laminin, alpha 3	LAMA3
210611_s_at	0.33	dystrobrevin, alpha	DTNA
1561153_at	0.33		
1562983_at	0.33		
243436_at	0.33		
219268_at	0.33	ethanolamine kinase 2	ETNK2
214455_at	0.33	histone cluster 1, H2bc	HIST1H2BC
1559206_at	0.33	presenilin 1 (Alzheimer disease 3)	PSEN1
238205_at	0.33	WD repeat domain 40B	WDR40B
218600_at	0.33	LIM domain containing 2	LIMD2
227463_at	0.33		
230706_s_at	0.33	calcium/calmodulin-dependent protein kinase II inhibitor 2	CAMK2N2
240698_s_at	0.33		
226070_at	0.33	chromosome 9 open reading frame 142	C9orf142
1569872_a_at	0.33		
1557717_at	0.33		
204622_x_at	0.32	nuclear receptor subfamily 4, group A, member 2	NR4A2
211371_at	0.32	mitogen-activated protein kinase kinase 5	MAP2K5
232997_at	0.32		
239058_at	0.32		
203045_at	0.32	ninjurin 1	NINJ1
219160_s_at	0.32	poly(A) polymerase gamma	PAPOLG
244313_at	0.32	complement component (3b/4b) receptor 1 (Knops blood group)	CR1
204686_at	0.32	insulin receptor substrate 1	IRS1
1563591_at	0.32		
1556599_s_at	0.32		
236919_at	0.32	chromosome 18 open reading frame 25	C18orf25
232322_x_at	0.32	START domain containing 10	STARD10
237516_at	0.32		
206961_s_at	0.32	Trf (TATA binding protein-related factor)-proximal homolog (Drosophila)	TRFP
204015_s_at	0.32	dual specificity phosphatase 4	DUSP4
1562133_x_at	0.32	zinc finger protein 90	ZNF90
226487_at	0.32	chromosome 12 open reading frame 34	C12orf34
1562854_at	0.32		
206543_at	0.32	SWI/SNF related, matrix associated, actin dependent regulator of chromatin, subfamily a, member 2	SMARCA2
233856_at	0.32		
229004_at	0.31		
242814_at	0.31	serpin peptidase inhibitor, clade B (ovalbumin), member 9	SERPINB9
236043_at	0.31		
205398_s_at	0.31	SMAD family member 3	SMAD3
220851_at	0.31		
1570622_at	0.31		
226256_at	0.31	mitochondrial ribosomal protein S22	MRPS22

233083_at	0.31	methylenetetrahydrofolate dehydrogenase (NADP+ dependent) 2-like	MTHFD2L
1568934_at	0.31	chemokine (C-X3-C motif) receptor 1	CX3CR1
229475_at	0.31	maelstrom homolog (Drosophila)	MAEL
216177_at	0.31		
224178_s_at	0.31	SRY (sex determining region Y)-box 6	SOX6
216213_at	0.31	NIMA (never in mitosis gene a)-related kinase 1	NEK1
234682_at	0.31	BTB (POZ) domain containing 9	BTBD9
213280_at	0.31	GTPase activating Rap/RanGAP domain-like 4	GARNL4
229857_s_at	0.31		
230482_at	0.31	ST6 (alpha-N-acetyl-neuraminyl-2,3-beta-galactosyl-1,3)-N-acetylgalactosaminide alpha-2,6-sialyltran	ST6GALNAC5
1563845_at	0.31		
223817_at	0.31	leucine-rich repeats and IQ motif containing 1	LRRIQ1
1554368_at	0.31	5'-nucleotidase, cytosolic IB	NT5C1B
1564331_at	0.31		
231074_at	0.31		
243566_at	0.31		
212705_x_at	0.31	patatin-like phospholipase domain containing 2	PNPLA2
229935_s_at	0.31	myeloid/lymphoid or mixed-lineage leukemia (trithorax homolog, Drosophila)	MLL
208366_at	0.31	protocadherin 11 X-linked	PCDH11X
210941_at	0.31	BH-protocadherin (brain-heart)	PCDH7
1555001_at	0.31	IQ motif containing F1	IQCF1
206924_at	0.31	interleukin 11	IL11
242897_at	0.31		
206411_s_at	0.30	v-abl Abelson murine leukemia viral oncogene homolog 2 (arg, Abelson-related gene)	ABL2
232555_at	0.30		
233974_s_at	0.30	family with sequence similarity 129, member B	FAM129B
1560274_at	0.30	Wilms tumor 1 associated protein	WTAP
228935_at	0.30	solute carrier family 4, sodium bicarbonate cotransporter, member 8	SLC4A8
1560370_x_at	0.30	ankylosis, progressive homolog (mouse)	ANKH
212750_at	0.30	protein phosphatase 1, regulatory (inhibitor) subunit 16B	PPP1R16B
235019_at	0.30	carboxypeptidase M	CPM
231789_at	0.30	protocadherin beta 15	PCDHB15
1555671_at	0.30	islet cell autoantigen 1,69kDa-like	ICA1L
217016_x_at	0.30		
235402_at	0.30	chromosome 11 open reading frame 66	C11orf66
239664_at	0.30		
217641_at	0.30	G protein-coupled receptor 135	GPR135
217487_x_at	0.30	folate hydrolase (prostate-specific membrane antigen) 1	FOLH1
214701_s_at	0.30	fibronectin 1	FN1
204661_at	0.30	CD52 molecule	CD52
1554825_at	0.30		
1552991_at	0.30	olfactory receptor, family 5, subfamily P, member 2	OR5P2
209909_s_at	0.29	transforming growth factor, beta 2	TGFB2
1554099_a_at	0.29	spindlin family, member 3	SPIN3

204926_at	0.29	inhibin, beta A (activin A, activin AB alpha polypeptide)	INHBA
238969_at	0.29	chromosome 3 open reading frame 55	C3orf55
236383_at	0.29		
1565918_a_at	0.29		
1564533_at	0.29		
238600_at	0.29	janus kinase and microtubule interacting protein 1	JAKMIP1
234823_at	0.29		
240744_at	0.29	carboxypeptidase A5	CPA5
242098_at	0.29		
241383_at	0.29		
206731_at	0.29	connector enhancer of kinase suppressor of Ras 2	CNKSR2
244158_at	0.29		
229499_at	0.29	calpain 13	CAPN13
216814_at	0.29	SERPINE1 mRNA binding protein 1 pseudogene	SERBP1P
207176_s_at	0.29	CD80 molecule	CD80
207326_at	0.29	betacellulin	BTC
206850_at	0.29	RAS-like, family 10, member A	RASL10A
1554400_at	0.28	t-complex-associated-testis-expressed 3	TCTE3
1557805_at	0.28		
236199_at	0.28	arachidonate 5-lipoxygenase	ALOX5
213411_at	0.28		
1555456_at	0.28		
231161_x_at	0.28		
226913_s_at	0.28	SRY (sex determining region Y)-box 8	SOX8
207113_s_at	0.28	tumor necrosis factor (TNF superfamily, member 2)	TNF
1553962_s_at	0.28	ras homolog gene family, member B	RHOB
207481_at	0.28		
231004_s_at	0.28	H1 histone family, member X	H1FX
203369_x_at	0.28	PDZ and LIM domain 7 (enigma)	PDLIM7
239292_at	0.28		
205442_at	0.28	microfibrillar-associated protein 3-like	MFAP3L
218723_s_at	0.28		
205617_at	0.28	proline rich Gla (G-carboxyglutamic acid) 2	PRRG2
56748_at	0.28	tripartite motif-containing 10	TRIM10
232075_at	0.28	WD repeat domain 61	WDR61
1553151_at	0.28	ATPase, H+ transporting, lysosomal 38kDa, V0 subunit d2	ATP6V0D2
1570385_at	0.28		
1556924_at	0.28	amyotrophic lateral sclerosis 2 (juvenile) chromosome region, candidate 10	ALS2CR10
218717_s_at	0.28	leprecan-like 1	LEPREL1
241001_at	0.28	chromosome 4 open reading frame 9	C4orf9
201566_x_at	0.28	inhibitor of DNA binding 2, dominant negative helix-loop-helix protein	ID2
217123_x_at	0.28	pro-melanin-concentrating hormone-like 1	PMCHL1
1554519_at	0.28	CD80 molecule	CD80
1565837_at	0.27		

224058_s_at	0.27	hydroxysteroid (17-beta) dehydrogenase 7 pseudogene 2	HSD17B7P2
1557094_at	0.27		
236421_at	0.27	ankyrin repeat domain 45	ANKRD45
214799_at	0.27	neurofascin homolog (chicken)	NFASC
240525_at	0.27		
1555560_at	0.27	UDP-glucose ceramide glucosyltransferase-like 2	UGCGL2
1555689_at	0.27	CD80 molecule	CD80
242500_at	0.27		
214871_x_at	0.27		
243943_x_at	0.27	chromosome 6 open reading frame 52	C6orf52
1555176_at	0.27		
1557541_at	0.27	chromosome 9 open reading frame 122	C9orf122
209032_s_at	0.27	cell adhesion molecule 1	CADM1
232729_at	0.27		
217619_x_at	0.27		
1566666_at	0.27		
233604_at	0.27		
243711_at	0.27	dimethylarginine dimethylaminohydrolase 1	DDAH1
211407_at	0.27	NADH dehydrogenase (ubiquinone) 1 beta subcomplex, 7, 18kDa	NDUFB7
244849_at	0.26	sema domain, immunoglobulin domain (Ig), short basic domain, secreted, (semaphorin) 3A	SEMA3A
1570015_at	0.26		
1565920_at	0.26		
240115_at	0.26		
203665_at	0.26	heme oxygenase (decycling) 1	HMOX1
230232_at	0.26		
1569001_at	0.26	bone morphogenetic protein 1	BMP1
214297_at	0.26	chondroitin sulfate proteoglycan 4 (melanoma-associated)	CSPG4
215926_x_at	0.26	small nuclear RNA activating complex, polypeptide 4, 190kDa	SNAPC4
210689_at	0.26	claudin 14	CLDN14
235404_at	0.26	AT rich interactive domain 5B (MRF1-like)	ARID5B
210770_s_at	0.26	calcium channel, voltage-dependent, P/Q type, alpha 1A subunit	CACNA1A
215646_s_at	0.26	chondroitin sulfate proteoglycan 2 (versican)	CSPG2
211719_x_at	0.26	fibronectin 1	FN1
244468_at	0.26	PDZ domain containing 1	PDZK1
1559344_at	0.26		
209840_s_at	0.26	leucine rich repeat neuronal 3	LRRN3
217484_at	0.26	complement component (3b/4b) receptor 1 (Knops blood group)	CR1
227347_x_at	0.26	hairy and enhancer of split 4 (Drosophila)	HES4
243254_at	0.26	human immunodeficiency virus type I enhancer binding protein 2	HIVEP2
233227_at	0.26	KIAA1109	KIAA1109
233723_at	0.26		
1561882_at	0.26	synaptotagmin-like 3	SYTL3

234458_at	0.26	alanyl (membrane) aminopeptidase (aminopeptidase N, aminopeptidase M, microsomal aminopeptidase, CD1	ANPEP
237064_x_at	0.26		
1566515_at	0.26	CWF19-like 2, cell cycle control (S. pombe)	CWF19L2
217236_x_at	0.26	immunoglobulin heavy constant gamma 1 (G1m marker)	IGHG1
213182_x_at	0.26	cyclin-dependent kinase inhibitor 1C (p57, Kip2)	CDKN1C
219993_at	0.26	SRY (sex determining region Y)-box 17	SOX17
1564360_a_at	0.26		
1563507_at	0.26		
236971_at	0.26		
1568970_at	0.26	ADAM metallopeptidase domain 18	ADAM18
1560407_at	0.26	MAP/microtubule affinity-regulating kinase 1	MARK1
206552_s_at	0.25	tachykinin, precursor 1 (substance K, substance P, neurokinin 1, neurokinin 2, neuromedin L, neuroki	TAC1
1563133_at	0.25		
234366_x_at	0.25	immunoglobulin lambda locus	IGL@
239459_s_at	0.25	cytochrome P450, family 19, subfamily A, polypeptide 1	CYP19A1
204942_s_at	0.25	aldehyde dehydrogenase 3 family, member B2	ALDH3B2
204103_at	0.25	chemokine (C-C motif) ligand 4	CCL4
220351_at	0.25	chemokine (C-C motif) receptor-like 1	CCRL1
1570627_at	0.25	transcription elongation factor B (SIII), polypeptide 3 (110kDa, elongin A)	TCEB3
232607_at	0.25		
216740_at	0.25		
206408_at	0.25	leucine rich repeat transmembrane neuronal 2	LRRTM2
229830_at	0.25		
236761_at	0.25	lipoma HMGIC fusion partner-like 3	LHFPL3
241032_at	0.25	ankyrin repeat domain 40	ANKRD40
237187_at	0.25		
241195_at	0.25		
216586_at	0.25	uracil-DNA glycosylase pseudogene 2	UNGP2
1563572_at	0.25		
240463_at	0.25		
208358_s_at	0.25	UDP glycosyltransferase 8 (UDP-galactose ceramide galactosyltransferase)	UGT8
1563389_at	0.25		
238989_at	0.25	C1GALT1-specific chaperone 1	C1GALT1C1
1552544_at	0.25	serpin peptidase inhibitor, clade A (alpha-1 antiproteinase, antitrypsin), member 12	SERPINA12
204035_at	0.24	secretogranin II (chromogranin C)	SCG2
237452_at	0.24	collagen, type XXVII, alpha 1	COL27A1
226612_at	0.24		
240733_at	0.24		
1553652_a_at	0.24	chromosome 18 open reading frame 54	C18orf54
1556066_at	0.24	jumonji domain containing 3	JMJD3
243146_at	0.24	adrenergic, alpha-1A-, receptor	ADRA1A
1559991_s_at	0.24		
229133_s_at	0.24	zinc finger protein 397	ZNF397
237135_at	0.24		

1559573_at	0.24		
233616_at	0.24		
1555799_at	0.24	Fc receptor-like 5	FCRL5
208404_x_at	0.24	potassium inwardly-rectifying channel, subfamily J, member 5	KCNJ5
1563263_at	0.24	phospholipase C, gamma 2 (phosphatidylinositol-specific)	PLCG2
1557739_at	0.24		
202008_s_at	0.24	nidogen 1	NID1
1561985_at	0.24	chromosome 14 open reading frame 39	C14orf39
1558406_a_at	0.24		
208123_at	0.24	potassium voltage-gated channel, Shab-related subfamily, member 2	KCNB2
213799_s_at	0.24	protein tyrosine phosphatase, receptor type, A	PTPRA
1552917_at	0.24	interleukin 29 (interferon, lambda 1)	IL29
1561415_at	0.23		
209399_at	0.23	holocarboxylase synthetase (biotin-(proprionyl-Coenzyme A-carboxylase (ATP-hydrolysing)) ligase)	HLCS
237488_at	0.23	heterogeneous nuclear ribonucleoprotein C (C1/C2)	HNRPC
215481_s_at	0.23	peroxisomal biogenesis factor 5	PEX5
1559405_a_at	0.23	transient receptor potential cation channel, subfamily V, member 6	TRPV6
242054_s_at	0.23		
207872_s_at	0.23	leukocyte immunoglobulin-like receptor, subfamily A (with TM domain), member 1	LILRA1
208275_x_at	0.23	undifferentiated embryonic cell transcription factor 1	UTF1
1557056_at	0.23		
1557280_s_at	0.23		
215282_at	0.23	anaphase promoting complex subunit 13	ANAPC13
1553658_at	0.23		
210247_at	0.23	synapsin II	SYN2
1569140_at	0.23	ubiquitin protein ligase E3 component n-recognin 2	UBR2
241861_at	0.23	synaptonemal complex protein 3	SYCP3
1568713_a_at	0.23	TBC1 (tre-2/USP6, BUB2, cdc16) domain family, member 1	TBC1D1
234489_at	0.23		
243137_at	0.23		
203961_at	0.23	nebulette	NEBL
1557620_a_at	0.23	coiled-coil domain containing 38	CCDC38
223828_s_at	0.23	lectin, galactoside-binding, soluble, 12 (galectin 12)	LGALS12
1563224_at	0.23		
1566902_at	0.23		
243014_at	0.22	zinc and ring finger 3	ZNRF3
233302_at	0.22		
216557_x_at	0.22	interferon, alpha-inducible protein 6	IFI6
204469_at	0.22	protein tyrosine phosphatase, receptor-type, Z polypeptide 1	PTPRZ1
224040_at	0.22	testis-specific transcript, Y-linked 5	TTTY5

239759_at	0.22		
236749_at	0.22	MAX binding protein	MNT
1553071_a_at	0.22	myozenin 3	MYOZ3
207407_x_at	0.22	cytochrome P450, family 4, subfamily A, polypeptide 11	CYP4A11
239738_at	0.22	dachshund homolog 2 (Drosophila)	DACH2
205463_s_at	0.22	platelet-derived growth factor alpha polypeptide	PDGFA
243422_at	0.22		
240380_at	0.22		
220883_at	0.22	glyoxalase I	GLO1
231313_at	0.22	leucine rich repeat containing 8 family, member B	LRRC8B
215473_at	0.22		
242756_at	0.22	Wiskott-Aldrich syndrome-like	WASL
204421_s_at	0.22	fibroblast growth factor 2 (basic)	FGF2
1554757_a_at	0.22	inositol polyphosphate-5-phosphatase, 40kDa	INPP5A
229188_s_at	0.22	zinc and ring finger 2	ZNRF2
214110_s_at	0.22		
223484_at	0.21	chromosome 15 open reading frame 48	C15orf48
1554233_at	0.21	C1q and tumor necrosis factor related protein 9	C1QTNF9
1568644_at	0.21	zinc finger protein 208	ZNF208
244858_at	0.21	TGFB-induced factor homeobox 1	TGIF1
217406_at	0.21	ret finger protein-like 3 antisense#ret finger protein-like 3#F-box protein 7#chromosome 22 open reading frame 28#bactericidal/permeability-increasing protein-like 2	RFPL3S#RFPL3#FBXO7#C22orf28#BPIL2
231688_at	0.21		
205002_at	0.21	AT hook, DNA binding motif, containing 1	AHDC1
214702_at	0.21	fibronectin 1	FN1
1561892_at	0.21	zinc finger, MYM-type 6	ZMYM6
211667_x_at	0.21		
1563229_at	0.21	deleted in lymphocytic leukemia, 2	DLEU2
1560517_s_at	0.21		
243474_at	0.21		
235735_at	0.21		
1568777_at	0.21		
238371_s_at	0.20		
215301_at	0.20		
218925_s_at	0.20	chromosome 11 open reading frame 1	C11orf1
217453_at	0.20		
220405_at	0.20	syntrophin, gamma 1	SNTG1
233358_at	0.20		
215704_at	0.20	filaggrin#null	FLG#null
221874_at	0.20	KIAA1324	KIAA1324
211737_x_at	0.20	pleiotrophin (heparin binding growth factor 8, neurite growth-promoting factor 1)	PTN
206942_s_at	0.20	pro-melanin-concentrating hormone	PMCH
232060_at	0.20		
219622_at	0.20	RAB20, member RAS oncogene family	RAB20
243880_at	0.20	golgi SNAP receptor complex member 2	GOSR2
244190_at	0.20	THAP domain containing 5	THAP5
1566176_at	0.20		
1553558_at	0.20	taste receptor, type 2, member 41	TAS2R41

213290_at	0.20	collagen, type VI, alpha 2	COL6A2
1553878_at	0.19	glutamic-oxaloacetic transaminase 1-like 1	GOT1L1
242676_at	0.19		
217315_s_at	0.19	kallikrein-related peptidase 13	KLK13
1570246_at	0.19		
1569810_at	0.19		
240621_at	0.19	ataxia telangiectasia mutated (includes complementation groups A, C and D)	ATM
237738_at	0.19		
234752_x_at	0.19	dystrophin (muscular dystrophy, Duchenne and Becker types)	DMD
213968_at	0.19	tetraspanin 5	TSPAN5
1559970_at	0.19		
228767_at	0.19	ataxin 2-like	ATXN2L
242798_at	0.19		
237124_at	0.19		
237448_at	0.19		
1560201_at	0.19	zinc finger protein 713	ZNF713
202133_at	0.18	WW domain containing transcription regulator 1	WWTR1
240770_at	0.18	transmembrane protein 171	TMEM171
1564070_s_at	0.18		
241567_at	0.18	nucleolar protein 4	NOL4
1560034_a_at	0.18		
215783_s_at	0.18	alkaline phosphatase, liver/bone/kidney	ALPL
240828_at	0.18		
210170_at	0.18	PDZ and LIM domain 3	PDLIM3
205285_s_at	0.18	FYN binding protein (FYB-120/130)	FYB
1558354_s_at	0.18		
240026_x_at	0.18		
222196_at	0.18		
229495_at	0.18	aminoacylase 1-like 2	ACY1L2
1569978_x_at	0.18		
1556062_at	0.18	ribonuclease P/MRP 30kDa subunit	RPP30
241911_at	0.17	cyclin-dependent kinase-like 3	CDKL3
205579_at	0.17	histamine receptor H1	HRH1
214032_at	0.17	zeta-chain (TCR) associated protein kinase 70kDa	ZAP70
1559545_at	0.17	small nuclear ribonucleoprotein polypeptide N	SNRPN
233627_at	0.17		
230594_at	0.17		
208399_s_at	0.17	endothelin 3	EDN3
1559497_at	0.17		
244536_at	0.17	tumor protein p53 binding protein, 2	TP53BP2
1569916_at	0.17		
233267_at	0.17	selenium binding protein 1	SELENBP1
1553776_at	0.17	ubiquitin-conjugating enzyme E2U (putative)	UBE2U
205242_at	0.17	chemokine (C-X-C motif) ligand 13 (B-cell chemoattractant)	CXCL13
225162_at	0.17		
1569981_at	0.17		
1564107_at	0.17		
1558578_a_at	0.17	solute carrier family 13 (sodium/sulfate symporters), member 4	SLC13A4

227794_at	0.17	glycine-N-acyltransferase-like 1	GLYATL1
228362_s_at	0.16		
242254_at	0.16	LSM domain containing 1	LSMD1
1563456_at	0.16		
224053_s_at	0.16	solute carrier family 4, sodium bicarbonate cotransporter, member 9	SLC4A9
242950_x_at	0.16	sorting nexin 4	SNX4
237648_x_at	0.16		
224045_x_at	0.16	chromosome 18 open reading frame 2	C18orf2
1557706_at	0.16		
216618_at	0.15		
1568812_at	0.15		
226681_at	0.15	ubiquitin-conjugating enzyme E2H (UBC8 homolog, yeast)	UBE2H
242152_at	0.15		
243287_s_at	0.15		
241075_at	0.15		
243204_at	0.15		
238272_at	0.15	thiopurine S-methyltransferase	TPMT
232477_at	0.15		
227126_at	0.15		
242613_at	0.15	PR domain containing 5	PRDM5
237411_at	0.15	ADAM metallopeptidase with thrombospondin type 1 motif, 6	ADAMTS6
1561720_at	0.14	RecQ protein-like 5	RECQL5
221451_s_at	0.14	olfactory receptor, family 2, subfamily W, member 1	OR2W1
243723_at	0.14		
207103_at	0.14	potassium voltage-gated channel, Shal-related subfamily, member 2	KCND2
241562_x_at	0.14		
1561407_at	0.14	centaurin, delta 1	CENTD1
244757_at	0.14	cytochrome P450, family 2, subfamily R, polypeptide 1	CYP2R1
1562249_at	0.14		
241256_at	0.14		
219584_at	0.14	phospholipase A1 member A	PLA1A
1560174_at	0.14	sperm associated antigen 16	SPAG16
1561553_at	0.14		
234789_at	0.14		
1563386_at	0.14		
235001_at	0.14		
211682_x_at	0.13	UDP glucuronosyltransferase 2 family, polypeptide B28	UGT2B28
217494_s_at	0.13	phosphatase and tensin homolog (mutated in multiple advanced cancers 1), pseudogene 1	PTENP1
209292_at	0.13	inhibitor of DNA binding 4, dominant negative helix-loop-helix protein	ID4
229222_at	0.13		
1555401_at	0.13	spermatogenesis and oogenesis specific basic helix-loop-helix 2	SOHLH2
1563135_at	0.13		
220884_at	0.13		

1570250_at	0.13		
1556685_at	0.12		
1563725_at	0.12	zinc finger protein 583	ZNF583
240329_at	0.12		
1568126_at	0.12	annexin A2	ANXA2
242800_at	0.12	Nance-Horan syndrome (congenital cataracts and dental anomalies)	NHS
1563016_at	0.12		
224393_s_at	0.12	cat eye syndrome chromosome region, candidate 6	CECR6
241561_at	0.12	membrane-associated ring finger (C3HC4) 3	3-Mar
1555055_at	0.11	KIAA0241	KIAA0241
1565637_at	0.11		
239665_at	0.11		
229072_at	0.11		
1555133_at	0.11	family with sequence similarity 9, member A	FAM9A
205111_s_at	0.11	phospholipase C, epsilon 1	PLCE1
1560855_at	0.10		
238868_at	0.10	uveal autoantigen with coiled-coil domains and ankyrin repeats	UACA
1553180_at	0.10	ADAM metallopeptidase with thrombospondin type 1 motif, 19	ADAMTS19
1561479_at	0.10		
235382_at	0.10		
233863_at	0.10	castor zinc finger 1	CASZ1
243549_at	0.10		
1564405_at	0.09		
1555854_at	0.09		
231028_at	0.09		
1558577_at	0.09		
211571_s_at	0.09	chondroitin sulfate proteoglycan 2 (versican)	CSPG2
243211_at	0.08		
1558387_at	0.08		
212759_s_at	0.08	transcription factor 7-like 2 (T-cell specific, HMG-box)	TCF7L2
240914_at	0.08		
213582_at	0.07	ATPase, Class VI, type 11A	ATP11A
234271_at	0.07	otopetrin 2	OTOP2
213148_at	0.07		
223823_at	0.07	potassium large conductance calcium-activated channel, subfamily M, beta member 2	KCNMB2
244608_at	0.07		
236882_at	0.07		
206935_at	0.07	protocadherin 8	PCDH8
232993_at	0.06	synaptojanin 1	SYNJ1
1554922_at	0.05	zinc finger protein 678	ZNF678
217528_at	0.05	chloride channel, calcium activated, family member 2	CLCA2
220817_at	0.05	transient receptor potential cation channel, subfamily C, member 4	TRPC4
242623_x_at	0.05		
237573_at	0.05		
206426_at	0.04	melan-A	MLANA

231734_at	0.03	retinol binding protein 2, cellular	RBP2
215172_at	0.03	protein tyrosine phosphatase, non-receptor type 20B#protein tyrosine phosphatase, non-receptor type 20A#protein tyrosine phosphatase, non-receptor type 20A	PTPN20B#PTPN20A#PTPN20A
207272_at	0.03	zinc finger protein 80	ZNF80
241676_x_at	0.02		
