# Supplementary material for: In Vitro Transformation of Primary Human CD34+ Cells by AML Fusion Oncogenes: Early Gene Expression Profiling Reveals Possible Drug Target in AML
Source: PLoS One. 2010 Aug 27;5(8):e12464. doi: 10.1371/journal.pone.0012464 (PMC2929205; doi:10.1371/journal.pone.0012464)
Supplement: Table S23 — Genes deregulated by NUP98-HOXA9 3 days after transduction. Primary human CD34+ cells were retrovirally transduced with either control MSCV-IRES-GFP vector or vector expressing NUP98-HOXA9 and sorted for GFP positivity. Total RNA was extracted 3 days after transduction and subjected to microarray analysis. Microarray data were analyzed by SAM as described in Materials and Methods. Significantly deregulated genes are listed and the false discovery rate (FDR) is shown. (0.21 MB PDF) [file pone.0012464.s023.pdf]

Table S23. Genes deregulated by NUP98-HOXA9 at 3 d detected by SAM **FDR = 3.36%**

Probe set ID	Fold Change	Gene Name	Gene Symbol
204439_at	325.52	interferon-induced protein 44-like	IFI44L
206367_at	208.35	renin	REN
1553428_at	85.31		
207952_at	65.41	interleukin 5 (colony-stimulating factor, eosinophil)	IL5
206442_at	62.91	semenogelin I	SEMG1
205751_at	47.89	SH3-domain GRB2-like 2	SH3GL2
1554507_at	47.35	N-acetylated alpha-linked acidic dipeptidase 2	NAALAD2
1564580_at	45.96		
203153_at	43.56	interferon-induced protein with tetratricopeptide repeats 1	IFIT1
1563072_at	39.92		
233821_at	36.89	RAB32, member RAS oncogene family	RAB32
202086_at	36.27	myxovirus (influenza virus) resistance 1, interferon-inducible protein p78 (mouse)	MX1
206987_x_at	35.34	fibroblast growth factor 18	FGF18
237219_at	32.20		
228230_at	31.53	eukaryotic translation elongation factor 1 alpha 2	EEF1A2
241897_at	29.37	RNA binding protein with multiple splicing	RBPMS
233096_at	28.95	KIAA1109	KIAA1109
237058_x_at	28.31	solute carrier family 6 (neurotransmitter transporter, GABA), member 13	SLC6A13
1554473_at	28.23	SLIT-ROBO Rho GTPase activating protein 1	SRGAP1
1556026_at	26.69	iduronate 2-sulfatase (Hunter syndrome)	IDS
211485_s_at	25.54	fibroblast growth factor 18	FGF18
204533_at	24.89	chemokine (C-X-C motif) ligand 10	CXCL10
210893_at	22.36		
233162_at	20.24		
243478_at	19.97		
215301_at	19.67		
217502_at	19.52	interferon-induced protein with tetratricopeptide repeats 2	IFIT2
1569873_at	19.10	lipase-like, ab-hydrolase domain containing 1	LIPL1
1560169_at	18.80	phosphatidylinositol-4-phosphate 5-kinase, type I, beta	PIP5K1B
1559310_at	18.57		
1562400_at	17.93		
242301_at	17.92	cerebellin 2 precursor	CBLN2
1566428_at	17.74		
237206_at	17.32	myocardin	MYOCD
213316_at	17.32	KIAA1462	KIAA1462
202869_at	17.13	2',5'-oligoadenylate synthetase 1, 40/46kDa	OAS1
211029_x_at	16.79	fibroblast growth factor 18	FGF18
209488_s_at	16.20	RNA binding protein with multiple splicing	RBPMS
224762_at	16.09	serine incorporator 2	SERINC2
214569_at	15.32	interferon, alpha 5	IFNA5
202411_at	15.30	interferon, alpha-inducible protein 27	IFI27
1561895_at	15.21		
207836_s_at	15.14	RNA binding protein with multiple splicing	RBPMS

1567575_at	15.01		
1566947_at	14.95		
219364_at	14.71		
229441_at	14.62	protease, serine, 23	PRSS23
236893_at	13.36		
234655_at	13.24	annexin A2	ANXA2P2
223595_at	13.10	transmembrane protein 133	TMEM133
232056_at	12.98	sciellin	SCEL
210661_at	12.87	glycine receptor, alpha 3	GLRA3
242340_at	12.82		
205278_at	12.48	glutamate decarboxylase 1 (brain, 67kDa)	GAD1
1553558_at	12.45	taste receptor, type 2, member 41	TAS2R41
215314_at	12.42	ankyrin 3, node of Ranvier (ankyrin G)	ANK3
229288_at	12.00		
237835_at	11.74		
216318_at	11.67	immunoglobulin heavy constant alpha 1	IGHA1
223812_at	11.59		
1564642_at	11.51	runt-related transcription factor 1; translocated to, 1 (cyclin D-related)	RUNX1T1
242344_at	11.48	gamma-aminobutyric acid (GABA) A receptor, beta 2	GABRB2
229638_at	11.44	iroquois homeobox protein 3	IRX3
231382_at	11.34	fibroblast growth factor 18	FGF18
235885_at	11.32		
239979_at	11.05	epithelial stromal interaction 1 (breast)	EPSTI1
1560748_at	10.99		
206382_s_at	10.97	brain-derived neurotrophic factor	BDNF
209465_x_at	10.74	pleiotrophin (heparin binding growth factor 8, neurite growth-promoting factor 1)	PTN
209396_s_at	10.73	chitinase 3-like 1 (cartilage glycoprotein-39)	CHI3L1
226757_at	10.72	interferon-induced protein with tetratricopeptide repeats 2	IFIT2
234240_at	10.69	gelsolin (amyloidosis, Finnish type)	GSN
229450_at	10.68	interferon-induced protein with tetratricopeptide repeats 3	IFIT3
238868_at	10.65	uveal autoantigen with coiled-coil domains and ankyrin repeats	UACA
1556713_at	10.55		
243564_at	10.47	phosphodiesterase 1C, calmodulin-dependent 70kDa	PDE1C
210809_s_at	10.44	periostin, osteoblast specific factor	POSTN
205984_at	10.37	corticotropin releasing hormone binding protein	CRHBP
1567333_at	10.26		
218400_at	10.26	2'-5'-oligoadenylate synthetase 3, 100kDa	OAS3
210026_s_at	10.13	caspase recruitment domain family, member 10	CARD10
213913_s_at	10.12		
205483_s_at	10.07	ISG15 ubiquitin-like modifier	ISG15
1565949_x_at	10.05	choroideremia-like (Rab escort protein 2)	CHML
212883_at	9.98	apolipoprotein E	APOE
227725_at	9.90	ST6 (alpha-N-acetyl-neuraminyl-2,3-beta-galactosyl-1,3)-N-acetylgalactosaminide alpha-2,6-sialyltran	ST6GALNAC1
230232_at	9.87		
235276_at	9.69	epithelial stromal interaction 1 (breast)	EPSTI1
211466_at	9.66	nuclear factor I/B	NFIB

220784_s_at	9.55	urotensin 2	UTS2
236892_s_at	9.41		
215168_at	9.39	translocase of inner mitochondrial membrane 17 homolog A (yeast)	TIMM17A
240472_at	9.24		
239984_at	9.22	sodium channel, voltage-gated, type VII, alpha	SCN7A
239738_at	9.19	dachshund homolog 2 (Drosophila)	DACH2
1561151_a_at	9.14		
221672_s_at	9.05		
1568644_at	9.04	zinc finger protein 208	ZNF208
206785_s_at	9.04	killer cell lectin-like receptor subfamily C, member 2	KLRC2
1552368_at	9.00	CCCTC-binding factor (zinc finger protein)-like	CTCFL
206655_s_at	8.99	glycoprotein Ib (platelet), beta polypeptide	GP1BB
238385_at	8.92	chromosome 6 open reading frame 58	C6orf58
242093_at	8.90	synaptotagmin-like 5	SYTL5
1559372_at	8.87		
204747_at	8.75	interferon-induced protein with tetratricopeptide repeats 3	IFIT3
207837_at	8.75	RNA binding protein with multiple splicing	RBPMS
213966_at	8.73	high-mobility group 20B	HMG20B
222153_at	8.58	myelin expression factor 2	MYEF2
232868_at	8.52	chromosome 9 open reading frame 11	C9orf11
215571_at	8.48		
230757_at	8.47		
217048_at	8.34	SHC (Src homology 2 domain containing) transforming protein 1 pseudogene 1	SHC1P1
204415_at	8.34	interferon, alpha-inducible protein 6	IFI6
229201_at	8.33		
1552372_at	8.30	chromosome 4 open reading frame 33	C4orf33
204994_at	8.27	myxovirus (influenza virus) resistance 2 (mouse)	MX2
224278_at	8.26	chromosome 2 open reading frame 14	C2orf14
244503_at	8.24	brain-derived neurotrophic factor	BDNF
1570051_at	8.23	ring finger protein 144	RNF144
216139_s_at	8.20	non-metastatic cells 3, protein expressed in#mitogen-activated protein kinase 8 interacting protein 3	NME3#MAPK8IP3
233401_at	8.17		
1570090_at	8.12		
242954_at	8.11		
223838_at	8.11	testis specific, 10	TSGA10
241067_at	8.09		
207517_at	8.07	laminin, gamma 2	LAMC2
205893_at	8.07	neuroligin 1	NLGN1
214059_at	8.03	interferon-induced protein 44	IFI44
1567287_at	7.98	olfactory receptor, family 5, subfamily K, member 1	OR5K1
237752_at	7.98		
1556158_at	7.87		
243346_at	7.82	leiomodin 3 (fetal)	LMOD3
243275_at	7.82	bone morphogenetic protein receptor, type IA	BMPR1A
218186_at	7.80	RAB25, member RAS oncogene family	RAB25
224052_at	7.77	heat shock transcription factor, Y-linked 1	HSFY1
208436_s_at	7.76	interferon regulatory factor 7	IRF7
224006_at	7.61		
1557334_a_at	7.61	cardiolipin synthase 1	CRLS1

1560153_at	7.59	Fraser syndrome 1	FRAS1
244142_at	7.51		
223652_at	7.48	arsenic (+3 oxidation state) methyltransferase	AS3MT
241291_at	7.47		
1561136_at	7.43	glycophorin E	GYPE
213578_at	7.42	bone morphogenetic protein receptor, type IA	BMPR1A
214457_at	7.34	homeobox A2	HOXA2
222063_s_at	7.29	CDP-diacylglycerol synthase (phosphatidate cytidylyltransferase) 1	CDS1
1553300_a_at	7.28	diacylglycerol kinase, eta	DGKH
206591_at	7.27	recombination activating gene 1	RAG1
1561985_at	7.25	chromosome 14 open reading frame 39	C14orf39
243183_at	7.21		
214024_s_at	7.18	DiGeorge syndrome critical region gene 6-like	DGCR6L
242501_at	7.17	ribosomal protein L5	RPL5
243885_x_at	7.17		
214453_s_at	7.14	interferon-induced protein 44	IFI44
232155_at	7.14	KIAA1618	KIAA1618
210016_at	7.11	myelin transcription factor 1-like	MYT1L
1563467_at	7.09		
205694_at	7.07	tyrosinase-related protein 1	TYRP1
211361_s_at	7.03	serpin peptidase inhibitor, clade B (ovalbumin), member 13	SERPINB13
1562742_at	7.00		
1562737_at	6.97		
1562613_at	6.89		
1554506_x_at	6.87	N-acetylated alpha-linked acidic dipeptidase 2	NAALAD2
215928_at	6.87		
213183_s_at	6.80	cyclin-dependent kinase inhibitor 1C (p57, Kip2)	CDKN1C
227019_at	6.79		
205651_x_at	6.75	Rap guanine nucleotide exchange factor (GEF) 4	RAPGEF4
1566937_at	6.74		
232037_at	6.73	putative neuronal cell adhesion molecule	PUNC
228617_at	6.71		
244789_at	6.71	chromosome 10 open reading frame 122	C10orf122
1555123_at	6.70	ST6 beta-galactosamide alpha-2,6-sialyltranferase 2	ST6GAL2
1563189_at	6.69		
235617_x_at	6.66		
1557274_at	6.66		
1552785_at	6.65	zinc finger protein 781	ZNF781
227842_at	6.64	RAB30, member RAS oncogene family	RAB30
1556558_s_at	6.63		
229057_at	6.61	sodium channel, voltage-gated, type II, alpha subunit	SCN2A
213268_at	6.59	period homolog 3	PER3
230130_at	6.57	slit homolog 2 (Drosophila)	SLIT2
237480_at	6.47		
242623_x_at	6.46		
234563_at	6.45		
1563589_at	6.44		
219211_at	6.44	ubiquitin specific peptidase 18	USP18
205552_s_at	6.44	2',5'-oligoadenylate synthetase 1, 40/46kDa	OAS1
206508_at	6.43	CD70 molecule	CD70

209487_at	6.41	RNA binding protein with multiple splicing	RBPMS
232113_at	6.41		
228607_at	6.38	2'-5'-oligoadenylate synthetase 2, 69/71kDa	OAS2
1565602_at	6.38		
212915_at	6.38	PDZ domain containing RING finger 3	PDZRN3
226442_at	6.36	ankyrin repeat and BTB (POZ) domain containing 1	ABTB1
215939_at	6.28		
1561795_at	6.28		
1555346_at	6.28	cell division cycle 20 homolog B (S. cerevisiae)	CDC20B
203792_x_at	6.27	polycomb group ring finger 2	PCGF2
240512_x_at	6.24	potassium channel tetramerisation domain containing 4	KCTD4
206891_at	6.23	actinin, alpha 3	ACTN3
227195_at	6.23	zinc finger protein 503	ZNF503
232895_s_at	6.21	SEC14-like 2 (S. cerevisiae)	SEC14L2
207901_at	6.19	interleukin 12B (natural killer cell stimulatory factor 2, cytotoxic lymphocyte maturation factor 2,	IL12B
202254_at	6.19	signal-induced proliferation-associated 1 like 1	SIPA1L1
206581_at	6.18	basonuclin 1	BNC1
1559433_at	6.18		
222096_x_at	6.17		
219612_s_at	6.15	fibrinogen gamma chain	FGG
204217_s_at	6.03	reticulon 2	RTN2
1564399_a_at	6.02	armadillo repeat containing, X-linked 4	ARMCX4
238135_at	5.98	angiotensin II receptor-associated protein	AGTRAP
1554474_a_at	5.97	monooxygenase, DBH-like 1	MOXD1
233139_at	5.97		
209795_at	5.96	CD69 molecule	CD69
242873_at	5.96		
228136_s_at	5.95	chromosome 17 open reading frame 70	C17orf70
208348_s_at	5.95	Cas-Br-M (murine) ecotropic retroviral transforming sequence b	CBLB
1569740_at	5.93		
205816_at	5.92	integrin, beta 8	ITGB8
228384_s_at	5.92	chromosome 10 open reading frame 33	C10orf33
223854_at	5.91	protocadherin beta 10	PCDHB10
239480_at	5.90		
1564403_at	5.89	topoisomerase I binding, arginine/serine-rich	TOPORS
1561642_at	5.86		
237542_at	5.82		
1567912_s_at	5.81		
235643_at	5.80	sterile alpha motif domain containing 9-like	SAMD9L
1570082_x_at	5.79		
210173_at	5.78	protein tyrosine phosphatase, receptor type, J	PTPRJ
1558606_s_at	5.74		
234570_at	5.72	zinc finger protein 71	ZNF71
1564257_at	5.71		
226279_at	5.71	protease, serine, 23	PRSS23
241767_at	5.67		
1560846_at	5.66		
233858_at	5.65		
204455_at	5.63	dystonin	DST
219737_s_at	5.63	protocadherin 9	PCDH9

244399_at	5.60		
231884_at	5.60	centrobin, centrosomal BRCA2 interacting protein	CNTROB
241132_at	5.59		
203187_at	5.59	dedicator of cytokinesis 1	DOCK1
1558987_at	5.57		
242006_at	5.55	chromosome 6 open reading frame 152	C6orf152
211597_s_at	5.55		
1556997_a_at	5.54		
205749_at	5.53	cytochrome P450, family 1, subfamily A, polypeptide 1	CYP1A1
231598_x_at	5.45		
237732_at	5.42		
234681_s_at	5.42	chromodomain helicase DNA binding protein 6	CHD6
229313_at	5.41	transmembrane protein 16E	TMEM16E
232276_at	5.38	heparan sulfate 6-O-sulfotransferase 3	HS6ST3
241791_at	5.37	titin	TTN
229151_at	5.35	solute carrier family 14 (urea transporter), member 1 (Kidd blood group)	SLC14A1
1554962_a_at	5.34	fibroblast growth factor receptor 4	FGFR4
242455_at	5.31	POU domain, class 3, transcription factor 2	POU3F2
227503_at	5.31		
214256_at	5.30	ATPase, Class V, type 10A	ATP10A
215598_at	5.29	tetratricopeptide repeat domain 12	TTC12
227850_x_at	5.29	CDC42 effector protein (Rho GTPase binding) 5	CDC42EP5
234556_at	5.29		
1565657_at	5.28	CKLF-like MARVEL transmembrane domain containing 6	CMTM6
204972_at	5.25	2'-5'-oligoadenylate synthetase 2, 69/71kDa	OAS2
233944_at	5.25		
239916_at	5.24	WD repeat domain 16	WDR16
242244_at	5.24		
238727_at	5.24		
239174_at	5.23		
205844_at	5.23	vanin 1	VNN1
206119_at	5.21	betaine-homocysteine methyltransferase	BHMT
206133_at	5.19		
1556232_at	5.18	kinesin family member 6	KIF6
203595_s_at	5.17		
220724_at	5.15		
232837_at	5.14	kinesin family member 13A	KIF13A
240726_at	5.14		
1553207_at	5.13	ADP-ribosylation factor-like 10	ARL10
240904_at	5.10		
242234_at	5.10		
244088_at	5.07		
207729_at	5.06	cadherin 9, type 2 (T1-cadherin)	CDH9
224508_at	5.04		
234340_at	4.99		
203038_at	4.98	protein tyrosine phosphatase, receptor type, K	PTPRK
213435_at	4.97	SATB family member 2	SATB2
1561856_at	4.96		
207780_at	4.95	cylicin, basic protein of sperm head cytoskeleton 2	CYLC2

204981_at	4.94	solute carrier family 22 (organic cation transporter), member 18	SLC22A18
228507_at	4.93		
237986_at	4.92		
240025_x_at	4.92		
1563341_at	4.89		
227609_at	4.87	epithelial stromal interaction 1 (breast)	EPSTI1
202011_at	4.87	tight junction protein 1 (zona occludens 1)	TJP1
1562579_at	4.86		
1555003_at	4.86	retinoblastoma-like 1 (p107)	RBL1
238919_at	4.86		
230846_at	4.84		
222929_at	4.84	retinoic acid induced 16	RAI16
207596_at	4.83		
205660_at	4.82	2'-5'-oligoadenylate synthetase-like	OASL
232979_at	4.82		
212094_at	4.80	paternally expressed 10	PEG10
242815_x_at	4.78		
205513_at	4.78	transcobalamin I (vitamin B12 binding protein, R binder family)	TCN1
231467_at	4.77		
235934_at	4.74		
231587_at	4.74		
213436_at	4.73	cannabinoid receptor 1 (brain)	CNR1
207705_s_at	4.73		
222940_at	4.72	sulfotransferase family 1E, estrogen-preferring, member 1	SULT1E1
1558148_x_at	4.70		
1558756_at	4.70		
220468_at	4.69	ADP-ribosylation factor-like 14	ARL14
220830_at	4.68	interphotoreceptor matrix proteoglycan 2	IMPG2
1557544_at	4.68	chromosome 10 open reading frame 80	C10orf80
1561422_at	4.68		
231911_at	4.65	KIAA1189	KIAA1189
211145_x_at	4.64	interferon, alpha 21	IFNA21
1562103_at	4.64	Janus kinase 1 (a protein tyrosine kinase)	JAK1
230030_at	4.63	heparan sulfate 6-O-sulfotransferase 2	HS6ST2
1563827_at	4.62		
1562255_at	4.61	synaptotagmin-like 3	SYTL3
1557620_a_at	4.60	coiled-coil domain containing 38	CCDC38
226603_at	4.60	sterile alpha motif domain containing 9-like	SAMD9L
234188_at	4.59		
1554604_at	4.58	membrane-bound transcription factor peptidase, site 2	MBTPS2
1569072_s_at	4.54	ATP-binding cassette, sub-family B (MDR/TAP), member 5	ABCB5
235521_at	4.54	homeobox A3	HOXA3
231128_at	4.53		
213776_at	4.50		
235737_at	4.50		
202350_s_at	4.50	matrilin 2	MATN2
234612_at	4.48		
228057_at	4.48	DNA-damage-inducible transcript 4-like	DDIT4L

1561191_at	4.48		
217026_at	4.48	cystic fibrosis transmembrane conductance regulator (ATP-binding cassette sub-family C, member 7)	CFTR
209818_s_at	4.47	hyaluronan binding protein 4	HABP4
236866_at	4.44	galactosamine (N-acetyl)-6-sulfate sulfatase (Morquio syndrome, mucopolysaccharidosis type IVA)	GALNS
226145_s_at	4.42	Fraser syndrome 1	FRAS1
1553613_s_at	4.41	forkhead box C1	FOXC1
1562052_at	4.41		
215034_s_at	4.40	transmembrane 4 L six family member 1	TM4SF1
207951_at	4.39	casein beta	CSN2
206094_x_at	4.38	UDP glucuronosyltransferase 1 family, polypeptide A6	UGT1A6
212327_at	4.38		
208557_at	4.38	homeobox A6	HOXA6
210831_s_at	4.38	prostaglandin E receptor 3 (subtype EP3)	PTGER3
206385_s_at	4.37	ankyrin 3, node of Ranvier (ankyrin G)	ANK3
1559072_a_at	4.36	leucine rich repeat containing 62	LRRC62
209498_at	4.36	carcinoembryonic antigen-related cell adhesion molecule 1 (biliary glycoprotein)	CEACAM1
236034_at	4.35		
1555617_x_at	4.35		
1569387_at	4.35		
231156_at	4.34		
1562214_at	4.34		
217380_s_at	4.34		
1555043_at	4.34	lipoma HMGIC fusion partner-like 5	LHFPL5
1559035_a_at	4.33	aryl hydrocarbon receptor	AHR
234765_at	4.32		
231688_at	4.30		
220064_at	4.29	tetratricopeptide repeat domain 21B	TTC21B
236860_at	4.28	neuropeptide Y receptor Y6 (pseudogene)	NPY6R
230036_at	4.28	sterile alpha motif domain containing 9-like	SAMD9L
1553467_at	4.25		
1558549_s_at	4.24	vanin 1	VNN1
226637_at	4.24	ubiquitin-conjugating enzyme E2H (UBC8 homolog, yeast)	UBE2H
236071_at	4.24		
234004_at	4.22	tetratricopeptide repeat domain 28	TTC28
207498_s_at	4.22	cytochrome P450, family 2, subfamily D, polypeptide 6	CYP2D6
215288_at	4.22	transient receptor potential cation channel, subfamily C, member 2	TRPC2
242625_at	4.21	radical S-adenosyl methionine domain containing 2	RSAD2
1557823_s_at	4.20		
230743_at	4.19		
234744_x_at	4.19		
212097_at	4.19	caveolin 1, caveolae protein, 22kDa	CAV1
239381_at	4.19	kallikrein-related peptidase 7	KLK7
210426_x_at	4.18	RAR-related orphan receptor A	RORA
243271_at	4.18		

207178_s_at	4.17	fyn-related kinase	FRK
212353_at	4.16	sulfatase 1	SULF1
1567878_at	4.15	defensin, beta 114	DEFB114
227273_at	4.15		
208429_x_at	4.14	hepatocyte nuclear factor 4, alpha	HNF4A
1561676_at	4.14		
1554712_a_at	4.13	glycine-N-acyltransferase-like 2	GLYATL2
220418_at	4.13	ubiquitin associated and SH3 domain containing, A	UBASH3A
1563771_a_at	4.11		
233468_at	4.11		
1560318_at	4.11	Rho GTPase activating protein 29	ARHGAP29
241238_at	4.09		
1552939_at	4.09	angiopoietin 1	ANGPT1
1554314_at	4.08	chromosome 6 open reading frame 141	C6orf141
238402_s_at	4.07		
1563121_at	4.07		
211305_x_at	4.01	Fc fragment of IgA, receptor for	FCAR
206460_at	4.01	adherens junction associated protein 1	AJAP1
234970_at	4.01	membrane targeting (tandem) C2 domain containing 1	MTAC2D1
205688_at	4.00	transcription factor AP-4 (activating enhancer binding protein 4)	TFAP4
239959_x_at	4.00	phosphodiesterase 3B, cGMP-inhibited	PDE3B
1561002_at	4.00		
1562313_at	3.99	BCL6 co-repressor-like 2	BCORL2
225615_at	3.99		
241431_at	3.98		
207051_at	3.98	solute carrier family 17 (sodium phosphate), member 4	SLC17A4
224349_at	3.98		
227826_s_at	3.98		
1569855_at	3.97		
228821_at	3.97	ST6 beta-galactosamide alpha-2,6-sialyltranferase 2	ST6GAL2
1562543_at	3.96		
1557008_at	3.95		
1553920_at	3.95	chromosome 9 open reading frame 84	C9orf84
243854_at	3.95	amyloid beta (A4) precursor-like protein 2	APLP2
230096_at	3.94		
216557_x_at	3.94	interferon, alpha-inducible protein 6	IFI6
215862_at	3.93		
230564_at	3.93	signal-induced proliferation-associated 1 like 3	SIPA1L3
1561780_at	3.92		
45297_at	3.91	EH-domain containing 2	EHD2
240539_at	3.91	autism susceptibility candidate 2	AUTS2
232956_at	3.90		
221451_s_at	3.89	olfactory receptor, family 2, subfamily W, member 1	OR2W1
1568933_at	3.89		
224091_at	3.89		
233072_at	3.89	netrin G2	NTNG2
205380_at	3.88	PDZ domain containing 1	PDZK1
213197_at	3.86	astrotactin 1	ASTN1
238713_at	3.86		
235497_at	3.86		

234230_at	3.85		
218943_s_at	3.85	DEAD (Asp-Glu-Ala-Asp) box polypeptide 58	DDX58
227997_at	3.83	interleukin 17 receptor D	IL17RD
230560_at	3.83	syntaxin binding protein 6 (amisyn)	STXBP6
1560615_a_at	3.82		
1552394_a_at	3.82	ENTH domain containing 1	ENTHD1
1564077_at	3.81		
238634_x_at	3.81		
209763_at	3.81	chordin-like 1	CHRDL1
1554041_at	3.80	chromosome 20 open reading frame 141	C20orf141
209309_at	3.79	alpha-2-glycoprotein 1, zinc-binding	AZGP1
1565346_a_at	3.79	ATPase, Na+/K+ transporting, alpha 4 polypeptide	ATP1A4
1553452_at	3.79	myosin IH	MYO1H
240715_at	3.79	T-box 5	TBX5
241030_at	3.78	fibrous sheath interacting protein 1	FSIP1
213844_at	3.77	homeobox A5	HOXA5
1569637_at	3.76	zinc finger protein 100	ZNF100
225949_at	3.75	nuclear receptor binding protein 2	NRBP2
1557403_s_at	3.75		
237485_at	3.74		
1558247_s_at	3.73		
208470_s_at	3.72	haptoglobin-related protein	HPR
242002_at	3.72	T-cell lymphoma breakpoint associated target 1	TCBA1
1565735_at	3.71		
221605_s_at	3.70	pipecolic acid oxidase	PIPOX
203882_at	3.69	interferon-stimulated transcription factor 3, gamma 48kDa	ISGF3G
241777_x_at	3.69	adaptor protein, phosphotyrosine interaction, PH domain and leucine zipper containing 2	APPL2
1561424_at	3.69		
1563854_s_at	3.68		
210029_at	3.67	indoleamine-pyrrole 2,3 dioxygenase	INDO
239956_at	3.67		
242579_at	3.66		
216514_at	3.66		
1554739_at	3.66	intracisternal A particle-promoted polypeptide	IPP
228426_at	3.65	C-type lectin domain family 2, member D	CLEC2D
212077_at	3.65	caldesmon 1	CALD1
233666_at	3.64	transmembrane protein 106B	TMEM106B
233321_x_at	3.64		
243533_x_at	3.64	ankyrin repeat and sterile alpha motif domain containing 1B	ANKS1B
227484_at	3.63		
213797_at	3.63	radical S-adenosyl methionine domain containing 2	RSAD2
1562168_at	3.61		
1569813_at	3.61	striatin, calmodulin binding protein	STRN
231653_at	3.60	coiled-coil domain containing 129	CCDC129
203485_at	3.60	reticulon 1	RTN1
244571_s_at	3.59	tetratricopeptide repeat domain 12	TTC12
214596_at	3.57		
1556209_at	3.57	C-type lectin domain family 2, member B	CLEC2B
218326_s_at	3.56	leucine-rich repeat-containing G protein-coupled receptor 4	LGR4

240980_at	3.55	mesoderm induction early response 1 homolog (Xenopus laevis)	MIER1
241770_x_at	3.55	solute carrier family 22 (organic anion/cation transporter), member 9	SLC22A9
1564767_at	3.54		
1563802_at	3.54		
203065_s_at	3.54	caveolin 1, caveolae protein, 22kDa	CAV1
242719_at	3.53		
205132_at	3.53	actin, alpha, cardiac muscle 1	ACTC1
236719_at	3.53		
237261_at	3.52		
230000_at	3.52	ring finger protein 213	RNF213
1560187_at	3.51		
231585_at	3.51	vacuolar protein sorting 13 homolog A (S. cerevisiae)	VPS13A
234709_at	3.51	calpain 13	CAPN13
233170_at	3.51		
238874_at	3.49		
222549_at	3.48	claudin 1	CLDN1
243616_at	3.48		
219179_at	3.48	dapper, antagonist of beta-catenin, homolog 1 (Xenopus laevis)	DACT1
237434_x_at	3.46		
215228_at	3.46	nescient helix loop helix 2	NHLH2
32128_at	3.46	chemokine (C-C motif) ligand 18 (pulmonary and activation-regulated)	CCL18
236286_at	3.45		
232712_at	3.45	fibroblast growth factor 10	FGF10
1565358_at	3.45	retinoic acid receptor, alpha	RARA
214043_at	3.44	protein tyrosine phosphatase, receptor type, D	PTPRD
203896_s_at	3.44	phospholipase C, beta 4	PLCB4
231241_at	3.44		
224403_at	3.43	Fc receptor-like 4	FCRL4
228678_at	3.42	family with sequence similarity 116, member B	FAM116B
229631_at	3.42	dynein heavy chain domain 1	DNHD1
219501_at	3.42		
1560734_at	3.41	olfactory receptor, family 4, subfamily N, member 4	OR4N4
222059_at	3.41	zinc finger protein 335	ZNF335
243814_at	3.41	zinc finger, MYND-type containing 8	ZMYND8
1561817_at	3.40		
210660_at	3.40	leukocyte immunoglobulin-like receptor, subfamily A (with TM domain), member 1	LILRA1
235157_at	3.39		
233888_s_at	3.38	SLIT-ROBO Rho GTPase activating protein 1	SRGAP1
202820_at	3.38	aryl hydrocarbon receptor	AHR
219352_at	3.38	hect domain and RLD 6	HERC6
219230_at	3.37	transmembrane protein 100	TMEM100
237460_x_at	3.37		
228531_at	3.37	sterile alpha motif domain containing 9	SAMD9
240420_at	3.36	arylacetamide deacetylase-like 2	AADACL2
230117_at	3.36	V-set and transmembrane domain containing 2	VSTM2
243161_x_at	3.36	zinc finger protein 42 homolog (mouse)	ZFP42
244848_at	3.36	chromodomain helicase DNA binding protein 1-like	CHD1L

238182_at	3.36		
223220_s_at	3.36	poly (ADP-ribose) polymerase family, member 9	PARP9
205645_at	3.35	RALBP1 associated Eps domain containing 2	REPS2
206526_at	3.34	RIB43A domain with coiled-coils 2	RIBC2
1554319_at	3.34	ribosomal protein S6 kinase, 90kDa, polypeptide 5	RPS6KA5
1557286_at	3.34		
227949_at	3.33	phosphatase and actin regulator 3	PHACTR3
1563010_at	3.33		
212850_s_at	3.33	low density lipoprotein receptor-related protein 4	LRP4
232193_at	3.33	glutathione S-transferase theta 1	GSTT1
242036_x_at	3.33	ATPase, Ca++ transporting, plasma membrane 3	ATP2B3
231294_at	3.32	STT3, subunit of the oligosaccharyltransferase complex, homolog B (S. cerevisiae)	STT3B
213524_s_at	3.32	G0/G1switch 2	G0S2
1562311_at	3.30		
231561_s_at	3.30	apolipoprotein C-II	APOC2
1554601_at	3.30	T-cell lymphoma breakpoint associated target 1	TCBA1
1562084_at	3.30		
235967_at	3.30		
220316_at	3.30	neuronal PAS domain protein 3	NPAS3
201236_s_at	3.30	BTG family, member 2	BTG2
206289_at	3.30	homeobox A4	HOXA4
216368_s_at	3.29	collagen, type IV, alpha 3 (Goodpasture antigen)	COL4A3
238330_s_at	3.29		
1561463_at	3.29		
234472_at	3.29	UDP-N-acetyl-alpha-D-galactosamine:polypeptide N-acetylgalactosaminyltransferase 13 (GalNAc-T13)	GALNT13
240443_at	3.28		
215816_at	3.28	immunoglobulin lambda-like polypeptide 2	IGLL2
220640_at	3.28	casein kinase 1, gamma 1	CSNK1G1
241497_at	3.27		
239856_at	3.27		
223980_s_at	3.27	SP110 nuclear body protein	SP110
1553705_a_at	3.27	cholinergic receptor, muscarinic 3	CHRM3
214933_at	3.27	calcium channel, voltage-dependent, P/Q type, alpha 1A subunit	CACNA1A
241834_at	3.26		
237009_at	3.26	CD69 molecule	CD69
238206_at	3.26		
205572_at	3.25	angiopoietin 2	ANGPT2
1562562_at	3.24		
224401_s_at	3.24	Fc receptor-like 4	FCRL4
216118_at	3.24		
214341_at	3.24	adaptor-related protein complex 1, gamma 2 subunit	AP1G2
234284_at	3.23	guanine nucleotide binding protein (G protein), gamma 8	GNG8
242758_x_at	3.23		
232080_at	3.23	HECT, C2 and WW domain containing E3 ubiquitin protein ligase 2	HECW2
210834_s_at	3.21	prostaglandin E receptor 3 (subtype EP3)	PTGER3
233927_at	3.21		
207452_s_at	3.21	contactin 5	CNTN5
234768_at	3.20		

1569127_at	3.20		
1559529_at	3.20	PTK2 protein tyrosine kinase 2	PTK2
234958_at	3.20		
203789_s_at	3.20	sema domain, immunoglobulin domain (Ig), short basic domain, secreted, (semaphorin) 3C	SEMA3C
226189_at	3.20	integrin, beta 8	ITGB8
241359_at	3.20		
207206_s_at	3.19	arachidonate 12-lipoxygenase	ALOX12
1564241_at	3.19	ATPase, Na+/K+ transporting, alpha 4 polypeptide	ATP1A4
242172_at	3.19	Meis1, myeloid ecotropic viral integration site 1 homolog (mouse)	MEIS1
1555875_at	3.18		
211753_s_at	3.18	relaxin 1	RLN1
206769_at	3.18	thymosin, beta 4, Y-linked	TMSB4Y
235368_at	3.18	ADAM metallopeptidase with thrombospondin type 1 motif, 5 (aggrecanase-2)	ADAMTS5
241837_at	3.18	AT rich interactive domain 5B (MRF1-like)	ARID5B
204546_at	3.18	KIAA0513	KIAA0513
234204_at	3.17		
240863_at	3.17	cytochrome P450, family 19, subfamily A, polypeptide 1	CYP19A1
220180_at	3.16	coiled-coil domain containing 68	CCDC68
231354_at	3.16		
228285_at	3.15	tudor domain containing 9	TDRD9
225929_s_at	3.15	ring finger protein 213	RNF213
231697_s_at	3.15	transmembrane protein 49	TMEM49
241969_at	3.15	AT rich interactive domain 5B (MRF1-like)	ARID5B
1555929_s_at	3.13		
1563187_at	3.13		
209732_at	3.12	C-type lectin domain family 2, member B	CLEC2B
204932_at	3.12	tumor necrosis factor receptor superfamily, member 11b (osteoprotegerin)	TNFRSF11B
212820_at	3.11	Dmx-like 2	DMXL2
202145_at	3.11	lymphocyte antigen 6 complex, locus E	LY6E
209859_at	3.11	tripartite motif-containing 9	TRIM9
1557729_at	3.10	G protein-coupled receptor kinase 5	GRK5
203596_s_at	3.10	interferon-induced protein with tetratricopeptide repeats 5	IFIT5
231577_s_at	3.10	guanylate binding protein 1, interferon-inducible, 67kDa	GBP1
215071_s_at	3.09	histone cluster 1, H1e#histone cluster 1, H2bd#histone cluster 1, H2be#histone cluster 1, H2bc#histone cluster 1, H4d	HIST1H1E#HIST1H2BD#HIST1H2BE#HIST1H2BC#HIST1H4D
241081_at	3.09	UDP-N-acetyl-alpha-D-galactosamine:polypeptide N-acetylgalactosaminyltransferase 10 (GalNAc-T10)	GALNT10
233555_s_at	3.09	nuclear receptor coactivator 3#sulfatase 2	NCOA3#SULF2
230366_at	3.09		
1568736_s_at	3.09		
204755_x_at	3.08	hepatic leukemia factor	HLF
215704_at	3.08	filaggrin#null	FLG#null

223179_at	3.08	yippee-like 3 (Drosophila)	YPEL3
1554540_at	3.08	chromosome 1 open reading frame 67	C1orf67
205626_s_at	3.08	calbindin 1, 28kDa	CALB1
220697_at	3.07		
1552899_at	3.07		
234577_at	3.07		
233880_at	3.06	ring finger protein 213	RNF213
1562894_at	3.06		
1560503_a_at	3.06		
1558183_at	3.06	zinc finger protein 17	ZNF17
227174_at	3.05	WD repeat domain 72	WDR72
240095_at	3.05		
212494_at	3.05	tensin like C1 domain containing phosphatase (tensin 2)	TENC1
206794_at	3.04	v-erb-a erythroblastic leukemia viral oncogene homolog 4 (avian)	ERBB4
240977_at	3.04	leucine-rich repeats and calponin homology (CH) domain containing 1	LRCH1
222793_at	3.04	DEAD (Asp-Glu-Ala-Asp) box polypeptide 58	DDX58
220154_at	3.04	dystonin	DST
1561529_at	3.04		
1568826_at	3.04		
230145_at	3.04	dihydrouridine synthase 3-like (S. cerevisiae)	DUS3L
228202_at	3.03	phospholamban	PLN
204509_at	3.03	carbonic anhydrase XII	CA12
232517_s_at	3.02	eukaryotic translation elongation factor 1 alpha 2#potassium voltage-gated channel, KQT-like subfamily, member 2#potassium voltage-gated channel, KQT-like subfamily, member 2#PTK6 protein tyrosine kinase 6#src-related kinase lacking C- terminal regulatory tyrosine and N-terminal myristylation sites#chromosome 20 open reading frame 195#chromosome 20 open reading frame 149#null	EEF1A2#KCNQ2#KCNQ2#PTK6#SRMS#C20orf195#C20orf149#null
240704_at	3.02		
1567612_at	3.02		
228985_at	3.02	oxysterol binding protein-like 8	OSBPL8
237130_at	3.01		
242765_at	3.01	myelin-associated oligodendrocyte basic protein	MOBP
215456_at	3.01		
1552791_a_at	3.00	triadin	TRDN
1558651_at	3.00		
213358_at	3.00	KIAA0802	KIAA0802
242459_at	3.00		
212224_at	2.99	aldehyde dehydrogenase 1 family, member A1	ALDH1A1
231733_at	2.99		
235431_s_at	2.99	pellino homolog 3 (Drosophila)	PELI3
204753_s_at	2.99	hepatic leukemia factor	HLF
1561813_at	2.99		
220483_s_at	2.99	ring finger protein 19	RNF19
205637_s_at	2.98	SH3-domain GRB2-like 3	SH3GL3
241184_x_at	2.98	zinc finger protein 407	ZNF407
208446_s_at	2.98	zinc finger, FYVE domain containing 9	ZFYVE9

243846_x_at	2.97		
236300_at	2.96		
206932_at	2.96	cholesterol 25-hydroxylase	CH25H
1565073_at	2.96		
234652_at	2.96		
244175_at	2.95		
204748_at	2.95	prostaglandin-endoperoxide synthase 2 (prostaglandin G/H synthase and cyclooxygenase)	PTGS2
1552715_a_at	2.95	relaxin/insulin-like family peptide receptor 1	RXFP1
234632_x_at	2.94		
206515_at	2.94	cytochrome P450, family 4, subfamily F, polypeptide 3	CYP4F3
232585_at	2.94	tousled-like kinase 2	TLK2
230840_at	2.94		
1554325_at	2.94	dedicator of cytokinesis 2	DOCK2
239791_at	2.93		
234810_at	2.93	zinc finger protein 1 homolog (mouse)	ZFP1
241618_at	2.93		
214485_at	2.92	outer dense fiber of sperm tails 1	ODF1
1552736_a_at	2.92	neuropilin (NRP) and tolloid (TLL)-like 1	NETO1
206224_at	2.92	cystatin SN	CST1
231880_at	2.92	family with sequence similarity 40, member B	FAM40B
243861_at	2.92		
219783_at	2.91	chromosome 2 open reading frame 18	C2orf18
222899_at	2.91	integrin, alpha 11	ITGA11
242721_at	2.91	autism susceptibility candidate 2	AUTS2
232504_at	2.90		
211824_x_at	2.90	NLR family, pyrin domain containing 1	NLRP1
232766_at	2.90	chromosome 20 open reading frame 179	C20orf179
206035_at	2.90	v-rel reticuloendotheliosis viral oncogene homolog (avian)	REL
227679_at	2.90		
232787_at	2.90		
221251_x_at	2.90	zinc finger, HIT type 4	ZNHIT4
1558425_x_at	2.90		
242907_at	2.89		
216956_s_at	2.89	integrin, alpha 2b (platelet glycoprotein IIb of IIb/IIIa complex, antigen CD41)	ITGA2B
1570181_a_at	2.89		
240217_s_at	2.89		
234276_at	2.89		
242321_at	2.89		
1563461_at	2.89		
1556461_at	2.88		
231578_at	2.87	guanylate binding protein 1, interferon-inducible, 67kDa	GBP1
219049_at	2.87		
228121_at	2.87		
234640_x_at	2.87		
1560282_at	2.87		
1568812_at	2.87		
209969_s_at	2.87	signal transducer and activator of transcription 1, 91kDa	STAT1

1569652_at	2.86	myeloid/lymphoid or mixed-lineage leukemia (trithorax homolog, Drosophila); translocated to, 3	MLLT3
209327_s_at	2.86		
1557520_a_at	2.86	transmembrane protein 59	TMEM59
1569263_at	2.86		
207017_at	2.86	RAB27B, member RAS oncogene family	RAB27B
237660_at	2.86		
233797_s_at	2.86	cystatin 11	CST11
239371_at	2.85	forkhead box K2	FOXK2
209010_s_at	2.85	triple functional domain (PTPRF interacting)	TRIO
219534_x_at	2.85	cyclin-dependent kinase inhibitor 1C (p57, Kip2)	CDKN1C
1559587_at	2.84	symplekin	SYMPK
230446_at	2.84		
243702_at	2.84		
1566869_at	2.84		
232449_at	2.84	beta-carotene dioxygenase 2	BCDO2
228152_s_at	2.83		
229374_at	2.83	EPH receptor A4	EPHA4
230167_at	2.83	ADAM metallopeptidase with thrombospondin type 1 motif, 14	ADAMTS14
229213_at	2.83		
243428_at	2.83	KCNQ1 overlapping transcript 1	KCNQ1OT1
1553271_at	2.82	DIP2 disco-interacting protein 2 homolog B (Drosophila)	DIP2B
229309_at	2.82		
226207_at	2.82		
206806_at	2.82	diacylglycerol kinase, iota	DGKI
228904_at	2.81	homeobox B3	HOXB3
235074_at	2.81	sprouty-related, EVH1 domain containing 1	SPRED1
244097_at	2.81	complement component (3d/Epstein Barr virus) receptor 2	CR2
237655_at	2.81		
213182_x_at	2.80	cyclin-dependent kinase inhibitor 1C (p57, Kip2)	CDKN1C
1558534_at	2.80		
211883_x_at	2.80	carcinoembryonic antigen-related cell adhesion molecule 1 (biliary glycoprotein)	CEACAM1
231947_at	2.79	myc target 1	MYCT1
232742_at	2.79		
1566798_at	2.79		
210262_at	2.79	cysteine-rich secretory protein 2	CRISP2
218986_s_at	2.79		
224213_at	2.79	chromosome 14 open reading frame 91	C14orf91
205547_s_at	2.79	transgelin	TAGLN
202269_x_at	2.78	guanylate binding protein 1, interferon-inducible, 67kDa	GBP1
216278_at	2.78		
1554996_at	2.78	zinc finger protein 479	ZNF479
201242_s_at	2.78	ATPase, Na+/K+ transporting, beta 1 polypeptide	ATP1B1
216821_at	2.78	Sec61 beta subunit#asparagine-linked glycosylation 2 homolog (S. cerevisiae, alpha-1,3-mannosyltransferase)#keratin 8-like 1	SEC61B#ALG2#KRT8L1
1552515_at	2.78	homeodomain interacting protein kinase 1	HIPK1

206552_s_at	2.78	tachykinin, precursor 1 (substance K, substance P, neurokinin 1, neurokinin 2, neuromedin L, neuroki	TAC1
228708_at	2.77		
1560031_at	2.77	FERM domain containing 4A	FRMD4A
238921_at	2.76		
240979_at	2.76		
211267_at	2.76	homeobox, ES cell expressed 1	HESX1
1562656_at	2.76		
219452_at	2.76	dipeptidase 2	DPEP2
215539_at	2.76		
1556739_at	2.76		
1556518_at	2.75		
239710_at	2.75		
243689_s_at	2.75		
1563273_at	2.74		
44783_s_at	2.74	hairy/enhancer-of-split related with YRPW motif 1	HEY1
220749_at	2.74	chromosome 10 open reading frame 68	C10orf68
231956_at	2.74	KIAA1618	KIAA1618
232610_at	2.73	poly (ADP-ribose) polymerase family, member 14	PARP14
232304_at	2.73	pellino homolog 1 (Drosophila)	PELI1
209905_at	2.73	homeobox A9	HOXA9
215616_s_at	2.73	jumonji domain containing 2B	JMJD2B
1559376_at	2.72	chromosome 1 open reading frame 203	C1orf203
215663_at	2.72	muscleblind-like (Drosophila)	MBNL1
1562901_at	2.72		
1565582_at	2.72		
1555394_at	2.72	phosphatidylinositol glycan anchor biosynthesis, class K	PIGK
1560251_at	2.72		
238755_at	2.71		
206697_s_at	2.71	haptoglobin	HP
212950_at	2.70	G protein-coupled receptor 116	GPR116
231616_at	2.70	glycophorin A (MNS blood group)	GYPA
225109_at	2.69	2-oxoglutarate and iron-dependent oxygenase domain containing 1	OGFOD1
1557535_at	2.69	palladin, cytoskeletal associated protein	PALLD
238595_at	2.69		
201601_x_at	2.69	interferon induced transmembrane protein 1 (9-27)	IFITM1
1563369_at	2.69		
206525_at	2.69	gamma-aminobutyric acid (GABA) receptor, rho 1	GABRR1
211488_s_at	2.68	integrin, beta 8	ITGB8
244194_at	2.68		
206828_at	2.68	TXK tyrosine kinase	TXK
226638_at	2.68	Rho GTPase activating protein 23	ARHGAP23
227807_at	2.68	poly (ADP-ribose) polymerase family, member 9	PARP9
226407_at	2.68	reticulocalbin 1, EF-hand calcium binding domain	RCN1
244839_at	2.68	titin	TTN
211590_x_at	2.67	thromboxane A2 receptor	TBXA2R
221525_at	2.67	zinc finger, MIZ-type containing 2	ZMIZ2
229957_at	2.67	branched chain keto acid dehydrogenase E1, alpha polypeptide	BCKDHA
1566046_at	2.67		
240180_at	2.67		

220168_at	2.66	cancer susceptibility candidate 1	CASC1
234067_at	2.66		
1554997_a_at	2.66	prostaglandin-endoperoxide synthase 2 (prostaglandin G/H synthase and cyclooxygenase)	PTGS2
223784_at	2.66	transmembrane protein 27	TMEM27
234832_at	2.65	anthrax toxin receptor 1	ANTXR1
1554844_at	2.65	eyes absent homolog 3 (Drosophila)	EYA3
224701_at	2.65	poly (ADP-ribose) polymerase family, member 14	PARP14
208194_s_at	2.65	signal transducing adaptor molecule (SH3 domain and ITAM motif) 2	STAM2
232029_at	2.65		
241421_at	2.65		
1558982_at	2.64		
209169_at	2.64	glycoprotein M6B	GPM6B
217033_x_at	2.64	neurotrophic tyrosine kinase, receptor, type 3	NTRK3
1557232_at	2.64		
226534_at	2.64	KIT ligand	KITLG
205208_at	2.63	aldehyde dehydrogenase 1 family, member L1	ALDH1L1
1566665_at	2.63		
208339_at	2.63	XK, Kell blood group complex subunit-related, Y-linked	XKRY
209374_s_at	2.63	immunoglobulin heavy constant mu	IGHM
206446_s_at	2.63	elastase 1, pancreatic	ELA1
1570628_at	2.63		
1561371_at	2.62		
213058_at	2.62	tetratricopeptide repeat domain 28	TTC28
210437_at	2.62	melanoma antigen family A, 9#null#null	MAGEA9#null#null
207489_at	2.62		
234389_x_at	2.62		
1553883_at	2.61	zinc finger protein 99	ZNF99
241508_at	2.61	ankyrin repeat domain 12	ANKRD12
1553011_at	2.61	TAF1-like RNA polymerase II, TATA box binding protein (TBP)-associated factor, 210kDa	TAF1L
1570039_at	2.61		
239401_at	2.61		
206620_at	2.60	GRB2-related adaptor protein	GRAP
1563469_at	2.60		
1562583_s_at	2.60		
204237_at	2.60	GULP, engulfment adaptor PTB domain containing 1	GULP1
202180_s_at	2.60	major vault protein	MVP
231985_at	2.60	microtubule associated monoxygenase, calponin and LIM domain containing 3	MICAL3
1560760_s_at	2.60		
211327_x_at	2.60	hemochromatosis	HFE
228718_at	2.59	zinc finger protein 44	ZNF44
210797_s_at	2.59	2'-5'-oligoadenylate synthetase-like	OASL
1552690_a_at	2.59	calcium channel, voltage-dependent, alpha 2/delta subunit 4	CACNA2D4
230093_at	2.59	testis specific A2 homolog (mouse)	TSGA2
212392_s_at	2.59	phosphodiesterase 4D interacting protein (myomegalin)	PDE4DIP

214375_at	2.59	PTPRF interacting protein, binding protein 1 (liprin beta 1)	PPFIBP1
234135_x_at	2.59		
229684_s_at	2.59	zinc finger protein 644	ZNF644
232375_at	2.59		
218665_at	2.59	frizzled homolog 4 (Drosophila)	FZD4
231854_at	2.58		
1555392_at	2.58		
210108_at	2.58	calcium channel, voltage-dependent, L type, alpha 1D subunit	CACNA1D
226550_at	2.58		
213832_at	2.58		
1557223_at	2.58	succinate dehydrogenase complex, subunit A, flavoprotein pseudogene 2	SDHALP2
1560483_at	2.58		
1559979_at	2.58	SYF2 homolog, RNA splicing factor (S. cerevisiae)	SYF2
233681_at	2.57	keratin associated protein 3-3	KRTAP3-3
1563466_at	2.57	myosin, light chain kinase	MYLK
210375_at	2.56	prostaglandin E receptor 3 (subtype EP3)	PTGER3
239367_at	2.56	brain-derived neurotrophic factor	BDNF
1568745_at	2.56		
232986_at	2.56	zinc finger protein 225#zinc finger protein 226#zinc finger protein 227#zinc finger protein 234#zinc finger protein 233	ZNF225#ZNF226#ZNF227#ZNF234#ZNF233
224346_at	2.56	RNA binding protein S1, serine-rich domain	RNPS1
221085_at	2.55	tumor necrosis factor (ligand) superfamily, member 15	TNFSF15
202458_at	2.55	protease, serine, 23	PRSS23
236884_at	2.55		
236942_at	2.55		
229170_s_at	2.55	tetratricopeptide repeat domain 18	TTC18
1559037_a_at	2.55		
237893_at	2.55		
221729_at	2.55		
1555051_at	2.55	chromosome 10 open reading frame 53	C10orf53
1562933_at	2.55		
1561740_at	2.55		
226756_at	2.54		
214022_s_at	2.54	interferon induced transmembrane protein 1 (9-27)	IFITM1
233430_at	2.54	TBC1 domain family, member 22B	TBC1D22B
242443_at	2.54	echinoderm microtubule associated protein like 5	EML5
244202_at	2.54	bromodomain and PHD finger containing, 3	BRPF3
1555370_a_at	2.54	calmodulin binding transcription activator 1	CAMTA1
227310_at	2.54	adenylosuccinate synthase	ADSS
1569411_at	2.54	transmembrane protein 67	TMEM67
53720_at	2.54		
243474_at	2.54		
206586_at	2.54	cannabinoid receptor 2 (macrophage)	CNR2
238067_at	2.54	TBC1 domain family, member 8B (with GRAM domain)	TBC1D8B
226211_at	2.54	maternally expressed 3	MEG3
224438_at	2.53		

219209_at	2.53	interferon induced with helicase C domain 1	IFIH1
1556359_at	2.53	chromosome 6 open reading frame 89	C6orf89
240728_at	2.53	phospholipase C, beta 4	PLCB4
1565723_at	2.53	LSM domain containing 1	LSMD1
204082_at	2.53	pre-B-cell leukemia homeobox 3	PBX3
231476_at	2.52		
241409_at	2.52		
243920_x_at	2.52		
1559633_a_at	2.52	cholinergic receptor, muscarinic 3	CHRM3
206521_s_at	2.51	general transcription factor IIA, 1, 19/37kDa	GTF2A1
230050_at	2.51	BTB (POZ) domain containing 14A	BTBD14A
1555244_at	2.51		
244658_at	2.51		
217558_at	2.51	cytochrome P450, family 2, subfamily C, polypeptide 9	CYP2C9
1556486_at	2.51		
241418_at	2.51		
214438_at	2.51	H2.0-like homeobox 1 (Drosophila)	HLX1
205729_at	2.51	oncostatin M receptor	OSMR
238327_at	2.51		
241099_at	2.50	elongation protein 4 homolog (S. cerevisiae)	ELP4
202708_s_at	2.50	histone cluster 2, H2be	HIST2H2BE
215478_at	2.50	regulating synaptic membrane exocytosis 2	RIMS2
209761_s_at	2.50	SP110 nuclear body protein	SP110
243095_at	2.50		
1557712_x_at	2.50		
231132_at	2.49		
240400_at	2.49		
240477_at	2.49	ets variant gene 1	ETV1
223875_s_at	2.49	enhancer of polycomb homolog 1 (Drosophila)	EPC1
232695_at	2.49	kinesin family member 6	KIF6
207788_s_at	2.49	sorbin and SH3 domain containing 3	SORBS3
214984_at	2.49		
239715_at	2.49		
224321_at	2.49	transmembrane protein with EGF-like and two follistatin-like domains 2	TMEFF2
216183_at	2.49	transglutaminase 2 (C polypeptide, protein-glutamine-gamma-glutamyltransferase)	TGM2
200923_at	2.49	lectin, galactoside-binding, soluble, 3 binding protein	LGALS3BP
213362_at	2.49	protein tyrosine phosphatase, receptor type, D	PTPRD

217394_at	2.49	T cell receptor alpha locus#T cell receptor alpha variable 6#T cell receptor alpha variable 16#T cell receptor alpha variable 15#T cell receptor alpha variable 14/delta variable 4#T cell receptor alpha variable 13-2#T cell receptor alpha variable 13-1#T cell receptor alpha variable 12-3#T cell receptor alpha variable 12-2#T cell receptor alpha variable 12-1#T cell receptor alpha variable 11#T cell receptor alpha variable 10#T cell receptor alpha variable 9-2#T cell receptor alpha variable 9-1#T cell receptor alpha variable 8-6#T cell receptor alpha variable 8-5#T cell receptor alpha variable 8-4#T cell receptor alpha variable 8-3#T cell receptor alpha variable 8-2#T cell receptor alpha variable 8-1#T cell receptor alpha variable 7#T cell receptor alpha variable 5#null	TRA@#TRAV6#TRAV16#TRAV15#TRAV14DV4#TRAV13-2#TRAV13-1#TRAV12-3#TRAV12-2#TRAV12-1#TRAV11#TRAV10#TRAV9-2#TRAV9-1#TRAV8-6#TRAV8-5#TRAV8-4#TRAV8-3#TRAV8-2#TRAV8-1#TRAV7#TRAV5#null
1568638_a_at	2.48	indoleamine-pyrrole 2,3 dioxygenase-like 1	INDOL1
218429_s_at	2.48		
1552438_a_at	2.48	ankyrin and armadillo repeat containing	ANKAR
1557211_a_at	2.48	chromosome 14 open reading frame 86	C14orf86
1560449_at	2.48		
1556113_at	2.48		
1557410_at	2.48		
240007_at	2.48		
206651_s_at	2.47	carboxypeptidase B2 (plasma, carboxypeptidase U)	CPB2
239556_at	2.47		
208250_s_at	2.47	deleted in malignant brain tumors 1	DMBT1
230640_at	2.47		
1561442_at	2.47		
210145_at	2.46	phospholipase A2, group IVA (cytosolic, calcium-dependent)	PLA2G4A
1553204_at	2.46		
208392_x_at	2.46	SP110 nuclear body protein	SP110
234968_at	2.46	DENN/MADD domain containing 4C	DENND4C
244263_at	2.46	histone deacetylase 7A	HDAC7A
218687_s_at	2.46	mucin 13, cell surface associated	MUC13
219194_at	2.46	sema domain, immunoglobulin domain (Ig), transmembrane domain (TM) and short cytoplasmic domain, (se	SEMA4G
221967_at	2.46	neurexophilin 4	NXPH4
220595_at	2.46	PDZ domain containing RING finger 4	PDZRN4
234178_at	2.46		
203020_at	2.46	RAB GTPase activating protein 1-like	RABGAP1L
225688_s_at	2.46	pleckstrin homology-like domain, family B, member 2	PHLDB2
231035_s_at	2.45	OTU domain containing 1	OTUD1
210861_s_at	2.45	WNT1 inducible signaling pathway protein 3	WISP3
1569270_at	2.45		
227497_at	2.45		
241063_at	2.45	ankyrin repeat domain 28	ANKRD28

242732_at	2.45	metastasis suppressor 1	MTSS1
244471_x_at	2.45	pannexin 2	PANX2
205081_at	2.45	cysteine-rich protein 1 (intestinal)	CRIP1
209417_s_at	2.45	interferon-induced protein 35	IFI35
202241_at	2.44	tribbles homolog 1 (Drosophila)	TRIB1
244511_at	2.44	phosphodiesterase 4D interacting protein (myomegalin)	PDE4DIP
234808_x_at	2.44		
238390_at	2.44	G protein-coupled receptor 39	GPR39
215197_at	2.44		
1569124_at	2.43		
1558760_at	2.43		
214776_x_at	2.43	xylulokinase homolog (H. influenzae)	XYLB
213690_s_at	2.42		
1569272_at	2.42	phosphoinositide-3-kinase, class 3	PIK3C3
242792_at	2.42	nuclear factor I/B	NFIB
241804_at	2.42		
233449_at	2.42		
1561877_at	2.42		
1554816_at	2.42	astrotactin 2	ASTN2
208965_s_at	2.42	interferon, gamma-inducible protein 16	IFI16
242135_at	2.41		
216887_s_at	2.41	LIM domain binding 3	LDB3
1554143_a_at	2.41	SGT1, suppressor of G2 allele of SKP1 like 1 (S. cerevisiae)	SUGT1L1
233817_at	2.41		
204633_s_at	2.41	ribosomal protein S6 kinase, 90kDa, polypeptide 5	RPS6KA5
220839_at	2.41	methyltransferase like 5	METTL5
213004_at	2.41	angiopoietin-like 2	ANGPTL2
1554840_at	2.41		
237431_at	2.41	ubiquitin specific peptidase 32	USP32
213348_at	2.40	cyclin-dependent kinase inhibitor 1C (p57, Kip2)	CDKN1C
238733_at	2.40		
216828_at	2.40	chromosome 20 open reading frame 80#null	C20orf80#null
243576_at	2.40		
214651_s_at	2.40	homeobox A9	HOXA9
201744_s_at	2.40	lumican	LUM
204917_s_at	2.40	myeloid/lymphoid or mixed-lineage leukemia (trithorax homolog, Drosophila); translocated to, 3	MLLT3
205600_x_at	2.40	homeobox B5	HOXB5
1556794_at	2.40		
242488_at	2.40		
224917_at	2.39	transmembrane protein 49	TMEM49
232779_at	2.39		
232768_at	2.39	cyclin B2	CCNB2
204547_at	2.39	RAB40B, member RAS oncogene family	RAB40B
1565595_at	2.39		
241751_at	2.39	oral-facial-digital syndrome 1	OFD1
229350_x_at	2.39	poly (ADP-ribose) polymerase family, member 10	PARP10
231543_at	2.39	pleckstrin homology domain containing, family A (phosphoinositide binding specific) member 8	PLEKHA8
244533_at	2.39		
212827_at	2.39	immunoglobulin heavy constant mu	IGHM

1568699_at	2.39	chromosome 14 open reading frame 179	C14orf179
1568615_a_at	2.38		
222969_at	2.38	UDP-Gal:betaGlcNAc beta 1,3-galactosyltransferase, polypeptide 1	B3GALT1
236349_at	2.38		
240448_at	2.38	KIAA0802	KIAA0802
1558785_a_at	2.38	regulator of G-protein signalling 5	RGS5
1564805_a_at	2.38	orofacial cleft 1 candidate 1	OFCC1
227057_at	2.38	Rho GTPase activating protein 27	ARHGAP27
242734_x_at	2.38	galactose-1-phosphate uridylyltransferase	GALT
1552943_at	2.37	gamma-aminobutyric acid (GABA) A receptor, gamma 1	GABRG1
238488_at	2.37	importin 11	IPO11
222970_at	2.37		
215962_at	2.37		
200872_at	2.37	S100 calcium binding protein A10	S100A10
236311_at	2.36	loss of heterozygosity, 12, chromosomal region 2	LOH12CR2
1563611_at	2.36		
215783_s_at	2.36	alkaline phosphatase, liver/bone/kidney	ALPL
244764_at	2.36		
204529_s_at	2.36		
220315_at	2.36	poly (ADP-ribose) polymerase family, member 11	PARP11
204044_at	2.35	quinolinate phosphoribosyltransferase (nicotinate-nucleotide pyrophosphorylase (carboxylating))	QPRT
232705_at	2.35	leucine rich repeat (in FLII) interacting protein 2	LRRFIP2
223316_at	2.35	coiled-coil domain containing 3	CCDC3
213338_at	2.35	transmembrane protein 158	TMEM158
223655_at	2.35	CD163 molecule-like 1	CD163L1
1552752_a_at	2.35	cell adhesion molecule 2	CADM2
239923_at	2.35		
235761_at	2.35		
225636_at	2.35	signal transducer and activator of transcription 2, 113kDa	STAT2
206553_at	2.35	2'-5'-oligoadenylate synthetase 2, 69/71kDa	OAS2
214639_s_at	2.35	homeobox A1	HOXA1
1556963_at	2.34		
1570169_at	2.34	CUB and Sushi multiple domains 2	CSMD2
224259_at	2.34	wingless-type MMTV integration site family, member 8A	WNT8A
240656_at	2.34	signal-induced proliferation-associated 1 like 1	SIPA1L1
201641_at	2.34	bone marrow stromal cell antigen 2	BST2
1557177_at	2.34		
243939_at	2.34		
240964_at	2.34		
201556_s_at	2.34	vesicle-associated membrane protein 2 (synaptobrevin 2)	VAMP2
1557256_a_at	2.34		
230753_at	2.34		
235301_at	2.33	KIAA1324-like	KIAA1324L
1556580_a_at	2.33		
226716_at	2.33	proline rich 12	PRR12
223943_s_at	2.33	guanine nucleotide binding protein (G protein), gamma 2	GNG2

242142_at	2.33		
217614_at	2.33		
232831_at	2.33		
239808_at	2.33		
234989_at	2.33		
229802_at	2.33		
218543_s_at	2.32	poly (ADP-ribose) polymerase family, member 12	PARP12
218675_at	2.32	solute carrier family 22 (organic cation transporter), member 17	SLC22A17
1561289_at	2.32		
238185_at	2.32		
217261_at	2.32	testis-specific transcript, Y-linked 2	TTTY2
216877_at	2.32		
207466_at	2.32	galanin	GAL
206747_at	2.32	G protein regulated inducer of neurite outgrowth 2	GPRIN2
204187_at	2.32	guanosine monophosphate reductase	GMPR
231735_s_at	2.31		
234522_at	2.31		
206310_at	2.31	serine peptidase inhibitor, Kazal type 2 (acrosin-trypsin inhibitor)	SPINK2
209108_at	2.31	tetraspanin 6	TSPAN6
215321_at	2.31		
220613_s_at	2.31	synaptotagmin-like 2	SYTL2
239130_at	2.31		
228206_at	2.31	heparan sulfate (glucosamine) 3-O-sulfotransferase 4	HS3ST4
204359_at	2.31	fibronectin leucine rich transmembrane protein 2	FLRT2
241216_at	2.31	kinesin family member 1B	KIF1B
237244_at	2.31		
241347_at	2.30	KIAA1618	KIAA1618
206916_x_at	2.30	tyrosine aminotransferase	TAT
1556801_at	2.30		
244265_at	2.30	arginine-glutamic acid dipeptide (RE) repeats	RERE
1557113_at	2.30		
239642_at	2.30		
241016_at	2.30	cullin 3	CUL3
233024_at	2.30	RAB18, member RAS oncogene family	RAB18
218045_x_at	2.30	parathymosin	PTMS
213813_x_at	2.29	RNA binding protein S1, serine-rich domain	RNPS1
1561615_s_at	2.29	solute carrier family 8 (sodium/calcium exchanger), member 1	SLC8A1
241951_at	2.29		
205991_s_at	2.29	paired related homeobox 1	PRRX1
236344_at	2.29		
1552908_at	2.29	chromosome 1 open reading frame 150	C1orf150
204684_at	2.29	neuronal pentraxin I	NPTX1
1558871_at	2.29		
221884_at	2.29	ecotropic viral integration site 1	EVI1
203476_at	2.29	trophoblast glycoprotein	TPBG
219691_at	2.29	sterile alpha motif domain containing 9	SAMD9
234074_at	2.28		
240226_at	2.28	ubiquitin specific peptidase 8	USP8
234097_s_at	2.28	chromosome 6 open reading frame 12	C6orf12

232158_x_at	2.28	NIPA-like domain containing 1	NPAL1
236338_at	2.28	insulin receptor substrate 2	IRS2
227496_at	2.28		
215591_at	2.27	SATB family member 2	SATB2
221943_x_at	2.27	ribosomal protein L38	RPL38
214294_at	2.27		
1560756_at	2.27		
1560705_at	2.27		
236806_at	2.27		
237632_at	2.27	hect (homologous to the E6-AP (UBE3A) carboxyl terminus) domain and RCC1 (CHC1)-like domain (RLD) 1	HERC1
1556964_s_at	2.26		
236385_at	2.26		
205942_s_at	2.26	acyl-CoA synthetase medium-chain family member 3	ACSM3
1565801_at	2.26		
230092_at	2.26	UBX domain containing 3	UBXD3
216563_at	2.25	ankyrin repeat domain 12	ANKRD12
224082_at	2.25		
216858_x_at	2.25		
1557826_at	2.25		
229540_at	2.24	recombining binding protein suppressor of hairless (Drosophila)	RBPSUH
215475_at	2.24		
234743_at	2.24	LIM domains containing 1	LIMD1
1562908_at	2.24		
203921_at	2.24	carbohydrate (N-acetylglucosamine-6-O) sulfotransferase 2	CHST2
1559412_at	2.24		
231833_at	2.24	proline rich 8	PRR8
244359_s_at	2.24		
242414_at	2.24		
1570111_at	2.24	chromosome 14 open reading frame 48	C14orf48
1569516_at	2.24	chromosome 6 open reading frame 153	C6orf153
240516_at	2.23		
240088_at	2.23	phosphodiesterase 5A, cGMP-specific	PDE5A
244310_at	2.23		
206756_at	2.23	carbohydrate (N-acetylglucosamine 6-O) sulfotransferase 7	CHST7
1570484_at	2.23		
219871_at	2.23		
220787_at	2.23		
204635_at	2.23	ribosomal protein S6 kinase, 90kDa, polypeptide 5	RPS6KA5
205608_s_at	2.23	angiopoietin 1	ANGPT1
236285_at	2.22		
234785_at	2.22		
224558_s_at	2.22	metastasis associated lung adenocarcinoma transcript 1 (non-coding RNA)	MALAT1
212599_at	2.22	autism susceptibility candidate 2	AUTS2
212328_at	2.22		
236453_at	2.22		
1554225_a_at	2.22		
205681_at	2.22	BCL2-related protein A1	BCL2A1

224548_at	2.22	hairy and enhancer of split 7 (Drosophila)	HES7
1554286_at	2.21		
205790_at	2.21	src kinase associated phosphoprotein 1	SKAP1
1562294_x_at	2.21	ankyrin repeat domain 30B	ANKRD30B
232907_at	2.21	zinc finger, UBR1 type 1	ZUBR1
209442_x_at	2.21	ankyrin 3, node of Ranvier (ankyrin G)	ANK3
219863_at	2.21	hect domain and RLD 5	HERC5
37170_at	2.21	BMP2 inducible kinase	BMP2K
241617_x_at	2.21		
203788_s_at	2.21	sema domain, immunoglobulin domain (Ig), short basic domain, secreted, (semaphorin) 3C	SEMA3C
220537_at	2.21	myotubularin related protein 8	MTMR8
229065_at	2.21	solute carrier family 35, member F3	SLC35F3
236116_at	2.21		
233175_at	2.21	zinc finger protein 460	ZNF460
205802_at	2.21	transient receptor potential cation channel, subfamily C, member 1	TRPC1
1557046_x_at	2.20		
1559063_at	2.20		
241346_at	2.20	Rho GTPase activating protein 30	ARHGAP30
203808_at	2.20	v-akt murine thymoma viral oncogene homolog 2	AKT2
237100_at	2.20	janus kinase and microtubule interacting protein 2	JAKMIP2
240835_at	2.20		
1559648_at	2.20		
243559_at	2.20		
220575_at	2.20	family with sequence similarity 106, member B	FAM106B
1561868_at	2.20		
228150_at	2.20	leucine zipper transcription regulator 2	LZTR2
232881_at	2.19		
1561014_at	2.19		
211621_at	2.19	androgen receptor (dihydrotestosterone receptor; testicular feminization; spinal and bulbar muscular	AR
209079_x_at	2.19	protocadherin gamma subfamily A, 1	PCDHGA1
224104_at	2.19		
229953_x_at	2.19	chromosome 6 open reading frame 152	C6orf152
244695_at	2.19		
207018_s_at	2.19	RAB27B, member RAS oncogene family	RAB27B
203461_at	2.19	chromodomain helicase DNA binding protein 2	CHD2
1558540_s_at	2.19		
215246_at	2.19	La ribonucleoprotein domain family, member 7	LARP7
210432_s_at	2.19	sodium channel, voltage-gated, type III, alpha subunit	SCN3A
226956_at	2.19		
213294_at	2.19		
205599_at	2.18	TNF receptor-associated factor 1	TRAF1
219684_at	2.18	receptor (chemosensory) transporter protein 4	RTP4
206194_at	2.18	homeobox C4	HOXC4
219232_s_at	2.18	egl nine homolog 3 (C. elegans)	EGLN3
1563487_at	2.18		
240948_at	2.18		
1561578_s_at	2.18	mitochondrial carrier triple repeat 6	MCART6
44790_s_at	2.18	chromosome 13 open reading frame 18	C13orf18
1563498_s_at	2.18	solute carrier family 25, member 45	SLC25A45

216062_at	2.18		
218999_at	2.18	transmembrane protein 140	TMEM140
230046_at	2.18		
1552480_s_at	2.17	protein tyrosine phosphatase, receptor type, C	PTPRC
242310_at	2.17		
202748_at	2.17	guanylate binding protein 2, interferon-inducible	GBP2
212729_at	2.17	discs, large homolog 3 (neuroendocrine-dlg, Drosophila)	DLG3
232601_at	2.17		
236499_at	2.17	chromosome 1 open reading frame 86	C1orf86
1556105_at	2.17		
1557585_at	2.17	ATPase, H+ transporting, lysosomal 50/57kDa, V1 subunit H	ATP6V1H
213261_at	2.17		
209210_s_at	2.16	pleckstrin homology domain containing, family C (with FERM domain) member 1	PLEKHC1
205146_x_at	2.16	amyloid beta (A4) precursor protein-binding, family A, member 3 (X11-like 2)	APBA3
1562098_at	2.16		
225540_at	2.16	microtubule-associated protein 2	MAP2
207608_x_at	2.16	cytochrome P450, family 1, subfamily A, polypeptide 2	CYP1A2
1558105_a_at	2.16		
1559406_at	2.16	ankyrin repeat domain 18A	ANKRD18A
243959_at	2.16	muskelin 1, intracellular mediator containing kelch motifs	MKLN1
242598_at	2.16	src kinase associated phosphoprotein 2	SKAP2
1563298_at	2.16		
216816_at	2.16		
1562934_at	2.15		
212274_at	2.15	lipin 1	LPIN1
239493_at	2.15	ribosomal protein L7	RPL7
205783_at	2.15	kallikrein-related peptidase 13	KLK13
1553894_at	2.15	coiled-coil domain containing 122	CCDC122
204621_s_at	2.15	nuclear receptor subfamily 4, group A, member 2	NR4A2
216004_s_at	2.15	crystallin, alpha A#PBX/knotted 1 homeobox 1#U2 small nuclear RNA auxiliary factor 1	CRYAA#PKNOX1#U2AF1
208448_x_at	2.15	interferon, alpha 16	IFNA16
227923_at	2.14	SH3 and multiple ankyrin repeat domains 3	SHANK3
1556649_at	2.14		
1558139_at	2.14		
229739_s_at	2.14	family with sequence similarity 116, member B	FAM116B
228056_s_at	2.14	napsin B aspartic peptidase pseudogene	NAPSB
243894_at	2.14	solute carrier family 41, member 2	SLC41A2
1563941_at	2.13		
229854_at	2.13	obscurin, cytoskeletal calmodulin and titin-interacting RhoGEF	OBSCN
236680_at	2.13		
202700_s_at	2.13	transmembrane protein 63A	TMEM63A
1557292_a_at	2.13	mucolipin 3	MCOLN3
204205_at	2.13	apolipoprotein B mRNA editing enzyme, catalytic polypeptide-like 3G	APOBEC3G
1564654_at	2.13		

221961_at	2.13	chloride channel 7	CLCN7
210163_at	2.13	chemokine (C-X-C motif) ligand 11	CXCL11
229461_x_at	2.13	neuronal growth regulator 1	NEGR1
228547_at	2.13	neurexin 1	NRXN1
1569551_at	2.13		
214748_at	2.12		
235046_at	2.12		
238414_at	2.12		
239255_at	2.12		
224042_at	2.12	ureidopropionase, beta	UPB1
223890_at	2.12		
215441_at	2.12		
241319_at	2.12	exocyst complex component 6	EXOC6
221656_s_at	2.12	Rho guanine nucleotide exchange factor (GEF) 10-like	ARHGEF10L
211028_s_at	2.12	ketohexokinase (fructokinase)	KHK
232679_at	2.12		
211148_s_at	2.12	angiopoietin 2	ANGPT2
1555938_x_at	2.12	vimentin	VIM
232150_at	2.12		
1558779_at	2.11	H2A histone family, member Y	H2AFY
1554253_a_at	2.11	LAG1 homolog, ceramide synthase 3 (S. cerevisiae)	LASS3
217569_x_at	2.11	calcium/calmodulin-dependent protein kinase (CaM kinase) II delta	CAMK2D
222108_at	2.11	adhesion molecule with Ig-like domain 2	AMIGO2
211095_at	2.11	neurofibromin 1 (neurofibromatosis, von Recklinghausen disease, Watson disease)	NF1
214645_at	2.11		
213689_x_at	2.11	ribosomal protein L5	RPL5
215074_at	2.11	myosin IB	MYO1B
239256_at	2.11		
1566459_at	2.11		
238032_at	2.11		
34408_at	2.11	reticulon 2	RTN2
242292_at	2.11		
204069_at	2.11	Meis1, myeloid ecotropic viral integration site 1 homolog (mouse)	MEIS1
33323_r_at	2.11	stratifin	SFN
44673_at	2.11	sialic acid binding Ig-like lectin 1, sialoadhesin	SIGLEC1
239593_at	2.11		
208331_at	2.10	basic charge, Y-linked, 2	BPY2
1552652_at	2.10	Hermansky-Pudlak syndrome 4	HPS4
214772_at	2.10	chromosome 11 open reading frame 41	C11orf41
1554335_at	2.10	pleckstrin homology, Sec7 and coiled-coil domains 4	PSCD4
221614_s_at	2.10	rabphilin 3A-like (without C2 domains)	RPH3AL
234046_at	2.10		
227599_at	2.10	chromosome 3 open reading frame 59	C3orf59
243676_at	2.10	chromosome 20 open reading frame 11	C20orf11
225415_at	2.10	deltex 3-like (Drosophila)	DTX3L
208269_s_at	2.10	ADAM metallopeptidase domain 28	ADAM28
239870_at	2.10	spermatogenesis associated, serine-rich 1	SPATS1
201243_s_at	2.10	ATPase, Na+/K+ transporting, beta 1 polypeptide	ATP1B1
225056_at	2.10	signal-induced proliferation-associated 1 like 2	SIPA1L2

237586_at	2.10	epidermal growth factor receptor pathway substrate 15	EPS15
206181_at	2.10	signaling lymphocytic activation molecule family member 1	SLAMF1
239913_at	2.10	solute carrier family 10 (sodium/bile acid cotransporter family), member 4	SLC10A4
212254_s_at	2.09	dystonin	DST
238076_at	2.09		
206144_at	2.09	membrane associated guanylate kinase, WW and PDZ domain containing 1	MAGI1
210832_x_at	2.09	prostaglandin E receptor 3 (subtype EP3)	PTGER3
210623_at	2.09		
223593_at	2.09	aminoadipate aminotransferase	AADAT
233362_at	2.09	zinc finger protein 341	ZNF341
235636_at	2.09		
222259_s_at	2.09	RAE1 RNA export 1 homolog (S. pombe)#SPO11 meiotic protein covalently bound to DSB homolog (S. cerevisiae)	RAE1#SPO11
242961_x_at	2.09	DEAD (Asp-Glu-Ala-Asp) box polypeptide 58	DDX58
216769_x_at	2.09		
240906_at	2.08	mitochondrial ribosomal protein S36	MRPS36
236502_at	2.08		
229092_at	2.08		
1556279_at	2.08		
206648_at	2.08	zinc finger protein 571	ZNF571
236982_at	2.08		
223924_at	2.08	tetratricopeptide repeat domain 25	TTC25
201508_at	2.08	insulin-like growth factor binding protein 4	IGFBP4
211565_at	2.08	SH3-domain GRB2-like 3	SH3GL3
239109_at	2.08		
234132_at	2.08		
1554866_at	2.08	transmembrane protein 135	TMEM135
1556809_a_at	2.08		
1569225_a_at	2.07	sex comb on midleg-like 4 (Drosophila)	SCML4
243476_at	2.07		
1559856_s_at	2.07	myeloid/lymphoid or mixed-lineage leukemia (trithorax homolog, Drosophila)	MLL
205767_at	2.07	epiregulin	EREG
225931_s_at	2.07	ring finger protein 213	RNF213
226791_at	2.07	kinesin family member C2	KIFC2
221843_s_at	2.07	KIAA1609	KIAA1609
231698_at	2.07	UDP-glucose pyrophosphorylase 2	UGP2
208782_at	2.07	follistatin-like 1	FSTL1
243351_at	2.07		
236786_at	2.07		
208012_x_at	2.07	SP110 nuclear body protein	SP110
1556090_at	2.07		
1562527_at	2.06		
240787_at	2.06		
218815_s_at	2.06	transmembrane protein 51	TMEM51
1556078_at	2.06		
241395_at	2.06	nitrilase 1	NIT1

205170_at	2.06	signal transducer and activator of transcription 2, 113kDa	STAT2
228582_x_at	2.06	metastasis associated lung adenocarcinoma transcript 1 (non-coding RNA)	MALAT1
209387_s_at	2.06	transmembrane 4 L six family member 1	TM4SF1
1569856_at	2.06	tripeptidyl peptidase II	TPP2
1560264_at	2.05		
203736_s_at	2.05	PTPRF interacting protein, binding protein 1 (liprin beta 1)	PPFIBP1
244502_at	2.05		
1553296_at	2.05	G protein-coupled receptor 128	GPR128
237627_at	2.05		
232086_at	2.05		
1559759_at	2.05	kinesin family member C3	KIFC3
220150_s_at	2.05	chromosome 6 open reading frame 60	C6orf60
216893_s_at	2.05	collagen, type IV, alpha 3 (Goodpasture antigen)	COL4A3
232300_at	2.05		
1569344_a_at	2.05		
215856_at	2.05	CD33 molecule-like 3	CD33L3
244204_at	2.05	proline rich 3	PRR3
240354_at	2.05	chromosome 12 open reading frame 54	C12orf54
1563138_at	2.05		
244637_at	2.05		
1564285_at	2.05	HFM1, ATP-dependent DNA helicase homolog (S. cerevisiae)	HFM1
244612_at	2.04		
211123_at	2.04	solute carrier family 5 (sodium iodide symporter), member 5	SLC5A5
243525_at	2.04		
233120_at	2.04		
218501_at	2.03	Rho guanine nucleotide exchange factor (GEF) 3	ARHGEF3
205253_at	2.03	pre-B-cell leukemia homeobox 1	PBX1
237982_at	2.03		
202728_s_at	2.03	latent transforming growth factor beta binding protein 1	LTBP1
234994_at	2.03	KIAA1913	KIAA1913
1562823_at	2.03		
217619_x_at	2.03		
243625_at	2.03	cAMP responsive element binding protein 1	CREB1
1561915_at	2.03		
228557_at	2.03	l(3)mbt-like 4 (Drosophila)	L3MBTL4
236103_at	2.02		
1553335_x_at	2.02		
229857_s_at	2.02		
241635_at	2.02		
225912_at	2.02	tumor protein p53 inducible nuclear protein 1	TP53INP1
204999_s_at	2.02	activating transcription factor 5	ATF5
235172_at	2.02		
241015_at	2.02		
226931_at	2.02	transmembrane and tetratricopeptide repeat containing 1	TMTC1
1553561_at	2.02	taste receptor, type 2, member 50	TAS2R50
212386_at	2.02		

204443_at	2.02	arylsulfatase A	ARSA
201579_at	2.01	FAT tumor suppressor homolog 1 (Drosophila)	FAT
214002_at	2.01	myosin, light chain 6, alkali, smooth muscle and non-muscle	MYL6
218010_x_at	2.01	chromosome 20 open reading frame 149	C20orf149
1558310_s_at	2.01		
227995_at	2.01		
217432_s_at	2.01	iduronate 2-sulfatase (Hunter syndrome)	IDS
221304_at	2.01	UDP glucuronosyltransferase 1 family, polypeptide A8	UGT1A8
231050_at	2.01	HRAS-like suppressor family, member 5	HRASLS5
1558603_at	2.01	plasminogen-like B2	PLGLB2
229605_at	2.01		
200999_s_at	2.01	cytoskeleton-associated protein 4	CKAP4
220028_at	2.01	activin A receptor, type IIB	ACVR2B
228154_at	2.01	chromosome 19 open reading frame 44	C19orf44
240958_at	2.01	unc-5 homolog C (C. elegans)	UNC5C
215094_at	2.01	coiled-coil domain containing 52	CCDC52
236270_at	2.01	nuclear factor of activated T-cells, cytoplasmic, calcineurin-dependent 4	NFATC4
200887_s_at	2.00	signal transducer and activator of transcription 1, 91kDa	STAT1
202807_s_at	2.00	target of myb1 (chicken)	TOM1
1569596_at	2.00		
243184_at	2.00	tight junction protein 1 (zona occludens 1)	TJP1
203826_s_at	2.00	phosphatidylinositol transfer protein, membrane-associated 1	PITPNM1
232212_at	0.50	pleckstrin homology domain containing, family A (phosphoinositide binding specific) member 8	PLEKHA8
1557534_at	0.50		
215220_s_at	0.49	translocated promoter region (to activated MET oncogene)	TPR
219688_at	0.49	Bardet-Biedl syndrome 7	BBS7
238795_at	0.49	chromosome 10 open reading frame 18	C10orf18
219522_at	0.48	four jointed box 1 (Drosophila)	FJX1
204446_s_at	0.48	arachidonate 5-lipoxygenase	ALOX5
222671_s_at	0.48	chromosome 1 open reading frame 142	C1orf142
239801_at	0.47		
214146_s_at	0.47	pro-platelet basic protein (chemokine (C-X-C motif) ligand 7)	PPBP
207867_at	0.47	paired box gene 4	PAX4
226218_at	0.47		
206834_at	0.47	hemoglobin, delta	HBD
210325_at	0.46	CD1a molecule	CD1A
237802_at	0.46	XK, Kell blood group complex subunit-related family, member 4	XKR4
238336_s_at	0.46		
235178_x_at	0.46	establishment of cohesion 1 homolog 2 (S. cerevisiae)	ESCO2
1557120_at	0.45	eukaryotic translation elongation factor 1 alpha 1	EEF1A1
1553723_at	0.45	G protein-coupled receptor 97	GPR97
223828_s_at	0.45	lectin, galactoside-binding, soluble, 12 (galectin 12)	LGALS12
225803_at	0.45	F-box protein 32	FBXO32

213515_x_at	0.44	hemoglobin, gamma G	HBG2
214732_at	0.44	Sp1 transcription factor	SP1
1552969_a_at	0.44	zinc finger, MYM-type 6	ZMYM6
204073_s_at	0.42	chromosome 11 open reading frame 9	C11orf9
232796_at	0.42		
238448_at	0.42	mitochondrial ribosomal protein L19	MRPL19
214139_at	0.41	AT rich interactive domain 4B (RBP1-like)	ARID4B
226777_at	0.41		
212667_at	0.41	secreted protein, acidic, cysteine-rich (osteonectin)	SPARC
216931_at	0.40		
238432_at	0.40		
235909_at	0.39		
230535_s_at	0.38		
210828_s_at	0.37	aryl hydrocarbon receptor nuclear translocator	ARNT
229622_at	0.37		
244239_at	0.36		
210945_at	0.35	collagen, type IV, alpha 6	COL4A6
205224_at	0.35	surfeit 2	SURF2
220811_at	0.35	proteoglycan 3	PRG3
231834_at	0.34	proline rich 8	PRR8
206395_at	0.33	diacylglycerol kinase, gamma 90kDa	DGKG
223690_at	0.32	latent transforming growth factor beta binding protein 2	LTBP2
207800_at	0.31	A kinase (PRKA) anchor protein 5	AKAP5
1556972_at	0.31		
241623_at	0.31	protein tyrosine phosphatase, non-receptor type 2	PTPN2
211096_at	0.30	pre-B-cell leukemia homeobox 2	PBX2
210997_at	0.29	hepatocyte growth factor (hepapoietin A; scatter factor)	HGF
231064_s_at	0.28	nucleoporin 50kDa	NUP50
220593_s_at	0.28	coiled-coil domain containing 40	CCDC40
215195_at	0.28	protein kinase C, alpha	PRKCA
234748_x_at	0.28		
231037_at	0.27		
1562789_at	0.27	zinc finger protein 229	ZNF229
231609_at	0.26	chromosome 10 open reading frame 82	C10orf82
1561651_s_at	0.25	SCL/TAL1 interrupting locus	STIL
1565939_at	0.25	chromosome 5 open reading frame 22	C5orf22
1564371_a_at	0.24	cancer susceptibility candidate 2	CASC2
216604_s_at	0.24	solute carrier family 7 (cationic amino acid transporter, y+ system), member 8	SLC7A8
1552604_at	0.23	chromosome 21 open reading frame 74	C21orf74
227744_s_at	0.23	heterogeneous nuclear ribonucleoprotein D (AU-rich element RNA binding protein 1, 37kDa)	HNRPD
233460_at	0.21		
238605_at	0.21		
234500_at	0.21		
204032_at	0.20	breast cancer anti-estrogen resistance 3	BCAR3
232943_at	0.20		
1555071_at	0.19	tolloid-like 1	TLL1
231877_at	0.19	RNA (guanine-9-) methyltransferase domain containing 2	RG9MTD2
243685_at	0.19		

234621_at	0.18		
232424_at	0.18	PR domain containing 16	PRDM16
1555676_s_at	0.17	GS homeobox 1	GSH1
211298_s_at	0.17	albumin	ALB
222847_s_at	0.17	egl nine homolog 3 (C. elegans)	EGLN3
1552912_a_at	0.17	interleukin 23 receptor	IL23R
238674_at	0.17		
1564075_a_at	0.17		
1565840_at	0.17	PR domain containing 4	PRDM4
242697_at	0.17	zinc finger protein 540	ZNF540
216716_at	0.16	ABO blood group (transferase A, alpha 1-3-N-acetylgalactosaminyltransferase; transferase B, alpha 1-	ABO
211517_s_at	0.16	interleukin 5 receptor, alpha	IL5RA
240506_at	0.15		
213228_at	0.15	phosphodiesterase 8B	PDE8B
223667_at	0.15	FK506 binding protein 7	FKBP7
206251_s_at	0.15	arginine vasopressin receptor 1A	AVPR1A
1559488_at	0.15		
237566_at	0.14		
225987_at	0.14	STEAP family member 4	STEAP4
244102_at	0.13		
219385_at	0.13	SLAM family member 8	SLAMF8
207739_s_at	0.12	G antigen 2	GAGE2
222324_at	0.12		
205557_at	0.11	bactericidal/permeability-increasing protein	BPI
242085_at	0.11	chromosome 2 open reading frame 18	C2orf18
216519_s_at	0.11	proline synthetase co-transcribed homolog (bacterial)	PROSC
221236_s_at	0.11	stathmin-like 4	STMN4
236868_at	0.11		
1563022_at	0.11		
1555340_x_at	0.10	RAP1A, member of RAS oncogene family	RAP1A
1562116_at	0.10		
227827_at	0.10		
1555339_at	0.09	RAP1A, member of RAS oncogene family	RAP1A
220187_at	0.09	STEAP family member 4	STEAP4
237069_s_at	0.08	transient receptor potential cation channel, subfamily M, member 1	TRPM1
230869_at	0.07		
1568919_at	0.07		
239818_x_at	0.06	tribbles homolog 1 (Drosophila)	TRIB1
1559653_at	0.06		
229912_at	0.05	sidekick homolog 1 (chicken)	SDK1
1566969_at	0.05		
206647_at	0.04	hemoglobin, zeta	HBZ
1559665_at	0.04		
243460_at	0.03		
