# Supplementary material for: In Vitro Transformation of Primary Human CD34+ Cells by AML Fusion Oncogenes: Early Gene Expression Profiling Reveals Possible Drug Target in AML
Source: PLoS One. 2010 Aug 27;5(8):e12464. doi: 10.1371/journal.pone.0012464 (PMC2929205; doi:10.1371/journal.pone.0012464)
Supplement: Table S24 — Genes deregulated by NUP98-HOXA9 8 days after transduction. Primary human CD34+ cells were retrovirally transduced with either control MSCV-IRES-GFP vector or vector expressing NUP98-HOXA9 and sorted for GFP positivity. Total RNA was extracted 8 days after transduction and subjected to microarray analysis. Microarray data were analyzed by SAM as described in Materials and Methods. Significantly deregulated genes are listed and the false discovery rate (FDR) is shown. (0.25 MB PDF) [file pone.0012464.s024.pdf]

Table S24. Genes deregulated by NUP98-HOXA9 at 8 d detected by SAM FDR = 5.29%

Probe set ID	Fold Change	Gene Name	Gene Symbol
204439_at	32.60	interferon-induced protein 44-like	IFI44L
224296_x_at	26.38		
237058_x_at	22.54	solute carrier family 6 (neurotransmitter transporter, GABA), member 13	SLC6A13
206367_at	21.47	renin	REN
219463_at	18.15	chromosome 20 open reading frame 103	C20orf103
237261_at	16.94		
1554524_a_at	15.89	olfactomedin 3	OLFM3
1559287_at	15.01		
229999_at	14.25		
229638_at	13.48	iroquois homeobox protein 3	IRX3
211597_s_at	13.31		
242513_x_at	12.72	KIAA2018	KIAA2018
1556499_s_at	12.36	collagen, type I, alpha 1	COL1A1
226103_at	12.31	nexilin (F actin binding protein)	NEXN
243806_at	11.87		
202411_at	11.84	interferon, alpha-inducible protein 27	IFI27
223316_at	11.67	coiled-coil domain containing 3	CCDC3
217276_x_at	11.56	serine hydrolase-like 2	SERHL2
230671_at	11.49		
202086_at	11.33	myxovirus (influenza virus) resistance 1, interferon-inducible protein p78 (mouse)	MX1
1563033_x_at	11.30		
229450_at	11.11	interferon-induced protein with tetratricopeptide repeats 3	IFIT3
214457_at	10.93	homeobox A2	HOXA2
209757_s_at	10.37	v-myc myelocytomatosis viral related oncogene, neuroblastoma derived (avian)	MYCN
208292_at	10.02	bone morphogenetic protein 10	BMP10
1553613_s_at	9.83	forkhead box C1	FOXC1
239791_at	9.71		
203153_at	9.70	interferon-induced protein with tetratricopeptide repeats 1	IFIT1
214023_x_at	9.59	tubulin, beta 2B	TUBB2B
224379_at	9.50	ferritin, heavy polypeptide-like 17	FTHL17
204415_at	9.49	interferon, alpha-inducible protein 6	IFI6
242625_at	9.42	radical S-adenosyl methionine domain containing 2	RSAD2
212097_at	9.33	caveolin 1, caveolae protein, 22kDa	CAV1
231947_at	9.26	myc target 1	MYCT1
200923_at	9.18	lectin, galactoside-binding, soluble, 3 binding protein	LGALS3BP
235904_at	9.16	UDP glycosyltransferase 3 family, polypeptide A1	UGT3A1
231382_at	9.12	fibroblast growth factor 18	FGF18
204747_at	9.03	interferon-induced protein with tetratricopeptide repeats 3	IFIT3
1570076_at	8.93		
229802_at	8.88		
236892_s_at	8.85		
244885_at	8.78		

1553808_a_at	8.77	NK2 transcription factor related, locus 3 (Drosophila)	NKX2-3
229859_at	8.40		
209487_at	8.30	RNA binding protein with multiple splicing	RBPMS
243530_at	8.26	cleavage and polyadenylation specific factor 6, 68kDa	CPSF6
224021_at	8.17	retinitis pigmentosa 1 (autosomal dominant)	RP1
1563329_s_at	7.98		
206085_s_at	7.92	cystathionase (cystathionine gamma-lyase)	CTH
1566115_at	7.85		
240681_at	7.76		
1560745_at	7.76		
210237_at	7.69	artemin	ARTN
217502_at	7.62	interferon-induced protein with tetratricopeptide repeats 2	IFIT2
239422_at	7.62	glypican 2 (cerebroglycan)	GPC2
1556593_s_at	7.57		
242964_at	7.56		
219211_at	7.44	ubiquitin specific peptidase 18	USP18
240143_at	7.34	ADAM metallopeptidase domain 23	ADAM23
224202_at	7.25	suppressor of fused homolog (Drosophila)	SUFU
201187_s_at	7.24	inositol 1,4,5-triphosphate receptor, type 3	ITPR3
227123_at	7.15	RAB3B, member RAS oncogene family	RAB3B
220280_s_at	7.15	ankyrin repeat and MYND domain containing 1	ANKMY1
223709_s_at	7.12	wingless-type MMTV integration site family, member 10A	WNT10A
232738_at	7.05		
231535_x_at	7.03	ropporin, rhophilin associated protein 1	ROPN1
240523_at	7.00		
202145_at	6.85	lymphocyte antigen 6 complex, locus E	LY6E
240395_at	6.84		
213797_at	6.84	radical S-adenosyl methionine domain containing 2	RSAD2
203878_s_at	6.82	matrix metallopeptidase 11 (stromelysin 3)	MMP11
214453_s_at	6.73	interferon-induced protein 44	IFI44
214212_x_at	6.71	pleckstrin homology domain containing, family C (with FERM domain) member 1	PLEKHC1
222869_s_at	6.66	elaC homolog 1 (E. coli)	ELAC1
1559105_at	6.66		
228371_s_at	6.66	chromosome 16 open reading frame 72	C16orf72
206310_at	6.64	serine peptidase inhibitor, Kazal type 2 (acrosin-trypsin inhibitor)	SPINK2
1569846_at	6.64		
210551_s_at	6.63	acetylserotonin O-methyltransferase	ASMT
208613_s_at	6.63	filamin B, beta (actin binding protein 278)	FLNB
1556578_a_at	6.61		
210029_at	6.60	indoleamine-pyrrole 2,3 dioxygenase	INDO
1561658_at	6.59		
1559458_at	6.58		
207199_at	6.58	telomerase reverse transcriptase	TERT
214961_at	6.50	KIAA0774	KIAA0774
235578_at	6.45	ATP-binding cassette, sub-family C (CFTR/MRP), member 9	ABCC9
243541_at	6.39	interleukin 31 receptor A	IL31RA
218400_at	6.32	2'-5'-oligoadenylate synthetase 3, 100kDa	OAS3
218758_s_at	6.25		

1570412_at	6.22		
205483_s_at	6.22	ISG15 ubiquitin-like modifier	ISG15
1552430_at	6.21	WD repeat domain 17	WDR17
222950_at	6.19	NIPA-like domain containing 2	NPAL2
203394_s_at	6.17	hairy and enhancer of split 1, (Drosophila)	HES1
213844_at	6.13	homeobox A5	HOXA5
1564438_at	6.05		
235065_at	6.03		
204972_at	6.02	2'-5'-oligoadenylate synthetase 2, 69/71kDa	OAS2
1569688_at	5.98	flavin containing monooxygenase 5	FMO5
214038_at	5.91	chemokine (C-C motif) ligand 8	CCL8
211165_x_at	5.79	EPH receptor B2	EPHB2
239492_at	5.77	SEC14-like 4 (S. cerevisiae)	SEC14L4
215234_at	5.71		
238498_at	5.67		
205609_at	5.61	angiopoietin 1	ANGPT1
1554158_at	5.60	zinc finger, MYND domain containing 11	ZMYND11
241362_at	5.60	chromosome 20 open reading frame 117	C20orf117
235276_at	5.56	epithelial stromal interaction 1 (breast)	EPSTI1
230755_at	5.55	rhomboid, veinlet-like 3 (Drosophila)	RHBDL3
214078_at	5.45		
242244_at	5.45		
231438_x_at	5.44		
1561460_at	5.44		
231304_at	5.42	protein phosphatase 3 (formerly 2B), regulatory subunit B, beta isoform	PPP3R2
210808_s_at	5.40	NADPH oxidase 1	NOX1
235608_at	5.38		
207710_at	5.29	late cornified envelope 2B	LCE2B
231174_s_at	5.23		
228038_at	5.16	SRY (sex determining region Y)-box 2	SOX2
235955_at	5.16	MARVEL domain containing 2	MARVELD2
237083_at	5.14		
210362_x_at	5.13	promyelocytic leukemia	PML
235521_at	5.08	homeobox A3	HOXA3
1556633_at	5.07	chromosome 1 open reading frame 204	C1orf204
1559737_at	5.07		
233622_x_at	5.04		
233857_s_at	5.04	ankyrin repeat and SOCS box-containing 2	ASB2
213953_at	5.03	keratin 20	KRT20
1553604_at	5.00	ATP-binding cassette, sub-family A (ABC1), member 13	ABCA13
1554633_a_at	4.99	myelin transcription factor 1-like	MYT1L
234569_at	4.98		
226014_at	4.89	eukaryotic translation initiation factor 3, subunit 5 epsilon, 47kDa	EIF3S5
206382_s_at	4.89	brain-derived neurotrophic factor	BDNF
241198_s_at	4.89	chromosome 11 open reading frame 70	C11orf70
232656_at	4.88		
209859_at	4.85	tripartite motif-containing 9	TRIM9
240401_at	4.85	apoptosis antagonizing transcription factor	AATF
244147_at	4.85		
240921_at	4.83		

211499_s_at	4.82	mitogen-activated protein kinase 11	MAPK11
1570579_at	4.82		
1560219_at	4.82		
233891_at	4.80		
1566219_at	4.78		
204292_x_at	4.78	serine/threonine kinase 11	STK11
206987_x_at	4.78	fibroblast growth factor 18	FGF18
232305_at	4.71	3-hydroxymethyl-3-methylglutaryl-Coenzyme A lyase-like 1	HMGCLL1
1570349_at	4.70		
212364_at	4.69	myosin IB	MYO1B
214059_at	4.69	interferon-induced protein 44	IFI44
214478_at	4.66	secreted phosphoprotein 2, 24kDa	SPP2
1556239_a_at	4.65		
1553474_at	4.63	ankyrin repeat domain 21	ANKRD21
206292_s_at	4.61	sulfotransferase family, cytosolic, 2A, dehydroepiandrosterone (DHEA)-preferring, member 1	SULT2A1
233318_at	4.56		
1568987_at	4.56		
1562368_at	4.56		
1561255_at	4.54		
226757_at	4.53	interferon-induced protein with tetratricopeptide repeats 2	IFIT2
1553574_at	4.51		
1569023_a_at	4.47		
211132_at	4.47	integrator complex subunit 3	INTS3
1566427_at	4.47		
212489_at	4.45	collagen, type V, alpha 1	COL5A1
210163_at	4.42	chemokine (C-X-C motif) ligand 11	CXCL11
213964_x_at	4.42		
1554319_at	4.40	ribosomal protein S6 kinase, 90kDa, polypeptide 5	RPS6KA5
207836_s_at	4.40	RNA binding protein with multiple splicing	RBPMS
1567078_x_at	4.39		
205989_s_at	4.39	myelin oligodendrocyte glycoprotein	MOG
224646_x_at	4.38	H19, imprinted maternally expressed untranslated mRNA	H19
1565633_at	4.37		
235586_at	4.37		
215132_at	4.37		
1564598_a_at	4.33		
241086_at	4.31		
229914_at	4.30		
241684_at	4.30		
235753_at	4.29	homeobox A7	HOXA7
223652_at	4.29	arsenic (+3 oxidation state) methyltransferase	AS3MT
223966_at	4.28		
243386_at	4.26		
233349_at	4.21	tousled-like kinase 2	TLK2
1557044_at	4.20		
238336_s_at	4.17		
204533_at	4.15	chemokine (C-X-C motif) ligand 10	CXCL10
203962_s_at	4.11	nebulette	NEBL

228617_at	4.10		
235504_at	4.10	gremlin 2, cysteine knot superfamily, homolog (Xenopus laevis)	GREM2
243947_s_at	4.09		
218986_s_at	4.07		
244259_s_at	4.06		
240235_at	4.06	chromosome 10 open reading frame 62	C10orf62
224074_at	4.05	visual system homeobox 1 homolog, CHX10-like (zebrafish)	VSX1
206760_s_at	4.04	Fc fragment of IgE, low affinity II, receptor for (CD23)	FCER2
234871_at	4.02	G protein-coupled receptor 98	GPR98
1561966_at	4.01		
1560525_at	4.01		
233170_at	4.00		
203903_s_at	3.98	hephaestin	HEPH
225739_at	3.95	RAB11 family interacting protein 4 (class II)	RAB11FIP4
210164_at	3.93	granzyme B (granzyme 2, cytotoxic T-lymphocyte-associated serine esterase 1)	GZMB
243538_at	3.90		
217415_at	3.90	polymerase (RNA) II (DNA directed) polypeptide A, 220kDa	POLR2A
218630_at	3.87	Meckel syndrome, type 1	MKS1
219604_s_at	3.85	zinc finger protein 3	ZNF3
239165_at	3.84		
203998_s_at	3.84	synaptotagmin I	SYT1
1562215_at	3.84		
228708_at	3.83		
1559975_at	3.82	B-cell translocation gene 1, anti-proliferative	BTG1
1563754_at	3.82	glutamate receptor, ionotropic, kainate 2	GRIK2
220205_at	3.82	transmembrane phosphatase with tensin homology	TPTE
237168_at	3.81		
1553776_at	3.79	ubiquitin-conjugating enzyme E2U (putative)	UBE2U
203395_s_at	3.78	hairy and enhancer of split 1, (Drosophila)	HES1
238049_at	3.75		
239576_at	3.74	mitochondrial tumor suppressor 1	MTUS1
229309_at	3.73		
207911_s_at	3.70	transglutaminase 5	TGM5
220021_at	3.69	transmembrane channel-like 7	TMC7
233288_at	3.69	ataxia telangiectasia and Rad3 related	ATR
230218_at	3.68		
206133_at	3.64		
219606_at	3.64	PHD finger protein 20-like 1	PHF20L1
212013_at	3.64	peroxidasin homolog (Drosophila)	PXDN
204994_at	3.64	myxovirus (influenza virus) resistance 2 (mouse)	MX2
236284_at	3.64	KIAA0146	KIAA0146
205734_s_at	3.62	AF4/FMR2 family, member 3	AFF3
1561468_at	3.62		
207840_at	3.59	CD160 molecule	CD160

234848_at	3.58	T cell receptor alpha locus#T cell receptor alpha variable 6#T cell receptor alpha variable 16#T cell receptor alpha variable 15#T cell receptor alpha variable 14/delta variable 4#T cell receptor alpha variable 13-2#T cell receptor alpha variable 13-1#T cell receptor alpha variable 12-3#T cell receptor alpha variable 12-2#T cell receptor alpha variable 12-1#T cell receptor alpha variable 11#T cell receptor alpha variable 10#T cell receptor alpha variable 9-2#T cell receptor alpha variable 9-1#T cell receptor alpha variable 8-6#T cell receptor alpha variable 8-5#T cell receptor alpha variable 8-4#T cell receptor alpha variable 8-3#T cell receptor alpha variable 8-2#T cell receptor alpha variable 8-1#T cell receptor alpha variable 7#T cell receptor alpha variable 5#null	TRA@#TRAV6#TRAV16#TRAV15#TRAV14DV4#TRAV13-2#TRAV13-1#TRAV12-3#TRAV12-2#TRAV12-1#TRAV11#TRAV10#TRAV9-2#TRAV9-1#TRAV8-6#TRAV8-5#TRAV8-4#TRAV8-3#TRAV8-2#TRAV8-1#TRAV7#TRAV5#null
1555516_at	3.57	forkhead box P2	FOXP2
237634_at	3.55		
1559889_at	3.55		
1566150_at	3.55	calmodulin-like 4	CALML4
234127_at	3.55		
241457_at	3.54		
1554874_at	3.54	microphthalmia-associated transcription factor	MITF
227225_at	3.53	zinc finger protein 503	ZNF503
1564736_a_at	3.52	caspase 12	CASP12
221062_at	3.52	heparan sulfate (glucosamine) 3-O-sulfotransferase 3B1	HS3ST3B1
232242_at	3.52		
203421_at	3.52	tumor protein p53 inducible protein 11	TP53I11
243789_at	3.51		
239744_at	3.51		
1565339_at	3.51	dynein, axonemal, heavy chain 10	DNAH10
228607_at	3.50	2'-5'-oligoadenylate synthetase 2, 69/71kDa	OAS2
220803_at	3.50	STAM binding protein-like 1	STAMBPL1
1569399_at	3.49		
203595_s_at	3.48		
238203_at	3.45		
217261_at	3.45	testis-specific transcript, Y-linked 2	TTTY2
209498_at	3.44	carcinoembryonic antigen-related cell adhesion molecule 1 (biliary glycoprotein)	CEACAM1
216719_s_at	3.44	RAB11 family interacting protein 4 (class II)	RAB11FIP4
221204_s_at	3.43	cartilage acidic protein 1	CRTAC1
236714_at	3.43		
240268_at	3.43		
236698_at	3.43		
1568449_at	3.42		
1559275_x_at	3.42		
205660_at	3.40	2'-5'-oligoadenylate synthetase-like	OASL
234919_s_at	3.39	syntrophin, gamma 1	SNTG1
203439_s_at	3.39	stanniocalcin 2	STC2

232368_at	3.38	BET3 like (S. cerevisiae)	BET3L
201601_x_at	3.38	interferon induced transmembrane protein 1 (9-27)	IFITM1
222947_at	3.36	zinc finger protein 224	ZNF224
239979_at	3.33	epithelial stromal interaction 1 (breast)	EPSTI1
216249_at	3.33	Pvt1 oncogene homolog, MYC activator (mouse)	PVT1
1562226_at	3.33		
208748_s_at	3.33	flotillin 1	FLOT1
1563797_at	3.33		
221594_at	3.32		
243868_at	3.31		
237493_at	3.31	interleukin 22 receptor, alpha 2	IL22RA2
242301_at	3.31	cerebellin 2 precursor	CBLN2
212732_at	3.29	maternally expressed 3	MEG3
1567703_at	3.29		
230220_at	3.29		
235144_at	3.29		
222241_at	3.26	family with sequence similarity 125, member B	FAM125B
236365_at	3.25	alpha-methylacyl-CoA racemase	AMACR
233872_x_at	3.24	Rho GTPase activating protein 5	ARHGAP5
234393_at	3.23	histone deacetylase 9	HDAC9
221232_s_at	3.23	ankyrin repeat domain 2 (stretch responsive muscle)	ANKRD2
233727_at	3.23		
219863_at	3.22	hect domain and RLD 5	HERC5
1554526_at	3.22	olfactomedin 3	OLFM3
208186_s_at	3.22	lipase, hormone-sensitive	LIPE
228877_at	3.22	erythropoietin receptor	EPOR
244639_at	3.22	transmembrane BAX inhibitor motif containing 4	TMBIM4
233146_at	3.22	kinase non-catalytic C-lobe domain (KIND) containing 1	KNDC1
216058_s_at	3.21	cytochrome P450, family 2, subfamily C, polypeptide 19	CYP2C19
228230_at	3.21	eukaryotic translation elongation factor 1 alpha 2#potassium voltage-gated channel, KQT-like subfamily, member 2#potassium voltage-gated channel, KQT-like subfamily, member 2#PTK6 protein tyrosine kinase 6#src-related kinase lacking C-terminal regulatory tyrosine and N-terminal myristylation sites#chromosome 20 open reading frame 195#chromosome 20 open reading frame 149#null	EEF1A2#KCNQ2#KCNQ2#PTK6#SRMS#C20orf195#C20orf149#null
202869_at	3.21	2',5'-oligoadenylate synthetase 1, 40/46kDa	OAS1
215768_at	3.20		
232666_at	3.19	2'-5'-oligoadenylate synthetase 3, 100kDa	OAS3
241115_at	3.19	KIAA1467	KIAA1467
240321_at	3.19		
241499_at	3.19	zinc finger protein 621	ZNF621
227195_at	3.18	zinc finger protein 503	ZNF503
231798_at	3.18	noggin	NOG
218279_s_at	3.18	histone cluster 2, H2aa3#histone cluster 2, H2aa4#histone cluster 2, H2aa4	HIST2H2AA3#HIST2H2AA4#HIST2H2AA4
238623_at	3.17		

209399_at	3.17	holocarboxylase synthetase (biotin-(proprionyl-Coenzyme A-carboxylase (ATP-hydrolysing)) ligase)	HLCS
1555505_a_at	3.17	tyrosinase (oculocutaneous albinism IA)	TYR
240413_at	3.17	interferon, gamma-inducible protein 16	IFI16
1561650_s_at	3.17		
1555018_at	3.17	olfactory receptor, family 2, subfamily C, member 3	OR2C3
227127_at	3.16	transmembrane protein 110	TMEM110
1561622_at	3.16		
231994_at	3.15	choline dehydrogenase	CHDH
240526_at	3.15	ATPase, Class VI, type 11A	ATP11A
234632_x_at	3.14		
238416_x_at	3.14		
224278_at	3.14	chromosome 2 open reading frame 14	C2orf14
205608_s_at	3.13	angiopoietin 1	ANGPT1
1553651_at	3.13	chromosome 18 open reading frame 54	C18orf54
205103_at	3.12	chromosome 1 open reading frame 61	C1orf61
205552_s_at	3.12	2',5'-oligoadenylate synthetase 1, 40/46kDa	OAS1
239282_at	3.11		
209969_s_at	3.11	signal transducer and activator of transcription 1, 91kDa	STAT1
218745_x_at	3.10	transmembrane protein 161A	TMEM161A
227609_at	3.10	epithelial stromal interaction 1 (breast)	EPSTI1
206386_at	3.09	serpin peptidase inhibitor, clade A (alpha-1 antiproteinase, antitrypsin), member 7	SERPINA7
209655_s_at	3.09	transmembrane protein 47	TMEM47
242631_x_at	3.09	deleted in liver cancer 1	DLC1
228904_at	3.08	homeobox B3	HOXB3
210534_s_at	3.06		
235175_at	3.06	guanylate binding protein 4	GBP4
234531_at	3.06		
214995_s_at	3.06	apolipoprotein B mRNA editing enzyme, catalytic polypeptide-like 3F	APOBEC3F
237723_at	3.05	SMAD specific E3 ubiquitin protein ligase 1	SMURF1
1562294_x_at	3.05	ankyrin repeat domain 30B	ANKRD30B
206847_s_at	3.04	homeobox A7	HOXA7
207051_at	3.04	solute carrier family 17 (sodium phosphate), member 4	SLC17A4
242088_at	3.02	kelch-like 24 (Drosophila)	KLHL24
239109_at	3.01		
239437_at	3.01		
1566762_at	3.01		
219670_at	3.00	chromosome 1 open reading frame 165	C1orf165
241844_x_at	3.00	transmembrane protein 156	TMEM156
216016_at	3.00	NLR family, pyrin domain containing 3	NLRP3
217139_at	2.99	voltage-dependent anion channel 1 pseudogene	VDAC1P
206553_at	2.99	2'-5'-oligoadenylate synthetase 2, 69/71kDa	OAS2
215818_at	2.99	nudix (nucleoside diphosphate linked moiety X)-type motif 7	NUDT7
1554540_at	2.98	chromosome 1 open reading frame 67	C1orf67
220358_at	2.98		
216183_at	2.98	transglutaminase 2 (C polypeptide, protein-glutamine-gamma-glutamyltransferase)	TGM2
1554140_at	2.97	WD repeat domain 78	WDR78

206938_at	2.96	steroid-5-alpha-reductase, alpha polypeptide 2 (3-oxo-5 alpha-steroid delta 4-dehydrogenase alpha 2)	SRD5A2
218943_s_at	2.96	DEAD (Asp-Glu-Ala-Asp) box polypeptide 58	DDX58
221577_x_at	2.95	growth differentiation factor 15	GDF15
232257_s_at	2.95		
232739_at	2.94	Spi-B transcription factor (Spi-1/PU.1 related)	SPIB
238464_at	2.94	KIAA1641	KIAA1641
200897_s_at	2.94	palladin, cytoskeletal associated protein	PALLD
216586_at	2.94	uracil-DNA glycosylase pseudogene 2	UNGP2
209905_at	2.94	homeobox A9	HOXA9
239956_at	2.93		
243753_at	2.93	chromosome 1 open reading frame 173	C1orf173
205793_x_at	2.92	tyrosine kinase, non-receptor, 1	TNK1
214022_s_at	2.90	interferon induced transmembrane protein 1 (9-27)	IFITM1
200907_s_at	2.90	palladin, cytoskeletal associated protein	PALLD
237354_at	2.90		
204720_s_at	2.88	DnaJ (Hsp40) homolog, subfamily C, member 6	DNAJC6
1568755_a_at	2.86		
235541_at	2.86	LAS1-like (S. cerevisiae)	LAS1L
234855_at	2.85		
1568827_at	2.84		
236235_at	2.84	itchy homolog E3 ubiquitin protein ligase (mouse)	ITCH
227915_at	2.84	ankyrin repeat and SOCS box-containing 2	ASB2
1556771_a_at	2.83		
231202_at	2.82		
1565325_at	2.82	small nucleolar RNA host gene (non-protein coding) 4	SNHG4
210273_at	2.82	BH-protocadherin (brain-heart)	PCDH7
219106_s_at	2.82	kelch repeat and BTB (POZ) domain containing 10	KBTBD10
203099_s_at	2.81	chromodomain protein, Y-like	CDYL
240211_at	2.81		
242005_at	2.80		
219937_at	2.80	thyrotropin-releasing hormone degrading enzyme	TRHDE
1558394_s_at	2.80	keratin 7	KRT7
236749_at	2.79	MAX binding protein	MNT
222293_at	2.79	cell adhesion molecule 4	CADM4
1554273_a_at	2.78		
226603_at	2.77	sterile alpha motif domain containing 9-like	SAMD9L
238592_at	2.77	PDZ and LIM domain 3	PDLIM3
234022_at	2.77		
208421_at	2.77		
224422_x_at	2.76	pro-melanin-concentrating hormone-like 2	PMCHL2
243500_at	2.76	CAS1 domain containing 1	CASD1
1553661_a_at	2.76	HUS1 checkpoint homolog b (S. pombe)	HUS1B
243583_at	2.75	transcription factor 7-like 2 (T-cell specific, HMG-box)	TCF7L2
1558164_s_at	2.75	peroxisome biogenesis factor 13	PEX13
201110_s_at	2.74	thrombospondin 1	THBS1
205028_at	2.74	trophinin	TRO
208401_s_at	2.73	glucagon-like peptide 1 receptor	GLP1R
237282_s_at	2.73	A kinase (PRKA) anchor protein 14	AKAP14
1556650_at	2.72	autism susceptibility candidate 2	AUTS2
228923_at	2.71	S100 calcium binding protein A6	S100A6
236286_at	2.71		
236895_at	2.71		

1565882_at	2.71		
207018_s_at	2.70	RAB27B, member RAS oncogene family	RAB27B
205224_at	2.70	surfeit 2	SURF2
219684_at	2.69	receptor (chemosensory) transporter protein 4	RTP4
214651_s_at	2.69	homeobox A9	HOXA9
1564027_a_at	2.69		
243343_at	2.68		
235643_at	2.68	sterile alpha motif domain containing 9-like	SAMD9L
228792_at	2.67		
219888_at	2.67	sperm associated antigen 4	SPAG4
1569938_at	2.67	sirtuin (silent mating type information regulation 2 homolog) 5 (S. cerevisiae)	SIRT5
1556092_s_at	2.67	HemK methyltransferase family member 1	HEMK1
226034_at	2.66		
236746_at	2.66	UDP-N-acetyl-alpha-D-galactosamine:polypeptide N-acetylgalactosaminyltransferase 1 (GalNAc-T1)	GALNT1
206806_at	2.66	diacylglycerol kinase, iota	DGKI
217699_at	2.66		
204505_s_at	2.66	erythrocyte membrane protein band 4.9 (dematin)	EPB49
226702_at	2.65		
1562850_at	2.65		
230803_s_at	2.65	Rho GTPase activating protein 24	ARHGAP24
1564653_s_at	2.65		
1554963_at	2.65	chromosome 6 open reading frame 192	C6orf192
1559520_at	2.65	glycophorin A (MNS blood group)	GYPA
226772_s_at	2.65	SAP30-like	SAP30L
204014_at	2.65	dual specificity phosphatase 4	DUSP4
206858_s_at	2.64	homeobox C6	HOXC6
215993_at	2.63		
219338_s_at	2.62	leucine rich repeat containing 49	LRRC49
209776_s_at	2.62	solute carrier family 19 (folate transporter), member 1	SLC19A1
232610_at	2.62	poly (ADP-ribose) polymerase family, member 14	PARP14
214767_s_at	2.62	heat shock protein, alpha-crystallin-related, B6	HSPB6
219352_at	2.62	hect domain and RLD 6	HERC6
233944_at	2.62		
213182_x_at	2.61	cyclin-dependent kinase inhibitor 1C (p57, Kip2)	CDKN1C
221460_at	2.61	olfactory receptor, family 2, subfamily C, member 1	OR2C1
210472_at	2.61	metallothionein 1G	MT1G
242873_at	2.61		
1557286_at	2.60		
244258_at	2.59		
1558871_at	2.59		
220758_s_at	2.59	roundabout homolog 4, magic roundabout (Drosophila)	ROBO4
220190_s_at	2.58	stonin 1	STON1
1562217_at	2.58		
204187_at	2.58	guanosine monophosphate reductase	GMPR
1554279_a_at	2.58	chromosome X open reading frame 34	CXorf34
234167_at	2.57	MKI67 (FHA domain) interacting nucleolar phosphoprotein	MKI67IP
210279_at	2.57	G protein-coupled receptor 18	GPR18
219668_at	2.57	ganglioside-induced differentiation-associated protein 1-like 1	GDAP1L1

1565900_at	2.56	methyltransferase 5 domain containing 1	METT5D1
240769_at	2.56		
242973_at	2.56	calcium channel, voltage-dependent, L type, alpha 1C subunit	CACNA1C
233813_at	2.56	protein phosphatase 1, regulatory (inhibitor) subunit 16B	PPP1R16B
205901_at	2.56	prepronociceptin	PNOC
233934_at	2.55		
220250_at	2.55	zinc finger protein 286A	ZNF286A
1564165_at	2.55	PRKR interacting protein 1 (IL11 inducible)	PRKRIP1
207346_at	2.55	syntaxin 2	STX2
209469_at	2.55	glycoprotein M6A	GPM6A
237291_at	2.54		
1569974_x_at	2.53		
238367_s_at	2.52		
200906_s_at	2.52	palladin, cytoskeletal associated protein	PALLD
243136_at	2.51		
209886_s_at	2.51	SMAD family member 6	SMAD6
1566569_at	2.50		
216240_at	2.50	Pvt1 oncogene homolog, MYC activator (mouse)	PVT1
239723_at	2.50		
206951_at	2.50	histone cluster 1, H4e	HIST1H4E
224365_s_at	2.48	tigger transposable element derived 7	TIGD7
222717_at	2.48	serum deprivation response (phosphatidylserine binding protein)	SDPR
1565820_x_at	2.48	phosphatase and actin regulator 4	PHACTR4
234474_x_at	2.47	interleukin 6 signal transducer (gp130, oncostatin M receptor)	IL6ST
204069_at	2.47	Meis1, myeloid ecotropic viral integration site 1 homolog (mouse)	MEIS1
229279_at	2.47		
1568903_at	2.47		
229151_at	2.46	solute carrier family 14 (urea transporter), member 1 (Kidd blood group)	SLC14A1
224389_s_at	2.46	collagen, type XXV, alpha 1	COL25A1
200887_s_at	2.45	signal transducer and activator of transcription 1, 91kDa	STAT1
201416_at	2.45	SRY (sex determining region Y)-box 4	SOX4
208436_s_at	2.45	interferon regulatory factor 7	IRF7
1569140_at	2.45	ubiquitin protein ligase E3 component n-recognin 2	UBR2
205195_at	2.44	adaptor-related protein complex 1, sigma 1 subunit	AP1S1
207566_at	2.43	major histocompatibility complex, class I-related	MR1
203706_s_at	2.43	frizzled homolog 7 (Drosophila)	FZD7
219884_at	2.43	LIM homeobox 6	LHX6
211791_s_at	2.43	potassium voltage-gated channel, shaker-related subfamily, beta member 2	KCNAB2
225627_s_at	2.43	cache domain containing 1	CACHD1
242462_at	2.42		
216758_at	2.42		
222882_s_at	2.42	chromosome 17 open reading frame 59	C17orf59
224701_at	2.42	poly (ADP-ribose) polymerase family, member 14	PARP14
229971_at	2.42	G protein-coupled receptor 114	GPR114
204015_s_at	2.41	dual specificity phosphatase 4	DUSP4

222558_at	2.40		
228215_at	2.40	adducin 3 (gamma)	ADD3
1554631_at	2.40	ataxia telangiectasia mutated (includes complementation groups A, C and D)	ATM
226101_at	2.39	protein kinase C, epsilon	PRKCE
237225_at	2.38		
217397_at	2.37	T cell receptor alpha locus#T cell receptor alpha variable 6#T cell receptor alpha variable 16#T cell receptor alpha variable 15#T cell receptor alpha variable 14/delta variable 4#T cell receptor alpha variable 13-2#T cell receptor alpha variable 13-1#T cell receptor alpha variable 12-3#T cell receptor alpha variable 12-2#T cell receptor alpha variable 12-1#T cell receptor alpha variable 11#T cell receptor alpha variable 10#T cell receptor alpha variable 9-2#T cell receptor alpha variable 9-1#T cell receptor alpha variable 8-6#T cell receptor alpha variable 8-5#T cell receptor alpha variable 8-4#T cell receptor alpha variable 8-3#T cell receptor alpha variable 8-2#T cell receptor alpha variable 8-1#T cell receptor alpha variable 7#T cell receptor alpha variable 5#null	TRA@#TRAV6#TRAV16#TRAV15#TRAV14DV4#TRAV13-2#TRAV13-1#TRAV12-3#TRAV12-2#TRAV12-1#TRAV11#TRAV10#TRAV9-2#TRAV9-1#TRAV8-6#TRAV8-5#TRAV8-4#TRAV8-3#TRAV8-2#TRAV8-1#TRAV7#TRAV5#null
243271_at	2.36		
202890_at	2.36	microtubule-associated protein 7	MAP7
1552825_at	2.36	zinc finger protein 396	ZNF396
1568638_a_at	2.36	indoleamine-pyrrole 2,3 dioxygenase-like 1	INDOL1
1556747_a_at	2.35		
210665_at	2.35	tissue factor pathway inhibitor (lipoprotein-associated coagulation inhibitor)	TFPI
1557535_at	2.35	palladin, cytoskeletal associated protein	PALLD
239846_at	2.34		
238566_at	2.34		
233388_at	2.33		
1556123_a_at	2.33		
236107_at	2.33	ubiquitin-conjugating enzyme E2Z (putative)	UBE2Z
1559441_s_at	2.32		
1553789_a_at	2.32	chromosome 21 open reading frame 58	C21orf58
244267_at	2.31	special AT-rich sequence binding protein 1 (binds to nuclear matrix/scaffold-associating DNA's)	SATB1
235268_at	2.31		
220816_at	2.31	endothelial differentiation, lysophosphatidic acid G-protein-coupled receptor, 7	EDG7
1562236_at	2.31	MYST histone acetyltransferase (monocytic leukemia) 4	MYST4
214999_s_at	2.30	RAB11 family interacting protein 3 (class II)	RAB11FIP3
231888_at	2.30		
228558_at	2.29	chromosome 14 open reading frame 80	C14orf80
1556682_s_at	2.29		
222972_at	2.28	visual system homeobox 1 homolog, CHX10-like (zebrafish)	VSX1

1566846_at	2.28		
220685_at	2.27	family with sequence similarity 120C	FAM120C
210360_s_at	2.27	metastasis suppressor 1	MTSS1
237431_at	2.26	ubiquitin specific peptidase 32	USP32
1562585_at	2.26		
205442_at	2.26	microfibrillar-associated protein 3-like	MFAP3L
225929_s_at	2.26	ring finger protein 213	RNF213
205789_at	2.26	CD1d molecule	CD1D
206385_s_at	2.25	ankyrin 3, node of Ranvier (ankyrin G)	ANK3
203596_s_at	2.25	interferon-induced protein with tetratricopeptide repeats 5	IFIT5
242234_at	2.25		
216439_at	2.24	tyrosine kinase, non-receptor, 2	TNK2
1557651_x_at	2.24	UDP-galactose-4-epimerase	GALE
241147_at	2.24		
1562282_at	2.23	zinc finger protein 568	ZNF568
202257_s_at	2.23	CD2 (cytoplasmic tail) binding protein 2	CD2BP2
1554303_at	2.23	histamine N-methyltransferase	HNMT
229085_at	2.22	leucine rich repeat containing 3B	LRRC3B
1564670_at	2.22		
217204_at	2.22	mitochondrial translational release factor 1-like	MTRF1L
220858_at	2.21	sorbin and SH3 domain containing 2	SORBS2
209980_s_at	2.21	serine hydroxymethyltransferase 1 (soluble)	SHMT1
241319_at	2.21	exocyst complex component 6	EXOC6
230352_at	2.21	phosphoribosyl pyrophosphate synthetase 2	PRPS2
228447_at	2.20	chromosome X and Y open reading frame 3	CXYorf3
216814_at	2.20	SERPINE1 mRNA binding protein 1 pseudogene	SERBP1P
226454_at	2.19	membrane-associated ring finger (C3HC4) 9	9-Mar
221942_s_at	2.19	guanylate cyclase 1, soluble, alpha 3	GUCY1A3
228531_at	2.18	sterile alpha motif domain containing 9	SAMD9
210797_s_at	2.18	2'-5'-oligoadenylate synthetase-like	OASL
1557113_at	2.18		
239951_at	2.18		
209614_at	2.18	alcohol dehydrogenase IB (class I), beta polypeptide	ADH1B
240293_at	2.18		
231577_s_at	2.18	guanylate binding protein 1, interferon-inducible, 67kDa	GBP1
213982_s_at	2.17	RAB GTPase activating protein 1-like	RABGAP1L
215745_at	2.17		
235157_at	2.17		
240603_s_at	2.16	exonuclease domain containing 1	EXOD1
1562245_a_at	2.16		
239330_at	2.16		
220030_at	2.16	serine/threonine/tyrosine kinase 1	STYK1
210827_s_at	2.16	E74-like factor 3 (ets domain transcription factor, epithelial-specific)	ELF3
219638_at	2.15	F-box protein 22	FBXO22
226771_at	2.15	ATPase, Class I, type 8B, member 2	ATP8B2
1555032_at	2.14	regulator of G-protein signalling 12	RGS12
1553674_at	2.12	leucine rich repeat containing 44	LRRC44
210387_at	2.12	histone cluster 1, H2bg	HIST1H2BG
204256_at	2.11	ELOVL family member 6, elongation of long chain fatty acids (FEN1/Elo2, SUR4/Elo3-like, yeast)	ELOVL6

224204_x_at	2.11	aryl hydrocarbon receptor nuclear translocator-like 2	ARNTL2
212093_s_at	2.10	mitochondrial tumor suppressor 1	MTUS1
224609_at	2.10	solute carrier family 44, member 2	SLC44A2
234306_s_at	2.10	SLAM family member 7	SLAMF7
210796_x_at	2.10	sialic acid binding Ig-like lectin 6	SIGLEC6
213294_at	2.09		
219531_at	2.09	centrosomal protein 72kDa	CEP72
207037_at	2.09	tumor necrosis factor receptor superfamily, member 11a, NFKB activator	TNFRSF11A
229124_at	2.08	prokineticin 1	PROK1
208622_s_at	2.08		
221558_s_at	2.08	lymphoid enhancer-binding factor 1	LEF1
1569159_at	2.07		
219522_at	2.07	four jointed box 1 (Drosophila)	FJX1
207125_at	2.06	zinc finger protein 225	ZNF225
231320_at	2.06	TBC1 domain family, member 25	TBC1D25
205653_at	2.06	cathepsin G	CTSG
205767_at	2.06	epiregulin	EREG
223279_s_at	2.06	uveal autoantigen with coiled-coil domains and ankyrin repeats	UACA
229134_at	2.05	vang-like 1 (van gogh, Drosophila)	VANGL1
1553842_at	2.05	chromosome X open reading frame 20	CXorf20
244092_at	2.05	zinc finger, RAN-binding domain containing 3	ZRANB3
227860_at	2.05	carboxypeptidase X (M14 family), member 1	CPXM1
1554163_at	2.05	twist homolog 2 (Drosophila)	TWIST2
217363_x_at	2.04		
216440_at	2.04	ELKS/RAB6-interacting/CAST family member 1	ERC1
243138_at	2.03		
205468_s_at	2.03	interferon regulatory factor 5	IRF5
214945_at	2.03		
244509_at	2.03	G protein-coupled receptor 155	GPR155
219683_at	2.02	frizzled homolog 3 (Drosophila)	FZD3
206289_at	2.02	homeobox A4	HOXA4
224058_s_at	2.02	hydroxysteroid (17-beta) dehydrogenase 7 pseudogene 2	HSD17B7P2
237843_at	2.01		
1553127_a_at	2.00	ring finger protein 168	RNF168
215810_x_at	2.00		
202709_at	0.50	fibromodulin	FMOD
244317_at	0.50	KIAA1324-like	KIAA1324L
1559375_s_at	0.50	ATPase, H+ transporting, lysosomal V0 subunit a1	ATP6V0A1
243064_at	0.50		
211178_s_at	0.50	proline-serine-threonine phosphatase interacting protein 1	PSTPIP1
228873_at	0.50	collagen, type XXII, alpha 1	COL22A1
201279_s_at	0.50	disabled homolog 2, mitogen-responsive phosphoprotein (Drosophila)	DAB2
205294_at	0.50	BAI1-associated protein 2	BAIAP2
207374_at	0.50	phospholipid scramblase 2	PLSCR2
236796_at	0.50	BTB and CNC homology 1, basic leucine zipper transcription factor 2	BACH2
230654_at	0.50		
228472_at	0.50		

217684_at	0.50	thymidylate synthetase	TYMS
243792_x_at	0.50	protein tyrosine phosphatase, non-receptor type 13 (APO-1/CD95 (Fas)-associated phosphatase)	PTPN13
223876_at	0.50	spermatogenesis associated 16	SPATA16
207056_s_at	0.50	solute carrier family 4, sodium bicarbonate cotransporter, member 8	SLC4A8
209771_x_at	0.50	CD24 molecule	CD24
217757_at	0.50	alpha-2-macroglobulin	A2M
210818_s_at	0.50	BTB and CNC homology 1, basic leucine zipper transcription factor 1	BACH1
206701_x_at	0.50	endothelin receptor type B	EDNRB
226805_at	0.49		
242611_at	0.49		
1562671_s_at	0.49		
211965_at	0.49	zinc finger protein 36, C3H type-like 1	ZFP36L1
1559347_at	0.49	CCR4-NOT transcription complex, subunit 4	CNOT4
209774_x_at	0.49	chemokine (C-X-C motif) ligand 2	CXCL2
1560754_at	0.49	CKLF-like MARVEL transmembrane domain containing 7	CMTM7
206945_at	0.49	lactase	LCT
232641_at	0.49	zinc finger protein 596#olfactory receptor, family 4, subfamily F, member 21#null	ZNF596#OR4F 21#null
240169_at	0.49		
1560034_a_at	0.49		
216112_at	0.49	protein kinase N2	PKN2
1557228_at	0.49	EH domain binding protein 1-like 1	EHBP1L1
242663_at	0.49		
208146_s_at	0.49	carboxypeptidase, vitellogenic-like	CPVL
229883_at	0.49	glutamate receptor, ionotropic, N-methyl D-aspartate 2D	GRIN2D
216379_x_at	0.49	CD24 molecule	CD24
209823_x_at	0.49	major histocompatibility complex, class II, DQ beta 1	HLA-DQB1
1560753_at	0.49		
228552_s_at	0.49	single stranded DNA binding protein 4	SSBP4
218086_at	0.49	neural proliferation, differentiation and control, 1	NPDC1
205798_at	0.49	interleukin 7 receptor	IL7R
206538_at	0.49	muscle RAS oncogene homolog	MRAS
1558019_at	0.49		
208322_s_at	0.49	ST3 beta-galactoside alpha-2,3-sialyltransferase 1	ST3GAL1
243493_at	0.49		
244627_at	0.49	dihydroxyacetone kinase 2 homolog (S. cerevisiae)	DAK
222913_at	0.49	Kruppel-like factor 3 (basic)	KLF3
232611_at	0.49		
213221_s_at	0.49	SNF1-like kinase 2	SNF1LK2
242117_at	0.49		
209253_at	0.49	sorbin and SH3 domain containing 3	SORBS3
221185_s_at	0.49	IQ motif containing G	IQCG
1561523_at	0.49	coiled-coil domain containing 65	CCDC65
1558640_a_at	0.49		
211812_s_at	0.49	beta-1,3-N-acetylgalactosaminyltransferase 1 (globoside blood group)	B3GALNT1
201340_s_at	0.49	ectodermal-neural cortex (with BTB-like domain)	ENC1
1554594_at	0.49		

1555168_a_at	0.49	calneuron 1	CALN1
226643_s_at	0.49	NudC domain containing 2	NUDCD2
236001_at	0.49		
204150_at	0.49	stabilin 1	STAB1
242677_at	0.49		
239715_at	0.49		
224235_at	0.49		
233799_at	0.49		
236654_s_at	0.49		
242129_at	0.48	SIN3 homolog B, transcription regulator (yeast)	SIN3B
236942_at	0.48		
223399_x_at	0.48		
219095_at	0.48	phospholipase A2, group IVB (cytosolic)	PLA2G4B
236577_at	0.48		
1553224_at	0.48	leucine zipper protein 1	LUZP1
241129_at	0.48	amyotrophic lateral sclerosis 2 (juvenile) chromosome region, candidate 2	ALS2CR2
217637_at	0.48		
233399_x_at	0.48		
226436_at	0.48	Ras association (RalGDS/AF-6) domain family 4	RASSF4
1555141_a_at	0.48		
1569380_a_at	0.48		
243263_at	0.48	chromosome 17 open reading frame 55	C17orf55
214455_at	0.48	histone cluster 1, H2bc	HIST1H2BC
205776_at	0.48	flavin containing monooxygenase 5	FMO5
228034_x_at	0.48	alkB, alkylation repair homolog 5 (E. coli)	ALKBH5
1556808_at	0.48		
242893_at	0.48		
241963_at	0.48	zinc finger protein 704	ZNF704
244668_at	0.48		
223291_at	0.48	mitochondrial ribosomal protein S15	MRPS15
1559131_a_at	0.48		
213839_at	0.48		
1566294_at	0.48		
222727_s_at	0.48	solute carrier family 24 (sodium/potassium/calcium exchanger), member 6	SLC24A6
226404_at	0.48	RNA binding motif protein 39	RBM39
1553401_at	0.48	MAS-related GPR, member X1	MRGPRX1
203866_at	0.48	notchless homolog 1 (Drosophila)	NLE1
1554578_at	0.48	zinc finger protein 70	ZNF70
1556938_a_at	0.48	dynein, light chain, LC8-type 1	DYNLL1
235437_at	0.48		
201341_at	0.48	ectodermal-neural cortex (with BTB-like domain)	ENC1
1560639_at	0.48		
217375_at	0.48		
242822_at	0.48		
230084_at	0.48	solute carrier family 30 (zinc transporter), member 2	SLC30A2
231875_at	0.48	kinesin family member 21A	KIF21A
236138_at	0.48		
208488_s_at	0.48	complement component (3b/4b) receptor 1 (Knops blood group)	CR1
235501_at	0.48		
1568633_a_at	0.48		

224067_at	0.48		
206721_at	0.47	chromosome 1 open reading frame 114	C1orf114
220210_at	0.47	cholinergic receptor, nicotinic, alpha 10	CHRNA10
205500_at	0.47	complement component 5	C5
244430_at	0.47		
220141_at	0.47	chromosome 11 open reading frame 63	C11orf63
204567_s_at	0.47	ATP-binding cassette, sub-family G (WHITE), member 1	ABCG1
212998_x_at	0.47	major histocompatibility complex, class II, DQ beta 1	HLA-DQB1
222350_at	0.47		
207277_at	0.47	CD209 molecule	CD209
215263_at	0.47	zinc finger, X-linked, duplicated A	ZXDA
240038_at	0.47		
227236_at	0.47	tetraspanin 2	TSPAN2
210375_at	0.47	prostaglandin E receptor 3 (subtype EP3)	PTGER3
206237_s_at	0.47	neuregulin 1	NRG1
213252_at	0.47	SH3 and PX domains 2A	SH3PXD2A
237772_at	0.47		
213121_at	0.47	small nuclear ribonucleoprotein 70kDa polypeptide (RNP antigen)	SNRP70
212099_at	0.47	ras homolog gene family, member B	RHOB
209883_at	0.47	glycosyltransferase 25 domain containing 2	GLT25D2
564_at	0.47	guanine nucleotide binding protein (G protein), alpha 11 (Gq class)	GNA11
224040_at	0.47	testis-specific transcript, Y-linked 5	TTTY5
238432_at	0.47		
228043_at	0.47	UTP15, U3 small nucleolar ribonucleoprotein, homolog (S. cerevisiae)	UTP15
206412_at	0.47	fer (fps/fes related) tyrosine kinase (phosphoprotein NCP94)	FER
1565638_at	0.47		
208868_s_at	0.47	GABA(A) receptor-associated protein like 1	GABARAPL1
1557683_at	0.47	small nuclear ribonucleoprotein 70kDa polypeptide (RNP antigen)	SNRP70
230134_s_at	0.47		
239158_at	0.47		
244682_at	0.47	calmodulin regulated spectrin-associated protein 1	CAMSAP1
203485_at	0.47	reticulon 1	RTN1
230697_at	0.47	Bardet-Biedl syndrome 5	BBS5
223660_at	0.47	adenosine A3 receptor	ADORA3
1560369_at	0.46	ankylosis, progressive homolog (mouse)	ANKH
1555689_at	0.46	CD80 molecule	CD80
225481_at	0.46	FERM domain containing 6	FRMD6
242664_at	0.46	protein tyrosine phosphatase, receptor type, M	PTPRM
208241_at	0.46	neuregulin 1	NRG1
1555675_at	0.46		
228005_at	0.46	zinc finger, X-linked, duplicated B	ZXDB
1552991_at	0.46	olfactory receptor, family 5, subfamily P, member 2	OR5P2
231193_s_at	0.46		
241216_at	0.46	kinesin family member 1B	KIF1B
211355_x_at	0.46	leptin receptor	LEPR
242937_at	0.46	forkhead box K2	FOXK2
1564767_at	0.46		

218380_at	0.46	NLR family, pyrin domain containing 1	NLRP1
235118_at	0.46		
210479_s_at	0.46	RAR-related orphan receptor A	RORA
210651_s_at	0.46	EPH receptor B2	EPHB2
213524_s_at	0.46	G0/G1switch 2	G0S2
1554306_at	0.46	inositol 1,4,5-trisphosphate 3-kinase B	ITPKB
241517_at	0.46	development and differentiation enhancing factor 1	DDEF1
213415_at	0.46	chloride intracellular channel 2	CLIC2
243017_at	0.46		
1556279_at	0.46		
205819_at	0.46	macrophage receptor with collagenous structure	MARCO
231710_at	0.46	calcyphosine	CAPS
215811_at	0.46		
229504_at	0.46		
242103_at	0.46	transmembrane protein 86A	TMEM86A
215990_s_at	0.46	B-cell CLL/lymphoma 6 (zinc finger protein 51)	BCL6
237525_at	0.46		
1563524_a_at	0.46	chromosome 14 open reading frame 85	C14orf85
229327_s_at	0.46	v-maf musculoaponeurotic fibrosarcoma oncogene homolog (avian)	MAF
1553934_at	0.46	chromosome 18 open reading frame 20	C18orf20
240958_at	0.46	unc-5 homolog C (C. elegans)	UNC5C
203695_s_at	0.46	deafness, autosomal dominant 5	DFNA5
1558969_a_at	0.46	ribosomal protein L32 pseudogene 3	RPL32P3
238467_at	0.46		
232858_at	0.46		
234403_at	0.46	ilvB (bacterial acetolactate synthase)-like#synapse defective 1, Rho GTPase, homolog 1 (C. elegans)#olfactory receptor, family 1, subfamily I, member 1#olfactory receptor, family 10, subfamily B, member 1 pseudogene	ILVBL#SYDE1#OR1I1#OR10B1P
244282_at	0.46		
220929_at	0.46	UDP-N-acetyl-alpha-D-galactosamine:polypeptide N-acetylgalactosaminyltransferase 8 (GalNAc-T8)	GALNT8
227136_s_at	0.46	chromosome 10 open reading frame 46	C10orf46
208791_at	0.46	clusterin	CLU
215845_x_at	0.46		
244710_at	0.46	leucine-rich repeats and guanylate kinase domain containing	LRGUK
236564_at	0.46		
228568_at	0.46		
228602_at	0.46	sarcoglycan, delta (35kDa dystrophin-associated glycoprotein)	SGCD
220150_s_at	0.46	chromosome 6 open reading frame 60	C6orf60
1564639_at	0.46		
244090_at	0.46		
218820_at	0.46	chromosome 14 open reading frame 132	C14orf132
241794_at	0.46	ARP6 actin-related protein 6 homolog (yeast)	ACTR6
1558745_at	0.45		
236316_at	0.45	family with sequence similarity 3, member C	FAM3C
205005_s_at	0.45	N-myristoyltransferase 2	NMT2
215062_at	0.45		
220582_at	0.45		

242742_at	0.45		
228791_at	0.45	chromosome 15 open reading frame 38	C15orf38
242284_at	0.45		
219962_at	0.45	angiotensin I converting enzyme (peptidyl-dipeptidase A) 2	ACE2
205695_at	0.45	serine dehydratase	SDS
243399_at	0.45		
219393_s_at	0.45	v-akt murine thymoma viral oncogene homolog 3 (protein kinase B, gamma)	AKT3
209395_at	0.45	chitinase 3-like 1 (cartilage glycoprotein-39)	CHI3L1
244231_at	0.45		
207498_s_at	0.45	cytochrome P450, family 2, subfamily D, polypeptide 6	CYP2D6
1569834_at	0.45		
221578_at	0.45	Ras association (RalGDS/AF-6) domain family 4	RASSF4
210510_s_at	0.45	neuropilin 1	NRP1
223503_at	0.45	transmembrane protein 163	TMEM163
237173_at	0.45		
1554288_at	0.45	KIAA1600	KIAA1600
220900_at	0.45		
243396_at	0.45		
243468_at	0.44		
1560792_at	0.44		
207123_s_at	0.44	matrilin 4	MATN4
236377_at	0.44	transmembrane protein 132D	TMEM132D
217340_at	0.44	leucine rich repeat containing 16	LRRC16
220791_x_at	0.44	sodium channel, voltage-gated, type XI, alpha subunit	SCN11A
209396_s_at	0.44	chitinase 3-like 1 (cartilage glycoprotein-39)	CHI3L1
204682_at	0.44	latent transforming growth factor beta binding protein 2	LTBP2
1564640_at	0.44	MAX gene associated	MGA
203305_at	0.44	coagulation factor XIII, A1 polypeptide	F13A1
226878_at	0.44	major histocompatibility complex, class II, DO alpha	HLA-DOA
216442_x_at	0.44	fibronectin 1	FN1
224323_s_at	0.44	maestro	MRO
239519_at	0.44	neuropilin 1	NRP1
239411_at	0.44	zinc finger protein 496	ZNF496
222378_at	0.44		
213125_at	0.44	olfactomedin-like 2B	OLFML2B
205249_at	0.44	early growth response 2 (Krox-20 homolog, Drosophila)	EGR2
1557961_s_at	0.44		
1554889_at	0.44	TIA1 cytotoxic granule-associated RNA binding protein	TIA1
211557_x_at	0.44	solute carrier organic anion transporter family, member 2B1	SLCO2B1
241292_at	0.44		
206729_at	0.44	tumor necrosis factor receptor superfamily, member 8	TNFRSF8
1563475_s_at	0.44		
1569672_at	0.44		
224429_x_at	0.44		
243719_at	0.44	serine/threonine kinase 19	STK19

201278_at	0.44	disabled homolog 2, mitogen-responsive phosphoprotein (Drosophila)	DAB2
238713_at	0.44		
230578_at	0.44		
222218_s_at	0.44	paired immunoglobin-like type 2 receptor alpha	PILRA
1553708_at	0.44		
1566672_at	0.44		
201280_s_at	0.44	disabled homolog 2, mitogen-responsive phosphoprotein (Drosophila)	DAB2
230928_at	0.44		
233908_x_at	0.44		
222245_s_at	0.44	fer-1-like 4 (C. elegans)	FER1L4
1562878_at	0.44		
228111_s_at	0.44	dynein, axonemal, heavy chain 1	DNAH1
213832_at	0.44		
225408_at	0.43	myelin basic protein	MBP
215748_at	0.43		
1564963_x_at	0.43	zinc finger protein 92	ZNF92
243625_at	0.43	cAMP responsive element binding protein 1	CREB1
205556_at	0.43	msh homeobox 2	MSX2
223937_at	0.43	forkhead box P1	FOXP1
1556429_a_at	0.43	WD repeat domain 67	WDR67
1552587_at	0.43	cyclic nucleotide binding domain containing 1	CNBD1
1553523_at	0.43	NLR family, pyrin domain containing 14	NLRP14
229242_at	0.43		
1560348_at	0.43		
242007_at	0.43		
207113_s_at	0.43	tumor necrosis factor (TNF superfamily, member 2)	TNF
1554418_s_at	0.43	sparc/osteonectin, cwcv and kazal-like domains proteoglycan (testican) 3	SPOCK3
239322_at	0.43		
238796_at	0.43		
212182_at	0.43	nudix (nucleoside diphosphate linked moiety X)-type motif 4	NUDT4
1561600_at	0.43		
266_s_at	0.43	CD24 molecule	CD24
1569690_at	0.43	coiled-coil domain containing 36	CCDC36
1557583_at	0.43	suppression of tumorigenicity 18 (breast carcinoma) (zinc finger protein)	ST18
210800_at	0.43	translocase of inner mitochondrial membrane 8 homolog A (yeast)	TIMM8A
234611_at	0.43		
233057_at	0.43		
222290_at	0.43	olfactory receptor, family 2, subfamily A, member 9 pseudogene	OR2A9P
216233_at	0.43	CD163 molecule	CD163
243226_at	0.43		
228298_at	0.43	family with sequence similarity 113, member B	FAM113B
227394_at	0.43	neural cell adhesion molecule 1	NCAM1
212096_s_at	0.43	mitochondrial tumor suppressor 1	MTUS1
1566537_at	0.43		
240510_at	0.43	nibrin	NBN
235292_at	0.43		

211460_at	0.42	testis-specific transcript, Y-linked 9A	TTTY9A
228436_at	0.42	potassium voltage-gated channel, Shaw-related subfamily, member 4	KCNC4
230952_at	0.42		
1564338_at	0.42		
237458_at	0.42		
234125_at	0.42		
220602_s_at	0.42		
239517_at	0.42		
1552414_at	0.42	WAP four-disulfide core domain 9	WFDC9
1554934_at	0.42	regulator of chromosome condensation (RCC1) and BTB (POZ) domain containing protein 1	RCBTB1
238281_at	0.42	RNA binding motif, single stranded interacting protein 1	RBMS1
230503_at	0.42	sterile alpha motif domain containing 4A	SAMD4A
217245_at	0.42		
227752_at	0.42	serine palmitoyltransferase, long chain base subunit 3	SPTLC3
230688_at	0.42		
1561673_at	0.42		
221872_at	0.42	retinoic acid receptor responder (tazarotene induced) 1	RARRES1
1552509_a_at	0.42	CD300 molecule-like family member g	CD300LG
1562903_at	0.42		
242636_at	0.42		
234783_at	0.42		
1555606_a_at	0.42	glycerophosphodiester phosphodiesterase domain containing 1	GDPD1
203757_s_at	0.42	carcinoembryonic antigen-related cell adhesion molecule 6 (non-specific cross reacting antigen)	CEACAM6
227189_at	0.42	copine V	CPNE5
240110_at	0.42		
228665_at	0.42	cysteine/tyrosine-rich 1	CYYR1
1558692_at	0.42	chromosome 1 open reading frame 85	C1orf85
244226_s_at	0.42	ring finger protein 43	RNF43
1560826_at	0.42		
224494_x_at	0.42	hydroxysteroid (17-beta) dehydrogenase 14	HSD17B14
202283_at	0.42	serpin peptidase inhibitor, clade F (alpha-2 antiplasmin, pigment epithelium derived factor), member	SERPINF1
203473_at	0.42	solute carrier organic anion transporter family, member 2B1	SLCO2B1
214366_s_at	0.42	arachidonate 5-lipoxygenase	ALOX5
215508_at	0.42	BUB1 budding uninhibited by benzimidazoles 1 homolog (yeast)	BUB1
232579_at	0.42		
234760_at	0.42	olfactory receptor, family 2, subfamily H, member 4 pseudogene	OR2H4P
207255_at	0.42	leptin receptor	LEPR
244269_at	0.41		
207789_s_at	0.41	dipeptidyl-peptidase 6	DPP6
208539_x_at	0.41	small proline-rich protein 2D	SPRR2D
234233_s_at	0.41	kelch repeat and BTB (POZ) domain containing 2	KBTBD2
233765_at	0.41		

234134_at	0.41		
210322_x_at	0.41	ubiquitously transcribed tetratricopeptide repeat gene, Y-linked	UTY
242083_at	0.41		
216467_s_at	0.41		
206392_s_at	0.41	retinoic acid receptor responder (tazarotene induced) 1	RARRES1
236011_at	0.41	forkhead box K1	FOXK1
1566881_at	0.41		
1560081_at	0.41		
237700_at	0.41		
1561090_at	0.41		
1562953_s_at	0.41	chromosome 4 open reading frame 12	C4orf12
236199_at	0.41	arachidonate 5-lipoxygenase	ALOX5
206135_at	0.41	suppression of tumorigenicity 18 (breast carcinoma) (zinc finger protein)	ST18
231034_s_at	0.41	NHS-like 1	NHSL1
233155_at	0.41	uridine phosphorylase 2	UPP2
219454_at	0.41	EGF-like-domain, multiple 6	EGFL6
234087_at	0.41		
242680_at	0.41		
1562894_at	0.41		
1562386_s_at	0.41	zinc finger protein 501	ZNF501
1564463_at	0.41		
1561365_at	0.41	neuropilin 1	NRP1
1570490_at	0.41		
243130_at	0.41		
208651_x_at	0.41	CD24 molecule	CD24
221466_at	0.41	pyrimidinergic receptor P2Y, G-protein coupled, 4	P2RY4
210761_s_at	0.41	growth factor receptor-bound protein 7	GRB7
230666_at	0.41		
1553193_at	0.41	zinc finger protein 441	ZNF441
204446_s_at	0.41	arachidonate 5-lipoxygenase	ALOX5
207323_s_at	0.41	myelin basic protein	MBP
1553354_a_at	0.41		
243439_at	0.41	zinc finger protein 418	ZNF418
211032_at	0.41	COBL-like 1	COBLL1
227855_at	0.41	zinc finger protein 219	ZNF219
203083_at	0.41	thrombospondin 2	THBS2
242790_at	0.41		
202075_s_at	0.41	phospholipid transfer protein	PLTP
229260_at	0.40	chromosome 5 open reading frame 15	C5orf15
242958_x_at	0.40		
1562106_at	0.40		
233321_x_at	0.40		
222853_at	0.40	fibronectin leucine rich transmembrane protein 3	FLRT3
201348_at	0.40	glutathione peroxidase 3 (plasma)	GPX3
240123_at	0.40		
220027_s_at	0.40	Ras interacting protein 1	RASIP1
204642_at	0.40	endothelial differentiation, sphingolipid G-protein-coupled receptor, 1	EDG1
224029_x_at	0.40	sodium channel, voltage-gated, type XI, alpha subunit	SCN11A
1570160_at	0.40		

215300_s_at	0.40	flavin containing monooxygenase 5	FMO5
237996_at	0.40		
227657_at	0.40	ring finger protein 150	RNF150
204580_at	0.40	matrix metallopeptidase 12 (macrophage elastase)	MMP12
222877_at	0.40		
243324_x_at	0.40		
220830_at	0.40	interphotoreceptor matrix proteoglycan 2	IMPG2
214627_at	0.40	eosinophil peroxidase	EPX
237696_at	0.40		
214523_at	0.40	CCAAT/enhancer binding protein (C/EBP), epsilon	CEBPE
200785_s_at	0.40	low density lipoprotein-related protein 1 (alpha-2-macroglobulin receptor)	LRP1
1557875_at	0.40		
1561606_at	0.40		
206211_at	0.39	selectin E (endothelial adhesion molecule 1)	SELE
210119_at	0.39	potassium inwardly-rectifying channel, subfamily J, member 15	KCNJ15
215298_at	0.39		
244840_x_at	0.39		
1554474_a_at	0.39	monooxygenase, DBH-like 1	MOXD1
218330_s_at	0.39	neuron navigator 2	NAV2
229140_at	0.39	zinc finger protein 579	ZNF579
202953_at	0.39	complement component 1, q subcomponent, B chain	C1QB
1557620_a_at	0.39	coiled-coil domain containing 38	CCDC38
229048_at	0.39		
206749_at	0.39	CD1b molecule	CD1B
205481_at	0.39	adenosine A1 receptor	ADORA1
237770_at	0.39		
239438_at	0.39	Rap guanine nucleotide exchange factor (GEF) 6	RAPGEF6
1556469_s_at	0.39		
1560142_at	0.39	glutamate receptor, ionotropic, kainate 2	GRIK2
213849_s_at	0.39	protein phosphatase 2 (formerly 2A), regulatory subunit B, beta isoform	PPP2R2B
203811_s_at	0.39	DnaJ (Hsp40) homolog, subfamily B, member 4	DNAJB4
228405_at	0.39	rhophilin, Rho GTPase binding protein 1	RHPN1
217206_at	0.39		
211751_at	0.39	phosphodiesterase 4D interacting protein (myomegalin)	PDE4DIP
1552747_a_at	0.39	chromosome 3 open reading frame 48	C3orf48
233683_at	0.39		
232898_at	0.39	disabled homolog 2, mitogen-responsive phosphoprotein (Drosophila)	DAB2
220682_s_at	0.39	kelch-like 5 (Drosophila)	KLHL5
242168_at	0.39	NADH dehydrogenase (ubiquinone) Fe-S protein 7, 20kDa (NADH-coenzyme Q reductase)	NDUFS7
244637_at	0.39		
1558148_x_at	0.39		
224276_at	0.39	zinc finger protein 33A	ZNF33A
226218_at	0.39		
1565662_at	0.39	mucin 6, oligomeric mucus/gel-forming	MUC6
213974_at	0.39	ADAMTS-like 3	ADAMTSL3
231366_at	0.39		
203290_at	0.39	major histocompatibility complex, class II, DQ alpha 1	HLA-DQA1

213680_at	0.39	keratin 6B	KRT6B
1569482_at	0.39		
234548_at	0.38		
1570354_s_at	0.38	zinc finger protein 169	ZNF169
210626_at	0.38	A kinase (PRKA) anchor protein 1	AKAP1
209552_at	0.38	paired box gene 8	PAX8
1561531_at	0.38		
238782_at	0.38		
240266_at	0.38	integrator complex subunit 7	INTS7
217319_x_at	0.38	SCL/TAL1 interrupting locus#T-cell acute lymphocytic leukemia 1#PDZK1 interacting protein 1#cytochrome P450, family 4, subfamily Z, polypeptide 1#cytochrome P450, family 4, subfamily A, polypeptide 22	STIL#TAL1#PDZK1IP1#CYP4Z1#CYP4A22
237979_at	0.38	EH-domain containing 4	EHD4
240440_at	0.38	N-acetylneuraminate pyruvate lyase (dihydrodipicolinate synthase)	NPL
1562797_at	0.38		
243163_at	0.38		
210834_s_at	0.38	prostaglandin E receptor 3 (subtype EP3)	PTGER3
1562399_at	0.38		
227023_at	0.38	GLI-Kruppel family member GLI4	GLI4
214345_at	0.38	EGF-like repeats and discoidin I-like domains 3	EDIL3
220908_at	0.38	coiled-coil domain containing 33	CCDC33
225353_s_at	0.38	complement component 1, q subcomponent, C chain	C1QC
237153_at	0.38		
1565784_at	0.38		
1568616_a_at	0.38		
201510_at	0.38	E74-like factor 3 (ets domain transcription factor, epithelial-specific)	ELF3
205696_s_at	0.38	GDNF family receptor alpha 1	GFRA1
205785_at	0.38	integrin, alpha M (complement component 3 receptor 3 subunit)	ITGAM
1559283_a_at	0.38	canopy 1 homolog (zebrafish)	CNPY1
214557_at	0.38	pituitary tumor-transforming 2	PTTG2
244439_at	0.38	sprouty-related, EVH1 domain containing 1	SPRED1
226122_at	0.38	methylenetetrahydrofolate dehydrogenase (NADP+ dependent) 1-like#pleckstrin homology domain containing, family G (with RhoGef domain) member 1	MTHFD1L#PLEKHG1
229247_at	0.38		
231067_s_at	0.38	A kinase (PRKA) anchor protein (gravin) 12	AKAP12
242447_at	0.38		
208027_s_at	0.38	tolloid-like 2	TLL2
239983_at	0.37	solute carrier family 30 (zinc transporter), member 8	SLC30A8
242043_s_at	0.37		
231583_at	0.37	keratin 74	KRT74
1555394_at	0.37	phosphatidylinositol glycan anchor biosynthesis, class K	PIGK
232771_at	0.37	serpin peptidase inhibitor, clade A (alpha-1 antiproteinase, antitrypsin), member 7	SERPINA7
206557_at	0.37	zinc finger protein 702	ZNF702
1555214_a_at	0.37	C-type lectin domain family 7, member A	CLEC7A
205729_at	0.37	oncostatin M receptor	OSMR

1559222_at	0.37		
243658_at	0.37	farnesyl-diphosphate farnesyltransferase 1	FDFT1
215417_at	0.37	exocyst complex component 6B	EXOC6B
238282_at	0.37		
215172_at	0.37	protein tyrosine phosphatase, non-receptor type 20B#protein tyrosine phosphatase, non-receptor type 20A#protein tyrosine phosphatase, non-receptor type 20A	PTPN20B#PTPN20A#PTPN20A
220897_at	0.37		
244368_x_at	0.37		
244190_at	0.37	THAP domain containing 5	THAP5
220338_at	0.37	Ral GEF with PH domain and SH3 binding motif 2	RALGPS2
1564760_at	0.37		
216363_at	0.37		
244608_at	0.37		
243485_at	0.36		
1556235_at	0.36		
228610_at	0.36	transmembrane 9 superfamily member 3	TM9SF3
1552919_at	0.36	chromosome 4 open reading frame 36	C4orf36
228080_at	0.36	layilin	LAYN
208048_at	0.36	tachykinin receptor 1	TACR1
1556175_at	0.36		
1553723_at	0.36	G protein-coupled receptor 97	GPR97
242747_at	0.36		
220880_at	0.36		
205831_at	0.36	CD2 molecule	CD2
211734_s_at	0.36	Fc fragment of IgE, high affinity I, receptor for; alpha polypeptide	FCER1A
209802_at	0.36	pleckstrin homology-like domain, family A, member 2	PHLDA2
1561624_at	0.36		
205033_s_at	0.36	defensin, alpha 1	DEFA1
207505_at	0.36	protein kinase, cGMP-dependent, type II	PRKG2
242897_at	0.36		
214368_at	0.36	RAS guanyl releasing protein 2 (calcium and DAG-regulated)	RASGRP2
1561300_at	0.36		
1554429_a_at	0.36	dystrophia myotonica-containing WD repeat motif	DMWD
222025_s_at	0.36	5-oxoprolinase (ATP-hydrolysing)	OPLAH
1553534_at	0.36	NLR family, pyrin domain containing 10	NLRP10
1558383_at	0.36		
236050_at	0.36	chromosome 11 open reading frame 35	C11orf35
243882_at	0.36		
205931_s_at	0.36	cAMP responsive element binding protein 5	CREB5
1562263_at	0.36	lysyl oxidase-like 2	LOXL2
243228_at	0.36		
234747_at	0.36		
231877_at	0.36	RNA (guanine-9-) methyltransferase domain containing 2	RG9MTD2
1560102_at	0.36		
238618_at	0.36	neurofibromin 2 (bilateral acoustic neuroma)	NF2
215306_at	0.36		
215369_at	0.36		
215468_at	0.35		

1569849_at	0.35		
1564765_at	0.35		
234225_at	0.35		
1561289_at	0.35		
211298_s_at	0.35	albumin	ALB
244865_at	0.35	HCLS1 associated protein X-1	HAX1
214862_x_at	0.35		
206371_at	0.35	folate receptor 3 (gamma)	FOLR3
206948_at	0.35	sialidase 3 (membrane sialidase)	NEU3
206186_at	0.35	membrane protein, palmitoylated 3 (MAGUK p55 subfamily member 3)	MPP3
1560570_a_at	0.35		
214169_at	0.35	unc-84 homolog A (C. elegans)	UNC84A
244041_at	0.35		
1559766_at	0.35		
235380_at	0.35		
240137_at	0.35		
234827_at	0.35		
213721_at	0.35	SRY (sex determining region Y)-box 2	SOX2
218232_at	0.35	complement component 1, q subcomponent, A chain	C1QA
223760_s_at	0.35		
221464_at	0.35	olfactory receptor, family 1, subfamily D, member 2	OR1D2
207826_s_at	0.35	inhibitor of DNA binding 3, dominant negative helix-loop-helix protein	ID3
210634_at	0.35	kelch-like 20 (Drosophila)	KLHL20
210934_at	0.35	B lymphoid tyrosine kinase	BLK
1559936_at	0.34		
232997_at	0.34		
234581_at	0.34		
1566696_at	0.34		
1566176_at	0.34		
1558881_at	0.34		
210832_x_at	0.34	prostaglandin E receptor 3 (subtype EP3)	PTGER3
1563941_at	0.34		
227842_at	0.34	RAB30, member RAS oncogene family	RAB30
1569264_at	0.34		
1559950_at	0.34		
205975_s_at	0.34	homeobox D1	HOXD1
221240_s_at	0.34	UDP-GlcNAc:betaGal beta-1,3-N-acetylglucosaminyltransferase 4	B3GNT4
1555661_at	0.34	olfactory receptor, family 8, subfamily D, member 1	OR8D1
238945_at	0.34		
1553599_a_at	0.34	synaptonemal complex protein 3	SYCP3
222542_x_at	0.34	chaperone, ABC1 activity of bc1 complex homolog (S. pombe)	CABC1
205579_at	0.34	histamine receptor H1	HRH1
242814_at	0.34	serpin peptidase inhibitor, clade B (ovalbumin), member 9	SERPINB9
235979_at	0.34	complement component 7	C7
211551_at	0.34	epidermal growth factor receptor (erythroblastic leukemia viral (v-erb-b) oncogene homolog, avian)	EGFR
242697_at	0.34	zinc finger protein 540	ZNF540
221344_at	0.34	olfactory receptor, family 12, subfamily D, member 2	OR12D2

1555947_at	0.34	family with sequence similarity 120A	FAM120A
223454_at	0.34	chemokine (C-X-C motif) ligand 16	CXCL16
202286_s_at	0.34	tumor-associated calcium signal transducer 2	TACSTD2
227570_at	0.34	transmembrane protein 86A	TMEM86A
233649_at	0.34	katanin p60 subunit A-like 2	KATNAL2
1557117_at	0.34		
217246_s_at	0.34		
239259_at	0.34	zinc finger protein 557	ZNF557
231156_at	0.34		
205342_s_at	0.34	sulfotransferase family, cytosolic, 1C, member 1	SULT1C1
1569776_at	0.34		
1562102_at	0.34	aldo-keto reductase family 1, member C2 (dihydrodiol dehydrogenase 2; bile acid binding protein; 3-a	AKR1C2
204058_at	0.34	malic enzyme 1, NADP(+)-dependent, cytosolic#phosphoglucomutase 3#RWD domain containing 2	ME1#PGM3#RWDD2
1553066_at	0.34	trace amine associated receptor 9	TAAR9
233072_at	0.34	netrin G2	NTNG2
1558577_at	0.34		
222301_at	0.34	chromosome 1 open reading frame 61	C1orf61
211842_s_at	0.34	solute carrier family 24 (sodium/potassium/calcium exchanger), member 1	SLC24A1
223736_at	0.34	intraflagellar transport 81 homolog (Chlamydomonas)	IFT81
244097_at	0.34	complement component (3d/Epstein Barr virus) receptor 2	CR2
231078_at	0.34		
216261_at	0.34	integrin, beta 3 (platelet glycoprotein IIIa, antigen CD61)	ITGB3
1569792_a_at	0.33		
243783_at	0.33		
220428_at	0.33	CD207 molecule, langerin	CD207
220963_s_at	0.33	chromosome 1 open reading frame 89	C1orf89
223756_at	0.33		
205772_s_at	0.33	A kinase (PRKA) anchor protein 7	AKAP7
241740_at	0.33	cAMP responsive element modulator	CREM
230254_at	0.33		
1569864_at	0.33	serine active site containing 1	SERAC1
239902_at	0.33		
1562338_at	0.33	membrane-associated ring finger (C3HC4) 1	1-Mar
242811_x_at	0.33		
1562537_at	0.33	Fc fragment of IgE, high affinity I, receptor for; alpha polypeptide	FCER1A
205557_at	0.33	bactericidal/permeability-increasing protein	BPI
203936_s_at	0.33	matrix metallopeptidase 9 (gelatinase B, 92kDa gelatinase, 92kDa type IV collagenase)	MMP9
237496_at	0.33	3'-phosphoadenosine 5'-phosphosulfate synthase 2	PAPSS2
232487_at	0.33	SFT2 domain containing 1	SFT2D1
215956_at	0.33		
221307_at	0.33	Kv channel interacting protein 1	KCNIP1
1559507_at	0.33		
242835_s_at	0.33		
229228_at	0.33	cAMP responsive element binding protein 5	CREB5

231796_at	0.33	EPH receptor A8#zinc finger and BTB domain containing 40	EPHA8#ZBTB 40
226023_at	0.32	mitogen-activated protein kinase kinase 7	MAP2K7
208950_s_at	0.32	aldehyde dehydrogenase 7 family, member A1	ALDH7A1
210690_at	0.32	killer cell lectin-like receptor subfamily C, member 4	KLRC4
217353_at	0.32	phenylalanine-tRNA synthetase 2 (mitochondrial)	FARS2
235212_at	0.32	chromosome 14 open reading frame 102	C14orf102
216341_s_at	0.32	gonadotropin-releasing hormone receptor	GNRHR
212698_s_at	0.32	septin 10	10-Sep
232324_x_at	0.32		
232748_at	0.32	pregnancy-associated plasma protein A, pappalysin 1	PAPPA
1558651_at	0.32		
238081_at	0.32		
216247_at	0.32		
1569573_at	0.32		
1559895_x_at	0.32		
1557194_a_at	0.32		
233424_at	0.32		
209369_at	0.32	annexin A3	ANXA3
1559413_at	0.32	t-complex 11 (mouse)-like 2	TCP11L2
232226_at	0.32	leucine rich repeat containing 4C	LRRC4C
234101_at	0.32		
222783_s_at	0.32	SPARC related modular calcium binding 1	SMOC1
233081_at	0.32		
234503_at	0.32		
207207_at	0.32		
215463_at	0.32	olfactory receptor, family 7, subfamily E, member 24	OR7E24
33197_at	0.32	myosin VIIA	MYO7A
244596_at	0.31		
236827_at	0.31		
204829_s_at	0.31	folate receptor 2 (fetal)	FOLR2
1557354_at	0.31	son of sevenless homolog 1 (Drosophila)	SOS1
237702_at	0.31		
240300_at	0.31	thymidine kinase 2, mitochondrial	TK2
202948_at	0.31	interleukin 1 receptor, type I	IL1R1
242880_at	0.31	voltage gated channel like 1	VGCNL1
225759_x_at	0.31	calmin (calponin-like, transmembrane)	CLMN
214285_at	0.31	fatty acid binding protein 3, muscle and heart (mammary-derived growth inhibitor)	FABP3
206029_at	0.31	ankyrin repeat domain 1 (cardiac muscle)	ANKRD1
244186_at	0.31	chromosome 10 open reading frame 11	C10orf11
216239_at	0.31	Bardet-Biedl syndrome 9	BBS9
224325_at	0.31	frizzled homolog 8 (Drosophila)	FZD8
237831_x_at	0.31	methylmalonic aciduria (cobalamin deficiency) cblA type	MMAA
227863_at	0.31	cathepsin D	CTSD
204445_s_at	0.31	arachidonate 5-lipoxygenase	ALOX5
1563945_at	0.31		
1555024_at	0.31	ADAM metallopeptidase domain 22	ADAM22
1559790_at	0.31		
237865_x_at	0.31		
1560742_at	0.31		
235617_x_at	0.31		

215770_at	0.31	olfactory receptor, family 7, subfamily E, member 2 pseudogene	OR7E2P
206363_at	0.31	v-maf musculoaponeurotic fibrosarcoma oncogene homolog (avian)	MAF
203472_s_at	0.31	solute carrier organic anion transporter family, member 2B1	SLCO2B1
1556272_a_at	0.31		
1562524_at	0.31		
1569624_at	0.31		
238301_at	0.30		
212942_s_at	0.30	KIAA1199	KIAA1199
221065_s_at	0.30	carbohydrate (N-acetylgalactosamine 4-0) sulfotransferase 8	CHST8
237675_at	0.30		
1569416_at	0.30		
222097_at	0.30		
244242_at	0.30		
1561759_at	0.30		
215779_s_at	0.30	histone cluster 1, H2bg	HIST1H2BG
240027_at	0.30	lin-7 homolog A (C. elegans)	LIN7A
244645_at	0.30	collagen, type XIV, alpha 1 (undulin)	COL14A1
209719_x_at	0.30	serpin peptidase inhibitor, clade B (ovalbumin), member 3	SERPINB3
239814_at	0.30		
240330_at	0.30		
203381_s_at	0.30	apolipoprotein E	APOE
228121_at	0.30		
1566973_at	0.30		
210920_x_at	0.30	EMI domain containing 2	EMID2
230507_at	0.30	ataxin 1	ATXN1
1563392_at	0.30		
242979_at	0.30		
208430_s_at	0.30	dystrobrevin, alpha	DTNA
206785_s_at	0.29	killer cell lectin-like receptor subfamily C, member 2	KLRC2
1561644_x_at	0.29		
234603_at	0.29		
1553627_s_at	0.29	chromosome 17 open reading frame 57	C17orf57
204631_at	0.29	myosin, heavy chain 2, skeletal muscle, adult	MYH2
215899_at	0.29		
1556111_s_at	0.29		
1570038_at	0.29	zinc finger protein 718	ZNF718
234094_x_at	0.29		
234682_at	0.29	BTB (POZ) domain containing 9	BTBD9
1562477_at	0.29	early B-cell factor 2	EBF2
1552765_x_at	0.29	transmembrane protein 67	TMEM67
223614_at	0.29	chromosome 8 open reading frame 57	C8orf57
221958_s_at	0.29	G protein-coupled receptor 177	GPR177
240457_at	0.29		
233433_at	0.29		
1566249_at	0.29		
216055_at	0.29	platelet-derived growth factor beta polypeptide (simian sarcoma viral (v-sis) oncogene homolog)	PDGFB
1554536_at	0.29	dihydropyrimidine dehydrogenase	DPYD

227660_at	0.29	anthrax toxin receptor 1	ANTXR1
1569786_at	0.29		
235185_s_at	0.29		
38918_at	0.29	SRY (sex determining region Y)-box 13	SOX13
241154_x_at	0.28		
224424_x_at	0.28		
233838_at	0.28		
234670_at	0.28		
1558356_at	0.28	uveal autoantigen with coiled-coil domains and ankyrin repeats	UACA
213663_s_at	0.28		
240290_at	0.28		
213249_at	0.28	F-box and leucine-rich repeat protein 7	FBXL7
1557775_a_at	0.28		
232060_at	0.28		
205403_at	0.28	interleukin 1 receptor, type II	IL1R2
214189_s_at	0.28	golgi associated, gamma adaptin ear containing, ARF binding protein 2	GGA2
237920_at	0.28	synaptonemal complex protein 2	SYCP2
240022_at	0.28	chromosome 19 open reading frame 7	C19orf7
1555854_at	0.28		
1557241_a_at	0.28		
216133_at	0.28	T cell receptor alpha locus	TRA@
237338_at	0.28	UDP-GlcNAc:betaGal beta-1,3-N-acetylglucosaminyltransferase 8	B3GNT8
240789_at	0.28	WD repeat domain 44	WDR44
243122_at	0.28		
214798_at	0.28	ATPase, Ca++ transporting, type 2C, member 2	ATP2C2
205799_s_at	0.28	solute carrier family 3 (cystine, dibasic and neutral amino acid transporters, activator of cystine,	SLC3A1
224124_at	0.28	zinc finger, RAN-binding domain containing 3	ZRANB3
241771_at	0.28	RIMS binding protein 2	RIMBP2
244655_at	0.28		
228503_at	0.28		
1559252_a_at	0.28	chromosome 20 open reading frame 29	C20orf29
230801_at	0.28	chromosome 20 open reading frame 77	C20orf77
221309_at	0.28	RNA binding motif protein 17	RBM17
210854_x_at	0.27	solute carrier family 6 (neurotransmitter transporter, creatine), member 8	SLC6A8
1564443_at	0.27	deleted in lymphocytic leukemia, 2	DLEU2
238608_at	0.27		
1559624_at	0.27	serine/threonine kinase 32A	STK32A
224056_at	0.27	guanine nucleotide binding protein (G protein), alpha 13	GNA13
240571_at	0.27		
1561407_at	0.27	centaurin, delta 1	CENTD1
230783_at	0.27		
227463_at	0.27		
243842_at	0.27		
1555176_at	0.27		
215569_at	0.27	general transcription factor II, i	GTF2I
242159_at	0.27		
232734_at	0.27	tetratricopeptide repeat domain 23	TTC23

202995_s_at	0.27	fibulin 1	FBLN1
233821_at	0.27	RAB32, member RAS oncogene family	RAB32
210952_at	0.27	adaptor-related protein complex 4, sigma 1 subunit	AP4S1
1552875_a_at	0.27	CD200 receptor 1	CD200R1
223918_at	0.27	acyl-CoA synthetase long-chain family member 6	ACSL6
203868_s_at	0.27	vascular cell adhesion molecule 1	VCAM1
224045_x_at	0.26	chromosome 18 open reading frame 2	C18orf2
1552858_at	0.26	melanoma antigen family B, 6	MAGEB6
1566108_at	0.26	myoneurin	MYNN
206345_s_at	0.26	paraoxonase 1	PON1
1556521_a_at	0.26		
1555107_a_at	0.26		
236877_at	0.26		
231001_at	0.26		
221372_s_at	0.26	purinergic receptor P2X, ligand-gated ion channel, 2	P2RX2
232837_at	0.26	kinesin family member 13A	KIF13A
239829_at	0.26		
241279_at	0.26	proteoglycan 1, secretory granule	PRG1
237411_at	0.26	ADAM metallopeptidase with thrombospondin type 1 motif, 6	ADAMTS6
210118_s_at	0.26	interleukin 1, alpha	IL1A
242256_x_at	0.26		
234931_at	0.26		
213856_at	0.26	CD47 molecule	CD47
221584_s_at	0.26	potassium large conductance calcium-activated channel, subfamily M, alpha member 1	KCNMA1
1565787_at	0.26		
204261_s_at	0.26	presenilin 2 (Alzheimer disease 4)	PSEN2
1557769_at	0.26	chimerin (chimaerin) 2	CHN2
231435_at	0.26	chromosome 7 open reading frame 34	C7orf34
243607_at	0.26		
208194_s_at	0.26	signal transducing adaptor molecule (SH3 domain and ITAM motif) 2	STAM2
1566740_at	0.26	phospholipase C, epsilon 1	PLCE1
215916_at	0.26	misshapen-like kinase 1 (zebrafish)	MINK1
1560929_at	0.26		
1563461_at	0.26		
241646_s_at	0.26		
224050_s_at	0.26		
1570644_at	0.26		
201131_s_at	0.26	cadherin 1, type 1, E-cadherin (epithelial)	CDH1
217369_at	0.26	immunoglobulin heavy constant gamma 1 (G1m marker)	IGHG1
1561417_x_at	0.26		
226225_at	0.25	mutated in colorectal cancers	MCC
1553811_at	0.25		
243066_at	0.25	N-acetylneuraminate pyruvate lyase (dihydrodipicolinate synthase)	NPL
1559538_at	0.25		
233282_at	0.25		
1560941_a_at	0.25		
224408_at	0.25	melanin-concentrating hormone receptor 2	MCHR2
1556508_s_at	0.25		

212328_at	0.25		
1562583_s_at	0.25		
207201_s_at	0.25	solute carrier family 22 (organic cation transporter), member 1	SLC22A1
1553208_s_at	0.25	ADP-ribosylation factor-like 10	ARL10
242686_at	0.25	START domain containing 13	STARD13
201789_at	0.25	dehydrogenase/reductase (SDR family) member 7	DHRS7
1561281_a_at	0.25		
1555867_at	0.25		
234650_at	0.25		
220824_at	0.25		
206183_s_at	0.25	hect domain and RLD 3	HERC3
1566780_at	0.25		
243401_at	0.25	formin-like 2	FMNL2
207500_at	0.25	caspase 5, apoptosis-related cysteine peptidase	CASP5
215798_at	0.24	aldehyde dehydrogenase 1 family, member L1	ALDH1L1
235099_at	0.24	CKLF-like MARVEL transmembrane domain containing 8	CMTM8
241974_at	0.24		
220811_at	0.24	proteoglycan 3	PRG3
206773_at	0.24	lymphocyte antigen 6 complex, locus H	LY6H
206676_at	0.24	carcinoembryonic antigen-related cell adhesion molecule 8	CEACAM8
242802_x_at	0.24		
225987_at	0.24	STEAP family member 4	STEAP4
216475_at	0.24		
203216_s_at	0.24	myosin VI	MYO6
200724_at	0.24	ribosomal protein L10	RPL10
217849_s_at	0.24	CDC42 binding protein kinase beta (DMPK-like)	CDC42BPB
1559129_a_at	0.24		
236762_at	0.24	ATPase, Class II, type 9B	ATP9B
1562059_at	0.24		
216214_at	0.24		
238780_s_at	0.24		
230117_at	0.24	V-set and transmembrane domain containing 2	VSTM2
241007_at	0.24		
244094_at	0.24		
224104_at	0.24		
237395_at	0.24	cytochrome P450, family 4, subfamily Z, polypeptide 1	CYP4Z1
203875_at	0.24	SWI/SNF related, matrix associated, actin dependent regulator of chromatin, subfamily a, member 1	SMARCA1
232276_at	0.24	heparan sulfate 6-O-sulfotransferase 3	HS6ST3
239075_at	0.24	death inducer-obliterator 1	DIDO1
1568448_at	0.24		
231981_at	0.24	prolactin receptor	PRLR
231683_at	0.24	glycine-N-acyltransferase	GLYAT
1566700_at	0.24	vaccinia related kinase 3	VRK3
221615_at	0.23	peptidylprolyl isomerase E (cyclophilin E)	PPIE
1561254_at	0.23		
1557617_at	0.23		
216568_x_at	0.23	aminolevulinate, delta-, synthase 2 (sideroblastic/hypochromic anemia)	ALAS2
1569809_at	0.23		

240492_at	0.23		
209335_at	0.23	decorin	DCN
1556763_at	0.23		
224213_at	0.23	chromosome 14 open reading frame 91	C14orf91
227885_at	0.23		
1553080_at	0.23	casein alpha s2-like A	CSN1S2A
1568736_s_at	0.23		
206355_at	0.23	guanine nucleotide binding protein (G protein), alpha activating activity polypeptide, olfactory typ	GNAL
237707_at	0.23		
1556410_a_at	0.23	keratin associated protein 19-1	KRTAP19-1
1552401_a_at	0.23		
209708_at	0.23	monooxygenase, DBH-like 1	MOXD1
234472_at	0.23	UDP-N-acetyl-alpha-D-galactosamine:polypeptide N-acetylgalactosaminyltransferase 13 (GalNAc-T13)	GALNT13
233048_at	0.23	family with sequence similarity 35, member A	FAM35A
201427_s_at	0.23	selenoprotein P, plasma, 1	SEPP1
219949_at	0.22	leucine rich repeat containing 2	LRRC2
205375_at	0.22	MyoD family inhibitor	MDFI
1553859_at	0.22	tryptophan hydroxylase 1 (tryptophan 5-monooxygenase)	TPH1
1561316_at	0.22	gamma-aminobutyric acid (GABA) A receptor, beta 3	GABRB3
1564200_at	0.22		
244784_at	0.22	DEAH (Asp-Glu-Ala-Asp/His) box polypeptide 57	DHX57
243095_at	0.22		
240354_at	0.22	chromosome 12 open reading frame 54	C12orf54
211249_at	0.22	G protein-coupled receptor 68	GPR68
241393_at	0.22		
1553296_at	0.22	G protein-coupled receptor 128	GPR128
242050_at	0.22	leucine rich repeat containing 7	LRRC7
209386_at	0.22	transmembrane 4 L six family member 1	TM4SF1
1565838_at	0.22		
203131_at	0.22	platelet-derived growth factor receptor, alpha polypeptide	PDGFRA
211673_s_at	0.22	molybdenum cofactor synthesis 1	MOCS1
1556221_a_at	0.22		
241739_at	0.22	2-oxoglutarate and iron-dependent oxygenase domain containing 1	OGFOD1
1558595_at	0.22		
242469_at	0.21		
230271_at	0.21	one cut domain, family member 2	ONECUT2
214070_s_at	0.21	ATPase, Class V, type 10B	ATP10B
227919_at	0.21		
240556_at	0.21	decorin	DCN
1557724_a_at	0.21	FK506 binding protein 5	FKBP5
243650_at	0.21	pleckstrin homology domain containing, family H (with MyTH4 domain) member 2	PLEKHH2
232246_at	0.21		
227142_at	0.21	pleckstrin homology domain containing, family G (with RhoGef domain) member 5	PLEKHG5
238492_at	0.21		
213409_s_at	0.21	Ras homolog enriched in brain	RHEB
202289_s_at	0.21	transforming, acidic coiled-coil containing protein 2	TACC2

231166_at	0.21		
1567853_at	0.21	zinc finger protein 28	ZNF28
1558474_at	0.21		
224030_s_at	0.21		
230513_at	0.21	HLA-B associated transcript 3	BAT3
237061_at	0.21	zinc finger protein 347	ZNF347
234236_at	0.21		
232335_at	0.21		
213709_at	0.21	basic helix-loop-helix domain containing, class B, 9	BHLHB9
1555216_a_at	0.21		
214967_at	0.21		
1564257_at	0.21		
209869_at	0.21	adrenergic, alpha-2A-, receptor	ADRA2A
239650_at	0.21		
241677_x_at	0.20		
244504_x_at	0.20	ADP-ribosylation factor 1	ARF1
1568900_a_at	0.20	zinc finger protein 568	ZNF568
234176_at	0.20		
230863_at	0.20	low density lipoprotein-related protein 2	LRP2
222259_s_at	0.20	RAE1 RNA export 1 homolog (S. pombe)#SPO11 meiotic protein covalently bound to DSB homolog (S. cerevisiae)	RAE1#SPO11
230378_at	0.20	secretoglobin, family 3A, member 1	SCGB3A1
1560990_a_at	0.20		
221030_s_at	0.20	Rho GTPase activating protein 24	ARHGAP24
1562598_at	0.20		
234819_at	0.20	T cell receptor alpha locus#T cell receptor delta variable 1#T cell receptor alpha variable 36/delta variable 7#T cell receptor alpha variable 35#T cell receptor alpha variable 34#T cell receptor alpha variable 33#T cell receptor alpha variable 32#T cell receptor alpha variable 31#T cell receptor alpha variable 30#T cell receptor alpha variable 29/delta variable 5#T cell receptor alpha variable 28#T cell receptor alpha variable 27#T cell receptor alpha variable 26-2#T cell receptor alpha variable 26-1#T cell receptor alpha variable 25#T cell receptor alpha variable 24#T cell receptor alpha variable 23/delta variable 6#T cell receptor alpha variable 22#T cell receptor alpha variable 21#T cell receptor alpha variable 20#T cell receptor alpha variable 19#T cell receptor alpha variable 18#T cell receptor alpha variable 17#T cell receptor alpha variable 8-7	TRA@#TRDV1 #TRAV36DV7#TRAV35#TRAV34#TRAV33#TRAV32#TRAV31#TRAV30#TRAV29DV5#TRAV28#TRAV27#TRAV26-2#TRAV26-1#TRAV25#TRAV24#TRAV23DV6#TRAV22#TRAV21#TRAV20#TRAV19#TRAV18#TRAV17#TRAV8-7
238503_at	0.20		
1560101_at	0.20	synapse defective 1, Rho GTPase, homolog 2 (C. elegans)	SYDE2
234097_s_at	0.20	chromosome 6 open reading frame 12	C6orf12
1569855_at	0.20		
1568856_at	0.20	neighbor of BRCA1 gene 1	NBR1
1554143_a_at	0.20	SGT1, suppressor of G2 allele of SKP1 like 1 (S. cerevisiae)	SUGT1L1

236197_at	0.20		
235756_at	0.20	sterile alpha motif domain containing 4A	SAMD4A
1564854_at	0.20		
235379_at	0.20		
1563224_at	0.20		
241781_at	0.20	chromosome 9 open reading frame 41	C9orf41
207703_at	0.20	neuroligin 4, Y-linked	NLGN4Y
1563963_at	0.19		
231580_at	0.19		
221312_at	0.19	glucagon-like peptide 2 receptor	GLP2R
222927_s_at	0.19	complexin 3	CPLX3
219114_at	0.19	chromosome 3 open reading frame 18	C3orf18
1560424_at	0.19		
221176_x_at	0.19	Williams-Beuren syndrome chromosome region 23	WBSCR23
238111_at	0.19	serologically defined colon cancer antigen 3	SDCCAG3
236945_at	0.19	chromosome 9 open reading frame 93	C9orf93
220623_s_at	0.19	testis specific, 10	TSGA10
216441_at	0.19		
228740_at	0.19		
220726_at	0.19		
1556362_at	0.19		
233604_at	0.19		
232268_at	0.18		
235453_at	0.18		
228376_at	0.18	glycoprotein, alpha-galactosyltransferase 1	GGTA1
244228_at	0.18	RAB GTPase activating protein 1	RABGAP1
212353_at	0.18	sulfatase 1	SULF1
1559627_at	0.18		
216910_at	0.18	X-prolyl aminopeptidase (aminopeptidase P) 2, membrane-bound	XPNPEP2
208280_at	0.18	CMT1A duplicated region transcript 1	CDRT1
216084_at	0.18	chromosome 9 open reading frame 144	C9orf144
1566033_at	0.18		
227140_at	0.18		
239739_at	0.18		
1556678_a_at	0.18		
207651_at	0.18	G protein-coupled receptor 171	GPR171
1556086_at	0.18	chromosome 4 open reading frame 27	C4orf27
1561214_at	0.18		
243975_at	0.18	reticulon 4 receptor-like 1	RTN4RL1
1558308_at	0.18		
216712_at	0.17	transmembrane protein 132A	TMEM132A
223612_s_at	0.17	ligand of numb-protein X 1	LNX1
228377_at	0.17	kelch-like 14 (Drosophila)	KLHL14
208394_x_at	0.17	endothelial cell-specific molecule 1	ESM1
241280_at	0.17	aldolase B, fructose-bisphosphate	ALDOB
216420_at	0.17	TAR DNA binding protein-like	TARDBPL
1569571_at	0.17	lipase-like, ab-hydrolase domain containing 1	LIPL1
225163_at	0.17	FERM domain containing 4A	FRMD4A
202674_s_at	0.17	LIM domain 7	LMO7
234790_at	0.17		
235150_at	0.17		
1562742_at	0.17		

1562677_at	0.17		
240874_at	0.17		
221244_s_at	0.17	3-phosphoinositide dependent protein kinase-1	PDPK1
237982_at	0.17		
238852_at	0.17		
216298_at	0.17		
240085_at	0.17		
238262_at	0.17	speedy homolog A (Drosophila)	SPDYA
207900_at	0.17	chemokine (C-C motif) ligand 17	CCL17
1561679_at	0.17		
1556620_at	0.17		
1559309_at	0.17	multiple coagulation factor deficiency 2	MCFD2
1555073_at	0.17		
234399_at	0.16	T cell receptor alpha locus#T cell receptor delta variable 1#T cell receptor alpha variable 36/delta variable 7#T cell receptor alpha variable 35#T cell receptor alpha variable 34#T cell receptor alpha variable 33#T cell receptor alpha variable 32#T cell receptor alpha variable 31#T cell receptor alpha variable 30#T cell receptor alpha variable 29/delta variable 5#T cell receptor alpha variable 28#T cell receptor alpha variable 27#T cell receptor alpha variable 26-2#T cell receptor alpha variable 26-1#T cell receptor alpha variable 25#T cell receptor alpha variable 24#T cell receptor alpha variable 23/delta variable 6#T cell receptor alpha variable 22#T cell receptor alpha variable 21#T cell receptor alpha variable 20#T cell receptor alpha variable 19#T cell receptor alpha variable 18#T cell receptor alpha variable 17#T cell receptor alpha variable 8-7	TRA@#TRDV1#TRAV36DV7#TRAV35#TRAV34#TRAV33#TRAV32#TRAV31#TRAV30#TRAV29DV5#TRAV28#TRAV27#TRAV26-2#TRAV26-1#TRAV25#TRAV24#TRAV23DV6#TRAV22#TRAV21#TRAV20#TRAV19#TRAV18#TRAV17#TRAV8-7
1561440_at	0.16		
214421_x_at	0.16	cytochrome P450, family 2, subfamily C, polypeptide 9	CYP2C9
235401_s_at	0.16	Fc receptor-like A	FCRLA
239153_at	0.16		
235116_at	0.16	TNF receptor-associated factor 1	TRAF1
221321_s_at	0.16	Kv channel interacting protein 2	KCNIP2
207425_s_at	0.16	septin 9	9-Sep
232321_at	0.16	mucin 17, cell surface associated	MUC17
236740_at	0.16		
1557906_at	0.16		
207723_s_at	0.16	killer cell lectin-like receptor subfamily C, member 3	KLRC3
240160_x_at	0.16		
236304_at	0.16		
244820_at	0.16	zinc and ring finger 3	ZNRF3
235763_at	0.15	solute carrier family 44, member 5	SLC44A5
235732_at	0.15		
230933_at	0.15	destrin (actin depolymerizing factor)	DSTN
1557120_at	0.15	eukaryotic translation elongation factor 1 alpha 1	EEF1A1
215518_at	0.15	syntaxin binding protein 5-like	STXBP5L

213270_at	0.15	membrane protein, palmitoylated 2 (MAGUK p55 subfamily member 2)	MPP2
228632_at	0.15		
1563478_at	0.15		
229992_at	0.15		
1565130_at	0.15		
234108_at	0.15	taste receptor, type 2, member 45	TAS2R45
231385_at	0.15	developmental pluripotency associated 3	DPPA3
229495_at	0.15	aminoacylase 1-like 2	ACY1L2
1552649_a_at	0.15	ring finger and FYVE-like domain containing 1	RFFL
1562937_at	0.15		
239092_at	0.15	integrin, alpha 8	ITGA8
206022_at	0.15	Norrie disease (pseudoglioma)	NDP
238428_at	0.15	potassium inwardly-rectifying channel, subfamily J, member 15	KCNJ15
1552745_at	0.15	solute carrier organic anion transporter family, member 6A1	SLCO6A1
232694_at	0.15	zinc finger protein 395	ZNF395
216837_at	0.15	EPH receptor A5	EPHA5
241547_at	0.15		
1560733_at	0.15		
239697_x_at	0.14		
1563856_at	0.14	insulin activator factor (insulin control element-binding transcription factor)	INSAF
244537_at	0.14	huntingtin interacting protein 2	HIP2
230923_at	0.14	family with sequence similarity 19 (chemokine (C-C motif)-like), member A1	FAM19A1
222168_at	0.14		
1555246_a_at	0.14	sodium channel, voltage-gated, type I, alpha subunit	SCN1A
235273_at	0.14	dyslexia susceptibility 1 candidate 1	DYX1C1
241766_at	0.14		
205862_at	0.14		
241898_at	0.14		
243968_x_at	0.14	Fc receptor-like 1	FCRL1
1561143_at	0.14		
1553301_a_at	0.14	transmembrane protein 182	TMEM182
220787_at	0.14		
214079_at	0.14	dehydrogenase/reductase (SDR family) member 2	DHRS2
1562879_at	0.14		
237320_at	0.13		
242102_at	0.13		
244856_at	0.13		
234765_at	0.13		
1555123_at	0.13	ST6 beta-galactosamide alpha-2,6-sialyltranferase 2	ST6GAL2
1570054_at	0.13		
230854_at	0.13	breast cancer anti-estrogen resistance 4	BCAR4
239914_at	0.13		
210355_at	0.13	parathyroid hormone-like hormone	PTHLH
216970_at	0.13	regulator of G-protein signalling 7	RGS7
1562311_at	0.13		
1553364_at	0.13	patatin-like phospholipase domain containing 1	PNPLA1
1554500_a_at	0.13	regulator of G-protein signalling 7	RGS7

222219_s_at	0.13	transducin-like enhancer of split 2 (E(sp1) homolog, Drosophila)#transducin-like enhancer of split 6 (E(sp1) homolog, Drosophila)	TLE2#TLE6
1561362_at	0.13		
214632_at	0.12	neuropilin 2	NRP2
215393_s_at	0.12	COBL-like 1	COBLL1
232450_at	0.12		
228767_at	0.12	ataxin 2-like	ATXN2L
220614_s_at	0.12	chromosome 6 open reading frame 103	C6orf103
236220_at	0.12		
241653_x_at	0.12		
1556725_a_at	0.12		
228170_at	0.12	oligodendrocyte transcription factor 1	OLIG1
236538_at	0.12	glutamate receptor, ionotropic, AMPA 2	GRIA2
1555925_at	0.11		
206218_at	0.11	melanoma antigen family B, 2	MAGEB2
240448_at	0.11	KIAA0802	KIAA0802
238318_at	0.11		
1569782_at	0.11		
1553793_a_at	0.11	KIAA1109	KIAA1109
220749_at	0.11	chromosome 10 open reading frame 68	C10orf68
231181_at	0.11		
215302_at	0.11		
215717_s_at	0.11	fibrillin 2 (congenital contractural arachnodactyly)	FBN2
216992_s_at	0.11	glutamate receptor, metabotropic 8	GRM8
1564209_at	0.11		
240179_at	0.11		
1560566_at	0.11	protocadherin 20	PCDH20
221126_at	0.11		
1566607_at	0.11		
212667_at	0.10	secreted protein, acidic, cysteine-rich (osteonectin)	SPARC
243421_at	0.10		
233320_at	0.10	testicular cell adhesion molecule 1 homolog (mouse)	TCAM1
208404_x_at	0.10	potassium inwardly-rectifying channel, subfamily J, member 5	KCNJ5
233967_at	0.10		
235565_at	0.10	zinc finger protein 425	ZNF425
242199_at	0.10		
240334_at	0.10	leucine rich repeat and fibronectin type III domain containing 5	LRFN5
1553792_at	0.10	KIAA1109	KIAA1109
211646_at	0.10		
207175_at	0.10	adiponectin, C1Q and collagen domain containing	ADIPOQ
241500_at	0.10		
229623_at	0.10		
1553883_at	0.10	zinc finger protein 99	ZNF99
1555492_a_at	0.10	bestrophin 3	BEST3
207322_at	0.10	intersectin 1 (SH3 domain protein)	ITSN1
215554_at	0.10	glycosylphosphatidylinositol specific phospholipase D1	GPLD1
242387_at	0.09	chromosome 8 open reading frame 42	C8orf42
1556963_at	0.09		
206415_at	0.09	tolloid-like 1	TLL1

1564949_at	0.09		
1553422_s_at	0.09		
1566860_at	0.09		
228933_at	0.09	Nance-Horan syndrome (congenital cataracts and dental anomalies)	NHS
234099_at	0.09		
1555339_at	0.08	RAP1A, member of RAS oncogene family	RAP1A
1557793_at	0.08	family with sequence similarity 62 (C2 domain containing), member C	FAM62C
225806_at	0.08	jub, ajuba homolog (Xenopus laevis)	JUB
1569783_at	0.07		
215817_at	0.07	serpin peptidase inhibitor, clade B (ovalbumin), member 13	SERPINB13
242107_x_at	0.07		
1563827_at	0.07		
1555340_x_at	0.07	RAP1A, member of RAS oncogene family	RAP1A
234853_s_at	0.07	YWHAQ pseudogene 2	YWHAQP2
228748_at	0.06	CD59 molecule, complement regulatory protein	CD59
237973_at	0.06		
1570163_at	0.06		
207400_at	0.05	neuropeptide Y receptor Y5	NPY5R
1556609_at	0.05		
242138_at	0.05	distal-less homeobox 1	DLX1
205433_at	0.05	butyrylcholinesterase	BCHE
244465_at	0.05		
215118_s_at	0.05	immunoglobulin heavy constant alpha 1	IGHA1
1555103_s_at	0.04	fibroblast growth factor 7 (keratinocyte growth factor)	FGF7
243644_at	0.04		
241069_at	0.04	zinc finger and BTB domain containing 16	ZBTB16
