# Supplementary material for: In Vitro Transformation of Primary Human CD34+ Cells by AML Fusion Oncogenes: Early Gene Expression Profiling Reveals Possible Drug Target in AML
Source: PLoS One. 2010 Aug 27;5(8):e12464. doi: 10.1371/journal.pone.0012464 (PMC2929205; doi:10.1371/journal.pone.0012464)
Supplement: Table S25 — Union lists for all 3 time points for each oncogene were generated from the lists shown in Tables S13 - S24 and subjected to GeneGo analysis as described in Materials and Methods. The top 10 enriched pathways for each oncogene are shown along with the corresponding p-values. (0.05 MB PDF) [file pone.0012464.s025.pdf]

Table S25. Top 10 pathways deregulated by each AML-associated oncogene

AML1-ETO		
	**Pathway**	**p-value**
1	Development_Regulation of epithelial-to-mesenchymal transition (EMT)	5.32E-16
2	Cytoskeleton remodeling_TGF, WNT and cytoskeletal remodeling	1.66E-13
3	Cell adhesion_Chemokines and adhesion	2.46E-11
4	Development_TGF-beta receptor signaling	7.04E-11
5	Development_TGF-beta-dependent induction of EMT via SMADs	1.34E-10
6	Development_TGF-beta-dependent induction of EMT via RhoA, PI3K and ILK.	3.50E-10
7	Development_PIP3 signaling in cardiac myocytes	6.26E-10
8	Some pathways of EMT in cancer cells	8.41E-10
9	Apoptosis and survival_Lymphotoxin-beta receptor signaling	9.48E-10
10	Apoptosis and survival_Anti-apoptotic TNFs/NF-kB/Bcl-2 pathway	6.97E-09

PML-RARA		
	**Pathway**	**p-value**
1	Cytoskeleton remodeling_TGF, WNT and cytoskeletal remodeling	5.08E-09
2	Development_TGF-beta-dependent induction of EMT via MAPK	1.60E-08
3	Cytoskeleton remodeling_Cytoskeleton remodeling	3.76E-08
4	Development_Regulation of epithelial-to-mesenchymal transition (EMT)	3.98E-08
5	Signal transduction_Activation of PKC via G-Protein coupled receptor	1.10E-07
6	Immune response_Classical complement pathway	1.10E-07
7	Development_FGFR signaling pathway	1.57E-07
8	Development_TGF-beta-dependent induction of EMT via SMADs	2.11E-07
9	Immune response_Lectin induced complement pathway	2.17E-07
10	Immune response_Th1 and Th2 cell differentiation	2.62E-07

MLL-AF9		
	**Pathway**	**p-value**
1	Development_Regulation of epithelial-to-mesenchymal transition (EMT)	1.37E-10
2	Cytoskeleton remodeling_TGF, WNT and cytoskeletal remodeling	8.70E-10
3	Cardiac Hypertrophy_NF-AT signaling in Cardiac Hypertrophy	6.69E-09
4	Cell adhesion_Chemokines and adhesion	5.70E-07
5	Signal transduction_PKA signaling	7.87E-07
6	Cell adhesion_Integrin inside-out signaling	4.03E-06
7	Muscle contraction_ GPCRs in the regulation of smooth muscle tone	5.65E-06
8	Blood coagulation_GPVI-dependent platelet activation	1.28E-05
9	Immune response_IL-6 signaling pathway	2.54E-05
10	Cytoskeleton remodeling_Cytoskeleton remodeling	2.81E-05

NUP98-HOXA9		
	**Pathway**	**p-value**
1	Development_Regulation of epithelial-to-mesenchymal transition (EMT)	2.14E-12
2	Immune response_IFN alpha/beta signaling pathway	2.29E-09
3	Cytoskeleton remodeling_TGF, WNT and cytoskeletal remodeling	9.92E-09
4	Development_TGF-beta-dependent induction of EMT via RhoA, PI3K and ILK	4.38E-08
5	Development_TGF-beta-dependent induction of EMT via MAPK	6.49E-08
6	Development_EGFR signaling pathway	8.84E-08

7	Immune response_Classical complement pathway	3.89E-07
8	Development_ERBB-family signaling	7.70E-07
9	Immune response_Alternative complement pathway	7.70E-07
10	Development_TGF-beta-dependent induction of EMT via SMADs	1.03E-06
